# Acute and short-term fluctuations in gravity are associated with changes in circulatory plasma protein levels

**DOI:** 10.1038/s41526-024-00370-y

**Published:** 2024-03-04

**Authors:** Alexander Lang, Stephan Binneboessel, Fabian Nienhaus, Raphael Romano Bruno, Georg Wolff, Kerstin Piayda, Susanne Pfeiler, Hakima Ezzahoini, Daniel Oehler, Malte Kelm, Holger Winkels, Norbert Gerdes, Christian Jung

**Affiliations:** 1 Division of Cardiology, Pulmonology, and Vascular Medicine, University Hospital and Medical Faculty, Heinrich-Heine University, Düsseldorf, Germany; 2 Department of Cardiology and Vascular Medicine, Medical Faculty, Justus-Liebig-University Giessen, Giessen, Germany; 3 Cardiovascular Research Institute Düsseldorf (CARID), Medical Faculty, Heinrich-Heine University, Düsseldorf, Germany; 4 Clinic III for Internal Medicine, Faculty of Medicine and University Hospital Cologne, Cologne, Germany

**Keywords:** Medical research, Biomarkers

## Abstract

Gravitational changes between micro- and hypergravity cause several adaptations and alterations in the human body. Besides muscular atrophy and immune system impairment, effects on the circulatory system have been described, which can be associated with a wide range of blood biomarker changes. This study examined nine individuals (seven males, two females) during a parabolic flight campaign (PFC). Thirty-one parabolas were performed in one flight day, resulting in ~22 s of microgravity during each parabola. Each participant was subjected to a single flight day with a total of 31 parabolas, totaling 11 min of microgravity during one parabolic flight. Before and after (1 hour (h) and 24 h), the flights blood was sampled to examine potential gravity-induced changes of circulating plasma proteins. Proximity Extension Assay (PEA) offers a proteomic solution, enabling the simultaneous analysis of a wide variety of plasma proteins. From 2925 unique proteins analyzed, 251 (8.58%) proteins demonstrated a differential regulation between baseline, 1 h and 24 h post flight. Pathway analysis indicated that parabolic flights led to altered levels of proteins associated with vesicle organization and apoptosis up to 24 h post microgravity exposure. Varying gravity conditions are associated with poorly understood physiological changes, including stress responses and fluid shifts. We provide a publicly available library of gravity-modulated circulating protein levels illustrating numerous changes in cellular pathways relevant for inter-organ function and communication.

## Introduction

The human cardiovascular system evolved in and adapted to terrestrial gravity (or weightiness) of ~9.81 m/s² corresponding to 1×*g*^1,2^. Although in all correctness, one should talk about weightiness, we will use the word “gravity” instead to signify what should be weightiness^3^. The effects of microgravity, as encountered in space, on the human body have drawn significant attention due to their importance for future space missions, including those to the Moon and Mars, and the growing commercial spaceflight sector. Microgravity influences many physiological systems and functions, such as wound healing, lung volume, breathing patterns, disorientation, motion sickness, and alterations in blood pressure and fluid distribution. Among these, the circulatory system plays a crucial role in maintaining overall health and well-being^4–6^. Over the next few years, preventive medicine will be an important cornerstone to allow safe and longer-lasting spaceflights. Future space missions head for the Moon and Mars, after more than two decades of continuous human presence in lower earth orbit on the International Space Station (ISS)^7,8^. Understanding the potential implications of microgravity-induced changes in the circulatory system is essential for developing effective countermeasures that can help astronauts maintain cognitive, motoric, and cardiovascular performance during space missions. The majority of them were professional male astronauts in their forties, who had undergone thorough psychological and physiological examinations^9^. Impairments of several psychomotor and cognitive skills, including basic postural functions, the speed and precision of intentional motions, internal timekeeping, and central control of concurrent tasks, during spaceflight were demonstrated in previous studies^10–13^. The aim of this study is to conduct an unbiased high-parametric characterization of the plasma proteome following 31 parabolas causing phases of hypergravity with 1.8× *g* and microgravity exposure, totaling approximately 11 min of weightlessness. We obtained plasma before and after a parabolic flight campaign (PFC) in which nine participants experienced such varying gravity conditions ranging from 0 to ~1.8× *g* while flying multiple parabolas. Proximity Extension Assay (PEA; Olink®, Uppsala, Sweden), a proteomic technology that allows for the simultaneous and highly sensitive detection of a large number of plasma proteins, was used to analyze changes in circulating plasma proteins.

By providing a comprehensive understanding of hypergravity- and microgravity-induced changes in the plasma proteome, our study contributes valuable insights into the effects of gravity changes on the human body.

## Results

### Characteristics of study participants

The demographic data of the nine participating volunteers, consisting of seven males (78%) and two females (22%), with a median age of 35 years, is summarized in Table 1. All participants were in good physical condition, with no history of primary cardiovascular or respiratory diseases and no regular intake of medication, except for oral contraceptives.

**Table 1:** Basic epidemiological data of the participating test volunteers

Subject ID	Age (years)	Height (cm)	Weight (kg)	BMI (kg/m^2^)	Sex
1	46	190	105	29.1	M
2	31	190	81	22.4	M
3	35	185	82	24.0	M
4	31	195	90	23.7	M
5	56	160	68	26.6	M
6	29	159	54	21.4	F
7	35	177	78	24.9	M
8	43	176	92	29.7	M
9	30	168	52	18.4	F
Mean	35	177	81	24.0	7 M/2 F

*BMI* body mass index.

### Significant protein level differences across time points

**Fig. 1:**
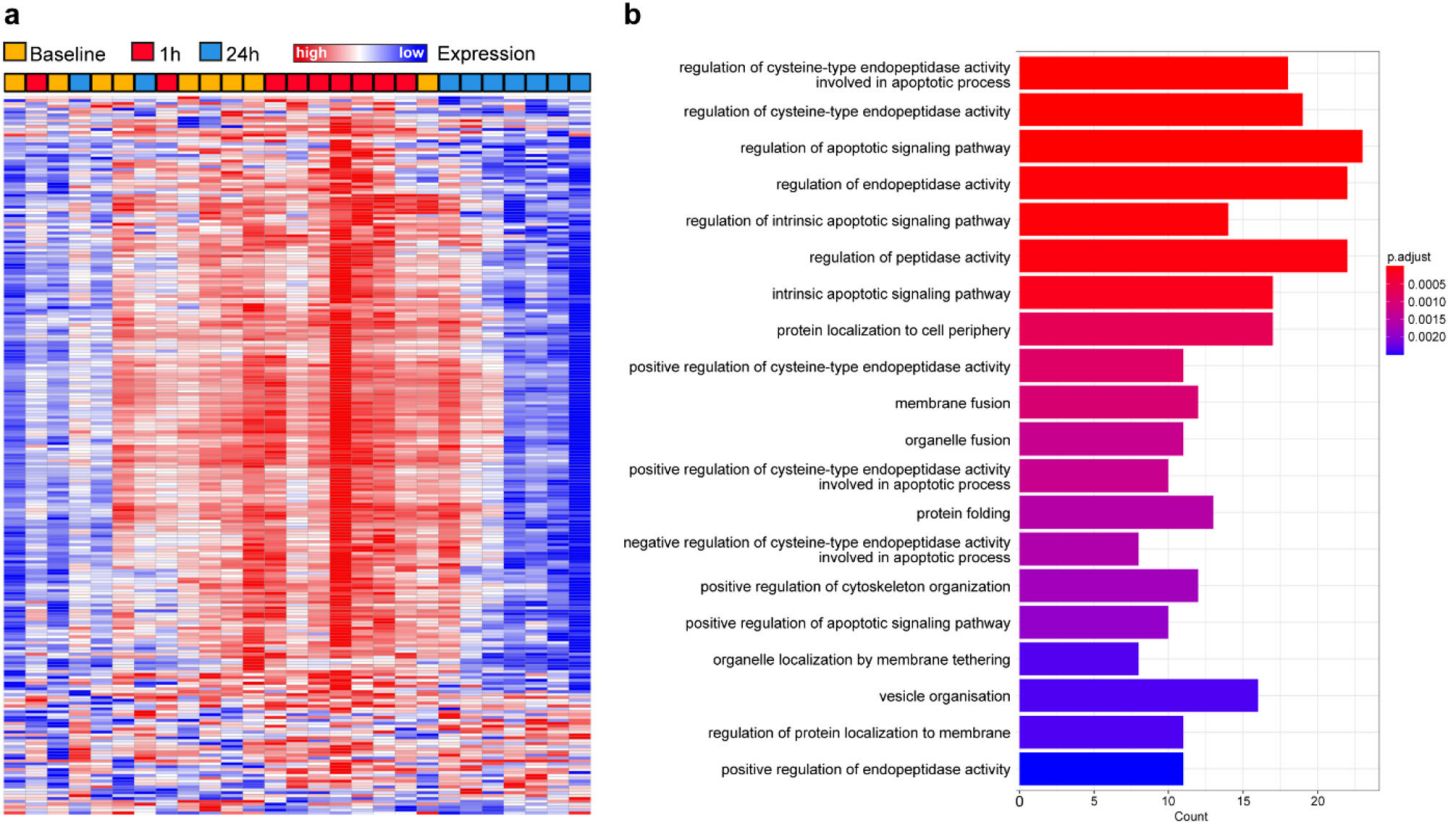
Differentially expressed proteins and their associated biological processes in response to varying gravity exposure. **a** Heatmap representation of protein levels of 200 out of 2950 proteins using the Olink proximity extension assay (PEA). Changes of plasma proteins were analyzed and visualized using the Morpheus software provided by the Broad Institute (https://software.broadinstitute.org/morpheus/). Each row represents a protein, and each column represents a sample. **b** Pathway analysis of differentially expressed proteins identified by one-way ANOVA (*n* = 251). The analysis was performed using the ClusterProfiler tool in R and focused on Gene Ontology (GO) terms associated with biological processes. The plot displays the top enriched biological processes, with the *x* axis representing the number of significant proteins involved in each process and the *y* axis showing the corresponding GO terms.

In this study, 2925 unique proteins were identified in plasma samples using the PEA method (see Supplementary Table 1). Out of these proteins, 251 (8.58%) exhibited significant differences in mean levels across the three time points (baseline, 1 h, and 24 h post flight), without isolating specific groups for analysis. The subsequent data processing and analysis produced a marker heatmap, revealing distinct plasma protein pattern changes across the time points (Fig. 1a). The pathway enrichment analysis of these significantly altered proteins suggested a time-dependent impact of varying gravity conditions on various protein sets, notably those involved in apoptosis, protein localization to the cell periphery, and vesicle organization (Fig. 1b and Supplementary Table 2).

### Detailed examination of individual protein changes

**Fig. 2:**
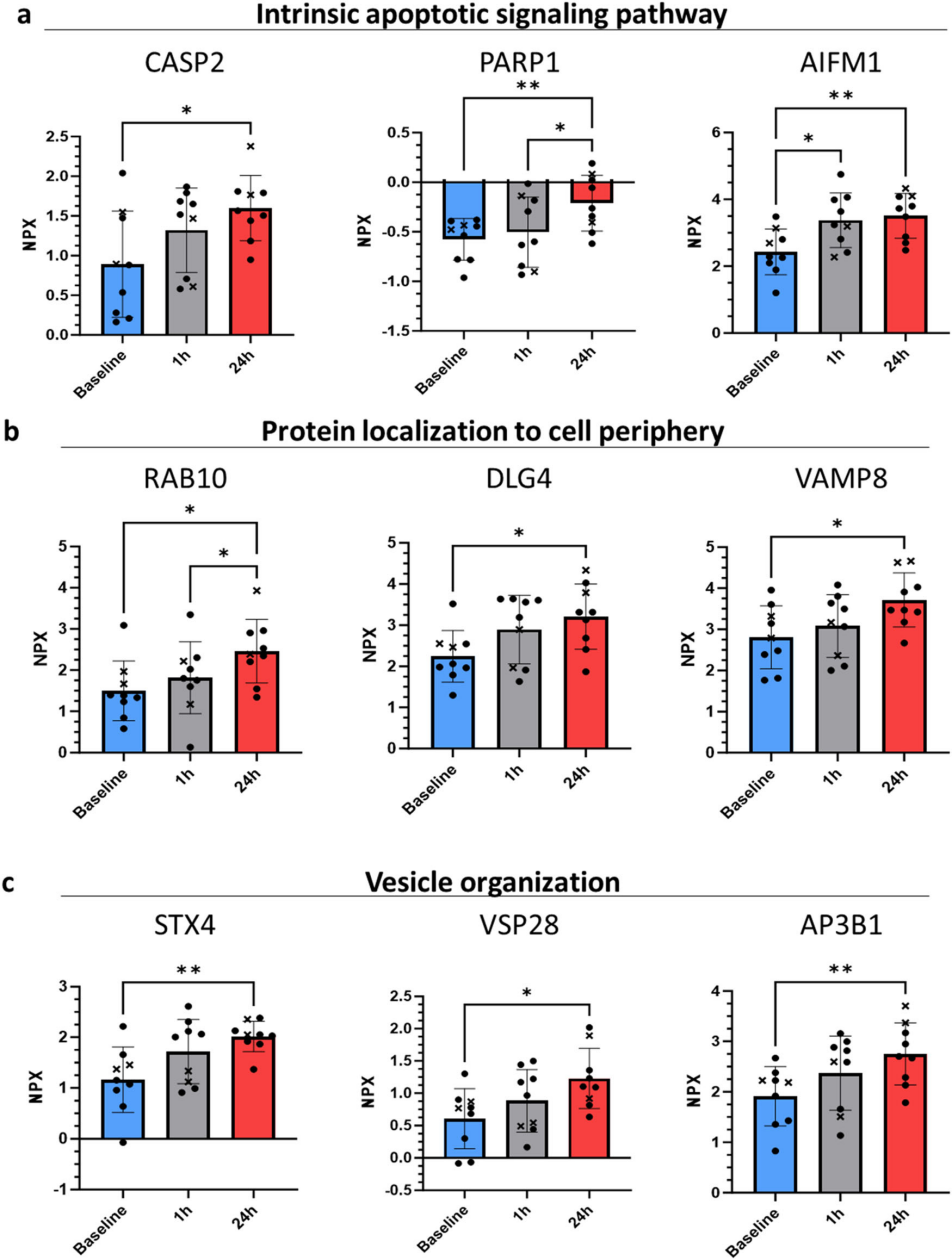
Plasma concentration of proteins associated with intrinsic apoptotic signaling pathway, protein localization to the cell periphery, and vesicle organization in response to varying gravity exposure. **a** Relative abundance of three proteins (CASP2, PARP1, AIFM1) involved in the intrinsic apoptotic signaling pathway. **b** Levels of three proteins (RAB10, DLG4, VAMP8) associated with protein localization to cell periphery. **c** Relative abundance of three proteins (STX4, VPS28, AP3B1) involved in vesicle organization. Values are presented as NPX (Normalized Protein eXpression), which is Olink’s arbitrary unit in Log2 scale. To differentiate by sex, the two female participants are marked with an “x”. Statistical analysis was performed using paired one-way ANOVA. Error bars indicate standard deviation (s.d.). Significance levels are indicated by asterisks: *P < 0.05, **P < 0.01.

We then focused on these pathways for a detailed examination of individual protein changes between the three time points, as they represent typical tissue responses to environmental stress. We isolated and analyzed three proteins per pathway to investigate the specific changes within the pathways. For the “intrinsic apoptotic pathways,” we focused on caspase 2 (CASP2), poly (ADP-ribose) polymerase 1 (PARP1), and apoptosis-inducing factor, mitochondria-associated 1 (AIFM1) (Fig. 2a). These proteins play a role in apoptosis but have distinct functions. CASP2 facilitates apoptosis by cleaving and activating other caspases, PARP1 induces apoptosis through protein cleavage, and AIFM1 triggers apoptosis by releasing specific mediators from the mitochondria. We observed significant alterations in proteins associated with protein localization to the cell periphery. Specifically, we found that Ras-related protein Rab-10 (RAB10), Disks large homolog 4 (DLG4), and Vesicle-associated membrane protein 8 (VAMP8) were significantly upregulated at 24 h post flight compared to baseline measurements (Fig. 2b). While there was no significant change in these proteins at 1 h post flight, the trend observed suggests a gradual increase of protein levels, potentially indicating a time-dependent response to exposure of varying gravity conditions. The “vesicle organization” pathway was investigated using the proteins adapter related protein complex 3 subunit beta-1 (AP3B1), Vacuolar protein sorting-associated protein 28 homolog (VPS28), and Syntaxin 4 (STX4) (Fig. 2c). The proteins AP3B1, VPS28, and STX4 are all involved in vesicle-mediated protein secretion and have diverse roles in vesicle structure. AP3B1 aids in the sorting of proteins into the right vesicles, VPS28 is involved in endosomal sorting of cell surface receptors, and STX4 is involved in vesicle docking and fusing with their target membranes. As compared to baseline levels, all the proteins mentioned were considerably elevated 24 h after exposure to gravitational changes.

## Discussion

In this study, we observed significant changes in the levels of plasma proteins involved in apoptosis, protein localization to cell periphery, and vesicle organization in response to varying gravity exposure. The findings provide insights into the cellular pathways affected by gravitational changes and contribute to a comprehensive repository of protein analyses for further investigations.

One of the primary foci of our study was the apoptosis pathway. Apoptosis is a critical cellular process that regulates homeostasis and maintains tissue integrity. Microgravity can induce cellular stress, which might trigger apoptosis as a response to DNA damage or other dysfunctions^14,15^. Nguyen et al. demonstrated that microgravity could lead to mitochondrial dysfunction and oxidative stress, which subsequently triggers apoptosis^16^. In addition, Man and colleagues reported that exposure to simulated microgravity induced apoptosis in osteocytes, that could contribute to bone loss experienced by astronauts during spaceflights^17^. Others found that microgravity exposure resulted in the activation of apoptotic pathways in lymphocytes likely impacting immune function^14^. Our study revealed that proteins associated with the intrinsic apoptotic pathways, such as CASP2, PARP1, and AIFM1, were altered in response to hyper- and microgravity. This suggests that cells may undergo apoptotic processes as a response to the stresses experienced in microgravity and varying gravity conditions in general. It is worth noting that these alterations in apoptotic proteins may vary across different cell types and tissues, with specific implications for each system. For example, increased apoptosis in immune cells might lead to compromised immune function, whereas increased apoptosis in musculoskeletal cells could result in muscle atrophy or bone loss^14,18^.

In addition to apoptosis, our study also investigated the impact of varying gravity conditions on protein localization to cell periphery. This is crucial for various cellular processes, including cell signaling, adhesion, and direct ligand-receptor communication^19^. Microgravity exposure can influence cellular architecture and organization, potentially affecting the overall health of astronauts during space missions^20^. In addition, microgravity conditions can alter protein distribution and localization, potentially leading to changes in cell behavior and function^21^. We discovered substantial changes in proteins involved in protein localization to the cell periphery, notably RAB10, DLG4, and VAMP8. As compared to baseline data, these proteins were considerably elevated at 24 h post flight, with a trend indicating a progressive rise of the protein levels. This data suggests that regulation of the protein localization to the cell periphery may be time-dependent. The observed changes in these plasma protein levels indicate that varying gravity conditions can impact cellular processes involving protein localization to the cell periphery. It is possible that cells adapt to varying gravity conditions by altering the expression of proteins which are involved in vesicle transport, signaling, and cell communication^22^. Further research is needed to elucidate the molecular mechanisms underlying these changes and determine the functional implications of these alterations in plasma proteins. Investigating the interplay between these proteins and other cellular pathways will provide valuable insights into how cells adapt to and function in an environment of varying gravity conditions.

In addition to investigating the impact of varying gravity conditions on vesicle organization, it is critical to evaluate the larger implications of these results on cellular and organismal processes. Vesicle architecture, particularly the synthesis and secretion of extracellular vesicles (EVs), is critical for cellular homeostasis, regulation of immune response, and the transfer of biomolecules (e.g., proteins, lipids, and nucleic acids) across cells^23,24^. Changes in vesicle organization and secretion under varying gravity conditions might have a substantial influence on a variety of physiological functions. Impaired vesicle-mediated transport of signaling molecules, such as cytokines and growth factors, might disrupt intercellular communication and jeopardize tissue repair and regeneration processes^25^. In addition, changes in EV-mediated transport of miRNAs and other regulatory molecules might lead to alterations in gene expression and cellular function, potentially contributing to the development of various pathological conditions^26^. The upregulation of vesicle organization proteins observed in our study may reflect a cellular compensatory mechanism in response to microgravity/ hypergravity-induced stress. It is possible that cells boost vesicle formation and secretion in response to the particular challenges posed by the microgravity/ hypergravity environment to maintain intercellular communication, facilitate tissue repair, and preserve homeostasis^27^. However, further studies will have to determine the exact processes driving the observed alterations in vesicle architecture and their functional implications.

While studying the biological impacts of gravitational variations, we must consider the possible influence of the substantial gravity shifts observed during parabolic flights. At these times, the body is subjected to forces of around 1.8× *g*, a significant change from microgravity circumstances. This might cause the organism to go into stress mode, affecting physiological processes and perhaps contributing to the alterations in protein levels found in our study. This hypergravity phase might stress cellular processes further, potentially changing the expression of proteins involved in apoptosis, vesicle architecture, and protein localization. Significant changes in gravitational forces may elicit cellular stress responses like reactive oxygen species (ROS) generation, that can drive apoptosis or changes in protein localization to the cell periphery^28^. As a result, the observed protein level changes may reflect physiological adaptations to occasional hypergravity exposure as well as a response to microgravity settings. This argument underlines the difficulties in assessing physiological responses during parabolic flying. Future studies should attempt to separate the effects of microgravity from the probable stress reactions caused by the alternating gravity circumstances encountered during parabolic flight. One of the constraints we faced was the sample size. Conducting experiments on parabolic flights presents logistical challenges, such as limited space and high demand among scientists for flight opportunities. While we aimed to increase the number of participants, we maximized our current resources to secure this valuable dataset for this study. Our goal is to set this dataset as a benchmark, allowing further findings or validating specific analytes in broader populations. Parabolic flights have been a long-standing method to investigate the effects of microgravity on the human body. Although there are no ideal alternatives for human research in microgravity, our focused approach on the proteome analysis offers a pioneering perspective, shedding light on the cellular and molecular changes that transpire during microgravity exposure. Such research would provide an even more nuanced understanding of how gravitational changes impact human physiology and help address the potential health risks tied to space travel. However, models that can distinctly differentiate between these contrasting gravitational effects are yet to be developed.

Due to the exploratory nature of this study, only a small sample size was investigated. However, the proteomic analysis revealed that the responses of the two female test candidates were consistent with those of the male candidates. This finding suggests that the effects of microgravity and hypergravity described in our study underly to a low gender difference. During parabolic flights, the time of induced microgravity is limited and we cannot distinguish conclusively whether microgravity or hypergravity may have induced the documented changes of protein levels. Moreover, it is important to note that this dataset only captures the initial response to gravitational alterations, and we must consider the possibility that some of the observed changes could be attributed to increased stress levels experienced during the flights. Finally, the microgravital environment in space is unique, and can only partially be mimicked by parabolic flights. Our findings emphasize the potential consequences of gravitational changes on physiological processes such as apoptosis, cell membrane location, and vesicle organization. The alterations in plasma proteins in these pathways highlight the complexity of cellular responses to changing gravitational circumstances. Using PEA technology, we provide a high-parametric analysis of changes in circulating plasma proteins in response to parabolic flight-induced gravity variations.

## Methods

### Study population

This observational study examined nine healthy volunteers (seven male (78%) and two female (22%)), median age 35 years). Before participation in the study, a medical examination confirmed the airworthiness of all participants. Inclusion criteria were defined as age >18 years, certified airworthiness, cardiorespiratory health (with blood pressure 140–90/90–60 mmHg and heart rate 50–100 bpm at rest), and written informed consent. The exclusion criteria were defined as age <18 years, history of cardiovascular and/or respiratory primary diseases or regular intake of medication, except for oral contraceptives, missing or withdrawal of informed consent, insufficient requirements for airworthiness, positive pregnancy test, or ametropia > ± 8 diopters. Study participants were not asked for any lifestyle restrictions. Anyway, due to the possibility of motion sickness during the parabolic flights, none of the subjects consumed any alcohol or other inebriant substances on the flight day or the day before. The study was conducted in accordance with the Declaration of Helsinki and approved by the Ethics Committee. All subjects provided written informed consent prior to inclusion.

### Institutional Review Board statement

The study was conducted in accordance with the Declaration of Helsinki, and the study protocol was approved by the Ethics Committee of the Université de Caen (#2020-107) and Comite de protection des personnes Ile de France (2023-A00517-38) and the Medical Faculty of the University Hospital Duesseldorf, Germany (Date of approval: July 29, 2020; Project Identification code: 2020-929).

### Informed consent statement

All subjects provided written informed consent prior to participation.

### Parabolic flights

The study was conducted as part of the 36th PFC organized by the German Aerospace Centre [Deutsches Zentrum für Luft-und Raumfahrt (DLR)] in June 2021. On each flight day, 31 parabolic flight maneuvers were performed. The experiments took place on board a specifically equipped Airbus A310 aircraft, operated by the company Novespace (Bordeaux, France) and flown by three well-trained pilots. The pilots operated simultaneously in the cockpit, and each of them was responsible for one space dimension (i.e., speed (thrust), vertical movement (pitch), and horizontal movement (roll)).

During the parabolic flight maneuvers, varying states of gravity could be experienced. These gravity states included regular earth gravity (1 ×*g*, “steady flight”), which is followed by a state of hypergravity (1.5–1.8× *g*, “pull-up”). By throttling back the engine force, a “free fall” induced a state of microgravity (~0× *g*) for the duration of ~22 s. The phase of microgravity was followed by a “pull-out” phase (1.5–1.8× *g*), during which the pilots steer the aircraft back to its horizontal flight path, again resulting in regular gravity (1 ×*g*). Because of slightly differing durations of the afore-mentioned phases, a protocol of the exact durations of the phases as well as the exact G-forces were provided by Novespace after the PFC. The first parabola on each flight day was a test run for all research teams aboard. After this test run, the individual experiments were commenced following each researcher´s study protocol^29^. This means that in our experimental design, each test participant encountered 31 parabolas.

### Blood sampling

To account for circadian variations, blood was drawn at the same time of the day. Baseline samples were obtained before the flight day at ~14.00–16.00 h. After landing, blood samples were collected again one hour (h) and 24 h post flight (again at ~14.00–16.00 h each). EDTA-anticoagulated blood (BD Vacutainer®) was immediately centrifuged (500×*g*; room temperature), and plasma was stored for later analysis at −80 °C.

### Proximity extension assay (PEA)

The plasma proteome profile (>3000 proteins) was analyzed in plasma (Citrate) samples using the Proximity Extension Assay (PEA) by Olink (Olink® Explore 3072; Uppsala, Sweden) to discover differences in the response to the gravitational changes. Briefly, in PEA, two different antigen-specific antibodies bind to the target protein bringing them in spatial proximity. The antibodies are conjugated to DNA oligonucleotides that contain complementary sequence segments allowing hybridize and serve as templates for polymerase chain reaction (PCR)-based amplification and subsequent readout by NGS.

Raw data were transformed to the normalized protein levels (NPX) by Olink for statistical ANOVA analysis with the R library rstatix (https://rpkgs.datanovia.com/rstatix/).

### Pathway analysis

Pathway analysis was conducted on proteins exhibiting statistically significant alterations, as determined through ANOVA analysis, utilizing R Version 4.2 and the library “ClusterProfiler”^30^ and the Gene Ontology Database “Biological Process”^31^.

### Heatmap

This graphical representation of the data was generated using the software Morpheus, a versatile online tool developed by the Broad Institute for the visualization and analysis of matrix-based data (https://software.broadinstitute.org/morpheus/).

### Statistics

Analysis of variance ANOVA Type III in R (version 4.1.3) using the “Anova” function from the “car” package (version 3.1) was performed to find significant differences in the dataset among the three time points. The analysis was carried out on the entire dataset, with the *P* value threshold for significance set at 0.05. The significantly regulated proteins were used to characterize associated pathways via ClusterProfiler (version 4.6.2). Statistics for selected proteins were calculated via multiple comparisons by paired one-way ANOVA followed by paired Tukey’s post hoc test to identify group differences in variance analysis using the GraphPad Prism software v10 and the R package rstatix version 0.7.0. Results with *P* value ≤ 0.05 were considered significant.

### Reporting summary

Further information on research design is available in the Nature Research Reporting Summary linked to this article.

### Supplementary information

Reporting Summary

Supplementary information

## Data Availability

Data are available at 10.5281/zenodo.10653443.

## Code availability

Available from the corresponding author upon reasonable request.
