# Supplementary material for: Acute and short-term fluctuations in gravity are associated with changes in circulatory plasma protein levels
Source: NPJ Microgravity. 2024 Mar 4;10:25. doi: 10.1038/s41526-024-00370-y (PMC10912449; doi:10.1038/s41526-024-00370-y)
Supplement: Supplementary file 2 — Supplementary information [file 41526_2024_370_MOESM2_ESM.pdf]

Supplementary information to

Acute and short-term microgravity-induced changes in circulatory plasma protein levels

Alexander Lang[1*], Stephan Binneboessel[1*], Fabian Nienhaus[1], Raphael Romano Bruno[1], Georg Wolff[1], Kerstin Piayda[2], Susanne Pfeiler[1], Hakima Ezzahoini[1], Daniel Oehler[1], Malte Kelm[1,3], Holger Winkels[4], Norbert Gerdes[1,3#], Christian Jung[1,3#]

[1]Division of Cardiology, Pulmonology, and Vascular Medicine, University Hospital and Medical Faculty, Heinrich-Heine University, Düsseldorf, Germany

[2]Medical Faculty, Department of Cardiology and Vascular Medicine, Justus-Liebig-University Giessen, Germany

[3]Cardiovascular Research Institute Düsseldorf (CARID), Medical Faculty, Heinrich-Heine University, Düsseldorf, Germany

[4]Clinic III for Experimental Cardiology, University Hospital of Cologne, Germany

[*,#] These authors contributed equally to this study

Corresponding author: Christian Jung, M.D. PhD, email: christian.jung@med.uni-duesseldorf.de, 𝕏: @cjungMD

This PDF file includes:

Supplemental table 1_Olink proximity extension assay (PEA) data (page 2-711)

Supplemental table 2_Pathway enrichment analysis (page 712- 839)

Supplemental table 1_Olink proximity extension assay (PEA) data

,"SampleID","Index","OlinkID","UniProt","Assay","MissingFreq","Panel","Panel_Lot_Nr","PlateID","QC_Warning","LOD","NPX","Normalization","Assay_Warning","condition","Value","group","alt_group"
1,"PFC_01",1,"OID30049","Q8IZC4","RTKN2",0.9545,"Cardiometabolic_II","B13805","20212097_SS211647","WARN",0.6387,-0.2763,"Plate control","PASS","PFC",0.825705946357795,"Baseline","Baseline"
2,"PFC_09",2,"OID30049","Q8IZC4","RTKN2",0.9545,"Cardiometabolic_II","B13805","20212097_SS211647","PASS",0.6387,-0.4805,"Plate control","PASS","PFC",0.716729181552894,"Baseline","Baseline"
3,"PFC_17",3,"OID30049","Q8IZC4","RTKN2",0.9545,"Cardiometabolic_II","B13805","20212097_SS211647","PASS",0.6387,0.1074,"Plate control","PASS","PFC",1.07728502162025,"1h","1h"
4,"PFC_25",4,"OID30049","Q8IZC4","RTKN2",0.9545,"Cardiometabolic_II","B13805","20212097_SS211647","PASS",0.6387,0.0305,"Plate control","PASS","PFC",1.02136604287095,"24h","24h"
5,"PFC_02",13,"OID30049","Q8IZC4","RTKN2",0.9545,"Cardiometabolic_II","B13805","20212097_SS211647","PASS",0.6387,-0.244,"Plate control","PASS","PFC",0.844400887423782,"Baseline","Baseline"
6,"PFC_10",14,"OID30049","Q8IZC4","RTKN2",0.9545,"Cardiometabolic_II","B13805","20212097_SS211647","PASS",0.6387,-0.9643,"Plate control","PASS","PFC",0.512527030862576,"1h","1h"
7,"PFC_18",15,"OID30049","Q8IZC4","RTKN2",0.9545,"Cardiometabolic_II","B13805","20212097_SS211647","PASS",0.6387,-0.0057,"Plate control","PASS","PFC",0.996056855761192,"1h","1h"
8,"PFC_26",16,"OID30049","Q8IZC4","RTKN2",0.9545,"Cardiometabolic_II","B13805","20212097_SS211647","PASS",0.6387,-0.4196,"Plate control","PASS","PFC",0.747631883156728,"24h","24h"
9,"PFC_03",25,"OID30049","Q8IZC4","RTKN2",0.9545,"Cardiometabolic_II","B13805","20212097_SS211647","PASS",0.6387,-0.3599,"Plate control","PASS","PFC",0.779218589105461,"Baseline","Baseline"
10,"PFC_11",26,"OID30049","Q8IZC4","RTKN2",0.9545,"Cardiometabolic_II","B13805","20212097_SS211647","PASS",0.6387,-0.771,"Plate control","PASS","PFC",0.586011141837257,"1h","1h"
11,"PFC_19",27,"OID30049","Q8IZC4","RTKN2",0.9545,"Cardiometabolic_II","B13805","20212097_SS211647","PASS",0.6387,0.2293,"Plate control","PASS","PFC",1.17226602419829,"24h","24h"
12,"PFC_27",28,"OID30049","Q8IZC4","RTKN2",0.9545,"Cardiometabolic_II","B13805","20212097_SS211647","PASS",0.6387,0.3124,"Plate control","PASS","PFC",1.24177173603258,"24h","24h"
13,"PFC_04",37,"OID30049","Q8IZC4","RTKN2",0.9545,"Cardiometabolic_II","B13805","20212097_SS211647","PASS",0.6387,-0.4459,"Plate control","PASS","PFC",0.734126202004236,"Baseline","Baseline"
14,"PFC_12",38,"OID30049","Q8IZC4","RTKN2",0.9545,"Cardiometabolic_II","B13805","20212097_SS211647","PASS",0.6387,0.1463,"Plate control","PASS","PFC",1.10672746667301,"1h","1h"
15,"PFC_20",39,"OID30049","Q8IZC4","RTKN2",0.9545,"Cardiometabolic_II","B13805","20212097_SS211647","PASS",0.6387,-1.0543,"Plate control","PASS","PFC",0.481530805082502,"24h","24h"
16,"PFC_05",49,"OID30049","Q8IZC4","RTKN2",0.9545,"Cardiometabolic_II","B13805","20212097_SS211647","PASS",0.6387,0.0074,"Plate control","PASS","PFC",1.00514246646013,"Baseline","Baseline"
17,"PFC_13",50,"OID30049","Q8IZC4","RTKN2",0.9545,"Cardiometabolic_II","B13805","20212097_SS211647","PASS",0.6387,0.5254,"Plate control","PASS","PFC",1.43933259242473,"1h","1h"
18,"PFC_21",51,"OID30049","Q8IZC4","RTKN2",0.9545,"Cardiometabolic_II","B13805","20212097_SS211647","PASS",0.6387,-0.5796,"Plate control","PASS","PFC",0.669149279250364,"24h","24h"
19,"PFC_06",61,"OID30049","Q8IZC4","RTKN2",0.9545,"Cardiometabolic_II","B13805","20212097_SS211647","PASS",0.6387,0.6191,"Plate control","PASS","PFC",1.53591672764744,"Baseline","Baseline"
20,"PFC_14",62,"OID30049","Q8IZC4","RTKN2",0.9545,"Cardiometabolic_II","B13805","20212097_SS211647","PASS",0.6387,0.2369,"Plate control","PASS","PFC",1.17845772051601,"1h","1h"
21,"PFC_22",63,"OID30049","Q8IZC4","RTKN2",0.9545,"Cardiometabolic_II","B13805","20212097_SS211647","PASS",0.6387,-0.0391,"Plate control","PASS","PFC",0.973261910445234,"24h","24h"
22,"PFC_07",73,"OID30049","Q8IZC4","RTKN2",0.9545,"Cardiometabolic_II","B13805","20212097_SS211647","PASS",0.6387,-0.3692,"Plate control","PASS","PFC",0.774211691883153,"Baseline","Baseline"
23,"PFC_15",74,"OID30049","Q8IZC4","RTKN2",0.9545,"Cardiometabolic_II","B13805","20212097_SS211647","PASS",0.6387,-0.3656,"Plate control","PASS","PFC",0.776146017815058,"1h","1h"
24,"PFC_23",75,"OID30049","Q8IZC4","RTKN2",0.9545,"Cardiometabolic_II","B13805","20212097_SS211647","PASS",0.6387,-0.3604,"Plate control","PASS","PFC",0.778948579313256,"24h","24h"
25,"PFC_08",85,"OID30049","Q8IZC4","RTKN2",0.9545,"Cardiometabolic_II","B13805","20212097_SS211647","PASS",0.6387,-1.0479,"Plate control","PASS","PFC",0.483671689211855,"Baseline","Baseline"
26,"PFC_16",86,"OID30049","Q8IZC4","RTKN2",0.9545,"Cardiometabolic_II","B13805","20212097_SS211647","PASS",0.6387,-0.1363,"Plate control","PASS","PFC",0.909849606501392,"1h","1h"
27,"PFC_24",87,"OID30049","Q8IZC4","RTKN2",0.9545,"Cardiometabolic_II","B13805","20212097_SS211647","PASS",0.6387,0.6542,"Plate control","PASS","PFC",1.57374304269623,"24h","24h"
28,"PFC_01",1,"OID30050","P78524","DENND2B",0.2727,"Cardiometabolic_II","B13805","20212097_SS211647","WARN",-0.6759,-0.3403,"Plate control","PASS","PFC",0.789877044470838,"Baseline","Baseline"
29,"PFC_09",2,"OID30050","P78524","DENND2B",0.2727,"Cardiometabolic_II","B13805","20212097_SS211647","PASS",-0.6759,-0.9352,"Plate control","PASS","PFC",0.522969965839315,"Baseline","Baseline"
30,"PFC_17",3,"OID30050","P78524","DENND2B",0.2727,"Cardiometabolic_II","B13805","20212097_SS211647","PASS",-0.6759,-0.3211,"Plate control","PASS","PFC",0.800459325120907,"1h","1h"
31,"PFC_25",4,"OID30050","P78524","DENND2B",0.2727,"Cardiometabolic_II","B13805","20212097_SS211647","PASS",-0.6759,-0.1663,"Plate control","PASS","PFC",0.891125172358942,"24h","24h"
32,"PFC_02",13,"OID30050","P78524","DENND2B",0.2727,"Cardiometabolic_II","B13805","20212097_SS211647","PASS",-0.6759,-0.1811,"Plate control","PASS","PFC",0.882030225401867,"Baseline","Baseline"
33,"PFC_10",14,"OID30050","P78524","DENND2B",0.2727,"Cardiometabolic_II","B13805","20212097_SS211647","PASS",-0.6759,-0.9459,"Plate control","PASS","PFC",0.51910561568392,"1h","1h"
34,"PFC_18",15,"OID30050","P78524","DENND2B",0.2727,"Cardiometabolic_II","B13805","20212097_SS211647","PASS",-0.6759,-0.3639,"Plate control","PASS","PFC",0.777061128690433,"1h","1h"
35,"PFC_26",16,"OID30050","P78524","DENND2B",0.2727,"Cardiometabolic_II","B13805","20212097_SS211647","PASS",-0.6759,-0.4511,"Plate control","PASS","PFC",0.731484905924886,"24h","24h"
36,"PFC_03",25,"OID30050","P78524","DENND2B",0.2727,"Cardiometabolic_II","B13805","20212097_SS211647","PASS",-0.6759,-0.6522,"Plate control","PASS","PFC",0.636309250330789,"Baseline","Baseline"
37,"PFC_11",26,"OID30050","P78524","DENND2B",0.2727,"Cardiometabolic_II","B13805","20212097_SS211647","PASS",-0.6759,-0.4476,"Plate control","PASS","PFC",0.733261653712541,"1h","1h"
38,"PFC_19",27,"OID30050","P78524","DENND2B",0.2727,"Cardiometabolic_II","B13805","20212097_SS211647","PASS",-0.6759,0.0814,"Plate control","PASS","PFC",1.05804427511742,"24h","24h"
39,"PFC_27",28,"OID30050","P78524","DENND2B",0.2727,"Cardiometabolic_II","B13805","20212097_SS211647","PASS",-0.6759,-0.108,"Plate control","PASS","PFC",0.927873476471283,"24h","24h"
40,"PFC_04",37,"OID30050","P78524","DENND2B",0.2727,"Cardiometabolic_II","B13805","20212097_SS211647","PASS",-0.6759,-0.9325,"Plate control","PASS","PFC",0.523949619187535,"Baseline","Baseline"
41,"PFC_12",38,"OID30050","P78524","DENND2B",0.2727,"Cardiometabolic_II","B13805","20212097_SS211647","PASS",-0.6759,-0.2499,"Plate control","PASS","PFC",0.840954703771755,"1h","1h"
42,"PFC_20",39,"OID30050","P78524","DENND2B",0.2727,"Cardiometabolic_II","B13805","20212097_SS211647","PASS",-0.6759,0.0919,"Plate control","PASS","PFC",1.06577285976233,"24h","24h"
43,"PFC_05",49,"OID30050","P78524","DENND2B",0.2727,"Cardiometabolic_II","B13805","20212097_SS211647","PASS",-0.6759,-0.4118,"Plate control","PASS","PFC",0.751684937469144,"Baseline","Baseline"
44,"PFC_13",50,"OID30050","P78524","DENND2B",0.2727,"Cardiometabolic_II","B13805","20212097_SS211647","PASS",-0.6759,-0.8841,"Plate control","PASS","PFC",0.541825425644138,"1h","1h"
45,"PFC_21",51,"OID30050","P78524","DENND2B",0.2727,"Cardiometabolic_II","B13805","20212097_SS211647","PASS",-0.6759,-0.2413,"Plate control","PASS","PFC",0.845982661159635,"24h","24h"
46,"PFC_06",61,"OID30050","P78524","DENND2B",0.2727,"Cardiometabolic_II","B13805","20212097_SS211647","PASS",-0.6759,-0.7005,"Plate control","PASS","PFC",0.615358902567635,"Baseline","Baseline"
47,"PFC_14",62,"OID30050","P78524","DENND2B",0.2727,"Cardiometabolic_II","B13805","20212097_SS211647","PASS",-0.6759,-0.1484,"Plate control","PASS","PFC",0.902250535814082,"1h","1h"
48,"PFC_22",63,"OID30050","P78524","DENND2B",0.2727,"Cardiometabolic_II","B13805","20212097_SS211647","PASS",-0.6759,-0.8404,"Plate control","PASS","PFC",0.558488701620358,"24h","24h"
49,"PFC_07",73,"OID30050","P78524","DENND2B",0.2727,"Cardiometabolic_II","B13805","20212097_SS211647","PASS",-0.6759,-0.3134,"Plate control","PASS","PFC",0.80474298455464,"Baseline","Baseline"
50,"PFC_15",74,"OID30050","P78524","DENND2B",0.2727,"Cardiometabolic_II","B13805","20212097_SS211647","PASS",-0.6759,-0.4533,"Plate control","PASS","PFC",0.730370297247165,"1h","1h"
51,"PFC_23",75,"OID30050","P78524","DENND2B",0.2727,"Cardiometabolic_II","B13805","20212097_SS211647","PASS",-0.6759,0.0947,"Plate control","PASS","PFC",1.06784333318036,"24h","24h"
52,"PFC_08",85,"OID30050","P78524","DENND2B",0.2727,"Cardiometabolic_II","B13805","20212097_SS211647","PASS",-0.6759,-0.6505,"Plate control","PASS","PFC",0.637059487401796,"Baseline","Baseline"
53,"PFC_16",86,"OID30050","P78524","DENND2B",0.2727,"Cardiometabolic_II","B13805","20212097_SS211647","PASS",-0.6759,-0.6475,"Plate control","PASS","PFC",0.6383855936668,"1h","1h"
54,"PFC_24",87,"OID30050","P78524","DENND2B",0.2727,"Cardiometabolic_II","B13805","20212097_SS211647","PASS",-0.6759,-0.5666,"Plate control","PASS","PFC",0.675206173513571,"24h","24h"
55,"PFC_01",1,"OID30051","Q9H2M3","BHMT2",0.9091,"Cardiometabolic_II","B13805","20212097_SS211647","WARN",0.3673,0.0543,"Plate control","PASS","PFC",1.03835516798211,"Baseline","Baseline"
56,"PFC_09",2,"OID30051","Q9H2M3","BHMT2",0.9091,"Cardiometabolic_II","B13805","20212097_SS211647","PASS",0.3673,0.0866,"Plate control","PASS","PFC",1.06186473425884,"Baseline","Baseline"
57,"PFC_17",3,"OID30051","Q9H2M3","BHMT2",0.9091,"Cardiometabolic_II","B13805","20212097_SS211647","PASS",0.3673,-0.0455,"Plate control","PASS","PFC",0.96895394488874,"1h","1h"
58,"PFC_25",4,"OID30051","Q9H2M3","BHMT2",0.9091,"Cardiometabolic_II","B13805","20212097_SS211647","PASS",0.3673,0.3008,"Plate control","PASS","PFC",1.23182729408534,"24h","24h"
59,"PFC_02",13,"OID30051","Q9H2M3","BHMT2",0.9091,"Cardiometabolic_II","B13805","20212097_SS211647","PASS",0.3673,0.1111,"Plate control","PASS","PFC",1.08005142069808,"Baseline","Baseline"
60,"PFC_10",14,"OID30051","Q9H2M3","BHMT2",0.9091,"Cardiometabolic_II","B13805","20212097_SS211647","PASS",0.3673,0.1011,"Plate control","PASS","PFC",1.07259096058138,"1h","1h"
61,"PFC_18",15,"OID30051","Q9H2M3","BHMT2",0.9091,"Cardiometabolic_II","B13805","20212097_SS211647","PASS",0.3673,-0.2246,"Plate control","PASS","PFC",0.8558322798834,"1h","1h"
62,"PFC_26",16,"OID30051","Q9H2M3","BHMT2",0.9091,"Cardiometabolic_II","B13805","20212097_SS211647","PASS",0.3673,0.0014,"Plate control","PASS","PFC",1.00097087704908,"24h","24h"
63,"PFC_03",25,"OID30051","Q9H2M3","BHMT2",0.9091,"Cardiometabolic_II","B13805","20212097_SS211647","PASS",0.3673,-0.7453,"Plate control","PASS","PFC",0.596543810819295,"Baseline","Baseline"
64,"PFC_11",26,"OID30051","Q9H2M3","BHMT2",0.9091,"Cardiometabolic_II","B13805","20212097_SS211647","PASS",0.3673,-0.4064,"Plate control","PASS","PFC",0.754503762395061,"1h","1h"
65,"PFC_19",27,"OID30051","Q9H2M3","BHMT2",0.9091,"Cardiometabolic_II","B13805","20212097_SS211647","PASS",0.3673,0.4417,"Plate control","PASS","PFC",1.35820382463652,"24h","24h"
66,"PFC_27",28,"OID30051","Q9H2M3","BHMT2",0.9091,"Cardiometabolic_II","B13805","20212097_SS211647","PASS",0.3673,0.4861,"Plate control","PASS","PFC",1.40065340341829,"24h","24h"
67,"PFC_04",37,"OID30051","Q9H2M3","BHMT2",0.9091,"Cardiometabolic_II","B13805","20212097_SS211647","PASS",0.3673,-0.1249,"Plate control","PASS","PFC",0.91706760728432,"Baseline","Baseline"
68,"PFC_12",38,"OID30051","Q9H2M3","BHMT2",0.9091,"Cardiometabolic_II","B13805","20212097_SS211647","PASS",0.3673,-0.3815,"Plate control","PASS","PFC",0.767639045323934,"1h","1h"
69,"PFC_20",39,"OID30051","Q9H2M3","BHMT2",0.9091,"Cardiometabolic_II","B13805","20212097_SS211647","PASS",0.3673,0.0389,"Plate control","PASS","PFC",1.0273302278059,"24h","24h"
70,"PFC_05",49,"OID30051","Q9H2M3","BHMT2",0.9091,"Cardiometabolic_II","B13805","20212097_SS211647","PASS",0.3673,0.1944,"Plate control","PASS","PFC",1.14424818218781,"Baseline","Baseline"
71,"PFC_13",50,"OID30051","Q9H2M3","BHMT2",0.9091,"Cardiometabolic_II","B13805","20212097_SS211647","PASS",0.3673,-0.0869,"Plate control","PASS","PFC",0.941543725117265,"1h","1h"
72,"PFC_21",51,"OID30051","Q9H2M3","BHMT2",0.9091,"Cardiometabolic_II","B13805","20212097_SS211647","PASS",0.3673,-0.1773,"Plate control","PASS","PFC",0.884356519441668,"24h","24h"
73,"PFC_06",61,"OID30051","Q9H2M3","BHMT2",0.9091,"Cardiometabolic_II","B13805","20212097_SS211647","PASS",0.3673,-0.075,"Plate control","PASS","PFC",0.949342120950519,"Baseline","Baseline"
74,"PFC_14",62,"OID30051","Q9H2M3","BHMT2",0.9091,"Cardiometabolic_II","B13805","20212097_SS211647","PASS",0.3673,-0.4047,"Plate control","PASS","PFC",0.755393356083144,"1h","1h"
75,"PFC_22",63,"OID30051","Q9H2M3","BHMT2",0.9091,"Cardiometabolic_II","B13805","20212097_SS211647","PASS",0.3673,-0.231,"Plate control","PASS","PFC",0.852044095074866,"24h","24h"
76,"PFC_07",73,"OID30051","Q9H2M3","BHMT2",0.9091,"Cardiometabolic_II","B13805","20212097_SS211647","PASS",0.3673,-0.1688,"Plate control","PASS","PFC",0.889582307283877,"Baseline","Baseline"
77,"PFC_15",74,"OID30051","Q9H2M3","BHMT2",0.9091,"Cardiometabolic_II","B13805","20212097_SS211647","PASS",0.3673,-0.6095,"Plate control","PASS","PFC",0.655423815155959,"1h","1h"
78,"PFC_23",75,"OID30051","Q9H2M3","BHMT2",0.9091,"Cardiometabolic_II","B13805","20212097_SS211647","PASS",0.3673,-0.1613,"Plate control","PASS","PFC",0.894218934847859,"24h","24h"
79,"PFC_08",85,"OID30051","Q9H2M3","BHMT2",0.9091,"Cardiometabolic_II","B13805","20212097_SS211647","PASS",0.3673,-0.3232,"Plate control","PASS","PFC",0.79929501685417,"Baseline","Baseline"
80,"PFC_16",86,"OID30051","Q9H2M3","BHMT2",0.9091,"Cardiometabolic_II","B13805","20212097_SS211647","PASS",0.3673,-0.345,"Plate control","PASS","PFC",0.787307976569203,"1h","1h"
81,"PFC_24",87,"OID30051","Q9H2M3","BHMT2",0.9091,"Cardiometabolic_II","B13805","20212097_SS211647","PASS",0.3673,-0.0911,"Plate control","PASS","PFC",0.938806671940363,"24h","24h"
82,"PFC_01",1,"OID30052","P55769","SNU13",0.75,"Cardiometabolic_II","B13805","20212097_SS211647","WARN",0.522,0.3818,"Plate control","PASS","PFC",1.30296650733025,"Baseline","Baseline"
83,"PFC_09",2,"OID30052","P55769","SNU13",0.75,"Cardiometabolic_II","B13805","20212097_SS211647","PASS",0.522,0.1388,"Plate control","PASS","PFC",1.10098895803959,"Baseline","Baseline"
84,"PFC_17",3,"OID30052","P55769","SNU13",0.75,"Cardiometabolic_II","B13805","20212097_SS211647","PASS",0.522,1.7907,"Plate control","PASS","PFC",3.45982723668367,"1h","1h"
85,"PFC_25",4,"OID30052","P55769","SNU13",0.75,"Cardiometabolic_II","B13805","20212097_SS211647","PASS",0.522,-0.0362,"Plate control","PASS","PFC",0.975220257921818,"24h","24h"
86,"PFC_02",13,"OID30052","P55769","SNU13",0.75,"Cardiometabolic_II","B13805","20212097_SS211647","PASS",0.522,0.3335,"Plate control","PASS","PFC",1.26006661008984,"Baseline","Baseline"
87,"PFC_10",14,"OID30052","P55769","SNU13",0.75,"Cardiometabolic_II","B13805","20212097_SS211647","PASS",0.522,0.7572,"Plate control","PASS","PFC",1.69020706503681,"1h","1h"
88,"PFC_18",15,"OID30052","P55769","SNU13",0.75,"Cardiometabolic_II","B13805","20212097_SS211647","PASS",0.522,-0.2033,"Plate control","PASS","PFC",0.868561554072981,"1h","1h"
89,"PFC_26",16,"OID30052","P55769","SNU13",0.75,"Cardiometabolic_II","B13805","20212097_SS211647","PASS",0.522,1.2705,"Plate control","PASS","PFC",2.41245160252782,"24h","24h"
90,"PFC_03",25,"OID30052","P55769","SNU13",0.75,"Cardiometabolic_II","B13805","20212097_SS211647","PASS",0.522,0.0387,"Plate control","PASS","PFC",1.02718781946694,"Baseline","Baseline"
91,"PFC_11",26,"OID30052","P55769","SNU13",0.75,"Cardiometabolic_II","B13805","20212097_SS211647","PASS",0.522,0.2856,"Plate control","PASS","PFC",1.21891709164322,"1h","1h"
92,"PFC_19",27,"OID30052","P55769","SNU13",0.75,"Cardiometabolic_II","B13805","20212097_SS211647","PASS",0.522,0.6432,"Plate control","PASS","PFC",1.56178948020311,"24h","24h"
93,"PFC_27",28,"OID30052","P55769","SNU13",0.75,"Cardiometabolic_II","B13805","20212097_SS211647","PASS",0.522,0.2698,"Plate control","PASS","PFC",1.20564067881769,"24h","24h"
94,"PFC_04",37,"OID30052","P55769","SNU13",0.75,"Cardiometabolic_II","B13805","20212097_SS211647","PASS",0.522,0.4234,"Plate control","PASS","PFC",1.34108436757817,"Baseline","Baseline"
95,"PFC_12",38,"OID30052","P55769","SNU13",0.75,"Cardiometabolic_II","B13805","20212097_SS211647","PASS",0.522,0.3407,"Plate control","PASS","PFC",1.26637089191791,"1h","1h"
96,"PFC_20",39,"OID30052","P55769","SNU13",0.75,"Cardiometabolic_II","B13805","20212097_SS211647","PASS",0.522,0.8058,"Plate control","PASS","PFC",1.74811488384162,"24h","24h"
97,"PFC_05",49,"OID30052","P55769","SNU13",0.75,"Cardiometabolic_II","B13805","20212097_SS211647","PASS",0.522,0.2061,"Plate control","PASS","PFC",1.15356556136056,"Baseline","Baseline"
98,"PFC_13",50,"OID30052","P55769","SNU13",0.75,"Cardiometabolic_II","B13805","20212097_SS211647","PASS",0.522,0.2005,"Plate control","PASS","PFC",1.14909653250499,"1h","1h"
99,"PFC_21",51,"OID30052","P55769","SNU13",0.75,"Cardiometabolic_II","B13805","20212097_SS211647","PASS",0.522,-0.1761,"Plate control","PASS","PFC",0.885092412522242,"24h","24h"
100,"PFC_06",61,"OID30052","P55769","SNU13",0.75,"Cardiometabolic_II","B13805","20212097_SS211647","PASS",0.522,-0.1914,"Plate control","PASS","PFC",0.875755470429191,"Baseline","Baseline"
101,"PFC_14",62,"OID30052","P55769","SNU13",0.75,"Cardiometabolic_II","B13805","20212097_SS211647","PASS",0.522,0.6827,"Plate control","PASS","PFC",1.60514096304585,"1h","1h"
102,"PFC_22",63,"OID30052","P55769","SNU13",0.75,"Cardiometabolic_II","B13805","20212097_SS211647","PASS",0.522,0.2181,"Plate control","PASS","PFC",1.16320066567978,"24h","24h"
103,"PFC_07",73,"OID30052","P55769","SNU13",0.75,"Cardiometabolic_II","B13805","20212097_SS211647","PASS",0.522,0.6122,"Plate control","PASS","PFC",1.52858841342735,"Baseline","Baseline"
104,"PFC_15",74,"OID30052","P55769","SNU13",0.75,"Cardiometabolic_II","B13805","20212097_SS211647","PASS",0.522,-0.3781,"Plate control","PASS","PFC",0.769450274003164,"1h","1h"
105,"PFC_23",75,"OID30052","P55769","SNU13",0.75,"Cardiometabolic_II","B13805","20212097_SS211647","PASS",0.522,0.7509,"Plate control","PASS","PFC",1.68284231478745,"24h","24h"
106,"PFC_08",85,"OID30052","P55769","SNU13",0.75,"Cardiometabolic_II","B13805","20212097_SS211647","PASS",0.522,1.6378,"Plate control","PASS","PFC",3.11190927408987,"Baseline","Baseline"
107,"PFC_16",86,"OID30052","P55769","SNU13",0.75,"Cardiometabolic_II","B13805","20212097_SS211647","PASS",0.522,0.4055,"Plate control","PASS","PFC",1.32454788417242,"1h","1h"
108,"PFC_24",87,"OID30052","P55769","SNU13",0.75,"Cardiometabolic_II","B13805","20212097_SS211647","PASS",0.522,0.3256,"Plate control","PASS","PFC",1.25318551546579,"24h","24h"
109,"PFC_01",1,"OID30053","Q9Y2W1","THRAP3",0.2727,"Cardiometabolic_II","B13805","20212097_SS211647","WARN",-0.2157,1.5125,"Plate control","PASS","PFC",2.85304005173806,"Baseline","Baseline"
110,"PFC_09",2,"OID30053","Q9Y2W1","THRAP3",0.2727,"Cardiometabolic_II","B13805","20212097_SS211647","PASS",-0.2157,-0.3854,"Plate control","PASS","PFC",0.765566708956628,"Baseline","Baseline"
111,"PFC_17",3,"OID30053","Q9Y2W1","THRAP3",0.2727,"Cardiometabolic_II","B13805","20212097_SS211647","PASS",-0.2157,0.97,"Plate control","PASS","PFC",1.95884059517385,"1h","1h"
112,"PFC_25",4,"OID30053","Q9Y2W1","THRAP3",0.2727,"Cardiometabolic_II","B13805","20212097_SS211647","PASS",-0.2157,-0.0812,"Plate control","PASS","PFC",0.945271065272406,"24h","24h"

Supplemental table 1_Olink proximity extension assay (PEA) data

,"SampleID","Index","OlinkID","UniProt","Assay","MissingFreq","Panel","Panel_Lot_Nr","PlateID","QC_Warning","LOD","NPX","Normalization","Assay_Warning","condition","Value","group","alt_group"
113,"PFC_02",13,"OID30053","Q9Y2W1","THRAP3",0.2727,"Cardiometabolic_II","B13805","20212097_SS211647","PASS",-0.2157,-0.2067,"Plate control","PASS","PFC",0.866517024816751,"Baseline","Baseline"
114,"PFC_10",14,"OID30053","Q9Y2W1","THRAP3",0.2727,"Cardiometabolic_II","B13805","20212097_SS211647","PASS",-0.2157,1.3672,"Plate control","PASS","PFC",2.57969409806264,"1h","1h"
115,"PFC_18",15,"OID30053","Q9Y2W1","THRAP3",0.2727,"Cardiometabolic_II","B13805","20212097_SS211647","PASS",-0.2157,0.0867,"Plate control","PASS","PFC",1.06193833966444,"1h","1h"
116,"PFC_26",16,"OID30053","Q9Y2W1","THRAP3",0.2727,"Cardiometabolic_II","B13805","20212097_SS211647","PASS",-0.2157,0.8205,"Plate control","PASS","PFC",1.76601794171104,"24h","24h"
117,"PFC_03",25,"OID30053","Q9Y2W1","THRAP3",0.2727,"Cardiometabolic_II","B13805","20212097_SS211647","PASS",-0.2157,0.0023,"Plate control","PASS","PFC",1.0015955099891,"Baseline","Baseline"
118,"PFC_11",26,"OID30053","Q9Y2W1","THRAP3",0.2727,"Cardiometabolic_II","B13805","20212097_SS211647","PASS",-0.2157,-0.059,"Plate control","PASS","PFC",0.959929261038908,"1h","1h"
119,"PFC_19",27,"OID30053","Q9Y2W1","THRAP3",0.2727,"Cardiometabolic_II","B13805","20212097_SS211647","PASS",-0.2157,1.2923,"Plate control","PASS","PFC",2.4491820249364,"24h","24h"
120,"PFC_27",28,"OID30053","Q9Y2W1","THRAP3",0.2727,"Cardiometabolic_II","B13805","20212097_SS211647","PASS",-0.2157,-0.5963,"Plate control","PASS","PFC",0.661448161373862,"24h","24h"
121,"PFC_04",37,"OID30053","Q9Y2W1","THRAP3",0.2727,"Cardiometabolic_II","B13805","20212097_SS211647","PASS",-0.2157,-0.2741,"Plate control","PASS","PFC",0.82696604553834,"Baseline","Baseline"
122,"PFC_12",38,"OID30053","Q9Y2W1","THRAP3",0.2727,"Cardiometabolic_II","B13805","20212097_SS211647","PASS",-0.2157,0.0663,"Plate control","PASS","PFC",1.04702798268725,"1h","1h"
123,"PFC_20",39,"OID30053","Q9Y2W1","THRAP3",0.2727,"Cardiometabolic_II","B13805","20212097_SS211647","PASS",-0.2157,-0.1438,"Plate control","PASS","PFC",0.905131932115196,"24h","24h"
124,"PFC_05",49,"OID30053","Q9Y2W1","THRAP3",0.2727,"Cardiometabolic_II","B13805","20212097_SS211647","PASS",-0.2157,2.5459,"Plate control","PASS","PFC",5.83972324981861,"Baseline","Baseline"
125,"PFC_13",50,"OID30053","Q9Y2W1","THRAP3",0.2727,"Cardiometabolic_II","B13805","20212097_SS211647","PASS",-0.2157,-0.5807,"Plate control","PASS","PFC",0.668639272875372,"1h","1h"
126,"PFC_21",51,"OID30053","Q9Y2W1","THRAP3",0.2727,"Cardiometabolic_II","B13805","20212097_SS211647","PASS",-0.2157,-0.7088,"Plate control","PASS","PFC",0.611828832173106,"24h","24h"
127,"PFC_06",61,"OID30053","Q9Y2W1","THRAP3",0.2727,"Cardiometabolic_II","B13805","20212097_SS211647","PASS",-0.2157,-0.0467,"Plate control","PASS","PFC",0.968148326748363,"Baseline","Baseline"
128,"PFC_14",62,"OID30053","Q9Y2W1","THRAP3",0.2727,"Cardiometabolic_II","B13805","20212097_SS211647","PASS",-0.2157,1.9406,"Plate control","PASS","PFC",3.83865259610896,"1h","1h"
129,"PFC_22",63,"OID30053","Q9Y2W1","THRAP3",0.2727,"Cardiometabolic_II","B13805","20212097_SS211647","PASS",-0.2157,-0.9749,"Plate control","PASS","PFC",0.508775110432492,"24h","24h"
130,"PFC_07",73,"OID30053","Q9Y2W1","THRAP3",0.2727,"Cardiometabolic_II","B13805","20212097_SS211647","PASS",-0.2157,-0.0417,"Plate control","PASS","PFC",0.971509494258729,"Baseline","Baseline"
131,"PFC_15",74,"OID30053","Q9Y2W1","THRAP3",0.2727,"Cardiometabolic_II","B13805","20212097_SS211647","PASS",-0.2157,-1.0731,"Plate control","PASS","PFC",0.475296604422966,"1h","1h"
132,"PFC_23",75,"OID30053","Q9Y2W1","THRAP3",0.2727,"Cardiometabolic_II","B13805","20212097_SS211647","PASS",-0.2157,2.67,"Plate control","PASS","PFC",6.36429187003935,"24h","24h"
133,"PFC_08",85,"OID30053","Q9Y2W1","THRAP3",0.2727,"Cardiometabolic_II","B13805","20212097_SS211647","PASS",-0.2157,0.4281,"Plate control","PASS","PFC",1.34546046550257,"Baseline","Baseline"
134,"PFC_16",86,"OID30053","Q9Y2W1","THRAP3",0.2727,"Cardiometabolic_II","B13805","20212097_SS211647","PASS",-0.2157,-0.3453,"Plate control","PASS","PFC",0.787144277498668,"1h","1h"
135,"PFC_24",87,"OID30053","Q9Y2W1","THRAP3",0.2727,"Cardiometabolic_II","B13805","20212097_SS211647","PASS",-0.2157,-0.079,"Plate control","PASS","PFC",0.946713631236714,"24h","24h"
136,"PFC_01",1,"OID30054","O43734","TRAF3IP2",0.5455,"Cardiometabolic_II","B13805","20212097_SS211647","WARN",0.0733,1.5415,"Plate control","PASS","PFC",2.9109700578108,"Baseline","Baseline"
137,"PFC_09",2,"OID30054","O43734","TRAF3IP2",0.5455,"Cardiometabolic_II","B13805","20212097_SS211647","PASS",0.0733,0.0057,"Plate control","PASS","PFC",1.00395875417754,"Baseline","Baseline"
138,"PFC_17",3,"OID30054","O43734","TRAF3IP2",0.5455,"Cardiometabolic_II","B13805","20212097_SS211647","PASS",0.0733,0.1526,"Plate control","PASS","PFC",1.11157092188835,"1h","1h"
139,"PFC_25",4,"OID30054","O43734","TRAF3IP2",0.5455,"Cardiometabolic_II","B13805","20212097_SS211647","PASS",0.0733,0.091,"Plate control","PASS","PFC",1.06510820339342,"24h","24h"
140,"PFC_02",13,"OID30054","O43734","TRAF3IP2",0.5455,"Cardiometabolic_II","B13805","20212097_SS211647","PASS",0.0733,-0.1795,"Plate control","PASS","PFC",0.883008970855519,"Baseline","Baseline"
141,"PFC_10",14,"OID30054","O43734","TRAF3IP2",0.5455,"Cardiometabolic_II","B13805","20212097_SS211647","PASS",0.0733,1.5535,"Plate control","PASS","PFC",2.93528380391818,"1h","1h"
142,"PFC_18",15,"OID30054","O43734","TRAF3IP2",0.5455,"Cardiometabolic_II","B13805","20212097_SS211647","PASS",0.0733,-0.5659,"Plate control","PASS","PFC",0.67553386508444,"1h","1h"
143,"PFC_26",16,"OID30054","O43734","TRAF3IP2",0.5455,"Cardiometabolic_II","B13805","20212097_SS211647","PASS",0.0733,-0.2752,"Plate control","PASS","PFC",0.826335755753675,"24h","24h"
144,"PFC_03",25,"OID30054","O43734","TRAF3IP2",0.5455,"Cardiometabolic_II","B13805","20212097_SS211647","PASS",0.0733,-0.0678,"Plate control","PASS","PFC",0.954091806589203,"Baseline","Baseline"
145,"PFC_11",26,"OID30054","O43734","TRAF3IP2",0.5455,"Cardiometabolic_II","B13805","20212097_SS211647","PASS",0.0733,-0.282,"Plate control","PASS","PFC",0.822450068712464,"1h","1h"
146,"PFC_19",27,"OID30054","O43734","TRAF3IP2",0.5455,"Cardiometabolic_II","B13805","20212097_SS211647","PASS",0.0733,1.4735,"Plate control","PASS","PFC",2.77694768800659,"24h","24h"
147,"PFC_27",28,"OID30054","O43734","TRAF3IP2",0.5455,"Cardiometabolic_II","B13805","20212097_SS211647","PASS",0.0733,-0.1319,"Plate control","PASS","PFC",0.912628744955273,"24h","24h"
148,"PFC_04",37,"OID30054","O43734","TRAF3IP2",0.5455,"Cardiometabolic_II","B13805","20212097_SS211647","PASS",0.0733,0.5549,"Plate control","PASS","PFC",1.46906680179797,"Baseline","Baseline"
149,"PFC_12",38,"OID30054","O43734","TRAF3IP2",0.5455,"Cardiometabolic_II","B13805","20212097_SS211647","PASS",0.0733,0.2749,"Plate control","PASS","PFC",1.20991022172681,"1h","1h"
150,"PFC_20",39,"OID30054","O43734","TRAF3IP2",0.5455,"Cardiometabolic_II","B13805","20212097_SS211647","PASS",0.0733,-0.2804,"Plate control","PASS","PFC",0.823362701003769,"24h","24h"
151,"PFC_05",49,"OID30054","O43734","TRAF3IP2",0.5455,"Cardiometabolic_II","B13805","20212097_SS211647","PASS",0.0733,-0.5543,"Plate control","PASS","PFC",0.680987395248109,"Baseline","Baseline"
152,"PFC_13",50,"OID30054","O43734","TRAF3IP2",0.5455,"Cardiometabolic_II","B13805","20212097_SS211647","PASS",0.0733,0.4841,"Plate control","PASS","PFC",1.39871303077783,"1h","1h"
153,"PFC_21",51,"OID30054","O43734","TRAF3IP2",0.5455,"Cardiometabolic_II","B13805","20212097_SS211647","PASS",0.0733,0.0045,"Plate control","PASS","PFC",1.00312403196104,"24h","24h"
154,"PFC_06",61,"OID30054","O43734","TRAF3IP2",0.5455,"Cardiometabolic_II","B13805","20212097_SS211647","PASS",0.0733,0.4816,"Plate control","PASS","PFC",1.39629134463074,"Baseline","Baseline"
155,"PFC_14",62,"OID30054","O43734","TRAF3IP2",0.5455,"Cardiometabolic_II","B13805","20212097_SS211647","PASS",0.0733,-0.3397,"Plate control","PASS","PFC",0.790205613417912,"1h","1h"
156,"PFC_22",63,"OID30054","O43734","TRAF3IP2",0.5455,"Cardiometabolic_II","B13805","20212097_SS211647","PASS",0.0733,0.8602,"Plate control","PASS","PFC",1.81528994581347,"24h","24h"
157,"PFC_07",73,"OID30054","O43734","TRAF3IP2",0.5455,"Cardiometabolic_II","B13805","20212097_SS211647","PASS",0.0733,-0.0445,"Plate control","PASS","PFC",0.969625805405944,"Baseline","Baseline"
158,"PFC_15",74,"OID30054","O43734","TRAF3IP2",0.5455,"Cardiometabolic_II","B13805","20212097_SS211647","PASS",0.0733,-0.1566,"Plate control","PASS","PFC",0.897136864337869,"1h","1h"
159,"PFC_23",75,"OID30054","O43734","TRAF3IP2",0.5455,"Cardiometabolic_II","B13805","20212097_SS211647","PASS",0.0733,0.0599,"Plate control","PASS","PFC",1.04239350512505,"24h","24h"
160,"PFC_08",85,"OID30054","O43734","TRAF3IP2",0.5455,"Cardiometabolic_II","B13805","20212097_SS211647","PASS",0.0733,0.3905,"Plate control","PASS","PFC",1.31084763031192,"Baseline","Baseline"
161,"PFC_16",86,"OID30054","O43734","TRAF3IP2",0.5455,"Cardiometabolic_II","B13805","20212097_SS211647","PASS",0.0733,-0.065,"Plate control","PASS","PFC",0.955945317593742,"1h","1h"
162,"PFC_24",87,"OID30054","O43734","TRAF3IP2",0.5455,"Cardiometabolic_II","B13805","20212097_SS211647","PASS",0.0733,0.1926,"Plate control","PASS","PFC",1.14282143410224,"24h","24h"
163,"PFC_01",1,"OID30055","O00567","NOP56",0.2727,"Cardiometabolic_II","B13805","20212097_SS211647","WARN",-0.6694,-0.8916,"Plate control","PASS","PFC",0.539015998773932,"Baseline","Baseline"
164,"PFC_09",2,"OID30055","O00567","NOP56",0.2727,"Cardiometabolic_II","B13805","20212097_SS211647","PASS",-0.6694,-0.4542,"Plate control","PASS","PFC",0.729914810634533,"Baseline","Baseline"
165,"PFC_17",3,"OID30055","O00567","NOP56",0.2727,"Cardiometabolic_II","B13805","20212097_SS211647","PASS",-0.6694,2.6234,"Plate control","PASS","PFC",6.16200562779713,"1h","1h"
166,"PFC_25",4,"OID30055","O00567","NOP56",0.2727,"Cardiometabolic_II","B13805","20212097_SS211647","PASS",-0.6694,0.5234,"Plate control","PASS","PFC",1.43733863619255,"24h","24h"
167,"PFC_02",13,"OID30055","O00567","NOP56",0.2727,"Cardiometabolic_II","B13805","20212097_SS211647","PASS",-0.6694,-0.6974,"Plate control","PASS","PFC",0.616682580482812,"Baseline","Baseline"
168,"PFC_10",14,"OID30055","O00567","NOP56",0.2727,"Cardiometabolic_II","B13805","20212097_SS211647","PASS",-0.6694,-0.9396,"Plate control","PASS","PFC",0.521377416897236,"1h","1h"
169,"PFC_18",15,"OID30055","O00567","NOP56",0.2727,"Cardiometabolic_II","B13805","20212097_SS211647","PASS",-0.6694,-0.5011,"Plate control","PASS","PFC",0.70656784469302,"1h","1h"
170,"PFC_26",16,"OID30055","O00567","NOP56",0.2727,"Cardiometabolic_II","B13805","20212097_SS211647","PASS",-0.6694,2.3664,"Plate control","PASS","PFC",5.15652801690338,"24h","24h"
171,"PFC_03",25,"OID30055","O00567","NOP56",0.2727,"Cardiometabolic_II","B13805","20212097_SS211647","PASS",-0.6694,0.3475,"Plate control","PASS","PFC",1.27235389459647,"Baseline","Baseline"
172,"PFC_11",26,"OID30055","O00567","NOP56",0.2727,"Cardiometabolic_II","B13805","20212097_SS211647","PASS",-0.6694,-1.2171,"Plate control","PASS","PFC",0.430146498789084,"1h","1h"
173,"PFC_19",27,"OID30055","O00567","NOP56",0.2727,"Cardiometabolic_II","B13805","20212097_SS211647","PASS",-0.6694,-0.2107,"Plate control","PASS","PFC",0.864117856975881,"24h","24h"
174,"PFC_27",28,"OID30055","O00567","NOP56",0.2727,"Cardiometabolic_II","B13805","20212097_SS211647","PASS",-0.6694,-0.6869,"Plate control","PASS","PFC",0.621187196815405,"24h","24h"
175,"PFC_04",37,"OID30055","O00567","NOP56",0.2727,"Cardiometabolic_II","B13805","20212097_SS211647","PASS",-0.6694,-0.6899,"Plate control","PASS","PFC",0.619896816453428,"Baseline","Baseline"
176,"PFC_12",38,"OID30055","O00567","NOP56",0.2727,"Cardiometabolic_II","B13805","20212097_SS211647","PASS",-0.6694,0.8258,"Plate control","PASS","PFC",1.77251765823326,"1h","1h"
177,"PFC_20",39,"OID30055","O00567","NOP56",0.2727,"Cardiometabolic_II","B13805","20212097_SS211647","PASS",-0.6694,-0.5944,"Plate control","PASS","PFC",0.662319849008778,"24h","24h"
178,"PFC_05",49,"OID30055","O00567","NOP56",0.2727,"Cardiometabolic_II","B13805","20212097_SS211647","PASS",-0.6694,-0.771,"Plate control","PASS","PFC",0.586011141837257,"Baseline","Baseline"
179,"PFC_13",50,"OID30055","O00567","NOP56",0.2727,"Cardiometabolic_II","B13805","20212097_SS211647","PASS",-0.6694,-1.4998,"Plate control","PASS","PFC",0.35360240689792,"1h","1h"
180,"PFC_21",51,"OID30055","O00567","NOP56",0.2727,"Cardiometabolic_II","B13805","20212097_SS211647","PASS",-0.6694,0.1337,"Plate control","PASS","PFC",1.09710377754074,"24h","24h"
181,"PFC_06",61,"OID30055","O00567","NOP56",0.2727,"Cardiometabolic_II","B13805","20212097_SS211647","PASS",-0.6694,-0.441,"Plate control","PASS","PFC",0.73662384290824,"Baseline","Baseline"
182,"PFC_14",62,"OID30055","O00567","NOP56",0.2727,"Cardiometabolic_II","B13805","20212097_SS211647","PASS",-0.6694,-0.1372,"Plate control","PASS","PFC",0.909282189785745,"1h","1h"
183,"PFC_22",63,"OID30055","O00567","NOP56",0.2727,"Cardiometabolic_II","B13805","20212097_SS211647","PASS",-0.6694,-0.3899,"Plate control","PASS","PFC",0.763182502426936,"24h","24h"
184,"PFC_07",73,"OID30055","O00567","NOP56",0.2727,"Cardiometabolic_II","B13805","20212097_SS211647","PASS",-0.6694,-0.1816,"Plate control","PASS","PFC",0.881724589985555,"Baseline","Baseline"
185,"PFC_15",74,"OID30055","O00567","NOP56",0.2727,"Cardiometabolic_II","B13805","20212097_SS211647","PASS",-0.6694,-0.7284,"Plate control","PASS","PFC",0.603572926593097,"1h","1h"
186,"PFC_23",75,"OID30055","O00567","NOP56",0.2727,"Cardiometabolic_II","B13805","20212097_SS211647","PASS",-0.6694,-0.4916,"Plate control","PASS","PFC",0.711235874415356,"24h","24h"
187,"PFC_08",85,"OID30055","O00567","NOP56",0.2727,"Cardiometabolic_II","B13805","20212097_SS211647","PASS",-0.6694,2.3469,"Plate control","PASS","PFC",5.08729939003008,"Baseline","Baseline"
188,"PFC_16",86,"OID30055","O00567","NOP56",0.2727,"Cardiometabolic_II","B13805","20212097_SS211647","PASS",-0.6694,-0.5293,"Plate control","PASS","PFC",0.692890845242857,"1h","1h"
189,"PFC_24",87,"OID30055","O00567","NOP56",0.2727,"Cardiometabolic_II","B13805","20212097_SS211647","PASS",-0.6694,-0.287,"Plate control","PASS","PFC",0.819604607637517,"24h","24h"
190,"PFC_01",1,"OID30056","Q15477","SKIV2L",1,"Cardiometabolic_II","B13805","20212097_SS211647","WARN",0.7042,0.1446,"Plate control","PASS","PFC",1.10542412218255,"Baseline","Baseline"
191,"PFC_09",2,"OID30056","Q15477","SKIV2L",1,"Cardiometabolic_II","B13805","20212097_SS211647","PASS",0.7042,0.1318,"Plate control","PASS","PFC",1.09565986520966,"Baseline","Baseline"
192,"PFC_17",3,"OID30056","Q15477","SKIV2L",1,"Cardiometabolic_II","B13805","20212097_SS211647","PASS",0.7042,0.3684,"Plate control","PASS","PFC",1.29092035996137,"1h","1h"
193,"PFC_25",4,"OID30056","Q15477","SKIV2L",1,"Cardiometabolic_II","B13805","20212097_SS211647","PASS",0.7042,-0.0303,"Plate control","PASS","PFC",0.979216654035547,"24h","24h"
194,"PFC_02",13,"OID30056","Q15477","SKIV2L",1,"Cardiometabolic_II","B13805","20212097_SS211647","PASS",0.7042,-0.1722,"Plate control","PASS","PFC",0.887488296739042,"Baseline","Baseline"
195,"PFC_10",14,"OID30056","Q15477","SKIV2L",1,"Cardiometabolic_II","B13805","20212097_SS211647","PASS",0.7042,0.3954,"Plate control","PASS","PFC",1.31530739029793,"1h","1h"
196,"PFC_18",15,"OID30056","Q15477","SKIV2L",1,"Cardiometabolic_II","B13805","20212097_SS211647","PASS",0.7042,-0.2312,"Plate control","PASS","PFC",0.851925984869388,"1h","1h"
197,"PFC_26",16,"OID30056","Q15477","SKIV2L",1,"Cardiometabolic_II","B13805","20212097_SS211647","PASS",0.7042,0.5252,"Plate control","PASS","PFC",1.43913307238906,"24h","24h"
198,"PFC_03",25,"OID30056","Q15477","SKIV2L",1,"Cardiometabolic_II","B13805","20212097_SS211647","PASS",0.7042,0.1275,"Plate control","PASS","PFC",1.0923990768175,"Baseline","Baseline"
199,"PFC_11",26,"OID30056","Q15477","SKIV2L",1,"Cardiometabolic_II","B13805","20212097_SS211647","PASS",0.7042,-0.0741,"Plate control","PASS","PFC",0.949934536148294,"1h","1h"
200,"PFC_19",27,"OID30056","Q15477","SKIV2L",1,"Cardiometabolic_II","B13805","20212097_SS211647","PASS",0.7042,-0.1572,"Plate control","PASS","PFC",0.896763833180039,"24h","24h"
201,"PFC_27",28,"OID30056","Q15477","SKIV2L",1,"Cardiometabolic_II","B13805","20212097_SS211647","PASS",0.7042,0.1182,"Plate control","PASS","PFC",1.0853798270462,"24h","24h"
202,"PFC_04",37,"OID30056","Q15477","SKIV2L",1,"Cardiometabolic_II","B13805","20212097_SS211647","PASS",0.7042,-0.038,"Plate control","PASS","PFC",0.974004268543241,"Baseline","Baseline"
203,"PFC_12",38,"OID30056","Q15477","SKIV2L",1,"Cardiometabolic_II","B13805","20212097_SS211647","PASS",0.7042,-0.063,"Plate control","PASS","PFC",0.957271458195298,"1h","1h"
204,"PFC_20",39,"OID30056","Q15477","SKIV2L",1,"Cardiometabolic_II","B13805","20212097_SS211647","PASS",0.7042,-0.1523,"Plate control","PASS","PFC",0.899814799110512,"24h","24h"
205,"PFC_05",49,"OID30056","Q15477","SKIV2L",1,"Cardiometabolic_II","B13805","20212097_SS211647","PASS",0.7042,-0.2557,"Plate control","PASS","PFC",0.837580639398472,"Baseline","Baseline"
206,"PFC_13",50,"OID30056","Q15477","SKIV2L",1,"Cardiometabolic_II","B13805","20212097_SS211647","PASS",0.7042,-0.2088,"Plate control","PASS","PFC",0.865256632310099,"1h","1h"
207,"PFC_21",51,"OID30056","Q15477","SKIV2L",1,"Cardiometabolic_II","B13805","20212097_SS211647","PASS",0.7042,-0.4402,"Plate control","PASS","PFC",0.737032427173259,"24h","24h"
208,"PFC_06",61,"OID30056","Q15477","SKIV2L",1,"Cardiometabolic_II","B13805","20212097_SS211647","PASS",0.7042,0.414,"Plate control","PASS","PFC",1.33237482509202,"Baseline","Baseline"
209,"PFC_14",62,"OID30056","Q15477","SKIV2L",1,"Cardiometabolic_II","B13805","20212097_SS211647","PASS",0.7042,0.1319,"Plate control","PASS","PFC",1.09573581319643,"1h","1h"
210,"PFC_22",63,"OID30056","Q15477","SKIV2L",1,"Cardiometabolic_II","B13805","20212097_SS211647","PASS",0.7042,0.1375,"Plate control","PASS","PFC",1.09999731327822,"24h","24h"
211,"PFC_07",73,"OID30056","Q15477","SKIV2L",1,"Cardiometabolic_II","B13805","20212097_SS211647","PASS",0.7042,0.0229,"Plate control","PASS","PFC",1.01599971681903,"Baseline","Baseline"
212,"PFC_15",74,"OID30056","Q15477","SKIV2L",1,"Cardiometabolic_II","B13805","20212097_SS211647","PASS",0.7042,-0.1654,"Plate control","PASS","PFC",0.891681258603905,"1h","1h"
213,"PFC_23",75,"OID30056","Q15477","SKIV2L",1,"Cardiometabolic_II","B13805","20212097_SS211647","PASS",0.7042,0.073,"Plate control","PASS","PFC",1.05190177920387,"24h","24h"
214,"PFC_08",85,"OID30056","Q15477","SKIV2L",1,"Cardiometabolic_II","B13805","20212097_SS211647","PASS",0.7042,0.0848,"Plate control","PASS","PFC",1.06054070901648,"Baseline","Baseline"
215,"PFC_16",86,"OID30056","Q15477","SKIV2L",1,"Cardiometabolic_II","B13805","20212097_SS211647","PASS",0.7042,-0.0636,"Plate control","PASS","PFC",0.956873422962767,"1h","1h"
216,"PFC_24",87,"OID30056","Q15477","SKIV2L",1,"Cardiometabolic_II","B13805","20212097_SS211647","PASS",0.7042,0.1168,"Plate control","PASS","PFC",1.08432707877173,"24h","24h"
217,"PFC_01",1,"OID30057","P25391","LAMA1",0.8864,"Cardiometabolic_II","B13805","20212097_SS211647","WARN",0.807,0.0326,"Plate control","PASS","PFC",1.02285383511537,"Baseline","Baseline"
218,"PFC_09",2,"OID30057","P25391","LAMA1",0.8864,"Cardiometabolic_II","B13805","20212097_SS211647","PASS",0.807,-0.0119,"Plate control","PASS","PFC",0.991785473686297,"Baseline","Baseline"
219,"PFC_17",3,"OID30057","P25391","LAMA1",0.8864,"Cardiometabolic_II","B13805","20212097_SS211647","PASS",0.807,-0.0412,"Plate control","PASS","PFC",0.971846252144469,"1h","1h"
220,"PFC_25",4,"OID30057","P25391","LAMA1",0.8864,"Cardiometabolic_II","B13805","20212097_SS211647","PASS",0.807,-0.1946,"Plate control","PASS","PFC",0.873815135332583,"24h","24h"
221,"PFC_02",13,"OID30057","P25391","LAMA1",0.8864,"Cardiometabolic_II","B13805","20212097_SS211647","PASS",0.807,0.0098,"Plate control","PASS","PFC",1.00681596605207,"Baseline","Baseline"
222,"PFC_10",14,"OID30057","P25391","LAMA1",0.8864,"Cardiometabolic_II","B13805","20212097_SS211647","PASS",0.807,0.0098,"Plate control","PASS","PFC",1.00681596605207,"1h","1h"
223,"PFC_18",15,"OID30057","P25391","LAMA1",0.8864,"Cardiometabolic_II","B13805","20212097_SS211647","PASS",0.807,-0.1785,"Plate control","PASS","PFC",0.883621238205257,"1h","1h"
224,"PFC_26",16,"OID30057","P25391","LAMA1",0.8864,"Cardiometabolic_II","B13805","20212097_SS211647","PASS",0.807,-0.5229,"Plate control","PASS","PFC",0.695971435307495,"24h","24h"

Supplemental table 1_Olink proximity extension assay (PEA) data

**,"SampleID","Index","OlinkID","UniProt","Assay","MissingFreq","Panel","Panel_Lot_Nr","PlateID","QC_Warning","LOD","NPX","Normalization","Assay_Warning","condition","Value","group","alt_group"**
225,"PFC_03",25,"OID30057","P25391","LAMA1",0.8864,"Cardiometabolic_II","B13805","20212097_SS211647","PASS",0.807,-0.3519,"Plate control","PASS","PFC",0.783551496718163,"Baseline","Baseline"
226,"PFC_11",26,"OID30057","P25391","LAMA1",0.8864,"Cardiometabolic_II","B13805","20212097_SS211647","PASS",0.807,-0.5394,"Plate control","PASS","PFC",0.688057004437503,"1h","1h"
227,"PFC_19",27,"OID30057","P25391","LAMA1",0.8864,"Cardiometabolic_II","B13805","20212097_SS211647","PASS",0.807,0.1394,"Plate control","PASS","PFC",1.10144694170327,"24h","24h"
228,"PFC_27",28,"OID30057","P25391","LAMA1",0.8864,"Cardiometabolic_II","B13805","20212097_SS211647","PASS",0.807,-0.406,"Plate control","PASS","PFC",0.75471298426028,"24h","24h"
229,"PFC_04",37,"OID30057","P25391","LAMA1",0.8864,"Cardiometabolic_II","B13805","20212097_SS211647","PASS",0.807,-0.1477,"Plate control","PASS","PFC",0.902688416726604,"Baseline","Baseline"
230,"PFC_12",38,"OID30057","P25391","LAMA1",0.8864,"Cardiometabolic_II","B13805","20212097_SS211647","PASS",0.807,-0.6952,"Plate control","PASS","PFC",0.617623691804283,"1h","1h"
231,"PFC_20",39,"OID30057","P25391","LAMA1",0.8864,"Cardiometabolic_II","B13805","20212097_SS211647","PASS",0.807,0.0892,"Plate control","PASS","PFC",1.06378013391184,"24h","24h"
232,"PFC_05",49,"OID30057","P25391","LAMA1",0.8864,"Cardiometabolic_II","B13805","20212097_SS211647","PASS",0.807,2.0147,"Plate control","PASS","PFC",4.04096540344093,"Baseline","Baseline"
233,"PFC_13",50,"OID30057","P25391","LAMA1",0.8864,"Cardiometabolic_II","B13805","20212097_SS211647","PASS",0.807,-0.1536,"Plate control","PASS","PFC",0.899004348991801,"1h","1h"
234,"PFC_21",51,"OID30057","P25391","LAMA1",0.8864,"Cardiometabolic_II","B13805","20212097_SS211647","PASS",0.807,-0.4213,"Plate control","PASS","PFC",0.746751429815551,"24h","24h"
235,"PFC_06",61,"OID30057","P25391","LAMA1",0.8864,"Cardiometabolic_II","B13805","20212097_SS211647","PASS",0.807,-0.585,"Plate control","PASS","PFC",0.666649338545599,"Baseline","Baseline"
236,"PFC_14",62,"OID30057","P25391","LAMA1",0.8864,"Cardiometabolic_II","B13805","20212097_SS211647","PASS",0.807,2.1391,"Plate control","PASS","PFC",4.40487170425068,"1h","1h"
237,"PFC_22",63,"OID30057","P25391","LAMA1",0.8864,"Cardiometabolic_II","B13805","20212097_SS211647","PASS",0.807,-0.5582,"Plate control","PASS","PFC",0.679148985707263,"24h","24h"
238,"PFC_07",73,"OID30057","P25391","LAMA1",0.8864,"Cardiometabolic_II","B13805","20212097_SS211647","PASS",0.807,-0.2684,"Plate control","PASS","PFC",0.830239800825795,"Baseline","Baseline"
239,"PFC_15",74,"OID30057","P25391","LAMA1",0.8864,"Cardiometabolic_II","B13805","20212097_SS211647","PASS",0.807,-0.8048,"Plate control","PASS","PFC",0.572441432031902,"1h","1h"
240,"PFC_23",75,"OID30057","P25391","LAMA1",0.8864,"Cardiometabolic_II","B13805","20212097_SS211647","PASS",0.807,2.1993,"Plate control","PASS","PFC",4.59256455308533,"24h","24h"
241,"PFC_08",85,"OID30057","P25391","LAMA1",0.8864,"Cardiometabolic_II","B13805","20212097_SS211647","PASS",0.807,-0.4253,"Plate control","PASS","PFC",0.744683862803909,"Baseline","Baseline"
242,"PFC_16",86,"OID30057","P25391","LAMA1",0.8864,"Cardiometabolic_II","B13805","20212097_SS211647","PASS",0.807,-0.7877,"Plate control","PASS","PFC",0.57926684572849,"1h","1h"
243,"PFC_24",87,"OID30057","P25391","LAMA1",0.8864,"Cardiometabolic_II","B13805","20212097_SS211647","PASS",0.807,-0.4578,"Plate control","PASS","PFC",0.728095703000294,"24h","24h"
244,"PFC_01",1,"OID30058","P06753","TPM3",0.7727,"Cardiometabolic_II","B13805","20212097_SS211647","WARN",0.3146,-0.5108,"Plate control","PASS","PFC",0.701833151009861,"Baseline","Baseline"
245,"PFC_09",2,"OID30058","P06753","TPM3",0.7727,"Cardiometabolic_II","B13805","20212097_SS211647","PASS",0.3146,-0.368,"Plate control","PASS","PFC",0.774855930959171,"Baseline","Baseline"
246,"PFC_17",3,"OID30058","P06753","TPM3",0.7727,"Cardiometabolic_II","B13805","20212097_SS211647","PASS",0.3146,0.0235,"Plate control","PASS","PFC",1.01642234709993,"1h","1h"
247,"PFC_25",4,"OID30058","P06753","TPM3",0.7727,"Cardiometabolic_II","B13805","20212097_SS211647","PASS",0.3146,0.2365,"Plate control","PASS","PFC",1.17813102794876,"24h","24h"
248,"PFC_02",13,"OID30058","P06753","TPM3",0.7727,"Cardiometabolic_II","B13805","20212097_SS211647","PASS",0.3146,0.5281,"Plate control","PASS","PFC",1.44202882181738,"Baseline","Baseline"
249,"PFC_10",14,"OID30058","P06753","TPM3",0.7727,"Cardiometabolic_II","B13805","20212097_SS211647","PASS",0.3146,-0.008,"Plate control","PASS","PFC",0.994470168673214,"1h","1h"
250,"PFC_18",15,"OID30058","P06753","TPM3",0.7727,"Cardiometabolic_II","B13805","20212097_SS211647","PASS",0.3146,-0.2716,"Plate control","PASS","PFC",0.828400310832826,"1h","1h"
251,"PFC_26",16,"OID30058","P06753","TPM3",0.7727,"Cardiometabolic_II","B13805","20212097_SS211647","PASS",0.3146,-0.5947,"Plate control","PASS","PFC",0.662182137786605,"24h","24h"
252,"PFC_03",25,"OID30058","P06753","TPM3",0.7727,"Cardiometabolic_II","B13805","20212097_SS211647","PASS",0.3146,-0.0538,"Plate control","PASS","PFC",0.963395439729813,"Baseline","Baseline"
253,"PFC_11",26,"OID30058","P06753","TPM3",0.7727,"Cardiometabolic_II","B13805","20212097_SS211647","PASS",0.3146,0.5288,"Plate control","PASS","PFC",1.44272866833597,"1h","1h"
254,"PFC_19",27,"OID30058","P06753","TPM3",0.7727,"Cardiometabolic_II","B13805","20212097_SS211647","PASS",0.3146,-0.2465,"Plate control","PASS","PFC",0.842938919252563,"24h","24h"
255,"PFC_27",28,"OID30058","P06753","TPM3",0.7727,"Cardiometabolic_II","B13805","20212097_SS211647","PASS",0.3146,-0.5871,"Plate control","PASS","PFC",0.66567966362081,"24h","24h"
256,"PFC_04",37,"OID30058","P06753","TPM3",0.7727,"Cardiometabolic_II","B13805","20212097_SS211647","PASS",0.3146,-0.0721,"Plate control","PASS","PFC",0.95125233825896,"Baseline","Baseline"
257,"PFC_12",38,"OID30058","P06753","TPM3",0.7727,"Cardiometabolic_II","B13805","20212097_SS211647","PASS",0.3146,0.4943,"Plate control","PASS","PFC",1.40863711431218,"1h","1h"
258,"PFC_20",39,"OID30058","P06753","TPM3",0.7727,"Cardiometabolic_II","B13805","20212097_SS211647","PASS",0.3146,0.5943,"Plate control","PASS","PFC",1.5097398774635,"24h","24h"
259,"PFC_05",49,"OID30058","P06753","TPM3",0.7727,"Cardiometabolic_II","B13805","20212097_SS211647","PASS",0.3146,0.066,"Plate control","PASS","PFC",1.04681028197458,"Baseline","Baseline"
260,"PFC_13",50,"OID30058","P06753","TPM3",0.7727,"Cardiometabolic_II","B13805","20212097_SS211647","PASS",0.3146,-1.1751,"Plate control","PASS","PFC",0.442853062252155,"1h","1h"
261,"PFC_21",51,"OID30058","P06753","TPM3",0.7727,"Cardiometabolic_II","B13805","20212097_SS211647","PASS",0.3146,0.9141,"Plate control","PASS","PFC",1.88439315946347,"24h","24h"
262,"PFC_06",61,"OID30058","P06753","TPM3",0.7727,"Cardiometabolic_II","B13805","20212097_SS211647","PASS",0.3146,0.066,"Plate control","PASS","PFC",1.04681028197458,"Baseline","Baseline"
263,"PFC_14",62,"OID30058","P06753","TPM3",0.7727,"Cardiometabolic_II","B13805","20212097_SS211647","PASS",0.3146,-0.1171,"Plate control","PASS","PFC",0.922039204810079,"1h","1h"
264,"PFC_22",63,"OID30058","P06753","TPM3",0.7727,"Cardiometabolic_II","B13805","20212097_SS211647","PASS",0.3146,-0.2408,"Plate control","PASS","PFC",0.846275907220562,"24h","24h"
265,"PFC_07",73,"OID30058","P06753","TPM3",0.7727,"Cardiometabolic_II","B13805","20212097_SS211647","PASS",0.3146,0.0665,"Plate control","PASS","PFC",1.0471731416475,"Baseline","Baseline"
266,"PFC_15",74,"OID30058","P06753","TPM3",0.7727,"Cardiometabolic_II","B13805","20212097_SS211647","PASS",0.3146,-0.3734,"Plate control","PASS","PFC",0.771961070361991,"1h","1h"
267,"PFC_23",75,"OID30058","P06753","TPM3",0.7727,"Cardiometabolic_II","B13805","20212097_SS211647","PASS",0.3146,0.0241,"Plate control","PASS","PFC",1.01684515318438,"24h","24h"
268,"PFC_08",85,"OID30058","P06753","TPM3",0.7727,"Cardiometabolic_II","B13805","20212097_SS211647","PASS",0.3146,-1.0107,"Plate control","PASS","PFC",0.496305380415879,"Baseline","Baseline"
269,"PFC_16",86,"OID30058","P06753","TPM3",0.7727,"Cardiometabolic_II","B13805","20212097_SS211647","PASS",0.3146,0.0322,"Plate control","PASS","PFC",1.02257027912562,"1h","1h"
270,"PFC_24",87,"OID30058","P06753","TPM3",0.7727,"Cardiometabolic_II","B13805","20212097_SS211647","PASS",0.3146,0.453,"Plate control","PASS","PFC",1.36888381309184,"24h","24h"
271,"PFC_01",1,"OID30059","P48507","GCLM",0.0682,"Cardiometabolic_II","B13805","20212097_SS211647","WARN",-0.5514,-0.3472,"Plate control","PASS","PFC",0.786108306834921,"Baseline","Baseline"
272,"PFC_09",2,"OID30059","P48507","GCLM",0.0682,"Cardiometabolic_II","B13805","20212097_SS211647","PASS",-0.5514,0.4447,"Plate control","PASS","PFC",1.36103106861706,"Baseline","Baseline"
273,"PFC_17",3,"OID30059","P48507","GCLM",0.0682,"Cardiometabolic_II","B13805","20212097_SS211647","PASS",-0.5514,0.3354,"Plate control","PASS","PFC",1.26172718539616,"1h","1h"
274,"PFC_25",4,"OID30059","P48507","GCLM",0.0682,"Cardiometabolic_II","B13805","20212097_SS211647","PASS",-0.5514,0.502,"Plate control","PASS","PFC",1.41617543821454,"24h","24h"
275,"PFC_02",13,"OID30059","P48507","GCLM",0.0682,"Cardiometabolic_II","B13805","20212097_SS211647","PASS",-0.5514,0.4206,"Plate control","PASS","PFC",1.33848409893563,"Baseline","Baseline"
276,"PFC_10",14,"OID30059","P48507","GCLM",0.0682,"Cardiometabolic_II","B13805","20212097_SS211647","PASS",-0.5514,-0.0279,"Plate control","PASS","PFC",0.980846988764834,"1h","1h"
277,"PFC_18",15,"OID30059","P48507","GCLM",0.0682,"Cardiometabolic_II","B13805","20212097_SS211647","PASS",-0.5514,-0.0617,"Plate control","PASS","PFC",0.958134435963762,"1h","1h"
278,"PFC_26",16,"OID30059","P48507","GCLM",0.0682,"Cardiometabolic_II","B13805","20212097_SS211647","PASS",-0.5514,0.5515,"Plate control","PASS","PFC",1.46560872788076,"24h","24h"
279,"PFC_03",25,"OID30059","P48507","GCLM",0.0682,"Cardiometabolic_II","B13805","20212097_SS211647","PASS",-0.5514,-0.5038,"Plate control","PASS","PFC",0.705246741423833,"Baseline","Baseline"
280,"PFC_11",26,"OID30059","P48507","GCLM",0.0682,"Cardiometabolic_II","B13805","20212097_SS211647","PASS",-0.5514,0.5245,"Plate control","PASS","PFC",1.43843497004134,"1h","1h"
281,"PFC_19",27,"OID30059","P48507","GCLM",0.0682,"Cardiometabolic_II","B13805","20212097_SS211647","PASS",-0.5514,0.1249,"Plate control","PASS","PFC",1.09043214704886,"24h","24h"
282,"PFC_27",28,"OID30059","P48507","GCLM",0.0682,"Cardiometabolic_II","B13805","20212097_SS211647","PASS",-0.5514,0.9324,"Plate control","PASS","PFC",1.9084481619334,"24h","24h"
283,"PFC_04",37,"OID30059","P48507","GCLM",0.0682,"Cardiometabolic_II","B13805","20212097_SS211647","PASS",-0.5514,0.4403,"Plate control","PASS","PFC",1.35688645471943,"Baseline","Baseline"
284,"PFC_12",38,"OID30059","P48507","GCLM",0.0682,"Cardiometabolic_II","B13805","20212097_SS211647","PASS",-0.5514,-0.0846,"Plate control","PASS","PFC",0.943045967535861,"1h","1h"
285,"PFC_20",39,"OID30059","P48507","GCLM",0.0682,"Cardiometabolic_II","B13805","20212097_SS211647","PASS",-0.5514,0.9853,"Plate control","PASS","PFC",1.97972494225957,"24h","24h"
286,"PFC_05",49,"OID30059","P48507","GCLM",0.0682,"Cardiometabolic_II","B13805","20212097_SS211647","PASS",-0.5514,-0.5082,"Plate control","PASS","PFC",0.703099122965178,"Baseline","Baseline"
287,"PFC_13",50,"OID30059","P48507","GCLM",0.0682,"Cardiometabolic_II","B13805","20212097_SS211647","PASS",-0.5514,0.3973,"Plate control","PASS","PFC",1.31704076451407,"1h","1h"
288,"PFC_21",51,"OID30059","P48507","GCLM",0.0682,"Cardiometabolic_II","B13805","20212097_SS211647","PASS",-0.5514,-0.2678,"Plate control","PASS","PFC",0.830585159662447,"24h","24h"
289,"PFC_06",61,"OID30059","P48507","GCLM",0.0682,"Cardiometabolic_II","B13805","20212097_SS211647","PASS",-0.5514,0.2297,"Plate control","PASS","PFC",1.17259109041577,"Baseline","Baseline"
290,"PFC_14",62,"OID30059","P48507","GCLM",0.0682,"Cardiometabolic_II","B13805","20212097_SS211647","PASS",-0.5514,-0.0683,"Plate control","PASS","PFC",0.953761200859253,"1h","1h"
291,"PFC_22",63,"OID30059","P48507","GCLM",0.0682,"Cardiometabolic_II","B13805","20212097_SS211647","PASS",-0.5514,0.098,"Plate control","PASS","PFC",1.07028869842662,"24h","24h"
292,"PFC_07",73,"OID30059","P48507","GCLM",0.0682,"Cardiometabolic_II","B13805","20212097_SS211647","PASS",-0.5514,0.5742,"Plate control","PASS","PFC",1.48885163952398,"Baseline","Baseline"
293,"PFC_15",74,"OID30059","P48507","GCLM",0.0682,"Cardiometabolic_II","B13805","20212097_SS211647","PASS",-0.5514,-0.155,"Plate control","PASS","PFC",0.898132372883934,"1h","1h"
294,"PFC_23",75,"OID30059","P48507","GCLM",0.0682,"Cardiometabolic_II","B13805","20212097_SS211647","PASS",-0.5514,0.5391,"Plate control","PASS","PFC",1.45306576492461,"24h","24h"
295,"PFC_08",85,"OID30059","P48507","GCLM",0.0682,"Cardiometabolic_II","B13805","20212097_SS211647","PASS",-0.5514,-0.0037,"Plate control","PASS","PFC",0.997438641323161,"Baseline","Baseline"
296,"PFC_16",86,"OID30059","P48507","GCLM",0.0682,"Cardiometabolic_II","B13805","20212097_SS211647","PASS",-0.5514,0.2657,"Plate control","PASS","PFC",1.20221922844335,"1h","1h"
297,"PFC_24",87,"OID30059","P48507","GCLM",0.0682,"Cardiometabolic_II","B13805","20212097_SS211647","PASS",-0.5514,0.8979,"Plate control","PASS","PFC",1.8633516964357,"24h","24h"
298,"PFC_01",1,"OID30060","Q9NZJ5","EIF2AK3",0.1364,"Cardiometabolic_II","B13805","20212097_SS211647","WARN",0.4329,-0.4495,"Plate control","PASS","PFC",0.732296598659977,"Baseline","Baseline"
299,"PFC_09",2,"OID30060","Q9NZJ5","EIF2AK3",0.1364,"Cardiometabolic_II","B13805","20212097_SS211647","PASS",0.4329,2.2324,"Plate control","PASS","PFC",4.69915058544535,"Baseline","Baseline"
300,"PFC_17",3,"OID30060","Q9NZJ5","EIF2AK3",0.1364,"Cardiometabolic_II","B13805","20212097_SS211647","PASS",0.4329,2.6646,"Plate control","PASS","PFC",6.34051488514782,"1h","1h"
301,"PFC_25",4,"OID30060","Q9NZJ5","EIF2AK3",0.1364,"Cardiometabolic_II","B13805","20212097_SS211647","PASS",0.4329,3.131,"Plate control","PASS","PFC",8.7604197663486,"24h","24h"
302,"PFC_02",13,"OID30060","Q9NZJ5","EIF2AK3",0.1364,"Cardiometabolic_II","B13805","20212097_SS211647","PASS",0.4329,0.7384,"Plate control","PASS","PFC",1.66832458132365,"Baseline","Baseline"
303,"PFC_10",14,"OID30060","Q9NZJ5","EIF2AK3",0.1364,"Cardiometabolic_II","B13805","20212097_SS211647","PASS",0.4329,1.2441,"Plate control","PASS","PFC",2.36870740430302,"1h","1h"
304,"PFC_18",15,"OID30060","Q9NZJ5","EIF2AK3",0.1364,"Cardiometabolic_II","B13805","20212097_SS211647","PASS",0.4329,2.0779,"Plate control","PASS","PFC",4.22192221906119,"1h","1h"
305,"PFC_26",16,"OID30060","Q9NZJ5","EIF2AK3",0.1364,"Cardiometabolic_II","B13805","20212097_SS211647","PASS",0.4329,2.3515,"Plate control","PASS","PFC",5.10354601451323,"24h","24h"
306,"PFC_03",25,"OID30060","Q9NZJ5","EIF2AK3",0.1364,"Cardiometabolic_II","B13805","20212097_SS211647","PASS",0.4329,1.0145,"Plate control","PASS","PFC",2.02020262275689,"Baseline","Baseline"
307,"PFC_11",26,"OID30060","Q9NZJ5","EIF2AK3",0.1364,"Cardiometabolic_II","B13805","20212097_SS211647","PASS",0.4329,2.7453,"Plate control","PASS","PFC",6.70529125850185,"1h","1h"
308,"PFC_19",27,"OID30060","Q9NZJ5","EIF2AK3",0.1364,"Cardiometabolic_II","B13805","20212097_SS211647","PASS",0.4329,1.7018,"Plate control","PASS","PFC",3.25306579059197,"24h","24h"
309,"PFC_27",28,"OID30060","Q9NZJ5","EIF2AK3",0.1364,"Cardiometabolic_II","B13805","20212097_SS211647","PASS",0.4329,3.0128,"Plate control","PASS","PFC",8.0712940742501,"24h","24h"
310,"PFC_04",37,"OID30060","Q9NZJ5","EIF2AK3",0.1364,"Cardiometabolic_II","B13805","20212097_SS211647","PASS",0.4329,2.6765,"Plate control","PASS","PFC",6.39303060326262,"Baseline","Baseline"
311,"PFC_12",38,"OID30060","Q9NZJ5","EIF2AK3",0.1364,"Cardiometabolic_II","B13805","20212097_SS211647","PASS",0.4329,1.4249,"Plate control","PASS","PFC",2.68495889194181,"1h","1h"
312,"PFC_20",39,"OID30060","Q9NZJ5","EIF2AK3",0.1364,"Cardiometabolic_II","B13805","20212097_SS211647","PASS",0.4329,3.399,"Plate control","PASS","PFC",10.5487489159389,"24h","24h"
313,"PFC_05",49,"OID30060","Q9NZJ5","EIF2AK3",0.1364,"Cardiometabolic_II","B13805","20212097_SS211647","PASS",0.4329,0.8812,"Plate control","PASS","PFC",1.84190671920176,"Baseline","Baseline"
314,"PFC_13",50,"OID30060","Q9NZJ5","EIF2AK3",0.1364,"Cardiometabolic_II","B13805","20212097_SS211647","PASS",0.4329,3.3866,"Plate control","PASS","PFC",10.458470682486,"1h","1h"
315,"PFC_21",51,"OID30060","Q9NZJ5","EIF2AK3",0.1364,"Cardiometabolic_II","B13805","20212097_SS211647","PASS",0.4329,1.6322,"Plate control","PASS","PFC",3.09985342498372,"24h","24h"
316,"PFC_06",61,"OID30060","Q9NZJ5","EIF2AK3",0.1364,"Cardiometabolic_II","B13805","20212097_SS211647","PASS",0.4329,1.2065,"Plate control","PASS","PFC",2.30777088397992,"Baseline","Baseline"
317,"PFC_14",62,"OID30060","Q9NZJ5","EIF2AK3",0.1364,"Cardiometabolic_II","B13805","20212097_SS211647","PASS",0.4329,2.3637,"Plate control","PASS","PFC",5.14688661293637,"1h","1h"
318,"PFC_22",63,"OID30060","Q9NZJ5","EIF2AK3",0.1364,"Cardiometabolic_II","B13805","20212097_SS211647","PASS",0.4329,2.4814,"Plate control","PASS","PFC",5.58439116386165,"24h","24h"
319,"PFC_07",73,"OID30060","Q9NZJ5","EIF2AK3",0.1364,"Cardiometabolic_II","B13805","20212097_SS211647","PASS",0.4329,3.1241,"Plate control","PASS","PFC",8.71862120553337,"Baseline","Baseline"
320,"PFC_15",74,"OID30060","Q9NZJ5","EIF2AK3",0.1364,"Cardiometabolic_II","B13805","20212097_SS211647","PASS",0.4329,1.8211,"Plate control","PASS","PFC",3.53350512134888,"1h","1h"
321,"PFC_23",75,"OID30060","Q9NZJ5","EIF2AK3",0.1364,"Cardiometabolic_II","B13805","20212097_SS211647","PASS",0.4329,2.1851,"Plate control","PASS","PFC",4.54758309645998,"24h","24h"
322,"PFC_08",85,"OID30060","Q9NZJ5","EIF2AK3",0.1364,"Cardiometabolic_II","B13805","20212097_SS211647","PASS",0.4329,2.0189,"Plate control","PASS","PFC",4.05274667590715,"Baseline","Baseline"
323,"PFC_16",86,"OID30060","Q9NZJ5","EIF2AK3",0.1364,"Cardiometabolic_II","B13805","20212097_SS211647","PASS",0.4329,3.3044,"Plate control","PASS","PFC",9.87923958228758,"1h","1h"
324,"PFC_24",87,"OID30060","Q9NZJ5","EIF2AK3",0.1364,"Cardiometabolic_II","B13805","20212097_SS211647","PASS",0.4329,2.6571,"Plate control","PASS","PFC",6.30763858948839,"24h","24h"
325,"PFC_01",1,"OID30061","Q9Y623","MYH4",0.9318,"Cardiometabolic_II","B13805","20212097_SS211647","WARN",0.4263,0.1105,"Plate control","PASS","PFC",1.07960233333138,"Baseline","Baseline"
326,"PFC_09",2,"OID30061","Q9Y623","MYH4",0.9318,"Cardiometabolic_II","B13805","20212097_SS211647","PASS",0.4263,0.1296,"Plate control","PASS","PFC",1.0939903406799,"Baseline","Baseline"
327,"PFC_17",3,"OID30061","Q9Y623","MYH4",0.9318,"Cardiometabolic_II","B13805","20212097_SS211647","PASS",0.4263,-0.6938,"Plate control","PASS","PFC",0.618223328471622,"1h","1h"
328,"PFC_25",4,"OID30061","Q9Y623","MYH4",0.9318,"Cardiometabolic_II","B13805","20212097_SS211647","PASS",0.4263,-0.2019,"Plate control","PASS","PFC",0.869404820551542,"24h","24h"
329,"PFC_02",13,"OID30061","Q9Y623","MYH4",0.9318,"Cardiometabolic_II","B13805","20212097_SS211647","PASS",0.4263,-0.2186,"Plate control","PASS","PFC",0.859398997915123,"Baseline","Baseline"
330,"PFC_10",14,"OID30061","Q9Y623","MYH4",0.9318,"Cardiometabolic_II","B13805","20212097_SS211647","PASS",0.4263,0.3602,"Plate control","PASS","PFC",1.28360383050446,"1h","1h"
331,"PFC_18",15,"OID30061","Q9Y623","MYH4",0.9318,"Cardiometabolic_II","B13805","20212097_SS211647","PASS",0.4263,-0.0746,"Plate control","PASS","PFC",0.949605370968844,"1h","1h"
332,"PFC_26",16,"OID30061","Q9Y623","MYH4",0.9318,"Cardiometabolic_II","B13805","20212097_SS211647","PASS",0.4263,-0.5129,"Plate control","PASS","PFC",0.700812299463822,"24h","24h"
333,"PFC_03",25,"OID30061","Q9Y623","MYH4",0.9318,"Cardiometabolic_II","B13805","20212097_SS211647","PASS",0.4263,0.5072,"Plate control","PASS","PFC",1.42128906202596,"Baseline","Baseline"
334,"PFC_11",26,"OID30061","Q9Y623","MYH4",0.9318,"Cardiometabolic_II","B13805","20212097_SS211647","PASS",0.4263,-0.3522,"Plate control","PASS","PFC",0.783388578704442,"1h","1h"
335,"PFC_19",27,"OID30061","Q9Y623","MYH4",0.9318,"Cardiometabolic_II","B13805","20212097_SS211647","PASS",0.4263,-0.2549,"Plate control","PASS","PFC",0.838045221523036,"24h","24h"
336,"PFC_27",28,"OID30061","Q9Y623","MYH4",0.9318,"Cardiometabolic_II","B13805","20212097_SS211647","PASS",0.4263,-0.1487,"Plate control","PASS","PFC",0.902062937595217,"24h","24h"

Supplemental table 1_Olink proximity extension assay (PEA) data

,"SampleID","Index","OlinkID","UniProt","Assay","MissingFreq","Panel","Panel_Lot_Nr","PlateID","QC_Warning","LOD","NPX","Normalization","Assay_Warning","condition","Value","group","alt_group"
337,"PFC_04",37,"OID30061","Q9Y623","MYH4",0.9318,"Cardiometabolic_II","B13805","20212097_SS211647","PASS",0.4263,-0.2995,"Plate control","PASS","PFC",0.812533950372229,"Baseline","Baseline"
338,"PFC_12",38,"OID30061","Q9Y623","MYH4",0.9318,"Cardiometabolic_II","B13805","20212097_SS211647","PASS",0.4263,0.5435,"Plate control","PASS","PFC",1.45750415882451,"1h","1h"
339,"PFC_20",39,"OID30061","Q9Y623","MYH4",0.9318,"Cardiometabolic_II","B13805","20212097_SS211647","PASS",0.4263,-0.1251,"Plate control","PASS","PFC",0.916940483530796,"24h","24h"
340,"PFC_05",49,"OID30061","Q9Y623","MYH4",0.9318,"Cardiometabolic_II","B13805","20212097_SS211647","PASS",0.4263,-0.1747,"Plate control","PASS","PFC",0.885951728431873,"Baseline","Baseline"
341,"PFC_13",50,"OID30061","Q9Y623","MYH4",0.9318,"Cardiometabolic_II","B13805","20212097_SS211647","PASS",0.4263,0.0869,"Plate control","PASS","PFC",1.06208556578236,"1h","1h"
342,"PFC_21",51,"OID30061","Q9Y623","MYH4",0.9318,"Cardiometabolic_II","B13805","20212097_SS211647","PASS",0.4263,0.3222,"Plate control","PASS","PFC",1.2502356100298,"24h","24h"
343,"PFC_06",61,"OID30061","Q9Y623","MYH4",0.9318,"Cardiometabolic_II","B13805","20212097_SS211647","PASS",0.4263,-0.2161,"Plate control","PASS","PFC",0.860889513956834,"Baseline","Baseline"
344,"PFC_14",62,"OID30061","Q9Y623","MYH4",0.9318,"Cardiometabolic_II","B13805","20212097_SS211647","PASS",0.4263,-0.1831,"Plate control","PASS","PFC",0.8808083190307,"1h","1h"
345,"PFC_22",63,"OID30061","Q9Y623","MYH4",0.9318,"Cardiometabolic_II","B13805","20212097_SS211647","PASS",0.4263,-0.2512,"Plate control","PASS","PFC",0.840197268085905,"24h","24h"
346,"PFC_07",73,"OID30061","Q9Y623","MYH4",0.9318,"Cardiometabolic_II","B13805","20212097_SS211647","PASS",0.4263,-0.436,"Plate control","PASS","PFC",0.739181215636921,"Baseline","Baseline"
347,"PFC_15",74,"OID30061","Q9Y623","MYH4",0.9318,"Cardiometabolic_II","B13805","20212097_SS211647","PASS",0.4263,0.1878,"Plate control","PASS","PFC",1.13902546381888,"1h","1h"
348,"PFC_23",75,"OID30061","Q9Y623","MYH4",0.9318,"Cardiometabolic_II","B13805","20212097_SS211647","PASS",0.4263,-0.1029,"Plate control","PASS","PFC",0.931159360641937,"24h","24h"
349,"PFC_08",85,"OID30061","Q9Y623","MYH4",0.9318,"Cardiometabolic_II","B13805","20212097_SS211647","PASS",0.4263,0.0552,"Plate control","PASS","PFC",1.03900312973228,"Baseline","Baseline"
350,"PFC_16",86,"OID30061","Q9Y623","MYH4",0.9318,"Cardiometabolic_II","B13805","20212097_SS211647","PASS",0.4263,-0.4825,"Plate control","PASS","PFC",0.715736272321307,"1h","1h"
351,"PFC_24",87,"OID30061","Q9Y623","MYH4",0.9318,"Cardiometabolic_II","B13805","20212097_SS211647","PASS",0.4263,-0.4091,"Plate control","PASS","PFC",0.753093031076616,"24h","24h"
352,"PFC_01",1,"OID30062","P23634","ATP2B4",0.9773,"Cardiometabolic_II","B13805","20212097_SS211647","WARN",1.0051,-0.3976,"Plate control","PASS","PFC",0.759120070086516,"Baseline","Baseline"
353,"PFC_09",2,"OID30062","P23634","ATP2B4",0.9773,"Cardiometabolic_II","B13805","20212097_SS211647","PASS",1.0051,0.6259,"Plate control","PASS","PFC",1.543173206736,"Baseline","Baseline"
354,"PFC_17",3,"OID30062","P23634","ATP2B4",0.9773,"Cardiometabolic_II","B13805","20212097_SS211647","PASS",1.0051,-0.396,"Plate control","PASS","PFC",0.759962428200758,"1h","1h"
355,"PFC_25",4,"OID30062","P23634","ATP2B4",0.9773,"Cardiometabolic_II","B13805","20212097_SS211647","PASS",1.0051,0.4536,"Plate control","PASS","PFC",1.36945323426477,"24h","24h"
356,"PFC_02",13,"OID30062","P23634","ATP2B4",0.9773,"Cardiometabolic_II","B13805","20212097_SS211647","PASS",1.0051,-0.8288,"Plate control","PASS","PFC",0.562997335661931,"Baseline","Baseline"
357,"PFC_10",14,"OID30062","P23634","ATP2B4",0.9773,"Cardiometabolic_II","B13805","20212097_SS211647","PASS",1.0051,0.04,"Plate control","PASS","PFC",1.02811382665607,"1h","1h"
358,"PFC_18",15,"OID30062","P23634","ATP2B4",0.9773,"Cardiometabolic_II","B13805","20212097_SS211647","PASS",1.0051,0.1096,"Plate control","PASS","PFC",1.07892905237835,"1h","1h"
359,"PFC_26",16,"OID30062","P23634","ATP2B4",0.9773,"Cardiometabolic_II","B13805","20212097_SS211647","PASS",1.0051,0.0621,"Plate control","PASS","PFC",1.04398428839045,"24h","24h"
360,"PFC_03",25,"OID30062","P23634","ATP2B4",0.9773,"Cardiometabolic_II","B13805","20212097_SS211647","PASS",1.0051,0.5207,"Plate control","PASS","PFC",1.43465117626147,"Baseline","Baseline"
361,"PFC_11",26,"OID30062","P23634","ATP2B4",0.9773,"Cardiometabolic_II","B13805","20212097_SS211647","PASS",1.0051,-0.0085,"Plate control","PASS","PFC",0.994125571294056,"1h","1h"
362,"PFC_19",27,"OID30062","P23634","ATP2B4",0.9773,"Cardiometabolic_II","B13805","20212097_SS211647","PASS",1.0051,0.3877,"Plate control","PASS","PFC",1.30830598858478,"24h","24h"
363,"PFC_27",28,"OID30062","P23634","ATP2B4",0.9773,"Cardiometabolic_II","B13805","20212097_SS211647","PASS",1.0051,0.123,"Plate control","PASS","PFC",1.08899701533611,"24h","24h"
364,"PFC_04",37,"OID30062","P23634","ATP2B4",0.9773,"Cardiometabolic_II","B13805","20212097_SS211647","PASS",1.0051,0.5458,"Plate control","PASS","PFC",1.45982962126907,"Baseline","Baseline"
365,"PFC_12",38,"OID30062","P23634","ATP2B4",0.9773,"Cardiometabolic_II","B13805","20212097_SS211647","PASS",1.0051,1.3815,"Plate control","PASS","PFC",2.60539118246131,"1h","1h"
366,"PFC_20",39,"OID30062","P23634","ATP2B4",0.9773,"Cardiometabolic_II","B13805","20212097_SS211647","PASS",1.0051,0.4511,"Plate control","PASS","PFC",1.36708220757557,"24h","24h"
367,"PFC_05",49,"OID30062","P23634","ATP2B4",0.9773,"Cardiometabolic_II","B13805","20212097_SS211647","PASS",1.0051,-0.6029,"Plate control","PASS","PFC",0.658429098275244,"Baseline","Baseline"
368,"PFC_13",50,"OID30062","P23634","ATP2B4",0.9773,"Cardiometabolic_II","B13805","20212097_SS211647","PASS",1.0051,0.4112,"Plate control","PASS","PFC",1.32979144364224,"1h","1h"
369,"PFC_21",51,"OID30062","P23634","ATP2B4",0.9773,"Cardiometabolic_II","B13805","20212097_SS211647","PASS",1.0051,0.5042,"Plate control","PASS","PFC",1.41833664526056,"24h","24h"
370,"PFC_06",61,"OID30062","P23634","ATP2B4",0.9773,"Cardiometabolic_II","B13805","20212097_SS211647","PASS",1.0051,0.1417,"Plate control","PASS","PFC",1.10320431130122,"Baseline","Baseline"
371,"PFC_14",62,"OID30062","P23634","ATP2B4",0.9773,"Cardiometabolic_II","B13805","20212097_SS211647","PASS",1.0051,0.1232,"Plate control","PASS","PFC",1.08914799284299,"1h","1h"
372,"PFC_22",63,"OID30062","P23634","ATP2B4",0.9773,"Cardiometabolic_II","B13805","20212097_SS211647","PASS",1.0051,0.1258,"Plate control","PASS","PFC",1.09111260624455,"24h","24h"
373,"PFC_07",73,"OID30062","P23634","ATP2B4",0.9773,"Cardiometabolic_II","B13805","20212097_SS211647","PASS",1.0051,0.4759,"Plate control","PASS","PFC",1.39078556645946,"Baseline","Baseline"
374,"PFC_15",74,"OID30062","P23634","ATP2B4",0.9773,"Cardiometabolic_II","B13805","20212097_SS211647","PASS",1.0051,-0.0491,"Plate control","PASS","PFC",0.966539099357874,"1h","1h"
375,"PFC_23",75,"OID30062","P23634","ATP2B4",0.9773,"Cardiometabolic_II","B13805","20212097_SS211647","PASS",1.0051,0.1435,"Plate control","PASS","PFC",1.10458159964406,"24h","24h"
376,"PFC_08",85,"OID30062","P23634","ATP2B4",0.9773,"Cardiometabolic_II","B13805","20212097_SS211647","PASS",1.0051,0.2621,"Plate control","PASS","PFC",1.19922303471687,"Baseline","Baseline"
377,"PFC_16",86,"OID30062","P23634","ATP2B4",0.9773,"Cardiometabolic_II","B13805","20212097_SS211647","PASS",1.0051,-0.1595,"Plate control","PASS","PFC",0.895335316738591,"1h","1h"
378,"PFC_24",87,"OID30062","P23634","ATP2B4",0.9773,"Cardiometabolic_II","B13805","20212097_SS211647","PASS",1.0051,0.324,"Plate control","PASS","PFC",1.25179645865399,"24h","24h"
379,"PFC_01",1,"OID30063","O14958","CASQ2",0.6136,"Cardiometabolic_II","B13805","20212097_SS211647","WARN",0.4892,0.5809,"Plate control","PASS","PFC",1.49578207505624,"Baseline","Baseline"
380,"PFC_09",2,"OID30063","O14958","CASQ2",0.6136,"Cardiometabolic_II","B13805","20212097_SS211647","PASS",0.4892,0.2628,"Plate control","PASS","PFC",1.19980504254719,"Baseline","Baseline"
381,"PFC_17",3,"OID30063","O14958","CASQ2",0.6136,"Cardiometabolic_II","B13805","20212097_SS211647","PASS",0.4892,0.6469,"Plate control","PASS","PFC",1.56580005576213,"1h","1h"
382,"PFC_25",4,"OID30063","O14958","CASQ2",0.6136,"Cardiometabolic_II","B13805","20212097_SS211647","PASS",0.4892,1.8541,"Plate control","PASS","PFC",3.61526148915055,"24h","24h"
383,"PFC_02",13,"OID30063","O14958","CASQ2",0.6136,"Cardiometabolic_II","B13805","20212097_SS211647","PASS",0.4892,-0.0477,"Plate control","PASS","PFC",0.967477489986477,"Baseline","Baseline"
384,"PFC_10",14,"OID30063","O14958","CASQ2",0.6136,"Cardiometabolic_II","B13805","20212097_SS211647","PASS",0.4892,0.5673,"Plate control","PASS","PFC",1.48174788679759,"1h","1h"
385,"PFC_18",15,"OID30063","O14958","CASQ2",0.6136,"Cardiometabolic_II","B13805","20212097_SS211647","PASS",0.4892,-0.0426,"Plate control","PASS","PFC",0.970903624098965,"1h","1h"
386,"PFC_26",16,"OID30063","O14958","CASQ2",0.6136,"Cardiometabolic_II","B13805","20212097_SS211647","PASS",0.4892,0.082,"Plate control","PASS","PFC",1.05848439487513,"24h","24h"
387,"PFC_03",25,"OID30063","O14958","CASQ2",0.6136,"Cardiometabolic_II","B13805","20212097_SS211647","PASS",0.4892,-0.4652,"Plate control","PASS","PFC",0.724370651221682,"Baseline","Baseline"
388,"PFC_11",26,"OID30063","O14958","CASQ2",0.6136,"Cardiometabolic_II","B13805","20212097_SS211647","PASS",0.4892,-0.0576,"Plate control","PASS","PFC",0.960861234327189,"1h","1h"
389,"PFC_19",27,"OID30063","O14958","CASQ2",0.6136,"Cardiometabolic_II","B13805","20212097_SS211647","PASS",0.4892,0.2449,"Plate control","PASS","PFC",1.18501062941714,"24h","24h"
390,"PFC_27",28,"OID30063","O14958","CASQ2",0.6136,"Cardiometabolic_II","B13805","20212097_SS211647","PASS",0.4892,0.6478,"Plate control","PASS","PFC",1.56677715740888,"24h","24h"
391,"PFC_04",37,"OID30063","O14958","CASQ2",0.6136,"Cardiometabolic_II","B13805","20212097_SS211647","PASS",0.4892,0.2969,"Plate control","PASS","PFC",1.22850182423682,"Baseline","Baseline"
392,"PFC_12",38,"OID30063","O14958","CASQ2",0.6136,"Cardiometabolic_II","B13805","20212097_SS211647","PASS",0.4892,-0.4388,"Plate control","PASS","PFC",0.737747995041228,"1h","1h"
393,"PFC_20",39,"OID30063","O14958","CASQ2",0.6136,"Cardiometabolic_II","B13805","20212097_SS211647","PASS",0.4892,0.0972,"Plate control","PASS","PFC",1.06969536887275,"24h","24h"
394,"PFC_05",49,"OID30063","O14958","CASQ2",0.6136,"Cardiometabolic_II","B13805","20212097_SS211647","PASS",0.4892,-0.0504,"Plate control","PASS","PFC",0.965668551631178,"Baseline","Baseline"
395,"PFC_13",50,"OID30063","O14958","CASQ2",0.6136,"Cardiometabolic_II","B13805","20212097_SS211647","PASS",0.4892,0.3396,"Plate control","PASS","PFC",1.26540570037096,"1h","1h"
396,"PFC_21",51,"OID30063","O14958","CASQ2",0.6136,"Cardiometabolic_II","B13805","20212097_SS211647","PASS",0.4892,0.178,"Plate control","PASS","PFC",1.1313144628459,"24h","24h"
397,"PFC_06",61,"OID30063","O14958","CASQ2",0.6136,"Cardiometabolic_II","B13805","20212097_SS211647","PASS",0.4892,0.5902,"Plate control","PASS","PFC",1.50545543338355,"Baseline","Baseline"
398,"PFC_14",62,"OID30063","O14958","CASQ2",0.6136,"Cardiometabolic_II","B13805","20212097_SS211647","PASS",0.4892,0.3088,"Plate control","PASS","PFC",1.23867696879113,"1h","1h"
399,"PFC_22",63,"OID30063","O14958","CASQ2",0.6136,"Cardiometabolic_II","B13805","20212097_SS211647","PASS",0.4892,0.906,"Plate control","PASS","PFC",1.87384289374485,"24h","24h"
400,"PFC_07",73,"OID30063","O14958","CASQ2",0.6136,"Cardiometabolic_II","B13805","20212097_SS211647","PASS",0.4892,1.9216,"Plate control","PASS","PFC",3.78842976100758,"Baseline","Baseline"
401,"PFC_15",74,"OID30063","O14958","CASQ2",0.6136,"Cardiometabolic_II","B13805","20212097_SS211647","PASS",0.4892,-0.0986,"Plate control","PASS","PFC",0.933938851854323,"1h","1h"
402,"PFC_23",75,"OID30063","O14958","CASQ2",0.6136,"Cardiometabolic_II","B13805","20212097_SS211647","PASS",0.4892,0.8131,"Plate control","PASS","PFC",1.75698271701774,"24h","24h"
403,"PFC_08",85,"OID30063","O14958","CASQ2",0.6136,"Cardiometabolic_II","B13805","20212097_SS211647","PASS",0.4892,0.1247,"Plate control","PASS","PFC",1.09028099153274,"Baseline","Baseline"
404,"PFC_16",86,"OID30063","O14958","CASQ2",0.6136,"Cardiometabolic_II","B13805","20212097_SS211647","PASS",0.4892,2.1108,"Plate control","PASS","PFC",4.31930741467384,"1h","1h"
405,"PFC_24",87,"OID30063","O14958","CASQ2",0.6136,"Cardiometabolic_II","B13805","20212097_SS211647","PASS",0.4892,-0.0492,"Plate control","PASS","PFC",0.966472106294542,"24h","24h"
406,"PFC_01",1,"OID30064","O95180","CACNA1H",0.8864,"Cardiometabolic_II","B13805","20212097_SS211647","WARN",1.3945,0.0231,"Plate control","PASS","PFC",1.01614057405011,"Baseline","Baseline"
407,"PFC_09",2,"OID30064","O95180","CACNA1H",0.8864,"Cardiometabolic_II","B13805","20212097_SS211647","PASS",1.3945,0.8414,"Plate control","PASS","PFC",1.79178806045537,"Baseline","Baseline"
408,"PFC_17",3,"OID30064","O95180","CACNA1H",0.8864,"Cardiometabolic_II","B13805","20212097_SS211647","PASS",1.3945,1.1224,"Plate control","PASS","PFC",2.17708841674941,"1h","1h"
409,"PFC_25",4,"OID30064","O95180","CACNA1H",0.8864,"Cardiometabolic_II","B13805","20212097_SS211647","PASS",1.3945,1.3001,"Plate control","PASS","PFC",2.46245950546083,"24h","24h"
410,"PFC_02",13,"OID30064","O95180","CACNA1H",0.8864,"Cardiometabolic_II","B13805","20212097_SS211647","PASS",1.3945,0.5582,"Plate control","PASS","PFC",1.47243097029528,"Baseline","Baseline"
411,"PFC_10",14,"OID30064","O95180","CACNA1H",0.8864,"Cardiometabolic_II","B13805","20212097_SS211647","PASS",1.3945,0.3508,"Plate control","PASS","PFC",1.27526758979699,"1h","1h"
412,"PFC_18",15,"OID30064","O95180","CACNA1H",0.8864,"Cardiometabolic_II","B13805","20212097_SS211647","PASS",1.3945,0.5893,"Plate control","PASS","PFC",1.50451657428919,"1h","1h"
413,"PFC_26",16,"OID30064","O95180","CACNA1H",0.8864,"Cardiometabolic_II","B13805","20212097_SS211647","PASS",1.3945,1.0154,"Plate control","PASS","PFC",2.0214632839136,"24h","24h"
414,"PFC_03",25,"OID30064","O95180","CACNA1H",0.8864,"Cardiometabolic_II","B13805","20212097_SS211647","PASS",1.3945,1.8521,"Plate control","PASS","PFC",3.61025314485598,"Baseline","Baseline"
415,"PFC_11",26,"OID30064","O95180","CACNA1H",0.8864,"Cardiometabolic_II","B13805","20212097_SS211647","PASS",1.3945,1.0364,"Plate control","PASS","PFC",2.05110308347552,"1h","1h"
416,"PFC_19",27,"OID30064","O95180","CACNA1H",0.8864,"Cardiometabolic_II","B13805","20212097_SS211647","PASS",1.3945,0.9093,"Plate control","PASS","PFC",1.87813400101399,"24h","24h"
417,"PFC_27",28,"OID30064","O95180","CACNA1H",0.8864,"Cardiometabolic_II","B13805","20212097_SS211647","PASS",1.3945,0.7808,"Plate control","PASS","PFC",1.71808331646309,"24h","24h"
418,"PFC_04",37,"OID30064","O95180","CACNA1H",0.8864,"Cardiometabolic_II","B13805","20212097_SS211647","PASS",1.3945,0.5254,"Plate control","PASS","PFC",1.43933259242473,"Baseline","Baseline"
419,"PFC_12",38,"OID30064","O95180","CACNA1H",0.8864,"Cardiometabolic_II","B13805","20212097_SS211647","PASS",1.3945,1.6321,"Plate control","PASS","PFC",3.09963856696405,"1h","1h"
420,"PFC_20",39,"OID30064","O95180","CACNA1H",0.8864,"Cardiometabolic_II","B13805","20212097_SS211647","PASS",1.3945,0.9142,"Plate control","PASS","PFC",1.88452378017094,"24h","24h"
421,"PFC_05",49,"OID30064","O95180","CACNA1H",0.8864,"Cardiometabolic_II","B13805","20212097_SS211647","PASS",1.3945,1.0352,"Plate control","PASS","PFC",2.04939773322585,"Baseline","Baseline"
422,"PFC_13",50,"OID30064","O95180","CACNA1H",0.8864,"Cardiometabolic_II","B13805","20212097_SS211647","PASS",1.3945,0.7132,"Plate control","PASS","PFC",1.63943647367621,"1h","1h"
423,"PFC_21",51,"OID30064","O95180","CACNA1H",0.8864,"Cardiometabolic_II","B13805","20212097_SS211647","PASS",1.3945,1.5807,"Plate control","PASS","PFC",2.99114946000604,"24h","24h"
424,"PFC_06",61,"OID30064","O95180","CACNA1H",0.8864,"Cardiometabolic_II","B13805","20212097_SS211647","PASS",1.3945,0.2582,"Plate control","PASS","PFC",1.19598558409148,"Baseline","Baseline"
425,"PFC_14",62,"OID30064","O95180","CACNA1H",0.8864,"Cardiometabolic_II","B13805","20212097_SS211647","PASS",1.3945,0.677,"Plate control","PASS","PFC",1.59881166070494,"1h","1h"
426,"PFC_22",63,"OID30064","O95180","CACNA1H",0.8864,"Cardiometabolic_II","B13805","20212097_SS211647","PASS",1.3945,0.6183,"Plate control","PASS","PFC",1.53506527066378,"24h","24h"
427,"PFC_07",73,"OID30064","O95180","CACNA1H",0.8864,"Cardiometabolic_II","B13805","20212097_SS211647","PASS",1.3945,0.9088,"Plate control","PASS","PFC",1.8774832021516,"Baseline","Baseline"
428,"PFC_15",74,"OID30064","O95180","CACNA1H",0.8864,"Cardiometabolic_II","B13805","20212097_SS211647","PASS",1.3945,0.6442,"Plate control","PASS","PFC",1.56287240544788,"1h","1h"
429,"PFC_23",75,"OID30064","O95180","CACNA1H",0.8864,"Cardiometabolic_II","B13805","20212097_SS211647","PASS",1.3945,0.1816,"Plate control","PASS","PFC",1.13414099068779,"24h","24h"
430,"PFC_08",85,"OID30064","O95180","CACNA1H",0.8864,"Cardiometabolic_II","B13805","20212097_SS211647","PASS",1.3945,1.5048,"Plate control","PASS","PFC",2.8378532751311,"Baseline","Baseline"
431,"PFC_16",86,"OID30064","O95180","CACNA1H",0.8864,"Cardiometabolic_II","B13805","20212097_SS211647","PASS",1.3945,0.9697,"Plate control","PASS","PFC",1.95843330807109,"1h","1h"
432,"PFC_24",87,"OID30064","O95180","CACNA1H",0.8864,"Cardiometabolic_II","B13805","20212097_SS211647","PASS",1.3945,0.1974,"Plate control","PASS","PFC",1.14663005501621,"24h","24h"
433,"PFC_01",1,"OID30065","P54709","ATP1B3",0.6591,"Cardiometabolic_II","B13805","20212097_SS211647","WARN",0.0371,-0.6644,"Plate control","PASS","PFC",0.630951055024485,"Baseline","Baseline"
434,"PFC_09",2,"OID30065","P54709","ATP1B3",0.6591,"Cardiometabolic_II","B13805","20212097_SS211647","PASS",0.0371,-0.7332,"Plate control","PASS","PFC",0.601568112170807,"Baseline","Baseline"
435,"PFC_17",3,"OID30065","P54709","ATP1B3",0.6591,"Cardiometabolic_II","B13805","20212097_SS211647","PASS",0.0371,-0.2734,"Plate control","PASS","PFC",0.827367389325094,"1h","1h"
436,"PFC_25",4,"OID30065","P54709","ATP1B3",0.6591,"Cardiometabolic_II","B13805","20212097_SS211647","PASS",0.0371,0.3583,"Plate control","PASS","PFC",1.28191446306537,"24h","24h"
437,"PFC_02",13,"OID30065","P54709","ATP1B3",0.6591,"Cardiometabolic_II","B13805","20212097_SS211647","PASS",0.0371,-0.3367,"Plate control","PASS","PFC",0.791850509436757,"Baseline","Baseline"
438,"PFC_10",14,"OID30065","P54709","ATP1B3",0.6591,"Cardiometabolic_II","B13805","20212097_SS211647","PASS",0.0371,-0.0532,"Plate control","PASS","PFC",0.963796187956976,"1h","1h"
439,"PFC_18",15,"OID30065","P54709","ATP1B3",0.6591,"Cardiometabolic_II","B13805","20212097_SS211647","PASS",0.0371,-0.1965,"Plate control","PASS","PFC",0.872665096042938,"1h","1h"
440,"PFC_26",16,"OID30065","P54709","ATP1B3",0.6591,"Cardiometabolic_II","B13805","20212097_SS211647","PASS",0.0371,-0.3764,"Plate control","PASS","PFC",0.770357490297056,"24h","24h"
441,"PFC_03",25,"OID30065","P54709","ATP1B3",0.6591,"Cardiometabolic_II","B13805","20212097_SS211647","PASS",0.0371,-0.1992,"Plate control","PASS","PFC",0.871033432898419,"Baseline","Baseline"
442,"PFC_11",26,"OID30065","P54709","ATP1B3",0.6591,"Cardiometabolic_II","B13805","20212097_SS211647","PASS",0.0371,0.2317,"Plate control","PASS","PFC",1.17421777410301,"1h","1h"
443,"PFC_19",27,"OID30065","P54709","ATP1B3",0.6591,"Cardiometabolic_II","B13805","20212097_SS211647","PASS",0.0371,-0.6578,"Plate control","PASS","PFC",0.633844124380393,"24h","24h"
444,"PFC_27",28,"OID30065","P54709","ATP1B3",0.6591,"Cardiometabolic_II","B13805","20212097_SS211647","PASS",0.0371,-0.0509,"Plate control","PASS","PFC",0.965333934402316,"24h","24h"
445,"PFC_04",37,"OID30065","P54709","ATP1B3",0.6591,"Cardiometabolic_II","B13805","20212097_SS211647","PASS",0.0371,0.0457,"Plate control","PASS","PFC",1.03218387656232,"Baseline","Baseline"
446,"PFC_12",38,"OID30065","P54709","ATP1B3",0.6591,"Cardiometabolic_II","B13805","20212097_SS211647","PASS",0.0371,0.1062,"Plate control","PASS","PFC",1.07638933368755,"1h","1h"
447,"PFC_20",39,"OID30065","P54709","ATP1B3",0.6591,"Cardiometabolic_II","B13805","20212097_SS211647","PASS",0.0371,0.3039,"Plate control","PASS","PFC",1.2344770365002,"24h","24h"
448,"PFC_05",49,"OID30065","P54709","ATP1B3",0.6591,"Cardiometabolic_II","B13805","20212097_SS211647","PASS",0.0371,0.3523,"Plate control","PASS","PFC",1.27659420153186,"Baseline","Baseline"

Supplemental table 1_Olink proximity extension assay (PEA) data

,"SampleID","Index","OlinkID","UniProt","Assay","MissingFreq","Panel","Panel_Lot_Nr","PlateID","QC_Warning","LOD","NPX","Normalization","Assay_Warning","condition","Value","group","alt_group"
449,"PFC_13",50,"OID30065","P54709","ATP1B3",0.6591,"Cardiometabolic_II","B13805","20212097_SS211647","PASS",0.0371,0.048,"Plate control","PASS","PFC",1.03383073624796,"1h","1h"
450,"PFC_21",51,"OID30065","P54709","ATP1B3",0.6591,"Cardiometabolic_II","B13805","20212097_SS211647","PASS",0.0371,-0.266,"Plate control","PASS","PFC",0.831622098374906,"24h","24h"
451,"PFC_06",61,"OID30065","P54709","ATP1B3",0.6591,"Cardiometabolic_II","B13805","20212097_SS211647","PASS",0.0371,-0.2678,"Plate control","PASS","PFC",0.830585159662447,"Baseline","Baseline"
452,"PFC_14",62,"OID30065","P54709","ATP1B3",0.6591,"Cardiometabolic_II","B13805","20212097_SS211647","PASS",0.0371,0.7035,"Plate control","PASS","PFC",1.6284506503414,"1h","1h"
453,"PFC_22",63,"OID30065","P54709","ATP1B3",0.6591,"Cardiometabolic_II","B13805","20212097_SS211647","PASS",0.0371,-0.2382,"Plate control","PASS","PFC",0.84780242611392,"24h","24h"
454,"PFC_07",73,"OID30065","P54709","ATP1B3",0.6591,"Cardiometabolic_II","B13805","20212097_SS211647","PASS",0.0371,0.0557,"Plate control","PASS","PFC",1.03936328318349,"Baseline","Baseline"
455,"PFC_15",74,"OID30065","P54709","ATP1B3",0.6591,"Cardiometabolic_II","B13805","20212097_SS211647","PASS",0.0371,-0.1785,"Plate control","PASS","PFC",0.883621238205257,"1h","1h"
456,"PFC_23",75,"OID30065","P54709","ATP1B3",0.6591,"Cardiometabolic_II","B13805","20212097_SS211647","PASS",0.0371,0.6456,"Plate control","PASS","PFC",1.56438976239696,"24h","24h"
457,"PFC_08",85,"OID30065","P54709","ATP1B3",0.6591,"Cardiometabolic_II","B13805","20212097_SS211647","PASS",0.0371,0.067,"Plate control","PASS","PFC",1.0475361270998,"Baseline","Baseline"
458,"PFC_16",86,"OID30065","P54709","ATP1B3",0.6591,"Cardiometabolic_II","B13805","20212097_SS211647","PASS",0.0371,-0.6462,"Plate control","PASS","PFC",0.638961096644901,"1h","1h"
459,"PFC_24",87,"OID30065","P54709","ATP1B3",0.6591,"Cardiometabolic_II","B13805","20212097_SS211647","PASS",0.0371,-0.677,"Plate control","PASS","PFC",0.625464540056633,"24h","24h"
460,"PFC_01",1,"OID30087","P35228","NOS2",0.2955,"Cardiometabolic_II","B13805","20212097_SS211647","WARN",-0.4366,-0.2469,"Plate control","PASS","PFC",0.842705239354867,"Baseline","Baseline"
461,"PFC_09",2,"OID30087","P35228","NOS2",0.2955,"Cardiometabolic_II","B13805","20212097_SS211647","PASS",-0.4366,-0.6322,"Plate control","PASS","PFC",0.64519179645099,"Baseline","Baseline"
462,"PFC_17",3,"OID30087","P35228","NOS2",0.2955,"Cardiometabolic_II","B13805","20212097_SS211647","PASS",-0.4366,-1.5099,"Plate control","PASS","PFC",0.351135556953128,"1h","1h"
463,"PFC_25",4,"OID30087","P35228","NOS2",0.2955,"Cardiometabolic_II","B13805","20212097_SS211647","PASS",-0.4366,-0.0767,"Plate control","PASS","PFC",0.948224122292167,"24h","24h"
464,"PFC_02",13,"OID30087","P35228","NOS2",0.2955,"Cardiometabolic_II","B13805","20212097_SS211647","PASS",-0.4366,0.1251,"Plate control","PASS","PFC",1.09058332352103,"Baseline","Baseline"
465,"PFC_10",14,"OID30087","P35228","NOS2",0.2955,"Cardiometabolic_II","B13805","20212097_SS211647","PASS",-0.4366,-0.0658,"Plate control","PASS","PFC",0.955415375897031,"1h","1h"
466,"PFC_18",15,"OID30087","P35228","NOS2",0.2955,"Cardiometabolic_II","B13805","20212097_SS211647","PASS",-0.4366,-0.9804,"Plate control","PASS","PFC",0.506839194748686,"1h","1h"
467,"PFC_26",16,"OID30087","P35228","NOS2",0.2955,"Cardiometabolic_II","B13805","20212097_SS211647","PASS",-0.4366,-0.5961,"Plate control","PASS","PFC",0.661539863915679,"24h","24h"
468,"PFC_03",25,"OID30087","P35228","NOS2",0.2955,"Cardiometabolic_II","B13805","20212097_SS211647","PASS",-0.4366,-0.5571,"Plate control","PASS","PFC",0.679667008393533,"Baseline","Baseline"
469,"PFC_11",26,"OID30087","P35228","NOS2",0.2955,"Cardiometabolic_II","B13805","20212097_SS211647","PASS",-0.4366,0.1586,"Plate control","PASS","PFC",1.11620344176851,"1h","1h"
470,"PFC_19",27,"OID30087","P35228","NOS2",0.2955,"Cardiometabolic_II","B13805","20212097_SS211647","PASS",-0.4366,-0.1073,"Plate control","PASS","PFC",0.928323792728746,"24h","24h"
471,"PFC_27",28,"OID30087","P35228","NOS2",0.2955,"Cardiometabolic_II","B13805","20212097_SS211647","PASS",-0.4366,-0.2409,"Plate control","PASS","PFC",0.846217249877588,"24h","24h"
472,"PFC_04",37,"OID30087","P35228","NOS2",0.2955,"Cardiometabolic_II","B13805","20212097_SS211647","PASS",-0.4366,-0.2842,"Plate control","PASS","PFC",0.821196850804407,"Baseline","Baseline"
473,"PFC_12",38,"OID30087","P35228","NOS2",0.2955,"Cardiometabolic_II","B13805","20212097_SS211647","PASS",-0.4366,-0.3302,"Plate control","PASS","PFC",0.795426206624774,"1h","1h"
474,"PFC_20",39,"OID30087","P35228","NOS2",0.2955,"Cardiometabolic_II","B13805","20212097_SS211647","PASS",-0.4366,0.003,"Plate control","PASS","PFC",1.00208160507963,"24h","24h"
475,"PFC_05",49,"OID30087","P35228","NOS2",0.2955,"Cardiometabolic_II","B13805","20212097_SS211647","PASS",-0.4366,0.1904,"Plate control","PASS","PFC",1.14108004658048,"Baseline","Baseline"
476,"PFC_13",50,"OID30087","P35228","NOS2",0.2955,"Cardiometabolic_II","B13805","20212097_SS211647","PASS",-0.4366,-0.6565,"Plate control","PASS","PFC",0.634415533235561,"1h","1h"
477,"PFC_21",51,"OID30087","P35228","NOS2",0.2955,"Cardiometabolic_II","B13805","20212097_SS211647","PASS",-0.4366,-1.2659,"Plate control","PASS","PFC",0.415839872012004,"24h","24h"
478,"PFC_06",61,"OID30087","P35228","NOS2",0.2955,"Cardiometabolic_II","B13805","20212097_SS211647","PASS",-0.4366,-0.1222,"Plate control","PASS","PFC",0.918785503933658,"Baseline","Baseline"
479,"PFC_14",62,"OID30087","P35228","NOS2",0.2955,"Cardiometabolic_II","B13805","20212097_SS211647","PASS",-0.4366,0.0088,"Plate control","PASS","PFC",1.00611833621187,"1h","1h"
480,"PFC_22",63,"OID30087","P35228","NOS2",0.2955,"Cardiometabolic_II","B13805","20212097_SS211647","PASS",-0.4366,-0.7655,"Plate control","PASS","PFC",0.588249461549154,"24h","24h"
481,"PFC_07",73,"OID30087","P35228","NOS2",0.2955,"Cardiometabolic_II","B13805","20212097_SS211647","PASS",-0.4366,-0.0027,"Plate control","PASS","PFC",0.998130252771747,"Baseline","Baseline"
482,"PFC_15",74,"OID30087","P35228","NOS2",0.2955,"Cardiometabolic_II","B13805","20212097_SS211647","PASS",-0.4366,-0.8167,"Plate control","PASS","PFC",0.567739096825422,"1h","1h"
483,"PFC_23",75,"OID30087","P35228","NOS2",0.2955,"Cardiometabolic_II","B13805","20212097_SS211647","PASS",-0.4366,0.3619,"Plate control","PASS","PFC",1.28511725684178,"24h","24h"
484,"PFC_08",85,"OID30087","P35228","NOS2",0.2955,"Cardiometabolic_II","B13805","20212097_SS211647","PASS",-0.4366,-0.3361,"Plate control","PASS","PFC",0.79217989929553,"Baseline","Baseline"
485,"PFC_16",86,"OID30087","P35228","NOS2",0.2955,"Cardiometabolic_II","B13805","20212097_SS211647","PASS",-0.4366,-0.2973,"Plate control","PASS","PFC",0.81377394793982,"1h","1h"
486,"PFC_24",87,"OID30087","P35228","NOS2",0.2955,"Cardiometabolic_II","B13805","20212097_SS211647","PASS",-0.4366,-0.5655,"Plate control","PASS","PFC",0.675721188809405,"24h","24h"
487,"PFC_01",1,"OID30088","E2RYF7","HCG22",0.9545,"Cardiometabolic_II","B13805","20212097_SS211647","WARN",0.679,0.1553,"Plate control","PASS","PFC",1.11365317181959,"Baseline","Baseline"
488,"PFC_09",2,"OID30088","E2RYF7","HCG22",0.9545,"Cardiometabolic_II","B13805","20212097_SS211647","PASS",0.679,-0.8124,"Plate control","PASS","PFC",0.569433786152785,"Baseline","Baseline"
489,"PFC_17",3,"OID30088","E2RYF7","HCG22",0.9545,"Cardiometabolic_II","B13805","20212097_SS211647","PASS",0.679,-0.276,"Plate control","PASS","PFC",0.82587766493572,"1h","1h"
490,"PFC_25",4,"OID30088","E2RYF7","HCG22",0.9545,"Cardiometabolic_II","B13805","20212097_SS211647","PASS",0.679,0.2254,"Plate control","PASS","PFC",1.16910134735062,"24h","24h"
491,"PFC_02",13,"OID30088","E2RYF7","HCG22",0.9545,"Cardiometabolic_II","B13805","20212097_SS211647","PASS",0.679,0.0458,"Plate control","PASS","PFC",1.03225542457635,"Baseline","Baseline"
492,"PFC_10",14,"OID30088","E2RYF7","HCG22",0.9545,"Cardiometabolic_II","B13805","20212097_SS211647","PASS",0.679,0.503,"Plate control","PASS","PFC",1.41715739650822,"1h","1h"
493,"PFC_18",15,"OID30088","E2RYF7","HCG22",0.9545,"Cardiometabolic_II","B13805","20212097_SS211647","PASS",0.679,0.4673,"Plate control","PASS","PFC",1.3825196635221,"1h","1h"
494,"PFC_26",16,"OID30088","E2RYF7","HCG22",0.9545,"Cardiometabolic_II","B13805","20212097_SS211647","PASS",0.679,-0.2554,"Plate control","PASS","PFC",0.837754827506151,"24h","24h"
495,"PFC_03",25,"OID30088","E2RYF7","HCG22",0.9545,"Cardiometabolic_II","B13805","20212097_SS211647","PASS",0.679,0.0124,"Plate control","PASS","PFC",1.0086320683199,"Baseline","Baseline"
496,"PFC_11",26,"OID30088","E2RYF7","HCG22",0.9545,"Cardiometabolic_II","B13805","20212097_SS211647","PASS",0.679,-0.8002,"Plate control","PASS","PFC",0.57426956131461,"1h","1h"
497,"PFC_19",27,"OID30088","E2RYF7","HCG22",0.9545,"Cardiometabolic_II","B13805","20212097_SS211647","PASS",0.679,0.0907,"Plate control","PASS","PFC",1.06488674339542,"24h","24h"
498,"PFC_27",28,"OID30088","E2RYF7","HCG22",0.9545,"Cardiometabolic_II","B13805","20212097_SS211647","PASS",0.679,-0.2053,"Plate control","PASS","PFC",0.867358306308781,"24h","24h"
499,"PFC_04",37,"OID30088","E2RYF7","HCG22",0.9545,"Cardiometabolic_II","B13805","20212097_SS211647","PASS",0.679,-0.0346,"Plate control","PASS","PFC",0.976302411759932,"Baseline","Baseline"
500,"PFC_12",38,"OID30088","E2RYF7","HCG22",0.9545,"Cardiometabolic_II","B13805","20212097_SS211647","PASS",0.679,-0.5429,"Plate control","PASS","PFC",0.686389790893435,"1h","1h"
501,"PFC_20",39,"OID30088","E2RYF7","HCG22",0.9545,"Cardiometabolic_II","B13805","20212097_SS211647","PASS",0.679,0.2544,"Plate control","PASS","PFC",1.19283955183549,"24h","24h"
502,"PFC_05",49,"OID30088","E2RYF7","HCG22",0.9545,"Cardiometabolic_II","B13805","20212097_SS211647","PASS",0.679,0.1222,"Plate control","PASS","PFC",1.08839331456432,"Baseline","Baseline"
503,"PFC_13",50,"OID30088","E2RYF7","HCG22",0.9545,"Cardiometabolic_II","B13805","20212097_SS211647","PASS",0.679,-0.1294,"Plate control","PASS","PFC",0.91421158108551,"1h","1h"
504,"PFC_21",51,"OID30088","E2RYF7","HCG22",0.9545,"Cardiometabolic_II","B13805","20212097_SS211647","PASS",0.679,-1.0188,"Plate control","PASS","PFC",0.493526685526933,"24h","24h"
505,"PFC_06",61,"OID30088","E2RYF7","HCG22",0.9545,"Cardiometabolic_II","B13805","20212097_SS211647","PASS",0.679,-0.6584,"Plate control","PASS","PFC",0.633580570827975,"Baseline","Baseline"
506,"PFC_14",62,"OID30088","E2RYF7","HCG22",0.9545,"Cardiometabolic_II","B13805","20212097_SS211647","PASS",0.679,-0.5317,"Plate control","PASS","PFC",0.691739142661778,"1h","1h"
507,"PFC_22",63,"OID30088","E2RYF7","HCG22",0.9545,"Cardiometabolic_II","B13805","20212097_SS211647","PASS",0.679,0.1601,"Plate control","PASS","PFC",1.11736458519926,"24h","24h"
508,"PFC_07",73,"OID30088","E2RYF7","HCG22",0.9545,"Cardiometabolic_II","B13805","20212097_SS211647","PASS",0.679,0.7539,"Plate control","PASS","PFC",1.68634532789813,"Baseline","Baseline"
509,"PFC_15",74,"OID30088","E2RYF7","HCG22",0.9545,"Cardiometabolic_II","B13805","20212097_SS211647","PASS",0.679,-0.0819,"Plate control","PASS","PFC",0.944812528141572,"1h","1h"
510,"PFC_23",75,"OID30088","E2RYF7","HCG22",0.9545,"Cardiometabolic_II","B13805","20212097_SS211647","PASS",0.679,0.1839,"Plate control","PASS","PFC",1.13595052396747,"24h","24h"
511,"PFC_08",85,"OID30088","E2RYF7","HCG22",0.9545,"Cardiometabolic_II","B13805","20212097_SS211647","PASS",0.679,0.0936,"Plate control","PASS","PFC",1.06702945364062,"Baseline","Baseline"
512,"PFC_16",86,"OID30088","E2RYF7","HCG22",0.9545,"Cardiometabolic_II","B13805","20212097_SS211647","PASS",0.679,0.4609,"Plate control","PASS","PFC",1.3764001934928,"1h","1h"
513,"PFC_24",87,"OID30088","E2RYF7","HCG22",0.9545,"Cardiometabolic_II","B13805","20212097_SS211647","PASS",0.679,-0.3099,"Plate control","PASS","PFC",0.806697673306051,"24h","24h"
514,"PFC_01",1,"OID30089","Q9NVZ3","NECAP2",0.0455,"Cardiometabolic_II","B13805","20212097_SS211647","WARN",-0.023,0.7846,"Plate control","PASS","PFC",1.72261464301388,"Baseline","Baseline"
515,"PFC_09",2,"OID30089","Q9NVZ3","NECAP2",0.0455,"Cardiometabolic_II","B13805","20212097_SS211647","PASS",-0.023,1.1355,"Plate control","PASS","PFC",2.19694689942271,"Baseline","Baseline"
516,"PFC_17",3,"OID30089","Q9NVZ3","NECAP2",0.0455,"Cardiometabolic_II","B13805","20212097_SS211647","PASS",-0.023,2.2798,"Plate control","PASS","PFC",4.8561062916385,"1h","1h"
517,"PFC_25",4,"OID30089","Q9NVZ3","NECAP2",0.0455,"Cardiometabolic_II","B13805","20212097_SS211647","PASS",-0.023,1.9378,"Plate control","PASS","PFC",3.83120971763198,"24h","24h"
518,"PFC_02",13,"OID30089","Q9NVZ3","NECAP2",0.0455,"Cardiometabolic_II","B13805","20212097_SS211647","PASS",-0.023,1.6037,"Plate control","PASS","PFC",3.03921765954387,"Baseline","Baseline"
519,"PFC_10",14,"OID30089","Q9NVZ3","NECAP2",0.0455,"Cardiometabolic_II","B13805","20212097_SS211647","PASS",-0.023,0.8626,"Plate control","PASS","PFC",1.8183122904909,"1h","1h"
520,"PFC_18",15,"OID30089","Q9NVZ3","NECAP2",0.0455,"Cardiometabolic_II","B13805","20212097_SS211647","PASS",-0.023,1.5861,"Plate control","PASS","PFC",3.00236629598974,"1h","1h"
521,"PFC_26",16,"OID30089","Q9NVZ3","NECAP2",0.0455,"Cardiometabolic_II","B13805","20212097_SS211647","PASS",-0.023,2.1806,"Plate control","PASS","PFC",4.5334205457821,"24h","24h"
522,"PFC_03",25,"OID30089","Q9NVZ3","NECAP2",0.0455,"Cardiometabolic_II","B13805","20212097_SS211647","PASS",-0.023,0.731,"Plate control","PASS","PFC",1.65978917117997,"Baseline","Baseline"
523,"PFC_11",26,"OID30089","Q9NVZ3","NECAP2",0.0455,"Cardiometabolic_II","B13805","20212097_SS211647","PASS",-0.023,2.1923,"Plate control","PASS","PFC",4.57033526308917,"1h","1h"
524,"PFC_19",27,"OID30089","Q9NVZ3","NECAP2",0.0455,"Cardiometabolic_II","B13805","20212097_SS211647","PASS",-0.023,1.7769,"Plate control","PASS","PFC",3.42689027690844,"24h","24h"
525,"PFC_27",28,"OID30089","Q9NVZ3","NECAP2",0.0455,"Cardiometabolic_II","B13805","20212097_SS211647","PASS",-0.023,2.9115,"Plate control","PASS","PFC",7.52400078822554,"24h","24h"
526,"PFC_04",37,"OID30089","Q9NVZ3","NECAP2",0.0455,"Cardiometabolic_II","B13805","20212097_SS211647","PASS",-0.023,1.9706,"Plate control","PASS","PFC",3.91931084700465,"Baseline","Baseline"
527,"PFC_12",38,"OID30089","Q9NVZ3","NECAP2",0.0455,"Cardiometabolic_II","B13805","20212097_SS211647","PASS",-0.023,0.4058,"Plate control","PASS","PFC",1.32482334480107,"1h","1h"
528,"PFC_20",39,"OID30089","Q9NVZ3","NECAP2",0.0455,"Cardiometabolic_II","B13805","20212097_SS211647","PASS",-0.023,2.7236,"Plate control","PASS","PFC",6.60518971813156,"24h","24h"
529,"PFC_05",49,"OID30089","Q9NVZ3","NECAP2",0.0455,"Cardiometabolic_II","B13805","20212097_SS211647","PASS",-0.023,0.7114,"Plate control","PASS","PFC",1.63739228178966,"Baseline","Baseline"
530,"PFC_13",50,"OID30089","Q9NVZ3","NECAP2",0.0455,"Cardiometabolic_II","B13805","20212097_SS211647","PASS",-0.023,1.6334,"Plate control","PASS","PFC",3.10243288319553,"1h","1h"
531,"PFC_21",51,"OID30089","Q9NVZ3","NECAP2",0.0455,"Cardiometabolic_II","B13805","20212097_SS211647","PASS",-0.023,0.6886,"Plate control","PASS","PFC",1.61171873771224,"24h","24h"
532,"PFC_06",61,"OID30089","Q9NVZ3","NECAP2",0.0455,"Cardiometabolic_II","B13805","20212097_SS211647","PASS",-0.023,1.6603,"Plate control","PASS","PFC",3.16082245367165,"Baseline","Baseline"
533,"PFC_14",62,"OID30089","Q9NVZ3","NECAP2",0.0455,"Cardiometabolic_II","B13805","20212097_SS211647","PASS",-0.023,1.7606,"Plate control","PASS","PFC",3.38839014830428,"1h","1h"
534,"PFC_22",63,"OID30089","Q9NVZ3","NECAP2",0.0455,"Cardiometabolic_II","B13805","20212097_SS211647","PASS",-0.023,1.1633,"Plate control","PASS","PFC",2.23969145963501,"24h","24h"
535,"PFC_07",73,"OID30089","Q9NVZ3","NECAP2",0.0455,"Cardiometabolic_II","B13805","20212097_SS211647","PASS",-0.023,2.0796,"Plate control","PASS","PFC",4.22690005422232,"Baseline","Baseline"
536,"PFC_15",74,"OID30089","Q9NVZ3","NECAP2",0.0455,"Cardiometabolic_II","B13805","20212097_SS211647","PASS",-0.023,0.4709,"Plate control","PASS","PFC",1.38597381393672,"1h","1h"
537,"PFC_23",75,"OID30089","Q9NVZ3","NECAP2",0.0455,"Cardiometabolic_II","B13805","20212097_SS211647","PASS",-0.023,2.1658,"Plate control","PASS","PFC",4.48715182767501,"24h","24h"
538,"PFC_08",85,"OID30089","Q9NVZ3","NECAP2",0.0455,"Cardiometabolic_II","B13805","20212097_SS211647","PASS",-0.023,1.7623,"Plate control","PASS","PFC",3.39238521186649,"Baseline","Baseline"
539,"PFC_16",86,"OID30089","Q9NVZ3","NECAP2",0.0455,"Cardiometabolic_II","B13805","20212097_SS211647","PASS",-0.023,1.3079,"Plate control","PASS","PFC",2.47580896572678,"1h","1h"
540,"PFC_24",87,"OID30089","Q9NVZ3","NECAP2",0.0455,"Cardiometabolic_II","B13805","20212097_SS211647","PASS",-0.023,2.2448,"Plate control","PASS","PFC",4.73971397434443,"24h","24h"
541,"PFC_01",1,"OID30090","Q9Y4C8","RBM19",0.7045,"Cardiometabolic_II","B13805","20212097_SS211647","WARN",-0.5065,-0.7456,"Plate control","PASS","PFC",0.596419775917743,"Baseline","Baseline"
542,"PFC_09",2,"OID30090","Q9Y4C8","RBM19",0.7045,"Cardiometabolic_II","B13805","20212097_SS211647","PASS",-0.5065,-1.7132,"Plate control","PASS","PFC",0.304982845037491,"Baseline","Baseline"
543,"PFC_17",3,"OID30090","Q9Y4C8","RBM19",0.7045,"Cardiometabolic_II","B13805","20212097_SS211647","PASS",-0.5065,-0.6367,"Plate control","PASS","PFC",0.643182473845912,"1h","1h"
544,"PFC_25",4,"OID30090","Q9Y4C8","RBM19",0.7045,"Cardiometabolic_II","B13805","20212097_SS211647","PASS",-0.5065,-1.3517,"Plate control","PASS","PFC",0.39183006377493,"24h","24h"
545,"PFC_02",13,"OID30090","Q9Y4C8","RBM19",0.7045,"Cardiometabolic_II","B13805","20212097_SS211647","PASS",-0.5065,-1.1511,"Plate control","PASS","PFC",0.450281777687777,"Baseline","Baseline"
546,"PFC_10",14,"OID30090","Q9Y4C8","RBM19",0.7045,"Cardiometabolic_II","B13805","20212097_SS211647","PASS",-0.5065,-0.4669,"Plate control","PASS","PFC",0.723517591615093,"1h","1h"
547,"PFC_18",15,"OID30090","Q9Y4C8","RBM19",0.7045,"Cardiometabolic_II","B13805","20212097_SS211647","PASS",-0.5065,-2.1146,"Plate control","PASS","PFC",0.230909590540531,"1h","1h"
548,"PFC_26",16,"OID30090","Q9Y4C8","RBM19",0.7045,"Cardiometabolic_II","B13805","20212097_SS211647","PASS",-0.5065,-1.7502,"Plate control","PASS","PFC",0.297260566829396,"24h","24h"
549,"PFC_03",25,"OID30090","Q9Y4C8","RBM19",0.7045,"Cardiometabolic_II","B13805","20212097_SS211647","PASS",-0.5065,2.5067,"Plate control","PASS","PFC",5.6831862644835,"Baseline","Baseline"
550,"PFC_11",26,"OID30090","Q9Y4C8","RBM19",0.7045,"Cardiometabolic_II","B13805","20212097_SS211647","PASS",-0.5065,-0.7154,"Plate control","PASS","PFC",0.609036247723181,"1h","1h"
551,"PFC_19",27,"OID30090","Q9Y4C8","RBM19",0.7045,"Cardiometabolic_II","B13805","20212097_SS211647","PASS",-0.5065,-0.9088,"Plate control","PASS","PFC",0.532627934489107,"24h","24h"
552,"PFC_27",28,"OID30090","Q9Y4C8","RBM19",0.7045,"Cardiometabolic_II","B13805","20212097_SS211647","PASS",-0.5065,-1.5523,"Plate control","PASS","PFC",0.340966049450757,"24h","24h"
553,"PFC_04",37,"OID30090","Q9Y4C8","RBM19",0.7045,"Cardiometabolic_II","B13805","20212097_SS211647","PASS",-0.5065,-0.5309,"Plate control","PASS","PFC",0.692122830661983,"Baseline","Baseline"
554,"PFC_12",38,"OID30090","Q9Y4C8","RBM19",0.7045,"Cardiometabolic_II","B13805","20212097_SS211647","PASS",-0.5065,2.3147,"Plate control","PASS","PFC",4.97501198096638,"1h","1h"
555,"PFC_20",39,"OID30090","Q9Y4C8","RBM19",0.7045,"Cardiometabolic_II","B13805","20212097_SS211647","PASS",-0.5065,-0.8362,"Plate control","PASS","PFC",0.560116953017288,"24h","24h"
556,"PFC_05",49,"OID30090","Q9Y4C8","RBM19",0.7045,"Cardiometabolic_II","B13805","20212097_SS211647","PASS",-0.5065,-1.2558,"Plate control","PASS","PFC",0.418761292372324,"Baseline","Baseline"
557,"PFC_13",50,"OID30090","Q9Y4C8","RBM19",0.7045,"Cardiometabolic_II","B13805","20212097_SS211647","PASS",-0.5065,-1.2179,"Plate control","PASS","PFC",0.429908041043429,"1h","1h"
558,"PFC_21",51,"OID30090","Q9Y4C8","RBM19",0.7045,"Cardiometabolic_II","B13805","20212097_SS211647","PASS",-0.5065,2.1982,"Plate control","PASS","PFC",4.5890642321969,"24h","24h"
559,"PFC_06",61,"OID30090","Q9Y4C8","RBM19",0.7045,"Cardiometabolic_II","B13805","20212097_SS211647","PASS",-0.5065,-0.2298,"Plate control","PASS","PFC",0.852753100255611,"Baseline","Baseline"
560,"PFC_14",62,"OID30090","Q9Y4C8","RBM19",0.7045,"Cardiometabolic_II","B13805","20212097_SS211647","PASS",-0.5065,-1.4521,"Plate control","PASS","PFC",0.365489027453096,"1h","1h"

Supplemental table 1_Olink proximity extension assay (PEA) data

,"SampleID","Index","OlinkID","UniProt","Assay","MissingFreq","Panel","Panel_Lot_Nr","PlateID","QC_Warning","LOD","NPX","Normalization","Assay_Warning","condition","Value","group","alt_group"
561,"PFC_22",63,"OID30090","Q9Y4C8","RBM19",0.7045,"Cardiometabolic_II","B13805","20212097_SS211647","PASS",-0.5065,-1.6351,"Plate control","PASS","PFC",0.321948090862887,"24h","24h"
562,"PFC_07",73,"OID30090","Q9Y4C8","RBM19",0.7045,"Cardiometabolic_II","B13805","20212097_SS211647","PASS",-0.5065,-2.6566,"Plate control","PASS","PFC",0.158592890106436,"Baseline","Baseline"
563,"PFC_15",74,"OID30090","Q9Y4C8","RBM19",0.7045,"Cardiometabolic_II","B13805","20212097_SS211647","PASS",-0.5065,-1.3722,"Plate control","PASS","PFC",0.386301718322445,"1h","1h"
564,"PFC_23",75,"OID30090","Q9Y4C8","RBM19",0.7045,"Cardiometabolic_II","B13805","20212097_SS211647","PASS",-0.5065,-1.0983,"Plate control","PASS","PFC",0.467066539586217,"24h","24h"
565,"PFC_08",85,"OID30090","Q9Y4C8","RBM19",0.7045,"Cardiometabolic_II","B13805","20212097_SS211647","PASS",-0.5065,-1.006,"Plate control","PASS","PFC",0.497924876547229,"Baseline","Baseline"
566,"PFC_16",86,"OID30090","Q9Y4C8","RBM19",0.7045,"Cardiometabolic_II","B13805","20212097_SS211647","PASS",-0.5065,-1.7953,"Plate control","PASS","PFC",0.288111669332814,"1h","1h"
567,"PFC_24",87,"OID30090","Q9Y4C8","RBM19",0.7045,"Cardiometabolic_II","B13805","20212097_SS211647","PASS",-0.5065,-0.6608,"Plate control","PASS","PFC",0.632527452023254,"24h","24h"
568,"PFC_01",1,"OID30091","Q07973","CYP24A1",0.75,"Cardiometabolic_II","B13805","20212097_SS211647","WARN",-0.716,-0.691,"Plate control","PASS","PFC",0.619424348891954,"Baseline","Baseline"
569,"PFC_09",2,"OID30091","Q07973","CYP24A1",0.75,"Cardiometabolic_II","B13805","20212097_SS211647","PASS",-0.716,-1.2137,"Plate control","PASS","PFC",0.431161420684502,"Baseline","Baseline"
570,"PFC_17",3,"OID30091","Q07973","CYP24A1",0.75,"Cardiometabolic_II","B13805","20212097_SS211647","PASS",-0.716,-1.3166,"Plate control","PASS","PFC",0.40147999281805,"1h","1h"
571,"PFC_25",4,"OID30091","Q07973","CYP24A1",0.75,"Cardiometabolic_II","B13805","20212097_SS211647","PASS",-0.716,-0.9186,"Plate control","PASS","PFC",0.529022137558711,"24h","24h"
572,"PFC_02",13,"OID30091","Q07973","CYP24A1",0.75,"Cardiometabolic_II","B13805","20212097_SS211647","PASS",-0.716,-1.0917,"Plate control","PASS","PFC",0.469208157199955,"Baseline","Baseline"
573,"PFC_10",14,"OID30091","Q07973","CYP24A1",0.75,"Cardiometabolic_II","B13805","20212097_SS211647","PASS",-0.716,-0.571,"Plate control","PASS","PFC",0.673150034638391,"1h","1h"
574,"PFC_18",15,"OID30091","Q07973","CYP24A1",0.75,"Cardiometabolic_II","B13805","20212097_SS211647","PASS",-0.716,-0.6104,"Plate control","PASS","PFC",0.655015068011491,"1h","1h"
575,"PFC_26",16,"OID30091","Q07973","CYP24A1",0.75,"Cardiometabolic_II","B13805","20212097_SS211647","PASS",-0.716,-1.4565,"Plate control","PASS","PFC",0.364376039706128,"24h","24h"
576,"PFC_03",25,"OID30091","Q07973","CYP24A1",0.75,"Cardiometabolic_II","B13805","20212097_SS211647","PASS",-0.716,-0.8383,"Plate control","PASS","PFC",0.559302234794549,"Baseline","Baseline"
577,"PFC_11",26,"OID30091","Q07973","CYP24A1",0.75,"Cardiometabolic_II","B13805","20212097_SS211647","PASS",-0.716,-0.6615,"Plate control","PASS","PFC",0.632220622232648,"1h","1h"
578,"PFC_19",27,"OID30091","Q07973","CYP24A1",0.75,"Cardiometabolic_II","B13805","20212097_SS211647","PASS",-0.716,-0.2668,"Plate control","PASS","PFC",0.831161076998763,"24h","24h"
579,"PFC_27",28,"OID30091","Q07973","CYP24A1",0.75,"Cardiometabolic_II","B13805","20212097_SS211647","PASS",-0.716,-1.6038,"Plate control","PASS","PFC",0.329009238461132,"24h","24h"
580,"PFC_04",37,"OID30091","Q07973","CYP24A1",0.75,"Cardiometabolic_II","B13805","20212097_SS211647","PASS",-0.716,-1.5465,"Plate control","PASS","PFC",0.342339578572415,"Baseline","Baseline"
581,"PFC_12",38,"OID30091","Q07973","CYP24A1",0.75,"Cardiometabolic_II","B13805","20212097_SS211647","PASS",-0.716,-1.1065,"Plate control","PASS","PFC",0.464419353747951,"1h","1h"
582,"PFC_20",39,"OID30091","Q07973","CYP24A1",0.75,"Cardiometabolic_II","B13805","20212097_SS211647","PASS",-0.716,-0.7773,"Plate control","PASS","PFC",0.583457711673432,"24h","24h"
583,"PFC_05",49,"OID30091","Q07973","CYP24A1",0.75,"Cardiometabolic_II","B13805","20212097_SS211647","PASS",-0.716,-1.4626,"Plate control","PASS","PFC",0.362838638245319,"Baseline","Baseline"
584,"PFC_13",50,"OID30091","Q07973","CYP24A1",0.75,"Cardiometabolic_II","B13805","20212097_SS211647","PASS",-0.716,-1.6044,"Plate control","PASS","PFC",0.328872435814804,"1h","1h"
585,"PFC_21",51,"OID30091","Q07973","CYP24A1",0.75,"Cardiometabolic_II","B13805","20212097_SS211647","PASS",-0.716,-1.1837,"Plate control","PASS","PFC",0.440221038656017,"24h","24h"
586,"PFC_06",61,"OID30091","Q07973","CYP24A1",0.75,"Cardiometabolic_II","B13805","20212097_SS211647","PASS",-0.716,-0.5969,"Plate control","PASS","PFC",0.661173130012104,"Baseline","Baseline"
587,"PFC_14",62,"OID30091","Q07973","CYP24A1",0.75,"Cardiometabolic_II","B13805","20212097_SS211647","PASS",-0.716,-1.1874,"Plate control","PASS","PFC",0.439093474678928,"1h","1h"
588,"PFC_22",63,"OID30091","Q07973","CYP24A1",0.75,"Cardiometabolic_II","B13805","20212097_SS211647","PASS",-0.716,-0.9952,"Plate control","PASS","PFC",0.501666323714414,"24h","24h"
589,"PFC_07",73,"OID30091","Q07973","CYP24A1",0.75,"Cardiometabolic_II","B13805","20212097_SS211647","PASS",-0.716,-0.8594,"Plate control","PASS","PFC",0.551181740319433,"Baseline","Baseline"
590,"PFC_15",74,"OID30091","Q07973","CYP24A1",0.75,"Cardiometabolic_II","B13805","20212097_SS211647","PASS",-0.716,-1.1604,"Plate control","PASS","PFC",0.447388475842207,"1h","1h"
591,"PFC_23",75,"OID30091","Q07973","CYP24A1",0.75,"Cardiometabolic_II","B13805","20212097_SS211647","PASS",-0.716,-1.1841,"Plate control","PASS","PFC",0.440099000386188,"24h","24h"
592,"PFC_08",85,"OID30091","Q07973","CYP24A1",0.75,"Cardiometabolic_II","B13805","20212097_SS211647","PASS",-0.716,-1.246,"Plate control","PASS","PFC",0.421615555125056,"Baseline","Baseline"
593,"PFC_16",86,"OID30091","Q07973","CYP24A1",0.75,"Cardiometabolic_II","B13805","20212097_SS211647","PASS",-0.716,-0.7445,"Plate control","PASS","PFC",0.596874696680297,"1h","1h"
594,"PFC_24",87,"OID30091","Q07973","CYP24A1",0.75,"Cardiometabolic_II","B13805","20212097_SS211647","PASS",-0.716,-0.6274,"Plate control","PASS","PFC",0.647341993232533,"24h","24h"
595,"PFC_01",1,"OID30092","Q04695","KRT17",0.9091,"Cardiometabolic_II","B13805","20212097_SS211647","WARN",0.6681,0.282,"Plate control","PASS","PFC",1.2158792831831,"Baseline","Baseline"
596,"PFC_09",2,"OID30092","Q04695","KRT17",0.9091,"Cardiometabolic_II","B13805","20212097_SS211647","PASS",0.6681,0.3003,"Plate control","PASS","PFC",1.23140044924827,"Baseline","Baseline"
597,"PFC_17",3,"OID30092","Q04695","KRT17",0.9091,"Cardiometabolic_II","B13805","20212097_SS211647","PASS",0.6681,0.1552,"Plate control","PASS","PFC",1.11357598193921,"1h","1h"
598,"PFC_25",4,"OID30092","Q04695","KRT17",0.9091,"Cardiometabolic_II","B13805","20212097_SS211647","PASS",0.6681,0.2074,"Plate control","PASS","PFC",1.15460549776042,"24h","24h"
599,"PFC_02",13,"OID30092","Q04695","KRT17",0.9091,"Cardiometabolic_II","B13805","20212097_SS211647","PASS",0.6681,0.2235,"Plate control","PASS","PFC",1.16756267809592,"Baseline","Baseline"
600,"PFC_10",14,"OID30092","Q04695","KRT17",0.9091,"Cardiometabolic_II","B13805","20212097_SS211647","PASS",0.6681,-0.1238,"Plate control","PASS","PFC",0.917767103028868,"1h","1h"
601,"PFC_18",15,"OID30092","Q04695","KRT17",0.9091,"Cardiometabolic_II","B13805","20212097_SS211647","PASS",0.6681,1.0842,"Plate control","PASS","PFC",2.12019946847914,"1h","1h"
602,"PFC_26",16,"OID30092","Q04695","KRT17",0.9091,"Cardiometabolic_II","B13805","20212097_SS211647","PASS",0.6681,-0.6132,"Plate control","PASS","PFC",0.653745039680002,"24h","24h"
603,"PFC_03",25,"OID30092","Q04695","KRT17",0.9091,"Cardiometabolic_II","B13805","20212097_SS211647","PASS",0.6681,0.3398,"Plate control","PASS","PFC",1.26558113500957,"Baseline","Baseline"
604,"PFC_11",26,"OID30092","Q04695","KRT17",0.9091,"Cardiometabolic_II","B13805","20212097_SS211647","PASS",0.6681,-0.2279,"Plate control","PASS","PFC",0.853876898576533,"1h","1h"
605,"PFC_19",27,"OID30092","Q04695","KRT17",0.9091,"Cardiometabolic_II","B13805","20212097_SS211647","PASS",0.6681,0.4228,"Plate control","PASS","PFC",1.34052674223217,"24h","24h"
606,"PFC_27",28,"OID30092","Q04695","KRT17",0.9091,"Cardiometabolic_II","B13805","20212097_SS211647","PASS",0.6681,0.2646,"Plate control","PASS","PFC",1.20130293145304,"24h","24h"
607,"PFC_04",37,"OID30092","Q04695","KRT17",0.9091,"Cardiometabolic_II","B13805","20212097_SS211647","PASS",0.6681,0.2093,"Plate control","PASS","PFC",1.15612709143076,"Baseline","Baseline"
608,"PFC_12",38,"OID30092","Q04695","KRT17",0.9091,"Cardiometabolic_II","B13805","20212097_SS211647","PASS",0.6681,0.3,"Plate control","PASS","PFC",1.23114441334492,"1h","1h"
609,"PFC_20",39,"OID30092","Q04695","KRT17",0.9091,"Cardiometabolic_II","B13805","20212097_SS211647","PASS",0.6681,-0.5856,"Plate control","PASS","PFC",0.66637214452481,"24h","24h"
610,"PFC_05",49,"OID30092","Q04695","KRT17",0.9091,"Cardiometabolic_II","B13805","20212097_SS211647","PASS",0.6681,-0.2575,"Plate control","PASS","PFC",0.83653627105918,"Baseline","Baseline"
611,"PFC_13",50,"OID30092","Q04695","KRT17",0.9091,"Cardiometabolic_II","B13805","20212097_SS211647","PASS",0.6681,-0.8971,"Plate control","PASS","PFC",0.536965014941483,"1h","1h"
612,"PFC_21",51,"OID30092","Q04695","KRT17",0.9091,"Cardiometabolic_II","B13805","20212097_SS211647","PASS",0.6681,-1.4336,"Plate control","PASS","PFC",0.370205953157065,"24h","24h"
613,"PFC_06",61,"OID30092","Q04695","KRT17",0.9091,"Cardiometabolic_II","B13805","20212097_SS211647","PASS",0.6681,1.6044,"Plate control","PASS","PFC",3.04069265495733,"Baseline","Baseline"
614,"PFC_14",62,"OID30092","Q04695","KRT17",0.9091,"Cardiometabolic_II","B13805","20212097_SS211647","PASS",0.6681,0.3678,"Plate control","PASS","PFC",1.29038359290198,"1h","1h"
615,"PFC_22",63,"OID30092","Q04695","KRT17",0.9091,"Cardiometabolic_II","B13805","20212097_SS211647","PASS",0.6681,-0.1521,"Plate control","PASS","PFC",0.899939548575492,"24h","24h"
616,"PFC_07",73,"OID30092","Q04695","KRT17",0.9091,"Cardiometabolic_II","B13805","20212097_SS211647","PASS",0.6681,0.1753,"Plate control","PASS","PFC",1.12919919076471,"Baseline","Baseline"
617,"PFC_15",74,"OID30092","Q04695","KRT17",0.9091,"Cardiometabolic_II","B13805","20212097_SS211647","PASS",0.6681,-0.2217,"Plate control","PASS","PFC",0.857554341506999,"1h","1h"
618,"PFC_23",75,"OID30092","Q04695","KRT17",0.9091,"Cardiometabolic_II","B13805","20212097_SS211647","PASS",0.6681,0.1672,"Plate control","PASS","PFC",1.12287707510006,"24h","24h"
619,"PFC_08",85,"OID30092","Q04695","KRT17",0.9091,"Cardiometabolic_II","B13805","20212097_SS211647","PASS",0.6681,0.311,"Plate control","PASS","PFC",1.24056729771539,"Baseline","Baseline"
620,"PFC_16",86,"OID30092","Q04695","KRT17",0.9091,"Cardiometabolic_II","B13805","20212097_SS211647","PASS",0.6681,0.011,"Plate control","PASS","PFC",1.0076537604105,"1h","1h"
621,"PFC_24",87,"OID30092","Q04695","KRT17",0.9091,"Cardiometabolic_II","B13805","20212097_SS211647","PASS",0.6681,-0.1214,"Plate control","PASS","PFC",0.91929512808369,"24h","24h"
622,"PFC_01",1,"OID30093","Q15059","BRD3",0.4318,"Cardiometabolic_II","B13805","20212097_SS211647","WARN",0.9497,0.447,"Plate control","PASS","PFC",1.36320260728251,"Baseline","Baseline"
623,"PFC_09",2,"OID30093","Q15059","BRD3",0.4318,"Cardiometabolic_II","B13805","20212097_SS211647","PASS",0.9497,0.8009,"Plate control","PASS","PFC",1.74218762085548,"Baseline","Baseline"
624,"PFC_17",3,"OID30093","Q15059","BRD3",0.4318,"Cardiometabolic_II","B13805","20212097_SS211647","PASS",0.9497,1.4785,"Plate control","PASS","PFC",2.78658855200339,"1h","1h"
625,"PFC_25",4,"OID30093","Q15059","BRD3",0.4318,"Cardiometabolic_II","B13805","20212097_SS211647","PASS",0.9497,1.3176,"Plate control","PASS","PFC",2.49251122188819,"24h","24h"
626,"PFC_02",13,"OID30093","Q15059","BRD3",0.4318,"Cardiometabolic_II","B13805","20212097_SS211647","PASS",0.9497,1.0596,"Plate control","PASS","PFC",2.0843535360494,"Baseline","Baseline"
627,"PFC_10",14,"OID30093","Q15059","BRD3",0.4318,"Cardiometabolic_II","B13805","20212097_SS211647","PASS",0.9497,0.2506,"Plate control","PASS","PFC",1.18970179519676,"1h","1h"
628,"PFC_18",15,"OID30093","Q15059","BRD3",0.4318,"Cardiometabolic_II","B13805","20212097_SS211647","PASS",0.9497,1.4157,"Plate control","PASS","PFC",2.6678915092274,"1h","1h"
629,"PFC_26",16,"OID30093","Q15059","BRD3",0.4318,"Cardiometabolic_II","B13805","20212097_SS211647","PASS",0.9497,0.0097,"Plate control","PASS","PFC",1.00674618130584,"24h","24h"
630,"PFC_03",25,"OID30093","Q15059","BRD3",0.4318,"Cardiometabolic_II","B13805","20212097_SS211647","PASS",0.9497,-0.0999,"Plate control","PASS","PFC",0.933097666696997,"Baseline","Baseline"
631,"PFC_11",26,"OID30093","Q15059","BRD3",0.4318,"Cardiometabolic_II","B13805","20212097_SS211647","PASS",0.9497,1.8773,"Plate control","PASS","PFC",3.67386852926259,"1h","1h"
632,"PFC_19",27,"OID30093","Q15059","BRD3",0.4318,"Cardiometabolic_II","B13805","20212097_SS211647","PASS",0.9497,0.7597,"Plate control","PASS","PFC",1.69313850986089,"24h","24h"
633,"PFC_27",28,"OID30093","Q15059","BRD3",0.4318,"Cardiometabolic_II","B13805","20212097_SS211647","PASS",0.9497,1.7332,"Plate control","PASS","PFC",3.32464430799505,"24h","24h"
634,"PFC_04",37,"OID30093","Q15059","BRD3",0.4318,"Cardiometabolic_II","B13805","20212097_SS211647","PASS",0.9497,1.3158,"Plate control","PASS","PFC",2.48940334226082,"Baseline","Baseline"
635,"PFC_12",38,"OID30093","Q15059","BRD3",0.4318,"Cardiometabolic_II","B13805","20212097_SS211647","PASS",0.9497,0.3475,"Plate control","PASS","PFC",1.27235389459647,"1h","1h"
636,"PFC_20",39,"OID30093","Q15059","BRD3",0.4318,"Cardiometabolic_II","B13805","20212097_SS211647","PASS",0.9497,2.4011,"Plate control","PASS","PFC",5.28205747569608,"24h","24h"
637,"PFC_05",49,"OID30093","Q15059","BRD3",0.4318,"Cardiometabolic_II","B13805","20212097_SS211647","PASS",0.9497,0.6326,"Plate control","PASS","PFC",1.55035650300306,"Baseline","Baseline"
638,"PFC_13",50,"OID30093","Q15059","BRD3",0.4318,"Cardiometabolic_II","B13805","20212097_SS211647","PASS",0.9497,1.5482,"Plate control","PASS","PFC",2.92452029329863,"1h","1h"
639,"PFC_21",51,"OID30093","Q15059","BRD3",0.4318,"Cardiometabolic_II","B13805","20212097_SS211647","PASS",0.9497,-0.1412,"Plate control","PASS","PFC",0.906764615952189,"24h","24h"
640,"PFC_06",61,"OID30093","Q15059","BRD3",0.4318,"Cardiometabolic_II","B13805","20212097_SS211647","PASS",0.9497,1.0025,"Plate control","PASS","PFC",2.00346874046939,"Baseline","Baseline"
641,"PFC_14",62,"OID30093","Q15059","BRD3",0.4318,"Cardiometabolic_II","B13805","20212097_SS211647","PASS",0.9497,0.7736,"Plate control","PASS","PFC",1.70953030762483,"1h","1h"
642,"PFC_22",63,"OID30093","Q15059","BRD3",0.4318,"Cardiometabolic_II","B13805","20212097_SS211647","PASS",0.9497,1.557,"Plate control","PASS","PFC",2.94241349171148,"24h","24h"
643,"PFC_07",73,"OID30093","Q15059","BRD3",0.4318,"Cardiometabolic_II","B13805","20212097_SS211647","PASS",0.9497,1.4481,"Plate control","PASS","PFC",2.72848478735807,"Baseline","Baseline"
644,"PFC_15",74,"OID30093","Q15059","BRD3",0.4318,"Cardiometabolic_II","B13805","20212097_SS211647","PASS",0.9497,0.0886,"Plate control","PASS","PFC",1.0633378121761,"1h","1h"
645,"PFC_23",75,"OID30093","Q15059","BRD3",0.4318,"Cardiometabolic_II","B13805","20212097_SS211647","PASS",0.9497,2.3199,"Plate control","PASS","PFC",4.9929760968813,"24h","24h"
646,"PFC_08",85,"OID30093","Q15059","BRD3",0.4318,"Cardiometabolic_II","B13805","20212097_SS211647","PASS",0.9497,0.1918,"Plate control","PASS","PFC",1.14218789500887,"Baseline","Baseline"
647,"PFC_16",86,"OID30093","Q15059","BRD3",0.4318,"Cardiometabolic_II","B13805","20212097_SS211647","PASS",0.9497,0.437,"Plate control","PASS","PFC",1.35378627905246,"1h","1h"
648,"PFC_24",87,"OID30093","Q15059","BRD3",0.4318,"Cardiometabolic_II","B13805","20212097_SS211647","PASS",0.9497,1.6893,"Plate control","PASS","PFC",3.22500187653833,"24h","24h"
649,"PFC_01",1,"OID30094","B6SEH8","ERVV-1",0.9545,"Cardiometabolic_II","B13805","20212097_SS211647","WARN",1.0414,0.186,"Plate control","PASS","PFC",1.1376052278725,"Baseline","Baseline"
650,"PFC_09",2,"OID30094","B6SEH8","ERVV-1",0.9545,"Cardiometabolic_II","B13805","20212097_SS211647","PASS",1.0414,0.5755,"Plate control","PASS","PFC",1.4901938354648,"Baseline","Baseline"
651,"PFC_17",3,"OID30094","B6SEH8","ERVV-1",0.9545,"Cardiometabolic_II","B13805","20212097_SS211647","PASS",1.0414,0.6378,"Plate control","PASS","PFC",1.55595463704494,"1h","1h"
652,"PFC_25",4,"OID30094","B6SEH8","ERVV-1",0.9545,"Cardiometabolic_II","B13805","20212097_SS211647","PASS",1.0414,0.514,"Plate control","PASS","PFC",1.42800397968506,"24h","24h"
653,"PFC_02",13,"OID30094","B6SEH8","ERVV-1",0.9545,"Cardiometabolic_II","B13805","20212097_SS211647","PASS",1.0414,-0.2622,"Plate control","PASS","PFC",0.833815444447526,"Baseline","Baseline"
654,"PFC_10",14,"OID30094","B6SEH8","ERVV-1",0.9545,"Cardiometabolic_II","B13805","20212097_SS211647","PASS",1.0414,-0.183,"Plate control","PASS","PFC",0.880869374126979,"1h","1h"
655,"PFC_18",15,"OID30094","B6SEH8","ERVV-1",0.9545,"Cardiometabolic_II","B13805","20212097_SS211647","PASS",1.0414,1.059,"Plate control","PASS","PFC",2.08348685801644,"1h","1h"
656,"PFC_26",16,"OID30094","B6SEH8","ERVV-1",0.9545,"Cardiometabolic_II","B13805","20212097_SS211647","PASS",1.0414,0.7599,"Plate control","PASS","PFC",1.69337324496799,"24h","24h"
657,"PFC_03",25,"OID30094","B6SEH8","ERVV-1",0.9545,"Cardiometabolic_II","B13805","20212097_SS211647","PASS",1.0414,0.6652,"Plate control","PASS","PFC",1.58578809489273,"Baseline","Baseline"
658,"PFC_11",26,"OID30094","B6SEH8","ERVV-1",0.9545,"Cardiometabolic_II","B13805","20212097_SS211647","PASS",1.0414,-0.3649,"Plate control","PASS","PFC",0.776522697587517,"1h","1h"
659,"PFC_19",27,"OID30094","B6SEH8","ERVV-1",0.9545,"Cardiometabolic_II","B13805","20212097_SS211647","PASS",1.0414,0.7283,"Plate control","PASS","PFC",1.65668578497767,"24h","24h"
660,"PFC_27",28,"OID30094","B6SEH8","ERVV-1",0.9545,"Cardiometabolic_II","B13805","20212097_SS211647","PASS",1.0414,0.961,"Plate control","PASS","PFC",1.94665874831648,"24h","24h"
661,"PFC_04",37,"OID30094","B6SEH8","ERVV-1",0.9545,"Cardiometabolic_II","B13805","20212097_SS211647","PASS",1.0414,-1.1165,"Plate control","PASS","PFC",0.461211374942808,"Baseline","Baseline"
662,"PFC_12",38,"OID30094","B6SEH8","ERVV-1",0.9545,"Cardiometabolic_II","B13805","20212097_SS211647","PASS",1.0414,-0.1138,"Plate control","PASS","PFC",0.924150678054395,"1h","1h"
663,"PFC_20",39,"OID30094","B6SEH8","ERVV-1",0.9545,"Cardiometabolic_II","B13805","20212097_SS211647","PASS",1.0414,-0.1769,"Plate control","PASS","PFC",0.884601749127374,"24h","24h"
664,"PFC_05",49,"OID30094","B6SEH8","ERVV-1",0.9545,"Cardiometabolic_II","B13805","20212097_SS211647","PASS",1.0414,0.5148,"Plate control","PASS","PFC",1.42879605281995,"Baseline","Baseline"
665,"PFC_13",50,"OID30094","B6SEH8","ERVV-1",0.9545,"Cardiometabolic_II","B13805","20212097_SS211647","PASS",1.0414,-0.0321,"Plate control","PASS","PFC",0.97799568160295,"1h","1h"
666,"PFC_21",51,"OID30094","B6SEH8","ERVV-1",0.9545,"Cardiometabolic_II","B13805","20212097_SS211647","PASS",1.0414,0.1449,"Plate control","PASS","PFC",1.10565401256798,"24h","24h"
667,"PFC_06",61,"OID30094","B6SEH8","ERVV-1",0.9545,"Cardiometabolic_II","B13805","20212097_SS211647","PASS",1.0414,0.7678,"Plate control","PASS","PFC",1.70267135876571,"Baseline","Baseline"
668,"PFC_14",62,"OID30094","B6SEH8","ERVV-1",0.9545,"Cardiometabolic_II","B13805","20212097_SS211647","PASS",1.0414,0.6452,"Plate control","PASS","PFC",1.56395608157943,"1h","1h"
669,"PFC_22",63,"OID30094","B6SEH8","ERVV-1",0.9545,"Cardiometabolic_II","B13805","20212097_SS211647","PASS",1.0414,0.2655,"Plate control","PASS","PFC",1.20205257702129,"24h","24h"
670,"PFC_07",73,"OID30094","B6SEH8","ERVV-1",0.9545,"Cardiometabolic_II","B13805","20212097_SS211647","PASS",1.0414,0.7391,"Plate control","PASS","PFC",1.66913425387141,"Baseline","Baseline"
671,"PFC_15",74,"OID30094","B6SEH8","ERVV-1",0.9545,"Cardiometabolic_II","B13805","20212097_SS211647","PASS",1.0414,-0.2981,"Plate control","PASS","PFC",0.813322820936242,"1h","1h"
672,"PFC_23",75,"OID30094","B6SEH8","ERVV-1",0.9545,"Cardiometabolic_II","B13805","20212097_SS211647","PASS",1.0414,0.9831,"Plate control","PASS","PFC",1.97670831323241,"24h","24h"

Supplemental table 1_Olink proximity extension assay (PEA) data

,"SampleID","Index","OlinkID","UniProt","Assay","MissingFreq","Panel","Panel_Lot_Nr","PlateID","QC_Warning","LOD","NPX","Normalization","Assay_Warning","condition","Value","group","alt_group"
673,"PFC_08",85,"OID30094","B6SEH8","ERVV-1",0.9545,"Cardiometabolic_II","B13805","20212097_SS211647","PASS",1.0414,0.153,"Plate control","PASS","PFC",1.11187915751711,"Baseline","Baseline"
674,"PFC_16",86,"OID30094","B6SEH8","ERVV-1",0.9545,"Cardiometabolic_II","B13805","20212097_SS211647","PASS",1.0414,0.4733,"Plate control","PASS","PFC",1.38828137399856,"1h","1h"
675,"PFC_24",87,"OID30094","B6SEH8","ERVV-1",0.9545,"Cardiometabolic_II","B13805","20212097_SS211647","PASS",1.0414,-0.281,"Plate control","PASS","PFC",0.823020345278708,"24h","24h"
676,"PFC_01",1,"OID30095","O95858","TSPAN15",0.8636,"Cardiometabolic_II","B13805","20212097_SS211647","WARN",1.2116,0.7118,"Plate control","PASS","PFC",1.63784632626812,"Baseline","Baseline"
677,"PFC_09",2,"OID30095","O95858","TSPAN15",0.8636,"Cardiometabolic_II","B13805","20212097_SS211647","PASS",1.2116,-0.3344,"Plate control","PASS","PFC",0.793113914834435,"Baseline","Baseline"
678,"PFC_17",3,"OID30095","O95858","TSPAN15",0.8636,"Cardiometabolic_II","B13805","20212097_SS211647","PASS",1.2116,1.4962,"Plate control","PASS","PFC",2.82098696569533,"1h","1h"
679,"PFC_25",4,"OID30095","O95858","TSPAN15",0.8636,"Cardiometabolic_II","B13805","20212097_SS211647","PASS",1.2116,0.0829,"Plate control","PASS","PFC",1.05914491780811,"24h","24h"
680,"PFC_02",13,"OID30095","O95858","TSPAN15",0.8636,"Cardiometabolic_II","B13805","20212097_SS211647","PASS",1.2116,-0.2131,"Plate control","PASS","PFC",0.862681545942089,"Baseline","Baseline"
681,"PFC_10",14,"OID30095","O95858","TSPAN15",0.8636,"Cardiometabolic_II","B13805","20212097_SS211647","PASS",1.2116,0.4469,"Plate control","PASS","PFC",1.36310812055283,"1h","1h"
682,"PFC_18",15,"OID30095","O95858","TSPAN15",0.8636,"Cardiometabolic_II","B13805","20212097_SS211647","PASS",1.2116,0.5824,"Plate control","PASS","PFC",1.49733807951323,"1h","1h"
683,"PFC_26",16,"OID30095","O95858","TSPAN15",0.8636,"Cardiometabolic_II","B13805","20212097_SS211647","PASS",1.2116,1.7537,"Plate control","PASS","PFC",3.37222313399937,"24h","24h"
684,"PFC_03",25,"OID30095","O95858","TSPAN15",0.8636,"Cardiometabolic_II","B13805","20212097_SS211647","PASS",1.2116,-0.5665,"Plate control","PASS","PFC",0.675252976861179,"Baseline","Baseline"
685,"PFC_11",26,"OID30095","O95858","TSPAN15",0.8636,"Cardiometabolic_II","B13805","20212097_SS211647","PASS",1.2116,0.0748,"Plate control","PASS","PFC",1.05321501922974,"1h","1h"
686,"PFC_19",27,"OID30095","O95858","TSPAN15",0.8636,"Cardiometabolic_II","B13805","20212097_SS211647","PASS",1.2116,0.9022,"Plate control","PASS","PFC",1.86891376226969,"24h","24h"
687,"PFC_27",28,"OID30095","O95858","TSPAN15",0.8636,"Cardiometabolic_II","B13805","20212097_SS211647","PASS",1.2116,0.6432,"Plate control","PASS","PFC",1.56178948020311,"24h","24h"
688,"PFC_04",37,"OID30095","O95858","TSPAN15",0.8636,"Cardiometabolic_II","B13805","20212097_SS211647","PASS",1.2116,0.2721,"Plate control","PASS","PFC",1.20756429056401,"Baseline","Baseline"
689,"PFC_12",38,"OID30095","O95858","TSPAN15",0.8636,"Cardiometabolic_II","B13805","20212097_SS211647","PASS",1.2116,0.1753,"Plate control","PASS","PFC",1.12919919076471,"1h","1h"
690,"PFC_20",39,"OID30095","O95858","TSPAN15",0.8636,"Cardiometabolic_II","B13805","20212097_SS211647","PASS",1.2116,0.9159,"Plate control","PASS","PFC",1.88674571801066,"24h","24h"
691,"PFC_05",49,"OID30095","O95858","TSPAN15",0.8636,"Cardiometabolic_II","B13805","20212097_SS211647","PASS",1.2116,0.5044,"Plate control","PASS","PFC",1.41853328209942,"Baseline","Baseline"
692,"PFC_13",50,"OID30095","O95858","TSPAN15",0.8636,"Cardiometabolic_II","B13805","20212097_SS211647","PASS",1.2116,0.0618,"Plate control","PASS","PFC",1.04376722053042,"1h","1h"
693,"PFC_21",51,"OID30095","O95858","TSPAN15",0.8636,"Cardiometabolic_II","B13805","20212097_SS211647","PASS",1.2116,0.4318,"Plate control","PASS","PFC",1.34891552197911,"24h","24h"
694,"PFC_06",61,"OID30095","O95858","TSPAN15",0.8636,"Cardiometabolic_II","B13805","20212097_SS211647","PASS",1.2116,-0.183,"Plate control","PASS","PFC",0.880869374126979,"Baseline","Baseline"
695,"PFC_14",62,"OID30095","O95858","TSPAN15",0.8636,"Cardiometabolic_II","B13805","20212097_SS211647","PASS",1.2116,0.3996,"Plate control","PASS","PFC",1.3191421162099,"1h","1h"
696,"PFC_22",63,"OID30095","O95858","TSPAN15",0.8636,"Cardiometabolic_II","B13805","20212097_SS211647","PASS",1.2116,0.3849,"Plate control","PASS","PFC",1.3057692749229,"24h","24h"
697,"PFC_07",73,"OID30095","O95858","TSPAN15",0.8636,"Cardiometabolic_II","B13805","20212097_SS211647","PASS",1.2116,-0.0319,"Plate control","PASS","PFC",0.978131269990864,"Baseline","Baseline"
698,"PFC_15",74,"OID30095","O95858","TSPAN15",0.8636,"Cardiometabolic_II","B13805","20212097_SS211647","PASS",1.2116,-0.7098,"Plate control","PASS","PFC",0.61140489168665,"1h","1h"
699,"PFC_23",75,"OID30095","O95858","TSPAN15",0.8636,"Cardiometabolic_II","B13805","20212097_SS211647","PASS",1.2116,0.9637,"Plate control","PASS","PFC",1.95030532629456,"24h","24h"
700,"PFC_08",85,"OID30095","O95858","TSPAN15",0.8636,"Cardiometabolic_II","B13805","20212097_SS211647","PASS",1.2116,2.0568,"Plate control","PASS","PFC",4.16062424111399,"Baseline","Baseline"
701,"PFC_16",86,"OID30095","O95858","TSPAN15",0.8636,"Cardiometabolic_II","B13805","20212097_SS211647","PASS",1.2116,-0.5008,"Plate control","PASS","PFC",0.706714786623175,"1h","1h"
702,"PFC_24",87,"OID30095","O95858","TSPAN15",0.8636,"Cardiometabolic_II","B13805","20212097_SS211647","PASS",1.2116,0.5701,"Plate control","PASS","PFC",1.4846264735281,"24h","24h"
703,"PFC_01",1,"OID30096","Q9H347","UBQLN3",0.75,"Cardiometabolic_II","B13805","20212097_SS211647","WARN",-0.214,-0.9,"Plate control","PASS","PFC",0.535886731268147,"Baseline","Baseline"
704,"PFC_09",2,"OID30096","Q9H347","UBQLN3",0.75,"Cardiometabolic_II","B13805","20212097_SS211647","PASS",-0.214,-0.8407,"Plate control","PASS","PFC",0.558372579233592,"Baseline","Baseline"
705,"PFC_17",3,"OID30096","Q9H347","UBQLN3",0.75,"Cardiometabolic_II","B13805","20212097_SS211647","PASS",-0.214,2.2243,"Plate control","PASS","PFC",4.67284116743497,"1h","1h"
706,"PFC_25",4,"OID30096","Q9H347","UBQLN3",0.75,"Cardiometabolic_II","B13805","20212097_SS211647","PASS",-0.214,-0.5822,"Plate control","PASS","PFC",0.667944436015916,"24h","24h"
707,"PFC_02",13,"OID30096","Q9H347","UBQLN3",0.75,"Cardiometabolic_II","B13805","20212097_SS211647","PASS",-0.214,-0.6602,"Plate control","PASS","PFC",0.632790567504786,"Baseline","Baseline"
708,"PFC_10",14,"OID30096","Q9H347","UBQLN3",0.75,"Cardiometabolic_II","B13805","20212097_SS211647","PASS",-0.214,-0.1151,"Plate control","PASS","PFC",0.923318308963027,"1h","1h"
709,"PFC_18",15,"OID30096","Q9H347","UBQLN3",0.75,"Cardiometabolic_II","B13805","20212097_SS211647","PASS",-0.214,-0.4609,"Plate control","PASS","PFC",0.726532882462307,"1h","1h"
710,"PFC_26",16,"OID30096","Q9H347","UBQLN3",0.75,"Cardiometabolic_II","B13805","20212097_SS211647","PASS",-0.214,2.297,"Plate control","PASS","PFC",4.9143479217826,"24h","24h"
711,"PFC_03",25,"OID30096","Q9H347","UBQLN3",0.75,"Cardiometabolic_II","B13805","20212097_SS211647","PASS",-0.214,2.7273,"Plate control","PASS","PFC",6.62215142313856,"Baseline","Baseline"
712,"PFC_11",26,"OID30096","Q9H347","UBQLN3",0.75,"Cardiometabolic_II","B13805","20212097_SS211647","PASS",-0.214,-0.3684,"Plate control","PASS","PFC",0.774641125057415,"1h","1h"
713,"PFC_19",27,"OID30096","Q9H347","UBQLN3",0.75,"Cardiometabolic_II","B13805","20212097_SS211647","PASS",-0.214,-0.5107,"Plate control","PASS","PFC",0.701881800062874,"24h","24h"
714,"PFC_27",28,"OID30096","Q9H347","UBQLN3",0.75,"Cardiometabolic_II","B13805","20212097_SS211647","PASS",-0.214,-0.3041,"Plate control","PASS","PFC",0.809947330415445,"24h","24h"
715,"PFC_04",37,"OID30096","Q9H347","UBQLN3",0.75,"Cardiometabolic_II","B13805","20212097_SS211647","PASS",-0.214,1.5258,"Plate control","PASS","PFC",2.87946343096741,"Baseline","Baseline"
716,"PFC_12",38,"OID30096","Q9H347","UBQLN3",0.75,"Cardiometabolic_II","B13805","20212097_SS211647","PASS",-0.214,2.3679,"Plate control","PASS","PFC",5.16189215430729,"1h","1h"
717,"PFC_20",39,"OID30096","Q9H347","UBQLN3",0.75,"Cardiometabolic_II","B13805","20212097_SS211647","PASS",-0.214,-0.4798,"Plate control","PASS","PFC",0.717077025101434,"24h","24h"
718,"PFC_05",49,"OID30096","Q9H347","UBQLN3",0.75,"Cardiometabolic_II","B13805","20212097_SS211647","PASS",-0.214,-1.2314,"Plate control","PASS","PFC",0.425903945518163,"Baseline","Baseline"
719,"PFC_13",50,"OID30096","Q9H347","UBQLN3",0.75,"Cardiometabolic_II","B13805","20212097_SS211647","PASS",-0.214,0.9386,"Plate control","PASS","PFC",1.9166673902706,"1h","1h"
720,"PFC_21",51,"OID30096","Q9H347","UBQLN3",0.75,"Cardiometabolic_II","B13805","20212097_SS211647","PASS",-0.214,2.4453,"Plate control","PASS","PFC",5.44638889298465,"24h","24h"
721,"PFC_06",61,"OID30096","Q9H347","UBQLN3",0.75,"Cardiometabolic_II","B13805","20212097_SS211647","PASS",-0.214,-0.7675,"Plate control","PASS","PFC",0.587434539629019,"Baseline","Baseline"
722,"PFC_14",62,"OID30096","Q9H347","UBQLN3",0.75,"Cardiometabolic_II","B13805","20212097_SS211647","PASS",-0.214,-0.4435,"Plate control","PASS","PFC",0.735348476398645,"1h","1h"
723,"PFC_22",63,"OID30096","Q9H347","UBQLN3",0.75,"Cardiometabolic_II","B13805","20212097_SS211647","PASS",-0.214,0.7583,"Plate control","PASS","PFC",1.69149627494894,"24h","24h"
724,"PFC_07",73,"OID30096","Q9H347","UBQLN3",0.75,"Cardiometabolic_II","B13805","20212097_SS211647","PASS",-0.214,-0.564,"Plate control","PASS","PFC",0.676424115525137,"Baseline","Baseline"
725,"PFC_15",74,"OID30096","Q9H347","UBQLN3",0.75,"Cardiometabolic_II","B13805","20212097_SS211647","PASS",-0.214,-0.3589,"Plate control","PASS","PFC",0.779758889505757,"1h","1h"
726,"PFC_23",75,"OID30096","Q9H347","UBQLN3",0.75,"Cardiometabolic_II","B13805","20212097_SS211647","PASS",-0.214,-0.6863,"Plate control","PASS","PFC",0.621445595036524,"24h","24h"
727,"PFC_08",85,"OID30096","Q9H347","UBQLN3",0.75,"Cardiometabolic_II","B13805","20212097_SS211647","PASS",-0.214,2.1748,"Plate control","PASS","PFC",4.51523163182035,"Baseline","Baseline"
728,"PFC_16",86,"OID30096","Q9H347","UBQLN3",0.75,"Cardiometabolic_II","B13805","20212097_SS211647","PASS",-0.214,-0.2471,"Plate control","PASS","PFC",0.842588423699958,"1h","1h"
729,"PFC_24",87,"OID30096","Q9H347","UBQLN3",0.75,"Cardiometabolic_II","B13805","20212097_SS211647","PASS",-0.214,-0.5005,"Plate control","PASS","PFC",0.706861759112223,"24h","24h"
730,"PFC_01",1,"OID30097","P06729","CD2",0.9091,"Cardiometabolic_II","B13805","20212097_SS211647","WARN",0.8652,0.1025,"Plate control","PASS","PFC",1.07363231452805,"Baseline","Baseline"
731,"PFC_09",2,"OID30097","P06729","CD2",0.9091,"Cardiometabolic_II","B13805","20212097_SS211647","PASS",0.8652,0.1233,"Plate control","PASS","PFC",1.08922348944552,"Baseline","Baseline"
732,"PFC_17",3,"OID30097","P06729","CD2",0.9091,"Cardiometabolic_II","B13805","20212097_SS211647","PASS",0.8652,-0.2347,"Plate control","PASS","PFC",0.849861704538896,"1h","1h"
733,"PFC_25",4,"OID30097","P06729","CD2",0.9091,"Cardiometabolic_II","B13805","20212097_SS211647","PASS",0.8652,-0.4192,"Plate control","PASS","PFC",0.747839199468306,"24h","24h"
734,"PFC_02",13,"OID30097","P06729","CD2",0.9091,"Cardiometabolic_II","B13805","20212097_SS211647","PASS",0.8652,1.3463,"Plate control","PASS","PFC",2.54259204079464,"Baseline","Baseline"
735,"PFC_10",14,"OID30097","P06729","CD2",0.9091,"Cardiometabolic_II","B13805","20212097_SS211647","PASS",0.8652,-0.4529,"Plate control","PASS","PFC",0.730572826967369,"1h","1h"
736,"PFC_18",15,"OID30097","P06729","CD2",0.9091,"Cardiometabolic_II","B13805","20212097_SS211647","PASS",0.8652,-0.2942,"Plate control","PASS","PFC",0.815524429810384,"1h","1h"
737,"PFC_26",16,"OID30097","P06729","CD2",0.9091,"Cardiometabolic_II","B13805","20212097_SS211647","PASS",0.8652,-0.1123,"Plate control","PASS","PFC",0.925112036395268,"24h","24h"
738,"PFC_03",25,"OID30097","P06729","CD2",0.9091,"Cardiometabolic_II","B13805","20212097_SS211647","PASS",0.8652,-0.4065,"Plate control","PASS","PFC",0.754451465991974,"Baseline","Baseline"
739,"PFC_11",26,"OID30097","P06729","CD2",0.9091,"Cardiometabolic_II","B13805","20212097_SS211647","PASS",0.8652,1.6915,"Plate control","PASS","PFC",3.22992351025043,"1h","1h"
740,"PFC_19",27,"OID30097","P06729","CD2",0.9091,"Cardiometabolic_II","B13805","20212097_SS211647","PASS",0.8652,-0.3263,"Plate control","PASS","PFC",0.797579370596264,"24h","24h"
741,"PFC_27",28,"OID30097","P06729","CD2",0.9091,"Cardiometabolic_II","B13805","20212097_SS211647","PASS",0.8652,0.0197,"Plate control","PASS","PFC",1.01374865476432,"24h","24h"
742,"PFC_04",37,"OID30097","P06729","CD2",0.9091,"Cardiometabolic_II","B13805","20212097_SS211647","PASS",0.8652,0.4706,"Plate control","PASS","PFC",1.38568563874749,"Baseline","Baseline"
743,"PFC_12",38,"OID30097","P06729","CD2",0.9091,"Cardiometabolic_II","B13805","20212097_SS211647","PASS",0.8652,-0.2317,"Plate control","PASS","PFC",0.85163078098005,"1h","1h"
744,"PFC_20",39,"OID30097","P06729","CD2",0.9091,"Cardiometabolic_II","B13805","20212097_SS211647","PASS",0.8652,1.574,"Plate control","PASS","PFC",2.97729051033308,"24h","24h"
745,"PFC_05",49,"OID30097","P06729","CD2",0.9091,"Cardiometabolic_II","B13805","20212097_SS211647","PASS",0.8652,-0.8201,"Plate control","PASS","PFC",0.566402681245062,"Baseline","Baseline"
746,"PFC_13",50,"OID30097","P06729","CD2",0.9091,"Cardiometabolic_II","B13805","20212097_SS211647","PASS",0.8652,0.0258,"Plate control","PASS","PFC",1.01804405910787,"1h","1h"
747,"PFC_21",51,"OID30097","P06729","CD2",0.9091,"Cardiometabolic_II","B13805","20212097_SS211647","PASS",0.8652,-0.0477,"Plate control","PASS","PFC",0.967477489986477,"24h","24h"
748,"PFC_06",61,"OID30097","P06729","CD2",0.9091,"Cardiometabolic_II","B13805","20212097_SS211647","PASS",0.8652,-0.238,"Plate control","PASS","PFC",0.847919964633148,"Baseline","Baseline"
749,"PFC_14",62,"OID30097","P06729","CD2",0.9091,"Cardiometabolic_II","B13805","20212097_SS211647","PASS",0.8652,0.0146,"Plate control","PASS","PFC",1.01017132869264,"1h","1h"
750,"PFC_22",63,"OID30097","P06729","CD2",0.9091,"Cardiometabolic_II","B13805","20212097_SS211647","PASS",0.8652,0.0803,"Plate control","PASS","PFC",1.05723786413849,"24h","24h"
751,"PFC_07",73,"OID30097","P06729","CD2",0.9091,"Cardiometabolic_II","B13805","20212097_SS211647","PASS",0.8652,-0.4145,"Plate control","PASS","PFC",0.750279476640792,"Baseline","Baseline"
752,"PFC_15",74,"OID30097","P06729","CD2",0.9091,"Cardiometabolic_II","B13805","20212097_SS211647","PASS",0.8652,-0.4737,"Plate control","PASS","PFC",0.720115387474401,"1h","1h"
753,"PFC_23",75,"OID30097","P06729","CD2",0.9091,"Cardiometabolic_II","B13805","20212097_SS211647","PASS",0.8652,0.0227,"Plate control","PASS","PFC",1.01585887911355,"24h","24h"
754,"PFC_08",85,"OID30097","P06729","CD2",0.9091,"Cardiometabolic_II","B13805","20212097_SS211647","PASS",0.8652,-0.6057,"Plate control","PASS","PFC",0.657152450378234,"Baseline","Baseline"
755,"PFC_16",86,"OID30097","P06729","CD2",0.9091,"Cardiometabolic_II","B13805","20212097_SS211647","PASS",0.8652,-0.5074,"Plate control","PASS","PFC",0.703489112022901,"1h","1h"
756,"PFC_24",87,"OID30097","P06729","CD2",0.9091,"Cardiometabolic_II","B13805","20212097_SS211647","PASS",0.8652,-0.4435,"Plate control","PASS","PFC",0.735348476398645,"24h","24h"
757,"PFC_01",1,"OID30126","Q9BW61","DDA1",0.5227,"Cardiometabolic_II","B13805","20212097_SS211647","WARN",-0.1961,-0.8567,"Plate control","PASS","PFC",0.55221424136663,"Baseline","Baseline"
758,"PFC_09",2,"OID30126","Q9BW61","DDA1",0.5227,"Cardiometabolic_II","B13805","20212097_SS211647","PASS",-0.1961,-1.0679,"Plate control","PASS","PFC",0.477012838138279,"Baseline","Baseline"
759,"PFC_17",3,"OID30126","Q9BW61","DDA1",0.5227,"Cardiometabolic_II","B13805","20212097_SS211647","PASS",-0.1961,0.1402,"Plate control","PASS","PFC",1.10205788295024,"1h","1h"
760,"PFC_25",4,"OID30126","Q9BW61","DDA1",0.5227,"Cardiometabolic_II","B13805","20212097_SS211647","PASS",-0.1961,-0.4675,"Plate control","PASS","PFC",0.723216751670109,"24h","24h"
761,"PFC_02",13,"OID30126","Q9BW61","DDA1",0.5227,"Cardiometabolic_II","B13805","20212097_SS211647","PASS",-0.1961,-0.0492,"Plate control","PASS","PFC",0.966472106294542,"Baseline","Baseline"
762,"PFC_10",14,"OID30126","Q9BW61","DDA1",0.5227,"Cardiometabolic_II","B13805","20212097_SS211647","PASS",-0.1961,-0.371,"Plate control","PASS","PFC",0.773246337455317,"1h","1h"
763,"PFC_18",15,"OID30126","Q9BW61","DDA1",0.5227,"Cardiometabolic_II","B13805","20212097_SS211647","PASS",-0.1961,0.0992,"Plate control","PASS","PFC",1.07117930988261,"1h","1h"
764,"PFC_26",16,"OID30126","Q9BW61","DDA1",0.5227,"Cardiometabolic_II","B13805","20212097_SS211647","PASS",-0.1961,-0.6412,"Plate control","PASS","PFC",0.641179408879808,"24h","24h"
765,"PFC_03",25,"OID30126","Q9BW61","DDA1",0.5227,"Cardiometabolic_II","B13805","20212097_SS211647","PASS",-0.1961,-0.8173,"Plate control","PASS","PFC",0.567503029865014,"Baseline","Baseline"
766,"PFC_11",26,"OID30126","Q9BW61","DDA1",0.5227,"Cardiometabolic_II","B13805","20212097_SS211647","PASS",-0.1961,0.0104,"Plate control","PASS","PFC",1.00723477612407,"1h","1h"
767,"PFC_19",27,"OID30126","Q9BW61","DDA1",0.5227,"Cardiometabolic_II","B13805","20212097_SS211647","PASS",-0.1961,-0.3653,"Plate control","PASS","PFC",0.776307429623983,"24h","24h"
768,"PFC_27",28,"OID30126","Q9BW61","DDA1",0.5227,"Cardiometabolic_II","B13805","20212097_SS211647","PASS",-0.1961,-0.0321,"Plate control","PASS","PFC",0.97799568160295,"24h","24h"
769,"PFC_04",37,"OID30126","Q9BW61","DDA1",0.5227,"Cardiometabolic_II","B13805","20212097_SS211647","PASS",-0.1961,-0.2511,"Plate control","PASS","PFC",0.840255508141077,"Baseline","Baseline"
770,"PFC_12",38,"OID30126","Q9BW61","DDA1",0.5227,"Cardiometabolic_II","B13805","20212097_SS211647","PASS",-0.1961,-0.3914,"Plate control","PASS","PFC",0.762389418091795,"1h","1h"
771,"PFC_20",39,"OID30126","Q9BW61","DDA1",0.5227,"Cardiometabolic_II","B13805","20212097_SS211647","PASS",-0.1961,0.2231,"Plate control","PASS","PFC",1.16723900585711,"24h","24h"
772,"PFC_05",49,"OID30126","Q9BW61","DDA1",0.5227,"Cardiometabolic_II","B13805","20212097_SS211647","PASS",-0.1961,-0.8344,"Plate control","PASS","PFC",0.560816227432839,"Baseline","Baseline"
773,"PFC_13",50,"OID30126","Q9BW61","DDA1",0.5227,"Cardiometabolic_II","B13805","20212097_SS211647","PASS",-0.1961,-0.3279,"Plate control","PASS","PFC",0.796695317083034,"1h","1h"
774,"PFC_21",51,"OID30126","Q9BW61","DDA1",0.5227,"Cardiometabolic_II","B13805","20212097_SS211647","PASS",-0.1961,-0.5676,"Plate control","PASS","PFC",0.674738318423054,"24h","24h"
775,"PFC_06",61,"OID30126","Q9BW61","DDA1",0.5227,"Cardiometabolic_II","B13805","20212097_SS211647","PASS",-0.1961,-0.5366,"Plate control","PASS","PFC",0.689393690509711,"Baseline","Baseline"
776,"PFC_14",62,"OID30126","Q9BW61","DDA1",0.5227,"Cardiometabolic_II","B13805","20212097_SS211647","PASS",-0.1961,-0.0417,"Plate control","PASS","PFC",0.971509494258729,"1h","1h"
777,"PFC_22",63,"OID30126","Q9BW61","DDA1",0.5227,"Cardiometabolic_II","B13805","20212097_SS211647","PASS",-0.1961,-0.416,"Plate control","PASS","PFC",0.749499800877774,"24h","24h"
778,"PFC_07",73,"OID30126","Q9BW61","DDA1",0.5227,"Cardiometabolic_II","B13805","20212097_SS211647","PASS",-0.1961,-0.7225,"Plate control","PASS","PFC",0.606046333475896,"Baseline","Baseline"
779,"PFC_15",74,"OID30126","Q9BW61","DDA1",0.5227,"Cardiometabolic_II","B13805","20212097_SS211647","PASS",-0.1961,-0.6927,"Plate control","PASS","PFC",0.618694879951591,"1h","1h"
780,"PFC_23",75,"OID30126","Q9BW61","DDA1",0.5227,"Cardiometabolic_II","B13805","20212097_SS211647","PASS",-0.1961,0.3105,"Plate control","PASS","PFC",1.24013742434872,"24h","24h"
781,"PFC_08",85,"OID30126","Q9BW61","DDA1",0.5227,"Cardiometabolic_II","B13805","20212097_SS211647","PASS",-0.1961,-0.5393,"Plate control","PASS","PFC",0.688104698567706,"Baseline","Baseline"
782,"PFC_16",86,"OID30126","Q9BW61","DDA1",0.5227,"Cardiometabolic_II","B13805","20212097_SS211647","PASS",-0.1961,0.0345,"Plate control","PASS","PFC",1.02420180022052,"1h","1h"
783,"PFC_24",87,"OID30126","Q9BW61","DDA1",0.5227,"Cardiometabolic_II","B13805","20212097_SS211647","PASS",-0.1961,-0.2511,"Plate control","PASS","PFC",0.840255508141077,"24h","24h"
784,"PFC_01",1,"OID30127","Q15370","ELOB",0.5227,"Cardiometabolic_II","B13805","20212097_SS211647","WARN",0.4654,0.3349,"Plate control","PASS","PFC",1.26128997984189,"Baseline","Baseline"

Supplemental table 1_Olink proximity extension assay (PEA) data

,"SampleID","Index","OlinkID","UniProt","Assay","MissingFreq","Panel","Panel_Lot_Nr","PlateID","QC_Warning","LOD","NPX","Normalization","Assay_Warning","condition","Value","group","alt_group"
785,"PFC_09",2,"OID30127","Q15370","ELOB",0.5227,"Cardiometabolic_II","B13805","20212097_SS211647","PASS",0.4654,0.4801,"Plate control","PASS","PFC",1.39484034596502,"Baseline","Baseline"
786,"PFC_17",3,"OID30127","Q15370","ELOB",0.5227,"Cardiometabolic_II","B13805","20212097_SS211647","PASS",0.4654,0.913,"Plate control","PASS","PFC",1.88295692908241,"1h","1h"
787,"PFC_25",4,"OID30127","Q15370","ELOB",0.5227,"Cardiometabolic_II","B13805","20212097_SS211647","PASS",0.4654,0.4732,"Plate control","PASS","PFC",1.38818514900148,"24h","24h"
788,"PFC_02",13,"OID30127","Q15370","ELOB",0.5227,"Cardiometabolic_II","B13805","20212097_SS211647","PASS",0.4654,-0.0164,"Plate control","PASS","PFC",0.988696753428805,"Baseline","Baseline"
789,"PFC_10",14,"OID30127","Q15370","ELOB",0.5227,"Cardiometabolic_II","B13805","20212097_SS211647","PASS",0.4654,0.4105,"Plate control","PASS","PFC",1.32914638131506,"1h","1h"
790,"PFC_18",15,"OID30127","Q15370","ELOB",0.5227,"Cardiometabolic_II","B13805","20212097_SS211647","PASS",0.4654,0.4122,"Plate control","PASS","PFC",1.33071350435711,"1h","1h"
791,"PFC_26",16,"OID30127","Q15370","ELOB",0.5227,"Cardiometabolic_II","B13805","20212097_SS211647","PASS",0.4654,0.2571,"Plate control","PASS","PFC",1.19507403820598,"24h","24h"
792,"PFC_03",25,"OID30127","Q15370","ELOB",0.5227,"Cardiometabolic_II","B13805","20212097_SS211647","PASS",0.4654,-0.0065,"Plate control","PASS","PFC",0.995504677670617,"Baseline","Baseline"
793,"PFC_11",26,"OID30127","Q15370","ELOB",0.5227,"Cardiometabolic_II","B13805","20212097_SS211647","PASS",0.4654,0.8819,"Plate control","PASS","PFC",1.84280063476398,"1h","1h"
794,"PFC_19",27,"OID30127","Q15370","ELOB",0.5227,"Cardiometabolic_II","B13805","20212097_SS211647","PASS",0.4654,0.4421,"Plate control","PASS","PFC",1.35858045090626,"24h","24h"
795,"PFC_27",28,"OID30127","Q15370","ELOB",0.5227,"Cardiometabolic_II","B13805","20212097_SS211647","PASS",0.4654,0.2505,"Plate control","PASS","PFC",1.18961933421017,"24h","24h"
796,"PFC_04",37,"OID30127","Q15370","ELOB",0.5227,"Cardiometabolic_II","B13805","20212097_SS211647","PASS",0.4654,0.3724,"Plate control","PASS","PFC",1.2945045175942,"Baseline","Baseline"
797,"PFC_12",38,"OID30127","Q15370","ELOB",0.5227,"Cardiometabolic_II","B13805","20212097_SS211647","PASS",0.4654,0.5863,"Plate control","PASS","PFC",1.5013912705938,"1h","1h"
798,"PFC_20",39,"OID30127","Q15370","ELOB",0.5227,"Cardiometabolic_II","B13805","20212097_SS211647","PASS",0.4654,0.9218,"Plate control","PASS","PFC",1.89447749264701,"24h","24h"
799,"PFC_05",49,"OID30127","Q15370","ELOB",0.5227,"Cardiometabolic_II","B13805","20212097_SS211647","PASS",0.4654,-0.4033,"Plate control","PASS","PFC",0.756126750155591,"Baseline","Baseline"
800,"PFC_13",50,"OID30127","Q15370","ELOB",0.5227,"Cardiometabolic_II","B13805","20212097_SS211647","PASS",0.4654,0.2271,"Plate control","PASS","PFC",1.17047977013811,"1h","1h"
801,"PFC_21",51,"OID30127","Q15370","ELOB",0.5227,"Cardiometabolic_II","B13805","20212097_SS211647","PASS",0.4654,-0.0908,"Plate control","PASS","PFC",0.939001911598456,"24h","24h"
802,"PFC_06",61,"OID30127","Q15370","ELOB",0.5227,"Cardiometabolic_II","B13805","20212097_SS211647","PASS",0.4654,0.0808,"Plate control","PASS","PFC",1.05760433836232,"Baseline","Baseline"
803,"PFC_14",62,"OID30127","Q15370","ELOB",0.5227,"Cardiometabolic_II","B13805","20212097_SS211647","PASS",0.4654,0.8693,"Plate control","PASS","PFC",1.82677632798949,"1h","1h"
804,"PFC_22",63,"OID30127","Q15370","ELOB",0.5227,"Cardiometabolic_II","B13805","20212097_SS211647","PASS",0.4654,0.4545,"Plate control","PASS","PFC",1.37030781017657,"24h","24h"
805,"PFC_07",73,"OID30127","Q15370","ELOB",0.5227,"Cardiometabolic_II","B13805","20212097_SS211647","PASS",0.4654,0.5138,"Plate control","PASS","PFC",1.42780603001974,"Baseline","Baseline"
806,"PFC_15",74,"OID30127","Q15370","ELOB",0.5227,"Cardiometabolic_II","B13805","20212097_SS211647","PASS",0.4654,-0.4295,"Plate control","PASS","PFC",0.742519078229278,"1h","1h"
807,"PFC_23",75,"OID30127","Q15370","ELOB",0.5227,"Cardiometabolic_II","B13805","20212097_SS211647","PASS",0.4654,0.3072,"Plate control","PASS","PFC",1.23730399355332,"24h","24h"
808,"PFC_08",85,"OID30127","Q15370","ELOB",0.5227,"Cardiometabolic_II","B13805","20212097_SS211647","PASS",0.4654,0.3944,"Plate control","PASS","PFC",1.31439600458749,"Baseline","Baseline"
809,"PFC_16",86,"OID30127","Q15370","ELOB",0.5227,"Cardiometabolic_II","B13805","20212097_SS211647","PASS",0.4654,0.5013,"Plate control","PASS","PFC",1.41548847227847,"1h","1h"
810,"PFC_24",87,"OID30127","Q15370","ELOB",0.5227,"Cardiometabolic_II","B13805","20212097_SS211647","PASS",0.4654,0.3676,"Plate control","PASS","PFC",1.29020472015093,"24h","24h"
811,"PFC_01",1,"OID30128","Q8NET8","TRPV3",0.9091,"Cardiometabolic_II","B13805","20212097_SS211647","WARN",0.3909,-0.4585,"Plate control","PASS","PFC",0.7277425144527,"Baseline","Baseline"
812,"PFC_09",2,"OID30128","Q8NET8","TRPV3",0.9091,"Cardiometabolic_II","B13805","20212097_SS211647","PASS",0.3909,-0.3793,"Plate control","PASS","PFC",0.76881052935676,"Baseline","Baseline"
813,"PFC_17",3,"OID30128","Q8NET8","TRPV3",0.9091,"Cardiometabolic_II","B13805","20212097_SS211647","PASS",0.3909,-0.4902,"Plate control","PASS","PFC",0.711926397002306,"1h","1h"
814,"PFC_25",4,"OID30128","Q8NET8","TRPV3",0.9091,"Cardiometabolic_II","B13805","20212097_SS211647","PASS",0.3909,-0.3986,"Plate control","PASS","PFC",0.758594070468865,"24h","24h"
815,"PFC_02",13,"OID30128","Q8NET8","TRPV3",0.9091,"Cardiometabolic_II","B13805","20212097_SS211647","PASS",0.3909,-1.7116,"Plate control","PASS","PFC",0.305321269463804,"Baseline","Baseline"
816,"PFC_10",14,"OID30128","Q8NET8","TRPV3",0.9091,"Cardiometabolic_II","B13805","20212097_SS211647","PASS",0.3909,-0.3096,"Plate control","PASS","PFC",0.80686543881372,"1h","1h"
817,"PFC_18",15,"OID30128","Q8NET8","TRPV3",0.9091,"Cardiometabolic_II","B13805","20212097_SS211647","PASS",0.3909,-0.1434,"Plate control","PASS","PFC",0.905382922766993,"1h","1h"
818,"PFC_26",16,"OID30128","Q8NET8","TRPV3",0.9091,"Cardiometabolic_II","B13805","20212097_SS211647","PASS",0.3909,0.4991,"Plate control","PASS","PFC",1.41333160516934,"24h","24h"
819,"PFC_03",25,"OID30128","Q8NET8","TRPV3",0.9091,"Cardiometabolic_II","B13805","20212097_SS211647","PASS",0.3909,-0.2205,"Plate control","PASS","PFC",0.858267931888545,"Baseline","Baseline"
820,"PFC_11",26,"OID30128","Q8NET8","TRPV3",0.9091,"Cardiometabolic_II","B13805","20212097_SS211647","PASS",0.3909,-0.2677,"Plate control","PASS","PFC",0.830642733433942,"1h","1h"
821,"PFC_19",27,"OID30128","Q8NET8","TRPV3",0.9091,"Cardiometabolic_II","B13805","20212097_SS211647","PASS",0.3909,-1.0511,"Plate control","PASS","PFC",0.482600059989426,"24h","24h"
822,"PFC_27",28,"OID30128","Q8NET8","TRPV3",0.9091,"Cardiometabolic_II","B13805","20212097_SS211647","PASS",0.3909,0.0103,"Plate control","PASS","PFC",1.00716496234914,"24h","24h"
823,"PFC_04",37,"OID30128","Q8NET8","TRPV3",0.9091,"Cardiometabolic_II","B13805","20212097_SS211647","PASS",0.3909,0.1203,"Plate control","PASS","PFC",1.08696086618511,"Baseline","Baseline"
824,"PFC_12",38,"OID30128","Q8NET8","TRPV3",0.9091,"Cardiometabolic_II","B13805","20212097_SS211647","PASS",0.3909,-0.337,"Plate control","PASS","PFC",0.791685865871272,"1h","1h"
825,"PFC_20",39,"OID30128","Q8NET8","TRPV3",0.9091,"Cardiometabolic_II","B13805","20212097_SS211647","PASS",0.3909,0.51,"Plate control","PASS","PFC",1.42405019559707,"24h","24h"
826,"PFC_05",49,"OID30128","Q8NET8","TRPV3",0.9091,"Cardiometabolic_II","B13805","20212097_SS211647","PASS",0.3909,-0.222,"Plate control","PASS","PFC",0.857376036634172,"Baseline","Baseline"
827,"PFC_13",50,"OID30128","Q8NET8","TRPV3",0.9091,"Cardiometabolic_II","B13805","20212097_SS211647","PASS",0.3909,-1.5967,"Plate control","PASS","PFC",0.330632397211918,"1h","1h"
828,"PFC_21",51,"OID30128","Q8NET8","TRPV3",0.9091,"Cardiometabolic_II","B13805","20212097_SS211647","PASS",0.3909,-0.1055,"Plate control","PASS","PFC",0.929482752610438,"24h","24h"
829,"PFC_06",61,"OID30128","Q8NET8","TRPV3",0.9091,"Cardiometabolic_II","B13805","20212097_SS211647","PASS",0.3909,-1.1425,"Plate control","PASS","PFC",0.452973953117201,"Baseline","Baseline"
830,"PFC_14",62,"OID30128","Q8NET8","TRPV3",0.9091,"Cardiometabolic_II","B13805","20212097_SS211647","PASS",0.3909,-0.3746,"Plate control","PASS","PFC",0.771319238162195,"1h","1h"
831,"PFC_22",63,"OID30128","Q8NET8","TRPV3",0.9091,"Cardiometabolic_II","B13805","20212097_SS211647","PASS",0.3909,-0.1437,"Plate control","PASS","PFC",0.905194673254291,"24h","24h"
832,"PFC_07",73,"OID30128","Q8NET8","TRPV3",0.9091,"Cardiometabolic_II","B13805","20212097_SS211647","PASS",0.3909,0.7129,"Plate control","PASS","PFC",1.63909559788817,"Baseline","Baseline"
833,"PFC_15",74,"OID30128","Q8NET8","TRPV3",0.9091,"Cardiometabolic_II","B13805","20212097_SS211647","PASS",0.3909,-0.4192,"Plate control","PASS","PFC",0.747839199468306,"1h","1h"
834,"PFC_23",75,"OID30128","Q8NET8","TRPV3",0.9091,"Cardiometabolic_II","B13805","20212097_SS211647","PASS",0.3909,-1.1878,"Plate control","PASS","PFC",0.438971748992881,"24h","24h"
835,"PFC_08",85,"OID30128","Q8NET8","TRPV3",0.9091,"Cardiometabolic_II","B13805","20212097_SS211647","PASS",0.3909,0.0641,"Plate control","PASS","PFC",1.04543256155707,"Baseline","Baseline"
836,"PFC_16",86,"OID30128","Q8NET8","TRPV3",0.9091,"Cardiometabolic_II","B13805","20212097_SS211647","PASS",0.3909,-0.8916,"Plate control","PASS","PFC",0.539015998773932,"1h","1h"
837,"PFC_24",87,"OID30128","Q8NET8","TRPV3",0.9091,"Cardiometabolic_II","B13805","20212097_SS211647","PASS",0.3909,0.0105,"Plate control","PASS","PFC",1.00730459473829,"24h","24h"
838,"PFC_01",1,"OID30129","P05000","IFNW1",0.7273,"Cardiometabolic_II","B13805","20212097_SS211647","WARN",-0.0069,-0.4447,"Plate control","PASS","PFC",0.73473708503664,"Baseline","Baseline"
839,"PFC_09",2,"OID30129","P05000","IFNW1",0.7273,"Cardiometabolic_II","B13805","20212097_SS211647","PASS",-0.0069,-0.3651,"Plate control","PASS","PFC",0.776415056145131,"Baseline","Baseline"
840,"PFC_17",3,"OID30129","P05000","IFNW1",0.7273,"Cardiometabolic_II","B13805","20212097_SS211647","PASS",-0.0069,2.0493,"Plate control","PASS","PFC",4.13905092803825,"1h","1h"
841,"PFC_25",4,"OID30129","P05000","IFNW1",0.7273,"Cardiometabolic_II","B13805","20212097_SS211647","PASS",-0.0069,0.1395,"Plate control","PASS","PFC",1.10152329083351,"24h","24h"
842,"PFC_02",13,"OID30129","P05000","IFNW1",0.7273,"Cardiometabolic_II","B13805","20212097_SS211647","PASS",-0.0069,-0.2955,"Plate control","PASS","PFC",0.814789898802887,"Baseline","Baseline"
843,"PFC_10",14,"OID30129","P05000","IFNW1",0.7273,"Cardiometabolic_II","B13805","20212097_SS211647","PASS",-0.0069,-0.4156,"Plate control","PASS","PFC",0.749707635157915,"1h","1h"
844,"PFC_18",15,"OID30129","P05000","IFNW1",0.7273,"Cardiometabolic_II","B13805","20212097_SS211647","PASS",-0.0069,-0.301,"Plate control","PASS","PFC",0.811689580977279,"1h","1h"
845,"PFC_26",16,"OID30129","P05000","IFNW1",0.7273,"Cardiometabolic_II","B13805","20212097_SS211647","PASS",-0.0069,2.0645,"Plate control","PASS","PFC",4.18288982878513,"24h","24h"
846,"PFC_03",25,"OID30129","P05000","IFNW1",0.7273,"Cardiometabolic_II","B13805","20212097_SS211647","PASS",-0.0069,-0.2233,"Plate control","PASS","PFC",0.856603810492351,"Baseline","Baseline"
847,"PFC_11",26,"OID30129","P05000","IFNW1",0.7273,"Cardiometabolic_II","B13805","20212097_SS211647","PASS",-0.0069,-0.1859,"Plate control","PASS","PFC",0.879100493402846,"1h","1h"
848,"PFC_19",27,"OID30129","P05000","IFNW1",0.7273,"Cardiometabolic_II","B13805","20212097_SS211647","PASS",-0.0069,-0.3855,"Plate control","PASS","PFC",0.765513645755095,"24h","24h"
849,"PFC_27",28,"OID30129","P05000","IFNW1",0.7273,"Cardiometabolic_II","B13805","20212097_SS211647","PASS",-0.0069,0.0011,"Plate control","PASS","PFC",1.00076275264658,"24h","24h"
850,"PFC_04",37,"OID30129","P05000","IFNW1",0.7273,"Cardiometabolic_II","B13805","20212097_SS211647","PASS",-0.0069,-0.5905,"Plate control","PASS","PFC",0.664112703235368,"Baseline","Baseline"
851,"PFC_12",38,"OID30129","P05000","IFNW1",0.7273,"Cardiometabolic_II","B13805","20212097_SS211647","PASS",-0.0069,-0.4581,"Plate control","PASS","PFC",0.727944315495799,"1h","1h"
852,"PFC_20",39,"OID30129","P05000","IFNW1",0.7273,"Cardiometabolic_II","B13805","20212097_SS211647","PASS",-0.0069,0.3359,"Plate control","PASS","PFC",1.26216454250059,"24h","24h"
853,"PFC_05",49,"OID30129","P05000","IFNW1",0.7273,"Cardiometabolic_II","B13805","20212097_SS211647","PASS",-0.0069,-0.2762,"Plate control","PASS","PFC",0.825763181916274,"Baseline","Baseline"
854,"PFC_13",50,"OID30129","P05000","IFNW1",0.7273,"Cardiometabolic_II","B13805","20212097_SS211647","PASS",-0.0069,-0.5716,"Plate control","PASS","PFC",0.67287013761621 3,"1h","1h"
855,"PFC_21",51,"OID30129","P05000","IFNW1",0.7273,"Cardiometabolic_II","B13805","20212097_SS211647","PASS",-0.0069,-0.4582,"Plate control","PASS","PFC",0.727893859989484,"24h","24h"
856,"PFC_06",61,"OID30129","P05000","IFNW1",0.7273,"Cardiometabolic_II","B13805","20212097_SS211647","PASS",-0.0069,-0.4487,"Plate control","PASS","PFC",0.732702782725861,"Baseline","Baseline"
857,"PFC_14",62,"OID30129","P05000","IFNW1",0.7273,"Cardiometabolic_II","B13805","20212097_SS211647","PASS",-0.0069,-0.6941,"Plate control","PASS","PFC",0.618094785909797,"1h","1h"
858,"PFC_22",63,"OID30129","P05000","IFNW1",0.7273,"Cardiometabolic_II","B13805","20212097_SS211647","PASS",-0.0069,-0.5995,"Plate control","PASS","PFC",0.659982648310642,"24h","24h"
859,"PFC_07",73,"OID30129","P05000","IFNW1",0.7273,"Cardiometabolic_II","B13805","20212097_SS211647","PASS",-0.0069,-0.426,"Plate control","PASS","PFC",0.74432262758327,"Baseline","Baseline"
860,"PFC_15",74,"OID30129","P05000","IFNW1",0.7273,"Cardiometabolic_II","B13805","20212097_SS211647","PASS",-0.0069,-0.4625,"Plate control","PASS","PFC",0.725727578349768,"1h","1h"
861,"PFC_23",75,"OID30129","P05000","IFNW1",0.7273,"Cardiometabolic_II","B13805","20212097_SS211647","PASS",-0.0069,-0.3605,"Plate control","PASS","PFC",0.778894588583299,"24h","24h"
862,"PFC_08",85,"OID30129","P05000","IFNW1",0.7273,"Cardiometabolic_II","B13805","20212097_SS211647","PASS",-0.0069,1.974,"Plate control","PASS","PFC",3.92855838105357,"Baseline","Baseline"
863,"PFC_16",86,"OID30129","P05000","IFNW1",0.7273,"Cardiometabolic_II","B13805","20212097_SS211647","PASS",-0.0069,0.0919,"Plate control","PASS","PFC",1.06577285976233,"1h","1h"
864,"PFC_24",87,"OID30129","P05000","IFNW1",0.7273,"Cardiometabolic_II","B13805","20212097_SS211647","PASS",-0.0069,-0.4685,"Plate control","PASS","PFC",0.722715629713455,"24h","24h"
865,"PFC_01",1,"OID30130","Q15018","ABRAXAS2",0.1364,"Cardiometabolic_II","B13805","20212097_SS211647","WARN",1.4474,1.8156,"Plate control","PASS","PFC",3.52005995109003,"Baseline","Baseline"
866,"PFC_09",2,"OID30130","Q15018","ABRAXAS2",0.1364,"Cardiometabolic_II","B13805","20212097_SS211647","PASS",1.4474,1.7771,"Plate control","PASS","PFC",3.42736537770586,"Baseline","Baseline"
867,"PFC_17",3,"OID30130","Q15018","ABRAXAS2",0.1364,"Cardiometabolic_II","B13805","20212097_SS211647","PASS",1.4474,2.554,"Plate control","PASS","PFC",5.87260254413641,"1h","1h"
868,"PFC_25",4,"OID30130","Q15018","ABRAXAS2",0.1364,"Cardiometabolic_II","B13805","20212097_SS211647","PASS",1.4474,2.5106,"Plate control","PASS","PFC",5.69857025838014,"24h","24h"
869,"PFC_02",13,"OID30130","Q15018","ABRAXAS2",0.1364,"Cardiometabolic_II","B13805","20212097_SS211647","PASS",1.4474,1.6199,"Plate control","PASS","PFC",3.07353731382006,"Baseline","Baseline"
870,"PFC_10",14,"OID30130","Q15018","ABRAXAS2",0.1364,"Cardiometabolic_II","B13805","20212097_SS211647","PASS",1.4474,1.6521,"Plate control","PASS","PFC",3.14290790889598,"1h","1h"
871,"PFC_18",15,"OID30130","Q15018","ABRAXAS2",0.1364,"Cardiometabolic_II","B13805","20212097_SS211647","PASS",1.4474,2.2571,"Plate control","PASS","PFC",4.78029615282391,"1h","1h"
872,"PFC_26",16,"OID30130","Q15018","ABRAXAS2",0.1364,"Cardiometabolic_II","B13805","20212097_SS211647","PASS",1.4474,2.2908,"Plate control","PASS","PFC",4.89327376572265,"24h","24h"
873,"PFC_03",25,"OID30130","Q15018","ABRAXAS2",0.1364,"Cardiometabolic_II","B13805","20212097_SS211647","PASS",1.4474,1.4215,"Plate control","PASS","PFC",2.67863869853628,"Baseline","Baseline"
874,"PFC_11",26,"OID30130","Q15018","ABRAXAS2",0.1364,"Cardiometabolic_II","B13805","20212097_SS211647","PASS",1.4474,2.095,"Plate control","PASS","PFC",4.27226163219141,"1h","1h"
875,"PFC_19",27,"OID30130","Q15018","ABRAXAS2",0.1364,"Cardiometabolic_II","B13805","20212097_SS211647","PASS",1.4474,2.2101,"Plate control","PASS","PFC",4.62707344879748,"24h","24h"
876,"PFC_27",28,"OID30130","Q15018","ABRAXAS2",0.1364,"Cardiometabolic_II","B13805","20212097_SS211647","PASS",1.4474,3.3775,"Plate control","PASS","PFC",10.3927100365902,"24h","24h"
877,"PFC_04",37,"OID30130","Q15018","ABRAXAS2",0.1364,"Cardiometabolic_II","B13805","20212097_SS211647","PASS",1.4474,1.7724,"Plate control","PASS","PFC",3.41621790299359,"Baseline","Baseline"
878,"PFC_12",38,"OID30130","Q15018","ABRAXAS2",0.1364,"Cardiometabolic_II","B13805","20212097_SS211647","PASS",1.4474,1.619,"Plate control","PASS","PFC",3.0716205394042,"1h","1h"
879,"PFC_20",39,"OID30130","Q15018","ABRAXAS2",0.1364,"Cardiometabolic_II","B13805","20212097_SS211647","PASS",1.4474,2.5385,"Plate control","PASS","PFC",5.80984631003075,"24h","24h"
880,"PFC_05",49,"OID30130","Q15018","ABRAXAS2",0.1364,"Cardiometabolic_II","B13805","20212097_SS211647","PASS",1.4474,1.5564,"Plate control","PASS","PFC",2.94119003077117,"Baseline","Baseline"
881,"PFC_13",50,"OID30130","Q15018","ABRAXAS2",0.1364,"Cardiometabolic_II","B13805","20212097_SS211647","PASS",1.4474,1.9398,"Plate control","PASS","PFC",3.83652458519408,"1h","1h"
882,"PFC_21",51,"OID30130","Q15018","ABRAXAS2",0.1364,"Cardiometabolic_II","B13805","20212097_SS211647","PASS",1.4474,1.5394,"Plate control","PASS","PFC",2.90673590574814,"24h","24h"
883,"PFC_06",61,"OID30130","Q15018","ABRAXAS2",0.1364,"Cardiometabolic_II","B13805","20212097_SS211647","PASS",1.4474,1.893,"Plate control","PASS","PFC",3.71406740922692,"Baseline","Baseline"
884,"PFC_14",62,"OID30130","Q15018","ABRAXAS2",0.1364,"Cardiometabolic_II","B13805","20212097_SS211647","PASS",1.4474,2.543,"Plate control","PASS","PFC",5.82799645559204,"1h","1h"
885,"PFC_22",63,"OID30130","Q15018","ABRAXAS2",0.1364,"Cardiometabolic_II","B13805","20212097_SS211647","PASS",1.4474,2.2184,"Plate control","PASS","PFC",4.6537702864357,"24h","24h"
886,"PFC_07",73,"OID30130","Q15018","ABRAXAS2",0.1364,"Cardiometabolic_II","B13805","20212097_SS211647","PASS",1.4474,2.103,"Plate control","PASS","PFC",4.2960178864805,"Baseline","Baseline"
887,"PFC_15",74,"OID30130","Q15018","ABRAXAS2",0.1364,"Cardiometabolic_II","B13805","20212097_SS211647","PASS",1.4474,1.1069,"Plate control","PASS","PFC",2.15382345554636,"1h","1h"
888,"PFC_23",75,"OID30130","Q15018","ABRAXAS2",0.1364,"Cardiometabolic_II","B13805","20212097_SS211647","PASS",1.4474,2.7499,"Plate control","PASS","PFC",6.72670504620629,"24h","24h"
889,"PFC_08",85,"OID30130","Q15018","ABRAXAS2",0.1364,"Cardiometabolic_II","B13805","20212097_SS211647","PASS",1.4474,1.6638,"Plate control","PASS","PFC",3.16849996588857,"Baseline","Baseline"
890,"PFC_16",86,"OID30130","Q15018","ABRAXAS2",0.1364,"Cardiometabolic_II","B13805","20212097_SS211647","PASS",1.4474,2.0526,"Plate control","PASS","PFC",4.14852937021926,"1h","1h"
891,"PFC_24",87,"OID30130","Q15018","ABRAXAS2",0.1364,"Cardiometabolic_II","B13805","20212097_SS211647","PASS",1.4474,2.3803,"Plate control","PASS","PFC",5.20644996004324,"24h","24h"
892,"PFC_01",1,"OID30131","P54296","MYOM2",0.6818,"Cardiometabolic_II","B13805","20212097_SS211647","WARN",-0.0486,-0.1372,"Plate control","PASS","PFC",0.909282189785745,"Baseline","Baseline"
893,"PFC_09",2,"OID30131","P54296","MYOM2",0.6818,"Cardiometabolic_II","B13805","20212097_SS211647","PASS",-0.0486,-1.5438,"Plate control","PASS","PFC",0.342980866096142,"Baseline","Baseline"
894,"PFC_17",3,"OID30131","P54296","MYOM2",0.6818,"Cardiometabolic_II","B13805","20212097_SS211647","PASS",-0.0486,-0.8824,"Plate control","PASS","PFC",0.542464262059169,"1h","1h"
895,"PFC_25",4,"OID30131","P54296","MYOM2",0.6818,"Cardiometabolic_II","B13805","20212097_SS211647","PASS",-0.0486,-1.1985,"Plate control","PASS","PFC",0.435728081751471,"24h","24h"
896,"PFC_02",13,"OID30131","P54296","MYOM2",0.6818,"Cardiometabolic_II","B13805","20212097_SS211647","PASS",-0.0486,-0.0072,"Plate control","PASS","PFC",0.995021772951113,"Baseline","Baseline"

Supplemental table 1_Olink proximity extension assay (PEA) data

,"SampleID","Index","OlinkID","UniProt","Assay","MissingFreq","Panel","Panel_Lot_Nr","PlateID","QC_Warning","LOD","NPX","Normalization","Assay_Warning","condition","Value","group","alt_group"
897,"PFC_10",14,"OID30131","P54296","MYOM2",0.6818,"Cardiometabolic_II","B13805","20212097_SS211647","PASS",-0.0486,-0.383,"Plate control","PASS","PFC",0.766841329836546,"1h","1h"
898,"PFC_18",15,"OID30131","P54296","MYOM2",0.6818,"Cardiometabolic_II","B13805","20212097_SS211647","PASS",-0.0486,-0.6381,"Plate control","PASS","PFC",0.642558628420891,"1h","1h"
899,"PFC_26",16,"OID30131","P54296","MYOM2",0.6818,"Cardiometabolic_II","B13805","20212097_SS211647","PASS",-0.0486,-1.0141,"Plate control","PASS","PFC",0.495137114487544,"24h","24h"
900,"PFC_03",25,"OID30131","P54296","MYOM2",0.6818,"Cardiometabolic_II","B13805","20212097_SS211647","PASS",-0.0486,-0.755,"Plate control","PASS","PFC",0.592546385470791,"Baseline","Baseline"
901,"PFC_11",26,"OID30131","P54296","MYOM2",0.6818,"Cardiometabolic_II","B13805","20212097_SS211647","PASS",-0.0486,0.0517,"Plate control","PASS","PFC",1.03648554749847,"1h","1h"
902,"PFC_19",27,"OID30131","P54296","MYOM2",0.6818,"Cardiometabolic_II","B13805","20212097_SS211647","PASS",-0.0486,-0.3842,"Plate control","PASS","PFC",0.766203754346641,"24h","24h"
903,"PFC_27",28,"OID30131","P54296","MYOM2",0.6818,"Cardiometabolic_II","B13805","20212097_SS211647","PASS",-0.0486,-1.3887,"Plate control","PASS","PFC",0.381908781932361,"24h","24h"
904,"PFC_04",37,"OID30131","P54296","MYOM2",0.6818,"Cardiometabolic_II","B13805","20212097_SS211647","PASS",-0.0486,-1.0603,"Plate control","PASS","PFC",0.479532333348785,"Baseline","Baseline"
905,"PFC_12",38,"OID30131","P54296","MYOM2",0.6818,"Cardiometabolic_II","B13805","20212097_SS211647","PASS",-0.0486,-0.0503,"Plate control","PASS","PFC",0.965735488994416,"1h","1h"
906,"PFC_20",39,"OID30131","P54296","MYOM2",0.6818,"Cardiometabolic_II","B13805","20212097_SS211647","PASS",-0.0486,0.0529,"Plate control","PASS","PFC",1.0373480305873,"24h","24h"
907,"PFC_05",49,"OID30131","P54296","MYOM2",0.6818,"Cardiometabolic_II","B13805","20212097_SS211647","PASS",-0.0486,-0.5635,"Plate control","PASS","PFC",0.676658586887851,"Baseline","Baseline"
908,"PFC_13",50,"OID30131","P54296","MYOM2",0.6818,"Cardiometabolic_II","B13805","20212097_SS211647","PASS",-0.0486,-1.3591,"Plate control","PASS","PFC",0.389825399731503,"1h","1h"
909,"PFC_21",51,"OID30131","P54296","MYOM2",0.6818,"Cardiometabolic_II","B13805","20212097_SS211647","PASS",-0.0486,0.7823,"Plate control","PASS","PFC",1.71987057233577,"24h","24h"
910,"PFC_06",61,"OID30131","P54296","MYOM2",0.6818,"Cardiometabolic_II","B13805","20212097_SS211647","PASS",-0.0486,-0.0947,"Plate control","PASS","PFC",0.936466960019026,"Baseline","Baseline"
911,"PFC_14",62,"OID30131","P54296","MYOM2",0.6818,"Cardiometabolic_II","B13805","20212097_SS211647","PASS",-0.0486,-0.6509,"Plate control","PASS","PFC",0.636882881490689,"1h","1h"
912,"PFC_22",63,"OID30131","P54296","MYOM2",0.6818,"Cardiometabolic_II","B13805","20212097_SS211647","PASS",-0.0486,-0.3922,"Plate control","PASS","PFC",0.761966776823155,"24h","24h"
913,"PFC_07",73,"OID30131","P54296","MYOM2",0.6818,"Cardiometabolic_II","B13805","20212097_SS211647","PASS",-0.0486,-0.747,"Plate control","PASS","PFC",0.595841287287023,"Baseline","Baseline"
914,"PFC_15",74,"OID30131","P54296","MYOM2",0.6818,"Cardiometabolic_II","B13805","20212097_SS211647","PASS",-0.0486,-0.1259,"Plate control","PASS","PFC",0.916432164711004,"1h","1h"
915,"PFC_23",75,"OID30131","P54296","MYOM2",0.6818,"Cardiometabolic_II","B13805","20212097_SS211647","PASS",-0.0486,-1.0275,"Plate control","PASS","PFC",0.490559487499159,"24h","24h"
916,"PFC_08",85,"OID30131","P54296","MYOM2",0.6818,"Cardiometabolic_II","B13805","20212097_SS211647","PASS",-0.0486,-1.3494,"Plate control","PASS","PFC",0.392455232555711,"Baseline","Baseline"
917,"PFC_16",86,"OID30131","P54296","MYOM2",0.6818,"Cardiometabolic_II","B13805","20212097_SS211647","PASS",-0.0486,-0.5975,"Plate control","PASS","PFC",0.660898213008892,"1h","1h"
918,"PFC_24",87,"OID30131","P54296","MYOM2",0.6818,"Cardiometabolic_II","B13805","20212097_SS211647","PASS",-0.0486,-1.0116,"Plate control","PASS","PFC",0.495995865561005,"24h","24h"
919,"PFC_01",1,"OID30132","Q16836","HADH",0.9318,"Cardiometabolic_II","B13805","20212097_SS211647","WARN",-0.0479,-0.8343,"Plate control","PASS","PFC",0.560855101598785,"Baseline","Baseline"
920,"PFC_09",2,"OID30132","Q16836","HADH",0.9318,"Cardiometabolic_II","B13805","20212097_SS211647","PASS",-0.0479,-0.6912,"Plate control","PASS","PFC",0.619338484395564,"Baseline","Baseline"
921,"PFC_17",3,"OID30132","Q16836","HADH",0.9318,"Cardiometabolic_II","B13805","20212097_SS211647","PASS",-0.0479,0.1695,"Plate control","PASS","PFC",1.12466863668991,"1h","1h"
922,"PFC_25",4,"OID30132","Q16836","HADH",0.9318,"Cardiometabolic_II","B13805","20212097_SS211647","PASS",-0.0479,-1.0417,"Plate control","PASS","PFC",0.485754747129365,"24h","24h"
923,"PFC_02",13,"OID30132","Q16836","HADH",0.9318,"Cardiometabolic_II","B13805","20212097_SS211647","PASS",-0.0479,-1.2457,"Plate control","PASS","PFC",0.421703236731174,"Baseline","Baseline"
924,"PFC_10",14,"OID30132","Q16836","HADH",0.9318,"Cardiometabolic_II","B13805","20212097_SS211647","PASS",-0.0479,-0.9084,"Plate control","PASS","PFC",0.532775630783646,"1h","1h"
925,"PFC_18",15,"OID30132","Q16836","HADH",0.9318,"Cardiometabolic_II","B13805","20212097_SS211647","PASS",-0.0479,-1.2682,"Plate control","PASS","PFC",0.415177452239707,"1h","1h"
926,"PFC_26",16,"OID30132","Q16836","HADH",0.9318,"Cardiometabolic_II","B13805","20212097_SS211647","PASS",-0.0479,0.0709,"Plate control","PASS","PFC",1.05037173526674,"24h","24h"
927,"PFC_03",25,"OID30132","Q16836","HADH",0.9318,"Cardiometabolic_II","B13805","20212097_SS211647","PASS",-0.0479,-0.8919,"Plate control","PASS","PFC",0.53890392520091,"Baseline","Baseline"
928,"PFC_11",26,"OID30132","Q16836","HADH",0.9318,"Cardiometabolic_II","B13805","20212097_SS211647","PASS",-0.0479,-0.2284,"Plate control","PASS","PFC",0.853581018669179,"1h","1h"
929,"PFC_19",27,"OID30132","Q16836","HADH",0.9318,"Cardiometabolic_II","B13805","20212097_SS211647","PASS",-0.0479,-1.0454,"Plate control","PASS","PFC",0.484510554992989,"24h","24h"
930,"PFC_27",28,"OID30132","Q16836","HADH",0.9318,"Cardiometabolic_II","B13805","20212097_SS211647","PASS",-0.0479,-0.9983,"Plate control","PASS","PFC",0.500589522367165,"24h","24h"
931,"PFC_04",37,"OID30132","Q16836","HADH",0.9318,"Cardiometabolic_II","B13805","20212097_SS211647","PASS",-0.0479,-1.4431,"Plate control","PASS","PFC",0.367776193277273,"Baseline","Baseline"
932,"PFC_12",38,"OID30132","Q16836","HADH",0.9318,"Cardiometabolic_II","B13805","20212097_SS211647","PASS",-0.0479,-0.5728,"Plate control","PASS","PFC",0.672310692668554,"1h","1h"
933,"PFC_20",39,"OID30132","Q16836","HADH",0.9318,"Cardiometabolic_II","B13805","20212097_SS211647","PASS",-0.0479,-0.8818,"Plate control","PASS","PFC",0.5426899135231,"24h","24h"
934,"PFC_05",49,"OID30132","Q16836","HADH",0.9318,"Cardiometabolic_II","B13805","20212097_SS211647","PASS",-0.0479,-0.565,"Plate control","PASS","PFC",0.675955416514063,"Baseline","Baseline"
935,"PFC_13",50,"OID30132","Q16836","HADH",0.9318,"Cardiometabolic_II","B13805","20212097_SS211647","PASS",-0.0479,-0.3191,"Plate control","PASS","PFC",0.801569766891372,"1h","1h"
936,"PFC_21",51,"OID30132","Q16836","HADH",0.9318,"Cardiometabolic_II","B13805","20212097_SS211647","PASS",-0.0479,-0.3827,"Plate control","PASS","PFC",0.767000806588815,"24h","24h"
937,"PFC_06",61,"OID30132","Q16836","HADH",0.9318,"Cardiometabolic_II","B13805","20212097_SS211647","PASS",-0.0479,-1.0967,"Plate control","PASS","PFC",0.467584820297023,"Baseline","Baseline"
938,"PFC_14",62,"OID30132","Q16836","HADH",0.9318,"Cardiometabolic_II","B13805","20212097_SS211647","PASS",-0.0479,-1.0475,"Plate control","PASS","PFC",0.483805810071172,"1h","1h"
939,"PFC_22",63,"OID30132","Q16836","HADH",0.9318,"Cardiometabolic_II","B13805","20212097_SS211647","PASS",-0.0479,-1.837,"Plate control","PASS","PFC",0.27990322216356,"24h","24h"
940,"PFC_07",73,"OID30132","Q16836","HADH",0.9318,"Cardiometabolic_II","B13805","20212097_SS211647","PASS",-0.0479,-0.6101,"Plate control","PASS","PFC",0.655151288728436,"Baseline","Baseline"
941,"PFC_15",74,"OID30132","Q16836","HADH",0.9318,"Cardiometabolic_II","B13805","20212097_SS211647","PASS",-0.0479,-1.0182,"Plate control","PASS","PFC",0.493731980192162,"1h","1h"
942,"PFC_23",75,"OID30132","Q16836","HADH",0.9318,"Cardiometabolic_II","B13805","20212097_SS211647","PASS",-0.0479,-0.7417,"Plate control","PASS","PFC",0.59803424318409,"24h","24h"
943,"PFC_08",85,"OID30132","Q16836","HADH",0.9318,"Cardiometabolic_II","B13805","20212097_SS211647","PASS",-0.0479,-0.1099,"Plate control","PASS","PFC",0.926652290306596,"Baseline","Baseline"
944,"PFC_16",86,"OID30132","Q16836","HADH",0.9318,"Cardiometabolic_II","B13805","20212097_SS211647","PASS",-0.0479,-0.4184,"Plate control","PASS","PFC",0.748254004572171,"1h","1h"
945,"PFC_24",87,"OID30132","Q16836","HADH",0.9318,"Cardiometabolic_II","B13805","20212097_SS211647","PASS",-0.0479,-0.3991,"Plate control","PASS","PFC",0.758331207351636,"24h","24h"
946,"PFC_01",1,"OID30133","Q14353","GAMT",0.9773,"Cardiometabolic_II","B13805","20212097_SS211647","WARN",0.9971,-0.078,"Plate control","PASS","PFC",0.947370070599268,"Baseline","Baseline"
947,"PFC_09",2,"OID30133","Q14353","GAMT",0.9773,"Cardiometabolic_II","B13805","20212097_SS211647","PASS",0.9971,0.7076,"Plate control","PASS","PFC",1.63308513210262,"Baseline","Baseline"
948,"PFC_17",3,"OID30133","Q14353","GAMT",0.9773,"Cardiometabolic_II","B13805","20212097_SS211647","PASS",0.9971,-0.3559,"Plate control","PASS","PFC",0.781382039571041,"1h","1h"
949,"PFC_25",4,"OID30133","Q14353","GAMT",0.9773,"Cardiometabolic_II","B13805","20212097_SS211647","PASS",0.9971,-0.347,"Plate control","PASS","PFC",0.786217292140332,"24h","24h"
950,"PFC_02",13,"OID30133","Q14353","GAMT",0.9773,"Cardiometabolic_II","B13805","20212097_SS211647","PASS",0.9971,-0.2429,"Plate control","PASS","PFC",0.845044956435438,"Baseline","Baseline"
951,"PFC_10",14,"OID30133","Q14353","GAMT",0.9773,"Cardiometabolic_II","B13805","20212097_SS211647","PASS",0.9971,-0.35,"Plate control","PASS","PFC",0.784584097896751,"1h","1h"
952,"PFC_18",15,"OID30133","Q14353","GAMT",0.9773,"Cardiometabolic_II","B13805","20212097_SS211647","PASS",0.9971,0.7304,"Plate control","PASS","PFC",1.6590990277906,"1h","1h"
953,"PFC_26",16,"OID30133","Q14353","GAMT",0.9773,"Cardiometabolic_II","B13805","20212097_SS211647","PASS",0.9971,-0.1128,"Plate control","PASS","PFC",0.924791472548094,"24h","24h"
954,"PFC_03",25,"OID30133","Q14353","GAMT",0.9773,"Cardiometabolic_II","B13805","20212097_SS211647","PASS",0.9971,-0.1312,"Plate control","PASS","PFC",0.913071662628106,"Baseline","Baseline"
955,"PFC_11",26,"OID30133","Q14353","GAMT",0.9773,"Cardiometabolic_II","B13805","20212097_SS211647","PASS",0.9971,-0.8115,"Plate control","PASS","PFC",0.569789128259281,"1h","1h"
956,"PFC_19",27,"OID30133","Q14353","GAMT",0.9773,"Cardiometabolic_II","B13805","20212097_SS211647","PASS",0.9971,-0.4009,"Plate control","PASS","PFC",0.757385654092162,"24h","24h"
957,"PFC_27",28,"OID30133","Q14353","GAMT",0.9773,"Cardiometabolic_II","B13805","20212097_SS211647","PASS",0.9971,0.3686,"Plate control","PASS","PFC",1.29109933192804,"24h","24h"
958,"PFC_04",37,"OID30133","Q14353","GAMT",0.9773,"Cardiometabolic_II","B13805","20212097_SS211647","PASS",0.9971,0.242,"Plate control","PASS","PFC",1.18263100024414,"Baseline","Baseline"
959,"PFC_12",38,"OID30133","Q14353","GAMT",0.9773,"Cardiometabolic_II","B13805","20212097_SS211647","PASS",0.9971,-0.0013,"Plate control","PASS","PFC",0.999099314526154,"1h","1h"
960,"PFC_20",39,"OID30133","Q14353","GAMT",0.9773,"Cardiometabolic_II","B13805","20212097_SS211647","PASS",0.9971,-0.1029,"Plate control","PASS","PFC",0.931159360641937,"24h","24h"
961,"PFC_05",49,"OID30133","Q14353","GAMT",0.9773,"Cardiometabolic_II","B13805","20212097_SS211647","PASS",0.9971,-0.0614,"Plate control","PASS","PFC",0.9583336951353,"Baseline","Baseline"
962,"PFC_13",50,"OID30133","Q14353","GAMT",0.9773,"Cardiometabolic_II","B13805","20212097_SS211647","PASS",0.9971,0.5767,"Plate control","PASS","PFC",1.49143385949145,"1h","1h"
963,"PFC_21",51,"OID30133","Q14353","GAMT",0.9773,"Cardiometabolic_II","B13805","20212097_SS211647","PASS",0.9971,-0.3257,"Plate control","PASS","PFC",0.797911143516922,"24h","24h"
964,"PFC_06",61,"OID30133","Q14353","GAMT",0.9773,"Cardiometabolic_II","B13805","20212097_SS211647","PASS",0.9971,-0.6365,"Plate control","PASS","PFC",0.643271644050243,"Baseline","Baseline"
965,"PFC_14",62,"OID30133","Q14353","GAMT",0.9773,"Cardiometabolic_II","B13805","20212097_SS211647","PASS",0.9971,-0.5195,"Plate control","PASS","PFC",0.697613565721825,"1h","1h"
966,"PFC_22",63,"OID30133","Q14353","GAMT",0.9773,"Cardiometabolic_II","B13805","20212097_SS211647","PASS",0.9971,-0.0813,"Plate control","PASS","PFC",0.945205546345769,"24h","24h"
967,"PFC_07",73,"OID30133","Q14353","GAMT",0.9773,"Cardiometabolic_II","B13805","20212097_SS211647","PASS",0.9971,0.1309,"Plate control","PASS","PFC",1.09497657017085,"Baseline","Baseline"
968,"PFC_15",74,"OID30133","Q14353","GAMT",0.9773,"Cardiometabolic_II","B13805","20212097_SS211647","PASS",0.9971,-0.2911,"Plate control","PASS","PFC",0.817278677083842,"1h","1h"
969,"PFC_23",75,"OID30133","Q14353","GAMT",0.9773,"Cardiometabolic_II","B13805","20212097_SS211647","PASS",0.9971,0.1714,"Plate control","PASS","PFC",1.12615077815046,"24h","24h"
970,"PFC_08",85,"OID30133","Q14353","GAMT",0.9773,"Cardiometabolic_II","B13805","20212097_SS211647","PASS",0.9971,-0.7124,"Plate control","PASS","PFC",0.610304020670122,"Baseline","Baseline"
971,"PFC_16",86,"OID30133","Q14353","GAMT",0.9773,"Cardiometabolic_II","B13805","20212097_SS211647","PASS",0.9971,-0.2616,"Plate control","PASS","PFC",0.834162290661825,"1h","1h"
972,"PFC_24",87,"OID30133","Q14353","GAMT",0.9773,"Cardiometabolic_II","B13805","20212097_SS211647","PASS",0.9971,-0.7323,"Plate control","PASS","PFC",0.601943506967142,"24h","24h"
973,"PFC_01",1,"OID30134","P23511","NFYA",0.7273,"Cardiometabolic_II","B13805","20212097_SS211647","WARN",-0.2691,-1.0477,"Plate control","PASS","PFC",0.483738744993239,"Baseline","Baseline"
974,"PFC_09",2,"OID30134","P23511","NFYA",0.7273,"Cardiometabolic_II","B13805","20212097_SS211647","PASS",-0.2691,-0.4182,"Plate control","PASS","PFC",0.748357741793244,"Baseline","Baseline"
975,"PFC_17",3,"OID30134","P23511","NFYA",0.7273,"Cardiometabolic_II","B13805","20212097_SS211647","PASS",-0.2691,-0.793,"Plate control","PASS","PFC",0.577142708758419,"1h","1h"
976,"PFC_25",4,"OID30134","P23511","NFYA",0.7273,"Cardiometabolic_II","B13805","20212097_SS211647","PASS",-0.2691,-0.7058,"Plate control","PASS","PFC",0.613102418178023,"24h","24h"
977,"PFC_02",13,"OID30134","P23511","NFYA",0.7273,"Cardiometabolic_II","B13805","20212097_SS211647","PASS",-0.2691,-0.3478,"Plate control","PASS","PFC",0.785781441555458,"Baseline","Baseline"
978,"PFC_10",14,"OID30134","P23511","NFYA",0.7273,"Cardiometabolic_II","B13805","20212097_SS211647","PASS",-0.2691,-0.7585,"Plate control","PASS","PFC",0.591110601294518,"1h","1h"
979,"PFC_18",15,"OID30134","P23511","NFYA",0.7273,"Cardiometabolic_II","B13805","20212097_SS211647","PASS",-0.2691,-0.4832,"Plate control","PASS","PFC",0.715389079152278,"1h","1h"
980,"PFC_26",16,"OID30134","P23511","NFYA",0.7273,"Cardiometabolic_II","B13805","20212097_SS211647","PASS",-0.2691,-0.6052,"Plate control","PASS","PFC",0.657380241533242,"24h","24h"
981,"PFC_03",25,"OID30134","P23511","NFYA",0.7273,"Cardiometabolic_II","B13805","20212097_SS211647","PASS",-0.2691,-0.7367,"Plate control","PASS","PFC",0.600110467676496,"Baseline","Baseline"
982,"PFC_11",26,"OID30134","P23511","NFYA",0.7273,"Cardiometabolic_II","B13805","20212097_SS211647","PASS",-0.2691,-0.2669,"Plate control","PASS","PFC",0.831103467299674,"1h","1h"
983,"PFC_19",27,"OID30134","P23511","NFYA",0.7273,"Cardiometabolic_II","B13805","20212097_SS211647","PASS",-0.2691,-0.2136,"Plate control","PASS","PFC",0.862382615105203,"24h","24h"
984,"PFC_27",28,"OID30134","P23511","NFYA",0.7273,"Cardiometabolic_II","B13805","20212097_SS211647","PASS",-0.2691,-0.1985,"Plate control","PASS","PFC",0.871456163502941,"24h","24h"
985,"PFC_04",37,"OID30134","P23511","NFYA",0.7273,"Cardiometabolic_II","B13805","20212097_SS211647","PASS",-0.2691,-0.5972,"Plate control","PASS","PFC",0.661035657218652,"Baseline","Baseline"
986,"PFC_12",38,"OID30134","P23511","NFYA",0.7273,"Cardiometabolic_II","B13805","20212097_SS211647","PASS",-0.2691,-0.3163,"Plate control","PASS","PFC",0.803126973832652,"1h","1h"
987,"PFC_20",39,"OID30134","P23511","NFYA",0.7273,"Cardiometabolic_II","B13805","20212097_SS211647","PASS",-0.2691,-0.2364,"Plate control","PASS","PFC",0.848860859612192,"24h","24h"
988,"PFC_05",49,"OID30134","P23511","NFYA",0.7273,"Cardiometabolic_II","B13805","20212097_SS211647","PASS",-0.2691,-0.7644,"Plate control","PASS","PFC",0.5886981503828,"Baseline","Baseline"
989,"PFC_13",50,"OID30134","P23511","NFYA",0.7273,"Cardiometabolic_II","B13805","20212097_SS211647","PASS",-0.2691,-0.7689,"Plate control","PASS","PFC",0.586864766096703,"1h","1h"
990,"PFC_21",51,"OID30134","P23511","NFYA",0.7273,"Cardiometabolic_II","B13805","20212097_SS211647","PASS",-0.2691,-0.7529,"Plate control","PASS","PFC",0.593409529416995,"24h","24h"
991,"PFC_06",61,"OID30134","P23511","NFYA",0.7273,"Cardiometabolic_II","B13805","20212097_SS211647","PASS",-0.2691,-0.8552,"Plate control","PASS","PFC",0.552788688563924,"Baseline","Baseline"
992,"PFC_14",62,"OID30134","P23511","NFYA",0.7273,"Cardiometabolic_II","B13805","20212097_SS211647","PASS",-0.2691,-0.7002,"Plate control","PASS","PFC",0.615486876159358,"1h","1h"
993,"PFC_22",63,"OID30134","P23511","NFYA",0.7273,"Cardiometabolic_II","B13805","20212097_SS211647","PASS",-0.2691,-0.677,"Plate control","PASS","PFC",0.625464540056633,"24h","24h"
994,"PFC_07",73,"OID30134","P23511","NFYA",0.7273,"Cardiometabolic_II","B13805","20212097_SS211647","PASS",-0.2691,-0.6064,"Plate control","PASS","PFC",0.656833675362065,"Baseline","Baseline"
995,"PFC_15",74,"OID30134","P23511","NFYA",0.7273,"Cardiometabolic_II","B13805","20212097_SS211647","PASS",-0.2691,-0.8375,"Plate control","PASS","PFC",0.55961246381405,"1h","1h"
996,"PFC_23",75,"OID30134","P23511","NFYA",0.7273,"Cardiometabolic_II","B13805","20212097_SS211647","PASS",-0.2691,-0.6294,"Plate control","PASS","PFC",0.646445208425081,"24h","24h"
997,"PFC_08",85,"OID30134","P23511","NFYA",0.7273,"Cardiometabolic_II","B13805","20212097_SS211647","PASS",-0.2691,-0.6141,"Plate control","PASS","PFC",0.653337339483579,"Baseline","Baseline"
998,"PFC_16",86,"OID30134","P23511","NFYA",0.7273,"Cardiometabolic_II","B13805","20212097_SS211647","PASS",-0.2691,-0.4464,"Plate control","PASS","PFC",0.733871817334739,"1h","1h"
999,"PFC_24",87,"OID30134","P23511","NFYA",0.7273,"Cardiometabolic_II","B13805","20212097_SS211647","PASS",-0.2691,-0.5654,"Plate control","PASS","PFC",0.675768027856391,"24h","24h"
1000,"PFC_01",1,"OID30135","Q07075","ENPEP",0.8409,"Cardiometabolic_II","B13805","20212097_SS211647","WARN",0.56,0.4919,"Plate control","PASS","PFC",1.40629571954344,"Baseline","Baseline"
1001,"PFC_09",2,"OID30135","Q07075","ENPEP",0.8409,"Cardiometabolic_II","B13805","20212097_SS211647","PASS",0.56,0.6643,"Plate control","PASS","PFC",1.58479913730448,"Baseline","Baseline"
1002,"PFC_17",3,"OID30135","Q07075","ENPEP",0.8409,"Cardiometabolic_II","B13805","20212097_SS211647","PASS",0.56,-0.2543,"Plate control","PASS","PFC",0.838393827218016,"1h","1h"
1003,"PFC_25",4,"OID30135","Q07075","ENPEP",0.8409,"Cardiometabolic_II","B13805","20212097_SS211647","PASS",0.56,1.0645,"Plate control","PASS","PFC",2.09144491439257,"24h","24h"
1004,"PFC_02",13,"OID30135","Q07075","ENPEP",0.8409,"Cardiometabolic_II","B13805","20212097_SS211647","PASS",0.56,0.3358,"Plate control","PASS","PFC",1.26207705895317,"Baseline","Baseline"
1005,"PFC_10",14,"OID30135","Q07075","ENPEP",0.8409,"Cardiometabolic_II","B13805","20212097_SS211647","PASS",0.56,-0.4622,"Plate control","PASS","PFC",0.725878504848802,"1h","1h"
1006,"PFC_18",15,"OID30135","Q07075","ENPEP",0.8409,"Cardiometabolic_II","B13805","20212097_SS211647","PASS",0.56,-0.3323,"Plate control","PASS","PFC",0.794269219281522,"1h","1h"
1007,"PFC_26",16,"OID30135","Q07075","ENPEP",0.8409,"Cardiometabolic_II","B13805","20212097_SS211647","PASS",0.56,-0.0873,"Plate control","PASS","PFC",0.941282709951947,"24h","24h"
1008,"PFC_03",25,"OID30135","Q07075","ENPEP",0.8409,"Cardiometabolic_II","B13805","20212097_SS211647","PASS",0.56,0.3525,"Plate control","PASS","PFC",1.2767711873336,"Baseline","Baseline"

Supplemental table 1_Olink proximity extension assay (PEA) data

,"SampleID","Index","OlinkID","UniProt","Assay","MissingFreq","Panel","Panel_Lot_Nr","PlateID","QC_Warning","LOD","NPX","Normalization","Assay_Warning","condition","Value","group","alt_group"
1009,"PFC_11",26,"OID30135","Q07075","ENPEP",0.8409,"Cardiometabolic_II","B13805","20212097_SS211647","PASS",0.56,0.2996,"Plate control","PASS","PFC",1.23080311494952,"1h","1h"
1010,"PFC_19",27,"OID30135","Q07075","ENPEP",0.8409,"Cardiometabolic_II","B13805","20212097_SS211647","PASS",0.56,0.1206,"Plate control","PASS","PFC",1.08718691684518,"24h","24h"
1011,"PFC_27",28,"OID30135","Q07075","ENPEP",0.8409,"Cardiometabolic_II","B13805","20212097_SS211647","PASS",0.56,-1.1095,"Plate control","PASS","PFC",0.463454624247937,"24h","24h"
1012,"PFC_04",37,"OID30135","Q07075","ENPEP",0.8409,"Cardiometabolic_II","B13805","20212097_SS211647","PASS",0.56,0.2678,"Plate control","PASS","PFC",1.20397046391535,"Baseline","Baseline"
1013,"PFC_12",38,"OID30135","Q07075","ENPEP",0.8409,"Cardiometabolic_II","B13805","20212097_SS211647","PASS",0.56,-0.9365,"Plate control","PASS","PFC",0.522498934387825,"1h","1h"
1014,"PFC_20",39,"OID30135","Q07075","ENPEP",0.8409,"Cardiometabolic_II","B13805","20212097_SS211647","PASS",0.56,0.4385,"Plate control","PASS","PFC",1.35519457075427,"24h","24h"
1015,"PFC_05",49,"OID30135","Q07075","ENPEP",0.8409,"Cardiometabolic_II","B13805","20212097_SS211647","PASS",0.56,0.4854,"Plate control","PASS","PFC",1.39997396699366,"Baseline","Baseline"
1016,"PFC_13",50,"OID30135","Q07075","ENPEP",0.8409,"Cardiometabolic_II","B13805","20212097_SS211647","PASS",0.56,0.0695,"Plate control","PASS","PFC",1.04935294257841,"1h","1h"
1017,"PFC_21",51,"OID30135","Q07075","ENPEP",0.8409,"Cardiometabolic_II","B13805","20212097_SS211647","PASS",0.56,0.1684,"Plate control","PASS","PFC",1.12381144653481,"24h","24h"
1018,"PFC_06",61,"OID30135","Q07075","ENPEP",0.8409,"Cardiometabolic_II","B13805","20212097_SS211647","PASS",0.56,0.1742,"Plate control","PASS","PFC",1.12833854755133,"Baseline","Baseline"
1019,"PFC_14",62,"OID30135","Q07075","ENPEP",0.8409,"Cardiometabolic_II","B13805","20212097_SS211647","PASS",0.56,0.1293,"Plate control","PASS","PFC",1.09376287543469,"1h","1h"
1020,"PFC_22",63,"OID30135","Q07075","ENPEP",0.8409,"Cardiometabolic_II","B13805","20212097_SS211647","PASS",0.56,0.1234,"Plate control","PASS","PFC",1.08929899128125,"24h","24h"
1021,"PFC_07",73,"OID30135","Q07075","ENPEP",0.8409,"Cardiometabolic_II","B13805","20212097_SS211647","PASS",0.56,0.8624,"Plate control","PASS","PFC",1.81806023635486,"Baseline","Baseline"
1022,"PFC_15",74,"OID30135","Q07075","ENPEP",0.8409,"Cardiometabolic_II","B13805","20212097_SS211647","PASS",0.56,0.1027,"Plate control","PASS","PFC",1.07378116188748,"1h","1h"
1023,"PFC_23",75,"OID30135","Q07075","ENPEP",0.8409,"Cardiometabolic_II","B13805","20212097_SS211647","PASS",0.56,0.6085,"Plate control","PASS","PFC",1.5246731502313,"24h","24h"
1024,"PFC_08",85,"OID30135","Q07075","ENPEP",0.8409,"Cardiometabolic_II","B13805","20212097_SS211647","PASS",0.56,0.7369,"Plate control","PASS","PFC",1.66659089103702,"Baseline","Baseline"
1025,"PFC_16",86,"OID30135","Q07075","ENPEP",0.8409,"Cardiometabolic_II","B13805","20212097_SS211647","PASS",0.56,0.7173,"Plate control","PASS","PFC",1.64410222049165,"1h","1h"
1026,"PFC_24",87,"OID30135","Q07075","ENPEP",0.8409,"Cardiometabolic_II","B13805","20212097_SS211647","PASS",0.56,0.3933,"Plate control","PASS","PFC",1.31339420967805,"24h","24h"
1027,"PFC_01",1,"OID30136","Q9BV94","EDEM2",0.8636,"Cardiometabolic_II","B13805","20212097_SS211647","WARN",0.3676,-0.5032,"Plate control","PASS","PFC",0.705540106297365,"Baseline","Baseline"
1028,"PFC_09",2,"OID30136","Q9BV94","EDEM2",0.8636,"Cardiometabolic_II","B13805","20212097_SS211647","PASS",0.3676,-0.3531,"Plate control","PASS","PFC",0.782900027881067,"Baseline","Baseline"
1029,"PFC_17",3,"OID30136","Q9BV94","EDEM2",0.8636,"Cardiometabolic_II","B13805","20212097_SS211647","PASS",0.3676,-0.0595,"Plate control","PASS","PFC",0.959596632551949,"1h","1h"
1030,"PFC_25",4,"OID30136","Q9BV94","EDEM2",0.8636,"Cardiometabolic_II","B13805","20212097_SS211647","PASS",0.3676,0.4573,"Plate control","PASS","PFC",1.37296990263793,"24h","24h"
1031,"PFC_02",13,"OID30136","Q9BV94","EDEM2",0.8636,"Cardiometabolic_II","B13805","20212097_SS211647","PASS",0.3676,-0.4283,"Plate control","PASS","PFC",0.743136945163993,"Baseline","Baseline"
1032,"PFC_10",14,"OID30136","Q9BV94","EDEM2",0.8636,"Cardiometabolic_II","B13805","20212097_SS211647","PASS",0.3676,-0.1837,"Plate control","PASS","PFC",0.880442077312034,"1h","1h"
1033,"PFC_18",15,"OID30136","Q9BV94","EDEM2",0.8636,"Cardiometabolic_II","B13805","20212097_SS211647","PASS",0.3676,-0.3191,"Plate control","PASS","PFC",0.801569766891372,"1h","1h"
1034,"PFC_26",16,"OID30136","Q9BV94","EDEM2",0.8636,"Cardiometabolic_II","B13805","20212097_SS211647","PASS",0.3676,0.2943,"Plate control","PASS","PFC",1.22628983334429,"24h","24h"
1035,"PFC_03",25,"OID30136","Q9BV94","EDEM2",0.8636,"Cardiometabolic_II","B13805","20212097_SS211647","PASS",0.3676,-0.9143,"Plate control","PASS","PFC",0.530601257572592,"Baseline","Baseline"
1036,"PFC_11",26,"OID30136","Q9BV94","EDEM2",0.8636,"Cardiometabolic_II","B13805","20212097_SS211647","PASS",0.3676,-0.0857,"Plate control","PASS","PFC",0.942327204966329,"1h","1h"
1037,"PFC_19",27,"OID30136","Q9BV94","EDEM2",0.8636,"Cardiometabolic_II","B13805","20212097_SS211647","PASS",0.3676,0.033,"Plate control","PASS","PFC",1.02313746973443,"24h","24h"
1038,"PFC_27",28,"OID30136","Q9BV94","EDEM2",0.8636,"Cardiometabolic_II","B13805","20212097_SS211647","PASS",0.3676,-0.3007,"Plate control","PASS","PFC",0.8118583846309,"24h","24h"
1039,"PFC_04",37,"OID30136","Q9BV94","EDEM2",0.8636,"Cardiometabolic_II","B13805","20212097_SS211647","PASS",0.3676,-0.5458,"Plate control","PASS","PFC",0.685011446151281,"Baseline","Baseline"
1040,"PFC_12",38,"OID30136","Q9BV94","EDEM2",0.8636,"Cardiometabolic_II","B13805","20212097_SS211647","PASS",0.3676,-0.6507,"Plate control","PASS","PFC",0.636971178325548,"1h","1h"
1041,"PFC_20",39,"OID30136","Q9BV94","EDEM2",0.8636,"Cardiometabolic_II","B13805","20212097_SS211647","PASS",0.3676,-0.4093,"Plate control","PASS","PFC",0.75298863745056,"24h","24h"
1042,"PFC_05",49,"OID30136","Q9BV94","EDEM2",0.8636,"Cardiometabolic_II","B13805","20212097_SS211647","PASS",0.3676,0.5142,"Plate control","PASS","PFC",1.42820195679394,"Baseline","Baseline"
1043,"PFC_13",50,"OID30136","Q9BV94","EDEM2",0.8636,"Cardiometabolic_II","B13805","20212097_SS211647","PASS",0.3676,-0.1283,"Plate control","PASS","PFC",0.914908898388858,"1h","1h"
1044,"PFC_21",51,"OID30136","Q9BV94","EDEM2",0.8636,"Cardiometabolic_II","B13805","20212097_SS211647","PASS",0.3676,-1.1258,"Plate control","PASS","PFC",0.458247844574841,"24h","24h"
1045,"PFC_06",61,"OID30136","Q9BV94","EDEM2",0.8636,"Cardiometabolic_II","B13805","20212097_SS211647","PASS",0.3676,-0.0431,"Plate control","PASS","PFC",0.970567192846606,"Baseline","Baseline"
1046,"PFC_14",62,"OID30136","Q9BV94","EDEM2",0.8636,"Cardiometabolic_II","B13805","20212097_SS211647","PASS",0.3676,-0.4095,"Plate control","PASS","PFC",0.752884258295531,"1h","1h"
1047,"PFC_22",63,"OID30136","Q9BV94","EDEM2",0.8636,"Cardiometabolic_II","B13805","20212097_SS211647","PASS",0.3676,0.241,"Plate control","PASS","PFC",1.18181154693437,"24h","24h"
1048,"PFC_07",73,"OID30136","Q9BV94","EDEM2",0.8636,"Cardiometabolic_II","B13805","20212097_SS211647","PASS",0.3676,0.4273,"Plate control","PASS","PFC",1.34471459061954,"Baseline","Baseline"
1049,"PFC_15",74,"OID30136","Q9BV94","EDEM2",0.8636,"Cardiometabolic_II","B13805","20212097_SS211647","PASS",0.3676,-0.0128,"Plate control","PASS","PFC",0.991166958656908,"1h","1h"
1050,"PFC_23",75,"OID30136","Q9BV94","EDEM2",0.8636,"Cardiometabolic_II","B13805","20212097_SS211647","PASS",0.3676,0.6511,"Plate control","PASS","PFC",1.57036508298777,"24h","24h"
1051,"PFC_08",85,"OID30136","Q9BV94","EDEM2",0.8636,"Cardiometabolic_II","B13805","20212097_SS211647","PASS",0.3676,-0.0787,"Plate control","PASS","PFC",0.946910515271733,"Baseline","Baseline"
1052,"PFC_16",86,"OID30136","Q9BV94","EDEM2",0.8636,"Cardiometabolic_II","B13805","20212097_SS211647","PASS",0.3676,-0.0176,"Plate control","PASS","PFC",0.987874720509598,"1h","1h"
1053,"PFC_24",87,"OID30136","Q9BV94","EDEM2",0.8636,"Cardiometabolic_II","B13805","20212097_SS211647","PASS",0.3676,0.3295,"Plate control","PASS","PFC",1.25657780236685,"24h","24h"
1054,"PFC_01",1,"OID30137","Q02127","DHODH",0.8409,"Cardiometabolic_II","B13805","20212097_SS211647","WARN",0.567,0.2587,"Plate control","PASS","PFC",1.19640015294444,"Baseline","Baseline"
1055,"PFC_09",2,"OID30137","Q02127","DHODH",0.8409,"Cardiometabolic_II","B13805","20212097_SS211647","PASS",0.567,-0.0238,"Plate control","PASS","PFC",0.983638425815153,"Baseline","Baseline"
1056,"PFC_17",3,"OID30137","Q02127","DHODH",0.8409,"Cardiometabolic_II","B13805","20212097_SS211647","PASS",0.567,0.6068,"Plate control","PASS","PFC",1.5228776094049,"1h","1h"
1057,"PFC_25",4,"OID30137","Q02127","DHODH",0.8409,"Cardiometabolic_II","B13805","20212097_SS211647","PASS",0.567,-0.2883,"Plate control","PASS","PFC",0.818866401673121,"24h","24h"
1058,"PFC_02",13,"OID30137","Q02127","DHODH",0.8409,"Cardiometabolic_II","B13805","20212097_SS211647","PASS",0.567,-0.522,"Plate control","PASS","PFC",0.696405740334518,"Baseline","Baseline"
1059,"PFC_10",14,"OID30137","Q02127","DHODH",0.8409,"Cardiometabolic_II","B13805","20212097_SS211647","PASS",0.567,0.225,"Plate control","PASS","PFC",1.16877724856125,"1h","1h"
1060,"PFC_18",15,"OID30137","Q02127","DHODH",0.8409,"Cardiometabolic_II","B13805","20212097_SS211647","PASS",0.567,0.3066,"Plate control","PASS","PFC",1.23678952027767,"1h","1h"
1061,"PFC_26",16,"OID30137","Q02127","DHODH",0.8409,"Cardiometabolic_II","B13805","20212097_SS211647","PASS",0.567,-0.2471,"Plate control","PASS","PFC",0.842588423699958,"24h","24h"
1062,"PFC_03",25,"OID30137","Q02127","DHODH",0.8409,"Cardiometabolic_II","B13805","20212097_SS211647","PASS",0.567,-0.1856,"Plate control","PASS","PFC",0.879283316219185,"Baseline","Baseline"
1063,"PFC_11",26,"OID30137","Q02127","DHODH",0.8409,"Cardiometabolic_II","B13805","20212097_SS211647","PASS",0.567,0.1699,"Plate control","PASS","PFC",1.12498050427977,"1h","1h"
1064,"PFC_19",27,"OID30137","Q02127","DHODH",0.8409,"Cardiometabolic_II","B13805","20212097_SS211647","PASS",0.567,0.3367,"Plate control","PASS","PFC",1.26286462922313,"24h","24h"
1065,"PFC_27",28,"OID30137","Q02127","DHODH",0.8409,"Cardiometabolic_II","B13805","20212097_SS211647","PASS",0.567,0.4137,"Plate control","PASS","PFC",1.33209779434044,"24h","24h"
1066,"PFC_04",37,"OID30137","Q02127","DHODH",0.8409,"Cardiometabolic_II","B13805","20212097_SS211647","PASS",0.567,0.8314,"Plate control","PASS","PFC",1.77941127625191,"Baseline","Baseline"
1067,"PFC_12",38,"OID30137","Q02127","DHODH",0.8409,"Cardiometabolic_II","B13805","20212097_SS211647","PASS",0.567,-0.0123,"Plate control","PASS","PFC",0.991510530481358,"1h","1h"
1068,"PFC_20",39,"OID30137","Q02127","DHODH",0.8409,"Cardiometabolic_II","B13805","20212097_SS211647","PASS",0.567,-0.6159,"Plate control","PASS","PFC",0.652522701704076,"24h","24h"
1069,"PFC_05",49,"OID30137","Q02127","DHODH",0.8409,"Cardiometabolic_II","B13805","20212097_SS211647","PASS",0.567,-0.1589,"Plate control","PASS","PFC",0.895707753669578,"Baseline","Baseline"
1070,"PFC_13",50,"OID30137","Q02127","DHODH",0.8409,"Cardiometabolic_II","B13805","20212097_SS211647","PASS",0.567,-0.488,"Plate control","PASS","PFC",0.71301285868207,"1h","1h"
1071,"PFC_21",51,"OID30137","Q02127","DHODH",0.8409,"Cardiometabolic_II","B13805","20212097_SS211647","PASS",0.567,-0.8971,"Plate control","PASS","PFC",0.536965014941483,"24h","24h"
1072,"PFC_06",61,"OID30137","Q02127","DHODH",0.8409,"Cardiometabolic_II","B13805","20212097_SS211647","PASS",0.567,0.5002,"Plate control","PASS","PFC",1.41440962759168,"Baseline","Baseline"
1073,"PFC_14",62,"OID30137","Q02127","DHODH",0.8409,"Cardiometabolic_II","B13805","20212097_SS211647","PASS",0.567,-0.4455,"Plate control","PASS","PFC",0.734329773226734,"1h","1h"
1074,"PFC_22",63,"OID30137","Q02127","DHODH",0.8409,"Cardiometabolic_II","B13805","20212097_SS211647","PASS",0.567,0.6083,"Plate control","PASS","PFC",1.52446180030223,"24h","24h"
1075,"PFC_07",73,"OID30137","Q02127","DHODH",0.8409,"Cardiometabolic_II","B13805","20212097_SS211647","PASS",0.567,0.3474,"Plate control","PASS","PFC",1.27226570480146,"Baseline","Baseline"
1076,"PFC_15",74,"OID30137","Q02127","DHODH",0.8409,"Cardiometabolic_II","B13805","20212097_SS211647","PASS",0.567,-0.1532,"Plate control","PASS","PFC",0.899253640481269,"1h","1h"
1077,"PFC_23",75,"OID30137","Q02127","DHODH",0.8409,"Cardiometabolic_II","B13805","20212097_SS211647","PASS",0.567,-0.2251,"Plate control","PASS","PFC",0.855535722409945,"24h","24h"
1078,"PFC_08",85,"OID30137","Q02127","DHODH",0.8409,"Cardiometabolic_II","B13805","20212097_SS211647","PASS",0.567,0.3677,"Plate control","PASS","PFC",1.29029415342683,"Baseline","Baseline"
1079,"PFC_16",86,"OID30137","Q02127","DHODH",0.8409,"Cardiometabolic_II","B13805","20212097_SS211647","PASS",0.567,0.2783,"Plate control","PASS","PFC",1.21276498023113,"1h","1h"
1080,"PFC_24",87,"OID30137","Q02127","DHODH",0.8409,"Cardiometabolic_II","B13805","20212097_SS211647","PASS",0.567,-0.5384,"Plate control","PASS","PFC",0.688534094537921,"24h","24h"
1081,"PFC_01",1,"OID30138","P57078","RIPK4",0.8409,"Cardiometabolic_II","B13805","20212097_SS211647","WARN",-0.0689,-1.3534,"Plate control","PASS","PFC",0.391368622661199,"Baseline","Baseline"
1082,"PFC_09",2,"OID30138","P57078","RIPK4",0.8409,"Cardiometabolic_II","B13805","20212097_SS211647","PASS",-0.0689,-0.9504,"Plate control","PASS","PFC",0.517488963622078,"Baseline","Baseline"
1083,"PFC_17",3,"OID30138","P57078","RIPK4",0.8409,"Cardiometabolic_II","B13805","20212097_SS211647","PASS",-0.0689,-1.0367,"Plate control","PASS","PFC",0.487441165448695,"1h","1h"
1084,"PFC_25",4,"OID30138","P57078","RIPK4",0.8409,"Cardiometabolic_II","B13805","20212097_SS211647","PASS",-0.0689,0.0772,"Plate control","PASS","PFC",1.05496855662739,"24h","24h"
1085,"PFC_02",13,"OID30138","P57078","RIPK4",0.8409,"Cardiometabolic_II","B13805","20212097_SS211647","PASS",-0.0689,-0.9538,"Plate control","PASS","PFC",0.516270833115448,"Baseline","Baseline"
1086,"PFC_10",14,"OID30138","P57078","RIPK4",0.8409,"Cardiometabolic_II","B13805","20212097_SS211647","PASS",-0.0689,-0.5589,"Plate control","PASS","PFC",0.678819540494397,"1h","1h"
1087,"PFC_18",15,"OID30138","P57078","RIPK4",0.8409,"Cardiometabolic_II","B13805","20212097_SS211647","PASS",-0.0689,-1.4886,"Plate control","PASS","PFC",0.356358193312178,"1h","1h"
1088,"PFC_26",16,"OID30138","P57078","RIPK4",0.8409,"Cardiometabolic_II","B13805","20212097_SS211647","PASS",-0.0689,-0.8078,"Plate control","PASS","PFC",0.571252310320986,"24h","24h"
1089,"PFC_03",25,"OID30138","P57078","RIPK4",0.8409,"Cardiometabolic_II","B13805","20212097_SS211647","PASS",-0.0689,-1.7152,"Plate control","PASS","PFC",0.304560341963655,"Baseline","Baseline"
1090,"PFC_11",26,"OID30138","P57078","RIPK4",0.8409,"Cardiometabolic_II","B13805","20212097_SS211647","PASS",-0.0689,-1.0396,"Plate control","PASS","PFC",0.486462331007362,"1h","1h"
1091,"PFC_19",27,"OID30138","P57078","RIPK4",0.8409,"Cardiometabolic_II","B13805","20212097_SS211647","PASS",-0.0689,-1.3074,"Plate control","PASS","PFC",0.404048392869516,"24h","24h"
1092,"PFC_27",28,"OID30138","P57078","RIPK4",0.8409,"Cardiometabolic_II","B13805","20212097_SS211647","PASS",-0.0689,-0.6644,"Plate control","PASS","PFC",0.630951055024485,"24h","24h"
1093,"PFC_04",37,"OID30138","P57078","RIPK4",0.8409,"Cardiometabolic_II","B13805","20212097_SS211647","PASS",-0.0689,1.5312,"Plate control","PASS","PFC",2.89026144338975,"Baseline","Baseline"
1094,"PFC_12",38,"OID30138","P57078","RIPK4",0.8409,"Cardiometabolic_II","B13805","20212097_SS211647","PASS",-0.0689,-1.1668,"Plate control","PASS","PFC",0.445408192710179,"1h","1h"
1095,"PFC_20",39,"OID30138","P57078","RIPK4",0.8409,"Cardiometabolic_II","B13805","20212097_SS211647","PASS",-0.0689,-1.0232,"Plate control","PASS","PFC",0.492023797307221,"24h","24h"
1096,"PFC_05",49,"OID30138","P57078","RIPK4",0.8409,"Cardiometabolic_II","B13805","20212097_SS211647","PASS",-0.0689,-1.1158,"Plate control","PASS","PFC",0.461435210396165,"Baseline","Baseline"
1097,"PFC_13",50,"OID30138","P57078","RIPK4",0.8409,"Cardiometabolic_II","B13805","20212097_SS211647","PASS",-0.0689,0.8301,"Plate control","PASS","PFC",1.77780858636339,"1h","1h"
1098,"PFC_21",51,"OID30138","P57078","RIPK4",0.8409,"Cardiometabolic_II","B13805","20212097_SS211647","PASS",-0.0689,-0.4244,"Plate control","PASS","PFC",0.745148565705136,"24h","24h"
1099,"PFC_06",61,"OID30138","P57078","RIPK4",0.8409,"Cardiometabolic_II","B13805","20212097_SS211647","PASS",-0.0689,-0.655,"Plate control","PASS","PFC",0.635075491269395,"Baseline","Baseline"
1100,"PFC_14",62,"OID30138","P57078","RIPK4",0.8409,"Cardiometabolic_II","B13805","20212097_SS211647","PASS",-0.0689,-0.8202,"Plate control","PASS","PFC",0.566363422563523,"1h","1h"
1101,"PFC_22",63,"OID30138","P57078","RIPK4",0.8409,"Cardiometabolic_II","B13805","20212097_SS211647","PASS",-0.0689,1.1625,"Plate control","PASS","PFC",2.2384498552562,"24h","24h"
1102,"PFC_07",73,"OID30138","P57078","RIPK4",0.8409,"Cardiometabolic_II","B13805","20212097_SS211647","PASS",-0.0689,-0.492,"Plate control","PASS","PFC",0.711038705293637,"Baseline","Baseline"
1103,"PFC_15",74,"OID30138","P57078","RIPK4",0.8409,"Cardiometabolic_II","B13805","20212097_SS211647","PASS",-0.0689,-0.6153,"Plate control","PASS","PFC",0.652794134705632,"1h","1h"
1104,"PFC_23",75,"OID30138","P57078","RIPK4",0.8409,"Cardiometabolic_II","B13805","20212097_SS211647","PASS",-0.0689,-1.2642,"Plate control","PASS","PFC",0.416330165823423,"24h","24h"
1105,"PFC_08",85,"OID30138","P57078","RIPK4",0.8409,"Cardiometabolic_II","B13805","20212097_SS211647","PASS",-0.0689,-1.1034,"Plate control","PASS","PFC",0.465418350657514,"Baseline","Baseline"
1106,"PFC_16",86,"OID30138","P57078","RIPK4",0.8409,"Cardiometabolic_II","B13805","20212097_SS211647","PASS",-0.0689,-0.4086,"Plate control","PASS","PFC",0.753354078465664,"1h","1h"
1107,"PFC_24",87,"OID30138","P57078","RIPK4",0.8409,"Cardiometabolic_II","B13805","20212097_SS211647","PASS",-0.0689,-0.5721,"Plate control","PASS","PFC",0.67263697900247,"24h","24h"
1108,"PFC_01",1,"OID30139","Q6ZN66","GBP6",0.8409,"Cardiometabolic_II","B13805","20212097_SS211647","WARN",0.201,0.2797,"Plate control","PASS","PFC",1.21394242591636,"Baseline","Baseline"
1109,"PFC_09",2,"OID30139","Q6ZN66","GBP6",0.8409,"Cardiometabolic_II","B13805","20212097_SS211647","PASS",0.201,0.0172,"Plate control","PASS","PFC",1.01199348338902,"Baseline","Baseline"
1110,"PFC_17",3,"OID30139","Q6ZN66","GBP6",0.8409,"Cardiometabolic_II","B13805","20212097_SS211647","PASS",0.201,0.044,"Plate control","PASS","PFC",1.03096831879479,"1h","1h"
1111,"PFC_25",4,"OID30139","Q6ZN66","GBP6",0.8409,"Cardiometabolic_II","B13805","20212097_SS211647","PASS",0.201,0.554,"Plate control","PASS","PFC",1.4681506360341,"24h","24h"
1112,"PFC_02",13,"OID30139","Q6ZN66","GBP6",0.8409,"Cardiometabolic_II","B13805","20212097_SS211647","PASS",0.201,-0.7463,"Plate control","PASS","PFC",0.596130461431276,"Baseline","Baseline"
1113,"PFC_10",14,"OID30139","Q6ZN66","GBP6",0.8409,"Cardiometabolic_II","B13805","20212097_SS211647","PASS",0.201,-8e-04,"Plate control","PASS","PFC",0.999445635972102,"1h","1h"
1114,"PFC_18",15,"OID30139","Q6ZN66","GBP6",0.8409,"Cardiometabolic_II","B13805","20212097_SS211647","PASS",0.201,-1.1495,"Plate control","PASS","PFC",0.450781433175857,"1h","1h"
1115,"PFC_26",16,"OID30139","Q6ZN66","GBP6",0.8409,"Cardiometabolic_II","B13805","20212097_SS211647","PASS",0.201,0.3607,"Plate control","PASS","PFC",1.28404877079032,"24h","24h"
1116,"PFC_03",25,"OID30139","Q6ZN66","GBP6",0.8409,"Cardiometabolic_II","B13805","20212097_SS211647","PASS",0.201,-0.9818,"Plate control","PASS","PFC",0.506347593491309,"Baseline","Baseline"
1117,"PFC_11",26,"OID30139","Q6ZN66","GBP6",0.8409,"Cardiometabolic_II","B13805","20212097_SS211647","PASS",0.201,-0.4494,"Plate control","PASS","PFC",0.732347359351458,"1h","1h"
1118,"PFC_19",27,"OID30139","Q6ZN66","GBP6",0.8409,"Cardiometabolic_II","B13805","20212097_SS211647","PASS",0.201,-0.2417,"Plate control","PASS","PFC",0.845748137474469,"24h","24h"
1119,"PFC_27",28,"OID30139","Q6ZN66","GBP6",0.8409,"Cardiometabolic_II","B13805","20212097_SS211647","PASS",0.201,-0.2564,"Plate control","PASS","PFC",0.83717434131389,"24h","24h"
1120,"PFC_04",37,"OID30139","Q6ZN66","GBP6",0.8409,"Cardiometabolic_II","B13805","20212097_SS211647","PASS",0.201,-0.199,"Plate control","PASS","PFC",0.871154192142256,"Baseline","Baseline"

Supplemental table 1_Olink proximity extension assay (PEA) data

,"SampleID","Index","OlinkID","UniProt","Assay","MissingFreq","Panel","Panel_Lot_Nr","PlateID","QC_Warning","LOD","NPX","Normalization","Assay_Warning","condition","Value","group","alt_group"
1121,"PFC_12",38,"OID30139","Q6ZN66","GBP6",0.8409,"Cardiometabolic_II","B13805","20212097_SS211647","PASS",0.201,-0.3487,"Plate control","PASS","PFC",0.785291398452073,"1h","1h"
1122,"PFC_20",39,"OID30139","Q6ZN66","GBP6",0.8409,"Cardiometabolic_II","B13805","20212097_SS211647","PASS",0.201,-0.5846,"Plate control","PASS","PFC",0.666834198615236,"24h","24h"
1123,"PFC_05",49,"OID30139","Q6ZN66","GBP6",0.8409,"Cardiometabolic_II","B13805","20212097_SS211647","PASS",0.201,-0.347,"Plate control","PASS","PFC",0.786217292140332,"Baseline","Baseline"
1124,"PFC_13",50,"OID30139","Q6ZN66","GBP6",0.8409,"Cardiometabolic_II","B13805","20212097_SS211647","PASS",0.201,-1.172,"Plate control","PASS","PFC",0.443805668634749,"1h","1h"
1125,"PFC_21",51,"OID30139","Q6ZN66","GBP6",0.8409,"Cardiometabolic_II","B13805","20212097_SS211647","PASS",0.201,-0.6556,"Plate control","PASS","PFC",0.63481142571236,"24h","24h"
1126,"PFC_06",61,"OID30139","Q6ZN66","GBP6",0.8409,"Cardiometabolic_II","B13805","20212097_SS211647","PASS",0.201,-0.018,"Plate control","PASS","PFC",0.987600861445373,"Baseline","Baseline"
1127,"PFC_14",62,"OID30139","Q6ZN66","GBP6",0.8409,"Cardiometabolic_II","B13805","20212097_SS211647","PASS",0.201,-0.7573,"Plate control","PASS","PFC",0.591602477807874,"1h","1h"
1128,"PFC_22",63,"OID30139","Q6ZN66","GBP6",0.8409,"Cardiometabolic_II","B13805","20212097_SS211647","PASS",0.201,-0.822,"Plate control","PASS","PFC",0.56565723142295,"24h","24h"
1129,"PFC_07",73,"OID30139","Q6ZN66","GBP6",0.8409,"Cardiometabolic_II","B13805","20212097_SS211647","PASS",0.201,-0.9166,"Plate control","PASS","PFC",0.529756026540462,"Baseline","Baseline"
1130,"PFC_15",74,"OID30139","Q6ZN66","GBP6",0.8409,"Cardiometabolic_II","B13805","20212097_SS211647","PASS",0.201,-0.4006,"Plate control","PASS","PFC",0.757543164387486,"1h","1h"
1131,"PFC_23",75,"OID30139","Q6ZN66","GBP6",0.8409,"Cardiometabolic_II","B13805","20212097_SS211647","PASS",0.201,-1.5754,"Plate control","PASS","PFC",0.335550078560234,"24h","24h"
1132,"PFC_08",85,"OID30139","Q6ZN66","GBP6",0.8409,"Cardiometabolic_II","B13805","20212097_SS211647","PASS",0.201,-0.2558,"Plate control","PASS","PFC",0.837522584744648,"Baseline","Baseline"
1133,"PFC_16",86,"OID30139","Q6ZN66","GBP6",0.8409,"Cardiometabolic_II","B13805","20212097_SS211647","PASS",0.201,-0.4175,"Plate control","PASS","PFC",0.748720935338564,"1h","1h"
1134,"PFC_24",87,"OID30139","Q6ZN66","GBP6",0.8409,"Cardiometabolic_II","B13805","20212097_SS211647","PASS",0.201,-0.4295,"Plate control","PASS","PFC",0.742519078229278,"24h","24h"
1135,"PFC_01",1,"OID30140","Q9BZL6","PRKD2",0.1364,"Cardiometabolic_II","B13805","20212097_SS211647","WARN",0.5334,0.7482,"Plate control","PASS","PFC",1.67969582503379,"Baseline","Baseline"
1136,"PFC_09",2,"OID30140","Q9BZL6","PRKD2",0.1364,"Cardiometabolic_II","B13805","20212097_SS211647","PASS",0.5334,0.9407,"Plate control","PASS","PFC",1.91945934022652,"Baseline","Baseline"
1137,"PFC_17",3,"OID30140","Q9BZL6","PRKD2",0.1364,"Cardiometabolic_II","B13805","20212097_SS211647","PASS",0.5334,1.3733,"Plate control","PASS","PFC",2.59062464695341,"1h","1h"
1138,"PFC_25",4,"OID30140","Q9BZL6","PRKD2",0.1364,"Cardiometabolic_II","B13805","20212097_SS211647","PASS",0.5334,1.1298,"Plate control","PASS","PFC",2.18828402091328,"24h","24h"
1139,"PFC_02",13,"OID30140","Q9BZL6","PRKD2",0.1364,"Cardiometabolic_II","B13805","20212097_SS211647","PASS",0.5334,0.6887,"Plate control","PASS","PFC",1.61183045741399,"Baseline","Baseline"
1140,"PFC_10",14,"OID30140","Q9BZL6","PRKD2",0.1364,"Cardiometabolic_II","B13805","20212097_SS211647","PASS",0.5334,0.5379,"Plate control","PASS","PFC",1.451857641313,"1h","1h"
1141,"PFC_18",15,"OID30140","Q9BZL6","PRKD2",0.1364,"Cardiometabolic_II","B13805","20212097_SS211647","PASS",0.5334,0.899,"Plate control","PASS","PFC",1.86477297287367,"1h","1h"
1142,"PFC_26",16,"OID30140","Q9BZL6","PRKD2",0.1364,"Cardiometabolic_II","B13805","20212097_SS211647","PASS",0.5334,1.0253,"Plate control","PASS","PFC",2.03538258611294,"24h","24h"
1143,"PFC_03",25,"OID30140","Q9BZL6","PRKD2",0.1364,"Cardiometabolic_II","B13805","20212097_SS211647","PASS",0.5334,0.6466,"Plate control","PASS","PFC",1.5654744906448,"Baseline","Baseline"
1144,"PFC_11",26,"OID30140","Q9BZL6","PRKD2",0.1364,"Cardiometabolic_II","B13805","20212097_SS211647","PASS",0.5334,1.3271,"Plate control","PASS","PFC",2.50897831213921,"1h","1h"
1145,"PFC_19",27,"OID30140","Q9BZL6","PRKD2",0.1364,"Cardiometabolic_II","B13805","20212097_SS211647","PASS",0.5334,1.122,"Plate control","PASS","PFC",2.17648488334161,"24h","24h"
1146,"PFC_27",28,"OID30140","Q9BZL6","PRKD2",0.1364,"Cardiometabolic_II","B13805","20212097_SS211647","PASS",0.5334,1.455,"Plate control","PASS","PFC",2.74156560995941,"24h","24h"
1147,"PFC_04",37,"OID30140","Q9BZL6","PRKD2",0.1364,"Cardiometabolic_II","B13805","20212097_SS211647","PASS",0.5334,1.408,"Plate control","PASS","PFC",2.65369028119432,"Baseline","Baseline"
1148,"PFC_12",38,"OID30140","Q9BZL6","PRKD2",0.1364,"Cardiometabolic_II","B13805","20212097_SS211647","PASS",0.5334,0.5002,"Plate control","PASS","PFC",1.41440962759168,"1h","1h"
1149,"PFC_20",39,"OID30140","Q9BZL6","PRKD2",0.1364,"Cardiometabolic_II","B13805","20212097_SS211647","PASS",0.5334,1.83,"Plate control","PASS","PFC",3.55537072466628,"24h","24h"
1150,"PFC_05",49,"OID30140","Q9BZL6","PRKD2",0.1364,"Cardiometabolic_II","B13805","20212097_SS211647","PASS",0.5334,0.2894,"Plate control","PASS","PFC",1.22213190161644,"Baseline","Baseline"
1151,"PFC_13",50,"OID30140","Q9BZL6","PRKD2",0.1364,"Cardiometabolic_II","B13805","20212097_SS211647","PASS",0.5334,1.4265,"Plate control","PASS","PFC",2.68793825844542,"1h","1h"
1152,"PFC_21",51,"OID30140","Q9BZL6","PRKD2",0.1364,"Cardiometabolic_II","B13805","20212097_SS211647","PASS",0.5334,0.6963,"Plate control","PASS","PFC",1.62034385326609,"24h","24h"
1153,"PFC_06",61,"OID30140","Q9BZL6","PRKD2",0.1364,"Cardiometabolic_II","B13805","20212097_SS211647","PASS",0.5334,1.0785,"Plate control","PASS","PFC",2.11183921615988,"Baseline","Baseline"
1154,"PFC_14",62,"OID30140","Q9BZL6","PRKD2",0.1364,"Cardiometabolic_II","B13805","20212097_SS211647","PASS",0.5334,1.2557,"Plate control","PASS","PFC",2.38782978727439,"1h","1h"
1155,"PFC_22",63,"OID30140","Q9BZL6","PRKD2",0.1364,"Cardiometabolic_II","B13805","20212097_SS211647","PASS",0.5334,0.6556,"Plate control","PASS","PFC",1.57527095369753,"24h","24h"
1156,"PFC_07",73,"OID30140","Q9BZL6","PRKD2",0.1364,"Cardiometabolic_II","B13805","20212097_SS211647","PASS",0.5334,1.3838,"Plate control","PASS","PFC",2.60954811011844,"Baseline","Baseline"
1157,"PFC_15",74,"OID30140","Q9BZL6","PRKD2",0.1364,"Cardiometabolic_II","B13805","20212097_SS211647","PASS",0.5334,0.6868,"Plate control","PASS","PFC",1.60970910670797,"1h","1h"
1158,"PFC_23",75,"OID30140","Q9BZL6","PRKD2",0.1364,"Cardiometabolic_II","B13805","20212097_SS211647","PASS",0.5334,1.4026,"Plate control","PASS","PFC",2.64377609827952,"24h","24h"
1159,"PFC_08",85,"OID30140","Q9BZL6","PRKD2",0.1364,"Cardiometabolic_II","B13805","20212097_SS211647","PASS",0.5334,1.4053,"Plate control","PASS","PFC",2.64872855114642,"Baseline","Baseline"
1160,"PFC_16",86,"OID30140","Q9BZL6","PRKD2",0.1364,"Cardiometabolic_II","B13805","20212097_SS211647","PASS",0.5334,1.0767,"Plate control","PASS","PFC",2.10920599147525,"1h","1h"
1161,"PFC_24",87,"OID30140","Q9BZL6","PRKD2",0.1364,"Cardiometabolic_II","B13805","20212097_SS211647","PASS",0.5334,1.6007,"Plate control","PASS","PFC",3.03290435044195,"24h","24h"
1162,"PFC_01",1,"OID30141","A6NHS7","MANSC4",0.6818,"Cardiometabolic_II","B13805","20212097_SS211647","WARN",0.4103,-0.9459,"Plate control","PASS","PFC",0.51910561568392,"Baseline","Baseline"
1163,"PFC_09",2,"OID30141","A6NHS7","MANSC4",0.6818,"Cardiometabolic_II","B13805","20212097_SS211647","PASS",0.4103,-0.0193,"Plate control","PASS","PFC",0.986711343695511,"Baseline","Baseline"
1164,"PFC_17",3,"OID30141","A6NHS7","MANSC4",0.6818,"Cardiometabolic_II","B13805","20212097_SS211647","PASS",0.4103,-0.1712,"Plate control","PASS","PFC",0.888103669997188,"1h","1h"
1165,"PFC_25",4,"OID30141","A6NHS7","MANSC4",0.6818,"Cardiometabolic_II","B13805","20212097_SS211647","PASS",0.4103,0.507,"Plate control","PASS","PFC",1.42109204318136,"24h","24h"
1166,"PFC_02",13,"OID30141","A6NHS7","MANSC4",0.6818,"Cardiometabolic_II","B13805","20212097_SS211647","PASS",0.4103,-0.2155,"Plate control","PASS","PFC",0.861247622301833,"Baseline","Baseline"
1167,"PFC_10",14,"OID30141","A6NHS7","MANSC4",0.6818,"Cardiometabolic_II","B13805","20212097_SS211647","PASS",0.4103,0.1336,"Plate control","PASS","PFC",1.09702773473719,"1h","1h"
1168,"PFC_18",15,"OID30141","A6NHS7","MANSC4",0.6818,"Cardiometabolic_II","B13805","20212097_SS211647","PASS",0.4103,0.1461,"Plate control","PASS","PFC",1.10657405230249,"1h","1h"
1169,"PFC_26",16,"OID30141","A6NHS7","MANSC4",0.6818,"Cardiometabolic_II","B13805","20212097_SS211647","PASS",0.4103,-0.4317,"Plate control","PASS","PFC",0.7413876561025,"24h","24h"
1170,"PFC_03",25,"OID30141","A6NHS7","MANSC4",0.6818,"Cardiometabolic_II","B13805","20212097_SS211647","PASS",0.4103,-0.5405,"Plate control","PASS","PFC",0.687532587137055,"Baseline","Baseline"
1171,"PFC_11",26,"OID30141","A6NHS7","MANSC4",0.6818,"Cardiometabolic_II","B13805","20212097_SS211647","PASS",0.4103,-0.0621,"Plate control","PASS","PFC",0.957868821514291,"1h","1h"
1172,"PFC_19",27,"OID30141","A6NHS7","MANSC4",0.6818,"Cardiometabolic_II","B13805","20212097_SS211647","PASS",0.4103,-0.1789,"Plate control","PASS","PFC",0.883376280337219,"24h","24h"
1173,"PFC_27",28,"OID30141","A6NHS7","MANSC4",0.6818,"Cardiometabolic_II","B13805","20212097_SS211647","PASS",0.4103,0.452,"Plate control","PASS","PFC",1.36793530390249,"24h","24h"
1174,"PFC_04",37,"OID30141","A6NHS7","MANSC4",0.6818,"Cardiometabolic_II","B13805","20212097_SS211647","PASS",0.4103,0.24,"Plate control","PASS","PFC",1.18099266142953,"Baseline","Baseline"
1175,"PFC_12",38,"OID30141","A6NHS7","MANSC4",0.6818,"Cardiometabolic_II","B13805","20212097_SS211647","PASS",0.4103,-1.7112,"Plate control","PASS","PFC",0.305405934231128,"1h","1h"
1176,"PFC_20",39,"OID30141","A6NHS7","MANSC4",0.6818,"Cardiometabolic_II","B13805","20212097_SS211647","PASS",0.4103,0.3288,"Plate control","PASS","PFC",1.25596825490334,"24h","24h"
1177,"PFC_05",49,"OID30141","A6NHS7","MANSC4",0.6818,"Cardiometabolic_II","B13805","20212097_SS211647","PASS",0.4103,1.5234,"Plate control","PASS","PFC",2.8746772723851,"Baseline","Baseline"
1178,"PFC_13",50,"OID30141","A6NHS7","MANSC4",0.6818,"Cardiometabolic_II","B13805","20212097_SS211647","PASS",0.4103,0.6399,"Plate control","PASS","PFC",1.55822114791812,"1h","1h"
1179,"PFC_21",51,"OID30141","A6NHS7","MANSC4",0.6818,"Cardiometabolic_II","B13805","20212097_SS211647","PASS",0.4103,-0.8676,"Plate control","PASS","PFC",0.548057816052502,"24h","24h"
1180,"PFC_06",61,"OID30141","A6NHS7","MANSC4",0.6818,"Cardiometabolic_II","B13805","20212097_SS211647","PASS",0.4103,-0.2282,"Plate control","PASS","PFC",0.853699358326963,"Baseline","Baseline"
1181,"PFC_14",62,"OID30141","A6NHS7","MANSC4",0.6818,"Cardiometabolic_II","B13805","20212097_SS211647","PASS",0.4103,1.6812,"Plate control","PASS","PFC",3.20694586388001,"1h","1h"
1182,"PFC_22",63,"OID30141","A6NHS7","MANSC4",0.6818,"Cardiometabolic_II","B13805","20212097_SS211647","PASS",0.4103,-0.8047,"Plate control","PASS","PFC",0.57248111202355,"24h","24h"
1183,"PFC_07",73,"OID30141","A6NHS7","MANSC4",0.6818,"Cardiometabolic_II","B13805","20212097_SS211647","PASS",0.4103,-0.0446,"Plate control","PASS","PFC",0.969558598395867,"Baseline","Baseline"
1184,"PFC_15",74,"OID30141","A6NHS7","MANSC4",0.6818,"Cardiometabolic_II","B13805","20212097_SS211647","PASS",0.4103,0.8959,"Plate control","PASS","PFC",1.86077033216492,"1h","1h"
1185,"PFC_23",75,"OID30141","A6NHS7","MANSC4",0.6818,"Cardiometabolic_II","B13805","20212097_SS211647","PASS",0.4103,1.6358,"Plate control","PASS","PFC",3.10759824068195,"24h","24h"
1186,"PFC_08",85,"OID30141","A6NHS7","MANSC4",0.6818,"Cardiometabolic_II","B13805","20212097_SS211647","PASS",0.4103,0.1284,"Plate control","PASS","PFC",1.09308076343064,"Baseline","Baseline"
1187,"PFC_16",86,"OID30141","A6NHS7","MANSC4",0.6818,"Cardiometabolic_II","B13805","20212097_SS211647","PASS",0.4103,0.5471,"Plate control","PASS","PFC",1.46114565393474,"1h","1h"
1188,"PFC_24",87,"OID30141","A6NHS7","MANSC4",0.6818,"Cardiometabolic_II","B13805","20212097_SS211647","PASS",0.4103,0.3726,"Plate control","PASS","PFC",1.29468398646507,"24h","24h"
1189,"PFC_01",1,"OID30142","O75521","ECI2",0.1364,"Cardiometabolic_II","B13805","20212097_SS211647","WARN",1.1171,0.5976,"Plate control","PASS","PFC",1.51319718745694,"Baseline","Baseline"
1190,"PFC_09",2,"OID30142","O75521","ECI2",0.1364,"Cardiometabolic_II","B13805","20212097_SS211647","PASS",1.1171,0.7624,"Plate control","PASS","PFC",1.69631018112029,"Baseline","Baseline"
1191,"PFC_17",3,"OID30142","O75521","ECI2",0.1364,"Cardiometabolic_II","B13805","20212097_SS211647","PASS",1.1171,2.5677,"Plate control","PASS","PFC",5.92863508601475,"1h","1h"
1192,"PFC_25",4,"OID30142","O75521","ECI2",0.1364,"Cardiometabolic_II","B13805","20212097_SS211647","PASS",1.1171,1.5008,"Plate control","PASS","PFC",2.82999597271256,"24h","24h"
1193,"PFC_02",13,"OID30142","O75521","ECI2",0.1364,"Cardiometabolic_II","B13805","20212097_SS211647","PASS",1.1171,1.7456,"Plate control","PASS","PFC",3.35334286480104,"Baseline","Baseline"
1194,"PFC_10",14,"OID30142","O75521","ECI2",0.1364,"Cardiometabolic_II","B13805","20212097_SS211647","PASS",1.1171,1.7291,"Plate control","PASS","PFC",3.31520940279319,"1h","1h"
1195,"PFC_18",15,"OID30142","O75521","ECI2",0.1364,"Cardiometabolic_II","B13805","20212097_SS211647","PASS",1.1171,1.5058,"Plate control","PASS","PFC",2.83982100701272,"1h","1h"
1196,"PFC_26",16,"OID30142","O75521","ECI2",0.1364,"Cardiometabolic_II","B13805","20212097_SS211647","PASS",1.1171,2.4982,"Plate control","PASS","PFC",5.64980079195018,"24h","24h"
1197,"PFC_03",25,"OID30142","O75521","ECI2",0.1364,"Cardiometabolic_II","B13805","20212097_SS211647","PASS",1.1171,1.6242,"Plate control","PASS","PFC",3.0827117584058,"Baseline","Baseline"
1198,"PFC_11",26,"OID30142","O75521","ECI2",0.1364,"Cardiometabolic_II","B13805","20212097_SS211647","PASS",1.1171,2.2112,"Plate control","PASS","PFC",4.63060276131646,"1h","1h"
1199,"PFC_19",27,"OID30142","O75521","ECI2",0.1364,"Cardiometabolic_II","B13805","20212097_SS211647","PASS",1.1171,2.1011,"Plate control","PASS","PFC",4.29036384232551,"24h","24h"
1200,"PFC_27",28,"OID30142","O75521","ECI2",0.1364,"Cardiometabolic_II","B13805","20212097_SS211647","PASS",1.1171,2.6718,"Plate control","PASS","PFC",6.37223732938287,"24h","24h"
1201,"PFC_04",37,"OID30142","O75521","ECI2",0.1364,"Cardiometabolic_II","B13805","20212097_SS211647","PASS",1.1171,2.2591,"Plate control","PASS","PFC",4.78692764596379,"Baseline","Baseline"
1202,"PFC_12",38,"OID30142","O75521","ECI2",0.1364,"Cardiometabolic_II","B13805","20212097_SS211647","PASS",1.1171,0.7064,"Plate control","PASS","PFC",1.63172733484699,"1h","1h"
1203,"PFC_20",39,"OID30142","O75521","ECI2",0.1364,"Cardiometabolic_II","B13805","20212097_SS211647","PASS",1.1171,2.8792,"Plate control","PASS","PFC",7.35742025353962,"24h","24h"
1204,"PFC_05",49,"OID30142","O75521","ECI2",0.1364,"Cardiometabolic_II","B13805","20212097_SS211647","PASS",1.1171,1.7253,"Plate control","PASS","PFC",3.30648876614398,"Baseline","Baseline"
1205,"PFC_13",50,"OID30142","O75521","ECI2",0.1364,"Cardiometabolic_II","B13805","20212097_SS211647","PASS",1.1171,2.0586,"Plate control","PASS","PFC",4.1658185457478,"1h","1h"
1206,"PFC_21",51,"OID30142","O75521","ECI2",0.1364,"Cardiometabolic_II","B13805","20212097_SS211647","PASS",1.1171,1.2616,"Plate control","PASS","PFC",2.3976149753943,"24h","24h"
1207,"PFC_06",61,"OID30142","O75521","ECI2",0.1364,"Cardiometabolic_II","B13805","20212097_SS211647","PASS",1.1171,1.4205,"Plate control","PASS","PFC",2.67678265100602,"Baseline","Baseline"
1208,"PFC_14",62,"OID30142","O75521","ECI2",0.1364,"Cardiometabolic_II","B13805","20212097_SS211647","PASS",1.1171,2.462,"Plate control","PASS","PFC",5.50980018553255,"1h","1h"
1209,"PFC_22",63,"OID30142","O75521","ECI2",0.1364,"Cardiometabolic_II","B13805","20212097_SS211647","PASS",1.1171,1.3793,"Plate control","PASS","PFC",2.60142118718553,"24h","24h"
1210,"PFC_07",73,"OID30142","O75521","ECI2",0.1364,"Cardiometabolic_II","B13805","20212097_SS211647","PASS",1.1171,1.9529,"Plate control","PASS","PFC",3.87151974497475,"Baseline","Baseline"
1211,"PFC_15",74,"OID30142","O75521","ECI2",0.1364,"Cardiometabolic_II","B13805","20212097_SS211647","PASS",1.1171,0.8864,"Plate control","PASS","PFC",1.84855760284481,"1h","1h"
1212,"PFC_23",75,"OID30142","O75521","ECI2",0.1364,"Cardiometabolic_II","B13805","20212097_SS211647","PASS",1.1171,2.8291,"Plate control","PASS","PFC",7.10630692132907,"24h","24h"
1213,"PFC_08",85,"OID30142","O75521","ECI2",0.1364,"Cardiometabolic_II","B13805","20212097_SS211647","PASS",1.1171,1.9399,"Plate control","PASS","PFC",3.83679052203058,"Baseline","Baseline"
1214,"PFC_16",86,"OID30142","O75521","ECI2",0.1364,"Cardiometabolic_II","B13805","20212097_SS211647","PASS",1.1171,1.8986,"Plate control","PASS","PFC",3.72851203938098,"1h","1h"
1215,"PFC_24",87,"OID30142","O75521","ECI2",0.1364,"Cardiometabolic_II","B13805","20212097_SS211647","PASS",1.1171,2.4009,"Plate control","PASS","PFC",5.28132527779997,"24h","24h"
1216,"PFC_01",1,"OID30143","P12270","TPR",0,"Cardiometabolic_II","B13805","20212097_SS211647","WARN",-1.2818,1.2815,"Plate control","PASS","PFC",2.4309159290955,"Baseline","Baseline"
1217,"PFC_09",2,"OID30143","P12270","TPR",0,"Cardiometabolic_II","B13805","20212097_SS211647","PASS",-1.2818,0.1232,"Plate control","PASS","PFC",1.08914799284299,"Baseline","Baseline"
1218,"PFC_17",3,"OID30143","P12270","TPR",0,"Cardiometabolic_II","B13805","20212097_SS211647","PASS",-1.2818,1.2979,"Plate control","PASS","PFC",2.458707303999,"1h","1h"
1219,"PFC_25",4,"OID30143","P12270","TPR",0,"Cardiometabolic_II","B13805","20212097_SS211647","PASS",-1.2818,0.4681,"Plate control","PASS","PFC",1.3832865078023,"24h","24h"
1220,"PFC_02",13,"OID30143","P12270","TPR",0,"Cardiometabolic_II","B13805","20212097_SS211647","PASS",-1.2818,0.4815,"Plate control","PASS","PFC",1.39619456444405,"Baseline","Baseline"
1221,"PFC_10",14,"OID30143","P12270","TPR",0,"Cardiometabolic_II","B13805","20212097_SS211647","PASS",-1.2818,1.1685,"Plate control","PASS","PFC",2.24777869181626,"1h","1h"
1222,"PFC_18",15,"OID30143","P12270","TPR",0,"Cardiometabolic_II","B13805","20212097_SS211647","PASS",-1.2818,-0.0624,"Plate control","PASS","PFC",0.957669659000455,"1h","1h"
1223,"PFC_26",16,"OID30143","P12270","TPR",0,"Cardiometabolic_II","B13805","20212097_SS211647","PASS",-1.2818,0.0928,"Plate control","PASS","PFC",1.06643793089482,"24h","24h"
1224,"PFC_03",25,"OID30143","P12270","TPR",0,"Cardiometabolic_II","B13805","20212097_SS211647","PASS",-1.2818,0.4265,"Plate control","PASS","PFC",1.34396912922271,"Baseline","Baseline"
1225,"PFC_11",26,"OID30143","P12270","TPR",0,"Cardiometabolic_II","B13805","20212097_SS211647","PASS",-1.2818,0.6508,"Plate control","PASS","PFC",1.5700385686984,"1h","1h"
1226,"PFC_19",27,"OID30143","P12270","TPR",0,"Cardiometabolic_II","B13805","20212097_SS211647","PASS",-1.2818,-0.0016,"Plate control","PASS","PFC",0.99889157926368,"24h","24h"
1227,"PFC_27",28,"OID30143","P12270","TPR",0,"Cardiometabolic_II","B13805","20212097_SS211647","PASS",-1.2818,1.3554,"Plate control","PASS","PFC",2.55868048108245,"24h","24h"
1228,"PFC_04",37,"OID30143","P12270","TPR",0,"Cardiometabolic_II","B13805","20212097_SS211647","PASS",-1.2818,0.7997,"Plate control","PASS","PFC",1.74073911243183,"Baseline","Baseline"
1229,"PFC_12",38,"OID30143","P12270","TPR",0,"Cardiometabolic_II","B13805","20212097_SS211647","PASS",-1.2818,0.7795,"Plate control","PASS","PFC",1.71653586377709,"1h","1h"
1230,"PFC_20",39,"OID30143","P12270","TPR",0,"Cardiometabolic_II","B13805","20212097_SS211647","PASS",-1.2818,0.7627,"Plate control","PASS","PFC",1.69666295558354,"24h","24h"
1231,"PFC_05",49,"OID30143","P12270","TPR",0,"Cardiometabolic_II","B13805","20212097_SS211647","PASS",-1.2818,0.6449,"Plate control","PASS","PFC",1.56363089986588,"Baseline","Baseline"
1232,"PFC_13",50,"OID30143","P12270","TPR",0,"Cardiometabolic_II","B13805","20212097_SS211647","PASS",-1.2818,0.8197,"Plate control","PASS","PFC",1.76503892489153,"1h","1h"

Supplemental table 1_Olink proximity extension assay (PEA) data

,"SampleID","Index","OlinkID","UniProt","Assay","MissingFreq","Panel","Panel_Lot_Nr","PlateID","QC_Warning","LOD","NPX","Normalization","Assay_Warning","condition","Value","group","alt_group"
1233,"PFC_21",51,"OID30143","P12270","TPR",0,"Cardiometabolic_II","B13805","20212097_SS211647","PASS",-1.2818,-0.3655,"Plate control","PASS","PFC",0.776199818022005,"24h","24h"
1234,"PFC_06",61,"OID30143","P12270","TPR",0,"Cardiometabolic_II","B13805","20212097_SS211647","PASS",-1.2818,0.2847,"Plate control","PASS","PFC",1.21815692872416,"Baseline","Baseline"
1235,"PFC_14",62,"OID30143","P12270","TPR",0,"Cardiometabolic_II","B13805","20212097_SS211647","PASS",-1.2818,0.6044,"Plate control","PASS","PFC",1.52034632747866,"1h","1h"
1236,"PFC_22",63,"OID30143","P12270","TPR",0,"Cardiometabolic_II","B13805","20212097_SS211647","PASS",-1.2818,0.3307,"Plate control","PASS","PFC",1.25762342920358,"24h","24h"
1237,"PFC_07",73,"OID30143","P12270","TPR",0,"Cardiometabolic_II","B13805","20212097_SS211647","PASS",-1.2818,-0.2747,"Plate control","PASS","PFC",0.826622191535995,"Baseline","Baseline"
1238,"PFC_15",74,"OID30143","P12270","TPR",0,"Cardiometabolic_II","B13805","20212097_SS211647","PASS",-1.2818,-0.1317,"Plate control","PASS","PFC",0.912755270933475,"1h","1h"
1239,"PFC_23",75,"OID30143","P12270","TPR",0,"Cardiometabolic_II","B13805","20212097_SS211647","PASS",-1.2818,0.3203,"Plate control","PASS","PFC",1.24859015893298,"24h","24h"
1240,"PFC_08",85,"OID30143","P12270","TPR",0,"Cardiometabolic_II","B13805","20212097_SS211647","PASS",-1.2818,-0.3526,"Plate control","PASS","PFC",0.783171407378327,"Baseline","Baseline"
1241,"PFC_16",86,"OID30143","P12270","TPR",0,"Cardiometabolic_II","B13805","20212097_SS211647","PASS",-1.2818,-0.0172,"Plate control","PASS","PFC",0.988148655514205,"1h","1h"
1242,"PFC_24",87,"OID30143","P12270","TPR",0,"Cardiometabolic_II","B13805","20212097_SS211647","PASS",-1.2818,0.6585,"Plate control","PASS","PFC",1.5784406327572,"24h","24h"
1243,"PFC_01",1,"OID30144","Q9NYX4","CALY",0.9318,"Cardiometabolic_II","B13805","20212097_SS211647","WARN",1.2384,0.2735,"Plate control","PASS","PFC",1.208736687019,"Baseline","Baseline"
1244,"PFC_09",2,"OID30144","Q9NYX4","CALY",0.9318,"Cardiometabolic_II","B13805","20212097_SS211647","PASS",1.2384,-0.2143,"Plate control","PASS","PFC",0.86196428594594,"Baseline","Baseline"
1245,"PFC_17",3,"OID30144","Q9NYX4","CALY",0.9318,"Cardiometabolic_II","B13805","20212097_SS211647","PASS",1.2384,-0.1855,"Plate control","PASS","PFC",0.87934426560666,"1h","1h"
1246,"PFC_25",4,"OID30144","Q9NYX4","CALY",0.9318,"Cardiometabolic_II","B13805","20212097_SS211647","PASS",1.2384,-0.1046,"Plate control","PASS","PFC",0.930062775024449,"24h","24h"
1247,"PFC_02",13,"OID30144","Q9NYX4","CALY",0.9318,"Cardiometabolic_II","B13805","20212097_SS211647","PASS",1.2384,0.0386,"Plate control","PASS","PFC",1.02711662270036,"Baseline","Baseline"
1248,"PFC_10",14,"OID30144","Q9NYX4","CALY",0.9318,"Cardiometabolic_II","B13805","20212097_SS211647","PASS",1.2384,0.3054,"Plate control","PASS","PFC",1.23576121539422,"1h","1h"
1249,"PFC_18",15,"OID30144","Q9NYX4","CALY",0.9318,"Cardiometabolic_II","B13805","20212097_SS211647","PASS",1.2384,0.4112,"Plate control","PASS","PFC",1.32979144364224,"1h","1h"
1250,"PFC_26",16,"OID30144","Q9NYX4","CALY",0.9318,"Cardiometabolic_II","B13805","20212097_SS211647","PASS",1.2384,-1.0796,"Plate control","PASS","PFC",0.473159992984023,"24h","24h"
1251,"PFC_03",25,"OID30144","Q9NYX4","CALY",0.9318,"Cardiometabolic_II","B13805","20212097_SS211647","PASS",1.2384,0.1455,"Plate control","PASS","PFC",1.1061139367768,"Baseline","Baseline"
1252,"PFC_11",26,"OID30144","Q9NYX4","CALY",0.9318,"Cardiometabolic_II","B13805","20212097_SS211647","PASS",1.2384,-0.2292,"Plate control","PASS","PFC",0.853107824057532,"1h","1h"
1253,"PFC_19",27,"OID30144","Q9NYX4","CALY",0.9318,"Cardiometabolic_II","B13805","20212097_SS211647","PASS",1.2384,0.2251,"Plate control","PASS","PFC",1.16885826483448,"24h","24h"
1254,"PFC_27",28,"OID30144","Q9NYX4","CALY",0.9318,"Cardiometabolic_II","B13805","20212097_SS211647","PASS",1.2384,0.4154,"Plate control","PASS","PFC",1.33366839723047,"24h","24h"
1255,"PFC_04",37,"OID30144","Q9NYX4","CALY",0.9318,"Cardiometabolic_II","B13805","20212097_SS211647","PASS",1.2384,-0.2361,"Plate control","PASS","PFC",0.849037393619622,"Baseline","Baseline"
1256,"PFC_12",38,"OID30144","Q9NYX4","CALY",0.9318,"Cardiometabolic_II","B13805","20212097_SS211647","PASS",1.2384,-0.3325,"Plate control","PASS","PFC",0.79415911781936,"1h","1h"
1257,"PFC_20",39,"OID30144","Q9NYX4","CALY",0.9318,"Cardiometabolic_II","B13805","20212097_SS211647","PASS",1.2384,0.3725,"Plate control","PASS","PFC",1.29459424891967,"24h","24h"
1258,"PFC_05",49,"OID30144","Q9NYX4","CALY",0.9318,"Cardiometabolic_II","B13805","20212097_SS211647","PASS",1.2384,0.3138,"Plate control","PASS","PFC",1.24297734371128,"Baseline","Baseline"
1259,"PFC_13",50,"OID30144","Q9NYX4","CALY",0.9318,"Cardiometabolic_II","B13805","20212097_SS211647","PASS",1.2384,0.0462,"Plate control","PASS","PFC",1.0325416662309,"1h","1h"
1260,"PFC_21",51,"OID30144","Q9NYX4","CALY",0.9318,"Cardiometabolic_II","B13805","20212097_SS211647","PASS",1.2384,0.0851,"Plate control","PASS","PFC",1.06076126518807,"24h","24h"
1261,"PFC_06",61,"OID30144","Q9NYX4","CALY",0.9318,"Cardiometabolic_II","B13805","20212097_SS211647","PASS",1.2384,0.507,"Plate control","PASS","PFC",1.42109204318136,"Baseline","Baseline"
1262,"PFC_14",62,"OID30144","Q9NYX4","CALY",0.9318,"Cardiometabolic_II","B13805","20212097_SS211647","PASS",1.2384,0.2017,"Plate control","PASS","PFC",1.15005272174383,"1h","1h"
1263,"PFC_22",63,"OID30144","Q9NYX4","CALY",0.9318,"Cardiometabolic_II","B13805","20212097_SS211647","PASS",1.2384,0.339,"Plate control","PASS","PFC",1.26487954235394,"24h","24h"
1264,"PFC_07",73,"OID30144","Q9NYX4","CALY",0.9318,"Cardiometabolic_II","B13805","20212097_SS211647","PASS",1.2384,-0.1746,"Plate control","PASS","PFC",0.88601314005448,"Baseline","Baseline"
1265,"PFC_15",74,"OID30144","Q9NYX4","CALY",0.9318,"Cardiometabolic_II","B13805","20212097_SS211647","PASS",1.2384,0.1935,"Plate control","PASS","PFC",1.14353458563209,"1h","1h"
1266,"PFC_23",75,"OID30144","Q9NYX4","CALY",0.9318,"Cardiometabolic_II","B13805","20212097_SS211647","PASS",1.2384,0.0744,"Plate control","PASS","PFC",1.05292304649919,"24h","24h"
1267,"PFC_08",85,"OID30144","Q9NYX4","CALY",0.9318,"Cardiometabolic_II","B13805","20212097_SS211647","PASS",1.2384,0.4488,"Plate control","PASS","PFC",1.36490448882948,"Baseline","Baseline"
1268,"PFC_16",86,"OID30144","Q9NYX4","CALY",0.9318,"Cardiometabolic_II","B13805","20212097_SS211647","PASS",1.2384,0.3337,"Plate control","PASS","PFC",1.26024130452208,"1h","1h"
1269,"PFC_24",87,"OID30144","Q9NYX4","CALY",0.9318,"Cardiometabolic_II","B13805","20212097_SS211647","PASS",1.2384,-0.2576,"Plate control","PASS","PFC",0.836478288792943,"24h","24h"
1270,"PFC_01",1,"OID30066","Q13503","MED21",0.9773,"Cardiometabolic_II","B13805","20212097_SS211647","WARN",0.3722,-0.8353,"Plate control","PASS","PFC",0.560466481167547,"Baseline","Baseline"
1271,"PFC_09",2,"OID30066","Q13503","MED21",0.9773,"Cardiometabolic_II","B13805","20212097_SS211647","PASS",0.3722,-0.786,"Plate control","PASS","PFC",0.579949827252717,"Baseline","Baseline"
1272,"PFC_17",3,"OID30066","Q13503","MED21",0.9773,"Cardiometabolic_II","B13805","20212097_SS211647","PASS",0.3722,-0.8989,"Plate control","PASS","PFC",0.536295480290688,"1h","1h"
1273,"PFC_25",4,"OID30066","Q13503","MED21",0.9773,"Cardiometabolic_II","B13805","20212097_SS211647","PASS",0.3722,-0.2319,"Plate control","PASS","PFC",0.8515127280681,"24h","24h"
1274,"PFC_02",13,"OID30066","Q13503","MED21",0.9773,"Cardiometabolic_II","B13805","20212097_SS211647","PASS",0.3722,-0.4319,"Plate control","PASS","PFC",0.741284885073504,"Baseline","Baseline"
1275,"PFC_10",14,"OID30066","Q13503","MED21",0.9773,"Cardiometabolic_II","B13805","20212097_SS211647","PASS",0.3722,0.0089,"Plate control","PASS","PFC",1.0061880774377,"1h","1h"
1276,"PFC_18",15,"OID30066","Q13503","MED21",0.9773,"Cardiometabolic_II","B13805","20212097_SS211647","PASS",0.3722,-0.3244,"Plate control","PASS","PFC",0.798630458369747,"1h","1h"
1277,"PFC_26",16,"OID30066","Q13503","MED21",0.9773,"Cardiometabolic_II","B13805","20212097_SS211647","PASS",0.3722,-0.6412,"Plate control","PASS","PFC",0.641179408879808,"24h","24h"
1278,"PFC_03",25,"OID30066","Q13503","MED21",0.9773,"Cardiometabolic_II","B13805","20212097_SS211647","PASS",0.3722,-0.5609,"Plate control","PASS","PFC",0.677879148773666,"Baseline","Baseline"
1279,"PFC_11",26,"OID30066","Q13503","MED21",0.9773,"Cardiometabolic_II","B13805","20212097_SS211647","PASS",0.3722,-0.4762,"Plate control","PASS","PFC",0.718868603166412,"1h","1h"
1280,"PFC_19",27,"OID30066","Q13503","MED21",0.9773,"Cardiometabolic_II","B13805","20212097_SS211647","PASS",0.3722,-0.9301,"Plate control","PASS","PFC",0.524821962664371,"24h","24h"
1281,"PFC_27",28,"OID30066","Q13503","MED21",0.9773,"Cardiometabolic_II","B13805","20212097_SS211647","PASS",0.3722,-0.8594,"Plate control","PASS","PFC",0.551181740319433,"24h","24h"
1282,"PFC_04",37,"OID30066","Q13503","MED21",0.9773,"Cardiometabolic_II","B13805","20212097_SS211647","PASS",0.3722,-0.1093,"Plate control","PASS","PFC",0.927037754309458,"Baseline","Baseline"
1283,"PFC_12",38,"OID30066","Q13503","MED21",0.9773,"Cardiometabolic_II","B13805","20212097_SS211647","PASS",0.3722,-0.6731,"Plate control","PASS","PFC",0.627157629499598,"1h","1h"
1284,"PFC_20",39,"OID30066","Q13503","MED21",0.9773,"Cardiometabolic_II","B13805","20212097_SS211647","PASS",0.3722,-1.0268,"Plate control","PASS","PFC",0.490797566200702,"24h","24h"
1285,"PFC_05",49,"OID30066","Q13503","MED21",0.9773,"Cardiometabolic_II","B13805","20212097_SS211647","PASS",0.3722,-0.349,"Plate control","PASS","PFC",0.785128118673572,"Baseline","Baseline"
1286,"PFC_13",50,"OID30066","Q13503","MED21",0.9773,"Cardiometabolic_II","B13805","20212097_SS211647","PASS",0.3722,-0.2732,"Plate control","PASS","PFC",0.827482094750325,"1h","1h"
1287,"PFC_21",51,"OID30066","Q13503","MED21",0.9773,"Cardiometabolic_II","B13805","20212097_SS211647","PASS",0.3722,-0.4102,"Plate control","PASS","PFC",0.752519045183301,"24h","24h"
1288,"PFC_06",61,"OID30066","Q13503","MED21",0.9773,"Cardiometabolic_II","B13805","20212097_SS211647","PASS",0.3722,-0.7021,"Plate control","PASS","PFC",0.61467682599975,"Baseline","Baseline"
1289,"PFC_14",62,"OID30066","Q13503","MED21",0.9773,"Cardiometabolic_II","B13805","20212097_SS211647","PASS",0.3722,-0.2013,"Plate control","PASS","PFC",0.869766471049515,"1h","1h"
1290,"PFC_22",63,"OID30066","Q13503","MED21",0.9773,"Cardiometabolic_II","B13805","20212097_SS211647","PASS",0.3722,-0.411,"Plate control","PASS","PFC",0.752101875694344,"24h","24h"
1291,"PFC_07",73,"OID30066","Q13503","MED21",0.9773,"Cardiometabolic_II","B13805","20212097_SS211647","PASS",0.3722,0.0713,"Plate control","PASS","PFC",1.05066300052555,"Baseline","Baseline"
1292,"PFC_15",74,"OID30066","Q13503","MED21",0.9773,"Cardiometabolic_II","B13805","20212097_SS211647","PASS",0.3722,-0.6034,"Plate control","PASS","PFC",0.658200943677173,"1h","1h"
1293,"PFC_23",75,"OID30066","Q13503","MED21",0.9773,"Cardiometabolic_II","B13805","20212097_SS211647","PASS",0.3722,0.077,"Plate control","PASS","PFC",1.05482231706806,"24h","24h"
1294,"PFC_08",85,"OID30066","Q13503","MED21",0.9773,"Cardiometabolic_II","B13805","20212097_SS211647","PASS",0.3722,-0.8635,"Plate control","PASS","PFC",0.549617558714681,"Baseline","Baseline"
1295,"PFC_16",86,"OID30066","Q13503","MED21",0.9773,"Cardiometabolic_II","B13805","20212097_SS211647","PASS",0.3722,-0.3319,"Plate control","PASS","PFC",0.794489468001047,"1h","1h"
1296,"PFC_24",87,"OID30066","Q13503","MED21",0.9773,"Cardiometabolic_II","B13805","20212097_SS211647","PASS",0.3722,0.2293,"Plate control","PASS","PFC",1.17226602419829,"24h","24h"
1297,"PFC_01",1,"OID30067","P08913","ADRA2A",0.5682,"Cardiometabolic_II","B13805","20212097_SS211647","WARN",-0.0472,-1.0971,"Plate control","PASS","PFC",0.46745519622762,"Baseline","Baseline"
1298,"PFC_09",2,"OID30067","P08913","ADRA2A",0.5682,"Cardiometabolic_II","B13805","20212097_SS211647","PASS",-0.0472,-0.0115,"Plate control","PASS","PFC",0.992060493132249,"Baseline","Baseline"
1299,"PFC_17",3,"OID30067","P08913","ADRA2A",0.5682,"Cardiometabolic_II","B13805","20212097_SS211647","PASS",-0.0472,-0.351,"Plate control","PASS","PFC",0.784040454075739,"1h","1h"
1300,"PFC_25",4,"OID30067","P08913","ADRA2A",0.5682,"Cardiometabolic_II","B13805","20212097_SS211647","PASS",-0.0472,0.9387,"Plate control","PASS","PFC",1.91680024813481,"24h","24h"
1301,"PFC_02",13,"OID30067","P08913","ADRA2A",0.5682,"Cardiometabolic_II","B13805","20212097_SS211647","PASS",-0.0472,-0.2407,"Plate control","PASS","PFC",0.846334568629495,"Baseline","Baseline"
1302,"PFC_10",14,"OID30067","P08913","ADRA2A",0.5682,"Cardiometabolic_II","B13805","20212097_SS211647","PASS",-0.0472,-1.039,"Plate control","PASS","PFC",0.486664687079119,"1h","1h"
1303,"PFC_18",15,"OID30067","P08913","ADRA2A",0.5682,"Cardiometabolic_II","B13805","20212097_SS211647","PASS",-0.0472,0.0319,"Plate control","PASS","PFC",1.02235766372068,"1h","1h"
1304,"PFC_26",16,"OID30067","P08913","ADRA2A",0.5682,"Cardiometabolic_II","B13805","20212097_SS211647","PASS",-0.0472,-0.4921,"Plate control","PASS","PFC",0.710989421554317,"24h","24h"
1305,"PFC_03",25,"OID30067","P08913","ADRA2A",0.5682,"Cardiometabolic_II","B13805","20212097_SS211647","PASS",-0.0472,0.6953,"Plate control","PASS","PFC",1.61922110565228,"Baseline","Baseline"
1306,"PFC_11",26,"OID30067","P08913","ADRA2A",0.5682,"Cardiometabolic_II","B13805","20212097_SS211647","PASS",-0.0472,-0.1116,"Plate control","PASS","PFC",0.925561012468545,"1h","1h"
1307,"PFC_19",27,"OID30067","P08913","ADRA2A",0.5682,"Cardiometabolic_II","B13805","20212097_SS211647","PASS",-0.0472,-0.5503,"Plate control","PASS","PFC",0.682878113100458,"24h","24h"
1308,"PFC_27",28,"OID30067","P08913","ADRA2A",0.5682,"Cardiometabolic_II","B13805","20212097_SS211647","PASS",-0.0472,0.4837,"Plate control","PASS","PFC",1.39832527893665,"24h","24h"
1309,"PFC_04",37,"OID30067","P08913","ADRA2A",0.5682,"Cardiometabolic_II","B13805","20212097_SS211647","PASS",-0.0472,0.3441,"Plate control","PASS","PFC",1.2693588682226,"Baseline","Baseline"
1310,"PFC_12",38,"OID30067","P08913","ADRA2A",0.5682,"Cardiometabolic_II","B13805","20212097_SS211647","PASS",-0.0472,0.4396,"Plate control","PASS","PFC",1.35622824899974,"1h","1h"
1311,"PFC_20",39,"OID30067","P08913","ADRA2A",0.5682,"Cardiometabolic_II","B13805","20212097_SS211647","PASS",-0.0472,0.0687,"Plate control","PASS","PFC",1.04877121905447,"24h","24h"
1312,"PFC_05",49,"OID30067","P08913","ADRA2A",0.5682,"Cardiometabolic_II","B13805","20212097_SS211647","PASS",-0.0472,-0.0984,"Plate control","PASS","PFC",0.934068332245408,"Baseline","Baseline"
1313,"PFC_13",50,"OID30067","P08913","ADRA2A",0.5682,"Cardiometabolic_II","B13805","20212097_SS211647","PASS",-0.0472,0.685,"Plate control","PASS","PFC",1.6077019814863,"1h","1h"
1314,"PFC_21",51,"OID30067","P08913","ADRA2A",0.5682,"Cardiometabolic_II","B13805","20212097_SS211647","PASS",-0.0472,0.7347,"Plate control","PASS","PFC",1.66405140368149,"24h","24h"
1315,"PFC_06",61,"OID30067","P08913","ADRA2A",0.5682,"Cardiometabolic_II","B13805","20212097_SS211647","PASS",-0.0472,-0.2896,"Plate control","PASS","PFC",0.818128860600113,"Baseline","Baseline"
1316,"PFC_14",62,"OID30067","P08913","ADRA2A",0.5682,"Cardiometabolic_II","B13805","20212097_SS211647","PASS",-0.0472,-0.0886,"Plate control","PASS","PFC",0.94043491028831,"1h","1h"
1317,"PFC_22",63,"OID30067","P08913","ADRA2A",0.5682,"Cardiometabolic_II","B13805","20212097_SS211647","PASS",-0.0472,0.8064,"Plate control","PASS","PFC",1.74884205558407,"24h","24h"
1318,"PFC_07",73,"OID30067","P08913","ADRA2A",0.5682,"Cardiometabolic_II","B13805","20212097_SS211647","PASS",-0.0472,0.862,"Plate control","PASS","PFC",1.81755623289704,"Baseline","Baseline"
1319,"PFC_15",74,"OID30067","P08913","ADRA2A",0.5682,"Cardiometabolic_II","B13805","20212097_SS211647","PASS",-0.0472,-0.6729,"Plate control","PASS","PFC",0.627244578034803,"1h","1h"
1320,"PFC_23",75,"OID30067","P08913","ADRA2A",0.5682,"Cardiometabolic_II","B13805","20212097_SS211647","PASS",-0.0472,0.2492,"Plate control","PASS","PFC",1.18854786135644,"24h","24h"
1321,"PFC_08",85,"OID30067","P08913","ADRA2A",0.5682,"Cardiometabolic_II","B13805","20212097_SS211647","PASS",-0.0472,-0.4786,"Plate control","PASS","PFC",0.717673721127765,"Baseline","Baseline"
1322,"PFC_16",86,"OID30067","P08913","ADRA2A",0.5682,"Cardiometabolic_II","B13805","20212097_SS211647","PASS",-0.0472,0.6376,"Plate control","PASS","PFC",1.55573895088156,"1h","1h"
1323,"PFC_24",87,"OID30067","P08913","ADRA2A",0.5682,"Cardiometabolic_II","B13805","20212097_SS211647","PASS",-0.0472,-0.3665,"Plate control","PASS","PFC",0.775661983727288,"24h","24h"
1324,"PFC_01",1,"OID30068","P49755","TMED10",0.9318,"Cardiometabolic_II","B13805","20212097_SS211647","WARN",0.3178,-0.4814,"Plate control","PASS","PFC",0.716282202057273,"Baseline","Baseline"
1325,"PFC_09",2,"OID30068","P49755","TMED10",0.9318,"Cardiometabolic_II","B13805","20212097_SS211647","PASS",0.3178,-0.388,"Plate control","PASS","PFC",0.764188260382583,"Baseline","Baseline"
1326,"PFC_17",3,"OID30068","P49755","TMED10",0.9318,"Cardiometabolic_II","B13805","20212097_SS211647","PASS",0.3178,-1.1544,"Plate control","PASS","PFC",0.449252986659898,"1h","1h"
1327,"PFC_25",4,"OID30068","P49755","TMED10",0.9318,"Cardiometabolic_II","B13805","20212097_SS211647","PASS",0.3178,-0.593,"Plate control","PASS","PFC",0.662962880149329,"24h","24h"
1328,"PFC_02",13,"OID30068","P49755","TMED10",0.9318,"Cardiometabolic_II","B13805","20212097_SS211647","PASS",0.3178,-0.2519,"Plate control","PASS","PFC",0.839789700713121,"Baseline","Baseline"
1329,"PFC_10",14,"OID30068","P49755","TMED10",0.9318,"Cardiometabolic_II","B13805","20212097_SS211647","PASS",0.3178,-0.4709,"Plate control","PASS","PFC",0.721514353261555,"1h","1h"
1330,"PFC_18",15,"OID30068","P49755","TMED10",0.9318,"Cardiometabolic_II","B13805","20212097_SS211647","PASS",0.3178,-1.0686,"Plate control","PASS","PFC",0.476781446206159,"1h","1h"
1331,"PFC_26",16,"OID30068","P49755","TMED10",0.9318,"Cardiometabolic_II","B13805","20212097_SS211647","PASS",0.3178,-0.2657,"Plate control","PASS","PFC",0.831795047309977,"24h","24h"
1332,"PFC_03",25,"OID30068","P49755","TMED10",0.9318,"Cardiometabolic_II","B13805","20212097_SS211647","PASS",0.3178,-0.4239,"Plate control","PASS","PFC",0.745406859275124,"Baseline","Baseline"
1333,"PFC_11",26,"OID30068","P49755","TMED10",0.9318,"Cardiometabolic_II","B13805","20212097_SS211647","PASS",0.3178,-0.7246,"Plate control","PASS","PFC",0.605164808663895,"1h","1h"
1334,"PFC_19",27,"OID30068","P49755","TMED10",0.9318,"Cardiometabolic_II","B13805","20212097_SS211647","PASS",0.3178,0.2422,"Plate control","PASS","PFC",1.18279495907733,"24h","24h"
1335,"PFC_27",28,"OID30068","P49755","TMED10",0.9318,"Cardiometabolic_II","B13805","20212097_SS211647","PASS",0.3178,-0.7172,"Plate control","PASS","PFC",0.608276848395302,"24h","24h"
1336,"PFC_04",37,"OID30068","P49755","TMED10",0.9318,"Cardiometabolic_II","B13805","20212097_SS211647","PASS",0.3178,0.4675,"Plate control","PASS","PFC",1.38271133472879,"Baseline","Baseline"
1337,"PFC_12",38,"OID30068","P49755","TMED10",0.9318,"Cardiometabolic_II","B13805","20212097_SS211647","PASS",0.3178,-0.6077,"Plate control","PASS","PFC",0.656242074811933,"1h","1h"
1338,"PFC_20",39,"OID30068","P49755","TMED10",0.9318,"Cardiometabolic_II","B13805","20212097_SS211647","PASS",0.3178,0.1836,"Plate control","PASS","PFC",1.13571433425458,"24h","24h"
1339,"PFC_05",49,"OID30068","P49755","TMED10",0.9318,"Cardiometabolic_II","B13805","20212097_SS211647","PASS",0.3178,-0.6471,"Plate control","PASS","PFC",0.638562616275955,"Baseline","Baseline"
1340,"PFC_13",50,"OID30068","P49755","TMED10",0.9318,"Cardiometabolic_II","B13805","20212097_SS211647","PASS",0.3178,-0.1326,"Plate control","PASS","PFC",0.912186042135307,"1h","1h"
1341,"PFC_21",51,"OID30068","P49755","TMED10",0.9318,"Cardiometabolic_II","B13805","20212097_SS211647","PASS",0.3178,-0.2758,"Plate control","PASS","PFC",0.825992163826982,"24h","24h"
1342,"PFC_06",61,"OID30068","P49755","TMED10",0.9318,"Cardiometabolic_II","B13805","20212097_SS211647","PASS",0.3178,-0.4268,"Plate control","PASS","PFC",0.743910001893387,"Baseline","Baseline"
1343,"PFC_14",62,"OID30068","P49755","TMED10",0.9318,"Cardiometabolic_II","B13805","20212097_SS211647","PASS",0.3178,-0.3268,"Plate control","PASS","PFC",0.797302998544656,"1h","1h"
1344,"PFC_22",63,"OID30068","P49755","TMED10",0.9318,"Cardiometabolic_II","B13805","20212097_SS211647","PASS",0.3178,0.0785,"Plate control","PASS","PFC",1.05591960807994,"24h","24h"

Supplemental table 1_Olink proximity extension assay (PEA) data

,"SampleID","Index","OlinkID","UniProt","Assay","MissingFreq","Panel","Panel_Lot_Nr","PlateID","QC_Warning","LOD","NPX","Normalization","Assay_Warning","condition","Value","group","alt_group"
1345,"PFC_07",73,"OID30068","P49755","TMED10",0.9318,"Cardiometabolic_II","B13805","20212097_SS211647","PASS",0.3178,0.178,"Plate control","PASS","PFC",1.1313144628459,"Baseline","Baseline"
1346,"PFC_15",74,"OID30068","P49755","TMED10",0.9318,"Cardiometabolic_II","B13805","20212097_SS211647","PASS",0.3178,-0.6972,"Plate control","PASS","PFC",0.616768076767219,"1h","1h"
1347,"PFC_23",75,"OID30068","P49755","TMED10",0.9318,"Cardiometabolic_II","B13805","20212097_SS211647","PASS",0.3178,-0.4099,"Plate control","PASS","PFC",0.752675543390527,"24h","24h"
1348,"PFC_08",85,"OID30068","P49755","TMED10",0.9318,"Cardiometabolic_II","B13805","20212097_SS211647","PASS",0.3178,-0.4876,"Plate control","PASS","PFC",0.713210575231217,"Baseline","Baseline"
1349,"PFC_16",86,"OID30068","P49755","TMED10",0.9318,"Cardiometabolic_II","B13805","20212097_SS211647","PASS",0.3178,0.2203,"Plate control","PASS","PFC",1.16497581119266,"1h","1h"
1350,"PFC_24",87,"OID30068","P49755","TMED10",0.9318,"Cardiometabolic_II","B13805","20212097_SS211647","PASS",0.3178,-0.7027,"Plate control","PASS","PFC",0.614421242245243,"24h","24h"
1351,"PFC_01",1,"OID30069","Q96DA2","RAB39B",0.2727,"Cardiometabolic_II","B13805","20212097_SS211647","WARN",-0.1959,-0.4766,"Plate control","PASS","PFC",0.718669318096274,"Baseline","Baseline"
1352,"PFC_09",2,"OID30069","Q96DA2","RAB39B",0.2727,"Cardiometabolic_II","B13805","20212097_SS211647","PASS",-0.1959,-0.2565,"Plate control","PASS","PFC",0.837116314821526,"Baseline","Baseline"
1353,"PFC_17",3,"OID30069","Q96DA2","RAB39B",0.2727,"Cardiometabolic_II","B13805","20212097_SS211647","PASS",-0.1959,0.6116,"Plate control","PASS","PFC",1.52795282355431,"1h","1h"
1354,"PFC_25",4,"OID30069","Q96DA2","RAB39B",0.2727,"Cardiometabolic_II","B13805","20212097_SS211647","PASS",-0.1959,0.1851,"Plate control","PASS","PFC",1.13689577411499,"24h","24h"
1355,"PFC_02",13,"OID30069","Q96DA2","RAB39B",0.2727,"Cardiometabolic_II","B13805","20212097_SS211647","PASS",-0.1959,0.1505,"Plate control","PASS","PFC",1.10995408618814,"Baseline","Baseline"
1356,"PFC_10",14,"OID30069","Q96DA2","RAB39B",0.2727,"Cardiometabolic_II","B13805","20212097_SS211647","PASS",-0.1959,-0.4531,"Plate control","PASS","PFC",0.730471555088122,"1h","1h"
1357,"PFC_18",15,"OID30069","Q96DA2","RAB39B",0.2727,"Cardiometabolic_II","B13805","20212097_SS211647","PASS",-0.1959,0.0225,"Plate control","PASS","PFC",1.01571806093096,"1h","1h"
1358,"PFC_26",16,"OID30069","Q96DA2","RAB39B",0.2727,"Cardiometabolic_II","B13805","20212097_SS211647","PASS",-0.1959,1.0912,"Plate control","PASS","PFC",2.13051173795643,"24h","24h"
1359,"PFC_03",25,"OID30069","Q96DA2","RAB39B",0.2727,"Cardiometabolic_II","B13805","20212097_SS211647","PASS",-0.1959,-0.1328,"Plate control","PASS","PFC",0.912059595063503,"Baseline","Baseline"
1360,"PFC_11",26,"OID30069","Q96DA2","RAB39B",0.2727,"Cardiometabolic_II","B13805","20212097_SS211647","PASS",-0.1959,-0.2135,"Plate control","PASS","PFC",0.862442392984745,"1h","1h"
1361,"PFC_19",27,"OID30069","Q96DA2","RAB39B",0.2727,"Cardiometabolic_II","B13805","20212097_SS211647","PASS",-0.1959,0.34,"Plate control","PASS","PFC",1.26575659397028,"24h","24h"
1362,"PFC_27",28,"OID30069","Q96DA2","RAB39B",0.2727,"Cardiometabolic_II","B13805","20212097_SS211647","PASS",-0.1959,0.4548,"Plate control","PASS","PFC",1.37059278730374,"24h","24h"
1363,"PFC_04",37,"OID30069","Q96DA2","RAB39B",0.2727,"Cardiometabolic_II","B13805","20212097_SS211647","PASS",-0.1959,0.0553,"Plate control","PASS","PFC",1.03907515043729,"Baseline","Baseline"
1364,"PFC_12",38,"OID30069","Q96DA2","RAB39B",0.2727,"Cardiometabolic_II","B13805","20212097_SS211647","PASS",-0.1959,-0.7813,"Plate control","PASS","PFC",0.581842263923111,"1h","1h"
1365,"PFC_20",39,"OID30069","Q96DA2","RAB39B",0.2727,"Cardiometabolic_II","B13805","20212097_SS211647","PASS",-0.1959,0.7061,"Plate control","PASS","PFC",1.63138806196264,"24h","24h"
1366,"PFC_05",49,"OID30069","Q96DA2","RAB39B",0.2727,"Cardiometabolic_II","B13805","20212097_SS211647","PASS",-0.1959,-0.8327,"Plate control","PASS","PFC",0.56147745485272,"Baseline","Baseline"
1367,"PFC_13",50,"OID30069","Q96DA2","RAB39B",0.2727,"Cardiometabolic_II","B13805","20212097_SS211647","PASS",-0.1959,-0.0427,"Plate control","PASS","PFC",0.970836328520315,"1h","1h"
1368,"PFC_21",51,"OID30069","Q96DA2","RAB39B",0.2727,"Cardiometabolic_II","B13805","20212097_SS211647","PASS",-0.1959,-0.2507,"Plate control","PASS","PFC",0.840488508734893,"24h","24h"
1369,"PFC_06",61,"OID30069","Q96DA2","RAB39B",0.2727,"Cardiometabolic_II","B13805","20212097_SS211647","PASS",-0.1959,-0.0611,"Plate control","PASS","PFC",0.958532995745926,"Baseline","Baseline"
1370,"PFC_14",62,"OID30069","Q96DA2","RAB39B",0.2727,"Cardiometabolic_II","B13805","20212097_SS211647","PASS",-0.1959,-0.1574,"Plate control","PASS","PFC",0.89663952393218,"1h","1h"
1371,"PFC_22",63,"OID30069","Q96DA2","RAB39B",0.2727,"Cardiometabolic_II","B13805","20212097_SS211647","PASS",-0.1959,-0.093,"Plate control","PASS","PFC",0.93757109645711,"24h","24h"
1372,"PFC_07",73,"OID30069","Q96DA2","RAB39B",0.2727,"Cardiometabolic_II","B13805","20212097_SS211647","PASS",-0.1959,-0.1298,"Plate control","PASS","PFC",0.913958142949514,"Baseline","Baseline"
1373,"PFC_15",74,"OID30069","Q96DA2","RAB39B",0.2727,"Cardiometabolic_II","B13805","20212097_SS211647","PASS",-0.1959,-0.4031,"Plate control","PASS","PFC",0.756231578846597,"1h","1h"
1374,"PFC_23",75,"OID30069","Q96DA2","RAB39B",0.2727,"Cardiometabolic_II","B13805","20212097_SS211647","PASS",-0.1959,-0.2861,"Plate control","PASS","PFC",0.820116063112857,"24h","24h"
1375,"PFC_08",85,"OID30069","Q96DA2","RAB39B",0.2727,"Cardiometabolic_II","B13805","20212097_SS211647","PASS",-0.1959,0.8155,"Plate control","PASS","PFC",1.75990798379145,"Baseline","Baseline"
1376,"PFC_16",86,"OID30069","Q96DA2","RAB39B",0.2727,"Cardiometabolic_II","B13805","20212097_SS211647","PASS",-0.1959,-0.0841,"Plate control","PASS","PFC",0.943372859005332,"1h","1h"
1377,"PFC_24",87,"OID30069","Q96DA2","RAB39B",0.2727,"Cardiometabolic_II","B13805","20212097_SS211647","PASS",-0.1959,0.3919,"Plate control","PASS","PFC",1.31212030219103,"24h","24h"
1378,"PFC_01",1,"OID30070","P46783","RPS10",1,"Cardiometabolic_II","B13805","20212097_SS211647","WARN",1.6349,-0.1591,"Plate control","PASS","PFC",0.895583590815277,"Baseline","Baseline"
1379,"PFC_09",2,"OID30070","P46783","RPS10",1,"Cardiometabolic_II","B13805","20212097_SS211647","PASS",1.6349,-0.0418,"Plate control","PASS","PFC",0.971442156685815,"Baseline","Baseline"
1380,"PFC_17",3,"OID30070","P46783","RPS10",1,"Cardiometabolic_II","B13805","20212097_SS211647","PASS",1.6349,1.003,"Plate control","PASS","PFC",2.00416321015927,"1h","1h"
1381,"PFC_25",4,"OID30070","P46783","RPS10",1,"Cardiometabolic_II","B13805","20212097_SS211647","PASS",1.6349,0.8562,"Plate control","PASS","PFC",1.81026386423039,"24h","24h"
1382,"PFC_02",13,"OID30070","P46783","RPS10",1,"Cardiometabolic_II","B13805","20212097_SS211647","PASS",1.6349,0.397,"Plate control","PASS","PFC",1.31676692205924,"Baseline","Baseline"
1383,"PFC_10",14,"OID30070","P46783","RPS10",1,"Cardiometabolic_II","B13805","20212097_SS211647","PASS",1.6349,-0.0596,"Plate control","PASS","PFC",0.959530120687068,"1h","1h"
1384,"PFC_18",15,"OID30070","P46783","RPS10",1,"Cardiometabolic_II","B13805","20212097_SS211647","PASS",1.6349,0.5664,"Plate control","PASS","PFC",1.48082381262826,"1h","1h"
1385,"PFC_26",16,"OID30070","P46783","RPS10",1,"Cardiometabolic_II","B13805","20212097_SS211647","PASS",1.6349,0.654,"Plate control","PASS","PFC",1.57352489070713,"24h","24h"
1386,"PFC_03",25,"OID30070","P46783","RPS10",1,"Cardiometabolic_II","B13805","20212097_SS211647","PASS",1.6349,0.7642,"Plate control","PASS","PFC",1.69842792868017,"Baseline","Baseline"
1387,"PFC_11",26,"OID30070","P46783","RPS10",1,"Cardiometabolic_II","B13805","20212097_SS211647","PASS",1.6349,0.755,"Plate control","PASS","PFC",1.68763159226004,"1h","1h"
1388,"PFC_19",27,"OID30070","P46783","RPS10",1,"Cardiometabolic_II","B13805","20212097_SS211647","PASS",1.6349,0.3764,"Plate control","PASS","PFC",1.29809862641096,"24h","24h"
1389,"PFC_27",28,"OID30070","P46783","RPS10",1,"Cardiometabolic_II","B13805","20212097_SS211647","PASS",1.6349,0.5904,"Plate control","PASS","PFC",1.50566414828805,"24h","24h"
1390,"PFC_04",37,"OID30070","P46783","RPS10",1,"Cardiometabolic_II","B13805","20212097_SS211647","PASS",1.6349,0.7754,"Plate control","PASS","PFC",1.7116645597668,"Baseline","Baseline"
1391,"PFC_12",38,"OID30070","P46783","RPS10",1,"Cardiometabolic_II","B13805","20212097_SS211647","PASS",1.6349,0.3091,"Plate control","PASS","PFC",1.23893457120822,"1h","1h"
1392,"PFC_20",39,"OID30070","P46783","RPS10",1,"Cardiometabolic_II","B13805","20212097_SS211647","PASS",1.6349,0.6583,"Plate control","PASS","PFC",1.57822182958897,"24h","24h"
1393,"PFC_05",49,"OID30070","P46783","RPS10",1,"Cardiometabolic_II","B13805","20212097_SS211647","PASS",1.6349,0.5042,"Plate control","PASS","PFC",1.41833664526056,"Baseline","Baseline"
1394,"PFC_13",50,"OID30070","P46783","RPS10",1,"Cardiometabolic_II","B13805","20212097_SS211647","PASS",1.6349,-0.0482,"Plate control","PASS","PFC",0.96714224593598,"1h","1h"
1395,"PFC_21",51,"OID30070","P46783","RPS10",1,"Cardiometabolic_II","B13805","20212097_SS211647","PASS",1.6349,0.5894,"Plate control","PASS","PFC",1.50462086304567,"24h","24h"
1396,"PFC_06",61,"OID30070","P46783","RPS10",1,"Cardiometabolic_II","B13805","20212097_SS211647","PASS",1.6349,0.6245,"Plate control","PASS","PFC",1.54167642847449,"Baseline","Baseline"
1397,"PFC_14",62,"OID30070","P46783","RPS10",1,"Cardiometabolic_II","B13805","20212097_SS211647","PASS",1.6349,0.043,"Plate control","PASS","PFC",1.03025395362007,"1h","1h"
1398,"PFC_22",63,"OID30070","P46783","RPS10",1,"Cardiometabolic_II","B13805","20212097_SS211647","PASS",1.6349,0.4129,"Plate control","PASS","PFC",1.33135932724162,"24h","24h"
1399,"PFC_07",73,"OID30070","P46783","RPS10",1,"Cardiometabolic_II","B13805","20212097_SS211647","PASS",1.6349,0.3664,"Plate control","PASS","PFC",1.28913200422585,"Baseline","Baseline"
1400,"PFC_15",74,"OID30070","P46783","RPS10",1,"Cardiometabolic_II","B13805","20212097_SS211647","PASS",1.6349,-0.3423,"Plate control","PASS","PFC",0.788782801025113,"1h","1h"
1401,"PFC_23",75,"OID30070","P46783","RPS10",1,"Cardiometabolic_II","B13805","20212097_SS211647","PASS",1.6349,0.3565,"Plate control","PASS","PFC",1.28031606069557,"24h","24h"
1402,"PFC_08",85,"OID30070","P46783","RPS10",1,"Cardiometabolic_II","B13805","20212097_SS211647","PASS",1.6349,0.0106,"Plate control","PASS","PFC",1.00737441819214,"Baseline","Baseline"
1403,"PFC_16",86,"OID30070","P46783","RPS10",1,"Cardiometabolic_II","B13805","20212097_SS211647","PASS",1.6349,0.5873,"Plate control","PASS","PFC",1.50243231647726,"1h","1h"
1404,"PFC_24",87,"OID30070","P46783","RPS10",1,"Cardiometabolic_II","B13805","20212097_SS211647","PASS",1.6349,1.026,"Plate control","PASS","PFC",2.03637039952924,"24h","24h"
1405,"PFC_01",1,"OID30071","O00291","HIP1",0.3864,"Cardiometabolic_II","B13805","20212097_SS211647","WARN",0.6171,0.6091,"Plate control","PASS","PFC",1.52530737584288,"Baseline","Baseline"
1406,"PFC_09",2,"OID30071","O00291","HIP1",0.3864,"Cardiometabolic_II","B13805","20212097_SS211647","PASS",0.6171,0.5139,"Plate control","PASS","PFC",1.42790500142219,"Baseline","Baseline"
1407,"PFC_17",3,"OID30071","O00291","HIP1",0.3864,"Cardiometabolic_II","B13805","20212097_SS211647","PASS",0.6171,0.8278,"Plate control","PASS","PFC",1.77497659347808,"1h","1h"
1408,"PFC_25",4,"OID30071","O00291","HIP1",0.3864,"Cardiometabolic_II","B13805","20212097_SS211647","PASS",0.6171,0.3384,"Plate control","PASS","PFC",1.26435360311439,"24h","24h"
1409,"PFC_02",13,"OID30071","O00291","HIP1",0.3864,"Cardiometabolic_II","B13805","20212097_SS211647","PASS",0.6171,0.2414,"Plate control","PASS","PFC",1.18213926009964,"Baseline","Baseline"
1410,"PFC_10",14,"OID30071","O00291","HIP1",0.3864,"Cardiometabolic_II","B13805","20212097_SS211647","PASS",0.6171,0.8483,"Plate control","PASS","PFC",1.80037819878658,"1h","1h"
1411,"PFC_18",15,"OID30071","O00291","HIP1",0.3864,"Cardiometabolic_II","B13805","20212097_SS211647","PASS",0.6171,0.8854,"Plate control","PASS","PFC",1.84727672432425,"1h","1h"
1412,"PFC_26",16,"OID30071","O00291","HIP1",0.3864,"Cardiometabolic_II","B13805","20212097_SS211647","PASS",0.6171,0.578,"Plate control","PASS","PFC",1.49277838329695,"24h","24h"
1413,"PFC_03",25,"OID30071","O00291","HIP1",0.3864,"Cardiometabolic_II","B13805","20212097_SS211647","PASS",0.6171,0.3918,"Plate control","PASS","PFC",1.31202935609421,"Baseline","Baseline"
1414,"PFC_11",26,"OID30071","O00291","HIP1",0.3864,"Cardiometabolic_II","B13805","20212097_SS211647","PASS",0.6171,1.0602,"Plate control","PASS","PFC",2.08522057459858,"1h","1h"
1415,"PFC_19",27,"OID30071","O00291","HIP1",0.3864,"Cardiometabolic_II","B13805","20212097_SS211647","PASS",0.6171,1.1392,"Plate control","PASS","PFC",2.20258851863642,"24h","24h"
1416,"PFC_27",28,"OID30071","O00291","HIP1",0.3864,"Cardiometabolic_II","B13805","20212097_SS211647","PASS",0.6171,0.8727,"Plate control","PASS","PFC",1.83108656949681,"24h","24h"
1417,"PFC_04",37,"OID30071","O00291","HIP1",0.3864,"Cardiometabolic_II","B13805","20212097_SS211647","PASS",0.6171,0.6747,"Plate control","PASS","PFC",1.5962648042645,"Baseline","Baseline"
1418,"PFC_12",38,"OID30071","O00291","HIP1",0.3864,"Cardiometabolic_II","B13805","20212097_SS211647","PASS",0.6171,0.7159,"Plate control","PASS","PFC",1.64250754761073,"1h","1h"
1419,"PFC_20",39,"OID30071","O00291","HIP1",0.3864,"Cardiometabolic_II","B13805","20212097_SS211647","PASS",0.6171,0.888,"Plate control","PASS","PFC",1.8506088560757,"24h","24h"
1420,"PFC_05",49,"OID30071","O00291","HIP1",0.3864,"Cardiometabolic_II","B13805","20212097_SS211647","PASS",0.6171,0.5675,"Plate control","PASS","PFC",1.48195331491046,"Baseline","Baseline"
1421,"PFC_13",50,"OID30071","O00291","HIP1",0.3864,"Cardiometabolic_II","B13805","20212097_SS211647","PASS",0.6171,1.0977,"Plate control","PASS","PFC",2.14013232257393,"1h","1h"
1422,"PFC_21",51,"OID30071","O00291","HIP1",0.3864,"Cardiometabolic_II","B13805","20212097_SS211647","PASS",0.6171,0.3657,"Plate control","PASS","PFC",1.28850666519644,"24h","24h"
1423,"PFC_06",61,"OID30071","O00291","HIP1",0.3864,"Cardiometabolic_II","B13805","20212097_SS211647","PASS",0.6171,0.3799,"Plate control","PASS","PFC",1.30125165642422,"Baseline","Baseline"
1424,"PFC_14",62,"OID30071","O00291","HIP1",0.3864,"Cardiometabolic_II","B13805","20212097_SS211647","PASS",0.6171,0.4413,"Plate control","PASS","PFC",1.35782730277527,"1h","1h"
1425,"PFC_22",63,"OID30071","O00291","HIP1",0.3864,"Cardiometabolic_II","B13805","20212097_SS211647","PASS",0.6171,0.2986,"Plate control","PASS","PFC",1.2299502828438,"24h","24h"
1426,"PFC_07",73,"OID30071","O00291","HIP1",0.3864,"Cardiometabolic_II","B13805","20212097_SS211647","PASS",0.6171,0.0972,"Plate control","PASS","PFC",1.06969536887275,"Baseline","Baseline"
1427,"PFC_15",74,"OID30071","O00291","HIP1",0.3864,"Cardiometabolic_II","B13805","20212097_SS211647","PASS",0.6171,0.5966,"Plate control","PASS","PFC",1.51214868251893,"1h","1h"
1428,"PFC_23",75,"OID30071","O00291","HIP1",0.3864,"Cardiometabolic_II","B13805","20212097_SS211647","PASS",0.6171,0.761,"Plate control","PASS","PFC",1.69466486989224,"24h","24h"
1429,"PFC_08",85,"OID30071","O00291","HIP1",0.3864,"Cardiometabolic_II","B13805","20212097_SS211647","PASS",0.6171,0.691,"Plate control","PASS","PFC",1.61440214900953,"Baseline","Baseline"
1430,"PFC_16",86,"OID30071","O00291","HIP1",0.3864,"Cardiometabolic_II","B13805","20212097_SS211647","PASS",0.6171,0.8231,"Plate control","PASS","PFC",1.76920349825475,"1h","1h"
1431,"PFC_24",87,"OID30071","O00291","HIP1",0.3864,"Cardiometabolic_II","B13805","20212097_SS211647","PASS",0.6171,1.101,"Plate control","PASS","PFC",2.14503323363589,"24h","24h"
1432,"PFC_01",1,"OID30072","P04141","CSF2",0.0909,"Cardiometabolic_II","B13805","20212097_SS211647","WARN",-0.7868,0.1904,"Plate control","PASS","PFC",1.14108004658048,"Baseline","Baseline"
1433,"PFC_09",2,"OID30072","P04141","CSF2",0.0909,"Cardiometabolic_II","B13805","20212097_SS211647","PASS",-0.7868,-0.6076,"Plate control","PASS","PFC",0.656287563622829,"Baseline","Baseline"
1434,"PFC_17",3,"OID30072","P04141","CSF2",0.0909,"Cardiometabolic_II","B13805","20212097_SS211647","PASS",-0.7868,-0.4,"Plate control","PASS","PFC",0.757858283255199,"1h","1h"
1435,"PFC_25",4,"OID30072","P04141","CSF2",0.0909,"Cardiometabolic_II","B13805","20212097_SS211647","PASS",-0.7868,0.2469,"Plate control","PASS","PFC",1.18665454218079,"24h","24h"
1436,"PFC_02",13,"OID30072","P04141","CSF2",0.0909,"Cardiometabolic_II","B13805","20212097_SS211647","PASS",-0.7868,0.2182,"Plate control","PASS","PFC",1.16328129540035,"Baseline","Baseline"
1437,"PFC_10",14,"OID30072","P04141","CSF2",0.0909,"Cardiometabolic_II","B13805","20212097_SS211647","PASS",-0.7868,0.6224,"Plate control","PASS","PFC",1.53943398272619,"1h","1h"
1438,"PFC_18",15,"OID30072","P04141","CSF2",0.0909,"Cardiometabolic_II","B13805","20212097_SS211647","PASS",-0.7868,0.2644,"Plate control","PASS","PFC",1.20113640704791,"1h","1h"
1439,"PFC_26",16,"OID30072","P04141","CSF2",0.0909,"Cardiometabolic_II","B13805","20212097_SS211647","PASS",-0.7868,-0.3133,"Plate control","PASS","PFC",0.804798767020972,"24h","24h"
1440,"PFC_03",25,"OID30072","P04141","CSF2",0.0909,"Cardiometabolic_II","B13805","20212097_SS211647","PASS",-0.7868,-0.4333,"Plate control","PASS","PFC",0.740565886650825,"Baseline","Baseline"
1441,"PFC_11",26,"OID30072","P04141","CSF2",0.0909,"Cardiometabolic_II","B13805","20212097_SS211647","PASS",-0.7868,0.9536,"Plate control","PASS","PFC",1.93669933693294,"1h","1h"
1442,"PFC_19",27,"OID30072","P04141","CSF2",0.0909,"Cardiometabolic_II","B13805","20212097_SS211647","PASS",-0.7868,0.3836,"Plate control","PASS","PFC",1.30459318750479,"24h","24h"
1443,"PFC_27",28,"OID30072","P04141","CSF2",0.0909,"Cardiometabolic_II","B13805","20212097_SS211647","PASS",-0.7868,0.4345,"Plate control","PASS","PFC",1.35144237761881,"24h","24h"
1444,"PFC_04",37,"OID30072","P04141","CSF2",0.0909,"Cardiometabolic_II","B13805","20212097_SS211647","PASS",-0.7868,-0.1324,"Plate control","PASS","PFC",0.912312506737613,"Baseline","Baseline"
1445,"PFC_12",38,"OID30072","P04141","CSF2",0.0909,"Cardiometabolic_II","B13805","20212097_SS211647","PASS",-0.7868,-0.2202,"Plate control","PASS","PFC",0.858446422245061,"1h","1h"
1446,"PFC_20",39,"OID30072","P04141","CSF2",0.0909,"Cardiometabolic_II","B13805","20212097_SS211647","PASS",-0.7868,-0.3177,"Plate control","PASS","PFC",0.802347992581281,"24h","24h"
1447,"PFC_05",49,"OID30072","P04141","CSF2",0.0909,"Cardiometabolic_II","B13805","20212097_SS211647","PASS",-0.7868,0.4224,"Plate control","PASS","PFC",1.34015512081948,"Baseline","Baseline"
1448,"PFC_13",50,"OID30072","P04141","CSF2",0.0909,"Cardiometabolic_II","B13805","20212097_SS211647","PASS",-0.7868,0.0188,"Plate control","PASS","PFC",1.01311644266238,"1h","1h"
1449,"PFC_21",51,"OID30072","P04141","CSF2",0.0909,"Cardiometabolic_II","B13805","20212097_SS211647","PASS",-0.7868,-0.8447,"Plate control","PASS","PFC",0.556826585909797,"24h","24h"
1450,"PFC_06",61,"OID30072","P04141","CSF2",0.0909,"Cardiometabolic_II","B13805","20212097_SS211647","PASS",-0.7868,-0.9321,"Plate control","PASS","PFC",0.524094909008568,"Baseline","Baseline"
1451,"PFC_14",62,"OID30072","P04141","CSF2",0.0909,"Cardiometabolic_II","B13805","20212097_SS211647","PASS",-0.7868,-0.8406,"Plate control","PASS","PFC",0.558411284012882,"1h","1h"
1452,"PFC_22",63,"OID30072","P04141","CSF2",0.0909,"Cardiometabolic_II","B13805","20212097_SS211647","PASS",-0.7868,0.0101,"Plate control","PASS","PFC",1.0070253493158,"24h","24h"
1453,"PFC_07",73,"OID30072","P04141","CSF2",0.0909,"Cardiometabolic_II","B13805","20212097_SS211647","PASS",-0.7868,-0.3656,"Plate control","PASS","PFC",0.776146017815058,"Baseline","Baseline"
1454,"PFC_15",74,"OID30072","P04141","CSF2",0.0909,"Cardiometabolic_II","B13805","20212097_SS211647","PASS",-0.7868,-0.5328,"Plate control","PASS","PFC",0.691211918941258,"1h","1h"
1455,"PFC_23",75,"OID30072","P04141","CSF2",0.0909,"Cardiometabolic_II","B13805","20212097_SS211647","PASS",-0.7868,-0.0767,"Plate control","PASS","PFC",0.948224122292167,"24h","24h"
1456,"PFC_08",85,"OID30072","P04141","CSF2",0.0909,"Cardiometabolic_II","B13805","20212097_SS211647","PASS",-0.7868,-0.5877,"Plate control","PASS","PFC",0.665402872792637,"Baseline","Baseline"

Supplemental table 1_Olink proximity extension assay (PEA) data

,"SampleID","Index","OlinkID","UniProt","Assay","MissingFreq","Panel","Panel_Lot_Nr","PlateID","QC_Warning","LOD","NPX","Normalization","Assay_Warning","condition","Value","group","alt_group"
1457,"PFC_16",86,"OID30072","P04141","CSF2",0.0909,"Cardiometabolic_II","B13805","20212097_SS211647","PASS",-0.7868,0.2126,"Plate control","PASS","PFC",1.15877462681521,"1h","1h"
1458,"PFC_24",87,"OID30072","P04141","CSF2",0.0909,"Cardiometabolic_II","B13805","20212097_SS211647","PASS",-0.7868,-0.667,"Plate control","PASS","PFC",0.6298149899737,"24h","24h"
1459,"PFC_01",1,"OID30074","A6NCE7","MAP1LC3B2",0.9773,"Cardiometabolic_II","B13805","20212097_SS211647","WARN",1.3484,0.1009,"Plate control","PASS","PFC",1.07244227820745,"Baseline","Baseline"
1460,"PFC_09",2,"OID30074","A6NCE7","MAP1LC3B2",0.9773,"Cardiometabolic_II","B13805","20212097_SS211647","PASS",1.3484,0.6025,"Plate control","PASS","PFC",1.51834538020759,"Baseline","Baseline"
1461,"PFC_17",3,"OID30074","A6NCE7","MAP1LC3B2",0.9773,"Cardiometabolic_II","B13805","20212097_SS211647","PASS",1.3484,0.309,"Plate control","PASS","PFC",1.23884869778391,"1h","1h"
1462,"PFC_25",4,"OID30074","A6NCE7","MAP1LC3B2",0.9773,"Cardiometabolic_II","B13805","20212097_SS211647","PASS",1.3484,-0.3527,"Plate control","PASS","PFC",0.783117123954377,"24h","24h"
1463,"PFC_02",13,"OID30074","A6NCE7","MAP1LC3B2",0.9773,"Cardiometabolic_II","B13805","20212097_SS211647","PASS",1.3484,1.0995,"Plate control","PASS","PFC",2.14280415703816,"Baseline","Baseline"
1464,"PFC_10",14,"OID30074","A6NCE7","MAP1LC3B2",0.9773,"Cardiometabolic_II","B13805","20212097_SS211647","PASS",1.3484,-0.1699,"Plate control","PASS","PFC",0.888904293181696,"1h","1h"
1465,"PFC_18",15,"OID30074","A6NCE7","MAP1LC3B2",0.9773,"Cardiometabolic_II","B13805","20212097_SS211647","PASS",1.3484,0.2531,"Plate control","PASS","PFC",1.19176517857852,"1h","1h"
1466,"PFC_26",16,"OID30074","A6NCE7","MAP1LC3B2",0.9773,"Cardiometabolic_II","B13805","20212097_SS211647","PASS",1.3484,0.0838,"Plate control","PASS","PFC",1.05980585292529,"24h","24h"
1467,"PFC_03",25,"OID30074","A6NCE7","MAP1LC3B2",0.9773,"Cardiometabolic_II","B13805","20212097_SS211647","PASS",1.3484,0.6925,"Plate control","PASS","PFC",1.61608154935877,"Baseline","Baseline"
1468,"PFC_11",26,"OID30074","A6NCE7","MAP1LC3B2",0.9773,"Cardiometabolic_II","B13805","20212097_SS211647","PASS",1.3484,0.46,"Plate control","PASS","PFC",1.37554181813974,"1h","1h"
1469,"PFC_19",27,"OID30074","A6NCE7","MAP1LC3B2",0.9773,"Cardiometabolic_II","B13805","20212097_SS211647","PASS",1.3484,0.1548,"Plate control","PASS","PFC",1.11326727591604,"24h","24h"
1470,"PFC_27",28,"OID30074","A6NCE7","MAP1LC3B2",0.9773,"Cardiometabolic_II","B13805","20212097_SS211647","PASS",1.3484,-0.2508,"Plate control","PASS","PFC",0.84043025252991,"24h","24h"
1471,"PFC_04",37,"OID30074","A6NCE7","MAP1LC3B2",0.9773,"Cardiometabolic_II","B13805","20212097_SS211647","PASS",1.3484,0.0514,"Plate control","PASS","PFC",1.03627003879565,"Baseline","Baseline"
1472,"PFC_12",38,"OID30074","A6NCE7","MAP1LC3B2",0.9773,"Cardiometabolic_II","B13805","20212097_SS211647","PASS",1.3484,0.0376,"Plate control","PASS","PFC",1.02640492639287,"1h","1h"
1473,"PFC_20",39,"OID30074","A6NCE7","MAP1LC3B2",0.9773,"Cardiometabolic_II","B13805","20212097_SS211647","PASS",1.3484,0.7454,"Plate control","PASS","PFC",1.67643901249579,"24h","24h"
1474,"PFC_05",49,"OID30074","A6NCE7","MAP1LC3B2",0.9773,"Cardiometabolic_II","B13805","20212097_SS211647","PASS",1.3484,0.6068,"Plate control","PASS","PFC",1.5228776094049,"Baseline","Baseline"
1475,"PFC_13",50,"OID30074","A6NCE7","MAP1LC3B2",0.9773,"Cardiometabolic_II","B13805","20212097_SS211647","PASS",1.3484,-0.1775,"Plate control","PASS","PFC",0.884233930093498,"1h","1h"
1476,"PFC_21",51,"OID30074","A6NCE7","MAP1LC3B2",0.9773,"Cardiometabolic_II","B13805","20212097_SS211647","PASS",1.3484,0.4181,"Plate control","PASS","PFC",1.33616669119782,"24h","24h"
1477,"PFC_06",61,"OID30074","A6NCE7","MAP1LC3B2",0.9773,"Cardiometabolic_II","B13805","20212097_SS211647","PASS",1.3484,-0.1576,"Plate control","PASS","PFC",0.896515231916046,"Baseline","Baseline"
1478,"PFC_14",62,"OID30074","A6NCE7","MAP1LC3B2",0.9773,"Cardiometabolic_II","B13805","20212097_SS211647","PASS",1.3484,0.1356,"Plate control","PASS","PFC",1.0985495927277,"1h","1h"
1479,"PFC_22",63,"OID30074","A6NCE7","MAP1LC3B2",0.9773,"Cardiometabolic_II","B13805","20212097_SS211647","PASS",1.3484,-0.0092,"Plate control","PASS","PFC",0.99364333555883,"24h","24h"
1480,"PFC_07",73,"OID30074","A6NCE7","MAP1LC3B2",0.9773,"Cardiometabolic_II","B13805","20212097_SS211647","PASS",1.3484,-0.0584,"Plate control","PASS","PFC",0.960328567423076,"Baseline","Baseline"
1481,"PFC_15",74,"OID30074","A6NCE7","MAP1LC3B2",0.9773,"Cardiometabolic_II","B13805","20212097_SS211647","PASS",1.3484,0.1947,"Plate control","PASS","PFC",1.14448614664902,"1h","1h"
1482,"PFC_23",75,"OID30074","A6NCE7","MAP1LC3B2",0.9773,"Cardiometabolic_II","B13805","20212097_SS211647","PASS",1.3484,0.492,"Plate control","PASS","PFC",1.40639319991312,"24h","24h"
1483,"PFC_08",85,"OID30074","A6NCE7","MAP1LC3B2",0.9773,"Cardiometabolic_II","B13805","20212097_SS211647","PASS",1.3484,-0.1099,"Plate control","PASS","PFC",0.926652290306596,"Baseline","Baseline"
1484,"PFC_16",86,"OID30074","A6NCE7","MAP1LC3B2",0.9773,"Cardiometabolic_II","B13805","20212097_SS211647","PASS",1.3484,-0.9158,"Plate control","PASS","PFC",0.530049867119785,"1h","1h"
1485,"PFC_24",87,"OID30074","A6NCE7","MAP1LC3B2",0.9773,"Cardiometabolic_II","B13805","20212097_SS211647","PASS",1.3484,-0.3777,"Plate control","PASS","PFC",0.769663640495877,"24h","24h"
1486,"PFC_01",1,"OID30075","Q9Y3B8","REXO2",1,"Cardiometabolic_II","B13805","20212097_SS211647","WARN",0.6144,-0.1023,"Plate control","PASS","PFC",0.931546699472488,"Baseline","Baseline"
1487,"PFC_09",2,"OID30075","Q9Y3B8","REXO2",1,"Cardiometabolic_II","B13805","20212097_SS211647","PASS",0.6144,0.0718,"Plate control","PASS","PFC",1.05102719568038,"Baseline","Baseline"
1488,"PFC_17",3,"OID30075","Q9Y3B8","REXO2",1,"Cardiometabolic_II","B13805","20212097_SS211647","PASS",0.6144,0.0878,"Plate control","PASS","PFC",1.06274833594353,"1h","1h"
1489,"PFC_25",4,"OID30075","Q9Y3B8","REXO2",1,"Cardiometabolic_II","B13805","20212097_SS211647","PASS",0.6144,0.1193,"Plate control","PASS","PFC",1.08620770338182,"24h","24h"
1490,"PFC_02",13,"OID30075","Q9Y3B8","REXO2",1,"Cardiometabolic_II","B13805","20212097_SS211647","PASS",0.6144,0.047,"Plate control","PASS","PFC",1.03311438768413,"Baseline","Baseline"
1491,"PFC_10",14,"OID30075","Q9Y3B8","REXO2",1,"Cardiometabolic_II","B13805","20212097_SS211647","PASS",0.6144,-0.1215,"Plate control","PASS","PFC",0.919231409609416,"1h","1h"
1492,"PFC_18",15,"OID30075","Q9Y3B8","REXO2",1,"Cardiometabolic_II","B13805","20212097_SS211647","PASS",0.6144,0.0055,"Plate control","PASS","PFC",1.00381958558822,"1h","1h"
1493,"PFC_26",16,"OID30075","Q9Y3B8","REXO2",1,"Cardiometabolic_II","B13805","20212097_SS211647","PASS",0.6144,-0.4652,"Plate control","PASS","PFC",0.724370651221682,"24h","24h"
1494,"PFC_03",25,"OID30075","Q9Y3B8","REXO2",1,"Cardiometabolic_II","B13805","20212097_SS211647","PASS",0.6144,0.0066,"Plate control","PASS","PFC",1.00458525163381,"Baseline","Baseline"
1495,"PFC_11",26,"OID30075","Q9Y3B8","REXO2",1,"Cardiometabolic_II","B13805","20212097_SS211647","PASS",0.6144,0.2658,"Plate control","PASS","PFC",1.20230256281833,"1h","1h"
1496,"PFC_19",27,"OID30075","Q9Y3B8","REXO2",1,"Cardiometabolic_II","B13805","20212097_SS211647","PASS",0.6144,-0.1003,"Plate control","PASS","PFC",0.932838992951706,"24h","24h"
1497,"PFC_27",28,"OID30075","Q9Y3B8","REXO2",1,"Cardiometabolic_II","B13805","20212097_SS211647","PASS",0.6144,-0.1239,"Plate control","PASS","PFC",0.91770349046555,"24h","24h"
1498,"PFC_04",37,"OID30075","Q9Y3B8","REXO2",1,"Cardiometabolic_II","B13805","20212097_SS211647","PASS",0.6144,-0.1476,"Plate control","PASS","PFC",0.902750988488249,"Baseline","Baseline"
1499,"PFC_12",38,"OID30075","Q9Y3B8","REXO2",1,"Cardiometabolic_II","B13805","20212097_SS211647","PASS",0.6144,-0.0647,"Plate control","PASS","PFC",0.956144121503579,"1h","1h"
1500,"PFC_20",39,"OID30075","Q9Y3B8","REXO2",1,"Cardiometabolic_II","B13805","20212097_SS211647","PASS",0.6144,0.507,"Plate control","PASS","PFC",1.42109204318136,"24h","24h"
1501,"PFC_05",49,"OID30075","Q9Y3B8","REXO2",1,"Cardiometabolic_II","B13805","20212097_SS211647","PASS",0.6144,0.1138,"Plate control","PASS","PFC",1.08207462673218,"Baseline","Baseline"
1502,"PFC_13",50,"OID30075","Q9Y3B8","REXO2",1,"Cardiometabolic_II","B13805","20212097_SS211647","PASS",0.6144,-0.1135,"Plate control","PASS","PFC",0.924342869767346,"1h","1h"
1503,"PFC_21",51,"OID30075","Q9Y3B8","REXO2",1,"Cardiometabolic_II","B13805","20212097_SS211647","PASS",0.6144,-0.0332,"Plate control","PASS","PFC",0.977250281364469,"24h","24h"
1504,"PFC_06",61,"OID30075","Q9Y3B8","REXO2",1,"Cardiometabolic_II","B13805","20212097_SS211647","PASS",0.6144,-0.248,"Plate control","PASS","PFC",0.842062953608416,"Baseline","Baseline"
1505,"PFC_14",62,"OID30075","Q9Y3B8","REXO2",1,"Cardiometabolic_II","B13805","20212097_SS211647","PASS",0.6144,0.0535,"Plate control","PASS","PFC",1.0377795412288,"1h","1h"
1506,"PFC_22",63,"OID30075","Q9Y3B8","REXO2",1,"Cardiometabolic_II","B13805","20212097_SS211647","PASS",0.6144,-0.2724,"Plate control","PASS","PFC",0.827941075499801,"24h","24h"
1507,"PFC_07",73,"OID30075","Q9Y3B8","REXO2",1,"Cardiometabolic_II","B13805","20212097_SS211647","PASS",0.6144,-0.4835,"Plate control","PASS","PFC",0.715240333641229,"Baseline","Baseline"
1508,"PFC_15",74,"OID30075","Q9Y3B8","REXO2",1,"Cardiometabolic_II","B13805","20212097_SS211647","PASS",0.6144,-0.3617,"Plate control","PASS","PFC",0.778246991642996,"1h","1h"
1509,"PFC_23",75,"OID30075","Q9Y3B8","REXO2",1,"Cardiometabolic_II","B13805","20212097_SS211647","PASS",0.6144,0.3522,"Plate control","PASS","PFC",1.27650571783136,"24h","24h"
1510,"PFC_08",85,"OID30075","Q9Y3B8","REXO2",1,"Cardiometabolic_II","B13805","20212097_SS211647","PASS",0.6144,-0.0673,"Plate control","PASS","PFC",0.954422526918224,"Baseline","Baseline"
1511,"PFC_16",86,"OID30075","Q9Y3B8","REXO2",1,"Cardiometabolic_II","B13805","20212097_SS211647","PASS",0.6144,-0.6448,"Plate control","PASS","PFC",0.639581449308887,"1h","1h"
1512,"PFC_24",87,"OID30075","Q9Y3B8","REXO2",1,"Cardiometabolic_II","B13805","20212097_SS211647","PASS",0.6144,-0.4551,"Plate control","PASS","PFC",0.729459608080624,"24h","24h"
1513,"PFC_01",1,"OID30076","Q6UWF7","NXPE4",0.7273,"Cardiometabolic_II","B13805","20212097_SS211647","WARN",0.493,0.0153,"Plate control","PASS","PFC",1.010661585806,"Baseline","Baseline"
1514,"PFC_09",2,"OID30076","Q6UWF7","NXPE4",0.7273,"Cardiometabolic_II","B13805","20212097_SS211647","PASS",0.493,0.4834,"Plate control","PASS","PFC",1.39803453559951,"Baseline","Baseline"
1515,"PFC_17",3,"OID30076","Q6UWF7","NXPE4",0.7273,"Cardiometabolic_II","B13805","20212097_SS211647","PASS",0.493,0.5434,"Plate control","PASS","PFC",1.45740313583591,"1h","1h"
1516,"PFC_25",4,"OID30076","Q6UWF7","NXPE4",0.7273,"Cardiometabolic_II","B13805","20212097_SS211647","PASS",0.493,0.4985,"Plate control","PASS","PFC",1.41274393928911,"24h","24h"
1517,"PFC_02",13,"OID30076","Q6UWF7","NXPE4",0.7273,"Cardiometabolic_II","B13805","20212097_SS211647","PASS",0.493,0.4648,"Plate control","PASS","PFC",1.38012601404321,"Baseline","Baseline"
1518,"PFC_10",14,"OID30076","Q6UWF7","NXPE4",0.7273,"Cardiometabolic_II","B13805","20212097_SS211647","PASS",0.493,0.3512,"Plate control","PASS","PFC",1.27562121807174,"1h","1h"
1519,"PFC_18",15,"OID30076","Q6UWF7","NXPE4",0.7273,"Cardiometabolic_II","B13805","20212097_SS211647","PASS",0.493,0.0134,"Plate control","PASS","PFC",1.00933144115043,"1h","1h"
1520,"PFC_26",16,"OID30076","Q6UWF7","NXPE4",0.7273,"Cardiometabolic_II","B13805","20212097_SS211647","PASS",0.493,0.2331,"Plate control","PASS","PFC",1.1753577951905,"24h","24h"
1521,"PFC_03",25,"OID30076","Q6UWF7","NXPE4",0.7273,"Cardiometabolic_II","B13805","20212097_SS211647","PASS",0.493,0.6118,"Plate control","PASS","PFC",1.52816465747551,"Baseline","Baseline"
1522,"PFC_11",26,"OID30076","Q6UWF7","NXPE4",0.7273,"Cardiometabolic_II","B13805","20212097_SS211647","PASS",0.493,0.1693,"Plate control","PASS","PFC",1.1245127353175,"1h","1h"
1523,"PFC_19",27,"OID30076","Q6UWF7","NXPE4",0.7273,"Cardiometabolic_II","B13805","20212097_SS211647","PASS",0.493,0.3801,"Plate control","PASS","PFC",1.30143206071198,"24h","24h"
1524,"PFC_27",28,"OID30076","Q6UWF7","NXPE4",0.7273,"Cardiometabolic_II","B13805","20212097_SS211647","PASS",0.493,0.3999,"Plate control","PASS","PFC",1.31941645262382,"24h","24h"
1525,"PFC_04",37,"OID30076","Q6UWF7","NXPE4",0.7273,"Cardiometabolic_II","B13805","20212097_SS211647","PASS",0.493,0.669,"Plate control","PASS","PFC",1.58997050189795,"Baseline","Baseline"
1526,"PFC_12",38,"OID30076","Q6UWF7","NXPE4",0.7273,"Cardiometabolic_II","B13805","20212097_SS211647","PASS",0.493,0.2788,"Plate control","PASS","PFC",1.21318536538748,"1h","1h"
1527,"PFC_20",39,"OID30076","Q6UWF7","NXPE4",0.7273,"Cardiometabolic_II","B13805","20212097_SS211647","PASS",0.493,0.5055,"Plate control","PASS","PFC",1.4196152721146,"24h","24h"
1528,"PFC_05",49,"OID30076","Q6UWF7","NXPE4",0.7273,"Cardiometabolic_II","B13805","20212097_SS211647","PASS",0.493,0.1373,"Plate control","PASS","PFC",1.0998448318404,"Baseline","Baseline"
1529,"PFC_13",50,"OID30076","Q6UWF7","NXPE4",0.7273,"Cardiometabolic_II","B13805","20212097_SS211647","PASS",0.493,0.2897,"Plate control","PASS","PFC",1.22238606322579,"1h","1h"
1530,"PFC_21",51,"OID30076","Q6UWF7","NXPE4",0.7273,"Cardiometabolic_II","B13805","20212097_SS211647","PASS",0.493,0.6786,"Plate control","PASS","PFC",1.60058578317728,"24h","24h"
1531,"PFC_06",61,"OID30076","Q6UWF7","NXPE4",0.7273,"Cardiometabolic_II","B13805","20212097_SS211647","PASS",0.493,0.5415,"Plate control","PASS","PFC",1.4554850289054,"Baseline","Baseline"
1532,"PFC_14",62,"OID30076","Q6UWF7","NXPE4",0.7273,"Cardiometabolic_II","B13805","20212097_SS211647","PASS",0.493,0.1935,"Plate control","PASS","PFC",1.14353458563209,"1h","1h"
1533,"PFC_22",63,"OID30076","Q6UWF7","NXPE4",0.7273,"Cardiometabolic_II","B13805","20212097_SS211647","PASS",0.493,0.406,"Plate control","PASS","PFC",1.32500701704521,"24h","24h"
1534,"PFC_07",73,"OID30076","Q6UWF7","NXPE4",0.7273,"Cardiometabolic_II","B13805","20212097_SS211647","PASS",0.493,0.4383,"Plate control","PASS","PFC",1.35500671391665,"Baseline","Baseline"
1535,"PFC_15",74,"OID30076","Q6UWF7","NXPE4",0.7273,"Cardiometabolic_II","B13805","20212097_SS211647","PASS",0.493,0.0936,"Plate control","PASS","PFC",1.06702945364062,"1h","1h"
1536,"PFC_23",75,"OID30076","Q6UWF7","NXPE4",0.7273,"Cardiometabolic_II","B13805","20212097_SS211647","PASS",0.493,-0.1365,"Plate control","PASS","PFC",0.909723483305892,"24h","24h"
1537,"PFC_08",85,"OID30076","Q6UWF7","NXPE4",0.7273,"Cardiometabolic_II","B13805","20212097_SS211647","PASS",0.493,0.5513,"Plate control","PASS","PFC",1.46540556545172,"Baseline","Baseline"
1538,"PFC_16",86,"OID30076","Q6UWF7","NXPE4",0.7273,"Cardiometabolic_II","B13805","20212097_SS211647","PASS",0.493,0.0016,"Plate control","PASS","PFC",1.00110965069616,"1h","1h"
1539,"PFC_24",87,"OID30076","Q6UWF7","NXPE4",0.7273,"Cardiometabolic_II","B13805","20212097_SS211647","PASS",0.493,0.2175,"Plate control","PASS","PFC",1.16271700470412,"24h","24h"
1540,"PFC_01",1,"OID30077","P55011","SLC12A2",0.9318,"Cardiometabolic_II","B13805","20212097_SS211647","WARN",1.2728,0.1971,"Plate control","PASS","PFC",1.14639164478815,"Baseline","Baseline"
1541,"PFC_09",2,"OID30077","P55011","SLC12A2",0.9318,"Cardiometabolic_II","B13805","20212097_SS211647","PASS",1.2728,1.0748,"Plate control","PASS","PFC",2.10643003845948,"Baseline","Baseline"
1542,"PFC_17",3,"OID30077","P55011","SLC12A2",0.9318,"Cardiometabolic_II","B13805","20212097_SS211647","PASS",1.2728,0.3967,"Plate control","PASS","PFC",1.31649313654243,"1h","1h"
1543,"PFC_25",4,"OID30077","P55011","SLC12A2",0.9318,"Cardiometabolic_II","B13805","20212097_SS211647","PASS",1.2728,0.8265,"Plate control","PASS","PFC",1.77337789784392,"24h","24h"
1544,"PFC_02",13,"OID30077","P55011","SLC12A2",0.9318,"Cardiometabolic_II","B13805","20212097_SS211647","PASS",1.2728,0.2409,"Plate control","PASS","PFC",1.18172963283915,"Baseline","Baseline"
1545,"PFC_10",14,"OID30077","P55011","SLC12A2",0.9318,"Cardiometabolic_II","B13805","20212097_SS211647","PASS",1.2728,0.8972,"Plate control","PASS","PFC",1.86244781185486,"1h","1h"
1546,"PFC_18",15,"OID30077","P55011","SLC12A2",0.9318,"Cardiometabolic_II","B13805","20212097_SS211647","PASS",1.2728,0.6382,"Plate control","PASS","PFC",1.55638609908341,"1h","1h"
1547,"PFC_26",16,"OID30077","P55011","SLC12A2",0.9318,"Cardiometabolic_II","B13805","20212097_SS211647","PASS",1.2728,0.9275,"Plate control","PASS","PFC",1.90197726333528,"24h","24h"
1548,"PFC_03",25,"OID30077","P55011","SLC12A2",0.9318,"Cardiometabolic_II","B13805","20212097_SS211647","PASS",1.2728,-0.0105,"Plate control","PASS","PFC",0.992748375440308,"Baseline","Baseline"
1549,"PFC_11",26,"OID30077","P55011","SLC12A2",0.9318,"Cardiometabolic_II","B13805","20212097_SS211647","PASS",1.2728,0.834,"Plate control","PASS","PFC",1.78262099179394,"1h","1h"
1550,"PFC_19",27,"OID30077","P55011","SLC12A2",0.9318,"Cardiometabolic_II","B13805","20212097_SS211647","PASS",1.2728,0.7448,"Plate control","PASS","PFC",1.67574194607179,"24h","24h"
1551,"PFC_27",28,"OID30077","P55011","SLC12A2",0.9318,"Cardiometabolic_II","B13805","20212097_SS211647","PASS",1.2728,1.6473,"Plate control","PASS","PFC",3.13246849581751,"24h","24h"
1552,"PFC_04",37,"OID30077","P55011","SLC12A2",0.9318,"Cardiometabolic_II","B13805","20212097_SS211647","PASS",1.2728,0.7222,"Plate control","PASS","PFC",1.6496957777643,"Baseline","Baseline"
1553,"PFC_12",38,"OID30077","P55011","SLC12A2",0.9318,"Cardiometabolic_II","B13805","20212097_SS211647","PASS",1.2728,-0.2512,"Plate control","PASS","PFC",0.840197268085905,"1h","1h"
1554,"PFC_20",39,"OID30077","P55011","SLC12A2",0.9318,"Cardiometabolic_II","B13805","20212097_SS211647","PASS",1.2728,0.8139,"Plate control","PASS","PFC",1.7579572652882,"24h","24h"
1555,"PFC_05",49,"OID30077","P55011","SLC12A2",0.9318,"Cardiometabolic_II","B13805","20212097_SS211647","PASS",1.2728,-0.2209,"Plate control","PASS","PFC",0.858030002475228,"Baseline","Baseline"
1556,"PFC_13",50,"OID30077","P55011","SLC12A2",0.9318,"Cardiometabolic_II","B13805","20212097_SS211647","PASS",1.2728,0.8842,"Plate control","PASS","PFC",1.84574084158466,"1h","1h"
1557,"PFC_21",51,"OID30077","P55011","SLC12A2",0.9318,"Cardiometabolic_II","B13805","20212097_SS211647","PASS",1.2728,0.2002,"Plate control","PASS","PFC",1.14885760944067,"24h","24h"
1558,"PFC_06",61,"OID30077","P55011","SLC12A2",0.9318,"Cardiometabolic_II","B13805","20212097_SS211647","PASS",1.2728,0.0404,"Plate control","PASS","PFC",1.02839891985665,"Baseline","Baseline"
1559,"PFC_14",62,"OID30077","P55011","SLC12A2",0.9318,"Cardiometabolic_II","B13805","20212097_SS211647","PASS",1.2728,0.4901,"Plate control","PASS","PFC",1.40454222781251,"1h","1h"
1560,"PFC_22",63,"OID30077","P55011","SLC12A2",0.9318,"Cardiometabolic_II","B13805","20212097_SS211647","PASS",1.2728,0.4214,"Plate control","PASS","PFC",1.33922651794238,"24h","24h"
1561,"PFC_07",73,"OID30077","P55011","SLC12A2",0.9318,"Cardiometabolic_II","B13805","20212097_SS211647","PASS",1.2728,0.6681,"Plate control","PASS","PFC",1.58897893600209,"Baseline","Baseline"
1562,"PFC_15",74,"OID30077","P55011","SLC12A2",0.9318,"Cardiometabolic_II","B13805","20212097_SS211647","PASS",1.2728,0.1374,"Plate control","PASS","PFC",1.09992106991701,"1h","1h"
1563,"PFC_23",75,"OID30077","P55011","SLC12A2",0.9318,"Cardiometabolic_II","B13805","20212097_SS211647","PASS",1.2728,0.6371,"Plate control","PASS","PFC",1.55519986626945,"24h","24h"
1564,"PFC_08",85,"OID30077","P55011","SLC12A2",0.9318,"Cardiometabolic_II","B13805","20212097_SS211647","PASS",1.2728,0.3659,"Plate control","PASS","PFC",1.28868530253077,"Baseline","Baseline"
1565,"PFC_16",86,"OID30077","P55011","SLC12A2",0.9318,"Cardiometabolic_II","B13805","20212097_SS211647","PASS",1.2728,0.5741,"Plate control","PASS","PFC",1.48874844376889,"1h","1h"
1566,"PFC_24",87,"OID30077","P55011","SLC12A2",0.9318,"Cardiometabolic_II","B13805","20212097_SS211647","PASS",1.2728,1.0079,"Plate control","PASS","PFC",2.01098176533183,"24h","24h"
1567,"PFC_01",1,"OID30078","P10109","FDX1",0.4091,"Cardiometabolic_II","B13805","20212097_SS211647","WARN",0.6811,-0.3238,"Plate control","PASS","PFC",0.798962668516433,"Baseline","Baseline"
1568,"PFC_09",2,"OID30078","P10109","FDX1",0.4091,"Cardiometabolic_II","B13805","20212097_SS211647","PASS",0.6811,1.284,"Plate control","PASS","PFC",2.43513203732597,"Baseline","Baseline"

Supplemental table 1_Olink proximity extension assay (PEA) data

,"SampleID","Index","OlinkID","UniProt","Assay","MissingFreq","Panel","Panel_Lot_Nr","PlateID","QC_Warning","LOD","NPX","Normalization","Assay_Warning","condition","Value","group","alt_group"
1569,"PFC_17",3,"OID30078","P10109","FDX1",0.4091,"Cardiometabolic_II","B13805","20212097_SS211647","PASS",0.6811,0.3498,"Plate control","PASS","PFC",1.27438394794497,"1h","1h"
1570,"PFC_25",4,"OID30078","P10109","FDX1",0.4091,"Cardiometabolic_II","B13805","20212097_SS211647","PASS",0.6811,1.0927,"Plate control","PASS","PFC",2.13272802722424,"24h","24h"
1571,"PFC_02",13,"OID30078","P10109","FDX1",0.4091,"Cardiometabolic_II","B13805","20212097_SS211647","PASS",0.6811,0.806,"Plate control","PASS","PFC",1.74835724082075,"Baseline","Baseline"
1572,"PFC_10",14,"OID30078","P10109","FDX1",0.4091,"Cardiometabolic_II","B13805","20212097_SS211647","PASS",0.6811,0.7469,"Plate control","PASS","PFC",1.6781829474037,"1h","1h"
1573,"PFC_18",15,"OID30078","P10109","FDX1",0.4091,"Cardiometabolic_II","B13805","20212097_SS211647","PASS",0.6811,1.0476,"Plate control","PASS","PFC",2.06708827447371,"1h","1h"
1574,"PFC_26",16,"OID30078","P10109","FDX1",0.4091,"Cardiometabolic_II","B13805","20212097_SS211647","PASS",0.6811,0.7975,"Plate control","PASS","PFC",1.73808664085702,"24h","24h"
1575,"PFC_03",25,"OID30078","P10109","FDX1",0.4091,"Cardiometabolic_II","B13805","20212097_SS211647","PASS",0.6811,-0.308,"Plate control","PASS","PFC",0.807760777609608,"Baseline","Baseline"
1576,"PFC_11",26,"OID30078","P10109","FDX1",0.4091,"Cardiometabolic_II","B13805","20212097_SS211647","PASS",0.6811,1.574,"Plate control","PASS","PFC",2.97729051033308,"1h","1h"
1577,"PFC_19",27,"OID30078","P10109","FDX1",0.4091,"Cardiometabolic_II","B13805","20212097_SS211647","PASS",0.6811,-0.195,"Plate control","PASS","PFC",0.873572895916694,"24h","24h"
1578,"PFC_27",28,"OID30078","P10109","FDX1",0.4091,"Cardiometabolic_II","B13805","20212097_SS211647","PASS",0.6811,1.2703,"Plate control","PASS","PFC",2.41211718890283,"24h","24h"
1579,"PFC_04",37,"OID30078","P10109","FDX1",0.4091,"Cardiometabolic_II","B13805","20212097_SS211647","PASS",0.6811,1.0313,"Plate control","PASS","PFC",2.04386513104842,"Baseline","Baseline"
1580,"PFC_12",38,"OID30078","P10109","FDX1",0.4091,"Cardiometabolic_II","B13805","20212097_SS211647","PASS",0.6811,0.6637,"Plate control","PASS","PFC",1.58414017490909,"1h","1h"
1581,"PFC_20",39,"OID30078","P10109","FDX1",0.4091,"Cardiometabolic_II","B13805","20212097_SS211647","PASS",0.6811,1.2912,"Plate control","PASS","PFC",2.44731532869242,"24h","24h"
1582,"PFC_05",49,"OID30078","P10109","FDX1",0.4091,"Cardiometabolic_II","B13805","20212097_SS211647","PASS",0.6811,1.224,"Plate control","PASS","PFC",2.33593478922624,"Baseline","Baseline"
1583,"PFC_13",50,"OID30078","P10109","FDX1",0.4091,"Cardiometabolic_II","B13805","20212097_SS211647","PASS",0.6811,0.5407,"Plate control","PASS","PFC",1.45467816036223,"1h","1h"
1584,"PFC_21",51,"OID30078","P10109","FDX1",0.4091,"Cardiometabolic_II","B13805","20212097_SS211647","PASS",0.6811,0.7457,"Plate control","PASS","PFC",1.67678765443603,"24h","24h"
1585,"PFC_06",61,"OID30078","P10109","FDX1",0.4091,"Cardiometabolic_II","B13805","20212097_SS211647","PASS",0.6811,0.475,"Plate control","PASS","PFC",1.38991821984234,"Baseline","Baseline"
1586,"PFC_14",62,"OID30078","P10109","FDX1",0.4091,"Cardiometabolic_II","B13805","20212097_SS211647","PASS",0.6811,0.8123,"Plate control","PASS","PFC",1.75600870900179,"1h","1h"
1587,"PFC_22",63,"OID30078","P10109","FDX1",0.4091,"Cardiometabolic_II","B13805","20212097_SS211647","PASS",0.6811,0.1965,"Plate control","PASS","PFC",1.14591497303428,"24h","24h"
1588,"PFC_07",73,"OID30078","P10109","FDX1",0.4091,"Cardiometabolic_II","B13805","20212097_SS211647","PASS",0.6811,-0.1401,"Plate control","PASS","PFC",0.907456253062831,"Baseline","Baseline"
1589,"PFC_15",74,"OID30078","P10109","FDX1",0.4091,"Cardiometabolic_II","B13805","20212097_SS211647","PASS",0.6811,0.4172,"Plate control","PASS","PFC",1.33533340698243,"1h","1h"
1590,"PFC_23",75,"OID30078","P10109","FDX1",0.4091,"Cardiometabolic_II","B13805","20212097_SS211647","PASS",0.6811,0.3513,"Plate control","PASS","PFC",1.27570964046127,"24h","24h"
1591,"PFC_08",85,"OID30078","P10109","FDX1",0.4091,"Cardiometabolic_II","B13805","20212097_SS211647","PASS",0.6811,0.5768,"Plate control","PASS","PFC",1.49153724139182,"Baseline","Baseline"
1592,"PFC_16",86,"OID30078","P10109","FDX1",0.4091,"Cardiometabolic_II","B13805","20212097_SS211647","PASS",0.6811,0.2221,"Plate control","PASS","PFC",1.16643021776813,"1h","1h"
1593,"PFC_24",87,"OID30078","P10109","FDX1",0.4091,"Cardiometabolic_II","B13805","20212097_SS211647","PASS",0.6811,0.6026,"Plate control","PASS","PFC",1.51845062753708,"24h","24h"
1594,"PFC_01",1,"OID30079","P30049","ATP5F1D",0.8636,"Cardiometabolic_II","B13805","20212097_SS211647","WARN",0.4311,-0.1758,"Plate control","PASS","PFC",0.88527648145269,"Baseline","Baseline"
1595,"PFC_09",2,"OID30079","P30049","ATP5F1D",0.8636,"Cardiometabolic_II","B13805","20212097_SS211647","PASS",0.4311,-0.1817,"Plate control","PASS","PFC",0.881663475612284,"Baseline","Baseline"
1596,"PFC_17",3,"OID30079","P30049","ATP5F1D",0.8636,"Cardiometabolic_II","B13805","20212097_SS211647","PASS",0.4311,0.3772,"Plate control","PASS","PFC",1.29881864474637,"1h","1h"
1597,"PFC_25",4,"OID30079","P30049","ATP5F1D",0.8636,"Cardiometabolic_II","B13805","20212097_SS211647","PASS",0.4311,0.1324,"Plate control","PASS","PFC",1.096115632105,"24h","24h"
1598,"PFC_02",13,"OID30079","P30049","ATP5F1D",0.8636,"Cardiometabolic_II","B13805","20212097_SS211647","PASS",0.4311,0.1158,"Plate control","PASS","PFC",1.0835757409382,"Baseline","Baseline"
1599,"PFC_10",14,"OID30079","P30049","ATP5F1D",0.8636,"Cardiometabolic_II","B13805","20212097_SS211647","PASS",0.4311,-0.5898,"Plate control","PASS","PFC",0.664435010914851,"1h","1h"
1600,"PFC_18",15,"OID30079","P30049","ATP5F1D",0.8636,"Cardiometabolic_II","B13805","20212097_SS211647","PASS",0.4311,0.1396,"Plate control","PASS","PFC",1.10159964525606,"1h","1h"
1601,"PFC_26",16,"OID30079","P30049","ATP5F1D",0.8636,"Cardiometabolic_II","B13805","20212097_SS211647","PASS",0.4311,-0.3139,"Plate control","PASS","PFC",0.804464130213796,"24h","24h"
1602,"PFC_03",25,"OID30079","P30049","ATP5F1D",0.8636,"Cardiometabolic_II","B13805","20212097_SS211647","PASS",0.4311,0.4121,"Plate control","PASS","PFC",1.33062126952239,"Baseline","Baseline"
1603,"PFC_11",26,"OID30079","P30049","ATP5F1D",0.8636,"Cardiometabolic_II","B13805","20212097_SS211647","PASS",0.4311,0.0954,"Plate control","PASS","PFC",1.06836157971456,"1h","1h"
1604,"PFC_19",27,"OID30079","P30049","ATP5F1D",0.8636,"Cardiometabolic_II","B13805","20212097_SS211647","PASS",0.4311,0.2295,"Plate control","PASS","PFC",1.17242854604109,"24h","24h"
1605,"PFC_27",28,"OID30079","P30049","ATP5F1D",0.8636,"Cardiometabolic_II","B13805","20212097_SS211647","PASS",0.4311,0.1104,"Plate control","PASS","PFC",1.07952750359346,"24h","24h"
1606,"PFC_04",37,"OID30079","P30049","ATP5F1D",0.8636,"Cardiometabolic_II","B13805","20212097_SS211647","PASS",0.4311,0.3523,"Plate control","PASS","PFC",1.27659420153186,"Baseline","Baseline"
1607,"PFC_12",38,"OID30079","P30049","ATP5F1D",0.8636,"Cardiometabolic_II","B13805","20212097_SS211647","PASS",0.4311,0.0678,"Plate control","PASS","PFC",1.0481171655534,"1h","1h"
1608,"PFC_20",39,"OID30079","P30049","ATP5F1D",0.8636,"Cardiometabolic_II","B13805","20212097_SS211647","PASS",0.4311,0.7655,"Plate control","PASS","PFC",1.69995905710904,"24h","24h"
1609,"PFC_05",49,"OID30079","P30049","ATP5F1D",0.8636,"Cardiometabolic_II","B13805","20212097_SS211647","PASS",0.4311,0.1968,"Plate control","PASS","PFC",1.14615328413094,"Baseline","Baseline"
1610,"PFC_13",50,"OID30079","P30049","ATP5F1D",0.8636,"Cardiometabolic_II","B13805","20212097_SS211647","PASS",0.4311,-0.1676,"Plate control","PASS","PFC",0.890322548860819,"1h","1h"
1611,"PFC_21",51,"OID30079","P30049","ATP5F1D",0.8636,"Cardiometabolic_II","B13805","20212097_SS211647","PASS",0.4311,0.6002,"Plate control","PASS","PFC",1.5159267040086,"24h","24h"
1612,"PFC_06",61,"OID30079","P30049","ATP5F1D",0.8636,"Cardiometabolic_II","B13805","20212097_SS211647","PASS",0.4311,0.4082,"Plate control","PASS","PFC",1.32702909314113,"Baseline","Baseline"
1613,"PFC_14",62,"OID30079","P30049","ATP5F1D",0.8636,"Cardiometabolic_II","B13805","20212097_SS211647","PASS",0.4311,-0.242,"Plate control","PASS","PFC",0.845572287377516,"1h","1h"
1614,"PFC_22",63,"OID30079","P30049","ATP5F1D",0.8636,"Cardiometabolic_II","B13805","20212097_SS211647","PASS",0.4311,0.0903,"Plate control","PASS","PFC",1.06459153502436,"24h","24h"
1615,"PFC_07",73,"OID30079","P30049","ATP5F1D",0.8636,"Cardiometabolic_II","B13805","20212097_SS211647","PASS",0.4311,0.3872,"Plate control","PASS","PFC",1.3078526428445,"Baseline","Baseline"
1616,"PFC_15",74,"OID30079","P30049","ATP5F1D",0.8636,"Cardiometabolic_II","B13805","20212097_SS211647","PASS",0.4311,0.3795,"Plate control","PASS","PFC",1.30089092286809,"1h","1h"
1617,"PFC_23",75,"OID30079","P30049","ATP5F1D",0.8636,"Cardiometabolic_II","B13805","20212097_SS211647","PASS",0.4311,-0.1382,"Plate control","PASS","PFC",0.908652141782786,"24h","24h"
1618,"PFC_08",85,"OID30079","P30049","ATP5F1D",0.8636,"Cardiometabolic_II","B13805","20212097_SS211647","PASS",0.4311,0.6227,"Plate control","PASS","PFC",1.53975413230909,"Baseline","Baseline"
1619,"PFC_16",86,"OID30079","P30049","ATP5F1D",0.8636,"Cardiometabolic_II","B13805","20212097_SS211647","PASS",0.4311,0.0352,"Plate control","PASS","PFC",1.02469886661293,"1h","1h"
1620,"PFC_24",87,"OID30079","P30049","ATP5F1D",0.8636,"Cardiometabolic_II","B13805","20212097_SS211647","PASS",0.4311,-0.0385,"Plate control","PASS","PFC",0.973666762875598,"24h","24h"
1621,"PFC_01",1,"OID30080","P33121","ACSL1",0.9545,"Cardiometabolic_II","B13805","20212097_SS211647","WARN",1.3357,-0.1872,"Plate control","PASS","PFC",0.878308700358387,"Baseline","Baseline"
1622,"PFC_09",2,"OID30080","P33121","ACSL1",0.9545,"Cardiometabolic_II","B13805","20212097_SS211647","PASS",1.3357,0.1259,"Plate control","PASS","PFC",1.09118823902842,"Baseline","Baseline"
1623,"PFC_17",3,"OID30080","P33121","ACSL1",0.9545,"Cardiometabolic_II","B13805","20212097_SS211647","PASS",1.3357,0.1837,"Plate control","PASS","PFC",1.1357930587018,"1h","1h"
1624,"PFC_25",4,"OID30080","P33121","ACSL1",0.9545,"Cardiometabolic_II","B13805","20212097_SS211647","PASS",1.3357,0.704,"Plate control","PASS","PFC",1.62901512614043,"24h","24h"
1625,"PFC_02",13,"OID30080","P33121","ACSL1",0.9545,"Cardiometabolic_II","B13805","20212097_SS211647","PASS",1.3357,-0.0596,"Plate control","PASS","PFC",0.959530120687068,"Baseline","Baseline"
1626,"PFC_10",14,"OID30080","P33121","ACSL1",0.9545,"Cardiometabolic_II","B13805","20212097_SS211647","PASS",1.3357,0.5478,"Plate control","PASS","PFC",1.46185477824875,"1h","1h"
1627,"PFC_18",15,"OID30080","P33121","ACSL1",0.9545,"Cardiometabolic_II","B13805","20212097_SS211647","PASS",1.3357,0.5125,"Plate control","PASS","PFC",1.42652002586903,"1h","1h"
1628,"PFC_26",16,"OID30080","P33121","ACSL1",0.9545,"Cardiometabolic_II","B13805","20212097_SS211647","PASS",1.3357,0.1817,"Plate control","PASS","PFC",1.13421960607536,"24h","24h"
1629,"PFC_03",25,"OID30080","P33121","ACSL1",0.9545,"Cardiometabolic_II","B13805","20212097_SS211647","PASS",1.3357,0.8749,"Plate control","PASS","PFC",1.83388096706159,"Baseline","Baseline"
1630,"PFC_11",26,"OID30080","P33121","ACSL1",0.9545,"Cardiometabolic_II","B13805","20212097_SS211647","PASS",1.3357,0.2696,"Plate control","PASS","PFC",1.20547355311477,"1h","1h"
1631,"PFC_19",27,"OID30080","P33121","ACSL1",0.9545,"Cardiometabolic_II","B13805","20212097_SS211647","PASS",1.3357,0.4783,"Plate control","PASS","PFC",1.39310113778951,"24h","24h"
1632,"PFC_27",28,"OID30080","P33121","ACSL1",0.9545,"Cardiometabolic_II","B13805","20212097_SS211647","PASS",1.3357,0.6557,"Plate control","PASS","PFC",1.57538014694386,"24h","24h"
1633,"PFC_04",37,"OID30080","P33121","ACSL1",0.9545,"Cardiometabolic_II","B13805","20212097_SS211647","PASS",1.3357,0.8937,"Plate control","PASS","PFC",1.85793496161568,"Baseline","Baseline"
1634,"PFC_12",38,"OID30080","P33121","ACSL1",0.9545,"Cardiometabolic_II","B13805","20212097_SS211647","PASS",1.3357,0.4048,"Plate control","PASS","PFC",1.32390536541905,"1h","1h"
1635,"PFC_20",39,"OID30080","P33121","ACSL1",0.9545,"Cardiometabolic_II","B13805","20212097_SS211647","PASS",1.3357,0.3968,"Plate control","PASS","PFC",1.31658439205566,"24h","24h"
1636,"PFC_05",49,"OID30080","P33121","ACSL1",0.9545,"Cardiometabolic_II","B13805","20212097_SS211647","PASS",1.3357,1.3001,"Plate control","PASS","PFC",2.46245950546083,"Baseline","Baseline"
1637,"PFC_13",50,"OID30080","P33121","ACSL1",0.9545,"Cardiometabolic_II","B13805","20212097_SS211647","PASS",1.3357,0.4997,"Plate control","PASS","PFC",1.41391951550378,"1h","1h"
1638,"PFC_21",51,"OID30080","P33121","ACSL1",0.9545,"Cardiometabolic_II","B13805","20212097_SS211647","PASS",1.3357,0.6666,"Plate control","PASS","PFC",1.58732770015876,"24h","24h"
1639,"PFC_06",61,"OID30080","P33121","ACSL1",0.9545,"Cardiometabolic_II","B13805","20212097_SS211647","PASS",1.3357,0.561,"Plate control","PASS","PFC",1.47529145708284,"Baseline","Baseline"
1640,"PFC_14",62,"OID30080","P33121","ACSL1",0.9545,"Cardiometabolic_II","B13805","20212097_SS211647","PASS",1.3357,1.2624,"Plate control","PASS","PFC",2.39894486413188,"1h","1h"
1641,"PFC_22",63,"OID30080","P33121","ACSL1",0.9545,"Cardiometabolic_II","B13805","20212097_SS211647","PASS",1.3357,0.8876,"Plate control","PASS","PFC",1.85009582947522,"24h","24h"
1642,"PFC_07",73,"OID30080","P33121","ACSL1",0.9545,"Cardiometabolic_II","B13805","20212097_SS211647","PASS",1.3357,0.4296,"Plate control","PASS","PFC",1.34686009618136,"Baseline","Baseline"
1643,"PFC_15",74,"OID30080","P33121","ACSL1",0.9545,"Cardiometabolic_II","B13805","20212097_SS211647","PASS",1.3357,-0.2279,"Plate control","PASS","PFC",0.853876898576533,"1h","1h"
1644,"PFC_23",75,"OID30080","P33121","ACSL1",0.9545,"Cardiometabolic_II","B13805","20212097_SS211647","PASS",1.3357,0.6987,"Plate control","PASS","PFC",1.62304162484348,"24h","24h"
1645,"PFC_08",85,"OID30080","P33121","ACSL1",0.9545,"Cardiometabolic_II","B13805","20212097_SS211647","PASS",1.3357,0.2167,"Plate control","PASS","PFC",1.16207243622209,"Baseline","Baseline"
1646,"PFC_16",86,"OID30080","P33121","ACSL1",0.9545,"Cardiometabolic_II","B13805","20212097_SS211647","PASS",1.3357,-0.0052,"Plate control","PASS","PFC",0.996402122588542,"1h","1h"
1647,"PFC_24",87,"OID30080","P33121","ACSL1",0.9545,"Cardiometabolic_II","B13805","20212097_SS211647","PASS",1.3357,-0.1265,"Plate control","PASS","PFC",0.916051110531799,"24h","24h"
1648,"PFC_01",1,"OID30081","O60701","UGDH",0.8182,"Cardiometabolic_II","B13805","20212097_SS211647","WARN",0.8953,0.0317,"Plate control","PASS","PFC",1.02221594467769,"Baseline","Baseline"
1649,"PFC_09",2,"OID30081","O60701","UGDH",0.8182,"Cardiometabolic_II","B13805","20212097_SS211647","PASS",0.8953,-0.488,"Plate control","PASS","PFC",0.71301285868207,"Baseline","Baseline"
1650,"PFC_17",3,"OID30081","O60701","UGDH",0.8182,"Cardiometabolic_II","B13805","20212097_SS211647","PASS",0.8953,1.0514,"Plate control","PASS","PFC",2.0725400775913,"1h","1h"
1651,"PFC_25",4,"OID30081","O60701","UGDH",0.8182,"Cardiometabolic_II","B13805","20212097_SS211647","PASS",0.8953,-0.3812,"Plate control","PASS","PFC",0.767798687973723,"24h","24h"
1652,"PFC_02",13,"OID30081","O60701","UGDH",0.8182,"Cardiometabolic_II","B13805","20212097_SS211647","PASS",0.8953,-0.0281,"Plate control","PASS","PFC",0.980711023924451,"Baseline","Baseline"
1653,"PFC_10",14,"OID30081","O60701","UGDH",0.8182,"Cardiometabolic_II","B13805","20212097_SS211647","PASS",0.8953,0.5632,"Plate control","PASS","PFC",1.47754288032175,"1h","1h"
1654,"PFC_18",15,"OID30081","O60701","UGDH",0.8182,"Cardiometabolic_II","B13805","20212097_SS211647","PASS",0.8953,-0.1544,"Plate control","PASS","PFC",0.898505973319796,"1h","1h"
1655,"PFC_26",16,"OID30081","O60701","UGDH",0.8182,"Cardiometabolic_II","B13805","20212097_SS211647","PASS",0.8953,1.0937,"Plate control","PASS","PFC",2.13420683409941,"24h","24h"
1656,"PFC_03",25,"OID30081","O60701","UGDH",0.8182,"Cardiometabolic_II","B13805","20212097_SS211647","PASS",0.8953,0.1045,"Plate control","PASS","PFC",1.07512171708826,"Baseline","Baseline"
1657,"PFC_11",26,"OID30081","O60701","UGDH",0.8182,"Cardiometabolic_II","B13805","20212097_SS211647","PASS",0.8953,-0.1638,"Plate control","PASS","PFC",0.892670713333269,"1h","1h"
1658,"PFC_19",27,"OID30081","O60701","UGDH",0.8182,"Cardiometabolic_II","B13805","20212097_SS211647","PASS",0.8953,0.3665,"Plate control","PASS","PFC",1.28922136314416,"24h","24h"
1659,"PFC_27",28,"OID30081","O60701","UGDH",0.8182,"Cardiometabolic_II","B13805","20212097_SS211647","PASS",0.8953,0.0884,"Plate control","PASS","PFC",1.06319041247201,"24h","24h"
1660,"PFC_04",37,"OID30081","O60701","UGDH",0.8182,"Cardiometabolic_II","B13805","20212097_SS211647","PASS",0.8953,0.4529,"Plate control","PASS","PFC",1.36878893258463,"Baseline","Baseline"
1661,"PFC_12",38,"OID30081","O60701","UGDH",0.8182,"Cardiometabolic_II","B13805","20212097_SS211647","PASS",0.8953,0.5753,"Plate control","PASS","PFC",1.48998726505239,"1h","1h"
1662,"PFC_20",39,"OID30081","O60701","UGDH",0.8182,"Cardiometabolic_II","B13805","20212097_SS211647","PASS",0.8953,-0.6187,"Plate control","PASS","PFC",0.651257505896261,"24h","24h"
1663,"PFC_05",49,"OID30081","O60701","UGDH",0.8182,"Cardiometabolic_II","B13805","20212097_SS211647","PASS",0.8953,0.1144,"Plate control","PASS","PFC",1.08252474251064,"Baseline","Baseline"
1664,"PFC_13",50,"OID30081","O60701","UGDH",0.8182,"Cardiometabolic_II","B13805","20212097_SS211647","PASS",0.8953,0.1354,"Plate control","PASS","PFC",1.09839731197266,"1h","1h"
1665,"PFC_21",51,"OID30081","O60701","UGDH",0.8182,"Cardiometabolic_II","B13805","20212097_SS211647","PASS",0.8953,-0.0683,"Plate control","PASS","PFC",0.953761200859253,"24h","24h"
1666,"PFC_06",61,"OID30081","O60701","UGDH",0.8182,"Cardiometabolic_II","B13805","20212097_SS211647","PASS",0.8953,0.7243,"Plate control","PASS","PFC",1.65209883845046,"Baseline","Baseline"
1667,"PFC_14",62,"OID30081","O60701","UGDH",0.8182,"Cardiometabolic_II","B13805","20212097_SS211647","PASS",0.8953,0.5454,"Plate control","PASS","PFC",1.45942492665981,"1h","1h"
1668,"PFC_22",63,"OID30081","O60701","UGDH",0.8182,"Cardiometabolic_II","B13805","20212097_SS211647","PASS",0.8953,-0.4676,"Plate control","PASS","PFC",0.723166623842192,"24h","24h"
1669,"PFC_07",73,"OID30081","O60701","UGDH",0.8182,"Cardiometabolic_II","B13805","20212097_SS211647","PASS",0.8953,0.3309,"Plate control","PASS","PFC",1.25779778491555,"Baseline","Baseline"
1670,"PFC_15",74,"OID30081","O60701","UGDH",0.8182,"Cardiometabolic_II","B13805","20212097_SS211647","PASS",0.8953,0.7286,"Plate control","PASS","PFC",1.65703031892262,"1h","1h"
1671,"PFC_23",75,"OID30081","O60701","UGDH",0.8182,"Cardiometabolic_II","B13805","20212097_SS211647","PASS",0.8953,0.1471,"Plate control","PASS","PFC",1.10734133687678,"24h","24h"
1672,"PFC_08",85,"OID30081","O60701","UGDH",0.8182,"Cardiometabolic_II","B13805","20212097_SS211647","PASS",0.8953,1.6901,"Plate control","PASS","PFC",3.2267906932242,"Baseline","Baseline"
1673,"PFC_16",86,"OID30081","O60701","UGDH",0.8182,"Cardiometabolic_II","B13805","20212097_SS211647","PASS",0.8953,0.5574,"Plate control","PASS","PFC",1.47161470753179,"1h","1h"
1674,"PFC_24",87,"OID30081","O60701","UGDH",0.8182,"Cardiometabolic_II","B13805","20212097_SS211647","PASS",0.8953,1.0248,"Plate control","PASS","PFC",2.03467729848657,"24h","24h"
1675,"PFC_01",1,"OID30082","Q9BY32","ITPA",0.0455,"Cardiometabolic_II","B13805","20212097_SS211647","WARN",1.307,3.2138,"Plate control","PASS","PFC",9.27791095532332,"Baseline","Baseline"
1676,"PFC_09",2,"OID30082","Q9BY32","ITPA",0.0455,"Cardiometabolic_II","B13805","20212097_SS211647","PASS",1.307,3.498,"Plate control","PASS","PFC",11.298035235078,"Baseline","Baseline"
1677,"PFC_17",3,"OID30082","Q9BY32","ITPA",0.0455,"Cardiometabolic_II","B13805","20212097_SS211647","PASS",1.307,5.2224,"Plate control","PASS","PFC",37.3335294506746,"1h","1h"
1678,"PFC_25",4,"OID30082","Q9BY32","ITPA",0.0455,"Cardiometabolic_II","B13805","20212097_SS211647","PASS",1.307,4.6596,"Plate control","PASS","PFC",25.2743134804369,"24h","24h"
1679,"PFC_02",13,"OID30082","Q9BY32","ITPA",0.0455,"Cardiometabolic_II","B13805","20212097_SS211647","PASS",1.307,3.4459,"Plate control","PASS","PFC",10.8973089070506,"Baseline","Baseline"
1680,"PFC_10",14,"OID30082","Q9BY32","ITPA",0.0455,"Cardiometabolic_II","B13805","20212097_SS211647","PASS",1.307,3.5575,"Plate control","PASS","PFC",11.7737337250049,"1h","1h"

Supplemental table 1_Olink proximity extension assay (PEA) data

**,"SampleID","Index","OlinkID","UniProt","Assay","MissingFreq","Panel","Panel_Lot_Nr","PlateID","QC_Warning","LOD","NPX","Normalization","Assay_Warning","condition","Value","group","alt_group"**
1681,"PFC_18",15,"OID30082","Q9BY32","ITPA",0.0455,"Cardiometabolic_II","B13805","20212097_SS211647","PASS",1.307,3.9789,"Plate control","PASS","PFC",15.7676964197191,"1h","1h"
1682,"PFC_26",16,"OID30082","Q9BY32","ITPA",0.0455,"Cardiometabolic_II","B13805","20212097_SS211647","PASS",1.307,5.0275,"Plate control","PASS","PFC",32.6158201150424,"24h","24h"
1683,"PFC_03",25,"OID30082","Q9BY32","ITPA",0.0455,"Cardiometabolic_II","B13805","20212097_SS211647","PASS",1.307,2.4949,"Plate control","PASS","PFC",5.63689228742684,"Baseline","Baseline"
1684,"PFC_11",26,"OID30082","Q9BY32","ITPA",0.0455,"Cardiometabolic_II","B13805","20212097_SS211647","PASS",1.307,4.7824,"Plate control","PASS","PFC",27.5198366209802,"1h","1h"
1685,"PFC_19",27,"OID30082","Q9BY32","ITPA",0.0455,"Cardiometabolic_II","B13805","20212097_SS211647","PASS",1.307,4.4236,"Plate control","PASS","PFC",21.4603247077597,"24h","24h"
1686,"PFC_27",28,"OID30082","Q9BY32","ITPA",0.0455,"Cardiometabolic_II","B13805","20212097_SS211647","PASS",1.307,5.1892,"Plate control","PASS","PFC",36.4842021600005,"24h","24h"
1687,"PFC_04",37,"OID30082","Q9BY32","ITPA",0.0455,"Cardiometabolic_II","B13805","20212097_SS211647","PASS",1.307,4.048,"Plate control","PASS","PFC",16.5412917799674,"Baseline","Baseline"
1688,"PFC_12",38,"OID30082","Q9BY32","ITPA",0.0455,"Cardiometabolic_II","B13805","20212097_SS211647","PASS",1.307,2.8586,"Plate control","PASS","PFC",7.25311136318037,"1h","1h"
1689,"PFC_20",39,"OID30082","Q9BY32","ITPA",0.0455,"Cardiometabolic_II","B13805","20212097_SS211647","PASS",1.307,5.0921,"Plate control","PASS","PFC",34.1094597598224,"24h","24h"
1690,"PFC_05",49,"OID30082","Q9BY32","ITPA",0.0455,"Cardiometabolic_II","B13805","20212097_SS211647","PASS",1.307,2.9171,"Plate control","PASS","PFC",7.55326288734492,"Baseline","Baseline"
1691,"PFC_13",50,"OID30082","Q9BY32","ITPA",0.0455,"Cardiometabolic_II","B13805","20212097_SS211647","PASS",1.307,4.4082,"Plate control","PASS","PFC",21.2324654902582,"1h","1h"
1692,"PFC_21",51,"OID30082","Q9BY32","ITPA",0.0455,"Cardiometabolic_II","B13805","20212097_SS211647","PASS",1.307,3.1612,"Plate control","PASS","PFC",8.94573486395056,"24h","24h"
1693,"PFC_06",61,"OID30082","Q9BY32","ITPA",0.0455,"Cardiometabolic_II","B13805","20212097_SS211647","PASS",1.307,4.3587,"Plate control","PASS","PFC",20.5163189520034,"Baseline","Baseline"
1694,"PFC_14",62,"OID30082","Q9BY32","ITPA",0.0455,"Cardiometabolic_II","B13805","20212097_SS211647","PASS",1.307,4.0023,"Plate control","PASS","PFC",16.0255281598256,"1h","1h"
1695,"PFC_22",63,"OID30082","Q9BY32","ITPA",0.0455,"Cardiometabolic_II","B13805","20212097_SS211647","PASS",1.307,3.6403,"Plate control","PASS","PFC",12.4692259076317,"24h","24h"
1696,"PFC_07",73,"OID30082","Q9BY32","ITPA",0.0455,"Cardiometabolic_II","B13805","20212097_SS211647","PASS",1.307,5.0749,"Plate control","PASS","PFC",33.7052168019844,"Baseline","Baseline"
1697,"PFC_15",74,"OID30082","Q9BY32","ITPA",0.0455,"Cardiometabolic_II","B13805","20212097_SS211647","PASS",1.307,3.165,"Plate control","PASS","PFC",8.96932862422807,"1h","1h"
1698,"PFC_23",75,"OID30082","Q9BY32","ITPA",0.0455,"Cardiometabolic_II","B13805","20212097_SS211647","PASS",1.307,4.7538,"Plate control","PASS","PFC",26.9796550943703,"24h","24h"
1699,"PFC_08",85,"OID30082","Q9BY32","ITPA",0.0455,"Cardiometabolic_II","B13805","20212097_SS211647","PASS",1.307,4.4058,"Plate control","PASS","PFC",21.1971735168172,"Baseline","Baseline"
1700,"PFC_16",86,"OID30082","Q9BY32","ITPA",0.0455,"Cardiometabolic_II","B13805","20212097_SS211647","PASS",1.307,4.5528,"Plate control","PASS","PFC",23.4708795263399,"1h","1h"
1701,"PFC_24",87,"OID30082","Q9BY32","ITPA",0.0455,"Cardiometabolic_II","B13805","20212097_SS211647","PASS",1.307,4.89,"Plate control","PASS","PFC",29.6508179804919,"24h","24h"
1702,"PFC_01",1,"OID30083","Q01780","EXOSC10",0.9318,"Cardiometabolic_II","B13805","20212097_SS211647","WARN",0.3814,0.1802,"Plate control","PASS","PFC",1.13304094723645,"Baseline","Baseline"
1703,"PFC_09",2,"OID30083","Q01780","EXOSC10",0.9318,"Cardiometabolic_II","B13805","20212097_SS211647","PASS",0.3814,-0.3255,"Plate control","PASS","PFC",0.798021765156346,"Baseline","Baseline"
1704,"PFC_17",3,"OID30083","Q01780","EXOSC10",0.9318,"Cardiometabolic_II","B13805","20212097_SS211647","PASS",0.3814,-0.4556,"Plate control","PASS","PFC",0.729206840449111,"1h","1h"
1705,"PFC_25",4,"OID30083","Q01780","EXOSC10",0.9318,"Cardiometabolic_II","B13805","20212097_SS211647","PASS",0.3814,-0.0363,"Plate control","PASS","PFC",0.975152663147281,"24h","24h"
1706,"PFC_02",13,"OID30083","Q01780","EXOSC10",0.9318,"Cardiometabolic_II","B13805","20212097_SS211647","PASS",0.3814,-0.3279,"Plate control","PASS","PFC",0.796695317083034,"Baseline","Baseline"
1707,"PFC_10",14,"OID30083","Q01780","EXOSC10",0.9318,"Cardiometabolic_II","B13805","20212097_SS211647","PASS",0.3814,0.3132,"Plate control","PASS","PFC",1.24246051144621,"1h","1h"
1708,"PFC_18",15,"OID30083","Q01780","EXOSC10",0.9318,"Cardiometabolic_II","B13805","20212097_SS211647","PASS",0.3814,-0.3991,"Plate control","PASS","PFC",0.758331207351636,"1h","1h"
1709,"PFC_26",16,"OID30083","Q01780","EXOSC10",0.9318,"Cardiometabolic_II","B13805","20212097_SS211647","PASS",0.3814,-0.6444,"Plate control","PASS","PFC",0.639758803525597,"24h","24h"
1710,"PFC_03",25,"OID30083","Q01780","EXOSC10",0.9318,"Cardiometabolic_II","B13805","20212097_SS211647","PASS",0.3814,-0.2149,"Plate control","PASS","PFC",0.86160587961088,"Baseline","Baseline"
1711,"PFC_11",26,"OID30083","Q01780","EXOSC10",0.9318,"Cardiometabolic_II","B13805","20212097_SS211647","PASS",0.3814,0.0061,"Plate control","PASS","PFC",1.00423714924145,"1h","1h"
1712,"PFC_19",27,"OID30083","Q01780","EXOSC10",0.9318,"Cardiometabolic_II","B13805","20212097_SS211647","PASS",0.3814,-0.0746,"Plate control","PASS","PFC",0.949605370968844,"24h","24h"
1713,"PFC_27",28,"OID30083","Q01780","EXOSC10",0.9318,"Cardiometabolic_II","B13805","20212097_SS211647","PASS",0.3814,-0.0088,"Plate control","PASS","PFC",0.993918870184885,"24h","24h"
1714,"PFC_04",37,"OID30083","Q01780","EXOSC10",0.9318,"Cardiometabolic_II","B13805","20212097_SS211647","PASS",0.3814,-0.5689,"Plate control","PASS","PFC",0.674130591421003,"Baseline","Baseline"
1715,"PFC_12",38,"OID30083","Q01780","EXOSC10",0.9318,"Cardiometabolic_II","B13805","20212097_SS211647","PASS",0.3814,-0.5181,"Plate control","PASS","PFC",0.69829086272191,"1h","1h"
1716,"PFC_20",39,"OID30083","Q01780","EXOSC10",0.9318,"Cardiometabolic_II","B13805","20212097_SS211647","PASS",0.3814,-0.206,"Plate control","PASS","PFC",0.866937563514687,"24h","24h"
1717,"PFC_05",49,"OID30083","Q01780","EXOSC10",0.9318,"Cardiometabolic_II","B13805","20212097_SS211647","PASS",0.3814,-0.2346,"Plate control","PASS","PFC",0.849920614504973,"Baseline","Baseline"
1718,"PFC_13",50,"OID30083","Q01780","EXOSC10",0.9318,"Cardiometabolic_II","B13805","20212097_SS211647","PASS",0.3814,-0.3082,"Plate control","PASS","PFC",0.807648805949958,"1h","1h"
1719,"PFC_21",51,"OID30083","Q01780","EXOSC10",0.9318,"Cardiometabolic_II","B13805","20212097_SS211647","PASS",0.3814,0.1147,"Plate control","PASS","PFC",1.08274987060881,"24h","24h"
1720,"PFC_06",61,"OID30083","Q01780","EXOSC10",0.9318,"Cardiometabolic_II","B13805","20212097_SS211647","PASS",0.3814,-0.4165,"Plate control","PASS","PFC",0.7492400890481,"Baseline","Baseline"
1721,"PFC_14",62,"OID30083","Q01780","EXOSC10",0.9318,"Cardiometabolic_II","B13805","20212097_SS211647","PASS",0.3814,0.1884,"Plate control","PASS","PFC",1.13949926971051,"1h","1h"
1722,"PFC_22",63,"OID30083","Q01780","EXOSC10",0.9318,"Cardiometabolic_II","B13805","20212097_SS211647","PASS",0.3814,-0.3557,"Plate control","PASS","PFC",0.781490369631269,"24h","24h"
1723,"PFC_07",73,"OID30083","Q01780","EXOSC10",0.9318,"Cardiometabolic_II","B13805","20212097_SS211647","PASS",0.3814,0.0876,"Plate control","PASS","PFC",1.06260101795253,"Baseline","Baseline"
1724,"PFC_15",74,"OID30083","Q01780","EXOSC10",0.9318,"Cardiometabolic_II","B13805","20212097_SS211647","PASS",0.3814,-0.7652,"Plate control","PASS","PFC",0.588371797304938,"1h","1h"
1725,"PFC_23",75,"OID30083","Q01780","EXOSC10",0.9318,"Cardiometabolic_II","B13805","20212097_SS211647","PASS",0.3814,0.1529,"Plate control","PASS","PFC",1.11180209059776,"24h","24h"
1726,"PFC_08",85,"OID30083","Q01780","EXOSC10",0.9318,"Cardiometabolic_II","B13805","20212097_SS211647","PASS",0.3814,-0.3845,"Plate control","PASS","PFC",0.766044443319496,"Baseline","Baseline"
1727,"PFC_16",86,"OID30083","Q01780","EXOSC10",0.9318,"Cardiometabolic_II","B13805","20212097_SS211647","PASS",0.3814,-0.6712,"Plate control","PASS","PFC",0.627984127451672,"1h","1h"
1728,"PFC_24",87,"OID30083","Q01780","EXOSC10",0.9318,"Cardiometabolic_II","B13805","20212097_SS211647","PASS",0.3814,-0.1637,"Plate control","PASS","PFC",0.892732590696562,"24h","24h"
1729,"PFC_01",1,"OID30084","Q08499","PDE4D",0.7045,"Cardiometabolic_II","B13805","20212097_SS211647","WARN",-0.6449,-0.7473,"Plate control","PASS","PFC",0.595717398455945,"Baseline","Baseline"
1730,"PFC_09",2,"OID30084","Q08499","PDE4D",0.7045,"Cardiometabolic_II","B13805","20212097_SS211647","PASS",-0.6449,0.0307,"Plate control","PASS","PFC",1.02150764408436,"Baseline","Baseline"
1731,"PFC_17",3,"OID30084","Q08499","PDE4D",0.7045,"Cardiometabolic_II","B13805","20212097_SS211647","PASS",-0.6449,-0.9469,"Plate control","PASS","PFC",0.518745923764118,"1h","1h"
1732,"PFC_25",4,"OID30084","Q08499","PDE4D",0.7045,"Cardiometabolic_II","B13805","20212097_SS211647","PASS",-0.6449,-0.6605,"Plate control","PASS","PFC",0.632658996085689,"24h","24h"
1733,"PFC_02",13,"OID30084","Q08499","PDE4D",0.7045,"Cardiometabolic_II","B13805","20212097_SS211647","PASS",-0.6449,-0.5796,"Plate control","PASS","PFC",0.669149279250364,"Baseline","Baseline"
1734,"PFC_10",14,"OID30084","Q08499","PDE4D",0.7045,"Cardiometabolic_II","B13805","20212097_SS211647","PASS",-0.6449,-1.104,"Plate control","PASS","PFC",0.465224828851508,"1h","1h"
1735,"PFC_18",15,"OID30084","Q08499","PDE4D",0.7045,"Cardiometabolic_II","B13805","20212097_SS211647","PASS",-0.6449,-0.1381,"Plate control","PASS","PFC",0.908715126932678,"1h","1h"
1736,"PFC_26",16,"OID30084","Q08499","PDE4D",0.7045,"Cardiometabolic_II","B13805","20212097_SS211647","PASS",-0.6449,-1.1301,"Plate control","PASS","PFC",0.456884055227646,"24h","24h"
1737,"PFC_03",25,"OID30084","Q08499","PDE4D",0.7045,"Cardiometabolic_II","B13805","20212097_SS211647","PASS",-0.6449,-0.9219,"Plate control","PASS","PFC",0.527813442791038,"Baseline","Baseline"
1738,"PFC_11",26,"OID30084","Q08499","PDE4D",0.7045,"Cardiometabolic_II","B13805","20212097_SS211647","PASS",-0.6449,-1.4429,"Plate control","PASS","PFC",0.36782718141771,"1h","1h"
1739,"PFC_19",27,"OID30084","Q08499","PDE4D",0.7045,"Cardiometabolic_II","B13805","20212097_SS211647","PASS",-0.6449,-1.0498,"Plate control","PASS","PFC",0.483035122707807,"24h","24h"
1740,"PFC_27",28,"OID30084","Q08499","PDE4D",0.7045,"Cardiometabolic_II","B13805","20212097_SS211647","PASS",-0.6449,0.2224,"Plate control","PASS","PFC",1.16667279533358,"24h","24h"
1741,"PFC_04",37,"OID30084","Q08499","PDE4D",0.7045,"Cardiometabolic_II","B13805","20212097_SS211647","PASS",-0.6449,-1.6017,"Plate control","PASS","PFC",0.329488496016973,"Baseline","Baseline"
1742,"PFC_12",38,"OID30084","Q08499","PDE4D",0.7045,"Cardiometabolic_II","B13805","20212097_SS211647","PASS",-0.6449,-0.6409,"Plate control","PASS","PFC",0.641312752253165,"1h","1h"
1743,"PFC_20",39,"OID30084","Q08499","PDE4D",0.7045,"Cardiometabolic_II","B13805","20212097_SS211647","PASS",-0.6449,-1.4779,"Plate control","PASS","PFC",0.359011011540483,"24h","24h"
1744,"PFC_05",49,"OID30084","Q08499","PDE4D",0.7045,"Cardiometabolic_II","B13805","20212097_SS211647","PASS",-0.6449,-0.993,"Plate control","PASS","PFC",0.502431910211893,"Baseline","Baseline"
1745,"PFC_13",50,"OID30084","Q08499","PDE4D",0.7045,"Cardiometabolic_II","B13805","20212097_SS211647","PASS",-0.6449,-0.7982,"Plate control","PASS","PFC",0.57506622004339,"1h","1h"
1746,"PFC_21",51,"OID30084","Q08499","PDE4D",0.7045,"Cardiometabolic_II","B13805","20212097_SS211647","PASS",-0.6449,-1.1647,"Plate control","PASS","PFC",0.446057005013409,"24h","24h"
1747,"PFC_06",61,"OID30084","Q08499","PDE4D",0.7045,"Cardiometabolic_II","B13805","20212097_SS211647","PASS",-0.6449,-0.413,"Plate control","PASS","PFC",0.751059963470465,"Baseline","Baseline"
1748,"PFC_14",62,"OID30084","Q08499","PDE4D",0.7045,"Cardiometabolic_II","B13805","20212097_SS211647","PASS",-0.6449,-0.7047,"Plate control","PASS","PFC",0.613570063670113,"1h","1h"
1749,"PFC_22",63,"OID30084","Q08499","PDE4D",0.7045,"Cardiometabolic_II","B13805","20212097_SS211647","PASS",-0.6449,-1.5441,"Plate control","PASS","PFC",0.34290955264491,"24h","24h"
1750,"PFC_07",73,"OID30084","Q08499","PDE4D",0.7045,"Cardiometabolic_II","B13805","20212097_SS211647","PASS",-0.6449,-0.5993,"Plate control","PASS","PFC",0.660074147675127,"Baseline","Baseline"
1751,"PFC_15",74,"OID30084","Q08499","PDE4D",0.7045,"Cardiometabolic_II","B13805","20212097_SS211647","PASS",-0.6449,-0.6495,"Plate control","PASS","PFC",0.637501216463279,"1h","1h"
1752,"PFC_23",75,"OID30084","Q08499","PDE4D",0.7045,"Cardiometabolic_II","B13805","20212097_SS211647","PASS",-0.6449,-0.5242,"Plate control","PASS","PFC",0.695344583945501,"24h","24h"
1753,"PFC_08",85,"OID30084","Q08499","PDE4D",0.7045,"Cardiometabolic_II","B13805","20212097_SS211647","PASS",-0.6449,-0.8607,"Plate control","PASS","PFC",0.550685298932478,"Baseline","Baseline"
1754,"PFC_16",86,"OID30084","Q08499","PDE4D",0.7045,"Cardiometabolic_II","B13805","20212097_SS211647","PASS",-0.6449,-0.817,"Plate control","PASS","PFC",0.567621051073032,"1h","1h"
1755,"PFC_24",87,"OID30084","Q08499","PDE4D",0.7045,"Cardiometabolic_II","B13805","20212097_SS211647","PASS",-0.6449,-0.3062,"Plate control","PASS","PFC",0.808769221368765,"24h","24h"
1756,"PFC_01",1,"OID30085","P21817","RYR1",0.8864,"Cardiometabolic_II","B13805","20212097_SS211647","WARN",0.504,-0.0721,"Plate control","PASS","PFC",0.95125233825896,"Baseline","Baseline"
1757,"PFC_09",2,"OID30085","P21817","RYR1",0.8864,"Cardiometabolic_II","B13805","20212097_SS211647","PASS",0.504,0.3599,"Plate control","PASS","PFC",1.28333694034173,"Baseline","Baseline"
1758,"PFC_17",3,"OID30085","P21817","RYR1",0.8864,"Cardiometabolic_II","B13805","20212097_SS211647","PASS",0.504,0.0039,"Plate control","PASS","PFC",1.00270693114403,"1h","1h"
1759,"PFC_25",4,"OID30085","P21817","RYR1",0.8864,"Cardiometabolic_II","B13805","20212097_SS211647","PASS",0.504,0.1345,"Plate control","PASS","PFC",1.09771230975825,"24h","24h"
1760,"PFC_02",13,"OID30085","P21817","RYR1",0.8864,"Cardiometabolic_II","B13805","20212097_SS211647","PASS",0.504,-0.1843,"Plate control","PASS","PFC",0.880075987877347,"Baseline","Baseline"
1761,"PFC_10",14,"OID30085","P21817","RYR1",0.8864,"Cardiometabolic_II","B13805","20212097_SS211647","PASS",0.504,0.2795,"Plate control","PASS","PFC",1.21377414942669,"1h","1h"
1762,"PFC_18",15,"OID30085","P21817","RYR1",0.8864,"Cardiometabolic_II","B13805","20212097_SS211647","PASS",0.504,0.0113,"Plate control","PASS","PFC",1.00786331790678,"1h","1h"
1763,"PFC_26",16,"OID30085","P21817","RYR1",0.8864,"Cardiometabolic_II","B13805","20212097_SS211647","PASS",0.504,0.1868,"Plate control","PASS","PFC",1.13823622509095,"24h","24h"
1764,"PFC_03",25,"OID30085","P21817","RYR1",0.8864,"Cardiometabolic_II","B13805","20212097_SS211647","PASS",0.504,0.1461,"Plate control","PASS","PFC",1.10657405230249,"Baseline","Baseline"
1765,"PFC_11",26,"OID30085","P21817","RYR1",0.8864,"Cardiometabolic_II","B13805","20212097_SS211647","PASS",0.504,-0.3145,"Plate control","PASS","PFC",0.80412963254922,"1h","1h"
1766,"PFC_19",27,"OID30085","P21817","RYR1",0.8864,"Cardiometabolic_II","B13805","20212097_SS211647","PASS",0.504,0.2226,"Plate control","PASS","PFC",1.16683454173647,"24h","24h"
1767,"PFC_27",28,"OID30085","P21817","RYR1",0.8864,"Cardiometabolic_II","B13805","20212097_SS211647","PASS",0.504,0.0274,"Plate control","PASS","PFC",1.01917373240678,"24h","24h"
1768,"PFC_04",37,"OID30085","P21817","RYR1",0.8864,"Cardiometabolic_II","B13805","20212097_SS211647","PASS",0.504,0.2185,"Plate control","PASS","PFC",1.16352321809771,"Baseline","Baseline"
1769,"PFC_12",38,"OID30085","P21817","RYR1",0.8864,"Cardiometabolic_II","B13805","20212097_SS211647","PASS",0.504,-0.2626,"Plate control","PASS","PFC",0.833584293763525,"1h","1h"
1770,"PFC_20",39,"OID30085","P21817","RYR1",0.8864,"Cardiometabolic_II","B13805","20212097_SS211647","PASS",0.504,-0.6652,"Plate control","PASS","PFC",0.630601278456215,"24h","24h"
1771,"PFC_05",49,"OID30085","P21817","RYR1",0.8864,"Cardiometabolic_II","B13805","20212097_SS211647","PASS",0.504,0.6238,"Plate control","PASS","PFC",1.54092858384859,"Baseline","Baseline"
1772,"PFC_13",50,"OID30085","P21817","RYR1",0.8864,"Cardiometabolic_II","B13805","20212097_SS211647","PASS",0.504,-0.2514,"Plate control","PASS","PFC",0.840080800085541,"1h","1h"
1773,"PFC_21",51,"OID30085","P21817","RYR1",0.8864,"Cardiometabolic_II","B13805","20212097_SS211647","PASS",0.504,0.1137,"Plate control","PASS","PFC",1.08199962563388,"24h","24h"
1774,"PFC_06",61,"OID30085","P21817","RYR1",0.8864,"Cardiometabolic_II","B13805","20212097_SS211647","PASS",0.504,0.0377,"Plate control","PASS","PFC",1.02647607382671,"Baseline","Baseline"
1775,"PFC_14",62,"OID30085","P21817","RYR1",0.8864,"Cardiometabolic_II","B13805","20212097_SS211647","PASS",0.504,0.5648,"Plate control","PASS","PFC",1.47918243680751,"1h","1h"
1776,"PFC_22",63,"OID30085","P21817","RYR1",0.8864,"Cardiometabolic_II","B13805","20212097_SS211647","PASS",0.504,-0.7216,"Plate control","PASS","PFC",0.606424522803625,"24h","24h"
1777,"PFC_07",73,"OID30085","P21817","RYR1",0.8864,"Cardiometabolic_II","B13805","20212097_SS211647","PASS",0.504,0.1091,"Plate control","PASS","PFC",1.07855518885236,"Baseline","Baseline"
1778,"PFC_15",74,"OID30085","P21817","RYR1",0.8864,"Cardiometabolic_II","B13805","20212097_SS211647","PASS",0.504,0.1022,"Plate control","PASS","PFC",1.07340908217526,"1h","1h"
1779,"PFC_23",75,"OID30085","P21817","RYR1",0.8864,"Cardiometabolic_II","B13805","20212097_SS211647","PASS",0.504,0.9916,"Plate control","PASS","PFC",1.9883889624319,"24h","24h"
1780,"PFC_08",85,"OID30085","P21817","RYR1",0.8864,"Cardiometabolic_II","B13805","20212097_SS211647","PASS",0.504,-0.2788,"Plate control","PASS","PFC",0.824276345998131,"Baseline","Baseline"
1781,"PFC_16",86,"OID30085","P21817","RYR1",0.8864,"Cardiometabolic_II","B13805","20212097_SS211647","PASS",0.504,0.3569,"Plate control","PASS","PFC",1.28067108889774,"1h","1h"
1782,"PFC_24",87,"OID30085","P21817","RYR1",0.8864,"Cardiometabolic_II","B13805","20212097_SS211647","PASS",0.504,0.2132,"Plate control","PASS","PFC",1.15925664786101,"24h","24h"
1783,"PFC_01",1,"OID30086","Q96HD9","ACY3",0,"Cardiometabolic_II","B13805","20212097_SS211647","WARN",-4.2927,0.1819,"Plate control","PASS","PFC",1.13437685319907,"Baseline","Baseline"
1784,"PFC_09",2,"OID30086","Q96HD9","ACY3",0,"Cardiometabolic_II","B13805","20212097_SS211647","PASS",-4.2927,-0.4869,"Plate control","PASS","PFC",0.713556711127075,"Baseline","Baseline"
1785,"PFC_17",3,"OID30086","Q96HD9","ACY3",0,"Cardiometabolic_II","B13805","20212097_SS211647","PASS",-4.2927,-0.165,"Plate control","PASS","PFC",0.891928519420093,"1h","1h"
1786,"PFC_25",4,"OID30086","Q96HD9","ACY3",0,"Cardiometabolic_II","B13805","20212097_SS211647","PASS",-4.2927,0.2694,"Plate control","PASS","PFC",1.20530645057879,"24h","24h"
1787,"PFC_02",13,"OID30086","Q96HD9","ACY3",0,"Cardiometabolic_II","B13805","20212097_SS211647","PASS",-4.2927,0.4696,"Plate control","PASS","PFC",1.38472548745537,"Baseline","Baseline"
1788,"PFC_10",14,"OID30086","Q96HD9","ACY3",0,"Cardiometabolic_II","B13805","20212097_SS211647","PASS",-4.2927,0.3218,"Plate control","PASS","PFC",1.24988901916447,"1h","1h"
1789,"PFC_18",15,"OID30086","Q96HD9","ACY3",0,"Cardiometabolic_II","B13805","20212097_SS211647","PASS",-4.2927,-0.0173,"Plate control","PASS","PFC",0.988080164642491,"1h","1h"
1790,"PFC_26",16,"OID30086","Q96HD9","ACY3",0,"Cardiometabolic_II","B13805","20212097_SS211647","PASS",-4.2927,-0.3682,"Plate control","PASS","PFC",0.774748520563688,"24h","24h"
1791,"PFC_03",25,"OID30086","Q96HD9","ACY3",0,"Cardiometabolic_II","B13805","20212097_SS211647","PASS",-4.2927,-0.0655,"Plate control","PASS","PFC",0.955614069597132,"Baseline","Baseline"
1792,"PFC_11",26,"OID30086","Q96HD9","ACY3",0,"Cardiometabolic_II","B13805","20212097_SS211647","PASS",-4.2927,1.0082,"Plate control","PASS","PFC",2.01139998071529,"1h","1h"

Supplemental table 1_Olink proximity extension assay (PEA) data

,"SampleID","Index","OlinkID","UniProt","Assay","MissingFreq","Panel","Panel_Lot_Nr","PlateID","QC_Warning","LOD","NPX","Normalization","Assay_Warning","condition","Value","group","alt_group"
1793,"PFC_19",27,"OID30086","Q96HD9","ACY3",0,"Cardiometabolic_II","B13805","20212097_SS211647","PASS",-4.2927,0.5595,"Plate control","PASS","PFC",1.47375836304483,"24h","24h"
1794,"PFC_27",28,"OID30086","Q96HD9","ACY3",0,"Cardiometabolic_II","B13805","20212097_SS211647","PASS",-4.2927,0.2579,"Plate control","PASS","PFC",1.19573691173667,"24h","24h"
1795,"PFC_04",37,"OID30086","Q96HD9","ACY3",0,"Cardiometabolic_II","B13805","20212097_SS211647","PASS",-4.2927,0.4759,"Plate control","PASS","PFC",1.39078556645946,"Baseline","Baseline"
1796,"PFC_12",38,"OID30086","Q96HD9","ACY3",0,"Cardiometabolic_II","B13805","20212097_SS211647","PASS",-4.2927,-0.3382,"Plate control","PASS","PFC",0.791027633869234,"1h","1h"
1797,"PFC_20",39,"OID30086","Q96HD9","ACY3",0,"Cardiometabolic_II","B13805","20212097_SS211647","PASS",-4.2927,0.9281,"Plate control","PASS","PFC",1.90276843795055,"24h","24h"
1798,"PFC_05",49,"OID30086","Q96HD9","ACY3",0,"Cardiometabolic_II","B13805","20212097_SS211647","PASS",-4.2927,-0.0696,"Plate control","PASS","PFC",0.952902162000121,"Baseline","Baseline"
1799,"PFC_13",50,"OID30086","Q96HD9","ACY3",0,"Cardiometabolic_II","B13805","20212097_SS211647","PASS",-4.2927,0.4592,"Plate control","PASS","PFC",1.3747792672369,"1h","1h"
1800,"PFC_21",51,"OID30086","Q96HD9","ACY3",0,"Cardiometabolic_II","B13805","20212097_SS211647","PASS",-4.2927,-0.9721,"Plate control","PASS","PFC",0.509763506160775,"24h","24h"
1801,"PFC_06",61,"OID30086","Q96HD9","ACY3",0,"Cardiometabolic_II","B13805","20212097_SS211647","PASS",-4.2927,0.4401,"Plate control","PASS","PFC",1.35669836335314,"Baseline","Baseline"
1802,"PFC_14",62,"OID30086","Q96HD9","ACY3",0,"Cardiometabolic_II","B13805","20212097_SS211647","PASS",-4.2927,0.1885,"Plate control","PASS","PFC",1.13957825651856,"1h","1h"
1803,"PFC_22",63,"OID30086","Q96HD9","ACY3",0,"Cardiometabolic_II","B13805","20212097_SS211647","PASS",-4.2927,0.4636,"Plate control","PASS","PFC",1.37897853478603,"24h","24h"
1804,"PFC_07",73,"OID30086","Q96HD9","ACY3",0,"Cardiometabolic_II","B13805","20212097_SS211647","PASS",-4.2927,-0.3232,"Plate control","PASS","PFC",0.79929501685417,"Baseline","Baseline"
1805,"PFC_15",74,"OID30086","Q96HD9","ACY3",0,"Cardiometabolic_II","B13805","20212097_SS211647","PASS",-4.2927,0.0922,"Plate control","PASS","PFC",1.0659945040422,"1h","1h"
1806,"PFC_23",75,"OID30086","Q96HD9","ACY3",0,"Cardiometabolic_II","B13805","20212097_SS211647","PASS",-4.2927,0.3616,"Plate control","PASS","PFC",1.28485005200361,"24h","24h"
1807,"PFC_08",85,"OID30086","Q96HD9","ACY3",0,"Cardiometabolic_II","B13805","20212097_SS211647","PASS",-4.2927,-0.2597,"Plate control","PASS","PFC",0.835261589135605,"Baseline","Baseline"
1808,"PFC_16",86,"OID30086","Q96HD9","ACY3",0,"Cardiometabolic_II","B13805","20212097_SS211647","PASS",-4.2927,-0.2702,"Plate control","PASS","PFC",0.829204585682062,"1h","1h"
1809,"PFC_24",87,"OID30086","Q96HD9","ACY3",0,"Cardiometabolic_II","B13805","20212097_SS211647","PASS",-4.2927,0.7012,"Plate control","PASS","PFC",1.62585657992728,"24h","24h"
1810,"PFC_01",1,"OID30098","Q96IW2","SHD",0.7955,"Cardiometabolic_II","B13805","20212097_SS211647","WARN",0.6686,-0.2039,"Plate control","PASS","PFC",0.868200404581703,"Baseline","Baseline"
1811,"PFC_09",2,"OID30098","Q96IW2","SHD",0.7955,"Cardiometabolic_II","B13805","20212097_SS211647","PASS",0.6686,-0.2321,"Plate control","PASS","PFC",0.851394691520625,"Baseline","Baseline"
1812,"PFC_17",3,"OID30098","Q96IW2","SHD",0.7955,"Cardiometabolic_II","B13805","20212097_SS211647","PASS",0.6686,1.0368,"Plate control","PASS","PFC",2.05167184884724,"1h","1h"
1813,"PFC_25",4,"OID30098","Q96IW2","SHD",0.7955,"Cardiometabolic_II","B13805","20212097_SS211647","PASS",0.6686,1.4819,"Plate control","PASS","PFC",2.79316345088764,"24h","24h"
1814,"PFC_02",13,"OID30098","Q96IW2","SHD",0.7955,"Cardiometabolic_II","B13805","20212097_SS211647","PASS",0.6686,-0.6452,"Plate control","PASS","PFC",0.639404144258391,"Baseline","Baseline"
1815,"PFC_10",14,"OID30098","Q96IW2","SHD",0.7955,"Cardiometabolic_II","B13805","20212097_SS211647","PASS",0.6686,-0.1401,"Plate control","PASS","PFC",0.907456253062831,"1h","1h"
1816,"PFC_18",15,"OID30098","Q96IW2","SHD",0.7955,"Cardiometabolic_II","B13805","20212097_SS211647","PASS",0.6686,0.1387,"Plate control","PASS","PFC",1.10091264594518,"1h","1h"
1817,"PFC_26",16,"OID30098","Q96IW2","SHD",0.7955,"Cardiometabolic_II","B13805","20212097_SS211647","PASS",0.6686,0.348,"Plate control","PASS","PFC",1.27279493527594,"24h","24h"
1818,"PFC_03",25,"OID30098","Q96IW2","SHD",0.7955,"Cardiometabolic_II","B13805","20212097_SS211647","PASS",0.6686,0.77,"Plate control","PASS","PFC",1.70526978353591,"Baseline","Baseline"
1819,"PFC_11",26,"OID30098","Q96IW2","SHD",0.7955,"Cardiometabolic_II","B13805","20212097_SS211647","PASS",0.6686,-0.2416,"Plate control","PASS","PFC",0.845806762299923,"1h","1h"
1820,"PFC_19",27,"OID30098","Q96IW2","SHD",0.7955,"Cardiometabolic_II","B13805","20212097_SS211647","PASS",0.6686,0.3404,"Plate control","PASS","PFC",1.26610758487146,"24h","24h"
1821,"PFC_27",28,"OID30098","Q96IW2","SHD",0.7955,"Cardiometabolic_II","B13805","20212097_SS211647","PASS",0.6686,0.2876,"Plate control","PASS","PFC",1.22060804134024,"24h","24h"
1822,"PFC_04",37,"OID30098","Q96IW2","SHD",0.7955,"Cardiometabolic_II","B13805","20212097_SS211647","PASS",0.6686,-0.2139,"Plate control","PASS","PFC",0.862203306325488,"Baseline","Baseline"
1823,"PFC_12",38,"OID30098","Q96IW2","SHD",0.7955,"Cardiometabolic_II","B13805","20212097_SS211647","PASS",0.6686,0.4804,"Plate control","PASS","PFC",1.39513042502004,"1h","1h"
1824,"PFC_20",39,"OID30098","Q96IW2","SHD",0.7955,"Cardiometabolic_II","B13805","20212097_SS211647","PASS",0.6686,-0.1564,"Plate control","PASS","PFC",0.897261242536529,"24h","24h"
1825,"PFC_05",49,"OID30098","Q96IW2","SHD",0.7955,"Cardiometabolic_II","B13805","20212097_SS211647","PASS",0.6686,-0.1834,"Plate control","PASS","PFC",0.880625179131911,"Baseline","Baseline"
1826,"PFC_13",50,"OID30098","Q96IW2","SHD",0.7955,"Cardiometabolic_II","B13805","20212097_SS211647","PASS",0.6686,-0.239,"Plate control","PASS","PFC",0.847332434946118,"1h","1h"
1827,"PFC_21",51,"OID30098","Q96IW2","SHD",0.7955,"Cardiometabolic_II","B13805","20212097_SS211647","PASS",0.6686,0.2486,"Plate control","PASS","PFC",1.18805366097021,"24h","24h"
1828,"PFC_06",61,"OID30098","Q96IW2","SHD",0.7955,"Cardiometabolic_II","B13805","20212097_SS211647","PASS",0.6686,0.8739,"Plate control","PASS","PFC",1.83261025808484,"Baseline","Baseline"
1829,"PFC_14",62,"OID30098","Q96IW2","SHD",0.7955,"Cardiometabolic_II","B13805","20212097_SS211647","PASS",0.6686,0.3558,"Plate control","PASS","PFC",1.27969499815108,"1h","1h"
1830,"PFC_22",63,"OID30098","Q96IW2","SHD",0.7955,"Cardiometabolic_II","B13805","20212097_SS211647","PASS",0.6686,-0.169,"Plate control","PASS","PFC",0.889458993537898,"24h","24h"
1831,"PFC_07",73,"OID30098","Q96IW2","SHD",0.7955,"Cardiometabolic_II","B13805","20212097_SS211647","PASS",0.6686,1.6898,"Plate control","PASS","PFC",3.22611977072245,"Baseline","Baseline"
1832,"PFC_15",74,"OID30098","Q96IW2","SHD",0.7955,"Cardiometabolic_II","B13805","20212097_SS211647","PASS",0.6686,-0.4597,"Plate control","PASS","PFC",0.727137446921617,"1h","1h"
1833,"PFC_23",75,"OID30098","Q96IW2","SHD",0.7955,"Cardiometabolic_II","B13805","20212097_SS211647","PASS",0.6686,-0.4562,"Plate control","PASS","PFC",0.728903634904,"24h","24h"
1834,"PFC_08",85,"OID30098","Q96IW2","SHD",0.7955,"Cardiometabolic_II","B13805","20212097_SS211647","PASS",0.6686,-0.3849,"Plate control","PASS","PFC",0.765832080142216,"Baseline","Baseline"
1835,"PFC_16",86,"OID30098","Q96IW2","SHD",0.7955,"Cardiometabolic_II","B13805","20212097_SS211647","PASS",0.6686,1.1502,"Plate control","PASS","PFC",2.21944660344099,"1h","1h"
1836,"PFC_24",87,"OID30098","Q96IW2","SHD",0.7955,"Cardiometabolic_II","B13805","20212097_SS211647","PASS",0.6686,0.3896,"Plate control","PASS","PFC",1.31003013602298,"24h","24h"
1837,"PFC_01",1,"OID30099","A6BM72","MEGF11",0,"Cardiometabolic_II","B13805","20212097_SS211647","WARN",-0.6643,0.6229,"Plate control","PASS","PFC",1.53996760235247,"Baseline","Baseline"
1838,"PFC_09",2,"OID30099","A6BM72","MEGF11",0,"Cardiometabolic_II","B13805","20212097_SS211647","PASS",-0.6643,0.7921,"Plate control","PASS","PFC",1.73159315177077,"Baseline","Baseline"
1839,"PFC_17",3,"OID30099","A6BM72","MEGF11",0,"Cardiometabolic_II","B13805","20212097_SS211647","PASS",-0.6643,0.6615,"Plate control","PASS","PFC",1.58172632279625,"1h","1h"
1840,"PFC_25",4,"OID30099","A6BM72","MEGF11",0,"Cardiometabolic_II","B13805","20212097_SS211647","PASS",-0.6643,0.6404,"Plate control","PASS","PFC",1.55876127980812,"24h","24h"
1841,"PFC_02",13,"OID30099","A6BM72","MEGF11",0,"Cardiometabolic_II","B13805","20212097_SS211647","PASS",-0.6643,0.4842,"Plate control","PASS","PFC",1.39880998553736,"Baseline","Baseline"
1842,"PFC_10",14,"OID30099","A6BM72","MEGF11",0,"Cardiometabolic_II","B13805","20212097_SS211647","PASS",-0.6643,0.7234,"Plate control","PASS","PFC",1.65106852696806,"1h","1h"
1843,"PFC_18",15,"OID30099","A6BM72","MEGF11",0,"Cardiometabolic_II","B13805","20212097_SS211647","PASS",-0.6643,0.2803,"Plate control","PASS","PFC",1.21444739537646,"1h","1h"
1844,"PFC_26",16,"OID30099","A6BM72","MEGF11",0,"Cardiometabolic_II","B13805","20212097_SS211647","PASS",-0.6643,0.5762,"Plate control","PASS","PFC",1.49091705746424,"24h","24h"
1845,"PFC_03",25,"OID30099","A6BM72","MEGF11",0,"Cardiometabolic_II","B13805","20212097_SS211647","PASS",-0.6643,0.5506,"Plate control","PASS","PFC",1.46469471870292,"Baseline","Baseline"
1846,"PFC_11",26,"OID30099","A6BM72","MEGF11",0,"Cardiometabolic_II","B13805","20212097_SS211647","PASS",-0.6643,0.4706,"Plate control","PASS","PFC",1.38568563874749,"1h","1h"
1847,"PFC_19",27,"OID30099","A6BM72","MEGF11",0,"Cardiometabolic_II","B13805","20212097_SS211647","PASS",-0.6643,0.4639,"Plate control","PASS","PFC",1.37926531512718,"24h","24h"
1848,"PFC_27",28,"OID30099","A6BM72","MEGF11",0,"Cardiometabolic_II","B13805","20212097_SS211647","PASS",-0.6643,0.9748,"Plate control","PASS","PFC",1.96536872024684,"24h","24h"
1849,"PFC_04",37,"OID30099","A6BM72","MEGF11",0,"Cardiometabolic_II","B13805","20212097_SS211647","PASS",-0.6643,0.8185,"Plate control","PASS","PFC",1.7635714171902,"Baseline","Baseline"
1850,"PFC_12",38,"OID30099","A6BM72","MEGF11",0,"Cardiometabolic_II","B13805","20212097_SS211647","PASS",-0.6643,0.0079,"Plate control","PASS","PFC",1.00549088266592,"1h","1h"
1851,"PFC_20",39,"OID30099","A6BM72","MEGF11",0,"Cardiometabolic_II","B13805","20212097_SS211647","PASS",-0.6643,0.6006,"Plate control","PASS","PFC",1.51634706640884,"24h","24h"
1852,"PFC_05",49,"OID30099","A6BM72","MEGF11",0,"Cardiometabolic_II","B13805","20212097_SS211647","PASS",-0.6643,0.2609,"Plate control","PASS","PFC",1.19822596376605,"Baseline","Baseline"
1853,"PFC_13",50,"OID30099","A6BM72","MEGF11",0,"Cardiometabolic_II","B13805","20212097_SS211647","PASS",-0.6643,0.479,"Plate control","PASS","PFC",1.39377723868747,"1h","1h"
1854,"PFC_21",51,"OID30099","A6BM72","MEGF11",0,"Cardiometabolic_II","B13805","20212097_SS211647","PASS",-0.6643,0.1448,"Plate control","PASS","PFC",1.10557737712785,"24h","24h"
1855,"PFC_06",61,"OID30099","A6BM72","MEGF11",0,"Cardiometabolic_II","B13805","20212097_SS211647","PASS",-0.6643,0.2641,"Plate control","PASS","PFC",1.20088666372094,"Baseline","Baseline"
1856,"PFC_14",62,"OID30099","A6BM72","MEGF11",0,"Cardiometabolic_II","B13805","20212097_SS211647","PASS",-0.6643,0.295,"Plate control","PASS","PFC",1.2268849772538,"1h","1h"
1857,"PFC_22",63,"OID30099","A6BM72","MEGF11",0,"Cardiometabolic_II","B13805","20212097_SS211647","PASS",-0.6643,0.2004,"Plate control","PASS","PFC",1.14901688596319,"24h","24h"
1858,"PFC_07",73,"OID30099","A6BM72","MEGF11",0,"Cardiometabolic_II","B13805","20212097_SS211647","PASS",-0.6643,0.6435,"Plate control","PASS","PFC",1.56211427896439,"Baseline","Baseline"
1859,"PFC_15",74,"OID30099","A6BM72","MEGF11",0,"Cardiometabolic_II","B13805","20212097_SS211647","PASS",-0.6643,0.2166,"Plate control","PASS","PFC",1.16199189029035,"1h","1h"
1860,"PFC_23",75,"OID30099","A6BM72","MEGF11",0,"Cardiometabolic_II","B13805","20212097_SS211647","PASS",-0.6643,0.3115,"Plate control","PASS","PFC",1.24099732009064,"24h","24h"
1861,"PFC_08",85,"OID30099","A6BM72","MEGF11",0,"Cardiometabolic_II","B13805","20212097_SS211647","PASS",-0.6643,0.3674,"Plate control","PASS","PFC",1.29002587219519,"Baseline","Baseline"
1862,"PFC_16",86,"OID30099","A6BM72","MEGF11",0,"Cardiometabolic_II","B13805","20212097_SS211647","PASS",-0.6643,0.2209,"Plate control","PASS","PFC",1.1654604117749,"1h","1h"
1863,"PFC_24",87,"OID30099","A6BM72","MEGF11",0,"Cardiometabolic_II","B13805","20212097_SS211647","PASS",-0.6643,0.1712,"Plate control","PASS","PFC",1.12599467132386,"24h","24h"
1864,"PFC_01",1,"OID30100","Q9UKX7","NUP50",0.9545,"Cardiometabolic_II","B13805","20212097_SS211647","WARN",0.8425,0.409,"Plate control","PASS","PFC",1.32776515838244,"Baseline","Baseline"
1865,"PFC_09",2,"OID30100","Q9UKX7","NUP50",0.9545,"Cardiometabolic_II","B13805","20212097_SS211647","PASS",0.8425,0.1312,"Plate control","PASS","PFC",1.09520428782303,"Baseline","Baseline"
1866,"PFC_17",3,"OID30100","Q9UKX7","NUP50",0.9545,"Cardiometabolic_II","B13805","20212097_SS211647","PASS",0.8425,0.6402,"Plate control","PASS","PFC",1.5585452045884,"1h","1h"
1867,"PFC_25",4,"OID30100","Q9UKX7","NUP50",0.9545,"Cardiometabolic_II","B13805","20212097_SS211647","PASS",0.8425,-0.05,"Plate control","PASS","PFC",0.965936328924846,"24h","24h"
1868,"PFC_02",13,"OID30100","Q9UKX7","NUP50",0.9545,"Cardiometabolic_II","B13805","20212097_SS211647","PASS",0.8425,0.304,"Plate control","PASS","PFC",1.23456260689354,"Baseline","Baseline"
1869,"PFC_10",14,"OID30100","Q9UKX7","NUP50",0.9545,"Cardiometabolic_II","B13805","20212097_SS211647","PASS",0.8425,0.2898,"Plate control","PASS","PFC",1.22247079550769,"1h","1h"
1870,"PFC_18",15,"OID30100","Q9UKX7","NUP50",0.9545,"Cardiometabolic_II","B13805","20212097_SS211647","PASS",0.8425,-0.0399,"Plate control","PASS","PFC",0.97272236905236,"1h","1h"
1871,"PFC_26",16,"OID30100","Q9UKX7","NUP50",0.9545,"Cardiometabolic_II","B13805","20212097_SS211647","PASS",0.8425,0.4142,"Plate control","PASS","PFC",1.33255954426618,"24h","24h"
1872,"PFC_03",25,"OID30100","Q9UKX7","NUP50",0.9545,"Cardiometabolic_II","B13805","20212097_SS211647","PASS",0.8425,0.4437,"Plate control","PASS","PFC",1.3600880006494,"Baseline","Baseline"
1873,"PFC_11",26,"OID30100","Q9UKX7","NUP50",0.9545,"Cardiometabolic_II","B13805","20212097_SS211647","PASS",0.8425,0.4674,"Plate control","PASS","PFC",1.38261549580404,"1h","1h"
1874,"PFC_19",27,"OID30100","Q9UKX7","NUP50",0.9545,"Cardiometabolic_II","B13805","20212097_SS211647","PASS",0.8425,0.3098,"Plate control","PASS","PFC",1.23953585187148,"24h","24h"
1875,"PFC_27",28,"OID30100","Q9UKX7","NUP50",0.9545,"Cardiometabolic_II","B13805","20212097_SS211647","PASS",0.8425,0.4245,"Plate control","PASS","PFC",1.34210728322883,"24h","24h"
1876,"PFC_04",37,"OID30100","Q9UKX7","NUP50",0.9545,"Cardiometabolic_II","B13805","20212097_SS211647","PASS",0.8425,0.1529,"Plate control","PASS","PFC",1.11180209059776,"Baseline","Baseline"
1877,"PFC_12",38,"OID30100","Q9UKX7","NUP50",0.9545,"Cardiometabolic_II","B13805","20212097_SS211647","PASS",0.8425,0.3562,"Plate control","PASS","PFC",1.28004985413427,"1h","1h"
1878,"PFC_20",39,"OID30100","Q9UKX7","NUP50",0.9545,"Cardiometabolic_II","B13805","20212097_SS211647","PASS",0.8425,0.3713,"Plate control","PASS","PFC",1.29351788340524,"24h","24h"
1879,"PFC_05",49,"OID30100","Q9UKX7","NUP50",0.9545,"Cardiometabolic_II","B13805","20212097_SS211647","PASS",0.8425,0.4662,"Plate control","PASS","PFC",1.38146594671508,"Baseline","Baseline"
1880,"PFC_13",50,"OID30100","Q9UKX7","NUP50",0.9545,"Cardiometabolic_II","B13805","20212097_SS211647","PASS",0.8425,-0.0435,"Plate control","PASS","PFC",0.970298131782807,"1h","1h"
1881,"PFC_21",51,"OID30100","Q9UKX7","NUP50",0.9545,"Cardiometabolic_II","B13805","20212097_SS211647","PASS",0.8425,0.2386,"Plate control","PASS","PFC",1.17984717488601,"24h","24h"
1882,"PFC_06",61,"OID30100","Q9UKX7","NUP50",0.9545,"Cardiometabolic_II","B13805","20212097_SS211647","PASS",0.8425,0.2235,"Plate control","PASS","PFC",1.16756267809592,"Baseline","Baseline"
1883,"PFC_14",62,"OID30100","Q9UKX7","NUP50",0.9545,"Cardiometabolic_II","B13805","20212097_SS211647","PASS",0.8425,0.6027,"Plate control","PASS","PFC",1.51855588216202,"1h","1h"
1884,"PFC_22",63,"OID30100","Q9UKX7","NUP50",0.9545,"Cardiometabolic_II","B13805","20212097_SS211647","PASS",0.8425,0.1721,"Plate control","PASS","PFC",1.12669732249796,"24h","24h"
1885,"PFC_07",73,"OID30100","Q9UKX7","NUP50",0.9545,"Cardiometabolic_II","B13805","20212097_SS211647","PASS",0.8425,0.3736,"Plate control","PASS","PFC",1.29558170410928,"Baseline","Baseline"
1886,"PFC_15",74,"OID30100","Q9UKX7","NUP50",0.9545,"Cardiometabolic_II","B13805","20212097_SS211647","PASS",0.8425,-0.1075,"Plate control","PASS","PFC",0.928195108644738,"1h","1h"
1887,"PFC_23",75,"OID30100","Q9UKX7","NUP50",0.9545,"Cardiometabolic_II","B13805","20212097_SS211647","PASS",0.8425,0.784,"Plate control","PASS","PFC",1.72189837667781,"24h","24h"
1888,"PFC_08",85,"OID30100","Q9UKX7","NUP50",0.9545,"Cardiometabolic_II","B13805","20212097_SS211647","PASS",0.8425,0.2116,"Plate control","PASS","PFC",1.1579717037538,"Baseline","Baseline"
1889,"PFC_16",86,"OID30100","Q9UKX7","NUP50",0.9545,"Cardiometabolic_II","B13805","20212097_SS211647","PASS",0.8425,0.1223,"Plate control","PASS","PFC",1.08846875885472,"1h","1h"
1890,"PFC_24",87,"OID30100","Q9UKX7","NUP50",0.9545,"Cardiometabolic_II","B13805","20212097_SS211647","PASS",0.8425,0.2185,"Plate control","PASS","PFC",1.16352321809771,"24h","24h"
1891,"PFC_01",1,"OID30101","Q96LB8","PGLYRP4",0.6818,"Cardiometabolic_II","B13805","20212097_SS211647","WARN",0.3625,-0.2429,"Plate control","PASS","PFC",0.845044956435438,"Baseline","Baseline"
1892,"PFC_09",2,"OID30101","Q96LB8","PGLYRP4",0.6818,"Cardiometabolic_II","B13805","20212097_SS211647","PASS",0.3625,0.0566,"Plate control","PASS","PFC",1.04001187402485,"Baseline","Baseline"
1893,"PFC_17",3,"OID30101","Q96LB8","PGLYRP4",0.6818,"Cardiometabolic_II","B13805","20212097_SS211647","PASS",0.3625,0.1056,"Plate control","PASS","PFC",1.07594176902336,"1h","1h"
1894,"PFC_25",4,"OID30101","Q96LB8","PGLYRP4",0.6818,"Cardiometabolic_II","B13805","20212097_SS211647","PASS",0.3625,1.6017,"Plate control","PASS","PFC",3.03500732829375,"24h","24h"
1895,"PFC_02",13,"OID30101","Q96LB8","PGLYRP4",0.6818,"Cardiometabolic_II","B13805","20212097_SS211647","PASS",0.3625,2.2982,"Plate control","PASS","PFC",4.91843726194312,"Baseline","Baseline"
1896,"PFC_10",14,"OID30101","Q96LB8","PGLYRP4",0.6818,"Cardiometabolic_II","B13805","20212097_SS211647","PASS",0.3625,-0.8124,"Plate control","PASS","PFC",0.569433786152785,"1h","1h"
1897,"PFC_18",15,"OID30101","Q96LB8","PGLYRP4",0.6818,"Cardiometabolic_II","B13805","20212097_SS211647","PASS",0.3625,-0.0115,"Plate control","PASS","PFC",0.992060493132249,"1h","1h"
1898,"PFC_26",16,"OID30101","Q96LB8","PGLYRP4",0.6818,"Cardiometabolic_II","B13805","20212097_SS211647","PASS",0.3625,0.093,"Plate control","PASS","PFC",1.06658578083176,"24h","24h"
1899,"PFC_03",25,"OID30101","Q96LB8","PGLYRP4",0.6818,"Cardiometabolic_II","B13805","20212097_SS211647","PASS",0.3625,0.2258,"Plate control","PASS","PFC",1.16942553601172,"Baseline","Baseline"
1900,"PFC_11",26,"OID30101","Q96LB8","PGLYRP4",0.6818,"Cardiometabolic_II","B13805","20212097_SS211647","PASS",0.3625,2.3124,"Plate control","PASS","PFC",4.96708694413031,"1h","1h"
1901,"PFC_19",27,"OID30101","Q96LB8","PGLYRP4",0.6818,"Cardiometabolic_II","B13805","20212097_SS211647","PASS",0.3625,0.3425,"Plate control","PASS","PFC",1.26795188453123,"24h","24h"
1902,"PFC_27",28,"OID30101","Q96LB8","PGLYRP4",0.6818,"Cardiometabolic_II","B13805","20212097_SS211647","PASS",0.3625,0.6059,"Plate control","PASS","PFC",1.52192788518094,"24h","24h"
1903,"PFC_04",37,"OID30101","Q96LB8","PGLYRP4",0.6818,"Cardiometabolic_II","B13805","20212097_SS211647","PASS",0.3625,0.0168,"Plate control","PASS","PFC",1.01171293811075,"Baseline","Baseline"
1904,"PFC_12",38,"OID30101","Q96LB8","PGLYRP4",0.6818,"Cardiometabolic_II","B13805","20212097_SS211647","PASS",0.3625,0.2109,"Plate control","PASS","PFC",1.15740998866261,"1h","1h"

Supplemental table 1_Olink proximity extension assay (PEA) data

,"SampleID","Index","OlinkID","UniProt","Assay","MissingFreq","Panel","Panel_Lot_Nr","PlateID","QC_Warning","LOD","NPX","Normalization","Assay_Warning","condition","Value","group","alt_group"
1905,"PFC_20",39,"OID30101","Q96LB8","PGLYRP4",0.6818,"Cardiometabolic_II","B13805","20212097_SS211647","PASS",0.3625,2.6945,"Plate control","PASS","PFC",6.47329387087239,"24h","24h"
1906,"PFC_05",49,"OID30101","Q96LB8","PGLYRP4",0.6818,"Cardiometabolic_II","B13805","20212097_SS211647","PASS",0.3625,0.2176,"Plate control","PASS","PFC",1.1627976008987,"Baseline","Baseline"
1907,"PFC_13",50,"OID30101","Q96LB8","PGLYRP4",0.6818,"Cardiometabolic_II","B13805","20212097_SS211647","PASS",0.3625,-0.3137,"Plate control","PASS","PFC",0.804575660353042,"1h","1h"
1908,"PFC_21",51,"OID30101","Q96LB8","PGLYRP4",0.6818,"Cardiometabolic_II","B13805","20212097_SS211647","PASS",0.3625,0.4542,"Plate control","PASS","PFC",1.37002289230256,"24h","24h"
1909,"PFC_06",61,"OID30101","Q96LB8","PGLYRP4",0.6818,"Cardiometabolic_II","B13805","20212097_SS211647","PASS",0.3625,0.3682,"Plate control","PASS","PFC",1.29074141280376,"Baseline","Baseline"
1910,"PFC_14",62,"OID30101","Q96LB8","PGLYRP4",0.6818,"Cardiometabolic_II","B13805","20212097_SS211647","PASS",0.3625,0.2643,"Plate control","PASS","PFC",1.20105315350189,"1h","1h"
1911,"PFC_22",63,"OID30101","Q96LB8","PGLYRP4",0.6818,"Cardiometabolic_II","B13805","20212097_SS211647","PASS",0.3625,-0.0627,"Plate control","PASS","PFC",0.957470537896994,"24h","24h"
1912,"PFC_07",73,"OID30101","Q96LB8","PGLYRP4",0.6818,"Cardiometabolic_II","B13805","20212097_SS211647","PASS",0.3625,1.41,"Plate control","PASS","PFC",2.65737162819302,"Baseline","Baseline"
1913,"PFC_15",74,"OID30101","Q96LB8","PGLYRP4",0.6818,"Cardiometabolic_II","B13805","20212097_SS211647","PASS",0.3625,-0.3285,"Plate control","PASS","PFC",0.796364049705242,"1h","1h"
1914,"PFC_23",75,"OID30101","Q96LB8","PGLYRP4",0.6818,"Cardiometabolic_II","B13805","20212097_SS211647","PASS",0.3625,0.1186,"Plate control","PASS","PFC",1.08568079995476,"24h","24h"
1915,"PFC_08",85,"OID30101","Q96LB8","PGLYRP4",0.6818,"Cardiometabolic_II","B13805","20212097_SS211647","PASS",0.3625,0.0168,"Plate control","PASS","PFC",1.01171293811075,"Baseline","Baseline"
1916,"PFC_16",86,"OID30101","Q96LB8","PGLYRP4",0.6818,"Cardiometabolic_II","B13805","20212097_SS211647","PASS",0.3625,1.3742,"Plate control","PASS","PFC",2.59224126690391,"1h","1h"
1917,"PFC_24",87,"OID30101","Q96LB8","PGLYRP4",0.6818,"Cardiometabolic_II","B13805","20212097_SS211647","PASS",0.3625,-0.3565,"Plate control","PASS","PFC",0.781057139482197,"24h","24h"
1918,"PFC_01",1,"OID30102","Q9NV35","NUDT15",0.6591,"Cardiometabolic_II","B13805","20212097_SS211647","WARN",0.0753,-0.3719,"Plate control","PASS","PFC",0.772764111718173,"Baseline","Baseline"
1919,"PFC_09",2,"OID30102","Q9NV35","NUDT15",0.6591,"Cardiometabolic_II","B13805","20212097_SS211647","PASS",0.0753,-0.5362,"Plate control","PASS","PFC",0.689584857527011,"Baseline","Baseline"
1920,"PFC_17",3,"OID30102","Q9NV35","NUDT15",0.6591,"Cardiometabolic_II","B13805","20212097_SS211647","PASS",0.0753,0.2055,"Plate control","PASS","PFC",1.15308590667899,"1h","1h"
1921,"PFC_25",4,"OID30102","Q9NV35","NUDT15",0.6591,"Cardiometabolic_II","B13805","20212097_SS211647","PASS",0.0753,-0.3808,"Plate control","PASS","PFC",0.768011596486069,"24h","24h"
1922,"PFC_02",13,"OID30102","Q9NV35","NUDT15",0.6591,"Cardiometabolic_II","B13805","20212097_SS211647","PASS",0.0753,-0.3649,"Plate control","PASS","PFC",0.776522697587517,"Baseline","Baseline"
1923,"PFC_10",14,"OID30102","Q9NV35","NUDT15",0.6591,"Cardiometabolic_II","B13805","20212097_SS211647","PASS",0.0753,-0.4703,"Plate control","PASS","PFC",0.721814485051698,"1h","1h"
1924,"PFC_18",15,"OID30102","Q9NV35","NUDT15",0.6591,"Cardiometabolic_II","B13805","20212097_SS211647","PASS",0.0753,-1.0962,"Plate control","PASS","PFC",0.467746900931765,"1h","1h"
1925,"PFC_26",16,"OID30102","Q9NV35","NUDT15",0.6591,"Cardiometabolic_II","B13805","20212097_SS211647","PASS",0.0753,0.4412,"Plate control","PASS","PFC",1.3577331886204,"24h","24h"
1926,"PFC_03",25,"OID30102","Q9NV35","NUDT15",0.6591,"Cardiometabolic_II","B13805","20212097_SS211647","PASS",0.0753,-0.4938,"Plate control","PASS","PFC",0.710152120436144,"Baseline","Baseline"
1927,"PFC_11",26,"OID30102","Q9NV35","NUDT15",0.6591,"Cardiometabolic_II","B13805","20212097_SS211647","PASS",0.0753,-0.5537,"Plate control","PASS","PFC",0.681270668844923,"1h","1h"
1928,"PFC_19",27,"OID30102","Q9NV35","NUDT15",0.6591,"Cardiometabolic_II","B13805","20212097_SS211647","PASS",0.0753,0.0719,"Plate control","PASS","PFC",1.05110004985902,"24h","24h"
1929,"PFC_27",28,"OID30102","Q9NV35","NUDT15",0.6591,"Cardiometabolic_II","B13805","20212097_SS211647","PASS",0.0753,-0.2573,"Plate control","PASS","PFC",0.836652247649423,"24h","24h"
1930,"PFC_04",37,"OID30102","Q9NV35","NUDT15",0.6591,"Cardiometabolic_II","B13805","20212097_SS211647","PASS",0.0753,0.7133,"Plate control","PASS","PFC",1.6395501146916,"Baseline","Baseline"
1931,"PFC_12",38,"OID30102","Q9NV35","NUDT15",0.6591,"Cardiometabolic_II","B13805","20212097_SS211647","PASS",0.0753,-0.3313,"Plate control","PASS","PFC",0.794819955600086,"1h","1h"
1932,"PFC_20",39,"OID30102","Q9NV35","NUDT15",0.6591,"Cardiometabolic_II","B13805","20212097_SS211647","PASS",0.0753,-0.2689,"Plate control","PASS","PFC",0.829952111492872,"24h","24h"
1933,"PFC_05",49,"OID30102","Q9NV35","NUDT15",0.6591,"Cardiometabolic_II","B13805","20212097_SS211647","PASS",0.0753,-0.6225,"Plate control","PASS","PFC",0.649544377286886,"Baseline","Baseline"
1934,"PFC_13",50,"OID30102","Q9NV35","NUDT15",0.6591,"Cardiometabolic_II","B13805","20212097_SS211647","PASS",0.0753,0.1704,"Plate control","PASS","PFC",1.12537046038248,"1h","1h"
1935,"PFC_21",51,"OID30102","Q9NV35","NUDT15",0.6591,"Cardiometabolic_II","B13805","20212097_SS211647","PASS",0.0753,-0.4836,"Plate control","PASS","PFC",0.715190758677317,"24h","24h"
1936,"PFC_06",61,"OID30102","Q9NV35","NUDT15",0.6591,"Cardiometabolic_II","B13805","20212097_SS211647","PASS",0.0753,-0.4479,"Plate control","PASS","PFC",0.733109192090477,"Baseline","Baseline"
1937,"PFC_14",62,"OID30102","Q9NV35","NUDT15",0.6591,"Cardiometabolic_II","B13805","20212097_SS211647","PASS",0.0753,-0.4756,"Plate control","PASS","PFC",0.719167634391184,"1h","1h"
1938,"PFC_22",63,"OID30102","Q9NV35","NUDT15",0.6591,"Cardiometabolic_II","B13805","20212097_SS211647","PASS",0.0753,-0.0972,"Plate control","PASS","PFC",0.93484559165083,"24h","24h"
1939,"PFC_07",73,"OID30102","Q9NV35","NUDT15",0.6591,"Cardiometabolic_II","B13805","20212097_SS211647","PASS",0.0753,0.1431,"Plate control","PASS","PFC",1.1042753870475,"Baseline","Baseline"
1940,"PFC_15",74,"OID30102","Q9NV35","NUDT15",0.6591,"Cardiometabolic_II","B13805","20212097_SS211647","PASS",0.0753,-0.0974,"Plate control","PASS","PFC",0.934716003516181,"1h","1h"
1941,"PFC_23",75,"OID30102","Q9NV35","NUDT15",0.6591,"Cardiometabolic_II","B13805","20212097_SS211647","PASS",0.0753,-0.1859,"Plate control","PASS","PFC",0.879100493402846,"24h","24h"
1942,"PFC_08",85,"OID30102","Q9NV35","NUDT15",0.6591,"Cardiometabolic_II","B13805","20212097_SS211647","PASS",0.0753,0.2612,"Plate control","PASS","PFC",1.19847515375849,"Baseline","Baseline"
1943,"PFC_16",86,"OID30102","Q9NV35","NUDT15",0.6591,"Cardiometabolic_II","B13805","20212097_SS211647","PASS",0.0753,-0.6095,"Plate control","PASS","PFC",0.655423815155959,"1h","1h"
1944,"PFC_24",87,"OID30102","Q9NV35","NUDT15",0.6591,"Cardiometabolic_II","B13805","20212097_SS211647","PASS",0.0753,0.1981,"Plate control","PASS","PFC",1.14718653838185,"24h","24h"
1945,"PFC_01",1,"OID30103","Q10587","TEF",0.3864,"Cardiometabolic_II","B13805","20212097_SS211647","WARN",-1.2376,-1.3531,"Plate control","PASS","PFC",0.391450013940534,"Baseline","Baseline"
1946,"PFC_09",2,"OID30103","Q10587","TEF",0.3864,"Cardiometabolic_II","B13805","20212097_SS211647","PASS",-1.2376,-1.8123,"Plate control","PASS","PFC",0.284736628831543,"Baseline","Baseline"
1947,"PFC_17",3,"OID30103","Q10587","TEF",0.3864,"Cardiometabolic_II","B13805","20212097_SS211647","PASS",-1.2376,-0.9427,"Plate control","PASS","PFC",0.52025830668712,"1h","1h"
1948,"PFC_25",4,"OID30103","Q10587","TEF",0.3864,"Cardiometabolic_II","B13805","20212097_SS211647","PASS",-1.2376,-1.0747,"Plate control","PASS","PFC",0.474769775810721,"24h","24h"
1949,"PFC_02",13,"OID30103","Q10587","TEF",0.3864,"Cardiometabolic_II","B13805","20212097_SS211647","PASS",-1.2376,-1.1821,"Plate control","PASS","PFC",0.440709530238026,"Baseline","Baseline"
1950,"PFC_10",14,"OID30103","Q10587","TEF",0.3864,"Cardiometabolic_II","B13805","20212097_SS211647","PASS",-1.2376,-1.4786,"Plate control","PASS","PFC",0.358836860563883,"1h","1h"
1951,"PFC_18",15,"OID30103","Q10587","TEF",0.3864,"Cardiometabolic_II","B13805","20212097_SS211647","PASS",-1.2376,-0.899,"Plate control","PASS","PFC",0.536258308408971,"1h","1h"
1952,"PFC_26",16,"OID30103","Q10587","TEF",0.3864,"Cardiometabolic_II","B13805","20212097_SS211647","PASS",-1.2376,-1.2589,"Plate control","PASS","PFC",0.41786244247452,"24h","24h"
1953,"PFC_03",25,"OID30103","Q10587","TEF",0.3864,"Cardiometabolic_II","B13805","20212097_SS211647","PASS",-1.2376,-1.4868,"Plate control","PASS","PFC",0.356803086411758,"Baseline","Baseline"
1954,"PFC_11",26,"OID30103","Q10587","TEF",0.3864,"Cardiometabolic_II","B13805","20212097_SS211647","PASS",-1.2376,-1.7287,"Plate control","PASS","PFC",0.301723715089987,"1h","1h"
1955,"PFC_19",27,"OID30103","Q10587","TEF",0.3864,"Cardiometabolic_II","B13805","20212097_SS211647","PASS",-1.2376,-1.0757,"Plate control","PASS","PFC",0.47444080450514,"24h","24h"
1956,"PFC_27",28,"OID30103","Q10587","TEF",0.3864,"Cardiometabolic_II","B13805","20212097_SS211647","PASS",-1.2376,-1.2131,"Plate control","PASS","PFC",0.431340772971045,"24h","24h"
1957,"PFC_04",37,"OID30103","Q10587","TEF",0.3864,"Cardiometabolic_II","B13805","20212097_SS211647","PASS",-1.2376,-0.6954,"Plate control","PASS","PFC",0.617538076914668,"Baseline","Baseline"
1958,"PFC_12",38,"OID30103","Q10587","TEF",0.3864,"Cardiometabolic_II","B13805","20212097_SS211647","PASS",-1.2376,-1.4966,"Plate control","PASS","PFC",0.354387592611244,"1h","1h"
1959,"PFC_20",39,"OID30103","Q10587","TEF",0.3864,"Cardiometabolic_II","B13805","20212097_SS211647","PASS",-1.2376,-1.0402,"Plate control","PASS","PFC",0.486260059075631,"24h","24h"
1960,"PFC_05",49,"OID30103","Q10587","TEF",0.3864,"Cardiometabolic_II","B13805","20212097_SS211647","PASS",-1.2376,-1.1838,"Plate control","PASS","PFC",0.440190525916343,"Baseline","Baseline"
1961,"PFC_13",50,"OID30103","Q10587","TEF",0.3864,"Cardiometabolic_II","B13805","20212097_SS211647","PASS",-1.2376,-1.3995,"Plate control","PASS","PFC",0.37906049122051,"1h","1h"
1962,"PFC_21",51,"OID30103","Q10587","TEF",0.3864,"Cardiometabolic_II","B13805","20212097_SS211647","PASS",-1.2376,-1.9955,"Plate control","PASS","PFC",0.250781007990261,"24h","24h"
1963,"PFC_06",61,"OID30103","Q10587","TEF",0.3864,"Cardiometabolic_II","B13805","20212097_SS211647","PASS",-1.2376,-1.3575,"Plate control","PASS","PFC",0.390257969757696,"Baseline","Baseline"
1964,"PFC_14",62,"OID30103","Q10587","TEF",0.3864,"Cardiometabolic_II","B13805","20212097_SS211647","PASS",-1.2376,-1.612,"Plate control","PASS","PFC",0.327144517863753,"1h","1h"
1965,"PFC_22",63,"OID30103","Q10587","TEF",0.3864,"Cardiometabolic_II","B13805","20212097_SS211647","PASS",-1.2376,-1.0977,"Plate control","PASS","PFC",0.467260827497481,"24h","24h"
1966,"PFC_07",73,"OID30103","Q10587","TEF",0.3864,"Cardiometabolic_II","B13805","20212097_SS211647","PASS",-1.2376,-1.0938,"Plate control","PASS","PFC",0.468525670383819,"Baseline","Baseline"
1967,"PFC_15",74,"OID30103","Q10587","TEF",0.3864,"Cardiometabolic_II","B13805","20212097_SS211647","PASS",-1.2376,-1.4912,"Plate control","PASS","PFC",0.355716549105771,"1h","1h"
1968,"PFC_23",75,"OID30103","Q10587","TEF",0.3864,"Cardiometabolic_II","B13805","20212097_SS211647","PASS",-1.2376,-1.3562,"Plate control","PASS","PFC",0.390609786318175,"24h","24h"
1969,"PFC_08",85,"OID30103","Q10587","TEF",0.3864,"Cardiometabolic_II","B13805","20212097_SS211647","PASS",-1.2376,-1.2102,"Plate control","PASS","PFC",0.432208694652999,"Baseline","Baseline"
1970,"PFC_16",86,"OID30103","Q10587","TEF",0.3864,"Cardiometabolic_II","B13805","20212097_SS211647","PASS",-1.2376,-1.6623,"Plate control","PASS","PFC",0.315935070930993,"1h","1h"
1971,"PFC_24",87,"OID30103","Q10587","TEF",0.3864,"Cardiometabolic_II","B13805","20212097_SS211647","PASS",-1.2376,-1.0337,"Plate control","PASS","PFC",0.488455825454715,"24h","24h"
1972,"PFC_01",1,"OID30104","Q13296","SCGB2A2",0.7955,"Cardiometabolic_II","B13805","20212097_SS211647","WARN",0.1924,-0.6201,"Plate control","PASS","PFC",0.650625828212112,"Baseline","Baseline"
1973,"PFC_09",2,"OID30104","Q13296","SCGB2A2",0.7955,"Cardiometabolic_II","B13805","20212097_SS211647","PASS",0.1924,-0.0183,"Plate control","PASS","PFC",0.987395516970415,"Baseline","Baseline"
1974,"PFC_17",3,"OID30104","Q13296","SCGB2A2",0.7955,"Cardiometabolic_II","B13805","20212097_SS211647","PASS",0.1924,0.039,"Plate control","PASS","PFC",1.02740143937896,"1h","1h"
1975,"PFC_25",4,"OID30104","Q13296","SCGB2A2",0.7955,"Cardiometabolic_II","B13805","20212097_SS211647","PASS",0.1924,-0.1112,"Plate control","PASS","PFC",0.925817668049411,"24h","24h"
1976,"PFC_02",13,"OID30104","Q13296","SCGB2A2",0.7955,"Cardiometabolic_II","B13805","20212097_SS211647","PASS",0.1924,-0.2972,"Plate control","PASS","PFC",0.813830356406529,"Baseline","Baseline"
1977,"PFC_10",14,"OID30104","Q13296","SCGB2A2",0.7955,"Cardiometabolic_II","B13805","20212097_SS211647","PASS",0.1924,0.5292,"Plate control","PASS","PFC",1.44312873311767,"1h","1h"
1978,"PFC_18",15,"OID30104","Q13296","SCGB2A2",0.7955,"Cardiometabolic_II","B13805","20212097_SS211647","PASS",0.1924,-0.1877,"Plate control","PASS","PFC",0.878004354500895,"1h","1h"
1979,"PFC_26",16,"OID30104","Q13296","SCGB2A2",0.7955,"Cardiometabolic_II","B13805","20212097_SS211647","PASS",0.1924,0.3715,"Plate control","PASS","PFC",1.29369721549008,"24h","24h"
1980,"PFC_03",25,"OID30104","Q13296","SCGB2A2",0.7955,"Cardiometabolic_II","B13805","20212097_SS211647","PASS",0.1924,0.016,"Plate control","PASS","PFC",1.0111520808513,"Baseline","Baseline"
1981,"PFC_11",26,"OID30104","Q13296","SCGB2A2",0.7955,"Cardiometabolic_II","B13805","20212097_SS211647","PASS",0.1924,0.1969,"Plate control","PASS","PFC",1.1462327321761,"1h","1h"
1982,"PFC_19",27,"OID30104","Q13296","SCGB2A2",0.7955,"Cardiometabolic_II","B13805","20212097_SS211647","PASS",0.1924,-0.3471,"Plate control","PASS","PFC",0.786162797599055,"24h","24h"
1983,"PFC_27",28,"OID30104","Q13296","SCGB2A2",0.7955,"Cardiometabolic_II","B13805","20212097_SS211647","PASS",0.1924,-0.3866,"Plate control","PASS","PFC",0.764930193225763,"24h","24h"
1984,"PFC_04",37,"OID30104","Q13296","SCGB2A2",0.7955,"Cardiometabolic_II","B13805","20212097_SS211647","PASS",0.1924,0.2009,"Plate control","PASS","PFC",1.14941517388471,"Baseline","Baseline"
1985,"PFC_12",38,"OID30104","Q13296","SCGB2A2",0.7955,"Cardiometabolic_II","B13805","20212097_SS211647","PASS",0.1924,-0.1978,"Plate control","PASS","PFC",0.8718790992674,"1h","1h"
1986,"PFC_20",39,"OID30104","Q13296","SCGB2A2",0.7955,"Cardiometabolic_II","B13805","20212097_SS211647","PASS",0.1924,-0.057,"Plate control","PASS","PFC",0.961260928388761,"24h","24h"
1987,"PFC_05",49,"OID30104","Q13296","SCGB2A2",0.7955,"Cardiometabolic_II","B13805","20212097_SS211647","PASS",0.1924,0.1553,"Plate control","PASS","PFC",1.11365317181959,"Baseline","Baseline"
1988,"PFC_13",50,"OID30104","Q13296","SCGB2A2",0.7955,"Cardiometabolic_II","B13805","20212097_SS211647","PASS",0.1924,-0.1795,"Plate control","PASS","PFC",0.883008970855519,"1h","1h"
1989,"PFC_21",51,"OID30104","Q13296","SCGB2A2",0.7955,"Cardiometabolic_II","B13805","20212097_SS211647","PASS",0.1924,-0.0323,"Plate control","PASS","PFC",0.977860112010275,"24h","24h"
1990,"PFC_06",61,"OID30104","Q13296","SCGB2A2",0.7955,"Cardiometabolic_II","B13805","20212097_SS211647","PASS",0.1924,-0.0876,"Plate control","PASS","PFC",0.941086996064477,"Baseline","Baseline"
1991,"PFC_14",62,"OID30104","Q13296","SCGB2A2",0.7955,"Cardiometabolic_II","B13805","20212097_SS211647","PASS",0.1924,-0.1277,"Plate control","PASS","PFC",0.915289477436593,"1h","1h"
1992,"PFC_22",63,"OID30104","Q13296","SCGB2A2",0.7955,"Cardiometabolic_II","B13805","20212097_SS211647","PASS",0.1924,-0.1929,"Plate control","PASS","PFC",0.874845402466674,"24h","24h"
1993,"PFC_07",73,"OID30104","Q13296","SCGB2A2",0.7955,"Cardiometabolic_II","B13805","20212097_SS211647","PASS",0.1924,-0.0627,"Plate control","PASS","PFC",0.957470537896994,"Baseline","Baseline"
1994,"PFC_15",74,"OID30104","Q13296","SCGB2A2",0.7955,"Cardiometabolic_II","B13805","20212097_SS211647","PASS",0.1924,0.3692,"Plate control","PASS","PFC",1.29163639671684,"1h","1h"
1995,"PFC_23",75,"OID30104","Q13296","SCGB2A2",0.7955,"Cardiometabolic_II","B13805","20212097_SS211647","PASS",0.1924,0.4818,"Plate control","PASS","PFC",1.39648492513016,"24h","24h"
1996,"PFC_08",85,"OID30104","Q13296","SCGB2A2",0.7955,"Cardiometabolic_II","B13805","20212097_SS211647","PASS",0.1924,0.1205,"Plate control","PASS","PFC",1.08711156140222,"Baseline","Baseline"
1997,"PFC_16",86,"OID30104","Q13296","SCGB2A2",0.7955,"Cardiometabolic_II","B13805","20212097_SS211647","PASS",0.1924,-0.0261,"Plate control","PASS","PFC",0.982071520893559,"1h","1h"
1998,"PFC_24",87,"OID30104","Q13296","SCGB2A2",0.7955,"Cardiometabolic_II","B13805","20212097_SS211647","PASS",0.1924,-0.2069,"Plate control","PASS","PFC",0.866396908376249,"24h","24h"
1999,"PFC_01",1,"OID30105","P20929","NEB",0.2955,"Cardiometabolic_II","B13805","20212097_SS211647","WARN",-0.4015,-0.7756,"Plate control","PASS","PFC",0.584145634416084,"Baseline","Baseline"
2000,"PFC_09",2,"OID30105","P20929","NEB",0.2955,"Cardiometabolic_II","B13805","20212097_SS211647","PASS",-0.4015,-0.5213,"Plate control","PASS","PFC",0.696743720495192,"Baseline","Baseline"
2001,"PFC_17",3,"OID30105","P20929","NEB",0.2955,"Cardiometabolic_II","B13805","20212097_SS211647","PASS",-0.4015,-0.2693,"Plate control","PASS","PFC",0.829722031803731,"1h","1h"
2002,"PFC_25",4,"OID30105","P20929","NEB",0.2955,"Cardiometabolic_II","B13805","20212097_SS211647","PASS",-0.4015,-0.5709,"Plate control","PASS","PFC",0.673196695460374,"24h","24h"
2003,"PFC_02",13,"OID30105","P20929","NEB",0.2955,"Cardiometabolic_II","B13805","20212097_SS211647","PASS",-0.4015,-0.419,"Plate control","PASS","PFC",0.747942879181195,"Baseline","Baseline"
2004,"PFC_10",14,"OID30105","P20929","NEB",0.2955,"Cardiometabolic_II","B13805","20212097_SS211647","PASS",-0.4015,-0.9029,"Plate control","PASS","PFC",0.534810612904742,"1h","1h"
2005,"PFC_18",15,"OID30105","P20929","NEB",0.2955,"Cardiometabolic_II","B13805","20212097_SS211647","PASS",-0.4015,0.0671,"Plate control","PASS","PFC",1.04760873928762,"1h","1h"
2006,"PFC_26",16,"OID30105","P20929","NEB",0.2955,"Cardiometabolic_II","B13805","20212097_SS211647","PASS",-0.4015,-0.6625,"Plate control","PASS","PFC",0.631782552131922,"24h","24h"
2007,"PFC_03",25,"OID30105","P20929","NEB",0.2955,"Cardiometabolic_II","B13805","20212097_SS211647","PASS",-0.4015,-0.0197,"Plate control","PASS","PFC",0.986437807143117,"Baseline","Baseline"
2008,"PFC_11",26,"OID30105","P20929","NEB",0.2955,"Cardiometabolic_II","B13805","20212097_SS211647","PASS",-0.4015,0.845,"Plate control","PASS","PFC",1.79626474576787,"1h","1h"
2009,"PFC_19",27,"OID30105","P20929","NEB",0.2955,"Cardiometabolic_II","B13805","20212097_SS211647","PASS",-0.4015,-0.4438,"Plate control","PASS","PFC",0.735195580879118,"24h","24h"
2010,"PFC_27",28,"OID30105","P20929","NEB",0.2955,"Cardiometabolic_II","B13805","20212097_SS211647","PASS",-0.4015,-0.2294,"Plate control","PASS","PFC",0.852989566398126,"24h","24h"
2011,"PFC_04",37,"OID30105","P20929","NEB",0.2955,"Cardiometabolic_II","B13805","20212097_SS211647","PASS",-0.4015,-0.2664,"Plate control","PASS","PFC",0.831391555731268,"Baseline","Baseline"
2012,"PFC_12",38,"OID30105","P20929","NEB",0.2955,"Cardiometabolic_II","B13805","20212097_SS211647","PASS",-0.4015,0.0667,"Plate control","PASS","PFC",1.04731832073245,"1h","1h"
2013,"PFC_20",39,"OID30105","P20929","NEB",0.2955,"Cardiometabolic_II","B13805","20212097_SS211647","PASS",-0.4015,0.0359,"Plate control","PASS","PFC",1.02519617424197,"24h","24h"
2014,"PFC_05",49,"OID30105","P20929","NEB",0.2955,"Cardiometabolic_II","B13805","20212097_SS211647","PASS",-0.4015,-0.4687,"Plate control","PASS","PFC",0.722615446997536,"Baseline","Baseline"
2015,"PFC_13",50,"OID30105","P20929","NEB",0.2955,"Cardiometabolic_II","B13805","20212097_SS211647","PASS",-0.4015,-0.1814,"Plate control","PASS","PFC",0.881846831441207,"1h","1h"
2016,"PFC_21",51,"OID30105","P20929","NEB",0.2955,"Cardiometabolic_II","B13805","20212097_SS211647","PASS",-0.4015,0.0415,"Plate control","PASS","PFC",1.02918333385451,"24h","24h"

Supplemental table 1_Olink proximity extension assay (PEA) data

,"SampleID","Index","OlinkID","UniProt","Assay","MissingFreq","Panel","Panel_Lot_Nr","PlateID","QC_Warning","LOD","NPX","Normalization","Assay_Warning","condition","Value","group","alt_group"
2017,"PFC_06",61,"OID30105","P20929","NEB",0.2955,"Cardiometabolic_II","B13805","20212097_SS211647","PASS",-0.4015,-0.2163,"Plate control","PASS","PFC",0.860770177600917,"Baseline","Baseline"
2018,"PFC_14",62,"OID30105","P20929","NEB",0.2955,"Cardiometabolic_II","B13805","20212097_SS211647","PASS",-0.4015,-0.174,"Plate control","PASS","PFC",0.886381699194867,"1h","1h"
2019,"PFC_22",63,"OID30105","P20929","NEB",0.2955,"Cardiometabolic_II","B13805","20212097_SS211647","PASS",-0.4015,-0.2679,"Plate control","PASS","PFC",0.830527589881523,"24h","24h"
2020,"PFC_07",73,"OID30105","P20929","NEB",0.2955,"Cardiometabolic_II","B13805","20212097_SS211647","PASS",-0.4015,-0.6679,"Plate control","PASS","PFC",0.629422213463689,"Baseline","Baseline"
2021,"PFC_15",74,"OID30105","P20929","NEB",0.2955,"Cardiometabolic_II","B13805","20212097_SS211647","PASS",-0.4015,-0.0198,"Plate control","PASS","PFC",0.986369434854266,"1h","1h"
2022,"PFC_23",75,"OID30105","P20929","NEB",0.2955,"Cardiometabolic_II","B13805","20212097_SS211647","PASS",-0.4015,0.2143,"Plate control","PASS","PFC",1.16014087393723,"24h","24h"
2023,"PFC_08",85,"OID30105","P20929","NEB",0.2955,"Cardiometabolic_II","B13805","20212097_SS211647","PASS",-0.4015,-0.9719,"Plate control","PASS","PFC",0.509834179286759,"Baseline","Baseline"
2024,"PFC_16",86,"OID30105","P20929","NEB",0.2955,"Cardiometabolic_II","B13805","20212097_SS211647","PASS",-0.4015,-0.5291,"Plate control","PASS","PFC",0.692986906968358,"1h","1h"
2025,"PFC_24",87,"OID30105","P20929","NEB",0.2955,"Cardiometabolic_II","B13805","20212097_SS211647","PASS",-0.4015,-0.3301,"Plate control","PASS","PFC",0.795481343278889,"24h","24h"
2026,"PFC_01",1,"OID30106","Q5TA50","CPTP",0.6136,"Cardiometabolic_II","B13805","20212097_SS211647","WARN",0.5679,0.4488,"Plate control","PASS","PFC",1.36490448882948,"Baseline","Baseline"
2027,"PFC_09",2,"OID30106","Q5TA50","CPTP",0.6136,"Cardiometabolic_II","B13805","20212097_SS211647","PASS",0.5679,0.1829,"Plate control","PASS","PFC",1.13516341588692,"Baseline","Baseline"
2028,"PFC_17",3,"OID30106","Q5TA50","CPTP",0.6136,"Cardiometabolic_II","B13805","20212097_SS211647","PASS",0.5679,1.713,"Plate control","PASS","PFC",3.27841843055007,"1h","1h"
2029,"PFC_25",4,"OID30106","Q5TA50","CPTP",0.6136,"Cardiometabolic_II","B13805","20212097_SS211647","PASS",0.5679,1.0171,"Plate control","PASS","PFC",2.02384667955414,"24h","24h"
2030,"PFC_02",13,"OID30106","Q5TA50","CPTP",0.6136,"Cardiometabolic_II","B13805","20212097_SS211647","PASS",0.5679,0.4318,"Plate control","PASS","PFC",1.34891552197911,"Baseline","Baseline"
2031,"PFC_10",14,"OID30106","Q5TA50","CPTP",0.6136,"Cardiometabolic_II","B13805","20212097_SS211647","PASS",0.5679,0.9539,"Plate control","PASS","PFC",1.93710210411352,"1h","1h"
2032,"PFC_18",15,"OID30106","Q5TA50","CPTP",0.6136,"Cardiometabolic_II","B13805","20212097_SS211647","PASS",0.5679,0.307,"Plate control","PASS","PFC",1.23713247868717,"1h","1h"
2033,"PFC_26",16,"OID30106","Q5TA50","CPTP",0.6136,"Cardiometabolic_II","B13805","20212097_SS211647","PASS",0.5679,2.0737,"Plate control","PASS","PFC",4.20964915588414,"24h","24h"
2034,"PFC_03",25,"OID30106","Q5TA50","CPTP",0.6136,"Cardiometabolic_II","B13805","20212097_SS211647","PASS",0.5679,0.4495,"Plate control","PASS","PFC",1.36556690530844,"Baseline","Baseline"
2035,"PFC_11",26,"OID30106","Q5TA50","CPTP",0.6136,"Cardiometabolic_II","B13805","20212097_SS211647","PASS",0.5679,0.3228,"Plate control","PASS","PFC",1.25075567653997,"1h","1h"
2036,"PFC_19",27,"OID30106","Q5TA50","CPTP",0.6136,"Cardiometabolic_II","B13805","20212097_SS211647","PASS",0.5679,0.7587,"Plate control","PASS","PFC",1.69196532231927,"24h","24h"
2037,"PFC_27",28,"OID30106","Q5TA50","CPTP",0.6136,"Cardiometabolic_II","B13805","20212097_SS211647","PASS",0.5679,0.2996,"Plate control","PASS","PFC",1.23080311494952,"24h","24h"
2038,"PFC_04",37,"OID30106","Q5TA50","CPTP",0.6136,"Cardiometabolic_II","B13805","20212097_SS211647","PASS",0.5679,0.4855,"Plate control","PASS","PFC",1.40007100915766,"Baseline","Baseline"
2039,"PFC_12",38,"OID30106","Q5TA50","CPTP",0.6136,"Cardiometabolic_II","B13805","20212097_SS211647","PASS",0.5679,-0.9586,"Plate control","PASS","PFC",0.514555999387103,"1h","1h"
2040,"PFC_20",39,"OID30106","Q5TA50","CPTP",0.6136,"Cardiometabolic_II","B13805","20212097_SS211647","PASS",0.5679,0.7386,"Plate control","PASS","PFC",1.66855587625138,"24h","24h"
2041,"PFC_05",49,"OID30106","Q5TA50","CPTP",0.6136,"Cardiometabolic_II","B13805","20212097_SS211647","PASS",0.5679,0.3123,"Plate control","PASS","PFC",1.2416856659578,"Baseline","Baseline"
2042,"PFC_13",50,"OID30106","Q5TA50","CPTP",0.6136,"Cardiometabolic_II","B13805","20212097_SS211647","PASS",0.5679,0.17,"Plate control","PASS","PFC",1.12505848468881,"1h","1h"
2043,"PFC_21",51,"OID30106","Q5TA50","CPTP",0.6136,"Cardiometabolic_II","B13805","20212097_SS211647","PASS",0.5679,0.1082,"Plate control","PASS","PFC",1.07788256093833,"24h","24h"
2044,"PFC_06",61,"OID30106","Q5TA50","CPTP",0.6136,"Cardiometabolic_II","B13805","20212097_SS211647","PASS",0.5679,0.7241,"Plate control","PASS","PFC",1.65186982479447,"Baseline","Baseline"
2045,"PFC_14",62,"OID30106","Q5TA50","CPTP",0.6136,"Cardiometabolic_II","B13805","20212097_SS211647","PASS",0.5679,0.1508,"Plate control","PASS","PFC",1.11018491865106,"1h","1h"
2046,"PFC_22",63,"OID30106","Q5TA50","CPTP",0.6136,"Cardiometabolic_II","B13805","20212097_SS211647","PASS",0.5679,-0.2401,"Plate control","PASS","PFC",0.846686622483995,"24h","24h"
2047,"PFC_07",73,"OID30106","Q5TA50","CPTP",0.6136,"Cardiometabolic_II","B13805","20212097_SS211647","PASS",0.5679,1.0508,"Plate control","PASS","PFC",2.07167831161609,"Baseline","Baseline"
2048,"PFC_15",74,"OID30106","Q5TA50","CPTP",0.6136,"Cardiometabolic_II","B13805","20212097_SS211647","PASS",0.5679,0.0169,"Plate control","PASS","PFC",1.01178306713827,"1h","1h"
2049,"PFC_23",75,"OID30106","Q5TA50","CPTP",0.6136,"Cardiometabolic_II","B13805","20212097_SS211647","PASS",0.5679,0.6023,"Plate control","PASS","PFC",1.51813490743291,"24h","24h"
2050,"PFC_08",85,"OID30106","Q5TA50","CPTP",0.6136,"Cardiometabolic_II","B13805","20212097_SS211647","PASS",0.5679,2.0486,"Plate control","PASS","PFC",4.13704313513456,"Baseline","Baseline"
2051,"PFC_16",86,"OID30106","Q5TA50","CPTP",0.6136,"Cardiometabolic_II","B13805","20212097_SS211647","PASS",0.5679,0.9475,"Plate control","PASS","PFC",1.9285278765039,"1h","1h"
2052,"PFC_24",87,"OID30106","Q5TA50","CPTP",0.6136,"Cardiometabolic_II","B13805","20212097_SS211647","PASS",0.5679,0.4713,"Plate control","PASS","PFC",1.38635814074985,"24h","24h"
2053,"PFC_01",1,"OID30107","Q86UW2","SLC51B",0,"Cardiometabolic_II","B13805","20212097_SS211647","WARN",-1.4753,0.4827,"Plate control","PASS","PFC",1.39735636954925,"Baseline","Baseline"
2054,"PFC_09",2,"OID30107","Q86UW2","SLC51B",0,"Cardiometabolic_II","B13805","20212097_SS211647","PASS",-1.4753,0.679,"Plate control","PASS","PFC",1.60102962131261,"Baseline","Baseline"
2055,"PFC_17",3,"OID30107","Q86UW2","SLC51B",0,"Cardiometabolic_II","B13805","20212097_SS211647","PASS",-1.4753,0.752,"Plate control","PASS","PFC",1.68412590721683,"1h","1h"
2056,"PFC_25",4,"OID30107","Q86UW2","SLC51B",0,"Cardiometabolic_II","B13805","20212097_SS211647","PASS",-1.4753,0.1341,"Plate control","PASS","PFC",1.09740800146925,"24h","24h"
2057,"PFC_02",13,"OID30107","Q86UW2","SLC51B",0,"Cardiometabolic_II","B13805","20212097_SS211647","PASS",-1.4753,-0.2861,"Plate control","PASS","PFC",0.820116063112857,"Baseline","Baseline"
2058,"PFC_10",14,"OID30107","Q86UW2","SLC51B",0,"Cardiometabolic_II","B13805","20212097_SS211647","PASS",-1.4753,-0.0081,"Plate control","PASS","PFC",0.99440123964278З,"1h","1h"
2059,"PFC_18",15,"OID30107","Q86UW2","SLC51B",0,"Cardiometabolic_II","B13805","20212097_SS211647","PASS",-1.4753,-0.0298,"Plate control","PASS","PFC",0.979556083482242,"1h","1h"
2060,"PFC_26",16,"OID30107","Q86UW2","SLC51B",0,"Cardiometabolic_II","B13805","20212097_SS211647","PASS",-1.4753,0.3898,"Plate control","PASS","PFC",1.31021175735077,"24h","24h"
2061,"PFC_03",25,"OID30107","Q86UW2","SLC51B",0,"Cardiometabolic_II","B13805","20212097_SS211647","PASS",-1.4753,1.1976,"Plate control","PASS","PFC",2.29357804542543,"Baseline","Baseline"
2062,"PFC_11",26,"OID30107","Q86UW2","SLC51B",0,"Cardiometabolic_II","B13805","20212097_SS211647","PASS",-1.4753,-0.4925,"Plate control","PASS","PFC",0.710792320754369,"1h","1h"
2063,"PFC_19",27,"OID30107","Q86UW2","SLC51B",0,"Cardiometabolic_II","B13805","20212097_SS211647","PASS",-1.4753,0.6146,"Plate control","PASS","PFC",1.53113341791326,"24h","24h"
2064,"PFC_27",28,"OID30107","Q86UW2","SLC51B",0,"Cardiometabolic_II","B13805","20212097_SS211647","PASS",-1.4753,0.5686,"Plate control","PASS","PFC",1.48308367872351,"24h","24h"
2065,"PFC_04",37,"OID30107","Q86UW2","SLC51B",0,"Cardiometabolic_II","B13805","20212097_SS211647","PASS",-1.4753,-0.1675,"Plate control","PASS","PFC",0.890384263456113,"Baseline","Baseline"
2066,"PFC_12",38,"OID30107","Q86UW2","SLC51B",0,"Cardiometabolic_II","B13805","20212097_SS211647","PASS",-1.4753,1.6112,"Plate control","PASS","PFC",3.05505848760194,"1h","1h"
2067,"PFC_20",39,"OID30107","Q86UW2","SLC51B",0,"Cardiometabolic_II","B13805","20212097_SS211647","PASS",-1.4753,0.2038,"Plate control","PASS","PFC",1.15172796788308,"24h","24h"
2068,"PFC_05",49,"OID30107","Q86UW2","SLC51B",0,"Cardiometabolic_II","B13805","20212097_SS211647","PASS",-1.4753,-0.3966,"Plate control","PASS","PFC",0.759646434425707,"Baseline","Baseline"
2069,"PFC_13",50,"OID30107","Q86UW2","SLC51B",0,"Cardiometabolic_II","B13805","20212097_SS211647","PASS",-1.4753,-0.1951,"Plate control","PASS","PFC",0.873512346556218,"1h","1h"
2070,"PFC_21",51,"OID30107","Q86UW2","SLC51B",0,"Cardiometabolic_II","B13805","20212097_SS211647","PASS",-1.4753,1.1957,"Plate control","PASS","PFC",2.2905594379884,"24h","24h"
2071,"PFC_06",61,"OID30107","Q86UW2","SLC51B",0,"Cardiometabolic_II","B13805","20212097_SS211647","PASS",-1.4753,-0.0734,"Plate control","PASS","PFC",0.950395559095927,"Baseline","Baseline"
2072,"PFC_14",62,"OID30107","Q86UW2","SLC51B",0,"Cardiometabolic_II","B13805","20212097_SS211647","PASS",-1.4753,0.195,"Plate control","PASS","PFC",1.14472416059868,"1h","1h"
2073,"PFC_22",63,"OID30107","Q86UW2","SLC51B",0,"Cardiometabolic_II","B13805","20212097_SS211647","PASS",-1.4753,-0.1603,"Plate control","PASS","PFC",0.894838975046085,"24h","24h"
2074,"PFC_07",73,"OID30107","Q86UW2","SLC51B",0,"Cardiometabolic_II","B13805","20212097_SS211647","PASS",-1.4753,0.3099,"Plate control","PASS","PFC",1.23962177292733,"Baseline","Baseline"
2075,"PFC_15",74,"OID30107","Q86UW2","SLC51B",0,"Cardiometabolic_II","B13805","20212097_SS211647","PASS",-1.4753,-0.2906,"Plate control","PASS","PFC",0.81756197337789,"1h","1h"
2076,"PFC_23",75,"OID30107","Q86UW2","SLC51B",0,"Cardiometabolic_II","B13805","20212097_SS211647","PASS",-1.4753,0.5906,"Plate control","PASS","PFC",1.50587289212858,"24h","24h"
2077,"PFC_08",85,"OID30107","Q86UW2","SLC51B",0,"Cardiometabolic_II","B13805","20212097_SS211647","PASS",-1.4753,0.1505,"Plate control","PASS","PFC",1.10995408618814,"Baseline","Baseline"
2078,"PFC_16",86,"OID30107","Q86UW2","SLC51B",0,"Cardiometabolic_II","B13805","20212097_SS211647","PASS",-1.4753,-0.3413,"Plate control","PASS","PFC",0.789329733130043,"1h","1h"
2079,"PFC_24",87,"OID30107","Q86UW2","SLC51B",0,"Cardiometabolic_II","B13805","20212097_SS211647","PASS",-1.4753,0.4346,"Plate control","PASS","PFC",1.35153605571278,"24h","24h"
2080,"PFC_01",1,"OID30108","Q8WZ42","TTN",0.25,"Cardiometabolic_II","B13805","20212097_SS211647","WARN",-0.3617,0.2689,"Plate control","PASS","PFC",1.2048887955731,"Baseline","Baseline"
2081,"PFC_09",2,"OID30108","Q8WZ42","TTN",0.25,"Cardiometabolic_II","B13805","20212097_SS211647","PASS",-0.3617,0.4862,"Plate control","PASS","PFC",1.40075049267886,"Baseline","Baseline"
2082,"PFC_17",3,"OID30108","Q8WZ42","TTN",0.25,"Cardiometabolic_II","B13805","20212097_SS211647","PASS",-0.3617,-0.8498,"Plate control","PASS","PFC",0.554861650860249,"1h","1h"
2083,"PFC_25",4,"OID30108","Q8WZ42","TTN",0.25,"Cardiometabolic_II","B13805","20212097_SS211647","PASS",-0.3617,-1.2136,"Plate control","PASS","PFC",0.431191307552601,"24h","24h"
2084,"PFC_02",13,"OID30108","Q8WZ42","TTN",0.25,"Cardiometabolic_II","B13805","20212097_SS211647","PASS",-0.3617,0.5913,"Plate control","PASS","PFC",1.50660372349967,"Baseline","Baseline"
2085,"PFC_10",14,"OID30108","Q8WZ42","TTN",0.25,"Cardiometabolic_II","B13805","20212097_SS211647","PASS",-0.3617,-0.0056,"Plate control","PASS","PFC",0.996125899554165,"1h","1h"
2086,"PFC_18",15,"OID30108","Q8WZ42","TTN",0.25,"Cardiometabolic_II","B13805","20212097_SS211647","PASS",-0.3617,0.2359,"Plate control","PASS","PFC",1.17764115890101,"1h","1h"
2087,"PFC_26",16,"OID30108","Q8WZ42","TTN",0.25,"Cardiometabolic_II","B13805","20212097_SS211647","PASS",-0.3617,-2.0062,"Plate control","PASS","PFC",0.248927927143368,"24h","24h"
2088,"PFC_03",25,"OID30108","Q8WZ42","TTN",0.25,"Cardiometabolic_II","B13805","20212097_SS211647","PASS",-0.3617,0.1896,"Plate control","PASS","PFC",1.14044747284971,"Baseline","Baseline"
2089,"PFC_11",26,"OID30108","Q8WZ42","TTN",0.25,"Cardiometabolic_II","B13805","20212097_SS211647","PASS",-0.3617,1.3092,"Plate control","PASS","PFC",2.47804090117007,"1h","1h"
2090,"PFC_19",27,"OID30108","Q8WZ42","TTN",0.25,"Cardiometabolic_II","B13805","20212097_SS211647","PASS",-0.3617,-0.0223,"Plate control","PASS","PFC",0.984661666967831,"24h","24h"
2091,"PFC_27",28,"OID30108","Q8WZ42","TTN",0.25,"Cardiometabolic_II","B13805","20212097_SS211647","PASS",-0.3617,0.5385,"Plate control","PASS","PFC",1.45246157750769,"24h","24h"
2092,"PFC_04",37,"OID30108","Q8WZ42","TTN",0.25,"Cardiometabolic_II","B13805","20212097_SS211647","PASS",-0.3617,-0.3688,"Plate control","PASS","PFC",0.774426378704245,"Baseline","Baseline"
2093,"PFC_12",38,"OID30108","Q8WZ42","TTN",0.25,"Cardiometabolic_II","B13805","20212097_SS211647","PASS",-0.3617,1.1639,"Plate control","PASS","PFC",2.24062311484618,"1h","1h"
2094,"PFC_20",39,"OID30108","Q8WZ42","TTN",0.25,"Cardiometabolic_II","B13805","20212097_SS211647","PASS",-0.3617,0.9562,"Plate control","PASS","PFC",1.94019276987054,"24h","24h"
2095,"PFC_05",49,"OID30108","Q8WZ42","TTN",0.25,"Cardiometabolic_II","B13805","20212097_SS211647","PASS",-0.3617,0.2558,"Plate control","PASS","PFC",1.1939976523796,"Baseline","Baseline"
2096,"PFC_13",50,"OID30108","Q8WZ42","TTN",0.25,"Cardiometabolic_II","B13805","20212097_SS211647","PASS",-0.3617,-0.401,"Plate control","PASS","PFC",0.757333157938488,"1h","1h"
2097,"PFC_21",51,"OID30108","Q8WZ42","TTN",0.25,"Cardiometabolic_II","B13805","20212097_SS211647","PASS",-0.3617,0.9643,"Plate control","PASS","PFC",1.95111660416625,"24h","24h"
2098,"PFC_06",61,"OID30108","Q8WZ42","TTN",0.25,"Cardiometabolic_II","B13805","20212097_SS211647","PASS",-0.3617,-1.034,"Plate control","PASS","PFC",0.488354264481114,"Baseline","Baseline"
2099,"PFC_14",62,"OID30108","Q8WZ42","TTN",0.25,"Cardiometabolic_II","B13805","20212097_SS211647","PASS",-0.3617,0.3306,"Plate control","PASS","PFC",1.25753626041124,"1h","1h"
2100,"PFC_22",63,"OID30108","Q8WZ42","TTN",0.25,"Cardiometabolic_II","B13805","20212097_SS211647","PASS",-0.3617,0.034,"Plate control","PASS","PFC",1.02384690042844,"24h","24h"
2101,"PFC_07",73,"OID30108","Q8WZ42","TTN",0.25,"Cardiometabolic_II","B13805","20212097_SS211647","PASS",-0.3617,-0.5527,"Plate control","PASS","PFC",0.681743053385324,"Baseline","Baseline"
2102,"PFC_15",74,"OID30108","Q8WZ42","TTN",0.25,"Cardiometabolic_II","B13805","20212097_SS211647","PASS",-0.3617,-0.7974,"Plate control","PASS","PFC",0.575385192896516,"1h","1h"
2103,"PFC_23",75,"OID30108","Q8WZ42","TTN",0.25,"Cardiometabolic_II","B13805","20212097_SS211647","PASS",-0.3617,0.4364,"Plate control","PASS","PFC",1.35322337222831,"24h","24h"
2104,"PFC_08",85,"OID30108","Q8WZ42","TTN",0.25,"Cardiometabolic_II","B13805","20212097_SS211647","PASS",-0.3617,-0.3538,"Plate control","PASS","PFC",0.78252025455921,"Baseline","Baseline"
2105,"PFC_16",86,"OID30108","Q8WZ42","TTN",0.25,"Cardiometabolic_II","B13805","20212097_SS211647","PASS",-0.3617,-0.1616,"Plate control","PASS","PFC",0.89403300657983,"1h","1h"
2106,"PFC_24",87,"OID30108","Q8WZ42","TTN",0.25,"Cardiometabolic_II","B13805","20212097_SS211647","PASS",-0.3617,-0.4643,"Plate control","PASS","PFC",0.724822678128669,"24h","24h"
2107,"PFC_01",1,"OID30109","Q9UFP1","GASK1A",0.3182,"Cardiometabolic_II","B13805","20212097_SS211647","WARN",-0.4326,-0.601,"Plate control","PASS","PFC",0.659296807246183,"Baseline","Baseline"
2108,"PFC_09",2,"OID30109","Q9UFP1","GASK1A",0.3182,"Cardiometabolic_II","B13805","20212097_SS211647","PASS",-0.4326,-0.2676,"Plate control","PASS","PFC",0.830700311196286,"Baseline","Baseline"
2109,"PFC_17",3,"OID30109","Q9UFP1","GASK1A",0.3182,"Cardiometabolic_II","B13805","20212097_SS211647","PASS",-0.4326,-0.6324,"Plate control","PASS","PFC",0.645102360075464,"1h","1h"
2110,"PFC_25",4,"OID30109","Q9UFP1","GASK1A",0.3182,"Cardiometabolic_II","B13805","20212097_SS211647","PASS",-0.4326,0.2929,"Plate control","PASS","PFC",1.22510041147198,"24h","24h"
2111,"PFC_02",13,"OID30109","Q9UFP1","GASK1A",0.3182,"Cardiometabolic_II","B13805","20212097_SS211647","PASS",-0.4326,-1.3769,"Plate control","PASS","PFC",0.385045275497786,"Baseline","Baseline"
2112,"PFC_10",14,"OID30109","Q9UFP1","GASK1A",0.3182,"Cardiometabolic_II","B13805","20212097_SS211647","PASS",-0.4326,-0.3521,"Plate control","PASS","PFC",0.783442880944854,"1h","1h"
2113,"PFC_18",15,"OID30109","Q9UFP1","GASK1A",0.3182,"Cardiometabolic_II","B13805","20212097_SS211647","PASS",-0.4326,-0.3754,"Plate control","PASS","PFC",0.770891646522533,"1h","1h"
2114,"PFC_26",16,"OID30109","Q9UFP1","GASK1A",0.3182,"Cardiometabolic_II","B13805","20212097_SS211647","PASS",-0.4326,-1.2248,"Plate control","PASS","PFC",0.427856822280199,"24h","24h"
2115,"PFC_03",25,"OID30109","Q9UFP1","GASK1A",0.3182,"Cardiometabolic_II","B13805","20212097_SS211647","PASS",-0.4326,0.1849,"Plate control","PASS","PFC",1.13673817781891,"Baseline","Baseline"
2116,"PFC_11",26,"OID30109","Q9UFP1","GASK1A",0.3182,"Cardiometabolic_II","B13805","20212097_SS211647","PASS",-0.4326,-0.6812,"Plate control","PASS","PFC",0.62364632422583,"1h","1h"
2117,"PFC_19",27,"OID30109","Q9UFP1","GASK1A",0.3182,"Cardiometabolic_II","B13805","20212097_SS211647","PASS",-0.4326,-0.0936,"Plate control","PASS","PFC",0.937181252671215,"24h","24h"
2118,"PFC_27",28,"OID30109","Q9UFP1","GASK1A",0.3182,"Cardiometabolic_II","B13805","20212097_SS211647","PASS",-0.4326,0.0219,"Plate control","PASS","PFC",1.01529572349355,"24h","24h"
2119,"PFC_04",37,"OID30109","Q9UFP1","GASK1A",0.3182,"Cardiometabolic_II","B13805","20212097_SS211647","PASS",-0.4326,0.3469,"Plate control","PASS","PFC",1.27182484750752,"Baseline","Baseline"
2120,"PFC_12",38,"OID30109","Q9UFP1","GASK1A",0.3182,"Cardiometabolic_II","B13805","20212097_SS211647","PASS",-0.4326,-0.0535,"Plate control","PASS","PFC",0.963595793010082,"1h","1h"
2121,"PFC_20",39,"OID30109","Q9UFP1","GASK1A",0.3182,"Cardiometabolic_II","B13805","20212097_SS211647","PASS",-0.4326,-0.8478,"Plate control","PASS","PFC",0.555631385854405,"24h","24h"
2122,"PFC_05",49,"OID30109","Q9UFP1","GASK1A",0.3182,"Cardiometabolic_II","B13805","20212097_SS211647","PASS",-0.4326,-0.675,"Plate control","PASS","PFC",0.626332219312064,"Baseline","Baseline"
2123,"PFC_13",50,"OID30109","Q9UFP1","GASK1A",0.3182,"Cardiometabolic_II","B13805","20212097_SS211647","PASS",-0.4326,0.0991,"Plate control","PASS","PFC",1.07110506396395,"1h","1h"
2124,"PFC_21",51,"OID30109","Q9UFP1","GASK1A",0.3182,"Cardiometabolic_II","B13805","20212097_SS211647","PASS",-0.4326,-0.2072,"Plate control","PASS","PFC",0.8662167649345,"24h","24h"
2125,"PFC_06",61,"OID30109","Q9UFP1","GASK1A",0.3182,"Cardiometabolic_II","B13805","20212097_SS211647","PASS",-0.4326,-0.0535,"Plate control","PASS","PFC",0.963595793010082,"Baseline","Baseline"
2126,"PFC_14",62,"OID30109","Q9UFP1","GASK1A",0.3182,"Cardiometabolic_II","B13805","20212097_SS211647","PASS",-0.4326,-1.1565,"Plate control","PASS","PFC",0.448599525640945,"1h","1h"
2127,"PFC_22",63,"OID30109","Q9UFP1","GASK1A",0.3182,"Cardiometabolic_II","B13805","20212097_SS211647","PASS",-0.4326,0.3784,"Plate control","PASS","PFC",1.29989942114432,"24h","24h"
2128,"PFC_07",73,"OID30109","Q9UFP1","GASK1A",0.3182,"Cardiometabolic_II","B13805","20212097_SS211647","PASS",-0.4326,-0.2984,"Plate control","PASS","PFC",0.813153712793311,"Baseline","Baseline"

Supplemental table 1_Olink proximity extension assay (PEA) data

,"SampleID","Index","OlinkID","UniProt","Assay","MissingFreq","Panel","Panel_Lot_Nr","PlateID","QC_Warning","LOD","NPX","Normalization","Assay_Warning","condition","Value","group","alt_group"
2129,"PFC_15",74,"OID30109","Q9UFP1","GASK1A",0.3182,"Cardiometabolic_II","B13805","20212097_SS211647","PASS",-0.4326,-0.3347,"Plate control","PASS","PFC",0.792949008578497,"1h","1h"
2130,"PFC_23",75,"OID30109","Q9UFP1","GASK1A",0.3182,"Cardiometabolic_II","B13805","20212097_SS211647","PASS",-0.4326,-0.0928,"Plate control","PASS","PFC",0.93770108041912,"24h","24h"
2131,"PFC_08",85,"OID30109","Q9UFP1","GASK1A",0.3182,"Cardiometabolic_II","B13805","20212097_SS211647","PASS",-0.4326,-1.7125,"Plate control","PASS","PFC",0.305130859542539,"Baseline","Baseline"
2132,"PFC_16",86,"OID30109","Q9UFP1","GASK1A",0.3182,"Cardiometabolic_II","B13805","20212097_SS211647","PASS",-0.4326,0.3431,"Plate control","PASS","PFC",1.26847932056518,"1h","1h"
2133,"PFC_24",87,"OID30109","Q9UFP1","GASK1A",0.3182,"Cardiometabolic_II","B13805","20212097_SS211647","PASS",-0.4326,-0.3504,"Plate control","PASS","PFC",0.78436659514828,"24h","24h"
2134,"PFC_01",1,"OID30110","Q99707","MTR",0.6136,"Cardiometabolic_II","B13805","20212097_SS211647","WARN",1.1181,0.9791,"Plate control","PASS","PFC",1.97123530476279,"Baseline","Baseline"
2135,"PFC_09",2,"OID30110","Q99707","MTR",0.6136,"Cardiometabolic_II","B13805","20212097_SS211647","PASS",1.1181,0.5568,"Plate control","PASS","PFC",1.47100280743042,"Baseline","Baseline"
2136,"PFC_17",3,"OID30110","Q99707","MTR",0.6136,"Cardiometabolic_II","B13805","20212097_SS211647","PASS",1.1181,2.5062,"Plate control","PASS","PFC",5.68121696348915,"1h","1h"
2137,"PFC_25",4,"OID30110","Q99707","MTR",0.6136,"Cardiometabolic_II","B13805","20212097_SS211647","PASS",1.1181,0.5446,"Plate control","PASS","PFC",1.45861587397905,"24h","24h"
2138,"PFC_02",13,"OID30110","Q99707","MTR",0.6136,"Cardiometabolic_II","B13805","20212097_SS211647","PASS",1.1181,0.447,"Plate control","PASS","PFC",1.36320260728251,"Baseline","Baseline"
2139,"PFC_10",14,"OID30110","Q99707","MTR",0.6136,"Cardiometabolic_II","B13805","20212097_SS211647","PASS",1.1181,-0.0092,"Plate control","PASS","PFC",0.99364333555883,"1h","1h"
2140,"PFC_18",15,"OID30110","Q99707","MTR",0.6136,"Cardiometabolic_II","B13805","20212097_SS211647","PASS",1.1181,0.3675,"Plate control","PASS","PFC",1.29011529307386,"1h","1h"
2141,"PFC_26",16,"OID30110","Q99707","MTR",0.6136,"Cardiometabolic_II","B13805","20212097_SS211647","PASS",1.1181,0.8014,"Plate control","PASS","PFC",1.74279152171618,"24h","24h"
2142,"PFC_03",25,"OID30110","Q99707","MTR",0.6136,"Cardiometabolic_II","B13805","20212097_SS211647","PASS",1.1181,0.4736,"Plate control","PASS","PFC",1.38857008901188,"Baseline","Baseline"
2143,"PFC_11",26,"OID30110","Q99707","MTR",0.6136,"Cardiometabolic_II","B13805","20212097_SS211647","PASS",1.1181,1.2417,"Plate control","PASS","PFC",2.36477020921654,"1h","1h"
2144,"PFC_19",27,"OID30110","Q99707","MTR",0.6136,"Cardiometabolic_II","B13805","20212097_SS211647","PASS",1.1181,0.8676,"Plate control","PASS","PFC",1.82462501347523,"24h","24h"
2145,"PFC_27",28,"OID30110","Q99707","MTR",0.6136,"Cardiometabolic_II","B13805","20212097_SS211647","PASS",1.1181,0.918,"Plate control","PASS","PFC",1.88949408199441,"24h","24h"
2146,"PFC_04",37,"OID30110","Q99707","MTR",0.6136,"Cardiometabolic_II","B13805","20212097_SS211647","PASS",1.1181,2.1836,"Plate control","PASS","PFC",4.54285733701831,"Baseline","Baseline"
2147,"PFC_12",38,"OID30110","Q99707","MTR",0.6136,"Cardiometabolic_II","B13805","20212097_SS211647","PASS",1.1181,-1.6362,"Plate control","PASS","PFC",0.321702711268455,"1h","1h"
2148,"PFC_20",39,"OID30110","Q99707","MTR",0.6136,"Cardiometabolic_II","B13805","20212097_SS211647","PASS",1.1181,1.7624,"Plate control","PASS","PFC",3.39262036224059,"24h","24h"
2149,"PFC_05",49,"OID30110","Q99707","MTR",0.6136,"Cardiometabolic_II","B13805","20212097_SS211647","PASS",1.1181,0.9426,"Plate control","PASS","PFC",1.92198889442337,"Baseline","Baseline"
2150,"PFC_13",50,"OID30110","Q99707","MTR",0.6136,"Cardiometabolic_II","B13805","20212097_SS211647","PASS",1.1181,1.7584,"Plate control","PASS","PFC",3.38322704919969,"1h","1h"
2151,"PFC_21",51,"OID30110","Q99707","MTR",0.6136,"Cardiometabolic_II","B13805","20212097_SS211647","PASS",1.1181,1.0622,"Plate control","PASS","PFC",2.08811330875009,"24h","24h"
2152,"PFC_06",61,"OID30110","Q99707","MTR",0.6136,"Cardiometabolic_II","B13805","20212097_SS211647","PASS",1.1181,0.7189,"Plate control","PASS","PFC",1.64592659966518,"Baseline","Baseline"
2153,"PFC_14",62,"OID30110","Q99707","MTR",0.6136,"Cardiometabolic_II","B13805","20212097_SS211647","PASS",1.1181,1.0696,"Plate control","PASS","PFC",2.09885136140529,"1h","1h"
2154,"PFC_22",63,"OID30110","Q99707","MTR",0.6136,"Cardiometabolic_II","B13805","20212097_SS211647","PASS",1.1181,1.5722,"Plate control","PASS","PFC",2.97357816575441,"24h","24h"
2155,"PFC_07",73,"OID30110","Q99707","MTR",0.6136,"Cardiometabolic_II","B13805","20212097_SS211647","PASS",1.1181,1.4121,"Plate control","PASS","PFC",2.66124253904478,"Baseline","Baseline"
2156,"PFC_15",74,"OID30110","Q99707","MTR",0.6136,"Cardiometabolic_II","B13805","20212097_SS211647","PASS",1.1181,0.8967,"Plate control","PASS","PFC",1.86180244846941,"1h","1h"
2157,"PFC_23",75,"OID30110","Q99707","MTR",0.6136,"Cardiometabolic_II","B13805","20212097_SS211647","PASS",1.1181,1.2667,"Plate control","PASS","PFC",2.40610566436725,"24h","24h"
2158,"PFC_08",85,"OID30110","Q99707","MTR",0.6136,"Cardiometabolic_II","B13805","20212097_SS211647","PASS",1.1181,2.0388,"Plate control","PASS","PFC",4.10903608467467,"Baseline","Baseline"
2159,"PFC_16",86,"OID30110","Q99707","MTR",0.6136,"Cardiometabolic_II","B13805","20212097_SS211647","PASS",1.1181,0.7496,"Plate control","PASS","PFC",1.68132660315975,"1h","1h"
2160,"PFC_24",87,"OID30110","Q99707","MTR",0.6136,"Cardiometabolic_II","B13805","20212097_SS211647","PASS",1.1181,-0.0097,"Plate control","PASS","PFC",0.993299024688541,"24h","24h"
2161,"PFC_01",1,"OID30111","P21673","SAT1",0.6818,"Cardiometabolic_II","B13805","20212097_SS211647","WARN",-0.0901,-0.449,"Plate control","PASS","PFC",0.732550437305669,"Baseline","Baseline"
2162,"PFC_09",2,"OID30111","P21673","SAT1",0.6818,"Cardiometabolic_II","B13805","20212097_SS211647","PASS",-0.0901,1.4528,"Plate control","PASS","PFC",2.73738811730761,"Baseline","Baseline"
2163,"PFC_17",3,"OID30111","P21673","SAT1",0.6818,"Cardiometabolic_II","B13805","20212097_SS211647","PASS",-0.0901,0.9996,"Plate control","PASS","PFC",1.99944555912093,"1h","1h"
2164,"PFC_25",4,"OID30111","P21673","SAT1",0.6818,"Cardiometabolic_II","B13805","20212097_SS211647","PASS",-0.0901,0.0376,"Plate control","PASS","PFC",1.02640492639287,"24h","24h"
2165,"PFC_02",13,"OID30111","P21673","SAT1",0.6818,"Cardiometabolic_II","B13805","20212097_SS211647","PASS",-0.0901,-0.4929,"Plate control","PASS","PFC",0.710595274594792,"Baseline","Baseline"
2166,"PFC_10",14,"OID30111","P21673","SAT1",0.6818,"Cardiometabolic_II","B13805","20212097_SS211647","PASS",-0.0901,-0.3443,"Plate control","PASS","PFC",0.787690073471928,"1h","1h"
2167,"PFC_18",15,"OID30111","P21673","SAT1",0.6818,"Cardiometabolic_II","B13805","20212097_SS211647","PASS",-0.0901,1.2303,"Plate control","PASS","PFC",2.34615771752455,"1h","1h"
2168,"PFC_26",16,"OID30111","P21673","SAT1",0.6818,"Cardiometabolic_II","B13805","20212097_SS211647","PASS",-0.0901,1.0405,"Plate control","PASS","PFC",2.05694041101674,"24h","24h"
2169,"PFC_03",25,"OID30111","P21673","SAT1",0.6818,"Cardiometabolic_II","B13805","20212097_SS211647","PASS",-0.0901,-0.1992,"Plate control","PASS","PFC",0.871033432898419,"Baseline","Baseline"
2170,"PFC_11",26,"OID30111","P21673","SAT1",0.6818,"Cardiometabolic_II","B13805","20212097_SS211647","PASS",-0.0901,-0.9739,"Plate control","PASS","PFC",0.509127888715339,"1h","1h"
2171,"PFC_19",27,"OID30111","P21673","SAT1",0.6818,"Cardiometabolic_II","B13805","20212097_SS211647","PASS",-0.0901,0.1303,"Plate control","PASS","PFC",1.09452127689955,"24h","24h"
2172,"PFC_27",28,"OID30111","P21673","SAT1",0.6818,"Cardiometabolic_II","B13805","20212097_SS211647","PASS",-0.0901,1.6664,"Plate control","PASS","PFC",3.17421533013357,"24h","24h"
2173,"PFC_04",37,"OID30111","P21673","SAT1",0.6818,"Cardiometabolic_II","B13805","20212097_SS211647","PASS",-0.0901,-0.9051,"Plate control","PASS","PFC",0.533995688980472,"Baseline","Baseline"
2174,"PFC_12",38,"OID30111","P21673","SAT1",0.6818,"Cardiometabolic_II","B13805","20212097_SS211647","PASS",-0.0901,-0.2509,"Plate control","PASS","PFC",0.8403720003628,"1h","1h"
2175,"PFC_20",39,"OID30111","P21673","SAT1",0.6818,"Cardiometabolic_II","B13805","20212097_SS211647","PASS",-0.0901,-0.6644,"Plate control","PASS","PFC",0.630951055024485,"24h","24h"
2176,"PFC_05",49,"OID30111","P21673","SAT1",0.6818,"Cardiometabolic_II","B13805","20212097_SS211647","PASS",-0.0901,-0.2786,"Plate control","PASS","PFC",0.824390622884065,"Baseline","Baseline"
2177,"PFC_13",50,"OID30111","P21673","SAT1",0.6818,"Cardiometabolic_II","B13805","20212097_SS211647","PASS",-0.0901,-0.8609,"Plate control","PASS","PFC",0.550608963031335,"1h","1h"
2178,"PFC_21",51,"OID30111","P21673","SAT1",0.6818,"Cardiometabolic_II","B13805","20212097_SS211647","PASS",-0.0901,-0.3312,"Plate control","PASS","PFC",0.794875050230626,"24h","24h"
2179,"PFC_06",61,"OID30111","P21673","SAT1",0.6818,"Cardiometabolic_II","B13805","20212097_SS211647","PASS",-0.0901,-0.2142,"Plate control","PASS","PFC",0.862024034828109,"Baseline","Baseline"
2180,"PFC_14",62,"OID30111","P21673","SAT1",0.6818,"Cardiometabolic_II","B13805","20212097_SS211647","PASS",-0.0901,-0.5253,"Plate control","PASS","PFC",0.694814612261117,"1h","1h"
2181,"PFC_22",63,"OID30111","P21673","SAT1",0.6818,"Cardiometabolic_II","B13805","20212097_SS211647","PASS",-0.0901,-0.7788,"Plate control","PASS","PFC",0.582851393826948,"24h","24h"
2182,"PFC_07",73,"OID30111","P21673","SAT1",0.6818,"Cardiometabolic_II","B13805","20212097_SS211647","PASS",-0.0901,0.1948,"Plate control","PASS","PFC",1.14456547913301,"Baseline","Baseline"
2183,"PFC_15",74,"OID30111","P21673","SAT1",0.6818,"Cardiometabolic_II","B13805","20212097_SS211647","PASS",-0.0901,-0.5423,"Plate control","PASS","PFC",0.686675311750707,"1h","1h"
2184,"PFC_23",75,"OID30111","P21673","SAT1",0.6818,"Cardiometabolic_II","B13805","20212097_SS211647","PASS",-0.0901,-0.415,"Plate control","PASS","PFC",0.750019494642909,"24h","24h"
2185,"PFC_08",85,"OID30111","P21673","SAT1",0.6818,"Cardiometabolic_II","B13805","20212097_SS211647","PASS",-0.0901,0.6118,"Plate control","PASS","PFC",1.52816465747551,"Baseline","Baseline"
2186,"PFC_16",86,"OID30111","P21673","SAT1",0.6818,"Cardiometabolic_II","B13805","20212097_SS211647","PASS",-0.0901,-0.0775,"Plate control","PASS","PFC",0.947698460948383,"1h","1h"
2187,"PFC_24",87,"OID30111","P21673","SAT1",0.6818,"Cardiometabolic_II","B13805","20212097_SS211647","PASS",-0.0901,-0.8469,"Plate control","PASS","PFC",0.555978114889256,"24h","24h"
2188,"PFC_01",1,"OID30112","O00425","IGF2BP3",0.9773,"Cardiometabolic_II","B13805","20212097_SS211647","WARN",1.2033,-0.1865,"Plate control","PASS","PFC",0.878734961801216,"Baseline","Baseline"
2189,"PFC_09",2,"OID30112","O00425","IGF2BP3",0.9773,"Cardiometabolic_II","B13805","20212097_SS211647","PASS",1.2033,0.2197,"Plate control","PASS","PFC",1.16449141210823,"Baseline","Baseline"
2190,"PFC_17",3,"OID30112","O00425","IGF2BP3",0.9773,"Cardiometabolic_II","B13805","20212097_SS211647","PASS",1.2033,1.1629,"Plate control","PASS","PFC",2.23907057138415,"1h","1h"
2191,"PFC_25",4,"OID30112","O00425","IGF2BP3",0.9773,"Cardiometabolic_II","B13805","20212097_SS211647","PASS",1.2033,0.0638,"Plate control","PASS","PFC",1.0452151925684,"24h","24h"
2192,"PFC_02",13,"OID30112","O00425","IGF2BP3",0.9773,"Cardiometabolic_II","B13805","20212097_SS211647","PASS",1.2033,0.2933,"Plate control","PASS","PFC",1.22544012852303,"Baseline","Baseline"
2193,"PFC_10",14,"OID30112","O00425","IGF2BP3",0.9773,"Cardiometabolic_II","B13805","20212097_SS211647","PASS",1.2033,0.428,"Plate control","PASS","PFC",1.34536720852183,"1h","1h"
2194,"PFC_18",15,"OID30112","O00425","IGF2BP3",0.9773,"Cardiometabolic_II","B13805","20212097_SS211647","PASS",1.2033,0.2586,"Plate control","PASS","PFC",1.19631722767916,"1h","1h"
2195,"PFC_26",16,"OID30112","O00425","IGF2BP3",0.9773,"Cardiometabolic_II","B13805","20212097_SS211647","PASS",1.2033,0.9678,"Plate control","PASS","PFC",1.95585578891474,"24h","24h"
2196,"PFC_03",25,"OID30112","O00425","IGF2BP3",0.9773,"Cardiometabolic_II","B13805","20212097_SS211647","PASS",1.2033,0.0214,"Plate control","PASS","PFC",1.01494390977765,"Baseline","Baseline"
2197,"PFC_11",26,"OID30112","O00425","IGF2BP3",0.9773,"Cardiometabolic_II","B13805","20212097_SS211647","PASS",1.2033,0.4199,"Plate control","PASS","PFC",1.33783481992886,"1h","1h"
2198,"PFC_19",27,"OID30112","O00425","IGF2BP3",0.9773,"Cardiometabolic_II","B13805","20212097_SS211647","PASS",1.2033,0.2873,"Plate control","PASS","PFC",1.2203542494217,"24h","24h"
2199,"PFC_27",28,"OID30112","O00425","IGF2BP3",0.9773,"Cardiometabolic_II","B13805","20212097_SS211647","PASS",1.2033,1.3772,"Plate control","PASS","PFC",2.59763728949273,"24h","24h"
2200,"PFC_04",37,"OID30112","O00425","IGF2BP3",0.9773,"Cardiometabolic_II","B13805","20212097_SS211647","PASS",1.2033,-0.229,"Plate control","PASS","PFC",0.853226098112067,"Baseline","Baseline"
2201,"PFC_12",38,"OID30112","O00425","IGF2BP3",0.9773,"Cardiometabolic_II","B13805","20212097_SS211647","PASS",1.2033,-0.348,"Plate control","PASS","PFC",0.78567251666758,"1h","1h"
2202,"PFC_20",39,"OID30112","O00425","IGF2BP3",0.9773,"Cardiometabolic_II","B13805","20212097_SS211647","PASS",1.2033,0.6676,"Plate control","PASS","PFC",1.58842833328506,"24h","24h"
2203,"PFC_05",49,"OID30112","O00425","IGF2BP3",0.9773,"Cardiometabolic_II","B13805","20212097_SS211647","PASS",1.2033,0.6344,"Plate control","PASS","PFC",1.55229203563012,"Baseline","Baseline"
2204,"PFC_13",50,"OID30112","O00425","IGF2BP3",0.9773,"Cardiometabolic_II","B13805","20212097_SS211647","PASS",1.2033,0.1201,"Plate control","PASS","PFC",1.08681019185734,"1h","1h"
2205,"PFC_21",51,"OID30112","O00425","IGF2BP3",0.9773,"Cardiometabolic_II","B13805","20212097_SS211647","PASS",1.2033,0.0953,"Plate control","PASS","PFC",1.06828752909931,"24h","24h"
2206,"PFC_06",61,"OID30112","O00425","IGF2BP3",0.9773,"Cardiometabolic_II","B13805","20212097_SS211647","PASS",1.2033,0.1902,"Plate control","PASS","PFC",1.14092187026127,"Baseline","Baseline"
2207,"PFC_14",62,"OID30112","O00425","IGF2BP3",0.9773,"Cardiometabolic_II","B13805","20212097_SS211647","PASS",1.2033,0.1869,"Plate control","PASS","PFC",1.13831512434838,"1h","1h"
2208,"PFC_22",63,"OID30112","O00425","IGF2BP3",0.9773,"Cardiometabolic_II","B13805","20212097_SS211647","PASS",1.2033,-0.1268,"Plate control","PASS","PFC",0.91586064286245,"24h","24h"
2209,"PFC_07",73,"OID30112","O00425","IGF2BP3",0.9773,"Cardiometabolic_II","B13805","20212097_SS211647","PASS",1.2033,0.4588,"Plate control","PASS","PFC",1.37439815032417,"Baseline","Baseline"
2210,"PFC_15",74,"OID30112","O00425","IGF2BP3",0.9773,"Cardiometabolic_II","B13805","20212097_SS211647","PASS",1.2033,0.4577,"Plate control","PASS","PFC",1.37335062350141,"1h","1h"
2211,"PFC_23",75,"OID30112","O00425","IGF2BP3",0.9773,"Cardiometabolic_II","B13805","20212097_SS211647","PASS",1.2033,0.8892,"Plate control","PASS","PFC",1.85214878960036,"24h","24h"
2212,"PFC_08",85,"OID30112","O00425","IGF2BP3",0.9773,"Cardiometabolic_II","B13805","20212097_SS211647","PASS",1.2033,0.615,"Plate control","PASS","PFC",1.53155799709438,"Baseline","Baseline"
2213,"PFC_16",86,"OID30112","O00425","IGF2BP3",0.9773,"Cardiometabolic_II","B13805","20212097_SS211647","PASS",1.2033,0.4013,"Plate control","PASS","PFC",1.32069744377585,"1h","1h"
2214,"PFC_24",87,"OID30112","O00425","IGF2BP3",0.9773,"Cardiometabolic_II","B13805","20212097_SS211647","PASS",1.2033,0.3127,"Plate control","PASS","PFC",1.24202998205534,"24h","24h"
2215,"PFC_01",1,"OID30113","O43290","SART1",0.0909,"Cardiometabolic_II","B13805","20212097_SS211647","WARN",-0.9222,-0.3837,"Plate control","PASS","PFC",0.766469346353601,"Baseline","Baseline"
2216,"PFC_09",2,"OID30113","O43290","SART1",0.0909,"Cardiometabolic_II","B13805","20212097_SS211647","PASS",-0.9222,-0.2752,"Plate control","PASS","PFC",0.826335755753675,"Baseline","Baseline"
2217,"PFC_17",3,"OID30113","O43290","SART1",0.0909,"Cardiometabolic_II","B13805","20212097_SS211647","PASS",-0.9222,-0.569,"Plate control","PASS","PFC",0.674083865868528,"1h","1h"
2218,"PFC_25",4,"OID30113","O43290","SART1",0.0909,"Cardiometabolic_II","B13805","20212097_SS211647","PASS",-0.9222,-0.8261,"Plate control","PASS","PFC",0.564051970269934,"24h","24h"
2219,"PFC_02",13,"OID30113","O43290","SART1",0.0909,"Cardiometabolic_II","B13805","20212097_SS211647","PASS",-0.9222,0.1597,"Plate control","PASS","PFC",1.11705482889784,"Baseline","Baseline"
2220,"PFC_10",14,"OID30113","O43290","SART1",0.0909,"Cardiometabolic_II","B13805","20212097_SS211647","PASS",-0.9222,0.0473,"Plate control","PASS","PFC",1.03332924011952,"1h","1h"
2221,"PFC_18",15,"OID30113","O43290","SART1",0.0909,"Cardiometabolic_II","B13805","20212097_SS211647","PASS",-0.9222,-0.0501,"Plate control","PASS","PFC",0.965869377640928,"1h","1h"
2222,"PFC_26",16,"OID30113","O43290","SART1",0.0909,"Cardiometabolic_II","B13805","20212097_SS211647","PASS",-0.9222,-1.1069,"Plate control","PASS","PFC",0.464290607210576,"24h","24h"
2223,"PFC_03",25,"OID30113","O43290","SART1",0.0909,"Cardiometabolic_II","B13805","20212097_SS211647","PASS",-0.9222,-0.4116,"Plate control","PASS","PFC",0.751789150351479,"Baseline","Baseline"
2224,"PFC_11",26,"OID30113","O43290","SART1",0.0909,"Cardiometabolic_II","B13805","20212097_SS211647","PASS",-0.9222,-0.0415,"Plate control","PASS","PFC",0.97164418340782,"1h","1h"
2225,"PFC_19",27,"OID30113","O43290","SART1",0.0909,"Cardiometabolic_II","B13805","20212097_SS211647","PASS",-0.9222,0.2605,"Plate control","PASS","PFC",1.19789379103771,"24h","24h"
2226,"PFC_27",28,"OID30113","O43290","SART1",0.0909,"Cardiometabolic_II","B13805","20212097_SS211647","PASS",-0.9222,-0.3875,"Plate control","PASS","PFC",0.764453153751505,"24h","24h"
2227,"PFC_04",37,"OID30113","O43290","SART1",0.0909,"Cardiometabolic_II","B13805","20212097_SS211647","PASS",-0.9222,-0.0262,"Plate control","PASS","PFC",0.982003451242119,"Baseline","Baseline"
2228,"PFC_12",38,"OID30113","O43290","SART1",0.0909,"Cardiometabolic_II","B13805","20212097_SS211647","PASS",-0.9222,-0.7003,"Plate control","PASS","PFC",0.615444215338598,"1h","1h"
2229,"PFC_20",39,"OID30113","O43290","SART1",0.0909,"Cardiometabolic_II","B13805","20212097_SS211647","PASS",-0.9222,0.1596,"Plate control","PASS","PFC",1.11697740324072,"24h","24h"
2230,"PFC_05",49,"OID30113","O43290","SART1",0.0909,"Cardiometabolic_II","B13805","20212097_SS211647","PASS",-0.9222,-0.2075,"Plate control","PASS","PFC",0.866036658948632,"Baseline","Baseline"
2231,"PFC_13",50,"OID30113","O43290","SART1",0.0909,"Cardiometabolic_II","B13805","20212097_SS211647","PASS",-0.9222,-0.5127,"Plate control","PASS","PFC",0.700909459412176,"1h","1h"
2232,"PFC_21",51,"OID30113","O43290","SART1",0.0909,"Cardiometabolic_II","B13805","20212097_SS211647","PASS",-0.9222,-0.6446,"Plate control","PASS","PFC",0.639670120270613,"24h","24h"
2233,"PFC_06",61,"OID30113","O43290","SART1",0.0909,"Cardiometabolic_II","B13805","20212097_SS211647","PASS",-0.9222,-0.1779,"Plate control","PASS","PFC",0.883988802374746,"Baseline","Baseline"
2234,"PFC_14",62,"OID30113","O43290","SART1",0.0909,"Cardiometabolic_II","B13805","20212097_SS211647","PASS",-0.9222,-0.3297,"Plate control","PASS","PFC",0.795701928117141,"1h","1h"
2235,"PFC_22",63,"OID30113","O43290","SART1",0.0909,"Cardiometabolic_II","B13805","20212097_SS211647","PASS",-0.9222,0.0798,"Plate control","PASS","PFC",1.05687151690294,"24h","24h"
2236,"PFC_07",73,"OID30113","O43290","SART1",0.0909,"Cardiometabolic_II","B13805","20212097_SS211647","PASS",-0.9222,-1.0596,"Plate control","PASS","PFC",0.479765060343534,"Baseline","Baseline"
2237,"PFC_15",74,"OID30113","O43290","SART1",0.0909,"Cardiometabolic_II","B13805","20212097_SS211647","PASS",-0.9222,-0.8488,"Plate control","PASS","PFC",0.555246384972422,"1h","1h"
2238,"PFC_23",75,"OID30113","O43290","SART1",0.0909,"Cardiometabolic_II","B13805","20212097_SS211647","PASS",-0.9222,-0.3299,"Plate control","PASS","PFC",0.795591628053127,"24h","24h"
2239,"PFC_08",85,"OID30113","O43290","SART1",0.0909,"Cardiometabolic_II","B13805","20212097_SS211647","PASS",-0.9222,-1.3962,"Plate control","PASS","PFC",0.379928541170029,"Baseline","Baseline"
2240,"PFC_16",86,"OID30113","O43290","SART1",0.0909,"Cardiometabolic_II","B13805","20212097_SS211647","PASS",-0.9222,-1.1069,"Plate control","PASS","PFC",0.464290607210576,"1h","1h"

Supplemental table 1_Olink proximity extension assay (PEA) data

**,"SampleID","Index","OlinkID","UniProt","Assay","MissingFreq","Panel","Panel_Lot_Nr","PlateID","QC_Warning","LOD","NPX","Normalization","Assay_Warning","condition","Value","group","alt_group"**
2241,"PFC_24",87,"OID30113","O43290","SART1",0.0909,"Cardiometabolic_II","B13805","20212097_SS211647","PASS",-0.9222,-0.5412,"Plate control","PASS","PFC",0.687199075162086,"24h","24h"
2242,"PFC_01",1,"OID30114","Q92935","EXTL1",0.8864,"Cardiometabolic_II","B13805","20212097_SS211647","WARN",0.6472,0.1818,"Plate control","PASS","PFC",1.13429822691233,"Baseline","Baseline"
2243,"PFC_09",2,"OID30114","Q92935","EXTL1",0.8864,"Cardiometabolic_II","B13805","20212097_SS211647","PASS",0.6472,0.4136,"Plate control","PASS","PFC",1.33200546355738,"Baseline","Baseline"
2244,"PFC_17",3,"OID30114","Q92935","EXTL1",0.8864,"Cardiometabolic_II","B13805","20212097_SS211647","PASS",0.6472,1.0901,"Plate control","PASS","PFC",2.12888792305884,"1h","1h"
2245,"PFC_25",4,"OID30114","Q92935","EXTL1",0.8864,"Cardiometabolic_II","B13805","20212097_SS211647","PASS",0.6472,0.5995,"Plate control","PASS","PFC",1.5151913501964,"24h","24h"
2246,"PFC_02",13,"OID30114","Q92935","EXTL1",0.8864,"Cardiometabolic_II","B13805","20212097_SS211647","PASS",0.6472,0.4245,"Plate control","PASS","PFC",1.34210728322883,"Baseline","Baseline"
2247,"PFC_10",14,"OID30114","Q92935","EXTL1",0.8864,"Cardiometabolic_II","B13805","20212097_SS211647","PASS",0.6472,0.3346,"Plate control","PASS","PFC",1.26102772923155,"1h","1h"
2248,"PFC_18",15,"OID30114","Q92935","EXTL1",0.8864,"Cardiometabolic_II","B13805","20212097_SS211647","PASS",0.6472,0.0715,"Plate control","PASS","PFC",1.05080866344121,"1h","1h"
2249,"PFC_26",16,"OID30114","Q92935","EXTL1",0.8864,"Cardiometabolic_II","B13805","20212097_SS211647","PASS",0.6472,0.7616,"Plate control","PASS","PFC",1.69536980777572,"24h","24h"
2250,"PFC_03",25,"OID30114","Q92935","EXTL1",0.8864,"Cardiometabolic_II","B13805","20212097_SS211647","PASS",0.6472,0.0577,"Plate control","PASS","PFC",1.04080514583424,"Baseline","Baseline"
2251,"PFC_11",26,"OID30114","Q92935","EXTL1",0.8864,"Cardiometabolic_II","B13805","20212097_SS211647","PASS",0.6472,0.7255,"Plate control","PASS","PFC",1.65347358729543,"1h","1h"
2252,"PFC_19",27,"OID30114","Q92935","EXTL1",0.8864,"Cardiometabolic_II","B13805","20212097_SS211647","PASS",0.6472,0.4457,"Plate control","PASS","PFC",1.36197479049622,"24h","24h"
2253,"PFC_27",28,"OID30114","Q92935","EXTL1",0.8864,"Cardiometabolic_II","B13805","20212097_SS211647","PASS",0.6472,0.3953,"Plate control","PASS","PFC",1.31521622329666,"24h","24h"
2254,"PFC_04",37,"OID30114","Q92935","EXTL1",0.8864,"Cardiometabolic_II","B13805","20212097_SS211647","PASS",0.6472,0.602,"Plate control","PASS","PFC",1.51781925297405,"Baseline","Baseline"
2255,"PFC_12",38,"OID30114","Q92935","EXTL1",0.8864,"Cardiometabolic_II","B13805","20212097_SS211647","PASS",0.6472,0.1071,"Plate control","PASS","PFC",1.07706102978733,"1h","1h"
2256,"PFC_20",39,"OID30114","Q92935","EXTL1",0.8864,"Cardiometabolic_II","B13805","20212097_SS211647","PASS",0.6472,0.4013,"Plate control","PASS","PFC",1.32069744377585,"24h","24h"
2257,"PFC_05",49,"OID30114","Q92935","EXTL1",0.8864,"Cardiometabolic_II","B13805","20212097_SS211647","PASS",0.6472,0.1039,"Plate control","PASS","PFC",1.07467467950138,"Baseline","Baseline"
2258,"PFC_13",50,"OID30114","Q92935","EXTL1",0.8864,"Cardiometabolic_II","B13805","20212097_SS211647","PASS",0.6472,0.1634,"Plate control","PASS","PFC",1.11992335429876,"1h","1h"
2259,"PFC_21",51,"OID30114","Q92935","EXTL1",0.8864,"Cardiometabolic_II","B13805","20212097_SS211647","PASS",0.6472,-0.5127,"Plate control","PASS","PFC",0.700909459412176,"24h","24h"
2260,"PFC_06",61,"OID30114","Q92935","EXTL1",0.8864,"Cardiometabolic_II","B13805","20212097_SS211647","PASS",0.6472,0.2788,"Plate control","PASS","PFC",1.21318536538748,"Baseline","Baseline"
2261,"PFC_14",62,"OID30114","Q92935","EXTL1",0.8864,"Cardiometabolic_II","B13805","20212097_SS211647","PASS",0.6472,0.3693,"Plate control","PASS","PFC",1.29172592923243,"1h","1h"
2262,"PFC_22",63,"OID30114","Q92935","EXTL1",0.8864,"Cardiometabolic_II","B13805","20212097_SS211647","PASS",0.6472,0.4551,"Plate control","PASS","PFC",1.37087782369641,"24h","24h"
2263,"PFC_07",73,"OID30114","Q92935","EXTL1",0.8864,"Cardiometabolic_II","B13805","20212097_SS211647","PASS",0.6472,0.6011,"Plate control","PASS","PFC",1.51687268333297,"Baseline","Baseline"
2264,"PFC_15",74,"OID30114","Q92935","EXTL1",0.8864,"Cardiometabolic_II","B13805","20212097_SS211647","PASS",0.6472,-0.5015,"Plate control","PASS","PFC",0.706371969644553,"1h","1h"
2265,"PFC_23",75,"OID30114","Q92935","EXTL1",0.8864,"Cardiometabolic_II","B13805","20212097_SS211647","PASS",0.6472,0.4356,"Plate control","PASS","PFC",1.35247319386904,"24h","24h"
2266,"PFC_08",85,"OID30114","Q92935","EXTL1",0.8864,"Cardiometabolic_II","B13805","20212097_SS211647","PASS",0.6472,0.7904,"Plate control","PASS","PFC",1.72955393031649,"Baseline","Baseline"
2267,"PFC_16",86,"OID30114","Q92935","EXTL1",0.8864,"Cardiometabolic_II","B13805","20212097_SS211647","PASS",0.6472,0.4087,"Plate control","PASS","PFC",1.32748908608445,"1h","1h"
2268,"PFC_24",87,"OID30114","Q92935","EXTL1",0.8864,"Cardiometabolic_II","B13805","20212097_SS211647","PASS",0.6472,0.1095,"Plate control","PASS","PFC",1.07885426930709,"24h","24h"
2269,"PFC_01",1,"OID30115","Q8N8E3","CEP112",0.7727,"Cardiometabolic_II","B13805","20212097_SS211647","WARN",0.5763,0.0028,"Plate control","PASS","PFC",1.0019426967004,"Baseline","Baseline"
2270,"PFC_09",2,"OID30115","Q8N8E3","CEP112",0.7727,"Cardiometabolic_II","B13805","20212097_SS211647","PASS",0.5763,0.3504,"Plate control","PASS","PFC",1.27491405955522,"Baseline","Baseline"
2271,"PFC_17",3,"OID30115","Q8N8E3","CEP112",0.7727,"Cardiometabolic_II","B13805","20212097_SS211647","PASS",0.5763,0.1089,"Plate control","PASS","PFC",1.07840567971814,"1h","1h"
2272,"PFC_25",4,"OID30115","Q8N8E3","CEP112",0.7727,"Cardiometabolic_II","B13805","20212097_SS211647","PASS",0.5763,-0.3726,"Plate control","PASS","PFC",0.772389255180598,"24h","24h"
2273,"PFC_02",13,"OID30115","Q8N8E3","CEP112",0.7727,"Cardiometabolic_II","B13805","20212097_SS211647","PASS",0.5763,-0.1177,"Plate control","PASS","PFC",0.921655819213291,"Baseline","Baseline"
2274,"PFC_10",14,"OID30115","Q8N8E3","CEP112",0.7727,"Cardiometabolic_II","B13805","20212097_SS211647","PASS",0.5763,-0.3403,"Plate control","PASS","PFC",0.789877044470838,"1h","1h"
2275,"PFC_18",15,"OID30115","Q8N8E3","CEP112",0.7727,"Cardiometabolic_II","B13805","20212097_SS211647","PASS",0.5763,-0.1497,"Plate control","PASS","PFC",0.901437891862705,"1h","1h"
2276,"PFC_26",16,"OID30115","Q8N8E3","CEP112",0.7727,"Cardiometabolic_II","B13805","20212097_SS211647","PASS",0.5763,0.2548,"Plate control","PASS","PFC",1.19317032303688,"24h","24h"
2277,"PFC_03",25,"OID30115","Q8N8E3","CEP112",0.7727,"Cardiometabolic_II","B13805","20212097_SS211647","PASS",0.5763,-0.0743,"Plate control","PASS","PFC",0.949802856386761,"Baseline","Baseline"
2278,"PFC_11",26,"OID30115","Q8N8E3","CEP112",0.7727,"Cardiometabolic_II","B13805","20212097_SS211647","PASS",0.5763,0.284,"Plate control","PASS","PFC",1.21756601866299,"1h","1h"
2279,"PFC_19",27,"OID30115","Q8N8E3","CEP112",0.7727,"Cardiometabolic_II","B13805","20212097_SS211647","PASS",0.5763,-0.0643,"Plate control","PASS","PFC",0.956409257698373,"24h","24h"
2280,"PFC_27",28,"OID30115","Q8N8E3","CEP112",0.7727,"Cardiometabolic_II","B13805","20212097_SS211647","PASS",0.5763,0.4069,"Plate control","PASS","PFC",1.32583385731357,"24h","24h"
2281,"PFC_04",37,"OID30115","Q8N8E3","CEP112",0.7727,"Cardiometabolic_II","B13805","20212097_SS211647","PASS",0.5763,0.4126,"Plate control","PASS","PFC",1.33108250763493,"Baseline","Baseline"
2282,"PFC_12",38,"OID30115","Q8N8E3","CEP112",0.7727,"Cardiometabolic_II","B13805","20212097_SS211647","PASS",0.5763,0.0782,"Plate control","PASS","PFC",1.05570005859798,"1h","1h"
2283,"PFC_20",39,"OID30115","Q8N8E3","CEP112",0.7727,"Cardiometabolic_II","B13805","20212097_SS211647","PASS",0.5763,0.2963,"Plate control","PASS","PFC",1.22799101091935,"24h","24h"
2284,"PFC_05",49,"OID30115","Q8N8E3","CEP112",0.7727,"Cardiometabolic_II","B13805","20212097_SS211647","PASS",0.5763,0.0061,"Plate control","PASS","PFC",1.00423714924145,"Baseline","Baseline"
2285,"PFC_13",50,"OID30115","Q8N8E3","CEP112",0.7727,"Cardiometabolic_II","B13805","20212097_SS211647","PASS",0.5763,-0.1246,"Plate control","PASS","PFC",0.917258325960961,"1h","1h"
2286,"PFC_21",51,"OID30115","Q8N8E3","CEP112",0.7727,"Cardiometabolic_II","B13805","20212097_SS211647","PASS",0.5763,-0.4252,"Plate control","PASS","PFC",0.74473548214487,"24h","24h"
2287,"PFC_06",61,"OID30115","Q8N8E3","CEP112",0.7727,"Cardiometabolic_II","B13805","20212097_SS211647","PASS",0.5763,0.3393,"Plate control","PASS","PFC",1.26514259400958,"Baseline","Baseline"
2288,"PFC_14",62,"OID30115","Q8N8E3","CEP112",0.7727,"Cardiometabolic_II","B13805","20212097_SS211647","PASS",0.5763,0.033,"Plate control","PASS","PFC",1.02313746973443,"1h","1h"
2289,"PFC_22",63,"OID30115","Q8N8E3","CEP112",0.7727,"Cardiometabolic_II","B13805","20212097_SS211647","PASS",0.5763,0.1799,"Plate control","PASS","PFC",1.13280536249012,"24h","24h"
2290,"PFC_07",73,"OID30115","Q8N8E3","CEP112",0.7727,"Cardiometabolic_II","B13805","20212097_SS211647","PASS",0.5763,-0.2816,"Plate control","PASS","PFC",0.822678131905787,"Baseline","Baseline"
2291,"PFC_15",74,"OID30115","Q8N8E3","CEP112",0.7727,"Cardiometabolic_II","B13805","20212097_SS211647","PASS",0.5763,0.1226,"Plate control","PASS","PFC",1.08869512310486,"1h","1h"
2292,"PFC_23",75,"OID30115","Q8N8E3","CEP112",0.7727,"Cardiometabolic_II","B13805","20212097_SS211647","PASS",0.5763,0.6076,"Plate control","PASS","PFC",1.52372230623999,"24h","24h"
2293,"PFC_08",85,"OID30115","Q8N8E3","CEP112",0.7727,"Cardiometabolic_II","B13805","20212097_SS211647","PASS",0.5763,0.1495,"Plate control","PASS","PFC",1.10918499122155,"Baseline","Baseline"
2294,"PFC_16",86,"OID30115","Q8N8E3","CEP112",0.7727,"Cardiometabolic_II","B13805","20212097_SS211647","PASS",0.5763,-0.2205,"Plate control","PASS","PFC",0.858267931888545,"1h","1h"
2295,"PFC_24",87,"OID30115","Q8N8E3","CEP112",0.7727,"Cardiometabolic_II","B13805","20212097_SS211647","PASS",0.5763,-0.2968,"Plate control","PASS","PFC",0.814056029376799,"24h","24h"
2296,"PFC_01",1,"OID30116","P16066","NPR1",0.5909,"Cardiometabolic_II","B13805","20212097_SS211647","WARN",-0.0155,-0.2911,"Plate control","PASS","PFC",0.817278677083842,"Baseline","Baseline"
2297,"PFC_09",2,"OID30116","P16066","NPR1",0.5909,"Cardiometabolic_II","B13805","20212097_SS211647","PASS",-0.0155,0.1455,"Plate control","PASS","PFC",1.1061139367768,"Baseline","Baseline"
2298,"PFC_17",3,"OID30116","P16066","NPR1",0.5909,"Cardiometabolic_II","B13805","20212097_SS211647","PASS",-0.0155,0.0872,"Plate control","PASS","PFC",1.06230644323128,"1h","1h"
2299,"PFC_25",4,"OID30116","P16066","NPR1",0.5909,"Cardiometabolic_II","B13805","20212097_SS211647","PASS",-0.0155,-0.7127,"Plate control","PASS","PFC",0.610177124710852,"24h","24h"
2300,"PFC_02",13,"OID30116","P16066","NPR1",0.5909,"Cardiometabolic_II","B13805","20212097_SS211647","PASS",-0.0155,-0.0532,"Plate control","PASS","PFC",0.963796187956976,"Baseline","Baseline"
2301,"PFC_10",14,"OID30116","P16066","NPR1",0.5909,"Cardiometabolic_II","B13805","20212097_SS211647","PASS",-0.0155,-0.4702,"Plate control","PASS","PFC",0.721864519153248,"1h","1h"
2302,"PFC_18",15,"OID30116","P16066","NPR1",0.5909,"Cardiometabolic_II","B13805","20212097_SS211647","PASS",-0.0155,-0.1662,"Plate control","PASS","PFC",0.891186942589785,"1h","1h"
2303,"PFC_26",16,"OID30116","P16066","NPR1",0.5909,"Cardiometabolic_II","B13805","20212097_SS211647","PASS",-0.0155,-0.3028,"Plate control","PASS","PFC",0.81067749585994,"24h","24h"
2304,"PFC_03",25,"OID30116","P16066","NPR1",0.5909,"Cardiometabolic_II","B13805","20212097_SS211647","PASS",-0.0155,1.0692,"Plate control","PASS","PFC",2.09826951690837,"Baseline","Baseline"
2305,"PFC_11",26,"OID30116","P16066","NPR1",0.5909,"Cardiometabolic_II","B13805","20212097_SS211647","PASS",-0.0155,-0.1925,"Plate control","PASS","PFC",0.875087994745209,"1h","1h"
2306,"PFC_19",27,"OID30116","P16066","NPR1",0.5909,"Cardiometabolic_II","B13805","20212097_SS211647","PASS",-0.0155,0.329,"Plate control","PASS","PFC",1.25614238114353,"24h","24h"
2307,"PFC_27",28,"OID30116","P16066","NPR1",0.5909,"Cardiometabolic_II","B13805","20212097_SS211647","PASS",-0.0155,-0.2293,"Plate control","PASS","PFC",0.85304869317858,"24h","24h"
2308,"PFC_04",37,"OID30116","P16066","NPR1",0.5909,"Cardiometabolic_II","B13805","20212097_SS211647","PASS",-0.0155,-0.2019,"Plate control","PASS","PFC",0.869404820551542,"Baseline","Baseline"
2309,"PFC_12",38,"OID30116","P16066","NPR1",0.5909,"Cardiometabolic_II","B13805","20212097_SS211647","PASS",-0.0155,0.75,"Plate control","PASS","PFC",1.68179283050743,"1h","1h"
2310,"PFC_20",39,"OID30116","P16066","NPR1",0.5909,"Cardiometabolic_II","B13805","20212097_SS211647","PASS",-0.0155,-0.0329,"Plate control","PASS","PFC",0.97745351597763,"24h","24h"
2311,"PFC_05",49,"OID30116","P16066","NPR1",0.5909,"Cardiometabolic_II","B13805","20212097_SS211647","PASS",-0.0155,0.1465,"Plate control","PASS","PFC",1.10688090231274,"Baseline","Baseline"
2312,"PFC_13",50,"OID30116","P16066","NPR1",0.5909,"Cardiometabolic_II","B13805","20212097_SS211647","PASS",-0.0155,0.1006,"Plate control","PASS","PFC",1.07221929329002,"1h","1h"
2313,"PFC_21",51,"OID30116","P16066","NPR1",0.5909,"Cardiometabolic_II","B13805","20212097_SS211647","PASS",-0.0155,0.7284,"Plate control","PASS","PFC",1.65680062166565,"24h","24h"
2314,"PFC_06",61,"OID30116","P16066","NPR1",0.5909,"Cardiometabolic_II","B13805","20212097_SS211647","PASS",-0.0155,0.02,"Plate control","PASS","PFC",1.01395947979003,"Baseline","Baseline"
2315,"PFC_14",62,"OID30116","P16066","NPR1",0.5909,"Cardiometabolic_II","B13805","20212097_SS211647","PASS",-0.0155,-0.1746,"Plate control","PASS","PFC",0.88601314005448,"1h","1h"
2316,"PFC_22",63,"OID30116","P16066","NPR1",0.5909,"Cardiometabolic_II","B13805","20212097_SS211647","PASS",-0.0155,0.3016,"Plate control","PASS","PFC",1.23251055360026,"24h","24h"
2317,"PFC_07",73,"OID30116","P16066","NPR1",0.5909,"Cardiometabolic_II","B13805","20212097_SS211647","PASS",-0.0155,0.2305,"Plate control","PASS","PFC",1.17324149329569,"Baseline","Baseline"
2318,"PFC_15",74,"OID30116","P16066","NPR1",0.5909,"Cardiometabolic_II","B13805","20212097_SS211647","PASS",-0.0155,-0.1377,"Plate control","PASS","PFC",0.908967111194767,"1h","1h"
2319,"PFC_23",75,"OID30116","P16066","NPR1",0.5909,"Cardiometabolic_II","B13805","20212097_SS211647","PASS",-0.0155,-0.1468,"Plate control","PASS","PFC",0.903251718749261,"24h","24h"
2320,"PFC_08",85,"OID30116","P16066","NPR1",0.5909,"Cardiometabolic_II","B13805","20212097_SS211647","PASS",-0.0155,-0.2536,"Plate control","PASS","PFC",0.83880071714442,"Baseline","Baseline"
2321,"PFC_16",86,"OID30116","P16066","NPR1",0.5909,"Cardiometabolic_II","B13805","20212097_SS211647","PASS",-0.0155,-0.1588,"Plate control","PASS","PFC",0.895769841551761,"1h","1h"
2322,"PFC_24",87,"OID30116","P16066","NPR1",0.5909,"Cardiometabolic_II","B13805","20212097_SS211647","PASS",-0.0155,0.0964,"Plate control","PASS","PFC",1.06910236823944,"24h","24h"
2323,"PFC_01",1,"OID30117","Q6NZY4","ZCCHC8",0.9545,"Cardiometabolic_II","B13805","20212097_SS211647","WARN",1.1862,-0.2155,"Plate control","PASS","PFC",0.861247622301833,"Baseline","Baseline"
2324,"PFC_09",2,"OID30117","Q6NZY4","ZCCHC8",0.9545,"Cardiometabolic_II","B13805","20212097_SS211647","PASS",1.1862,-0.5002,"Plate control","PASS","PFC",0.707008762166518,"Baseline","Baseline"
2325,"PFC_17",3,"OID30117","Q6NZY4","ZCCHC8",0.9545,"Cardiometabolic_II","B13805","20212097_SS211647","PASS",1.1862,0.1269,"Plate control","PASS","PFC",1.09194485527267,"1h","1h"
2326,"PFC_25",4,"OID30117","Q6NZY4","ZCCHC8",0.9545,"Cardiometabolic_II","B13805","20212097_SS211647","PASS",1.1862,-0.2049,"Plate control","PASS","PFC",0.867598822435677,"24h","24h"
2327,"PFC_02",13,"OID30117","Q6NZY4","ZCCHC8",0.9545,"Cardiometabolic_II","B13805","20212097_SS211647","PASS",1.1862,-0.6243,"Plate control","PASS","PFC",0.648734468902315,"Baseline","Baseline"
2328,"PFC_10",14,"OID30117","Q6NZY4","ZCCHC8",0.9545,"Cardiometabolic_II","B13805","20212097_SS211647","PASS",1.1862,-0.3352,"Plate control","PASS","PFC",0.792674241010026,"1h","1h"
2329,"PFC_18",15,"OID30117","Q6NZY4","ZCCHC8",0.9545,"Cardiometabolic_II","B13805","20212097_SS211647","PASS",1.1862,-0.1052,"Plate control","PASS","PFC",0.929676053212412,"1h","1h"
2330,"PFC_26",16,"OID30117","Q6NZY4","ZCCHC8",0.9545,"Cardiometabolic_II","B13805","20212097_SS211647","PASS",1.1862,-0.1894,"Plate control","PASS","PFC",0.876970367207276,"24h","24h"
2331,"PFC_03",25,"OID30117","Q6NZY4","ZCCHC8",0.9545,"Cardiometabolic_II","B13805","20212097_SS211647","PASS",1.1862,-0.8107,"Plate control","PASS","PFC",0.570105174059898,"Baseline","Baseline"
2332,"PFC_11",26,"OID30117","Q6NZY4","ZCCHC8",0.9545,"Cardiometabolic_II","B13805","20212097_SS211647","PASS",1.1862,-0.1069,"Plate control","PASS","PFC",0.928581214421152,"1h","1h"
2333,"PFC_19",27,"OID30117","Q6NZY4","ZCCHC8",0.9545,"Cardiometabolic_II","B13805","20212097_SS211647","PASS",1.1862,0.3401,"Plate control","PASS","PFC",1.26584433257247,"24h","24h"
2334,"PFC_27",28,"OID30117","Q6NZY4","ZCCHC8",0.9545,"Cardiometabolic_II","B13805","20212097_SS211647","PASS",1.1862,-1.156,"Plate control","PASS","PFC",0.448755025333631,"24h","24h"
2335,"PFC_04",37,"OID30117","Q6NZY4","ZCCHC8",0.9545,"Cardiometabolic_II","B13805","20212097_SS211647","PASS",1.1862,-0.3457,"Plate control","PASS","PFC",0.786926065016083,"Baseline","Baseline"
2336,"PFC_12",38,"OID30117","Q6NZY4","ZCCHC8",0.9545,"Cardiometabolic_II","B13805","20212097_SS211647","PASS",1.1862,-0.0978,"Plate control","PASS","PFC",0.934456881134845,"1h","1h"
2337,"PFC_20",39,"OID30117","Q6NZY4","ZCCHC8",0.9545,"Cardiometabolic_II","B13805","20212097_SS211647","PASS",1.1862,0.2856,"Plate control","PASS","PFC",1.21891709164322,"24h","24h"
2338,"PFC_05",49,"OID30117","Q6NZY4","ZCCHC8",0.9545,"Cardiometabolic_II","B13805","20212097_SS211647","PASS",1.1862,-0.9278,"Plate control","PASS","PFC",0.5256593213483,"Baseline","Baseline"
2339,"PFC_13",50,"OID30117","Q6NZY4","ZCCHC8",0.9545,"Cardiometabolic_II","B13805","20212097_SS211647","PASS",1.1862,0.3906,"Plate control","PASS","PFC",1.3109384944949,"1h","1h"
2340,"PFC_21",51,"OID30117","Q6NZY4","ZCCHC8",0.9545,"Cardiometabolic_II","B13805","20212097_SS211647","PASS",1.1862,-0.3204,"Plate control","PASS","PFC",0.800847804646059,"24h","24h"
2341,"PFC_06",61,"OID30117","Q6NZY4","ZCCHC8",0.9545,"Cardiometabolic_II","B13805","20212097_SS211647","PASS",1.1862,-1.0419,"Plate control","PASS","PFC",0.485687411890112,"Baseline","Baseline"
2342,"PFC_14",62,"OID30117","Q6NZY4","ZCCHC8",0.9545,"Cardiometabolic_II","B13805","20212097_SS211647","PASS",1.1862,-0.6658,"Plate control","PASS","PFC",0.630339073285095,"1h","1h"
2343,"PFC_22",63,"OID30117","Q6NZY4","ZCCHC8",0.9545,"Cardiometabolic_II","B13805","20212097_SS211647","PASS",1.1862,-0.4014,"Plate control","PASS","PFC",0.75712320970757,"24h","24h"
2344,"PFC_07",73,"OID30117","Q6NZY4","ZCCHC8",0.9545,"Cardiometabolic_II","B13805","20212097_SS211647","PASS",1.1862,-0.0225,"Plate control","PASS","PFC",0.984525173337414,"Baseline","Baseline"
2345,"PFC_15",74,"OID30117","Q6NZY4","ZCCHC8",0.9545,"Cardiometabolic_II","B13805","20212097_SS211647","PASS",1.1862,-0.4538,"Plate control","PASS","PFC",0.730117214049524,"1h","1h"
2346,"PFC_23",75,"OID30117","Q6NZY4","ZCCHC8",0.9545,"Cardiometabolic_II","B13805","20212097_SS211647","PASS",1.1862,-0.3515,"Plate control","PASS","PFC",0.783768773442031,"24h","24h"
2347,"PFC_08",85,"OID30117","Q6NZY4","ZCCHC8",0.9545,"Cardiometabolic_II","B13805","20212097_SS211647","PASS",1.1862,-0.0272,"Plate control","PASS","PFC",0.981323014167354,"Baseline","Baseline"
2348,"PFC_16",86,"OID30117","Q6NZY4","ZCCHC8",0.9545,"Cardiometabolic_II","B13805","20212097_SS211647","PASS",1.1862,-0.6843,"Plate control","PASS","PFC",0.622307698987522,"1h","1h"
2349,"PFC_24",87,"OID30117","Q6NZY4","ZCCHC8",0.9545,"Cardiometabolic_II","B13805","20212097_SS211647","PASS",1.1862,-0.4029,"Plate control","PASS","PFC",0.756336422070954,"24h","24h"
2350,"PFC_01",1,"OID30118","P14415","ATP1B2",0.9773,"Cardiometabolic_II","B13805","20212097_SS211647","WARN",0.9198,0.2845,"Plate control","PASS","PFC",1.21798806802083,"Baseline","Baseline"
2351,"PFC_09",2,"OID30118","P14415","ATP1B2",0.9773,"Cardiometabolic_II","B13805","20212097_SS211647","PASS",0.9198,0.0167,"Plate control","PASS","PFC",1.01164281394404,"Baseline","Baseline"
2352,"PFC_17",3,"OID30118","P14415","ATP1B2",0.9773,"Cardiometabolic_II","B13805","20212097_SS211647","PASS",0.9198,0.767,"Plate control","PASS","PFC",1.70172745901308,"1h","1h"

Supplemental table 1_Olink proximity extension assay (PEA) data

,"SampleID","Index","OlinkID","UniProt","Assay","MissingFreq","Panel","Panel_Lot_Nr","PlateID","QC_Warning","LOD","NPX","Normalization","Assay_Warning","condition","Value","group","alt_group"
2353,"PFC_25",4,"OID30118","P14415","ATP1B2",0.9773,"Cardiometabolic_II","B13805","20212097_SS211647","PASS",0.9198,0.4698,"Plate control","PASS","PFC",1.38491746447539,"24h","24h"
2354,"PFC_02",13,"OID30118","P14415","ATP1B2",0.9773,"Cardiometabolic_II","B13805","20212097_SS211647","PASS",0.9198,0.4147,"Plate control","PASS","PFC",1.33302145424997,"Baseline","Baseline"
2355,"PFC_10",14,"OID30118","P14415","ATP1B2",0.9773,"Cardiometabolic_II","B13805","20212097_SS211647","PASS",0.9198,0.4415,"Plate control","PASS","PFC",1.35801555065664,"1h","1h"
2356,"PFC_18",15,"OID30118","P14415","ATP1B2",0.9773,"Cardiometabolic_II","B13805","20212097_SS211647","PASS",0.9198,-0.2101,"Plate control","PASS","PFC",0.864477308230228,"1h","1h"
2357,"PFC_26",16,"OID30118","P14415","ATP1B2",0.9773,"Cardiometabolic_II","B13805","20212097_SS211647","PASS",0.9198,-0.7136,"Plate control","PASS","PFC",0.609796595118277,"24h","24h"
2358,"PFC_03",25,"OID30118","P14415","ATP1B2",0.9773,"Cardiometabolic_II","B13805","20212097_SS211647","PASS",0.9198,0.1434,"Plate control","PASS","PFC",1.10450503853534,"Baseline","Baseline"
2359,"PFC_11",26,"OID30118","P14415","ATP1B2",0.9773,"Cardiometabolic_II","B13805","20212097_SS211647","PASS",0.9198,-0.5322,"Plate control","PASS","PFC",0.691499445682289,"1h","1h"
2360,"PFC_19",27,"OID30118","P14415","ATP1B2",0.9773,"Cardiometabolic_II","B13805","20212097_SS211647","PASS",0.9198,0.6559,"Plate control","PASS","PFC",1.57559855614391,"24h","24h"
2361,"PFC_27",28,"OID30118","P14415","ATP1B2",0.9773,"Cardiometabolic_II","B13805","20212097_SS211647","PASS",0.9198,0.7023,"Plate control","PASS","PFC",1.62709670633658,"24h","24h"
2362,"PFC_04",37,"OID30118","P14415","ATP1B2",0.9773,"Cardiometabolic_II","B13805","20212097_SS211647","PASS",0.9198,-0.0184,"Plate control","PASS","PFC",0.987327078300478,"Baseline","Baseline"
2363,"PFC_12",38,"OID30118","P14415","ATP1B2",0.9773,"Cardiometabolic_II","B13805","20212097_SS211647","PASS",0.9198,0.2545,"Plate control","PASS","PFC",1.19292223603829,"1h","1h"
2364,"PFC_20",39,"OID30118","P14415","ATP1B2",0.9773,"Cardiometabolic_II","B13805","20212097_SS211647","PASS",0.9198,-0.1019,"Plate control","PASS","PFC",0.931805014868271,"24h","24h"
2365,"PFC_05",49,"OID30118","P14415","ATP1B2",0.9773,"Cardiometabolic_II","B13805","20212097_SS211647","PASS",0.9198,0.2935,"Plate control","PASS","PFC",1.22561002237291,"Baseline","Baseline"
2366,"PFC_13",50,"OID30118","P14415","ATP1B2",0.9773,"Cardiometabolic_II","B13805","20212097_SS211647","PASS",0.9198,0.0194,"Plate control","PASS","PFC",1.01353787357388,"1h","1h"
2367,"PFC_21",51,"OID30118","P14415","ATP1B2",0.9773,"Cardiometabolic_II","B13805","20212097_SS211647","PASS",0.9198,-0.4398,"Plate control","PASS","PFC",0.737236804284184,"24h","24h"
2368,"PFC_06",61,"OID30118","P14415","ATP1B2",0.9773,"Cardiometabolic_II","B13805","20212097_SS211647","PASS",0.9198,-0.0473,"Plate control","PASS","PFC",0.967745768893888,"Baseline","Baseline"
2369,"PFC_14",62,"OID30118","P14415","ATP1B2",0.9773,"Cardiometabolic_II","B13805","20212097_SS211647","PASS",0.9198,-0.1334,"Plate control","PASS","PFC",0.911680359006797,"1h","1h"
2370,"PFC_22",63,"OID30118","P14415","ATP1B2",0.9773,"Cardiometabolic_II","B13805","20212097_SS211647","PASS",0.9198,-0.2089,"Plate control","PASS","PFC",0.865196659369112,"24h","24h"
2371,"PFC_07",73,"OID30118","P14415","ATP1B2",0.9773,"Cardiometabolic_II","B13805","20212097_SS211647","PASS",0.9198,0.3081,"Plate control","PASS","PFC",1.23807610475946,"Baseline","Baseline"
2372,"PFC_15",74,"OID30118","P14415","ATP1B2",0.9773,"Cardiometabolic_II","B13805","20212097_SS211647","PASS",0.9198,-0.116,"Plate control","PASS","PFC",0.922742492653798,"1h","1h"
2373,"PFC_23",75,"OID30118","P14415","ATP1B2",0.9773,"Cardiometabolic_II","B13805","20212097_SS211647","PASS",0.9198,0.5473,"Plate control","PASS","PFC",1.46134822577371,"24h","24h"
2374,"PFC_08",85,"OID30118","P14415","ATP1B2",0.9773,"Cardiometabolic_II","B13805","20212097_SS211647","PASS",0.9198,-0.0927,"Plate control","PASS","PFC",0.937766079157789,"Baseline","Baseline"
2375,"PFC_16",86,"OID30118","P14415","ATP1B2",0.9773,"Cardiometabolic_II","B13805","20212097_SS211647","PASS",0.9198,0.2632,"Plate control","PASS","PFC",1.20013774526043,"1h","1h"
2376,"PFC_24",87,"OID30118","P14415","ATP1B2",0.9773,"Cardiometabolic_II","B13805","20212097_SS211647","PASS",0.9198,-0.2548,"Plate control","PASS","PFC",0.838103312404538,"24h","24h"
2377,"PFC_01",1,"OID30119","Q96K76","USP47",0.4091,"Cardiometabolic_II","B13805","20212097_SS211647","WARN",0.361,-0.0625,"Plate control","PASS","PFC",0.957603280698574,"Baseline","Baseline"
2378,"PFC_09",2,"OID30119","Q96K76","USP47",0.4091,"Cardiometabolic_II","B13805","20212097_SS211647","PASS",0.361,0.4656,"Plate control","PASS","PFC",1.38089153063422,"Baseline","Baseline"
2379,"PFC_17",3,"OID30119","Q96K76","USP47",0.4091,"Cardiometabolic_II","B13805","20212097_SS211647","PASS",0.361,0.9326,"Plate control","PASS","PFC",1.90871274736518,"1h","1h"
2380,"PFC_25",4,"OID30119","Q96K76","USP47",0.4091,"Cardiometabolic_II","B13805","20212097_SS211647","PASS",0.361,0.7348,"Plate control","PASS","PFC",1.66416675093295,"24h","24h"
2381,"PFC_02",13,"OID30119","Q96K76","USP47",0.4091,"Cardiometabolic_II","B13805","20212097_SS211647","PASS",0.361,0.1729,"Plate control","PASS","PFC",1.12732226941197,"Baseline","Baseline"
2382,"PFC_10",14,"OID30119","Q96K76","USP47",0.4091,"Cardiometabolic_II","B13805","20212097_SS211647","PASS",0.361,-0.2,"Plate control","PASS","PFC",0.870550563296124,"1h","1h"
2383,"PFC_18",15,"OID30119","Q96K76","USP47",0.4091,"Cardiometabolic_II","B13805","20212097_SS211647","PASS",0.361,0.9008,"Plate control","PASS","PFC",1.86710103672483,"1h","1h"
2384,"PFC_26",16,"OID30119","Q96K76","USP47",0.4091,"Cardiometabolic_II","B13805","20212097_SS211647","PASS",0.361,0.3063,"Plate control","PASS","PFC",1.236532363865,"24h","24h"
2385,"PFC_03",25,"OID30119","Q96K76","USP47",0.4091,"Cardiometabolic_II","B13805","20212097_SS211647","PASS",0.361,0.1417,"Plate control","PASS","PFC",1.10320431130122,"Baseline","Baseline"
2386,"PFC_11",26,"OID30119","Q96K76","USP47",0.4091,"Cardiometabolic_II","B13805","20212097_SS211647","PASS",0.361,1.1363,"Plate control","PASS","PFC",2.19816548329401,"1h","1h"
2387,"PFC_19",27,"OID30119","Q96K76","USP47",0.4091,"Cardiometabolic_II","B13805","20212097_SS211647","PASS",0.361,0.5518,"Plate control","PASS","PFC",1.46591352433724,"24h","24h"
2388,"PFC_27",28,"OID30119","Q96K76","USP47",0.4091,"Cardiometabolic_II","B13805","20212097_SS211647","PASS",0.361,1.566,"Plate control","PASS","PFC",2.9608265960001,"24h","24h"
2389,"PFC_04",37,"OID30119","Q96K76","USP47",0.4091,"Cardiometabolic_II","B13805","20212097_SS211647","PASS",0.361,0.8052,"Plate control","PASS","PFC",1.74738801445852,"Baseline","Baseline"
2390,"PFC_12",38,"OID30119","Q96K76","USP47",0.4091,"Cardiometabolic_II","B13805","20212097_SS211647","PASS",0.361,0.1159,"Plate control","PASS","PFC",1.08365085128828,"1h","1h"
2391,"PFC_20",39,"OID30119","Q96K76","USP47",0.4091,"Cardiometabolic_II","B13805","20212097_SS211647","PASS",0.361,1.0591,"Plate control","PASS","PFC",2.08363127932578,"24h","24h"
2392,"PFC_05",49,"OID30119","Q96K76","USP47",0.4091,"Cardiometabolic_II","B13805","20212097_SS211647","PASS",0.361,0.5628,"Plate control","PASS","PFC",1.47713327523503,"Baseline","Baseline"
2393,"PFC_13",50,"OID30119","Q96K76","USP47",0.4091,"Cardiometabolic_II","B13805","20212097_SS211647","PASS",0.361,0.627,"Plate control","PASS","PFC",1.54435026618357,"1h","1h"
2394,"PFC_21",51,"OID30119","Q96K76","USP47",0.4091,"Cardiometabolic_II","B13805","20212097_SS211647","PASS",0.361,0.1508,"Plate control","PASS","PFC",1.11018491865106,"24h","24h"
2395,"PFC_06",61,"OID30119","Q96K76","USP47",0.4091,"Cardiometabolic_II","B13805","20212097_SS211647","PASS",0.361,0.1663,"Plate control","PASS","PFC",1.1221768063771,"Baseline","Baseline"
2396,"PFC_14",62,"OID30119","Q96K76","USP47",0.4091,"Cardiometabolic_II","B13805","20212097_SS211647","PASS",0.361,0.7953,"Plate control","PASS","PFC",1.73543821101679,"1h","1h"
2397,"PFC_22",63,"OID30119","Q96K76","USP47",0.4091,"Cardiometabolic_II","B13805","20212097_SS211647","PASS",0.361,0.1326,"Plate control","PASS","PFC",1.09626759653012,"24h","24h"
2398,"PFC_07",73,"OID30119","Q96K76","USP47",0.4091,"Cardiometabolic_II","B13805","20212097_SS211647","PASS",0.361,0.6625,"Plate control","PASS","PFC",1.58282307199771,"Baseline","Baseline"
2399,"PFC_15",74,"OID30119","Q96K76","USP47",0.4091,"Cardiometabolic_II","B13805","20212097_SS211647","PASS",0.361,0.1701,"Plate control","PASS","PFC",1.12513647050322,"1h","1h"
2400,"PFC_23",75,"OID30119","Q96K76","USP47",0.4091,"Cardiometabolic_II","B13805","20212097_SS211647","PASS",0.361,0.8241,"Plate control","PASS","PFC",1.77043024177919,"24h","24h"
2401,"PFC_08",85,"OID30119","Q96K76","USP47",0.4091,"Cardiometabolic_II","B13805","20212097_SS211647","PASS",0.361,-0.0335,"Plate control","PASS","PFC",0.977047089008363,"Baseline","Baseline"
2402,"PFC_16",86,"OID30119","Q96K76","USP47",0.4091,"Cardiometabolic_II","B13805","20212097_SS211647","PASS",0.361,1.1255,"Plate control","PASS","PFC",2.18177147869036,"1h","1h"
2403,"PFC_24",87,"OID30119","Q96K76","USP47",0.4091,"Cardiometabolic_II","B13805","20212097_SS211647","PASS",0.361,0.8738,"Plate control","PASS","PFC",1.83248323562381,"24h","24h"
2404,"PFC_01",1,"OID30120","P05976","MYL1",0.7955,"Cardiometabolic_II","B13805","20212097_SS211647","WARN",0.1723,-0.1158,"Plate control","PASS","PFC",0.92287042079233,"Baseline","Baseline"
2405,"PFC_09",2,"OID30120","P05976","MYL1",0.7955,"Cardiometabolic_II","B13805","20212097_SS211647","PASS",0.1723,0.9019,"Plate control","PASS","PFC",1.86852517298202,"Baseline","Baseline"
2406,"PFC_17",3,"OID30120","P05976","MYL1",0.7955,"Cardiometabolic_II","B13805","20212097_SS211647","PASS",0.1723,0.1515,"Plate control","PASS","PFC",1.11072371443555,"1h","1h"
2407,"PFC_25",4,"OID30120","P05976","MYL1",0.7955,"Cardiometabolic_II","B13805","20212097_SS211647","PASS",0.1723,-0.4291,"Plate control","PASS","PFC",0.742724976773793,"24h","24h"
2408,"PFC_02",13,"OID30120","P05976","MYL1",0.7955,"Cardiometabolic_II","B13805","20212097_SS211647","PASS",0.1723,0.1428,"Plate control","PASS","PFC",1.10404578330938,"Baseline","Baseline"
2409,"PFC_10",14,"OID30120","P05976","MYL1",0.7955,"Cardiometabolic_II","B13805","20212097_SS211647","PASS",0.1723,-0.1542,"Plate control","PASS","PFC",0.898630541330418,"1h","1h"
2410,"PFC_18",15,"OID30120","P05976","MYL1",0.7955,"Cardiometabolic_II","B13805","20212097_SS211647","PASS",0.1723,-0.1021,"Plate control","PASS","PFC",0.93167584821785,"1h","1h"
2411,"PFC_26",16,"OID30120","P05976","MYL1",0.7955,"Cardiometabolic_II","B13805","20212097_SS211647","PASS",0.1723,-0.0117,"Plate control","PASS","PFC",0.991922973877825,"24h","24h"
2412,"PFC_03",25,"OID30120","P05976","MYL1",0.7955,"Cardiometabolic_II","B13805","20212097_SS211647","PASS",0.1723,-0.5492,"Plate control","PASS","PFC",0.683398980188516,"Baseline","Baseline"
2413,"PFC_11",26,"OID30120","P05976","MYL1",0.7955,"Cardiometabolic_II","B13805","20212097_SS211647","PASS",0.1723,0.1079,"Plate control","PASS","PFC",1.07765844486352,"1h","1h"
2414,"PFC_19",27,"OID30120","P05976","MYL1",0.7955,"Cardiometabolic_II","B13805","20212097_SS211647","PASS",0.1723,-0.0751,"Plate control","PASS","PFC",0.949276319849586,"24h","24h"
2415,"PFC_27",28,"OID30120","P05976","MYL1",0.7955,"Cardiometabolic_II","B13805","20212097_SS211647","PASS",0.1723,0.2349,"Plate control","PASS","PFC",1.17682516308728,"24h","24h"
2416,"PFC_04",37,"OID30120","P05976","MYL1",0.7955,"Cardiometabolic_II","B13805","20212097_SS211647","PASS",0.1723,-0.2913,"Plate control","PASS","PFC",0.817165386054609,"Baseline","Baseline"
2417,"PFC_12",38,"OID30120","P05976","MYL1",0.7955,"Cardiometabolic_II","B13805","20212097_SS211647","PASS",0.1723,-0.154,"Plate control","PASS","PFC",0.89875512661103,"1h","1h"
2418,"PFC_20",39,"OID30120","P05976","MYL1",0.7955,"Cardiometabolic_II","B13805","20212097_SS211647","PASS",0.1723,0.0402,"Plate control","PASS","PFC",1.02825636337578,"24h","24h"
2419,"PFC_05",49,"OID30120","P05976","MYL1",0.7955,"Cardiometabolic_II","B13805","20212097_SS211647","PASS",0.1723,0.0613,"Plate control","PASS","PFC",1.04340554105528,"Baseline","Baseline"
2420,"PFC_13",50,"OID30120","P05976","MYL1",0.7955,"Cardiometabolic_II","B13805","20212097_SS211647","PASS",0.1723,-0.0212,"Plate control","PASS","PFC",0.985412720260296,"1h","1h"
2421,"PFC_21",51,"OID30120","P05976","MYL1",0.7955,"Cardiometabolic_II","B13805","20212097_SS211647","PASS",0.1723,-0.0971,"Plate control","PASS","PFC",0.93491039245524,"24h","24h"
2422,"PFC_06",61,"OID30120","P05976","MYL1",0.7955,"Cardiometabolic_II","B13805","20212097_SS211647","PASS",0.1723,-0.5563,"Plate control","PASS","PFC",0.6800440003247,"Baseline","Baseline"
2423,"PFC_14",62,"OID30120","P05976","MYL1",0.7955,"Cardiometabolic_II","B13805","20212097_SS211647","PASS",0.1723,-1.0062,"Plate control","PASS","PFC",0.497855854286736,"1h","1h"
2424,"PFC_22",63,"OID30120","P05976","MYL1",0.7955,"Cardiometabolic_II","B13805","20212097_SS211647","PASS",0.1723,-0.0789,"Plate control","PASS","PFC",0.946779254699453,"24h","24h"
2425,"PFC_07",73,"OID30120","P05976","MYL1",0.7955,"Cardiometabolic_II","B13805","20212097_SS211647","PASS",0.1723,-0.2179,"Plate control","PASS","PFC",0.859816082086858,"Baseline","Baseline"
2426,"PFC_15",74,"OID30120","P05976","MYL1",0.7955,"Cardiometabolic_II","B13805","20212097_SS211647","PASS",0.1723,0.1045,"Plate control","PASS","PFC",1.07512171708826,"1h","1h"
2427,"PFC_23",75,"OID30120","P05976","MYL1",0.7955,"Cardiometabolic_II","B13805","20212097_SS211647","PASS",0.1723,-0.0093,"Plate control","PASS","PFC",0.993574463838117,"24h","24h"
2428,"PFC_08",85,"OID30120","P05976","MYL1",0.7955,"Cardiometabolic_II","B13805","20212097_SS211647","PASS",0.1723,-0.2008,"Plate control","PASS","PFC",0.870067961379367,"Baseline","Baseline"
2429,"PFC_16",86,"OID30120","P05976","MYL1",0.7955,"Cardiometabolic_II","B13805","20212097_SS211647","PASS",0.1723,-1.0838,"Plate control","PASS","PFC",0.471784524137031,"1h","1h"
2430,"PFC_24",87,"OID30120","P05976","MYL1",0.7955,"Cardiometabolic_II","B13805","20212097_SS211647","PASS",0.1723,0.1637,"Plate control","PASS","PFC",1.12015626002826,"24h","24h"
2431,"PFC_01",1,"OID30121","Q9Y2Y0","ARL2BP",0.0227,"Cardiometabolic_II","B13805","20212097_SS211647","WARN",-0.5393,1.3813,"Plate control","PASS","PFC",2.60503002358504,"Baseline","Baseline"
2432,"PFC_09",2,"OID30121","Q9Y2Y0","ARL2BP",0.0227,"Cardiometabolic_II","B13805","20212097_SS211647","PASS",-0.5393,1.3444,"Plate control","PASS","PFC",2.53924570284944,"Baseline","Baseline"
2433,"PFC_17",3,"OID30121","Q9Y2Y0","ARL2BP",0.0227,"Cardiometabolic_II","B13805","20212097_SS211647","PASS",-0.5393,2.8225,"Plate control","PASS","PFC",7.07387144074799,"1h","1h"
2434,"PFC_25",4,"OID30121","Q9Y2Y0","ARL2BP",0.0227,"Cardiometabolic_II","B13805","20212097_SS211647","PASS",-0.5393,2.7325,"Plate control","PASS","PFC",6.64606314359803,"24h","24h"
2435,"PFC_02",13,"OID30121","Q9Y2Y0","ARL2BP",0.0227,"Cardiometabolic_II","B13805","20212097_SS211647","PASS",-0.5393,1.354,"Plate control","PASS","PFC",2.5561987264061,"Baseline","Baseline"
2436,"PFC_10",14,"OID30121","Q9Y2Y0","ARL2BP",0.0227,"Cardiometabolic_II","B13805","20212097_SS211647","PASS",-0.5393,1.7068,"Plate control","PASS","PFC",3.26435961690177,"1h","1h"
2437,"PFC_18",15,"OID30121","Q9Y2Y0","ARL2BP",0.0227,"Cardiometabolic_II","B13805","20212097_SS211647","PASS",-0.5393,1.9987,"Plate control","PASS","PFC",3.99639725810461,"1h","1h"
2438,"PFC_26",16,"OID30121","Q9Y2Y0","ARL2BP",0.0227,"Cardiometabolic_II","B13805","20212097_SS211647","PASS",-0.5393,2.5506,"Plate control","PASS","PFC",5.85877887481166,"24h","24h"
2439,"PFC_03",25,"OID30121","Q9Y2Y0","ARL2BP",0.0227,"Cardiometabolic_II","B13805","20212097_SS211647","PASS",-0.5393,0.8134,"Plate control","PASS","PFC",1.757348109292,"Baseline","Baseline"
2440,"PFC_11",26,"OID30121","Q9Y2Y0","ARL2BP",0.0227,"Cardiometabolic_II","B13805","20212097_SS211647","PASS",-0.5393,2.2722,"Plate control","PASS","PFC",4.8305919817732,"1h","1h"
2441,"PFC_19",27,"OID30121","Q9Y2Y0","ARL2BP",0.0227,"Cardiometabolic_II","B13805","20212097_SS211647","PASS",-0.5393,2.16,"Plate control","PASS","PFC",4.46914855228888,"24h","24h"
2442,"PFC_27",28,"OID30121","Q9Y2Y0","ARL2BP",0.0227,"Cardiometabolic_II","B13805","20212097_SS211647","PASS",-0.5393,3.0361,"Plate control","PASS","PFC",8.20270645168912,"24h","24h"
2443,"PFC_04",37,"OID30121","Q9Y2Y0","ARL2BP",0.0227,"Cardiometabolic_II","B13805","20212097_SS211647","PASS",-0.5393,2.6664,"Plate control","PASS","PFC",6.34843066026714,"Baseline","Baseline"
2444,"PFC_12",38,"OID30121","Q9Y2Y0","ARL2BP",0.0227,"Cardiometabolic_II","B13805","20212097_SS211647","PASS",-0.5393,1.2049,"Plate control","PASS","PFC",2.30521290287745,"1h","1h"
2445,"PFC_20",39,"OID30121","Q9Y2Y0","ARL2BP",0.0227,"Cardiometabolic_II","B13805","20212097_SS211647","PASS",-0.5393,2.5529,"Plate control","PASS","PFC",5.86812661503033,"24h","24h"
2446,"PFC_05",49,"OID30121","Q9Y2Y0","ARL2BP",0.0227,"Cardiometabolic_II","B13805","20212097_SS211647","PASS",-0.5393,1.1751,"Plate control","PASS","PFC",2.25808532273536,"Baseline","Baseline"
2447,"PFC_13",50,"OID30121","Q9Y2Y0","ARL2BP",0.0227,"Cardiometabolic_II","B13805","20212097_SS211647","PASS",-0.5393,2.7847,"Plate control","PASS","PFC",6.89093619880183,"1h","1h"
2448,"PFC_21",51,"OID30121","Q9Y2Y0","ARL2BP",0.0227,"Cardiometabolic_II","B13805","20212097_SS211647","PASS",-0.5393,1.7272,"Plate control","PASS","PFC",3.31084621324377,"24h","24h"
2449,"PFC_06",61,"OID30121","Q9Y2Y0","ARL2BP",0.0227,"Cardiometabolic_II","B13805","20212097_SS211647","PASS",-0.5393,2.4047,"Plate control","PASS","PFC",5.29525440988884,"Baseline","Baseline"
2450,"PFC_14",62,"OID30121","Q9Y2Y0","ARL2BP",0.0227,"Cardiometabolic_II","B13805","20212097_SS211647","PASS",-0.5393,2.1523,"Plate control","PASS","PFC",4.44535920497651,"1h","1h"
2451,"PFC_22",63,"OID30121","Q9Y2Y0","ARL2BP",0.0227,"Cardiometabolic_II","B13805","20212097_SS211647","PASS",-0.5393,2.3626,"Plate control","PASS","PFC",5.14296380368385,"24h","24h"
2452,"PFC_07",73,"OID30121","Q9Y2Y0","ARL2BP",0.0227,"Cardiometabolic_II","B13805","20212097_SS211647","PASS",-0.5393,3.1881,"Plate control","PASS","PFC",9.11409873706724,"Baseline","Baseline"
2453,"PFC_15",74,"OID30121","Q9Y2Y0","ARL2BP",0.0227,"Cardiometabolic_II","B13805","20212097_SS211647","PASS",-0.5393,1.3552,"Plate control","PASS","PFC",2.55832579723555,"1h","1h"
2454,"PFC_23",75,"OID30121","Q9Y2Y0","ARL2BP",0.0227,"Cardiometabolic_II","B13805","20212097_SS211647","PASS",-0.5393,2.5672,"Plate control","PASS","PFC",5.92658073368022,"24h","24h"
2455,"PFC_08",85,"OID30121","Q9Y2Y0","ARL2BP",0.0227,"Cardiometabolic_II","B13805","20212097_SS211647","PASS",-0.5393,2.3698,"Plate control","PASS","PFC",5.16869473964418,"Baseline","Baseline"
2456,"PFC_16",86,"OID30121","Q9Y2Y0","ARL2BP",0.0227,"Cardiometabolic_II","B13805","20212097_SS211647","PASS",-0.5393,2.7641,"Plate control","PASS","PFC",6.79324082682853,"1h","1h"
2457,"PFC_24",87,"OID30121","Q9Y2Y0","ARL2BP",0.0227,"Cardiometabolic_II","B13805","20212097_SS211647","PASS",-0.5393,2.6984,"Plate control","PASS","PFC",6.49081663165591,"24h","24h"
2458,"PFC_01",1,"OID30122","Q14088","RAB33A",0.0909,"Cardiometabolic_II","B13805","20212097_SS211647","WARN",0.7039,1.977,"Plate control","PASS","PFC",3.9367360881352,"Baseline","Baseline"
2459,"PFC_09",2,"OID30122","Q14088","RAB33A",0.0909,"Cardiometabolic_II","B13805","20212097_SS211647","PASS",0.7039,2.1249,"Plate control","PASS","PFC",4.36172858819543,"Baseline","Baseline"
2460,"PFC_17",3,"OID30122","Q14088","RAB33A",0.0909,"Cardiometabolic_II","B13805","20212097_SS211647","PASS",0.7039,3.297,"Plate control","PASS","PFC",9.8286958435652,"1h","1h"
2461,"PFC_25",4,"OID30122","Q14088","RAB33A",0.0909,"Cardiometabolic_II","B13805","20212097_SS211647","PASS",0.7039,3.1713,"Plate control","PASS","PFC",9.0085817762563,"24h","24h"
2462,"PFC_02",13,"OID30122","Q14088","RAB33A",0.0909,"Cardiometabolic_II","B13805","20212097_SS211647","PASS",0.7039,1.477,"Plate control","PASS","PFC",2.7836927836622,"Baseline","Baseline"
2463,"PFC_10",14,"OID30122","Q14088","RAB33A",0.0909,"Cardiometabolic_II","B13805","20212097_SS211647","PASS",0.7039,1.6726,"Plate control","PASS","PFC",3.18788591396718,"1h","1h"
2464,"PFC_18",15,"OID30122","Q14088","RAB33A",0.0909,"Cardiometabolic_II","B13805","20212097_SS211647","PASS",0.7039,2.6195,"Plate control","PASS","PFC",6.14537053290999,"1h","1h"

Supplemental table 1_Olink proximity extension assay (PEA) data

,"SampleID","Index","OlinkID","UniProt","Assay","MissingFreq","Panel","Panel_Lot_Nr","PlateID","QC_Warning","LOD","NPX","Normalization","Assay_Warning","condition","Value","group","alt_group"
2465,"PFC_26",16,"OID30122","Q14088","RAB33A",0.0909,"Cardiometabolic_II","B13805","20212097_SS211647","PASS",0.7039,3.2204,"Plate control","PASS","PFC",9.3204525116896,"24h","24h"
2466,"PFC_03",25,"OID30122","Q14088","RAB33A",0.0909,"Cardiometabolic_II","B13805","20212097_SS211647","PASS",0.7039,1.2817,"Plate control","PASS","PFC",2.43125294895988,"Baseline","Baseline"
2467,"PFC_11",26,"OID30122","Q14088","RAB33A",0.0909,"Cardiometabolic_II","B13805","20212097_SS211647","PASS",0.7039,2.2572,"Plate control","PASS","PFC",4.78062750918777,"1h","1h"
2468,"PFC_19",27,"OID30122","Q14088","RAB33A",0.0909,"Cardiometabolic_II","B13805","20212097_SS211647","PASS",0.7039,2.6,"Plate control","PASS","PFC",6.06286626604159,"24h","24h"
2469,"PFC_27",28,"OID30122","Q14088","RAB33A",0.0909,"Cardiometabolic_II","B13805","20212097_SS211647","PASS",0.7039,3.7509,"Plate control","PASS","PFC",13.4627385182996,"24h","24h"
2470,"PFC_04",37,"OID30122","Q14088","RAB33A",0.0909,"Cardiometabolic_II","B13805","20212097_SS211647","PASS",0.7039,2.9194,"Plate control","PASS","PFC",7.56531419373195,"Baseline","Baseline"
2471,"PFC_12",38,"OID30122","Q14088","RAB33A",0.0909,"Cardiometabolic_II","B13805","20212097_SS211647","PASS",0.7039,1.4875,"Plate control","PASS","PFC",2.80402653132277,"1h","1h"
2472,"PFC_20",39,"OID30122","Q14088","RAB33A",0.0909,"Cardiometabolic_II","B13805","20212097_SS211647","PASS",0.7039,2.7998,"Plate control","PASS","PFC",6.96343910181771,"24h","24h"
2473,"PFC_05",49,"OID30122","Q14088","RAB33A",0.0909,"Cardiometabolic_II","B13805","20212097_SS211647","PASS",0.7039,1.6631,"Plate control","PASS","PFC",3.16696297302295,"Baseline","Baseline"
2474,"PFC_13",50,"OID30122","Q14088","RAB33A",0.0909,"Cardiometabolic_II","B13805","20212097_SS211647","PASS",0.7039,3.2175,"Plate control","PASS","PFC",9.30173603763298,"1h","1h"
2475,"PFC_21",51,"OID30122","Q14088","RAB33A",0.0909,"Cardiometabolic_II","B13805","20212097_SS211647","PASS",0.7039,1.8669,"Plate control","PASS","PFC",3.64747982928068,"24h","24h"
2476,"PFC_06",61,"OID30122","Q14088","RAB33A",0.0909,"Cardiometabolic_II","B13805","20212097_SS211647","PASS",0.7039,2.808,"Plate control","PASS","PFC",7.00313063755411,"Baseline","Baseline"
2477,"PFC_14",62,"OID30122","Q14088","RAB33A",0.0909,"Cardiometabolic_II","B13805","20212097_SS211647","PASS",0.7039,2.6331,"Plate control","PASS","PFC",6.20357563496983,"1h","1h"
2478,"PFC_22",63,"OID30122","Q14088","RAB33A",0.0909,"Cardiometabolic_II","B13805","20212097_SS211647","PASS",0.7039,2.7018,"Plate control","PASS","PFC",6.50613158118394,"24h","24h"
2479,"PFC_07",73,"OID30122","Q14088","RAB33A",0.0909,"Cardiometabolic_II","B13805","20212097_SS211647","PASS",0.7039,3.2317,"Plate control","PASS","PFC",9.39374219282406,"Baseline","Baseline"
2480,"PFC_15",74,"OID30122","Q14088","RAB33A",0.0909,"Cardiometabolic_II","B13805","20212097_SS211647","PASS",0.7039,2.029,"Plate control","PASS","PFC",4.08121863699383,"1h","1h"
2481,"PFC_23",75,"OID30122","Q14088","RAB33A",0.0909,"Cardiometabolic_II","B13805","20212097_SS211647","PASS",0.7039,2.8504,"Plate control","PASS","PFC",7.21200301553253,"24h","24h"
2482,"PFC_08",85,"OID30122","Q14088","RAB33A",0.0909,"Cardiometabolic_II","B13805","20212097_SS211647","PASS",0.7039,2.4779,"Plate control","PASS","PFC",5.57085976671909,"Baseline","Baseline"
2483,"PFC_16",86,"OID30122","Q14088","RAB33A",0.0909,"Cardiometabolic_II","B13805","20212097_SS211647","PASS",0.7039,3.0327,"Plate control","PASS","PFC",8.18339789118227,"1h","1h"
2484,"PFC_24",87,"OID30122","Q14088","RAB33A",0.0909,"Cardiometabolic_II","B13805","20212097_SS211647","PASS",0.7039,3.2402,"Plate control","PASS","PFC",9.44925114499993,"24h","24h"
2485,"PFC_01",1,"OID30123","P38935","IGHMBP2",0.0682,"Cardiometabolic_II","B13805","20212097_SS211647","WARN",0.1792,1.2028,"Plate control","PASS","PFC",2.30185985500209,"Baseline","Baseline"
2486,"PFC_09",2,"OID30123","P38935","IGHMBP2",0.0682,"Cardiometabolic_II","B13805","20212097_SS211647","PASS",0.1792,1.3862,"Plate control","PASS","PFC",2.61389284516164,"Baseline","Baseline"
2487,"PFC_17",3,"OID30123","P38935","IGHMBP2",0.0682,"Cardiometabolic_II","B13805","20212097_SS211647","PASS",0.1792,2.4557,"Plate control","PASS","PFC",5.48579229731002,"1h","1h"
2488,"PFC_25",4,"OID30123","P38935","IGHMBP2",0.0682,"Cardiometabolic_II","B13805","20212097_SS211647","PASS",0.1792,2.0507,"Plate control","PASS","PFC",4.14306943758925,"24h","24h"
2489,"PFC_02",13,"OID30123","P38935","IGHMBP2",0.0682,"Cardiometabolic_II","B13805","20212097_SS211647","PASS",0.1792,1.5104,"Plate control","PASS","PFC",2.84889016177698,"Baseline","Baseline"
2490,"PFC_10",14,"OID30123","P38935","IGHMBP2",0.0682,"Cardiometabolic_II","B13805","20212097_SS211647","PASS",0.1792,1.5161,"Plate control","PASS","PFC",2.86016821760625,"1h","1h"
2491,"PFC_18",15,"OID30123","P38935","IGHMBP2",0.0682,"Cardiometabolic_II","B13805","20212097_SS211647","PASS",0.1792,1.6273,"Plate control","PASS","PFC",3.08934287637035,"1h","1h"
2492,"PFC_26",16,"OID30123","P38935","IGHMBP2",0.0682,"Cardiometabolic_II","B13805","20212097_SS211647","PASS",0.1792,2.5478,"Plate control","PASS","PFC",5.84741911299499,"24h","24h"
2493,"PFC_03",25,"OID30123","P38935","IGHMBP2",0.0682,"Cardiometabolic_II","B13805","20212097_SS211647","PASS",0.1792,0.8075,"Plate control","PASS","PFC",1.75017598949042,"Baseline","Baseline"
2494,"PFC_11",26,"OID30123","P38935","IGHMBP2",0.0682,"Cardiometabolic_II","B13805","20212097_SS211647","PASS",0.1792,1.6441,"Plate control","PASS","PFC",3.12552815828416,"1h","1h"
2495,"PFC_19",27,"OID30123","P38935","IGHMBP2",0.0682,"Cardiometabolic_II","B13805","20212097_SS211647","PASS",0.1792,1.8523,"Plate control","PASS","PFC",3.61075366690642,"24h","24h"
2496,"PFC_27",28,"OID30123","P38935","IGHMBP2",0.0682,"Cardiometabolic_II","B13805","20212097_SS211647","PASS",0.1792,2.832,"Plate control","PASS","PFC",7.12060586601997,"24h","24h"
2497,"PFC_04",37,"OID30123","P38935","IGHMBP2",0.0682,"Cardiometabolic_II","B13805","20212097_SS211647","PASS",0.1792,1.8239,"Plate control","PASS","PFC",3.54036965008897,"Baseline","Baseline"
2498,"PFC_12",38,"OID30123","P38935","IGHMBP2",0.0682,"Cardiometabolic_II","B13805","20212097_SS211647","PASS",0.1792,0.163,"Plate control","PASS","PFC",1.11961288865424,"1h","1h"
2499,"PFC_20",39,"OID30123","P38935","IGHMBP2",0.0682,"Cardiometabolic_II","B13805","20212097_SS211647","PASS",0.1792,2.8995,"Plate control","PASS","PFC",7.46167746377138,"24h","24h"
2500,"PFC_05",49,"OID30123","P38935","IGHMBP2",0.0682,"Cardiometabolic_II","B13805","20212097_SS211647","PASS",0.1792,1.1131,"Plate control","PASS","PFC",2.16309945639997,"Baseline","Baseline"
2501,"PFC_13",50,"OID30123","P38935","IGHMBP2",0.0682,"Cardiometabolic_II","B13805","20212097_SS211647","PASS",0.1792,1.4314,"Plate control","PASS","PFC",2.69708315005043,"1h","1h"
2502,"PFC_21",51,"OID30123","P38935","IGHMBP2",0.0682,"Cardiometabolic_II","B13805","20212097_SS211647","PASS",0.1792,0.6504,"Plate control","PASS","PFC",1.5696033219163,"24h","24h"
2503,"PFC_06",61,"OID30123","P38935","IGHMBP2",0.0682,"Cardiometabolic_II","B13805","20212097_SS211647","PASS",0.1792,1.9256,"Plate control","PASS","PFC",3.79894809340475,"Baseline","Baseline"
2504,"PFC_14",62,"OID30123","P38935","IGHMBP2",0.0682,"Cardiometabolic_II","B13805","20212097_SS211647","PASS",0.1792,2.0812,"Plate control","PASS","PFC",4.23159043681009,"1h","1h"
2505,"PFC_22",63,"OID30123","P38935","IGHMBP2",0.0682,"Cardiometabolic_II","B13805","20212097_SS211647","PASS",0.1792,1.6389,"Plate control","PASS","PFC",3.1142828911246,"24h","24h"
2506,"PFC_07",73,"OID30123","P38935","IGHMBP2",0.0682,"Cardiometabolic_II","B13805","20212097_SS211647","PASS",0.1792,2.7075,"Plate control","PASS","PFC",6.53188775676055,"Baseline","Baseline"
2507,"PFC_15",74,"OID30123","P38935","IGHMBP2",0.0682,"Cardiometabolic_II","B13805","20212097_SS211647","PASS",0.1792,1.4242,"Plate control","PASS","PFC",2.68365645775976,"1h","1h"
2508,"PFC_23",75,"OID30123","P38935","IGHMBP2",0.0682,"Cardiometabolic_II","B13805","20212097_SS211647","PASS",0.1792,2.3922,"Plate control","PASS","PFC",5.24957271323178,"24h","24h"
2509,"PFC_08",85,"OID30123","P38935","IGHMBP2",0.0682,"Cardiometabolic_II","B13805","20212097_SS211647","PASS",0.1792,1.6643,"Plate control","PASS","PFC",3.16959827460896,"Baseline","Baseline"
2510,"PFC_16",86,"OID30123","P38935","IGHMBP2",0.0682,"Cardiometabolic_II","B13805","20212097_SS211647","PASS",0.1792,2.012,"Plate control","PASS","PFC",4.03340981957838,"1h","1h"
2511,"PFC_24",87,"OID30123","P38935","IGHMBP2",0.0682,"Cardiometabolic_II","B13805","20212097_SS211647","PASS",0.1792,2.2745,"Plate control","PASS","PFC",4.83829923953337,"24h","24h"
2512,"PFC_01",1,"OID30124","P05026","ATP1B1",0.2727,"Cardiometabolic_II","B13805","20212097_SS211647","WARN",0.279,0.656,"Plate control","PASS","PFC",1.57570777209869,"Baseline","Baseline"
2513,"PFC_09",2,"OID30124","P05026","ATP1B1",0.2727,"Cardiometabolic_II","B13805","20212097_SS211647","PASS",0.279,0.1041,"Plate control","PASS","PFC",1.07482367137329,"Baseline","Baseline"
2514,"PFC_17",3,"OID30124","P05026","ATP1B1",0.2727,"Cardiometabolic_II","B13805","20212097_SS211647","PASS",0.279,1.0056,"Plate control","PASS","PFC",2.00777833494254,"1h","1h"
2515,"PFC_25",4,"OID30124","P05026","ATP1B1",0.2727,"Cardiometabolic_II","B13805","20212097_SS211647","PASS",0.279,0.331,"Plate control","PASS","PFC",1.25788497183602,"24h","24h"
2516,"PFC_02",13,"OID30124","P05026","ATP1B1",0.2727,"Cardiometabolic_II","B13805","20212097_SS211647","PASS",0.279,0.4877,"Plate control","PASS","PFC",1.40220763944248,"Baseline","Baseline"
2517,"PFC_10",14,"OID30124","P05026","ATP1B1",0.2727,"Cardiometabolic_II","B13805","20212097_SS211647","PASS",0.279,0.1183,"Plate control","PASS","PFC",1.08545506245033,"1h","1h"
2518,"PFC_18",15,"OID30124","P05026","ATP1B1",0.2727,"Cardiometabolic_II","B13805","20212097_SS211647","PASS",0.279,0.2569,"Plate control","PASS","PFC",1.19490837724896,"1h","1h"
2519,"PFC_26",16,"OID30124","P05026","ATP1B1",0.2727,"Cardiometabolic_II","B13805","20212097_SS211647","PASS",0.279,0.5955,"Plate control","PASS","PFC",1.51099616619419,"24h","24h"
2520,"PFC_03",25,"OID30124","P05026","ATP1B1",0.2727,"Cardiometabolic_II","B13805","20212097_SS211647","PASS",0.279,0.5156,"Plate control","PASS","PFC",1.42958856529524,"Baseline","Baseline"
2521,"PFC_11",26,"OID30124","P05026","ATP1B1",0.2727,"Cardiometabolic_II","B13805","20212097_SS211647","PASS",0.279,0.5304,"Plate control","PASS","PFC",1.44432959320716,"1h","1h"
2522,"PFC_19",27,"OID30124","P05026","ATP1B1",0.2727,"Cardiometabolic_II","B13805","20212097_SS211647","PASS",0.279,0.5102,"Plate control","PASS","PFC",1.4242476245571,"24h","24h"
2523,"PFC_27",28,"OID30124","P05026","ATP1B1",0.2727,"Cardiometabolic_II","B13805","20212097_SS211647","PASS",0.279,0.9597,"Plate control","PASS","PFC",1.94490542105933,"24h","24h"
2524,"PFC_04",37,"OID30124","P05026","ATP1B1",0.2727,"Cardiometabolic_II","B13805","20212097_SS211647","PASS",0.279,0.4213,"Plate control","PASS","PFC",1.33913369305098,"Baseline","Baseline"
2525,"PFC_12",38,"OID30124","P05026","ATP1B1",0.2727,"Cardiometabolic_II","B13805","20212097_SS211647","PASS",0.279,0.1183,"Plate control","PASS","PFC",1.08545506245033,"1h","1h"
2526,"PFC_20",39,"OID30124","P05026","ATP1B1",0.2727,"Cardiometabolic_II","B13805","20212097_SS211647","PASS",0.279,0.5009,"Plate control","PASS","PFC",1.41509606994203,"24h","24h"
2527,"PFC_05",49,"OID30124","P05026","ATP1B1",0.2727,"Cardiometabolic_II","B13805","20212097_SS211647","PASS",0.279,0.496,"Plate control","PASS","PFC",1.41029796048439,"Baseline","Baseline"
2528,"PFC_13",50,"OID30124","P05026","ATP1B1",0.2727,"Cardiometabolic_II","B13805","20212097_SS211647","PASS",0.279,0.2571,"Plate control","PASS","PFC",1.19507403820598,"1h","1h"
2529,"PFC_21",51,"OID30124","P05026","ATP1B1",0.2727,"Cardiometabolic_II","B13805","20212097_SS211647","PASS",0.279,0.9252,"Plate control","PASS","PFC",1.89894747367226,"24h","24h"
2530,"PFC_06",61,"OID30124","P05026","ATP1B1",0.2727,"Cardiometabolic_II","B13805","20212097_SS211647","PASS",0.279,0.6623,"Plate control","PASS","PFC",1.58260366133651,"Baseline","Baseline"
2531,"PFC_14",62,"OID30124","P05026","ATP1B1",0.2727,"Cardiometabolic_II","B13805","20212097_SS211647","PASS",0.279,0.3691,"Plate control","PASS","PFC",1.29154687040695,"1h","1h"
2532,"PFC_22",63,"OID30124","P05026","ATP1B1",0.2727,"Cardiometabolic_II","B13805","20212097_SS211647","PASS",0.279,0.7968,"Plate control","PASS","PFC",1.73724352051756,"24h","24h"
2533,"PFC_07",73,"OID30124","P05026","ATP1B1",0.2727,"Cardiometabolic_II","B13805","20212097_SS211647","PASS",0.279,0.7899,"Plate control","PASS","PFC",1.72895461646046,"Baseline","Baseline"
2534,"PFC_15",74,"OID30124","P05026","ATP1B1",0.2727,"Cardiometabolic_II","B13805","20212097_SS211647","PASS",0.279,0.2496,"Plate control","PASS","PFC",1.1888774424836,"1h","1h"
2535,"PFC_23",75,"OID30124","P05026","ATP1B1",0.2727,"Cardiometabolic_II","B13805","20212097_SS211647","PASS",0.279,0.786,"Plate control","PASS","PFC",1.72428709003519,"24h","24h"
2536,"PFC_08",85,"OID30124","P05026","ATP1B1",0.2727,"Cardiometabolic_II","B13805","20212097_SS211647","PASS",0.279,1.0799,"Plate control","PASS","PFC",2.11388955238619,"Baseline","Baseline"
2537,"PFC_16",86,"OID30124","P05026","ATP1B1",0.2727,"Cardiometabolic_II","B13805","20212097_SS211647","PASS",0.279,0.4545,"Plate control","PASS","PFC",1.37030781017657,"1h","1h"
2538,"PFC_24",87,"OID30124","P05026","ATP1B1",0.2727,"Cardiometabolic_II","B13805","20212097_SS211647","PASS",0.279,0.4596,"Plate control","PASS","PFC",1.37516048983232,"24h","24h"
2539,"PFC_01",1,"OID30125","O15305","PMM2",0,"Cardiometabolic_II","B13805","20212097_SS211647","WARN",0.8398,2.8071,"Plate control","PASS","PFC",6.9987632197334,"Baseline","Baseline"
2540,"PFC_09",2,"OID30125","O15305","PMM2",0,"Cardiometabolic_II","B13805","20212097_SS211647","PASS",0.8398,3.7517,"Plate control","PASS","PFC",13.4702059159078,"Baseline","Baseline"
2541,"PFC_17",3,"OID30125","O15305","PMM2",0,"Cardiometabolic_II","B13805","20212097_SS211647","PASS",0.8398,5.2971,"Plate control","PASS","PFC",39.3175085618325,"1h","1h"
2542,"PFC_25",4,"OID30125","O15305","PMM2",0,"Cardiometabolic_II","B13805","20212097_SS211647","PASS",0.8398,4.862,"Plate control","PASS","PFC",29.0808997263526,"24h","24h"
2543,"PFC_02",13,"OID30125","O15305","PMM2",0,"Cardiometabolic_II","B13805","20212097_SS211647","PASS",0.8398,2.6404,"Plate control","PASS","PFC",6.23504511923247,"Baseline","Baseline"
2544,"PFC_10",14,"OID30125","O15305","PMM2",0,"Cardiometabolic_II","B13805","20212097_SS211647","PASS",0.8398,3.7266,"Plate control","PASS","PFC",13.2378782292015,"1h","1h"
2545,"PFC_18",15,"OID30125","O15305","PMM2",0,"Cardiometabolic_II","B13805","20212097_SS211647","PASS",0.8398,4.4063,"Plate control","PASS","PFC",21.2045211705245,"1h","1h"
2546,"PFC_26",16,"OID30125","O15305","PMM2",0,"Cardiometabolic_II","B13805","20212097_SS211647","PASS",0.8398,5.202,"Plate control","PASS","PFC",36.8093405872194,"24h","24h"
2547,"PFC_03",25,"OID30125","O15305","PMM2",0,"Cardiometabolic_II","B13805","20212097_SS211647","PASS",0.8398,2.4066,"Plate control","PASS","PFC",5.30223274630639,"Baseline","Baseline"
2548,"PFC_11",26,"OID30125","O15305","PMM2",0,"Cardiometabolic_II","B13805","20212097_SS211647","PASS",0.8398,3.9954,"Plate control","PASS","PFC",15.9490656122251,"1h","1h"
2549,"PFC_19",27,"OID30125","O15305","PMM2",0,"Cardiometabolic_II","B13805","20212097_SS211647","PASS",0.8398,4.3143,"Plate control","PASS","PFC",19.8945312238333,"24h","24h"
2550,"PFC_27",28,"OID30125","O15305","PMM2",0,"Cardiometabolic_II","B13805","20212097_SS211647","PASS",0.8398,5.4951,"Plate control","PASS","PFC",45.1013902463505,"24h","24h"
2551,"PFC_04",37,"OID30125","O15305","PMM2",0,"Cardiometabolic_II","B13805","20212097_SS211647","PASS",0.8398,4.0627,"Plate control","PASS","PFC",16.7106969527675,"Baseline","Baseline"
2552,"PFC_12",38,"OID30125","O15305","PMM2",0,"Cardiometabolic_II","B13805","20212097_SS211647","PASS",0.8398,2.5135,"Plate control","PASS","PFC",5.71003662788034,"1h","1h"
2553,"PFC_20",39,"OID30125","O15305","PMM2",0,"Cardiometabolic_II","B13805","20212097_SS211647","PASS",0.8398,4.3963,"Plate control","PASS","PFC",21.0580508437836,"24h","24h"
2554,"PFC_05",49,"OID30125","O15305","PMM2",0,"Cardiometabolic_II","B13805","20212097_SS211647","PASS",0.8398,2.4478,"Plate control","PASS","PFC",5.45583494776722,"Baseline","Baseline"
2555,"PFC_13",50,"OID30125","O15305","PMM2",0,"Cardiometabolic_II","B13805","20212097_SS211647","PASS",0.8398,4.3346,"Plate control","PASS","PFC",20.1764436677049,"1h","1h"
2556,"PFC_21",51,"OID30125","O15305","PMM2",0,"Cardiometabolic_II","B13805","20212097_SS211647","PASS",0.8398,3.3256,"Plate control","PASS","PFC",10.0254841237263,"24h","24h"
2557,"PFC_06",61,"OID30125","O15305","PMM2",0,"Cardiometabolic_II","B13805","20212097_SS211647","PASS",0.8398,3.8367,"Plate control","PASS","PFC",14.287682288709,"Baseline","Baseline"
2558,"PFC_14",62,"OID30125","O15305","PMM2",0,"Cardiometabolic_II","B13805","20212097_SS211647","PASS",0.8398,3.8568,"Plate control","PASS","PFC",14.4881351070612,"1h","1h"
2559,"PFC_22",63,"OID30125","O15305","PMM2",0,"Cardiometabolic_II","B13805","20212097_SS211647","PASS",0.8398,4.1008,"Plate control","PASS","PFC",17.1578871159925,"24h","24h"
2560,"PFC_07",73,"OID30125","O15305","PMM2",0,"Cardiometabolic_II","B13805","20212097_SS211647","PASS",0.8398,5.1253,"Plate control","PASS","PFC",34.9035046704695,"Baseline","Baseline"
2561,"PFC_15",74,"OID30125","O15305","PMM2",0,"Cardiometabolic_II","B13805","20212097_SS211647","PASS",0.8398,2.983,"Plate control","PASS","PFC",7.90628521200586,"1h","1h"
2562,"PFC_23",75,"OID30125","O15305","PMM2",0,"Cardiometabolic_II","B13805","20212097_SS211647","PASS",0.8398,4.4465,"Plate control","PASS","PFC",21.8036838259283,"24h","24h"
2563,"PFC_08",85,"OID30125","O15305","PMM2",0,"Cardiometabolic_II","B13805","20212097_SS211647","PASS",0.8398,4.2518,"Plate control","PASS","PFC",19.051068367903,"Baseline","Baseline"
2564,"PFC_16",86,"OID30125","O15305","PMM2",0,"Cardiometabolic_II","B13805","20212097_SS211647","PASS",0.8398,4.1543,"Plate control","PASS","PFC",17.8061042196902,"1h","1h"
2565,"PFC_24",87,"OID30125","O15305","PMM2",0,"Cardiometabolic_II","B13805","20212097_SS211647","PASS",0.8398,4.9342,"Plate control","PASS","PFC",30.573292028705,"24h","24h"
2566,"PFC_01",1,"OID30145","P37058","HSD17B3",0.8182,"Cardiometabolic_II","B13805","20212097_SS211647","WARN",0.7767,0.1549,"Plate control","PASS","PFC",1.11334444439781,"Baseline","Baseline"
2567,"PFC_09",2,"OID30145","P37058","HSD17B3",0.8182,"Cardiometabolic_II","B13805","20212097_SS211647","PASS",0.7767,0.5807,"Plate control","PASS","PFC",1.49557473000302,"Baseline","Baseline"
2568,"PFC_17",3,"OID30145","P37058","HSD17B3",0.8182,"Cardiometabolic_II","B13805","20212097_SS211647","PASS",0.7767,0.6233,"Plate control","PASS","PFC",1.5403946312292,"1h","1h"
2569,"PFC_25",4,"OID30145","P37058","HSD17B3",0.8182,"Cardiometabolic_II","B13805","20212097_SS211647","PASS",0.7767,-0.0476,"Plate control","PASS","PFC",0.967544552740112,"24h","24h"
2570,"PFC_02",13,"OID30145","P37058","HSD17B3",0.8182,"Cardiometabolic_II","B13805","20212097_SS211647","PASS",0.7767,0.0806,"Plate control","PASS","PFC",1.05745773343137,"Baseline","Baseline"
2571,"PFC_10",14,"OID30145","P37058","HSD17B3",0.8182,"Cardiometabolic_II","B13805","20212097_SS211647","PASS",0.7767,-0.1511,"Plate control","PASS","PFC",0.900563555375555,"1h","1h"
2572,"PFC_18",15,"OID30145","P37058","HSD17B3",0.8182,"Cardiometabolic_II","B13805","20212097_SS211647","PASS",0.7767,0.2029,"Plate control","PASS","PFC",1.15100970664937,"1h","1h"
2573,"PFC_26",16,"OID30145","P37058","HSD17B3",0.8182,"Cardiometabolic_II","B13805","20212097_SS211647","PASS",0.7767,0.1439,"Plate control","PASS","PFC",1.10488789715254,"24h","24h"
2574,"PFC_03",25,"OID30145","P37058","HSD17B3",0.8182,"Cardiometabolic_II","B13805","20212097_SS211647","PASS",0.7767,0.433,"Plate control","PASS","PFC",1.35003798512275,"Baseline","Baseline"
2575,"PFC_11",26,"OID30145","P37058","HSD17B3",0.8182,"Cardiometabolic_II","B13805","20212097_SS211647","PASS",0.7767,0.5163,"Plate control","PASS","PFC",1.43028237429912,"1h","1h"
2576,"PFC_19",27,"OID30145","P37058","HSD17B3",0.8182,"Cardiometabolic_II","B13805","20212097_SS211647","PASS",0.7767,0.7574,"Plate control","PASS","PFC",1.69044139373121,"24h","24h"

Supplemental table 1_Olink proximity extension assay (PEA) data

,"SampleID","Index","OlinkID","UniProt","Assay","MissingFreq","Panel","Panel_Lot_Nr","PlateID","QC_Warning","LOD","NPX","Normalization","Assay_Warning","condition","Value","group","alt_group"
2577,"PFC_27",28,"OID30145","P37058","HSD17B3",0.8182,"Cardiometabolic_II","B13805","20212097_SS211647","PASS",0.7767,0.0834,"Plate control","PASS","PFC",1.05951205308092,"24h","24h"
2578,"PFC_04",37,"OID30145","P37058","HSD17B3",0.8182,"Cardiometabolic_II","B13805","20212097_SS211647","PASS",0.7767,0.354,"Plate control","PASS","PFC",1.27809936320305,"Baseline","Baseline"
2579,"PFC_12",38,"OID30145","P37058","HSD17B3",0.8182,"Cardiometabolic_II","B13805","20212097_SS211647","PASS",0.7767,0.3111,"Plate control","PASS","PFC",1.2406532902681,"1h","1h"
2580,"PFC_20",39,"OID30145","P37058","HSD17B3",0.8182,"Cardiometabolic_II","B13805","20212097_SS211647","PASS",0.7767,0.2184,"Plate control","PASS","PFC",1.16344257160893,"24h","24h"
2581,"PFC_05",49,"OID30145","P37058","HSD17B3",0.8182,"Cardiometabolic_II","B13805","20212097_SS211647","PASS",0.7767,0.1508,"Plate control","PASS","PFC",1.11018491865106,"Baseline","Baseline"
2582,"PFC_13",50,"OID30145","P37058","HSD17B3",0.8182,"Cardiometabolic_II","B13805","20212097_SS211647","PASS",0.7767,-0.869,"Plate control","PASS","PFC",0.547526235396787,"1h","1h"
2583,"PFC_21",51,"OID30145","P37058","HSD17B3",0.8182,"Cardiometabolic_II","B13805","20212097_SS211647","PASS",0.7767,0.1391,"Plate control","PASS","PFC",1.10121792606267,"24h","24h"
2584,"PFC_06",61,"OID30145","P37058","HSD17B3",0.8182,"Cardiometabolic_II","B13805","20212097_SS211647","PASS",0.7767,0.577,"Plate control","PASS","PFC",1.49174402669147,"Baseline","Baseline"
2585,"PFC_14",62,"OID30145","P37058","HSD17B3",0.8182,"Cardiometabolic_II","B13805","20212097_SS211647","PASS",0.7767,0.022,"Plate control","PASS","PFC",1.01536610086943,"1h","1h"
2586,"PFC_22",63,"OID30145","P37058","HSD17B3",0.8182,"Cardiometabolic_II","B13805","20212097_SS211647","PASS",0.7767,0.0517,"Plate control","PASS","PFC",1.03648554749847,"24h","24h"
2587,"PFC_07",73,"OID30145","P37058","HSD17B3",0.8182,"Cardiometabolic_II","B13805","20212097_SS211647","PASS",0.7767,0.4154,"Plate control","PASS","PFC",1.33366839723047,"Baseline","Baseline"
2588,"PFC_15",74,"OID30145","P37058","HSD17B3",0.8182,"Cardiometabolic_II","B13805","20212097_SS211647","PASS",0.7767,-0.4219,"Plate control","PASS","PFC",0.746440929197922,"1h","1h"
2589,"PFC_23",75,"OID30145","P37058","HSD17B3",0.8182,"Cardiometabolic_II","B13805","20212097_SS211647","PASS",0.7767,0.2201,"Plate control","PASS","PFC",1.16481432244668,"24h","24h"
2590,"PFC_08",85,"OID30145","P37058","HSD17B3",0.8182,"Cardiometabolic_II","B13805","20212097_SS211647","PASS",0.7767,-0.1908,"Plate control","PASS","PFC",0.876119762637488,"Baseline","Baseline"
2591,"PFC_16",86,"OID30145","P37058","HSD17B3",0.8182,"Cardiometabolic_II","B13805","20212097_SS211647","PASS",0.7767,-0.0878,"Plate control","PASS","PFC",0.940956542747424,"1h","1h"
2592,"PFC_24",87,"OID30145","P37058","HSD17B3",0.8182,"Cardiometabolic_II","B13805","20212097_SS211647","PASS",0.7767,0.8077,"Plate control","PASS","PFC",1.75041863221926,"24h","24h"
2593,"PFC_01",1,"OID30146","Q9BZC7","ABCA2",0.0682,"Cardiometabolic_II","B13805","20212097_SS211647","WARN",-0.776,0.0576,"Plate control","PASS","PFC",1.04073300521924,"Baseline","Baseline"
2594,"PFC_09",2,"OID30146","Q9BZC7","ABCA2",0.0682,"Cardiometabolic_II","B13805","20212097_SS211647","PASS",-0.776,0.092,"Plate control","PASS","PFC",1.06584673606794,"Baseline","Baseline"
2595,"PFC_17",3,"OID30146","Q9BZC7","ABCA2",0.0682,"Cardiometabolic_II","B13805","20212097_SS211647","PASS",-0.776,-0.1938,"Plate control","PASS","PFC",0.874299815700005,"1h","1h"
2596,"PFC_25",4,"OID30146","Q9BZC7","ABCA2",0.0682,"Cardiometabolic_II","B13805","20212097_SS211647","PASS",-0.776,-0.1778,"Plate control","PASS","PFC",0.884050077932972,"24h","24h"
2597,"PFC_02",13,"OID30146","Q9BZC7","ABCA2",0.0682,"Cardiometabolic_II","B13805","20212097_SS211647","PASS",-0.776,0.2022,"Plate control","PASS","PFC",1.15045136872088,"Baseline","Baseline"
2598,"PFC_10",14,"OID30146","Q9BZC7","ABCA2",0.0682,"Cardiometabolic_II","B13805","20212097_SS211647","PASS",-0.776,-0.1711,"Plate control","PASS","PFC",0.888165230786188,"1h","1h"
2599,"PFC_18",15,"OID30146","Q9BZC7","ABCA2",0.0682,"Cardiometabolic_II","B13805","20212097_SS211647","PASS",-0.776,-0.101,"Plate control","PASS","PFC",0.932386486436833,"1h","1h"
2600,"PFC_26",16,"OID30146","Q9BZC7","ABCA2",0.0682,"Cardiometabolic_II","B13805","20212097_SS211647","PASS",-0.776,-0.3659,"Plate control","PASS","PFC",0.775984639567286,"24h","24h"
2601,"PFC_03",25,"OID30146","Q9BZC7","ABCA2",0.0682,"Cardiometabolic_II","B13805","20212097_SS211647","PASS",-0.776,-0.0253,"Plate control","PASS","PFC",0.982616247994675,"Baseline","Baseline"
2602,"PFC_11",26,"OID30146","Q9BZC7","ABCA2",0.0682,"Cardiometabolic_II","B13805","20212097_SS211647","PASS",-0.776,0.2807,"Plate control","PASS","PFC",1.21478415837478,"1h","1h"
2603,"PFC_19",27,"OID30146","Q9BZC7","ABCA2",0.0682,"Cardiometabolic_II","B13805","20212097_SS211647","PASS",-0.776,-0.482,"Plate control","PASS","PFC",0.715984370600572,"24h","24h"
2604,"PFC_27",28,"OID30146","Q9BZC7","ABCA2",0.0682,"Cardiometabolic_II","B13805","20212097_SS211647","PASS",-0.776,0.1792,"Plate control","PASS","PFC",1.13225585522201,"24h","24h"
2605,"PFC_04",37,"OID30146","Q9BZC7","ABCA2",0.0682,"Cardiometabolic_II","B13805","20212097_SS211647","PASS",-0.776,-0.2009,"Plate control","PASS","PFC",0.87000765495402,"Baseline","Baseline"
2606,"PFC_12",38,"OID30146","Q9BZC7","ABCA2",0.0682,"Cardiometabolic_II","B13805","20212097_SS211647","PASS",-0.776,-1.1084,"Plate control","PASS","PFC",0.463808125489151,"1h","1h"
2607,"PFC_20",39,"OID30146","Q9BZC7","ABCA2",0.0682,"Cardiometabolic_II","B13805","20212097_SS211647","PASS",-0.776,0.7931,"Plate control","PASS","PFC",1.7327938167525,"24h","24h"
2608,"PFC_05",49,"OID30146","Q9BZC7","ABCA2",0.0682,"Cardiometabolic_II","B13805","20212097_SS211647","PASS",-0.776,-0.5056,"Plate control","PASS","PFC",0.7043673784903,"Baseline","Baseline"
2609,"PFC_13",50,"OID30146","Q9BZC7","ABCA2",0.0682,"Cardiometabolic_II","B13805","20212097_SS211647","PASS",-0.776,0.2165,"Plate control","PASS","PFC",1.16191134994144,"1h","1h"
2610,"PFC_21",51,"OID30146","Q9BZC7","ABCA2",0.0682,"Cardiometabolic_II","B13805","20212097_SS211647","PASS",-0.776,-0.2486,"Plate control","PASS","PFC",0.841712822283936,"24h","24h"
2611,"PFC_06",61,"OID30146","Q9BZC7","ABCA2",0.0682,"Cardiometabolic_II","B13805","20212097_SS211647","PASS",-0.776,0.3644,"Plate control","PASS","PFC",1.28734612596014,"Baseline","Baseline"
2612,"PFC_14",62,"OID30146","Q9BZC7","ABCA2",0.0682,"Cardiometabolic_II","B13805","20212097_SS211647","PASS",-0.776,0.1438,"Plate control","PASS","PFC",1.10481131481364,"1h","1h"
2613,"PFC_22",63,"OID30146","Q9BZC7","ABCA2",0.0682,"Cardiometabolic_II","B13805","20212097_SS211647","PASS",-0.776,0.3939,"Plate control","PASS","PFC",1.3139405485742,"24h","24h"
2614,"PFC_07",73,"OID30146","Q9BZC7","ABCA2",0.0682,"Cardiometabolic_II","B13805","20212097_SS211647","PASS",-0.776,-0.3716,"Plate control","PASS","PFC",0.772924820206372,"Baseline","Baseline"
2615,"PFC_15",74,"OID30146","Q9BZC7","ABCA2",0.0682,"Cardiometabolic_II","B13805","20212097_SS211647","PASS",-0.776,-0.3973,"Plate control","PASS","PFC",0.759277941081009,"1h","1h"
2616,"PFC_23",75,"OID30146","Q9BZC7","ABCA2",0.0682,"Cardiometabolic_II","B13805","20212097_SS211647","PASS",-0.776,0.3516,"Plate control","PASS","PFC",1.27597494440667,"24h","24h"
2617,"PFC_08",85,"OID30146","Q9BZC7","ABCA2",0.0682,"Cardiometabolic_II","B13805","20212097_SS211647","PASS",-0.776,-0.0691,"Plate control","PASS","PFC",0.953232469958292,"Baseline","Baseline"
2618,"PFC_16",86,"OID30146","Q9BZC7","ABCA2",0.0682,"Cardiometabolic_II","B13805","20212097_SS211647","PASS",-0.776,0.2277,"Plate control","PASS","PFC",1.17096666022858,"1h","1h"
2619,"PFC_24",87,"OID30146","Q9BZC7","ABCA2",0.0682,"Cardiometabolic_II","B13805","20212097_SS211647","PASS",-0.776,0.3167,"Plate control","PASS","PFC",1.24547839868773,"24h","24h"
2620,"PFC_01",1,"OID30147","Q6P4F2","FDX2",0.8864,"Cardiometabolic_II","B13805","20212097_SS211647","WARN",1.7438,0.1878,"Plate control","PASS","PFC",1.13902546381888,"Baseline","Baseline"
2621,"PFC_09",2,"OID30147","Q6P4F2","FDX2",0.8864,"Cardiometabolic_II","B13805","20212097_SS211647","PASS",1.7438,0.2822,"Plate control","PASS","PFC",1.2160478515265,"Baseline","Baseline"
2622,"PFC_17",3,"OID30147","Q6P4F2","FDX2",0.8864,"Cardiometabolic_II","B13805","20212097_SS211647","PASS",1.7438,0.8594,"Plate control","PASS","PFC",1.8142836143673,"1h","1h"
2623,"PFC_25",4,"OID30147","Q6P4F2","FDX2",0.8864,"Cardiometabolic_II","B13805","20212097_SS211647","PASS",1.7438,0.2273,"Plate control","PASS","PFC",1.17064204433635,"24h","24h"
2624,"PFC_02",13,"OID30147","Q6P4F2","FDX2",0.8864,"Cardiometabolic_II","B13805","20212097_SS211647","PASS",1.7438,0.6,"Plate control","PASS","PFC",1.5157165665104,"Baseline","Baseline"
2625,"PFC_10",14,"OID30147","Q6P4F2","FDX2",0.8864,"Cardiometabolic_II","B13805","20212097_SS211647","PASS",1.7438,0.0786,"Plate control","PASS","PFC",1.05599280138652,"1h","1h"
2626,"PFC_18",15,"OID30147","Q6P4F2","FDX2",0.8864,"Cardiometabolic_II","B13805","20212097_SS211647","PASS",1.7438,-0.4959,"Plate control","PASS","PFC",0.709119168531511,"1h","1h"
2627,"PFC_26",16,"OID30147","Q6P4F2","FDX2",0.8864,"Cardiometabolic_II","B13805","20212097_SS211647","PASS",1.7438,-0.2964,"Plate control","PASS","PFC",0.814281764925576,"24h","24h"
2628,"PFC_03",25,"OID30147","Q6P4F2","FDX2",0.8864,"Cardiometabolic_II","B13805","20212097_SS211647","PASS",1.7438,-1.4427,"Plate control","PASS","PFC",0.367878176627093,"Baseline","Baseline"
2629,"PFC_11",26,"OID30147","Q6P4F2","FDX2",0.8864,"Cardiometabolic_II","B13805","20212097_SS211647","PASS",1.7438,-0.6395,"Plate control","PASS","PFC",0.641935388085608,"1h","1h"
2630,"PFC_19",27,"OID30147","Q6P4F2","FDX2",0.8864,"Cardiometabolic_II","B13805","20212097_SS211647","PASS",1.7438,0.0256,"Plate control","PASS","PFC",1.01790293801601,"24h","24h"
2631,"PFC_27",28,"OID30147","Q6P4F2","FDX2",0.8864,"Cardiometabolic_II","B13805","20212097_SS211647","PASS",1.7438,0.2764,"Plate control","PASS","PFC",1.21116884471004,"24h","24h"
2632,"PFC_04",37,"OID30147","Q6P4F2","FDX2",0.8864,"Cardiometabolic_II","B13805","20212097_SS211647","PASS",1.7438,0.4944,"Plate control","PASS","PFC",1.4087347569806,"Baseline","Baseline"
2633,"PFC_12",38,"OID30147","Q6P4F2","FDX2",0.8864,"Cardiometabolic_II","B13805","20212097_SS211647","PASS",1.7438,-0.5402,"Plate control","PASS","PFC",0.6876755703851,"1h","1h"
2634,"PFC_20",39,"OID30147","Q6P4F2","FDX2",0.8864,"Cardiometabolic_II","B13805","20212097_SS211647","PASS",1.7438,0.3195,"Plate control","PASS","PFC",1.24789798546328,"24h","24h"
2635,"PFC_05",49,"OID30147","Q6P4F2","FDX2",0.8864,"Cardiometabolic_II","B13805","20212097_SS211647","PASS",1.7438,0.555,"Plate control","PASS","PFC",1.46916863327831,"Baseline","Baseline"
2636,"PFC_13",50,"OID30147","Q6P4F2","FDX2",0.8864,"Cardiometabolic_II","B13805","20212097_SS211647","PASS",1.7438,-0.3047,"Plate control","PASS","PFC",0.809610552826142,"1h","1h"
2637,"PFC_21",51,"OID30147","Q6P4F2","FDX2",0.8864,"Cardiometabolic_II","B13805","20212097_SS211647","PASS",1.7438,0.5509,"Plate control","PASS","PFC",1.46499932507677,"24h","24h"
2638,"PFC_06",61,"OID30147","Q6P4F2","FDX2",0.8864,"Cardiometabolic_II","B13805","20212097_SS211647","PASS",1.7438,0.7287,"Plate control","PASS","PFC",1.6571451794927,"Baseline","Baseline"
2639,"PFC_14",62,"OID30147","Q6P4F2","FDX2",0.8864,"Cardiometabolic_II","B13805","20212097_SS211647","PASS",1.7438,-0.1096,"Plate control","PASS","PFC",0.926845002269281,"1h","1h"
2640,"PFC_22",63,"OID30147","Q6P4F2","FDX2",0.8864,"Cardiometabolic_II","B13805","20212097_SS211647","PASS",1.7438,-0.0158,"Plate control","PASS","PFC",0.989108026364921,"24h","24h"
2641,"PFC_07",73,"OID30147","Q6P4F2","FDX2",0.8864,"Cardiometabolic_II","B13805","20212097_SS211647","PASS",1.7438,0.7777,"Plate control","PASS","PFC",1.71439553766989,"Baseline","Baseline"
2642,"PFC_15",74,"OID30147","Q6P4F2","FDX2",0.8864,"Cardiometabolic_II","B13805","20212097_SS211647","PASS",1.7438,1.1189,"Plate control","PASS","PFC",2.17181316880973,"1h","1h"
2643,"PFC_23",75,"OID30147","Q6P4F2","FDX2",0.8864,"Cardiometabolic_II","B13805","20212097_SS211647","PASS",1.7438,0.3803,"Plate control","PASS","PFC",1.30161249001081,"24h","24h"
2644,"PFC_08",85,"OID30147","Q6P4F2","FDX2",0.8864,"Cardiometabolic_II","B13805","20212097_SS211647","PASS",1.7438,-1.5702,"Plate control","PASS","PFC",0.336761705894917,"Baseline","Baseline"
2645,"PFC_16",86,"OID30147","Q6P4F2","FDX2",0.8864,"Cardiometabolic_II","B13805","20212097_SS211647","PASS",1.7438,1.0395,"Plate control","PASS","PFC",2.05551514258772,"1h","1h"
2646,"PFC_24",87,"OID30147","Q6P4F2","FDX2",0.8864,"Cardiometabolic_II","B13805","20212097_SS211647","PASS",1.7438,-0.1418,"Plate control","PASS","PFC",0.906387581557534,"24h","24h"
2647,"PFC_01",1,"OID30148","Q16774","GUK1",0.9318,"Cardiometabolic_II","B13805","20212097_SS211647","WARN",0.6414,-0.4522,"Plate control","PASS","PFC",0.730927389124374,"Baseline","Baseline"
2648,"PFC_09",2,"OID30148","Q16774","GUK1",0.9318,"Cardiometabolic_II","B13805","20212097_SS211647","PASS",0.6414,-0.6459,"Plate control","PASS","PFC",0.639093978685232,"Baseline","Baseline"
2649,"PFC_17",3,"OID30148","Q16774","GUK1",0.9318,"Cardiometabolic_II","B13805","20212097_SS211647","PASS",0.6414,0.216,"Plate control","PASS","PFC",1.16150873192572,"1h","1h"
2650,"PFC_25",4,"OID30148","Q16774","GUK1",0.9318,"Cardiometabolic_II","B13805","20212097_SS211647","PASS",0.6414,-0.2531,"Plate control","PASS","PFC",0.839091473701852,"24h","24h"
2651,"PFC_02",13,"OID30148","Q16774","GUK1",0.9318,"Cardiometabolic_II","B13805","20212097_SS211647","PASS",0.6414,0.5159,"Plate control","PASS","PFC",1.42988587079066,"Baseline","Baseline"
2652,"PFC_10",14,"OID30148","Q16774","GUK1",0.9318,"Cardiometabolic_II","B13805","20212097_SS211647","PASS",0.6414,-0.273,"Plate control","PASS","PFC",0.827596816078205,"1h","1h"
2653,"PFC_18",15,"OID30148","Q16774","GUK1",0.9318,"Cardiometabolic_II","B13805","20212097_SS211647","PASS",0.6414,0.2479,"Plate control","PASS","PFC",1.18747735356275,"1h","1h"
2654,"PFC_26",16,"OID30148","Q16774","GUK1",0.9318,"Cardiometabolic_II","B13805","20212097_SS211647","PASS",0.6414,-0.4459,"Plate control","PASS","PFC",0.734126202004236,"24h","24h"
2655,"PFC_03",25,"OID30148","Q16774","GUK1",0.9318,"Cardiometabolic_II","B13805","20212097_SS211647","PASS",0.6414,-0.5077,"Plate control","PASS","PFC",0.703342840783187,"Baseline","Baseline"
2656,"PFC_11",26,"OID30148","Q16774","GUK1",0.9318,"Cardiometabolic_II","B13805","20212097_SS211647","PASS",0.6414,0.019,"Plate control","PASS","PFC",1.01325690015909,"1h","1h"
2657,"PFC_19",27,"OID30148","Q16774","GUK1",0.9318,"Cardiometabolic_II","B13805","20212097_SS211647","PASS",0.6414,-0.4876,"Plate control","PASS","PFC",0.713210575231217,"24h","24h"
2658,"PFC_27",28,"OID30148","Q16774","GUK1",0.9318,"Cardiometabolic_II","B13805","20212097_SS211647","PASS",0.6414,-0.7135,"Plate control","PASS","PFC",0.609838864462266,"24h","24h"
2659,"PFC_04",37,"OID30148","Q16774","GUK1",0.9318,"Cardiometabolic_II","B13805","20212097_SS211647","PASS",0.6414,-0.1094,"Plate control","PASS","PFC",0.926973499175829,"Baseline","Baseline"
2660,"PFC_12",38,"OID30148","Q16774","GUK1",0.9318,"Cardiometabolic_II","B13805","20212097_SS211647","PASS",0.6414,0.0213,"Plate control","PASS","PFC",1.01487356166481,"1h","1h"
2661,"PFC_20",39,"OID30148","Q16774","GUK1",0.9318,"Cardiometabolic_II","B13805","20212097_SS211647","PASS",0.6414,0.1275,"Plate control","PASS","PFC",1.0923990768175,"24h","24h"
2662,"PFC_05",49,"OID30148","Q16774","GUK1",0.9318,"Cardiometabolic_II","B13805","20212097_SS211647","PASS",0.6414,-0.0838,"Plate control","PASS","PFC",0.943569048274062,"Baseline","Baseline"
2663,"PFC_13",50,"OID30148","Q16774","GUK1",0.9318,"Cardiometabolic_II","B13805","20212097_SS211647","PASS",0.6414,-0.4682,"Plate control","PASS","PFC",0.722865929830253,"1h","1h"
2664,"PFC_21",51,"OID30148","Q16774","GUK1",0.9318,"Cardiometabolic_II","B13805","20212097_SS211647","PASS",0.6414,-0.5895,"Plate control","PASS","PFC",0.664573190657532,"24h","24h"
2665,"PFC_06",61,"OID30148","Q16774","GUK1",0.9318,"Cardiometabolic_II","B13805","20212097_SS211647","PASS",0.6414,-0.1298,"Plate control","PASS","PFC",0.913958142949514,"Baseline","Baseline"
2666,"PFC_14",62,"OID30148","Q16774","GUK1",0.9318,"Cardiometabolic_II","B13805","20212097_SS211647","PASS",0.6414,-1.0139,"Plate control","PASS","PFC",0.495205759824546,"1h","1h"
2667,"PFC_22",63,"OID30148","Q16774","GUK1",0.9318,"Cardiometabolic_II","B13805","20212097_SS211647","PASS",0.6414,-0.0036,"Plate control","PASS","PFC",0.997507780897529,"24h","24h"
2668,"PFC_07",73,"OID30148","Q16774","GUK1",0.9318,"Cardiometabolic_II","B13805","20212097_SS211647","PASS",0.6414,-0.9288,"Plate control","PASS","PFC",0.525295088319904,"Baseline","Baseline"
2669,"PFC_15",74,"OID30148","Q16774","GUK1",0.9318,"Cardiometabolic_II","B13805","20212097_SS211647","PASS",0.6414,-0.9409,"Plate control","PASS","PFC",0.520907819831446,"1h","1h"
2670,"PFC_23",75,"OID30148","Q16774","GUK1",0.9318,"Cardiometabolic_II","B13805","20212097_SS211647","PASS",0.6414,0.2551,"Plate control","PASS","PFC",1.19341846162907,"24h","24h"
2671,"PFC_08",85,"OID30148","Q16774","GUK1",0.9318,"Cardiometabolic_II","B13805","20212097_SS211647","PASS",0.6414,-0.9935,"Plate control","PASS","PFC",0.502257810751779,"Baseline","Baseline"
2672,"PFC_16",86,"OID30148","Q16774","GUK1",0.9318,"Cardiometabolic_II","B13805","20212097_SS211647","PASS",0.6414,-0.5859,"Plate control","PASS","PFC",0.66623359073908,"1h","1h"
2673,"PFC_24",87,"OID30148","Q16774","GUK1",0.9318,"Cardiometabolic_II","B13805","20212097_SS211647","PASS",0.6414,-0.3896,"Plate control","PASS","PFC",0.763341218268323,"24h","24h"
2674,"PFC_01",1,"OID30149","Q9UNN8","PROCR",0.6136,"Cardiometabolic_II","B13805","20212097_SS211647","WARN",0.2918,-0.1469,"Plate control","PASS","PFC",0.903189112280842,"Baseline","Baseline"
2675,"PFC_09",2,"OID30149","Q9UNN8","PROCR",0.6136,"Cardiometabolic_II","B13805","20212097_SS211647","PASS",0.2918,0.5767,"Plate control","PASS","PFC",1.49143385949145,"Baseline","Baseline"
2676,"PFC_17",3,"OID30149","Q9UNN8","PROCR",0.6136,"Cardiometabolic_II","B13805","20212097_SS211647","PASS",0.2918,-0.1028,"Plate control","PASS","PFC",0.931223905927429,"1h","1h"
2677,"PFC_25",4,"OID30149","Q9UNN8","PROCR",0.6136,"Cardiometabolic_II","B13805","20212097_SS211647","PASS",0.2918,-0.3394,"Plate control","PASS","PFC",0.790369949141548,"24h","24h"
2678,"PFC_02",13,"OID30149","Q9UNN8","PROCR",0.6136,"Cardiometabolic_II","B13805","20212097_SS211647","PASS",0.2918,0.783,"Plate control","PASS","PFC",1.72070526122288,"Baseline","Baseline"
2679,"PFC_10",14,"OID30149","Q9UNN8","PROCR",0.6136,"Cardiometabolic_II","B13805","20212097_SS211647","PASS",0.2918,-0.3238,"Plate control","PASS","PFC",0.798962668516433,"1h","1h"
2680,"PFC_18",15,"OID30149","Q9UNN8","PROCR",0.6136,"Cardiometabolic_II","B13805","20212097_SS211647","PASS",0.2918,0.2072,"Plate control","PASS","PFC",1.15444544654549,"1h","1h"
2681,"PFC_26",16,"OID30149","Q9UNN8","PROCR",0.6136,"Cardiometabolic_II","B13805","20212097_SS211647","PASS",0.2918,0.0509,"Plate control","PASS","PFC",1.0359109571955,"24h","24h"
2682,"PFC_03",25,"OID30149","Q9UNN8","PROCR",0.6136,"Cardiometabolic_II","B13805","20212097_SS211647","PASS",0.2918,0.5869,"Plate control","PASS","PFC",1.50201581153011,"Baseline","Baseline"
2683,"PFC_11",26,"OID30149","Q9UNN8","PROCR",0.6136,"Cardiometabolic_II","B13805","20212097_SS211647","PASS",0.2918,0.6938,"Plate control","PASS","PFC",1.61753844273753,"1h","1h"
2684,"PFC_19",27,"OID30149","Q9UNN8","PROCR",0.6136,"Cardiometabolic_II","B13805","20212097_SS211647","PASS",0.2918,0.1571,"Plate control","PASS","PFC",1.1150435049753,"24h","24h"
2685,"PFC_27",28,"OID30149","Q9UNN8","PROCR",0.6136,"Cardiometabolic_II","B13805","20212097_SS211647","PASS",0.2918,0.492,"Plate control","PASS","PFC",1.40639319991312,"24h","24h"
2686,"PFC_04",37,"OID30149","Q9UNN8","PROCR",0.6136,"Cardiometabolic_II","B13805","20212097_SS211647","PASS",0.2918,0.0096,"Plate control","PASS","PFC",1.00667640139654,"Baseline","Baseline"
2687,"PFC_12",38,"OID30149","Q9UNN8","PROCR",0.6136,"Cardiometabolic_II","B13805","20212097_SS211647","PASS",0.2918,0.3813,"Plate control","PASS","PFC",1.30251501179252,"1h","1h"
2688,"PFC_20",39,"OID30149","Q9UNN8","PROCR",0.6136,"Cardiometabolic_II","B13805","20212097_SS211647","PASS",0.2918,0.4733,"Plate control","PASS","PFC",1.38828137399856,"24h","24h"

Supplemental table 1_Olink proximity extension assay (PEA) data

,"SampleID","Index","OlinkID","UniProt","Assay","MissingFreq","Panel","Panel_Lot_Nr","PlateID","QC_Warning","LOD","NPX","Normalization","Assay_Warning","condition","Value","group","alt_group"
2689,"PFC_05",49,"OID30149","Q9UNN8","PROCR",0.6136,"Cardiometabolic_II","B13805","20212097_SS211647","PASS",0.2918,0.864,"Plate control","PASS","PFC",1.8200776481618,"Baseline","Baseline"
2690,"PFC_13",50,"OID30149","Q9UNN8","PROCR",0.6136,"Cardiometabolic_II","B13805","20212097_SS211647","PASS",0.2918,-0.4603,"Plate control","PASS","PFC",0.72683510183414,"1h","1h"
2691,"PFC_21",51,"OID30149","Q9UNN8","PROCR",0.6136,"Cardiometabolic_II","B13805","20212097_SS211647","PASS",0.2918,-0.4064,"Plate control","PASS","PFC",0.754503762395061,"24h","24h"
2692,"PFC_06",61,"OID30149","Q9UNN8","PROCR",0.6136,"Cardiometabolic_II","B13805","20212097_SS211647","PASS",0.2918,0.5206,"Plate control","PASS","PFC",1.43455173726601,"Baseline","Baseline"
2693,"PFC_14",62,"OID30149","Q9UNN8","PROCR",0.6136,"Cardiometabolic_II","B13805","20212097_SS211647","PASS",0.2918,0.2306,"Plate control","PASS","PFC",1.17332281901751,"1h","1h"
2694,"PFC_22",63,"OID30149","Q9UNN8","PROCR",0.6136,"Cardiometabolic_II","B13805","20212097_SS211647","PASS",0.2918,0.1256,"Plate control","PASS","PFC",1.09096135640329,"24h","24h"
2695,"PFC_07",73,"OID30149","Q9UNN8","PROCR",0.6136,"Cardiometabolic_II","B13805","20212097_SS211647","PASS",0.2918,0.2754,"Plate control","PASS","PFC",1.21032961732779,"Baseline","Baseline"
2696,"PFC_15",74,"OID30149","Q9UNN8","PROCR",0.6136,"Cardiometabolic_II","B13805","20212097_SS211647","PASS",0.2918,-0.7248,"Plate control","PASS","PFC",0.605080920822512,"1h","1h"
2697,"PFC_23",75,"OID30149","Q9UNN8","PROCR",0.6136,"Cardiometabolic_II","B13805","20212097_SS211647","PASS",0.2918,0.0086,"Plate control","PASS","PFC",1.00597886826167,"24h","24h"
2698,"PFC_08",85,"OID30149","Q9UNN8","PROCR",0.6136,"Cardiometabolic_II","B13805","20212097_SS211647","PASS",0.2918,0.2492,"Plate control","PASS","PFC",1.18854786135644,"Baseline","Baseline"
2699,"PFC_16",86,"OID30149","Q9UNN8","PROCR",0.6136,"Cardiometabolic_II","B13805","20212097_SS211647","PASS",0.2918,-0.2313,"Plate control","PASS","PFC",0.851866935906447,"1h","1h"
2700,"PFC_24",87,"OID30149","Q9UNN8","PROCR",0.6136,"Cardiometabolic_II","B13805","20212097_SS211647","PASS",0.2918,-0.4192,"Plate control","PASS","PFC",0.747839199468306,"24h","24h"
2701,"PFC_01",1,"OID30150","P10082","PYY",0.4091,"Cardiometabolic_II","B13805","20212097_SS211647","WARN",0.3367,0.7061,"Plate control","PASS","PFC",1.63138806196264,"Baseline","Baseline"
2702,"PFC_09",2,"OID30150","P10082","PYY",0.4091,"Cardiometabolic_II","B13805","20212097_SS211647","PASS",0.3367,0.7378,"Plate control","PASS","PFC",1.66763088889505,"Baseline","Baseline"
2703,"PFC_17",3,"OID30150","P10082","PYY",0.4091,"Cardiometabolic_II","B13805","20212097_SS211647","PASS",0.3367,0.1184,"Plate control","PASS","PFC",1.08553030306955,"1h","1h"
2704,"PFC_25",4,"OID30150","P10082","PYY",0.4091,"Cardiometabolic_II","B13805","20212097_SS211647","PASS",0.3367,0.6037,"Plate control","PASS","PFC",1.51960882977194,"24h","24h"
2705,"PFC_02",13,"OID30150","P10082","PYY",0.4091,"Cardiometabolic_II","B13805","20212097_SS211647","PASS",0.3367,3.0208,"Plate control","PASS","PFC",8.11617515386965,"Baseline","Baseline"
2706,"PFC_10",14,"OID30150","P10082","PYY",0.4091,"Cardiometabolic_II","B13805","20212097_SS211647","PASS",0.3367,-0.1586,"Plate control","PASS","PFC",0.895894030227686,"1h","1h"
2707,"PFC_18",15,"OID30150","P10082","PYY",0.4091,"Cardiometabolic_II","B13805","20212097_SS211647","PASS",0.3367,-0.0704,"Plate control","PASS","PFC",0.952373907319402,"1h","1h"
2708,"PFC_26",16,"OID30150","P10082","PYY",0.4091,"Cardiometabolic_II","B13805","20212097_SS211647","PASS",0.3367,-0.561,"Plate control","PASS","PFC",0.6778321634,"24h","24h"
2709,"PFC_03",25,"OID30150","P10082","PYY",0.4091,"Cardiometabolic_II","B13805","20212097_SS211647","PASS",0.3367,-0.4234,"Plate control","PASS","PFC",0.745665242378356,"Baseline","Baseline"
2710,"PFC_11",26,"OID30150","P10082","PYY",0.4091,"Cardiometabolic_II","B13805","20212097_SS211647","PASS",0.3367,2.5365,"Plate control","PASS","PFC",5.80179773298966,"1h","1h"
2711,"PFC_19",27,"OID30150","P10082","PYY",0.4091,"Cardiometabolic_II","B13805","20212097_SS211647","PASS",0.3367,0.6105,"Plate control","PASS","PFC",1.52678826176688,"24h","24h"
2712,"PFC_27",28,"OID30150","P10082","PYY",0.4091,"Cardiometabolic_II","B13805","20212097_SS211647","PASS",0.3367,1.319,"Plate control","PASS","PFC",2.49493114382809,"24h","24h"
2713,"PFC_04",37,"OID30150","P10082","PYY",0.4091,"Cardiometabolic_II","B13805","20212097_SS211647","PASS",0.3367,0.4858,"Plate control","PASS","PFC",1.40036217601161,"Baseline","Baseline"
2714,"PFC_12",38,"OID30150","P10082","PYY",0.4091,"Cardiometabolic_II","B13805","20212097_SS211647","PASS",0.3367,-0.2297,"Plate control","PASS","PFC",0.852812210644913,"1h","1h"
2715,"PFC_20",39,"OID30150","P10082","PYY",0.4091,"Cardiometabolic_II","B13805","20212097_SS211647","PASS",0.3367,1.2904,"Plate control","PASS","PFC",2.44595862510927,"24h","24h"
2716,"PFC_05",49,"OID30150","P10082","PYY",0.4091,"Cardiometabolic_II","B13805","20212097_SS211647","PASS",0.3367,1.0537,"Plate control","PASS","PFC",2.0758468359879,"Baseline","Baseline"
2717,"PFC_13",50,"OID30150","P10082","PYY",0.4091,"Cardiometabolic_II","B13805","20212097_SS211647","PASS",0.3367,-0.8528,"Plate control","PASS","PFC",0.553709047294761,"1h","1h"
2718,"PFC_21",51,"OID30150","P10082","PYY",0.4091,"Cardiometabolic_II","B13805","20212097_SS211647","PASS",0.3367,0.5524,"Plate control","PASS","PFC",1.46652330742508,"24h","24h"
2719,"PFC_06",61,"OID30150","P10082","PYY",0.4091,"Cardiometabolic_II","B13805","20212097_SS211647","PASS",0.3367,2.0165,"Plate control","PASS","PFC",4.04601032076673,"Baseline","Baseline"
2720,"PFC_14",62,"OID30150","P10082","PYY",0.4091,"Cardiometabolic_II","B13805","20212097_SS211647","PASS",0.3367,1.0547,"Plate control","PASS","PFC",2.07728620215821,"1h","1h"
2721,"PFC_22",63,"OID30150","P10082","PYY",0.4091,"Cardiometabolic_II","B13805","20212097_SS211647","PASS",0.3367,0.6016,"Plate control","PASS","PFC",1.51739848245361,"24h","24h"
2722,"PFC_07",73,"OID30150","P10082","PYY",0.4091,"Cardiometabolic_II","B13805","20212097_SS211647","PASS",0.3367,-0.0442,"Plate control","PASS","PFC",0.969827454389046,"Baseline","Baseline"
2723,"PFC_15",74,"OID30150","P10082","PYY",0.4091,"Cardiometabolic_II","B13805","20212097_SS211647","PASS",0.3367,0.1453,"Plate control","PASS","PFC",1.10596060745369,"1h","1h"
2724,"PFC_23",75,"OID30150","P10082","PYY",0.4091,"Cardiometabolic_II","B13805","20212097_SS211647","PASS",0.3367,2.02,"Plate control","PASS","PFC",4.05583791916012,"24h","24h"
2725,"PFC_08",85,"OID30150","P10082","PYY",0.4091,"Cardiometabolic_II","B13805","20212097_SS211647","PASS",0.3367,-1.1069,"Plate control","PASS","PFC",0.464290607210576,"Baseline","Baseline"
2726,"PFC_16",86,"OID30150","P10082","PYY",0.4091,"Cardiometabolic_II","B13805","20212097_SS211647","PASS",0.3367,-0.0819,"Plate control","PASS","PFC",0.944812528141572,"1h","1h"
2727,"PFC_24",87,"OID30150","P10082","PYY",0.4091,"Cardiometabolic_II","B13805","20212097_SS211647","PASS",0.3367,4.0004,"Plate control","PASS","PFC",16.0044367569923,"24h","24h"
2728,"PFC_01",1,"OID30151","O15018","PDZD2",0,"Cardiometabolic_II","B13805","20212097_SS211647","WARN",-0.6741,0.7742,"Plate control","PASS","PFC",1.71024142915583,"Baseline","Baseline"
2729,"PFC_09",2,"OID30151","O15018","PDZD2",0,"Cardiometabolic_II","B13805","20212097_SS211647","PASS",-0.6741,2.0815,"Plate control","PASS","PFC",4.23247046279922,"Baseline","Baseline"
2730,"PFC_17",3,"OID30151","O15018","PDZD2",0,"Cardiometabolic_II","B13805","20212097_SS211647","PASS",-0.6741,2.5214,"Plate control","PASS","PFC",5.74138976902212,"1h","1h"
2731,"PFC_25",4,"OID30151","O15018","PDZD2",0,"Cardiometabolic_II","B13805","20212097_SS211647","PASS",-0.6741,2.3227,"Plate control","PASS","PFC",5.00267593506989,"24h","24h"
2732,"PFC_02",13,"OID30151","O15018","PDZD2",0,"Cardiometabolic_II","B13805","20212097_SS211647","PASS",-0.6741,1.4531,"Plate control","PASS","PFC",2.73795740035178,"Baseline","Baseline"
2733,"PFC_10",14,"OID30151","O15018","PDZD2",0,"Cardiometabolic_II","B13805","20212097_SS211647","PASS",-0.6741,1.2892,"Plate control","PASS","PFC",2.44392497980611,"1h","1h"
2734,"PFC_18",15,"OID30151","O15018","PDZD2",0,"Cardiometabolic_II","B13805","20212097_SS211647","PASS",-0.6741,2.48,"Plate control","PASS","PFC",5.57897466540162,"1h","1h"
2735,"PFC_26",16,"OID30151","O15018","PDZD2",0,"Cardiometabolic_II","B13805","20212097_SS211647","PASS",-0.6741,2.2564,"Plate control","PASS","PFC",4.7779773012659,"24h","24h"
2736,"PFC_03",25,"OID30151","O15018","PDZD2",0,"Cardiometabolic_II","B13805","20212097_SS211647","PASS",-0.6741,-0.4553,"Plate control","PASS","PFC",0.729358490515587,"Baseline","Baseline"
2737,"PFC_11",26,"OID30151","O15018","PDZD2",0,"Cardiometabolic_II","B13805","20212097_SS211647","PASS",-0.6741,2.7309,"Plate control","PASS","PFC",6.63869650939478,"1h","1h"
2738,"PFC_19",27,"OID30151","O15018","PDZD2",0,"Cardiometabolic_II","B13805","20212097_SS211647","PASS",-0.6741,1.8042,"Plate control","PASS","PFC",3.49235447410981,"24h","24h"
2739,"PFC_27",28,"OID30151","O15018","PDZD2",0,"Cardiometabolic_II","B13805","20212097_SS211647","PASS",-0.6741,2.5149,"Plate control","PASS","PFC",5.71558037139175,"24h","24h"
2740,"PFC_04",37,"OID30151","O15018","PDZD2",0,"Cardiometabolic_II","B13805","20212097_SS211647","PASS",-0.6741,2.66,"Plate control","PASS","PFC",6.32033049490702,"Baseline","Baseline"
2741,"PFC_12",38,"OID30151","O15018","PDZD2",0,"Cardiometabolic_II","B13805","20212097_SS211647","PASS",-0.6741,-0.2516,"Plate control","PASS","PFC",0.83996434822995,"1h","1h"
2742,"PFC_20",39,"OID30151","O15018","PDZD2",0,"Cardiometabolic_II","B13805","20212097_SS211647","PASS",-0.6741,3.2538,"Plate control","PASS","PFC",9.5387485356517,"24h","24h"
2743,"PFC_05",49,"OID30151","O15018","PDZD2",0,"Cardiometabolic_II","B13805","20212097_SS211647","PASS",-0.6741,0.7282,"Plate control","PASS","PFC",1.65657095624928,"Baseline","Baseline"
2744,"PFC_13",50,"OID30151","O15018","PDZD2",0,"Cardiometabolic_II","B13805","20212097_SS211647","PASS",-0.6741,2.6667,"Plate control","PASS","PFC",6.34975091657612,"1h","1h"
2745,"PFC_21",51,"OID30151","O15018","PDZD2",0,"Cardiometabolic_II","B13805","20212097_SS211647","PASS",-0.6741,-0.2439,"Plate control","PASS","PFC",0.844459418861742,"24h","24h"
2746,"PFC_06",61,"OID30151","O15018","PDZD2",0,"Cardiometabolic_II","B13805","20212097_SS211647","PASS",-0.6741,1.7642,"Plate control","PASS","PFC",3.39685585736033,"Baseline","Baseline"
2747,"PFC_14",62,"OID30151","O15018","PDZD2",0,"Cardiometabolic_II","B13805","20212097_SS211647","PASS",-0.6741,1.9022,"Plate control","PASS","PFC",3.73782752453938,"1h","1h"
2748,"PFC_22",63,"OID30151","O15018","PDZD2",0,"Cardiometabolic_II","B13805","20212097_SS211647","PASS",-0.6741,2.1968,"Plate control","PASS","PFC",4.58461313652374,"24h","24h"
2749,"PFC_07",73,"OID30151","O15018","PDZD2",0,"Cardiometabolic_II","B13805","20212097_SS211647","PASS",-0.6741,2.3097,"Plate control","PASS","PFC",4.95779974708403,"Baseline","Baseline"
2750,"PFC_15",74,"OID30151","O15018","PDZD2",0,"Cardiometabolic_II","B13805","20212097_SS211647","PASS",-0.6741,1.3398,"Plate control","PASS","PFC",2.53116227001914,"1h","1h"
2751,"PFC_23",75,"OID30151","O15018","PDZD2",0,"Cardiometabolic_II","B13805","20212097_SS211647","PASS",-0.6741,1.9788,"Plate control","PASS","PFC",3.94165088104118,"24h","24h"
2752,"PFC_08",85,"OID30151","O15018","PDZD2",0,"Cardiometabolic_II","B13805","20212097_SS211647","PASS",-0.6741,1.9052,"Plate control","PASS","PFC",3.74560820530125,"Baseline","Baseline"
2753,"PFC_16",86,"OID30151","O15018","PDZD2",0,"Cardiometabolic_II","B13805","20212097_SS211647","PASS",-0.6741,2.5138,"Plate control","PASS","PFC",5.71122412007895,"1h","1h"
2754,"PFC_24",87,"OID30151","O15018","PDZD2",0,"Cardiometabolic_II","B13805","20212097_SS211647","PASS",-0.6741,2.5799,"Plate control","PASS","PFC",5.9789825486867,"24h","24h"
2755,"PFC_01",1,"OID30152","Q16206","ENOX2",0.2955,"Cardiometabolic_II","B13805","20212097_SS211647","WARN",0.4071,0.2696,"Plate control","PASS","PFC",1.20547355311477,"Baseline","Baseline"
2756,"PFC_09",2,"OID30152","Q16206","ENOX2",0.2955,"Cardiometabolic_II","B13805","20212097_SS211647","PASS",0.4071,1.0453,"Plate control","PASS","PFC",2.06379546818876,"Baseline","Baseline"
2757,"PFC_17",3,"OID30152","Q16206","ENOX2",0.2955,"Cardiometabolic_II","B13805","20212097_SS211647","PASS",0.4071,0.4513,"Plate control","PASS","PFC",1.36727173854811,"1h","1h"
2758,"PFC_25",4,"OID30152","Q16206","ENOX2",0.2955,"Cardiometabolic_II","B13805","20212097_SS211647","PASS",0.4071,0.4848,"Plate control","PASS","PFC",1.39939185524394,"24h","24h"
2759,"PFC_02",13,"OID30152","Q16206","ENOX2",0.2955,"Cardiometabolic_II","B13805","20212097_SS211647","PASS",0.4071,0.7812,"Plate control","PASS","PFC",1.71855973634856,"Baseline","Baseline"
2760,"PFC_10",14,"OID30152","Q16206","ENOX2",0.2955,"Cardiometabolic_II","B13805","20212097_SS211647","PASS",0.4071,0.7643,"Plate control","PASS","PFC",1.69854565881335,"1h","1h"
2761,"PFC_18",15,"OID30152","Q16206","ENOX2",0.2955,"Cardiometabolic_II","B13805","20212097_SS211647","PASS",0.4071,0.4718,"Plate control","PASS","PFC",1.38683869913771,"1h","1h"
2762,"PFC_26",16,"OID30152","Q16206","ENOX2",0.2955,"Cardiometabolic_II","B13805","20212097_SS211647","PASS",0.4071,0.5864,"Plate control","PASS","PFC",1.50149534271324,"24h","24h"
2763,"PFC_03",25,"OID30152","Q16206","ENOX2",0.2955,"Cardiometabolic_II","B13805","20212097_SS211647","PASS",0.4071,0.2309,"Plate control","PASS","PFC",1.17356683000813,"Baseline","Baseline"
2764,"PFC_11",26,"OID30152","Q16206","ENOX2",0.2955,"Cardiometabolic_II","B13805","20212097_SS211647","PASS",0.4071,1.4394,"Plate control","PASS","PFC",2.71208049774764,"1h","1h"
2765,"PFC_19",27,"OID30152","Q16206","ENOX2",0.2955,"Cardiometabolic_II","B13805","20212097_SS211647","PASS",0.4071,0.3913,"Plate control","PASS","PFC",1.31157472015667,"24h","24h"
2766,"PFC_27",28,"OID30152","Q16206","ENOX2",0.2955,"Cardiometabolic_II","B13805","20212097_SS211647","PASS",0.4071,0.8682,"Plate control","PASS","PFC",1.82538401150369,"24h","24h"
2767,"PFC_04",37,"OID30152","Q16206","ENOX2",0.2955,"Cardiometabolic_II","B13805","20212097_SS211647","PASS",0.4071,0.9388,"Plate control","PASS","PFC",1.91693311520835,"Baseline","Baseline"
2768,"PFC_12",38,"OID30152","Q16206","ENOX2",0.2955,"Cardiometabolic_II","B13805","20212097_SS211647","PASS",0.4071,0.6503,"Plate control","PASS","PFC",1.5694945290751,"1h","1h"
2769,"PFC_20",39,"OID30152","Q16206","ENOX2",0.2955,"Cardiometabolic_II","B13805","20212097_SS211647","PASS",0.4071,1.6238,"Plate control","PASS","PFC",3.08185716769718,"24h","24h"
2770,"PFC_05",49,"OID30152","Q16206","ENOX2",0.2955,"Cardiometabolic_II","B13805","20212097_SS211647","PASS",0.4071,0.4372,"Plate control","PASS","PFC",1.35397396669016,"Baseline","Baseline"
2771,"PFC_13",50,"OID30152","Q16206","ENOX2",0.2955,"Cardiometabolic_II","B13805","20212097_SS211647","PASS",0.4071,1.1197,"Plate control","PASS","PFC",2.17301781171653,"1h","1h"
2772,"PFC_21",51,"OID30152","Q16206","ENOX2",0.2955,"Cardiometabolic_II","B13805","20212097_SS211647","PASS",0.4071,0.0987,"Plate control","PASS","PFC",1.07080813174733,"24h","24h"
2773,"PFC_06",61,"OID30152","Q16206","ENOX2",0.2955,"Cardiometabolic_II","B13805","20212097_SS211647","PASS",0.4071,0.6471,"Plate control","PASS","PFC",1.56601713678749,"Baseline","Baseline"
2774,"PFC_14",62,"OID30152","Q16206","ENOX2",0.2955,"Cardiometabolic_II","B13805","20212097_SS211647","PASS",0.4071,0.7328,"Plate control","PASS","PFC",1.66186132431935,"1h","1h"
2775,"PFC_22",63,"OID30152","Q16206","ENOX2",0.2955,"Cardiometabolic_II","B13805","20212097_SS211647","PASS",0.4071,0.6388,"Plate control","PASS","PFC",1.55703351648261,"24h","24h"
2776,"PFC_07",73,"OID30152","Q16206","ENOX2",0.2955,"Cardiometabolic_II","B13805","20212097_SS211647","PASS",0.4071,0.8977,"Plate control","PASS","PFC",1.86309339894498,"Baseline","Baseline"
2777,"PFC_15",74,"OID30152","Q16206","ENOX2",0.2955,"Cardiometabolic_II","B13805","20212097_SS211647","PASS",0.4071,0.3918,"Plate control","PASS","PFC",1.31202935609421,"1h","1h"
2778,"PFC_23",75,"OID30152","Q16206","ENOX2",0.2955,"Cardiometabolic_II","B13805","20212097_SS211647","PASS",0.4071,0.8781,"Plate control","PASS","PFC",1.83795315974802,"24h","24h"
2779,"PFC_08",85,"OID30152","Q16206","ENOX2",0.2955,"Cardiometabolic_II","B13805","20212097_SS211647","PASS",0.4071,0.7277,"Plate control","PASS","PFC",1.65599693198199,"Baseline","Baseline"
2780,"PFC_16",86,"OID30152","Q16206","ENOX2",0.2955,"Cardiometabolic_II","B13805","20212097_SS211647","PASS",0.4071,0.8618,"Plate control","PASS","PFC",1.81730428356557,"1h","1h"
2781,"PFC_24",87,"OID30152","Q16206","ENOX2",0.2955,"Cardiometabolic_II","B13805","20212097_SS211647","PASS",0.4071,1.1953,"Plate control","PASS","PFC",2.28992444809422,"24h","24h"
2782,"PFC_01",1,"OID30153","P0C7L1","SPINK8",0.6591,"Cardiometabolic_II","B13805","20212097_SS211647","WARN",0.2944,0.0939,"Plate control","PASS","PFC",1.06725135924902,"Baseline","Baseline"
2783,"PFC_09",2,"OID30153","P0C7L1","SPINK8",0.6591,"Cardiometabolic_II","B13805","20212097_SS211647","PASS",0.2944,0.2257,"Plate control","PASS","PFC",1.1693444804196,"Baseline","Baseline"
2784,"PFC_17",3,"OID30153","P0C7L1","SPINK8",0.6591,"Cardiometabolic_II","B13805","20212097_SS211647","PASS",0.2944,-0.2994,"Plate control","PASS","PFC",0.812590272885877,"1h","1h"
2785,"PFC_25",4,"OID30153","P0C7L1","SPINK8",0.6591,"Cardiometabolic_II","B13805","20212097_SS211647","PASS",0.2944,-0.056,"Plate control","PASS","PFC",0.961927454664775,"24h","24h"
2786,"PFC_02",13,"OID30153","P0C7L1","SPINK8",0.6591,"Cardiometabolic_II","B13805","20212097_SS211647","PASS",0.2944,-0.2777,"Plate control","PASS","PFC",0.824905064962554,"Baseline","Baseline"
2787,"PFC_10",14,"OID30153","P0C7L1","SPINK8",0.6591,"Cardiometabolic_II","B13805","20212097_SS211647","PASS",0.2944,0.1755,"Plate control","PASS","PFC",1.12935574186283,"1h","1h"
2788,"PFC_18",15,"OID30153","P0C7L1","SPINK8",0.6591,"Cardiometabolic_II","B13805","20212097_SS211647","PASS",0.2944,-0.0787,"Plate control","PASS","PFC",0.946910515271733,"1h","1h"
2789,"PFC_26",16,"OID30153","P0C7L1","SPINK8",0.6591,"Cardiometabolic_II","B13805","20212097_SS211647","PASS",0.2944,0.5451,"Plate control","PASS","PFC",1.45912147932901,"24h","24h"
2790,"PFC_03",25,"OID30153","P0C7L1","SPINK8",0.6591,"Cardiometabolic_II","B13805","20212097_SS211647","PASS",0.2944,-0.236,"Plate control","PASS","PFC",0.84909624644684,"Baseline","Baseline"
2791,"PFC_11",26,"OID30153","P0C7L1","SPINK8",0.6591,"Cardiometabolic_II","B13805","20212097_SS211647","PASS",0.2944,-0.5252,"Plate control","PASS","PFC",0.694862774809235,"1h","1h"
2792,"PFC_19",27,"OID30153","P0C7L1","SPINK8",0.6591,"Cardiometabolic_II","B13805","20212097_SS211647","PASS",0.2944,0.4903,"Plate control","PASS","PFC",1.4047369522065,"24h","24h"
2793,"PFC_27",28,"OID30153","P0C7L1","SPINK8",0.6591,"Cardiometabolic_II","B13805","20212097_SS211647","PASS",0.2944,-0.0664,"Plate control","PASS","PFC",0.955018112426933,"24h","24h"
2794,"PFC_04",37,"OID30153","P0C7L1","SPINK8",0.6591,"Cardiometabolic_II","B13805","20212097_SS211647","PASS",0.2944,0.1568,"Plate control","PASS","PFC",1.11481166230279,"Baseline","Baseline"
2795,"PFC_12",38,"OID30153","P0C7L1","SPINK8",0.6591,"Cardiometabolic_II","B13805","20212097_SS211647","PASS",0.2944,-0.1291,"Plate control","PASS","PFC",0.914401705806788,"1h","1h"
2796,"PFC_20",39,"OID30153","P0C7L1","SPINK8",0.6591,"Cardiometabolic_II","B13805","20212097_SS211647","PASS",0.2944,-0.6803,"Plate control","PASS","PFC",0.624035496424508,"24h","24h"
2797,"PFC_05",49,"OID30153","P0C7L1","SPINK8",0.6591,"Cardiometabolic_II","B13805","20212097_SS211647","PASS",0.2944,0.1712,"Plate control","PASS","PFC",1.12599467132386,"Baseline","Baseline"
2798,"PFC_13",50,"OID30153","P0C7L1","SPINK8",0.6591,"Cardiometabolic_II","B13805","20212097_SS211647","PASS",0.2944,0.0866,"Plate control","PASS","PFC",1.06186473425884,"1h","1h"
2799,"PFC_21",51,"OID30153","P0C7L1","SPINK8",0.6591,"Cardiometabolic_II","B13805","20212097_SS211647","PASS",0.2944,-1.0142,"Plate control","PASS","PFC",0.495102795387478,"24h","24h"
2800,"PFC_06",61,"OID30153","P0C7L1","SPINK8",0.6591,"Cardiometabolic_II","B13805","20212097_SS211647","PASS",0.2944,-0.379,"Plate control","PASS","PFC",0.768970415635134,"Baseline","Baseline"

Supplemental table 1_Olink proximity extension assay (PEA) data

**,"SampleID","Index","OlinkID","UniProt","Assay","MissingFreq","Panel","Panel_Lot_Nr","PlateID","QC_Warning","LOD","NPX","Normalization","Assay_Warning","condition","Value","group","alt_group"**
2801,"PFC_14",62,"OID30153","P0C7L1","SPINK8",0.6591,"Cardiometabolic_II","B13805","20212097_SS211647","PASS",0.2944,0.2465,"Plate control","PASS","PFC",1.18632557728703,"1h","1h"
2802,"PFC_22",63,"OID30153","P0C7L1","SPINK8",0.6591,"Cardiometabolic_II","B13805","20212097_SS211647","PASS",0.2944,-0.3447,"Plate control","PASS","PFC",0.787471709683543,"24h","24h"
2803,"PFC_07",73,"OID30153","P0C7L1","SPINK8",0.6591,"Cardiometabolic_II","B13805","20212097_SS211647","PASS",0.2944,-0.5594,"Plate control","PASS","PFC",0.678584320331998,"Baseline","Baseline"
2804,"PFC_15",74,"OID30153","P0C7L1","SPINK8",0.6591,"Cardiometabolic_II","B13805","20212097_SS211647","PASS",0.2944,0.0116,"Plate control","PASS","PFC",1.00807291898384,"1h","1h"
2805,"PFC_23",75,"OID30153","P0C7L1","SPINK8",0.6591,"Cardiometabolic_II","B13805","20212097_SS211647","PASS",0.2944,0.3872,"Plate control","PASS","PFC",1.3078526428445,"24h","24h"
2806,"PFC_08",85,"OID30153","P0C7L1","SPINK8",0.6591,"Cardiometabolic_II","B13805","20212097_SS211647","PASS",0.2944,0.8447,"Plate control","PASS","PFC",1.79589126184789,"Baseline","Baseline"
2807,"PFC_16",86,"OID30153","P0C7L1","SPINK8",0.6591,"Cardiometabolic_II","B13805","20212097_SS211647","PASS",0.2944,-0.4361,"Plate control","PASS","PFC",0.739129981275035,"1h","1h"
2808,"PFC_24",87,"OID30153","P0C7L1","SPINK8",0.6591,"Cardiometabolic_II","B13805","20212097_SS211647","PASS",0.2944,0.3925,"Plate control","PASS","PFC",1.31266611117376,"24h","24h"
2809,"PFC_01",1,"OID30154","Q7Z7H5","TMED4",1,"Cardiometabolic_II","B13805","20212097_SS211647","WARN",0.5026,-0.589,"Plate control","PASS","PFC",0.664803554090857,"Baseline","Baseline"
2810,"PFC_09",2,"OID30154","Q7Z7H5","TMED4",1,"Cardiometabolic_II","B13805","20212097_SS211647","PASS",0.5026,-1.4127,"Plate control","PASS","PFC",0.375608079119315,"Baseline","Baseline"
2811,"PFC_17",3,"OID30154","Q7Z7H5","TMED4",1,"Cardiometabolic_II","B13805","20212097_SS211647","PASS",0.5026,-0.4714,"Plate control","PASS","PFC",0.72126433876842,"1h","1h"
2812,"PFC_25",4,"OID30154","Q7Z7H5","TMED4",1,"Cardiometabolic_II","B13805","20212097_SS211647","PASS",0.5026,-0.3865,"Plate control","PASS","PFC",0.764983215984047,"24h","24h"
2813,"PFC_02",13,"OID30154","Q7Z7H5","TMED4",1,"Cardiometabolic_II","B13805","20212097_SS211647","PASS",0.5026,-0.6416,"Plate control","PASS","PFC",0.641001660842258,"Baseline","Baseline"
2814,"PFC_10",14,"OID30154","Q7Z7H5","TMED4",1,"Cardiometabolic_II","B13805","20212097_SS211647","PASS",0.5026,-0.1059,"Plate control","PASS","PFC",0.92922508099322,"1h","1h"
2815,"PFC_18",15,"OID30154","Q7Z7H5","TMED4",1,"Cardiometabolic_II","B13805","20212097_SS211647","PASS",0.5026,-0.6572,"Plate control","PASS","PFC",0.634107787564448,"1h","1h"
2816,"PFC_26",16,"OID30154","Q7Z7H5","TMED4",1,"Cardiometabolic_II","B13805","20212097_SS211647","PASS",0.5026,-1.3199,"Plate control","PASS","PFC",0.400562702723379,"24h","24h"
2817,"PFC_03",25,"OID30154","Q7Z7H5","TMED4",1,"Cardiometabolic_II","B13805","20212097_SS211647","PASS",0.5026,-1.2052,"Plate control","PASS","PFC",0.433709214544455,"Baseline","Baseline"
2818,"PFC_11",26,"OID30154","Q7Z7H5","TMED4",1,"Cardiometabolic_II","B13805","20212097_SS211647","PASS",0.5026,-0.8388,"Plate control","PASS","PFC",0.55910842899691,"1h","1h"
2819,"PFC_19",27,"OID30154","Q7Z7H5","TMED4",1,"Cardiometabolic_II","B13805","20212097_SS211647","PASS",0.5026,-1.1373,"Plate control","PASS","PFC",0.454609582665707,"24h","24h"
2820,"PFC_27",28,"OID30154","Q7Z7H5","TMED4",1,"Cardiometabolic_II","B13805","20212097_SS211647","PASS",0.5026,-1.1402,"Plate control","PASS","PFC",0.453696677584201,"24h","24h"
2821,"PFC_04",37,"OID30154","Q7Z7H5","TMED4",1,"Cardiometabolic_II","B13805","20212097_SS211647","PASS",0.5026,-0.9961,"Plate control","PASS","PFC",0.501353465572015,"Baseline","Baseline"
2822,"PFC_12",38,"OID30154","Q7Z7H5","TMED4",1,"Cardiometabolic_II","B13805","20212097_SS211647","PASS",0.5026,0.0707,"Plate control","PASS","PFC",1.05022613291799,"1h","1h"
2823,"PFC_20",39,"OID30154","Q7Z7H5","TMED4",1,"Cardiometabolic_II","B13805","20212097_SS211647","PASS",0.5026,-0.1471,"Plate control","PASS","PFC",0.903063912361901,"24h","24h"
2824,"PFC_05",49,"OID30154","Q7Z7H5","TMED4",1,"Cardiometabolic_II","B13805","20212097_SS211647","PASS",0.5026,0.1002,"Plate control","PASS","PFC",1.07192205218626,"Baseline","Baseline"
2825,"PFC_13",50,"OID30154","Q7Z7H5","TMED4",1,"Cardiometabolic_II","B13805","20212097_SS211647","PASS",0.5026,-0.414,"Plate control","PASS","PFC",0.750539548757186,"1h","1h"
2826,"PFC_21",51,"OID30154","Q7Z7H5","TMED4",1,"Cardiometabolic_II","B13805","20212097_SS211647","PASS",0.5026,-0.9739,"Plate control","PASS","PFC",0.509127888715339,"24h","24h"
2827,"PFC_06",61,"OID30154","Q7Z7H5","TMED4",1,"Cardiometabolic_II","B13805","20212097_SS211647","PASS",0.5026,-0.0535,"Plate control","PASS","PFC",0.963595793010082,"Baseline","Baseline"
2828,"PFC_14",62,"OID30154","Q7Z7H5","TMED4",1,"Cardiometabolic_II","B13805","20212097_SS211647","PASS",0.5026,-0.6301,"Plate control","PASS","PFC",0.646131627334955,"1h","1h"
2829,"PFC_22",63,"OID30154","Q7Z7H5","TMED4",1,"Cardiometabolic_II","B13805","20212097_SS211647","PASS",0.5026,-2.6913,"Plate control","PASS","PFC",0.15482388915273,"24h","24h"
2830,"PFC_07",73,"OID30154","Q7Z7H5","TMED4",1,"Cardiometabolic_II","B13805","20212097_SS211647","PASS",0.5026,-0.3987,"Plate control","PASS","PFC",0.758541490557053,"Baseline","Baseline"
2831,"PFC_15",74,"OID30154","Q7Z7H5","TMED4",1,"Cardiometabolic_II","B13805","20212097_SS211647","PASS",0.5026,-0.4413,"Plate control","PASS","PFC",0.736470682211274,"1h","1h"
2832,"PFC_23",75,"OID30154","Q7Z7H5","TMED4",1,"Cardiometabolic_II","B13805","20212097_SS211647","PASS",0.5026,-0.5799,"Plate control","PASS","PFC",0.669010148035741,"24h","24h"
2833,"PFC_08",85,"OID30154","Q7Z7H5","TMED4",1,"Cardiometabolic_II","B13805","20212097_SS211647","PASS",0.5026,-1.3614,"Plate control","PASS","PFC",0.389204420191287,"Baseline","Baseline"
2834,"PFC_16",86,"OID30154","Q7Z7H5","TMED4",1,"Cardiometabolic_II","B13805","20212097_SS211647","PASS",0.5026,-0.5282,"Plate control","PASS","PFC",0.693419349568856,"1h","1h"
2835,"PFC_24",87,"OID30154","Q7Z7H5","TMED4",1,"Cardiometabolic_II","B13805","20212097_SS211647","PASS",0.5026,-0.8313,"Plate control","PASS","PFC",0.562022580427211,"24h","24h"
2836,"PFC_01",1,"OID30155","Q9Y2L6","FRMD4B",0.1136,"Cardiometabolic_II","B13805","20212097_SS211647","WARN",0.6008,0.6584,"Plate control","PASS","PFC",1.57833122738152,"Baseline","Baseline"
2837,"PFC_09",2,"OID30155","Q9Y2L6","FRMD4B",0.1136,"Cardiometabolic_II","B13805","20212097_SS211647","PASS",0.6008,2.1645,"Plate control","PASS","PFC",4.48311031520489,"Baseline","Baseline"
2838,"PFC_17",3,"OID30155","Q9Y2L6","FRMD4B",0.1136,"Cardiometabolic_II","B13805","20212097_SS211647","PASS",0.6008,3.4439,"Plate control","PASS","PFC",10.8822124956141,"1h","1h"
2839,"PFC_25",4,"OID30155","Q9Y2L6","FRMD4B",0.1136,"Cardiometabolic_II","B13805","20212097_SS211647","PASS",0.6008,3.2976,"Plate control","PASS","PFC",9.83278433337135,"24h","24h"
2840,"PFC_02",13,"OID30155","Q9Y2L6","FRMD4B",0.1136,"Cardiometabolic_II","B13805","20212097_SS211647","PASS",0.6008,1.0527,"Plate control","PASS","PFC",2.0744084671645,"Baseline","Baseline"
2841,"PFC_10",14,"OID30155","Q9Y2L6","FRMD4B",0.1136,"Cardiometabolic_II","B13805","20212097_SS211647","PASS",0.6008,1.487,"Plate control","PASS","PFC",2.80305489816149,"1h","1h"
2842,"PFC_18",15,"OID30155","Q9Y2L6","FRMD4B",0.1136,"Cardiometabolic_II","B13805","20212097_SS211647","PASS",0.6008,2.1586,"Plate control","PASS","PFC",4.46481376707402,"1h","1h"
2843,"PFC_26",16,"OID30155","Q9Y2L6","FRMD4B",0.1136,"Cardiometabolic_II","B13805","20212097_SS211647","PASS",0.6008,2.8982,"Plate control","PASS","PFC",7.45495683926923,"24h","24h"
2844,"PFC_03",25,"OID30155","Q9Y2L6","FRMD4B",0.1136,"Cardiometabolic_II","B13805","20212097_SS211647","PASS",0.6008,1.4616,"Plate control","PASS","PFC",2.75413637815168,"Baseline","Baseline"
2845,"PFC_11",26,"OID30155","Q9Y2L6","FRMD4B",0.1136,"Cardiometabolic_II","B13805","20212097_SS211647","PASS",0.6008,3.0328,"Plate control","PASS","PFC",8.18396514075898,"1h","1h"
2846,"PFC_19",27,"OID30155","Q9Y2L6","FRMD4B",0.1136,"Cardiometabolic_II","B13805","20212097_SS211647","PASS",0.6008,2.5096,"Plate control","PASS","PFC",5.69462167910371,"24h","24h"
2847,"PFC_27",28,"OID30155","Q9Y2L6","FRMD4B",0.1136,"Cardiometabolic_II","B13805","20212097_SS211647","PASS",0.6008,3.7724,"Plate control","PASS","PFC",13.6648716119744,"24h","24h"
2848,"PFC_04",37,"OID30155","Q9Y2L6","FRMD4B",0.1136,"Cardiometabolic_II","B13805","20212097_SS211647","PASS",0.6008,3.2684,"Plate control","PASS","PFC",9.63577028232425,"Baseline","Baseline"
2849,"PFC_12",38,"OID30155","Q9Y2L6","FRMD4B",0.1136,"Cardiometabolic_II","B13805","20212097_SS211647","PASS",0.6008,1.4559,"Plate control","PASS","PFC",2.74327642115928,"1h","1h"
2850,"PFC_20",39,"OID30155","Q9Y2L6","FRMD4B",0.1136,"Cardiometabolic_II","B13805","20212097_SS211647","PASS",0.6008,3.7364,"Plate control","PASS","PFC",13.3281071578132,"24h","24h"
2851,"PFC_05",49,"OID30155","Q9Y2L6","FRMD4B",0.1136,"Cardiometabolic_II","B13805","20212097_SS211647","PASS",0.6008,1.7997,"Plate control","PASS","PFC",3.48147822486365,"Baseline","Baseline"
2852,"PFC_13",50,"OID30155","Q9Y2L6","FRMD4B",0.1136,"Cardiometabolic_II","B13805","20212097_SS211647","PASS",0.6008,3.1563,"Plate control","PASS","PFC",8.91540291973278,"1h","1h"
2853,"PFC_21",51,"OID30155","Q9Y2L6","FRMD4B",0.1136,"Cardiometabolic_II","B13805","20212097_SS211647","PASS",0.6008,1.9743,"Plate control","PASS","PFC",3.92937538674605,"24h","24h"
2854,"PFC_06",61,"OID30155","Q9Y2L6","FRMD4B",0.1136,"Cardiometabolic_II","B13805","20212097_SS211647","PASS",0.6008,2.969,"Plate control","PASS","PFC",7.82993320317949,"Baseline","Baseline"
2855,"PFC_14",62,"OID30155","Q9Y2L6","FRMD4B",0.1136,"Cardiometabolic_II","B13805","20212097_SS211647","PASS",0.6008,2.4119,"Plate control","PASS","PFC",5.32174727612619,"1h","1h"
2856,"PFC_22",63,"OID30155","Q9Y2L6","FRMD4B",0.1136,"Cardiometabolic_II","B13805","20212097_SS211647","PASS",0.6008,2.4022,"Plate control","PASS","PFC",5.28608637901505,"24h","24h"
2857,"PFC_07",73,"OID30155","Q9Y2L6","FRMD4B",0.1136,"Cardiometabolic_II","B13805","20212097_SS211647","PASS",0.6008,2.7782,"Plate control","PASS","PFC",6.859959219437,"Baseline","Baseline"
2858,"PFC_15",74,"OID30155","Q9Y2L6","FRMD4B",0.1136,"Cardiometabolic_II","B13805","20212097_SS211647","PASS",0.6008,1.8939,"Plate control","PASS","PFC",3.71638508789115,"1h","1h"
2859,"PFC_23",75,"OID30155","Q9Y2L6","FRMD4B",0.1136,"Cardiometabolic_II","B13805","20212097_SS211647","PASS",0.6008,2.9581,"Plate control","PASS","PFC",7.7709985902418,"24h","24h"
2860,"PFC_08",85,"OID30155","Q9Y2L6","FRMD4B",0.1136,"Cardiometabolic_II","B13805","20212097_SS211647","PASS",0.6008,2.2385,"Plate control","PASS","PFC",4.71906158778391,"Baseline","Baseline"
2861,"PFC_16",86,"OID30155","Q9Y2L6","FRMD4B",0.1136,"Cardiometabolic_II","B13805","20212097_SS211647","PASS",0.6008,3.8582,"Plate control","PASS","PFC",14.5022013049205,"1h","1h"
2862,"PFC_24",87,"OID30155","Q9Y2L6","FRMD4B",0.1136,"Cardiometabolic_II","B13805","20212097_SS211647","PASS",0.6008,3.9292,"Plate control","PASS","PFC",15.2337582368994,"24h","24h"
2863,"PFC_01",1,"OID30156","P55010","EIF5",0.2045,"Cardiometabolic_II","B13805","20212097_SS211647","WARN",0.769,0.7673,"Plate control","PASS","PFC",1.70208136008472,"Baseline","Baseline"
2864,"PFC_09",2,"OID30156","P55010","EIF5",0.2045,"Cardiometabolic_II","B13805","20212097_SS211647","PASS",0.769,1.0982,"Plate control","PASS","PFC",2.14087416446062,"Baseline","Baseline"
2865,"PFC_17",3,"OID30156","P55010","EIF5",0.2045,"Cardiometabolic_II","B13805","20212097_SS211647","PASS",0.769,2.6533,"Plate control","PASS","PFC",6.2910463874371,"1h","1h"
2866,"PFC_25",4,"OID30156","P55010","EIF5",0.2045,"Cardiometabolic_II","B13805","20212097_SS211647","PASS",0.769,2.1091,"Plate control","PASS","PFC",4.31422075540944,"24h","24h"
2867,"PFC_02",13,"OID30156","P55010","EIF5",0.2045,"Cardiometabolic_II","B13805","20212097_SS211647","PASS",0.769,1.4568,"Plate control","PASS","PFC",2.74498829995169,"Baseline","Baseline"
2868,"PFC_10",14,"OID30156","P55010","EIF5",0.2045,"Cardiometabolic_II","B13805","20212097_SS211647","PASS",0.769,1.1881,"Plate control","PASS","PFC",2.27852468426681,"1h","1h"
2869,"PFC_18",15,"OID30156","P55010","EIF5",0.2045,"Cardiometabolic_II","B13805","20212097_SS211647","PASS",0.769,1.5469,"Plate control","PASS","PFC",2.92188622035249,"1h","1h"
2870,"PFC_26",16,"OID30156","P55010","EIF5",0.2045,"Cardiometabolic_II","B13805","20212097_SS211647","PASS",0.769,2.0459,"Plate control","PASS","PFC",4.12930791019948,"24h","24h"
2871,"PFC_03",25,"OID30156","P55010","EIF5",0.2045,"Cardiometabolic_II","B13805","20212097_SS211647","PASS",0.769,0.1838,"Plate control","PASS","PFC",1.13587178860597,"Baseline","Baseline"
2872,"PFC_11",26,"OID30156","P55010","EIF5",0.2045,"Cardiometabolic_II","B13805","20212097_SS211647","PASS",0.769,1.5923,"Plate control","PASS","PFC",3.01529676726524,"1h","1h"
2873,"PFC_19",27,"OID30156","P55010","EIF5",0.2045,"Cardiometabolic_II","B13805","20212097_SS211647","PASS",0.769,1.604,"Plate control","PASS","PFC",3.03984971280303,"24h","24h"
2874,"PFC_27",28,"OID30156","P55010","EIF5",0.2045,"Cardiometabolic_II","B13805","20212097_SS211647","PASS",0.769,2.4112,"Plate control","PASS","PFC",5.31916577456895,"24h","24h"
2875,"PFC_04",37,"OID30156","P55010","EIF5",0.2045,"Cardiometabolic_II","B13805","20212097_SS211647","PASS",0.769,1.4196,"Plate control","PASS","PFC",2.67511330784262,"Baseline","Baseline"
2876,"PFC_12",38,"OID30156","P55010","EIF5",0.2045,"Cardiometabolic_II","B13805","20212097_SS211647","PASS",0.769,0.7783,"Plate control","PASS","PFC",1.715108683014,"1h","1h"
2877,"PFC_20",39,"OID30156","P55010","EIF5",0.2045,"Cardiometabolic_II","B13805","20212097_SS211647","PASS",0.769,2.175,"Plate control","PASS","PFC",4.51585761922452,"24h","24h"
2878,"PFC_05",49,"OID30156","P55010","EIF5",0.2045,"Cardiometabolic_II","B13805","20212097_SS211647","PASS",0.769,0.6606,"Plate control","PASS","PFC",1.5807398982831,"Baseline","Baseline"
2879,"PFC_13",50,"OID30156","P55010","EIF5",0.2045,"Cardiometabolic_II","B13805","20212097_SS211647","PASS",0.769,1.8453,"Plate control","PASS","PFC",3.59327661471943,"1h","1h"
2880,"PFC_21",51,"OID30156","P55010","EIF5",0.2045,"Cardiometabolic_II","B13805","20212097_SS211647","PASS",0.769,0.569,"Plate control","PASS","PFC",1.48349493384112,"24h","24h"
2881,"PFC_06",61,"OID30156","P55010","EIF5",0.2045,"Cardiometabolic_II","B13805","20212097_SS211647","PASS",0.769,1.3686,"Plate control","PASS","PFC",2.58219866385609,"Baseline","Baseline"
2882,"PFC_14",62,"OID30156","P55010","EIF5",0.2045,"Cardiometabolic_II","B13805","20212097_SS211647","PASS",0.769,1.8344,"Plate control","PASS","PFC",3.56623061560663,"1h","1h"
2883,"PFC_22",63,"OID30156","P55010","EIF5",0.2045,"Cardiometabolic_II","B13805","20212097_SS211647","PASS",0.769,1.2918,"Plate control","PASS","PFC",2.44833335020139,"24h","24h"
2884,"PFC_07",73,"OID30156","P55010","EIF5",0.2045,"Cardiometabolic_II","B13805","20212097_SS211647","PASS",0.769,1.904,"Plate control","PASS","PFC",3.7424939913256,"Baseline","Baseline"
2885,"PFC_15",74,"OID30156","P55010","EIF5",0.2045,"Cardiometabolic_II","B13805","20212097_SS211647","PASS",0.769,0.9977,"Plate control","PASS","PFC",1.99681406321577,"1h","1h"
2886,"PFC_23",75,"OID30156","P55010","EIF5",0.2045,"Cardiometabolic_II","B13805","20212097_SS211647","PASS",0.769,2.337,"Plate control","PASS","PFC",5.0525090473832,"24h","24h"
2887,"PFC_08",85,"OID30156","P55010","EIF5",0.2045,"Cardiometabolic_II","B13805","20212097_SS211647","PASS",0.769,1.4843,"Plate control","PASS","PFC",2.79781389403501,"Baseline","Baseline"
2888,"PFC_16",86,"OID30156","P55010","EIF5",0.2045,"Cardiometabolic_II","B13805","20212097_SS211647","PASS",0.769,1.8409,"Plate control","PASS","PFC",3.58233436326111,"1h","1h"
2889,"PFC_24",87,"OID30156","P55010","EIF5",0.2045,"Cardiometabolic_II","B13805","20212097_SS211647","PASS",0.769,1.9866,"Plate control","PASS","PFC",3.96301931845184,"24h","24h"
2890,"PFC_01",1,"OID30157","Q01581","HMGCS1",0.8636,"Cardiometabolic_II","B13805","20212097_SS211647","WARN",0.335,0.0363,"Plate control","PASS","PFC",1.02548045838538,"Baseline","Baseline"
2891,"PFC_09",2,"OID30157","Q01581","HMGCS1",0.8636,"Cardiometabolic_II","B13805","20212097_SS211647","PASS",0.335,-0.1603,"Plate control","PASS","PFC",0.894838975046085,"Baseline","Baseline"
2892,"PFC_17",3,"OID30157","Q01581","HMGCS1",0.8636,"Cardiometabolic_II","B13805","20212097_SS211647","PASS",0.335,0.0402,"Plate control","PASS","PFC",1.02825636337578,"1h","1h"
2893,"PFC_25",4,"OID30157","Q01581","HMGCS1",0.8636,"Cardiometabolic_II","B13805","20212097_SS211647","PASS",0.335,0.483,"Plate control","PASS","PFC",1.39764697185107,"24h","24h"
2894,"PFC_02",13,"OID30157","Q01581","HMGCS1",0.8636,"Cardiometabolic_II","B13805","20212097_SS211647","PASS",0.335,-0.7492,"Plate control","PASS","PFC",0.59493336715911,"Baseline","Baseline"
2895,"PFC_10",14,"OID30157","Q01581","HMGCS1",0.8636,"Cardiometabolic_II","B13805","20212097_SS211647","PASS",0.335,-0.1859,"Plate control","PASS","PFC",0.879100493402846,"1h","1h"
2896,"PFC_18",15,"OID30157","Q01581","HMGCS1",0.8636,"Cardiometabolic_II","B13805","20212097_SS211647","PASS",0.335,0.0048,"Plate control","PASS","PFC",1.00333264742883,"1h","1h"
2897,"PFC_26",16,"OID30157","Q01581","HMGCS1",0.8636,"Cardiometabolic_II","B13805","20212097_SS211647","PASS",0.335,-0.0394,"Plate control","PASS","PFC",0.973059547361322,"24h","24h"
2898,"PFC_03",25,"OID30157","Q01581","HMGCS1",0.8636,"Cardiometabolic_II","B13805","20212097_SS211647","PASS",0.335,-0.4373,"Plate control","PASS","PFC",0.738515445853457,"Baseline","Baseline"
2899,"PFC_11",26,"OID30157","Q01581","HMGCS1",0.8636,"Cardiometabolic_II","B13805","20212097_SS211647","PASS",0.335,-0.1336,"Plate control","PASS","PFC",0.9115539820327,"1h","1h"
2900,"PFC_19",27,"OID30157","Q01581","HMGCS1",0.8636,"Cardiometabolic_II","B13805","20212097_SS211647","PASS",0.335,0.3293,"Plate control","PASS","PFC",1.25640361576865,"24h","24h"
2901,"PFC_27",28,"OID30157","Q01581","HMGCS1",0.8636,"Cardiometabolic_II","B13805","20212097_SS211647","PASS",0.335,-0.0773,"Plate control","PASS","PFC",0.947829848958541,"24h","24h"
2902,"PFC_04",37,"OID30157","Q01581","HMGCS1",0.8636,"Cardiometabolic_II","B13805","20212097_SS211647","PASS",0.335,-0.1552,"Plate control","PASS","PFC",0.898007873929337,"Baseline","Baseline"
2903,"PFC_12",38,"OID30157","Q01581","HMGCS1",0.8636,"Cardiometabolic_II","B13805","20212097_SS211647","PASS",0.335,-0.3534,"Plate control","PASS","PFC",0.782737245322399,"1h","1h"
2904,"PFC_20",39,"OID30157","Q01581","HMGCS1",0.8636,"Cardiometabolic_II","B13805","20212097_SS211647","PASS",0.335,0.2107,"Plate control","PASS","PFC",1.15724954868964,"24h","24h"
2905,"PFC_05",49,"OID30157","Q01581","HMGCS1",0.8636,"Cardiometabolic_II","B13805","20212097_SS211647","PASS",0.335,0.3193,"Plate control","PASS","PFC",1.24772500205981,"Baseline","Baseline"
2906,"PFC_13",50,"OID30157","Q01581","HMGCS1",0.8636,"Cardiometabolic_II","B13805","20212097_SS211647","PASS",0.335,0.2363,"Plate control","PASS","PFC",1.17796771562889,"1h","1h"
2907,"PFC_21",51,"OID30157","Q01581","HMGCS1",0.8636,"Cardiometabolic_II","B13805","20212097_SS211647","PASS",0.335,-0.1889,"Plate control","PASS","PFC",0.877274354649975,"24h","24h"
2908,"PFC_06",61,"OID30157","Q01581","HMGCS1",0.8636,"Cardiometabolic_II","B13805","20212097_SS211647","PASS",0.335,-0.0193,"Plate control","PASS","PFC",0.986711343695511,"Baseline","Baseline"
2909,"PFC_14",62,"OID30157","Q01581","HMGCS1",0.8636,"Cardiometabolic_II","B13805","20212097_SS211647","PASS",0.335,-0.3356,"Plate control","PASS","PFC",0.792454495508525,"1h","1h"
2910,"PFC_22",63,"OID30157","Q01581","HMGCS1",0.8636,"Cardiometabolic_II","B13805","20212097_SS211647","PASS",0.335,-0.1324,"Plate control","PASS","PFC",0.912312506737613,"24h","24h"
2911,"PFC_07",73,"OID30157","Q01581","HMGCS1",0.8636,"Cardiometabolic_II","B13805","20212097_SS211647","PASS",0.335,-0.1187,"Plate control","PASS","PFC",0.921017197435768,"Baseline","Baseline"
2912,"PFC_15",74,"OID30157","Q01581","HMGCS1",0.8636,"Cardiometabolic_II","B13805","20212097_SS211647","PASS",0.335,-0.1212,"Plate control","PASS","PFC",0.919422578282887,"1h","1h"

Supplemental table 1_Olink proximity extension assay (PEA) data

,"SampleID","Index","OlinkID","UniProt","Assay","MissingFreq","Panel","Panel_Lot_Nr","PlateID","QC_Warning","LOD","NPX","Normalization","Assay_Warning","condition","Value","group","alt_group"
2913,"PFC_23",75,"OID30157","Q01581","HMGCS1",0.8636,"Cardiometabolic_II","B13805","20212097_SS211647","PASS",0.335,0.3534,"Plate control","PASS","PFC",1.27756792713769,"24h","24h"
2914,"PFC_08",85,"OID30157","Q01581","HMGCS1",0.8636,"Cardiometabolic_II","B13805","20212097_SS211647","PASS",0.335,-0.2332,"Plate control","PASS","PFC",0.850745782923134,"Baseline","Baseline"
2915,"PFC_16",86,"OID30157","Q01581","HMGCS1",0.8636,"Cardiometabolic_II","B13805","20212097_SS211647","PASS",0.335,0.1774,"Plate control","PASS","PFC",1.13084406021201,"1h","1h"
2916,"PFC_24",87,"OID30157","Q01581","HMGCS1",0.8636,"Cardiometabolic_II","B13805","20212097_SS211647","PASS",0.335,0.2474,"Plate control","PASS","PFC",1.18706587658059,"24h","24h"
2917,"PFC_01",1,"OID30158","Q12986","NFX1",0.0682,"Cardiometabolic_II","B13805","20212097_SS211647","WARN",0.4441,1.5705,"Plate control","PASS","PFC",2.97007631291711,"Baseline","Baseline"
2918,"PFC_09",2,"OID30158","Q12986","NFX1",0.0682,"Cardiometabolic_II","B13805","20212097_SS211647","PASS",0.4441,2.0513,"Plate control","PASS","PFC",4.14479285007768,"Baseline","Baseline"
2919,"PFC_17",3,"OID30158","Q12986","NFX1",0.0682,"Cardiometabolic_II","B13805","20212097_SS211647","PASS",0.4441,2.5162,"Plate control","PASS","PFC",5.72073295246178,"1h","1h"
2920,"PFC_25",4,"OID30158","Q12986","NFX1",0.0682,"Cardiometabolic_II","B13805","20212097_SS211647","PASS",0.4441,2.3404,"Plate control","PASS","PFC",5.06443033948582,"24h","24h"
2921,"PFC_02",13,"OID30158","Q12986","NFX1",0.0682,"Cardiometabolic_II","B13805","20212097_SS211647","PASS",0.4441,2.4035,"Plate control","PASS","PFC",5.29085177235068,"Baseline","Baseline"
2922,"PFC_10",14,"OID30158","Q12986","NFX1",0.0682,"Cardiometabolic_II","B13805","20212097_SS211647","PASS",0.4441,1.8247,"Plate control","PASS","PFC",3.54233339229648,"1h","1h"
2923,"PFC_18",15,"OID30158","Q12986","NFX1",0.0682,"Cardiometabolic_II","B13805","20212097_SS211647","PASS",0.4441,2.3561,"Plate control","PASS","PFC",5.11984452365794,"1h","1h"
2924,"PFC_26",16,"OID30158","Q12986","NFX1",0.0682,"Cardiometabolic_II","B13805","20212097_SS211647","PASS",0.4441,2.1891,"Plate control","PASS","PFC",4.56020916943212,"24h","24h"
2925,"PFC_03",25,"OID30158","Q12986","NFX1",0.0682,"Cardiometabolic_II","B13805","20212097_SS211647","PASS",0.4441,1.107,"Plate control","PASS","PFC",2.1539727523861,"Baseline","Baseline"
2926,"PFC_11",26,"OID30158","Q12986","NFX1",0.0682,"Cardiometabolic_II","B13805","20212097_SS211647","PASS",0.4441,3.1698,"Plate control","PASS","PFC",8.99922023420591,"1h","1h"
2927,"PFC_19",27,"OID30158","Q12986","NFX1",0.0682,"Cardiometabolic_II","B13805","20212097_SS211647","PASS",0.4441,2.8885,"Plate control","PASS","PFC",7.4050013575413,"24h","24h"
2928,"PFC_27",28,"OID30158","Q12986","NFX1",0.0682,"Cardiometabolic_II","B13805","20212097_SS211647","PASS",0.4441,3.7527,"Plate control","PASS","PFC",13.4795459878082,"24h","24h"
2929,"PFC_04",37,"OID30158","Q12986","NFX1",0.0682,"Cardiometabolic_II","B13805","20212097_SS211647","PASS",0.4441,2.7859,"Plate control","PASS","PFC",6.89667030281519,"Baseline","Baseline"
2930,"PFC_12",38,"OID30158","Q12986","NFX1",0.0682,"Cardiometabolic_II","B13805","20212097_SS211647","PASS",0.4441,1.0982,"Plate control","PASS","PFC",2.14087416446062,"1h","1h"
2931,"PFC_20",39,"OID30158","Q12986","NFX1",0.0682,"Cardiometabolic_II","B13805","20212097_SS211647","PASS",0.4441,3.8627,"Plate control","PASS","PFC",14.5475066453026,"24h","24h"
2932,"PFC_05",49,"OID30158","Q12986","NFX1",0.0682,"Cardiometabolic_II","B13805","20212097_SS211647","PASS",0.4441,1.6062,"Plate control","PASS","PFC",3.04448878818935,"Baseline","Baseline"
2933,"PFC_13",50,"OID30158","Q12986","NFX1",0.0682,"Cardiometabolic_II","B13805","20212097_SS211647","PASS",0.4441,2.6796,"Plate control","PASS","PFC",6.40678243716847,"1h","1h"
2934,"PFC_21",51,"OID30158","Q12986","NFX1",0.0682,"Cardiometabolic_II","B13805","20212097_SS211647","PASS",0.4441,1.3687,"Plate control","PASS","PFC",2.58237765443171,"24h","24h"
2935,"PFC_06",61,"OID30158","Q12986","NFX1",0.0682,"Cardiometabolic_II","B13805","20212097_SS211647","PASS",0.4441,3.001,"Plate control","PASS","PFC",8.00554709970064,"Baseline","Baseline"
2936,"PFC_14",62,"OID30158","Q12986","NFX1",0.0682,"Cardiometabolic_II","B13805","20212097_SS211647","PASS",0.4441,2.1878,"Plate control","PASS","PFC",4.55610185527551,"1h","1h"
2937,"PFC_22",63,"OID30158","Q12986","NFX1",0.0682,"Cardiometabolic_II","B13805","20212097_SS211647","PASS",0.4441,2.2446,"Plate control","PASS","PFC",4.73905695601093,"24h","24h"
2938,"PFC_07",73,"OID30158","Q12986","NFX1",0.0682,"Cardiometabolic_II","B13805","20212097_SS211647","PASS",0.4441,2.5995,"Plate control","PASS","PFC",6.06076540078562,"Baseline","Baseline"
2939,"PFC_15",74,"OID30158","Q12986","NFX1",0.0682,"Cardiometabolic_II","B13805","20212097_SS211647","PASS",0.4441,1.864,"Plate control","PASS","PFC",3.6401552963236,"1h","1h"
2940,"PFC_23",75,"OID30158","Q12986","NFX1",0.0682,"Cardiometabolic_II","B13805","20212097_SS211647","PASS",0.4441,2.5697,"Plate control","PASS","PFC",5.93685961889824,"24h","24h"
2941,"PFC_08",85,"OID30158","Q12986","NFX1",0.0682,"Cardiometabolic_II","B13805","20212097_SS211647","PASS",0.4441,0.8065,"Plate control","PASS","PFC",1.74896328027936,"Baseline","Baseline"
2942,"PFC_16",86,"OID30158","Q12986","NFX1",0.0682,"Cardiometabolic_II","B13805","20212097_SS211647","PASS",0.4441,2.8624,"Plate control","PASS","PFC",7.27224094541945,"1h","1h"
2943,"PFC_24",87,"OID30158","Q12986","NFX1",0.0682,"Cardiometabolic_II","B13805","20212097_SS211647","PASS",0.4441,3.5867,"Plate control","PASS","PFC",12.0144608188594,"24h","24h"
2944,"PFC_01",1,"OID30159","P22033","MMUT",0.5,"Cardiometabolic_II","B13805","20212097_SS211647","WARN",0.07,-0.9938,"Plate control","PASS","PFC",0.502153380034403,"Baseline","Baseline"
2945,"PFC_09",2,"OID30159","P22033","MMUT",0.5,"Cardiometabolic_II","B13805","20212097_SS211647","PASS",0.07,-0.3716,"Plate control","PASS","PFC",0.772924820206372,"Baseline","Baseline"
2946,"PFC_17",3,"OID30159","P22033","MMUT",0.5,"Cardiometabolic_II","B13805","20212097_SS211647","PASS",0.07,0.1453,"Plate control","PASS","PFC",1.10596060745369,"1h","1h"
2947,"PFC_25",4,"OID30159","P22033","MMUT",0.5,"Cardiometabolic_II","B13805","20212097_SS211647","PASS",0.07,0.8135,"Plate control","PASS","PFC",1.75746992360243,"24h","24h"
2948,"PFC_02",13,"OID30159","P22033","MMUT",0.5,"Cardiometabolic_II","B13805","20212097_SS211647","PASS",0.07,-0.2111,"Plate control","PASS","PFC",0.86387830584376,"Baseline","Baseline"
2949,"PFC_10",14,"OID30159","P22033","MMUT",0.5,"Cardiometabolic_II","B13805","20212097_SS211647","PASS",0.07,-0.3674,"Plate control","PASS","PFC",0.775178251501529,"1h","1h"
2950,"PFC_18",15,"OID30159","P22033","MMUT",0.5,"Cardiometabolic_II","B13805","20212097_SS211647","PASS",0.07,0.2595,"Plate control","PASS","PFC",1.19706376203321,"1h","1h"
2951,"PFC_26",16,"OID30159","P22033","MMUT",0.5,"Cardiometabolic_II","B13805","20212097_SS211647","PASS",0.07,0.1462,"Plate control","PASS","PFC",1.10665075682928,"24h","24h"
2952,"PFC_03",25,"OID30159","P22033","MMUT",0.5,"Cardiometabolic_II","B13805","20212097_SS211647","PASS",0.07,-0.2347,"Plate control","PASS","PFC",0.849861704538896,"Baseline","Baseline"
2953,"PFC_11",26,"OID30159","P22033","MMUT",0.5,"Cardiometabolic_II","B13805","20212097_SS211647","PASS",0.07,0.2823,"Plate control","PASS","PFC",1.21613214446181,"1h","1h"
2954,"PFC_19",27,"OID30159","P22033","MMUT",0.5,"Cardiometabolic_II","B13805","20212097_SS211647","PASS",0.07,0.3891,"Plate control","PASS","PFC",1.30957619284227,"24h","24h"
2955,"PFC_27",28,"OID30159","P22033","MMUT",0.5,"Cardiometabolic_II","B13805","20212097_SS211647","PASS",0.07,0.3428,"Plate control","PASS","PFC",1.2682155751289,"24h","24h"
2956,"PFC_04",37,"OID30159","P22033","MMUT",0.5,"Cardiometabolic_II","B13805","20212097_SS211647","PASS",0.07,0.3248,"Plate control","PASS","PFC",1.25249079449574,"Baseline","Baseline"
2957,"PFC_12",38,"OID30159","P22033","MMUT",0.5,"Cardiometabolic_II","B13805","20212097_SS211647","PASS",0.07,-0.6836,"Plate control","PASS","PFC",0.622609717830721,"1h","1h"
2958,"PFC_20",39,"OID30159","P22033","MMUT",0.5,"Cardiometabolic_II","B13805","20212097_SS211647","PASS",0.07,-0.3901,"Plate control","PASS","PFC",0.763076710200102,"24h","24h"
2959,"PFC_05",49,"OID30159","P22033","MMUT",0.5,"Cardiometabolic_II","B13805","20212097_SS211647","PASS",0.07,0.4469,"Plate control","PASS","PFC",1.36310812055283,"Baseline","Baseline"
2960,"PFC_13",50,"OID30159","P22033","MMUT",0.5,"Cardiometabolic_II","B13805","20212097_SS211647","PASS",0.07,0.5625,"Plate control","PASS","PFC",1.4768261459395,"1h","1h"
2961,"PFC_21",51,"OID30159","P22033","MMUT",0.5,"Cardiometabolic_II","B13805","20212097_SS211647","PASS",0.07,-0.7776,"Plate control","PASS","PFC",0.583336397666792,"24h","24h"
2962,"PFC_06",61,"OID30159","P22033","MMUT",0.5,"Cardiometabolic_II","B13805","20212097_SS211647","PASS",0.07,-0.4027,"Plate control","PASS","PFC",0.756441279830674,"Baseline","Baseline"
2963,"PFC_14",62,"OID30159","P22033","MMUT",0.5,"Cardiometabolic_II","B13805","20212097_SS211647","PASS",0.07,0.6625,"Plate control","PASS","PFC",1.58282307199771,"1h","1h"
2964,"PFC_22",63,"OID30159","P22033","MMUT",0.5,"Cardiometabolic_II","B13805","20212097_SS211647","PASS",0.07,-0.9801,"Plate control","PASS","PFC",0.506944599955155,"24h","24h"
2965,"PFC_07",73,"OID30159","P22033","MMUT",0.5,"Cardiometabolic_II","B13805","20212097_SS211647","PASS",0.07,0.4444,"Plate control","PASS","PFC",1.36074807958667,"Baseline","Baseline"
2966,"PFC_15",74,"OID30159","P22033","MMUT",0.5,"Cardiometabolic_II","B13805","20212097_SS211647","PASS",0.07,-0.2811,"Plate control","PASS","PFC",0.822963299832588,"1h","1h"
2967,"PFC_23",75,"OID30159","P22033","MMUT",0.5,"Cardiometabolic_II","B13805","20212097_SS211647","PASS",0.07,-0.7199,"Plate control","PASS","PFC",0.607139524444005,"24h","24h"
2968,"PFC_08",85,"OID30159","P22033","MMUT",0.5,"Cardiometabolic_II","B13805","20212097_SS211647","PASS",0.07,-0.0178,"Plate control","PASS","PFC",0.987737781486253,"Baseline","Baseline"
2969,"PFC_16",86,"OID30159","P22033","MMUT",0.5,"Cardiometabolic_II","B13805","20212097_SS211647","PASS",0.07,0.9222,"Plate control","PASS","PFC",1.8950028261634,"1h","1h"
2970,"PFC_24",87,"OID30159","P22033","MMUT",0.5,"Cardiometabolic_II","B13805","20212097_SS211647","PASS",0.07,-0.3458,"Plate control","PASS","PFC",0.786871521348117,"24h","24h"
2971,"PFC_01",1,"OID30160","P19838","NFKB1",0,"Cardiometabolic_II","B13805","20212097_SS211647","WARN",-1.6949,1.5598,"Plate control","PASS","PFC",2.94812970869304,"Baseline","Baseline"
2972,"PFC_09",2,"OID30160","P19838","NFKB1",0,"Cardiometabolic_II","B13805","20212097_SS211647","PASS",-1.6949,1.9226,"Plate control","PASS","PFC",3.79105661070673,"Baseline","Baseline"
2973,"PFC_17",3,"OID30160","P19838","NFKB1",0,"Cardiometabolic_II","B13805","20212097_SS211647","PASS",-1.6949,2.7996,"Plate control","PASS","PFC",6.96247383109064,"1h","1h"
2974,"PFC_25",4,"OID30160","P19838","NFKB1",0,"Cardiometabolic_II","B13805","20212097_SS211647","PASS",-1.6949,2.7525,"Plate control","PASS","PFC",6.73883872773434,"24h","24h"
2975,"PFC_02",13,"OID30160","P19838","NFKB1",0,"Cardiometabolic_II","B13805","20212097_SS211647","PASS",-1.6949,1.8789,"Plate control","PASS","PFC",3.67794524003369,"Baseline","Baseline"
2976,"PFC_10",14,"OID30160","P19838","NFKB1",0,"Cardiometabolic_II","B13805","20212097_SS211647","PASS",-1.6949,1.8148,"Plate control","PASS","PFC",3.5181085564771,"1h","1h"
2977,"PFC_18",15,"OID30160","P19838","NFKB1",0,"Cardiometabolic_II","B13805","20212097_SS211647","PASS",-1.6949,2.4766,"Plate control","PASS","PFC",5.56584217425037,"1h","1h"
2978,"PFC_26",16,"OID30160","P19838","NFKB1",0,"Cardiometabolic_II","B13805","20212097_SS211647","PASS",-1.6949,2.4338,"Plate control","PASS","PFC",5.40314725096435,"24h","24h"
2979,"PFC_03",25,"OID30160","P19838","NFKB1",0,"Cardiometabolic_II","B13805","20212097_SS211647","PASS",-1.6949,0.385,"Plate control","PASS","PFC",1.30585978708892,"Baseline","Baseline"
2980,"PFC_11",26,"OID30160","P19838","NFKB1",0,"Cardiometabolic_II","B13805","20212097_SS211647","PASS",-1.6949,2.8083,"Plate control","PASS","PFC",7.00458704905195,"1h","1h"
2981,"PFC_19",27,"OID30160","P19838","NFKB1",0,"Cardiometabolic_II","B13805","20212097_SS211647","PASS",-1.6949,2.4197,"Plate control","PASS","PFC",5.35059747798759,"24h","24h"
2982,"PFC_27",28,"OID30160","P19838","NFKB1",0,"Cardiometabolic_II","B13805","20212097_SS211647","PASS",-1.6949,3.6015,"Plate control","PASS","PFC",12.1383464644058,"24h","24h"
2983,"PFC_04",37,"OID30160","P19838","NFKB1",0,"Cardiometabolic_II","B13805","20212097_SS211647","PASS",-1.6949,2.8686,"Plate control","PASS","PFC",7.30356074233393,"Baseline","Baseline"
2984,"PFC_12",38,"OID30160","P19838","NFKB1",0,"Cardiometabolic_II","B13805","20212097_SS211647","PASS",-1.6949,0.9472,"Plate control","PASS","PFC",1.92812689209846,"1h","1h"
2985,"PFC_20",39,"OID30160","P19838","NFKB1",0,"Cardiometabolic_II","B13805","20212097_SS211647","PASS",-1.6949,3.5961,"Plate control","PASS","PFC",12.0929976202004,"24h","24h"
2986,"PFC_05",49,"OID30160","P19838","NFKB1",0,"Cardiometabolic_II","B13805","20212097_SS211647","PASS",-1.6949,1.768,"Plate control","PASS","PFC",3.40581483099578,"Baseline","Baseline"
2987,"PFC_13",50,"OID30160","P19838","NFKB1",0,"Cardiometabolic_II","B13805","20212097_SS211647","PASS",-1.6949,2.9481,"Plate control","PASS","PFC",7.71732038202091,"1h","1h"
2988,"PFC_21",51,"OID30160","P19838","NFKB1",0,"Cardiometabolic_II","B13805","20212097_SS211647","PASS",-1.6949,1.4462,"Plate control","PASS","PFC",2.72489379358859,"24h","24h"
2989,"PFC_06",61,"OID30160","P19838","NFKB1",0,"Cardiometabolic_II","B13805","20212097_SS211647","PASS",-1.6949,2.5703,"Plate control","PASS","PFC",5.93932920290193,"Baseline","Baseline"
2990,"PFC_14",62,"OID30160","P19838","NFKB1",0,"Cardiometabolic_II","B13805","20212097_SS211647","PASS",-1.6949,2.7578,"Plate control","PASS","PFC",6.76364059434324,"1h","1h"
2991,"PFC_22",63,"OID30160","P19838","NFKB1",0,"Cardiometabolic_II","B13805","20212097_SS211647","PASS",-1.6949,2.3052,"Plate control","PASS","PFC",4.94235965755087,"24h","24h"
2992,"PFC_07",73,"OID30160","P19838","NFKB1",0,"Cardiometabolic_II","B13805","20212097_SS211647","PASS",-1.6949,2.7326,"Plate control","PASS","PFC",6.64652382955698,"Baseline","Baseline"
2993,"PFC_15",74,"OID30160","P19838","NFKB1",0,"Cardiometabolic_II","B13805","20212097_SS211647","PASS",-1.6949,1.5727,"Plate control","PASS","PFC",2.974608908019,"1h","1h"
2994,"PFC_23",75,"OID30160","P19838","NFKB1",0,"Cardiometabolic_II","B13805","20212097_SS211647","PASS",-1.6949,2.8621,"Plate control","PASS","PFC",7.27072888264491,"24h","24h"
2995,"PFC_08",85,"OID30160","P19838","NFKB1",0,"Cardiometabolic_II","B13805","20212097_SS211647","PASS",-1.6949,1.5584,"Plate control","PASS","PFC",2.94527021343948,"Baseline","Baseline"
2996,"PFC_16",86,"OID30160","P19838","NFKB1",0,"Cardiometabolic_II","B13805","20212097_SS211647","PASS",-1.6949,2.7682,"Plate control","PASS","PFC",6.81257401982699,"1h","1h"
2997,"PFC_24",87,"OID30160","P19838","NFKB1",0,"Cardiometabolic_II","B13805","20212097_SS211647","PASS",-1.6949,2.9563,"Plate control","PASS","PFC",7.76130903378528,"24h","24h"
2998,"PFC_01",1,"OID30161","Q01484","ANK2",0.5227,"Cardiometabolic_II","B13805","20212097_SS211647","WARN",-0.1547,-0.6736,"Plate control","PASS","PFC",0.626940310888892,"Baseline","Baseline"
2999,"PFC_09",2,"OID30161","Q01484","ANK2",0.5227,"Cardiometabolic_II","B13805","20212097_SS211647","PASS",-0.1547,-0.3998,"Plate control","PASS","PFC",0.757963352004302,"Baseline","Baseline"
3000,"PFC_17",3,"OID30161","Q01484","ANK2",0.5227,"Cardiometabolic_II","B13805","20212097_SS211647","PASS",-0.1547,-0.0452,"Plate control","PASS","PFC",0.969155454147848,"1h","1h"
3001,"PFC_25",4,"OID30161","Q01484","ANK2",0.5227,"Cardiometabolic_II","B13805","20212097_SS211647","PASS",-0.1547,-0.8741,"Plate control","PASS","PFC",0.545594119514208,"24h","24h"
3002,"PFC_02",13,"OID30161","Q01484","ANK2",0.5227,"Cardiometabolic_II","B13805","20212097_SS211647","PASS",-0.1547,-0.8848,"Plate control","PASS","PFC",0.541562594076331,"Baseline","Baseline"
3003,"PFC_10",14,"OID30161","Q01484","ANK2",0.5227,"Cardiometabolic_II","B13805","20212097_SS211647","PASS",-0.1547,-0.1863,"Plate control","PASS","PFC",0.87885678877767,"1h","1h"
3004,"PFC_18",15,"OID30161","Q01484","ANK2",0.5227,"Cardiometabolic_II","B13805","20212097_SS211647","PASS",-0.1547,-0.1001,"Plate control","PASS","PFC",0.932968320859403,"1h","1h"
3005,"PFC_26",16,"OID30161","Q01484","ANK2",0.5227,"Cardiometabolic_II","B13805","20212097_SS211647","PASS",-0.1547,-0.3924,"Plate control","PASS","PFC",0.761861153119994,"24h","24h"
3006,"PFC_03",25,"OID30161","Q01484","ANK2",0.5227,"Cardiometabolic_II","B13805","20212097_SS211647","PASS",-0.1547,-0.2685,"Plate control","PASS","PFC",0.830182254982492,"Baseline","Baseline"
3007,"PFC_11",26,"OID30161","Q01484","ANK2",0.5227,"Cardiometabolic_II","B13805","20212097_SS211647","PASS",-0.1547,-0.1583,"Plate control","PASS","PFC",0.896080345524943,"1h","1h"
3008,"PFC_19",27,"OID30161","Q01484","ANK2",0.5227,"Cardiometabolic_II","B13805","20212097_SS211647","PASS",-0.1547,0.6345,"Plate control","PASS","PFC",1.55239963604401,"24h","24h"
3009,"PFC_27",28,"OID30161","Q01484","ANK2",0.5227,"Cardiometabolic_II","B13805","20212097_SS211647","PASS",-0.1547,0.1259,"Plate control","PASS","PFC",1.09118823902842,"24h","24h"
3010,"PFC_04",37,"OID30161","Q01484","ANK2",0.5227,"Cardiometabolic_II","B13805","20212097_SS211647","PASS",-0.1547,0.9039,"Plate control","PASS","PFC",1.87111729520001,"Baseline","Baseline"
3011,"PFC_12",38,"OID30161","Q01484","ANK2",0.5227,"Cardiometabolic_II","B13805","20212097_SS211647","PASS",-0.1547,-0.78,"Plate control","PASS","PFC",0.582366793234228,"1h","1h"
3012,"PFC_20",39,"OID30161","Q01484","ANK2",0.5227,"Cardiometabolic_II","B13805","20212097_SS211647","PASS",-0.1547,0.0144,"Plate control","PASS","PFC",1.01003129891732,"24h","24h"
3013,"PFC_05",49,"OID30161","Q01484","ANK2",0.5227,"Cardiometabolic_II","B13805","20212097_SS211647","PASS",-0.1547,-0.5057,"Plate control","PASS","PFC",0.70431855715609,"Baseline","Baseline"
3014,"PFC_13",50,"OID30161","Q01484","ANK2",0.5227,"Cardiometabolic_II","B13805","20212097_SS211647","PASS",-0.1547,0.9008,"Plate control","PASS","PFC",1.86710103672483,"1h","1h"
3015,"PFC_21",51,"OID30161","Q01484","ANK2",0.5227,"Cardiometabolic_II","B13805","20212097_SS211647","PASS",-0.1547,-0.3358,"Plate control","PASS","PFC",0.79234464560306,"24h","24h"
3016,"PFC_06",61,"OID30161","Q01484","ANK2",0.5227,"Cardiometabolic_II","B13805","20212097_SS211647","PASS",-0.1547,-1.2297,"Plate control","PASS","PFC",0.426406105322456,"Baseline","Baseline"
3017,"PFC_14",62,"OID30161","Q01484","ANK2",0.5227,"Cardiometabolic_II","B13805","20212097_SS211647","PASS",-0.1547,-0.2711,"Plate control","PASS","PFC",0.828687462259416,"1h","1h"
3018,"PFC_22",63,"OID30161","Q01484","ANK2",0.5227,"Cardiometabolic_II","B13805","20212097_SS211647","PASS",-0.1547,0.4852,"Plate control","PASS","PFC",1.39977990284386,"24h","24h"
3019,"PFC_07",73,"OID30161","Q01484","ANK2",0.5227,"Cardiometabolic_II","B13805","20212097_SS211647","PASS",-0.1547,-0.5459,"Plate control","PASS","PFC",0.684963966421567,"Baseline","Baseline"
3020,"PFC_15",74,"OID30161","Q01484","ANK2",0.5227,"Cardiometabolic_II","B13805","20212097_SS211647","PASS",-0.1547,-0.8812,"Plate control","PASS","PFC",0.542915658852354,"1h","1h"
3021,"PFC_23",75,"OID30161","Q01484","ANK2",0.5227,"Cardiometabolic_II","B13805","20212097_SS211647","PASS",-0.1547,0.2119,"Plate control","PASS","PFC",1.15821252223782,"24h","24h"
3022,"PFC_08",85,"OID30161","Q01484","ANK2",0.5227,"Cardiometabolic_II","B13805","20212097_SS211647","PASS",-0.1547,-0.0786,"Plate control","PASS","PFC",0.946976152381906,"Baseline","Baseline"
3023,"PFC_16",86,"OID30161","Q01484","ANK2",0.5227,"Cardiometabolic_II","B13805","20212097_SS211647","PASS",-0.1547,0.1547,"Plate control","PASS","PFC",1.11319011278299,"1h","1h"
3024,"PFC_24",87,"OID30161","Q01484","ANK2",0.5227,"Cardiometabolic_II","B13805","20212097_SS211647","PASS",-0.1547,0.1809,"Plate control","PASS","PFC",1.13359083552602,"24h","24h"

Supplemental table 1_Olink proximity extension assay (PEA) data

,"SampleID","Index","OlinkID","UniProt","Assay","MissingFreq","Panel","Panel_Lot_Nr","PlateID","QC_Warning","LOD","NPX","Normalization","Assay_Warning","condition","Value","group","alt_group"
3025,"PFC_01",1,"OID30162","P59901","LILRA4",0.8409,"Cardiometabolic_II","B13805","20212097_SS211647","WARN",0.2633,-0.3885,"Plate control","PASS","PFC",0.7639234588028,"Baseline","Baseline"
3026,"PFC_09",2,"OID30162","P59901","LILRA4",0.8409,"Cardiometabolic_II","B13805","20212097_SS211647","PASS",0.2633,-0.3023,"Plate control","PASS","PFC",0.81095850396242,"Baseline","Baseline"
3027,"PFC_17",3,"OID30162","P59901","LILRA4",0.8409,"Cardiometabolic_II","B13805","20212097_SS211647","PASS",0.2633,-0.0625,"Plate control","PASS","PFC",0.957603280698574,"1h","1h"
3028,"PFC_25",4,"OID30162","P59901","LILRA4",0.8409,"Cardiometabolic_II","B13805","20212097_SS211647","PASS",0.2633,-0.6287,"Plate control","PASS","PFC",0.646758941702618,"24h","24h"
3029,"PFC_02",13,"OID30162","P59901","LILRA4",0.8409,"Cardiometabolic_II","B13805","20212097_SS211647","PASS",0.2633,-0.6173,"Plate control","PASS","PFC",0.651889796861775,"Baseline","Baseline"
3030,"PFC_10",14,"OID30162","P59901","LILRA4",0.8409,"Cardiometabolic_II","B13805","20212097_SS211647","PASS",0.2633,-0.8097,"Plate control","PASS","PFC",0.570500477839943,"1h","1h"
3031,"PFC_18",15,"OID30162","P59901","LILRA4",0.8409,"Cardiometabolic_II","B13805","20212097_SS211647","PASS",0.2633,-0.6077,"Plate control","PASS","PFC",0.656242074811933,"1h","1h"
3032,"PFC_26",16,"OID30162","P59901","LILRA4",0.8409,"Cardiometabolic_II","B13805","20212097_SS211647","PASS",0.2633,-0.1912,"Plate control","PASS","PFC",0.875876884331805,"24h","24h"
3033,"PFC_03",25,"OID30162","P59901","LILRA4",0.8409,"Cardiometabolic_II","B13805","20212097_SS211647","PASS",0.2633,0.0836,"Plate control","PASS","PFC",1.05965894282078,"Baseline","Baseline"
3034,"PFC_11",26,"OID30162","P59901","LILRA4",0.8409,"Cardiometabolic_II","B13805","20212097_SS211647","PASS",0.2633,-1.999,"Plate control","PASS","PFC",0.250173346865645,"1h","1h"
3035,"PFC_19",27,"OID30162","P59901","LILRA4",0.8409,"Cardiometabolic_II","B13805","20212097_SS211647","PASS",0.2633,-1.5107,"Plate control","PASS","PFC",0.350940900031437,"24h","24h"
3036,"PFC_27",28,"OID30162","P59901","LILRA4",0.8409,"Cardiometabolic_II","B13805","20212097_SS211647","PASS",0.2633,-0.8311,"Plate control","PASS","PFC",0.562100498701377,"24h","24h"
3037,"PFC_04",37,"OID30162","P59901","LILRA4",0.8409,"Cardiometabolic_II","B13805","20212097_SS211647","PASS",0.2633,0.2935,"Plate control","PASS","PFC",1.22561002237291,"Baseline","Baseline"
3038,"PFC_12",38,"OID30162","P59901","LILRA4",0.8409,"Cardiometabolic_II","B13805","20212097_SS211647","PASS",0.2633,-0.7322,"Plate control","PASS","PFC",0.601985231957674,"1h","1h"
3039,"PFC_20",39,"OID30162","P59901","LILRA4",0.8409,"Cardiometabolic_II","B13805","20212097_SS211647","PASS",0.2633,-1.0809,"Plate control","PASS","PFC",0.472733824651537,"24h","24h"
3040,"PFC_05",49,"OID30162","P59901","LILRA4",0.8409,"Cardiometabolic_II","B13805","20212097_SS211647","PASS",0.2633,-1.486,"Plate control","PASS","PFC",0.357000994921266,"Baseline","Baseline"
3041,"PFC_13",50,"OID30162","P59901","LILRA4",0.8409,"Cardiometabolic_II","B13805","20212097_SS211647","PASS",0.2633,-0.6712,"Plate control","PASS","PFC",0.627984127451672,"1h","1h"
3042,"PFC_21",51,"OID30162","P59901","LILRA4",0.8409,"Cardiometabolic_II","B13805","20212097_SS211647","PASS",0.2633,-0.8311,"Plate control","PASS","PFC",0.562100498701377,"24h","24h"
3043,"PFC_06",61,"OID30162","P59901","LILRA4",0.8409,"Cardiometabolic_II","B13805","20212097_SS211647","PASS",0.2633,-1.0998,"Plate control","PASS","PFC",0.466581173170141,"Baseline","Baseline"
3044,"PFC_14",62,"OID30162","P59901","LILRA4",0.8409,"Cardiometabolic_II","B13805","20212097_SS211647","PASS",0.2633,-0.3571,"Plate control","PASS","PFC",0.780732374487407,"1h","1h"
3045,"PFC_22",63,"OID30162","P59901","LILRA4",0.8409,"Cardiometabolic_II","B13805","20212097_SS211647","PASS",0.2633,-1.433,"Plate control","PASS","PFC",0.37035994950508,"24h","24h"
3046,"PFC_07",73,"OID30162","P59901","LILRA4",0.8409,"Cardiometabolic_II","B13805","20212097_SS211647","PASS",0.2633,-0.249,"Plate control","PASS","PFC",0.84147948228538,"Baseline","Baseline"
3047,"PFC_15",74,"OID30162","P59901","LILRA4",0.8409,"Cardiometabolic_II","B13805","20212097_SS211647","PASS",0.2633,-1.0001,"Plate control","PASS","PFC",0.499965343842077,"1h","1h"
3048,"PFC_23",75,"OID30162","P59901","LILRA4",0.8409,"Cardiometabolic_II","B13805","20212097_SS211647","PASS",0.2633,0.3397,"Plate control","PASS","PFC",1.26549341465021,"24h","24h"
3049,"PFC_08",85,"OID30162","P59901","LILRA4",0.8409,"Cardiometabolic_II","B13805","20212097_SS211647","PASS",0.2633,-0.1622,"Plate control","PASS","PFC",0.893661266011763,"Baseline","Baseline"
3050,"PFC_16",86,"OID30162","P59901","LILRA4",0.8409,"Cardiometabolic_II","B13805","20212097_SS211647","PASS",0.2633,-1.1021,"Plate control","PASS","PFC",0.465837924108925,"1h","1h"
3051,"PFC_24",87,"OID30162","P59901","LILRA4",0.8409,"Cardiometabolic_II","B13805","20212097_SS211647","PASS",0.2633,-0.7345,"Plate control","PASS","PFC",0.601026288510645,"24h","24h"
3052,"PFC_01",1,"OID30163","O43896","KIF1C",0,"Cardiometabolic_II","B13805","20212097_SS211647","WARN",-0.1559,0.2687,"Plate control","PASS","PFC",1.20472177409611,"Baseline","Baseline"
3053,"PFC_09",2,"OID30163","O43896","KIF1C",0,"Cardiometabolic_II","B13805","20212097_SS211647","PASS",-0.1559,0.389,"Plate control","PASS","PFC",1.30948542308357,"Baseline","Baseline"
3054,"PFC_17",3,"OID30163","O43896","KIF1C",0,"Cardiometabolic_II","B13805","20212097_SS211647","PASS",-0.1559,0.7016,"Plate control","PASS","PFC",1.62630742558662,"1h","1h"
3055,"PFC_25",4,"OID30163","O43896","KIF1C",0,"Cardiometabolic_II","B13805","20212097_SS211647","PASS",-0.1559,0.7614,"Plate control","PASS","PFC",1.69513479590543,"24h","24h"
3056,"PFC_02",13,"OID30163","O43896","KIF1C",0,"Cardiometabolic_II","B13805","20212097_SS211647","PASS",-0.1559,0.9306,"Plate control","PASS","PFC",1.90606854289285,"Baseline","Baseline"
3057,"PFC_10",14,"OID30163","O43896","KIF1C",0,"Cardiometabolic_II","B13805","20212097_SS211647","PASS",-0.1559,0.5878,"Plate control","PASS","PFC",1.50295311008078,"1h","1h"
3058,"PFC_18",15,"OID30163","O43896","KIF1C",0,"Cardiometabolic_II","B13805","20212097_SS211647","PASS",-0.1559,0.6029,"Plate control","PASS","PFC",1.51876641330023,"1h","1h"
3059,"PFC_26",16,"OID30163","O43896","KIF1C",0,"Cardiometabolic_II","B13805","20212097_SS211647","PASS",-0.1559,0.8785,"Plate control","PASS","PFC",1.83846281921883,"24h","24h"
3060,"PFC_03",25,"OID30163","O43896","KIF1C",0,"Cardiometabolic_II","B13805","20212097_SS211647","PASS",-0.1559,0.2614,"Plate control","PASS","PFC",1.19864130921,"Baseline","Baseline"
3061,"PFC_11",26,"OID30163","O43896","KIF1C",0,"Cardiometabolic_II","B13805","20212097_SS211647","PASS",-0.1559,1.202,"Plate control","PASS","PFC",2.30058378670121,"1h","1h"
3062,"PFC_19",27,"OID30163","O43896","KIF1C",0,"Cardiometabolic_II","B13805","20212097_SS211647","PASS",-0.1559,0.9258,"Plate control","PASS","PFC",1.89973738797138,"24h","24h"
3063,"PFC_27",28,"OID30163","O43896","KIF1C",0,"Cardiometabolic_II","B13805","20212097_SS211647","PASS",-0.1559,1.2067,"Plate control","PASS","PFC",2.30809083113278,"24h","24h"
3064,"PFC_04",37,"OID30163","O43896","KIF1C",0,"Cardiometabolic_II","B13805","20212097_SS211647","PASS",-0.1559,1.2305,"Plate control","PASS","PFC",2.34648298659138,"Baseline","Baseline"
3065,"PFC_12",38,"OID30163","O43896","KIF1C",0,"Cardiometabolic_II","B13805","20212097_SS211647","PASS",-0.1559,0.159,"Plate control","PASS","PFC",1.11651296198257,"1h","1h"
3066,"PFC_20",39,"OID30163","O43896","KIF1C",0,"Cardiometabolic_II","B13805","20212097_SS211647","PASS",-0.1559,0.9163,"Plate control","PASS","PFC",1.88726890752673,"24h","24h"
3067,"PFC_05",49,"OID30163","O43896","KIF1C",0,"Cardiometabolic_II","B13805","20212097_SS211647","PASS",-0.1559,0.5419,"Plate control","PASS","PFC",1.4558886309916,"Baseline","Baseline"
3068,"PFC_13",50,"OID30163","O43896","KIF1C",0,"Cardiometabolic_II","B13805","20212097_SS211647","PASS",-0.1559,0.964,"Plate control","PASS","PFC",1.95071092305528,"1h","1h"
3069,"PFC_21",51,"OID30163","O43896","KIF1C",0,"Cardiometabolic_II","B13805","20212097_SS211647","PASS",-0.1559,0.4788,"Plate control","PASS","PFC",1.39358403352708,"24h","24h"
3070,"PFC_06",61,"OID30163","O43896","KIF1C",0,"Cardiometabolic_II","B13805","20212097_SS211647","PASS",-0.1559,0.5828,"Plate control","PASS","PFC",1.49775328733786,"Baseline","Baseline"
3071,"PFC_14",62,"OID30163","O43896","KIF1C",0,"Cardiometabolic_II","B13805","20212097_SS211647","PASS",-0.1559,0.9271,"Plate control","PASS","PFC",1.90144999636235,"1h","1h"
3072,"PFC_22",63,"OID30163","O43896","KIF1C",0,"Cardiometabolic_II","B13805","20212097_SS211647","PASS",-0.1559,0.8662,"Plate control","PASS","PFC",1.82285524515392,"24h","24h"
3073,"PFC_07",73,"OID30163","O43896","KIF1C",0,"Cardiometabolic_II","B13805","20212097_SS211647","PASS",-0.1559,0.5515,"Plate control","PASS","PFC",1.46560872788076,"Baseline","Baseline"
3074,"PFC_15",74,"OID30163","O43896","KIF1C",0,"Cardiometabolic_II","B13805","20212097_SS211647","PASS",-0.1559,0.4653,"Plate control","PASS","PFC",1.38060441216623,"1h","1h"
3075,"PFC_23",75,"OID30163","O43896","KIF1C",0,"Cardiometabolic_II","B13805","20212097_SS211647","PASS",-0.1559,1.1933,"Plate control","PASS","PFC",2.28675213813019,"24h","24h"
3076,"PFC_08",85,"OID30163","O43896","KIF1C",0,"Cardiometabolic_II","B13805","20212097_SS211647","PASS",-0.1559,0.2525,"Plate control","PASS","PFC",1.19126964042587,"Baseline","Baseline"
3077,"PFC_16",86,"OID30163","O43896","KIF1C",0,"Cardiometabolic_II","B13805","20212097_SS211647","PASS",-0.1559,0.665,"Plate control","PASS","PFC",1.58556827322057,"1h","1h"
3078,"PFC_24",87,"OID30163","O43896","KIF1C",0,"Cardiometabolic_II","B13805","20212097_SS211647","PASS",-0.1559,1.09,"Plate control","PASS","PFC",2.12874036490672,"24h","24h"
3079,"PFC_01",1,"OID30164","Q03013","GSTM4",0.6818,"Cardiometabolic_II","B13805","20212097_SS211647","WARN",-0.0466,-0.6884,"Plate control","PASS","PFC",0.62054167122563,"Baseline","Baseline"
3080,"PFC_09",2,"OID30164","Q03013","GSTM4",0.6818,"Cardiometabolic_II","B13805","20212097_SS211647","PASS",-0.0466,-1.2119,"Plate control","PASS","PFC",0.43169970139326,"Baseline","Baseline"
3081,"PFC_17",3,"OID30164","Q03013","GSTM4",0.6818,"Cardiometabolic_II","B13805","20212097_SS211647","PASS",-0.0466,-0.9004,"Plate control","PASS","PFC",0.535738172512964,"1h","1h"
3082,"PFC_25",4,"OID30164","Q03013","GSTM4",0.6818,"Cardiometabolic_II","B13805","20212097_SS211647","PASS",-0.0466,-0.2922,"Plate control","PASS","PFC",0.816655770733371,"24h","24h"
3083,"PFC_02",13,"OID30164","Q03013","GSTM4",0.6818,"Cardiometabolic_II","B13805","20212097_SS211647","PASS",-0.0466,-0.8053,"Plate control","PASS","PFC",0.572243073324508,"Baseline","Baseline"
3084,"PFC_10",14,"OID30164","Q03013","GSTM4",0.6818,"Cardiometabolic_II","B13805","20212097_SS211647","PASS",-0.0466,0.0325,"Plate control","PASS","PFC",1.02278293874728,"1h","1h"
3085,"PFC_18",15,"OID30164","Q03013","GSTM4",0.6818,"Cardiometabolic_II","B13805","20212097_SS211647","PASS",-0.0466,-1.09,"Plate control","PASS","PFC",0.469761374607006,"1h","1h"
3086,"PFC_26",16,"OID30164","Q03013","GSTM4",0.6818,"Cardiometabolic_II","B13805","20212097_SS211647","PASS",-0.0466,-0.4357,"Plate control","PASS","PFC",0.739334940032074,"24h","24h"
3087,"PFC_03",25,"OID30164","Q03013","GSTM4",0.6818,"Cardiometabolic_II","B13805","20212097_SS211647","PASS",-0.0466,-0.0613,"Plate control","PASS","PFC",0.958400124067406,"Baseline","Baseline"
3088,"PFC_11",26,"OID30164","Q03013","GSTM4",0.6818,"Cardiometabolic_II","B13805","20212097_SS211647","PASS",-0.0466,-0.4234,"Plate control","PASS","PFC",0.745665242378356,"1h","1h"
3089,"PFC_19",27,"OID30164","Q03013","GSTM4",0.6818,"Cardiometabolic_II","B13805","20212097_SS211647","PASS",-0.0466,0.3655,"Plate control","PASS","PFC",1.28832805262478,"24h","24h"
3090,"PFC_27",28,"OID30164","Q03013","GSTM4",0.6818,"Cardiometabolic_II","B13805","20212097_SS211647","PASS",-0.0466,-0.8869,"Plate control","PASS","PFC",0.540774864099991,"24h","24h"
3091,"PFC_04",37,"OID30164","Q03013","GSTM4",0.6818,"Cardiometabolic_II","B13805","20212097_SS211647","PASS",-0.0466,0.185,"Plate control","PASS","PFC",1.13681697323601,"Baseline","Baseline"
3092,"PFC_12",38,"OID30164","Q03013","GSTM4",0.6818,"Cardiometabolic_II","B13805","20212097_SS211647","PASS",-0.0466,-1.0015,"Plate control","PASS","PFC",0.499480409775762,"1h","1h"
3093,"PFC_20",39,"OID30164","Q03013","GSTM4",0.6818,"Cardiometabolic_II","B13805","20212097_SS211647","PASS",-0.0466,-0.1724,"Plate control","PASS","PFC",0.887365273264444,"24h","24h"
3094,"PFC_05",49,"OID30164","Q03013","GSTM4",0.6818,"Cardiometabolic_II","B13805","20212097_SS211647","PASS",-0.0466,1.6683,"Plate control","PASS","PFC",3.17839846105057,"Baseline","Baseline"
3095,"PFC_13",50,"OID30164","Q03013","GSTM4",0.6818,"Cardiometabolic_II","B13805","20212097_SS211647","PASS",-0.0466,-0.3491,"Plate control","PASS","PFC",0.785073699625431,"1h","1h"
3096,"PFC_21",51,"OID30164","Q03013","GSTM4",0.6818,"Cardiometabolic_II","B13805","20212097_SS211647","PASS",-0.0466,-0.2746,"Plate control","PASS","PFC",0.826679490605952,"24h","24h"
3097,"PFC_06",61,"OID30164","Q03013","GSTM4",0.6818,"Cardiometabolic_II","B13805","20212097_SS211647","PASS",-0.0466,0.1015,"Plate control","PASS","PFC",1.07288838717164,"Baseline","Baseline"
3098,"PFC_14",62,"OID30164","Q03013","GSTM4",0.6818,"Cardiometabolic_II","B13805","20212097_SS211647","PASS",-0.0466,1.4981,"Plate control","PASS","PFC",2.82470459558661,"1h","1h"
3099,"PFC_22",63,"OID30164","Q03013","GSTM4",0.6818,"Cardiometabolic_II","B13805","20212097_SS211647","PASS",-0.0466,-0.7858,"Plate control","PASS","PFC",0.580030230943273,"24h","24h"
3100,"PFC_07",73,"OID30164","Q03013","GSTM4",0.6818,"Cardiometabolic_II","B13805","20212097_SS211647","PASS",-0.0466,-1.4033,"Plate control","PASS","PFC",0.378063375077796,"Baseline","Baseline"
3101,"PFC_15",74,"OID30164","Q03013","GSTM4",0.6818,"Cardiometabolic_II","B13805","20212097_SS211647","PASS",-0.0466,-0.8352,"Plate control","PASS","PFC",0.560505331090089,"1h","1h"
3102,"PFC_23",75,"OID30164","Q03013","GSTM4",0.6818,"Cardiometabolic_II","B13805","20212097_SS211647","PASS",-0.0466,1.3536,"Plate control","PASS","PFC",2.55549009587163,"24h","24h"
3103,"PFC_08",85,"OID30164","Q03013","GSTM4",0.6818,"Cardiometabolic_II","B13805","20212097_SS211647","PASS",-0.0466,-0.9756,"Plate control","PASS","PFC",0.508528311087894,"Baseline","Baseline"
3104,"PFC_16",86,"OID30164","Q03013","GSTM4",0.6818,"Cardiometabolic_II","B13805","20212097_SS211647","PASS",-0.0466,-0.2028,"Plate control","PASS","PFC",0.868862626738059,"1h","1h"
3105,"PFC_24",87,"OID30164","Q03013","GSTM4",0.6818,"Cardiometabolic_II","B13805","20212097_SS211647","PASS",-0.0466,0.011,"Plate control","PASS","PFC",1.0076537604105,"24h","24h"
3106,"PFC_01",1,"OID30165","O94766","B3GAT3",1,"Cardiometabolic_II","B13805","20212097_SS211647","WARN",0.9532,-0.6756,"Plate control","PASS","PFC",0.626071789223586,"Baseline","Baseline"
3107,"PFC_09",2,"OID30165","O94766","B3GAT3",1,"Cardiometabolic_II","B13805","20212097_SS211647","PASS",0.9532,0.2343,"Plate control","PASS","PFC",1.17633583702054,"Baseline","Baseline"
3108,"PFC_17",3,"OID30165","O94766","B3GAT3",1,"Cardiometabolic_II","B13805","20212097_SS211647","PASS",0.9532,0.0091,"Plate control","PASS","PFC",1.00632757439246,"1h","1h"
3109,"PFC_25",4,"OID30165","O94766","B3GAT3",1,"Cardiometabolic_II","B13805","20212097_SS211647","PASS",0.9532,-0.2739,"Plate control","PASS","PFC",0.82708069532165,"24h","24h"
3110,"PFC_02",13,"OID30165","O94766","B3GAT3",1,"Cardiometabolic_II","B13805","20212097_SS211647","PASS",0.9532,-0.1402,"Plate control","PASS","PFC",0.907393355168402,"Baseline","Baseline"
3111,"PFC_10",14,"OID30165","O94766","B3GAT3",1,"Cardiometabolic_II","B13805","20212097_SS211647","PASS",0.9532,-0.0815,"Plate control","PASS","PFC",0.945074522115987,"1h","1h"
3112,"PFC_18",15,"OID30165","O94766","B3GAT3",1,"Cardiometabolic_II","B13805","20212097_SS211647","PASS",0.9532,-0.3029,"Plate control","PASS","PFC",0.810621305925297,"1h","1h"
3113,"PFC_26",16,"OID30165","O94766","B3GAT3",1,"Cardiometabolic_II","B13805","20212097_SS211647","PASS",0.9532,-0.0229,"Plate control","PASS","PFC",0.984252242836128,"24h","24h"
3114,"PFC_03",25,"OID30165","O94766","B3GAT3",1,"Cardiometabolic_II","B13805","20212097_SS211647","PASS",0.9532,0.5288,"Plate control","PASS","PFC",1.44272866833597,"Baseline","Baseline"
3115,"PFC_11",26,"OID30165","O94766","B3GAT3",1,"Cardiometabolic_II","B13805","20212097_SS211647","PASS",0.9532,0.1069,"Plate control","PASS","PFC",1.07691172777318,"1h","1h"
3116,"PFC_19",27,"OID30165","O94766","B3GAT3",1,"Cardiometabolic_II","B13805","20212097_SS211647","PASS",0.9532,0.5001,"Plate control","PASS","PFC",1.41431159158484,"24h","24h"
3117,"PFC_27",28,"OID30165","O94766","B3GAT3",1,"Cardiometabolic_II","B13805","20212097_SS211647","PASS",0.9532,0.0861,"Plate control","PASS","PFC",1.06149678375014,"24h","24h"
3118,"PFC_04",37,"OID30165","O94766","B3GAT3",1,"Cardiometabolic_II","B13805","20212097_SS211647","PASS",0.9532,-0.5403,"Plate control","PASS","PFC",0.687627905998766,"Baseline","Baseline"
3119,"PFC_12",38,"OID30165","O94766","B3GAT3",1,"Cardiometabolic_II","B13805","20212097_SS211647","PASS",0.9532,-0.2204,"Plate control","PASS","PFC",0.858327424550095,"1h","1h"
3120,"PFC_20",39,"OID30165","O94766","B3GAT3",1,"Cardiometabolic_II","B13805","20212097_SS211647","PASS",0.9532,0.1943,"Plate control","PASS","PFC",1.1441688716964,"24h","24h"
3121,"PFC_05",49,"OID30165","O94766","B3GAT3",1,"Cardiometabolic_II","B13805","20212097_SS211647","PASS",0.9532,-0.0297,"Plate control","PASS","PFC",0.979623983489196,"Baseline","Baseline"
3122,"PFC_13",50,"OID30165","O94766","B3GAT3",1,"Cardiometabolic_II","B13805","20212097_SS211647","PASS",0.9532,0.0825,"Plate control","PASS","PFC",1.05885130118847,"1h","1h"
3123,"PFC_21",51,"OID30165","O94766","B3GAT3",1,"Cardiometabolic_II","B13805","20212097_SS211647","PASS",0.9532,0.3417,"Plate control","PASS","PFC",1.26724897761734,"24h","24h"
3124,"PFC_06",61,"OID30165","O94766","B3GAT3",1,"Cardiometabolic_II","B13805","20212097_SS211647","PASS",0.9532,-0.1545,"Plate control","PASS","PFC",0.898443695789984,"Baseline","Baseline"
3125,"PFC_14",62,"OID30165","O94766","B3GAT3",1,"Cardiometabolic_II","B13805","20212097_SS211647","PASS",0.9532,0.0213,"Plate control","PASS","PFC",1.01487356166481,"1h","1h"
3126,"PFC_22",63,"OID30165","O94766","B3GAT3",1,"Cardiometabolic_II","B13805","20212097_SS211647","PASS",0.9532,-0.3493,"Plate control","PASS","PFC",0.784964872844618,"24h","24h"
3127,"PFC_07",73,"OID30165","O94766","B3GAT3",1,"Cardiometabolic_II","B13805","20212097_SS211647","PASS",0.9532,0.0675,"Plate control","PASS","PFC",1.04789923837507,"Baseline","Baseline"
3128,"PFC_15",74,"OID30165","O94766","B3GAT3",1,"Cardiometabolic_II","B13805","20212097_SS211647","PASS",0.9532,0.0477,"Plate control","PASS","PFC",1.03361577954023,"1h","1h"
3129,"PFC_23",75,"OID30165","O94766","B3GAT3",1,"Cardiometabolic_II","B13805","20212097_SS211647","PASS",0.9532,0.478,"Plate control","PASS","PFC",1.39281148066904,"24h","24h"
3130,"PFC_08",85,"OID30165","O94766","B3GAT3",1,"Cardiometabolic_II","B13805","20212097_SS211647","PASS",0.9532,-0.2897,"Plate control","PASS","PFC",0.818072154194124,"Baseline","Baseline"
3131,"PFC_16",86,"OID30165","O94766","B3GAT3",1,"Cardiometabolic_II","B13805","20212097_SS211647","PASS",0.9532,-0.3432,"Plate control","PASS","PFC",0.788290886160153,"1h","1h"
3132,"PFC_24",87,"OID30165","O94766","B3GAT3",1,"Cardiometabolic_II","B13805","20212097_SS211647","PASS",0.9532,-0.0048,"Plate control","PASS","PFC",0.996678422218825,"24h","24h"
3133,"PFC_01",1,"OID30166","Q3SXY8","ARL13B",0,"Cardiometabolic_II","B13805","20212097_SS211647","WARN",-0.1372,1.9976,"Plate control","PASS","PFC",3.99335131881746,"Baseline","Baseline"
3134,"PFC_09",2,"OID30166","Q3SXY8","ARL13B",0,"Cardiometabolic_II","B13805","20212097_SS211647","PASS",-0.1372,2.5471,"Plate control","PASS","PFC",5.84458261573896,"Baseline","Baseline"
3135,"PFC_17",3,"OID30166","Q3SXY8","ARL13B",0,"Cardiometabolic_II","B13805","20212097_SS211647","PASS",-0.1372,3.6549,"Plate control","PASS","PFC",12.596054502881,"1h","1h"
3136,"PFC_25",4,"OID30166","Q3SXY8","ARL13B",0,"Cardiometabolic_II","B13805","20212097_SS211647","PASS",-0.1372,2.9799,"Plate control","PASS","PFC",7.88931477136518,"24h","24h"

Supplemental table 1_Olink proximity extension assay (PEA) data

,"SampleID","Index","OlinkID","UniProt","Assay","MissingFreq","Panel","Panel_Lot_Nr","PlateID","QC_Warning","LOD","NPX","Normalization","Assay_Warning","condition","Value","group","alt_group"
3137,"PFC_02",13,"OID30166","Q3SXY8","ARL13B",0,"Cardiometabolic_II","B13805","20212097_SS211647","PASS",-0.1372,2.0289,"Plate control","PASS","PFC",4.08093575827863,"Baseline","Baseline"
3138,"PFC_10",14,"OID30166","Q3SXY8","ARL13B",0,"Cardiometabolic_II","B13805","20212097_SS211647","PASS",-0.1372,1.9512,"Plate control","PASS","PFC",3.86696042564703,"1h","1h"
3139,"PFC_18",15,"OID30166","Q3SXY8","ARL13B",0,"Cardiometabolic_II","B13805","20212097_SS211647","PASS",-0.1372,2.6551,"Plate control","PASS","PFC",6.29890040392791,"1h","1h"
3140,"PFC_26",16,"OID30166","Q3SXY8","ARL13B",0,"Cardiometabolic_II","B13805","20212097_SS211647","PASS",-0.1372,3.5382,"Plate control","PASS","PFC",11.6172766241128,"24h","24h"
3141,"PFC_03",25,"OID30166","Q3SXY8","ARL13B",0,"Cardiometabolic_II","B13805","20212097_SS211647","PASS",-0.1372,1.2392,"Plate control","PASS","PFC",2.36067592316165,"Baseline","Baseline"
3142,"PFC_11",26,"OID30166","Q3SXY8","ARL13B",0,"Cardiometabolic_II","B13805","20212097_SS211647","PASS",-0.1372,3.1246,"Plate control","PASS","PFC",8.72164337305834,"1h","1h"
3143,"PFC_19",27,"OID30166","Q3SXY8","ARL13B",0,"Cardiometabolic_II","B13805","20212097_SS211647","PASS",-0.1372,2.4595,"Plate control","PASS","PFC",5.50026069709644,"24h","24h"
3144,"PFC_27",28,"OID30166","Q3SXY8","ARL13B",0,"Cardiometabolic_II","B13805","20212097_SS211647","PASS",-0.1372,4.0081,"Plate control","PASS","PFC",16.0900845273962,"24h","24h"
3145,"PFC_04",37,"OID30166","Q3SXY8","ARL13B",0,"Cardiometabolic_II","B13805","20212097_SS211647","PASS",-0.1372,2.8613,"Plate control","PASS","PFC",7.26669825209577,"Baseline","Baseline"
3146,"PFC_12",38,"OID30166","Q3SXY8","ARL13B",0,"Cardiometabolic_II","B13805","20212097_SS211647","PASS",-0.1372,1.8118,"Plate control","PASS","PFC",3.51080045641345,"1h","1h"
3147,"PFC_20",39,"OID30166","Q3SXY8","ARL13B",0,"Cardiometabolic_II","B13805","20212097_SS211647","PASS",-0.1372,3.8259,"Plate control","PASS","PFC",14.1811241924252,"24h","24h"
3148,"PFC_05",49,"OID30166","Q3SXY8","ARL13B",0,"Cardiometabolic_II","B13805","20212097_SS211647","PASS",-0.1372,1.9082,"Plate control","PASS","PFC",3.75340508236772,"Baseline","Baseline"
3149,"PFC_13",50,"OID30166","Q3SXY8","ARL13B",0,"Cardiometabolic_II","B13805","20212097_SS211647","PASS",-0.1372,3.4174,"Plate control","PASS","PFC",10.6841482906525,"1h","1h"
3150,"PFC_21",51,"OID30166","Q3SXY8","ARL13B",0,"Cardiometabolic_II","B13805","20212097_SS211647","PASS",-0.1372,1.8927,"Plate control","PASS","PFC",3.71329517091499,"24h","24h"
3151,"PFC_06",61,"OID30166","Q3SXY8","ARL13B",0,"Cardiometabolic_II","B13805","20212097_SS211647","PASS",-0.1372,2.7484,"Plate control","PASS","PFC",6.7197147858396,"Baseline","Baseline"
3152,"PFC_14",62,"OID30166","Q3SXY8","ARL13B",0,"Cardiometabolic_II","B13805","20212097_SS211647","PASS",-0.1372,3.2943,"Plate control","PASS","PFC",9.81031866675436,"1h","1h"
3153,"PFC_22",63,"OID30166","Q3SXY8","ARL13B",0,"Cardiometabolic_II","B13805","20212097_SS211647","PASS",-0.1372,2.4542,"Plate control","PASS","PFC",5.48009156921025,"24h","24h"
3154,"PFC_07",73,"OID30166","Q3SXY8","ARL13B",0,"Cardiometabolic_II","B13805","20212097_SS211647","PASS",-0.1372,3.3861,"Plate control","PASS","PFC",10.4548466807806,"Baseline","Baseline"
3155,"PFC_15",74,"OID30166","Q3SXY8","ARL13B",0,"Cardiometabolic_II","B13805","20212097_SS211647","PASS",-0.1372,1.9339,"Plate control","PASS","PFC",3.82086689403931,"1h","1h"
3156,"PFC_23",75,"OID30166","Q3SXY8","ARL13B",0,"Cardiometabolic_II","B13805","20212097_SS211647","PASS",-0.1372,3.3781,"Plate control","PASS","PFC",10.3970331420885,"24h","24h"
3157,"PFC_08",85,"OID30166","Q3SXY8","ARL13B",0,"Cardiometabolic_II","B13805","20212097_SS211647","PASS",-0.1372,2.6975,"Plate control","PASS","PFC",6.48676871227596,"Baseline","Baseline"
3158,"PFC_16",86,"OID30166","Q3SXY8","ARL13B",0,"Cardiometabolic_II","B13805","20212097_SS211647","PASS",-0.1372,3.4403,"Plate control","PASS","PFC",10.8550916377554,"1h","1h"
3159,"PFC_24",87,"OID30166","Q3SXY8","ARL13B",0,"Cardiometabolic_II","B13805","20212097_SS211647","PASS",-0.1372,3.8326,"Plate control","PASS","PFC",14.2471357172685,"24h","24h"
3160,"PFC_01",1,"OID30167","O95670","ATP6V1G2",0.9773,"Cardiometabolic_II","B13805","20212097_SS211647","WARN",0.1989,-0.6782,"Plate control","PASS","PFC",0.624944509582233,"Baseline","Baseline"
3161,"PFC_09",2,"OID30167","O95670","ATP6V1G2",0.9773,"Cardiometabolic_II","B13805","20212097_SS211647","PASS",0.1989,-0.6819,"Plate control","PASS","PFC",0.623343802540071,"Baseline","Baseline"
3162,"PFC_17",3,"OID30167","O95670","ATP6V1G2",0.9773,"Cardiometabolic_II","B13805","20212097_SS211647","PASS",0.1989,-0.6917,"Plate control","PASS","PFC",0.619123875224512,"1h","1h"
3163,"PFC_25",4,"OID30167","O95670","ATP6V1G2",0.9773,"Cardiometabolic_II","B13805","20212097_SS211647","PASS",0.1989,-0.3985,"Plate control","PASS","PFC",0.758646654025365,"24h","24h"
3164,"PFC_02",13,"OID30167","O95670","ATP6V1G2",0.9773,"Cardiometabolic_II","B13805","20212097_SS211647","PASS",0.1989,0.1011,"Plate control","PASS","PFC",1.07259096058138,"Baseline","Baseline"
3165,"PFC_10",14,"OID30167","O95670","ATP6V1G2",0.9773,"Cardiometabolic_II","B13805","20212097_SS211647","PASS",0.1989,-1.037,"Plate control","PASS","PFC",0.487339815445774,"1h","1h"
3166,"PFC_18",15,"OID30167","O95670","ATP6V1G2",0.9773,"Cardiometabolic_II","B13805","20212097_SS211647","PASS",0.1989,-0.5162,"Plate control","PASS","PFC",0.69921110340917,"1h","1h"
3167,"PFC_26",16,"OID30167","O95670","ATP6V1G2",0.9773,"Cardiometabolic_II","B13805","20212097_SS211647","PASS",0.1989,-0.1999,"Plate control","PASS","PFC",0.870610907354314,"24h","24h"
3168,"PFC_03",25,"OID30167","O95670","ATP6V1G2",0.9773,"Cardiometabolic_II","B13805","20212097_SS211647","PASS",0.1989,-1.147,"Plate control","PASS","PFC",0.451563255075911,"Baseline","Baseline"
3169,"PFC_11",26,"OID30167","O95670","ATP6V1G2",0.9773,"Cardiometabolic_II","B13805","20212097_SS211647","PASS",0.1989,-2.5251,"Plate control","PASS","PFC",0.17372773517396,"1h","1h"
3170,"PFC_19",27,"OID30167","O95670","ATP6V1G2",0.9773,"Cardiometabolic_II","B13805","20212097_SS211647","PASS",0.1989,-0.0841,"Plate control","PASS","PFC",0.943372859005332,"24h","24h"
3171,"PFC_27",28,"OID30167","O95670","ATP6V1G2",0.9773,"Cardiometabolic_II","B13805","20212097_SS211647","PASS",0.1989,0.1211,"Plate control","PASS","PFC",1.08756377241858,"24h","24h"
3172,"PFC_04",37,"OID30167","O95670","ATP6V1G2",0.9773,"Cardiometabolic_II","B13805","20212097_SS211647","PASS",0.1989,-0.6067,"Plate control","PASS","PFC",0.656697104839026,"Baseline","Baseline"
3173,"PFC_12",38,"OID30167","O95670","ATP6V1G2",0.9773,"Cardiometabolic_II","B13805","20212097_SS211647","PASS",0.1989,0.0291,"Plate control","PASS","PFC",1.02037538382934,"1h","1h"
3174,"PFC_20",39,"OID30167","O95670","ATP6V1G2",0.9773,"Cardiometabolic_II","B13805","20212097_SS211647","PASS",0.1989,-0.5369,"Plate control","PASS","PFC",0.689250350025774,"24h","24h"
3175,"PFC_05",49,"OID30167","O95670","ATP6V1G2",0.9773,"Cardiometabolic_II","B13805","20212097_SS211647","PASS",0.1989,-0.5008,"Plate control","PASS","PFC",0.706714786623175,"Baseline","Baseline"
3176,"PFC_13",50,"OID30167","O95670","ATP6V1G2",0.9773,"Cardiometabolic_II","B13805","20212097_SS211647","PASS",0.1989,-0.3185,"Plate control","PASS","PFC",0.801903199716338,"1h","1h"
3177,"PFC_21",51,"OID30167","O95670","ATP6V1G2",0.9773,"Cardiometabolic_II","B13805","20212097_SS211647","PASS",0.1989,0.0638,"Plate control","PASS","PFC",1.0452151925684,"24h","24h"
3178,"PFC_06",61,"OID30167","O95670","ATP6V1G2",0.9773,"Cardiometabolic_II","B13805","20212097_SS211647","PASS",0.1989,0.1102,"Plate control","PASS","PFC",1.07937785967714,"Baseline","Baseline"
3179,"PFC_14",62,"OID30167","O95670","ATP6V1G2",0.9773,"Cardiometabolic_II","B13805","20212097_SS211647","PASS",0.1989,-1.0533,"Plate control","PASS","PFC",0.481864692505592,"1h","1h"
3180,"PFC_22",63,"OID30167","O95670","ATP6V1G2",0.9773,"Cardiometabolic_II","B13805","20212097_SS211647","PASS",0.1989,-1.3188,"Plate control","PASS","PFC",0.400868232985002,"24h","24h"
3181,"PFC_07",73,"OID30167","O95670","ATP6V1G2",0.9773,"Cardiometabolic_II","B13805","20212097_SS211647","PASS",0.1989,-0.3152,"Plate control","PASS","PFC",0.803739561057482,"Baseline","Baseline"
3182,"PFC_15",74,"OID30167","O95670","ATP6V1G2",0.9773,"Cardiometabolic_II","B13805","20212097_SS211647","PASS",0.1989,-0.9412,"Plate control","PASS","PFC",0.520799511356899,"1h","1h"
3183,"PFC_23",75,"OID30167","O95670","ATP6V1G2",0.9773,"Cardiometabolic_II","B13805","20212097_SS211647","PASS",0.1989,-0.035,"Plate control","PASS","PFC",0.97603176077622
5,"24h","24h"
3184,"PFC_08",85,"OID30167","O95670","ATP6V1G2",0.9773,"Cardiometabolic_II","B13805","20212097_SS211647","PASS",0.1989,0.0972,"Plate control","PASS","PFC",1.06969536887275,"Baseline","Baseline"
3185,"PFC_16",86,"OID30167","O95670","ATP6V1G2",0.9773,"Cardiometabolic_II","B13805","20212097_SS211647","PASS",0.1989,-0.6771,"Plate control","PASS","PFC",0.625421187660882,"1h","1h"
3186,"PFC_24",87,"OID30167","O95670","ATP6V1G2",0.9773,"Cardiometabolic_II","B13805","20212097_SS211647","PASS",0.1989,-0.0736,"Plate control","PASS","PFC",0.950263815427473,"24h","24h"
3187,"PFC_01",1,"OID30168","O00327","ARNTL",0.9545,"Cardiometabolic_II","B13805","20212097_SS211647","WARN",0.4836,-0.1594,"Plate control","PASS","PFC",0.895397378804519,"Baseline","Baseline"
3188,"PFC_09",2,"OID30168","O00327","ARNTL",0.9545,"Cardiometabolic_II","B13805","20212097_SS211647","PASS",0.4836,-0.2187,"Plate control","PASS","PFC",0.859339430980341,"Baseline","Baseline"
3189,"PFC_17",3,"OID30168","O00327","ARNTL",0.9545,"Cardiometabolic_II","B13805","20212097_SS211647","PASS",0.4836,0.0403,"Plate control","PASS","PFC",1.0283276391459,"1h","1h"
3190,"PFC_25",4,"OID30168","O00327","ARNTL",0.9545,"Cardiometabolic_II","B13805","20212097_SS211647","PASS",0.4836,0.1535,"Plate control","PASS","PFC",1.11226457225211,"24h","24h"
3191,"PFC_02",13,"OID30168","O00327","ARNTL",0.9545,"Cardiometabolic_II","B13805","20212097_SS211647","PASS",0.4836,0.3957,"Plate control","PASS","PFC",1.3155809292201,"Baseline","Baseline"
3192,"PFC_10",14,"OID30168","O00327","ARNTL",0.9545,"Cardiometabolic_II","B13805","20212097_SS211647","PASS",0.4836,0.0547,"Plate control","PASS","PFC",1.03864310107911,"1h","1h"
3193,"PFC_18",15,"OID30168","O00327","ARNTL",0.9545,"Cardiometabolic_II","B13805","20212097_SS211647","PASS",0.4836,0.168,"Plate control","PASS","PFC",1.12349990303165,"1h","1h"
3194,"PFC_26",16,"OID30168","O00327","ARNTL",0.9545,"Cardiometabolic_II","B13805","20212097_SS211647","PASS",0.4836,0.0791,"Plate control","PASS","PFC",1.0563588440297,"24h","24h"
3195,"PFC_03",25,"OID30168","O00327","ARNTL",0.9545,"Cardiometabolic_II","B13805","20212097_SS211647","PASS",0.4836,0.2947,"Plate control","PASS","PFC",1.22662988021884,"Baseline","Baseline"
3196,"PFC_11",26,"OID30168","O00327","ARNTL",0.9545,"Cardiometabolic_II","B13805","20212097_SS211647","PASS",0.4836,0.1661,"Plate control","PASS","PFC",1.12202125042178,"1h","1h"
3197,"PFC_19",27,"OID30168","O00327","ARNTL",0.9545,"Cardiometabolic_II","B13805","20212097_SS211647","PASS",0.4836,0.0817,"Plate control","PASS","PFC",1.05826431211619,"24h","24h"
3198,"PFC_27",28,"OID30168","O00327","ARNTL",0.9545,"Cardiometabolic_II","B13805","20212097_SS211647","PASS",0.4836,0.2923,"Plate control","PASS","PFC",1.2245910124682,"24h","24h"
3199,"PFC_04",37,"OID30168","O00327","ARNTL",0.9545,"Cardiometabolic_II","B13805","20212097_SS211647","PASS",0.4836,0.1432,"Plate control","PASS","PFC",1.10435193223743,"Baseline","Baseline"
3200,"PFC_12",38,"OID30168","O00327","ARNTL",0.9545,"Cardiometabolic_II","B13805","20212097_SS211647","PASS",0.4836,0.1542,"Plate control","PASS","PFC",1.11280437733566,"1h","1h"
3201,"PFC_20",39,"OID30168","O00327","ARNTL",0.9545,"Cardiometabolic_II","B13805","20212097_SS211647","PASS",0.4836,-0.0328,"Plate control","PASS","PFC",0.977521270240659,"24h","24h"
3202,"PFC_05",49,"OID30168","O00327","ARNTL",0.9545,"Cardiometabolic_II","B13805","20212097_SS211647","PASS",0.4836,-0.2426,"Plate control","PASS","PFC",0.845220696865603,"Baseline","Baseline"
3203,"PFC_13",50,"OID30168","O00327","ARNTL",0.9545,"Cardiometabolic_II","B13805","20212097_SS211647","PASS",0.4836,-0.0269,"Plate control","PASS","PFC",0.981527095769553,"1h","1h"
3204,"PFC_21",51,"OID30168","O00327","ARNTL",0.9545,"Cardiometabolic_II","B13805","20212097_SS211647","PASS",0.4836,-0.1113,"Plate control","PASS","PFC",0.925753497482787,"24h","24h"
3205,"PFC_06",61,"OID30168","O00327","ARNTL",0.9545,"Cardiometabolic_II","B13805","20212097_SS211647","PASS",0.4836,-0.1249,"Plate control","PASS","PFC",0.91706760728432,"Baseline","Baseline"
3206,"PFC_14",62,"OID30168","O00327","ARNTL",0.9545,"Cardiometabolic_II","B13805","20212097_SS211647","PASS",0.4836,0.0086,"Plate control","PASS","PFC",1.00597886826167,"1h","1h"
3207,"PFC_22",63,"OID30168","O00327","ARNTL",0.9545,"Cardiometabolic_II","B13805","20212097_SS211647","PASS",0.4836,0.0502,"Plate control","PASS","PFC",1.03540845198238,"24h","24h"
3208,"PFC_07",73,"OID30168","O00327","ARNTL",0.9545,"Cardiometabolic_II","B13805","20212097_SS211647","PASS",0.4836,0.2697,"Plate control","PASS","PFC",1.20555711307017,"Baseline","Baseline"
3209,"PFC_15",74,"OID30168","O00327","ARNTL",0.9545,"Cardiometabolic_II","B13805","20212097_SS211647","PASS",0.4836,-0.0464,"Plate control","PASS","PFC",0.968349668466469,"1h","1h"
3210,"PFC_23",75,"OID30168","O00327","ARNTL",0.9545,"Cardiometabolic_II","B13805","20212097_SS211647","PASS",0.4836,0.1455,"Plate control","PASS","PFC",1.1061139367768,"24h","24h"
3211,"PFC_08",85,"OID30168","O00327","ARNTL",0.9545,"Cardiometabolic_II","B13805","20212097_SS211647","PASS",0.4836,0.3958,"Plate control","PASS","PFC",1.31567212150174,"Baseline","Baseline"
3212,"PFC_16",86,"OID30168","O00327","ARNTL",0.9545,"Cardiometabolic_II","B13805","20212097_SS211647","PASS",0.4836,-0.0683,"Plate control","PASS","PFC",0.953761200859253,"1h","1h"
3213,"PFC_24",87,"OID30168","O00327","ARNTL",0.9545,"Cardiometabolic_II","B13805","20212097_SS211647","PASS",0.4836,0.158,"Plate control","PASS","PFC",1.11573932232496,"24h","24h"
3214,"PFC_01",1,"OID30169","P48668","KRT6C",1,"Cardiometabolic_II","B13805","20212097_SS211647","WARN",0.9553,-0.7064,"Plate control","PASS","PFC",0.612847489065184,"Baseline","Baseline"
3215,"PFC_09",2,"OID30169","P48668","KRT6C",1,"Cardiometabolic_II","B13805","20212097_SS211647","PASS",0.9553,0.1262,"Plate control","PASS","PFC",1.09141516883737,"Baseline","Baseline"
3216,"PFC_17",3,"OID30169","P48668","KRT6C",1,"Cardiometabolic_II","B13805","20212097_SS211647","PASS",0.9553,-0.4682,"Plate control","PASS","PFC",0.722865929830253,"1h","1h"
3217,"PFC_25",4,"OID30169","P48668","KRT6C",1,"Cardiometabolic_II","B13805","20212097_SS211647","PASS",0.9553,-0.0768,"Plate control","PASS","PFC",0.948158398682309,"24h","24h"
3218,"PFC_02",13,"OID30169","P48668","KRT6C",1,"Cardiometabolic_II","B13805","20212097_SS211647","PASS",0.9553,-0.2303,"Plate control","PASS","PFC",0.852457609759591,"Baseline","Baseline"
3219,"PFC_10",14,"OID30169","P48668","KRT6C",1,"Cardiometabolic_II","B13805","20212097_SS211647","PASS",0.9553,0.3866,"Plate control","PASS","PFC",1.30730883531075,"1h","1h"
3220,"PFC_18",15,"OID30169","P48668","KRT6C",1,"Cardiometabolic_II","B13805","20212097_SS211647","PASS",0.9553,-0.012,"Plate control","PASS","PFC",0.991716730738293,"1h","1h"
3221,"PFC_26",16,"OID30169","P48668","KRT6C",1,"Cardiometabolic_II","B13805","20212097_SS211647","PASS",0.9553,-0.128,"Plate control","PASS","PFC",0.915099168127928,"24h","24h"
3222,"PFC_03",25,"OID30169","P48668","KRT6C",1,"Cardiometabolic_II","B13805","20212097_SS211647","PASS",0.9553,-0.2055,"Plate control","PASS","PFC",0.867238073249983,"Baseline","Baseline"
3223,"PFC_11",26,"OID30169","P48668","KRT6C",1,"Cardiometabolic_II","B13805","20212097_SS211647","PASS",0.9553,0.3685,"Plate control","PASS","PFC",1.29100984284336,"1h","1h"
3224,"PFC_19",27,"OID30169","P48668","KRT6C",1,"Cardiometabolic_II","B13805","20212097_SS211647","PASS",0.9553,-0.2149,"Plate control","PASS","PFC",0.86160587961088,"24h","24h"
3225,"PFC_27",28,"OID30169","P48668","KRT6C",1,"Cardiometabolic_II","B13805","20212097_SS211647","PASS",0.9553,0.0507,"Plate control","PASS","PFC",1.03576735939731,"24h","24h"
3226,"PFC_04",37,"OID30169","P48668","KRT6C",1,"Cardiometabolic_II","B13805","20212097_SS211647","PASS",0.9553,0.118,"Plate control","PASS","PFC",1.08522937188181,"Baseline","Baseline"
3227,"PFC_12",38,"OID30169","P48668","KRT6C",1,"Cardiometabolic_II","B13805","20212097_SS211647","PASS",0.9553,-0.0801,"Plate control","PASS","PFC",0.945992073279177,"1h","1h"
3228,"PFC_20",39,"OID30169","P48668","KRT6C",1,"Cardiometabolic_II","B13805","20212097_SS211647","PASS",0.9553,-0.0161,"Plate control","PASS","PFC",0.988902368516412,"24h","24h"
3229,"PFC_05",49,"OID30169","P48668","KRT6C",1,"Cardiometabolic_II","B13805","20212097_SS211647","PASS",0.9553,-0.2909,"Plate control","PASS","PFC",0.817391983819636,"Baseline","Baseline"
3230,"PFC_13",50,"OID30169","P48668","KRT6C",1,"Cardiometabolic_II","B13805","20212097_SS211647","PASS",0.9553,-0.1966,"Plate control","PASS","PFC",0.872604609604173,"1h","1h"
3231,"PFC_21",51,"OID30169","P48668","KRT6C",1,"Cardiometabolic_II","B13805","20212097_SS211647","PASS",0.9553,0.2326,"Plate control","PASS","PFC",1.17495051779943,"24h","24h"
3232,"PFC_06",61,"OID30169","P48668","KRT6C",1,"Cardiometabolic_II","B13805","20212097_SS211647","PASS",0.9553,0.483,"Plate control","PASS","PFC",1.39764697185107,"Baseline","Baseline"
3233,"PFC_14",62,"OID30169","P48668","KRT6C",1,"Cardiometabolic_II","B13805","20212097_SS211647","PASS",0.9553,-0.188,"Plate control","PASS","PFC",0.877821797609519,"1h","1h"
3234,"PFC_22",63,"OID30169","P48668","KRT6C",1,"Cardiometabolic_II","B13805","20212097_SS211647","PASS",0.9553,-0.414,"Plate control","PASS","PFC",0.750539548757186,"24h","24h"
3235,"PFC_07",73,"OID30169","P48668","KRT6C",1,"Cardiometabolic_II","B13805","20212097_SS211647","PASS",0.9553,-0.2043,"Plate control","PASS","PFC",0.867959721683941,"Baseline","Baseline"
3236,"PFC_15",74,"OID30169","P48668","KRT6C",1,"Cardiometabolic_II","B13805","20212097_SS211647","PASS",0.9553,-5e-04,"Plate control","PASS","PFC",0.999653486459409,"1h","1h"
3237,"PFC_23",75,"OID30169","P48668","KRT6C",1,"Cardiometabolic_II","B13805","20212097_SS211647","PASS",0.9553,-0.057,"Plate control","PASS","PFC",0.961260928388761,"24h","24h"
3238,"PFC_08",85,"OID30169","P48668","KRT6C",1,"Cardiometabolic_II","B13805","20212097_SS211647","PASS",0.9553,-0.2168,"Plate control","PASS","PFC",0.860471909079041,"Baseline","Baseline"
3239,"PFC_16",86,"OID30169","P48668","KRT6C",1,"Cardiometabolic_II","B13805","20212097_SS211647","PASS",0.9553,0.0517,"Plate control","PASS","PFC",1.03648554749847,"1h","1h"
3240,"PFC_24",87,"OID30169","P48668","KRT6C",1,"Cardiometabolic_II","B13805","20212097_SS211647","PASS",0.9553,0.1594,"Plate control","PASS","PFC",1.11682256802576,"24h","24h"
3241,"PFC_01",1,"OID30170","P00966","ASS1",0.0227,"Cardiometabolic_II","B13805","20212097_SS211647","WARN",-2.0526,0.2852,"Plate control","PASS","PFC",1.21857918291132,"Baseline","Baseline"
3242,"PFC_09",2,"OID30170","P00966","ASS1",0.0227,"Cardiometabolic_II","B13805","20212097_SS211647","PASS",-2.0526,-0.2796,"Plate control","PASS","PFC",0.823819396842863,"Baseline","Baseline"
3243,"PFC_17",3,"OID30170","P00966","ASS1",0.0227,"Cardiometabolic_II","B13805","20212097_SS211647","PASS",-2.0526,-0.5438,"Plate control","PASS","PFC",0.685961732192284,"1h","1h"
3244,"PFC_25",4,"OID30170","P00966","ASS1",0.0227,"Cardiometabolic_II","B13805","20212097_SS211647","PASS",-2.0526,-0.9867,"Plate control","PASS","PFC",0.504630741025395,"24h","24h"
3245,"PFC_02",13,"OID30170","P00966","ASS1",0.0227,"Cardiometabolic_II","B13805","20212097_SS211647","PASS",-2.0526,0.0838,"Plate control","PASS","PFC",1.05980585292529,"Baseline","Baseline"
3246,"PFC_10",14,"OID30170","P00966","ASS1",0.0227,"Cardiometabolic_II","B13805","20212097_SS211647","PASS",-2.0526,0.0199,"Plate control","PASS","PFC",1.01388919991031,"1h","1h"
3247,"PFC_18",15,"OID30170","P00966","ASS1",0.0227,"Cardiometabolic_II","B13805","20212097_SS211647","PASS",-2.0526,-1.1907,"Plate control","PASS","PFC",0.43809024636827,"1h","1h"
3248,"PFC_26",16,"OID30170","P00966","ASS1",0.0227,"Cardiometabolic_II","B13805","20212097_SS211647","PASS",-2.0526,-0.5956,"Plate control","PASS","PFC",0.661769175895873,"24h","24h"

Supplemental table 1_Olink proximity extension assay (PEA) data

**,"SampleID","Index","OlinkID","UniProt","Assay","MissingFreq","Panel","Panel_Lot_Nr","PlateID","QC_Warning","LOD","NPX","Normalization","Assay_Warning","condition","Value","group","alt_group"**
3249,"PFC_03",25,"OID30170","P00966","ASS1",0.0227,"Cardiometabolic_II","B13805","20212097_SS211647","PASS",-2.0526,0.4063,"Plate control","PASS","PFC",1.32528257315778,"Baseline","Baseline"
3250,"PFC_11",26,"OID30170","P00966","ASS1",0.0227,"Cardiometabolic_II","B13805","20212097_SS211647","PASS",-2.0526,-0.4017,"Plate control","PASS","PFC",0.756965786730287,"1h","1h"
3251,"PFC_19",27,"OID30170","P00966","ASS1",0.0227,"Cardiometabolic_II","B13805","20212097_SS211647","PASS",-2.0526,0.2336,"Plate control","PASS","PFC",1.17576521375763,"24h","24h"
3252,"PFC_27",28,"OID30170","P00966","ASS1",0.0227,"Cardiometabolic_II","B13805","20212097_SS211647","PASS",-2.0526,0.048,"Plate control","PASS","PFC",1.03383073624796,"24h","24h"
3253,"PFC_04",37,"OID30170","P00966","ASS1",0.0227,"Cardiometabolic_II","B13805","20212097_SS211647","PASS",-2.0526,-0.1401,"Plate control","PASS","PFC",0.907456253062831,"Baseline","Baseline"
3254,"PFC_12",38,"OID30170","P00966","ASS1",0.0227,"Cardiometabolic_II","B13805","20212097_SS211647","PASS",-2.0526,-0.9223,"Plate control","PASS","PFC",0.52767122116435,"1h","1h"
3255,"PFC_20",39,"OID30170","P00966","ASS1",0.0227,"Cardiometabolic_II","B13805","20212097_SS211647","PASS",-2.0526,-0.5329,"Plate control","PASS","PFC",0.691164009442416,"24h","24h"
3256,"PFC_05",49,"OID30170","P00966","ASS1",0.0227,"Cardiometabolic_II","B13805","20212097_SS211647","PASS",-2.0526,0.0489,"Plate control","PASS","PFC",1.03447587463018,"Baseline","Baseline"
3257,"PFC_13",50,"OID30170","P00966","ASS1",0.0227,"Cardiometabolic_II","B13805","20212097_SS211647","PASS",-2.0526,-0.5849,"Plate control","PASS","PFC",0.666695548758048,"1h","1h"
3258,"PFC_21",51,"OID30170","P00966","ASS1",0.0227,"Cardiometabolic_II","B13805","20212097_SS211647","PASS",-2.0526,-1.0365,"Plate control","PASS","PFC",0.487508743826667,"24h","24h"
3259,"PFC_06",61,"OID30170","P00966","ASS1",0.0227,"Cardiometabolic_II","B13805","20212097_SS211647","PASS",-2.0526,-0.0161,"Plate control","PASS","PFC",0.988902368516412,"Baseline","Baseline"
3260,"PFC_14",62,"OID30170","P00966","ASS1",0.0227,"Cardiometabolic_II","B13805","20212097_SS211647","PASS",-2.0526,-0.4387,"Plate control","PASS","PFC",0.737799133607807,"1h","1h"
3261,"PFC_22",63,"OID30170","P00966","ASS1",0.0227,"Cardiometabolic_II","B13805","20212097_SS211647","PASS",-2.0526,-0.9991,"Plate control","PASS","PFC",0.500312013543222,"24h","24h"
3262,"PFC_07",73,"OID30170","P00966","ASS1",0.0227,"Cardiometabolic_II","B13805","20212097_SS211647","PASS",-2.0526,-0.4493,"Plate control","PASS","PFC",0.732398123561523,"Baseline","Baseline"
3263,"PFC_15",74,"OID30170","P00966","ASS1",0.0227,"Cardiometabolic_II","B13805","20212097_SS211647","PASS",-2.0526,-1.0571,"Plate control","PASS","PFC",0.480597150583841,"1h","1h"
3264,"PFC_23",75,"OID30170","P00966","ASS1",0.0227,"Cardiometabolic_II","B13805","20212097_SS211647","PASS",-2.0526,0.1428,"Plate control","PASS","PFC",1.10404578330938,"24h","24h"
3265,"PFC_08",85,"OID30170","P00966","ASS1",0.0227,"Cardiometabolic_II","B13805","20212097_SS211647","PASS",-2.0526,-1.052,"Plate control","PASS","PFC",0.482299092292069,"Baseline","Baseline"
3266,"PFC_16",86,"OID30170","P00966","ASS1",0.0227,"Cardiometabolic_II","B13805","20212097_SS211647","PASS",-2.0526,-0.9527,"Plate control","PASS","PFC",0.516664620059759,"1h","1h"
3267,"PFC_24",87,"OID30170","P00966","ASS1",0.0227,"Cardiometabolic_II","B13805","20212097_SS211647","PASS",-2.0526,-0.4219,"Plate control","PASS","PFC",0.746440929197922,"24h","24h"
3268,"PFC_01",1,"OID30171","Q96PU4","UHRF2",0.9545,"Cardiometabolic_II","B13805","20212097_SS211647","WARN",0.7053,0.491,"Plate control","PASS","PFC",1.40541870020672,"Baseline","Baseline"
3269,"PFC_09",2,"OID30171","Q96PU4","UHRF2",0.9545,"Cardiometabolic_II","B13805","20212097_SS211647","PASS",0.7053,-0.3846,"Plate control","PASS","PFC",0.765991347005088,"Baseline","Baseline"
3270,"PFC_17",3,"OID30171","Q96PU4","UHRF2",0.9545,"Cardiometabolic_II","B13805","20212097_SS211647","PASS",0.7053,-0.0565,"Plate control","PASS","PFC",0.961594133776667,"1h","1h"
3271,"PFC_25",4,"OID30171","Q96PU4","UHRF2",0.9545,"Cardiometabolic_II","B13805","20212097_SS211647","PASS",0.7053,0.0129,"Plate control","PASS","PFC",1.00898169413913,"24h","24h"
3272,"PFC_02",13,"OID30171","Q96PU4","UHRF2",0.9545,"Cardiometabolic_II","B13805","20212097_SS211647","PASS",0.7053,-0.268,"Plate control","PASS","PFC",0.830470024090894,"Baseline","Baseline"
3273,"PFC_10",14,"OID30171","Q96PU4","UHRF2",0.9545,"Cardiometabolic_II","B13805","20212097_SS211647","PASS",0.7053,0.0661,"Plate control","PASS","PFC",1.0468828438489,"1h","1h"
3274,"PFC_18",15,"OID30171","Q96PU4","UHRF2",0.9545,"Cardiometabolic_II","B13805","20212097_SS211647","PASS",0.7053,-0.4394,"Plate control","PASS","PFC",0.737441238068332,"1h","1h"
3275,"PFC_26",16,"OID30171","Q96PU4","UHRF2",0.9545,"Cardiometabolic_II","B13805","20212097_SS211647","PASS",0.7053,0.2467,"Plate control","PASS","PFC",1.18649004833285,"24h","24h"
3276,"PFC_03",25,"OID30171","Q96PU4","UHRF2",0.9545,"Cardiometabolic_II","B13805","20212097_SS211647","PASS",0.7053,-0.1819,"Plate control","PASS","PFC",0.881541259573385,"Baseline","Baseline"
3277,"PFC_11",26,"OID30171","Q96PU4","UHRF2",0.9545,"Cardiometabolic_II","B13805","20212097_SS211647","PASS",0.7053,0.2713,"Plate control","PASS","PFC",1.20689486035995,"1h","1h"
3278,"PFC_19",27,"OID30171","Q96PU4","UHRF2",0.9545,"Cardiometabolic_II","B13805","20212097_SS211647","PASS",0.7053,0.2011,"Plate control","PASS","PFC",1.14957452770743,"24h","24h"
3279,"PFC_27",28,"OID30171","Q96PU4","UHRF2",0.9545,"Cardiometabolic_II","B13805","20212097_SS211647","PASS",0.7053,0.6628,"Plate control","PASS","PFC",1.58315224502623,"24h","24h"
3280,"PFC_04",37,"OID30171","Q96PU4","UHRF2",0.9545,"Cardiometabolic_II","B13805","20212097_SS211647","PASS",0.7053,0.202,"Plate control","PASS","PFC",1.15029189335061,"Baseline","Baseline"
3281,"PFC_12",38,"OID30171","Q96PU4","UHRF2",0.9545,"Cardiometabolic_II","B13805","20212097_SS211647","PASS",0.7053,0.5277,"Plate control","PASS","PFC",1.44162906195358,"1h","1h"
3282,"PFC_20",39,"OID30171","Q96PU4","UHRF2",0.9545,"Cardiometabolic_II","B13805","20212097_SS211647","PASS",0.7053,0.4559,"Plate control","PASS","PFC",1.37163821057964,"24h","24h"
3283,"PFC_05",49,"OID30171","Q96PU4","UHRF2",0.9545,"Cardiometabolic_II","B13805","20212097_SS211647","PASS",0.7053,-0.3457,"Plate control","PASS","PFC",0.786926065016083,"Baseline","Baseline"
3284,"PFC_13",50,"OID30171","Q96PU4","UHRF2",0.9545,"Cardiometabolic_II","B13805","20212097_SS211647","PASS",0.7053,0.302,"Plate control","PASS","PFC",1.23285232546381,"1h","1h"
3285,"PFC_21",51,"OID30171","Q96PU4","UHRF2",0.9545,"Cardiometabolic_II","B13805","20212097_SS211647","PASS",0.7053,-0.348,"Plate control","PASS","PFC",0.78567251666758,"24h","24h"
3286,"PFC_06",61,"OID30171","Q96PU4","UHRF2",0.9545,"Cardiometabolic_II","B13805","20212097_SS211647","PASS",0.7053,-0.1461,"Plate control","PASS","PFC",0.903690085556642,"Baseline","Baseline"
3287,"PFC_14",62,"OID30171","Q96PU4","UHRF2",0.9545,"Cardiometabolic_II","B13805","20212097_SS211647","PASS",0.7053,-0.208,"Plate control","PASS","PFC",0.865736565519658,"1h","1h"
3288,"PFC_22",63,"OID30171","Q96PU4","UHRF2",0.9545,"Cardiometabolic_II","B13805","20212097_SS211647","PASS",0.7053,-0.1947,"Plate control","PASS","PFC",0.873754569181932,"24h","24h"
3289,"PFC_07",73,"OID30171","Q96PU4","UHRF2",0.9545,"Cardiometabolic_II","B13805","20212097_SS211647","PASS",0.7053,0.3766,"Plate control","PASS","PFC",1.29827859356564,"Baseline","Baseline"
3290,"PFC_15",74,"OID30171","Q96PU4","UHRF2",0.9545,"Cardiometabolic_II","B13805","20212097_SS211647","PASS",0.7053,0.2854,"Plate control","PASS","PFC",1.21874812556624,"1h","1h"
3291,"PFC_23",75,"OID30171","Q96PU4","UHRF2",0.9545,"Cardiometabolic_II","B13805","20212097_SS211647","PASS",0.7053,0.2918,"Plate control","PASS","PFC",1.22416667510069,"24h","24h"
3292,"PFC_08",85,"OID30171","Q96PU4","UHRF2",0.9545,"Cardiometabolic_II","B13805","20212097_SS211647","PASS",0.7053,-0.3284,"Plate control","PASS","PFC",0.796419251367939,"Baseline","Baseline"
3293,"PFC_16",86,"OID30171","Q96PU4","UHRF2",0.9545,"Cardiometabolic_II","B13805","20212097_SS211647","PASS",0.7053,-2e-04,"Plate control","PASS","PFC",0.999861380172504,"1h","1h"
3294,"PFC_24",87,"OID30171","Q96PU4","UHRF2",0.9545,"Cardiometabolic_II","B13805","20212097_SS211647","PASS",0.7053,-0.6238,"Plate control","PASS","PFC",0.648959342101646,"24h","24h"
3295,"PFC_01",1,"OID30172","P20382","PMCH",0.4091,"Cardiometabolic_II","B13805","20212097_SS211647","WARN",-1.9689,-1.255,"Plate control","PASS","PFC",0.418993567333974,"Baseline","Baseline"
3296,"PFC_09",2,"OID30172","P20382","PMCH",0.4091,"Cardiometabolic_II","B13805","20212097_SS211647","PASS",-1.9689,-2.0399,"Plate control","PASS","PFC",0.24318059226309,"Baseline","Baseline"
3297,"PFC_17",3,"OID30172","P20382","PMCH",0.4091,"Cardiometabolic_II","B13805","20212097_SS211647","PASS",-1.9689,0.7008,"Plate control","PASS","PFC",1.62540585925157,"1h","1h"
3298,"PFC_25",4,"OID30172","P20382","PMCH",0.4091,"Cardiometabolic_II","B13805","20212097_SS211647","PASS",-1.9689,-1.3259,"Plate control","PASS","PFC",0.398900268605925,"24h","24h"
3299,"PFC_02",13,"OID30172","P20382","PMCH",0.4091,"Cardiometabolic_II","B13805","20212097_SS211647","PASS",-1.9689,-2.2579,"Plate control","PASS","PFC",0.209076091526607,"Baseline","Baseline"
3300,"PFC_10",14,"OID30172","P20382","PMCH",0.4091,"Cardiometabolic_II","B13805","20212097_SS211647","PASS",-1.9689,-1.4852,"Plate control","PASS","PFC",0.357199013204987,"1h","1h"
3301,"PFC_18",15,"OID30172","P20382","PMCH",0.4091,"Cardiometabolic_II","B13805","20212097_SS211647","PASS",-1.9689,-2.4066,"Plate control","PASS","PFC",0.188599793303418,"1h","1h"
3302,"PFC_26",16,"OID30172","P20382","PMCH",0.4091,"Cardiometabolic_II","B13805","20212097_SS211647","PASS",-1.9689,0.3829,"Plate control","PASS","PFC",1.30396034848212,"24h","24h"
3303,"PFC_03",25,"OID30172","P20382","PMCH",0.4091,"Cardiometabolic_II","B13805","20212097_SS211647","PASS",-1.9689,-1.8044,"Plate control","PASS","PFC",0.286300084251146,"Baseline","Baseline"
3304,"PFC_11",26,"OID30172","P20382","PMCH",0.4091,"Cardiometabolic_II","B13805","20212097_SS211647","PASS",-1.9689,-2.0063,"Plate control","PASS","PFC",0.248910673372259,"1h","1h"
3305,"PFC_19",27,"OID30172","P20382","PMCH",0.4091,"Cardiometabolic_II","B13805","20212097_SS211647","PASS",-1.9689,-1.0142,"Plate control","PASS","PFC",0.495102795387478,"24h","24h"
3306,"PFC_27",28,"OID30172","P20382","PMCH",0.4091,"Cardiometabolic_II","B13805","20212097_SS211647","PASS",-1.9689,-1.7784,"Plate control","PASS","PFC",0.291506508475881,"24h","24h"
3307,"PFC_04",37,"OID30172","P20382","PMCH",0.4091,"Cardiometabolic_II","B13805","20212097_SS211647","PASS",-1.9689,-1.6663,"Plate control","PASS","PFC",0.315060326130519,"Baseline","Baseline"
3308,"PFC_12",38,"OID30172","P20382","PMCH",0.4091,"Cardiometabolic_II","B13805","20212097_SS211647","PASS",-1.9689,-2.2418,"Plate control","PASS","PFC",0.21142237917811,"1h","1h"
3309,"PFC_20",39,"OID30172","P20382","PMCH",0.4091,"Cardiometabolic_II","B13805","20212097_SS211647","PASS",-1.9689,-2.1602,"Plate control","PASS","PFC",0.223725250676759,"24h","24h"
3310,"PFC_05",49,"OID30172","P20382","PMCH",0.4091,"Cardiometabolic_II","B13805","20212097_SS211647","PASS",-1.9689,0.0244,"Plate control","PASS","PFC",1.01705662217579,"Baseline","Baseline"
3311,"PFC_13",50,"OID30172","P20382","PMCH",0.4091,"Cardiometabolic_II","B13805","20212097_SS211647","PASS",-1.9689,-1.9595,"Plate control","PASS","PFC",0.257117551377092,"1h","1h"
3312,"PFC_21",51,"OID30172","P20382","PMCH",0.4091,"Cardiometabolic_II","B13805","20212097_SS211647","PASS",-1.9689,-2.3944,"Plate control","PASS","PFC",0.190201430259567,"24h","24h"
3313,"PFC_06",61,"OID30172","P20382","PMCH",0.4091,"Cardiometabolic_II","B13805","20212097_SS211647","PASS",-1.9689,-2.1052,"Plate control","PASS","PFC",0.232419013303103,"Baseline","Baseline"
3314,"PFC_14",62,"OID30172","P20382","PMCH",0.4091,"Cardiometabolic_II","B13805","20212097_SS211647","PASS",-1.9689,0.4461,"Plate control","PASS","PFC",1.36235246244466,"1h","1h"
3315,"PFC_22",63,"OID30172","P20382","PMCH",0.4091,"Cardiometabolic_II","B13805","20212097_SS211647","PASS",-1.9689,-2.5718,"Plate control","PASS","PFC",0.168194216118452,"24h","24h"
3316,"PFC_07",73,"OID30172","P20382","PMCH",0.4091,"Cardiometabolic_II","B13805","20212097_SS211647","PASS",-1.9689,-1.2243,"Plate control","PASS","PFC",0.428005131853828,"Baseline","Baseline"
3317,"PFC_15",74,"OID30172","P20382","PMCH",0.4091,"Cardiometabolic_II","B13805","20212097_SS211647","PASS",-1.9689,-2.2422,"Plate control","PASS","PFC",0.211363768573226,"1h","1h"
3318,"PFC_23",75,"OID30172","P20382","PMCH",0.4091,"Cardiometabolic_II","B13805","20212097_SS211647","PASS",-1.9689,0.4487,"Plate control","PASS","PFC",1.36480988413845,"24h","24h"
3319,"PFC_08",85,"OID30172","P20382","PMCH",0.4091,"Cardiometabolic_II","B13805","20212097_SS211647","PASS",-1.9689,0.5394,"Plate control","PASS","PFC",1.45336795287407,"Baseline","Baseline"
3320,"PFC_16",86,"OID30172","P20382","PMCH",0.4091,"Cardiometabolic_II","B13805","20212097_SS211647","PASS",-1.9689,-1.9227,"Plate control","PASS","PFC",0.263760421002457,"1h","1h"
3321,"PFC_24",87,"OID30172","P20382","PMCH",0.4091,"Cardiometabolic_II","B13805","20212097_SS211647","PASS",-1.9689,-1.6695,"Plate control","PASS","PFC",0.314362274415193,"24h","24h"
3322,"PFC_01",1,"OID30173","P35606","COPB2",0.0682,"Cardiometabolic_II","B13805","20212097_SS211647","WARN",0.3351,0.7984,"Plate control","PASS","PFC",1.7391712539995,"Baseline","Baseline"
3323,"PFC_09",2,"OID30173","P35606","COPB2",0.0682,"Cardiometabolic_II","B13805","20212097_SS211647","PASS",0.3351,0.8615,"Plate control","PASS","PFC",1.81692642505156,"Baseline","Baseline"
3324,"PFC_17",3,"OID30173","P35606","COPB2",0.0682,"Cardiometabolic_II","B13805","20212097_SS211647","PASS",0.3351,0.9315,"Plate control","PASS","PFC",1.90725798129224,"1h","1h"
3325,"PFC_25",4,"OID30173","P35606","COPB2",0.0682,"Cardiometabolic_II","B13805","20212097_SS211647","PASS",0.3351,1.5155,"Plate control","PASS","PFC",2.85897895440215,"24h","24h"
3326,"PFC_02",13,"OID30173","P35606","COPB2",0.0682,"Cardiometabolic_II","B13805","20212097_SS211647","PASS",0.3351,0.9004,"Plate control","PASS","PFC",1.86658343815477,"Baseline","Baseline"
3327,"PFC_10",14,"OID30173","P35606","COPB2",0.0682,"Cardiometabolic_II","B13805","20212097_SS211647","PASS",0.3351,0.9378,"Plate control","PASS","PFC",1.91560485881599,"1h","1h"
3328,"PFC_18",15,"OID30173","P35606","COPB2",0.0682,"Cardiometabolic_II","B13805","20212097_SS211647","PASS",0.3351,0.8286,"Plate control","PASS","PFC",1.77596112243931,"1h","1h"
3329,"PFC_26",16,"OID30173","P35606","COPB2",0.0682,"Cardiometabolic_II","B13805","20212097_SS211647","PASS",0.3351,1.2554,"Plate control","PASS","PFC",2.3873333036512,"24h","24h"
3330,"PFC_03",25,"OID30173","P35606","COPB2",0.0682,"Cardiometabolic_II","B13805","20212097_SS211647","PASS",0.3351,0.4445,"Plate control","PASS","PFC",1.36084240272511,"Baseline","Baseline"
3331,"PFC_11",26,"OID30173","P35606","COPB2",0.0682,"Cardiometabolic_II","B13805","20212097_SS211647","PASS",0.3351,1.9134,"Plate control","PASS","PFC",3.76695813595498,"1h","1h"
3332,"PFC_19",27,"OID30173","P35606","COPB2",0.0682,"Cardiometabolic_II","B13805","20212097_SS211647","PASS",0.3351,1.1184,"Plate control","PASS","PFC",2.1710606061391,"24h","24h"
3333,"PFC_27",28,"OID30173","P35606","COPB2",0.0682,"Cardiometabolic_II","B13805","20212097_SS211647","PASS",0.3351,1.7718,"Plate control","PASS","PFC",3.41479743330785,"24h","24h"
3334,"PFC_04",37,"OID30173","P35606","COPB2",0.0682,"Cardiometabolic_II","B13805","20212097_SS211647","PASS",0.3351,1.7784,"Plate control","PASS","PFC",3.43045513881807,"Baseline","Baseline"
3335,"PFC_12",38,"OID30173","P35606","COPB2",0.0682,"Cardiometabolic_II","B13805","20212097_SS211647","PASS",0.3351,0.7072,"Plate control","PASS","PFC",1.6326324075245,"1h","1h"
3336,"PFC_20",39,"OID30173","P35606","COPB2",0.0682,"Cardiometabolic_II","B13805","20212097_SS211647","PASS",0.3351,1.7558,"Plate control","PASS","PFC",3.37713534687372,"24h","24h"
3337,"PFC_05",49,"OID30173","P35606","COPB2",0.0682,"Cardiometabolic_II","B13805","20212097_SS211647","PASS",0.3351,0.565,"Plate control","PASS","PFC",1.47938750924884,"Baseline","Baseline"
3338,"PFC_13",50,"OID30173","P35606","COPB2",0.0682,"Cardiometabolic_II","B13805","20212097_SS211647","PASS",0.3351,1.1017,"Plate control","PASS","PFC",2.14607426278738,"1h","1h"
3339,"PFC_21",51,"OID30173","P35606","COPB2",0.0682,"Cardiometabolic_II","B13805","20212097_SS211647","PASS",0.3351,0.3647,"Plate control","PASS","PFC",1.28761384989628,"24h","24h"
3340,"PFC_06",61,"OID30173","P35606","COPB2",0.0682,"Cardiometabolic_II","B13805","20212097_SS211647","PASS",0.3351,1.0319,"Plate control","PASS","PFC",2.04471532744136,"Baseline","Baseline"
3341,"PFC_14",62,"OID30173","P35606","COPB2",0.0682,"Cardiometabolic_II","B13805","20212097_SS211647","PASS",0.3351,1.3949,"Plate control","PASS","PFC",2.62970323695431,"1h","1h"
3342,"PFC_22",63,"OID30173","P35606","COPB2",0.0682,"Cardiometabolic_II","B13805","20212097_SS211647","PASS",0.3351,1.3805,"Plate control","PASS","PFC",2.60358588864837,"24h","24h"
3343,"PFC_07",73,"OID30173","P35606","COPB2",0.0682,"Cardiometabolic_II","B13805","20212097_SS211647","PASS",0.3351,1.7369,"Plate control","PASS","PFC",3.33318178207405,"Baseline","Baseline"
3344,"PFC_15",74,"OID30173","P35606","COPB2",0.0682,"Cardiometabolic_II","B13805","20212097_SS211647","PASS",0.3351,0.7712,"Plate control","PASS","PFC",1.70668877712801,"1h","1h"
3345,"PFC_23",75,"OID30173","P35606","COPB2",0.0682,"Cardiometabolic_II","B13805","20212097_SS211647","PASS",0.3351,1.601,"Plate control","PASS","PFC",3.03353509074888,"24h","24h"
3346,"PFC_08",85,"OID30173","P35606","COPB2",0.0682,"Cardiometabolic_II","B13805","20212097_SS211647","PASS",0.3351,1.2152,"Plate control","PASS","PFC",2.32172966653331,"Baseline","Baseline"
3347,"PFC_16",86,"OID30173","P35606","COPB2",0.0682,"Cardiometabolic_II","B13805","20212097_SS211647","PASS",0.3351,1.5003,"Plate control","PASS","PFC",2.8290153407882,"1h","1h"
3348,"PFC_24",87,"OID30173","P35606","COPB2",0.0682,"Cardiometabolic_II","B13805","20212097_SS211647","PASS",0.3351,1.3477,"Plate control","PASS","PFC",2.54506058505221,"24h","24h"
3349,"PFC_01",1,"OID30174","P13224","GP1BB",0,"Cardiometabolic_II","B13805","20212097_SS211647","WARN",-0.7173,1.7525,"Plate control","PASS","PFC",3.36941936386717,"Baseline","Baseline"
3350,"PFC_09",2,"OID30174","P13224","GP1BB",0,"Cardiometabolic_II","B13805","20212097_SS211647","PASS",-0.7173,3.1677,"Plate control","PASS","PFC",8.98613041666735,"Baseline","Baseline"
3351,"PFC_17",3,"OID30174","P13224","GP1BB",0,"Cardiometabolic_II","B13805","20212097_SS211647","PASS",-0.7173,3.3826,"Plate control","PASS","PFC",10.4295138059244,"1h","1h"
3352,"PFC_25",4,"OID30174","P13224","GP1BB",0,"Cardiometabolic_II","B13805","20212097_SS211647","PASS",-0.7173,2.9685,"Plate control","PASS","PFC",7.82722002530267,"24h","24h"
3353,"PFC_02",13,"OID30174","P13224","GP1BB",0,"Cardiometabolic_II","B13805","20212097_SS211647","PASS",-0.7173,2.7978,"Plate control","PASS","PFC",6.95379241357654,"Baseline","Baseline"
3354,"PFC_10",14,"OID30174","P13224","GP1BB",0,"Cardiometabolic_II","B13805","20212097_SS211647","PASS",-0.7173,2.5056,"Plate control","PASS","PFC",5.67885470302922,"1h","1h"
3355,"PFC_18",15,"OID30174","P13224","GP1BB",0,"Cardiometabolic_II","B13805","20212097_SS211647","PASS",-0.7173,3.1474,"Plate control","PASS","PFC",8.86057300781804,"1h","1h"
3356,"PFC_26",16,"OID30174","P13224","GP1BB",0,"Cardiometabolic_II","B13805","20212097_SS211647","PASS",-0.7173,3.4537,"Plate control","PASS","PFC",10.9563852865557,"24h","24h"
3357,"PFC_03",25,"OID30174","P13224","GP1BB",0,"Cardiometabolic_II","B13805","20212097_SS211647","PASS",-0.7173,1.4408,"Plate control","PASS","PFC",2.71471359445816,"Baseline","Baseline"
3358,"PFC_11",26,"OID30174","P13224","GP1BB",0,"Cardiometabolic_II","B13805","20212097_SS211647","PASS",-0.7173,4.1912,"Plate control","PASS","PFC",18.2674075389102,"1h","1h"
3359,"PFC_19",27,"OID30174","P13224","GP1BB",0,"Cardiometabolic_II","B13805","20212097_SS211647","PASS",-0.7173,3.7734,"Plate control","PASS","PFC",13.6743466626279,"24h","24h"
3360,"PFC_27",28,"OID30174","P13224","GP1BB",0,"Cardiometabolic_II","B13805","20212097_SS211647","PASS",-0.7173,3.7718,"Plate control","PASS","PFC",13.6591897332314,"24h","24h"

Supplemental table 1_Olink proximity extension assay (PEA) data

,"SampleID","Index","OlinkID","UniProt","Assay","MissingFreq","Panel","Panel_Lot_Nr","PlateID","QC_Warning","LOD","NPX","Normalization","Assay_Warning","condition","Value","group","alt_group"
3361,"PFC_04",37,"OID30174","P13224","GP1BB",0,"Cardiometabolic_II","B13805","20212097_SS211647","PASS",-0.7173,3.3259,"Plate control","PASS","PFC",10.0275690813124,"Baseline","Baseline"
3362,"PFC_12",38,"OID30174","P13224","GP1BB",0,"Cardiometabolic_II","B13805","20212097_SS211647","PASS",-0.7173,1.7762,"Plate control","PASS","PFC",3.42522794269238,"1h","1h"
3363,"PFC_20",39,"OID30174","P13224","GP1BB",0,"Cardiometabolic_II","B13805","20212097_SS211647","PASS",-0.7173,4.7933,"Plate control","PASS","PFC",27.728544794136,"24h","24h"
3364,"PFC_05",49,"OID30174","P13224","GP1BB",0,"Cardiometabolic_II","B13805","20212097_SS211647","PASS",-0.7173,1.7214,"Plate control","PASS","PFC",3.29756249153626,"Baseline","Baseline"
3365,"PFC_13",50,"OID30174","P13224","GP1BB",0,"Cardiometabolic_II","B13805","20212097_SS211647","PASS",-0.7173,3.2933,"Plate control","PASS","PFC",9.80352102818427,"1h","1h"
3366,"PFC_21",51,"OID30174","P13224","GP1BB",0,"Cardiometabolic_II","B13805","20212097_SS211647","PASS",-0.7173,2.2962,"Plate control","PASS","PFC",4.91162358407419,"24h","24h"
3367,"PFC_06",61,"OID30174","P13224","GP1BB",0,"Cardiometabolic_II","B13805","20212097_SS211647","PASS",-0.7173,2.6446,"Plate control","PASS","PFC",6.25322314305973,"Baseline","Baseline"
3368,"PFC_14",62,"OID30174","P13224","GP1BB",0,"Cardiometabolic_II","B13805","20212097_SS211647","PASS",-0.7173,3.2413,"Plate control","PASS","PFC",9.45645858631897,"1h","1h"
3369,"PFC_22",63,"OID30174","P13224","GP1BB",0,"Cardiometabolic_II","B13805","20212097_SS211647","PASS",-0.7173,2.9348,"Plate control","PASS","PFC",7.64650243694977,"24h","24h"
3370,"PFC_07",73,"OID30174","P13224","GP1BB",0,"Cardiometabolic_II","B13805","20212097_SS211647","PASS",-0.7173,2.9766,"Plate control","PASS","PFC",7.87128948885303,"Baseline","Baseline"
3371,"PFC_15",74,"OID30174","P13224","GP1BB",0,"Cardiometabolic_II","B13805","20212097_SS211647","PASS",-0.7173,1.9886,"Plate control","PASS","PFC",3.96851703963549,"1h","1h"
3372,"PFC_23",75,"OID30174","P13224","GP1BB",0,"Cardiometabolic_II","B13805","20212097_SS211647","PASS",-0.7173,3.6756,"Plate control","PASS","PFC",12.7780873339159,"24h","24h"
3373,"PFC_08",85,"OID30174","P13224","GP1BB",0,"Cardiometabolic_II","B13805","20212097_SS211647","PASS",-0.7173,3.2042,"Plate control","PASS","PFC",9.216378711622,"Baseline","Baseline"
3374,"PFC_16",86,"OID30174","P13224","GP1BB",0,"Cardiometabolic_II","B13805","20212097_SS211647","PASS",-0.7173,2.0154,"Plate control","PASS","PFC",4.04292656782719,"1h","1h"
3375,"PFC_24",87,"OID30174","P13224","GP1BB",0,"Cardiometabolic_II","B13805","20212097_SS211647","PASS",-0.7173,3.8743,"Plate control","PASS","PFC",14.6649474878671,"24h","24h"
3376,"PFC_01",1,"OID30175","Q14807","KIF22",0,"Cardiometabolic_II","B13805","20212097_SS211647","WARN",0.8287,2.3441,"Plate control","PASS","PFC",5.07743547289041,"Baseline","Baseline"
3377,"PFC_09",2,"OID30175","Q14807","KIF22",0,"Cardiometabolic_II","B13805","20212097_SS211647","PASS",0.8287,2.8396,"Plate control","PASS","PFC",7.15821561347532,"Baseline","Baseline"
3378,"PFC_17",3,"OID30175","Q14807","KIF22",0,"Cardiometabolic_II","B13805","20212097_SS211647","PASS",0.8287,2.4008,"Plate control","PASS","PFC",5.28095921691423,"1h","1h"
3379,"PFC_25",4,"OID30175","Q14807","KIF22",0,"Cardiometabolic_II","B13805","20212097_SS211647","PASS",0.8287,2.5735,"Plate control","PASS","PFC",5.95251767771395,"24h","24h"
3380,"PFC_02",13,"OID30175","Q14807","KIF22",0,"Cardiometabolic_II","B13805","20212097_SS211647","PASS",0.8287,3.5131,"Plate control","PASS","PFC",11.4169073780332,"Baseline","Baseline"
3381,"PFC_10",14,"OID30175","Q14807","KIF22",0,"Cardiometabolic_II","B13805","20212097_SS211647","PASS",0.8287,2.736,"Plate control","PASS","PFC",6.66220616704439,"1h","1h"
3382,"PFC_18",15,"OID30175","Q14807","KIF22",0,"Cardiometabolic_II","B13805","20212097_SS211647","PASS",0.8287,3.418,"Plate control","PASS","PFC",10.6885926271208,"1h","1h"
3383,"PFC_26",16,"OID30175","Q14807","KIF22",0,"Cardiometabolic_II","B13805","20212097_SS211647","PASS",0.8287,1.7639,"Plate control","PASS","PFC",3.39614957447849,"24h","24h"
3384,"PFC_03",25,"OID30175","Q14807","KIF22",0,"Cardiometabolic_II","B13805","20212097_SS211647","PASS",0.8287,1.2126,"Plate control","PASS","PFC",2.31754925363041,"Baseline","Baseline"
3385,"PFC_11",26,"OID30175","Q14807","KIF22",0,"Cardiometabolic_II","B13805","20212097_SS211647","PASS",0.8287,4.0017,"Plate control","PASS","PFC",16.0188647157493,"1h","1h"
3386,"PFC_19",27,"OID30175","Q14807","KIF22",0,"Cardiometabolic_II","B13805","20212097_SS211647","PASS",0.8287,3.3456,"Plate control","PASS","PFC",10.1654346667368,"24h","24h"
3387,"PFC_27",28,"OID30175","Q14807","KIF22",0,"Cardiometabolic_II","B13805","20212097_SS211647","PASS",0.8287,3.3341,"Plate control","PASS","PFC",10.0847261283865,"24h","24h"
3388,"PFC_04",37,"OID30175","Q14807","KIF22",0,"Cardiometabolic_II","B13805","20212097_SS211647","PASS",0.8287,4.3708,"Plate control","PASS","PFC",20.6891145910982,"Baseline","Baseline"
3389,"PFC_12",38,"OID30175","Q14807","KIF22",0,"Cardiometabolic_II","B13805","20212097_SS211647","PASS",0.8287,1.5155,"Plate control","PASS","PFC",2.85897895440215,"1h","1h"
3390,"PFC_20",39,"OID30175","Q14807","KIF22",0,"Cardiometabolic_II","B13805","20212097_SS211647","PASS",0.8287,4.5276,"Plate control","PASS","PFC",23.0644662288752,"24h","24h"
3391,"PFC_05",49,"OID30175","Q14807","KIF22",0,"Cardiometabolic_II","B13805","20212097_SS211647","PASS",0.8287,2.9395,"Plate control","PASS","PFC",7.67145377055132,"Baseline","Baseline"
3392,"PFC_13",50,"OID30175","Q14807","KIF22",0,"Cardiometabolic_II","B13805","20212097_SS211647","PASS",0.8287,4.4473,"Plate control","PASS","PFC",21.8157777083304,"1h","1h"
3393,"PFC_21",51,"OID30175","Q14807","KIF22",0,"Cardiometabolic_II","B13805","20212097_SS211647","PASS",0.8287,2.3116,"Plate control","PASS","PFC",4.96433336980504,"24h","24h"
3394,"PFC_06",61,"OID30175","Q14807","KIF22",0,"Cardiometabolic_II","B13805","20212097_SS211647","PASS",0.8287,3.2131,"Plate control","PASS","PFC",9.27341037678466,"Baseline","Baseline"
3395,"PFC_14",62,"OID30175","Q14807","KIF22",0,"Cardiometabolic_II","B13805","20212097_SS211647","PASS",0.8287,3.7556,"Plate control","PASS","PFC",13.506668835818,"1h","1h"
3396,"PFC_22",63,"OID30175","Q14807","KIF22",0,"Cardiometabolic_II","B13805","20212097_SS211647","PASS",0.8287,3.9417,"Plate control","PASS","PFC",15.3663221541438,"24h","24h"
3397,"PFC_07",73,"OID30175","Q14807","KIF22",0,"Cardiometabolic_II","B13805","20212097_SS211647","PASS",0.8287,2.977,"Plate control","PASS","PFC",7.8734721762704,"Baseline","Baseline"
3398,"PFC_15",74,"OID30175","Q14807","KIF22",0,"Cardiometabolic_II","B13805","20212097_SS211647","PASS",0.8287,2.8293,"Plate control","PASS","PFC",7.1072921329385,"1h","1h"
3399,"PFC_23",75,"OID30175","Q14807","KIF22",0,"Cardiometabolic_II","B13805","20212097_SS211647","PASS",0.8287,4.1308,"Plate control","PASS","PFC",17.5184107969436,"24h","24h"
3400,"PFC_08",85,"OID30175","Q14807","KIF22",0,"Cardiometabolic_II","B13805","20212097_SS211647","PASS",0.8287,1.0481,"Plate control","PASS","PFC",2.06780479683512,"Baseline","Baseline"
3401,"PFC_16",86,"OID30175","Q14807","KIF22",0,"Cardiometabolic_II","B13805","20212097_SS211647","PASS",0.8287,3.2641,"Plate control","PASS","PFC",9.60709330976752,"1h","1h"
3402,"PFC_24",87,"OID30175","Q14807","KIF22",0,"Cardiometabolic_II","B13805","20212097_SS211647","PASS",0.8287,4.0648,"Plate control","PASS","PFC",16.7350389068581,"24h","24h"
3403,"PFC_01",1,"OID30176","P50461","CSRP3",0.25,"Cardiometabolic_II","B13805","20212097_SS211647","WARN",-0.1533,-2.0125,"Plate control","PASS","PFC",0.247843271865666,"Baseline","Baseline"
3404,"PFC_09",2,"OID30176","P50461","CSRP3",0.25,"Cardiometabolic_II","B13805","20212097_SS211647","PASS",-0.1533,-0.8131,"Plate control","PASS","PFC",0.569157562174188,"Baseline","Baseline"
3405,"PFC_17",3,"OID30176","P50461","CSRP3",0.25,"Cardiometabolic_II","B13805","20212097_SS211647","PASS",-0.1533,-0.5082,"Plate control","PASS","PFC",0.703099122965178,"1h","1h"
3406,"PFC_25",4,"OID30176","P50461","CSRP3",0.25,"Cardiometabolic_II","B13805","20212097_SS211647","PASS",-0.1533,-0.3982,"Plate control","PASS","PFC",0.758804426565518,"24h","24h"
3407,"PFC_02",13,"OID30176","P50461","CSRP3",0.25,"Cardiometabolic_II","B13805","20212097_SS211647","PASS",-0.1533,1.2,"Plate control","PASS","PFC",2.29739670999407,"Baseline","Baseline"
3408,"PFC_10",14,"OID30176","P50461","CSRP3",0.25,"Cardiometabolic_II","B13805","20212097_SS211647","PASS",-0.1533,-0.0948,"Plate control","PASS","PFC",0.936402051325314,"1h","1h"
3409,"PFC_18",15,"OID30176","P50461","CSRP3",0.25,"Cardiometabolic_II","B13805","20212097_SS211647","PASS",-0.1533,1.0753,"Plate control","PASS","PFC",2.10716019800028,"1h","1h"
3410,"PFC_26",16,"OID30176","P50461","CSRP3",0.25,"Cardiometabolic_II","B13805","20212097_SS211647","PASS",-0.1533,-0.5123,"Plate control","PASS","PFC",0.701103819721239,"24h","24h"
3411,"PFC_03",25,"OID30176","P50461","CSRP3",0.25,"Cardiometabolic_II","B13805","20212097_SS211647","PASS",-0.1533,1.3353,"Plate control","PASS","PFC",2.52327946432594,"Baseline","Baseline"
3412,"PFC_11",26,"OID30176","P50461","CSRP3",0.25,"Cardiometabolic_II","B13805","20212097_SS211647","PASS",-0.1533,2.6275,"Plate control","PASS","PFC",6.17954235983662,"1h","1h"
3413,"PFC_19",27,"OID30176","P50461","CSRP3",0.25,"Cardiometabolic_II","B13805","20212097_SS211647","PASS",-0.1533,-0.5645,"Plate control","PASS","PFC",0.676189725409926,"24h","24h"
3414,"PFC_27",28,"OID30176","P50461","CSRP3",0.25,"Cardiometabolic_II","B13805","20212097_SS211647","PASS",-0.1533,0.4541,"Plate control","PASS","PFC",1.36992793284313,"24h","24h"
3415,"PFC_04",37,"OID30176","P50461","CSRP3",0.25,"Cardiometabolic_II","B13805","20212097_SS211647","PASS",-0.1533,0.6406,"Plate control","PASS","PFC",1.55897738498429,"Baseline","Baseline"
3416,"PFC_12",38,"OID30176","P50461","CSRP3",0.25,"Cardiometabolic_II","B13805","20212097_SS211647","PASS",-0.1533,2.3575,"Plate control","PASS","PFC",5.12481526320081,"1h","1h"
3417,"PFC_20",39,"OID30176","P50461","CSRP3",0.25,"Cardiometabolic_II","B13805","20212097_SS211647","PASS",-0.1533,0.9708,"Plate control","PASS","PFC",1.95992710825997,"24h","24h"
3418,"PFC_05",49,"OID30176","P50461","CSRP3",0.25,"Cardiometabolic_II","B13805","20212097_SS211647","PASS",-0.1533,0.0817,"Plate control","PASS","PFC",1.05826431211619,"Baseline","Baseline"
3419,"PFC_13",50,"OID30176","P50461","CSRP3",0.25,"Cardiometabolic_II","B13805","20212097_SS211647","PASS",-0.1533,0.8044,"Plate control","PASS","PFC",1.74641932540053,"1h","1h"
3420,"PFC_21",51,"OID30176","P50461","CSRP3",0.25,"Cardiometabolic_II","B13805","20212097_SS211647","PASS",-0.1533,1.9725,"Plate control","PASS","PFC",3.92447589999327,"24h","24h"
3421,"PFC_06",61,"OID30176","P50461","CSRP3",0.25,"Cardiometabolic_II","B13805","20212097_SS211647","PASS",-0.1533,-0.3852,"Plate control","PASS","PFC",0.765672846394513,"Baseline","Baseline"
3422,"PFC_14",62,"OID30176","P50461","CSRP3",0.25,"Cardiometabolic_II","B13805","20212097_SS211647","PASS",-0.1533,-0.6922,"Plate control","PASS","PFC",0.618909340418444,"1h","1h"
3423,"PFC_22",63,"OID30176","P50461","CSRP3",0.25,"Cardiometabolic_II","B13805","20212097_SS211647","PASS",-0.1533,0.6405,"Plate control","PASS","PFC",1.55886932865139,"24h","24h"
3424,"PFC_07",73,"OID30176","P50461","CSRP3",0.25,"Cardiometabolic_II","B13805","20212097_SS211647","PASS",-0.1533,0.0213,"Plate control","PASS","PFC",1.01487356166481,"Baseline","Baseline"
3425,"PFC_15",74,"OID30176","P50461","CSRP3",0.25,"Cardiometabolic_II","B13805","20212097_SS211647","PASS",-0.1533,0.4665,"Plate control","PASS","PFC",1.38175324435278,"1h","1h"
3426,"PFC_23",75,"OID30176","P50461","CSRP3",0.25,"Cardiometabolic_II","B13805","20212097_SS211647","PASS",-0.1533,0.1035,"Plate control","PASS","PFC",1.07437675771437,"24h","24h"
3427,"PFC_08",85,"OID30176","P50461","CSRP3",0.25,"Cardiometabolic_II","B13805","20212097_SS211647","PASS",-0.1533,-1.1803,"Plate control","PASS","PFC",0.441259731222883,"Baseline","Baseline"
3428,"PFC_16",86,"OID30176","P50461","CSRP3",0.25,"Cardiometabolic_II","B13805","20212097_SS211647","PASS",-0.1533,-0.2713,"Plate control","PASS","PFC",0.82857258974635,"1h","1h"
3429,"PFC_24",87,"OID30176","P50461","CSRP3",0.25,"Cardiometabolic_II","B13805","20212097_SS211647","PASS",-0.1533,0.7313,"Plate control","PASS","PFC",1.66013435052303,"24h","24h"
3430,"PFC_01",1,"OID30177","Q14781","CBX2",1,"Cardiometabolic_II","B13805","20212097_SS211647","WARN",-0.2233,-1.0475,"Plate control","PASS","PFC",0.483805810071172,"Baseline","Baseline"
3431,"PFC_09",2,"OID30177","Q14781","CBX2",1,"Cardiometabolic_II","B13805","20212097_SS211647","PASS",-0.2233,-0.6474,"Plate control","PASS","PFC",0.638429844717843,"Baseline","Baseline"
3432,"PFC_17",3,"OID30177","Q14781","CBX2",1,"Cardiometabolic_II","B13805","20212097_SS211647","PASS",-0.2233,-0.7917,"Plate control","PASS","PFC",0.577663001432588,"1h","1h"
3433,"PFC_25",4,"OID30177","Q14781","CBX2",1,"Cardiometabolic_II","B13805","20212097_SS211647","PASS",-0.2233,-1.1633,"Plate control","PASS","PFC",0.446490071522156,"24h","24h"
3434,"PFC_02",13,"OID30177","Q14781","CBX2",1,"Cardiometabolic_II","B13805","20212097_SS211647","PASS",-0.2233,-0.5382,"Plate control","PASS","PFC",0.688629552247662,"Baseline","Baseline"
3435,"PFC_10",14,"OID30177","Q14781","CBX2",1,"Cardiometabolic_II","B13805","20212097_SS211647","PASS",-0.2233,-0.9005,"Plate control","PASS","PFC",0.535701039259539,"1h","1h"
3436,"PFC_18",15,"OID30177","Q14781","CBX2",1,"Cardiometabolic_II","B13805","20212097_SS211647","PASS",-0.2233,-1.0697,"Plate control","PASS","PFC",0.476418057072249,"1h","1h"
3437,"PFC_26",16,"OID30177","Q14781","CBX2",1,"Cardiometabolic_II","B13805","20212097_SS211647","PASS",-0.2233,-1.2415,"Plate control","PASS","PFC",0.42293269559454,"24h","24h"
3438,"PFC_03",25,"OID30177","Q14781","CBX2",1,"Cardiometabolic_II","B13805","20212097_SS211647","PASS",-0.2233,-1.0541,"Plate control","PASS","PFC",0.481597564053753,"Baseline","Baseline"
3439,"PFC_11",26,"OID30177","Q14781","CBX2",1,"Cardiometabolic_II","B13805","20212097_SS211647","PASS",-0.2233,-1.1989,"Plate control","PASS","PFC",0.43560728902113,"1h","1h"
3440,"PFC_19",27,"OID30177","Q14781","CBX2",1,"Cardiometabolic_II","B13805","20212097_SS211647","PASS",-0.2233,-0.5549,"Plate control","PASS","PFC",0.680704239436979,"24h","24h"
3441,"PFC_27",28,"OID30177","Q14781","CBX2",1,"Cardiometabolic_II","B13805","20212097_SS211647","PASS",-0.2233,-0.3448,"Plate control","PASS","PFC",0.787417128195681,"24h","24h"
3442,"PFC_04",37,"OID30177","Q14781","CBX2",1,"Cardiometabolic_II","B13805","20212097_SS211647","PASS",-0.2233,-0.554,"Plate control","PASS","PFC",0.681129017320244,"Baseline","Baseline"
3443,"PFC_12",38,"OID30177","Q14781","CBX2",1,"Cardiometabolic_II","B13805","20212097_SS211647","PASS",-0.2233,-1.2526,"Plate control","PASS","PFC",0.419691165522646,"1h","1h"
3444,"PFC_20",39,"OID30177","Q14781","CBX2",1,"Cardiometabolic_II","B13805","20212097_SS211647","PASS",-0.2233,-0.7658,"Plate control","PASS","PFC",0.588127151229731,"24h","24h"
3445,"PFC_05",49,"OID30177","Q14781","CBX2",1,"Cardiometabolic_II","B13805","20212097_SS211647","PASS",-0.2233,-0.7192,"Plate control","PASS","PFC",0.607434181857261,"Baseline","Baseline"
3446,"PFC_13",50,"OID30177","Q14781","CBX2",1,"Cardiometabolic_II","B13805","20212097_SS211647","PASS",-0.2233,-1.2444,"Plate control","PASS","PFC",0.422083401119313,"1h","1h"
3447,"PFC_21",51,"OID30177","Q14781","CBX2",1,"Cardiometabolic_II","B13805","20212097_SS211647","PASS",-0.2233,-1.0971,"Plate control","PASS","PFC",0.46745519622762,"24h","24h"
3448,"PFC_06",61,"OID30177","Q14781","CBX2",1,"Cardiometabolic_II","B13805","20212097_SS211647","PASS",-0.2233,-0.6917,"Plate control","PASS","PFC",0.619123875224512,"Baseline","Baseline"
3449,"PFC_14",62,"OID30177","Q14781","CBX2",1,"Cardiometabolic_II","B13805","20212097_SS211647","PASS",-0.2233,-0.6072,"Plate control","PASS","PFC",0.656469550400132,"1h","1h"
3450,"PFC_22",63,"OID30177","Q14781","CBX2",1,"Cardiometabolic_II","B13805","20212097_SS211647","PASS",-0.2233,-1.3427,"Plate control","PASS","PFC",0.394282067155158,"24h","24h"
3451,"PFC_07",73,"OID30177","Q14781","CBX2",1,"Cardiometabolic_II","B13805","20212097_SS211647","PASS",-0.2233,-0.9985,"Plate control","PASS","PFC",0.500520130733928,"Baseline","Baseline"
3452,"PFC_15",74,"OID30177","Q14781","CBX2",1,"Cardiometabolic_II","B13805","20212097_SS211647","PASS",-0.2233,-1.1807,"Plate control","PASS","PFC",0.441137405006244,"1h","1h"
3453,"PFC_23",75,"OID30177","Q14781","CBX2",1,"Cardiometabolic_II","B13805","20212097_SS211647","PASS",-0.2233,-0.3659,"Plate control","PASS","PFC",0.775984639567286,"24h","24h"
3454,"PFC_08",85,"OID30177","Q14781","CBX2",1,"Cardiometabolic_II","B13805","20212097_SS211647","PASS",-0.2233,-0.8684,"Plate control","PASS","PFC",0.547753992514074,"Baseline","Baseline"
3455,"PFC_16",86,"OID30177","Q14781","CBX2",1,"Cardiometabolic_II","B13805","20212097_SS211647","PASS",-0.2233,-0.8335,"Plate control","PASS","PFC",0.561166191949274,"1h","1h"
3456,"PFC_24",87,"OID30177","Q14781","CBX2",1,"Cardiometabolic_II","B13805","20212097_SS211647","PASS",-0.2233,-1.2945,"Plate control","PASS","PFC",0.407677431951677,"24h","24h"
3457,"PFC_01",1,"OID30178","Q96A35","MRPL24",0.5909,"Cardiometabolic_II","B13805","20212097_SS211647","WARN",0.2827,0.0865,"Plate control","PASS","PFC",1.06179113395499,"Baseline","Baseline"
3458,"PFC_09",2,"OID30178","Q96A35","MRPL24",0.5909,"Cardiometabolic_II","B13805","20212097_SS211647","PASS",0.2827,-0.4575,"Plate control","PASS","PFC",0.728247121988208,"Baseline","Baseline"
3459,"PFC_17",3,"OID30178","Q96A35","MRPL24",0.5909,"Cardiometabolic_II","B13805","20212097_SS211647","PASS",0.2827,0.4016,"Plate control","PASS","PFC",1.32097210364467,"1h","1h"
3460,"PFC_25",4,"OID30178","Q96A35","MRPL24",0.5909,"Cardiometabolic_II","B13805","20212097_SS211647","PASS",0.2827,-0.1352,"Plate control","PASS","PFC",0.91054359669674,"24h","24h"
3461,"PFC_02",13,"OID30178","Q96A35","MRPL24",0.5909,"Cardiometabolic_II","B13805","20212097_SS211647","PASS",0.2827,0.0257,"Plate control","PASS","PFC",1.0179734961165,"Baseline","Baseline"
3462,"PFC_10",14,"OID30178","Q96A35","MRPL24",0.5909,"Cardiometabolic_II","B13805","20212097_SS211647","PASS",0.2827,-0.3305,"Plate control","PASS","PFC",0.795260819591264,"1h","1h"
3463,"PFC_18",15,"OID30178","Q96A35","MRPL24",0.5909,"Cardiometabolic_II","B13805","20212097_SS211647","PASS",0.2827,0.4422,"Plate control","PASS","PFC",1.35867462379092,"1h","1h"
3464,"PFC_26",16,"OID30178","Q96A35","MRPL24",0.5909,"Cardiometabolic_II","B13805","20212097_SS211647","PASS",0.2827,0.358,"Plate control","PASS","PFC",1.28164792416019,"24h","24h"
3465,"PFC_03",25,"OID30178","Q96A35","MRPL24",0.5909,"Cardiometabolic_II","B13805","20212097_SS211647","PASS",0.2827,-0.0762,"Plate control","PASS","PFC",0.948552808684341,"Baseline","Baseline"
3466,"PFC_11",26,"OID30178","Q96A35","MRPL24",0.5909,"Cardiometabolic_II","B13805","20212097_SS211647","PASS",0.2827,0.316,"Plate control","PASS","PFC",1.24487423538217,"1h","1h"
3467,"PFC_19",27,"OID30178","Q96A35","MRPL24",0.5909,"Cardiometabolic_II","B13805","20212097_SS211647","PASS",0.2827,-0.0822,"Plate control","PASS","PFC",0.944616080325356,"24h","24h"
3468,"PFC_27",28,"OID30178","Q96A35","MRPL24",0.5909,"Cardiometabolic_II","B13805","20212097_SS211647","PASS",0.2827,-0.3878,"Plate control","PASS","PFC",0.764294206713674,"24h","24h"
3469,"PFC_04",37,"OID30178","Q96A35","MRPL24",0.5909,"Cardiometabolic_II","B13805","20212097_SS211647","PASS",0.2827,0.2137,"Plate control","PASS","PFC",1.1596584852286,"Baseline","Baseline"
3470,"PFC_12",38,"OID30178","Q96A35","MRPL24",0.5909,"Cardiometabolic_II","B13805","20212097_SS211647","PASS",0.2827,-0.3305,"Plate control","PASS","PFC",0.795260819591264,"1h","1h"
3471,"PFC_20",39,"OID30178","Q96A35","MRPL24",0.5909,"Cardiometabolic_II","B13805","20212097_SS211647","PASS",0.2827,0.3336,"Plate control","PASS","PFC",1.26015395427874,"24h","24h"
3472,"PFC_05",49,"OID30178","Q96A35","MRPL24",0.5909,"Cardiometabolic_II","B13805","20212097_SS211647","PASS",0.2827,-0.0175,"Plate control","PASS","PFC",0.987943197140516,"Baseline","Baseline"

Supplemental table 1_Olink proximity extension assay (PEA) data

,"SampleID","Index","OlinkID","UniProt","Assay","MissingFreq","Panel","Panel_Lot_Nr","PlateID","QC_Warning","LOD","NPX","Normalization","Assay_Warning","condition","Value","group","alt_group"
3473,"PFC_13",50,"OID30178","Q96A35","MRPL24",0.5909,"Cardiometabolic_II","B13805","20212097_SS211647","PASS",0.2827,0.0585,"Plate control","PASS","PFC",1.04138245080425,"1h","1h"
3474,"PFC_21",51,"OID30178","Q96A35","MRPL24",0.5909,"Cardiometabolic_II","B13805","20212097_SS211647","PASS",0.2827,-0.1664,"Plate control","PASS","PFC",0.891063406409537,"24h","24h"
3475,"PFC_06",61,"OID30178","Q96A35","MRPL24",0.5909,"Cardiometabolic_II","B13805","20212097_SS211647","PASS",0.2827,0.2386,"Plate control","PASS","PFC",1.17984717488601,"Baseline","Baseline"
3476,"PFC_14",62,"OID30178","Q96A35","MRPL24",0.5909,"Cardiometabolic_II","B13805","20212097_SS211647","PASS",0.2827,0.501,"Plate control","PASS","PFC",1.41519416032666,"1h","1h"
3477,"PFC_22",63,"OID30178","Q96A35","MRPL24",0.5909,"Cardiometabolic_II","B13805","20212097_SS211647","PASS",0.2827,-0.2841,"Plate control","PASS","PFC",0.821253773805367,"24h","24h"
3478,"PFC_07",73,"OID30178","Q96A35","MRPL24",0.5909,"Cardiometabolic_II","B13805","20212097_SS211647","PASS",0.2827,0.1974,"Plate control","PASS","PFC",1.14663005501621,"Baseline","Baseline"
3479,"PFC_15",74,"OID30178","Q96A35","MRPL24",0.5909,"Cardiometabolic_II","B13805","20212097_SS211647","PASS",0.2827,-0.094,"Plate control","PASS","PFC",0.936921446872426,"1h","1h"
3480,"PFC_23",75,"OID30178","Q96A35","MRPL24",0.5909,"Cardiometabolic_II","B13805","20212097_SS211647","PASS",0.2827,0.0262,"Plate control","PASS","PFC",1.01832635998898,"24h","24h"
3481,"PFC_08",85,"OID30178","Q96A35","MRPL24",0.5909,"Cardiometabolic_II","B13805","20212097_SS211647","PASS",0.2827,0.3018,"Plate control","PASS","PFC",1.23268142768712,"Baseline","Baseline"
3482,"PFC_16",86,"OID30178","Q96A35","MRPL24",0.5909,"Cardiometabolic_II","B13805","20212097_SS211647","PASS",0.2827,0.0405,"Plate control","PASS","PFC",1.02847020550837,"1h","1h"
3483,"PFC_24",87,"OID30178","Q96A35","MRPL24",0.5909,"Cardiometabolic_II","B13805","20212097_SS211647","PASS",0.2827,0.3412,"Plate control","PASS","PFC",1.2668098586873,"24h","24h"
3484,"PFC_01",1,"OID30179","Q58F21","BRDT",0.0909,"Cardiometabolic_II","B13805","20212097_SS211647","WARN",0.2758,1.1234,"Plate control","PASS","PFC",2.17859798256251,"Baseline","Baseline"
3485,"PFC_09",2,"OID30179","Q58F21","BRDT",0.0909,"Cardiometabolic_II","B13805","20212097_SS211647","PASS",0.2758,1.1604,"Plate control","PASS","PFC",2.23519391758472,"Baseline","Baseline"
3486,"PFC_17",3,"OID30179","Q58F21","BRDT",0.0909,"Cardiometabolic_II","B13805","20212097_SS211647","PASS",0.2758,1.394,"Plate control","PASS","PFC",2.62806325429874,"1h","1h"
3487,"PFC_25",4,"OID30179","Q58F21","BRDT",0.0909,"Cardiometabolic_II","B13805","20212097_SS211647","PASS",0.2758,0.8221,"Plate control","PASS","PFC",1.76797760474949,"24h","24h"
3488,"PFC_02",13,"OID30179","Q58F21","BRDT",0.0909,"Cardiometabolic_II","B13805","20212097_SS211647","PASS",0.2758,0.8733,"Plate control","PASS","PFC",1.83184825536976,"Baseline","Baseline"
3489,"PFC_10",14,"OID30179","Q58F21","BRDT",0.0909,"Cardiometabolic_II","B13805","20212097_SS211647","PASS",0.2758,0.2146,"Plate control","PASS","PFC",1.1603821435344,"1h","1h"
3490,"PFC_18",15,"OID30179","Q58F21","BRDT",0.0909,"Cardiometabolic_II","B13805","20212097_SS211647","PASS",0.2758,1.0797,"Plate control","PASS","PFC",2.1135965253811,"1h","1h"
3491,"PFC_26",16,"OID30179","Q58F21","BRDT",0.0909,"Cardiometabolic_II","B13805","20212097_SS211647","PASS",0.2758,1.6919,"Plate control","PASS","PFC",3.23081915935785,"24h","24h"
3492,"PFC_03",25,"OID30179","Q58F21","BRDT",0.0909,"Cardiometabolic_II","B13805","20212097_SS211647","PASS",0.2758,1.0992,"Plate control","PASS","PFC",2.14235861976522,"Baseline","Baseline"
3493,"PFC_11",26,"OID30179","Q58F21","BRDT",0.0909,"Cardiometabolic_II","B13805","20212097_SS211647","PASS",0.2758,0.837,"Plate control","PASS","PFC",1.78633170470552,"1h","1h"
3494,"PFC_19",27,"OID30179","Q58F21","BRDT",0.0909,"Cardiometabolic_II","B13805","20212097_SS211647","PASS",0.2758,0.3016,"Plate control","PASS","PFC",1.23251055360026,"24h","24h"
3495,"PFC_27",28,"OID30179","Q58F21","BRDT",0.0909,"Cardiometabolic_II","B13805","20212097_SS211647","PASS",0.2758,1.4782,"Plate control","PASS","PFC",2.78600915744688,"24h","24h"
3496,"PFC_04",37,"OID30179","Q58F21","BRDT",0.0909,"Cardiometabolic_II","B13805","20212097_SS211647","PASS",0.2758,1.1924,"Plate control","PASS","PFC",2.28532603278438,"Baseline","Baseline"
3497,"PFC_12",38,"OID30179","Q58F21","BRDT",0.0909,"Cardiometabolic_II","B13805","20212097_SS211647","PASS",0.2758,0.793,"Plate control","PASS","PFC",1.73267371280017,"1h","1h"
3498,"PFC_20",39,"OID30179","Q58F21","BRDT",0.0909,"Cardiometabolic_II","B13805","20212097_SS211647","PASS",0.2758,1.5574,"Plate control","PASS","PFC",2.94322941506357,"24h","24h"
3499,"PFC_05",49,"OID30179","Q58F21","BRDT",0.0909,"Cardiometabolic_II","B13805","20212097_SS211647","PASS",0.2758,0.7783,"Plate control","PASS","PFC",1.715108683014,"Baseline","Baseline"
3500,"PFC_13",50,"OID30179","Q58F21","BRDT",0.0909,"Cardiometabolic_II","B13805","20212097_SS211647","PASS",0.2758,0.6304,"Plate control","PASS","PFC",1.54799412915527,"1h","1h"
3501,"PFC_21",51,"OID30179","Q58F21","BRDT",0.0909,"Cardiometabolic_II","B13805","20212097_SS211647","PASS",0.2758,0.7138,"Plate control","PASS","PFC",1.64011843793852,"24h","24h"
3502,"PFC_06",61,"OID30179","Q58F21","BRDT",0.0909,"Cardiometabolic_II","B13805","20212097_SS211647","PASS",0.2758,0.5987,"Plate control","PASS","PFC",1.51435138261647,"Baseline","Baseline"
3503,"PFC_14",62,"OID30179","Q58F21","BRDT",0.0909,"Cardiometabolic_II","B13805","20212097_SS211647","PASS",0.2758,0.737,"Plate control","PASS","PFC",1.66670641431844,"1h","1h"
3504,"PFC_22",63,"OID30179","Q58F21","BRDT",0.0909,"Cardiometabolic_II","B13805","20212097_SS211647","PASS",0.2758,0.5156,"Plate control","PASS","PFC",1.42958856529524,"24h","24h"
3505,"PFC_07",73,"OID30179","Q58F21","BRDT",0.0909,"Cardiometabolic_II","B13805","20212097_SS211647","PASS",0.2758,0.236,"Plate control","PASS","PFC",1.17772278959498,"Baseline","Baseline"
3506,"PFC_15",74,"OID30179","Q58F21","BRDT",0.0909,"Cardiometabolic_II","B13805","20212097_SS211647","PASS",0.2758,0.8401,"Plate control","PASS","PFC",1.79017422297711,"1h","1h"
3507,"PFC_23",75,"OID30179","Q58F21","BRDT",0.0909,"Cardiometabolic_II","B13805","20212097_SS211647","PASS",0.2758,1.4751,"Plate control","PASS","PFC",2.78002912994179,"24h","24h"
3508,"PFC_08",85,"OID30179","Q58F21","BRDT",0.0909,"Cardiometabolic_II","B13805","20212097_SS211647","PASS",0.2758,1.025,"Plate control","PASS","PFC",2.03495938420537,"Baseline","Baseline"
3509,"PFC_16",86,"OID30179","Q58F21","BRDT",0.0909,"Cardiometabolic_II","B13805","20212097_SS211647","PASS",0.2758,0.4656,"Plate control","PASS","PFC",1.38089153063422,"1h","1h"
3510,"PFC_24",87,"OID30179","Q58F21","BRDT",0.0909,"Cardiometabolic_II","B13805","20212097_SS211647","PASS",0.2758,0.9349,"Plate control","PASS","PFC",1.91175811761991,"24h","24h"
3511,"PFC_01",1,"OID30180","Q96EU7","C1GALT1C1",0.8182,"Cardiometabolic_II","B13805","20212097_SS211647","WARN",0.645,0.1493,"Plate control","PASS","PFC",1.1090312361894,"Baseline","Baseline"
3512,"PFC_09",2,"OID30180","Q96EU7","C1GALT1C1",0.8182,"Cardiometabolic_II","B13805","20212097_SS211647","PASS",0.645,-0.0732,"Plate control","PASS","PFC",0.950527321029199,"Baseline","Baseline"
3513,"PFC_17",3,"OID30180","Q96EU7","C1GALT1C1",0.8182,"Cardiometabolic_II","B13805","20212097_SS211647","PASS",0.645,-0.8002,"Plate control","PASS","PFC",0.57426956131461,"1h","1h"
3514,"PFC_25",4,"OID30180","Q96EU7","C1GALT1C1",0.8182,"Cardiometabolic_II","B13805","20212097_SS211647","PASS",0.645,-0.1708,"Plate control","PASS","PFC",0.888349938757671,"24h","24h"
3515,"PFC_02",13,"OID30180","Q96EU7","C1GALT1C1",0.8182,"Cardiometabolic_II","B13805","20212097_SS211647","PASS",0.645,0.555,"Plate control","PASS","PFC",1.46916863327831,"Baseline","Baseline"
3516,"PFC_10",14,"OID30180","Q96EU7","C1GALT1C1",0.8182,"Cardiometabolic_II","B13805","20212097_SS211647","PASS",0.645,-0.3384,"Plate control","PASS","PFC",0.790917981755083,"1h","1h"
3517,"PFC_18",15,"OID30180","Q96EU7","C1GALT1C1",0.8182,"Cardiometabolic_II","B13805","20212097_SS211647","PASS",0.645,0.2378,"Plate control","PASS","PFC",1.17919311005384,"1h","1h"
3518,"PFC_26",16,"OID30180","Q96EU7","C1GALT1C1",0.8182,"Cardiometabolic_II","B13805","20212097_SS211647","PASS",0.645,-0.9179,"Plate control","PASS","PFC",0.529278882982675,"24h","24h"
3519,"PFC_03",25,"OID30180","Q96EU7","C1GALT1C1",0.8182,"Cardiometabolic_II","B13805","20212097_SS211647","PASS",0.645,0.4237,"Plate control","PASS","PFC",1.34136326722942,"Baseline","Baseline"
3520,"PFC_11",26,"OID30180","Q96EU7","C1GALT1C1",0.8182,"Cardiometabolic_II","B13805","20212097_SS211647","PASS",0.645,0.9111,"Plate control","PASS","PFC",1.88047874535499,"1h","1h"
3521,"PFC_19",27,"OID30180","Q96EU7","C1GALT1C1",0.8182,"Cardiometabolic_II","B13805","20212097_SS211647","PASS",0.645,0.0581,"Plate control","PASS","PFC",1.04109375830351,"24h","24h"
3522,"PFC_27",28,"OID30180","Q96EU7","C1GALT1C1",0.8182,"Cardiometabolic_II","B13805","20212097_SS211647","PASS",0.645,-0.5534,"Plate control","PASS","PFC",0.681412349828271,"24h","24h"
3523,"PFC_04",37,"OID30180","Q96EU7","C1GALT1C1",0.8182,"Cardiometabolic_II","B13805","20212097_SS211647","PASS",0.645,0.7389,"Plate control","PASS","PFC",1.66890287876907,"Baseline","Baseline"
3524,"PFC_12",38,"OID30180","Q96EU7","C1GALT1C1",0.8182,"Cardiometabolic_II","B13805","20212097_SS211647","PASS",0.645,-0.0825,"Plate control","PASS","PFC",0.944419673355068,"1h","1h"
3525,"PFC_20",39,"OID30180","Q96EU7","C1GALT1C1",0.8182,"Cardiometabolic_II","B13805","20212097_SS211647","PASS",0.645,0.6631,"Plate control","PASS","PFC",1.58348148651147,"24h","24h"
3526,"PFC_05",49,"OID30180","Q96EU7","C1GALT1C1",0.8182,"Cardiometabolic_II","B13805","20212097_SS211647","PASS",0.645,-0.1385,"Plate control","PASS","PFC",0.908463212525778,"Baseline","Baseline"
3527,"PFC_13",50,"OID30180","Q96EU7","C1GALT1C1",0.8182,"Cardiometabolic_II","B13805","20212097_SS211647","PASS",0.645,0.0204,"Plate control","PASS","PFC",1.01424064802827,"1h","1h"
3528,"PFC_21",51,"OID30180","Q96EU7","C1GALT1C1",0.8182,"Cardiometabolic_II","B13805","20212097_SS211647","PASS",0.645,-0.0542,"Plate control","PASS","PFC",0.963128366822566,"24h","24h"
3529,"PFC_06",61,"OID30180","Q96EU7","C1GALT1C1",0.8182,"Cardiometabolic_II","B13805","20212097_SS211647","PASS",0.645,0.7206,"Plate control","PASS","PFC",1.6478672207556,"Baseline","Baseline"
3530,"PFC_14",62,"OID30180","Q96EU7","C1GALT1C1",0.8182,"Cardiometabolic_II","B13805","20212097_SS211647","PASS",0.645,0.3779,"Plate control","PASS","PFC",1.29944898839349,"1h","1h"
3531,"PFC_22",63,"OID30180","Q96EU7","C1GALT1C1",0.8182,"Cardiometabolic_II","B13805","20212097_SS211647","PASS",0.645,-0.5809,"Plate control","PASS","PFC",0.668546586214709,"24h","24h"
3532,"PFC_07",73,"OID30180","Q96EU7","C1GALT1C1",0.8182,"Cardiometabolic_II","B13805","20212097_SS211647","PASS",0.645,-1.1018,"Plate control","PASS","PFC",0.465934802454327,"Baseline","Baseline"
3533,"PFC_15",74,"OID30180","Q96EU7","C1GALT1C1",0.8182,"Cardiometabolic_II","B13805","20212097_SS211647","PASS",0.645,-0.7234,"Plate control","PASS","PFC",0.605668380001373,"1h","1h"
3534,"PFC_23",75,"OID30180","Q96EU7","C1GALT1C1",0.8182,"Cardiometabolic_II","B13805","20212097_SS211647","PASS",0.645,0.6548,"Plate control","PASS","PFC",1.57439768014661,"24h","24h"
3535,"PFC_08",85,"OID30180","Q96EU7","C1GALT1C1",0.8182,"Cardiometabolic_II","B13805","20212097_SS211647","PASS",0.645,0.0607,"Plate control","PASS","PFC",1.04297169111272,"Baseline","Baseline"
3536,"PFC_16",86,"OID30180","Q96EU7","C1GALT1C1",0.8182,"Cardiometabolic_II","B13805","20212097_SS211647","PASS",0.645,-0.0322,"Plate control","PASS","PFC",0.97792789445737,"1h","1h"
3537,"PFC_24",87,"OID30180","Q96EU7","C1GALT1C1",0.8182,"Cardiometabolic_II","B13805","20212097_SS211647","PASS",0.645,-0.0583,"Plate control","PASS","PFC",0.960395134633986,"24h","24h"
3538,"PFC_01",1,"OID30181","Q5VVQ6","YOD1",0,"Cardiometabolic_II","B13805","20212097_SS211647","WARN",-0.0971,3.1739,"Plate control","PASS","PFC",9.02483152431894,"Baseline","Baseline"
3539,"PFC_09",2,"OID30181","Q5VVQ6","YOD1",0,"Cardiometabolic_II","B13805","20212097_SS211647","PASS",-0.0971,3.3821,"Plate control","PASS","PFC",10.4258998381689,"Baseline","Baseline"
3540,"PFC_17",3,"OID30181","Q5VVQ6","YOD1",0,"Cardiometabolic_II","B13805","20212097_SS211647","PASS",-0.0971,3.8851,"Plate control","PASS","PFC",14.7751410709149,"1h","1h"
3541,"PFC_25",4,"OID30181","Q5VVQ6","YOD1",0,"Cardiometabolic_II","B13805","20212097_SS211647","PASS",-0.0971,3.6873,"Plate control","PASS","PFC",12.8821366884581,"24h","24h"
3542,"PFC_02",13,"OID30181","Q5VVQ6","YOD1",0,"Cardiometabolic_II","B13805","20212097_SS211647","PASS",-0.0971,3.438,"Plate control","PASS","PFC",10.8377998198829,"Baseline","Baseline"
3543,"PFC_10",14,"OID30181","Q5VVQ6","YOD1",0,"Cardiometabolic_II","B13805","20212097_SS211647","PASS",-0.0971,3.2059,"Plate control","PASS","PFC",9.22724523441152,"1h","1h"
3544,"PFC_18",15,"OID30181","Q5VVQ6","YOD1",0,"Cardiometabolic_II","B13805","20212097_SS211647","PASS",-0.0971,3.1582,"Plate control","PASS","PFC",8.92715205887814,"1h","1h"
3545,"PFC_26",16,"OID30181","Q5VVQ6","YOD1",0,"Cardiometabolic_II","B13805","20212097_SS211647","PASS",-0.0971,3.6595,"Plate control","PASS","PFC",12.636280829619,"24h","24h"
3546,"PFC_03",25,"OID30181","Q5VVQ6","YOD1",0,"Cardiometabolic_II","B13805","20212097_SS211647","PASS",-0.0971,1.5886,"Plate control","PASS","PFC",3.00757351072716,"Baseline","Baseline"
3547,"PFC_11",26,"OID30181","Q5VVQ6","YOD1",0,"Cardiometabolic_II","B13805","20212097_SS211647","PASS",-0.0971,4.073,"Plate control","PASS","PFC",16.830428467262,"1h","1h"
3548,"PFC_19",27,"OID30181","Q5VVQ6","YOD1",0,"Cardiometabolic_II","B13805","20212097_SS211647","PASS",-0.0971,3.6951,"Plate control","PASS","PFC",12.9519731960155,"24h","24h"
3549,"PFC_27",28,"OID30181","Q5VVQ6","YOD1",0,"Cardiometabolic_II","B13805","20212097_SS211647","PASS",-0.0971,4.9593,"Plate control","PASS","PFC",31.1098600563785,"24h","24h"
3550,"PFC_04",37,"OID30181","Q5VVQ6","YOD1",0,"Cardiometabolic_II","B13805","20212097_SS211647","PASS",-0.0971,3.8103,"Plate control","PASS","PFC",14.0286084048826,"Baseline","Baseline"
3551,"PFC_12",38,"OID30181","Q5VVQ6","YOD1",0,"Cardiometabolic_II","B13805","20212097_SS211647","PASS",-0.0971,2.3092,"Plate control","PASS","PFC",4.95608180234013,"1h","1h"
3552,"PFC_20",39,"OID30181","Q5VVQ6","YOD1",0,"Cardiometabolic_II","B13805","20212097_SS211647","PASS",-0.0971,4.4124,"Plate control","PASS","PFC",21.2943678913174,"24h","24h"
3553,"PFC_05",49,"OID30181","Q5VVQ6","YOD1",0,"Cardiometabolic_II","B13805","20212097_SS211647","PASS",-0.0971,2.9094,"Plate control","PASS","PFC",7.51305675141849,"Baseline","Baseline"
3554,"PFC_13",50,"OID30181","Q5VVQ6","YOD1",0,"Cardiometabolic_II","B13805","20212097_SS211647","PASS",-0.0971,3.6248,"Plate control","PASS","PFC",12.3359763552749,"1h","1h"
3555,"PFC_21",51,"OID30181","Q5VVQ6","YOD1",0,"Cardiometabolic_II","B13805","20212097_SS211647","PASS",-0.0971,2.3917,"Plate control","PASS","PFC",5.24775366520433,"24h","24h"
3556,"PFC_06",61,"OID30181","Q5VVQ6","YOD1",0,"Cardiometabolic_II","B13805","20212097_SS211647","PASS",-0.0971,3.8346,"Plate control","PASS","PFC",14.2669001376625,"Baseline","Baseline"
3557,"PFC_14",62,"OID30181","Q5VVQ6","YOD1",0,"Cardiometabolic_II","B13805","20212097_SS211647","PASS",-0.0971,3.7443,"Plate control","PASS","PFC",13.4012902303756,"1h","1h"
3558,"PFC_22",63,"OID30181","Q5VVQ6","YOD1",0,"Cardiometabolic_II","B13805","20212097_SS211647","PASS",-0.0971,3.2601,"Plate control","PASS","PFC",9.58049368315556,"24h","24h"
3559,"PFC_07",73,"OID30181","Q5VVQ6","YOD1",0,"Cardiometabolic_II","B13805","20212097_SS211647","PASS",-0.0971,4.0288,"Plate control","PASS","PFC",16.3226115966816,"Baseline","Baseline"
3560,"PFC_15",74,"OID30181","Q5VVQ6","YOD1",0,"Cardiometabolic_II","B13805","20212097_SS211647","PASS",-0.0971,2.1103,"Plate control","PASS","PFC",4.31781071616868,"1h","1h"
3561,"PFC_23",75,"OID30181","Q5VVQ6","YOD1",0,"Cardiometabolic_II","B13805","20212097_SS211647","PASS",-0.0971,4.7,"Plate control","PASS","PFC",25.9920766833995,"24h","24h"
3562,"PFC_08",85,"OID30181","Q5VVQ6","YOD1",0,"Cardiometabolic_II","B13805","20212097_SS211647","PASS",-0.0971,2.758,"Plate control","PASS","PFC",6.76457829902013,"Baseline","Baseline"
3563,"PFC_16",86,"OID30181","Q5VVQ6","YOD1",0,"Cardiometabolic_II","B13805","20212097_SS211647","PASS",-0.0971,3.3738,"Plate control","PASS","PFC",10.3660905785621,"1h","1h"
3564,"PFC_24",87,"OID30181","Q5VVQ6","YOD1",0,"Cardiometabolic_II","B13805","20212097_SS211647","PASS",-0.0971,4.3496,"Plate control","PASS","PFC",20.3873166889911,"24h","24h"
3565,"PFC_01",1,"OID30182","A6NDB9","PALM3",0.8864,"Cardiometabolic_II","B13805","20212097_SS211647","WARN",0.9433,0.081,"Plate control","PASS","PFC",1.05775096361843,"Baseline","Baseline"
3566,"PFC_09",2,"OID30182","A6NDB9","PALM3",0.8864,"Cardiometabolic_II","B13805","20212097_SS211647","PASS",0.9433,0.3851,"Plate control","PASS","PFC",1.30595030552897,"Baseline","Baseline"
3567,"PFC_17",3,"OID30182","A6NDB9","PALM3",0.8864,"Cardiometabolic_II","B13805","20212097_SS211647","PASS",0.9433,0.5694,"Plate control","PASS","PFC",1.48390630299867,"1h","1h"
3568,"PFC_25",4,"OID30182","A6NDB9","PALM3",0.8864,"Cardiometabolic_II","B13805","20212097_SS211647","PASS",0.9433,0.2995,"Plate control","PASS","PFC",1.23071780513527,"24h","24h"
3569,"PFC_02",13,"OID30182","A6NDB9","PALM3",0.8864,"Cardiometabolic_II","B13805","20212097_SS211647","PASS",0.9433,0.6008,"Plate control","PASS","PFC",1.51655729131895,"Baseline","Baseline"
3570,"PFC_10",14,"OID30182","A6NDB9","PALM3",0.8864,"Cardiometabolic_II","B13805","20212097_SS211647","PASS",0.9433,0.2066,"Plate control","PASS","PFC",1.15396542600605,"1h","1h"
3571,"PFC_18",15,"OID30182","A6NDB9","PALM3",0.8864,"Cardiometabolic_II","B13805","20212097_SS211647","PASS",0.9433,-0.0399,"Plate control","PASS","PFC",0.97272236905236,"1h","1h"
3572,"PFC_26",16,"OID30182","A6NDB9","PALM3",0.8864,"Cardiometabolic_II","B13805","20212097_SS211647","PASS",0.9433,0.6989,"Plate control","PASS","PFC",1.62326664178535,"24h","24h"
3573,"PFC_03",25,"OID30182","A6NDB9","PALM3",0.8864,"Cardiometabolic_II","B13805","20212097_SS211647","PASS",0.9433,0.273,"Plate control","PASS","PFC",1.20831784338994,"Baseline","Baseline"
3574,"PFC_11",26,"OID30182","A6NDB9","PALM3",0.8864,"Cardiometabolic_II","B13805","20212097_SS211647","PASS",0.9433,-0.2987,"Plate control","PASS","PFC",0.812984639811774,"1h","1h"
3575,"PFC_19",27,"OID30182","A6NDB9","PALM3",0.8864,"Cardiometabolic_II","B13805","20212097_SS211647","PASS",0.9433,0.3422,"Plate control","PASS","PFC",1.26768824876079,"24h","24h"
3576,"PFC_27",28,"OID30182","A6NDB9","PALM3",0.8864,"Cardiometabolic_II","B13805","20212097_SS211647","PASS",0.9433,0.3344,"Plate control","PASS","PFC",1.26085292578526,"24h","24h"
3577,"PFC_04",37,"OID30182","A6NDB9","PALM3",0.8864,"Cardiometabolic_II","B13805","20212097_SS211647","PASS",0.9433,0.3404,"Plate control","PASS","PFC",1.26610758487146,"Baseline","Baseline"
3578,"PFC_12",38,"OID30182","A6NDB9","PALM3",0.8864,"Cardiometabolic_II","B13805","20212097_SS211647","PASS",0.9433,0.2223,"Plate control","PASS","PFC",1.1665919305403,"1h","1h"
3579,"PFC_20",39,"OID30182","A6NDB9","PALM3",0.8864,"Cardiometabolic_II","B13805","20212097_SS211647","PASS",0.9433,0.6823,"Plate control","PASS","PFC",1.60469598516256,"24h","24h"
3580,"PFC_05",49,"OID30182","A6NDB9","PALM3",0.8864,"Cardiometabolic_II","B13805","20212097_SS211647","PASS",0.9433,0.4312,"Plate control","PASS","PFC",1.34835464042472,"Baseline","Baseline"
3581,"PFC_13",50,"OID30182","A6NDB9","PALM3",0.8864,"Cardiometabolic_II","B13805","20212097_SS211647","PASS",0.9433,0.1843,"Plate control","PASS","PFC",1.13626551999436,"1h","1h"
3582,"PFC_21",51,"OID30182","A6NDB9","PALM3",0.8864,"Cardiometabolic_II","B13805","20212097_SS211647","PASS",0.9433,0.3784,"Plate control","PASS","PFC",1.29989942114432,"24h","24h"
3583,"PFC_06",61,"OID30182","A6NDB9","PALM3",0.8864,"Cardiometabolic_II","B13805","20212097_SS211647","PASS",0.9433,0.0597,"Plate control","PASS","PFC",1.04224900871718,"Baseline","Baseline"
3584,"PFC_14",62,"OID30182","A6NDB9","PALM3",0.8864,"Cardiometabolic_II","B13805","20212097_SS211647","PASS",0.9433,-0.0437,"Plate control","PASS","PFC",0.97016362922316,"1h","1h"

Supplemental table 1_Olink proximity extension assay (PEA) data

,"SampleID","Index","OlinkID","UniProt","Assay","MissingFreq","Panel","Panel_Lot_Nr","PlateID","QC_Warning","LOD","NPX","Normalization","Assay_Warning","condition","Value","group","alt_group"
3585,"PFC_22",63,"OID30182","A6NDB9","PALM3",0.8864,"Cardiometabolic_II","B13805","20212097_SS211647","PASS",0.9433,0.2091,"Plate control","PASS","PFC",1.15596682929278,"24h","24h"
3586,"PFC_07",73,"OID30182","A6NDB9","PALM3",0.8864,"Cardiometabolic_II","B13805","20212097_SS211647","PASS",0.9433,0.2689,"Plate control","PASS","PFC",1.2048887955731,"Baseline","Baseline"
3587,"PFC_15",74,"OID30182","A6NDB9","PALM3",0.8864,"Cardiometabolic_II","B13805","20212097_SS211647","PASS",0.9433,-0.1174,"Plate control","PASS","PFC",0.921847492080987,"1h","1h"
3588,"PFC_23",75,"OID30182","A6NDB9","PALM3",0.8864,"Cardiometabolic_II","B13805","20212097_SS211647","PASS",0.9433,0.3738,"Plate control","PASS","PFC",1.29576132232026,"24h","24h"
3589,"PFC_08",85,"OID30182","A6NDB9","PALM3",0.8864,"Cardiometabolic_II","B13805","20212097_SS211647","PASS",0.9433,0.4487,"Plate control","PASS","PFC",1.36480988413845,"Baseline","Baseline"
3590,"PFC_16",86,"OID30182","A6NDB9","PALM3",0.8864,"Cardiometabolic_II","B13805","20212097_SS211647","PASS",0.9433,0.2021,"Plate control","PASS","PFC",1.15037162827224,"1h","1h"
3591,"PFC_24",87,"OID30182","A6NDB9","PALM3",0.8864,"Cardiometabolic_II","B13805","20212097_SS211647","PASS",0.9433,0.208,"Plate control","PASS","PFC",1.15508578455358,"24h","24h"
3592,"PFC_01",1,"OID30183","O75534","CSDE1",0.0227,"Cardiometabolic_II","B13805","20212097_SS211647","WARN",0.3508,2.0169,"Plate control","PASS","PFC",4.04713226855308,"Baseline","Baseline"
3593,"PFC_09",2,"OID30183","O75534","CSDE1",0.0227,"Cardiometabolic_II","B13805","20212097_SS211647","PASS",0.3508,3.0642,"Plate control","PASS","PFC",8.36404022345426,"Baseline","Baseline"
3594,"PFC_17",3,"OID30183","O75534","CSDE1",0.0227,"Cardiometabolic_II","B13805","20212097_SS211647","PASS",0.3508,4.5051,"Plate control","PASS","PFC",22.7075476119183,"1h","1h"
3595,"PFC_25",4,"OID30183","O75534","CSDE1",0.0227,"Cardiometabolic_II","B13805","20212097_SS211647","PASS",0.3508,4.0713,"Plate control","PASS","PFC",16.8106080084088,"24h","24h"
3596,"PFC_02",13,"OID30183","O75534","CSDE1",0.0227,"Cardiometabolic_II","B13805","20212097_SS211647","PASS",0.3508,2.7578,"Plate control","PASS","PFC",6.76364059434324,"Baseline","Baseline"
3597,"PFC_10",14,"OID30183","O75534","CSDE1",0.0227,"Cardiometabolic_II","B13805","20212097_SS211647","PASS",0.3508,2.031,"Plate control","PASS","PFC",4.0868803308572,"1h","1h"
3598,"PFC_18",15,"OID30183","O75534","CSDE1",0.0227,"Cardiometabolic_II","B13805","20212097_SS211647","PASS",0.3508,3.7606,"Plate control","PASS","PFC",13.5535605932171,"1h","1h"
3599,"PFC_26",16,"OID30183","O75534","CSDE1",0.0227,"Cardiometabolic_II","B13805","20212097_SS211647","PASS",0.3508,3.8058,"Plate control","PASS","PFC",13.984919070733,"24h","24h"
3600,"PFC_03",25,"OID30183","O75534","CSDE1",0.0227,"Cardiometabolic_II","B13805","20212097_SS211647","PASS",0.3508,0.855,"Plate control","PASS","PFC",1.80875875512218,"Baseline","Baseline"
3601,"PFC_11",26,"OID30183","O75534","CSDE1",0.0227,"Cardiometabolic_II","B13805","20212097_SS211647","PASS",0.3508,3.8499,"Plate control","PASS","PFC",14.4190079176657,"1h","1h"
3602,"PFC_19",27,"OID30183","O75534","CSDE1",0.0227,"Cardiometabolic_II","B13805","20212097_SS211647","PASS",0.3508,3.0856,"Plate control","PASS","PFC",8.48903168593021,"24h","24h"
3603,"PFC_27",28,"OID30183","O75534","CSDE1",0.0227,"Cardiometabolic_II","B13805","20212097_SS211647","PASS",0.3508,5.3919,"Plate control","PASS","PFC",41.9878496701128,"24h","24h"
3604,"PFC_04",37,"OID30183","O75534","CSDE1",0.0227,"Cardiometabolic_II","B13805","20212097_SS211647","PASS",0.3508,4.1012,"Plate control","PASS","PFC",17.1626449519697,"Baseline","Baseline"
3605,"PFC_12",38,"OID30183","O75534","CSDE1",0.0227,"Cardiometabolic_II","B13805","20212097_SS211647","PASS",0.3508,1.936,"Plate control","PASS","PFC",3.8264326323675,"1h","1h"
3606,"PFC_20",39,"OID30183","O75534","CSDE1",0.0227,"Cardiometabolic_II","B13805","20212097_SS211647","PASS",0.3508,5.0049,"Plate control","PASS","PFC",32.1088702578793,"24h","24h"
3607,"PFC_05",49,"OID30183","O75534","CSDE1",0.0227,"Cardiometabolic_II","B13805","20212097_SS211647","PASS",0.3508,2.1666,"Plate control","PASS","PFC",4.48964072299002,"Baseline","Baseline"
3608,"PFC_13",50,"OID30183","O75534","CSDE1",0.0227,"Cardiometabolic_II","B13805","20212097_SS211647","PASS",0.3508,4.4382,"Plate control","PASS","PFC",21.6786047242131,"1h","1h"
3609,"PFC_21",51,"OID30183","O75534","CSDE1",0.0227,"Cardiometabolic_II","B13805","20212097_SS211647","PASS",0.3508,1.8445,"Plate control","PASS","PFC",3.59128463142195,"24h","24h"
3610,"PFC_06",61,"OID30183","O75534","CSDE1",0.0227,"Cardiometabolic_II","B13805","20212097_SS211647","PASS",0.3508,3.9187,"Plate control","PASS","PFC",15.1232887415323,"Baseline","Baseline"
3611,"PFC_14",62,"OID30183","O75534","CSDE1",0.0227,"Cardiometabolic_II","B13805","20212097_SS211647","PASS",0.3508,4.3772,"Plate control","PASS","PFC",20.7810983159419,"1h","1h"
3612,"PFC_22",63,"OID30183","O75534","CSDE1",0.0227,"Cardiometabolic_II","B13805","20212097_SS211647","PASS",0.3508,3.8386,"Plate control","PASS","PFC",14.3065112714018,"24h","24h"
3613,"PFC_07",73,"OID30183","O75534","CSDE1",0.0227,"Cardiometabolic_II","B13805","20212097_SS211647","PASS",0.3508,4.3675,"Plate control","PASS","PFC",20.6418446891817,"Baseline","Baseline"
3614,"PFC_15",74,"OID30183","O75534","CSDE1",0.0227,"Cardiometabolic_II","B13805","20212097_SS211647","PASS",0.3508,2.176,"Plate control","PASS","PFC",4.51898885828049,"1h","1h"
3615,"PFC_23",75,"OID30183","O75534","CSDE1",0.0227,"Cardiometabolic_II","B13805","20212097_SS211647","PASS",0.3508,4.2092,"Plate control","PASS","PFC",18.4967513213542,"24h","24h"
3616,"PFC_08",85,"OID30183","O75534","CSDE1",0.0227,"Cardiometabolic_II","B13805","20212097_SS211647","PASS",0.3508,3.2019,"Plate control","PASS","PFC",9.20169731164462,"Baseline","Baseline"
3617,"PFC_16",86,"OID30183","O75534","CSDE1",0.0227,"Cardiometabolic_II","B13805","20212097_SS211647","PASS",0.3508,4.5808,"Plate control","PASS","PFC",23.9308543829858,"1h","1h"
3618,"PFC_24",87,"OID30183","O75534","CSDE1",0.0227,"Cardiometabolic_II","B13805","20212097_SS211647","PASS",0.3508,4.5174,"Plate control","PASS","PFC",22.9019732154476,"24h","24h"
3619,"PFC_01",1,"OID30184","Q13563","PKD2",0,"Cardiometabolic_II","B13805","20212097_SS211647","WARN",-1.6259,-0.0411,"Plate control","PASS","PFC",0.971913617728117,"Baseline","Baseline"
3620,"PFC_09",2,"OID30184","Q13563","PKD2",0,"Cardiometabolic_II","B13805","20212097_SS211647","PASS",-1.6259,0.904,"Plate control","PASS","PFC",1.8712469956628,"Baseline","Baseline"
3621,"PFC_17",3,"OID30184","Q13563","PKD2",0,"Cardiometabolic_II","B13805","20212097_SS211647","PASS",-1.6259,2.0582,"Plate control","PASS","PFC",4.16466369569952,"1h","1h"
3622,"PFC_25",4,"OID30184","Q13563","PKD2",0,"Cardiometabolic_II","B13805","20212097_SS211647","PASS",-1.6259,1.4014,"Plate control","PASS","PFC",2.64157798143908,"24h","24h"
3623,"PFC_02",13,"OID30184","Q13563","PKD2",0,"Cardiometabolic_II","B13805","20212097_SS211647","PASS",-1.6259,0.741,"Plate control","PASS","PFC",1.67133391784371,"Baseline","Baseline"
3624,"PFC_10",14,"OID30184","Q13563","PKD2",0,"Cardiometabolic_II","B13805","20212097_SS211647","PASS",-1.6259,0.7382,"Plate control","PASS","PFC",1.66809331845798,"1h","1h"
3625,"PFC_18",15,"OID30184","Q13563","PKD2",0,"Cardiometabolic_II","B13805","20212097_SS211647","PASS",-1.6259,1.3611,"Plate control","PASS","PFC",2.56880966812592,"1h","1h"
3626,"PFC_26",16,"OID30184","Q13563","PKD2",0,"Cardiometabolic_II","B13805","20212097_SS211647","PASS",-1.6259,1.8903,"Plate control","PASS","PFC",3.70712304198297,"24h","24h"
3627,"PFC_03",25,"OID30184","Q13563","PKD2",0,"Cardiometabolic_II","B13805","20212097_SS211647","PASS",-1.6259,1.5629,"Plate control","PASS","PFC",2.95447133172017,"Baseline","Baseline"
3628,"PFC_11",26,"OID30184","Q13563","PKD2",0,"Cardiometabolic_II","B13805","20212097_SS211647","PASS",-1.6259,1.7451,"Plate control","PASS","PFC",3.35218088609215,"1h","1h"
3629,"PFC_19",27,"OID30184","Q13563","PKD2",0,"Cardiometabolic_II","B13805","20212097_SS211647","PASS",-1.6259,1.0118,"Plate control","PASS","PFC",2.01642535450249,"24h","24h"
3630,"PFC_27",28,"OID30184","Q13563","PKD2",0,"Cardiometabolic_II","B13805","20212097_SS211647","PASS",-1.6259,1.4719,"Plate control","PASS","PFC",2.77386966160553,"24h","24h"
3631,"PFC_04",37,"OID30184","Q13563","PKD2",0,"Cardiometabolic_II","B13805","20212097_SS211647","PASS",-1.6259,1.3636,"Plate control","PASS","PFC",2.57326493515291,"Baseline","Baseline"
3632,"PFC_12",38,"OID30184","Q13563","PKD2",0,"Cardiometabolic_II","B13805","20212097_SS211647","PASS",-1.6259,1.8116,"Plate control","PASS","PFC",3.51031378985981,"1h","1h"
3633,"PFC_20",39,"OID30184","Q13563","PKD2",0,"Cardiometabolic_II","B13805","20212097_SS211647","PASS",-1.6259,1.9536,"Plate control","PASS","PFC",3.87339867386587,"24h","24h"
3634,"PFC_05",49,"OID30184","Q13563","PKD2",0,"Cardiometabolic_II","B13805","20212097_SS211647","PASS",-1.6259,-0.5452,"Plate control","PASS","PFC",0.685296393651871,"Baseline","Baseline"
3635,"PFC_13",50,"OID30184","Q13563","PKD2",0,"Cardiometabolic_II","B13805","20212097_SS211647","PASS",-1.6259,1.3776,"Plate control","PASS","PFC",2.59835760733071,"1h","1h"
3636,"PFC_21",51,"OID30184","Q13563","PKD2",0,"Cardiometabolic_II","B13805","20212097_SS211647","PASS",-1.6259,1.4209,"Plate control","PASS","PFC",2.67752491563999,"24h","24h"
3637,"PFC_06",61,"OID30184","Q13563","PKD2",0,"Cardiometabolic_II","B13805","20212097_SS211647","PASS",-1.6259,0.6389,"Plate control","PASS","PFC",1.5571414455623,"Baseline","Baseline"
3638,"PFC_14",62,"OID30184","Q13563","PKD2",0,"Cardiometabolic_II","B13805","20212097_SS211647","PASS",-1.6259,1.548,"Plate control","PASS","PFC",2.92411489680007,"1h","1h"
3639,"PFC_22",63,"OID30184","Q13563","PKD2",0,"Cardiometabolic_II","B13805","20212097_SS211647","PASS",-1.6259,1.2714,"Plate control","PASS","PFC",2.41395703767253,"24h","24h"
3640,"PFC_07",73,"OID30184","Q13563","PKD2",0,"Cardiometabolic_II","B13805","20212097_SS211647","PASS",-1.6259,1.4409,"Plate control","PASS","PFC",2.71490177058717,"Baseline","Baseline"
3641,"PFC_15",74,"OID30184","Q13563","PKD2",0,"Cardiometabolic_II","B13805","20212097_SS211647","PASS",-1.6259,0.3029,"Plate control","PASS","PFC",1.23362165870848,"1h","1h"
3642,"PFC_23",75,"OID30184","Q13563","PKD2",0,"Cardiometabolic_II","B13805","20212097_SS211647","PASS",-1.6259,1.419,"Plate control","PASS","PFC",2.67400099081027,"24h","24h"
3643,"PFC_08",85,"OID30184","Q13563","PKD2",0,"Cardiometabolic_II","B13805","20212097_SS211647","PASS",-1.6259,0.7276,"Plate control","PASS","PFC",1.6558821509996,"Baseline","Baseline"
3644,"PFC_16",86,"OID30184","Q13563","PKD2",0,"Cardiometabolic_II","B13805","20212097_SS211647","PASS",-1.6259,1.4724,"Plate control","PASS","PFC",2.77483117818162,"1h","1h"
3645,"PFC_24",87,"OID30184","Q13563","PKD2",0,"Cardiometabolic_II","B13805","20212097_SS211647","PASS",-1.6259,1.2394,"Plate control","PASS","PFC",2.36100320501864,"24h","24h"
3646,"PFC_01",1,"OID30185","Q99598","TSNAX",0,"Cardiometabolic_II","B13805","20212097_SS211647","WARN",-0.0568,0.8235,"Plate control","PASS","PFC",1.76969409362923,"Baseline","Baseline"
3647,"PFC_09",2,"OID30185","Q99598","TSNAX",0,"Cardiometabolic_II","B13805","20212097_SS211647","PASS",-0.0568,1.7598,"Plate control","PASS","PFC",3.38651174669358,"Baseline","Baseline"
3648,"PFC_17",3,"OID30185","Q99598","TSNAX",0,"Cardiometabolic_II","B13805","20212097_SS211647","PASS",-0.0568,1.983,"Plate control","PASS","PFC",3.95314260600293,"1h","1h"
3649,"PFC_25",4,"OID30185","Q99598","TSNAX",0,"Cardiometabolic_II","B13805","20212097_SS211647","PASS",-0.0568,2.2068,"Plate control","PASS","PFC",4.61650164268552,"24h","24h"
3650,"PFC_02",13,"OID30185","Q99598","TSNAX",0,"Cardiometabolic_II","B13805","20212097_SS211647","PASS",-0.0568,1.5808,"Plate control","PASS","PFC",2.99135679787322,"Baseline","Baseline"
3651,"PFC_10",14,"OID30185","Q99598","TSNAX",0,"Cardiometabolic_II","B13805","20212097_SS211647","PASS",-0.0568,1.291,"Plate control","PASS","PFC",2.44697608226373,"1h","1h"
3652,"PFC_18",15,"OID30185","Q99598","TSNAX",0,"Cardiometabolic_II","B13805","20212097_SS211647","PASS",-0.0568,1.6976,"Plate control","PASS","PFC",3.24360917820182,"1h","1h"
3653,"PFC_26",16,"OID30185","Q99598","TSNAX",0,"Cardiometabolic_II","B13805","20212097_SS211647","PASS",-0.0568,1.8917,"Plate control","PASS","PFC",3.71072220266254,"24h","24h"
3654,"PFC_03",25,"OID30185","Q99598","TSNAX",0,"Cardiometabolic_II","B13805","20212097_SS211647","PASS",-0.0568,0.3943,"Plate control","PASS","PFC",1.31430490075647,"Baseline","Baseline"
3655,"PFC_11",26,"OID30185","Q99598","TSNAX",0,"Cardiometabolic_II","B13805","20212097_SS211647","PASS",-0.0568,2.0641,"Plate control","PASS","PFC",4.18173024622827,"1h","1h"
3656,"PFC_19",27,"OID30185","Q99598","TSNAX",0,"Cardiometabolic_II","B13805","20212097_SS211647","PASS",-0.0568,1.5566,"Plate control","PASS","PFC",2.94159779455001,"24h","24h"
3657,"PFC_27",28,"OID30185","Q99598","TSNAX",0,"Cardiometabolic_II","B13805","20212097_SS211647","PASS",-0.0568,3.2213,"Plate control","PASS","PFC",9.32626872651481,"24h","24h"
3658,"PFC_04",37,"OID30185","Q99598","TSNAX",0,"Cardiometabolic_II","B13805","20212097_SS211647","PASS",-0.0568,1.9937,"Plate control","PASS","PFC",3.98257077395614,"Baseline","Baseline"
3659,"PFC_12",38,"OID30185","Q99598","TSNAX",0,"Cardiometabolic_II","B13805","20212097_SS211647","PASS",-0.0568,0.4452,"Plate control","PASS","PFC",1.36150284778937,"1h","1h"
3660,"PFC_20",39,"OID30185","Q99598","TSNAX",0,"Cardiometabolic_II","B13805","20212097_SS211647","PASS",-0.0568,2.3284,"Plate control","PASS","PFC",5.02248029932671,"24h","24h"
3661,"PFC_05",49,"OID30185","Q99598","TSNAX",0,"Cardiometabolic_II","B13805","20212097_SS211647","PASS",-0.0568,1.4373,"Plate control","PASS","PFC",2.70813564053318,"Baseline","Baseline"
3662,"PFC_13",50,"OID30185","Q99598","TSNAX",0,"Cardiometabolic_II","B13805","20212097_SS211647","PASS",-0.0568,2.1894,"Plate control","PASS","PFC",4.561157536871,"1h","1h"
3663,"PFC_21",51,"OID30185","Q99598","TSNAX",0,"Cardiometabolic_II","B13805","20212097_SS211647","PASS",-0.0568,1.2539,"Plate control","PASS","PFC",2.38485243355805,"24h","24h"
3664,"PFC_06",61,"OID30185","Q99598","TSNAX",0,"Cardiometabolic_II","B13805","20212097_SS211647","PASS",-0.0568,1.7777,"Plate control","PASS","PFC",3.42879107533978,"Baseline","Baseline"
3665,"PFC_14",62,"OID30185","Q99598","TSNAX",0,"Cardiometabolic_II","B13805","20212097_SS211647","PASS",-0.0568,1.7374,"Plate control","PASS","PFC",3.3343371750541,"1h","1h"
3666,"PFC_22",63,"OID30185","Q99598","TSNAX",0,"Cardiometabolic_II","B13805","20212097_SS211647","PASS",-0.0568,1.1068,"Plate control","PASS","PFC",2.15367416905473,"24h","24h"
3667,"PFC_07",73,"OID30185","Q99598","TSNAX",0,"Cardiometabolic_II","B13805","20212097_SS211647","PASS",-0.0568,2.1344,"Plate control","PASS","PFC",4.3905448991037,"Baseline","Baseline"
3668,"PFC_15",74,"OID30185","Q99598","TSNAX",0,"Cardiometabolic_II","B13805","20212097_SS211647","PASS",-0.0568,0.2486,"Plate control","PASS","PFC",1.18805366097021,"1h","1h"
3669,"PFC_23",75,"OID30185","Q99598","TSNAX",0,"Cardiometabolic_II","B13805","20212097_SS211647","PASS",-0.0568,2.4373,"Plate control","PASS","PFC",5.41627128106636,"24h","24h"
3670,"PFC_08",85,"OID30185","Q99598","TSNAX",0,"Cardiometabolic_II","B13805","20212097_SS211647","PASS",-0.0568,1.5211,"Plate control","PASS","PFC",2.87009800235266,"Baseline","Baseline"
3671,"PFC_16",86,"OID30185","Q99598","TSNAX",0,"Cardiometabolic_II","B13805","20212097_SS211647","PASS",-0.0568,2.0193,"Plate control","PASS","PFC",4.05387049166667,"1h","1h"
3672,"PFC_24",87,"OID30185","Q99598","TSNAX",0,"Cardiometabolic_II","B13805","20212097_SS211647","PASS",-0.0568,2.3346,"Plate control","PASS","PFC",5.04411091692621,"24h","24h"
3673,"PFC_01",1,"OID30186","Q86VP3","PACS2",0,"Cardiometabolic_II","B13805","20212097_SS211647","WARN",-0.075,2.1986,"Plate control","PASS","PFC",4.59033676737316,"Baseline","Baseline"
3674,"PFC_09",2,"OID30186","Q86VP3","PACS2",0,"Cardiometabolic_II","B13805","20212097_SS211647","PASS",-0.075,2.248,"Plate control","PASS","PFC",4.75023866429364,"Baseline","Baseline"
3675,"PFC_17",3,"OID30186","Q86VP3","PACS2",0,"Cardiometabolic_II","B13805","20212097_SS211647","PASS",-0.075,3.85,"Plate control","PASS","PFC",14.4200074017733,"1h","1h"
3676,"PFC_25",4,"OID30186","Q86VP3","PACS2",0,"Cardiometabolic_II","B13805","20212097_SS211647","PASS",-0.075,3.4878,"Plate control","PASS","PFC",11.2184386915057,"24h","24h"
3677,"PFC_02",13,"OID30186","Q86VP3","PACS2",0,"Cardiometabolic_II","B13805","20212097_SS211647","PASS",-0.075,2.6362,"Plate control","PASS","PFC",6.21691993864184,"Baseline","Baseline"
3678,"PFC_10",14,"OID30186","Q86VP3","PACS2",0,"Cardiometabolic_II","B13805","20212097_SS211647","PASS",-0.075,2.1927,"Plate control","PASS","PFC",4.57160260477264,"1h","1h"
3679,"PFC_18",15,"OID30186","Q86VP3","PACS2",0,"Cardiometabolic_II","B13805","20212097_SS211647","PASS",-0.075,3.175,"Plate control","PASS","PFC",9.03171523844905,"1h","1h"
3680,"PFC_26",16,"OID30186","Q86VP3","PACS2",0,"Cardiometabolic_II","B13805","20212097_SS211647","PASS",-0.075,3.1837,"Plate control","PASS","PFC",9.08634446961439,"24h","24h"
3681,"PFC_03",25,"OID30186","Q86VP3","PACS2",0,"Cardiometabolic_II","B13805","20212097_SS211647","PASS",-0.075,1.8563,"Plate control","PASS","PFC",3.62077869301717,"Baseline","Baseline"
3682,"PFC_11",26,"OID30186","Q86VP3","PACS2",0,"Cardiometabolic_II","B13805","20212097_SS211647","PASS",-0.075,3.5871,"Plate control","PASS","PFC",12.0177923965498,"1h","1h"
3683,"PFC_19",27,"OID30186","Q86VP3","PACS2",0,"Cardiometabolic_II","B13805","20212097_SS211647","PASS",-0.075,3.0401,"Plate control","PASS","PFC",8.2254807403676,"24h","24h"
3684,"PFC_27",28,"OID30186","Q86VP3","PACS2",0,"Cardiometabolic_II","B13805","20212097_SS211647","PASS",-0.075,4.2353,"Plate control","PASS","PFC",18.8344238966674,"24h","24h"
3685,"PFC_04",37,"OID30186","Q86VP3","PACS2",0,"Cardiometabolic_II","B13805","20212097_SS211647","PASS",-0.075,2.9417,"Plate control","PASS","PFC",7.68316107707189,"Baseline","Baseline"
3686,"PFC_12",38,"OID30186","Q86VP3","PACS2",0,"Cardiometabolic_II","B13805","20212097_SS211647","PASS",-0.075,2.173,"Plate control","PASS","PFC",4.50960164858169,"1h","1h"
3687,"PFC_20",39,"OID30186","Q86VP3","PACS2",0,"Cardiometabolic_II","B13805","20212097_SS211647","PASS",-0.075,4.2061,"Plate control","PASS","PFC",18.457048981769,"24h","24h"
3688,"PFC_05",49,"OID30186","Q86VP3","PACS2",0,"Cardiometabolic_II","B13805","20212097_SS211647","PASS",-0.075,2.5365,"Plate control","PASS","PFC",5.80179773298966,"Baseline","Baseline"
3689,"PFC_13",50,"OID30186","Q86VP3","PACS2",0,"Cardiometabolic_II","B13805","20212097_SS211647","PASS",-0.075,3.293,"Plate control","PASS","PFC",9.80148265523741,"1h","1h"
3690,"PFC_21",51,"OID30186","Q86VP3","PACS2",0,"Cardiometabolic_II","B13805","20212097_SS211647","PASS",-0.075,2.3861,"Plate control","PASS","PFC",5.22742334039032,"24h","24h"
3691,"PFC_06",61,"OID30186","Q86VP3","PACS2",0,"Cardiometabolic_II","B13805","20212097_SS211647","PASS",-0.075,3.4285,"Plate control","PASS","PFC",10.7666684645846,"Baseline","Baseline"
3692,"PFC_14",62,"OID30186","Q86VP3","PACS2",0,"Cardiometabolic_II","B13805","20212097_SS211647","PASS",-0.075,3.8567,"Plate control","PASS","PFC",14.4871309008646,"1h","1h"
3693,"PFC_22",63,"OID30186","Q86VP3","PACS2",0,"Cardiometabolic_II","B13805","20212097_SS211647","PASS",-0.075,2.9496,"Plate control","PASS","PFC",7.72534841304943,"24h","24h"
3694,"PFC_07",73,"OID30186","Q86VP3","PACS2",0,"Cardiometabolic_II","B13805","20212097_SS211647","PASS",-0.075,3.8273,"Plate control","PASS","PFC",14.1948923204337,"Baseline","Baseline"
3695,"PFC_15",74,"OID30186","Q86VP3","PACS2",0,"Cardiometabolic_II","B13805","20212097_SS211647","PASS",-0.075,2.214,"Plate control","PASS","PFC",4.63959861802174,"1h","1h"
3696,"PFC_23",75,"OID30186","Q86VP3","PACS2",0,"Cardiometabolic_II","B13805","20212097_SS211647","PASS",-0.075,4.0297,"Plate control","PASS","PFC",16.3327973484394,"24h","24h"

Supplemental table 1_Olink proximity extension assay (PEA) data

,"SampleID","Index","OlinkID","UniProt","Assay","MissingFreq","Panel","Panel_Lot_Nr","PlateID","QC_Warning","LOD","NPX","Normalization","Assay_Warning","condition","Value","group","alt_group"
3697,"PFC_08",85,"OID30186","Q86VP3","PACS2",0,"Cardiometabolic_II","B13805","20212097_SS211647","PASS",-0.075,2.7912,"Plate control","PASS","PFC",6.92205305848079,"Baseline","Baseline"
3698,"PFC_16",86,"OID30186","Q86VP3","PACS2",0,"Cardiometabolic_II","B13805","20212097_SS211647","PASS",-0.075,3.5515,"Plate control","PASS","PFC",11.724869823046,"1h","1h"
3699,"PFC_24",87,"OID30186","Q86VP3","PACS2",0,"Cardiometabolic_II","B13805","20212097_SS211647","PASS",-0.075,4.3292,"Plate control","PASS","PFC",20.1010645043905,"24h","24h"
3700,"PFC_01",1,"OID30187","Q5W0V3","FHIP2A",0.8182,"Cardiometabolic_II","B13805","20212097_SS211647","WARN",0.6979,-0.2874,"Plate control","PASS","PFC",0.819377396487943,"Baseline","Baseline"
3701,"PFC_09",2,"OID30187","Q5W0V3","FHIP2A",0.8182,"Cardiometabolic_II","B13805","20212097_SS211647","PASS",0.6979,0.2732,"Plate control","PASS","PFC",1.20848536342255,"Baseline","Baseline"
3702,"PFC_17",3,"OID30187","Q5W0V3","FHIP2A",0.8182,"Cardiometabolic_II","B13805","20212097_SS211647","PASS",0.6979,0.9258,"Plate control","PASS","PFC",1.89973738797138,"1h","1h"
3703,"PFC_25",4,"OID30187","Q5W0V3","FHIP2A",0.8182,"Cardiometabolic_II","B13805","20212097_SS211647","PASS",0.6979,0.2786,"Plate control","PASS","PFC",1.21301719384141,"24h","24h"
3704,"PFC_02",13,"OID30187","Q5W0V3","FHIP2A",0.8182,"Cardiometabolic_II","B13805","20212097_SS211647","PASS",0.6979,0.4348,"Plate control","PASS","PFC",1.35172343138166,"Baseline","Baseline"
3705,"PFC_10",14,"OID30187","Q5W0V3","FHIP2A",0.8182,"Cardiometabolic_II","B13805","20212097_SS211647","PASS",0.6979,0.2932,"Plate control","PASS","PFC",1.22535519042979,"1h","1h"
3706,"PFC_18",15,"OID30187","Q5W0V3","FHIP2A",0.8182,"Cardiometabolic_II","B13805","20212097_SS211647","PASS",0.6979,-0.2134,"Plate control","PASS","PFC",0.862502175007917,"1h","1h"
3707,"PFC_26",16,"OID30187","Q5W0V3","FHIP2A",0.8182,"Cardiometabolic_II","B13805","20212097_SS211647","PASS",0.6979,0.6208,"Plate control","PASS","PFC",1.53772764217754,"24h","24h"
3708,"PFC_03",25,"OID30187","Q5W0V3","FHIP2A",0.8182,"Cardiometabolic_II","B13805","20212097_SS211647","PASS",0.6979,0.9552,"Plate control","PASS","PFC",1.93884839670042,"Baseline","Baseline"
3709,"PFC_11",26,"OID30187","Q5W0V3","FHIP2A",0.8182,"Cardiometabolic_II","B13805","20212097_SS211647","PASS",0.6979,0.2983,"Plate control","PASS","PFC",1.22969454846272,"1h","1h"
3710,"PFC_19",27,"OID30187","Q5W0V3","FHIP2A",0.8182,"Cardiometabolic_II","B13805","20212097_SS211647","PASS",0.6979,0.2882,"Plate control","PASS","PFC",1.2211157835284,"24h","24h"
3711,"PFC_27",28,"OID30187","Q5W0V3","FHIP2A",0.8182,"Cardiometabolic_II","B13805","20212097_SS211647","PASS",0.6979,0.1982,"Plate control","PASS","PFC",1.14726605804922,"24h","24h"
3712,"PFC_04",37,"OID30187","Q5W0V3","FHIP2A",0.8182,"Cardiometabolic_II","B13805","20212097_SS211647","PASS",0.6979,0.3785,"Plate control","PASS","PFC",1.29998952642897,"Baseline","Baseline"
3713,"PFC_12",38,"OID30187","Q5W0V3","FHIP2A",0.8182,"Cardiometabolic_II","B13805","20212097_SS211647","PASS",0.6979,0.2593,"Plate control","PASS","PFC",1.19689782526101,"1h","1h"
3714,"PFC_20",39,"OID30187","Q5W0V3","FHIP2A",0.8182,"Cardiometabolic_II","B13805","20212097_SS211647","PASS",0.6979,0.5631,"Plate control","PASS","PFC",1.47744046840295,"24h","24h"
3715,"PFC_05",49,"OID30187","Q5W0V3","FHIP2A",0.8182,"Cardiometabolic_II","B13805","20212097_SS211647","PASS",0.6979,-0.0445,"Plate control","PASS","PFC",0.969625805405944,"Baseline","Baseline"
3716,"PFC_13",50,"OID30187","Q5W0V3","FHIP2A",0.8182,"Cardiometabolic_II","B13805","20212097_SS211647","PASS",0.6979,0.9459,"Plate control","PASS","PFC",1.92639025621501,"1h","1h"
3717,"PFC_21",51,"OID30187","Q5W0V3","FHIP2A",0.8182,"Cardiometabolic_II","B13805","20212097_SS211647","PASS",0.6979,0.0109,"Plate control","PASS","PFC",1.0075839175948,"24h","24h"
3718,"PFC_06",61,"OID30187","Q5W0V3","FHIP2A",0.8182,"Cardiometabolic_II","B13805","20212097_SS211647","PASS",0.6979,0.6005,"Plate control","PASS","PFC",1.51624196488204,"Baseline","Baseline"
3719,"PFC_14",62,"OID30187","Q5W0V3","FHIP2A",0.8182,"Cardiometabolic_II","B13805","20212097_SS211647","PASS",0.6979,0.7403,"Plate control","PASS","PFC",1.67052317827121,"1h","1h"
3720,"PFC_22",63,"OID30187","Q5W0V3","FHIP2A",0.8182,"Cardiometabolic_II","B13805","20212097_SS211647","PASS",0.6979,0.1938,"Plate control","PASS","PFC",1.14377240168963,"24h","24h"
3721,"PFC_07",73,"OID30187","Q5W0V3","FHIP2A",0.8182,"Cardiometabolic_II","B13805","20212097_SS211647","PASS",0.6979,-0.0481,"Plate control","PASS","PFC",0.967209285451463,"Baseline","Baseline"
3722,"PFC_15",74,"OID30187","Q5W0V3","FHIP2A",0.8182,"Cardiometabolic_II","B13805","20212097_SS211647","PASS",0.6979,0.0971,"Plate control","PASS","PFC",1.06962122580948,"1h","1h"
3723,"PFC_23",75,"OID30187","Q5W0V3","FHIP2A",0.8182,"Cardiometabolic_II","B13805","20212097_SS211647","PASS",0.6979,0.5771,"Plate control","PASS","PFC",1.49184743009173,"24h","24h"
3724,"PFC_08",85,"OID30187","Q5W0V3","FHIP2A",0.8182,"Cardiometabolic_II","B13805","20212097_SS211647","PASS",0.6979,0.0601,"Plate control","PASS","PFC",1.04253802156575,"Baseline","Baseline"
3725,"PFC_16",86,"OID30187","Q5W0V3","FHIP2A",0.8182,"Cardiometabolic_II","B13805","20212097_SS211647","PASS",0.6979,0.1413,"Plate control","PASS","PFC",1.10289848051714,"1h","1h"
3726,"PFC_24",87,"OID30187","Q5W0V3","FHIP2A",0.8182,"Cardiometabolic_II","B13805","20212097_SS211647","PASS",0.6979,0.2315,"Plate control","PASS","PFC",1.17405500423772,"24h","24h"
3727,"PFC_01",1,"OID30188","P41227","NAA10",0.0455,"Cardiometabolic_II","B13805","20212097_SS211647","WARN",1.5824,3.3176,"Plate control","PASS","PFC",9.97004488755276,"Baseline","Baseline"
3728,"PFC_09",2,"OID30188","P41227","NAA10",0.0455,"Cardiometabolic_II","B13805","20212097_SS211647","PASS",1.5824,3.4156,"Plate control","PASS","PFC",10.6708263659539,"Baseline","Baseline"
3729,"PFC_17",3,"OID30188","P41227","NAA10",0.0455,"Cardiometabolic_II","B13805","20212097_SS211647","PASS",1.5824,5.3262,"Plate control","PASS","PFC",40.1186178899934,"1h","1h"
3730,"PFC_25",4,"OID30188","P41227","NAA10",0.0455,"Cardiometabolic_II","B13805","20212097_SS211647","PASS",1.5824,4.5789,"Plate control","PASS","PFC",23.899358679075,"24h","24h"
3731,"PFC_02",13,"OID30188","P41227","NAA10",0.0455,"Cardiometabolic_II","B13805","20212097_SS211647","PASS",1.5824,3.7679,"Plate control","PASS","PFC",13.62231506433,"Baseline","Baseline"
3732,"PFC_10",14,"OID30188","P41227","NAA10",0.0455,"Cardiometabolic_II","B13805","20212097_SS211647","PASS",1.5824,3.7269,"Plate control","PASS","PFC",13.2406312548207,"1h","1h"
3733,"PFC_18",15,"OID30188","P41227","NAA10",0.0455,"Cardiometabolic_II","B13805","20212097_SS211647","PASS",1.5824,4.6166,"Plate control","PASS","PFC",24.5321198638731,"1h","1h"
3734,"PFC_26",16,"OID30188","P41227","NAA10",0.0455,"Cardiometabolic_II","B13805","20212097_SS211647","PASS",1.5824,5.2212,"Plate control","PASS","PFC",37.3024892049247,"24h","24h"
3735,"PFC_03",25,"OID30188","P41227","NAA10",0.0455,"Cardiometabolic_II","B13805","20212097_SS211647","PASS",1.5824,2.5657,"Plate control","PASS","PFC",5.92042194685546,"Baseline","Baseline"
3736,"PFC_11",26,"OID30188","P41227","NAA10",0.0455,"Cardiometabolic_II","B13805","20212097_SS211647","PASS",1.5824,4.7169,"Plate control","PASS","PFC",26.2983430680231,"1h","1h"
3737,"PFC_19",27,"OID30188","P41227","NAA10",0.0455,"Cardiometabolic_II","B13805","20212097_SS211647","PASS",1.5824,4.6079,"Plate control","PASS","PFC",24.3846270133103,"24h","24h"
3738,"PFC_27",28,"OID30188","P41227","NAA10",0.0455,"Cardiometabolic_II","B13805","20212097_SS211647","PASS",1.5824,5.9245,"Plate control","PASS","PFC",60.7368423072612,"24h","24h"
3739,"PFC_04",37,"OID30188","P41227","NAA10",0.0455,"Cardiometabolic_II","B13805","20212097_SS211647","PASS",1.5824,4.7974,"Plate control","PASS","PFC",27.8074587207489,"Baseline","Baseline"
3740,"PFC_12",38,"OID30188","P41227","NAA10",0.0455,"Cardiometabolic_II","B13805","20212097_SS211647","PASS",1.5824,3.3538,"Plate control","PASS","PFC",10.2233775463184,"1h","1h"
3741,"PFC_20",39,"OID30188","P41227","NAA10",0.0455,"Cardiometabolic_II","B13805","20212097_SS211647","PASS",1.5824,5.486,"Plate control","PASS","PFC",44.8178022683906,"24h","24h"
3742,"PFC_05",49,"OID30188","P41227","NAA10",0.0455,"Cardiometabolic_II","B13805","20212097_SS211647","PASS",1.5824,3.868,"Plate control","PASS","PFC",14.6010478166952,"Baseline","Baseline"
3743,"PFC_13",50,"OID30188","P41227","NAA10",0.0455,"Cardiometabolic_II","B13805","20212097_SS211647","PASS",1.5824,5.6562,"Plate control","PASS","PFC",50.4296392550524,"1h","1h"
3744,"PFC_21",51,"OID30188","P41227","NAA10",0.0455,"Cardiometabolic_II","B13805","20212097_SS211647","PASS",1.5824,3.857,"Plate control","PASS","PFC",14.4901437282852,"24h","24h"
3745,"PFC_06",61,"OID30188","P41227","NAA10",0.0455,"Cardiometabolic_II","B13805","20212097_SS211647","PASS",1.5824,4.4679,"Plate control","PASS","PFC",22.1295161098434,"Baseline","Baseline"
3746,"PFC_14",62,"OID30188","P41227","NAA10",0.0455,"Cardiometabolic_II","B13805","20212097_SS211647","PASS",1.5824,4.7135,"Plate control","PASS","PFC",26.2364387256759,"1h","1h"
3747,"PFC_22",63,"OID30188","P41227","NAA10",0.0455,"Cardiometabolic_II","B13805","20212097_SS211647","PASS",1.5824,4.3507,"Plate control","PASS","PFC",20.4028671687523,"24h","24h"
3748,"PFC_07",73,"OID30188","P41227","NAA10",0.0455,"Cardiometabolic_II","B13805","20212097_SS211647","PASS",1.5824,5.1575,"Plate control","PASS","PFC",35.6912865133443,"Baseline","Baseline"
3749,"PFC_15",74,"OID30188","P41227","NAA10",0.0455,"Cardiometabolic_II","B13805","20212097_SS211647","PASS",1.5824,4.2568,"Plate control","PASS","PFC",19.1172088861137,"1h","1h"
3750,"PFC_23",75,"OID30188","P41227","NAA10",0.0455,"Cardiometabolic_II","B13805","20212097_SS211647","PASS",1.5824,5.3534,"Plate control","PASS","PFC",40.882173668406,"24h","24h"
3751,"PFC_08",85,"OID30188","P41227","NAA10",0.0455,"Cardiometabolic_II","B13805","20212097_SS211647","PASS",1.5824,4.863,"Plate control","PASS","PFC",29.1010640576235,"Baseline","Baseline"
3752,"PFC_16",86,"OID30188","P41227","NAA10",0.0455,"Cardiometabolic_II","B13805","20212097_SS211647","PASS",1.5824,5.2794,"Plate control","PASS","PFC",38.8380806377433,"1h","1h"
3753,"PFC_24",87,"OID30188","P41227","NAA10",0.0455,"Cardiometabolic_II","B13805","20212097_SS211647","PASS",1.5824,5.2665,"Plate control","PASS","PFC",38.4923540866419,"24h","24h"
3754,"PFC_01",1,"OID30189","Q86VR7","VSIG10L",0.7273,"Cardiometabolic_II","B13805","20212097_SS211647","WARN",0.1702,-0.0962,"Plate control","PASS","PFC",0.93549380186353,"Baseline","Baseline"
3755,"PFC_09",2,"OID30189","Q86VR7","VSIG10L",0.7273,"Cardiometabolic_II","B13805","20212097_SS211647","PASS",0.1702,0.9936,"Plate control","PASS","PFC",1.99114736637639,"Baseline","Baseline"
3756,"PFC_17",3,"OID30189","Q86VR7","VSIG10L",0.7273,"Cardiometabolic_II","B13805","20212097_SS211647","PASS",0.1702,-0.0947,"Plate control","PASS","PFC",0.936466960019026,"1h","1h"
3757,"PFC_25",4,"OID30189","Q86VR7","VSIG10L",0.7273,"Cardiometabolic_II","B13805","20212097_SS211647","PASS",0.1702,0.1152,"Plate control","PASS","PFC",1.08312518815266,"24h","24h"
3758,"PFC_02",13,"OID30189","Q86VR7","VSIG10L",0.7273,"Cardiometabolic_II","B13805","20212097_SS211647","PASS",0.1702,-0.2712,"Plate control","PASS","PFC",0.828630024012294,"Baseline","Baseline"
3759,"PFC_10",14,"OID30189","Q86VR7","VSIG10L",0.7273,"Cardiometabolic_II","B13805","20212097_SS211647","PASS",0.1702,-0.1311,"Plate control","PASS","PFC",0.913134954126456,"1h","1h"
3760,"PFC_18",15,"OID30189","Q86VR7","VSIG10L",0.7273,"Cardiometabolic_II","B13805","20212097_SS211647","PASS",0.1702,1.068,"Plate control","PASS","PFC",2.09652495103386,"1h","1h"
3761,"PFC_26",16,"OID30189","Q86VR7","VSIG10L",0.7273,"Cardiometabolic_II","B13805","20212097_SS211647","PASS",0.1702,-1.416,"Plate control","PASS","PFC",0.374749900438887,"24h","24h"
3762,"PFC_03",25,"OID30189","Q86VR7","VSIG10L",0.7273,"Cardiometabolic_II","B13805","20212097_SS211647","PASS",0.1702,0.097,"Plate control","PASS","PFC",1.06954708788524,"Baseline","Baseline"
3763,"PFC_11",26,"OID30189","Q86VR7","VSIG10L",0.7273,"Cardiometabolic_II","B13805","20212097_SS211647","PASS",0.1702,-0.4395,"Plate control","PASS","PFC",0.737390124308321,"1h","1h"
3764,"PFC_19",27,"OID30189","Q86VR7","VSIG10L",0.7273,"Cardiometabolic_II","B13805","20212097_SS211647","PASS",0.1702,0.2513,"Plate control","PASS","PFC",1.19027918217192,"24h","24h"
3765,"PFC_27",28,"OID30189","Q86VR7","VSIG10L",0.7273,"Cardiometabolic_II","B13805","20212097_SS211647","PASS",0.1702,0.6079,"Plate control","PASS","PFC",1.52403918833189,"24h","24h"
3766,"PFC_04",37,"OID30189","Q86VR7","VSIG10L",0.7273,"Cardiometabolic_II","B13805","20212097_SS211647","PASS",0.1702,0.6545,"Plate control","PASS","PFC",1.57407032738941,"Baseline","Baseline"
3767,"PFC_12",38,"OID30189","Q86VR7","VSIG10L",0.7273,"Cardiometabolic_II","B13805","20212097_SS211647","PASS",0.1702,-0.796,"Plate control","PASS","PFC",0.575943821174679,"1h","1h"
3768,"PFC_20",39,"OID30189","Q86VR7","VSIG10L",0.7273,"Cardiometabolic_II","B13805","20212097_SS211647","PASS",0.1702,-0.6501,"Plate control","PASS","PFC",0.637236142285248,"24h","24h"
3769,"PFC_05",49,"OID30189","Q86VR7","VSIG10L",0.7273,"Cardiometabolic_II","B13805","20212097_SS211647","PASS",0.1702,-1.3743,"Plate control","PASS","PFC",0.385739823083072,"Baseline","Baseline"
3770,"PFC_13",50,"OID30189","Q86VR7","VSIG10L",0.7273,"Cardiometabolic_II","B13805","20212097_SS211647","PASS",0.1702,-0.088,"Plate control","PASS","PFC",0.940826107513788,"1h","1h"
3771,"PFC_21",51,"OID30189","Q86VR7","VSIG10L",0.7273,"Cardiometabolic_II","B13805","20212097_SS211647","PASS",0.1702,0.1366,"Plate control","PASS","PFC",1.09931131324232,"24h","24h"
3772,"PFC_06",61,"OID30189","Q86VR7","VSIG10L",0.7273,"Cardiometabolic_II","B13805","20212097_SS211647","PASS",0.1702,-0.6279,"Plate control","PASS","PFC",0.647117680466485,"Baseline","Baseline"
3773,"PFC_14",62,"OID30189","Q86VR7","VSIG10L",0.7273,"Cardiometabolic_II","B13805","20212097_SS211647","PASS",0.1702,-0.0679,"Plate control","PASS","PFC",0.954025676276558,"1h","1h"
3774,"PFC_22",63,"OID30189","Q86VR7","VSIG10L",0.7273,"Cardiometabolic_II","B13805","20212097_SS211647","PASS",0.1702,0.5169,"Plate control","PASS","PFC",1.43087733572639,"24h","24h"
3775,"PFC_07",73,"OID30189","Q86VR7","VSIG10L",0.7273,"Cardiometabolic_II","B13805","20212097_SS211647","PASS",0.1702,-0.3808,"Plate control","PASS","PFC",0.768011596486069,"Baseline","Baseline"
3776,"PFC_15",74,"OID30189","Q86VR7","VSIG10L",0.7273,"Cardiometabolic_II","B13805","20212097_SS211647","PASS",0.1702,-0.5946,"Plate control","PASS","PFC",0.662228038345561,"1h","1h"
3777,"PFC_23",75,"OID30189","Q86VR7","VSIG10L",0.7273,"Cardiometabolic_II","B13805","20212097_SS211647","PASS",0.1702,-0.1274,"Plate control","PASS","PFC",0.915479826323081,"24h","24h"
3778,"PFC_08",85,"OID30189","Q86VR7","VSIG10L",0.7273,"Cardiometabolic_II","B13805","20212097_SS211647","PASS",0.1702,0.0933,"Plate control","PASS","PFC",1.0668075941714,"Baseline","Baseline"
3779,"PFC_16",86,"OID30189","Q86VR7","VSIG10L",0.7273,"Cardiometabolic_II","B13805","20212097_SS211647","PASS",0.1702,-0.3512,"Plate control","PASS","PFC",0.783931770523245,"1h","1h"
3780,"PFC_24",87,"OID30189","Q86VR7","VSIG10L",0.7273,"Cardiometabolic_II","B13805","20212097_SS211647","PASS",0.1702,-0.0396,"Plate control","PASS","PFC",0.972924662014724,"24h","24h"
3781,"PFC_01",1,"OID30190","Q93052","LPP",0.0682,"Cardiometabolic_II","B13805","20212097_SS211647","WARN",1.4803,2.2257,"Plate control","PASS","PFC",4.67737792167842,"Baseline","Baseline"
3782,"PFC_09",2,"OID30190","Q93052","LPP",0.0682,"Cardiometabolic_II","B13805","20212097_SS211647","PASS",1.4803,3.0595,"Plate control","PASS","PFC",8.3368362587151,"Baseline","Baseline"
3783,"PFC_17",3,"OID30190","Q93052","LPP",0.0682,"Cardiometabolic_II","B13805","20212097_SS211647","PASS",1.4803,4.1584,"Plate control","PASS","PFC",17.8567794214393,"1h","1h"
3784,"PFC_25",4,"OID30190","Q93052","LPP",0.0682,"Cardiometabolic_II","B13805","20212097_SS211647","PASS",1.4803,3.3316,"Plate control","PASS","PFC",10.0672657623036,"24h","24h"
3785,"PFC_02",13,"OID30190","Q93052","LPP",0.0682,"Cardiometabolic_II","B13805","20212097_SS211647","PASS",1.4803,2.8966,"Plate control","PASS","PFC",7.44669361052021,"Baseline","Baseline"
3786,"PFC_10",14,"OID30190","Q93052","LPP",0.0682,"Cardiometabolic_II","B13805","20212097_SS211647","PASS",1.4803,2.7162,"Plate control","PASS","PFC",6.57139653187018,"1h","1h"
3787,"PFC_18",15,"OID30190","Q93052","LPP",0.0682,"Cardiometabolic_II","B13805","20212097_SS211647","PASS",1.4803,3.5741,"Plate control","PASS","PFC",11.9099875501511,"1h","1h"
3788,"PFC_26",16,"OID30190","Q93052","LPP",0.0682,"Cardiometabolic_II","B13805","20212097_SS211647","PASS",1.4803,3.4792,"Plate control","PASS","PFC",11.1517637650691,"24h","24h"
3789,"PFC_03",25,"OID30190","Q93052","LPP",0.0682,"Cardiometabolic_II","B13805","20212097_SS211647","PASS",1.4803,2.157,"Plate control","PASS","PFC",4.45986487491079,"Baseline","Baseline"
3790,"PFC_11",26,"OID30190","Q93052","LPP",0.0682,"Cardiometabolic_II","B13805","20212097_SS211647","PASS",1.4803,3.9869,"Plate control","PASS","PFC",15.8553739633596,"1h","1h"
3791,"PFC_19",27,"OID30190","Q93052","LPP",0.0682,"Cardiometabolic_II","B13805","20212097_SS211647","PASS",1.4803,3.024,"Plate control","PASS","PFC",8.13419738633565,"24h","24h"
3792,"PFC_27",28,"OID30190","Q93052","LPP",0.0682,"Cardiometabolic_II","B13805","20212097_SS211647","PASS",1.4803,5.1222,"Plate control","PASS","PFC",34.8285860660581,"24h","24h"
3793,"PFC_04",37,"OID30190","Q93052","LPP",0.0682,"Cardiometabolic_II","B13805","20212097_SS211647","PASS",1.4803,3.711,"Plate control","PASS","PFC",13.0955069054529,"Baseline","Baseline"
3794,"PFC_12",38,"OID30190","Q93052","LPP",0.0682,"Cardiometabolic_II","B13805","20212097_SS211647","PASS",1.4803,2.4299,"Plate control","PASS","PFC",5.38856078794597,"1h","1h"
3795,"PFC_20",39,"OID30190","Q93052","LPP",0.0682,"Cardiometabolic_II","B13805","20212097_SS211647","PASS",1.4803,4.6224,"Plate control","PASS","PFC",24.630943723619,"24h","24h"
3796,"PFC_05",49,"OID30190","Q93052","LPP",0.0682,"Cardiometabolic_II","B13805","20212097_SS211647","PASS",1.4803,1.8204,"Plate control","PASS","PFC",3.53179106983534,"Baseline","Baseline"
3797,"PFC_13",50,"OID30190","Q93052","LPP",0.0682,"Cardiometabolic_II","B13805","20212097_SS211647","PASS",1.4803,3.72,"Plate control","PASS","PFC",13.1774562762812,"1h","1h"
3798,"PFC_21",51,"OID30190","Q93052","LPP",0.0682,"Cardiometabolic_II","B13805","20212097_SS211647","PASS",1.4803,2.6955,"Plate control","PASS","PFC",6.47778237168406,"24h","24h"
3799,"PFC_06",61,"OID30190","Q93052","LPP",0.0682,"Cardiometabolic_II","B13805","20212097_SS211647","PASS",1.4803,3.8622,"Plate control","PASS","PFC",14.5424657372682,"Baseline","Baseline"
3800,"PFC_14",62,"OID30190","Q93052","LPP",0.0682,"Cardiometabolic_II","B13805","20212097_SS211647","PASS",1.4803,3.4951,"Plate control","PASS","PFC",11.2753475615876,"1h","1h"
3801,"PFC_22",63,"OID30190","Q93052","LPP",0.0682,"Cardiometabolic_II","B13805","20212097_SS211647","PASS",1.4803,3.0552,"Plate control","PASS","PFC",8.3120250378582,"24h","24h"
3802,"PFC_07",73,"OID30190","Q93052","LPP",0.0682,"Cardiometabolic_II","B13805","20212097_SS211647","PASS",1.4803,4.0209,"Plate control","PASS","PFC",16.2334754875196,"Baseline","Baseline"
3803,"PFC_15",74,"OID30190","Q93052","LPP",0.0682,"Cardiometabolic_II","B13805","20212097_SS211647","PASS",1.4803,3.016,"Plate control","PASS","PFC",8.08921664681043,"1h","1h"
3804,"PFC_23",75,"OID30190","Q93052","LPP",0.0682,"Cardiometabolic_II","B13805","20212097_SS211647","PASS",1.4803,3.8655,"Plate control","PASS","PFC",14.5757680384615,"24h","24h"
3805,"PFC_08",85,"OID30190","Q93052","LPP",0.0682,"Cardiometabolic_II","B13805","20212097_SS211647","PASS",1.4803,3.0957,"Plate control","PASS","PFC",8.54867009887674,"Baseline","Baseline"
3806,"PFC_16",86,"OID30190","Q93052","LPP",0.0682,"Cardiometabolic_II","B13805","20212097_SS211647","PASS",1.4803,3.336,"Plate control","PASS","PFC",10.0980162568969,"1h","1h"
3807,"PFC_24",87,"OID30190","Q93052","LPP",0.0682,"Cardiometabolic_II","B13805","20212097_SS211647","PASS",1.4803,4.3118,"Plate control","PASS","PFC",19.8600864809822,"24h","24h"
3808,"PFC_01",1,"OID30191","O75427","LRCH4",0,"Cardiometabolic_II","B13805","20212097_SS211647","WARN",-2.562,-0.0755,"Plate control","PASS","PFC",0.949013161050957,"Baseline","Baseline"

Supplemental table 1_Olink proximity extension assay (PEA) data

,"SampleID","Index","OlinkID","UniProt","Assay","MissingFreq","Panel","Panel_Lot_Nr","PlateID","QC_Warning","LOD","NPX","Normalization","Assay_Warning","condition","Value","group","alt_group"
3809,"PFC_09",2,"OID30191","O75427","LRCH4",0,"Cardiometabolic_II","B13805","20212097_SS211647","PASS",-2.562,0.2202,"Plate control","PASS","PFC",1.16489506402128,"Baseline","Baseline"
3810,"PFC_17",3,"OID30191","O75427","LRCH4",0,"Cardiometabolic_II","B13805","20212097_SS211647","PASS",-2.562,1.6256,"Plate control","PASS","PFC",3.08570469250096,"1h","1h"
3811,"PFC_25",4,"OID30191","O75427","LRCH4",0,"Cardiometabolic_II","B13805","20212097_SS211647","PASS",-2.562,0.5378,"Plate control","PASS","PFC",1.4517570096976,"24h","24h"
3812,"PFC_02",13,"OID30191","O75427","LRCH4",0,"Cardiometabolic_II","B13805","20212097_SS211647","PASS",-2.562,0.4202,"Plate control","PASS","PFC",1.33811304378541,"Baseline","Baseline"
3813,"PFC_10",14,"OID30191","O75427","LRCH4",0,"Cardiometabolic_II","B13805","20212097_SS211647","PASS",-2.562,0.2906,"Plate control","PASS","PFC",1.22314886523934,"1h","1h"
3814,"PFC_18",15,"OID30191","O75427","LRCH4",0,"Cardiometabolic_II","B13805","20212097_SS211647","PASS",-2.562,0.9738,"Plate control","PASS","PFC",1.96400690248424,"1h","1h"
3815,"PFC_26",16,"OID30191","O75427","LRCH4",0,"Cardiometabolic_II","B13805","20212097_SS211647","PASS",-2.562,1.2991,"Plate control","PASS","PFC",2.46075325000877,"24h","24h"
3816,"PFC_03",25,"OID30191","O75427","LRCH4",0,"Cardiometabolic_II","B13805","20212097_SS211647","PASS",-2.562,0.0161,"Plate control","PASS","PFC",1.01122217100181,"Baseline","Baseline"
3817,"PFC_11",26,"OID30191","O75427","LRCH4",0,"Cardiometabolic_II","B13805","20212097_SS211647","PASS",-2.562,1.017,"Plate control","PASS","PFC",2.02370640205386,"1h","1h"
3818,"PFC_19",27,"OID30191","O75427","LRCH4",0,"Cardiometabolic_II","B13805","20212097_SS211647","PASS",-2.562,0.4065,"Plate control","PASS","PFC",1.3254663090689,"24h","24h"
3819,"PFC_27",28,"OID30191","O75427","LRCH4",0,"Cardiometabolic_II","B13805","20212097_SS211647","PASS",-2.562,2.1365,"Plate control","PASS","PFC",4.39694046971745,"24h","24h"
3820,"PFC_04",37,"OID30191","O75427","LRCH4",0,"Cardiometabolic_II","B13805","20212097_SS211647","PASS",-2.562,0.9364,"Plate control","PASS","PFC",1.91374684592553,"Baseline","Baseline"
3821,"PFC_12",38,"OID30191","O75427","LRCH4",0,"Cardiometabolic_II","B13805","20212097_SS211647","PASS",-2.562,-0.1757,"Plate control","PASS","PFC",0.885337846269121,"1h","1h"
3822,"PFC_20",39,"OID30191","O75427","LRCH4",0,"Cardiometabolic_II","B13805","20212097_SS211647","PASS",-2.562,1.466,"Plate control","PASS","PFC",2.76254889628771,"24h","24h"
3823,"PFC_05",49,"OID30191","O75427","LRCH4",0,"Cardiometabolic_II","B13805","20212097_SS211647","PASS",-2.562,0.111,"Plate control","PASS","PFC",1.07997655983288,"Baseline","Baseline"
3824,"PFC_13",50,"OID30191","O75427","LRCH4",0,"Cardiometabolic_II","B13805","20212097_SS211647","PASS",-2.562,0.8553,"Plate control","PASS","PFC",1.80913491504038,"1h","1h"
3825,"PFC_21",51,"OID30191","O75427","LRCH4",0,"Cardiometabolic_II","B13805","20212097_SS211647","PASS",-2.562,0.1943,"Plate control","PASS","PFC",1.1441688716964,"24h","24h"
3826,"PFC_06",61,"OID30191","O75427","LRCH4",0,"Cardiometabolic_II","B13805","20212097_SS211647","PASS",-2.562,0.6807,"Plate control","PASS","PFC",1.60291730685712,"Baseline","Baseline"
3827,"PFC_14",62,"OID30191","O75427","LRCH4",0,"Cardiometabolic_II","B13805","20212097_SS211647","PASS",-2.562,1.2929,"Plate control","PASS","PFC",2.45020082294396,"1h","1h"
3828,"PFC_22",63,"OID30191","O75427","LRCH4",0,"Cardiometabolic_II","B13805","20212097_SS211647","PASS",-2.562,0.2943,"Plate control","PASS","PFC",1.22628983334429,"24h","24h"
3829,"PFC_07",73,"OID30191","O75427","LRCH4",0,"Cardiometabolic_II","B13805","20212097_SS211647","PASS",-2.562,0.7254,"Plate control","PASS","PFC",1.6533589812119,"Baseline","Baseline"
3830,"PFC_15",74,"OID30191","O75427","LRCH4",0,"Cardiometabolic_II","B13805","20212097_SS211647","PASS",-2.562,0.3697,"Plate control","PASS","PFC",1.29208412136047,"1h","1h"
3831,"PFC_23",75,"OID30191","O75427","LRCH4",0,"Cardiometabolic_II","B13805","20212097_SS211647","PASS",-2.562,1.5524,"Plate control","PASS","PFC",2.93304661485016,"24h","24h"
3832,"PFC_08",85,"OID30191","O75427","LRCH4",0,"Cardiometabolic_II","B13805","20212097_SS211647","PASS",-2.562,0.9867,"Plate control","PASS","PFC",1.98164701176949,"Baseline","Baseline"
3833,"PFC_16",86,"OID30191","O75427","LRCH4",0,"Cardiometabolic_II","B13805","20212097_SS211647","PASS",-2.562,0.5025,"Plate control","PASS","PFC",1.41666633228118,"1h","1h"
3834,"PFC_24",87,"OID30191","O75427","LRCH4",0,"Cardiometabolic_II","B13805","20212097_SS211647","PASS",-2.562,1.301,"Plate control","PASS","PFC",2.46399614689151,"24h","24h"
3835,"PFC_01",1,"OID30192","P35609","ACTN2",0.3636,"Cardiometabolic_II","B13805","20212097_SS211647","WARN",-0.6259,-1.3162,"Plate control","PASS","PFC",0.401591322140887,"Baseline","Baseline"
3836,"PFC_09",2,"OID30192","P35609","ACTN2",0.3636,"Cardiometabolic_II","B13805","20212097_SS211647","PASS",-0.6259,0.6957,"Plate control","PASS","PFC",1.61967011131244,"Baseline","Baseline"
3837,"PFC_17",3,"OID30192","P35609","ACTN2",0.3636,"Cardiometabolic_II","B13805","20212097_SS211647","PASS",-0.6259,-1.4496,"Plate control","PASS","PFC",0.366122920743419,"1h","1h"
3838,"PFC_25",4,"OID30192","P35609","ACTN2",0.3636,"Cardiometabolic_II","B13805","20212097_SS211647","PASS",-0.6259,-1.5123,"Plate control","PASS","PFC",0.35055190986062,"24h","24h"
3839,"PFC_02",13,"OID30192","P35609","ACTN2",0.3636,"Cardiometabolic_II","B13805","20212097_SS211647","PASS",-0.6259,-0.3295,"Plate control","PASS","PFC",0.79581224347305,"Baseline","Baseline"
3840,"PFC_10",14,"OID30192","P35609","ACTN2",0.3636,"Cardiometabolic_II","B13805","20212097_SS211647","PASS",-0.6259,-1.0346,"Plate control","PASS","PFC",0.488151205879966,"1h","1h"
3841,"PFC_18",15,"OID30192","P35609","ACTN2",0.3636,"Cardiometabolic_II","B13805","20212097_SS211647","PASS",-0.6259,-0.1995,"Plate control","PASS","PFC",0.870852325418737,"1h","1h"
3842,"PFC_26",16,"OID30192","P35609","ACTN2",0.3636,"Cardiometabolic_II","B13805","20212097_SS211647","PASS",-0.6259,-0.8438,"Plate control","PASS","PFC",0.557174060781857,"24h","24h"
3843,"PFC_03",25,"OID30192","P35609","ACTN2",0.3636,"Cardiometabolic_II","B13805","20212097_SS211647","PASS",-0.6259,-0.2829,"Plate control","PASS","PFC",0.82193715762728,"Baseline","Baseline"
3844,"PFC_11",26,"OID30192","P35609","ACTN2",0.3636,"Cardiometabolic_II","B13805","20212097_SS211647","PASS",-0.6259,0.757,"Plate control","PASS","PFC",1.68997276882503,"1h","1h"
3845,"PFC_19",27,"OID30192","P35609","ACTN2",0.3636,"Cardiometabolic_II","B13805","20212097_SS211647","PASS",-0.6259,-1.0574,"Plate control","PASS","PFC",0.480497223605843,"24h","24h"
3846,"PFC_27",28,"OID30192","P35609","ACTN2",0.3636,"Cardiometabolic_II","B13805","20212097_SS211647","PASS",-0.6259,-1.0806,"Plate control","PASS","PFC",0.472832137108247,"24h","24h"
3847,"PFC_04",37,"OID30192","P35609","ACTN2",0.3636,"Cardiometabolic_II","B13805","20212097_SS211647","PASS",-0.6259,-0.926,"Plate control","PASS","PFC",0.526315577354719,"Baseline","Baseline"
3848,"PFC_12",38,"OID30192","P35609","ACTN2",0.3636,"Cardiometabolic_II","B13805","20212097_SS211647","PASS",-0.6259,0.6328,"Plate control","PASS","PFC",1.550571442949,"1h","1h"
3849,"PFC_20",39,"OID30192","P35609","ACTN2",0.3636,"Cardiometabolic_II","B13805","20212097_SS211647","PASS",-0.6259,0.0693,"Plate control","PASS","PFC",1.04920748145453,"24h","24h"
3850,"PFC_05",49,"OID30192","P35609","ACTN2",0.3636,"Cardiometabolic_II","B13805","20212097_SS211647","PASS",-0.6259,0.8049,"Plate control","PASS","PFC",1.74702469311244,"Baseline","Baseline"
3851,"PFC_13",50,"OID30192","P35609","ACTN2",0.3636,"Cardiometabolic_II","B13805","20212097_SS211647","PASS",-0.6259,-0.6346,"Plate control","PASS","PFC",0.644119377811943,"1h","1h"
3852,"PFC_21",51,"OID30192","P35609","ACTN2",0.3636,"Cardiometabolic_II","B13805","20212097_SS211647","PASS",-0.6259,0.6746,"Plate control","PASS","PFC",1.59615416345421,"24h","24h"
3853,"PFC_06",61,"OID30192","P35609","ACTN2",0.3636,"Cardiometabolic_II","B13805","20212097_SS211647","PASS",-0.6259,-0.8213,"Plate control","PASS","PFC",0.565931756618379,"Baseline","Baseline"
3854,"PFC_14",62,"OID30192","P35609","ACTN2",0.3636,"Cardiometabolic_II","B13805","20212097_SS211647","PASS",-0.6259,0.3387,"Plate control","PASS","PFC",1.26461654539267,"1h","1h"
3855,"PFC_22",63,"OID30192","P35609","ACTN2",0.3636,"Cardiometabolic_II","B13805","20212097_SS211647","PASS",-0.6259,-0.7654,"Plate control","PASS","PFC",0.588290237307892,"24h","24h"
3856,"PFC_07",73,"OID30192","P35609","ACTN2",0.3636,"Cardiometabolic_II","B13805","20212097_SS211647","PASS",-0.6259,-1.0937,"Plate control","PASS","PFC",0.468558147234112,"Baseline","Baseline"
3857,"PFC_15",74,"OID30192","P35609","ACTN2",0.3636,"Cardiometabolic_II","B13805","20212097_SS211647","PASS",-0.6259,-0.3935,"Plate control","PASS","PFC",0.761280484415716,"1h","1h"
3858,"PFC_23",75,"OID30192","P35609","ACTN2",0.3636,"Cardiometabolic_II","B13805","20212097_SS211647","PASS",-0.6259,0.9312,"Plate control","PASS","PFC",1.90686141937733,"24h","24h"
3859,"PFC_08",85,"OID30192","P35609","ACTN2",0.3636,"Cardiometabolic_II","B13805","20212097_SS211647","PASS",-0.6259,-0.9451,"Plate control","PASS","PFC",0.519393548783688,"Baseline","Baseline"
3860,"PFC_16",86,"OID30192","P35609","ACTN2",0.3636,"Cardiometabolic_II","B13805","20212097_SS211647","PASS",-0.6259,-1.2742,"Plate control","PASS","PFC",0.413454363303299,"1h","1h"
3861,"PFC_24",87,"OID30192","P35609","ACTN2",0.3636,"Cardiometabolic_II","B13805","20212097_SS211647","PASS",-0.6259,-0.4686,"Plate control","PASS","PFC",0.722665536619461,"24h","24h"
3862,"PFC_01",1,"OID30246","Q0VD83","APOBR",0,"Cardiometabolic_II","B13805","20212097_SS211647","PASS",-5.3422,0.0964,"Plate control","PASS","PFC",1.06910236823944,"Baseline","Baseline"
3863,"PFC_09",2,"OID30246","Q0VD83","APOBR",0,"Cardiometabolic_II","B13805","20212097_SS211647","PASS",-5.3422,0.4284,"Plate control","PASS","PFC",1.34574027523243,"Baseline","Baseline"
3864,"PFC_17",3,"OID30246","Q0VD83","APOBR",0,"Cardiometabolic_II","B13805","20212097_SS211647","PASS",-5.3422,0.2216,"Plate control","PASS","PFC",1.16602603390352,"1h","1h"
3865,"PFC_25",4,"OID30246","Q0VD83","APOBR",0,"Cardiometabolic_II","B13805","20212097_SS211647","PASS",-5.3422,-0.2936,"Plate control","PASS","PFC",0.815863667423493,"24h","24h"
3866,"PFC_02",13,"OID30246","Q0VD83","APOBR",0,"Cardiometabolic_II","B13805","20212097_SS211647","PASS",-5.3422,-0.3658,"Plate control","PASS","PFC",0.776038428587957,"Baseline","Baseline"
3867,"PFC_10",14,"OID30246","Q0VD83","APOBR",0,"Cardiometabolic_II","B13805","20212097_SS211647","PASS",-5.3422,0.1779,"Plate control","PASS","PFC",1.13123604882053,"1h","1h"
3868,"PFC_18",15,"OID30246","Q0VD83","APOBR",0,"Cardiometabolic_II","B13805","20212097_SS211647","PASS",-5.3422,0.3498,"Plate control","PASS","PFC",1.27438394794497,"1h","1h"
3869,"PFC_26",16,"OID30246","Q0VD83","APOBR",0,"Cardiometabolic_II","B13805","20212097_SS211647","PASS",-5.3422,0.1672,"Plate control","PASS","PFC",1.12287707510006,"24h","24h"
3870,"PFC_03",25,"OID30246","Q0VD83","APOBR",0,"Cardiometabolic_II","B13805","20212097_SS211647","PASS",-5.3422,0.0878,"Plate control","PASS","PFC",1.06274833594353,"Baseline","Baseline"
3871,"PFC_11",26,"OID30246","Q0VD83","APOBR",0,"Cardiometabolic_II","B13805","20212097_SS211647","PASS",-5.3422,-0.4567,"Plate control","PASS","PFC",0.72865105992472,"1h","1h"
3872,"PFC_19",27,"OID30246","Q0VD83","APOBR",0,"Cardiometabolic_II","B13805","20212097_SS211647","PASS",-5.3422,0.2609,"Plate control","PASS","PFC",1.19822596376605,"24h","24h"
3873,"PFC_27",28,"OID30246","Q0VD83","APOBR",0,"Cardiometabolic_II","B13805","20212097_SS211647","PASS",-5.3422,0.3972,"Plate control","PASS","PFC",1.31694947736862,"24h","24h"
3874,"PFC_04",37,"OID30246","Q0VD83","APOBR",0,"Cardiometabolic_II","B13805","20212097_SS211647","PASS",-5.3422,0.0332,"Plate control","PASS","PFC",1.02327931653677,"Baseline","Baseline"
3875,"PFC_12",38,"OID30246","Q0VD83","APOBR",0,"Cardiometabolic_II","B13805","20212097_SS211647","PASS",-5.3422,0.3346,"Plate control","PASS","PFC",1.26102772923155,"1h","1h"
3876,"PFC_20",39,"OID30246","Q0VD83","APOBR",0,"Cardiometabolic_II","B13805","20212097_SS211647","PASS",-5.3422,-0.2875,"Plate control","PASS","PFC",0.81932060354304,"24h","24h"
3877,"PFC_05",49,"OID30246","Q0VD83","APOBR",0,"Cardiometabolic_II","B13805","20212097_SS211647","PASS",-5.3422,0.2342,"Plate control","PASS","PFC",1.17625430245946,"Baseline","Baseline"
3878,"PFC_13",50,"OID30246","Q0VD83","APOBR",0,"Cardiometabolic_II","B13805","20212097_SS211647","PASS",-5.3422,-0.192,"Plate control","PASS","PFC",0.875391329694264,"1h","1h"
3879,"PFC_21",51,"OID30246","Q0VD83","APOBR",0,"Cardiometabolic_II","B13805","20212097_SS211647","PASS",-5.3422,0.2631,"Plate control","PASS","PFC",1.20005456093398,"24h","24h"
3880,"PFC_06",61,"OID30246","Q0VD83","APOBR",0,"Cardiometabolic_II","B13805","20212097_SS211647","PASS",-5.3422,-0.1854,"Plate control","PASS","PFC",0.879405219218972,"Baseline","Baseline"
3881,"PFC_14",62,"OID30246","Q0VD83","APOBR",0,"Cardiometabolic_II","B13805","20212097_SS211647","PASS",-5.3422,0.2272,"Plate control","PASS","PFC",1.17056090442523,"1h","1h"
3882,"PFC_22",63,"OID30246","Q0VD83","APOBR",0,"Cardiometabolic_II","B13805","20212097_SS211647","PASS",-5.3422,-0.061,"Plate control","PASS","PFC",0.958599438492977,"24h","24h"
3883,"PFC_07",73,"OID30246","Q0VD83","APOBR",0,"Cardiometabolic_II","B13805","20212097_SS211647","PASS",-5.3422,0.0811,"Plate control","PASS","PFC",1.0578242838693,"Baseline","Baseline"
3884,"PFC_15",74,"OID30246","Q0VD83","APOBR",0,"Cardiometabolic_II","B13805","20212097_SS211647","PASS",-5.3422,-0.4067,"Plate control","PASS","PFC",0.754346884059904,"1h","1h"
3885,"PFC_23",75,"OID30246","Q0VD83","APOBR",0,"Cardiometabolic_II","B13805","20212097_SS211647","PASS",-5.3422,0.0489,"Plate control","PASS","PFC",1.03447587463018,"24h","24h"
3886,"PFC_08",85,"OID30246","Q0VD83","APOBR",0,"Cardiometabolic_II","B13805","20212097_SS211647","PASS",-5.3422,0.0374,"Plate control","PASS","PFC",1.02626264631903,"Baseline","Baseline"
3887,"PFC_16",86,"OID30246","Q0VD83","APOBR",0,"Cardiometabolic_II","B13805","20212097_SS211647","PASS",-5.3422,-0.2029,"Plate control","PASS","PFC",0.868802403857248,"1h","1h"
3888,"PFC_24",87,"OID30246","Q0VD83","APOBR",0,"Cardiometabolic_II","B13805","20212097_SS211647","PASS",-5.3422,-0.3404,"Plate control","PASS","PFC",0.78982229626365\2,"24h","24h"
3889,"PFC_01",1,"OID30203","O43423","ANP32C",0.8409,"Cardiometabolic_II","B13805","20212097_SS211647","PASS",0.7069,0.1939,"Plate control","PASS","PFC",1.14385168469889,"Baseline","Baseline"
3890,"PFC_09",2,"OID30203","O43423","ANP32C",0.8409,"Cardiometabolic_II","B13805","20212097_SS211647","PASS",0.7069,-0.1823,"Plate control","PASS","PFC",0.881296878317938,"Baseline","Baseline"
3891,"PFC_17",3,"OID30203","O43423","ANP32C",0.8409,"Cardiometabolic_II","B13805","20212097_SS211647","PASS",0.7069,7.4665,"Plate control","PASS","PFC",176.864415277156,"1h","1h"
3892,"PFC_25",4,"OID30203","O43423","ANP32C",0.8409,"Cardiometabolic_II","B13805","20212097_SS211647","PASS",0.7069,0.1125,"Plate control","PASS","PFC",1.08110001783426,"24h","24h"
3893,"PFC_02",13,"OID30203","O43423","ANP32C",0.8409,"Cardiometabolic_II","B13805","20212097_SS211647","PASS",0.7069,0.2985,"Plate control","PASS","PFC",1.22986503214132,"Baseline","Baseline"
3894,"PFC_10",14,"OID30203","O43423","ANP32C",0.8409,"Cardiometabolic_II","B13805","20212097_SS211647","PASS",0.7069,0.4239,"Plate control","PASS","PFC",1.34154923255262,"1h","1h"
3895,"PFC_18",15,"OID30203","O43423","ANP32C",0.8409,"Cardiometabolic_II","B13805","20212097_SS211647","PASS",0.7069,0.0126,"Plate control","PASS","PFC",1.00877190410723,"1h","1h"
3896,"PFC_26",16,"OID30203","O43423","ANP32C",0.8409,"Cardiometabolic_II","B13805","20212097_SS211647","PASS",0.7069,7.5982,"Plate control","PASS","PFC",193.76980983772,"24h","24h"
3897,"PFC_03",25,"OID30203","O43423","ANP32C",0.8409,"Cardiometabolic_II","B13805","20212097_SS211647","PASS",0.7069,1.7568,"Plate control","PASS","PFC",3.37947701018268,"Baseline","Baseline"
3898,"PFC_11",26,"OID30203","O43423","ANP32C",0.8409,"Cardiometabolic_II","B13805","20212097_SS211647","PASS",0.7069,-0.1494,"Plate control","PASS","PFC",0.901625360093449,"1h","1h"
3899,"PFC_19",27,"OID30203","O43423","ANP32C",0.8409,"Cardiometabolic_II","B13805","20212097_SS211647","PASS",0.7069,0.0092,"Plate control","PASS","PFC",1.00639733012208,"24h","24h"
3900,"PFC_27",28,"OID30203","O43423","ANP32C",0.8409,"Cardiometabolic_II","B13805","20212097_SS211647","PASS",0.7069,0.0761,"Plate control","PASS","PFC",1.05416448987281,"24h","24h"
3901,"PFC_04",37,"OID30203","O43423","ANP32C",0.8409,"Cardiometabolic_II","B13805","20212097_SS211647","PASS",0.7069,0.2648,"Plate control","PASS","PFC",1.20146947894495,"Baseline","Baseline"
3902,"PFC_12",38,"OID30203","O43423","ANP32C",0.8409,"Cardiometabolic_II","B13805","20212097_SS211647","PASS",0.7069,1.8284,"Plate control","PASS","PFC",3.55142987802976,"1h","1h"
3903,"PFC_20",39,"OID30203","O43423","ANP32C",0.8409,"Cardiometabolic_II","B13805","20212097_SS211647","PASS",0.7069,-0.4515,"Plate control","PASS","PFC",0.731282123357752,"24h","24h"
3904,"PFC_05",49,"OID30203","O43423","ANP32C",0.8409,"Cardiometabolic_II","B13805","20212097_SS211647","PASS",0.7069,-0.2975,"Plate control","PASS","PFC",0.81366114273553\6,"Baseline","Baseline"
3905,"PFC_13",50,"OID30203","O43423","ANP32C",0.8409,"Cardiometabolic_II","B13805","20212097_SS211647","PASS",0.7069,-0.1261,"Plate control","PASS","PFC",0.91630512904242,"1h","1h"
3906,"PFC_21",51,"OID30203","O43423","ANP32C",0.8409,"Cardiometabolic_II","B13805","20212097_SS211647","PASS",0.7069,1.3865,"Plate control","PASS","PFC",2.6144364454157,"24h","24h"
3907,"PFC_06",61,"OID30203","O43423","ANP32C",0.8409,"Cardiometabolic_II","B13805","20212097_SS211647","PASS",0.7069,0.1502,"Plate control","PASS","PFC",1.1097233017205,"Baseline","Baseline"
3908,"PFC_14",62,"OID30203","O43423","ANP32C",0.8409,"Cardiometabolic_II","B13805","20212097_SS211647","PASS",0.7069,0.3357,"Plate control","PASS","PFC",1.26198958146944,"1h","1h"
3909,"PFC_22",63,"OID30203","O43423","ANP32C",0.8409,"Cardiometabolic_II","B13805","20212097_SS211647","PASS",0.7069,-0.2508,"Plate control","PASS","PFC",0.84043025252991,"24h","24h"
3910,"PFC_07",73,"OID30203","O43423","ANP32C",0.8409,"Cardiometabolic_II","B13805","20212097_SS211647","PASS",0.7069,-0.077,"Plate control","PASS","PFC",0.948026965128645,"Baseline","Baseline"
3911,"PFC_15",74,"OID30203","O43423","ANP32C",0.8409,"Cardiometabolic_II","B13805","20212097_SS211647","PASS",0.7069,0.0035,"Plate control","PASS","PFC",1.00242896028785,"1h","1h"
3912,"PFC_23",75,"OID30203","O43423","ANP32C",0.8409,"Cardiometabolic_II","B13805","20212097_SS211647","PASS",0.7069,-0.1249,"Plate control","PASS","PFC",0.9170676072843\2,"24h","24h"
3913,"PFC_08",85,"OID30203","O43423","ANP32C",0.8409,"Cardiometabolic_II","B13805","20212097_SS211647","PASS",0.7069,7.3544,"Plate control","PASS","PFC",163.642083420283,"Baseline","Baseline"
3914,"PFC_16",86,"OID30203","O43423","ANP32C",0.8409,"Cardiometabolic_II","B13805","20212097_SS211647","PASS",0.7069,0.036,"Plate control","PASS","PFC",1.02526723788859,"1h","1h"
3915,"PFC_24",87,"OID30203","O43423","ANP32C",0.8409,"Cardiometabolic_II","B13805","20212097_SS211647","PASS",0.7069,-0.7185,"Plate control","PASS","PFC",0.607728982273875,"24h","24h"
3916,"PFC_01",1,"OID30204","Q9BQI0","AIF1L",0.0227,"Cardiometabolic_II","B13805","20212097_SS211647","PASS",-0.1491,0.1666,"Plate control","PASS","PFC",1.12241018074751,"Baseline","Baseline"
3917,"PFC_09",2,"OID30204","Q9BQI0","AIF1L",0.0227,"Cardiometabolic_II","B13805","20212097_SS211647","PASS",-0.1491,0.0473,"Plate control","PASS","PFC",1.03332924011952,"Baseline","Baseline"
3918,"PFC_17",3,"OID30204","Q9BQI0","AIF1L",0.0227,"Cardiometabolic_II","B13805","20212097_SS211647","PASS",-0.1491,0.7697,"Plate control","PASS","PFC",1.70491521951918,"1h","1h"
3919,"PFC_25",4,"OID30204","Q9BQI0","AIF1L",0.0227,"Cardiometabolic_II","B13805","20212097_SS211647","PASS",-0.1491,-0.1579,"Plate control","PASS","PFC",0.896328826196108,"24h","24h"
3920,"PFC_02",13,"OID30204","Q9BQI0","AIF1L",0.0227,"Cardiometabolic_II","B13805","20212097_SS211647","PASS",-0.1491,0.5698,"Plate control","PASS","PFC",1.48431778622778,"Baseline","Baseline"

Supplemental table 1_Olink proximity extension assay (PEA) data

,"SampleID","Index","OlinkID","UniProt","Assay","MissingFreq","Panel","Panel_Lot_Nr","PlateID","QC_Warning","LOD","NPX","Normalization","Assay_Warning","condition","Value","group","alt_group"
3921,"PFC_10",14,"OID30204","Q9BQI0","AIF1L",0.0227,"Cardiometabolic_II","B13805","20212097_SS211647","PASS",-0.1491,0.2289,"Plate control","PASS","PFC",1.17194104809581,"1h","1h"
3922,"PFC_18",15,"OID30204","Q9BQI0","AIF1L",0.0227,"Cardiometabolic_II","B13805","20212097_SS211647","PASS",-0.1491,0.2263,"Plate control","PASS","PFC",1.16983089825816,"1h","1h"
3923,"PFC_26",16,"OID30204","Q9BQI0","AIF1L",0.0227,"Cardiometabolic_II","B13805","20212097_SS211647","PASS",-0.1491,0.4927,"Plate control","PASS","PFC",1.40707575172473,"24h","24h"
3924,"PFC_03",25,"OID30204","Q9BQI0","AIF1L",0.0227,"Cardiometabolic_II","B13805","20212097_SS211647","PASS",-0.1491,-0.0806,"Plate control","PASS","PFC",0.945664274216494,"Baseline","Baseline"
3925,"PFC_11",26,"OID30204","Q9BQI0","AIF1L",0.0227,"Cardiometabolic_II","B13805","20212097_SS211647","PASS",-0.1491,1.0099,"Plate control","PASS","PFC",2.01377151127118,"1h","1h"
3926,"PFC_19",27,"OID30204","Q9BQI0","AIF1L",0.0227,"Cardiometabolic_II","B13805","20212097_SS211647","PASS",-0.1491,-0.1049,"Plate control","PASS","PFC",0.929869394014296,"24h","24h"
3927,"PFC_27",28,"OID30204","Q9BQI0","AIF1L",0.0227,"Cardiometabolic_II","B13805","20212097_SS211647","PASS",-0.1491,0.1018,"Plate control","PASS","PFC",1.0731115112377,"24h","24h"
3928,"PFC_04",37,"OID30204","Q9BQI0","AIF1L",0.0227,"Cardiometabolic_II","B13805","20212097_SS211647","PASS",-0.1491,0.1767,"Plate control","PASS","PFC",1.13029550434286,"Baseline","Baseline"
3929,"PFC_12",38,"OID30204","Q9BQI0","AIF1L",0.0227,"Cardiometabolic_II","B13805","20212097_SS211647","PASS",-0.1491,0.0664,"Plate control","PASS","PFC",1.04710055965196,"1h","1h"
3930,"PFC_20",39,"OID30204","Q9BQI0","AIF1L",0.0227,"Cardiometabolic_II","B13805","20212097_SS211647","PASS",-0.1491,0.644,"Plate control","PASS","PFC",1.56265576034463,"24h","24h"
3931,"PFC_05",49,"OID30204","Q9BQI0","AIF1L",0.0227,"Cardiometabolic_II","B13805","20212097_SS211647","PASS",-0.1491,0.4339,"Plate control","PASS","PFC",1.35088044539319,"Baseline","Baseline"
3932,"PFC_13",50,"OID30204","Q9BQI0","AIF1L",0.0227,"Cardiometabolic_II","B13805","20212097_SS211647","PASS",-0.1491,-0.1358,"Plate control","PASS","PFC",0.910164990994944,"1h","1h"
3933,"PFC_21",51,"OID30204","Q9BQI0","AIF1L",0.0227,"Cardiometabolic_II","B13805","20212097_SS211647","PASS",-0.1491,-0.0365,"Plate control","PASS","PFC",0.975017487653333,"24h","24h"
3934,"PFC_06",61,"OID30204","Q9BQI0","AIF1L",0.0227,"Cardiometabolic_II","B13805","20212097_SS211647","PASS",-0.1491,0.0834,"Plate control","PASS","PFC",1.05951205308092,"Baseline","Baseline"
3935,"PFC_14",62,"OID30204","Q9BQI0","AIF1L",0.0227,"Cardiometabolic_II","B13805","20212097_SS211647","PASS",-0.1491,0.7761,"Plate control","PASS","PFC",1.71249526610568,"1h","1h"
3936,"PFC_22",63,"OID30204","Q9BQI0","AIF1L",0.0227,"Cardiometabolic_II","B13805","20212097_SS211647","PASS",-0.1491,-0.0383,"Plate control","PASS","PFC",0.973801751106351,"24h","24h"
3937,"PFC_07",73,"OID30204","Q9BQI0","AIF1L",0.0227,"Cardiometabolic_II","B13805","20212097_SS211647","PASS",-0.1491,0.3679,"Plate control","PASS","PFC",1.29047303857682,"Baseline","Baseline"
3938,"PFC_15",74,"OID30204","Q9BQI0","AIF1L",0.0227,"Cardiometabolic_II","B13805","20212097_SS211647","PASS",-0.1491,0.0799,"Plate control","PASS","PFC",1.0569447761931,"1h","1h"
3939,"PFC_23",75,"OID30204","Q9BQI0","AIF1L",0.0227,"Cardiometabolic_II","B13805","20212097_SS211647","PASS",-0.1491,0.294,"Plate control","PASS","PFC",1.22603486005316,"24h","24h"
3940,"PFC_08",85,"OID30204","Q9BQI0","AIF1L",0.0227,"Cardiometabolic_II","B13805","20212097_SS211647","PASS",-0.1491,0.0102,"Plate control","PASS","PFC",1.00709515341316,"Baseline","Baseline"
3941,"PFC_16",86,"OID30204","Q9BQI0","AIF1L",0.0227,"Cardiometabolic_II","B13805","20212097_SS211647","PASS",-0.1491,-0.0517,"Plate control","PASS","PFC",0.964798787994174,"1h","1h"
3942,"PFC_24",87,"OID30204","Q9BQI0","AIF1L",0.0227,"Cardiometabolic_II","B13805","20212097_SS211647","PASS",-0.1491,-0.0317,"Plate control","PASS","PFC",0.978266877176623,"24h","24h"
3943,"PFC_01",1,"OID30205","Q8TER0","SNED1",0,"Cardiometabolic_II","B13805","20212097_SS211647","PASS",-2.2732,-0.7348,"Plate control","PASS","PFC",0.600901321600968,"Baseline","Baseline"
3944,"PFC_09",2,"OID30205","Q8TER0","SNED1",0,"Cardiometabolic_II","B13805","20212097_SS211647","PASS",-2.2732,-1.3499,"Plate control","PASS","PFC",0.392319241503555,"Baseline","Baseline"
3945,"PFC_17",3,"OID30205","Q8TER0","SNED1",0,"Cardiometabolic_II","B13805","20212097_SS211647","PASS",-2.2732,-1.5483,"Plate control","PASS","PFC",0.341912719831501,"1h","1h"
3946,"PFC_25",4,"OID30205","Q8TER0","SNED1",0,"Cardiometabolic_II","B13805","20212097_SS211647","PASS",-2.2732,-1.2611,"Plate control","PASS","PFC",0.417225719692268,"24h","24h"
3947,"PFC_02",13,"OID30205","Q8TER0","SNED1",0,"Cardiometabolic_II","B13805","20212097_SS211647","PASS",-2.2732,-1.4096,"Plate control","PASS","PFC",0.376416037072012,"Baseline","Baseline"
3948,"PFC_10",14,"OID30205","Q8TER0","SNED1",0,"Cardiometabolic_II","B13805","20212097_SS211647","PASS",-2.2732,-1.214,"Plate control","PASS","PFC",0.431071772508798,"1h","1h"
3949,"PFC_18",15,"OID30205","Q8TER0","SNED1",0,"Cardiometabolic_II","B13805","20212097_SS211647","PASS",-2.2732,-1.1927,"Plate control","PASS","PFC",0.437483345099167,"1h","1h"
3950,"PFC_26",16,"OID30205","Q8TER0","SNED1",0,"Cardiometabolic_II","B13805","20212097_SS211647","PASS",-2.2732,-1.1444,"Plate control","PASS","PFC",0.452377788296742,"24h","24h"
3951,"PFC_03",25,"OID30205","Q8TER0","SNED1",0,"Cardiometabolic_II","B13805","20212097_SS211647","PASS",-2.2732,-1.4679,"Plate control","PASS","PFC",0.361508130602166,"Baseline","Baseline"
3952,"PFC_11",26,"OID30205","Q8TER0","SNED1",0,"Cardiometabolic_II","B13805","20212097_SS211647","PASS",-2.2732,-1.1546,"Plate control","PASS","PFC",0.449190711288385,"1h","1h"
3953,"PFC_19",27,"OID30205","Q8TER0","SNED1",0,"Cardiometabolic_II","B13805","20212097_SS211647","PASS",-2.2732,-0.4391,"Plate control","PASS","PFC",0.7375946006077,"24h","24h"
3954,"PFC_27",28,"OID30205","Q8TER0","SNED1",0,"Cardiometabolic_II","B13805","20212097_SS211647","PASS",-2.2732,-0.8032,"Plate control","PASS","PFC",0.573076642065468,"24h","24h"
3955,"PFC_04",37,"OID30205","Q8TER0","SNED1",0,"Cardiometabolic_II","B13805","20212097_SS211647","PASS",-2.2732,-0.9568,"Plate control","PASS","PFC",0.515198393522473,"Baseline","Baseline"
3956,"PFC_12",38,"OID30205","Q8TER0","SNED1",0,"Cardiometabolic_II","B13805","20212097_SS211647","PASS",-2.2732,-1.2405,"Plate control","PASS","PFC",0.423225951823181,"1h","1h"
3957,"PFC_20",39,"OID30205","Q8TER0","SNED1",0,"Cardiometabolic_II","B13805","20212097_SS211647","PASS",-2.2732,-1.3135,"Plate control","PASS","PFC",0.40234360297736,"24h","24h"
3958,"PFC_05",49,"OID30205","Q8TER0","SNED1",0,"Cardiometabolic_II","B13805","20212097_SS211647","PASS",-2.2732,-1.0461,"Plate control","PASS","PFC",0.484275526028381,"Baseline","Baseline"
3959,"PFC_13",50,"OID30205","Q8TER0","SNED1",0,"Cardiometabolic_II","B13805","20212097_SS211647","PASS",-2.2732,-0.9231,"Plate control","PASS","PFC",0.52737460244522 9,"1h","1h"
3960,"PFC_21",51,"OID30205","Q8TER0","SNED1",0,"Cardiometabolic_II","B13805","20212097_SS211647","PASS",-2.2732,-1.3354,"Plate control","PASS","PFC",0.396282180321739,"24h","24h"
3961,"PFC_06",61,"OID30205","Q8TER0","SNED1",0,"Cardiometabolic_II","B13805","20212097_SS211647","PASS",-2.2732,-1.0437,"Plate control","PASS","PFC",0.48508181461158,"Baseline","Baseline"
3962,"PFC_14",62,"OID30205","Q8TER0","SNED1",0,"Cardiometabolic_II","B13805","20212097_SS211647","PASS",-2.2732,-1.5199,"Plate control","PASS","PFC",0.348710086491255,"1h","1h"
3963,"PFC_22",63,"OID30205","Q8TER0","SNED1",0,"Cardiometabolic_II","B13805","20212097_SS211647","PASS",-2.2732,-1.2394,"Plate control","PASS","PFC",0.423548768538035,"24h","24h"
3964,"PFC_07",73,"OID30205","Q8TER0","SNED1",0,"Cardiometabolic_II","B13805","20212097_SS211647","PASS",-2.2732,-0.9573,"Plate control","PASS","PFC",0.515019870303027,"Baseline","Baseline"
3965,"PFC_15",74,"OID30205","Q8TER0","SNED1",0,"Cardiometabolic_II","B13805","20212097_SS211647","PASS",-2.2732,-1.5714,"Plate control","PASS","PFC",0.336481711844958,"1h","1h"
3966,"PFC_23",75,"OID30205","Q8TER0","SNED1",0,"Cardiometabolic_II","B13805","20212097_SS211647","PASS",-2.2732,-0.8602,"Plate control","PASS","PFC",0.550876184989766,"24h","24h"
3967,"PFC_08",85,"OID30205","Q8TER0","SNED1",0,"Cardiometabolic_II","B13805","20212097_SS211647","PASS",-2.2732,-1.6838,"Plate control","PASS","PFC",0.311261705889519,"Baseline","Baseline"
3968,"PFC_16",86,"OID30205","Q8TER0","SNED1",0,"Cardiometabolic_II","B13805","20212097_SS211647","PASS",-2.2732,-1.07,"Plate control","PASS","PFC",0.476318999021969,"1h","1h"
3969,"PFC_24",87,"OID30205","Q8TER0","SNED1",0,"Cardiometabolic_II","B13805","20212097_SS211647","PASS",-2.2732,-1.388,"Plate control","PASS","PFC",0.382094130191292,"24h","24h"
3970,"PFC_01",1,"OID30206","Q9BTK6","PAGR1",0,"Cardiometabolic_II","B13805","20212097_SS211647","PASS",-0.8118,0.4373,"Plate control","PASS","PFC",1.35406782026659,"Baseline","Baseline"
3971,"PFC_09",2,"OID30206","Q9BTK6","PAGR1",0,"Cardiometabolic_II","B13805","20212097_SS211647","PASS",-0.8118,0.7907,"Plate control","PASS","PFC",1.72991361834183,"Baseline","Baseline"
3972,"PFC_17",3,"OID30206","Q9BTK6","PAGR1",0,"Cardiometabolic_II","B13805","20212097_SS211647","PASS",-0.8118,1.0658,"Plate control","PASS","PFC",2.09333034662774,"1h","1h"
3973,"PFC_25",4,"OID30206","Q9BTK6","PAGR1",0,"Cardiometabolic_II","B13805","20212097_SS211647","PASS",-0.8118,0.8829,"Plate control","PASS","PFC",1.84407840962016,"24h","24h"
3974,"PFC_02",13,"OID30206","Q9BTK6","PAGR1",0,"Cardiometabolic_II","B13805","20212097_SS211647","PASS",-0.8118,0.6132,"Plate control","PASS","PFC",1.52964831746866,"Baseline","Baseline"
3975,"PFC_10",14,"OID30206","Q9BTK6","PAGR1",0,"Cardiometabolic_II","B13805","20212097_SS211647","PASS",-0.8118,0.3437,"Plate control","PASS","PFC",1.26900697599923,"1h","1h"
3976,"PFC_18",15,"OID30206","Q9BTK6","PAGR1",0,"Cardiometabolic_II","B13805","20212097_SS211647","PASS",-0.8118,0.9409,"Plate control","PASS","PFC",1.91972545223755,"1h","1h"
3977,"PFC_26",16,"OID30206","Q9BTK6","PAGR1",0,"Cardiometabolic_II","B13805","20212097_SS211647","PASS",-0.8118,0.9338,"Plate control","PASS","PFC",1.91030103045317,"24h","24h"
3978,"PFC_03",25,"OID30206","Q9BTK6","PAGR1",0,"Cardiometabolic_II","B13805","20212097_SS211647","PASS",-0.8118,-0.6873,"Plate control","PASS","PFC",0.621014991027671,"Baseline","Baseline"
3979,"PFC_11",26,"OID30206","Q9BTK6","PAGR1",0,"Cardiometabolic_II","B13805","20212097_SS211647","PASS",-0.8118,0.8364,"Plate control","PASS","PFC",1.78558894469803,"1h","1h"
3980,"PFC_19",27,"OID30206","Q9BTK6","PAGR1",0,"Cardiometabolic_II","B13805","20212097_SS211647","PASS",-0.8118,0.4008,"Plate control","PASS","PFC",1.32023980422856,"24h","24h"
3981,"PFC_27",28,"OID30206","Q9BTK6","PAGR1",0,"Cardiometabolic_II","B13805","20212097_SS211647","PASS",-0.8118,1.9318,"Plate control","PASS","PFC",3.81530925135689,"24h","24h"
3982,"PFC_04",37,"OID30206","Q9BTK6","PAGR1",0,"Cardiometabolic_II","B13805","20212097_SS211647","PASS",-0.8118,0.3152,"Plate control","PASS","PFC",1.24418412188682,"Baseline","Baseline"
3983,"PFC_12",38,"OID30206","Q9BTK6","PAGR1",0,"Cardiometabolic_II","B13805","20212097_SS211647","PASS",-0.8118,0.1099,"Plate control","PASS","PFC",1.07915343269603,"1h","1h"
3984,"PFC_20",39,"OID30206","Q9BTK6","PAGR1",0,"Cardiometabolic_II","B13805","20212097_SS211647","PASS",-0.8118,0.9195,"Plate control","PASS","PFC",1.89145964988165,"24h","24h"
3985,"PFC_05",49,"OID30206","Q9BTK6","PAGR1",0,"Cardiometabolic_II","B13805","20212097_SS211647","PASS",-0.8118,0.1202,"Plate control","PASS","PFC",1.08688552641024,"Baseline","Baseline"
3986,"PFC_13",50,"OID30206","Q9BTK6","PAGR1",0,"Cardiometabolic_II","B13805","20212097_SS211647","PASS",-0.8118,0.9805,"Plate control","PASS","PFC",1.97314913187852,"1h","1h"
3987,"PFC_21",51,"OID30206","Q9BTK6","PAGR1",0,"Cardiometabolic_II","B13805","20212097_SS211647","PASS",-0.8118,0.4077,"Plate control","PASS","PFC",1.3265692595916,"24h","24h"
3988,"PFC_06",61,"OID30206","Q9BTK6","PAGR1",0,"Cardiometabolic_II","B13805","20212097_SS211647","PASS",-0.8118,1.0352,"Plate control","PASS","PFC",2.04939773322585,"Baseline","Baseline"
3989,"PFC_14",62,"OID30206","Q9BTK6","PAGR1",0,"Cardiometabolic_II","B13805","20212097_SS211647","PASS",-0.8118,0.791,"Plate control","PASS","PFC",1.73027338116998,"1h","1h"
3990,"PFC_22",63,"OID30206","Q9BTK6","PAGR1",0,"Cardiometabolic_II","B13805","20212097_SS211647","PASS",-0.8118,0.5899,"Plate control","PASS","PFC",1.50514241527308,"24h","24h"
3991,"PFC_07",73,"OID30206","Q9BTK6","PAGR1",0,"Cardiometabolic_II","B13805","20212097_SS211647","PASS",-0.8118,0.8152,"Plate control","PASS","PFC",1.7595420592616,"Baseline","Baseline"
3992,"PFC_15",74,"OID30206","Q9BTK6","PAGR1",0,"Cardiometabolic_II","B13805","20212097_SS211647","PASS",-0.8118,-0.3189,"Plate control","PASS","PFC",0.801680895758849,"1h","1h"
3993,"PFC_23",75,"OID30206","Q9BTK6","PAGR1",0,"Cardiometabolic_II","B13805","20212097_SS211647","PASS",-0.8118,0.496,"Plate control","PASS","PFC",1.41029796048439,"24h","24h"
3994,"PFC_08",85,"OID30206","Q9BTK6","PAGR1",0,"Cardiometabolic_II","B13805","20212097_SS211647","PASS",-0.8118,0.6391,"Plate control","PASS","PFC",1.5573573261662,"Baseline","Baseline"
3995,"PFC_16",86,"OID30206","Q9BTK6","PAGR1",0,"Cardiometabolic_II","B13805","20212097_SS211647","PASS",-0.8118,0.7799,"Plate control","PASS","PFC",1.7170118545581,"1h","1h"
3996,"PFC_24",87,"OID30206","Q9BTK6","PAGR1",0,"Cardiometabolic_II","B13805","20212097_SS211647","PASS",-0.8118,1.0754,"Plate control","PASS","PFC",2.10730626027738,"24h","24h"
3997,"PFC_01",1,"OID30207","Q9H173","SIL1",0,"Cardiometabolic_II","B13805","20212097_SS211647","PASS",-1.4834,-0.7496,"Plate control","PASS","PFC",0.594768439469572,"Baseline","Baseline"
3998,"PFC_09",2,"OID30207","Q9H173","SIL1",0,"Cardiometabolic_II","B13805","20212097_SS211647","PASS",-1.4834,-1.2133,"Plate control","PASS","PFC",0.431280980587504,"Baseline","Baseline"
3999,"PFC_17",3,"OID30207","Q9H173","SIL1",0,"Cardiometabolic_II","B13805","20212097_SS211647","PASS",-1.4834,-1.0162,"Plate control","PASS","PFC",0.494416912701552,"1h","1h"
4000,"PFC_25",4,"OID30207","Q9H173","SIL1",0,"Cardiometabolic_II","B13805","20212097_SS211647","PASS",-1.4834,-1.0263,"Plate control","PASS","PFC",0.490967693154372,"24h","24h"
4001,"PFC_02",13,"OID30207","Q9H173","SIL1",0,"Cardiometabolic_II","B13805","20212097_SS211647","PASS",-1.4834,-1.1795,"Plate control","PASS","PFC",0.44150448542776,"Baseline","Baseline"
4002,"PFC_10",14,"OID30207","Q9H173","SIL1",0,"Cardiometabolic_II","B13805","20212097_SS211647","PASS",-1.4834,-1.0733,"Plate control","PASS","PFC",0.475230718889651,"1h","1h"
4003,"PFC_18",15,"OID30207","Q9H173","SIL1",0,"Cardiometabolic_II","B13805","20212097_SS211647","PASS",-1.4834,-1.3014,"Plate control","PASS","PFC",0.405732282017437,"1h","1h"
4004,"PFC_26",16,"OID30207","Q9H173","SIL1",0,"Cardiometabolic_II","B13805","20212097_SS211647","PASS",-1.4834,-1.2652,"Plate control","PASS","PFC",0.416041687733238,"24h","24h"
4005,"PFC_03",25,"OID30207","Q9H173","SIL1",0,"Cardiometabolic_II","B13805","20212097_SS211647","PASS",-1.4834,-1.2484,"Plate control","PASS","PFC",0.42091475827315,"Baseline","Baseline"
4006,"PFC_11",26,"OID30207","Q9H173","SIL1",0,"Cardiometabolic_II","B13805","20212097_SS211647","PASS",-1.4834,-1.0927,"Plate control","PASS","PFC",0.468883039578894,"1h","1h"
4007,"PFC_19",27,"OID30207","Q9H173","SIL1",0,"Cardiometabolic_II","B13805","20212097_SS211647","PASS",-1.4834,-0.8481,"Plate control","PASS","PFC",0.555515857567977,"24h","24h"
4008,"PFC_27",28,"OID30207","Q9H173","SIL1",0,"Cardiometabolic_II","B13805","20212097_SS211647","PASS",-1.4834,-0.8967,"Plate control","PASS","PFC",0.537113913896774,"24h","24h"
4009,"PFC_04",37,"OID30207","Q9H173","SIL1",0,"Cardiometabolic_II","B13805","20212097_SS211647","PASS",-1.4834,-1.0051,"Plate control","PASS","PFC",0.498235595157209,"Baseline","Baseline"
4010,"PFC_12",38,"OID30207","Q9H173","SIL1",0,"Cardiometabolic_II","B13805","20212097_SS211647","PASS",-1.4834,-1.1299,"Plate control","PASS","PFC",0.45694739719702,"1h","1h"
4011,"PFC_20",39,"OID30207","Q9H173","SIL1",0,"Cardiometabolic_II","B13805","20212097_SS211647","PASS",-1.4834,-1.346,"Plate control","PASS","PFC",0.393381222676783,"24h","24h"
4012,"PFC_05",49,"OID30207","Q9H173","SIL1",0,"Cardiometabolic_II","B13805","20212097_SS211647","PASS",-1.4834,-0.8984,"Plate control","PASS","PFC",0.536481378352562,"Baseline","Baseline"
4013,"PFC_13",50,"OID30207","Q9H173","SIL1",0,"Cardiometabolic_II","B13805","20212097_SS211647","PASS",-1.4834,-0.9307,"Plate control","PASS","PFC",0.524603740727263,"1h","1h"
4014,"PFC_21",51,"OID30207","Q9H173","SIL1",0,"Cardiometabolic_II","B13805","20212097_SS211647","PASS",-1.4834,-1.1829,"Plate control","PASS","PFC",0.440465216727711,"24h","24h"
4015,"PFC_06",61,"OID30207","Q9H173","SIL1",0,"Cardiometabolic_II","B13805","20212097_SS211647","PASS",-1.4834,-1.1101,"Plate control","PASS","PFC",0.463261918962983,"Baseline","Baseline"
4016,"PFC_14",62,"OID30207","Q9H173","SIL1",0,"Cardiometabolic_II","B13805","20212097_SS211647","PASS",-1.4834,-0.973,"Plate control","PASS","PFC",0.509445598308361,"1h","1h"
4017,"PFC_22",63,"OID30207","Q9H173","SIL1",0,"Cardiometabolic_II","B13805","20212097_SS211647","PASS",-1.4834,-0.6973,"Plate control","PASS","PFC",0.616725327143478,"24h","24h"
4018,"PFC_07",73,"OID30207","Q9H173","SIL1",0,"Cardiometabolic_II","B13805","20212097_SS211647","PASS",-1.4834,-1.1802,"Plate control","PASS","PFC",0.441290318076789,"Baseline","Baseline"
4019,"PFC_15",74,"OID30207","Q9H173","SIL1",0,"Cardiometabolic_II","B13805","20212097_SS211647","PASS",-1.4834,-1.2453,"Plate control","PASS","PFC",0.421820173905188,"1h","1h"
4020,"PFC_23",75,"OID30207","Q9H173","SIL1",0,"Cardiometabolic_II","B13805","20212097_SS211647","PASS",-1.4834,-0.9776,"Plate control","PASS","PFC",0.507823829579957,"24h","24h"
4021,"PFC_08",85,"OID30207","Q9H173","SIL1",0,"Cardiometabolic_II","B13805","20212097_SS211647","PASS",-1.4834,-1.4396,"Plate control","PASS","PFC",0.368669507045562,"Baseline","Baseline"
4022,"PFC_16",86,"OID30207","Q9H173","SIL1",0,"Cardiometabolic_II","B13805","20212097_SS211647","PASS",-1.4834,-1.1407,"Plate control","PASS","PFC",0.453539465542097,"1h","1h"
4023,"PFC_24",87,"OID30207","Q9H173","SIL1",0,"Cardiometabolic_II","B13805","20212097_SS211647","PASS",-1.4834,-1.1791,"Plate control","PASS","PFC",0.441626913434812,"24h","24h"
4024,"PFC_01",1,"OID30208","P20645","M6PR",0.2273,"Cardiometabolic_II","B13805","20212097_SS211647","PASS",0.2224,0.1348,"Plate control","PASS","PFC",1.09794059635063,"Baseline","Baseline"
4025,"PFC_09",2,"OID30208","P20645","M6PR",0.2273,"Cardiometabolic_II","B13805","20212097_SS211647","PASS",0.2224,0.3841,"Plate control","PASS","PFC",1.30504540340815,"Baseline","Baseline"
4026,"PFC_17",3,"OID30208","P20645","M6PR",0.2273,"Cardiometabolic_II","B13805","20212097_SS211647","PASS",0.2224,1.2688,"Plate control","PASS","PFC",2.40961056388069,"1h","1h"
4027,"PFC_25",4,"OID30208","P20645","M6PR",0.2273,"Cardiometabolic_II","B13805","20212097_SS211647","PASS",0.2224,0.2669,"Plate control","PASS","PFC",1.20321962228011,"24h","24h"
4028,"PFC_02",13,"OID30208","P20645","M6PR",0.2273,"Cardiometabolic_II","B13805","20212097_SS211647","PASS",0.2224,0.4392,"Plate control","PASS","PFC",1.35585227480844,"Baseline","Baseline"
4029,"PFC_10",14,"OID30208","P20645","M6PR",0.2273,"Cardiometabolic_II","B13805","20212097_SS211647","PASS",0.2224,0.2425,"Plate control","PASS","PFC",1.18304093994891,"1h","1h"
4030,"PFC_18",15,"OID30208","P20645","M6PR",0.2273,"Cardiometabolic_II","B13805","20212097_SS211647","PASS",0.2224,0.4056,"Plate control","PASS","PFC",1.32463969801756,"1h","1h"
4031,"PFC_26",16,"OID30208","P20645","M6PR",0.2273,"Cardiometabolic_II","B13805","20212097_SS211647","PASS",0.2224,1.0081,"Plate control","PASS","PFC",2.01126056592453,"24h","24h"
4032,"PFC_03",25,"OID30208","P20645","M6PR",0.2273,"Cardiometabolic_II","B13805","20212097_SS211647","PASS",0.2224,0.0502,"Plate control","PASS","PFC",1.03540845198238,"Baseline","Baseline"

Supplemental table 1_Olink proximity extension assay (PEA) data

,"SampleID","Index","OlinkID","UniProt","Assay","MissingFreq","Panel","Panel_Lot_Nr","PlateID","QC_Warning","LOD","NPX","Normalization","Assay_Warning","condition","Value","group","alt_group"
4033,"PFC_11",26,"OID30208","P20645","M6PR",0.2273,"Cardiometabolic_II","B13805","20212097_SS211647","PASS",0.2224,0.6544,"Plate control","PASS","PFC",1.57396122492972,"1h","1h"
4034,"PFC_19",27,"OID30208","P20645","M6PR",0.2273,"Cardiometabolic_II","B13805","20212097_SS211647","PASS",0.2224,0.6148,"Plate control","PASS","PFC",1.53134569278903,"24h","24h"
4035,"PFC_27",28,"OID30208","P20645","M6PR",0.2273,"Cardiometabolic_II","B13805","20212097_SS211647","PASS",0.2224,0.7923,"Plate control","PASS","PFC",1.73183321819273,"24h","24h"
4036,"PFC_04",37,"OID30208","P20645","M6PR",0.2273,"Cardiometabolic_II","B13805","20212097_SS211647","PASS",0.2224,0.2652,"Plate control","PASS","PFC",1.20180264320194,"Baseline","Baseline"
4037,"PFC_12",38,"OID30208","P20645","M6PR",0.2273,"Cardiometabolic_II","B13805","20212097_SS211647","PASS",0.2224,0.3848,"Plate control","PASS","PFC",1.3056787690305,"1h","1h"
4038,"PFC_20",39,"OID30208","P20645","M6PR",0.2273,"Cardiometabolic_II","B13805","20212097_SS211647","PASS",0.2224,0.9605,"Plate control","PASS","PFC",1.94598420470128,"24h","24h"
4039,"PFC_05",49,"OID30208","P20645","M6PR",0.2273,"Cardiometabolic_II","B13805","20212097_SS211647","PASS",0.2224,0.2219,"Plate control","PASS","PFC",1.16626852741256,"Baseline","Baseline"
4040,"PFC_13",50,"OID30208","P20645","M6PR",0.2273,"Cardiometabolic_II","B13805","20212097_SS211647","PASS",0.2224,0.456,"Plate control","PASS","PFC",1.3717332885906,"1h","1h"
4041,"PFC_21",51,"OID30208","P20645","M6PR",0.2273,"Cardiometabolic_II","B13805","20212097_SS211647","PASS",0.2224,0.1958,"Plate control","PASS","PFC",1.14535910648635,"24h","24h"
4042,"PFC_06",61,"OID30208","P20645","M6PR",0.2273,"Cardiometabolic_II","B13805","20212097_SS211647","PASS",0.2224,0.4831,"Plate control","PASS","PFC",1.39774385271446,"Baseline","Baseline"
4043,"PFC_14",62,"OID30208","P20645","M6PR",0.2273,"Cardiometabolic_II","B13805","20212097_SS211647","PASS",0.2224,0.4447,"Plate control","PASS","PFC",1.36103106861706,"1h","1h"
4044,"PFC_22",63,"OID30208","P20645","M6PR",0.2273,"Cardiometabolic_II","B13805","20212097_SS211647","PASS",0.2224,0.1714,"Plate control","PASS","PFC",1.12615077815046,"24h","24h"
4045,"PFC_07",73,"OID30208","P20645","M6PR",0.2273,"Cardiometabolic_II","B13805","20212097_SS211647","PASS",0.2224,0.33,"Plate control","PASS","PFC",1.25701337452183,"Baseline","Baseline"
4046,"PFC_15",74,"OID30208","P20645","M6PR",0.2273,"Cardiometabolic_II","B13805","20212097_SS211647","PASS",0.2224,0.0223,"Plate control","PASS","PFC",1.01557726226857,"1h","1h"
4047,"PFC_23",75,"OID30208","P20645","M6PR",0.2273,"Cardiometabolic_II","B13805","20212097_SS211647","PASS",0.2224,0.3601,"Plate control","PASS","PFC",1.28351486095034,"24h","24h"
4048,"PFC_08",85,"OID30208","P20645","M6PR",0.2273,"Cardiometabolic_II","B13805","20212097_SS211647","PASS",0.2224,0.3994,"Plate control","PASS","PFC",1.31895925695731,"Baseline","Baseline"
4049,"PFC_16",86,"OID30208","P20645","M6PR",0.2273,"Cardiometabolic_II","B13805","20212097_SS211647","PASS",0.2224,0.3761,"Plate control","PASS","PFC",1.29782872245351,"1h","1h"
4050,"PFC_24",87,"OID30208","P20645","M6PR",0.2273,"Cardiometabolic_II","B13805","20212097_SS211647","PASS",0.2224,0.4605,"Plate control","PASS","PFC",1.37601862722618,"24h","24h"
4051,"PFC_01",1,"OID30209","P13929","ENO3",0,"Cardiometabolic_II","B13805","20212097_SS211647","PASS",-2.221,-0.3653,"Plate control","PASS","PFC",0.776307429623983,"Baseline","Baseline"
4052,"PFC_09",2,"OID30209","P13929","ENO3",0,"Cardiometabolic_II","B13805","20212097_SS211647","PASS",-2.221,-0.3327,"Plate control","PASS","PFC",0.794049031619444,"Baseline","Baseline"
4053,"PFC_17",3,"OID30209","P13929","ENO3",0,"Cardiometabolic_II","B13805","20212097_SS211647","PASS",-2.221,-0.7852,"Plate control","PASS","PFC",0.580271508903667,"1h","1h"
4054,"PFC_25",4,"OID30209","P13929","ENO3",0,"Cardiometabolic_II","B13805","20212097_SS211647","PASS",-2.221,-1.5721,"Plate control","PASS","PFC",0.336318489501235,"24h","24h"
4055,"PFC_02",13,"OID30209","P13929","ENO3",0,"Cardiometabolic_II","B13805","20212097_SS211647","PASS",-2.221,0.3242,"Plate control","PASS","PFC",1.25197000652033,"Baseline","Baseline"
4056,"PFC_10",14,"OID30209","P13929","ENO3",0,"Cardiometabolic_II","B13805","20212097_SS211647","PASS",-2.221,-0.7004,"Plate control","PASS","PFC",0.615401557474759,"1h","1h"
4057,"PFC_18",15,"OID30209","P13929","ENO3",0,"Cardiometabolic_II","B13805","20212097_SS211647","PASS",-2.221,-0.3513,"Plate control","PASS","PFC",0.783877434396764,"1h","1h"
4058,"PFC_26",16,"OID30209","P13929","ENO3",0,"Cardiometabolic_II","B13805","20212097_SS211647","PASS",-2.221,-1.332,"Plate control","PASS","PFC",0.397217200048052,"24h","24h"
4059,"PFC_03",25,"OID30209","P13929","ENO3",0,"Cardiometabolic_II","B13805","20212097_SS211647","PASS",-2.221,-0.6457,"Plate control","PASS","PFC",0.639182582064496,"Baseline","Baseline"
4060,"PFC_11",26,"OID30209","P13929","ENO3",0,"Cardiometabolic_II","B13805","20212097_SS211647","PASS",-2.221,0.6107,"Plate control","PASS","PFC",1.52699993423435,"1h","1h"
4061,"PFC_19",27,"OID30209","P13929","ENO3",0,"Cardiometabolic_II","B13805","20212097_SS211647","PASS",-2.221,-0.4668,"Plate control","PASS","PFC",0.723567743771086,"24h","24h"
4062,"PFC_27",28,"OID30209","P13929","ENO3",0,"Cardiometabolic_II","B13805","20212097_SS211647","PASS",-2.221,0.1404,"Plate control","PASS","PFC",1.10221067120334,"24h","24h"
4063,"PFC_04",37,"OID30209","P13929","ENO3",0,"Cardiometabolic_II","B13805","20212097_SS211647","PASS",-2.221,-0.5203,"Plate control","PASS","PFC",0.697226833855615,"Baseline","Baseline"
4064,"PFC_12",38,"OID30209","P13929","ENO3",0,"Cardiometabolic_II","B13805","20212097_SS211647","PASS",-2.221,-0.351,"Plate control","PASS","PFC",0.784040454075739,"1h","1h"
4065,"PFC_20",39,"OID30209","P13929","ENO3",0,"Cardiometabolic_II","B13805","20212097_SS211647","PASS",-2.221,0.0483,"Plate control","PASS","PFC",1.03404573765933,"24h","24h"
4066,"PFC_05",49,"OID30209","P13929","ENO3",0,"Cardiometabolic_II","B13805","20212097_SS211647","PASS",-2.221,0.1637,"Plate control","PASS","PFC",1.12015626002826,"Baseline","Baseline"
4067,"PFC_13",50,"OID30209","P13929","ENO3",0,"Cardiometabolic_II","B13805","20212097_SS211647","PASS",-2.221,-0.5062,"Plate control","PASS","PFC",0.704074501239146,"1h","1h"
4068,"PFC_21",51,"OID30209","P13929","ENO3",0,"Cardiometabolic_II","B13805","20212097_SS211647","PASS",-2.221,-0.14,"Plate control","PASS","PFC",0.907519155317161,"24h","24h"
4069,"PFC_06",61,"OID30209","P13929","ENO3",0,"Cardiometabolic_II","B13805","20212097_SS211647","PASS",-2.221,-0.2372,"Plate control","PASS","PFC",0.848390281686933,"Baseline","Baseline"
4070,"PFC_14",62,"OID30209","P13929","ENO3",0,"Cardiometabolic_II","B13805","20212097_SS211647","PASS",-2.221,-0.0331,"Plate control","PASS","PFC",0.97731802153986,"1h","1h"
4071,"PFC_22",63,"OID30209","P13929","ENO3",0,"Cardiometabolic_II","B13805","20212097_SS211647","PASS",-2.221,-0.7755,"Plate control","PASS","PFC",0.584186125709342,"24h","24h"
4072,"PFC_07",73,"OID30209","P13929","ENO3",0,"Cardiometabolic_II","B13805","20212097_SS211647","PASS",-2.221,-1.1318,"Plate control","PASS","PFC",0.456346002875923,"Baseline","Baseline"
4073,"PFC_15",74,"OID30209","P13929","ENO3",0,"Cardiometabolic_II","B13805","20212097_SS211647","PASS",-2.221,-0.2424,"Plate control","PASS","PFC",0.845337877456351,"1h","1h"
4074,"PFC_23",75,"OID30209","P13929","ENO3",0,"Cardiometabolic_II","B13805","20212097_SS211647","PASS",-2.221,-0.121,"Plate control","PASS","PFC",0.919550046151658,"24h","24h"
4075,"PFC_08",85,"OID30209","P13929","ENO3",0,"Cardiometabolic_II","B13805","20212097_SS211647","PASS",-2.221,-1.1544,"Plate control","PASS","PFC",0.449252986659898,"Baseline","Baseline"
4076,"PFC_16",86,"OID30209","P13929","ENO3",0,"Cardiometabolic_II","B13805","20212097_SS211647","PASS",-2.221,-1.2321,"Plate control","PASS","PFC",0.425697345760312,"1h","1h"
4077,"PFC_24",87,"OID30209","P13929","ENO3",0,"Cardiometabolic_II","B13805","20212097_SS211647","PASS",-2.221,-0.1863,"Plate control","PASS","PFC",0.87885678877767,"24h","24h"
4078,"PFC_01",1,"OID30210","Q96ID5","IGSF21",0,"Cardiometabolic_II","B13805","20212097_SS211647","PASS",-1.8197,1.0235,"Plate control","PASS","PFC",2.03284469419986,"Baseline","Baseline"
4079,"PFC_09",2,"OID30210","Q96ID5","IGSF21",0,"Cardiometabolic_II","B13805","20212097_SS211647","PASS",-1.8197,1.1912,"Plate control","PASS","PFC",2.28342594236378,"Baseline","Baseline"
4080,"PFC_17",3,"OID30210","Q96ID5","IGSF21",0,"Cardiometabolic_II","B13805","20212097_SS211647","PASS",-1.8197,1.0213,"Plate control","PASS","PFC",2.02974712332962,"1h","1h"
4081,"PFC_25",4,"OID30210","Q96ID5","IGSF21",0,"Cardiometabolic_II","B13805","20212097_SS211647","PASS",-1.8197,0.4088,"Plate control","PASS","PFC",1.32758110380523,"24h","24h"
4082,"PFC_02",13,"OID30210","Q96ID5","IGSF21",0,"Cardiometabolic_II","B13805","20212097_SS211647","PASS",-1.8197,0.4589,"Plate control","PASS","PFC",1.3744934196462,"Baseline","Baseline"
4083,"PFC_10",14,"OID30210","Q96ID5","IGSF21",0,"Cardiometabolic_II","B13805","20212097_SS211647","PASS",-1.8197,1.0027,"Plate control","PASS","PFC",2.00374649946349,"1h","1h"
4084,"PFC_18",15,"OID30210","Q96ID5","IGSF21",0,"Cardiometabolic_II","B13805","20212097_SS211647","PASS",-1.8197,0.8571,"Plate control","PASS","PFC",1.81139351791528,"1h","1h"
4085,"PFC_26",16,"OID30210","Q96ID5","IGSF21",0,"Cardiometabolic_II","B13805","20212097_SS211647","PASS",-1.8197,0.8686,"Plate control","PASS","PFC",1.82589018558348,"24h","24h"
4086,"PFC_03",25,"OID30210","Q96ID5","IGSF21",0,"Cardiometabolic_II","B13805","20212097_SS211647","PASS",-1.8197,1.0745,"Plate control","PASS","PFC",2.10599206418549,"Baseline","Baseline"
4087,"PFC_11",26,"OID30210","Q96ID5","IGSF21",0,"Cardiometabolic_II","B13805","20212097_SS211647","PASS",-1.8197,0.5493,"Plate control","PASS","PFC",1.46337548944616,"1h","1h"
4088,"PFC_19",27,"OID30210","Q96ID5","IGSF21",0,"Cardiometabolic_II","B13805","20212097_SS211647","PASS",-1.8197,1.1167,"Plate control","PASS","PFC",2.16850384310152,"24h","24h"
4089,"PFC_27",28,"OID30210","Q96ID5","IGSF21",0,"Cardiometabolic_II","B13805","20212097_SS211647","PASS",-1.8197,1.2049,"Plate control","PASS","PFC",2.30521290287745,"24h","24h"
4090,"PFC_04",37,"OID30210","Q96ID5","IGSF21",0,"Cardiometabolic_II","B13805","20212097_SS211647","PASS",-1.8197,0.5936,"Plate control","PASS","PFC",1.50900752479012,"Baseline","Baseline"
4091,"PFC_12",38,"OID30210","Q96ID5","IGSF21",0,"Cardiometabolic_II","B13805","20212097_SS211647","PASS",-1.8197,1.2331,"Plate control","PASS","PFC",2.35071559038101,"1h","1h"
4092,"PFC_20",39,"OID30210","Q96ID5","IGSF21",0,"Cardiometabolic_II","B13805","20212097_SS211647","PASS",-1.8197,0.4022,"Plate control","PASS","PFC",1.32152159475376,"24h","24h"
4093,"PFC_05",49,"OID30210","Q96ID5","IGSF21",0,"Cardiometabolic_II","B13805","20212097_SS211647","PASS",-1.8197,0.882,"Plate control","PASS","PFC",1.84292837239741,"Baseline","Baseline"
4094,"PFC_13",50,"OID30210","Q96ID5","IGSF21",0,"Cardiometabolic_II","B13805","20212097_SS211647","PASS",-1.8197,0.6888,"Plate control","PASS","PFC",1.61194218485984,"1h","1h"
4095,"PFC_21",51,"OID30210","Q96ID5","IGSF21",0,"Cardiometabolic_II","B13805","20212097_SS211647","PASS",-1.8197,1.1383,"Plate control","PASS","PFC",2.20121490091517,"24h","24h"
4096,"PFC_06",61,"OID30210","Q96ID5","IGSF21",0,"Cardiometabolic_II","B13805","20212097_SS211647","PASS",-1.8197,0.0885,"Plate control","PASS","PFC",1.06326410976981,"Baseline","Baseline"
4097,"PFC_14",62,"OID30210","Q96ID5","IGSF21",0,"Cardiometabolic_II","B13805","20212097_SS211647","PASS",-1.8197,0.7137,"Plate control","PASS","PFC",1.64000475753132,"1h","1h"
4098,"PFC_22",63,"OID30210","Q96ID5","IGSF21",0,"Cardiometabolic_II","B13805","20212097_SS211647","PASS",-1.8197,0.5386,"Plate control","PASS","PFC",1.4525622579617,"24h","24h"
4099,"PFC_07",73,"OID30210","Q96ID5","IGSF21",0,"Cardiometabolic_II","B13805","20212097_SS211647","PASS",-1.8197,0.7386,"Plate control","PASS","PFC",1.66855587625138,"Baseline","Baseline"
4100,"PFC_15",74,"OID30210","Q96ID5","IGSF21",0,"Cardiometabolic_II","B13805","20212097_SS211647","PASS",-1.8197,0.1948,"Plate control","PASS","PFC",1.14456547913301,"1h","1h"
4101,"PFC_23",75,"OID30210","Q96ID5","IGSF21",0,"Cardiometabolic_II","B13805","20212097_SS211647","PASS",-1.8197,0.7874,"Plate control","PASS","PFC",1.72596116079693,"24h","24h"
4102,"PFC_08",85,"OID30210","Q96ID5","IGSF21",0,"Cardiometabolic_II","B13805","20212097_SS211647","PASS",-1.8197,0.6168,"Plate control","PASS","PFC",1.53347006084738,"Baseline","Baseline"
4103,"PFC_16",86,"OID30210","Q96ID5","IGSF21",0,"Cardiometabolic_II","B13805","20212097_SS211647","PASS",-1.8197,0.4043,"Plate control","PASS","PFC",1.32344661428347,"1h","1h"
4104,"PFC_24",87,"OID30210","Q96ID5","IGSF21",0,"Cardiometabolic_II","B13805","20212097_SS211647","PASS",-1.8197,0.2577,"Plate control","PASS","PFC",1.19557115889224,"24h","24h"
4105,"PFC_01",1,"OID30211","P23327","HRC",0,"Cardiometabolic_II","B13805","20212097_SS211647","PASS",-3.3374,0.3591,"Plate control","PASS","PFC",1.28262550450633,"Baseline","Baseline"
4106,"PFC_09",2,"OID30211","P23327","HRC",0,"Cardiometabolic_II","B13805","20212097_SS211647","PASS",-3.3374,0.0863,"Plate control","PASS","PFC",1.06164394865116,"Baseline","Baseline"
4107,"PFC_17",3,"OID30211","P23327","HRC",0,"Cardiometabolic_II","B13805","20212097_SS211647","PASS",-3.3374,0.1001,"Plate control","PASS","PFC",1.07184775478641,"1h","1h"
4108,"PFC_25",4,"OID30211","P23327","HRC",0,"Cardiometabolic_II","B13805","20212097_SS211647","PASS",-3.3374,0.3395,"Plate control","PASS","PFC",1.26531799217138,"24h","24h"
4109,"PFC_02",13,"OID30211","P23327","HRC",0,"Cardiometabolic_II","B13805","20212097_SS211647","PASS",-3.3374,1.6756,"Plate control","PASS","PFC",3.19452183347899,"Baseline","Baseline"
4110,"PFC_10",14,"OID30211","P23327","HRC",0,"Cardiometabolic_II","B13805","20212097_SS211647","PASS",-3.3374,0.3846,"Plate control","PASS","PFC",1.30549777606477,"1h","1h"
4111,"PFC_18",15,"OID30211","P23327","HRC",0,"Cardiometabolic_II","B13805","20212097_SS211647","PASS",-3.3374,0.262,"Plate control","PASS","PFC",1.19913991379111,"1h","1h"
4112,"PFC_26",16,"OID30211","P23327","HRC",0,"Cardiometabolic_II","B13805","20212097_SS211647","PASS",-3.3374,0.1305,"Plate control","PASS","PFC",1.09467302028479,"24h","24h"
4113,"PFC_03",25,"OID30211","P23327","HRC",0,"Cardiometabolic_II","B13805","20212097_SS211647","PASS",-3.3374,0.1469,"Plate control","PASS","PFC",1.10718783741168,"Baseline","Baseline"
4114,"PFC_11",26,"OID30211","P23327","HRC",0,"Cardiometabolic_II","B13805","20212097_SS211647","PASS",-3.3374,1.611,"Plate control","PASS","PFC",3.0546349959214,"1h","1h"
4115,"PFC_19",27,"OID30211","P23327","HRC",0,"Cardiometabolic_II","B13805","20212097_SS211647","PASS",-3.3374,0.4253,"Plate control","PASS","PFC",1.34285171191271,"24h","24h"
4116,"PFC_27",28,"OID30211","P23327","HRC",0,"Cardiometabolic_II","B13805","20212097_SS211647","PASS",-3.3374,0.1621,"Plate control","PASS","PFC",1.11891465560173,"24h","24h"
4117,"PFC_04",37,"OID30211","P23327","HRC",0,"Cardiometabolic_II","B13805","20212097_SS211647","PASS",-3.3374,0.6608,"Plate control","PASS","PFC",1.58095905055396,"Baseline","Baseline"
4118,"PFC_12",38,"OID30211","P23327","HRC",0,"Cardiometabolic_II","B13805","20212097_SS211647","PASS",-3.3374,0.2179,"Plate control","PASS","PFC",1.16303942300416,"1h","1h"
4119,"PFC_20",39,"OID30211","P23327","HRC",0,"Cardiometabolic_II","B13805","20212097_SS211647","PASS",-3.3374,1.5355,"Plate control","PASS","PFC",2.89888881333625,"24h","24h"
4120,"PFC_05",49,"OID30211","P23327","HRC",0,"Cardiometabolic_II","B13805","20212097_SS211647","PASS",-3.3374,0.0299,"Plate control","PASS","PFC",1.02094135699225,"Baseline","Baseline"
4121,"PFC_13",50,"OID30211","P23327","HRC",0,"Cardiometabolic_II","B13805","20212097_SS211647","PASS",-3.3374,0.5701,"Plate control","PASS","PFC",1.4846264735281,"1h","1h"
4122,"PFC_21",51,"OID30211","P23327","HRC",0,"Cardiometabolic_II","B13805","20212097_SS211647","PASS",-3.3374,0.1668,"Plate control","PASS","PFC",1.12256579062375,"24h","24h"
4123,"PFC_06",61,"OID30211","P23327","HRC",0,"Cardiometabolic_II","B13805","20212097_SS211647","PASS",-3.3374,-0.3965,"Plate control","PASS","PFC",0.759699090929046,"Baseline","Baseline"
4124,"PFC_14",62,"OID30211","P23327","HRC",0,"Cardiometabolic_II","B13805","20212097_SS211647","PASS",-3.3374,0.0841,"Plate control","PASS","PFC",1.06002625627196,"1h","1h"
4125,"PFC_22",63,"OID30211","P23327","HRC",0,"Cardiometabolic_II","B13805","20212097_SS211647","PASS",-3.3374,0.548,"Plate control","PASS","PFC",1.46205744840003,"24h","24h"
4126,"PFC_07",73,"OID30211","P23327","HRC",0,"Cardiometabolic_II","B13805","20212097_SS211647","PASS",-3.3374,0.2176,"Plate control","PASS","PFC",1.1627976008987,"Baseline","Baseline"
4127,"PFC_15",74,"OID30211","P23327","HRC",0,"Cardiometabolic_II","B13805","20212097_SS211647","PASS",-3.3374,-0.2347,"Plate control","PASS","PFC",0.849861704538896,"1h","1h"
4128,"PFC_23",75,"OID30211","P23327","HRC",0,"Cardiometabolic_II","B13805","20212097_SS211647","PASS",-3.3374,0.0297,"Plate control","PASS","PFC",1.02079983427746,"24h","24h"
4129,"PFC_08",85,"OID30211","P23327","HRC",0,"Cardiometabolic_II","B13805","20212097_SS211647","PASS",-3.3374,-0.1786,"Plate control","PASS","PFC",0.883559992370906,"Baseline","Baseline"
4130,"PFC_16",86,"OID30211","P23327","HRC",0,"Cardiometabolic_II","B13805","20212097_SS211647","PASS",-3.3374,0.2597,"Plate control","PASS","PFC",1.19722972181072,"1h","1h"
4131,"PFC_24",87,"OID30211","P23327","HRC",0,"Cardiometabolic_II","B13805","20212097_SS211647","PASS",-3.3374,-0.0642,"Plate control","PASS","PFC",0.956475553234019,"24h","24h"
4132,"PFC_01",1,"OID30193","P32241","VIPR1",1,"Cardiometabolic_II","B13805","20212097_SS211647","PASS",1.1068,-0.1149,"Plate control","PASS","PFC",0.923446316932181,"Baseline","Baseline"
4133,"PFC_09",2,"OID30193","P32241","VIPR1",1,"Cardiometabolic_II","B13805","20212097_SS211647","PASS",1.1068,0.0332,"Plate control","PASS","PFC",1.02327931653677,"Baseline","Baseline"
4134,"PFC_17",3,"OID30193","P32241","VIPR1",1,"Cardiometabolic_II","B13805","20212097_SS211647","PASS",1.1068,0.1469,"Plate control","PASS","PFC",1.10718783741168,"1h","1h"
4135,"PFC_25",4,"OID30193","P32241","VIPR1",1,"Cardiometabolic_II","B13805","20212097_SS211647","PASS",1.1068,-0.3516,"Plate control","PASS","PFC",0.783714448613255,"24h","24h"
4136,"PFC_02",13,"OID30193","P32241","VIPR1",1,"Cardiometabolic_II","B13805","20212097_SS211647","PASS",1.1068,0.3576,"Plate control","PASS","PFC",1.28129262515933,"Baseline","Baseline"
4137,"PFC_10",14,"OID30193","P32241","VIPR1",1,"Cardiometabolic_II","B13805","20212097_SS211647","PASS",1.1068,0.1157,"Plate control","PASS","PFC",1.0835006357942,"1h","1h"
4138,"PFC_18",15,"OID30193","P32241","VIPR1",1,"Cardiometabolic_II","B13805","20212097_SS211647","PASS",1.1068,-0.1223,"Plate control","PASS","PFC",0.918721820782615,"1h","1h"
4139,"PFC_26",16,"OID30193","P32241","VIPR1",1,"Cardiometabolic_II","B13805","20212097_SS211647","PASS",1.1068,0.2927,"Plate control","PASS","PFC",1.22493058826428,"24h","24h"
4140,"PFC_03",25,"OID30193","P32241","VIPR1",1,"Cardiometabolic_II","B13805","20212097_SS211647","PASS",1.1068,0.1292,"Plate control","PASS","PFC",1.09368706419681,"Baseline","Baseline"
4141,"PFC_11",26,"OID30193","P32241","VIPR1",1,"Cardiometabolic_II","B13805","20212097_SS211647","PASS",1.1068,0.0777,"Plate control","PASS","PFC",1.05533424423287,"1h","1h"
4142,"PFC_19",27,"OID30193","P32241","VIPR1",1,"Cardiometabolic_II","B13805","20212097_SS211647","PASS",1.1068,0.4122,"Plate control","PASS","PFC",1.33071350435711,"24h","24h"
4143,"PFC_27",28,"OID30193","P32241","VIPR1",1,"Cardiometabolic_II","B13805","20212097_SS211647","PASS",1.1068,-0.3181,"Plate control","PASS","PFC",0.802125565318118,"24h","24h"
4144,"PFC_04",37,"OID30193","P32241","VIPR1",1,"Cardiometabolic_II","B13805","20212097_SS211647","PASS",1.1068,-0.4312,"Plate control","PASS","PFC",0.741644646014651,"Baseline","Baseline"

Supplemental table 1_Olink proximity extension assay (PEA) data

,"SampleID","Index","OlinkID","UniProt","Assay","MissingFreq","Panel","Panel_Lot_Nr","PlateID","QC_Warning","LOD","NPX","Normalization","Assay_Warning","condition","Value","group","alt_group"
4145,"PFC_12",38,"OID30193","P32241","VIPR1",1,"Cardiometabolic_II","B13805","20212097_SS211647","PASS",1.1068,-0.0864,"Plate control","PASS","PFC",0.941870095858957,"1h","1h"
4146,"PFC_20",39,"OID30193","P32241","VIPR1",1,"Cardiometabolic_II","B13805","20212097_SS211647","PASS",1.1068,-0.0902,"Plate control","PASS","PFC",0.939392512732587,"24h","24h"
4147,"PFC_05",49,"OID30193","P32241","VIPR1",1,"Cardiometabolic_II","B13805","20212097_SS211647","PASS",1.1068,0.1112,"Plate control","PASS","PFC",1.08012628675243,"Baseline","Baseline"
4148,"PFC_13",50,"OID30193","P32241","VIPR1",1,"Cardiometabolic_II","B13805","20212097_SS211647","PASS",1.1068,-0.5135,"Plate control","PASS","PFC",0.700520900421065,"1h","1h"
4149,"PFC_21",51,"OID30193","P32241","VIPR1",1,"Cardiometabolic_II","B13805","20212097_SS211647","PASS",1.1068,-0.0533,"Plate control","PASS","PFC",0.963729385011185,"24h","24h"
4150,"PFC_06",61,"OID30193","P32241","VIPR1",1,"Cardiometabolic_II","B13805","20212097_SS211647","PASS",1.1068,-0.1072,"Plate control","PASS","PFC",0.928388141460835,"Baseline","Baseline"
4151,"PFC_14",62,"OID30193","P32241","VIPR1",1,"Cardiometabolic_II","B13805","20212097_SS211647","PASS",1.1068,0.0268,"Plate control","PASS","PFC",1.01874995809481,"1h","1h"
4152,"PFC_22",63,"OID30193","P32241","VIPR1",1,"Cardiometabolic_II","B13805","20212097_SS211647","PASS",1.1068,-0.1545,"Plate control","PASS","PFC",0.898443695789984,"24h","24h"
4153,"PFC_07",73,"OID30193","P32241","VIPR1",1,"Cardiometabolic_II","B13805","20212097_SS211647","PASS",1.1068,0.2824,"Plate control","PASS","PFC",1.21621644324006,"Baseline","Baseline"
4154,"PFC_15",74,"OID30193","P32241","VIPR1",1,"Cardiometabolic_II","B13805","20212097_SS211647","PASS",1.1068,-0.4443,"Plate control","PASS","PFC",0.73494082565536,"1h","1h"
4155,"PFC_23",75,"OID30193","P32241","VIPR1",1,"Cardiometabolic_II","B13805","20212097_SS211647","PASS",1.1068,0.2075,"Plate control","PASS","PFC",1.15468553168869,"24h","24h"
4156,"PFC_08",85,"OID30193","P32241","VIPR1",1,"Cardiometabolic_II","B13805","20212097_SS211647","PASS",1.1068,-0.4285,"Plate control","PASS","PFC",0.743033931648849,"Baseline","Baseline"
4157,"PFC_16",86,"OID30193","P32241","VIPR1",1,"Cardiometabolic_II","B13805","20212097_SS211647","PASS",1.1068,-0.1355,"Plate control","PASS","PFC",0.910354274163631,"1h","1h"
4158,"PFC_24",87,"OID30193","P32241","VIPR1",1,"Cardiometabolic_II","B13805","20212097_SS211647","PASS",1.1068,-0.3927,"Plate control","PASS","PFC",0.761702745017505,"24h","24h"
4159,"PFC_01",1,"OID30194","Q8ND90","PNMA1",0.0682,"Cardiometabolic_II","B13805","20212097_SS211647","PASS",0.5529,1.8762,"Plate control","PASS","PFC",3.67106841211547,"Baseline","Baseline"
4160,"PFC_09",2,"OID30194","Q8ND90","PNMA1",0.0682,"Cardiometabolic_II","B13805","20212097_SS211647","PASS",0.5529,2.6052,"Plate control","PASS","PFC",6.08475848113504,"Baseline","Baseline"
4161,"PFC_17",3,"OID30194","Q8ND90","PNMA1",0.0682,"Cardiometabolic_II","B13805","20212097_SS211647","PASS",0.5529,3.7574,"Plate control","PASS","PFC",13.5235311498496,"1h","1h"
4162,"PFC_25",4,"OID30194","Q8ND90","PNMA1",0.0682,"Cardiometabolic_II","B13805","20212097_SS211647","PASS",0.5529,3.1231,"Plate control","PASS","PFC",8.71258001178647,"24h","24h"
4163,"PFC_02",13,"OID30194","Q8ND90","PNMA1",0.0682,"Cardiometabolic_II","B13805","20212097_SS211647","PASS",0.5529,1.9222,"Plate control","PASS","PFC",3.7900056523268,"Baseline","Baseline"
4164,"PFC_10",14,"OID30194","Q8ND90","PNMA1",0.0682,"Cardiometabolic_II","B13805","20212097_SS211647","PASS",0.5529,2.2246,"Plate control","PASS","PFC",4.67381295847472,"1h","1h"
4165,"PFC_18",15,"OID30194","Q8ND90","PNMA1",0.0682,"Cardiometabolic_II","B13805","20212097_SS211647","PASS",0.5529,3.0972,"Plate control","PASS","PFC",8.55756295098201,"1h","1h"
4166,"PFC_26",16,"OID30194","Q8ND90","PNMA1",0.0682,"Cardiometabolic_II","B13805","20212097_SS211647","PASS",0.5529,3.0691,"Plate control","PASS","PFC",8.39249632395551,"24h","24h"
4167,"PFC_03",25,"OID30194","Q8ND90","PNMA1",0.0682,"Cardiometabolic_II","B13805","20212097_SS211647","PASS",0.5529,1.3139,"Plate control","PASS","PFC",2.48612700664289,"Baseline","Baseline"
4168,"PFC_11",26,"OID30194","Q8ND90","PNMA1",0.0682,"Cardiometabolic_II","B13805","20212097_SS211647","PASS",0.5529,2.8827,"Plate control","PASS","PFC",7.37529113515651,"1h","1h"
4169,"PFC_19",27,"OID30194","Q8ND90","PNMA1",0.0682,"Cardiometabolic_II","B13805","20212097_SS211647","PASS",0.5529,3.0165,"Plate control","PASS","PFC",8.09202064153346,"24h","24h"
4170,"PFC_27",28,"OID30194","Q8ND90","PNMA1",0.0682,"Cardiometabolic_II","B13805","20212097_SS211647","PASS",0.5529,4.0222,"Plate control","PASS","PFC",16.2481099241057,"24h","24h"
4171,"PFC_04",37,"OID30194","Q8ND90","PNMA1",0.0682,"Cardiometabolic_II","B13805","20212097_SS211647","PASS",0.5529,2.6515,"Plate control","PASS","PFC",6.28320216401668,"Baseline","Baseline"
4172,"PFC_12",38,"OID30194","Q8ND90","PNMA1",0.0682,"Cardiometabolic_II","B13805","20212097_SS211647","PASS",0.5529,1.6001,"Plate control","PASS","PFC",3.03164326323619,"1h","1h"
4173,"PFC_20",39,"OID30194","Q8ND90","PNMA1",0.0682,"Cardiometabolic_II","B13805","20212097_SS211647","PASS",0.5529,3.1213,"Plate control","PASS","PFC",8.70171640977628,"24h","24h"
4174,"PFC_05",49,"OID30194","Q8ND90","PNMA1",0.0682,"Cardiometabolic_II","B13805","20212097_SS211647","PASS",0.5529,0.9707,"Plate control","PASS","PFC",1.95979126117321,"Baseline","Baseline"
4175,"PFC_13",50,"OID30194","Q8ND90","PNMA1",0.0682,"Cardiometabolic_II","B13805","20212097_SS211647","PASS",0.5529,2.9979,"Plate control","PASS","PFC",7.98836359844707,"1h","1h"
4176,"PFC_21",51,"OID30194","Q8ND90","PNMA1",0.0682,"Cardiometabolic_II","B13805","20212097_SS211647","PASS",0.5529,2.3234,"Plate control","PASS","PFC",5.00510383753883,"24h","24h"
4177,"PFC_06",61,"OID30194","Q8ND90","PNMA1",0.0682,"Cardiometabolic_II","B13805","20212097_SS211647","PASS",0.5529,2.3259,"Plate control","PASS","PFC",5.01378454065621,"Baseline","Baseline"
4178,"PFC_14",62,"OID30194","Q8ND90","PNMA1",0.0682,"Cardiometabolic_II","B13805","20212097_SS211647","PASS",0.5529,2.3413,"Plate control","PASS","PFC",5.06759068119507,"1h","1h"
4179,"PFC_22",63,"OID30194","Q8ND90","PNMA1",0.0682,"Cardiometabolic_II","B13805","20212097_SS211647","PASS",0.5529,2.2094,"Plate control","PASS","PFC",4.62482892332609,"24h","24h"
4180,"PFC_07",73,"OID30194","Q8ND90","PNMA1",0.0682,"Cardiometabolic_II","B13805","20212097_SS211647","PASS",0.5529,2.9111,"Plate control","PASS","PFC",7.52191498141997,"Baseline","Baseline"
4181,"PFC_15",74,"OID30194","Q8ND90","PNMA1",0.0682,"Cardiometabolic_II","B13805","20212097_SS211647","PASS",0.5529,2.0621,"Plate control","PASS","PFC",4.17593715356182,"1h","1h"
4182,"PFC_23",75,"OID30194","Q8ND90","PNMA1",0.0682,"Cardiometabolic_II","B13805","20212097_SS211647","PASS",0.5529,1.9057,"Plate control","PASS","PFC",3.74690655915933,"24h","24h"
4183,"PFC_08",85,"OID30194","Q8ND90","PNMA1",0.0682,"Cardiometabolic_II","B13805","20212097_SS211647","PASS",0.5529,2.215,"Plate control","PASS","PFC",4.64281565753488,"Baseline","Baseline"
4184,"PFC_16",86,"OID30194","Q8ND90","PNMA1",0.0682,"Cardiometabolic_II","B13805","20212097_SS211647","PASS",0.5529,3.1306,"Plate control","PASS","PFC",8.75799119893046,"1h","1h"
4185,"PFC_24",87,"OID30194","Q8ND90","PNMA1",0.0682,"Cardiometabolic_II","B13805","20212097_SS211647","PASS",0.5529,3.3291,"Plate control","PASS","PFC",10.0498356265294,"24h","24h"
4186,"PFC_01",1,"OID30195","Q5JTV8","TOR1AIP1",0.0682,"Cardiometabolic_II","B13805","20212097_SS211647","PASS",-1.6734,-0.7853,"Plate control","PASS","PFC",0.580231288941566,"Baseline","Baseline"
4187,"PFC_09",2,"OID30195","Q5JTV8","TOR1AIP1",0.0682,"Cardiometabolic_II","B13805","20212097_SS211647","PASS",-1.6734,-0.2,"Plate control","PASS","PFC",0.870550563296124,"Baseline","Baseline"
4188,"PFC_17",3,"OID30195","Q5JTV8","TOR1AIP1",0.0682,"Cardiometabolic_II","B13805","20212097_SS211647","PASS",-1.6734,1.8061,"Plate control","PASS","PFC",3.49695686382014,"1h","1h"
4189,"PFC_25",4,"OID30195","Q5JTV8","TOR1AIP1",0.0682,"Cardiometabolic_II","B13805","20212097_SS211647","PASS",-1.6734,-0.9853,"Plate control","PASS","PFC",0.505120675430116,"24h","24h"
4190,"PFC_02",13,"OID30195","Q5JTV8","TOR1AIP1",0.0682,"Cardiometabolic_II","B13805","20212097_SS211647","PASS",-1.6734,-0.1498,"Plate control","PASS","PFC",0.901375411114828,"Baseline","Baseline"
4191,"PFC_10",14,"OID30195","Q5JTV8","TOR1AIP1",0.0682,"Cardiometabolic_II","B13805","20212097_SS211647","PASS",-1.6734,-0.7667,"Plate control","PASS","PFC",0.587760372836743,"1h","1h"
4192,"PFC_18",15,"OID30195","Q5JTV8","TOR1AIP1",0.0682,"Cardiometabolic_II","B13805","20212097_SS211647","PASS",-1.6734,-0.0159,"Plate control","PASS","PFC",0.989039468996991,"1h","1h"
4193,"PFC_26",16,"OID30195","Q5JTV8","TOR1AIP1",0.0682,"Cardiometabolic_II","B13805","20212097_SS211647","PASS",-1.6734,1.1421,"Plate control","PASS","PFC",2.20702045378269,"24h","24h"
4194,"PFC_03",25,"OID30195","Q5JTV8","TOR1AIP1",0.0682,"Cardiometabolic_II","B13805","20212097_SS211647","PASS",-1.6734,-1.4586,"Plate control","PASS","PFC",0.363846036466842,"Baseline","Baseline"
4195,"PFC_11",26,"OID30195","Q5JTV8","TOR1AIP1",0.0682,"Cardiometabolic_II","B13805","20212097_SS211647","PASS",-1.6734,0.3361,"Plate control","PASS","PFC",1.26233952778817,"1h","1h"
4196,"PFC_19",27,"OID30195","Q5JTV8","TOR1AIP1",0.0682,"Cardiometabolic_II","B13805","20212097_SS211647","PASS",-1.6734,0.0863,"Plate control","PASS","PFC",1.06164394865116,"24h","24h"
4197,"PFC_27",28,"OID30195","Q5JTV8","TOR1AIP1",0.0682,"Cardiometabolic_II","B13805","20212097_SS211647","PASS",-1.6734,0.7973,"Plate control","PASS","PFC",1.73784570758669,"24h","24h"
4198,"PFC_04",37,"OID30195","Q5JTV8","TOR1AIP1",0.0682,"Cardiometabolic_II","B13805","20212097_SS211647","PASS",-1.6734,-0.2407,"Plate control","PASS","PFC",0.846334568629495,"Baseline","Baseline"
4199,"PFC_12",38,"OID30195","Q5JTV8","TOR1AIP1",0.0682,"Cardiometabolic_II","B13805","20212097_SS211647","PASS",-1.6734,-1.7375,"Plate control","PASS","PFC",0.299888894010226,"1h","1h"
4200,"PFC_20",39,"OID30195","Q5JTV8","TOR1AIP1",0.0682,"Cardiometabolic_II","B13805","20212097_SS211647","PASS",-1.6734,0.5499,"Plate control","PASS","PFC",1.46398421677544,"24h","24h"
4201,"PFC_05",49,"OID30195","Q5JTV8","TOR1AIP1",0.0682,"Cardiometabolic_II","B13805","20212097_SS211647","PASS",-1.6734,-0.0839,"Plate control","PASS","PFC",0.943503647318165,"Baseline","Baseline"
4202,"PFC_13",50,"OID30195","Q5JTV8","TOR1AIP1",0.0682,"Cardiometabolic_II","B13805","20212097_SS211647","PASS",-1.6734,-0.0947,"Plate control","PASS","PFC",0.936466960019026,"1h","1h"
4203,"PFC_21",51,"OID30195","Q5JTV8","TOR1AIP1",0.0682,"Cardiometabolic_II","B13805","20212097_SS211647","PASS",-1.6734,-1.1981,"Plate control","PASS","PFC",0.435848907977311,"24h","24h"
4204,"PFC_06",61,"OID30195","Q5JTV8","TOR1AIP1",0.0682,"Cardiometabolic_II","B13805","20212097_SS211647","PASS",-1.6734,-0.2036,"Plate control","PASS","PFC",0.868380960552611,"Baseline","Baseline"
4205,"PFC_14",62,"OID30195","Q5JTV8","TOR1AIP1",0.0682,"Cardiometabolic_II","B13805","20212097_SS211647","PASS",-1.6734,0.822,"Plate control","PASS","PFC",1.76785506212734,"1h","1h"
4206,"PFC_22",63,"OID30195","Q5JTV8","TOR1AIP1",0.0682,"Cardiometabolic_II","B13805","20212097_SS211647","PASS",-1.6734,-0.2738,"Plate control","PASS","PFC",0.827138026173769,"24h","24h"
4207,"PFC_07",73,"OID30195","Q5JTV8","TOR1AIP1",0.0682,"Cardiometabolic_II","B13805","20212097_SS211647","PASS",-1.6734,-0.7201,"Plate control","PASS","PFC",0.60705536286786,"Baseline","Baseline"
4208,"PFC_15",74,"OID30195","Q5JTV8","TOR1AIP1",0.0682,"Cardiometabolic_II","B13805","20212097_SS211647","PASS",-1.6734,-0.8802,"Plate control","PASS","PFC",0.543292109763441,"1h","1h"
4209,"PFC_23",75,"OID30195","Q5JTV8","TOR1AIP1",0.0682,"Cardiometabolic_II","B13805","20212097_SS211647","PASS",-1.6734,0.6514,"Plate control","PASS","PFC",1.57069166518094,"24h","24h"
4210,"PFC_08",85,"OID30195","Q5JTV8","TOR1AIP1",0.0682,"Cardiometabolic_II","B13805","20212097_SS211647","PASS",-1.6734,0.2192,"Plate control","PASS","PFC",1.16408790006603,"Baseline","Baseline"
4211,"PFC_16",86,"OID30195","Q5JTV8","TOR1AIP1",0.0682,"Cardiometabolic_II","B13805","20212097_SS211647","PASS",-1.6734,-1.2879,"Plate control","PASS","PFC",0.409546735562603,"1h","1h"
4212,"PFC_24",87,"OID30195","Q5JTV8","TOR1AIP1",0.0682,"Cardiometabolic_II","B13805","20212097_SS211647","PASS",-1.6734,0.4073,"Plate control","PASS","PFC",1.32620150747839,"24h","24h"
4213,"PFC_01",1,"OID30196","Q9UBV2","SEL1L",0,"Cardiometabolic_II","B13805","20212097_SS211647","PASS",-2.1153,0.0947,"Plate control","PASS","PFC",1.06784333318036,"Baseline","Baseline"
4214,"PFC_09",2,"OID30196","Q9UBV2","SEL1L",0,"Cardiometabolic_II","B13805","20212097_SS211647","PASS",-2.1153,0.1612,"Plate control","PASS","PFC",1.11821685799382,"Baseline","Baseline"
4215,"PFC_17",3,"OID30196","Q9UBV2","SEL1L",0,"Cardiometabolic_II","B13805","20212097_SS211647","PASS",-2.1153,0.9305,"Plate control","PASS","PFC",1.90593642886798,"1h","1h"
4216,"PFC_25",4,"OID30196","Q9UBV2","SEL1L",0,"Cardiometabolic_II","B13805","20212097_SS211647","PASS",-2.1153,0.668,"Plate control","PASS","PFC",1.58886880019221,"24h","24h"
4217,"PFC_02",13,"OID30196","Q9UBV2","SEL1L",0,"Cardiometabolic_II","B13805","20212097_SS211647","PASS",-2.1153,0.2488,"Plate control","PASS","PFC",1.18821837159591,"Baseline","Baseline"
4218,"PFC_10",14,"OID30196","Q9UBV2","SEL1L",0,"Cardiometabolic_II","B13805","20212097_SS211647","PASS",-2.1153,-0.0073,"Plate control","PASS","PFC",0.994952805687713,"1h","1h"
4219,"PFC_18",15,"OID30196","Q9UBV2","SEL1L",0,"Cardiometabolic_II","B13805","20212097_SS211647","PASS",-2.1153,0.0814,"Plate control","PASS","PFC",1.05804427511742,"1h","1h"
4220,"PFC_26",16,"OID30196","Q9UBV2","SEL1L",0,"Cardiometabolic_II","B13805","20212097_SS211647","PASS",-2.1153,0.2842,"Plate control","PASS","PFC",1.21773482085379,"24h","24h"
4221,"PFC_03",25,"OID30196","Q9UBV2","SEL1L",0,"Cardiometabolic_II","B13805","20212097_SS211647","PASS",-2.1153,-0.0138,"Plate control","PASS","PFC",0.990480172123622,"Baseline","Baseline"
4222,"PFC_11",26,"OID30196","Q9UBV2","SEL1L",0,"Cardiometabolic_II","B13805","20212097_SS211647","PASS",-2.1153,0.3874,"Plate control","PASS","PFC",1.30803396228671,"1h","1h"
4223,"PFC_19",27,"OID30196","Q9UBV2","SEL1L",0,"Cardiometabolic_II","B13805","20212097_SS211647","PASS",-2.1153,0.3521,"Plate control","PASS","PFC",1.27641724026386,"24h","24h"
4224,"PFC_27",28,"OID30196","Q9UBV2","SEL1L",0,"Cardiometabolic_II","B13805","20212097_SS211647","PASS",-2.1153,0.2527,"Plate control","PASS","PFC",1.19143479691189,"24h","24h"
4225,"PFC_04",37,"OID30196","Q9UBV2","SEL1L",0,"Cardiometabolic_II","B13805","20212097_SS211647","PASS",-2.1153,0.1974,"Plate control","PASS","PFC",1.14663005501621,"Baseline","Baseline"
4226,"PFC_12",38,"OID30196","Q9UBV2","SEL1L",0,"Cardiometabolic_II","B13805","20212097_SS211647","PASS",-2.1153,-0.0433,"Plate control","PASS","PFC",0.970432652989761,"1h","1h"
4227,"PFC_20",39,"OID30196","Q9UBV2","SEL1L",0,"Cardiometabolic_II","B13805","20212097_SS211647","PASS",-2.1153,0.3521,"Plate control","PASS","PFC",1.27641724026386,"24h","24h"
4228,"PFC_05",49,"OID30196","Q9UBV2","SEL1L",0,"Cardiometabolic_II","B13805","20212097_SS211647","PASS",-2.1153,0.105,"Plate control","PASS","PFC",1.07549439045738,"Baseline","Baseline"
4229,"PFC_13",50,"OID30196","Q9UBV2","SEL1L",0,"Cardiometabolic_II","B13805","20212097_SS211647","PASS",-2.1153,0.3921,"Plate control","PASS","PFC",1.31230221329745,"1h","1h"
4230,"PFC_21",51,"OID30196","Q9UBV2","SEL1L",0,"Cardiometabolic_II","B13805","20212097_SS211647","PASS",-2.1153,-0.1387,"Plate control","PASS","PFC",0.908337281511972,"24h","24h"
4231,"PFC_06",61,"OID30196","Q9UBV2","SEL1L",0,"Cardiometabolic_II","B13805","20212097_SS211647","PASS",-2.1153,0.2151,"Plate control","PASS","PFC",1.16078437103667,"Baseline","Baseline"
4232,"PFC_14",62,"OID30196","Q9UBV2","SEL1L",0,"Cardiometabolic_II","B13805","20212097_SS211647","PASS",-2.1153,0.0979,"Plate control","PASS","PFC",1.07021451423831,"1h","1h"
4233,"PFC_22",63,"OID30196","Q9UBV2","SEL1L",0,"Cardiometabolic_II","B13805","20212097_SS211647","PASS",-2.1153,0.3006,"Plate control","PASS","PFC",1.23165653839833,"24h","24h"
4234,"PFC_07",73,"OID30196","Q9UBV2","SEL1L",0,"Cardiometabolic_II","B13805","20212097_SS211647","PASS",-2.1153,0.4956,"Plate control","PASS","PFC",1.40990699706391,"Baseline","Baseline"
4235,"PFC_15",74,"OID30196","Q9UBV2","SEL1L",0,"Cardiometabolic_II","B13805","20212097_SS211647","PASS",-2.1153,-0.2839,"Plate control","PASS","PFC",0.821367631644776,"1h","1h"
4236,"PFC_23",75,"OID30196","Q9UBV2","SEL1L",0,"Cardiometabolic_II","B13805","20212097_SS211647","PASS",-2.1153,0.2305,"Plate control","PASS","PFC",1.17324149329569,"24h","24h"
4237,"PFC_08",85,"OID30196","Q9UBV2","SEL1L",0,"Cardiometabolic_II","B13805","20212097_SS211647","PASS",-2.1153,-0.4187,"Plate control","PASS","PFC",0.748098425702507,"Baseline","Baseline"
4238,"PFC_16",86,"OID30196","Q9UBV2","SEL1L",0,"Cardiometabolic_II","B13805","20212097_SS211647","PASS",-2.1153,0.2837,"Plate control","PASS","PFC",1.21731285924951,"1h","1h"
4239,"PFC_24",87,"OID30196","Q9UBV2","SEL1L",0,"Cardiometabolic_II","B13805","20212097_SS211647","PASS",-2.1153,0.1966,"Plate control","PASS","PFC",1.14599440456041,"24h","24h"
4240,"PFC_01",1,"OID30197","P46926","GNPDA1",0,"Cardiometabolic_II","B13805","20212097_SS211647","PASS",-0.4585,1.5565,"Plate control","PASS","PFC",2.94139390559458,"Baseline","Baseline"
4241,"PFC_09",2,"OID30197","P46926","GNPDA1",0,"Cardiometabolic_II","B13805","20212097_SS211647","PASS",-0.4585,0.6822,"Plate control","PASS","PFC",1.6045847599676,"Baseline","Baseline"
4242,"PFC_17",3,"OID30197","P46926","GNPDA1",0,"Cardiometabolic_II","B13805","20212097_SS211647","PASS",-0.4585,0.3888,"Plate control","PASS","PFC",1.30930390244011,"1h","1h"
4243,"PFC_25",4,"OID30197","P46926","GNPDA1",0,"Cardiometabolic_II","B13805","20212097_SS211647","PASS",-0.4585,0.3425,"Plate control","PASS","PFC",1.26795188453123,"24h","24h"
4244,"PFC_02",13,"OID30197","P46926","GNPDA1",0,"Cardiometabolic_II","B13805","20212097_SS211647","PASS",-0.4585,-0.0989,"Plate control","PASS","PFC",0.933744664920448,"Baseline","Baseline"
4245,"PFC_10",14,"OID30197","P46926","GNPDA1",0,"Cardiometabolic_II","B13805","20212097_SS211647","PASS",-0.4585,1.4999,"Plate control","PASS","PFC",2.82823107991197,"1h","1h"
4246,"PFC_18",15,"OID30197","P46926","GNPDA1",0,"Cardiometabolic_II","B13805","20212097_SS211647","PASS",-0.4585,0.0236,"Plate control","PASS","PFC",1.01649280257012,"1h","1h"
4247,"PFC_26",16,"OID30197","P46926","GNPDA1",0,"Cardiometabolic_II","B13805","20212097_SS211647","PASS",-0.4585,-0.3489,"Plate control","PASS","PFC",0.785182541493886,"24h","24h"
4248,"PFC_03",25,"OID30197","P46926","GNPDA1",0,"Cardiometabolic_II","B13805","20212097_SS211647","PASS",-0.4585,0.8284,"Plate control","PASS","PFC",1.77571493901488,"Baseline","Baseline"
4249,"PFC_11",26,"OID30197","P46926","GNPDA1",0,"Cardiometabolic_II","B13805","20212097_SS211647","PASS",-0.4585,0.0869,"Plate control","PASS","PFC",1.06208556578236,"1h","1h"
4250,"PFC_19",27,"OID30197","P46926","GNPDA1",0,"Cardiometabolic_II","B13805","20212097_SS211647","PASS",-0.4585,1.3183,"Plate control","PASS","PFC",2.49372088931979,"24h","24h"
4251,"PFC_27",28,"OID30197","P46926","GNPDA1",0,"Cardiometabolic_II","B13805","20212097_SS211647","PASS",-0.4585,0.5869,"Plate control","PASS","PFC",1.50201581153011,"24h","24h"
4252,"PFC_04",37,"OID30197","P46926","GNPDA1",0,"Cardiometabolic_II","B13805","20212097_SS211647","PASS",-0.4585,0.3059,"Plate control","PASS","PFC",1.23618957181959,"Baseline","Baseline"
4253,"PFC_12",38,"OID30197","P46926","GNPDA1",0,"Cardiometabolic_II","B13805","20212097_SS211647","PASS",-0.4585,0.4734,"Plate control","PASS","PFC",1.38837760566567,"1h","1h"
4254,"PFC_20",39,"OID30197","P46926","GNPDA1",0,"Cardiometabolic_II","B13805","20212097_SS211647","PASS",-0.4585,-0.2763,"Plate control","PASS","PFC",0.825705946357795,"24h","24h"
4255,"PFC_05",49,"OID30197","P46926","GNPDA1",0,"Cardiometabolic_II","B13805","20212097_SS211647","PASS",-0.4585,0.1787,"Plate control","PASS","PFC",1.13186351323676,"Baseline","Baseline"
4256,"PFC_13",50,"OID30197","P46926","GNPDA1",0,"Cardiometabolic_II","B13805","20212097_SS211647","PASS",-0.4585,0.5944,"Plate control","PASS","PFC",1.50984452828432,"1h","1h"

Supplemental table 1_Olink proximity extension assay (PEA) data

**,"SampleID","Index","OlinkID","UniProt","Assay","MissingFreq","Panel","Panel_Lot_Nr","PlateID","QC_Warning","LOD","NPX","Normalization","Assay_Warning","condition","Value","group","alt_group"**
4257,"PFC_21",51,"OID30197","P46926","GNPDA1",0,"Cardiometabolic_II","B13805","20212097_SS211647","PASS",-0.4585,0.9219,"Plate control","PASS","PFC",1.89460881237142,"24h","24h"
4258,"PFC_06",61,"OID30197","P46926","GNPDA1",0,"Cardiometabolic_II","B13805","20212097_SS211647","PASS",-0.4585,-0.0752,"Plate control","PASS","PFC",0.949210523309479,"Baseline","Baseline"
4259,"PFC_14",62,"OID30197","P46926","GNPDA1",0,"Cardiometabolic_II","B13805","20212097_SS211647","PASS",-0.4585,0.0895,"Plate control","PASS","PFC",1.06400136377294,"1h","1h"
4260,"PFC_22",63,"OID30197","P46926","GNPDA1",0,"Cardiometabolic_II","B13805","20212097_SS211647","PASS",-0.4585,0.3325,"Plate control","PASS","PFC",1.25919350110321,"24h","24h"
4261,"PFC_07",73,"OID30197","P46926","GNPDA1",0,"Cardiometabolic_II","B13805","20212097_SS211647","PASS",-0.4585,0.4905,"Plate control","PASS","PFC",1.4049317035969,"Baseline","Baseline"
4262,"PFC_15",74,"OID30197","P46926","GNPDA1",0,"Cardiometabolic_II","B13805","20212097_SS211647","PASS",-0.4585,-0.167,"Plate control","PASS","PFC",0.890692900606681,"1h","1h"
4263,"PFC_23",75,"OID30197","P46926","GNPDA1",0,"Cardiometabolic_II","B13805","20212097_SS211647","PASS",-0.4585,0.4948,"Plate control","PASS","PFC",1.40912539534206,"24h","24h"
4264,"PFC_08",85,"OID30197","P46926","GNPDA1",0,"Cardiometabolic_II","B13805","20212097_SS211647","PASS",-0.4585,-0.2104,"Plate control","PASS","PFC",0.864297563916608,"Baseline","Baseline"
4265,"PFC_16",86,"OID30197","P46926","GNPDA1",0,"Cardiometabolic_II","B13805","20212097_SS211647","PASS",-0.4585,0.2857,"Plate control","PASS","PFC",1.21900158346599,"1h","1h"
4266,"PFC_24",87,"OID30197","P46926","GNPDA1",0,"Cardiometabolic_II","B13805","20212097_SS211647","PASS",-0.4585,0.0722,"Plate control","PASS","PFC",1.0513186426966,"24h","24h"
4267,"PFC_01",1,"OID30198","Q8NFP7","NUDT10",0.9091,"Cardiometabolic_II","B13805","20212097_SS211647","PASS",0.9955,0.3361,"Plate control","PASS","PFC",1.26233952778817,"Baseline","Baseline"
4268,"PFC_09",2,"OID30198","Q8NFP7","NUDT10",0.9091,"Cardiometabolic_II","B13805","20212097_SS211647","PASS",0.9955,0.1593,"Plate control","PASS","PFC",1.11674515846718,"Baseline","Baseline"
4269,"PFC_17",3,"OID30198","Q8NFP7","NUDT10",0.9091,"Cardiometabolic_II","B13805","20212097_SS211647","PASS",0.9955,0.4675,"Plate control","PASS","PFC",1.38271133472879,"1h","1h"
4270,"PFC_25",4,"OID30198","Q8NFP7","NUDT10",0.9091,"Cardiometabolic_II","B13805","20212097_SS211647","PASS",0.9955,0.3864,"Plate control","PASS","PFC",1.30712761638552,"24h","24h"
4271,"PFC_02",13,"OID30198","Q8NFP7","NUDT10",0.9091,"Cardiometabolic_II","B13805","20212097_SS211647","PASS",0.9955,0.023,"Plate control","PASS","PFC",1.0160701429937,"Baseline","Baseline"
4272,"PFC_10",14,"OID30198","Q8NFP7","NUDT10",0.9091,"Cardiometabolic_II","B13805","20212097_SS211647","PASS",0.9955,0.3644,"Plate control","PASS","PFC",1.28734612596014,"1h","1h"
4273,"PFC_18",15,"OID30198","Q8NFP7","NUDT10",0.9091,"Cardiometabolic_II","B13805","20212097_SS211647","PASS",0.9955,0.2613,"Plate control","PASS","PFC",1.19855822860499,"1h","1h"
4274,"PFC_26",16,"OID30198","Q8NFP7","NUDT10",0.9091,"Cardiometabolic_II","B13805","20212097_SS211647","PASS",0.9955,0.3288,"Plate control","PASS","PFC",1.25596825490334,"24h","24h"
4275,"PFC_03",25,"OID30198","Q8NFP7","NUDT10",0.9091,"Cardiometabolic_II","B13805","20212097_SS211647","PASS",0.9955,0.01,"Plate control","PASS","PFC",1.00695555005672,"Baseline","Baseline"
4276,"PFC_11",26,"OID30198","Q8NFP7","NUDT10",0.9091,"Cardiometabolic_II","B13805","20212097_SS211647","PASS",0.9955,0.1914,"Plate control","PASS","PFC",1.14187125717858,"1h","1h"
4277,"PFC_19",27,"OID30198","Q8NFP7","NUDT10",0.9091,"Cardiometabolic_II","B13805","20212097_SS211647","PASS",0.9955,0.4178,"Plate control","PASS","PFC",1.33588887203183,"24h","24h"
4278,"PFC_27",28,"OID30198","Q8NFP7","NUDT10",0.9091,"Cardiometabolic_II","B13805","20212097_SS211647","PASS",0.9955,0.5234,"Plate control","PASS","PFC",1.43733863619255,"24h","24h"
4279,"PFC_04",37,"OID30198","Q8NFP7","NUDT10",0.9091,"Cardiometabolic_II","B13805","20212097_SS211647","PASS",0.9955,0.2594,"Plate control","PASS","PFC",1.19698079077165,"Baseline","Baseline"
4280,"PFC_12",38,"OID30198","Q8NFP7","NUDT10",0.9091,"Cardiometabolic_II","B13805","20212097_SS211647","PASS",0.9955,-0.0185,"Plate control","PASS","PFC",0.987258644374183,"1h","1h"
4281,"PFC_20",39,"OID30198","Q8NFP7","NUDT10",0.9091,"Cardiometabolic_II","B13805","20212097_SS211647","PASS",0.9955,0.1615,"Plate control","PASS","PFC",1.11844940883049,"24h","24h"
4282,"PFC_05",49,"OID30198","Q8NFP7","NUDT10",0.9091,"Cardiometabolic_II","B13805","20212097_SS211647","PASS",0.9955,-0.0773,"Plate control","PASS","PFC",0.947829848958541,"Baseline","Baseline"
4283,"PFC_13",50,"OID30198","Q8NFP7","NUDT10",0.9091,"Cardiometabolic_II","B13805","20212097_SS211647","PASS",0.9955,-0.0827,"Plate control","PASS","PFC",0.944288758062864,"1h","1h"
4284,"PFC_21",51,"OID30198","Q8NFP7","NUDT10",0.9091,"Cardiometabolic_II","B13805","20212097_SS211647","PASS",0.9955,-0.0376,"Plate control","PASS","PFC",0.974274357308802,"24h","24h"
4285,"PFC_06",61,"OID30198","Q8NFP7","NUDT10",0.9091,"Cardiometabolic_II","B13805","20212097_SS211647","PASS",0.9955,0.2002,"Plate control","PASS","PFC",1.14885760944067,"Baseline","Baseline"
4286,"PFC_14",62,"OID30198","Q8NFP7","NUDT10",0.9091,"Cardiometabolic_II","B13805","20212097_SS211647","PASS",0.9955,0.2443,"Plate control","PASS","PFC",1.18451789981845,"1h","1h"
4287,"PFC_22",63,"OID30198","Q8NFP7","NUDT10",0.9091,"Cardiometabolic_II","B13805","20212097_SS211647","PASS",0.9955,0.1609,"Plate control","PASS","PFC",1.11798435550971,"24h","24h"
4288,"PFC_07",73,"OID30198","Q8NFP7","NUDT10",0.9091,"Cardiometabolic_II","B13805","20212097_SS211647","PASS",0.9955,0.3279,"Plate control","PASS","PFC",1.25518498547391,"Baseline","Baseline"
4289,"PFC_15",74,"OID30198","Q8NFP7","NUDT10",0.9091,"Cardiometabolic_II","B13805","20212097_SS211647","PASS",0.9955,-0.2824,"Plate control","PASS","PFC",0.8222206874292,"1h","1h"
4290,"PFC_23",75,"OID30198","Q8NFP7","NUDT10",0.9091,"Cardiometabolic_II","B13805","20212097_SS211647","PASS",0.9955,0.1056,"Plate control","PASS","PFC",1.07594176902336,"24h","24h"
4291,"PFC_08",85,"OID30198","Q8NFP7","NUDT10",0.9091,"Cardiometabolic_II","B13805","20212097_SS211647","PASS",0.9955,-0.0136,"Plate control","PASS","PFC",0.990617491349387,"Baseline","Baseline"
4292,"PFC_16",86,"OID30198","Q8NFP7","NUDT10",0.9091,"Cardiometabolic_II","B13805","20212097_SS211647","PASS",0.9955,0.109,"Plate control","PASS","PFC",1.07848043169445,"1h","1h"
4293,"PFC_24",87,"OID30198","Q8NFP7","NUDT10",0.9091,"Cardiometabolic_II","B13805","20212097_SS211647","PASS",0.9955,0.1956,"Plate control","PASS","PFC",1.14520033700458,"24h","24h"
4294,"PFC_01",1,"OID30199","Q14324","MYBPC2",0.0909,"Cardiometabolic_II","B13805","20212097_SS211647","PASS",-0.8263,0.2932,"Plate control","PASS","PFC",1.22535519042979,"Baseline","Baseline"
4295,"PFC_09",2,"OID30199","Q14324","MYBPC2",0.0909,"Cardiometabolic_II","B13805","20212097_SS211647","PASS",-0.8263,-0.3283,"Plate control","PASS","PFC",0.796474456857057,"Baseline","Baseline"
4296,"PFC_17",3,"OID30199","Q14324","MYBPC2",0.0909,"Cardiometabolic_II","B13805","20212097_SS211647","PASS",-0.8263,2.0396,"Plate control","PASS","PFC",4.1113152499566,"1h","1h"
4297,"PFC_25",4,"OID30199","Q14324","MYBPC2",0.0909,"Cardiometabolic_II","B13805","20212097_SS211647","PASS",-0.8263,-1.0859,"Plate control","PASS","PFC",0.471098289865866,"24h","24h"
4298,"PFC_02",13,"OID30199","Q14324","MYBPC2",0.0909,"Cardiometabolic_II","B13805","20212097_SS211647","PASS",-0.8263,0.2905,"Plate control","PASS","PFC",1.22306408595886,"Baseline","Baseline"
4299,"PFC_10",14,"OID30199","Q14324","MYBPC2",0.0909,"Cardiometabolic_II","B13805","20212097_SS211647","PASS",-0.8263,0.2305,"Plate control","PASS","PFC",1.17324149329569,"1h","1h"
4300,"PFC_18",15,"OID30199","Q14324","MYBPC2",0.0909,"Cardiometabolic_II","B13805","20212097_SS211647","PASS",-0.8263,1.318,"Plate control","PASS","PFC",2.49320238855019,"1h","1h"
4301,"PFC_26",16,"OID30199","Q14324","MYBPC2",0.0909,"Cardiometabolic_II","B13805","20212097_SS211647","PASS",-0.8263,2.2091,"Plate control","PASS","PFC",4.62386731717112,"24h","24h"
4302,"PFC_03",25,"OID30199","Q14324","MYBPC2",0.0909,"Cardiometabolic_II","B13805","20212097_SS211647","PASS",-0.8263,0.2999,"Plate control","PASS","PFC",1.23105907987449,"Baseline","Baseline"
4303,"PFC_11",26,"OID30199","Q14324","MYBPC2",0.0909,"Cardiometabolic_II","B13805","20212097_SS211647","PASS",-0.8263,0.7951,"Plate control","PASS","PFC",1.73519764487135,"1h","1h"
4304,"PFC_19",27,"OID30199","Q14324","MYBPC2",0.0909,"Cardiometabolic_II","B13805","20212097_SS211647","PASS",-0.8263,-0.5672,"Plate control","PASS","PFC",0.674925421545068,"24h","24h"
4305,"PFC_27",28,"OID30199","Q14324","MYBPC2",0.0909,"Cardiometabolic_II","B13805","20212097_SS211647","PASS",-0.8263,0.0049,"Plate control","PASS","PFC",1.00340219555873,"24h","24h"
4306,"PFC_04",37,"OID30199","Q14324","MYBPC2",0.0909,"Cardiometabolic_II","B13805","20212097_SS211647","PASS",-0.8263,-0.6517,"Plate control","PASS","PFC",0.636529816531206,"Baseline","Baseline"
4307,"PFC_12",38,"OID30199","Q14324","MYBPC2",0.0909,"Cardiometabolic_II","B13805","20212097_SS211647","PASS",-0.8263,0.6928,"Plate control","PASS","PFC",1.61641763901234,"1h","1h"
4308,"PFC_20",39,"OID30199","Q14324","MYBPC2",0.0909,"Cardiometabolic_II","B13805","20212097_SS211647","PASS",-0.8263,-0.1384,"Plate control","PASS","PFC",0.908526184579639,"24h","24h"
4309,"PFC_05",49,"OID30199","Q14324","MYBPC2",0.0909,"Cardiometabolic_II","B13805","20212097_SS211647","PASS",-0.8263,-0.0653,"Plate control","PASS","PFC",0.955746555019718,"Baseline","Baseline"
4310,"PFC_13",50,"OID30199","Q14324","MYBPC2",0.0909,"Cardiometabolic_II","B13805","20212097_SS211647","PASS",-0.8263,-0.0015,"Plate control","PASS","PFC",0.998960819551523,"1h","1h"
4311,"PFC_21",51,"OID30199","Q14324","MYBPC2",0.0909,"Cardiometabolic_II","B13805","20212097_SS211647","PASS",-0.8263,0.5722,"Plate control","PASS","PFC",1.48678908287721,"24h","24h"
4312,"PFC_06",61,"OID30199","Q14324","MYBPC2",0.0909,"Cardiometabolic_II","B13805","20212097_SS211647","PASS",-0.8263,-0.7319,"Plate control","PASS","PFC",0.60211042428361,"Baseline","Baseline"
4313,"PFC_14",62,"OID30199","Q14324","MYBPC2",0.0909,"Cardiometabolic_II","B13805","20212097_SS211647","PASS",-0.8263,0.0038,"Plate control","PASS","PFC",1.00263743120452,"1h","1h"
4314,"PFC_22",63,"OID30199","Q14324","MYBPC2",0.0909,"Cardiometabolic_II","B13805","20212097_SS211647","PASS",-0.8263,-0.7343,"Plate control","PASS","PFC",0.601109614221675,"24h","24h"
4315,"PFC_07",73,"OID30199","Q14324","MYBPC2",0.0909,"Cardiometabolic_II","B13805","20212097_SS211647","PASS",-0.8263,-0.9612,"Plate control","PASS","PFC",0.513629510584334,"Baseline","Baseline"
4316,"PFC_15",74,"OID30199","Q14324","MYBPC2",0.0909,"Cardiometabolic_II","B13805","20212097_SS211647","PASS",-0.8263,-6e-04,"Plate control","PASS","PFC",0.999584198161219,"1h","1h"
4317,"PFC_23",75,"OID30199","Q14324","MYBPC2",0.0909,"Cardiometabolic_II","B13805","20212097_SS211647","PASS",-0.8263,-0.2612,"Plate control","PASS","PFC",0.834393601622812,"24h","24h"
4318,"PFC_08",85,"OID30199","Q14324","MYBPC2",0.0909,"Cardiometabolic_II","B13805","20212097_SS211647","PASS",-0.8263,2.0502,"Plate control","PASS","PFC",4.14163380792951,"Baseline","Baseline"
4319,"PFC_16",86,"OID30199","Q14324","MYBPC2",0.0909,"Cardiometabolic_II","B13805","20212097_SS211647","PASS",-0.8263,-1.2467,"Plate control","PASS","PFC",0.421411035602494,"1h","1h"
4320,"PFC_24",87,"OID30199","Q14324","MYBPC2",0.0909,"Cardiometabolic_II","B13805","20212097_SS211647","PASS",-0.8263,-0.4329,"Plate control","PASS","PFC",0.740771243580565,"24h","24h"
4321,"PFC_01",1,"OID30200","P35520","CBS",0,"Cardiometabolic_II","B13805","20212097_SS211647","PASS",-2.1048,-1.608,"Plate control","PASS","PFC",0.32805281364799,"Baseline","Baseline"
4322,"PFC_09",2,"OID30200","P35520","CBS",0,"Cardiometabolic_II","B13805","20212097_SS211647","PASS",-2.1048,-1.0471,"Plate control","PASS","PFC",0.483939968121843,"Baseline","Baseline"
4323,"PFC_17",3,"OID30200","P35520","CBS",0,"Cardiometabolic_II","B13805","20212097_SS211647","PASS",-2.1048,-0.834,"Plate control","PASS","PFC",0.560971740265242,"1h","1h"
4324,"PFC_25",4,"OID30200","P35520","CBS",0,"Cardiometabolic_II","B13805","20212097_SS211647","PASS",-2.1048,-1.595,"Plate control","PASS","PFC",0.331022227598849,"24h","24h"
4325,"PFC_02",13,"OID30200","P35520","CBS",0,"Cardiometabolic_II","B13805","20212097_SS211647","PASS",-2.1048,-1.0842,"Plate control","PASS","PFC",0.471653735823884,"Baseline","Baseline"
4326,"PFC_10",14,"OID30200","P35520","CBS",0,"Cardiometabolic_II","B13805","20212097_SS211647","PASS",-2.1048,-1.4552,"Plate control","PASS","PFC",0.364704523772936,"1h","1h"
4327,"PFC_18",15,"OID30200","P35520","CBS",0,"Cardiometabolic_II","B13805","20212097_SS211647","PASS",-2.1048,-0.9564,"Plate control","PASS","PFC",0.515341256652153,"1h","1h"
4328,"PFC_26",16,"OID30200","P35520","CBS",0,"Cardiometabolic_II","B13805","20212097_SS211647","PASS",-2.1048,-1.3087,"Plate control","PASS","PFC",0.403684472351327,"24h","24h"
4329,"PFC_03",25,"OID30200","P35520","CBS",0,"Cardiometabolic_II","B13805","20212097_SS211647","PASS",-2.1048,-1.6563,"Plate control","PASS","PFC",0.317251743999806,"Baseline","Baseline"
4330,"PFC_11",26,"OID30200","P35520","CBS",0,"Cardiometabolic_II","B13805","20212097_SS211647","PASS",-2.1048,-1.3619,"Plate control","PASS","PFC",0.389069555589633,"1h","1h"
4331,"PFC_19",27,"OID30200","P35520","CBS",0,"Cardiometabolic_II","B13805","20212097_SS211647","PASS",-2.1048,-1.0956,"Plate control","PASS","PFC",0.467941471856205,"24h","24h"
4332,"PFC_27",28,"OID30200","P35520","CBS",0,"Cardiometabolic_II","B13805","20212097_SS211647","PASS",-2.1048,-0.7183,"Plate control","PASS","PFC",0.607813237239971,"24h","24h"
4333,"PFC_04",37,"OID30200","P35520","CBS",0,"Cardiometabolic_II","B13805","20212097_SS211647","PASS",-2.1048,-1.4324,"Plate control","PASS","PFC",0.370514009911695,"Baseline","Baseline"
4334,"PFC_12",38,"OID30200","P35520","CBS",0,"Cardiometabolic_II","B13805","20212097_SS211647","PASS",-2.1048,-1.642,"Plate control","PASS","PFC",0.320411981040048,"1h","1h"
4335,"PFC_20",39,"OID30200","P35520","CBS",0,"Cardiometabolic_II","B13805","20212097_SS211647","PASS",-2.1048,-1.0726,"Plate control","PASS","PFC",0.475461358221617,"24h","24h"
4336,"PFC_05",49,"OID30200","P35520","CBS",0,"Cardiometabolic_II","B13805","20212097_SS211647","PASS",-2.1048,-1.0791,"Plate control","PASS","PFC",0.473324006161245,"Baseline","Baseline"
4337,"PFC_13",50,"OID30200","P35520","CBS",0,"Cardiometabolic_II","B13805","20212097_SS211647","PASS",-2.1048,-1.1962,"Plate control","PASS","PFC",0.436423290258435,"1h","1h"
4338,"PFC_21",51,"OID30200","P35520","CBS",0,"Cardiometabolic_II","B13805","20212097_SS211647","PASS",-2.1048,-1.5453,"Plate control","PASS","PFC",0.342624447085893,"24h","24h"
4339,"PFC_06",61,"OID30200","P35520","CBS",0,"Cardiometabolic_II","B13805","20212097_SS211647","PASS",-2.1048,-1.7672,"Plate control","PASS","PFC",0.293778352954466,"Baseline","Baseline"
4340,"PFC_14",62,"OID30200","P35520","CBS",0,"Cardiometabolic_II","B13805","20212097_SS211647","PASS",-2.1048,-0.6929,"Plate control","PASS","PFC",0.61860911657406,"1h","1h"
4341,"PFC_22",63,"OID30200","P35520","CBS",0,"Cardiometabolic_II","B13805","20212097_SS211647","PASS",-2.1048,-0.8664,"Plate control","PASS","PFC",0.548513867368596,"24h","24h"
4342,"PFC_07",73,"OID30200","P35520","CBS",0,"Cardiometabolic_II","B13805","20212097_SS211647","PASS",-2.1048,-1.4119,"Plate control","PASS","PFC",0.375816418222671,"Baseline","Baseline"
4343,"PFC_15",74,"OID30200","P35520","CBS",0,"Cardiometabolic_II","B13805","20212097_SS211647","PASS",-2.1048,-1.5571,"Plate control","PASS","PFC",0.339833504196766,"1h","1h"
4344,"PFC_23",75,"OID30200","P35520","CBS",0,"Cardiometabolic_II","B13805","20212097_SS211647","PASS",-2.1048,-0.6855,"Plate control","PASS","PFC",0.621790293207974,"24h","24h"
4345,"PFC_08",85,"OID30200","P35520","CBS",0,"Cardiometabolic_II","B13805","20212097_SS211647","PASS",-2.1048,-1.46,"Plate control","PASS","PFC",0.363493129330078,"Baseline","Baseline"
4346,"PFC_16",86,"OID30200","P35520","CBS",0,"Cardiometabolic_II","B13805","20212097_SS211647","PASS",-2.1048,-1.8846,"Plate control","PASS","PFC",0.270818837898758,"1h","1h"
4347,"PFC_24",87,"OID30200","P35520","CBS",0,"Cardiometabolic_II","B13805","20212097_SS211647","PASS",-2.1048,-1.4899,"Plate control","PASS","PFC",0.356037226663976,"24h","24h"
4348,"PFC_01",1,"OID30201","O14841","OPLAH",0,"Cardiometabolic_II","B13805","20212097_SS211647","PASS",0.1807,2.1574,"Plate control","PASS","PFC",4.46110158345256,"Baseline","Baseline"
4349,"PFC_09",2,"OID30201","O14841","OPLAH",0,"Cardiometabolic_II","B13805","20212097_SS211647","PASS",0.1807,1.9254,"Plate control","PASS","PFC",3.79842148387537,"Baseline","Baseline"
4350,"PFC_17",3,"OID30201","O14841","OPLAH",0,"Cardiometabolic_II","B13805","20212097_SS211647","PASS",0.1807,2.4066,"Plate control","PASS","PFC",5.30223274630639,"1h","1h"
4351,"PFC_25",4,"OID30201","O14841","OPLAH",0,"Cardiometabolic_II","B13805","20212097_SS211647","PASS",0.1807,2.7633,"Plate control","PASS","PFC",6.78947489848129,"24h","24h"
4352,"PFC_02",13,"OID30201","O14841","OPLAH",0,"Cardiometabolic_II","B13805","20212097_SS211647","PASS",0.1807,1.1042,"Plate control","PASS","PFC",2.14979635011021,"Baseline","Baseline"
4353,"PFC_10",14,"OID30201","O14841","OPLAH",0,"Cardiometabolic_II","B13805","20212097_SS211647","PASS",0.1807,2.391,"Plate control","PASS","PFC",5.24520805686259,"1h","1h"
4354,"PFC_18",15,"OID30201","O14841","OPLAH",0,"Cardiometabolic_II","B13805","20212097_SS211647","PASS",0.1807,2.2871,"Plate control","PASS","PFC",4.88074033650466,"1h","1h"
4355,"PFC_26",16,"OID30201","O14841","OPLAH",0,"Cardiometabolic_II","B13805","20212097_SS211647","PASS",0.1807,2.396,"Plate control","PASS","PFC",5.26341810011603,"24h","24h"
4356,"PFC_03",25,"OID30201","O14841","OPLAH",0,"Cardiometabolic_II","B13805","20212097_SS211647","PASS",0.1807,1.332,"Plate control","PASS","PFC",2.51751434700972,"Baseline","Baseline"
4357,"PFC_11",26,"OID30201","O14841","OPLAH",0,"Cardiometabolic_II","B13805","20212097_SS211647","PASS",0.1807,2.033,"Plate control","PASS","PFC",4.09254987893773,"1h","1h"
4358,"PFC_19",27,"OID30201","O14841","OPLAH",0,"Cardiometabolic_II","B13805","20212097_SS211647","PASS",0.1807,3.0064,"Plate control","PASS","PFC",8.03556796959623,"24h","24h"
4359,"PFC_27",28,"OID30201","O14841","OPLAH",0,"Cardiometabolic_II","B13805","20212097_SS211647","PASS",0.1807,3.3032,"Plate control","PASS","PFC",9.87102567835271,"24h","24h"
4360,"PFC_04",37,"OID30201","O14841","OPLAH",0,"Cardiometabolic_II","B13805","20212097_SS211647","PASS",0.1807,2.5143,"Plate control","PASS","PFC",5.71320382256362,"Baseline","Baseline"
4361,"PFC_12",38,"OID30201","O14841","OPLAH",0,"Cardiometabolic_II","B13805","20212097_SS211647","PASS",0.1807,1.8668,"Plate control","PASS","PFC",3.64722701400671,"1h","1h"
4362,"PFC_20",39,"OID30201","O14841","OPLAH",0,"Cardiometabolic_II","B13805","20212097_SS211647","PASS",0.1807,2.444,"Plate control","PASS","PFC",5.44148340962382,"24h","24h"
4363,"PFC_05",49,"OID30201","O14841","OPLAH",0,"Cardiometabolic_II","B13805","20212097_SS211647","PASS",0.1807,1.545,"Plate control","PASS","PFC",2.91804068848035,"Baseline","Baseline"
4364,"PFC_13",50,"OID30201","O14841","OPLAH",0,"Cardiometabolic_II","B13805","20212097_SS211647","PASS",0.1807,2.8338,"Plate control","PASS","PFC",7.12949554071828,"1h","1h"
4365,"PFC_21",51,"OID30201","O14841","OPLAH",0,"Cardiometabolic_II","B13805","20212097_SS211647","PASS",0.1807,2.1523,"Plate control","PASS","PFC",4.44535920497651,"24h","24h"
4366,"PFC_06",61,"OID30201","O14841","OPLAH",0,"Cardiometabolic_II","B13805","20212097_SS211647","PASS",0.1807,3.2954,"Plate control","PASS","PFC",9.81780151328121,"Baseline","Baseline"
4367,"PFC_14",62,"OID30201","O14841","OPLAH",0,"Cardiometabolic_II","B13805","20212097_SS211647","PASS",0.1807,2.5115,"Plate control","PASS","PFC",5.70212632057537,"1h","1h"
4368,"PFC_22",63,"OID30201","O14841","OPLAH",0,"Cardiometabolic_II","B13805","20212097_SS211647","PASS",0.1807,2.3469,"Plate control","PASS","PFC",5.08729939003008,"24h","24h"

Supplemental table 1_Olink proximity extension assay (PEA) data

**,"SampleID","Index","OlinkID","UniProt","Assay","MissingFreq","Panel","Panel_Lot_Nr","PlateID","QC_Warning","LOD","NPX","Normalization","Assay_Warning","condition","Value","group","alt_group"**
4369,"PFC_07",73,"OID30201","O14841","OPLAH",0,"Cardiometabolic_II","B13805","20212097_SS211647","PASS",0.1807,3.0563,"Plate control","PASS","PFC",8.31836505695426,"Baseline","Baseline"
4370,"PFC_15",74,"OID30201","O14841","OPLAH",0,"Cardiometabolic_II","B13805","20212097_SS211647","PASS",0.1807,2.2743,"Plate control","PASS","PFC",4.83762855532742,"1h","1h"
4371,"PFC_23",75,"OID30201","O14841","OPLAH",0,"Cardiometabolic_II","B13805","20212097_SS211647","PASS",0.1807,2.5039,"Plate control","PASS","PFC",5.67216696443757,"24h","24h"
4372,"PFC_08",85,"OID30201","O14841","OPLAH",0,"Cardiometabolic_II","B13805","20212097_SS211647","PASS",0.1807,1.6786,"Plate control","PASS","PFC",3.20117156635455,"Baseline","Baseline"
4373,"PFC_16",86,"OID30201","O14841","OPLAH",0,"Cardiometabolic_II","B13805","20212097_SS211647","PASS",0.1807,3.0375,"Plate control","PASS","PFC",8.21067027112339,"1h","1h"
4374,"PFC_24",87,"OID30201","O14841","OPLAH",0,"Cardiometabolic_II","B13805","20212097_SS211647","PASS",0.1807,3.4521,"Plate control","PASS","PFC",10.9442410019089,"24h","24h"
4375,"PFC_01",1,"OID30202","Q8WXC3","PYDC1",0.0682,"Cardiometabolic_II","B13805","20212097_SS211647","PASS",-0.3814,0.1048,"Plate control","PASS","PFC",1.0753453056105,"Baseline","Baseline"
4376,"PFC_09",2,"OID30202","Q8WXC3","PYDC1",0.0682,"Cardiometabolic_II","B13805","20212097_SS211647","PASS",-0.3814,0.636,"Plate control","PASS","PFC",1.5540145375681,"Baseline","Baseline"
4377,"PFC_17",3,"OID30202","Q8WXC3","PYDC1",0.0682,"Cardiometabolic_II","B13805","20212097_SS211647","PASS",-0.3814,0.0177,"Plate control","PASS","PFC",1.01234427438784,"1h","1h"
4378,"PFC_25",4,"OID30202","Q8WXC3","PYDC1",0.0682,"Cardiometabolic_II","B13805","20212097_SS211647","PASS",-0.3814,0.1344,"Plate control","PASS","PFC",1.09763622477592,"24h","24h"
4379,"PFC_02",13,"OID30202","Q8WXC3","PYDC1",0.0682,"Cardiometabolic_II","B13805","20212097_SS211647","PASS",-0.3814,0.6306,"Plate control","PASS","PFC",1.54820874158396,"Baseline","Baseline"
4380,"PFC_10",14,"OID30202","Q8WXC3","PYDC1",0.0682,"Cardiometabolic_II","B13805","20212097_SS211647","PASS",-0.3814,0.1307,"Plate control","PASS","PFC",1.09482478470758,"1h","1h"
4381,"PFC_18",15,"OID30202","Q8WXC3","PYDC1",0.0682,"Cardiometabolic_II","B13805","20212097_SS211647","PASS",-0.3814,0.9621,"Plate control","PASS","PFC",1.94814356742874,"1h","1h"
4382,"PFC_26",16,"OID30202","Q8WXC3","PYDC1",0.0682,"Cardiometabolic_II","B13805","20212097_SS211647","PASS",-0.3814,-0.0847,"Plate control","PASS","PFC",0.942980602835902,"24h","24h"
4383,"PFC_03",25,"OID30202","Q8WXC3","PYDC1",0.0682,"Cardiometabolic_II","B13805","20212097_SS211647","PASS",-0.3814,0.4475,"Plate control","PASS","PFC",1.3636751391832,"Baseline","Baseline"
4384,"PFC_11",26,"OID30202","Q8WXC3","PYDC1",0.0682,"Cardiometabolic_II","B13805","20212097_SS211647","PASS",-0.3814,0.7341,"Plate control","PASS","PFC",1.66335948804801,"1h","1h"
4385,"PFC_19",27,"OID30202","Q8WXC3","PYDC1",0.0682,"Cardiometabolic_II","B13805","20212097_SS211647","PASS",-0.3814,-0.1897,"Plate control","PASS","PFC",0.87678802530516,"24h","24h"
4386,"PFC_27",28,"OID30202","Q8WXC3","PYDC1",0.0682,"Cardiometabolic_II","B13805","20212097_SS211647","PASS",-0.3814,0.5958,"Plate control","PASS","PFC",1.51131040168451,"24h","24h"
4387,"PFC_04",37,"OID30202","Q8WXC3","PYDC1",0.0682,"Cardiometabolic_II","B13805","20212097_SS211647","PASS",-0.3814,0.617,"Plate control","PASS","PFC",1.53368265967309,"Baseline","Baseline"
4388,"PFC_12",38,"OID30202","Q8WXC3","PYDC1",0.0682,"Cardiometabolic_II","B13805","20212097_SS211647","PASS",-0.3814,0.3171,"Plate control","PASS","PFC",1.24582376649987,"1h","1h"
4389,"PFC_20",39,"OID30202","Q8WXC3","PYDC1",0.0682,"Cardiometabolic_II","B13805","20212097_SS211647","PASS",-0.3814,0.0341,"Plate control","PASS","PFC",1.02391787054729,"24h","24h"
4390,"PFC_05",49,"OID30202","Q8WXC3","PYDC1",0.0682,"Cardiometabolic_II","B13805","20212097_SS211647","PASS",-0.3814,-0.284,"Plate control","PASS","PFC",0.821310700752066,"Baseline","Baseline"
4391,"PFC_13",50,"OID30202","Q8WXC3","PYDC1",0.0682,"Cardiometabolic_II","B13805","20212097_SS211647","PASS",-0.3814,0.7382,"Plate control","PASS","PFC",1.66809331845798,"1h","1h"
4392,"PFC_21",51,"OID30202","Q8WXC3","PYDC1",0.0682,"Cardiometabolic_II","B13805","20212097_SS211647","PASS",-0.3814,0.1441,"Plate control","PASS","PFC",1.10504107775612,"24h","24h"
4393,"PFC_06",61,"OID30202","Q8WXC3","PYDC1",0.0682,"Cardiometabolic_II","B13805","20212097_SS211647","PASS",-0.3814,0.8365,"Plate control","PASS","PFC",1.78571271658185,"Baseline","Baseline"
4394,"PFC_14",62,"OID30202","Q8WXC3","PYDC1",0.0682,"Cardiometabolic_II","B13805","20212097_SS211647","PASS",-0.3814,-0.0466,"Plate control","PASS","PFC",0.96821543600247,"1h","1h"
4395,"PFC_22",63,"OID30202","Q8WXC3","PYDC1",0.0682,"Cardiometabolic_II","B13805","20212097_SS211647","PASS",-0.3814,0.5887,"Plate control","PASS","PFC",1.50389099353112,"24h","24h"
4396,"PFC_07",73,"OID30202","Q8WXC3","PYDC1",0.0682,"Cardiometabolic_II","B13805","20212097_SS211647","PASS",-0.3814,0.0901,"Plate control","PASS","PFC",1.06444396152942,"Baseline","Baseline"
4397,"PFC_15",74,"OID30202","Q8WXC3","PYDC1",0.0682,"Cardiometabolic_II","B13805","20212097_SS211647","PASS",-0.3814,0.5751,"Plate control","PASS","PFC",1.48978072327474,"1h","1h"
4398,"PFC_23",75,"OID30202","Q8WXC3","PYDC1",0.0682,"Cardiometabolic_II","B13805","20212097_SS211647","PASS",-0.3814,-0.3146,"Plate control","PASS","PFC",0.804073896462147,"24h","24h"
4399,"PFC_08",85,"OID30202","Q8WXC3","PYDC1",0.0682,"Cardiometabolic_II","B13805","20212097_SS211647","PASS",-0.3814,-0.3598,"Plate control","PASS","PFC",0.779272602294202,"Baseline","Baseline"
4400,"PFC_16",86,"OID30202","Q8WXC3","PYDC1",0.0682,"Cardiometabolic_II","B13805","20212097_SS211647","PASS",-0.3814,0.072,"Plate control","PASS","PFC",1.05117290908771,"1h","1h"
4401,"PFC_24",87,"OID30202","Q8WXC3","PYDC1",0.0682,"Cardiometabolic_II","B13805","20212097_SS211647","PASS",-0.3814,0.4013,"Plate control","PASS","PFC",1.32069744377585,"24h","24h"
4402,"PFC_01",1,"OID30212","P29536","LMOD1",0,"Cardiometabolic_II","B13805","20212097_SS211647","PASS",-2.7168,0.488,"Plate control","PASS","PFC",1.4024992506424,"Baseline","Baseline"
4403,"PFC_09",2,"OID30212","P29536","LMOD1",0,"Cardiometabolic_II","B13805","20212097_SS211647","PASS",-2.7168,0.2845,"Plate control","PASS","PFC",1.21798806802083,"Baseline","Baseline"
4404,"PFC_17",3,"OID30212","P29536","LMOD1",0,"Cardiometabolic_II","B13805","20212097_SS211647","PASS",-2.7168,0.5677,"Plate control","PASS","PFC",1.48215877150369,"1h","1h"
4405,"PFC_25",4,"OID30212","P29536","LMOD1",0,"Cardiometabolic_II","B13805","20212097_SS211647","PASS",-2.7168,0.3157,"Plate control","PASS","PFC",1.24461539797504,"24h","24h"
4406,"PFC_02",13,"OID30212","P29536","LMOD1",0,"Cardiometabolic_II","B13805","20212097_SS211647","PASS",-2.7168,0.9363,"Plate control","PASS","PFC",1.9136141996997,"Baseline","Baseline"
4407,"PFC_10",14,"OID30212","P29536","LMOD1",0,"Cardiometabolic_II","B13805","20212097_SS211647","PASS",-2.7168,0.5681,"Plate control","PASS","PFC",1.48256977014701,"1h","1h"
4408,"PFC_18",15,"OID30212","P29536","LMOD1",0,"Cardiometabolic_II","B13805","20212097_SS211647","PASS",-2.7168,0.3028,"Plate control","PASS","PFC",1.23353615353444,"1h","1h"
4409,"PFC_26",16,"OID30212","P29536","LMOD1",0,"Cardiometabolic_II","B13805","20212097_SS211647","PASS",-2.7168,0.1853,"Plate control","PASS","PFC",1.13705339226008,"24h","24h"
4410,"PFC_03",25,"OID30212","P29536","LMOD1",0,"Cardiometabolic_II","B13805","20212097_SS211647","PASS",-2.7168,0.3098,"Plate control","PASS","PFC",1.23953585187148,"Baseline","Baseline"
4411,"PFC_11",26,"OID30212","P29536","LMOD1",0,"Cardiometabolic_II","B13805","20212097_SS211647","PASS",-2.7168,1.2842,"Plate control","PASS","PFC",2.43546964170758,"1h","1h"
4412,"PFC_19",27,"OID30212","P29536","LMOD1",0,"Cardiometabolic_II","B13805","20212097_SS211647","PASS",-2.7168,0.3842,"Plate control","PASS","PFC",1.30513586539748,"24h","24h"
4413,"PFC_27",28,"OID30212","P29536","LMOD1",0,"Cardiometabolic_II","B13805","20212097_SS211647","PASS",-2.7168,0.3596,"Plate control","PASS","PFC",1.28307010567147,"24h","24h"
4414,"PFC_04",37,"OID30212","P29536","LMOD1",0,"Cardiometabolic_II","B13805","20212097_SS211647","PASS",-2.7168,1.688,"Plate control","PASS","PFC",3.222097164195,"Baseline","Baseline"
4415,"PFC_12",38,"OID30212","P29536","LMOD1",0,"Cardiometabolic_II","B13805","20212097_SS211647","PASS",-2.7168,1.0659,"Plate control","PASS","PFC",2.09347545025936,"1h","1h"
4416,"PFC_20",39,"OID30212","P29536","LMOD1",0,"Cardiometabolic_II","B13805","20212097_SS211647","PASS",-2.7168,0.6653,"Plate control","PASS","PFC",1.58589801715699,"24h","24h"
4417,"PFC_05",49,"OID30212","P29536","LMOD1",0,"Cardiometabolic_II","B13805","20212097_SS211647","PASS",-2.7168,0.4892,"Plate control","PASS","PFC",1.40366630201972,"Baseline","Baseline"
4418,"PFC_13",50,"OID30212","P29536","LMOD1",0,"Cardiometabolic_II","B13805","20212097_SS211647","PASS",-2.7168,1.1363,"Plate control","PASS","PFC",2.19816548329401,"1h","1h"
4419,"PFC_21",51,"OID30212","P29536","LMOD1",0,"Cardiometabolic_II","B13805","20212097_SS211647","PASS",-2.7168,0.8303,"Plate control","PASS","PFC",1.77805506004909,"24h","24h"
4420,"PFC_06",61,"OID30212","P29536","LMOD1",0,"Cardiometabolic_II","B13805","20212097_SS211647","PASS",-2.7168,0.55,"Plate control","PASS","PFC",1.46408569594563,"Baseline","Baseline"
4421,"PFC_14",62,"OID30212","P29536","LMOD1",0,"Cardiometabolic_II","B13805","20212097_SS211647","PASS",-2.7168,1.0212,"Plate control","PASS","PFC",2.02960643685592,"1h","1h"
4422,"PFC_22",63,"OID30212","P29536","LMOD1",0,"Cardiometabolic_II","B13805","20212097_SS211647","PASS",-2.7168,1.056,"Plate control","PASS","PFC",2.07915887035056,"24h","24h"
4423,"PFC_07",73,"OID30212","P29536","LMOD1",0,"Cardiometabolic_II","B13805","20212097_SS211647","PASS",-2.7168,0.3127,"Plate control","PASS","PFC",1.24202998205534,"Baseline","Baseline"
4424,"PFC_15",74,"OID30212","P29536","LMOD1",0,"Cardiometabolic_II","B13805","20212097_SS211647","PASS",-2.7168,1.1372,"Plate control","PASS","PFC",2.19953719809607,"1h","1h"
4425,"PFC_23",75,"OID30212","P29536","LMOD1",0,"Cardiometabolic_II","B13805","20212097_SS211647","PASS",-2.7168,0.3027,"Plate control","PASS","PFC",1.23345065428696,"24h","24h"
4426,"PFC_08",85,"OID30212","P29536","LMOD1",0,"Cardiometabolic_II","B13805","20212097_SS211647","PASS",-2.7168,0.1925,"Plate control","PASS","PFC",1.14274222250205,"Baseline","Baseline"
4427,"PFC_16",86,"OID30212","P29536","LMOD1",0,"Cardiometabolic_II","B13805","20212097_SS211647","PASS",-2.7168,0.5921,"Plate control","PASS","PFC",1.50743939367375,"1h","1h"
4428,"PFC_24",87,"OID30212","P29536","LMOD1",0,"Cardiometabolic_II","B13805","20212097_SS211647","PASS",-2.7168,0.7213,"Plate control","PASS","PFC",1.64866696492166,"24h","24h"
4429,"PFC_01",1,"OID30213","Q13316","DMP1",0,"Cardiometabolic_II","B13805","20212097_SS211647","PASS",-5.7936,1.0086,"Plate control","PASS","PFC",2.01195773652333,"Baseline","Baseline"
4430,"PFC_09",2,"OID30213","Q13316","DMP1",0,"Cardiometabolic_II","B13805","20212097_SS211647","PASS",-5.7936,1.0305,"Plate control","PASS","PFC",2.04273208574189,"Baseline","Baseline"
4431,"PFC_17",3,"OID30213","Q13316","DMP1",0,"Cardiometabolic_II","B13805","20212097_SS211647","PASS",-5.7936,0.5348,"Plate control","PASS","PFC",1.44874130244336,"1h","1h"
4432,"PFC_25",4,"OID30213","Q13316","DMP1",0,"Cardiometabolic_II","B13805","20212097_SS211647","PASS",-5.7936,1.4631,"Plate control","PASS","PFC",2.75700140010309,"24h","24h"
4433,"PFC_02",13,"OID30213","Q13316","DMP1",0,"Cardiometabolic_II","B13805","20212097_SS211647","PASS",-5.7936,0.5444,"Plate control","PASS","PFC",1.45841368089822,"Baseline","Baseline"
4434,"PFC_10",14,"OID30213","Q13316","DMP1",0,"Cardiometabolic_II","B13805","20212097_SS211647","PASS",-5.7936,1.4108,"Plate control","PASS","PFC",2.65884559654748,"1h","1h"
4435,"PFC_18",15,"OID30213","Q13316","DMP1",0,"Cardiometabolic_II","B13805","20212097_SS211647","PASS",-5.7936,0.7853,"Plate control","PASS","PFC",1.72345066365545,"1h","1h"
4436,"PFC_26",16,"OID30213","Q13316","DMP1",0,"Cardiometabolic_II","B13805","20212097_SS211647","PASS",-5.7936,0.5958,"Plate control","PASS","PFC",1.51131040168451,"24h","24h"
4437,"PFC_03",25,"OID30213","Q13316","DMP1",0,"Cardiometabolic_II","B13805","20212097_SS211647","PASS",-5.7936,0.8344,"Plate control","PASS","PFC",1.78311530780331,"Baseline","Baseline"
4438,"PFC_11",26,"OID30213","Q13316","DMP1",0,"Cardiometabolic_II","B13805","20212097_SS211647","PASS",-5.7936,0.3787,"Plate control","PASS","PFC",1.30016975573623,"1h","1h"
4439,"PFC_19",27,"OID30213","Q13316","DMP1",0,"Cardiometabolic_II","B13805","20212097_SS211647","PASS",-5.7936,1.1583,"Plate control","PASS","PFC",2.23194271583801,"24h","24h"
4440,"PFC_27",28,"OID30213","Q13316","DMP1",0,"Cardiometabolic_II","B13805","20212097_SS211647","PASS",-5.7936,1.0974,"Plate control","PASS","PFC",2.13968734083558,"24h","24h"
4441,"PFC_04",37,"OID30213","Q13316","DMP1",0,"Cardiometabolic_II","B13805","20212097_SS211647","PASS",-5.7936,1.0333,"Plate control","PASS","PFC",2.04670049462467,"Baseline","Baseline"
4442,"PFC_12",38,"OID30213","Q13316","DMP1",0,"Cardiometabolic_II","B13805","20212097_SS211647","PASS",-5.7936,0.8809,"Plate control","PASS","PFC",1.84152374528695,"1h","1h"
4443,"PFC_20",39,"OID30213","Q13316","DMP1",0,"Cardiometabolic_II","B13805","20212097_SS211647","PASS",-5.7936,0.2811,"Plate control","PASS","PFC",1.21512101475658,"24h","24h"
4444,"PFC_05",49,"OID30213","Q13316","DMP1",0,"Cardiometabolic_II","B13805","20212097_SS211647","PASS",-5.7936,0.2919,"Plate control","PASS","PFC",1.22425153080947,"Baseline","Baseline"
4445,"PFC_13",50,"OID30213","Q13316","DMP1",0,"Cardiometabolic_II","B13805","20212097_SS211647","PASS",-5.7936,1.1924,"Plate control","PASS","PFC",2.28532603278438,"1h","1h"
4446,"PFC_21",51,"OID30213","Q13316","DMP1",0,"Cardiometabolic_II","B13805","20212097_SS211647","PASS",-5.7936,0.9782,"Plate control","PASS","PFC",1.97000596767851,"24h","24h"
4447,"PFC_06",61,"OID30213","Q13316","DMP1",0,"Cardiometabolic_II","B13805","20212097_SS211647","PASS",-5.7936,0.3181,"Plate control","PASS","PFC",1.24668760508014,"Baseline","Baseline"
4448,"PFC_14",62,"OID30213","Q13316","DMP1",0,"Cardiometabolic_II","B13805","20212097_SS211647","PASS",-5.7936,0.2021,"Plate control","PASS","PFC",1.15037162827224,"1h","1h"
4449,"PFC_22",63,"OID30213","Q13316","DMP1",0,"Cardiometabolic_II","B13805","20212097_SS211647","PASS",-5.7936,0.8731,"Plate control","PASS","PFC",1.8315943248806,"24h","24h"
4450,"PFC_07",73,"OID30213","Q13316","DMP1",0,"Cardiometabolic_II","B13805","20212097_SS211647","PASS",-5.7936,1.7046,"Plate control","PASS","PFC",3.25938551076954,"Baseline","Baseline"
4451,"PFC_15",74,"OID30213","Q13316","DMP1",0,"Cardiometabolic_II","B13805","20212097_SS211647","PASS",-5.7936,0.3406,"Plate control","PASS","PFC",1.26628311681867,"1h","1h"
4452,"PFC_23",75,"OID30213","Q13316","DMP1",0,"Cardiometabolic_II","B13805","20212097_SS211647","PASS",-5.7936,0.2762,"Plate control","PASS","PFC",1.21100095269371,"24h","24h"
4453,"PFC_08",85,"OID30213","Q13316","DMP1",0,"Cardiometabolic_II","B13805","20212097_SS211647","PASS",-5.7936,0.2849,"Plate control","PASS","PFC",1.21832581283816,"Baseline","Baseline"
4454,"PFC_16",86,"OID30213","Q13316","DMP1",0,"Cardiometabolic_II","B13805","20212097_SS211647","PASS",-5.7936,1.5976,"Plate control","PASS","PFC",3.02639437491389,"1h","1h"
4455,"PFC_24",87,"OID30213","Q13316","DMP1",0,"Cardiometabolic_II","B13805","20212097_SS211647","PASS",-5.7936,0.2821,"Plate control","PASS","PFC",1.21596356443373,"24h","24h"
4456,"PFC_01",1,"OID30214","P35914","HMGCL",0,"Cardiometabolic_II","B13805","20212097_SS211647","PASS",-0.4945,0.7499,"Plate control","PASS","PFC",1.68167626155157,"Baseline","Baseline"
4457,"PFC_09",2,"OID30214","P35914","HMGCL",0,"Cardiometabolic_II","B13805","20212097_SS211647","PASS",-0.4945,1.1337,"Plate control","PASS","PFC",2.19420755508147,"Baseline","Baseline"
4458,"PFC_17",3,"OID30214","P35914","HMGCL",0,"Cardiometabolic_II","B13805","20212097_SS211647","PASS",-0.4945,3.0079,"Plate control","PASS","PFC",8.04392706132734,"1h","1h"
4459,"PFC_25",4,"OID30214","P35914","HMGCL",0,"Cardiometabolic_II","B13805","20212097_SS211647","PASS",-0.4945,2.4935,"Plate control","PASS","PFC",5.63142486627057,"24h","24h"
4460,"PFC_02",13,"OID30214","P35914","HMGCL",0,"Cardiometabolic_II","B13805","20212097_SS211647","PASS",-0.4945,1.0036,"Plate control","PASS","PFC",2.00499689155352,"Baseline","Baseline"
4461,"PFC_10",14,"OID30214","P35914","HMGCL",0,"Cardiometabolic_II","B13805","20212097_SS211647","PASS",-0.4945,1.204,"Plate control","PASS","PFC",2.30377528469872,"1h","1h"
4462,"PFC_18",15,"OID30214","P35914","HMGCL",0,"Cardiometabolic_II","B13805","20212097_SS211647","PASS",-0.4945,1.5015,"Plate control","PASS","PFC",2.8313694284987,"1h","1h"
4463,"PFC_26",16,"OID30214","P35914","HMGCL",0,"Cardiometabolic_II","B13805","20212097_SS211647","PASS",-0.4945,2.4808,"Plate control","PASS","PFC",5.58206916374724,"24h","24h"
4464,"PFC_03",25,"OID30214","P35914","HMGCL",0,"Cardiometabolic_II","B13805","20212097_SS211647","PASS",-0.4945,0.5146,"Plate control","PASS","PFC",1.42859799335758,"Baseline","Baseline"
4465,"PFC_11",26,"OID30214","P35914","HMGCL",0,"Cardiometabolic_II","B13805","20212097_SS211647","PASS",-0.4945,1.9117,"Plate control","PASS","PFC",3.76252195425702,"1h","1h"
4466,"PFC_19",27,"OID30214","P35914","HMGCL",0,"Cardiometabolic_II","B13805","20212097_SS211647","PASS",-0.4945,1.7428,"Plate control","PASS","PFC",3.34684096789595,"24h","24h"
4467,"PFC_27",28,"OID30214","P35914","HMGCL",0,"Cardiometabolic_II","B13805","20212097_SS211647","PASS",-0.4945,2.647,"Plate control","PASS","PFC",6.26363437005234,"24h","24h"
4468,"PFC_04",37,"OID30214","P35914","HMGCL",0,"Cardiometabolic_II","B13805","20212097_SS211647","PASS",-0.4945,1.6496,"Plate control","PASS","PFC",3.13746638059312,"Baseline","Baseline"
4469,"PFC_12",38,"OID30214","P35914","HMGCL",0,"Cardiometabolic_II","B13805","20212097_SS211647","PASS",-0.4945,0.5423,"Plate control","PASS","PFC",1.45629234499557,"1h","1h"
4470,"PFC_20",39,"OID30214","P35914","HMGCL",0,"Cardiometabolic_II","B13805","20212097_SS211647","PASS",-0.4945,2.5697,"Plate control","PASS","PFC",5.93685961889824,"24h","24h"
4471,"PFC_05",49,"OID30214","P35914","HMGCL",0,"Cardiometabolic_II","B13805","20212097_SS211647","PASS",-0.4945,0.2075,"Plate control","PASS","PFC",1.15468553168869,"Baseline","Baseline"
4472,"PFC_13",50,"OID30214","P35914","HMGCL",0,"Cardiometabolic_II","B13805","20212097_SS211647","PASS",-0.4945,1.8769,"Plate control","PASS","PFC",3.67285005781411,"1h","1h"
4473,"PFC_21",51,"OID30214","P35914","HMGCL",0,"Cardiometabolic_II","B13805","20212097_SS211647","PASS",-0.4945,1.4089,"Plate control","PASS","PFC",2.65534625580882,"24h","24h"
4474,"PFC_06",61,"OID30214","P35914","HMGCL",0,"Cardiometabolic_II","B13805","20212097_SS211647","PASS",-0.4945,1.7928,"Plate control","PASS","PFC",3.464867059738,"Baseline","Baseline"
4475,"PFC_14",62,"OID30214","P35914","HMGCL",0,"Cardiometabolic_II","B13805","20212097_SS211647","PASS",-0.4945,1.7395,"Plate control","PASS","PFC",3.33919420062681,"1h","1h"
4476,"PFC_22",63,"OID30214","P35914","HMGCL",0,"Cardiometabolic_II","B13805","20212097_SS211647","PASS",-0.4945,1.4153,"Plate control","PASS","PFC",2.66715191517058,"24h","24h"
4477,"PFC_07",73,"OID30214","P35914","HMGCL",0,"Cardiometabolic_II","B13805","20212097_SS211647","PASS",-0.4945,2.4856,"Plate control","PASS","PFC",5.60067223219334,"Baseline","Baseline"
4478,"PFC_15",74,"OID30214","P35914","HMGCL",0,"Cardiometabolic_II","B13805","20212097_SS211647","PASS",-0.4945,0.6468,"Plate control","PASS","PFC",1.56569152653412,"1h","1h"
4479,"PFC_23",75,"OID30214","P35914","HMGCL",0,"Cardiometabolic_II","B13805","20212097_SS211647","PASS",-0.4945,1.6251,"Plate control","PASS","PFC",3.08463545404274,"24h","24h"
4480,"PFC_08",85,"OID30214","P35914","HMGCL",0,"Cardiometabolic_II","B13805","20212097_SS211647","PASS",-0.4945,1.3408,"Plate control","PASS","PFC",2.53291734620293,"Baseline","Baseline"

Supplemental table 1_Olink proximity extension assay (PEA) data

,"SampleID","Index","OlinkID","UniProt","Assay","MissingFreq","Panel","Panel_Lot_Nr","PlateID","QC_Warning","LOD","NPX","Normalization","Assay_Warning","condition","Value","group","alt_group"
4481,"PFC_16",86,"OID30214","P35914","HMGCL",0,"Cardiometabolic_II","B13805","20212097_SS211647","PASS",-0.4945,2.1683,"Plate control","PASS","PFC",4.4949342102435,"1h","1h"
4482,"PFC_24",87,"OID30214","P35914","HMGCL",0,"Cardiometabolic_II","B13805","20212097_SS211647","PASS",-0.4945,2.4162,"Plate control","PASS","PFC",5.33763258041861,"24h","24h"
4483,"PFC_01",1,"OID30215","Q9Y5X3","SNX5",0.3409,"Cardiometabolic_II","B13805","20212097_SS211647","PASS",0.7934,0.6096,"Plate control","PASS","PFC",1.52583609871181,"Baseline","Baseline"
4484,"PFC_09",2,"OID30215","Q9Y5X3","SNX5",0.3409,"Cardiometabolic_II","B13805","20212097_SS211647","PASS",0.7934,0.9034,"Plate control","PASS","PFC",1.87046892772119,"Baseline","Baseline"
4485,"PFC_17",3,"OID30215","Q9Y5X3","SNX5",0.3409,"Cardiometabolic_II","B13805","20212097_SS211647","PASS",0.7934,1.7079,"Plate control","PASS","PFC",3.26684951583895,"1h","1h"
4486,"PFC_25",4,"OID30215","Q9Y5X3","SNX5",0.3409,"Cardiometabolic_II","B13805","20212097_SS211647","PASS",0.7934,1.107,"Plate control","PASS","PFC",2.1539727523861,"24h","24h"
4487,"PFC_02",13,"OID30215","Q9Y5X3","SNX5",0.3409,"Cardiometabolic_II","B13805","20212097_SS211647","PASS",0.7934,0.4098,"Plate control","PASS","PFC",1.32850163189817,"Baseline","Baseline"
4488,"PFC_10",14,"OID30215","Q9Y5X3","SNX5",0.3409,"Cardiometabolic_II","B13805","20212097_SS211647","PASS",0.7934,0.3695,"Plate control","PASS","PFC",1.29190501288246,"1h","1h"
4489,"PFC_18",15,"OID30215","Q9Y5X3","SNX5",0.3409,"Cardiometabolic_II","B13805","20212097_SS211647","PASS",0.7934,0.8412,"Plate control","PASS","PFC",1.79153968310352,"1h","1h"
4490,"PFC_26",16,"OID30215","Q9Y5X3","SNX5",0.3409,"Cardiometabolic_II","B13805","20212097_SS211647","PASS",0.7934,1.5275,"Plate control","PASS","PFC",2.88285844716339,"24h","24h"
4491,"PFC_03",25,"OID30215","Q9Y5X3","SNX5",0.3409,"Cardiometabolic_II","B13805","20212097_SS211647","PASS",0.7934,0.2595,"Plate control","PASS","PFC",1.19706376203321,"Baseline","Baseline"
4492,"PFC_11",26,"OID30215","Q9Y5X3","SNX5",0.3409,"Cardiometabolic_II","B13805","20212097_SS211647","PASS",0.7934,1.2793,"Plate control","PASS","PFC",2.42721179252695,"1h","1h"
4493,"PFC_19",27,"OID30215","Q9Y5X3","SNX5",0.3409,"Cardiometabolic_II","B13805","20212097_SS211647","PASS",0.7934,0.7841,"Plate control","PASS","PFC",1.72201773371486,"24h","24h"
4494,"PFC_27",28,"OID30215","Q9Y5X3","SNX5",0.3409,"Cardiometabolic_II","B13805","20212097_SS211647","PASS",0.7934,1.8478,"Plate control","PASS","PFC",3.59950868672503,"24h","24h"
4495,"PFC_04",37,"OID30215","Q9Y5X3","SNX5",0.3409,"Cardiometabolic_II","B13805","20212097_SS211647","PASS",0.7934,1.1518,"Plate control","PASS","PFC",2.2219094139096,"Baseline","Baseline"
4496,"PFC_12",38,"OID30215","Q9Y5X3","SNX5",0.3409,"Cardiometabolic_II","B13805","20212097_SS211647","PASS",0.7934,0.6525,"Plate control","PASS","PFC",1.57188971440552,"1h","1h"
4497,"PFC_20",39,"OID30215","Q9Y5X3","SNX5",0.3409,"Cardiometabolic_II","B13805","20212097_SS211647","PASS",0.7934,1.3831,"Plate control","PASS","PFC",2.60828225660071,"24h","24h"
4498,"PFC_05",49,"OID30215","Q9Y5X3","SNX5",0.3409,"Cardiometabolic_II","B13805","20212097_SS211647","PASS",0.7934,0.6672,"Plate control","PASS","PFC",1.58798798848433,"Baseline","Baseline"
4499,"PFC_13",50,"OID30215","Q9Y5X3","SNX5",0.3409,"Cardiometabolic_II","B13805","20212097_SS211647","PASS",0.7934,1.3673,"Plate control","PASS","PFC",2.57987291502897,"1h","1h"
4500,"PFC_21",51,"OID30215","Q9Y5X3","SNX5",0.3409,"Cardiometabolic_II","B13805","20212097_SS211647","PASS",0.7934,0.767,"Plate control","PASS","PFC",1.70172745901308,"24h","24h"
4501,"PFC_06",61,"OID30215","Q9Y5X3","SNX5",0.3409,"Cardiometabolic_II","B13805","20212097_SS211647","PASS",0.7934,1.1464,"Plate control","PASS","PFC",2.21360836366808,"Baseline","Baseline"
4502,"PFC_14",62,"OID30215","Q9Y5X3","SNX5",0.3409,"Cardiometabolic_II","B13805","20212097_SS211647","PASS",0.7934,1.2098,"Plate control","PASS","PFC",2.31305568797754,"1h","1h"
4503,"PFC_22",63,"OID30215","Q9Y5X3","SNX5",0.3409,"Cardiometabolic_II","B13805","20212097_SS211647","PASS",0.7934,1.0198,"Plate control","PASS","PFC",2.02763784980371,"24h","24h"
4504,"PFC_07",73,"OID30215","Q9Y5X3","SNX5",0.3409,"Cardiometabolic_II","B13805","20212097_SS211647","PASS",0.7934,1.3499,"Plate control","PASS","PFC",2.54894456914099,"Baseline","Baseline"
4505,"PFC_15",74,"OID30215","Q9Y5X3","SNX5",0.3409,"Cardiometabolic_II","B13805","20212097_SS211647","PASS",0.7934,0.3378,"Plate control","PASS","PFC",1.26382788256135,"1h","1h"
4506,"PFC_23",75,"OID30215","Q9Y5X3","SNX5",0.3409,"Cardiometabolic_II","B13805","20212097_SS211647","PASS",0.7934,0.846,"Plate control","PASS","PFC",1.79751025322206,"24h","24h"
4507,"PFC_08",85,"OID30215","Q9Y5X3","SNX5",0.3409,"Cardiometabolic_II","B13805","20212097_SS211647","PASS",0.7934,1.1435,"Plate control","PASS","PFC",2.20916319928811,"Baseline","Baseline"
4508,"PFC_16",86,"OID30215","Q9Y5X3","SNX5",0.3409,"Cardiometabolic_II","B13805","20212097_SS211647","PASS",0.7934,1.1894,"Plate control","PASS","PFC",2.2805787684355,"1h","1h"
4509,"PFC_24",87,"OID30215","Q9Y5X3","SNX5",0.3409,"Cardiometabolic_II","B13805","20212097_SS211647","PASS",0.7934,1.1489,"Plate control","PASS","PFC",2.2174475801253,"24h","24h"
4510,"PFC_01",1,"OID30216","Q14643","ITPR1",0.0227,"Cardiometabolic_II","B13805","20212097_SS211647","PASS",-0.2818,1.1866,"Plate control","PASS","PFC",2.27615688596355,"Baseline","Baseline"
4511,"PFC_09",2,"OID30216","Q14643","ITPR1",0.0227,"Cardiometabolic_II","B13805","20212097_SS211647","PASS",-0.2818,1.4743,"Plate control","PASS","PFC",2.77848798179564,"Baseline","Baseline"
4512,"PFC_17",3,"OID30216","Q14643","ITPR1",0.0227,"Cardiometabolic_II","B13805","20212097_SS211647","PASS",-0.2818,1.6756,"Plate control","PASS","PFC",3.19452183347899,"1h","1h"
4513,"PFC_25",4,"OID30216","Q14643","ITPR1",0.0227,"Cardiometabolic_II","B13805","20212097_SS211647","PASS",-0.2818,1.304,"Plate control","PASS","PFC",2.46912521378708,"24h","24h"
4514,"PFC_02",13,"OID30216","Q14643","ITPR1",0.0227,"Cardiometabolic_II","B13805","20212097_SS211647","PASS",-0.2818,0.3021,"Plate control","PASS","PFC",1.23293778323686,"Baseline","Baseline"
4515,"PFC_10",14,"OID30216","Q14643","ITPR1",0.0227,"Cardiometabolic_II","B13805","20212097_SS211647","PASS",-0.2818,0.5225,"Plate control","PASS","PFC",1.43644225731587,"1h","1h"
4516,"PFC_18",15,"OID30216","Q14643","ITPR1",0.0227,"Cardiometabolic_II","B13805","20212097_SS211647","PASS",-0.2818,1.0603,"Plate control","PASS","PFC",2.08536511608417,"1h","1h"
4517,"PFC_26",16,"OID30216","Q14643","ITPR1",0.0227,"Cardiometabolic_II","B13805","20212097_SS211647","PASS",-0.2818,1.775,"Plate control","PASS","PFC",3.42238010273041,"24h","24h"
4518,"PFC_03",25,"OID30216","Q14643","ITPR1",0.0227,"Cardiometabolic_II","B13805","20212097_SS211647","PASS",-0.2818,-0.47,"Plate control","PASS","PFC",0.721964597761248,"Baseline","Baseline"
4519,"PFC_11",26,"OID30216","Q14643","ITPR1",0.0227,"Cardiometabolic_II","B13805","20212097_SS211647","PASS",-0.2818,1.498,"Plate control","PASS","PFC",2.82450880876951,"1h","1h"
4520,"PFC_19",27,"OID30216","Q14643","ITPR1",0.0227,"Cardiometabolic_II","B13805","20212097_SS211647","PASS",-0.2818,1.3981,"Plate control","PASS","PFC",2.63554257902794,"24h","24h"
4521,"PFC_27",28,"OID30216","Q14643","ITPR1",0.0227,"Cardiometabolic_II","B13805","20212097_SS211647","PASS",-0.2818,2.5619,"Plate control","PASS","PFC",5.90484831564983,"24h","24h"
4522,"PFC_04",37,"OID30216","Q14643","ITPR1",0.0227,"Cardiometabolic_II","B13805","20212097_SS211647","PASS",-0.2818,1.334,"Plate control","PASS","PFC",2.52100678316597,"Baseline","Baseline"
4523,"PFC_12",38,"OID30216","Q14643","ITPR1",0.0227,"Cardiometabolic_II","B13805","20212097_SS211647","PASS",-0.2818,0.0918,"Plate control","PASS","PFC",1.06569898857725,"1h","1h"
4524,"PFC_20",39,"OID30216","Q14643","ITPR1",0.0227,"Cardiometabolic_II","B13805","20212097_SS211647","PASS",-0.2818,1.9268,"Plate control","PASS","PFC",3.80210928411679,"24h","24h"
4525,"PFC_05",49,"OID30216","Q14643","ITPR1",0.0227,"Cardiometabolic_II","B13805","20212097_SS211647","PASS",-0.2818,1.953,"Plate control","PASS","PFC",3.87178810757495,"Baseline","Baseline"
4526,"PFC_13",50,"OID30216","Q14643","ITPR1",0.0227,"Cardiometabolic_II","B13805","20212097_SS211647","PASS",-0.2818,1.4164,"Plate control","PASS","PFC",2.6691862923527,"1h","1h"
4527,"PFC_21",51,"OID30216","Q14643","ITPR1",0.0227,"Cardiometabolic_II","B13805","20212097_SS211647","PASS",-0.2818,0.3696,"Plate control","PASS","PFC",1.29199456401775,"24h","24h"
4528,"PFC_06",61,"OID30216","Q14643","ITPR1",0.0227,"Cardiometabolic_II","B13805","20212097_SS211647","PASS",-0.2818,1.8164,"Plate control","PASS","PFC",3.52201242808597,"Baseline","Baseline"
4529,"PFC_14",62,"OID30216","Q14643","ITPR1",0.0227,"Cardiometabolic_II","B13805","20212097_SS211647","PASS",-0.2818,2.2479,"Plate control","PASS","PFC",4.749909414251,"1h","1h"
4530,"PFC_22",63,"OID30216","Q14643","ITPR1",0.0227,"Cardiometabolic_II","B13805","20212097_SS211647","PASS",-0.2818,0.7838,"Plate control","PASS","PFC",1.72165968742187,"24h","24h"
4531,"PFC_07",73,"OID30216","Q14643","ITPR1",0.0227,"Cardiometabolic_II","B13805","20212097_SS211647","PASS",-0.2818,1.397,"Plate control","PASS","PFC",2.63353384411849,"Baseline","Baseline"
4532,"PFC_15",74,"OID30216","Q14643","ITPR1",0.0227,"Cardiometabolic_II","B13805","20212097_SS211647","PASS",-0.2818,0.386,"Plate control","PASS","PFC",1.30676525389317,"1h","1h"
4533,"PFC_23",75,"OID30216","Q14643","ITPR1",0.0227,"Cardiometabolic_II","B13805","20212097_SS211647","PASS",-0.2818,2.2184,"Plate control","PASS","PFC",4.6537702864357,"24h","24h"
4534,"PFC_08",85,"OID30216","Q14643","ITPR1",0.0227,"Cardiometabolic_II","B13805","20212097_SS211647","PASS",-0.2818,0.6505,"Plate control","PASS","PFC",1.56971212229871,"Baseline","Baseline"
4535,"PFC_16",86,"OID30216","Q14643","ITPR1",0.0227,"Cardiometabolic_II","B13805","20212097_SS211647","PASS",-0.2818,0.8325,"Plate control","PASS","PFC",1.78076852691223,"1h","1h"
4536,"PFC_24",87,"OID30216","Q14643","ITPR1",0.0227,"Cardiometabolic_II","B13805","20212097_SS211647","PASS",-0.2818,1.6495,"Plate control","PASS","PFC",3.13724891553239,"24h","24h"
4537,"PFC_01",1,"OID30217","Q99807","COQ7",0,"Cardiometabolic_II","B13805","20212097_SS211647","PASS",-1.3475,-0.0251,"Plate control","PASS","PFC",0.982752476973504,"Baseline","Baseline"
4538,"PFC_09",2,"OID30217","Q99807","COQ7",0,"Cardiometabolic_II","B13805","20212097_SS211647","PASS",-1.3475,-0.39,"Plate control","PASS","PFC",0.76312960448028,"Baseline","Baseline"
4539,"PFC_17",3,"OID30217","Q99807","COQ7",0,"Cardiometabolic_II","B13805","20212097_SS211647","PASS",-1.3475,-0.1209,"Plate control","PASS","PFC",0.919613786712899,"1h","1h"
4540,"PFC_25",4,"OID30217","Q99807","COQ7",0,"Cardiometabolic_II","B13805","20212097_SS211647","PASS",-1.3475,-0.0514,"Plate control","PASS","PFC",0.964999433122854,"24h","24h"
4541,"PFC_02",13,"OID30217","Q99807","COQ7",0,"Cardiometabolic_II","B13805","20212097_SS211647","PASS",-1.3475,-0.1752,"Plate control","PASS","PFC",0.885644734161661,"Baseline","Baseline"
4542,"PFC_10",14,"OID30217","Q99807","COQ7",0,"Cardiometabolic_II","B13805","20212097_SS211647","PASS",-1.3475,-0.5597,"Plate control","PASS","PFC",0.678443227359713,"1h","1h"
4543,"PFC_18",15,"OID30217","Q99807","COQ7",0,"Cardiometabolic_II","B13805","20212097_SS211647","PASS",-1.3475,-0.1152,"Plate control","PASS","PFC",0.923254311632769,"1h","1h"
4544,"PFC_26",16,"OID30217","Q99807","COQ7",0,"Cardiometabolic_II","B13805","20212097_SS211647","PASS",-1.3475,0.2423,"Plate control","PASS","PFC",1.18287694701789,"24h","24h"
4545,"PFC_03",25,"OID30217","Q99807","COQ7",0,"Cardiometabolic_II","B13805","20212097_SS211647","PASS",-1.3475,-0.1537,"Plate control","PASS","PFC",0.898942036918416,"Baseline","Baseline"
4546,"PFC_11",26,"OID30217","Q99807","COQ7",0,"Cardiometabolic_II","B13805","20212097_SS211647","PASS",-1.3475,0.0296,"Plate control","PASS","PFC",1.02072908027693,"1h","1h"
4547,"PFC_19",27,"OID30217","Q99807","COQ7",0,"Cardiometabolic_II","B13805","20212097_SS211647","PASS",-1.3475,0.052,"Plate control","PASS","PFC",1.03670110101972,"24h","24h"
4548,"PFC_27",28,"OID30217","Q99807","COQ7",0,"Cardiometabolic_II","B13805","20212097_SS211647","PASS",-1.3475,0.0745,"Plate control","PASS","PFC",1.05299603209275,"24h","24h"
4549,"PFC_04",37,"OID30217","Q99807","COQ7",0,"Cardiometabolic_II","B13805","20212097_SS211647","PASS",-1.3475,-0.1983,"Plate control","PASS","PFC",0.871576981353746,"Baseline","Baseline"
4550,"PFC_12",38,"OID30217","Q99807","COQ7",0,"Cardiometabolic_II","B13805","20212097_SS211647","PASS",-1.3475,-0.0997,"Plate control","PASS","PFC",0.933227030466975,"1h","1h"
4551,"PFC_20",39,"OID30217","Q99807","COQ7",0,"Cardiometabolic_II","B13805","20212097_SS211647","PASS",-1.3475,0.303,"Plate control","PASS","PFC",1.2337071698095,"24h","24h"
4552,"PFC_05",49,"OID30217","Q99807","COQ7",0,"Cardiometabolic_II","B13805","20212097_SS211647","PASS",-1.3475,-0.0889,"Plate control","PASS","PFC",0.940239372677496,"Baseline","Baseline"
4553,"PFC_13",50,"OID30217","Q99807","COQ7",0,"Cardiometabolic_II","B13805","20212097_SS211647","PASS",-1.3475,-0.3291,"Plate control","PASS","PFC",0.796032920069036,"1h","1h"
4554,"PFC_21",51,"OID30217","Q99807","COQ7",0,"Cardiometabolic_II","B13805","20212097_SS211647","PASS",-1.3475,0.0848,"Plate control","PASS","PFC",1.06054070901648,"24h","24h"
4555,"PFC_06",61,"OID30217","Q99807","COQ7",0,"Cardiometabolic_II","B13805","20212097_SS211647","PASS",-1.3475,-0.1056,"Plate control","PASS","PFC",0.929418328008315,"Baseline","Baseline"
4556,"PFC_14",62,"OID30217","Q99807","COQ7",0,"Cardiometabolic_II","B13805","20212097_SS211647","PASS",-1.3475,-0.1134,"Plate control","PASS","PFC",0.924406942553319,"1h","1h"
4557,"PFC_22",63,"OID30217","Q99807","COQ7",0,"Cardiometabolic_II","B13805","20212097_SS211647","PASS",-1.3475,-0.1463,"Plate control","PASS","PFC",0.903564816192873,"24h","24h"
4558,"PFC_07",73,"OID30217","Q99807","COQ7",0,"Cardiometabolic_II","B13805","20212097_SS211647","PASS",-1.3475,-0.1333,"Plate control","PASS","PFC",0.911743554063987,"Baseline","Baseline"
4559,"PFC_15",74,"OID30217","Q99807","COQ7",0,"Cardiometabolic_II","B13805","20212097_SS211647","PASS",-1.3475,-0.3364,"Plate control","PASS","PFC",0.79201518724247,"1h","1h"
4560,"PFC_23",75,"OID30217","Q99807","COQ7",0,"Cardiometabolic_II","B13805","20212097_SS211647","PASS",-1.3475,-0.0837,"Plate control","PASS","PFC",0.943634453763365,"24h","24h"
4561,"PFC_08",85,"OID30217","Q99807","COQ7",0,"Cardiometabolic_II","B13805","20212097_SS211647","PASS",-1.3475,-0.2024,"Plate control","PASS","PFC",0.869103560008966,"Baseline","Baseline"
4562,"PFC_16",86,"OID30217","Q99807","COQ7",0,"Cardiometabolic_II","B13805","20212097_SS211647","PASS",-1.3475,0.1445,"Plate control","PASS","PFC",1.10534750267665,"1h","1h"
4563,"PFC_24",87,"OID30217","Q99807","COQ7",0,"Cardiometabolic_II","B13805","20212097_SS211647","PASS",-1.3475,0.0414,"Plate control","PASS","PFC",1.02911199877421,"24h","24h"
4564,"PFC_01",1,"OID30218","Q99942","RNF5",0,"Cardiometabolic_II","B13805","20212097_SS211647","PASS",-0.5824,1.4161,"Plate control","PASS","PFC",2.66863130837166,"Baseline","Baseline"
4565,"PFC_09",2,"OID30218","Q99942","RNF5",0,"Cardiometabolic_II","B13805","20212097_SS211647","PASS",-0.5824,1.7203,"Plate control","PASS","PFC",3.29504918405052,"Baseline","Baseline"
4566,"PFC_17",3,"OID30218","Q99942","RNF5",0,"Cardiometabolic_II","B13805","20212097_SS211647","PASS",-0.5824,3.085,"Plate control","PASS","PFC",8.48550193094574,"1h","1h"
4567,"PFC_25",4,"OID30218","Q99942","RNF5",0,"Cardiometabolic_II","B13805","20212097_SS211647","PASS",-0.5824,2.5925,"Plate control","PASS","PFC",6.0314296102626,"24h","24h"
4568,"PFC_02",13,"OID30218","Q99942","RNF5",0,"Cardiometabolic_II","B13805","20212097_SS211647","PASS",-0.5824,1.5297,"Plate control","PASS","PFC",2.88725794020679,"Baseline","Baseline"
4569,"PFC_10",14,"OID30218","Q99942","RNF5",0,"Cardiometabolic_II","B13805","20212097_SS211647","PASS",-0.5824,1.6308,"Plate control","PASS","PFC",3.09684676753261,"1h","1h"
4570,"PFC_18",15,"OID30218","Q99942","RNF5",0,"Cardiometabolic_II","B13805","20212097_SS211647","PASS",-0.5824,1.9003,"Plate control","PASS","PFC",3.7329081218679,"1h","1h"
4571,"PFC_26",16,"OID30218","Q99942","RNF5",0,"Cardiometabolic_II","B13805","20212097_SS211647","PASS",-0.5824,2.9439,"Plate control","PASS","PFC",7.69488624996173,"24h","24h"
4572,"PFC_03",25,"OID30218","Q99942","RNF5",0,"Cardiometabolic_II","B13805","20212097_SS211647","PASS",-0.5824,0.374,"Plate control","PASS","PFC",1.29594096543333,"Baseline","Baseline"
4573,"PFC_11",26,"OID30218","Q99942","RNF5",0,"Cardiometabolic_II","B13805","20212097_SS211647","PASS",-0.5824,2.2243,"Plate control","PASS","PFC",4.67284116743497,"1h","1h"
4574,"PFC_19",27,"OID30218","Q99942","RNF5",0,"Cardiometabolic_II","B13805","20212097_SS211647","PASS",-0.5824,2.2676,"Plate control","PASS","PFC",4.81521427894932,"24h","24h"
4575,"PFC_27",28,"OID30218","Q99942","RNF5",0,"Cardiometabolic_II","B13805","20212097_SS211647","PASS",-0.5824,2.8465,"Plate control","PASS","PFC",7.19253332307583,"24h","24h"
4576,"PFC_04",37,"OID30218","Q99942","RNF5",0,"Cardiometabolic_II","B13805","20212097_SS211647","PASS",-0.5824,2.5403,"Plate control","PASS","PFC",5.81709957537294,"Baseline","Baseline"
4577,"PFC_12",38,"OID30218","Q99942","RNF5",0,"Cardiometabolic_II","B13805","20212097_SS211647","PASS",-0.5824,0.7122,"Plate control","PASS","PFC",1.63830049665189,"1h","1h"
4578,"PFC_20",39,"OID30218","Q99942","RNF5",0,"Cardiometabolic_II","B13805","20212097_SS211647","PASS",-0.5824,2.6769,"Plate control","PASS","PFC",6.39480337346455,"24h","24h"
4579,"PFC_05",49,"OID30218","Q99942","RNF5",0,"Cardiometabolic_II","B13805","20212097_SS211647","PASS",-0.5824,1.1985,"Plate control","PASS","PFC",2.29500930025065,"Baseline","Baseline"
4580,"PFC_13",50,"OID30218","Q99942","RNF5",0,"Cardiometabolic_II","B13805","20212097_SS211647","PASS",-0.5824,2.3928,"Plate control","PASS","PFC",5.25175640320106,"1h","1h"
4581,"PFC_21",51,"OID30218","Q99942","RNF5",0,"Cardiometabolic_II","B13805","20212097_SS211647","PASS",-0.5824,0.8836,"Plate control","PASS","PFC",1.84497337914881,"24h","24h"
4582,"PFC_06",61,"OID30218","Q99942","RNF5",0,"Cardiometabolic_II","B13805","20212097_SS211647","PASS",-0.5824,2.2058,"Plate control","PASS","PFC",4.61330283633772,"Baseline","Baseline"
4583,"PFC_14",62,"OID30218","Q99942","RNF5",0,"Cardiometabolic_II","B13805","20212097_SS211647","PASS",-0.5824,2.1767,"Plate control","PASS","PFC",4.52118201737145,"1h","1h"
4584,"PFC_22",63,"OID30218","Q99942","RNF5",0,"Cardiometabolic_II","B13805","20212097_SS211647","PASS",-0.5824,2.0218,"Plate control","PASS","PFC",4.06090140398273,"24h","24h"
4585,"PFC_07",73,"OID30218","Q99942","RNF5",0,"Cardiometabolic_II","B13805","20212097_SS211647","PASS",-0.5824,2.6128,"Plate control","PASS","PFC",6.11689707075905,"Baseline","Baseline"
4586,"PFC_15",74,"OID30218","Q99942","RNF5",0,"Cardiometabolic_II","B13805","20212097_SS211647","PASS",-0.5824,1.4608,"Plate control","PASS","PFC",2.75260958401571,"1h","1h"
4587,"PFC_23",75,"OID30218","Q99942","RNF5",0,"Cardiometabolic_II","B13805","20212097_SS211647","PASS",-0.5824,2.2832,"Plate control","PASS","PFC",4.8675641754426,"24h","24h"
4588,"PFC_08",85,"OID30218","Q99942","RNF5",0,"Cardiometabolic_II","B13805","20212097_SS211647","PASS",-0.5824,2.35,"Plate control","PASS","PFC",5.09824250927705,"Baseline","Baseline"
4589,"PFC_16",86,"OID30218","Q99942","RNF5",0,"Cardiometabolic_II","B13805","20212097_SS211647","PASS",-0.5824,2.3477,"Plate control","PASS","PFC",5.09012117010443,"1h","1h"
4590,"PFC_24",87,"OID30218","Q99942","RNF5",0,"Cardiometabolic_II","B13805","20212097_SS211647","PASS",-0.5824,2.7565,"Plate control","PASS","PFC",6.7575486815096,"24h","24h"
4591,"PFC_01",1,"OID30219","P36776","LONP1",0,"Cardiometabolic_II","B13805","20212097_SS211647","PASS",-0.4957,2.0089,"Plate control","PASS","PFC",4.02475230975079,"Baseline","Baseline"
4592,"PFC_09",2,"OID30219","P36776","LONP1",0,"Cardiometabolic_II","B13805","20212097_SS211647","PASS",-0.4957,2.2541,"Plate control","PASS","PFC",4.77036613444674,"Baseline","Baseline"

Supplemental table 1_Olink proximity extension assay (PEA) data

,"SampleID","Index","OlinkID","UniProt","Assay","MissingFreq","Panel","Panel_Lot_Nr","PlateID","QC_Warning","LOD","NPX","Normalization","Assay_Warning","condition","Value","group","alt_group"
4593,"PFC_17",3,"OID30219","P36776","LONP1",0,"Cardiometabolic_II","B13805","20212097_SS211647","PASS",-0.4957,5.4515,"Plate control","PASS","PFC",43.7587614655052,"1h","1h"
4594,"PFC_25",4,"OID30219","P36776","LONP1",0,"Cardiometabolic_II","B13805","20212097_SS211647","PASS",-0.4957,3.9084,"Plate control","PASS","PFC",15.0157018034646,"24h","24h"
4595,"PFC_02",13,"OID30219","P36776","LONP1",0,"Cardiometabolic_II","B13805","20212097_SS211647","PASS",-0.4957,1.8686,"Plate control","PASS","PFC",3.65178037116696,"Baseline","Baseline"
4596,"PFC_10",14,"OID30219","P36776","LONP1",0,"Cardiometabolic_II","B13805","20212097_SS211647","PASS",-0.4957,2.054,"Plate control","PASS","PFC",4.15255708217223,"1h","1h"
4597,"PFC_18",15,"OID30219","P36776","LONP1",0,"Cardiometabolic_II","B13805","20212097_SS211647","PASS",-0.4957,2.7045,"Plate control","PASS","PFC",6.5183191904231,"1h","1h"
4598,"PFC_26",16,"OID30219","P36776","LONP1",0,"Cardiometabolic_II","B13805","20212097_SS211647","PASS",-0.4957,4.1731,"Plate control","PASS","PFC",18.039656964728,"24h","24h"
4599,"PFC_03",25,"OID30219","P36776","LONP1",0,"Cardiometabolic_II","B13805","20212097_SS211647","PASS",-0.4957,1.5877,"Plate control","PASS","PFC",3.00569787384021,"Baseline","Baseline"
4600,"PFC_11",26,"OID30219","P36776","LONP1",0,"Cardiometabolic_II","B13805","20212097_SS211647","PASS",-0.4957,2.8055,"Plate control","PASS","PFC",6.99100564545205,"1h","1h"
4601,"PFC_19",27,"OID30219","P36776","LONP1",0,"Cardiometabolic_II","B13805","20212097_SS211647","PASS",-0.4957,3.2982,"Plate control","PASS","PFC",9.83687452388624,"24h","24h"
4602,"PFC_27",28,"OID30219","P36776","LONP1",0,"Cardiometabolic_II","B13805","20212097_SS211647","PASS",-0.4957,3.0389,"Plate control","PASS","PFC",8.21864182244717,"24h","24h"
4603,"PFC_04",37,"OID30219","P36776","LONP1",0,"Cardiometabolic_II","B13805","20212097_SS211647","PASS",-0.4957,2.0697,"Plate control","PASS","PFC",4.19799369547553,"Baseline","Baseline"
4604,"PFC_12",38,"OID30219","P36776","LONP1",0,"Cardiometabolic_II","B13805","20212097_SS211647","PASS",-0.4957,1.9405,"Plate control","PASS","PFC",3.83838653020779,"1h","1h"
4605,"PFC_20",39,"OID30219","P36776","LONP1",0,"Cardiometabolic_II","B13805","20212097_SS211647","PASS",-0.4957,3.4323,"Plate control","PASS","PFC",10.7950648119618,"24h","24h"
4606,"PFC_05",49,"OID30219","P36776","LONP1",0,"Cardiometabolic_II","B13805","20212097_SS211647","PASS",-0.4957,1.7424,"Plate control","PASS","PFC",3.34591315517177,"Baseline","Baseline"
4607,"PFC_13",50,"OID30219","P36776","LONP1",0,"Cardiometabolic_II","B13805","20212097_SS211647","PASS",-0.4957,3.3398,"Plate control","PASS","PFC",10.1246490800766,"1h","1h"
4608,"PFC_21",51,"OID30219","P36776","LONP1",0,"Cardiometabolic_II","B13805","20212097_SS211647","PASS",-0.4957,2.7214,"Plate control","PASS","PFC",6.59512498307252,"24h","24h"
4609,"PFC_06",61,"OID30219","P36776","LONP1",0,"Cardiometabolic_II","B13805","20212097_SS211647","PASS",-0.4957,2.2492,"Plate control","PASS","PFC",4.75419144542577,"Baseline","Baseline"
4610,"PFC_14",62,"OID30219","P36776","LONP1",0,"Cardiometabolic_II","B13805","20212097_SS211647","PASS",-0.4957,3.1027,"Plate control","PASS","PFC",8.59024929509986,"1h","1h"
4611,"PFC_22",63,"OID30219","P36776","LONP1",0,"Cardiometabolic_II","B13805","20212097_SS211647","PASS",-0.4957,2.2371,"Plate control","PASS","PFC",4.71448440307873,"24h","24h"
4612,"PFC_07",73,"OID30219","P36776","LONP1",0,"Cardiometabolic_II","B13805","20212097_SS211647","PASS",-0.4957,3.0566,"Plate control","PASS","PFC",8.32009499219882,"Baseline","Baseline"
4613,"PFC_15",74,"OID30219","P36776","LONP1",0,"Cardiometabolic_II","B13805","20212097_SS211647","PASS",-0.4957,1.469,"Plate control","PASS","PFC",2.76829943210296,"1h","1h"
4614,"PFC_23",75,"OID30219","P36776","LONP1",0,"Cardiometabolic_II","B13805","20212097_SS211647","PASS",-0.4957,3.2523,"Plate control","PASS","PFC",9.52883605467051,"24h","24h"
4615,"PFC_08",85,"OID30219","P36776","LONP1",0,"Cardiometabolic_II","B13805","20212097_SS211647","PASS",-0.4957,1.9277,"Plate control","PASS","PFC",3.8044819032957,"Baseline","Baseline"
4616,"PFC_16",86,"OID30219","P36776","LONP1",0,"Cardiometabolic_II","B13805","20212097_SS211647","PASS",-0.4957,2.3916,"Plate control","PASS","PFC",5.2473899312448,"1h","1h"
4617,"PFC_24",87,"OID30219","P36776","LONP1",0,"Cardiometabolic_II","B13805","20212097_SS211647","PASS",-0.4957,3.6102,"Plate control","PASS","PFC",12.2117664646452,"24h","24h"
4618,"PFC_01",1,"OID30220","Q14457","BECN1",0,"Cardiometabolic_II","B13805","20212097_SS211647","PASS",-0.6929,1.1646,"Plate control","PASS","PFC",2.24171053575113,"Baseline","Baseline"
4619,"PFC_09",2,"OID30220","Q14457","BECN1",0,"Cardiometabolic_II","B13805","20212097_SS211647","PASS",-0.6929,1.9686,"Plate control","PASS","PFC",3.91388129282769,"Baseline","Baseline"
4620,"PFC_17",3,"OID30220","Q14457","BECN1",0,"Cardiometabolic_II","B13805","20212097_SS211647","PASS",-0.6929,3.1625,"Plate control","PASS","PFC",8.95379942102481,"1h","1h"
4621,"PFC_25",4,"OID30220","Q14457","BECN1",0,"Cardiometabolic_II","B13805","20212097_SS211647","PASS",-0.6929,2.5594,"Plate control","PASS","PFC",5.89462485375877,"24h","24h"
4622,"PFC_02",13,"OID30220","Q14457","BECN1",0,"Cardiometabolic_II","B13805","20212097_SS211647","PASS",-0.6929,1.7712,"Plate control","PASS","PFC",3.41337755425602,"Baseline","Baseline"
4623,"PFC_10",14,"OID30220","Q14457","BECN1",0,"Cardiometabolic_II","B13805","20212097_SS211647","PASS",-0.6929,1.1003,"Plate control","PASS","PFC",2.14399270947236,"1h","1h"
4624,"PFC_18",15,"OID30220","Q14457","BECN1",0,"Cardiometabolic_II","B13805","20212097_SS211647","PASS",-0.6929,2.3704,"Plate control","PASS","PFC",5.17084478641442,"1h","1h"
4625,"PFC_26",16,"OID30220","Q14457","BECN1",0,"Cardiometabolic_II","B13805","20212097_SS211647","PASS",-0.6929,2.3262,"Plate control","PASS","PFC",5.01482723624917,"24h","24h"
4626,"PFC_03",25,"OID30220","Q14457","BECN1",0,"Cardiometabolic_II","B13805","20212097_SS211647","PASS",-0.6929,0.4765,"Plate control","PASS","PFC",1.39136409820991,"Baseline","Baseline"
4627,"PFC_11",26,"OID30220","Q14457","BECN1",0,"Cardiometabolic_II","B13805","20212097_SS211647","PASS",-0.6929,2.5169,"Plate control","PASS","PFC",5.72350934290555,"1h","1h"
4628,"PFC_19",27,"OID30220","Q14457","BECN1",0,"Cardiometabolic_II","B13805","20212097_SS211647","PASS",-0.6929,2.0613,"Plate control","PASS","PFC",4.17362216422112,"24h","24h"
4629,"PFC_27",28,"OID30220","Q14457","BECN1",0,"Cardiometabolic_II","B13805","20212097_SS211647","PASS",-0.6929,3.3944,"Plate control","PASS","PFC",10.5151680366999,"24h","24h"
4630,"PFC_04",37,"OID30220","Q14457","BECN1",0,"Cardiometabolic_II","B13805","20212097_SS211647","PASS",-0.6929,1.6146,"Plate control","PASS","PFC",3.06226683582651,"Baseline","Baseline"
4631,"PFC_12",38,"OID30220","Q14457","BECN1",0,"Cardiometabolic_II","B13805","20212097_SS211647","PASS",-0.6929,0.9498,"Plate control","PASS","PFC",1.93160486199512,"1h","1h"
4632,"PFC_20",39,"OID30220","Q14457","BECN1",0,"Cardiometabolic_II","B13805","20212097_SS211647","PASS",-0.6929,2.8583,"Plate control","PASS","PFC",7.25160327787706,"24h","24h"
4633,"PFC_05",49,"OID30220","Q14457","BECN1",0,"Cardiometabolic_II","B13805","20212097_SS211647","PASS",-0.6929,0.4714,"Plate control","PASS","PFC",1.38645423910397,"Baseline","Baseline"
4634,"PFC_13",50,"OID30220","Q14457","BECN1",0,"Cardiometabolic_II","B13805","20212097_SS211647","PASS",-0.6929,2.0066,"Plate control","PASS","PFC",4.01834100653526,"1h","1h"
4635,"PFC_21",51,"OID30220","Q14457","BECN1",0,"Cardiometabolic_II","B13805","20212097_SS211647","PASS",-0.6929,1.3784,"Plate control","PASS","PFC",2.59979884228864,"24h","24h"
4636,"PFC_06",61,"OID30220","Q14457","BECN1",0,"Cardiometabolic_II","B13805","20212097_SS211647","PASS",-0.6929,2.0682,"Plate control","PASS","PFC",4.19363122250436,"Baseline","Baseline"
4637,"PFC_14",62,"OID30220","Q14457","BECN1",0,"Cardiometabolic_II","B13805","20212097_SS211647","PASS",-0.6929,2.4019,"Plate control","PASS","PFC",5.28498728253341,"1h","1h"
4638,"PFC_22",63,"OID30220","Q14457","BECN1",0,"Cardiometabolic_II","B13805","20212097_SS211647","PASS",-0.6929,1.5574,"Plate control","PASS","PFC",2.94322941506357,"24h","24h"
4639,"PFC_07",73,"OID30220","Q14457","BECN1",0,"Cardiometabolic_II","B13805","20212097_SS211647","PASS",-0.6929,2.2199,"Plate control","PASS","PFC",4.65861142434493,"Baseline","Baseline"
4640,"PFC_15",74,"OID30220","Q14457","BECN1",0,"Cardiometabolic_II","B13805","20212097_SS211647","PASS",-0.6929,1.3038,"Plate control","PASS","PFC",2.46878294407588,"1h","1h"
4641,"PFC_23",75,"OID30220","Q14457","BECN1",0,"Cardiometabolic_II","B13805","20212097_SS211647","PASS",-0.6929,1.7973,"Plate control","PASS","PFC",3.47569141517339,"24h","24h"
4642,"PFC_08",85,"OID30220","Q14457","BECN1",0,"Cardiometabolic_II","B13805","20212097_SS211647","PASS",-0.6929,0.9474,"Plate control","PASS","PFC",1.92839420577061,"Baseline","Baseline"
4643,"PFC_16",86,"OID30220","Q14457","BECN1",0,"Cardiometabolic_II","B13805","20212097_SS211647","PASS",-0.6929,2.3305,"Plate control","PASS","PFC",5.02979639064308,"1h","1h"
4644,"PFC_24",87,"OID30220","Q14457","BECN1",0,"Cardiometabolic_II","B13805","20212097_SS211647","PASS",-0.6929,2.8373,"Plate control","PASS","PFC",7.14681280225911,"24h","24h"
4645,"PFC_01",1,"OID30221","I3L3R5","CCER2",0,"Cardiometabolic_II","B13805","20212097_SS211647","PASS",-2.9788,0.7852,"Plate control","PASS","PFC",1.72333120729871,"Baseline","Baseline"
4646,"PFC_09",2,"OID30221","I3L3R5","CCER2",0,"Cardiometabolic_II","B13805","20212097_SS211647","PASS",-2.9788,0.8608,"Plate control","PASS","PFC",1.81604506068901,"Baseline","Baseline"
4647,"PFC_17",3,"OID30221","I3L3R5","CCER2",0,"Cardiometabolic_II","B13805","20212097_SS211647","PASS",-2.9788,0.2949,"Plate control","PASS","PFC",1.22679993901475,"1h","1h"
4648,"PFC_25",4,"OID30221","I3L3R5","CCER2",0,"Cardiometabolic_II","B13805","20212097_SS211647","PASS",-2.9788,0.6072,"Plate control","PASS","PFC",1.52329989927252,"24h","24h"
4649,"PFC_02",13,"OID30221","I3L3R5","CCER2",0,"Cardiometabolic_II","B13805","20212097_SS211647","PASS",-2.9788,0.8211,"Plate control","PASS","PFC",1.76675256067444,"Baseline","Baseline"
4650,"PFC_10",14,"OID30221","I3L3R5","CCER2",0,"Cardiometabolic_II","B13805","20212097_SS211647","PASS",-2.9788,1.035,"Plate control","PASS","PFC",2.0491136460656,"1h","1h"
4651,"PFC_18",15,"OID30221","I3L3R5","CCER2",0,"Cardiometabolic_II","B13805","20212097_SS211647","PASS",-2.9788,0.8056,"Plate control","PASS","PFC",1.7478725604579 8,"1h","1h"
4652,"PFC_26",16,"OID30221","I3L3R5","CCER2",0,"Cardiometabolic_II","B13805","20212097_SS211647","PASS",-2.9788,0.4599,"Plate control","PASS","PFC",1.37544647615078,"24h","24h"
4653,"PFC_03",25,"OID30221","I3L3R5","CCER2",0,"Cardiometabolic_II","B13805","20212097_SS211647","PASS",-2.9788,0.974,"Plate control","PASS","PFC",1.96427919052678,"Baseline","Baseline"
4654,"PFC_11",26,"OID30221","I3L3R5","CCER2",0,"Cardiometabolic_II","B13805","20212097_SS211647","PASS",-2.9788,0.9351,"Plate control","PASS","PFC",1.91202316194079,"1h","1h"
4655,"PFC_19",27,"OID30221","I3L3R5","CCER2",0,"Cardiometabolic_II","B13805","20212097_SS211647","PASS",-2.9788,0.7733,"Plate control","PASS","PFC",1.70917485774913,"24h","24h"
4656,"PFC_27",28,"OID30221","I3L3R5","CCER2",0,"Cardiometabolic_II","B13805","20212097_SS211647","PASS",-2.9788,0.762,"Plate control","PASS","PFC",1.6958399292663,"24h","24h"
4657,"PFC_04",37,"OID30221","I3L3R5","CCER2",0,"Cardiometabolic_II","B13805","20212097_SS211647","PASS",-2.9788,0.3944,"Plate control","PASS","PFC",1.31439600458749,"Baseline","Baseline"
4658,"PFC_12",38,"OID30221","I3L3R5","CCER2",0,"Cardiometabolic_II","B13805","20212097_SS211647","PASS",-2.9788,1.1048,"Plate control","PASS","PFC",2.150690611221,"1h","1h"
4659,"PFC_20",39,"OID30221","I3L3R5","CCER2",0,"Cardiometabolic_II","B13805","20212097_SS211647","PASS",-2.9788,0.6726,"Plate control","PASS","PFC",1.59394295698359,"24h","24h"
4660,"PFC_05",49,"OID30221","I3L3R5","CCER2",0,"Cardiometabolic_II","B13805","20212097_SS211647","PASS",-2.9788,0.6557,"Plate control","PASS","PFC",1.57538014694386,"Baseline","Baseline"
4661,"PFC_13",50,"OID30221","I3L3R5","CCER2",0,"Cardiometabolic_II","B13805","20212097_SS211647","PASS",-2.9788,0.2602,"Plate control","PASS","PFC",1.19764472192368,"1h","1h"
4662,"PFC_21",51,"OID30221","I3L3R5","CCER2",0,"Cardiometabolic_II","B13805","20212097_SS211647","PASS",-2.9788,0.8081,"Plate control","PASS","PFC",1.75090401860088,"24h","24h"
4663,"PFC_06",61,"OID30221","I3L3R5","CCER2",0,"Cardiometabolic_II","B13805","20212097_SS211647","PASS",-2.9788,0.7084,"Plate control","PASS","PFC",1.63399095791159,"Baseline","Baseline"
4664,"PFC_14",62,"OID30221","I3L3R5","CCER2",0,"Cardiometabolic_II","B13805","20212097_SS211647","PASS",-2.9788,0.4183,"Plate control","PASS","PFC",1.33635193607268,"1h","1h"
4665,"PFC_22",63,"OID30221","I3L3R5","CCER2",0,"Cardiometabolic_II","B13805","20212097_SS211647","PASS",-2.9788,0.2318,"Plate control","PASS","PFC",1.1742991674978,"24h","24h"
4666,"PFC_07",73,"OID30221","I3L3R5","CCER2",0,"Cardiometabolic_II","B13805","20212097_SS211647","PASS",-2.9788,0.6325,"Plate control","PASS","PFC",1.55024904420345,"Baseline","Baseline"
4667,"PFC_15",74,"OID30221","I3L3R5","CCER2",0,"Cardiometabolic_II","B13805","20212097_SS211647","PASS",-2.9788,0.7538,"Plate control","PASS","PFC",1.68622844339814,"1h","1h"
4668,"PFC_23",75,"OID30221","I3L3R5","CCER2",0,"Cardiometabolic_II","B13805","20212097_SS211647","PASS",-2.9788,0.5333,"Plate control","PASS","PFC",1.44723579880696,"24h","24h"
4669,"PFC_08",85,"OID30221","I3L3R5","CCER2",0,"Cardiometabolic_II","B13805","20212097_SS211647","PASS",-2.9788,0.2517,"Plate control","PASS","PFC",1.19060924338969,"Baseline","Baseline"
4670,"PFC_16",86,"OID30221","I3L3R5","CCER2",0,"Cardiometabolic_II","B13805","20212097_SS211647","PASS",-2.9788,0.68,"Plate control","PASS","PFC",1.60213975517924,"1h","1h"
4671,"PFC_24",87,"OID30221","I3L3R5","CCER2",0,"Cardiometabolic_II","B13805","20212097_SS211647","PASS",-2.9788,1.0952,"Plate control","PASS","PFC",2.13642696723336,"24h","24h"
4672,"PFC_01",1,"OID30222","Q8N668","COMMD1",0,"Cardiometabolic_II","B13805","20212097_SS211647","PASS",-2.0586,0.5618,"Plate control","PASS","PFC",1.47610975923459,"Baseline","Baseline"
4673,"PFC_09",2,"OID30222","Q8N668","COMMD1",0,"Cardiometabolic_II","B13805","20212097_SS211647","PASS",-2.0586,1.5244,"Plate control","PASS","PFC",2.87667053756474,"Baseline","Baseline"
4674,"PFC_17",3,"OID30222","Q8N668","COMMD1",0,"Cardiometabolic_II","B13805","20212097_SS211647","PASS",-2.0586,2.2344,"Plate control","PASS","PFC",4.70566950893343,"1h","1h"
4675,"PFC_25",4,"OID30222","Q8N668","COMMD1",0,"Cardiometabolic_II","B13805","20212097_SS211647","PASS",-2.0586,2.1112,"Plate control","PASS","PFC",4.3205051470097,"24h","24h"
4676,"PFC_02",13,"OID30222","Q8N668","COMMD1",0,"Cardiometabolic_II","B13805","20212097_SS211647","PASS",-2.0586,1.1224,"Plate control","PASS","PFC",2.17708841674941,"Baseline","Baseline"
4677,"PFC_10",14,"OID30222","Q8N668","COMMD1",0,"Cardiometabolic_II","B13805","20212097_SS211647","PASS",-2.0586,0.8911,"Plate control","PASS","PFC",1.85458963877373,"1h","1h"
4678,"PFC_18",15,"OID30222","Q8N668","COMMD1",0,"Cardiometabolic_II","B13805","20212097_SS211647","PASS",-2.0586,1.6112,"Plate control","PASS","PFC",3.05505848760194,"1h","1h"
4679,"PFC_26",16,"OID30222","Q8N668","COMMD1",0,"Cardiometabolic_II","B13805","20212097_SS211647","PASS",-2.0586,2.0573,"Plate control","PASS","PFC",4.16206645349696,"24h","24h"
4680,"PFC_03",25,"OID30222","Q8N668","COMMD1",0,"Cardiometabolic_II","B13805","20212097_SS211647","PASS",-2.0586,0.3962,"Plate control","PASS","PFC",1.31603695384453,"Baseline","Baseline"
4681,"PFC_11",26,"OID30222","Q8N668","COMMD1",0,"Cardiometabolic_II","B13805","20212097_SS211647","PASS",-2.0586,2.2352,"Plate control","PASS","PFC",4.70827960978238,"1h","1h"
4682,"PFC_19",27,"OID30222","Q8N668","COMMD1",0,"Cardiometabolic_II","B13805","20212097_SS211647","PASS",-2.0586,1.4039,"Plate control","PASS","PFC",2.64615945566272,"24h","24h"
4683,"PFC_27",28,"OID30222","Q8N668","COMMD1",0,"Cardiometabolic_II","B13805","20212097_SS211647","PASS",-2.0586,2.5986,"Plate control","PASS","PFC",6.05698567766056,"24h","24h"
4684,"PFC_04",37,"OID30222","Q8N668","COMMD1",0,"Cardiometabolic_II","B13805","20212097_SS211647","PASS",-2.0586,2.011,"Plate control","PASS","PFC",4.03061504164202,"Baseline","Baseline"
4685,"PFC_12",38,"OID30222","Q8N668","COMMD1",0,"Cardiometabolic_II","B13805","20212097_SS211647","PASS",-2.0586,0.6389,"Plate control","PASS","PFC",1.5571414455623,"1h","1h"
4686,"PFC_20",39,"OID30222","Q8N668","COMMD1",0,"Cardiometabolic_II","B13805","20212097_SS211647","PASS",-2.0586,2.4168,"Plate control","PASS","PFC",5.33985290107369,"24h","24h"
4687,"PFC_05",49,"OID30222","Q8N668","COMMD1",0,"Cardiometabolic_II","B13805","20212097_SS211647","PASS",-2.0586,0.6536,"Plate control","PASS","PFC",1.57308867744531,"Baseline","Baseline"
4688,"PFC_13",50,"OID30222","Q8N668","COMMD1",0,"Cardiometabolic_II","B13805","20212097_SS211647","PASS",-2.0586,2.4487,"Plate control","PASS","PFC",5.45923953655379,"1h","1h"
4689,"PFC_21",51,"OID30222","Q8N668","COMMD1",0,"Cardiometabolic_II","B13805","20212097_SS211647","PASS",-2.0586,1.2282,"Plate control","PASS","PFC",2.3427451133611,"24h","24h"
4690,"PFC_06",61,"OID30222","Q8N668","COMMD1",0,"Cardiometabolic_II","B13805","20212097_SS211647","PASS",-2.0586,1.5301,"Plate control","PASS","PFC",2.88805856907271,"Baseline","Baseline"
4691,"PFC_14",62,"OID30222","Q8N668","COMMD1",0,"Cardiometabolic_II","B13805","20212097_SS211647","PASS",-2.0586,1.8194,"Plate control","PASS","PFC",3.5293438670468,"1h","1h"
4692,"PFC_22",63,"OID30222","Q8N668","COMMD1",0,"Cardiometabolic_II","B13805","20212097_SS211647","PASS",-2.0586,1.8655,"Plate control","PASS","PFC",3.64394200961537,"24h","24h"
4693,"PFC_07",73,"OID30222","Q8N668","COMMD1",0,"Cardiometabolic_II","B13805","20212097_SS211647","PASS",-2.0586,2.3097,"Plate control","PASS","PFC",4.95779974708403,"Baseline","Baseline"
4694,"PFC_15",74,"OID30222","Q8N668","COMMD1",0,"Cardiometabolic_II","B13805","20212097_SS211647","PASS",-2.0586,0.68,"Plate control","PASS","PFC",1.60213975517924,"1h","1h"
4695,"PFC_23",75,"OID30222","Q8N668","COMMD1",0,"Cardiometabolic_II","B13805","20212097_SS211647","PASS",-2.0586,1.891,"Plate control","PASS","PFC",3.70892218574185,"24h","24h"
4696,"PFC_08",85,"OID30222","Q8N668","COMMD1",0,"Cardiometabolic_II","B13805","20212097_SS211647","PASS",-2.0586,1.3072,"Plate control","PASS","PFC",2.47460798710664,"Baseline","Baseline"
4697,"PFC_16",86,"OID30222","Q8N668","COMMD1",0,"Cardiometabolic_II","B13805","20212097_SS211647","PASS",-2.0586,2.2618,"Plate control","PASS","PFC",4.79589475689248,"1h","1h"
4698,"PFC_24",87,"OID30222","Q8N668","COMMD1",0,"Cardiometabolic_II","B13805","20212097_SS211647","PASS",-2.0586,1.9373,"Plate control","PASS","PFC",3.82988215158798,"24h","24h"
4699,"PFC_01",1,"OID30223","P11532","DMD",0,"Cardiometabolic_II","B13805","20212097_SS211647","PASS",-1.0092,0.3591,"Plate control","PASS","PFC",1.28262550450633,"Baseline","Baseline"
4700,"PFC_09",2,"OID30223","P11532","DMD",0,"Cardiometabolic_II","B13805","20212097_SS211647","PASS",-1.0092,1.4031,"Plate control","PASS","PFC",2.64469252004842,"Baseline","Baseline"
4701,"PFC_17",3,"OID30223","P11532","DMD",0,"Cardiometabolic_II","B13805","20212097_SS211647","PASS",-1.0092,1.8276,"Plate control","PASS","PFC",3.54946109305777,"1h","1h"
4702,"PFC_25",4,"OID30223","P11532","DMD",0,"Cardiometabolic_II","B13805","20212097_SS211647","PASS",-1.0092,2.0155,"Plate control","PASS","PFC",4.04320681185477,"24h","24h"
4703,"PFC_02",13,"OID30223","P11532","DMD",0,"Cardiometabolic_II","B13805","20212097_SS211647","PASS",-1.0092,0.6525,"Plate control","PASS","PFC",1.57188971440552,"Baseline","Baseline"
4704,"PFC_10",14,"OID30223","P11532","DMD",0,"Cardiometabolic_II","B13805","20212097_SS211647","PASS",-1.0092,0.8363,"Plate control","PASS","PFC",1.78546518139312,"1h","1h"

Supplemental table 1_Olink proximity extension assay (PEA) data

,"SampleID","Index","OlinkID","UniProt","Assay","MissingFreq","Panel","Panel_Lot_Nr","PlateID","QC_Warning","LOD","NPX","Normalization","Assay_Warning","condition","Value","group","alt_group"
4705,"PFC_18",15,"OID30223","P11532","DMD",0,"Cardiometabolic_II","B13805","20212097_SS211647","PASS",-1.0092,1.0105,"Plate control","PASS","PFC",2.01460918947659,"1h","1h"
4706,"PFC_26",16,"OID30223","P11532","DMD",0,"Cardiometabolic_II","B13805","20212097_SS211647","PASS",-1.0092,1.8035,"Plate control","PASS","PFC",3.49066038417175,"24h","24h"
4707,"PFC_03",25,"OID30223","P11532","DMD",0,"Cardiometabolic_II","B13805","20212097_SS211647","PASS",-1.0092,0.7988,"Plate control","PASS","PFC",1.73965352151338,"Baseline","Baseline"
4708,"PFC_11",26,"OID30223","P11532","DMD",0,"Cardiometabolic_II","B13805","20212097_SS211647","PASS",-1.0092,2.0855,"Plate control","PASS","PFC",4.24422164574239,"1h","1h"
4709,"PFC_19",27,"OID30223","P11532","DMD",0,"Cardiometabolic_II","B13805","20212097_SS211647","PASS",-1.0092,1.0052,"Plate control","PASS","PFC",2.00722173775004,"24h","24h"
4710,"PFC_27",28,"OID30223","P11532","DMD",0,"Cardiometabolic_II","B13805","20212097_SS211647","PASS",-1.0092,1.7919,"Plate control","PASS","PFC",3.46270623725356,"24h","24h"
4711,"PFC_04",37,"OID30223","P11532","DMD",0,"Cardiometabolic_II","B13805","20212097_SS211647","PASS",-1.0092,1.2899,"Plate control","PASS","PFC",2.44511106732595,"Baseline","Baseline"
4712,"PFC_12",38,"OID30223","P11532","DMD",0,"Cardiometabolic_II","B13805","20212097_SS211647","PASS",-1.0092,1.0382,"Plate control","PASS","PFC",2.05366376995752,"1h","1h"
4713,"PFC_20",39,"OID30223","P11532","DMD",0,"Cardiometabolic_II","B13805","20212097_SS211647","PASS",-1.0092,1.9965,"Plate control","PASS","PFC",3.99030770105782,"24h","24h"
4714,"PFC_05",49,"OID30223","P11532","DMD",0,"Cardiometabolic_II","B13805","20212097_SS211647","PASS",-1.0092,0.6569,"Plate control","PASS","PFC",1.5766910564288,"Baseline","Baseline"
4715,"PFC_13",50,"OID30223","P11532","DMD",0,"Cardiometabolic_II","B13805","20212097_SS211647","PASS",-1.0092,1.6804,"Plate control","PASS","PFC",3.20516804845366,"1h","1h"
4716,"PFC_21",51,"OID30223","P11532","DMD",0,"Cardiometabolic_II","B13805","20212097_SS211647","PASS",-1.0092,1.2329,"Plate control","PASS","PFC",2.35038973459138,"24h","24h"
4717,"PFC_06",61,"OID30223","P11532","DMD",0,"Cardiometabolic_II","B13805","20212097_SS211647","PASS",-1.0092,0.3815,"Plate control","PASS","PFC",1.30269559123066,"Baseline","Baseline"
4718,"PFC_14",62,"OID30223","P11532","DMD",0,"Cardiometabolic_II","B13805","20212097_SS211647","PASS",-1.0092,1.5387,"Plate control","PASS","PFC",2.90532589078918,"1h","1h"
4719,"PFC_22",63,"OID30223","P11532","DMD",0,"Cardiometabolic_II","B13805","20212097_SS211647","PASS",-1.0092,1.066,"Plate control","PASS","PFC",2.09362056394915,"24h","24h"
4720,"PFC_07",73,"OID30223","P11532","DMD",0,"Cardiometabolic_II","B13805","20212097_SS211647","PASS",-1.0092,1.7621,"Plate control","PASS","PFC",3.39191496001362,"Baseline","Baseline"
4721,"PFC_15",74,"OID30223","P11532","DMD",0,"Cardiometabolic_II","B13805","20212097_SS211647","PASS",-1.0092,0.6336,"Plate control","PASS","PFC",1.55143150076477,"1h","1h"
4722,"PFC_23",75,"OID30223","P11532","DMD",0,"Cardiometabolic_II","B13805","20212097_SS211647","PASS",-1.0092,1.5328,"Plate control","PASS","PFC",2.8934686240125,"24h","24h"
4723,"PFC_08",85,"OID30223","P11532","DMD",0,"Cardiometabolic_II","B13805","20212097_SS211647","PASS",-1.0092,0.8961,"Plate control","PASS","PFC",1.86102830758789,"Baseline","Baseline"
4724,"PFC_16",86,"OID30223","P11532","DMD",0,"Cardiometabolic_II","B13805","20212097_SS211647","PASS",-1.0092,1.9872,"Plate control","PASS","PFC",3.96466783462763,"1h","1h"
4725,"PFC_24",87,"OID30223","P11532","DMD",0,"Cardiometabolic_II","B13805","20212097_SS211647","PASS",-1.0092,1.9525,"Plate control","PASS","PFC",3.87044648056938,"24h","24h"
4726,"PFC_01",1,"OID30224","P05305","EDN1",0,"Cardiometabolic_II","B13805","20212097_SS211647","PASS",-2.1414,1.4354,"Plate control","PASS","PFC",2.70457142853642,"Baseline","Baseline"
4727,"PFC_09",2,"OID30224","P05305","EDN1",0,"Cardiometabolic_II","B13805","20212097_SS211647","PASS",-2.1414,1.5417,"Plate control","PASS","PFC",2.91137363192143,"Baseline","Baseline"
4728,"PFC_17",3,"OID30224","P05305","EDN1",0,"Cardiometabolic_II","B13805","20212097_SS211647","PASS",-2.1414,1.6026,"Plate control","PASS","PFC",3.03690125507416,"1h","1h"
4729,"PFC_25",4,"OID30224","P05305","EDN1",0,"Cardiometabolic_II","B13805","20212097_SS211647","PASS",-2.1414,1.169,"Plate control","PASS","PFC",2.24855784755724,"24h","24h"
4730,"PFC_02",13,"OID30224","P05305","EDN1",0,"Cardiometabolic_II","B13805","20212097_SS211647","PASS",-2.1414,1.4566,"Plate control","PASS","PFC",2.74460779014707,"Baseline","Baseline"
4731,"PFC_10",14,"OID30224","P05305","EDN1",0,"Cardiometabolic_II","B13805","20212097_SS211647","PASS",-2.1414,1.717,"Plate control","PASS","PFC",3.2875207491794,"1h","1h"
4732,"PFC_18",15,"OID30224","P05305","EDN1",0,"Cardiometabolic_II","B13805","20212097_SS211647","PASS",-2.1414,1.347,"Plate control","PASS","PFC",2.54382601348715,"1h","1h"
4733,"PFC_26",16,"OID30224","P05305","EDN1",0,"Cardiometabolic_II","B13805","20212097_SS211647","PASS",-2.1414,1.4602,"Plate control","PASS","PFC",2.75146504388923,"24h","24h"
4734,"PFC_03",25,"OID30224","P05305","EDN1",0,"Cardiometabolic_II","B13805","20212097_SS211647","PASS",-2.1414,1.1431,"Plate control","PASS","PFC",2.208550774095,"Baseline","Baseline"
4735,"PFC_11",26,"OID30224","P05305","EDN1",0,"Cardiometabolic_II","B13805","20212097_SS211647","PASS",-2.1414,1.542,"Plate control","PASS","PFC",2.91197909799817,"1h","1h"
4736,"PFC_19",27,"OID30224","P05305","EDN1",0,"Cardiometabolic_II","B13805","20212097_SS211647","PASS",-2.1414,1.2465,"Plate control","PASS","PFC",2.37265115457405,"24h","24h"
4737,"PFC_27",28,"OID30224","P05305","EDN1",0,"Cardiometabolic_II","B13805","20212097_SS211647","PASS",-2.1414,1.5616,"Plate control","PASS","PFC",2.9518102823088,"24h","24h"
4738,"PFC_04",37,"OID30224","P05305","EDN1",0,"Cardiometabolic_II","B13805","20212097_SS211647","PASS",-2.1414,1.4463,"Plate control","PASS","PFC",2.72508267537969,"Baseline","Baseline"
4739,"PFC_12",38,"OID30224","P05305","EDN1",0,"Cardiometabolic_II","B13805","20212097_SS211647","PASS",-2.1414,1.0353,"Plate control","PASS","PFC",2.04953979157523,"1h","1h"
4740,"PFC_20",39,"OID30224","P05305","EDN1",0,"Cardiometabolic_II","B13805","20212097_SS211647","PASS",-2.1414,1.4093,"Plate control","PASS","PFC",2.65608257618803,"24h","24h"
4741,"PFC_05",49,"OID30224","P05305","EDN1",0,"Cardiometabolic_II","B13805","20212097_SS211647","PASS",-2.1414,0.969,"Plate control","PASS","PFC",1.95748330079487,"Baseline","Baseline"
4742,"PFC_13",50,"OID30224","P05305","EDN1",0,"Cardiometabolic_II","B13805","20212097_SS211647","PASS",-2.1414,1.9058,"Plate control","PASS","PFC",3.74716628393233,"1h","1h"
4743,"PFC_21",51,"OID30224","P05305","EDN1",0,"Cardiometabolic_II","B13805","20212097_SS211647","PASS",-2.1414,1.2085,"Plate control","PASS","PFC",2.31097235231918,"24h","24h"
4744,"PFC_06",61,"OID30224","P05305","EDN1",0,"Cardiometabolic_II","B13805","20212097_SS211647","PASS",-2.1414,1.3311,"Plate control","PASS","PFC",2.51594432960006,"Baseline","Baseline"
4745,"PFC_14",62,"OID30224","P05305","EDN1",0,"Cardiometabolic_II","B13805","20212097_SS211647","PASS",-2.1414,0.9059,"Plate control","PASS","PFC",1.87371301335435,"1h","1h"
4746,"PFC_22",63,"OID30224","P05305","EDN1",0,"Cardiometabolic_II","B13805","20212097_SS211647","PASS",-2.1414,1.1037,"Plate control","PASS","PFC",2.14905141656538,"24h","24h"
4747,"PFC_07",73,"OID30224","P05305","EDN1",0,"Cardiometabolic_II","B13805","20212097_SS211647","PASS",-2.1414,1.3746,"Plate control","PASS","PFC",2.59296008843933,"Baseline","Baseline"
4748,"PFC_15",74,"OID30224","P05305","EDN1",0,"Cardiometabolic_II","B13805","20212097_SS211647","PASS",-2.1414,1.5858,"Plate control","PASS","PFC",3.00174203637763,"1h","1h"
4749,"PFC_23",75,"OID30224","P05305","EDN1",0,"Cardiometabolic_II","B13805","20212097_SS211647","PASS",-2.1414,0.9563,"Plate control","PASS","PFC",1.94032725844632,"24h","24h"
4750,"PFC_08",85,"OID30224","P05305","EDN1",0,"Cardiometabolic_II","B13805","20212097_SS211647","PASS",-2.1414,1.104,"Plate control","PASS","PFC",2.149498345711,"Baseline","Baseline"
4751,"PFC_16",86,"OID30224","P05305","EDN1",0,"Cardiometabolic_II","B13805","20212097_SS211647","PASS",-2.1414,1.3555,"Plate control","PASS","PFC",2.55885784144536,"1h","1h"
4752,"PFC_24",87,"OID30224","P05305","EDN1",0,"Cardiometabolic_II","B13805","20212097_SS211647","PASS",-2.1414,1.7,"Plate control","PASS","PFC",3.24900958542494,"24h","24h"
4753,"PFC_01",1,"OID30225","Q14160","SCRIB",0,"Cardiometabolic_II","B13805","20212097_SS211647","PASS",-2.9417,0.6606,"Plate control","PASS","PFC",1.5807398982831,"Baseline","Baseline"
4754,"PFC_09",2,"OID30225","Q14160","SCRIB",0,"Cardiometabolic_II","B13805","20212097_SS211647","PASS",-2.9417,1.2565,"Plate control","PASS","PFC",2.38915424844683,"Baseline","Baseline"
4755,"PFC_17",3,"OID30225","Q14160","SCRIB",0,"Cardiometabolic_II","B13805","20212097_SS211647","PASS",-2.9417,2.4449,"Plate control","PASS","PFC",5.44487904266186,"1h","1h"
4756,"PFC_25",4,"OID30225","Q14160","SCRIB",0,"Cardiometabolic_II","B13805","20212097_SS211647","PASS",-2.9417,1.1387,"Plate control","PASS","PFC",2.20182529189037,"24h","24h"
4757,"PFC_02",13,"OID30225","Q14160","SCRIB",0,"Cardiometabolic_II","B13805","20212097_SS211647","PASS",-2.9417,0.9982,"Plate control","PASS","PFC",1.99750622617055,"Baseline","Baseline"
4758,"PFC_10",14,"OID30225","Q14160","SCRIB",0,"Cardiometabolic_II","B13805","20212097_SS211647","PASS",-2.9417,0.8567,"Plate control","PASS","PFC",1.81089136260807,"1h","1h"
4759,"PFC_18",15,"OID30225","Q14160","SCRIB",0,"Cardiometabolic_II","B13805","20212097_SS211647","PASS",-2.9417,1.0398,"Plate control","PASS","PFC",2.05594261938954,"1h","1h"
4760,"PFC_26",16,"OID30225","Q14160","SCRIB",0,"Cardiometabolic_II","B13805","20212097_SS211647","PASS",-2.9417,2.3971,"Plate control","PASS","PFC",5.26743278620195,"24h","24h"
4761,"PFC_03",25,"OID30225","Q14160","SCRIB",0,"Cardiometabolic_II","B13805","20212097_SS211647","PASS",-2.9417,-0.0854,"Plate control","PASS","PFC",0.942523176774806,"Baseline","Baseline"
4762,"PFC_11",26,"OID30225","Q14160","SCRIB",0,"Cardiometabolic_II","B13805","20212097_SS211647","PASS",-2.9417,1.7818,"Plate control","PASS","PFC",3.43854922778878,"1h","1h"
4763,"PFC_19",27,"OID30225","Q14160","SCRIB",0,"Cardiometabolic_II","B13805","20212097_SS211647","PASS",-2.9417,1.0824,"Plate control","PASS","PFC",2.11755581950529,"24h","24h"
4764,"PFC_27",28,"OID30225","Q14160","SCRIB",0,"Cardiometabolic_II","B13805","20212097_SS211647","PASS",-2.9417,1.677,"Plate control","PASS","PFC",3.19762332140989,"24h","24h"
4765,"PFC_04",37,"OID30225","Q14160","SCRIB",0,"Cardiometabolic_II","B13805","20212097_SS211647","PASS",-2.9417,1.4183,"Plate control","PASS","PFC",2.67270387214535,"Baseline","Baseline"
4766,"PFC_12",38,"OID30225","Q14160","SCRIB",0,"Cardiometabolic_II","B13805","20212097_SS211647","PASS",-2.9417,-0.0779,"Plate control","PASS","PFC",0.947435739564493,"1h","1h"
4767,"PFC_20",39,"OID30225","Q14160","SCRIB",0,"Cardiometabolic_II","B13805","20212097_SS211647","PASS",-2.9417,2.0304,"Plate control","PASS","PFC",4.08518099850076,"24h","24h"
4768,"PFC_05",49,"OID30225","Q14160","SCRIB",0,"Cardiometabolic_II","B13805","20212097_SS211647","PASS",-2.9417,0.7055,"Plate control","PASS","PFC",1.63070972780671,"Baseline","Baseline"
4769,"PFC_13",50,"OID30225","Q14160","SCRIB",0,"Cardiometabolic_II","B13805","20212097_SS211647","PASS",-2.9417,1.6505,"Plate control","PASS","PFC",3.13942424459741,"1h","1h"
4770,"PFC_21",51,"OID30225","Q14160","SCRIB",0,"Cardiometabolic_II","B13805","20212097_SS211647","PASS",-2.9417,0.4992,"Plate control","PASS","PFC",1.41342957324635,"24h","24h"
4771,"PFC_06",61,"OID30225","Q14160","SCRIB",0,"Cardiometabolic_II","B13805","20212097_SS211647","PASS",-2.9417,0.9342,"Plate control","PASS","PFC",1.91083075179406,"Baseline","Baseline"
4772,"PFC_14",62,"OID30225","Q14160","SCRIB",0,"Cardiometabolic_II","B13805","20212097_SS211647","PASS",-2.9417,1.5665,"Plate control","PASS","PFC",2.96185291814147,"1h","1h"
4773,"PFC_22",63,"OID30225","Q14160","SCRIB",0,"Cardiometabolic_II","B13805","20212097_SS211647","PASS",-2.9417,1.3317,"Plate control","PASS","PFC",2.51699089904408,"24h","24h"
4774,"PFC_07",73,"OID30225","Q14160","SCRIB",0,"Cardiometabolic_II","B13805","20212097_SS211647","PASS",-2.9417,0.9822,"Plate control","PASS","PFC",1.97547556297251,"Baseline","Baseline"
4775,"PFC_15",74,"OID30225","Q14160","SCRIB",0,"Cardiometabolic_II","B13805","20212097_SS211647","PASS",-2.9417,0.5897,"Plate control","PASS","PFC",1.50493377269112,"1h","1h"
4776,"PFC_23",75,"OID30225","Q14160","SCRIB",0,"Cardiometabolic_II","B13805","20212097_SS211647","PASS",-2.9417,1.1562,"Plate control","PASS","PFC",2.22869624312743,"24h","24h"
4777,"PFC_08",85,"OID30225","Q14160","SCRIB",0,"Cardiometabolic_II","B13805","20212097_SS211647","PASS",-2.9417,2.1526,"Plate control","PASS","PFC",4.4462836875534,"Baseline","Baseline"
4778,"PFC_16",86,"OID30225","Q14160","SCRIB",0,"Cardiometabolic_II","B13805","20212097_SS211647","PASS",-2.9417,1.6483,"Plate control","PASS","PFC",3.13464051019944,"1h","1h"
4779,"PFC_24",87,"OID30225","Q14160","SCRIB",0,"Cardiometabolic_II","B13805","20212097_SS211647","PASS",-2.9417,1.5902,"Plate control","PASS","PFC",3.01091086676709,"24h","24h"
4780,"PFC_01",1,"OID30226","Q8WZ75","ROBO4",0,"Cardiometabolic_II","B13805","20212097_SS211647","PASS",-1.8816,0.2552,"Plate control","PASS","PFC",1.19350118596024,"Baseline","Baseline"
4781,"PFC_09",2,"OID30226","Q8WZ75","ROBO4",0,"Cardiometabolic_II","B13805","20212097_SS211647","PASS",-1.8816,0.6905,"Plate control","PASS","PFC",1.61384273680494,"Baseline","Baseline"
4782,"PFC_17",3,"OID30226","Q8WZ75","ROBO4",0,"Cardiometabolic_II","B13805","20212097_SS211647","PASS",-1.8816,0.1222,"Plate control","PASS","PFC",1.08839331456432,"1h","1h"
4783,"PFC_25",4,"OID30226","Q8WZ75","ROBO4",0,"Cardiometabolic_II","B13805","20212097_SS211647","PASS",-1.8816,0.4202,"Plate control","PASS","PFC",1.33811304378541,"24h","24h"
4784,"PFC_02",13,"OID30226","Q8WZ75","ROBO4",0,"Cardiometabolic_II","B13805","20212097_SS211647","PASS",-1.8816,0.3862,"Plate control","PASS","PFC",1.30694642258082,"Baseline","Baseline"
4785,"PFC_10",14,"OID30226","Q8WZ75","ROBO4",0,"Cardiometabolic_II","B13805","20212097_SS211647","PASS",-1.8816,0.2661,"Plate control","PASS","PFC",1.20255260060385,"1h","1h"
4786,"PFC_18",15,"OID30226","Q8WZ75","ROBO4",0,"Cardiometabolic_II","B13805","20212097_SS211647","PASS",-1.8816,0.5509,"Plate control","PASS","PFC",1.46499932507677,"1h","1h"
4787,"PFC_26",16,"OID30226","Q8WZ75","ROBO4",0,"Cardiometabolic_II","B13805","20212097_SS211647","PASS",-1.8816,0.0525,"Plate control","PASS","PFC",1.03706045651031,"24h","24h"
4788,"PFC_03",25,"OID30226","Q8WZ75","ROBO4",0,"Cardiometabolic_II","B13805","20212097_SS211647","PASS",-1.8816,0.372,"Plate control","PASS","PFC",1.29414565448285,"Baseline","Baseline"
4789,"PFC_11",26,"OID30226","Q8WZ75","ROBO4",0,"Cardiometabolic_II","B13805","20212097_SS211647","PASS",-1.8816,0.3442,"Plate control","PASS","PFC",1.26944685652407,"1h","1h"
4790,"PFC_19",27,"OID30226","Q8WZ75","ROBO4",0,"Cardiometabolic_II","B13805","20212097_SS211647","PASS",-1.8816,0.3935,"Plate control","PASS","PFC",1.3135762973978,"24h","24h"
4791,"PFC_27",28,"OID30226","Q8WZ75","ROBO4",0,"Cardiometabolic_II","B13805","20212097_SS211647","PASS",-1.8816,0.4665,"Plate control","PASS","PFC",1.38175324435278,"24h","24h"
4792,"PFC_04",37,"OID30226","Q8WZ75","ROBO4",0,"Cardiometabolic_II","B13805","20212097_SS211647","PASS",-1.8816,0.1805,"Plate control","PASS","PFC",1.13327658097634,"Baseline","Baseline"
4793,"PFC_12",38,"OID30226","Q8WZ75","ROBO4",0,"Cardiometabolic_II","B13805","20212097_SS211647","PASS",-1.8816,0.1315,"Plate control","PASS","PFC",1.09543205283268,"1h","1h"
4794,"PFC_20",39,"OID30226","Q8WZ75","ROBO4",0,"Cardiometabolic_II","B13805","20212097_SS211647","PASS",-1.8816,0.2088,"Plate control","PASS","PFC",1.15572647773893,"24h","24h"
4795,"PFC_05",49,"OID30226","Q8WZ75","ROBO4",0,"Cardiometabolic_II","B13805","20212097_SS211647","PASS",-1.8816,0.1548,"Plate control","PASS","PFC",1.1132672759160４,"Baseline","Baseline"
4796,"PFC_13",50,"OID30226","Q8WZ75","ROBO4",0,"Cardiometabolic_II","B13805","20212097_SS211647","PASS",-1.8816,0.1035,"Plate control","PASS","PFC",1.07437675771437,"1h","1h"
4797,"PFC_21",51,"OID30226","Q8WZ75","ROBO4",0,"Cardiometabolic_II","B13805","20212097_SS211647","PASS",-1.8816,0.2797,"Plate control","PASS","PFC",1.21394242591636,"24h","24h"
4798,"PFC_06",61,"OID30226","Q8WZ75","ROBO4",0,"Cardiometabolic_II","B13805","20212097_SS211647","PASS",-1.8816,0.1889,"Plate control","PASS","PFC",1.13989425850593,"Baseline","Baseline"
4799,"PFC_14",62,"OID30226","Q8WZ75","ROBO4",0,"Cardiometabolic_II","B13805","20212097_SS211647","PASS",-1.8816,0.2101,"Plate control","PASS","PFC",1.15676836219937,"1h","1h"
4800,"PFC_22",63,"OID30226","Q8WZ75","ROBO4",0,"Cardiometabolic_II","B13805","20212097_SS211647","PASS",-1.8816,0.1306,"Plate control","PASS","PFC",1.09474889986631,"24h","24h"
4801,"PFC_07",73,"OID30226","Q8WZ75","ROBO4",0,"Cardiometabolic_II","B13805","20212097_SS211647","PASS",-1.8816,0.4863,"Plate control","PASS","PFC",1.40084758866938,"Baseline","Baseline"
4802,"PFC_15",74,"OID30226","Q8WZ75","ROBO4",0,"Cardiometabolic_II","B13805","20212097_SS211647","PASS",-1.8816,0.0722,"Plate control","PASS","PFC",1.0513186426966,"1h","1h"
4803,"PFC_23",75,"OID30226","Q8WZ75","ROBO4",0,"Cardiometabolic_II","B13805","20212097_SS211647","PASS",-1.8816,0.0685,"Plate control","PASS","PFC",1.04862583856901,"24h","24h"
4804,"PFC_08",85,"OID30226","Q8WZ75","ROBO4",0,"Cardiometabolic_II","B13805","20212097_SS211647","PASS",-1.8816,-0.1693,"Plate control","PASS","PFC",0.889274054968933,"Baseline","Baseline"
4805,"PFC_16",86,"OID30226","Q8WZ75","ROBO4",0,"Cardiometabolic_II","B13805","20212097_SS211647","PASS",-1.8816,0.1807,"Plate control","PASS","PFC",1.13343369735995,"1h","1h"
4806,"PFC_24",87,"OID30226","Q8WZ75","ROBO4",0,"Cardiometabolic_II","B13805","20212097_SS211647","PASS",-1.8816,0.1932,"Plate control","PASS","PFC",1.14329681902187,"24h","24h"
4807,"PFC_01",1,"OID30227","P55809","OXCT1",0,"Cardiometabolic_II","B13805","20212097_SS211647","PASS",-2.0457,1.5925,"Plate control","PASS","PFC",3.0157148051313,"Baseline","Baseline"
4808,"PFC_09",2,"OID30227","P55809","OXCT1",0,"Cardiometabolic_II","B13805","20212097_SS211647","PASS",-2.0457,2.1996,"Plate control","PASS","PFC",4.59351964933668,"Baseline","Baseline"
4809,"PFC_17",3,"OID30227","P55809","OXCT1",0,"Cardiometabolic_II","B13805","20212097_SS211647","PASS",-2.0457,4.669,"Plate control","PASS","PFC",25.4395280303672,"1h","1h"
4810,"PFC_25",4,"OID30227","P55809","OXCT1",0,"Cardiometabolic_II","B13805","20212097_SS211647","PASS",-2.0457,3.4458,"Plate control","PASS","PFC",10.8965535893338,"24h","24h"
4811,"PFC_02",13,"OID30227","P55809","OXCT1",0,"Cardiometabolic_II","B13805","20212097_SS211647","PASS",-2.0457,1.7507,"Plate control","PASS","PFC",3.36521807895215,"Baseline","Baseline"
4812,"PFC_10",14,"OID30227","P55809","OXCT1",0,"Cardiometabolic_II","B13805","20212097_SS211647","PASS",-2.0457,1.8642,"Plate control","PASS","PFC",3.64065996397977,"1h","1h"
4813,"PFC_18",15,"OID30227","P55809","OXCT1",0,"Cardiometabolic_II","B13805","20212097_SS211647","PASS",-2.0457,2.2869,"Plate control","PASS","PFC",4.88006376912116,"1h","1h"
4814,"PFC_26",16,"OID30227","P55809","OXCT1",0,"Cardiometabolic_II","B13805","20212097_SS211647","PASS",-2.0457,3.6894,"Plate control","PASS","PFC",12.9009016975265,"24h","24h"
4815,"PFC_03",25,"OID30227","P55809","OXCT1",0,"Cardiometabolic_II","B13805","20212097_SS211647","PASS",-2.0457,1.7807,"Plate control","PASS","PFC",3.43592846425921,"Baseline","Baseline"
4816,"PFC_11",26,"OID30227","P55809","OXCT1",0,"Cardiometabolic_II","B13805","20212097_SS211647","PASS",-2.0457,2.4522,"Plate control","PASS","PFC",5.47249981258995,"1h","1h"

Supplemental table 1_Olink proximity extension assay (PEA) data

,"SampleID","Index","OlinkID","UniProt","Assay","MissingFreq","Panel","Panel_Lot_Nr","PlateID","QC_Warning","LOD","NPX","Normalization","Assay_Warning","condition","Value","group","alt_group"
4817,"PFC_19",27,"OID30227","P55809","OXCT1",0,"Cardiometabolic_II","B13805","20212097_SS211647","PASS",-2.0457,2.8966,"Plate control","PASS","PFC",7.44669361052021,"24h","24h"
4818,"PFC_27",28,"OID30227","P55809","OXCT1",0,"Cardiometabolic_II","B13805","20212097_SS211647","PASS",-2.0457,2.9779,"Plate control","PASS","PFC",7.87838543617275,"24h","24h"
4819,"PFC_04",37,"OID30227","P55809","OXCT1",0,"Cardiometabolic_II","B13805","20212097_SS211647","PASS",-2.0457,2.2432,"Plate control","PASS","PFC",4.73446037709104,"Baseline","Baseline"
4820,"PFC_12",38,"OID30227","P55809","OXCT1",0,"Cardiometabolic_II","B13805","20212097_SS211647","PASS",-2.0457,2.1516,"Plate control","PASS","PFC",4.44320282641986,"1h","1h"
4821,"PFC_20",39,"OID30227","P55809","OXCT1",0,"Cardiometabolic_II","B13805","20212097_SS211647","PASS",-2.0457,3.0472,"Plate control","PASS","PFC",8.26606094141483,"24h","24h"
4822,"PFC_05",49,"OID30227","P55809","OXCT1",0,"Cardiometabolic_II","B13805","20212097_SS211647","PASS",-2.0457,1.2803,"Plate control","PASS","PFC",2.42889479075292,"Baseline","Baseline"
4823,"PFC_13",50,"OID30227","P55809","OXCT1",0,"Cardiometabolic_II","B13805","20212097_SS211647","PASS",-2.0457,2.7807,"Plate control","PASS","PFC",6.87185692851842,"1h","1h"
4824,"PFC_21",51,"OID30227","P55809","OXCT1",0,"Cardiometabolic_II","B13805","20212097_SS211647","PASS",-2.0457,2.6903,"Plate control","PASS","PFC",6.45447610481263,"24h","24h"
4825,"PFC_06",61,"OID30227","P55809","OXCT1",0,"Cardiometabolic_II","B13805","20212097_SS211647","PASS",-2.0457,2.1023,"Plate control","PASS","PFC",4.29393395120728,"Baseline","Baseline"
4826,"PFC_14",62,"OID30227","P55809","OXCT1",0,"Cardiometabolic_II","B13805","20212097_SS211647","PASS",-2.0457,2.918,"Plate control","PASS","PFC",7.55797632797765,"1h","1h"
4827,"PFC_22",63,"OID30227","P55809","OXCT1",0,"Cardiometabolic_II","B13805","20212097_SS211647","PASS",-2.0457,2.4011,"Plate control","PASS","PFC",5.28205747569608,"24h","24h"
4828,"PFC_07",73,"OID30227","P55809","OXCT1",0,"Cardiometabolic_II","B13805","20212097_SS211647","PASS",-2.0457,3.0445,"Plate control","PASS","PFC",8.25060549688105,"Baseline","Baseline"
4829,"PFC_15",74,"OID30227","P55809","OXCT1",0,"Cardiometabolic_II","B13805","20212097_SS211647","PASS",-2.0457,1.5044,"Plate control","PASS","PFC",2.83706656419883,"1h","1h"
4830,"PFC_23",75,"OID30227","P55809","OXCT1",0,"Cardiometabolic_II","B13805","20212097_SS211647","PASS",-2.0457,2.8109,"Plate control","PASS","PFC",7.01722197624214,"24h","24h"
4831,"PFC_08",85,"OID30227","P55809","OXCT1",0,"Cardiometabolic_II","B13805","20212097_SS211647","PASS",-2.0457,1.9097,"Plate control","PASS","PFC",3.75730960504816,"Baseline","Baseline"
4832,"PFC_16",86,"OID30227","P55809","OXCT1",0,"Cardiometabolic_II","B13805","20212097_SS211647","PASS",-2.0457,2.6964,"Plate control","PASS","PFC",6.48182468334407,"1h","1h"
4833,"PFC_24",87,"OID30227","P55809","OXCT1",0,"Cardiometabolic_II","B13805","20212097_SS211647","PASS",-2.0457,3.0516,"Plate control","PASS","PFC",8.29130965027864,"24h","24h"
4834,"PFC_01",1,"OID30228","Q9BY49","PECR",0.4318,"Cardiometabolic_II","B13805","20212097_SS211647","PASS",0.2364,0.0921,"Plate control","PASS","PFC",1.06592061749445,"Baseline","Baseline"
4835,"PFC_09",2,"OID30228","Q9BY49","PECR",0.4318,"Cardiometabolic_II","B13805","20212097_SS211647","PASS",0.2364,0.3154,"Plate control","PASS","PFC",1.24435661438605,"Baseline","Baseline"
4836,"PFC_17",3,"OID30228","Q9BY49","PECR",0.4318,"Cardiometabolic_II","B13805","20212097_SS211647","PASS",0.2364,0.3864,"Plate control","PASS","PFC",1.30712761638552,"1h","1h"
4837,"PFC_25",4,"OID30228","Q9BY49","PECR",0.4318,"Cardiometabolic_II","B13805","20212097_SS211647","PASS",0.2364,0.4572,"Plate control","PASS","PFC",1.37287473891439,"24h","24h"
4838,"PFC_02",13,"OID30228","Q9BY49","PECR",0.4318,"Cardiometabolic_II","B13805","20212097_SS211647","PASS",0.2364,0.2094,"Plate control","PASS","PFC",1.15620723083152,"Baseline","Baseline"
4839,"PFC_10",14,"OID30228","Q9BY49","PECR",0.4318,"Cardiometabolic_II","B13805","20212097_SS211647","PASS",0.2364,0.1442,"Plate control","PASS","PFC",1.10511767602152,"1h","1h"
4840,"PFC_18",15,"OID30228","Q9BY49","PECR",0.4318,"Cardiometabolic_II","B13805","20212097_SS211647","PASS",0.2364,0.6556,"Plate control","PASS","PFC",1.57527095369753,"1h","1h"
4841,"PFC_26",16,"OID30228","Q9BY49","PECR",0.4318,"Cardiometabolic_II","B13805","20212097_SS211647","PASS",0.2364,0.1399,"Plate control","PASS","PFC",1.10182874028119,"24h","24h"
4842,"PFC_03",25,"OID30228","Q9BY49","PECR",0.4318,"Cardiometabolic_II","B13805","20212097_SS211647","PASS",0.2364,0.0461,"Plate control","PASS","PFC",1.0324700983768,"Baseline","Baseline"
4843,"PFC_11",26,"OID30228","Q9BY49","PECR",0.4318,"Cardiometabolic_II","B13805","20212097_SS211647","PASS",0.2364,0.4477,"Plate control","PASS","PFC",1.36386419780303,"1h","1h"
4844,"PFC_19",27,"OID30228","Q9BY49","PECR",0.4318,"Cardiometabolic_II","B13805","20212097_SS211647","PASS",0.2364,0.557,"Plate control","PASS","PFC",1.47120674585574,"24h","24h"
4845,"PFC_27",28,"OID30228","Q9BY49","PECR",0.4318,"Cardiometabolic_II","B13805","20212097_SS211647","PASS",0.2364,1.128,"Plate control","PASS","PFC",2.18555547820191,"24h","24h"
4846,"PFC_04",37,"OID30228","Q9BY49","PECR",0.4318,"Cardiometabolic_II","B13805","20212097_SS211647","PASS",0.2364,0.2671,"Plate control","PASS","PFC",1.20338643550021,"Baseline","Baseline"
4847,"PFC_12",38,"OID30228","Q9BY49","PECR",0.4318,"Cardiometabolic_II","B13805","20212097_SS211647","PASS",0.2364,0.1229,"Plate control","PASS","PFC",1.08892153443102,"1h","1h"
4848,"PFC_20",39,"OID30228","Q9BY49","PECR",0.4318,"Cardiometabolic_II","B13805","20212097_SS211647","PASS",0.2364,0.903,"Plate control","PASS","PFC",1.86995039550291,"24h","24h"
4849,"PFC_05",49,"OID30228","Q9BY49","PECR",0.4318,"Cardiometabolic_II","B13805","20212097_SS211647","PASS",0.2364,0.0451,"Plate control","PASS","PFC",1.03175469260849,"Baseline","Baseline"
4850,"PFC_13",50,"OID30228","Q9BY49","PECR",0.4318,"Cardiometabolic_II","B13805","20212097_SS211647","PASS",0.2364,0.1483,"Plate control","PASS","PFC",1.10826278067204,"1h","1h"
4851,"PFC_21",51,"OID30228","Q9BY49","PECR",0.4318,"Cardiometabolic_II","B13805","20212097_SS211647","PASS",0.2364,0.2315,"Plate control","PASS","PFC",1.17405500423772,"24h","24h"
4852,"PFC_06",61,"OID30228","Q9BY49","PECR",0.4318,"Cardiometabolic_II","B13805","20212097_SS211647","PASS",0.2364,0.353,"Plate control","PASS","PFC",1.27721375919539,"Baseline","Baseline"
4853,"PFC_14",62,"OID30228","Q9BY49","PECR",0.4318,"Cardiometabolic_II","B13805","20212097_SS211647","PASS",0.2364,0.6387,"Plate control","PASS","PFC",1.55692559488373,"1h","1h"
4854,"PFC_22",63,"OID30228","Q9BY49","PECR",0.4318,"Cardiometabolic_II","B13805","20212097_SS211647","PASS",0.2364,0.1874,"Plate control","PASS","PFC",1.13870970267916,"24h","24h"
4855,"PFC_07",73,"OID30228","Q9BY49","PECR",0.4318,"Cardiometabolic_II","B13805","20212097_SS211647","PASS",0.2364,0.3826,"Plate control","PASS","PFC",1.30368922574056,"Baseline","Baseline"
4856,"PFC_15",74,"OID30228","Q9BY49","PECR",0.4318,"Cardiometabolic_II","B13805","20212097_SS211647","PASS",0.2364,-0.4225,"Plate control","PASS","PFC",0.74613055768702,"1h","1h"
4857,"PFC_23",75,"OID30228","Q9BY49","PECR",0.4318,"Cardiometabolic_II","B13805","20212097_SS211647","PASS",0.2364,0.4237,"Plate control","PASS","PFC",1.34136326722942,"24h","24h"
4858,"PFC_08",85,"OID30228","Q9BY49","PECR",0.4318,"Cardiometabolic_II","B13805","20212097_SS211647","PASS",0.2364,-0.3541,"Plate control","PASS","PFC",0.782357550963974,"Baseline","Baseline"
4859,"PFC_16",86,"OID30228","Q9BY49","PECR",0.4318,"Cardiometabolic_II","B13805","20212097_SS211647","PASS",0.2364,0.5009,"Plate control","PASS","PFC",1.41509606994203,"1h","1h"
4860,"PFC_24",87,"OID30228","Q9BY49","PECR",0.4318,"Cardiometabolic_II","B13805","20212097_SS211647","PASS",0.2364,0.184,"Plate control","PASS","PFC",1.13602926478669,"24h","24h"
4861,"PFC_01",1,"OID30229","Q9NZN3","EHD3",0,"Cardiometabolic_II","B13805","20212097_SS211647","PASS",-2.083,5.1922,"Plate control","PASS","PFC",36.5601478605431,"Baseline","Baseline"
4862,"PFC_09",2,"OID30229","Q9NZN3","EHD3",0,"Cardiometabolic_II","B13805","20212097_SS211647","PASS",-2.083,4.4646,"Plate control","PASS","PFC",22.0789552194116,"Baseline","Baseline"
4863,"PFC_17",3,"OID30229","Q9NZN3","EHD3",0,"Cardiometabolic_II","B13805","20212097_SS211647","PASS",-2.083,6.5271,"Plate control","PASS","PFC",92.2258963160816,"1h","1h"
4864,"PFC_25",4,"OID30229","Q9NZN3","EHD3",0,"Cardiometabolic_II","B13805","20212097_SS211647","PASS",-2.083,5.8279,"Plate control","PASS","PFC",56.8031881518169,"24h","24h"
4865,"PFC_02",13,"OID30229","Q9NZN3","EHD3",0,"Cardiometabolic_II","B13805","20212097_SS211647","PASS",-2.083,4.888,"Plate control","PASS","PFC",29.6097416972113,"Baseline","Baseline"
4866,"PFC_10",14,"OID30229","Q9NZN3","EHD3",0,"Cardiometabolic_II","B13805","20212097_SS211647","PASS",-2.083,5.421,"Plate control","PASS","PFC",42.8433682233102,"1h","1h"
4867,"PFC_18",15,"OID30229","Q9NZN3","EHD3",0,"Cardiometabolic_II","B13805","20212097_SS211647","PASS",-2.083,5.0333,"Plate control","PASS","PFC",32.7472079139947,"1h","1h"
4868,"PFC_26",16,"OID30229","Q9NZN3","EHD3",0,"Cardiometabolic_II","B13805","20212097_SS211647","PASS",-2.083,6.2109,"Plate control","PASS","PFC",74.0742392744072,"24h","24h"
4869,"PFC_03",25,"OID30229","Q9NZN3","EHD3",0,"Cardiometabolic_II","B13805","20212097_SS211647","PASS",-2.083,3.8816,"Plate control","PASS","PFC",14.7393397998718,"Baseline","Baseline"
4870,"PFC_11",26,"OID30229","Q9NZN3","EHD3",0,"Cardiometabolic_II","B13805","20212097_SS211647","PASS",-2.083,5.9712,"Plate control","PASS","PFC",62.7350589049231,"1h","1h"
4871,"PFC_19",27,"OID30229","Q9NZN3","EHD3",0,"Cardiometabolic_II","B13805","20212097_SS211647","PASS",-2.083,6.1223,"Plate control","PASS","PFC",69.662000566702,"24h","24h"
4872,"PFC_27",28,"OID30229","Q9NZN3","EHD3",0,"Cardiometabolic_II","B13805","20212097_SS211647","PASS",-2.083,6.2488,"Plate control","PASS","PFC",76.0459757821383,"24h","24h"
4873,"PFC_04",37,"OID30229","Q9NZN3","EHD3",0,"Cardiometabolic_II","B13805","20212097_SS211647","PASS",-2.083,6.0754,"Plate control","PASS","PFC",67.4338003288762,"Baseline","Baseline"
4874,"PFC_12",38,"OID30229","Q9NZN3","EHD3",0,"Cardiometabolic_II","B13805","20212097_SS211647","PASS",-2.083,4.7615,"Plate control","PASS","PFC",27.1240367642901,"1h","1h"
4875,"PFC_20",39,"OID30229","Q9NZN3","EHD3",0,"Cardiometabolic_II","B13805","20212097_SS211647","PASS",-2.083,6.3384,"Plate control","PASS","PFC",80.9186305993209,"24h","24h"
4876,"PFC_05",49,"OID30229","Q9NZN3","EHD3",0,"Cardiometabolic_II","B13805","20212097_SS211647","PASS",-2.083,3.8942,"Plate control","PASS","PFC",14.8686318752002,"Baseline","Baseline"
4877,"PFC_13",50,"OID30229","Q9NZN3","EHD3",0,"Cardiometabolic_II","B13805","20212097_SS211647","PASS",-2.083,6.4976,"Plate control","PASS","PFC",90.3592255102743,"1h","1h"
4878,"PFC_21",51,"OID30229","Q9NZN3","EHD3",0,"Cardiometabolic_II","B13805","20212097_SS211647","PASS",-2.083,5.3619,"Plate control","PASS","PFC",41.123752218937,"24h","24h"
4879,"PFC_06",61,"OID30229","Q9NZN3","EHD3",0,"Cardiometabolic_II","B13805","20212097_SS211647","PASS",-2.083,6.2359,"Plate control","PASS","PFC",75.3690341696646,"Baseline","Baseline"
4880,"PFC_14",62,"OID30229","Q9NZN3","EHD3",0,"Cardiometabolic_II","B13805","20212097_SS211647","PASS",-2.083,5.2525,"Plate control","PASS","PFC",38.1206284936278,"1h","1h"
4881,"PFC_22",63,"OID30229","Q9NZN3","EHD3",0,"Cardiometabolic_II","B13805","20212097_SS211647","PASS",-2.083,5.9982,"Plate control","PASS","PFC",63.9201992374577,"24h","24h"
4882,"PFC_07",73,"OID30229","Q9NZN3","EHD3",0,"Cardiometabolic_II","B13805","20212097_SS211647","PASS",-2.083,6.299,"Plate control","PASS","PFC",78.7386460640733,"Baseline","Baseline"
4883,"PFC_15",74,"OID30229","Q9NZN3","EHD3",0,"Cardiometabolic_II","B13805","20212097_SS211647","PASS",-2.083,5.1779,"Plate control","PASS","PFC",36.1995535622569,"1h","1h"
4884,"PFC_23",75,"OID30229","Q9NZN3","EHD3",0,"Cardiometabolic_II","B13805","20212097_SS211647","PASS",-2.083,5.2921,"Plate control","PASS","PFC",39.1814803159455,"24h","24h"
4885,"PFC_08",85,"OID30229","Q9NZN3","EHD3",0,"Cardiometabolic_II","B13805","20212097_SS211647","PASS",-2.083,5.4398,"Plate control","PASS","PFC",43.4053208060743,"Baseline","Baseline"
4886,"PFC_16",86,"OID30229","Q9NZN3","EHD3",0,"Cardiometabolic_II","B13805","20212097_SS211647","PASS",-2.083,5.8811,"Plate control","PASS","PFC",58.9369296762073,"1h","1h"
4887,"PFC_24",87,"OID30229","Q9NZN3","EHD3",0,"Cardiometabolic_II","B13805","20212097_SS211647","PASS",-2.083,6.7541,"Plate control","PASS","PFC",107.941063757118,"24h","24h"
4888,"PFC_01",1,"OID30230","P14902","IDO1",0,"Cardiometabolic_II","B13805","20212097_SS211647","PASS",-3.788,-0.5814,"Plate control","PASS","PFC",0.66831492577007,"Baseline","Baseline"
4889,"PFC_09",2,"OID30230","P14902","IDO1",0,"Cardiometabolic_II","B13805","20212097_SS211647","PASS",-3.788,-0.2613,"Plate control","PASS","PFC",0.834335767869956,"Baseline","Baseline"
4890,"PFC_17",3,"OID30230","P14902","IDO1",0,"Cardiometabolic_II","B13805","20212097_SS211647","PASS",-3.788,-1.9675,"Plate control","PASS","PFC",0.255695734686821,"1h","1h"
4891,"PFC_25",4,"OID30230","P14902","IDO1",0,"Cardiometabolic_II","B13805","20212097_SS211647","PASS",-3.788,-1.3965,"Plate control","PASS","PFC",0.379849545464523,"24h","24h"
4892,"PFC_02",13,"OID30230","P14902","IDO1",0,"Cardiometabolic_II","B13805","20212097_SS211647","PASS",-3.788,-0.445,"Plate control","PASS","PFC",0.734584316639154,"Baseline","Baseline"
4893,"PFC_10",14,"OID30230","P14902","IDO1",0,"Cardiometabolic_II","B13805","20212097_SS211647","PASS",-3.788,-0.8894,"Plate control","PASS","PFC",0.539838584128139,"1h","1h"
4894,"PFC_18",15,"OID30230","P14902","IDO1",0,"Cardiometabolic_II","B13805","20212097_SS211647","PASS",-3.788,-0.9399,"Plate control","PASS","PFC",0.521269010782877,"1h","1h"
4895,"PFC_26",16,"OID30230","P14902","IDO1",0,"Cardiometabolic_II","B13805","20212097_SS211647","PASS",-3.788,-2.1375,"Plate control","PASS","PFC",0.22727328238189,"24h","24h"
4896,"PFC_03",25,"OID30230","P14902","IDO1",0,"Cardiometabolic_II","B13805","20212097_SS211647","PASS",-3.788,-1.5845,"Plate control","PASS","PFC",0.333440210820826,"Baseline","Baseline"
4897,"PFC_11",26,"OID30230","P14902","IDO1",0,"Cardiometabolic_II","B13805","20212097_SS211647","PASS",-3.788,-0.6284,"Plate control","PASS","PFC",0.646893445427848,"1h","1h"
4898,"PFC_19",27,"OID30230","P14902","IDO1",0,"Cardiometabolic_II","B13805","20212097_SS211647","PASS",-3.788,-0.5847,"Plate control","PASS","PFC",0.666787978792645,"24h","24h"
4899,"PFC_27",28,"OID30230","P14902","IDO1",0,"Cardiometabolic_II","B13805","20212097_SS211647","PASS",-3.788,-0.403,"Plate control","PASS","PFC",0.756283998641981,"24h","24h"
4900,"PFC_04",37,"OID30230","P14902","IDO1",0,"Cardiometabolic_II","B13805","20212097_SS211647","PASS",-3.788,-0.2768,"Plate control","PASS","PFC",0.825419828066835,"Baseline","Baseline"
4901,"PFC_12",38,"OID30230","P14902","IDO1",0,"Cardiometabolic_II","B13805","20212097_SS211647","PASS",-3.788,-1.7705,"Plate control","PASS","PFC",0.293107136510273,"1h","1h"
4902,"PFC_20",39,"OID30230","P14902","IDO1",0,"Cardiometabolic_II","B13805","20212097_SS211647","PASS",-3.788,-0.734,"Plate control","PASS","PFC",0.601234624449089,"24h","24h"
4903,"PFC_05",49,"OID30230","P14902","IDO1",0,"Cardiometabolic_II","B13805","20212097_SS211647","PASS",-3.788,-0.2972,"Plate control","PASS","PFC",0.813830356406529,"Baseline","Baseline"
4904,"PFC_13",50,"OID30230","P14902","IDO1",0,"Cardiometabolic_II","B13805","20212097_SS211647","PASS",-3.788,-0.9551,"Plate control","PASS","PFC",0.515805835475491,"1h","1h"
4905,"PFC_21",51,"OID30230","P14902","IDO1",0,"Cardiometabolic_II","B13805","20212097_SS211647","PASS",-3.788,-1.9396,"Plate control","PASS","PFC",0.260688708448618,"24h","24h"
4906,"PFC_06",61,"OID30230","P14902","IDO1",0,"Cardiometabolic_II","B13805","20212097_SS211647","PASS",-3.788,-1.3153,"Plate control","PASS","PFC",0.401841926003584,"Baseline","Baseline"
4907,"PFC_14",62,"OID30230","P14902","IDO1",0,"Cardiometabolic_II","B13805","20212097_SS211647","PASS",-3.788,-0.726,"Plate control","PASS","PFC",0.604577837916681,"1h","1h"
4908,"PFC_22",63,"OID30230","P14902","IDO1",0,"Cardiometabolic_II","B13805","20212097_SS211647","PASS",-3.788,-1.0559,"Plate control","PASS","PFC",0.480997066352972,"24h","24h"
4909,"PFC_07",73,"OID30230","P14902","IDO1",0,"Cardiometabolic_II","B13805","20212097_SS211647","PASS",-3.788,-1.1482,"Plate control","PASS","PFC",0.451187811483637,"Baseline","Baseline"
4910,"PFC_15",74,"OID30230","P14902","IDO1",0,"Cardiometabolic_II","B13805","20212097_SS211647","PASS",-3.788,-1.6555,"Plate control","PASS","PFC",0.317427714506186,"1h","1h"
4911,"PFC_23",75,"OID30230","P14902","IDO1",0,"Cardiometabolic_II","B13805","20212097_SS211647","PASS",-3.788,-0.1743,"Plate control","PASS","PFC",0.886197400464744,"24h","24h"
4912,"PFC_08",85,"OID30230","P14902","IDO1",0,"Cardiometabolic_II","B13805","20212097_SS211647","PASS",-3.788,-2.2179,"Plate control","PASS","PFC",0.214954020521714,"Baseline","Baseline"
4913,"PFC_16",86,"OID30230","P14902","IDO1",0,"Cardiometabolic_II","B13805","20212097_SS211647","PASS",-3.788,-1.8763,"Plate control","PASS","PFC",0.272381382047832,"1h","1h"
4914,"PFC_24",87,"OID30230","P14902","IDO1",0,"Cardiometabolic_II","B13805","20212097_SS211647","PASS",-3.788,-1.4574,"Plate control","PASS","PFC",0.364148800990814,"24h","24h"
4915,"PFC_01",1,"OID30231","Q96C92","ENTR1",0,"Cardiometabolic_II","B13805","20212097_SS211647","PASS",-2.4506,-0.3415,"Plate control","PASS","PFC",0.7892203163786,"Baseline","Baseline"
4916,"PFC_09",2,"OID30231","Q96C92","ENTR1",0,"Cardiometabolic_II","B13805","20212097_SS211647","PASS",-2.4506,0.8149,"Plate control","PASS","PFC",1.7591762108157,"Baseline","Baseline"
4917,"PFC_17",3,"OID30231","Q96C92","ENTR1",0,"Cardiometabolic_II","B13805","20212097_SS211647","PASS",-2.4506,0.9292,"Plate control","PASS","PFC",1.90421977961243,"1h","1h"
4918,"PFC_25",4,"OID30231","Q96C92","ENTR1",0,"Cardiometabolic_II","B13805","20212097_SS211647","PASS",-2.4506,0.6971,"Plate control","PASS","PFC",1.62124261185059,"24h","24h"
4919,"PFC_02",13,"OID30231","Q96C92","ENTR1",0,"Cardiometabolic_II","B13805","20212097_SS211647","PASS",-2.4506,0.665,"Plate control","PASS","PFC",1.58556827322057,"Baseline","Baseline"
4920,"PFC_10",14,"OID30231","Q96C92","ENTR1",0,"Cardiometabolic_II","B13805","20212097_SS211647","PASS",-2.4506,0.0477,"Plate control","PASS","PFC",1.03361577954023,"1h","1h"
4921,"PFC_18",15,"OID30231","Q96C92","ENTR1",0,"Cardiometabolic_II","B13805","20212097_SS211647","PASS",-2.4506,0.6591,"Plate control","PASS","PFC",1.57909722428667,"1h","1h"
4922,"PFC_26",16,"OID30231","Q96C92","ENTR1",0,"Cardiometabolic_II","B13805","20212097_SS211647","PASS",-2.4506,0.6037,"Plate control","PASS","PFC",1.51960882977194,"24h","24h"
4923,"PFC_03",25,"OID30231","Q96C92","ENTR1",0,"Cardiometabolic_II","B13805","20212097_SS211647","PASS",-2.4506,0.0219,"Plate control","PASS","PFC",1.01529572349355,"Baseline","Baseline"
4924,"PFC_11",26,"OID30231","Q96C92","ENTR1",0,"Cardiometabolic_II","B13805","20212097_SS211647","PASS",-2.4506,1.4659,"Plate control","PASS","PFC",2.76235741762607,"1h","1h"
4925,"PFC_19",27,"OID30231","Q96C92","ENTR1",0,"Cardiometabolic_II","B13805","20212097_SS211647","PASS",-2.4506,0.2081,"Plate control","PASS","PFC",1.15516585177396,"24h","24h"
4926,"PFC_27",28,"OID30231","Q96C92","ENTR1",0,"Cardiometabolic_II","B13805","20212097_SS211647","PASS",-2.4506,1.0885,"Plate control","PASS","PFC",2.12652821953963,"24h","24h"
4927,"PFC_04",37,"OID30231","Q96C92","ENTR1",0,"Cardiometabolic_II","B13805","20212097_SS211647","PASS",-2.4506,0.367,"Plate control","PASS","PFC",1.28966825065589,"Baseline","Baseline"
4928,"PFC_12",38,"OID30231","Q96C92","ENTR1",0,"Cardiometabolic_II","B13805","20212097_SS211647","PASS",-2.4506,-0.2213,"Plate control","PASS","PFC",0.857792139020808,"1h","1h"

Supplemental table 1_Olink proximity extension assay (PEA) data

,"SampleID","Index","OlinkID","UniProt","Assay","MissingFreq","Panel","Panel_Lot_Nr","PlateID","QC_Warning","LOD","NPX","Normalization","Assay_Warning","condition","Value","group","alt_group"
4929,"PFC_20",39,"OID30231","Q96C92","ENTR1",0,"Cardiometabolic_II","B13805","20212097_SS211647","PASS",-2.4506,1.5892,"Plate control","PASS","PFC",3.00882458552238,"24h","24h"
4930,"PFC_05",49,"OID30231","Q96C92","ENTR1",0,"Cardiometabolic_II","B13805","20212097_SS211647","PASS",-2.4506,1.432,"Plate control","PASS","PFC",2.6982050686794,"Baseline","Baseline"
4931,"PFC_13",50,"OID30231","Q96C92","ENTR1",0,"Cardiometabolic_II","B13805","20212097_SS211647","PASS",-2.4506,0.7345,"Plate control","PASS","PFC",1.66382073316297,"1h","1h"
4932,"PFC_21",51,"OID30231","Q96C92","ENTR1",0,"Cardiometabolic_II","B13805","20212097_SS211647","PASS",-2.4506,-0.2568,"Plate control","PASS","PFC",0.836942259475024,"24h","24h"
4933,"PFC_06",61,"OID30231","Q96C92","ENTR1",0,"Cardiometabolic_II","B13805","20212097_SS211647","PASS",-2.4506,0.5101,"Plate control","PASS","PFC",1.4241489066559,"Baseline","Baseline"
4934,"PFC_14",62,"OID30231","Q96C92","ENTR1",0,"Cardiometabolic_II","B13805","20212097_SS211647","PASS",-2.4506,1.8309,"Plate control","PASS","PFC",3.55758937230082,"1h","1h"
4935,"PFC_22",63,"OID30231","Q96C92","ENTR1",0,"Cardiometabolic_II","B13805","20212097_SS211647","PASS",-2.4506,0.1898,"Plate control","PASS","PFC",1.14060558339892,"24h","24h"
4936,"PFC_07",73,"OID30231","Q96C92","ENTR1",0,"Cardiometabolic_II","B13805","20212097_SS211647","PASS",-2.4506,0.6952,"Plate control","PASS","PFC",1.61910887368758,"Baseline","Baseline"
4937,"PFC_15",74,"OID30231","Q96C92","ENTR1",0,"Cardiometabolic_II","B13805","20212097_SS211647","PASS",-2.4506,0.2858,"Plate control","PASS","PFC",1.21908608114549,"1h","1h"
4938,"PFC_23",75,"OID30231","Q96C92","ENTR1",0,"Cardiometabolic_II","B13805","20212097_SS211647","PASS",-2.4506,1.8049,"Plate control","PASS","PFC",3.49404938622487,"24h","24h"
4939,"PFC_08",85,"OID30231","Q96C92","ENTR1",0,"Cardiometabolic_II","B13805","20212097_SS211647","PASS",-2.4506,0.1438,"Plate control","PASS","PFC",1.10481131481364,"Baseline","Baseline"
4940,"PFC_16",86,"OID30231","Q96C92","ENTR1",0,"Cardiometabolic_II","B13805","20212097_SS211647","PASS",-2.4506,1.1445,"Plate control","PASS","PFC",2.21069500535329,"1h","1h"
4941,"PFC_24",87,"OID30231","Q96C92","ENTR1",0,"Cardiometabolic_II","B13805","20212097_SS211647","PASS",-2.4506,1.0744,"Plate control","PASS","PFC",2.10584609299837,"24h","24h"
4942,"PFC_01",1,"OID30232","O75506","HSBP1",0,"Cardiometabolic_II","B13805","20212097_SS211647","PASS",-3.2261,1.9044,"Plate control","PASS","PFC",3.74353177484964,"Baseline","Baseline"
4943,"PFC_09",2,"OID30232","O75506","HSBP1",0,"Cardiometabolic_II","B13805","20212097_SS211647","PASS",-3.2261,2.2986,"Plate control","PASS","PFC",4.91980113137519,"Baseline","Baseline"
4944,"PFC_17",3,"OID30232","O75506","HSBP1",0,"Cardiometabolic_II","B13805","20212097_SS211647","PASS",-3.2261,2.6213,"Plate control","PASS","PFC",6.15304268131507,"1h","1h"
4945,"PFC_25",4,"OID30232","O75506","HSBP1",0,"Cardiometabolic_II","B13805","20212097_SS211647","PASS",-3.2261,3.7529,"Plate control","PASS","PFC",13.4814147791993,"24h","24h"
4946,"PFC_02",13,"OID30232","O75506","HSBP1",0,"Cardiometabolic_II","B13805","20212097_SS211647","PASS",-3.2261,1.2345,"Plate control","PASS","PFC",2.35299784619662,"Baseline","Baseline"
4947,"PFC_10",14,"OID30232","O75506","HSBP1",0,"Cardiometabolic_II","B13805","20212097_SS211647","PASS",-3.2261,2.2947,"Plate control","PASS","PFC",4.90651952087534,"1h","1h"
4948,"PFC_18",15,"OID30232","O75506","HSBP1",0,"Cardiometabolic_II","B13805","20212097_SS211647","PASS",-3.2261,2.6298,"Plate control","PASS","PFC",6.18940188139979,"1h","1h"
4949,"PFC_26",16,"OID30232","O75506","HSBP1",0,"Cardiometabolic_II","B13805","20212097_SS211647","PASS",-3.2261,2.4981,"Plate control","PASS","PFC",5.64940919117322,"24h","24h"
4950,"PFC_03",25,"OID30232","O75506","HSBP1",0,"Cardiometabolic_II","B13805","20212097_SS211647","PASS",-3.2261,0.3352,"Plate control","PASS","PFC",1.26155228499137,"Baseline","Baseline"
4951,"PFC_11",26,"OID30232","O75506","HSBP1",0,"Cardiometabolic_II","B13805","20212097_SS211647","PASS",-3.2261,2.4017,"Plate control","PASS","PFC",5.28425467850799,"1h","1h"
4952,"PFC_19",27,"OID30232","O75506","HSBP1",0,"Cardiometabolic_II","B13805","20212097_SS211647","PASS",-3.2261,3.0263,"Plate control","PASS","PFC",8.14717557951884,"24h","24h"
4953,"PFC_27",28,"OID30232","O75506","HSBP1",0,"Cardiometabolic_II","B13805","20212097_SS211647","PASS",-3.2261,3.3309,"Plate control","PASS","PFC",10.0623822793244,"24h","24h"
4954,"PFC_04",37,"OID30232","O75506","HSBP1",0,"Cardiometabolic_II","B13805","20212097_SS211647","PASS",-3.2261,3.3931,"Plate control","PASS","PFC",10.5056971775942,"Baseline","Baseline"
4955,"PFC_12",38,"OID30232","O75506","HSBP1",0,"Cardiometabolic_II","B13805","20212097_SS211647","PASS",-3.2261,0.6726,"Plate control","PASS","PFC",1.59394295698359,"1h","1h"
4956,"PFC_20",39,"OID30232","O75506","HSBP1",0,"Cardiometabolic_II","B13805","20212097_SS211647","PASS",-3.2261,2.5457,"Plate control","PASS","PFC",5.8389137483891,"24h","24h"
4957,"PFC_05",49,"OID30232","O75506","HSBP1",0,"Cardiometabolic_II","B13805","20212097_SS211647","PASS",-3.2261,2.0504,"Plate control","PASS","PFC",4.14220800008794,"Baseline","Baseline"
4958,"PFC_13",50,"OID30232","O75506","HSBP1",0,"Cardiometabolic_II","B13805","20212097_SS211647","PASS",-3.2261,3.6336,"Plate control","PASS","PFC",12.4114520061182,"1h","1h"
4959,"PFC_21",51,"OID30232","O75506","HSBP1",0,"Cardiometabolic_II","B13805","20212097_SS211647","PASS",-3.2261,1.3099,"Plate control","PASS","PFC",2.47924354585466,"24h","24h"
4960,"PFC_06",61,"OID30232","O75506","HSBP1",0,"Cardiometabolic_II","B13805","20212097_SS211647","PASS",-3.2261,2.6328,"Plate control","PASS","PFC",6.20228577179599,"Baseline","Baseline"
4961,"PFC_14",62,"OID30232","O75506","HSBP1",0,"Cardiometabolic_II","B13805","20212097_SS211647","PASS",-3.2261,3.1577,"Plate control","PASS","PFC",8.92405867981083,"1h","1h"
4962,"PFC_22",63,"OID30232","O75506","HSBP1",0,"Cardiometabolic_II","B13805","20212097_SS211647","PASS",-3.2261,3.2658,"Plate control","PASS","PFC",9.61842050254661,"24h","24h"
4963,"PFC_07",73,"OID30232","O75506","HSBP1",0,"Cardiometabolic_II","B13805","20212097_SS211647","PASS",-3.2261,3.9101,"Plate control","PASS","PFC",15.0334059876082,"Baseline","Baseline"
4964,"PFC_15",74,"OID30232","O75506","HSBP1",0,"Cardiometabolic_II","B13805","20212097_SS211647","PASS",-3.2261,1.5705,"Plate control","PASS","PFC",2.97007631291711,"1h","1h"
4965,"PFC_23",75,"OID30232","O75506","HSBP1",0,"Cardiometabolic_II","B13805","20212097_SS211647","PASS",-3.2261,3.1607,"Plate control","PASS","PFC",8.94263504568967,"24h","24h"
4966,"PFC_08",85,"OID30232","O75506","HSBP1",0,"Cardiometabolic_II","B13805","20212097_SS211647","PASS",-3.2261,1.4744,"Plate control","PASS","PFC",2.77868057858154,"Baseline","Baseline"
4967,"PFC_16",86,"OID30232","O75506","HSBP1",0,"Cardiometabolic_II","B13805","20212097_SS211647","PASS",-3.2261,3.5479,"Plate control","PASS","PFC",11.6956488784991,"1h","1h"
4968,"PFC_24",87,"OID30232","O75506","HSBP1",0,"Cardiometabolic_II","B13805","20212097_SS211647","PASS",-3.2261,2.9425,"Plate control","PASS","PFC",7.68742270768827,"24h","24h"
4969,"PFC_01",1,"OID30233","P01225","FSHB",0.6818,"Cardiometabolic_II","B13805","20212097_SS211647","PASS",-0.6663,-0.7038,"Plate control","PASS","PFC",0.613952948009274,"Baseline","Baseline"
4970,"PFC_09",2,"OID30233","P01225","FSHB",0.6818,"Cardiometabolic_II","B13805","20212097_SS211647","PASS",-0.6663,-0.8043,"Plate control","PASS","PFC",0.5726398594971,"Baseline","Baseline"
4971,"PFC_17",3,"OID30233","P01225","FSHB",0.6818,"Cardiometabolic_II","B13805","20212097_SS211647","PASS",-0.6663,-1.0303,"Plate control","PASS","PFC",0.489608327017774,"1h","1h"
4972,"PFC_25",4,"OID30233","P01225","FSHB",0.6818,"Cardiometabolic_II","B13805","20212097_SS211647","PASS",-0.6663,-0.3828,"Plate control","PASS","PFC",0.766947643986654,"24h","24h"
4973,"PFC_02",13,"OID30233","P01225","FSHB",0.6818,"Cardiometabolic_II","B13805","20212097_SS211647","PASS",-0.6663,-1.0629,"Plate control","PASS","PFC",0.478668906748099,"Baseline","Baseline"
4974,"PFC_10",14,"OID30233","P01225","FSHB",0.6818,"Cardiometabolic_II","B13805","20212097_SS211647","PASS",-0.6663,-0.6985,"Plate control","PASS","PFC",0.616212562719742,"1h","1h"
4975,"PFC_18",15,"OID30233","P01225","FSHB",0.6818,"Cardiometabolic_II","B13805","20212097_SS211647","PASS",-0.6663,-1.009,"Plate control","PASS","PFC",0.496890546661277,"1h","1h"
4976,"PFC_26",16,"OID30233","P01225","FSHB",0.6818,"Cardiometabolic_II","B13805","20212097_SS211647","PASS",-0.6663,-0.6884,"Plate control","PASS","PFC",0.62054167122563,"24h","24h"
4977,"PFC_03",25,"OID30233","P01225","FSHB",0.6818,"Cardiometabolic_II","B13805","20212097_SS211647","PASS",-0.6663,-1.253,"Plate control","PASS","PFC",0.419574818553271,"Baseline","Baseline"
4978,"PFC_11",26,"OID30233","P01225","FSHB",0.6818,"Cardiometabolic_II","B13805","20212097_SS211647","PASS",-0.6663,-1.0998,"Plate control","PASS","PFC",0.466581173170141,"1h","1h"
4979,"PFC_19",27,"OID30233","P01225","FSHB",0.6818,"Cardiometabolic_II","B13805","20212097_SS211647","PASS",-0.6663,-0.7607,"Plate control","PASS","PFC",0.590209889604695,"24h","24h"
4980,"PFC_27",28,"OID30233","P01225","FSHB",0.6818,"Cardiometabolic_II","B13805","20212097_SS211647","PASS",-0.6663,-0.9207,"Plate control","PASS","PFC",0.528252648305833,"24h","24h"
4981,"PFC_04",37,"OID30233","P01225","FSHB",0.6818,"Cardiometabolic_II","B13805","20212097_SS211647","PASS",-0.6663,-1.1232,"Plate control","PASS","PFC",0.459074435508856,"Baseline","Baseline"
4982,"PFC_12",38,"OID30233","P01225","FSHB",0.6818,"Cardiometabolic_II","B13805","20212097_SS211647","PASS",-0.6663,-0.9765,"Plate control","PASS","PFC",0.508211173549965,"1h","1h"
4983,"PFC_20",39,"OID30233","P01225","FSHB",0.6818,"Cardiometabolic_II","B13805","20212097_SS211647","PASS",-0.6663,-1.0961,"Plate control","PASS","PFC",0.467779323800003,"24h","24h"
4984,"PFC_05",49,"OID30233","P01225","FSHB",0.6818,"Cardiometabolic_II","B13805","20212097_SS211647","PASS",-0.6663,-1.195,"Plate control","PASS","PFC",0.436786447958347,"Baseline","Baseline"
4985,"PFC_13",50,"OID30233","P01225","FSHB",0.6818,"Cardiometabolic_II","B13805","20212097_SS211647","PASS",-0.6663,-0.9468,"Plate control","PASS","PFC",0.51878188173776,"1h","1h"
4986,"PFC_21",51,"OID30233","P01225","FSHB",0.6818,"Cardiometabolic_II","B13805","20212097_SS211647","PASS",-0.6663,-1.0401,"Plate control","PASS","PFC",0.48629376522268,"24h","24h"
4987,"PFC_06",61,"OID30233","P01225","FSHB",0.6818,"Cardiometabolic_II","B13805","20212097_SS211647","PASS",-0.6663,-0.9442,"Plate control","PASS","PFC",0.519717664426653,"Baseline","Baseline"
4988,"PFC_14",62,"OID30233","P01225","FSHB",0.6818,"Cardiometabolic_II","B13805","20212097_SS211647","PASS",-0.6663,-1.2531,"Plate control","PASS","PFC",0.419545736850926,"1h","1h"
4989,"PFC_22",63,"OID30233","P01225","FSHB",0.6818,"Cardiometabolic_II","B13805","20212097_SS211647","PASS",-0.6663,-0.9829,"Plate control","PASS","PFC",0.505961669888534,"24h","24h"
4990,"PFC_07",73,"OID30233","P01225","FSHB",0.6818,"Cardiometabolic_II","B13805","20212097_SS211647","PASS",-0.6663,-0.6674,"Plate control","PASS","PFC",0.629640392385354,"Baseline","Baseline"
4991,"PFC_15",74,"OID30233","P01225","FSHB",0.6818,"Cardiometabolic_II","B13805","20212097_SS211647","PASS",-0.6663,-1.101,"Plate control","PASS","PFC",0.466193243218416,"1h","1h"
4992,"PFC_23",75,"OID30233","P01225","FSHB",0.6818,"Cardiometabolic_II","B13805","20212097_SS211647","PASS",-0.6663,-1.2552,"Plate control","PASS","PFC",0.418935486517949,"24h","24h"
4993,"PFC_08",85,"OID30233","P01225","FSHB",0.6818,"Cardiometabolic_II","B13805","20212097_SS211647","PASS",-0.6663,-0.9088,"Plate control","PASS","PFC",0.532627934489107,"Baseline","Baseline"
4994,"PFC_16",86,"OID30233","P01225","FSHB",0.6818,"Cardiometabolic_II","B13805","20212097_SS211647","PASS",-0.6663,-0.0041,"Plate control","PASS","PFC",0.997162130944604,"1h","1h"
4995,"PFC_24",87,"OID30233","P01225","FSHB",0.6818,"Cardiometabolic_II","B13805","20212097_SS211647","PASS",-0.6663,-1.1216,"Plate control","PASS","PFC",0.459583847775809,"24h","24h"
4996,"PFC_01",1,"OID30234","O95980","RECK",0,"Cardiometabolic_II","B13805","20212097_SS211647","PASS",-3.9135,0.2382,"Plate control","PASS","PFC",1.17952009713361,"Baseline","Baseline"
4997,"PFC_09",2,"OID30234","O95980","RECK",0,"Cardiometabolic_II","B13805","20212097_SS211647","PASS",-3.9135,0.4168,"Plate control","PASS","PFC",1.33496322526842,"Baseline","Baseline"
4998,"PFC_17",3,"OID30234","O95980","RECK",0,"Cardiometabolic_II","B13805","20212097_SS211647","PASS",-3.9135,0.2135,"Plate control","PASS","PFC",1.15949773356942,"1h","1h"
4999,"PFC_25",4,"OID30234","O95980","RECK",0,"Cardiometabolic_II","B13805","20212097_SS211647","PASS",-3.9135,0.2042,"Plate control","PASS","PFC",1.15204733895275,"24h","24h"
5000,"PFC_02",13,"OID30234","O95980","RECK",0,"Cardiometabolic_II","B13805","20212097_SS211647","PASS",-3.9135,0.1951,"Plate control","PASS","PFC",1.14480350958112,"Baseline","Baseline"
5001,"PFC_10",14,"OID30234","O95980","RECK",0,"Cardiometabolic_II","B13805","20212097_SS211647","PASS",-3.9135,0.1091,"Plate control","PASS","PFC",1.07855518885236,"1h","1h"
5002,"PFC_18",15,"OID30234","O95980","RECK",0,"Cardiometabolic_II","B13805","20212097_SS211647","PASS",-3.9135,0.0732,"Plate control","PASS","PFC",1.05204761386262,"1h","1h"
5003,"PFC_26",16,"OID30234","O95980","RECK",0,"Cardiometabolic_II","B13805","20212097_SS211647","PASS",-3.9135,0.246,"Plate control","PASS","PFC",1.18591449941095,"24h","24h"
5004,"PFC_03",25,"OID30234","O95980","RECK",0,"Cardiometabolic_II","B13805","20212097_SS211647","PASS",-3.9135,0.036,"Plate control","PASS","PFC",1.02526723788859,"Baseline","Baseline"
5005,"PFC_11",26,"OID30234","O95980","RECK",0,"Cardiometabolic_II","B13805","20212097_SS211647","PASS",-3.9135,0.0911,"Plate control","PASS","PFC",1.06518203362697,"1h","1h"
5006,"PFC_19",27,"OID30234","O95980","RECK",0,"Cardiometabolic_II","B13805","20212097_SS211647","PASS",-3.9135,0.2919,"Plate control","PASS","PFC",1.22425153080947,"24h","24h"
5007,"PFC_27",28,"OID30234","O95980","RECK",0,"Cardiometabolic_II","B13805","20212097_SS211647","PASS",-3.9135,0.3789,"Plate control","PASS","PFC",1.3003500100303,"24h","24h"
5008,"PFC_04",37,"OID30234","O95980","RECK",0,"Cardiometabolic_II","B13805","20212097_SS211647","PASS",-3.9135,0.2298,"Plate control","PASS","PFC",1.17267237105354,"Baseline","Baseline"
5009,"PFC_12",38,"OID30234","O95980","RECK",0,"Cardiometabolic_II","B13805","20212097_SS211647","PASS",-3.9135,-0.1297,"Plate control","PASS","PFC",0.914021495896128,"1h","1h"
5010,"PFC_20",39,"OID30234","O95980","RECK",0,"Cardiometabolic_II","B13805","20212097_SS211647","PASS",-3.9135,0.1339,"Plate control","PASS","PFC",1.0972558789614,"24h","24h"
5011,"PFC_05",49,"OID30234","O95980","RECK",0,"Cardiometabolic_II","B13805","20212097_SS211647","PASS",-3.9135,0.0412,"Plate control","PASS","PFC",1.02896934344646,"Baseline","Baseline"
5012,"PFC_13",50,"OID30234","O95980","RECK",0,"Cardiometabolic_II","B13805","20212097_SS211647","PASS",-3.9135,0.1806,"Plate control","PASS","PFC",1.13335513644552,"1h","1h"
5013,"PFC_21",51,"OID30234","O95980","RECK",0,"Cardiometabolic_II","B13805","20212097_SS211647","PASS",-3.9135,-0.0803,"Plate control","PASS","PFC",0.945860940021167,"24h","24h"
5014,"PFC_06",61,"OID30234","O95980","RECK",0,"Cardiometabolic_II","B13805","20212097_SS211647","PASS",-3.9135,0.0866,"Plate control","PASS","PFC",1.06186473425884,"Baseline","Baseline"
5015,"PFC_14",62,"OID30234","O95980","RECK",0,"Cardiometabolic_II","B13805","20212097_SS211647","PASS",-3.9135,-0.033,"Plate control","PASS","PFC",0.977385766410805,"1h","1h"
5016,"PFC_22",63,"OID30234","O95980","RECK",0,"Cardiometabolic_II","B13805","20212097_SS211647","PASS",-3.9135,0.0667,"Plate control","PASS","PFC",1.04731832073245,"24h","24h"
5017,"PFC_07",73,"OID30234","O95980","RECK",0,"Cardiometabolic_II","B13805","20212097_SS211647","PASS",-3.9135,0.4319,"Plate control","PASS","PFC",1.34900902491873,"Baseline","Baseline"
5018,"PFC_15",74,"OID30234","O95980","RECK",0,"Cardiometabolic_II","B13805","20212097_SS211647","PASS",-3.9135,-0.3495,"Plate control","PASS","PFC",0.784856061149354,"1h","1h"
5019,"PFC_23",75,"OID30234","O95980","RECK",0,"Cardiometabolic_II","B13805","20212097_SS211647","PASS",-3.9135,-0.1019,"Plate control","PASS","PFC",0.931805014868271,"24h","24h"
5020,"PFC_08",85,"OID30234","O95980","RECK",0,"Cardiometabolic_II","B13805","20212097_SS211647","PASS",-3.9135,0.0471,"Plate control","PASS","PFC",1.03318600019851,"Baseline","Baseline"
5021,"PFC_16",86,"OID30234","O95980","RECK",0,"Cardiometabolic_II","B13805","20212097_SS211647","PASS",-3.9135,0.0101,"Plate control","PASS","PFC",1.0070253493158,"1h","1h"
5022,"PFC_24",87,"OID30234","O95980","RECK",0,"Cardiometabolic_II","B13805","20212097_SS211647","PASS",-3.9135,0.0308,"Plate control","PASS","PFC",1.02157845205269,"24h","24h"
5023,"PFC_01",1,"OID30235","Q8NC42","RNF149",0,"Cardiometabolic_II","B13805","20212097_SS211647","PASS",-3.0103,-0.0361,"Plate control","PASS","PFC",0.97528785738183,"Baseline","Baseline"
5024,"PFC_09",2,"OID30235","Q8NC42","RNF149",0,"Cardiometabolic_II","B13805","20212097_SS211647","PASS",-3.0103,-0.1699,"Plate control","PASS","PFC",0.888904293181696,"Baseline","Baseline"
5025,"PFC_17",3,"OID30235","Q8NC42","RNF149",0,"Cardiometabolic_II","B13805","20212097_SS211647","PASS",-3.0103,-0.8492,"Plate control","PASS","PFC",0.55509245932553,"1h","1h"
5026,"PFC_25",4,"OID30235","Q8NC42","RNF149",0,"Cardiometabolic_II","B13805","20212097_SS211647","PASS",-3.0103,-0.2122,"Plate control","PASS","PFC",0.863219882593732,"24h","24h"
5027,"PFC_02",13,"OID30235","Q8NC42","RNF149",0,"Cardiometabolic_II","B13805","20212097_SS211647","PASS",-3.0103,0.383,"Plate control","PASS","PFC",1.30405073525856,"Baseline","Baseline"
5028,"PFC_10",14,"OID30235","Q8NC42","RNF149",0,"Cardiometabolic_II","B13805","20212097_SS211647","PASS",-3.0103,0.137,"Plate control","PASS","PFC",1.09961614931455,"1h","1h"
5029,"PFC_18",15,"OID30235","Q8NC42","RNF149",0,"Cardiometabolic_II","B13805","20212097_SS211647","PASS",-3.0103,-0.3893,"Plate control","PASS","PFC",0.763499967117173,"1h","1h"
5030,"PFC_26",16,"OID30235","Q8NC42","RNF149",0,"Cardiometabolic_II","B13805","20212097_SS211647","PASS",-3.0103,-0.4665,"Plate control","PASS","PFC",0.723718221098446,"24h","24h"
5031,"PFC_03",25,"OID30235","Q8NC42","RNF149",0,"Cardiometabolic_II","B13805","20212097_SS211647","PASS",-3.0103,-0.4142,"Plate control","PASS","PFC",0.750435509094408,"Baseline","Baseline"
5032,"PFC_11",26,"OID30235","Q8NC42","RNF149",0,"Cardiometabolic_II","B13805","20212097_SS211647","PASS",-3.0103,0.4172,"Plate control","PASS","PFC",1.33533340698243,"1h","1h"
5033,"PFC_19",27,"OID30235","Q8NC42","RNF149",0,"Cardiometabolic_II","B13805","20212097_SS211647","PASS",-3.0103,0.0071,"Plate control","PASS","PFC",1.00493347469019,"24h","24h"
5034,"PFC_27",28,"OID30235","Q8NC42","RNF149",0,"Cardiometabolic_II","B13805","20212097_SS211647","PASS",-3.0103,-0.0022,"Plate control","PASS","PFC",0.998476238308279,"24h","24h"
5035,"PFC_04",37,"OID30235","Q8NC42","RNF149",0,"Cardiometabolic_II","B13805","20212097_SS211647","PASS",-3.0103,-0.3122,"Plate control","PASS","PFC",0.805412629410481,"Baseline","Baseline"
5036,"PFC_12",38,"OID30235","Q8NC42","RNF149",0,"Cardiometabolic_II","B13805","20212097_SS211647","PASS",-3.0103,0.0373,"Plate control","PASS","PFC",1.02619151367835,"1h","1h"
5037,"PFC_20",39,"OID30235","Q8NC42","RNF149",0,"Cardiometabolic_II","B13805","20212097_SS211647","PASS",-3.0103,0.1393,"Plate control","PASS","PFC",1.10137059786496,"24h","24h"
5038,"PFC_05",49,"OID30235","Q8NC42","RNF149",0,"Cardiometabolic_II","B13805","20212097_SS211647","PASS",-3.0103,-0.1846,"Plate control","PASS","PFC",0.879893000246708,"Baseline","Baseline"
5039,"PFC_13",50,"OID30235","Q8NC42","RNF149",0,"Cardiometabolic_II","B13805","20212097_SS211647","PASS",-3.0103,-0.3677,"Plate control","PASS","PFC",0.775017074474182,"1h","1h"
5040,"PFC_21",51,"OID30235","Q8NC42","RNF149",0,"Cardiometabolic_II","B13805","20212097_SS211647","PASS",-3.0103,-0.0513,"Plate control","PASS","PFC",0.965066324104723,"24h","24h"

Supplemental table 1_Olink proximity extension assay (PEA) data

,"SampleID","Index","OlinkID","UniProt","Assay","MissingFreq","Panel","Panel_Lot_Nr","PlateID","QC_Warning","LOD","NPX","Normalization","Assay_Warning","condition","Value","group","alt_group"
5041,"PFC_06",61,"OID30235","Q8NC42","RNF149",0,"Cardiometabolic_II","B13805","20212097_SS211647","PASS",-3.0103,-0.1098,"Plate control","PASS","PFC",0.926716523174951,"Baseline","Baseline"
5042,"PFC_14",62,"OID30235","Q8NC42","RNF149",0,"Cardiometabolic_II","B13805","20212097_SS211647","PASS",-3.0103,-0.1067,"Plate control","PASS","PFC",0.928709952034497,"1h","1h"
5043,"PFC_22",63,"OID30235","Q8NC42","RNF149",0,"Cardiometabolic_II","B13805","20212097_SS211647","PASS",-3.0103,-0.3386,"Plate control","PASS","PFC",0.790808344840889,"24h","24h"
5044,"PFC_07",73,"OID30235","Q8NC42","RNF149",0,"Cardiometabolic_II","B13805","20212097_SS211647","PASS",-3.0103,-0.4634,"Plate control","PASS","PFC",0.725274987112689,"Baseline","Baseline"
5045,"PFC_15",74,"OID30235","Q8NC42","RNF149",0,"Cardiometabolic_II","B13805","20212097_SS211647","PASS",-3.0103,-0.0845,"Plate control","PASS","PFC",0.943111336766714,"1h","1h"
5046,"PFC_23",75,"OID30235","Q8NC42","RNF149",0,"Cardiometabolic_II","B13805","20212097_SS211647","PASS",-3.0103,-0.2643,"Plate control","PASS","PFC",0.832602618030947,"24h","24h"
5047,"PFC_08",85,"OID30235","Q8NC42","RNF149",0,"Cardiometabolic_II","B13805","20212097_SS211647","PASS",-3.0103,-0.491,"Plate control","PASS","PFC",0.711531730617298,"Baseline","Baseline"
5048,"PFC_16",86,"OID30235","Q8NC42","RNF149",0,"Cardiometabolic_II","B13805","20212097_SS211647","PASS",-3.0103,-0.2416,"Plate control","PASS","PFC",0.845806762299923,"1h","1h"
5049,"PFC_24",87,"OID30235","Q8NC42","RNF149",0,"Cardiometabolic_II","B13805","20212097_SS211647","PASS",-3.0103,0.108,"Plate control","PASS","PFC",1.07773314504367,"24h","24h"
5050,"PFC_01",1,"OID30236","Q9H7C9","AAMDC",0,"Cardiometabolic_II","B13805","20212097_SS211647","PASS",-0.3951,0.9277,"Plate control","PASS","PFC",1.90224095164785,"Baseline","Baseline"
5051,"PFC_09",2,"OID30236","Q9H7C9","AAMDC",0,"Cardiometabolic_II","B13805","20212097_SS211647","PASS",-0.3951,1.5512,"Plate control","PASS","PFC",2.93060798959665,"Baseline","Baseline"
5052,"PFC_17",3,"OID30236","Q9H7C9","AAMDC",0,"Cardiometabolic_II","B13805","20212097_SS211647","PASS",-0.3951,1.5688,"Plate control","PASS","PFC",2.9665785840586,"1h","1h"
5053,"PFC_25",4,"OID30236","Q9H7C9","AAMDC",0,"Cardiometabolic_II","B13805","20212097_SS211647","PASS",-0.3951,2.1767,"Plate control","PASS","PFC",4.52118201737145,"24h","24h"
5054,"PFC_02",13,"OID30236","Q9H7C9","AAMDC",0,"Cardiometabolic_II","B13805","20212097_SS211647","PASS",-0.3951,1.0948,"Plate control","PASS","PFC",2.13583470601047,"Baseline","Baseline"
5055,"PFC_10",14,"OID30236","Q9H7C9","AAMDC",0,"Cardiometabolic_II","B13805","20212097_SS211647","PASS",-0.3951,1.2783,"Plate control","PASS","PFC",2.42552996046225,"1h","1h"
5056,"PFC_18",15,"OID30236","Q9H7C9","AAMDC",0,"Cardiometabolic_II","B13805","20212097_SS211647","PASS",-0.3951,1.7838,"Plate control","PASS","PFC",3.44331937484373,"1h","1h"
5057,"PFC_26",16,"OID30236","Q9H7C9","AAMDC",0,"Cardiometabolic_II","B13805","20212097_SS211647","PASS",-0.3951,1.4073,"Plate control","PASS","PFC",2.65240301495677,"24h","24h"
5058,"PFC_03",25,"OID30236","Q9H7C9","AAMDC",0,"Cardiometabolic_II","B13805","20212097_SS211647","PASS",-0.3951,0.6609,"Plate control","PASS","PFC",1.58106863808278,"Baseline","Baseline"
5059,"PFC_11",26,"OID30236","Q9H7C9","AAMDC",0,"Cardiometabolic_II","B13805","20212097_SS211647","PASS",-0.3951,2.1325,"Plate control","PASS","PFC",4.38476644673688,"1h","1h"
5060,"PFC_19",27,"OID30236","Q9H7C9","AAMDC",0,"Cardiometabolic_II","B13805","20212097_SS211647","PASS",-0.3951,1.7278,"Plate control","PASS","PFC",3.31222344184134,"24h","24h"
5061,"PFC_27",28,"OID30236","Q9H7C9","AAMDC",0,"Cardiometabolic_II","B13805","20212097_SS211647","PASS",-0.3951,2.7542,"Plate control","PASS","PFC",6.74678412005177,"24h","24h"
5062,"PFC_04",37,"OID30236","Q9H7C9","AAMDC",0,"Cardiometabolic_II","B13805","20212097_SS211647","PASS",-0.3951,1.9012,"Plate control","PASS","PFC",3.73523755764715,"Baseline","Baseline"
5063,"PFC_12",38,"OID30236","Q9H7C9","AAMDC",0,"Cardiometabolic_II","B13805","20212097_SS211647","PASS",-0.3951,1.0016,"Plate control","PASS","PFC",2.00221930139232,"1h","1h"
5064,"PFC_20",39,"OID30236","Q9H7C9","AAMDC",0,"Cardiometabolic_II","B13805","20212097_SS211647","PASS",-0.3951,2.0485,"Plate control","PASS","PFC",4.13675638709411,"24h","24h"
5065,"PFC_05",49,"OID30236","Q9H7C9","AAMDC",0,"Cardiometabolic_II","B13805","20212097_SS211647","PASS",-0.3951,0.92,"Plate control","PASS","PFC",1.89211529345119,"Baseline","Baseline"
5066,"PFC_13",50,"OID30236","Q9H7C9","AAMDC",0,"Cardiometabolic_II","B13805","20212097_SS211647","PASS",-0.3951,2.0295,"Plate control","PASS","PFC",4.08263332472211,"1h","1h"
5067,"PFC_21",51,"OID30236","Q9H7C9","AAMDC",0,"Cardiometabolic_II","B13805","20212097_SS211647","PASS",-0.3951,1.4006,"Plate control","PASS","PFC",2.64011358562928,"24h","24h"
5068,"PFC_06",61,"OID30236","Q9H7C9","AAMDC",0,"Cardiometabolic_II","B13805","20212097_SS211647","PASS",-0.3951,1.8195,"Plate control","PASS","PFC",3.52958851100048,"Baseline","Baseline"
5069,"PFC_14",62,"OID30236","Q9H7C9","AAMDC",0,"Cardiometabolic_II","B13805","20212097_SS211647","PASS",-0.3951,1.7586,"Plate control","PASS","PFC",3.3836960965689,"1h","1h"
5070,"PFC_22",63,"OID30236","Q9H7C9","AAMDC",0,"Cardiometabolic_II","B13805","20212097_SS211647","PASS",-0.3951,1.5831,"Plate control","PASS","PFC",2.99612953752519,"24h","24h"
5071,"PFC_07",73,"OID30236","Q9H7C9","AAMDC",0,"Cardiometabolic_II","B13805","20212097_SS211647","PASS",-0.3951,2.4828,"Plate control","PASS","PFC",5.58981292107571,"Baseline","Baseline"
5072,"PFC_15",74,"OID30236","Q9H7C9","AAMDC",0,"Cardiometabolic_II","B13805","20212097_SS211647","PASS",-0.3951,0.855,"Plate control","PASS","PFC",1.80875875512218,"1h","1h"
5073,"PFC_23",75,"OID30236","Q9H7C9","AAMDC",0,"Cardiometabolic_II","B13805","20212097_SS211647","PASS",-0.3951,1.9109,"Plate control","PASS","PFC",3.7604361474314,"24h","24h"
5074,"PFC_08",85,"OID30236","Q9H7C9","AAMDC",0,"Cardiometabolic_II","B13805","20212097_SS211647","PASS",-0.3951,1.0679,"Plate control","PASS","PFC",2.09637963603427,"Baseline","Baseline"
5075,"PFC_16",86,"OID30236","Q9H7C9","AAMDC",0,"Cardiometabolic_II","B13805","20212097_SS211647","PASS",-0.3951,1.9472,"Plate control","PASS","PFC",3.85625378419692,"1h","1h"
5076,"PFC_24",87,"OID30236","Q9H7C9","AAMDC",0,"Cardiometabolic_II","B13805","20212097_SS211647","PASS",-0.3951,2.2352,"Plate control","PASS","PFC",4.70827960978238,"24h","24h"
5077,"PFC_01",1,"OID30237","Q8TAE8","GADD45GIP1",0,"Cardiometabolic_II","B13805","20212097_SS211647","PASS",-0.1898,0.6306,"Plate control","PASS","PFC",1.54820874158396,"Baseline","Baseline"
5078,"PFC_09",2,"OID30237","Q8TAE8","GADD45GIP1",0,"Cardiometabolic_II","B13805","20212097_SS211647","PASS",-0.1898,1.5078,"Plate control","PASS","PFC",2.84376056492387,"Baseline","Baseline"
5079,"PFC_17",3,"OID30237","Q8TAE8","GADD45GIP1",0,"Cardiometabolic_II","B13805","20212097_SS211647","PASS",-0.1898,2.7447,"Plate control","PASS","PFC",6.70250318606701,"1h","1h"
5080,"PFC_25",4,"OID30237","Q8TAE8","GADD45GIP1",0,"Cardiometabolic_II","B13805","20212097_SS211647","PASS",-0.1898,1.8942,"Plate control","PASS","PFC",3.71715796880006,"24h","24h"
5081,"PFC_02",13,"OID30237","Q8TAE8","GADD45GIP1",0,"Cardiometabolic_II","B13805","20212097_SS211647","PASS",-0.1898,1.1303,"Plate control","PASS","PFC",2.1890425537991,"Baseline","Baseline"
5082,"PFC_10",14,"OID30237","Q8TAE8","GADD45GIP1",0,"Cardiometabolic_II","B13805","20212097_SS211647","PASS",-0.1898,0.7104,"Plate control","PASS","PFC",1.63625772120023,"1h","1h"
5083,"PFC_18",15,"OID30237","Q8TAE8","GADD45GIP1",0,"Cardiometabolic_II","B13805","20212097_SS211647","PASS",-0.1898,1.8445,"Plate control","PASS","PFC",3.59128463142195,"1h","1h"
5084,"PFC_26",16,"OID30237","Q8TAE8","GADD45GIP1",0,"Cardiometabolic_II","B13805","20212097_SS211647","PASS",-0.1898,2.4022,"Plate control","PASS","PFC",5.28608637901505,"24h","24h"
5085,"PFC_03",25,"OID30237","Q8TAE8","GADD45GIP1",0,"Cardiometabolic_II","B13805","20212097_SS211647","PASS",-0.1898,0.3228,"Plate control","PASS","PFC",1.25075567653997,"Baseline","Baseline"
5086,"PFC_11",26,"OID30237","Q8TAE8","GADD45GIP1",0,"Cardiometabolic_II","B13805","20212097_SS211647","PASS",-0.1898,1.9282,"Plate control","PASS","PFC",3.80580066475883,"1h","1h"
5087,"PFC_19",27,"OID30237","Q8TAE8","GADD45GIP1",0,"Cardiometabolic_II","B13805","20212097_SS211647","PASS",-0.1898,1.5437,"Plate control","PASS","PFC",2.91541245162014,"24h","24h"
5088,"PFC_27",28,"OID30237","Q8TAE8","GADD45GIP1",0,"Cardiometabolic_II","B13805","20212097_SS211647","PASS",-0.1898,2.315,"Plate control","PASS","PFC",4.97604661319387,"24h","24h"
5089,"PFC_04",37,"OID30237","Q8TAE8","GADD45GIP1",0,"Cardiometabolic_II","B13805","20212097_SS211647","PASS",-0.1898,1.2922,"Plate control","PASS","PFC",2.44901226645832,"Baseline","Baseline"
5090,"PFC_12",38,"OID30237","Q8TAE8","GADD45GIP1",0,"Cardiometabolic_II","B13805","20212097_SS211647","PASS",-0.1898,0.671,"Plate control","PASS","PFC",1.59217619755756,"1h","1h"
5091,"PFC_20",39,"OID30237","Q8TAE8","GADD45GIP1",0,"Cardiometabolic_II","B13805","20212097_SS211647","PASS",-0.1898,2.4086,"Plate control","PASS","PFC",5.30958829896653,"24h","24h"
5092,"PFC_05",49,"OID30237","Q8TAE8","GADD45GIP1",0,"Cardiometabolic_II","B13805","20212097_SS211647","PASS",-0.1898,0.6196,"Plate control","PASS","PFC",1.53644912807475,"Baseline","Baseline"
5093,"PFC_13",50,"OID30237","Q8TAE8","GADD45GIP1",0,"Cardiometabolic_II","B13805","20212097_SS211647","PASS",-0.1898,1.5897,"Plate control","PASS","PFC",3.00986754538224,"1h","1h"
5094,"PFC_21",51,"OID30237","Q8TAE8","GADD45GIP1",0,"Cardiometabolic_II","B13805","20212097_SS211647","PASS",-0.1898,1.1903,"Plate control","PASS","PFC",2.28200191135977,"24h","24h"
5095,"PFC_06",61,"OID30237","Q8TAE8","GADD45GIP1",0,"Cardiometabolic_II","B13805","20212097_SS211647","PASS",-0.1898,1.2419,"Plate control","PASS","PFC",2.36509805870145,"Baseline","Baseline"
5096,"PFC_14",62,"OID30237","Q8TAE8","GADD45GIP1",0,"Cardiometabolic_II","B13805","20212097_SS211647","PASS",-0.1898,1.7745,"Plate control","PASS","PFC",3.42119420168376,"1h","1h"
5097,"PFC_22",63,"OID30237","Q8TAE8","GADD45GIP1",0,"Cardiometabolic_II","B13805","20212097_SS211647","PASS",-0.1898,1.029,"Plate control","PASS","PFC",2.04060931849692,"24h","24h"
5098,"PFC_07",73,"OID30237","Q8TAE8","GADD45GIP1",0,"Cardiometabolic_II","B13805","20212097_SS211647","PASS",-0.1898,2.0384,"Plate control","PASS","PFC",4.10789697588521,"Baseline","Baseline"
5099,"PFC_15",74,"OID30237","Q8TAE8","GADD45GIP1",0,"Cardiometabolic_II","B13805","20212097_SS211647","PASS",-0.1898,0.3832,"Plate control","PASS","PFC",1.30423152760793,"1h","1h"
5100,"PFC_23",75,"OID30237","Q8TAE8","GADD45GIP1",0,"Cardiometabolic_II","B13805","20212097_SS211647","PASS",-0.1898,1.3923,"Plate control","PASS","PFC",2.62496829924773,"24h","24h"
5101,"PFC_08",85,"OID30237","Q8TAE8","GADD45GIP1",0,"Cardiometabolic_II","B13805","20212097_SS211647","PASS",-0.1898,1.3098,"Plate control","PASS","PFC",2.47907170374295,"Baseline","Baseline"
5102,"PFC_16",86,"OID30237","Q8TAE8","GADD45GIP1",0,"Cardiometabolic_II","B13805","20212097_SS211647","PASS",-0.1898,1.5214,"Plate control","PASS","PFC",2.87069488451106,"1h","1h"
5103,"PFC_24",87,"OID30237","Q8TAE8","GADD45GIP1",0,"Cardiometabolic_II","B13805","20212097_SS211647","PASS",-0.1898,1.8905,"Plate control","PASS","PFC",3.70763699398349,"24h","24h"
5104,"PFC_01",1,"OID30238","Q5GAN6","RNASE10",0,"Cardiometabolic_II","B13805","20212097_SS211647","PASS",-1.6729,0.7402,"Plate control","PASS","PFC",1.67040739044105,"Baseline","Baseline"
5105,"PFC_09",2,"OID30238","Q5GAN6","RNASE10",0,"Cardiometabolic_II","B13805","20212097_SS211647","PASS",-1.6729,0.4556,"Plate control","PASS","PFC",1.37135301608541,"Baseline","Baseline"
5106,"PFC_17",3,"OID30238","Q5GAN6","RNASE10",0,"Cardiometabolic_II","B13805","20212097_SS211647","PASS",-1.6729,0.288,"Plate control","PASS","PFC",1.22094651266913,"1h","1h"
5107,"PFC_25",4,"OID30238","Q5GAN6","RNASE10",0,"Cardiometabolic_II","B13805","20212097_SS211647","PASS",-1.6729,0.7191,"Plate control","PASS","PFC",1.64615478935811,"24h","24h"
5108,"PFC_02",13,"OID30238","Q5GAN6","RNASE10",0,"Cardiometabolic_II","B13805","20212097_SS211647","PASS",-1.6729,1.0143,"Plate control","PASS","PFC",2.01992258261782,"Baseline","Baseline"
5109,"PFC_10",14,"OID30238","Q5GAN6","RNASE10",0,"Cardiometabolic_II","B13805","20212097_SS211647","PASS",-1.6729,0.7327,"Plate control","PASS","PFC",1.66174613686234,"1h","1h"
5110,"PFC_18",15,"OID30238","Q5GAN6","RNASE10",0,"Cardiometabolic_II","B13805","20212097_SS211647","PASS",-1.6729,0.061,"Plate control","PASS","PFC",1.04318859352986,"1h","1h"
5111,"PFC_26",16,"OID30238","Q5GAN6","RNASE10",0,"Cardiometabolic_II","B13805","20212097_SS211647","PASS",-1.6729,0.6309,"Plate control","PASS","PFC",1.54853071601639,"24h","24h"
5112,"PFC_03",25,"OID30238","Q5GAN6","RNASE10",0,"Cardiometabolic_II","B13805","20212097_SS211647","PASS",-1.6729,0.7027,"Plate control","PASS","PFC",1.62754789587964,"Baseline","Baseline"
5113,"PFC_11",26,"OID30238","Q5GAN6","RNASE10",0,"Cardiometabolic_II","B13805","20212097_SS211647","PASS",-1.6729,0.9364,"Plate control","PASS","PFC",1.91374684592553,"1h","1h"
5114,"PFC_19",27,"OID30238","Q5GAN6","RNASE10",0,"Cardiometabolic_II","B13805","20212097_SS211647","PASS",-1.6729,0.6629,"Plate control","PASS","PFC",1.58326198458097,"24h","24h"
5115,"PFC_27",28,"OID30238","Q5GAN6","RNASE10",0,"Cardiometabolic_II","B13805","20212097_SS211647","PASS",-1.6729,0.5009,"Plate control","PASS","PFC",1.41509606994203,"24h","24h"
5116,"PFC_04",37,"OID30238","Q5GAN6","RNASE10",0,"Cardiometabolic_II","B13805","20212097_SS211647","PASS",-1.6729,0.1645,"Plate control","PASS","PFC",1.12077757880122,"Baseline","Baseline"
5117,"PFC_12",38,"OID30238","Q5GAN6","RNASE10",0,"Cardiometabolic_II","B13805","20212097_SS211647","PASS",-1.6729,0.9456,"Plate control","PASS","PFC",1.925989716269,"1h","1h"
5118,"PFC_20",39,"OID30238","Q5GAN6","RNASE10",0,"Cardiometabolic_II","B13805","20212097_SS211647","PASS",-1.6729,0.8059,"Plate control","PASS","PFC",1.74823605813146,"24h","24h"
5119,"PFC_05",49,"OID30238","Q5GAN6","RNASE10",0,"Cardiometabolic_II","B13805","20212097_SS211647","PASS",-1.6729,0.5737,"Plate control","PASS","PFC",1.48833573227095,"Baseline","Baseline"
5120,"PFC_13",50,"OID30238","Q5GAN6","RNASE10",0,"Cardiometabolic_II","B13805","20212097_SS211647","PASS",-1.6729,-0.4308,"Plate control","PASS","PFC",0.741850302081463,"1h","1h"
5121,"PFC_21",51,"OID30238","Q5GAN6","RNASE10",0,"Cardiometabolic_II","B13805","20212097_SS211647","PASS",-1.6729,0.8089,"Plate control","PASS","PFC",1.75187519519046,"24h","24h"
5122,"PFC_06",61,"OID30238","Q5GAN6","RNASE10",0,"Cardiometabolic_II","B13805","20212097_SS211647","PASS",-1.6729,1.1988,"Plate control","PASS","PFC",2.29548658364082,"Baseline","Baseline"
5123,"PFC_14",62,"OID30238","Q5GAN6","RNASE10",0,"Cardiometabolic_II","B13805","20212097_SS211647","PASS",-1.6729,0.4285,"Plate control","PASS","PFC",1.34583355807308,"1h","1h"
5124,"PFC_22",63,"OID30238","Q5GAN6","RNASE10",0,"Cardiometabolic_II","B13805","20212097_SS211647","PASS",-1.6729,-0.3145,"Plate control","PASS","PFC",0.80412963254922,"24h","24h"
5125,"PFC_07",73,"OID30238","Q5GAN6","RNASE10",0,"Cardiometabolic_II","B13805","20212097_SS211647","PASS",-1.6729,0.6968,"Plate control","PASS","PFC",1.62090551897643,"Baseline","Baseline"
5126,"PFC_15",74,"OID30238","Q5GAN6","RNASE10",0,"Cardiometabolic_II","B13805","20212097_SS211647","PASS",-1.6729,0.8433,"Plate control","PASS","PFC",1.79414936340834,"1h","1h"
5127,"PFC_23",75,"OID30238","Q5GAN6","RNASE10",0,"Cardiometabolic_II","B13805","20212097_SS211647","PASS",-1.6729,0.6705,"Plate control","PASS","PFC",1.5916244869461,"24h","24h"
5128,"PFC_08",85,"OID30238","Q5GAN6","RNASE10",0,"Cardiometabolic_II","B13805","20212097_SS211647","PASS",-1.6729,0.2197,"Plate control","PASS","PFC",1.16449141210823,"Baseline","Baseline"
5129,"PFC_16",86,"OID30238","Q5GAN6","RNASE10",0,"Cardiometabolic_II","B13805","20212097_SS211647","PASS",-1.6729,0.6879,"Plate control","PASS","PFC",1.61093691658933,"1h","1h"
5130,"PFC_24",87,"OID30238","Q5GAN6","RNASE10",0,"Cardiometabolic_II","B13805","20212097_SS211647","PASS",-1.6729,1.5452,"Plate control","PASS","PFC",2.91844524285647,"24h","24h"
5131,"PFC_01",1,"OID30239","P30084","ECHS1",0,"Cardiometabolic_II","B13805","20212097_SS211647","PASS",-1.2919,1.5126,"Plate control","PASS","PFC",2.85323781625876,"Baseline","Baseline"
5132,"PFC_09",2,"OID30239","P30084","ECHS1",0,"Cardiometabolic_II","B13805","20212097_SS211647","PASS",-1.2919,1.9316,"Plate control","PASS","PFC",3.81478037384663,"Baseline","Baseline"
5133,"PFC_17",3,"OID30239","P30084","ECHS1",0,"Cardiometabolic_II","B13805","20212097_SS211647","PASS",-1.2919,4.5396,"Plate control","PASS","PFC",23.2571111427198,"1h","1h"
5134,"PFC_25",4,"OID30239","P30084","ECHS1",0,"Cardiometabolic_II","B13805","20212097_SS211647","PASS",-1.2919,3.4123,"Plate control","PASS","PFC",10.6464459646822,"24h","24h"
5135,"PFC_02",13,"OID30239","P30084","ECHS1",0,"Cardiometabolic_II","B13805","20212097_SS211647","PASS",-1.2919,2.3001,"Plate control","PASS","PFC",4.92491901092167,"Baseline","Baseline"
5136,"PFC_10",14,"OID30239","P30084","ECHS1",0,"Cardiometabolic_II","B13805","20212097_SS211647","PASS",-1.2919,1.1161,"Plate control","PASS","PFC",2.16760217521616,"1h","1h"
5137,"PFC_18",15,"OID30239","P30084","ECHS1",0,"Cardiometabolic_II","B13805","20212097_SS211647","PASS",-1.2919,2.2369,"Plate control","PASS","PFC",4.71383088206404,"1h","1h"
5138,"PFC_26",16,"OID30239","P30084","ECHS1",0,"Cardiometabolic_II","B13805","20212097_SS211647","PASS",-1.2919,3.5369,"Plate control","PASS","PFC",11.6068131118118,"24h","24h"
5139,"PFC_03",25,"OID30239","P30084","ECHS1",0,"Cardiometabolic_II","B13805","20212097_SS211647","PASS",-1.2919,0.8779,"Plate control","PASS","PFC",1.83769838299807,"Baseline","Baseline"
5140,"PFC_11",26,"OID30239","P30084","ECHS1",0,"Cardiometabolic_II","B13805","20212097_SS211647","PASS",-1.2919,2.7539,"Plate control","PASS","PFC",6.74538131159254,"1h","1h"
5141,"PFC_19",27,"OID30239","P30084","ECHS1",0,"Cardiometabolic_II","B13805","20212097_SS211647","PASS",-1.2919,2.9005,"Plate control","PASS","PFC",7.46685129737458,"24h","24h"
5142,"PFC_27",28,"OID30239","P30084","ECHS1",0,"Cardiometabolic_II","B13805","20212097_SS211647","PASS",-1.2919,2.9969,"Plate control","PASS","PFC",7.98282840531489,"24h","24h"
5143,"PFC_04",37,"OID30239","P30084","ECHS1",0,"Cardiometabolic_II","B13805","20212097_SS211647","PASS",-1.2919,1.9587,"Plate control","PASS","PFC",3.88711556492035,"Baseline","Baseline"
5144,"PFC_12",38,"OID30239","P30084","ECHS1",0,"Cardiometabolic_II","B13805","20212097_SS211647","PASS",-1.2919,1.3387,"Plate control","PASS","PFC",2.52923309078533,"1h","1h"
5145,"PFC_20",39,"OID30239","P30084","ECHS1",0,"Cardiometabolic_II","B13805","20212097_SS211647","PASS",-1.2919,3.3381,"Plate control","PASS","PFC",10.1127257240619,"24h","24h"
5146,"PFC_05",49,"OID30239","P30084","ECHS1",0,"Cardiometabolic_II","B13805","20212097_SS211647","PASS",-1.2919,1.8667,"Plate control","PASS","PFC",3.64697421625595,"Baseline","Baseline"
5147,"PFC_13",50,"OID30239","P30084","ECHS1",0,"Cardiometabolic_II","B13805","20212097_SS211647","PASS",-1.2919,2.5274,"Plate control","PASS","PFC",5.76531725913632,"1h","1h"
5148,"PFC_21",51,"OID30239","P30084","ECHS1",0,"Cardiometabolic_II","B13805","20212097_SS211647","PASS",-1.2919,2.2712,"Plate control","PASS","PFC",4.82724483072877,"24h","24h"
5149,"PFC_06",61,"OID30239","P30084","ECHS1",0,"Cardiometabolic_II","B13805","20212097_SS211647","PASS",-1.2919,2.3897,"Plate control","PASS","PFC",5.24048377415847,"Baseline","Baseline"
5150,"PFC_14",62,"OID30239","P30084","ECHS1",0,"Cardiometabolic_II","B13805","20212097_SS211647","PASS",-1.2919,2.8141,"Plate control","PASS","PFC",7.03280394722277,"1h","1h"
5151,"PFC_22",63,"OID30239","P30084","ECHS1",0,"Cardiometabolic_II","B13805","20212097_SS211647","PASS",-1.2919,1.8487,"Plate control","PASS","PFC",3.60175487764344,"24h","24h"
5152,"PFC_07",73,"OID30239","P30084","ECHS1",0,"Cardiometabolic_II","B13805","20212097_SS211647","PASS",-1.2919,3.1026,"Plate control","PASS","PFC",8.58965388502751,"Baseline","Baseline"

Supplemental table 1_Olink proximity extension assay (PEA) data

,"SampleID","Index","OlinkID","UniProt","Assay","MissingFreq","Panel","Panel_Lot_Nr","PlateID","QC_Warning","LOD","NPX","Normalization","Assay_Warning","condition","Value","group","alt_group"
5153,"PFC_15",74,"OID30239","P30084","ECHS1",0,"Cardiometabolic_II","B13805","20212097_SS211647","PASS",-1.2919,0.856,"Plate control","PASS","PFC",1.81001292576581,"1h","1h"
5154,"PFC_23",75,"OID30239","P30084","ECHS1",0,"Cardiometabolic_II","B13805","20212097_SS211647","PASS",-1.2919,2.8494,"Plate control","PASS","PFC",7.20700576809019,"24h","24h"
5155,"PFC_08",85,"OID30239","P30084","ECHS1",0,"Cardiometabolic_II","B13805","20212097_SS211647","PASS",-1.2919,1.9466,"Plate control","PASS","PFC",3.85465034678264,"Baseline","Baseline"
5156,"PFC_16",86,"OID30239","P30084","ECHS1",0,"Cardiometabolic_II","B13805","20212097_SS211647","PASS",-1.2919,2.4361,"Plate control","PASS","PFC",5.41176802637583,"1h","1h"
5157,"PFC_24",87,"OID30239","P30084","ECHS1",0,"Cardiometabolic_II","B13805","20212097_SS211647","PASS",-1.2919,3.1063,"Plate control","PASS","PFC",8.61171156717253,"24h","24h"
5158,"PFC_01",1,"OID30240","Q5SW79","CEP170",0,"Cardiometabolic_II","B13805","20212097_SS211647","PASS",-1.8553,1.0162,"Plate control","PASS","PFC",2.02258453202153,"Baseline","Baseline"
5159,"PFC_09",2,"OID30240","Q5SW79","CEP170",0,"Cardiometabolic_II","B13805","20212097_SS211647","PASS",-1.8553,3.1665,"Plate control","PASS","PFC",8.97865907118839,"Baseline","Baseline"
5160,"PFC_17",3,"OID30240","Q5SW79","CEP170",0,"Cardiometabolic_II","B13805","20212097_SS211647","PASS",-1.8553,3.7941,"Plate control","PASS","PFC",13.8719625140822,"1h","1h"
5161,"PFC_25",4,"OID30240","Q5SW79","CEP170",0,"Cardiometabolic_II","B13805","20212097_SS211647","PASS",-1.8553,3.7935,"Plate control","PASS","PFC",13.8661945265613,"24h","24h"
5162,"PFC_02",13,"OID30240","Q5SW79","CEP170",0,"Cardiometabolic_II","B13805","20212097_SS211647","PASS",-1.8553,1.9785,"Plate control","PASS","PFC",3.9408313229968,"Baseline","Baseline"
5163,"PFC_10",14,"OID30240","Q5SW79","CEP170",0,"Cardiometabolic_II","B13805","20212097_SS211647","PASS",-1.8553,2.3584,"Plate control","PASS","PFC",5.12801328673806,"1h","1h"
5164,"PFC_18",15,"OID30240","Q5SW79","CEP170",0,"Cardiometabolic_II","B13805","20212097_SS211647","PASS",-1.8553,3.0731,"Plate control","PASS","PFC",8.41579755204901,"1h","1h"
5165,"PFC_26",16,"OID30240","Q5SW79","CEP170",0,"Cardiometabolic_II","B13805","20212097_SS211647","PASS",-1.8553,3.6942,"Plate control","PASS","PFC",12.9438958543983,"24h","24h"
5166,"PFC_03",25,"OID30240","Q5SW79","CEP170",0,"Cardiometabolic_II","B13805","20212097_SS211647","PASS",-1.8553,1.7341,"Plate control","PASS","PFC",3.32671897609603,"Baseline","Baseline"
5167,"PFC_11",26,"OID30240","Q5SW79","CEP170",0,"Cardiometabolic_II","B13805","20212097_SS211647","PASS",-1.8553,3.6557,"Plate control","PASS","PFC",12.6030411755508,"1h","1h"
5168,"PFC_19",27,"OID30240","Q5SW79","CEP170",0,"Cardiometabolic_II","B13805","20212097_SS211647","PASS",-1.8553,2.8167,"Plate control","PASS","PFC",7.04548977226767,"24h","24h"
5169,"PFC_27",28,"OID30240","Q5SW79","CEP170",0,"Cardiometabolic_II","B13805","20212097_SS211647","PASS",-1.8553,3.9202,"Plate control","PASS","PFC",15.1390209160774,"24h","24h"
5170,"PFC_04",37,"OID30240","Q5SW79","CEP170",0,"Cardiometabolic_II","B13805","20212097_SS211647","PASS",-1.8553,3.5554,"Plate control","PASS","PFC",11.7566082383297,"Baseline","Baseline"
5171,"PFC_12",38,"OID30240","Q5SW79","CEP170",0,"Cardiometabolic_II","B13805","20212097_SS211647","PASS",-1.8553,1.9731,"Plate control","PASS","PFC",3.92610838307821,"1h","1h"
5172,"PFC_20",39,"OID30240","Q5SW79","CEP170",0,"Cardiometabolic_II","B13805","20212097_SS211647","PASS",-1.8553,3.804,"Plate control","PASS","PFC",13.9674814581402,"24h","24h"
5173,"PFC_05",49,"OID30240","Q5SW79","CEP170",0,"Cardiometabolic_II","B13805","20212097_SS211647","PASS",-1.8553,2.1694,"Plate control","PASS","PFC",4.49836273320856,"Baseline","Baseline"
5174,"PFC_13",50,"OID30240","Q5SW79","CEP170",0,"Cardiometabolic_II","B13805","20212097_SS211647","PASS",-1.8553,3.8968,"Plate control","PASS","PFC",14.8954520260307,"1h","1h"
5175,"PFC_21",51,"OID30240","Q5SW79","CEP170",0,"Cardiometabolic_II","B13805","20212097_SS211647","PASS",-1.8553,2.4343,"Plate control","PASS","PFC",5.40502016363822,"24h","24h"
5176,"PFC_06",61,"OID30240","Q5SW79","CEP170",0,"Cardiometabolic_II","B13805","20212097_SS211647","PASS",-1.8553,2.911,"Plate control","PASS","PFC",7.52139362007301,"Baseline","Baseline"
5177,"PFC_14",62,"OID30240","Q5SW79","CEP170",0,"Cardiometabolic_II","B13805","20212097_SS211647","PASS",-1.8553,3.3819,"Plate control","PASS","PFC",10.4244546017319,"1h","1h"
5178,"PFC_22",63,"OID30240","Q5SW79","CEP170",0,"Cardiometabolic_II","B13805","20212097_SS211647","PASS",-1.8553,3.4783,"Plate control","PASS","PFC",11.1448091023161,"24h","24h"
5179,"PFC_07",73,"OID30240","Q5SW79","CEP170",0,"Cardiometabolic_II","B13805","20212097_SS211647","PASS",-1.8553,3.9733,"Plate control","PASS","PFC",15.7066107799897,"Baseline","Baseline"
5180,"PFC_15",74,"OID30240","Q5SW79","CEP170",0,"Cardiometabolic_II","B13805","20212097_SS211647","PASS",-1.8553,2.3854,"Plate control","PASS","PFC",5.224887593991,"1h","1h"
5181,"PFC_23",75,"OID30240","Q5SW79","CEP170",0,"Cardiometabolic_II","B13805","20212097_SS211647","PASS",-1.8553,3.5546,"Plate control","PASS","PFC",11.7500907976323,"24h","24h"
5182,"PFC_08",85,"OID30240","Q5SW79","CEP170",0,"Cardiometabolic_II","B13805","20212097_SS211647","PASS",-1.8553,3.0662,"Plate control","PASS","PFC",8.37564328602408,"Baseline","Baseline"
5183,"PFC_16",86,"OID30240","Q5SW79","CEP170",0,"Cardiometabolic_II","B13805","20212097_SS211647","PASS",-1.8553,4.2215,"Plate control","PASS","PFC",18.6551234230204,"1h","1h"
5184,"PFC_24",87,"OID30240","Q5SW79","CEP170",0,"Cardiometabolic_II","B13805","20212097_SS211647","PASS",-1.8553,4.0864,"Plate control","PASS","PFC",16.9874806200408,"24h","24h"
5185,"PFC_01",1,"OID30241","P50053","KHK",0,"Cardiometabolic_II","B13805","20212097_SS211647","PASS",-3.5085,3.3234,"Plate control","PASS","PFC",10.0102076750777,"Baseline","Baseline"
5186,"PFC_09",2,"OID30241","P50053","KHK",0,"Cardiometabolic_II","B13805","20212097_SS211647","PASS",-3.5085,3.5122,"Plate control","PASS","PFC",11.409787361669,"Baseline","Baseline"
5187,"PFC_17",3,"OID30241","P50053","KHK",0,"Cardiometabolic_II","B13805","20212097_SS211647","PASS",-3.5085,3.9025,"Plate control","PASS","PFC",14.9544194589651,"1h","1h"
5188,"PFC_25",4,"OID30241","P50053","KHK",0,"Cardiometabolic_II","B13805","20212097_SS211647","PASS",-3.5085,3.1561,"Plate control","PASS","PFC",8.91416706811799,"24h","24h"
5189,"PFC_02",13,"OID30241","P50053","KHK",0,"Cardiometabolic_II","B13805","20212097_SS211647","PASS",-3.5085,3.4025,"Plate control","PASS","PFC",10.5743714081423,"Baseline","Baseline"
5190,"PFC_10",14,"OID30241","P50053","KHK",0,"Cardiometabolic_II","B13805","20212097_SS211647","PASS",-3.5085,3.1286,"Plate control","PASS","PFC",8.74585845683569,"1h","1h"
5191,"PFC_18",15,"OID30241","P50053","KHK",0,"Cardiometabolic_II","B13805","20212097_SS211647","PASS",-3.5085,3.3734,"Plate control","PASS","PFC",10.3632168863756,"1h","1h"
5192,"PFC_26",16,"OID30241","P50053","KHK",0,"Cardiometabolic_II","B13805","20212097_SS211647","PASS",-3.5085,3.9157,"Plate control","PASS","PFC",15.0918734211576,"24h","24h"
5193,"PFC_03",25,"OID30241","P50053","KHK",0,"Cardiometabolic_II","B13805","20212097_SS211647","PASS",-3.5085,2.5799,"Plate control","PASS","PFC",5.9789825486867,"Baseline","Baseline"
5194,"PFC_11",26,"OID30241","P50053","KHK",0,"Cardiometabolic_II","B13805","20212097_SS211647","PASS",-3.5085,3.487,"Plate control","PASS","PFC",11.212219592646,"1h","1h"
5195,"PFC_19",27,"OID30241","P50053","KHK",0,"Cardiometabolic_II","B13805","20212097_SS211647","PASS",-3.5085,3.8762,"Plate control","PASS","PFC",14.6842736484619,"24h","24h"
5196,"PFC_27",28,"OID30241","P50053","KHK",0,"Cardiometabolic_II","B13805","20212097_SS211647","PASS",-3.5085,4.3382,"Plate control","PASS","PFC",20.2268534181765,"24h","24h"
5197,"PFC_04",37,"OID30241","P50053","KHK",0,"Cardiometabolic_II","B13805","20212097_SS211647","PASS",-3.5085,3.5657,"Plate control","PASS","PFC",11.8408438937109,"Baseline","Baseline"
5198,"PFC_12",38,"OID30241","P50053","KHK",0,"Cardiometabolic_II","B13805","20212097_SS211647","PASS",-3.5085,2.3235,"Plate control","PASS","PFC",5.00545077692403,"1h","1h"
5199,"PFC_20",39,"OID30241","P50053","KHK",0,"Cardiometabolic_II","B13805","20212097_SS211647","PASS",-3.5085,3.2156,"Plate control","PASS","PFC",9.28949390371627,"24h","24h"
5200,"PFC_05",49,"OID30241","P50053","KHK",0,"Cardiometabolic_II","B13805","20212097_SS211647","PASS",-3.5085,3.6149,"Plate control","PASS","PFC",12.251614730102,"Baseline","Baseline"
5201,"PFC_13",50,"OID30241","P50053","KHK",0,"Cardiometabolic_II","B13805","20212097_SS211647","PASS",-3.5085,3.4619,"Plate control","PASS","PFC",11.0188365770437,"1h","1h"
5202,"PFC_21",51,"OID30241","P50053","KHK",0,"Cardiometabolic_II","B13805","20212097_SS211647","PASS",-3.5085,2.665,"Plate control","PASS","PFC",6.34227309288228,"24h","24h"
5203,"PFC_06",61,"OID30241","P50053","KHK",0,"Cardiometabolic_II","B13805","20212097_SS211647","PASS",-3.5085,3.9384,"Plate control","PASS","PFC",15.3312136173407,"Baseline","Baseline"
5204,"PFC_14",62,"OID30241","P50053","KHK",0,"Cardiometabolic_II","B13805","20212097_SS211647","PASS",-3.5085,3.9441,"Plate control","PASS","PFC",15.3919061232951,"1h","1h"
5205,"PFC_22",63,"OID30241","P50053","KHK",0,"Cardiometabolic_II","B13805","20212097_SS211647","PASS",-3.5085,3.1519,"Plate control","PASS","PFC",8.88825372108763,"24h","24h"
5206,"PFC_07",73,"OID30241","P50053","KHK",0,"Cardiometabolic_II","B13805","20212097_SS211647","PASS",-3.5085,3.4071,"Plate control","PASS","PFC",10.6081413572336,"Baseline","Baseline"
5207,"PFC_15",74,"OID30241","P50053","KHK",0,"Cardiometabolic_II","B13805","20212097_SS211647","PASS",-3.5085,2.9435,"Plate control","PASS","PFC",7.69275307021334,"1h","1h"
5208,"PFC_23",75,"OID30241","P50053","KHK",0,"Cardiometabolic_II","B13805","20212097_SS211647","PASS",-3.5085,4.2079,"Plate control","PASS","PFC",18.4800915661257,"24h","24h"
5209,"PFC_08",85,"OID30241","P50053","KHK",0,"Cardiometabolic_II","B13805","20212097_SS211647","PASS",-3.5085,3.2187,"Plate control","PASS","PFC",9.30947622276978,"Baseline","Baseline"
5210,"PFC_16",86,"OID30241","P50053","KHK",0,"Cardiometabolic_II","B13805","20212097_SS211647","PASS",-3.5085,3.0589,"Plate control","PASS","PFC",8.33336978686911,"1h","1h"
5211,"PFC_24",87,"OID30241","P50053","KHK",0,"Cardiometabolic_II","B13805","20212097_SS211647","PASS",-3.5085,4.242,"Plate control","PASS","PFC",18.9220960039063,"24h","24h"
5212,"PFC_01",1,"OID30242","O75348","ATP6V1G1",0,"Cardiometabolic_II","B13805","20212097_SS211647","PASS",-0.7985,1.1999,"Plate control","PASS","PFC",2.29723747210768,"Baseline","Baseline"
5213,"PFC_09",2,"OID30242","O75348","ATP6V1G1",0,"Cardiometabolic_II","B13805","20212097_SS211647","PASS",-0.7985,2.0421,"Plate control","PASS","PFC",4.11844579275157,"Baseline","Baseline"
5214,"PFC_17",3,"OID30242","O75348","ATP6V1G1",0,"Cardiometabolic_II","B13805","20212097_SS211647","PASS",-0.7985,2.7449,"Plate control","PASS","PFC",6.70343241471196,"1h","1h"
5215,"PFC_25",4,"OID30242","O75348","ATP6V1G1",0,"Cardiometabolic_II","B13805","20212097_SS211647","PASS",-0.7985,2.3103,"Plate control","PASS","PFC",4.95986206685153,"24h","24h"
5216,"PFC_02",13,"OID30242","O75348","ATP6V1G1",0,"Cardiometabolic_II","B13805","20212097_SS211647","PASS",-0.7985,1.5059,"Plate control","PASS","PFC",2.84001785522731,"Baseline","Baseline"
5217,"PFC_10",14,"OID30242","O75348","ATP6V1G1",0,"Cardiometabolic_II","B13805","20212097_SS211647","PASS",-0.7985,1.0894,"Plate control","PASS","PFC",2.1278552307487,"1h","1h"
5218,"PFC_18",15,"OID30242","O75348","ATP6V1G1",0,"Cardiometabolic_II","B13805","20212097_SS211647","PASS",-0.7985,2.1178,"Plate control","PASS","PFC",4.34031575029393,"1h","1h"
5219,"PFC_26",16,"OID30242","O75348","ATP6V1G1",0,"Cardiometabolic_II","B13805","20212097_SS211647","PASS",-0.7985,2.4796,"Plate control","PASS","PFC",5.57742805959272,"24h","24h"
5220,"PFC_03",25,"OID30242","O75348","ATP6V1G1",0,"Cardiometabolic_II","B13805","20212097_SS211647","PASS",-0.7985,0.3659,"Plate control","PASS","PFC",1.28868530253077,"Baseline","Baseline"
5221,"PFC_11",26,"OID30242","O75348","ATP6V1G1",0,"Cardiometabolic_II","B13805","20212097_SS211647","PASS",-0.7985,2.2357,"Plate control","PASS","PFC",4.70991165794684,"1h","1h"
5222,"PFC_19",27,"OID30242","O75348","ATP6V1G1",0,"Cardiometabolic_II","B13805","20212097_SS211647","PASS",-0.7985,1.9644,"Plate control","PASS","PFC",3.90250369990154,"24h","24h"
5223,"PFC_27",28,"OID30242","O75348","ATP6V1G1",0,"Cardiometabolic_II","B13805","20212097_SS211647","PASS",-0.7985,3.2918,"Plate control","PASS","PFC",9.79333340080554,"24h","24h"
5224,"PFC_04",37,"OID30242","O75348","ATP6V1G1",0,"Cardiometabolic_II","B13805","20212097_SS211647","PASS",-0.7985,2.6278,"Plate control","PASS","PFC",6.18082749315913,"Baseline","Baseline"
5225,"PFC_12",38,"OID30242","O75348","ATP6V1G1",0,"Cardiometabolic_II","B13805","20212097_SS211647","PASS",-0.7985,1.0985,"Plate control","PASS","PFC",2.14131939301766,"1h","1h"
5226,"PFC_20",39,"OID30242","O75348","ATP6V1G1",0,"Cardiometabolic_II","B13805","20212097_SS211647","PASS",-0.7985,2.6984,"Plate control","PASS","PFC",6.49081663165591,"24h","24h"
5227,"PFC_05",49,"OID30242","O75348","ATP6V1G1",0,"Cardiometabolic_II","B13805","20212097_SS211647","PASS",-0.7985,1.6394,"Plate control","PASS","PFC",3.11536240638226,"Baseline","Baseline"
5228,"PFC_13",50,"OID30242","O75348","ATP6V1G1",0,"Cardiometabolic_II","B13805","20212097_SS211647","PASS",-0.7985,2.5611,"Plate control","PASS","PFC",5.90157488015344,"1h","1h"
5229,"PFC_21",51,"OID30242","O75348","ATP6V1G1",0,"Cardiometabolic_II","B13805","20212097_SS211647","PASS",-0.7985,1.435,"Plate control","PASS","PFC",2.70382166605625,"24h","24h"
5230,"PFC_06",61,"OID30242","O75348","ATP6V1G1",0,"Cardiometabolic_II","B13805","20212097_SS211647","PASS",-0.7985,2.2571,"Plate control","PASS","PFC",4.78029615282391,"Baseline","Baseline"
5231,"PFC_14",62,"OID30242","O75348","ATP6V1G1",0,"Cardiometabolic_II","B13805","20212097_SS211647","PASS",-0.7985,2.3915,"Plate control","PASS","PFC",5.24702622249652,"1h","1h"
5232,"PFC_22",63,"OID30242","O75348","ATP6V1G1",0,"Cardiometabolic_II","B13805","20212097_SS211647","PASS",-0.7985,2.4627,"Plate control","PASS","PFC",5.51247420592632,"24h","24h"
5233,"PFC_07",73,"OID30242","O75348","ATP6V1G1",0,"Cardiometabolic_II","B13805","20212097_SS211647","PASS",-0.7985,2.6084,"Plate control","PASS","PFC",6.09826988641739,"Baseline","Baseline"
5234,"PFC_15",74,"OID30242","O75348","ATP6V1G1",0,"Cardiometabolic_II","B13805","20212097_SS211647","PASS",-0.7985,0.916,"Plate control","PASS","PFC",1.88687650179072,"1h","1h"
5235,"PFC_23",75,"OID30242","O75348","ATP6V1G1",0,"Cardiometabolic_II","B13805","20212097_SS211647","PASS",-0.7985,2.7205,"Plate control","PASS","PFC",6.5910120130493,"24h","24h"
5236,"PFC_08",85,"OID30242","O75348","ATP6V1G1",0,"Cardiometabolic_II","B13805","20212097_SS211647","PASS",-0.7985,1.7656,"Plate control","PASS","PFC",3.40015378675127,"Baseline","Baseline"
5237,"PFC_16",86,"OID30242","O75348","ATP6V1G1",0,"Cardiometabolic_II","B13805","20212097_SS211647","PASS",-0.7985,2.6244,"Plate control","PASS","PFC",6.16627828524379,"1h","1h"
5238,"PFC_24",87,"OID30242","O75348","ATP6V1G1",0,"Cardiometabolic_II","B13805","20212097_SS211647","PASS",-0.7985,2.5461,"Plate control","PASS","PFC",5.84053286347663,"24h","24h"
5239,"PFC_01",1,"OID30243","Q53T59","HS1BP3",0,"Cardiometabolic_II","B13805","20212097_SS211647","PASS",-2.2295,2.0242,"Plate control","PASS","PFC",4.06766255184907,"Baseline","Baseline"
5240,"PFC_09",2,"OID30243","Q53T59","HS1BP3",0,"Cardiometabolic_II","B13805","20212097_SS211647","PASS",-2.2295,2.6368,"Plate control","PASS","PFC",6.21950602068155,"Baseline","Baseline"
5241,"PFC_17",3,"OID30243","Q53T59","HS1BP3",0,"Cardiometabolic_II","B13805","20212097_SS211647","PASS",-2.2295,3.7878,"Plate control","PASS","PFC",13.8115181214997,"1h","1h"
5242,"PFC_25",4,"OID30243","Q53T59","HS1BP3",0,"Cardiometabolic_II","B13805","20212097_SS211647","PASS",-2.2295,3.3678,"Plate control","PASS","PFC",10.3230687432158,"24h","24h"
5243,"PFC_02",13,"OID30243","Q53T59","HS1BP3",0,"Cardiometabolic_II","B13805","20212097_SS211647","PASS",-2.2295,2.1512,"Plate control","PASS","PFC",4.44197107977938,"Baseline","Baseline"
5244,"PFC_10",14,"OID30243","Q53T59","HS1BP3",0,"Cardiometabolic_II","B13805","20212097_SS211647","PASS",-2.2295,2.2716,"Plate control","PASS","PFC",4.82858341274478,"1h","1h"
5245,"PFC_18",15,"OID30243","Q53T59","HS1BP3",0,"Cardiometabolic_II","B13805","20212097_SS211647","PASS",-2.2295,3.0007,"Plate control","PASS","PFC",8.00388256605136,"1h","1h"
5246,"PFC_26",16,"OID30243","Q53T59","HS1BP3",0,"Cardiometabolic_II","B13805","20212097_SS211647","PASS",-2.2295,3.4451,"Plate control","PASS","PFC",10.8912678309917,"24h","24h"
5247,"PFC_03",25,"OID30243","Q53T59","HS1BP3",0,"Cardiometabolic_II","B13805","20212097_SS211647","PASS",-2.2295,0.7044,"Plate control","PASS","PFC",1.62946684765615,"Baseline","Baseline"
5248,"PFC_11",26,"OID30243","Q53T59","HS1BP3",0,"Cardiometabolic_II","B13805","20212097_SS211647","PASS",-2.2295,3.2372,"Plate control","PASS","PFC",9.42962239512322,"1h","1h"
5249,"PFC_19",27,"OID30243","Q53T59","HS1BP3",0,"Cardiometabolic_II","B13805","20212097_SS211647","PASS",-2.2295,3.0273,"Plate control","PASS","PFC",8.15282472892112,"24h","24h"
5250,"PFC_27",28,"OID30243","Q53T59","HS1BP3",0,"Cardiometabolic_II","B13805","20212097_SS211647","PASS",-2.2295,4.5364,"Plate control","PASS","PFC",23.2055823878108,"24h","24h"
5251,"PFC_04",37,"OID30243","Q53T59","HS1BP3",0,"Cardiometabolic_II","B13805","20212097_SS211647","PASS",-2.2295,3.4584,"Plate control","PASS","PFC",10.9921371125187,"Baseline","Baseline"
5252,"PFC_12",38,"OID30243","Q53T59","HS1BP3",0,"Cardiometabolic_II","B13805","20212097_SS211647","PASS",-2.2295,1.5316,"Plate control","PASS","PFC",2.89106290511903,"1h","1h"
5253,"PFC_20",39,"OID30243","Q53T59","HS1BP3",0,"Cardiometabolic_II","B13805","20212097_SS211647","PASS",-2.2295,3.5593,"Plate control","PASS","PFC",11.7884325673182,"24h","24h"
5254,"PFC_05",49,"OID30243","Q53T59","HS1BP3",0,"Cardiometabolic_II","B13805","20212097_SS211647","PASS",-2.2295,1.8629,"Plate control","PASS","PFC",3.637380874435,"Baseline","Baseline"
5255,"PFC_13",50,"OID30243","Q53T59","HS1BP3",0,"Cardiometabolic_II","B13805","20212097_SS211647","PASS",-2.2295,3.6929,"Plate control","PASS","PFC",12.9322374754272,"1h","1h"
5256,"PFC_21",51,"OID30243","Q53T59","HS1BP3",0,"Cardiometabolic_II","B13805","20212097_SS211647","PASS",-2.2295,2.193,"Plate control","PASS","PFC",4.57255334165614,"24h","24h"
5257,"PFC_06",61,"OID30243","Q53T59","HS1BP3",0,"Cardiometabolic_II","B13805","20212097_SS211647","PASS",-2.2295,3.2016,"Plate control","PASS","PFC",9.1997840714107,"Baseline","Baseline"
5258,"PFC_14",62,"OID30243","Q53T59","HS1BP3",0,"Cardiometabolic_II","B13805","20212097_SS211647","PASS",-2.2295,3.201,"Plate control","PASS","PFC",9.19595878427742,"1h","1h"
5259,"PFC_22",63,"OID30243","Q53T59","HS1BP3",0,"Cardiometabolic_II","B13805","20212097_SS211647","PASS",-2.2295,3.0875,"Plate control","PASS","PFC",8.50021893292122,"24h","24h"
5260,"PFC_07",73,"OID30243","Q53T59","HS1BP3",0,"Cardiometabolic_II","B13805","20212097_SS211647","PASS",-2.2295,3.9161,"Plate control","PASS","PFC",15.0960583570906,"Baseline","Baseline"
5261,"PFC_15",74,"OID30243","Q53T59","HS1BP3",0,"Cardiometabolic_II","B13805","20212097_SS211647","PASS",-2.2295,1.8296,"Plate control","PASS","PFC",3.55438510323128,"1h","1h"
5262,"PFC_23",75,"OID30243","Q53T59","HS1BP3",0,"Cardiometabolic_II","B13805","20212097_SS211647","PASS",-2.2295,3.1771,"Plate control","PASS","PFC",9.04487145798402,"24h","24h"
5263,"PFC_08",85,"OID30243","Q53T59","HS1BP3",0,"Cardiometabolic_II","B13805","20212097_SS211647","PASS",-2.2295,2.46,"Plate control","PASS","PFC",5.50216727255897,"Baseline","Baseline"
5264,"PFC_16",86,"OID30243","Q53T59","HS1BP3",0,"Cardiometabolic_II","B13805","20212097_SS211647","PASS",-2.2295,3.3353,"Plate control","PASS","PFC",10.0931178573038,"1h","1h"

Supplemental table 1_Olink proximity extension assay (PEA) data

,"SampleID","Index","OlinkID","UniProt","Assay","MissingFreq","Panel","Panel_Lot_Nr","PlateID","QC_Warning","LOD","NPX","Normalization","Assay_Warning","condition","Value","group","alt_group"
5265,"PFC_24",87,"OID30243","Q53T59","HS1BP3",0,"Cardiometabolic_II","B13805","20212097_SS211647","PASS",-2.2295,3.6597,"Plate control","PASS","PFC",12.6380327115334,"24h","24h"
5266,"PFC_01",1,"OID30244","Q8IVF2","AHNAK2",0,"Cardiometabolic_II","B13805","20212097_SS211647","PASS",-2.8511,0.573,"Plate control","PASS","PFC",1.48761376243451,"Baseline","Baseline"
5267,"PFC_09",2,"OID30244","Q8IVF2","AHNAK2",0,"Cardiometabolic_II","B13805","20212097_SS211647","PASS",-2.8511,0.1807,"Plate control","PASS","PFC",1.13343369735995,"Baseline","Baseline"
5268,"PFC_17",3,"OID30244","Q8IVF2","AHNAK2",0,"Cardiometabolic_II","B13805","20212097_SS211647","PASS",-2.8511,0.4697,"Plate control","PASS","PFC",1.38482147263867,"1h","1h"
5269,"PFC_25",4,"OID30244","Q8IVF2","AHNAK2",0,"Cardiometabolic_II","B13805","20212097_SS211647","PASS",-2.8511,0.0879,"Plate control","PASS","PFC",1.06282200259786,"24h","24h"
5270,"PFC_02",13,"OID30244","Q8IVF2","AHNAK2",0,"Cardiometabolic_II","B13805","20212097_SS211647","PASS",-2.8511,3.4472,"Plate control","PASS","PFC",10.9071328031277,"Baseline","Baseline"
5271,"PFC_10",14,"OID30244","Q8IVF2","AHNAK2",0,"Cardiometabolic_II","B13805","20212097_SS211647","PASS",-2.8511,0.5065,"Plate control","PASS","PFC",1.42059961554597,"1h","1h"
5272,"PFC_18",15,"OID30244","Q8IVF2","AHNAK2",0,"Cardiometabolic_II","B13805","20212097_SS211647","PASS",-2.8511,0.3684,"Plate control","PASS","PFC",1.29092035996137,"1h","1h"
5273,"PFC_26",16,"OID30244","Q8IVF2","AHNAK2",0,"Cardiometabolic_II","B13805","20212097_SS211647","PASS",-2.8511,0.4715,"Plate control","PASS","PFC",1.38655034411936,"24h","24h"
5274,"PFC_03",25,"OID30244","Q8IVF2","AHNAK2",0,"Cardiometabolic_II","B13805","20212097_SS211647","PASS",-2.8511,0.2985,"Plate control","PASS","PFC",1.22986503214132,"Baseline","Baseline"
5275,"PFC_11",26,"OID30244","Q8IVF2","AHNAK2",0,"Cardiometabolic_II","B13805","20212097_SS211647","PASS",-2.8511,3.4011,"Plate control","PASS","PFC",10.5641149513922,"1h","1h"
5276,"PFC_19",27,"OID30244","Q8IVF2","AHNAK2",0,"Cardiometabolic_II","B13805","20212097_SS211647","PASS",-2.8511,0.445,"Plate control","PASS","PFC",1.36131411649947,"24h","24h"
5277,"PFC_27",28,"OID30244","Q8IVF2","AHNAK2",0,"Cardiometabolic_II","B13805","20212097_SS211647","PASS",-2.8511,0.2485,"Plate control","PASS","PFC",1.18797131421962,"24h","24h"
5278,"PFC_04",37,"OID30244","Q8IVF2","AHNAK2",0,"Cardiometabolic_II","B13805","20212097_SS211647","PASS",-2.8511,0.7676,"Plate control","PASS","PFC",1.70243533475568,"Baseline","Baseline"
5279,"PFC_12",38,"OID30244","Q8IVF2","AHNAK2",0,"Cardiometabolic_II","B13805","20212097_SS211647","PASS",-2.8511,0.4748,"Plate control","PASS","PFC",1.38972554961847,"1h","1h"
5280,"PFC_20",39,"OID30244","Q8IVF2","AHNAK2",0,"Cardiometabolic_II","B13805","20212097_SS211647","PASS",-2.8511,3.332,"Plate control","PASS","PFC",10.0700573880389,"24h","24h"
5281,"PFC_05",49,"OID30244","Q8IVF2","AHNAK2",0,"Cardiometabolic_II","B13805","20212097_SS211647","PASS",-2.8511,0.503,"Plate control","PASS","PFC",1.41715739650822,"Baseline","Baseline"
5282,"PFC_13",50,"OID30244","Q8IVF2","AHNAK2",0,"Cardiometabolic_II","B13805","20212097_SS211647","PASS",-2.8511,0.4439,"Plate control","PASS","PFC",1.36027656195176,"1h","1h"
5283,"PFC_21",51,"OID30244","Q8IVF2","AHNAK2",0,"Cardiometabolic_II","B13805","20212097_SS211647","PASS",-2.8511,0.2328,"Plate control","PASS","PFC",1.17511341181787,"24h","24h"
5284,"PFC_06",61,"OID30244","Q8IVF2","AHNAK2",0,"Cardiometabolic_II","B13805","20212097_SS211647","PASS",-2.8511,-0.1528,"Plate control","PASS","PFC",0.899503001098596,"Baseline","Baseline"
5285,"PFC_14",62,"OID30244","Q8IVF2","AHNAK2",0,"Cardiometabolic_II","B13805","20212097_SS211647","PASS",-2.8511,0.6019,"Plate control","PASS","PFC",1.51771404940659,"1h","1h"
5286,"PFC_22",63,"OID30244","Q8IVF2","AHNAK2",0,"Cardiometabolic_II","B13805","20212097_SS211647","PASS",-2.8511,0.193,"Plate control","PASS","PFC",1.14313833541404,"24h","24h"
5287,"PFC_07",73,"OID30244","Q8IVF2","AHNAK2",0,"Cardiometabolic_II","B13805","20212097_SS211647","PASS",-2.8511,0.2184,"Plate control","PASS","PFC",1.16344257160893,"Baseline","Baseline"
5288,"PFC_15",74,"OID30244","Q8IVF2","AHNAK2",0,"Cardiometabolic_II","B13805","20212097_SS211647","PASS",-2.8511,-0.0698,"Plate control","PASS","PFC",0.952770070866804,"1h","1h"
5289,"PFC_23",75,"OID30244","Q8IVF2","AHNAK2",0,"Cardiometabolic_II","B13805","20212097_SS211647","PASS",-2.8511,0.6004,"Plate control","PASS","PFC",1.51613687064007,"24h","24h"
5290,"PFC_08",85,"OID30244","Q8IVF2","AHNAK2",0,"Cardiometabolic_II","B13805","20212097_SS211647","PASS",-2.8511,0.2919,"Plate control","PASS","PFC",1.22425153080947,"Baseline","Baseline"
5291,"PFC_16",86,"OID30244","Q8IVF2","AHNAK2",0,"Cardiometabolic_II","B13805","20212097_SS211647","PASS",-2.8511,0.1685,"Plate control","PASS","PFC",1.12388934590813,"1h","1h"
5292,"PFC_24",87,"OID30244","Q8IVF2","AHNAK2",0,"Cardiometabolic_II","B13805","20212097_SS211647","PASS",-2.8511,0.0631,"Plate control","PASS","PFC",1.04470817400716,"24h","24h"
5293,"PFC_01",1,"OID30245","P12829","MYL4",0.1364,"Cardiometabolic_II","B13805","20212097_SS211647","PASS",-0.1254,0.9936,"Plate control","PASS","PFC",1.99114736637639,"Baseline","Baseline"
5294,"PFC_09",2,"OID30245","P12829","MYL4",0.1364,"Cardiometabolic_II","B13805","20212097_SS211647","PASS",-0.1254,0.7734,"Plate control","PASS","PFC",1.70929333282849,"Baseline","Baseline"
5295,"PFC_17",3,"OID30245","P12829","MYL4",0.1364,"Cardiometabolic_II","B13805","20212097_SS211647","PASS",-0.1254,-0.1907,"Plate control","PASS","PFC",0.876180492736539,"1h","1h"
5296,"PFC_25",4,"OID30245","P12829","MYL4",0.1364,"Cardiometabolic_II","B13805","20212097_SS211647","PASS",-0.1254,-0.3772,"Plate control","PASS","PFC",0.769930431815814,"24h","24h"
5297,"PFC_02",13,"OID30245","P12829","MYL4",0.1364,"Cardiometabolic_II","B13805","20212097_SS211647","PASS",-0.1254,0.6125,"Plate control","PASS","PFC",1.52890630750301,"Baseline","Baseline"
5298,"PFC_10",14,"OID30245","P12829","MYL4",0.1364,"Cardiometabolic_II","B13805","20212097_SS211647","PASS",-0.1254,0.7127,"Plate control","PASS","PFC",1.63886838673914,"1h","1h"
5299,"PFC_18",15,"OID30245","P12829","MYL4",0.1364,"Cardiometabolic_II","B13805","20212097_SS211647","PASS",-0.1254,-0.0245,"Plate control","PASS","PFC",0.983161277240399,"1h","1h"
5300,"PFC_26",16,"OID30245","P12829","MYL4",0.1364,"Cardiometabolic_II","B13805","20212097_SS211647","PASS",-0.1254,-0.0955,"Plate control","PASS","PFC",0.935947816423077,"24h","24h"
5301,"PFC_03",25,"OID30245","P12829","MYL4",0.1364,"Cardiometabolic_II","B13805","20212097_SS211647","PASS",-0.1254,1.2819,"Plate control","PASS","PFC",2.43159001554838,"Baseline","Baseline"
5302,"PFC_11",26,"OID30245","P12829","MYL4",0.1364,"Cardiometabolic_II","B13805","20212097_SS211647","PASS",-0.1254,0.509,"Plate control","PASS","PFC",1.4230634612346,"1h","1h"
5303,"PFC_19",27,"OID30245","P12829","MYL4",0.1364,"Cardiometabolic_II","B13805","20212097_SS211647","PASS",-0.1254,0.3249,"Plate control","PASS","PFC",1.25257761355091,"24h","24h"
5304,"PFC_27",28,"OID30245","P12829","MYL4",0.1364,"Cardiometabolic_II","B13805","20212097_SS211647","PASS",-0.1254,0.8828,"Plate control","PASS","PFC",1.84395059227499,"24h","24h"
5305,"PFC_04",37,"OID30245","P12829","MYL4",0.1364,"Cardiometabolic_II","B13805","20212097_SS211647","PASS",-0.1254,-0.3295,"Plate control","PASS","PFC",0.79581224347305,"Baseline","Baseline"
5306,"PFC_12",38,"OID30245","P12829","MYL4",0.1364,"Cardiometabolic_II","B13805","20212097_SS211647","PASS",-0.1254,1.3948,"Plate control","PASS","PFC",2.62952096613297,"1h","1h"
5307,"PFC_20",39,"OID30245","P12829","MYL4",0.1364,"Cardiometabolic_II","B13805","20212097_SS211647","PASS",-0.1254,-0.3289,"Plate control","PASS","PFC",0.796143281313353,"24h","24h"
5308,"PFC_05",49,"OID30245","P12829","MYL4",0.1364,"Cardiometabolic_II","B13805","20212097_SS211647","PASS",-0.1254,0.1597,"Plate control","PASS","PFC",1.11705482889784,"Baseline","Baseline"
5309,"PFC_13",50,"OID30245","P12829","MYL4",0.1364,"Cardiometabolic_II","B13805","20212097_SS211647","PASS",-0.1254,0.0048,"Plate control","PASS","PFC",1.00333264742883,"1h","1h"
5310,"PFC_21",51,"OID30245","P12829","MYL4",0.1364,"Cardiometabolic_II","B13805","20212097_SS211647","PASS",-0.1254,1.2657,"Plate control","PASS","PFC",2.4044384568867,"24h","24h"
5311,"PFC_06",61,"OID30245","P12829","MYL4",0.1364,"Cardiometabolic_II","B13805","20212097_SS211647","PASS",-0.1254,0.4395,"Plate control","PASS","PFC",1.35613424567899,"Baseline","Baseline"
5312,"PFC_14",62,"OID30245","P12829","MYL4",0.1364,"Cardiometabolic_II","B13805","20212097_SS211647","PASS",-0.1254,0.3573,"Plate control","PASS","PFC",1.28102621554827,"1h","1h"
5313,"PFC_22",63,"OID30245","P12829","MYL4",0.1364,"Cardiometabolic_II","B13805","20212097_SS211647","PASS",-0.1254,0.2758,"Plate control","PASS","PFC",1.21066523847733,"24h","24h"
5314,"PFC_07",73,"OID30245","P12829","MYL4",0.1364,"Cardiometabolic_II","B13805","20212097_SS211647","PASS",-0.1254,0.0236,"Plate control","PASS","PFC",1.01649280257012,"Baseline","Baseline"
5315,"PFC_15",74,"OID30245","P12829","MYL4",0.1364,"Cardiometabolic_II","B13805","20212097_SS211647","PASS",-0.1254,-0.1812,"Plate control","PASS","PFC",0.881969089844297,"1h","1h"
5316,"PFC_23",75,"OID30245","P12829","MYL4",0.1364,"Cardiometabolic_II","B13805","20212097_SS211647","PASS",-0.1254,0.7193,"Plate control","PASS","PFC",1.64638301068704,"24h","24h"
5317,"PFC_08",85,"OID30245","P12829","MYL4",0.1364,"Cardiometabolic_II","B13805","20212097_SS211647","PASS",-0.1254,-0.1287,"Plate control","PASS","PFC",0.914655266941912,"Baseline","Baseline"
5318,"PFC_16",86,"OID30245","P12829","MYL4",0.1364,"Cardiometabolic_II","B13805","20212097_SS211647","PASS",-0.1254,-0.0213,"Plate control","PASS","PFC",0.98534441902259,"1h","1h"
5319,"PFC_24",87,"OID30245","P12829","MYL4",0.1364,"Cardiometabolic_II","B13805","20212097_SS211647","PASS",-0.1254,0.044,"Plate control","PASS","PFC",1.03096831879479,"24h","24h"
5320,"PFC_01",1,"OID30247","O75061","DNAJC6",0,"Cardiometabolic_II","B13805","20212097_SS211647","PASS",-1.6433,3.5214,"Plate control","PASS","PFC",11.4827795380442,"Baseline","Baseline"
5321,"PFC_09",2,"OID30247","O75061","DNAJC6",0,"Cardiometabolic_II","B13805","20212097_SS211647","PASS",-1.6433,3.9541,"Plate control","PASS","PFC",15.498965296804,"Baseline","Baseline"
5322,"PFC_17",3,"OID30247","O75061","DNAJC6",0,"Cardiometabolic_II","B13805","20212097_SS211647","PASS",-1.6433,5.7686,"Plate control","PASS","PFC",54.5157050263249,"1h","1h"
5323,"PFC_25",4,"OID30247","O75061","DNAJC6",0,"Cardiometabolic_II","B13805","20212097_SS211647","PASS",-1.6433,4.6253,"Plate control","PASS","PFC",24.6805048396653,"24h","24h"
5324,"PFC_02",13,"OID30247","O75061","DNAJC6",0,"Cardiometabolic_II","B13805","20212097_SS211647","PASS",-1.6433,3.769,"Plate control","PASS","PFC",13.6327055211979,"Baseline","Baseline"
5325,"PFC_10",14,"OID30247","O75061","DNAJC6",0,"Cardiometabolic_II","B13805","20212097_SS211647","PASS",-1.6433,3.7064,"Plate control","PASS","PFC",13.0538186787759,"1h","1h"
5326,"PFC_18",15,"OID30247","O75061","DNAJC6",0,"Cardiometabolic_II","B13805","20212097_SS211647","PASS",-1.6433,4.1298,"Plate control","PASS","PFC",17.5062721673062,"1h","1h"
5327,"PFC_26",16,"OID30247","O75061","DNAJC6",0,"Cardiometabolic_II","B13805","20212097_SS211647","PASS",-1.6433,5.1252,"Plate control","PASS","PFC",34.9010854277296,"24h","24h"
5328,"PFC_03",25,"OID30247","O75061","DNAJC6",0,"Cardiometabolic_II","B13805","20212097_SS211647","PASS",-1.6433,2.3408,"Plate control","PASS","PFC",5.06583469240586,"Baseline","Baseline"
5329,"PFC_11",26,"OID30247","O75061","DNAJC6",0,"Cardiometabolic_II","B13805","20212097_SS211647","PASS",-1.6433,5.1719,"Plate control","PASS","PFC",36.0493164771032,"1h","1h"
5330,"PFC_19",27,"OID30247","O75061","DNAJC6",0,"Cardiometabolic_II","B13805","20212097_SS211647","PASS",-1.6433,4.6994,"Plate control","PASS","PFC",25.9812691301208,"24h","24h"
5331,"PFC_27",28,"OID30247","O75061","DNAJC6",0,"Cardiometabolic_II","B13805","20212097_SS211647","PASS",-1.6433,6.0567,"Plate control","PASS","PFC",66.5653737377995,"24h","24h"
5332,"PFC_04",37,"OID30247","O75061","DNAJC6",0,"Cardiometabolic_II","B13805","20212097_SS211647","PASS",-1.6433,4.8002,"Plate control","PASS","PFC",27.8614801790522,"Baseline","Baseline"
5333,"PFC_12",38,"OID30247","O75061","DNAJC6",0,"Cardiometabolic_II","B13805","20212097_SS211647","PASS",-1.6433,3.0818,"Plate control","PASS","PFC",8.46670134360727,"1h","1h"
5334,"PFC_20",39,"OID30247","O75061","DNAJC6",0,"Cardiometabolic_II","B13805","20212097_SS211647","PASS",-1.6433,5.6754,"Plate control","PASS","PFC",51.105264150616,"24h","24h"
5335,"PFC_05",49,"OID30247","O75061","DNAJC6",0,"Cardiometabolic_II","B13805","20212097_SS211647","PASS",-1.6433,3.3709,"Plate control","PASS","PFC",10.3452743504724,"Baseline","Baseline"
5336,"PFC_13",50,"OID30247","O75061","DNAJC6",0,"Cardiometabolic_II","B13805","20212097_SS211647","PASS",-1.6433,4.9868,"Plate control","PASS","PFC",31.7085499813433,"1h","1h"
5337,"PFC_21",51,"OID30247","O75061","DNAJC6",0,"Cardiometabolic_II","B13805","20212097_SS211647","PASS",-1.6433,3.5886,"Plate control","PASS","PFC",12.0302940429086,"24h","24h"
5338,"PFC_06",61,"OID30247","O75061","DNAJC6",0,"Cardiometabolic_II","B13805","20212097_SS211647","PASS",-1.6433,5.0483,"Plate control","PASS","PFC",33.0894636050987,"Baseline","Baseline"
5339,"PFC_14",62,"OID30247","O75061","DNAJC6",0,"Cardiometabolic_II","B13805","20212097_SS211647","PASS",-1.6433,5.0176,"Plate control","PASS","PFC",32.3927714067759,"1h","1h"
5340,"PFC_22",63,"OID30247","O75061","DNAJC6",0,"Cardiometabolic_II","B13805","20212097_SS211647","PASS",-1.6433,4.3787,"Plate control","PASS","PFC",20.8027160917797,"24h","24h"
5341,"PFC_07",73,"OID30247","O75061","DNAJC6",0,"Cardiometabolic_II","B13805","20212097_SS211647","PASS",-1.6433,5.1934,"Plate control","PASS","PFC",36.5905703872547,"Baseline","Baseline"
5342,"PFC_15",74,"OID30247","O75061","DNAJC6",0,"Cardiometabolic_II","B13805","20212097_SS211647","PASS",-1.6433,3.6473,"Plate control","PASS","PFC",12.52987398327,"1h","1h"
5343,"PFC_23",75,"OID30247","O75061","DNAJC6",0,"Cardiometabolic_II","B13805","20212097_SS211647","PASS",-1.6433,5.0951,"Plate control","PASS","PFC",34.180462184522,"24h","24h"
5344,"PFC_08",85,"OID30247","O75061","DNAJC6",0,"Cardiometabolic_II","B13805","20212097_SS211647","PASS",-1.6433,4.3833,"Plate control","PASS","PFC",20.8691509309077,"Baseline","Baseline"
5345,"PFC_16",86,"OID30247","O75061","DNAJC6",0,"Cardiometabolic_II","B13805","20212097_SS211647","PASS",-1.6433,4.9904,"Plate control","PASS","PFC",31.7877720741314,"1h","1h"
5346,"PFC_24",87,"OID30247","O75061","DNAJC6",0,"Cardiometabolic_II","B13805","20212097_SS211647","PASS",-1.6433,6.0873,"Plate control","PASS","PFC",67.9923250723125,"24h","24h"
5347,"PFC_01",1,"OID30248","Q8WUF8","FAM172A",0,"Cardiometabolic_II","B13805","20212097_SS211647","PASS",-1.1525,1.4768,"Plate control","PASS","PFC",2.78330690864873,"Baseline","Baseline"
5348,"PFC_09",2,"OID30248","Q8WUF8","FAM172A",0,"Cardiometabolic_II","B13805","20212097_SS211647","PASS",-1.1525,2.1356,"Plate control","PASS","PFC",4.39419837091079,"Baseline","Baseline"
5349,"PFC_17",3,"OID30248","Q8WUF8","FAM172A",0,"Cardiometabolic_II","B13805","20212097_SS211647","PASS",-1.1525,3.2834,"Plate control","PASS","PFC",9.73647801978871,"1h","1h"
5350,"PFC_25",4,"OID30248","Q8WUF8","FAM172A",0,"Cardiometabolic_II","B13805","20212097_SS211647","PASS",-1.1525,2.4735,"Plate control","PASS","PFC",5.55389537601318,"24h","24h"
5351,"PFC_02",13,"OID30248","Q8WUF8","FAM172A",0,"Cardiometabolic_II","B13805","20212097_SS211647","PASS",-1.1525,1.3262,"Plate control","PASS","PFC",2.50741361812459,"Baseline","Baseline"
5352,"PFC_10",14,"OID30248","Q8WUF8","FAM172A",0,"Cardiometabolic_II","B13805","20212097_SS211647","PASS",-1.1525,1.8011,"Plate control","PASS","PFC",3.48485831216904,"1h","1h"
5353,"PFC_18",15,"OID30248","Q8WUF8","FAM172A",0,"Cardiometabolic_II","B13805","20212097_SS211647","PASS",-1.1525,2.1673,"Plate control","PASS","PFC",4.49181963882176,"1h","1h"
5354,"PFC_26",16,"OID30248","Q8WUF8","FAM172A",0,"Cardiometabolic_II","B13805","20212097_SS211647","PASS",-1.1525,3.0695,"Plate control","PASS","PFC",8.39482354062726,"24h","24h"
5355,"PFC_03",25,"OID30248","Q8WUF8","FAM172A",0,"Cardiometabolic_II","B13805","20212097_SS211647","PASS",-1.1525,1.072,"Plate control","PASS","PFC",2.10234581817541,"Baseline","Baseline"
5356,"PFC_11",26,"OID30248","Q8WUF8","FAM172A",0,"Cardiometabolic_II","B13805","20212097_SS211647","PASS",-1.1525,2.4741,"Plate control","PASS","PFC",5.55620565654182,"1h","1h"
5357,"PFC_19",27,"OID30248","Q8WUF8","FAM172A",0,"Cardiometabolic_II","B13805","20212097_SS211647","PASS",-1.1525,2.4366,"Plate control","PASS","PFC",5.41364392730057,"24h","24h"
5358,"PFC_27",28,"OID30248","Q8WUF8","FAM172A",0,"Cardiometabolic_II","B13805","20212097_SS211647","PASS",-1.1525,3.2006,"Plate control","PASS","PFC",9.19340947654129,"24h","24h"
5359,"PFC_04",37,"OID30248","Q8WUF8","FAM172A",0,"Cardiometabolic_II","B13805","20212097_SS211647","PASS",-1.1525,2.4579,"Plate control","PASS","PFC",5.49416409408461,"Baseline","Baseline"
5360,"PFC_12",38,"OID30248","Q8WUF8","FAM172A",0,"Cardiometabolic_II","B13805","20212097_SS211647","PASS",-1.1525,1.3379,"Plate control","PASS","PFC",2.52783097494163,"1h","1h"
5361,"PFC_20",39,"OID30248","Q8WUF8","FAM172A",0,"Cardiometabolic_II","B13805","20212097_SS211647","PASS",-1.1525,2.5339,"Plate control","PASS","PFC",5.79135124972652,"24h","24h"
5362,"PFC_05",49,"OID30248","Q8WUF8","FAM172A",0,"Cardiometabolic_II","B13805","20212097_SS211647","PASS",-1.1525,1.3267,"Plate control","PASS","PFC",2.50828277206874,"Baseline","Baseline"
5363,"PFC_13",50,"OID30248","Q8WUF8","FAM172A",0,"Cardiometabolic_II","B13805","20212097_SS211647","PASS",-1.1525,2.7967,"Plate control","PASS","PFC",6.94849243258385,"1h","1h"
5364,"PFC_21",51,"OID30248","Q8WUF8","FAM172A",0,"Cardiometabolic_II","B13805","20212097_SS211647","PASS",-1.1525,1.8759,"Plate control","PASS","PFC",3.67030511426404,"24h","24h"
5365,"PFC_06",61,"OID30248","Q8WUF8","FAM172A",0,"Cardiometabolic_II","B13805","20212097_SS211647","PASS",-1.1525,2.2782,"Plate control","PASS","PFC",4.85072368272708,"Baseline","Baseline"
5366,"PFC_14",62,"OID30248","Q8WUF8","FAM172A",0,"Cardiometabolic_II","B13805","20212097_SS211647","PASS",-1.1525,2.456,"Plate control","PASS","PFC",5.48693315436239,"1h","1h"
5367,"PFC_22",63,"OID30248","Q8WUF8","FAM172A",0,"Cardiometabolic_II","B13805","20212097_SS211647","PASS",-1.1525,2.443,"Plate control","PASS","PFC",5.43771296762696,"24h","24h"
5368,"PFC_07",73,"OID30248","Q8WUF8","FAM172A",0,"Cardiometabolic_II","B13805","20212097_SS211647","PASS",-1.1525,3.0071,"Plate control","PASS","PFC",8.0394677975215,"Baseline","Baseline"
5369,"PFC_15",74,"OID30248","Q8WUF8","FAM172A",0,"Cardiometabolic_II","B13805","20212097_SS211647","PASS",-1.1525,1.5047,"Plate control","PASS","PFC",2.83765657694857,"1h","1h"
5370,"PFC_23",75,"OID30248","Q8WUF8","FAM172A",0,"Cardiometabolic_II","B13805","20212097_SS211647","PASS",-1.1525,2.4676,"Plate control","PASS","PFC",5.53122872118732,"24h","24h"
5371,"PFC_08",85,"OID30248","Q8WUF8","FAM172A",0,"Cardiometabolic_II","B13805","20212097_SS211647","PASS",-1.1525,2.4407,"Plate control","PASS","PFC",5.42905086274413,"Baseline","Baseline"
5372,"PFC_16",86,"OID30248","Q8WUF8","FAM172A",0,"Cardiometabolic_II","B13805","20212097_SS211647","PASS",-1.1525,2.662,"Plate control","PASS","PFC",6.3290984094836,"1h","1h"
5373,"PFC_24",87,"OID30248","Q8WUF8","FAM172A",0,"Cardiometabolic_II","B13805","20212097_SS211647","PASS",-1.1525,2.8408,"Plate control","PASS","PFC",7.16417212674051,"24h","24h"
5374,"PFC_01",1,"OID30249","Q13137","CALCOCO2",0,"Cardiometabolic_II","B13805","20212097_SS211647","PASS",-1.5243,1.5684,"Plate control","PASS","PFC",2.96575618783962,"Baseline","Baseline"
5375,"PFC_09",2,"OID30249","Q13137","CALCOCO2",0,"Cardiometabolic_II","B13805","20212097_SS211647","PASS",-1.5243,2.2663,"Plate control","PASS","PFC",4.81087728539482,"Baseline","Baseline"
5376,"PFC_17",3,"OID30249","Q13137","CALCOCO2",0,"Cardiometabolic_II","B13805","20212097_SS211647","PASS",-1.5243,3.2374,"Plate control","PASS","PFC",9.4309297089726,"1h","1h"

Supplemental table 1_Olink proximity extension assay (PEA) data

,"SampleID","Index","OlinkID","UniProt","Assay","MissingFreq","Panel","Panel_Lot_Nr","PlateID","QC_Warning","LOD","NPX","Normalization","Assay_Warning","condition","Value","group","alt_group"
5377,"PFC_25",4,"OID30249","Q13137","CALCOCO2",0,"Cardiometabolic_II","B13805","20212097_SS211647","PASS",-1.5243,3.1011,"Plate control","PASS","PFC",8.58072768465101,"24h","24h"
5378,"PFC_02",13,"OID30249","Q13137","CALCOCO2",0,"Cardiometabolic_II","B13805","20212097_SS211647","PASS",-1.5243,1.8741,"Plate control","PASS","PFC",3.66572865884401,"Baseline","Baseline"
5379,"PFC_10",14,"OID30249","Q13137","CALCOCO2",0,"Cardiometabolic_II","B13805","20212097_SS211647","PASS",-1.5243,1.5938,"Plate control","PASS","PFC",3.01843346430637,"1h","1h"
5380,"PFC_18",15,"OID30249","Q13137","CALCOCO2",0,"Cardiometabolic_II","B13805","20212097_SS211647","PASS",-1.5243,2.3834,"Plate control","PASS","PFC",5.21764938008873,"1h","1h"
5381,"PFC_26",16,"OID30249","Q13137","CALCOCO2",0,"Cardiometabolic_II","B13805","20212097_SS211647","PASS",-1.5243,2.8474,"Plate control","PASS","PFC",7.19702165868958,"24h","24h"
5382,"PFC_03",25,"OID30249","Q13137","CALCOCO2",0,"Cardiometabolic_II","B13805","20212097_SS211647","PASS",-1.5243,0.4039,"Plate control","PASS","PFC",1.32307972783136,"Baseline","Baseline"
5383,"PFC_11",26,"OID30249","Q13137","CALCOCO2",0,"Cardiometabolic_II","B13805","20212097_SS211647","PASS",-1.5243,3.011,"Plate control","PASS","PFC",8.06123008328403,"1h","1h"
5384,"PFC_19",27,"OID30249","Q13137","CALCOCO2",0,"Cardiometabolic_II","B13805","20212097_SS211647","PASS",-1.5243,2.2697,"Plate control","PASS","PFC",4.82222845228066,"24h","24h"
5385,"PFC_27",28,"OID30249","Q13137","CALCOCO2",0,"Cardiometabolic_II","B13805","20212097_SS211647","PASS",-1.5243,3.5671,"Plate control","PASS","PFC",11.852339897289,"24h","24h"
5386,"PFC_04",37,"OID30249","Q13137","CALCOCO2",0,"Cardiometabolic_II","B13805","20212097_SS211647","PASS",-1.5243,2.4105,"Plate control","PASS","PFC",5.31658552526026,"Baseline","Baseline"
5387,"PFC_12",38,"OID30249","Q13137","CALCOCO2",0,"Cardiometabolic_II","B13805","20212097_SS211647","PASS",-1.5243,1.0788,"Plate control","PASS","PFC",2.11227840644137,"1h","1h"
5388,"PFC_20",39,"OID30249","Q13137","CALCOCO2",0,"Cardiometabolic_II","B13805","20212097_SS211647","PASS",-1.5243,3.3391,"Plate control","PASS","PFC",10.1197377612915,"24h","24h"
5389,"PFC_05",49,"OID30249","Q13137","CALCOCO2",0,"Cardiometabolic_II","B13805","20212097_SS211647","PASS",-1.5243,1.6384,"Plate control","PASS","PFC",3.11320374993359,"Baseline","Baseline"
5390,"PFC_13",50,"OID30249","Q13137","CALCOCO2",0,"Cardiometabolic_II","B13805","20212097_SS211647","PASS",-1.5243,2.9793,"Plate control","PASS","PFC",7.88603437977652,"1h","1h"
5391,"PFC_21",51,"OID30249","Q13137","CALCOCO2",0,"Cardiometabolic_II","B13805","20212097_SS211647","PASS",-1.5243,1.2752,"Plate control","PASS","PFC",2.42032368329005,"24h","24h"
5392,"PFC_06",61,"OID30249","Q13137","CALCOCO2",0,"Cardiometabolic_II","B13805","20212097_SS211647","PASS",-1.5243,2.6595,"Plate control","PASS","PFC",6.31814041480952,"Baseline","Baseline"
5393,"PFC_14",62,"OID30249","Q13137","CALCOCO2",0,"Cardiometabolic_II","B13805","20212097_SS211647","PASS",-1.5243,3.1199,"Plate control","PASS","PFC",8.6932763073282,"1h","1h"
5394,"PFC_22",63,"OID30249","Q13137","CALCOCO2",0,"Cardiometabolic_II","B13805","20212097_SS211647","PASS",-1.5243,2.4651,"Plate control","PASS","PFC",5.52165213208309,"24h","24h"
5395,"PFC_07",73,"OID30249","Q13137","CALCOCO2",0,"Cardiometabolic_II","B13805","20212097_SS211647","PASS",-1.5243,3.1679,"Plate control","PASS","PFC",8.9873762452121,"Baseline","Baseline"
5396,"PFC_15",74,"OID30249","Q13137","CALCOCO2",0,"Cardiometabolic_II","B13805","20212097_SS211647","PASS",-1.5243,2.1051,"Plate control","PASS","PFC",4.30227576252602,"1h","1h"
5397,"PFC_23",75,"OID30249","Q13137","CALCOCO2",0,"Cardiometabolic_II","B13805","20212097_SS211647","PASS",-1.5243,2.6788,"Plate control","PASS","PFC",6.40323074745073,"24h","24h"
5398,"PFC_08",85,"OID30249","Q13137","CALCOCO2",0,"Cardiometabolic_II","B13805","20212097_SS211647","PASS",-1.5243,2.2199,"Plate control","PASS","PFC",4.65861142434493,"Baseline","Baseline"
5399,"PFC_16",86,"OID30249","Q13137","CALCOCO2",0,"Cardiometabolic_II","B13805","20212097_SS211647","PASS",-1.5243,3.1388,"Plate control","PASS","PFC",8.80791166431669,"1h","1h"
5400,"PFC_24",87,"OID30249","Q13137","CALCOCO2",0,"Cardiometabolic_II","B13805","20212097_SS211647","PASS",-1.5243,3.4019,"Plate control","PASS","PFC",10.5699745650668,"24h","24h"
5401,"PFC_01",1,"OID30250","P58107","EPPK1",0,"Cardiometabolic_II","B13805","20212097_SS211647","PASS",-3.0143,0.6258,"Plate control","PASS","PFC",1.54306624582729,"Baseline","Baseline"
5402,"PFC_09",2,"OID30250","P58107","EPPK1",0,"Cardiometabolic_II","B13805","20212097_SS211647","PASS",-3.0143,1.7779,"Plate control","PASS","PFC",3.42926643966208,"Baseline","Baseline"
5403,"PFC_17",3,"OID30250","P58107","EPPK1",0,"Cardiometabolic_II","B13805","20212097_SS211647","PASS",-3.0143,2.3814,"Plate control","PASS","PFC",5.21042119352954,"1h","1h"
5404,"PFC_25",4,"OID30250","P58107","EPPK1",0,"Cardiometabolic_II","B13805","20212097_SS211647","PASS",-3.0143,1.6168,"Plate control","PASS","PFC",3.06694012169475,"24h","24h"
5405,"PFC_02",13,"OID30250","P58107","EPPK1",0,"Cardiometabolic_II","B13805","20212097_SS211647","PASS",-3.0143,1.3847,"Plate control","PASS","PFC",2.61117653882253,"Baseline","Baseline"
5406,"PFC_10",14,"OID30250","P58107","EPPK1",0,"Cardiometabolic_II","B13805","20212097_SS211647","PASS",-3.0143,0.962,"Plate control","PASS","PFC",1.94800853708648,"1h","1h"
5407,"PFC_18",15,"OID30250","P58107","EPPK1",0,"Cardiometabolic_II","B13805","20212097_SS211647","PASS",-3.0143,1.9687,"Plate control","PASS","PFC",3.9141525918084,"1h","1h"
5408,"PFC_26",16,"OID30250","P58107","EPPK1",0,"Cardiometabolic_II","B13805","20212097_SS211647","PASS",-3.0143,1.4053,"Plate control","PASS","PFC",2.64872855114642,"24h","24h"
5409,"PFC_03",25,"OID30250","P58107","EPPK1",0,"Cardiometabolic_II","B13805","20212097_SS211647","PASS",-3.0143,0.5259,"Plate control","PASS","PFC",1.43983151354034,"Baseline","Baseline"
5410,"PFC_11",26,"OID30250","P58107","EPPK1",0,"Cardiometabolic_II","B13805","20212097_SS211647","PASS",-3.0143,1.8527,"Plate control","PASS","PFC",3.61175491919261,"1h","1h"
5411,"PFC_19",27,"OID30250","P58107","EPPK1",0,"Cardiometabolic_II","B13805","20212097_SS211647","PASS",-3.0143,1.0534,"Plate control","PASS","PFC",2.07541522065091,"24h","24h"
5412,"PFC_27",28,"OID30250","P58107","EPPK1",0,"Cardiometabolic_II","B13805","20212097_SS211647","PASS",-3.0143,2.1538,"Plate control","PASS","PFC",4.44998354086822,"24h","24h"
5413,"PFC_04",37,"OID30250","P58107","EPPK1",0,"Cardiometabolic_II","B13805","20212097_SS211647","PASS",-3.0143,2.228,"Plate control","PASS","PFC",4.68484072487524,"Baseline","Baseline"
5414,"PFC_12",38,"OID30250","P58107","EPPK1",0,"Cardiometabolic_II","B13805","20212097_SS211647","PASS",-3.0143,1.0092,"Plate control","PASS","PFC",2.01279466024415,"1h","1h"
5415,"PFC_20",39,"OID30250","P58107","EPPK1",0,"Cardiometabolic_II","B13805","20212097_SS211647","PASS",-3.0143,1.6994,"Plate control","PASS","PFC",3.2476586412651,"24h","24h"
5416,"PFC_05",49,"OID30250","P58107","EPPK1",0,"Cardiometabolic_II","B13805","20212097_SS211647","PASS",-3.0143,1.2913,"Plate control","PASS","PFC",2.44748496954366,"Baseline","Baseline"
5417,"PFC_13",50,"OID30250","P58107","EPPK1",0,"Cardiometabolic_II","B13805","20212097_SS211647","PASS",-3.0143,2.9777,"Plate control","PASS","PFC",7.87729333574265,"1h","1h"
5418,"PFC_21",51,"OID30250","P58107","EPPK1",0,"Cardiometabolic_II","B13805","20212097_SS211647","PASS",-3.0143,1.4348,"Plate control","PASS","PFC",2.70344686276332,"24h","24h"
5419,"PFC_06",61,"OID30250","P58107","EPPK1",0,"Cardiometabolic_II","B13805","20212097_SS211647","PASS",-3.0143,0.5809,"Plate control","PASS","PFC",1.49578207505624,"Baseline","Baseline"
5420,"PFC_14",62,"OID30250","P58107","EPPK1",0,"Cardiometabolic_II","B13805","20212097_SS211647","PASS",-3.0143,2.655,"Plate control","PASS","PFC",6.29846381255363,"1h","1h"
5421,"PFC_22",63,"OID30250","P58107","EPPK1",0,"Cardiometabolic_II","B13805","20212097_SS211647","PASS",-3.0143,2.109,"Plate control","PASS","PFC",4.31392172677781,"24h","24h"
5422,"PFC_07",73,"OID30250","P58107","EPPK1",0,"Cardiometabolic_II","B13805","20212097_SS211647","PASS",-3.0143,1.7298,"Plate control","PASS","PFC",3.31681834272825,"Baseline","Baseline"
5423,"PFC_15",74,"OID30250","P58107","EPPK1",0,"Cardiometabolic_II","B13805","20212097_SS211647","PASS",-3.0143,0.65,"Plate control","PASS","PFC",1.5691681957935,"1h","1h"
5424,"PFC_23",75,"OID30250","P58107","EPPK1",0,"Cardiometabolic_II","B13805","20212097_SS211647","PASS",-3.0143,2.2811,"Plate control","PASS","PFC",4.86048405902633,"24h","24h"
5425,"PFC_08",85,"OID30250","P58107","EPPK1",0,"Cardiometabolic_II","B13805","20212097_SS211647","PASS",-3.0143,0.8432,"Plate control","PASS","PFC",1.79402500676099,"Baseline","Baseline"
5426,"PFC_16",86,"OID30250","P58107","EPPK1",0,"Cardiometabolic_II","B13805","20212097_SS211647","PASS",-3.0143,1.5231,"Plate control","PASS","PFC",2.87407956219851,"1h","1h"
5427,"PFC_24",87,"OID30250","P58107","EPPK1",0,"Cardiometabolic_II","B13805","20212097_SS211647","PASS",-3.0143,0.8823,"Plate control","PASS","PFC",1.84331163842658,"24h","24h"
5428,"PFC_01",1,"OID30251","P08590","MYL3",0,"Cardiometabolic_II","B13805","20212097_SS211647","PASS",-3.0269,-0.2476,"Plate control","PASS","PFC",0.842296455402001,"Baseline","Baseline"
5429,"PFC_09",2,"OID30251","P08590","MYL3",0,"Cardiometabolic_II","B13805","20212097_SS211647","PASS",-3.0269,-0.6454,"Plate control","PASS","PFC",0.639315510166214,"Baseline","Baseline"
5430,"PFC_17",3,"OID30251","P08590","MYL3",0,"Cardiometabolic_II","B13805","20212097_SS211647","PASS",-3.0269,-0.767,"Plate control","PASS","PFC",0.587638164209885,"1h","1h"
5431,"PFC_25",4,"OID30251","P08590","MYL3",0,"Cardiometabolic_II","B13805","20212097_SS211647","PASS",-3.0269,-0.7508,"Plate control","PASS","PFC",0.594273930678222,"24h","24h"
5432,"PFC_02",13,"OID30251","P08590","MYL3",0,"Cardiometabolic_II","B13805","20212097_SS211647","PASS",-3.0269,0.2863,"Plate control","PASS","PFC",1.21950865740815,"Baseline","Baseline"
5433,"PFC_10",14,"OID30251","P08590","MYL3",0,"Cardiometabolic_II","B13805","20212097_SS211647","PASS",-3.0269,0.1471,"Plate control","PASS","PFC",1.10734133687678,"1h","1h"
5434,"PFC_18",15,"OID30251","P08590","MYL3",0,"Cardiometabolic_II","B13805","20212097_SS211647","PASS",-3.0269,0.0422,"Plate control","PASS","PFC",1.02968281788859,"1h","1h"
5435,"PFC_26",16,"OID30251","P08590","MYL3",0,"Cardiometabolic_II","B13805","20212097_SS211647","PASS",-3.0269,-1.1539,"Plate control","PASS","PFC",0.449408712864165,"24h","24h"
5436,"PFC_03",25,"OID30251","P08590","MYL3",0,"Cardiometabolic_II","B13805","20212097_SS211647","PASS",-3.0269,0.7837,"Plate control","PASS","PFC",1.72154035520183,"Baseline","Baseline"
5437,"PFC_11",26,"OID30251","P08590","MYL3",0,"Cardiometabolic_II","B13805","20212097_SS211647","PASS",-3.0269,1.6907,"Plate control","PASS","PFC",3.22813295684349,"1h","1h"
5438,"PFC_19",27,"OID30251","P08590","MYL3",0,"Cardiometabolic_II","B13805","20212097_SS211647","PASS",-3.0269,-0.4518,"Plate control","PASS","PFC",0.731130073324658,"24h","24h"
5439,"PFC_27",28,"OID30251","P08590","MYL3",0,"Cardiometabolic_II","B13805","20212097_SS211647","PASS",-3.0269,-0.4815,"Plate control","PASS","PFC",0.716232554879049,"24h","24h"
5440,"PFC_04",37,"OID30251","P08590","MYL3",0,"Cardiometabolic_II","B13805","20212097_SS211647","PASS",-3.0269,-0.1001,"Plate control","PASS","PFC",0.932968320859403,"Baseline","Baseline"
5441,"PFC_12",38,"OID30251","P08590","MYL3",0,"Cardiometabolic_II","B13805","20212097_SS211647","PASS",-3.0269,1.6776,"Plate control","PASS","PFC",3.19895345213746,"1h","1h"
5442,"PFC_20",39,"OID30251","P08590","MYL3",0,"Cardiometabolic_II","B13805","20212097_SS211647","PASS",-3.0269,0.7511,"Plate control","PASS","PFC",1.6830756224399,"24h","24h"
5443,"PFC_05",49,"OID30251","P08590","MYL3",0,"Cardiometabolic_II","B13805","20212097_SS211647","PASS",-3.0269,0.5398,"Plate control","PASS","PFC",1.45377096790078,"Baseline","Baseline"
5444,"PFC_13",50,"OID30251","P08590","MYL3",0,"Cardiometabolic_II","B13805","20212097_SS211647","PASS",-3.0269,-0.4469,"Plate control","PASS","PFC",0.733617520812975,"1h","1h"
5445,"PFC_21",51,"OID30251","P08590","MYL3",0,"Cardiometabolic_II","B13805","20212097_SS211647","PASS",-3.0269,2.0425,"Plate control","PASS","PFC",4.11958782669959,"24h","24h"
5446,"PFC_06",61,"OID30251","P08590","MYL3",0,"Cardiometabolic_II","B13805","20212097_SS211647","PASS",-3.0269,-1.0093,"Plate control","PASS","PFC",0.496787231919059,"Baseline","Baseline"
5447,"PFC_14",62,"OID30251","P08590","MYL3",0,"Cardiometabolic_II","B13805","20212097_SS211647","PASS",-3.0269,0.0741,"Plate control","PASS","PFC",1.0527041200699,"1h","1h"
5448,"PFC_22",63,"OID30251","P08590","MYL3",0,"Cardiometabolic_II","B13805","20212097_SS211647","PASS",-3.0269,-0.5495,"Plate control","PASS","PFC",0.683256886139947,"24h","24h"
5449,"PFC_07",73,"OID30251","P08590","MYL3",0,"Cardiometabolic_II","B13805","20212097_SS211647","PASS",-3.0269,-1.0437,"Plate control","PASS","PFC",0.48508181461158,"Baseline","Baseline"
5450,"PFC_15",74,"OID30251","P08590","MYL3",0,"Cardiometabolic_II","B13805","20212097_SS211647","PASS",-3.0269,0.0078,"Plate control","PASS","PFC",1.00542118976428,"1h","1h"
5451,"PFC_23",75,"OID30251","P08590","MYL3",0,"Cardiometabolic_II","B13805","20212097_SS211647","PASS",-3.0269,0.3209,"Plate control","PASS","PFC",1.24910954097696,"24h","24h"
5452,"PFC_08",85,"OID30251","P08590","MYL3",0,"Cardiometabolic_II","B13805","20212097_SS211647","PASS",-3.0269,-2.0349,"Plate control","PASS","PFC",0.244024854121819,"Baseline","Baseline"
5453,"PFC_16",86,"OID30251","P08590","MYL3",0,"Cardiometabolic_II","B13805","20212097_SS211647","PASS",-3.0269,-0.3383,"Plate control","PASS","PFC",0.790972805912032,"1h","1h"
5454,"PFC_24",87,"OID30251","P08590","MYL3",0,"Cardiometabolic_II","B13805","20212097_SS211647","PASS",-3.0269,0.0674,"Plate control","PASS","PFC",1.04782660605208,"24h","24h"
5455,"PFC_01",1,"OID30252","Q86X76","NIT1",0,"Cardiometabolic_II","B13805","20212097_SS211647","PASS",-3.0747,3.2529,"Plate control","PASS","PFC",9.53279981036039,"Baseline","Baseline"
5456,"PFC_09",2,"OID30252","Q86X76","NIT1",0,"Cardiometabolic_II","B13805","20212097_SS211647","PASS",-3.0747,3.6923,"Plate control","PASS","PFC",12.9268602273054,"Baseline","Baseline"
5457,"PFC_17",3,"OID30252","Q86X76","NIT1",0,"Cardiometabolic_II","B13805","20212097_SS211647","PASS",-3.0747,4.106,"Plate control","PASS","PFC",17.2198419965408,"1h","1h"
5458,"PFC_25",4,"OID30252","Q86X76","NIT1",0,"Cardiometabolic_II","B13805","20212097_SS211647","PASS",-3.0747,3.6846,"Plate control","PASS","PFC",12.8580503490908,"24h","24h"
5459,"PFC_02",13,"OID30252","Q86X76","NIT1",0,"Cardiometabolic_II","B13805","20212097_SS211647","PASS",-3.0747,3.3197,"Plate control","PASS","PFC",9.98456794279212,"Baseline","Baseline"
5460,"PFC_10",14,"OID30252","Q86X76","NIT1",0,"Cardiometabolic_II","B13805","20212097_SS211647","PASS",-3.0747,3.4674,"Plate control","PASS","PFC",11.0609239664323,"1h","1h"
5461,"PFC_18",15,"OID30252","Q86X76","NIT1",0,"Cardiometabolic_II","B13805","20212097_SS211647","PASS",-3.0747,3.7725,"Plate control","PASS","PFC",13.6658188215248,"1h","1h"
5462,"PFC_26",16,"OID30252","Q86X76","NIT1",0,"Cardiometabolic_II","B13805","20212097_SS211647","PASS",-3.0747,4.0582,"Plate control","PASS","PFC",16.6586547827981,"24h","24h"
5463,"PFC_03",25,"OID30252","Q86X76","NIT1",0,"Cardiometabolic_II","B13805","20212097_SS211647","PASS",-3.0747,2.6084,"Plate control","PASS","PFC",6.09826988641739,"Baseline","Baseline"
5464,"PFC_11",26,"OID30252","Q86X76","NIT1",0,"Cardiometabolic_II","B13805","20212097_SS211647","PASS",-3.0747,3.8888,"Plate control","PASS","PFC",14.8130826887906,"1h","1h"
5465,"PFC_19",27,"OID30252","Q86X76","NIT1",0,"Cardiometabolic_II","B13805","20212097_SS211647","PASS",-3.0747,3.9189,"Plate control","PASS","PFC",15.12538541985,"24h","24h"
5466,"PFC_27",28,"OID30252","Q86X76","NIT1",0,"Cardiometabolic_II","B13805","20212097_SS211647","PASS",-3.0747,4.1494,"Plate control","PASS","PFC",17.7457297766577,"24h","24h"
5467,"PFC_04",37,"OID30252","Q86X76","NIT1",0,"Cardiometabolic_II","B13805","20212097_SS211647","PASS",-3.0747,3.9548,"Plate control","PASS","PFC",15.5064872663656,"Baseline","Baseline"
5468,"PFC_12",38,"OID30252","Q86X76","NIT1",0,"Cardiometabolic_II","B13805","20212097_SS211647","PASS",-3.0747,3.2006,"Plate control","PASS","PFC",9.19340947654129,"1h","1h"
5469,"PFC_20",39,"OID30252","Q86X76","NIT1",0,"Cardiometabolic_II","B13805","20212097_SS211647","PASS",-3.0747,3.9761,"Plate control","PASS","PFC",15.7371239609265,"24h","24h"
5470,"PFC_05",49,"OID30252","Q86X76","NIT1",0,"Cardiometabolic_II","B13805","20212097_SS211647","PASS",-3.0747,3.1346,"Plate control","PASS","PFC",8.78230720011649,"Baseline","Baseline"
5471,"PFC_13",50,"OID30252","Q86X76","NIT1",0,"Cardiometabolic_II","B13805","20212097_SS211647","PASS",-3.0747,4.0059,"Plate control","PASS","PFC",16.0655670729769,"1h","1h"
5472,"PFC_21",51,"OID30252","Q86X76","NIT1",0,"Cardiometabolic_II","B13805","20212097_SS211647","PASS",-3.0747,3.4683,"Plate control","PASS","PFC",11.0678262825884,"24h","24h"
5473,"PFC_06",61,"OID30252","Q86X76","NIT1",0,"Cardiometabolic_II","B13805","20212097_SS211647","PASS",-3.0747,3.8742,"Plate control","PASS","PFC",14.6639310263949,"Baseline","Baseline"
5474,"PFC_14",62,"OID30252","Q86X76","NIT1",0,"Cardiometabolic_II","B13805","20212097_SS211647","PASS",-3.0747,3.794,"Plate control","PASS","PFC",13.871001016235,"1h","1h"
5475,"PFC_22",63,"OID30252","Q86X76","NIT1",0,"Cardiometabolic_II","B13805","20212097_SS211647","PASS",-3.0747,4.0102,"Plate control","PASS","PFC",16.1135224546105,"24h","24h"
5476,"PFC_07",73,"OID30252","Q86X76","NIT1",0,"Cardiometabolic_II","B13805","20212097_SS211647","PASS",-3.0747,3.927,"Plate control","PASS","PFC",15.2105456196771,"Baseline","Baseline"
5477,"PFC_15",74,"OID30252","Q86X76","NIT1",0,"Cardiometabolic_II","B13805","20212097_SS211647","PASS",-3.0747,3.2372,"Plate control","PASS","PFC",9.42962239512322,"1h","1h"
5478,"PFC_23",75,"OID30252","Q86X76","NIT1",0,"Cardiometabolic_II","B13805","20212097_SS211647","PASS",-3.0747,3.7087,"Plate control","PASS","PFC",13.0746461768738,"24h","24h"
5479,"PFC_08",85,"OID30252","Q86X76","NIT1",0,"Cardiometabolic_II","B13805","20212097_SS211647","PASS",-3.0747,3.6814,"Plate control","PASS","PFC",12.8295618871757,"Baseline","Baseline"
5480,"PFC_16",86,"OID30252","Q86X76","NIT1",0,"Cardiometabolic_II","B13805","20212097_SS211647","PASS",-3.0747,3.6817,"Plate control","PASS","PFC",12.83222999697,"1h","1h"
5481,"PFC_24",87,"OID30252","Q86X76","NIT1",0,"Cardiometabolic_II","B13805","20212097_SS211647","PASS",-3.0747,4.1856,"Plate control","PASS","PFC",18.1966377672195,"24h","24h"
5482,"PFC_01",1,"OID30253","A0A0B4J2D5","GATD3B",0,"Cardiometabolic_II","B13805","20212097_SS211647","PASS",-1.8978,-0.2724,"Plate control","PASS","PFC",0.827941075499801,"Baseline","Baseline"
5483,"PFC_09",2,"OID30253","A0A0B4J2D5","GATD3B",0,"Cardiometabolic_II","B13805","20212097_SS211647","PASS",-1.8978,0.3544,"Plate control","PASS","PFC",1.27845377672096,"Baseline","Baseline"
5484,"PFC_17",3,"OID30253","A0A0B4J2D5","GATD3B",0,"Cardiometabolic_II","B13805","20212097_SS211647","PASS",-1.8978,2.0409,"Plate control","PASS","PFC",4.11502159012739,"1h","1h"
5485,"PFC_25",4,"OID30253","A0A0B4J2D5","GATD3B",0,"Cardiometabolic_II","B13805","20212097_SS211647","PASS",-1.8978,1.6656,"Plate control","PASS","PFC",3.17245565933774,"24h","24h"
5486,"PFC_02",13,"OID30253","A0A0B4J2D5","GATD3B",0,"Cardiometabolic_II","B13805","20212097_SS211647","PASS",-1.8978,-0.3796,"Plate control","PASS","PFC",0.768650676322345,"Baseline","Baseline"
5487,"PFC_10",14,"OID30253","A0A0B4J2D5","GATD3B",0,"Cardiometabolic_II","B13805","20212097_SS211647","PASS",-1.8978,-0.501,"Plate control","PASS","PFC",0.706616821941365,"1h","1h"
5488,"PFC_18",15,"OID30253","A0A0B4J2D5","GATD3B",0,"Cardiometabolic_II","B13805","20212097_SS211647","PASS",-1.8978,0.6313,"Plate control","PASS","PFC",1.54896011942152,"1h","1h"

Supplemental table 1_Olink proximity extension assay (PEA) data

,"SampleID","Index","OlinkID","UniProt","Assay","MissingFreq","Panel","Panel_Lot_Nr","PlateID","QC_Warning","LOD","NPX","Normalization","Assay_Warning","condition","Value","group","alt_group"
5489,"PFC_26",16,"OID30253","A0A0B4J2D5","GATD3B",0,"Cardiometabolic_II","B13805","20212097_SS211647","PASS",-1.8978,1.6723,"Plate control","PASS","PFC",3.18722308064576,"24h","24h"
5490,"PFC_03",25,"OID30253","A0A0B4J2D5","GATD3B",0,"Cardiometabolic_II","B13805","20212097_SS211647","PASS",-1.8978,-0.3293,"Plate control","PASS","PFC",0.795922574122975,"Baseline","Baseline"
5491,"PFC_11",26,"OID30253","A0A0B4J2D5","GATD3B",0,"Cardiometabolic_II","B13805","20212097_SS211647","PASS",-1.8978,1.0059,"Plate control","PASS","PFC",2.00819588412211,"1h","1h"
5492,"PFC_19",27,"OID30253","A0A0B4J2D5","GATD3B",0,"Cardiometabolic_II","B13805","20212097_SS211647","PASS",-1.8978,1.1129,"Plate control","PASS","PFC",2.16279960792646,"24h","24h"
5493,"PFC_27",28,"OID30253","A0A0B4J2D5","GATD3B",0,"Cardiometabolic_II","B13805","20212097_SS211647","PASS",-1.8978,1.3259,"Plate control","PASS","PFC",2.50689227032811,"24h","24h"
5494,"PFC_04",37,"OID30253","A0A0B4J2D5","GATD3B",0,"Cardiometabolic_II","B13805","20212097_SS211647","PASS",-1.8978,1.2207,"Plate control","PASS","PFC",2.33059771170734,"Baseline","Baseline"
5495,"PFC_12",38,"OID30253","A0A0B4J2D5","GATD3B",0,"Cardiometabolic_II","B13805","20212097_SS211647","PASS",-1.8978,0.8037,"Plate control","PASS","PFC",1.74557216299799,"1h","1h"
5496,"PFC_20",39,"OID30253","A0A0B4J2D5","GATD3B",0,"Cardiometabolic_II","B13805","20212097_SS211647","PASS",-1.8978,1.673,"Plate control","PASS","PFC",3.18876990616214,"24h","24h"
5497,"PFC_05",49,"OID30253","A0A0B4J2D5","GATD3B",0,"Cardiometabolic_II","B13805","20212097_SS211647","PASS",-1.8978,0.1023,"Plate control","PASS","PFC",1.07348348780182,"Baseline","Baseline"
5498,"PFC_13",50,"OID30253","A0A0B4J2D5","GATD3B",0,"Cardiometabolic_II","B13805","20212097_SS211647","PASS",-1.8978,1.6035,"Plate control","PASS","PFC",3.03879636371618,"1h","1h"
5499,"PFC_21",51,"OID30253","A0A0B4J2D5","GATD3B",0,"Cardiometabolic_II","B13805","20212097_SS211647","PASS",-1.8978,0.9076,"Plate control","PASS","PFC",1.87592220481639,"24h","24h"
5500,"PFC_06",61,"OID30253","A0A0B4J2D5","GATD3B",0,"Cardiometabolic_II","B13805","20212097_SS211647","PASS",-1.8978,0.8518,"Plate control","PASS","PFC",1.80475124593455,"Baseline","Baseline"
5501,"PFC_14",62,"OID30253","A0A0B4J2D5","GATD3B",0,"Cardiometabolic_II","B13805","20212097_SS211647","PASS",-1.8978,1.285,"Plate control","PASS","PFC",2.43682052735038,"1h","1h"
5502,"PFC_22",63,"OID30253","A0A0B4J2D5","GATD3B",0,"Cardiometabolic_II","B13805","20212097_SS211647","PASS",-1.8978,0.576,"Plate control","PASS","PFC",1.49071038679892,"24h","24h"
5503,"PFC_07",73,"OID30253","A0A0B4J2D5","GATD3B",0,"Cardiometabolic_II","B13805","20212097_SS211647","PASS",-1.8978,1.2832,"Plate control","PASS","PFC",2.4337820877213,"Baseline","Baseline"
5504,"PFC_15",74,"OID30253","A0A0B4J2D5","GATD3B",0,"Cardiometabolic_II","B13805","20212097_SS211647","PASS",-1.8978,0.0556,"Plate control","PASS","PFC",1.03929124250733,"1h","1h"
5505,"PFC_23",75,"OID30253","A0A0B4J2D5","GATD3B",0,"Cardiometabolic_II","B13805","20212097_SS211647","PASS",-1.8978,0.4895,"Plate control","PASS","PFC",1.40395821657154,"24h","24h"
5506,"PFC_08",85,"OID30253","A0A0B4J2D5","GATD3B",0,"Cardiometabolic_II","B13805","20212097_SS211647","PASS",-1.8978,0.5074,"Plate control","PASS","PFC",1.42148610818506,"Baseline","Baseline"
5507,"PFC_16",86,"OID30253","A0A0B4J2D5","GATD3B",0,"Cardiometabolic_II","B13805","20212097_SS211647","PASS",-1.8978,1.3189,"Plate control","PASS","PFC",2.49475821437264,"1h","1h"
5508,"PFC_24",87,"OID30253","A0A0B4J2D5","GATD3B",0,"Cardiometabolic_II","B13805","20212097_SS211647","PASS",-1.8978,0.6781,"Plate control","PASS","PFC",1.60003115853053,"24h","24h"
5509,"PFC_01",1,"OID30254","Q12841","FSTL1",0,"Cardiometabolic_II","B13805","20212097_SS211647","PASS",-4.2536,0.1408,"Plate control","PASS","PFC",1.10251631125974,"Baseline","Baseline"
5510,"PFC_09",2,"OID30254","Q12841","FSTL1",0,"Cardiometabolic_II","B13805","20212097_SS211647","PASS",-4.2536,0.2363,"Plate control","PASS","PFC",1.17796771562889,"Baseline","Baseline"
5511,"PFC_17",3,"OID30254","Q12841","FSTL1",0,"Cardiometabolic_II","B13805","20212097_SS211647","PASS",-4.2536,0.068,"Plate control","PASS","PFC",1.04826247551693,"1h","1h"
5512,"PFC_25",4,"OID30254","Q12841","FSTL1",0,"Cardiometabolic_II","B13805","20212097_SS211647","PASS",-4.2536,0.2128,"Plate control","PASS","PFC",1.15893527822355,"24h","24h"
5513,"PFC_02",13,"OID30254","Q12841","FSTL1",0,"Cardiometabolic_II","B13805","20212097_SS211647","PASS",-4.2536,0.1416,"Plate control","PASS","PFC",1.10312784565555,"Baseline","Baseline"
5514,"PFC_10",14,"OID30254","Q12841","FSTL1",0,"Cardiometabolic_II","B13805","20212097_SS211647","PASS",-4.2536,0.0945,"Plate control","PASS","PFC",1.06769530892173,"1h","1h"
5515,"PFC_18",15,"OID30254","Q12841","FSTL1",0,"Cardiometabolic_II","B13805","20212097_SS211647","PASS",-4.2536,0.0746,"Plate control","PASS","PFC",1.05306902274546,"1h","1h"
5516,"PFC_26",16,"OID30254","Q12841","FSTL1",0,"Cardiometabolic_II","B13805","20212097_SS211647","PASS",-4.2536,-0.2287,"Plate control","PASS","PFC",0.853403539939709,"24h","24h"
5517,"PFC_03",25,"OID30254","Q12841","FSTL1",0,"Cardiometabolic_II","B13805","20212097_SS211647","PASS",-4.2536,0.1451,"Plate control","PASS","PFC",1.10580729938507,"Baseline","Baseline"
5518,"PFC_11",26,"OID30254","Q12841","FSTL1",0,"Cardiometabolic_II","B13805","20212097_SS211647","PASS",-4.2536,0.1948,"Plate control","PASS","PFC",1.14456547913301,"1h","1h"
5519,"PFC_19",27,"OID30254","Q12841","FSTL1",0,"Cardiometabolic_II","B13805","20212097_SS211647","PASS",-4.2536,0.2726,"Plate control","PASS","PFC",1.2079828729863,"24h","24h"
5520,"PFC_27",28,"OID30254","Q12841","FSTL1",0,"Cardiometabolic_II","B13805","20212097_SS211647","PASS",-4.2536,0.2846,"Plate control","PASS","PFC",1.21807249544636,"24h","24h"
5521,"PFC_04",37,"OID30254","Q12841","FSTL1",0,"Cardiometabolic_II","B13805","20212097_SS211647","PASS",-4.2536,0.2357,"Plate control","PASS","PFC",1.17747791448671,"Baseline","Baseline"
5522,"PFC_12",38,"OID30254","Q12841","FSTL1",0,"Cardiometabolic_II","B13805","20212097_SS211647","PASS",-4.2536,0.259,"Plate control","PASS","PFC",1.19664896323071,"1h","1h"
5523,"PFC_20",39,"OID30254","Q12841","FSTL1",0,"Cardiometabolic_II","B13805","20212097_SS211647","PASS",-4.2536,0.2615,"Plate control","PASS","PFC",1.19872439557392,"24h","24h"
5524,"PFC_05",49,"OID30254","Q12841","FSTL1",0,"Cardiometabolic_II","B13805","20212097_SS211647","PASS",-4.2536,0.0953,"Plate control","PASS","PFC",1.06828752909931,"Baseline","Baseline"
5525,"PFC_13",50,"OID30254","Q12841","FSTL1",0,"Cardiometabolic_II","B13805","20212097_SS211647","PASS",-4.2536,0.1371,"Plate control","PASS","PFC",1.09969237153954,"1h","1h"
5526,"PFC_21",51,"OID30254","Q12841","FSTL1",0,"Cardiometabolic_II","B13805","20212097_SS211647","PASS",-4.2536,0.2922,"Plate control","PASS","PFC",1.22450613322916,"24h","24h"
5527,"PFC_06",61,"OID30254","Q12841","FSTL1",0,"Cardiometabolic_II","B13805","20212097_SS211647","PASS",-4.2536,0.1754,"Plate control","PASS","PFC",1.12927746360095,"Baseline","Baseline"
5528,"PFC_14",62,"OID30254","Q12841","FSTL1",0,"Cardiometabolic_II","B13805","20212097_SS211647","PASS",-4.2536,0.1748,"Plate control","PASS","PFC",1.12880790795509,"1h","1h"
5529,"PFC_22",63,"OID30254","Q12841","FSTL1",0,"Cardiometabolic_II","B13805","20212097_SS211647","PASS",-4.2536,0.1904,"Plate control","PASS","PFC",1.14108004658048,"24h","24h"
5530,"PFC_07",73,"OID30254","Q12841","FSTL1",0,"Cardiometabolic_II","B13805","20212097_SS211647","PASS",-4.2536,0.4153,"Plate control","PASS","PFC",1.33357595758529,"Baseline","Baseline"
5531,"PFC_15",74,"OID30254","Q12841","FSTL1",0,"Cardiometabolic_II","B13805","20212097_SS211647","PASS",-4.2536,-0.0141,"Plate control","PASS","PFC",0.990274228975089,"1h","1h"
5532,"PFC_23",75,"OID30254","Q12841","FSTL1",0,"Cardiometabolic_II","B13805","20212097_SS211647","PASS",-4.2536,0.2794,"Plate control","PASS","PFC",1.21369001992948,"24h","24h"
5533,"PFC_08",85,"OID30254","Q12841","FSTL1",0,"Cardiometabolic_II","B13805","20212097_SS211647","PASS",-4.2536,-0.3734,"Plate control","PASS","PFC",0.771961070361991,"Baseline","Baseline"
5534,"PFC_16",86,"OID30254","Q12841","FSTL1",0,"Cardiometabolic_II","B13805","20212097_SS211647","PASS",-4.2536,0.2791,"Plate control","PASS","PFC",1.21343766642349,"1h","1h"
5535,"PFC_24",87,"OID30254","Q12841","FSTL1",0,"Cardiometabolic_II","B13805","20212097_SS211647","PASS",-4.2536,0.13,"Plate control","PASS","PFC",1.09429370126074,"24h","24h"
5536,"PFC_01",1,"OID30255","Q9NR61","DLL4",0.8636,"Cardiometabolic_II","B13805","20212097_SS211647","PASS",-0.9658,-1.6844,"Plate control","PASS","PFC",0.311132282699868,"Baseline","Baseline"
5537,"PFC_09",2,"OID30255","Q9NR61","DLL4",0.8636,"Cardiometabolic_II","B13805","20212097_SS211647","PASS",-0.9658,-1.4839,"Plate control","PASS","PFC",0.357521027200782,"Baseline","Baseline"
5538,"PFC_17",3,"OID30255","Q9NR61","DLL4",0.8636,"Cardiometabolic_II","B13805","20212097_SS211647","PASS",-0.9658,-1.8034,"Plate control","PASS","PFC",0.286498601140102,"1h","1h"
5539,"PFC_25",4,"OID30255","Q9NR61","DLL4",0.8636,"Cardiometabolic_II","B13805","20212097_SS211647","PASS",-0.9658,-1.9046,"Plate control","PASS","PFC",0.267090394928641,"24h","24h"
5540,"PFC_02",13,"OID30255","Q9NR61","DLL4",0.8636,"Cardiometabolic_II","B13805","20212097_SS211647","PASS",-0.9658,-1.9697,"Plate control","PASS","PFC",0.255306115321569,"Baseline","Baseline"
5541,"PFC_10",14,"OID30255","Q9NR61","DLL4",0.8636,"Cardiometabolic_II","B13805","20212097_SS211647","PASS",-0.9658,-1.5718,"Plate control","PASS","PFC",0.336388432236905,"1h","1h"
5542,"PFC_18",15,"OID30255","Q9NR61","DLL4",0.8636,"Cardiometabolic_II","B13805","20212097_SS211647","PASS",-0.9658,-2.1956,"Plate control","PASS","PFC",0.218302415675066,"1h","1h"
5543,"PFC_26",16,"OID30255","Q9NR61","DLL4",0.8636,"Cardiometabolic_II","B13805","20212097_SS211647","PASS",-0.9658,-1.6124,"Plate control","PASS","PFC",0.327053826716719,"24h","24h"
5544,"PFC_03",25,"OID30255","Q9NR61","DLL4",0.8636,"Cardiometabolic_II","B13805","20212097_SS211647","PASS",-0.9658,-2.3565,"Plate control","PASS","PFC",0.195264284870549,"Baseline","Baseline"
5545,"PFC_11",26,"OID30255","Q9NR61","DLL4",0.8636,"Cardiometabolic_II","B13805","20212097_SS211647","PASS",-0.9658,-1.8686,"Plate control","PASS","PFC",0.273839031475061,"1h","1h"
5546,"PFC_19",27,"OID30255","Q9NR61","DLL4",0.8636,"Cardiometabolic_II","B13805","20212097_SS211647","PASS",-0.9658,-1.668,"Plate control","PASS","PFC",0.314689293376214,"24h","24h"
5547,"PFC_27",28,"OID30255","Q9NR61","DLL4",0.8636,"Cardiometabolic_II","B13805","20212097_SS211647","PASS",-0.9658,-1.6574,"Plate control","PASS","PFC",0.317009943826161,"24h","24h"
5548,"PFC_04",37,"OID30255","Q9NR61","DLL4",0.8636,"Cardiometabolic_II","B13805","20212097_SS211647","PASS",-0.9658,-2.5806,"Plate control","PASS","PFC",0.167171405256085,"Baseline","Baseline"
5549,"PFC_12",38,"OID30255","Q9NR61","DLL4",0.8636,"Cardiometabolic_II","B13805","20212097_SS211647","PASS",-0.9658,-1.7148,"Plate control","PASS","PFC",0.304644795727829,"1h","1h"
5550,"PFC_20",39,"OID30255","Q9NR61","DLL4",0.8636,"Cardiometabolic_II","B13805","20212097_SS211647","PASS",-0.9658,-1.6998,"Plate control","PASS","PFC",0.30782877450785,"24h","24h"
5551,"PFC_05",49,"OID30255","Q9NR61","DLL4",0.8636,"Cardiometabolic_II","B13805","20212097_SS211647","PASS",-0.9658,3.8423,"Plate control","PASS","PFC",14.3432494779061,"Baseline","Baseline"
5552,"PFC_13",50,"OID30255","Q9NR61","DLL4",0.8636,"Cardiometabolic_II","B13805","20212097_SS211647","PASS",-0.9658,-1.8622,"Plate control","PASS","PFC",0.275056518768282,"1h","1h"
5553,"PFC_21",51,"OID30255","Q9NR61","DLL4",0.8636,"Cardiometabolic_II","B13805","20212097_SS211647","PASS",-0.9658,-1.7214,"Plate control","PASS","PFC",0.303254298460352,"24h","24h"
5554,"PFC_06",61,"OID30255","Q9NR61","DLL4",0.8636,"Cardiometabolic_II","B13805","20212097_SS211647","PASS",-0.9658,-2.8041,"Plate control","PASS","PFC",0.143179812435176,"Baseline","Baseline"
5555,"PFC_14",62,"OID30255","Q9NR61","DLL4",0.8636,"Cardiometabolic_II","B13805","20212097_SS211647","PASS",-0.9658,3.9731,"Plate control","PASS","PFC",15.7044335323128,"1h","1h"
5556,"PFC_22",63,"OID30255","Q9NR61","DLL4",0.8636,"Cardiometabolic_II","B13805","20212097_SS211647","PASS",-0.9658,-1.7101,"Plate control","PASS","PFC",0.305638883415744,"24h","24h"
5557,"PFC_07",73,"OID30255","Q9NR61","DLL4",0.8636,"Cardiometabolic_II","B13805","20212097_SS211647","PASS",-0.9658,-2.5425,"Plate control","PASS","PFC",0.171645031234362,"Baseline","Baseline"
5558,"PFC_15",74,"OID30255","Q9NR61","DLL4",0.8636,"Cardiometabolic_II","B13805","20212097_SS211647","PASS",-0.9658,-1.5451,"Plate control","PASS","PFC",0.342671948212242,"1h","1h"
5559,"PFC_23",75,"OID30255","Q9NR61","DLL4",0.8636,"Cardiometabolic_II","B13805","20212097_SS211647","PASS",-0.9658,3.6646,"Plate control","PASS","PFC",12.6810297702956,"24h","24h"
5560,"PFC_08",85,"OID30255","Q9NR61","DLL4",0.8636,"Cardiometabolic_II","B13805","20212097_SS211647","PASS",-0.9658,-2.3537,"Plate control","PASS","PFC",0.195643624152473,"Baseline","Baseline"
5561,"PFC_16",86,"OID30255","Q9NR61","DLL4",0.8636,"Cardiometabolic_II","B13805","20212097_SS211647","PASS",-0.9658,-2.5412,"Plate control","PASS","PFC",0.171799768790521,"1h","1h"
5562,"PFC_24",87,"OID30255","Q9NR61","DLL4",0.8636,"Cardiometabolic_II","B13805","20212097_SS211647","PASS",-0.9658,-1.6366,"Plate control","PASS","PFC",0.321613528701438,"24h","24h"
5563,"PFC_01",1,"OID30256","Q6YN16","HSDL2",0,"Cardiometabolic_II","B13805","20212097_SS211647","PASS",-1.9867,-0.2016,"Plate control","PASS","PFC",0.869585626999752,"Baseline","Baseline"
5564,"PFC_09",2,"OID30256","Q6YN16","HSDL2",0,"Cardiometabolic_II","B13805","20212097_SS211647","PASS",-1.9867,0.6734,"Plate control","PASS","PFC",1.59482707174288,"Baseline","Baseline"
5565,"PFC_17",3,"OID30256","Q6YN16","HSDL2",0,"Cardiometabolic_II","B13805","20212097_SS211647","PASS",-1.9867,1.7081,"Plate control","PASS","PFC",3.267302428738,"1h","1h"
5566,"PFC_25",4,"OID30256","Q6YN16","HSDL2",0,"Cardiometabolic_II","B13805","20212097_SS211647","PASS",-1.9867,1.2132,"Plate control","PASS","PFC",2.31851329572201,"24h","24h"
5567,"PFC_02",13,"OID30256","Q6YN16","HSDL2",0,"Cardiometabolic_II","B13805","20212097_SS211647","PASS",-1.9867,-0.3531,"Plate control","PASS","PFC",0.782900027881067,"Baseline","Baseline"
5568,"PFC_10",14,"OID30256","Q6YN16","HSDL2",0,"Cardiometabolic_II","B13805","20212097_SS211647","PASS",-1.9867,-0.0376,"Plate control","PASS","PFC",0.974274357308802,"1h","1h"
5569,"PFC_18",15,"OID30256","Q6YN16","HSDL2",0,"Cardiometabolic_II","B13805","20212097_SS211647","PASS",-1.9867,0.7384,"Plate control","PASS","PFC",1.66832458132365,"1h","1h"
5570,"PFC_26",16,"OID30256","Q6YN16","HSDL2",0,"Cardiometabolic_II","B13805","20212097_SS211647","PASS",-1.9867,1.0865,"Plate control","PASS","PFC",2.12358226790998,"24h","24h"
5571,"PFC_03",25,"OID30256","Q6YN16","HSDL2",0,"Cardiometabolic_II","B13805","20212097_SS211647","PASS",-1.9867,-0.1198,"Plate control","PASS","PFC",0.920315224562546,"Baseline","Baseline"
5572,"PFC_11",26,"OID30256","Q6YN16","HSDL2",0,"Cardiometabolic_II","B13805","20212097_SS211647","PASS",-1.9867,0.1908,"Plate control","PASS","PFC",1.14139646501019,"1h","1h"
5573,"PFC_19",27,"OID30256","Q6YN16","HSDL2",0,"Cardiometabolic_II","B13805","20212097_SS211647","PASS",-1.9867,0.2878,"Plate control","PASS","PFC",1.22077726527417,"24h","24h"
5574,"PFC_27",28,"OID30256","Q6YN16","HSDL2",0,"Cardiometabolic_II","B13805","20212097_SS211647","PASS",-1.9867,1.011,"Plate control","PASS","PFC",2.01530752082101,"24h","24h"
5575,"PFC_04",37,"OID30256","Q6YN16","HSDL2",0,"Cardiometabolic_II","B13805","20212097_SS211647","PASS",-1.9867,0.4883,"Plate control","PASS","PFC",1.40279092248747,"Baseline","Baseline"
5576,"PFC_12",38,"OID30256","Q6YN16","HSDL2",0,"Cardiometabolic_II","B13805","20212097_SS211647","PASS",-1.9867,0.0942,"Plate control","PASS","PFC",1.06747331100619,"1h","1h"
5577,"PFC_20",39,"OID30256","Q6YN16","HSDL2",0,"Cardiometabolic_II","B13805","20212097_SS211647","PASS",-1.9867,-0.0066,"Plate control","PASS","PFC",0.995435676935972,"24h","24h"
5578,"PFC_05",49,"OID30256","Q6YN16","HSDL2",0,"Cardiometabolic_II","B13805","20212097_SS211647","PASS",-1.9867,-0.0118,"Plate control","PASS","PFC",0.991854221399365,"Baseline","Baseline"
5579,"PFC_13",50,"OID30256","Q6YN16","HSDL2",0,"Cardiometabolic_II","B13805","20212097_SS211647","PASS",-1.9867,0.5241,"Plate control","PASS","PFC",1.4380362064667,"1h","1h"
5580,"PFC_21",51,"OID30256","Q6YN16","HSDL2",0,"Cardiometabolic_II","B13805","20212097_SS211647","PASS",-1.9867,0.2691,"Plate control","PASS","PFC",1.2050558402058,"24h","24h"
5581,"PFC_06",61,"OID30256","Q6YN16","HSDL2",0,"Cardiometabolic_II","B13805","20212097_SS211647","PASS",-1.9867,0.5289,"Plate control","PASS","PFC",1.44282867413274,"Baseline","Baseline"
5582,"PFC_14",62,"OID30256","Q6YN16","HSDL2",0,"Cardiometabolic_II","B13805","20212097_SS211647","PASS",-1.9867,0.5799,"Plate control","PASS","PFC",1.49474563717167,"1h","1h"
5583,"PFC_22",63,"OID30256","Q6YN16","HSDL2",0,"Cardiometabolic_II","B13805","20212097_SS211647","PASS",-1.9867,0.4696,"Plate control","PASS","PFC",1.38472548745537,"24h","24h"
5584,"PFC_07",73,"OID30256","Q6YN16","HSDL2",0,"Cardiometabolic_II","B13805","20212097_SS211647","PASS",-1.9867,0.6364,"Plate control","PASS","PFC",1.5544454616222,"Baseline","Baseline"
5585,"PFC_15",74,"OID30256","Q6YN16","HSDL2",0,"Cardiometabolic_II","B13805","20212097_SS211647","PASS",-1.9867,0.3367,"Plate control","PASS","PFC",1.26286462922313,"1h","1h"
5586,"PFC_23",75,"OID30256","Q6YN16","HSDL2",0,"Cardiometabolic_II","B13805","20212097_SS211647","PASS",-1.9867,0.499,"Plate control","PASS","PFC",1.41323364388273,"24h","24h"
5587,"PFC_08",85,"OID30256","Q6YN16","HSDL2",0,"Cardiometabolic_II","B13805","20212097_SS211647","PASS",-1.9867,0.0853,"Plate control","PASS","PFC",1.0609083281175,"Baseline","Baseline"
5588,"PFC_16",86,"OID30256","Q6YN16","HSDL2",0,"Cardiometabolic_II","B13805","20212097_SS211647","PASS",-1.9867,0.4453,"Plate control","PASS","PFC",1.36159722324616,"1h","1h"
5589,"PFC_24",87,"OID30256","Q6YN16","HSDL2",0,"Cardiometabolic_II","B13805","20212097_SS211647","PASS",-1.9867,0.8748,"Plate control","PASS","PFC",1.83375385652478,"24h","24h"
5590,"PFC_01",1,"OID30257","Q6ZRY4","RBPMS2",0,"Cardiometabolic_II","B13805","20212097_SS211647","PASS",-2.122,2.2685,"Plate control","PASS","PFC",4.81821910308642,"Baseline","Baseline"
5591,"PFC_09",2,"OID30257","Q6ZRY4","RBPMS2",0,"Cardiometabolic_II","B13805","20212097_SS211647","PASS",-2.122,4.8913,"Plate control","PASS","PFC",29.6775481169802,"Baseline","Baseline"
5592,"PFC_17",3,"OID30257","Q6ZRY4","RBPMS2",0,"Cardiometabolic_II","B13805","20212097_SS211647","PASS",-2.122,4.9126,"Plate control","PASS","PFC",30.1189589589585,"1h","1h"
5593,"PFC_25",4,"OID30257","Q6ZRY4","RBPMS2",0,"Cardiometabolic_II","B13805","20212097_SS211647","PASS",-2.122,4.8358,"Plate control","PASS","PFC",28.5575438965043,"24h","24h"
5594,"PFC_02",13,"OID30257","Q6ZRY4","RBPMS2",0,"Cardiometabolic_II","B13805","20212097_SS211647","PASS",-2.122,3.3098,"Plate control","PASS","PFC",9.91628681497181,"Baseline","Baseline"
5595,"PFC_10",14,"OID30257","Q6ZRY4","RBPMS2",0,"Cardiometabolic_II","B13805","20212097_SS211647","PASS",-2.122,3.4561,"Plate control","PASS","PFC",10.9746269855367,"1h","1h"
5596,"PFC_18",15,"OID30257","Q6ZRY4","RBPMS2",0,"Cardiometabolic_II","B13805","20212097_SS211647","PASS",-2.122,4.7248,"Plate control","PASS","PFC",26.4427441841176,"1h","1h"
5597,"PFC_26",16,"OID30257","Q6ZRY4","RBPMS2",0,"Cardiometabolic_II","B13805","20212097_SS211647","PASS",-2.122,4.69,"Plate control","PASS","PFC",25.812536295108,"24h","24h"
5598,"PFC_03",25,"OID30257","Q6ZRY4","RBPMS2",0,"Cardiometabolic_II","B13805","20212097_SS211647","PASS",-2.122,1.937,"Plate control","PASS","PFC",3.82908583278119,"Baseline","Baseline"
5599,"PFC_11",26,"OID30257","Q6ZRY4","RBPMS2",0,"Cardiometabolic_II","B13805","20212097_SS211647","PASS",-2.122,5.1379,"Plate control","PASS","PFC",35.2096748664452,"1h","1h"
5600,"PFC_19",27,"OID30257","Q6ZRY4","RBPMS2",0,"Cardiometabolic_II","B13805","20212097_SS211647","PASS",-2.122,4.3773,"Plate control","PASS","PFC",20.7825388018354,"24h","24h"

Supplemental table 1_Olink proximity extension assay (PEA) data

,"SampleID","Index","OlinkID","UniProt","Assay","MissingFreq","Panel","Panel_Lot_Nr","PlateID","QC_Warning","LOD","NPX","Normalization","Assay_Warning","condition","Value","group","alt_group"
5601,"PFC_27",28,"OID30257","Q6ZRY4","RBPMS2",0,"Cardiometabolic_II","B13805","20212097_SS211647","PASS",-2.122,5.6772,"Plate control","PASS","PFC",51.1690661896866,"24h","24h"
5602,"PFC_04",37,"OID30257","Q6ZRY4","RBPMS2",0,"Cardiometabolic_II","B13805","20212097_SS211647","PASS",-2.122,4.5554,"Plate control","PASS","PFC",23.5132164766595,"Baseline","Baseline"
5603,"PFC_12",38,"OID30257","Q6ZRY4","RBPMS2",0,"Cardiometabolic_II","B13805","20212097_SS211647","PASS",-2.122,2.5261,"Plate control","PASS","PFC",5.7601245216289,"1h","1h"
5604,"PFC_20",39,"OID30257","Q6ZRY4","RBPMS2",0,"Cardiometabolic_II","B13805","20212097_SS211647","PASS",-2.122,5.4228,"Plate control","PASS","PFC",42.8968557514294,"24h","24h"
5605,"PFC_05",49,"OID30257","Q6ZRY4","RBPMS2",0,"Cardiometabolic_II","B13805","20212097_SS211647","PASS",-2.122,3.6005,"Plate control","PASS","PFC",12.1299357190563,"Baseline","Baseline"
5606,"PFC_13",50,"OID30257","Q6ZRY4","RBPMS2",0,"Cardiometabolic_II","B13805","20212097_SS211647","PASS",-2.122,4.9021,"Plate control","PASS","PFC",29.9005475764594,"1h","1h"
5607,"PFC_21",51,"OID30257","Q6ZRY4","RBPMS2",0,"Cardiometabolic_II","B13805","20212097_SS211647","PASS",-2.122,3.3571,"Plate control","PASS","PFC",10.246789119322,"24h","24h"
5608,"PFC_06",61,"OID30257","Q6ZRY4","RBPMS2",0,"Cardiometabolic_II","B13805","20212097_SS211647","PASS",-2.122,3.7745,"Plate control","PASS","PFC",13.6847768067351,"Baseline","Baseline"
5609,"PFC_14",62,"OID30257","Q6ZRY4","RBPMS2",0,"Cardiometabolic_II","B13805","20212097_SS211647","PASS",-2.122,5.0894,"Plate control","PASS","PFC",34.0456836919793,"1h","1h"
5610,"PFC_22",63,"OID30257","Q6ZRY4","RBPMS2",0,"Cardiometabolic_II","B13805","20212097_SS211647","PASS",-2.122,4.4643,"Plate control","PASS","PFC",22.074364507066,"24h","24h"
5611,"PFC_07",73,"OID30257","Q6ZRY4","RBPMS2",0,"Cardiometabolic_II","B13805","20212097_SS211647","PASS",-2.122,4.858,"Plate control","PASS","PFC",29.0003820245547,"Baseline","Baseline"
5612,"PFC_15",74,"OID30257","Q6ZRY4","RBPMS2",0,"Cardiometabolic_II","B13805","20212097_SS211647","PASS",-2.122,3.2044,"Plate control","PASS","PFC",9.21765646157062,"1h","1h"
5613,"PFC_23",75,"OID30257","Q6ZRY4","RBPMS2",0,"Cardiometabolic_II","B13805","20212097_SS211647","PASS",-2.122,5.1052,"Plate control","PASS","PFC",34.4205918711436,"24h","24h"
5614,"PFC_08",85,"OID30257","Q6ZRY4","RBPMS2",0,"Cardiometabolic_II","B13805","20212097_SS211647","PASS",-2.122,3.791,"Plate control","PASS","PFC",13.8421870493598,"Baseline","Baseline"
5615,"PFC_16",86,"OID30257","Q6ZRY4","RBPMS2",0,"Cardiometabolic_II","B13805","20212097_SS211647","PASS",-2.122,5.0682,"Plate control","PASS","PFC",33.5490497800349,"1h","1h"
5616,"PFC_24",87,"OID30257","Q6ZRY4","RBPMS2",0,"Cardiometabolic_II","B13805","20212097_SS211647","PASS",-2.122,5.1061,"Plate control","PASS","PFC",34.4420712528026,"24h","24h"
5617,"PFC_01",1,"OID30258","P43487","RANBP1",0,"Cardiometabolic_II","B13805","20212097_SS211647","PASS",-2.9298,2.6761,"Plate control","PASS","PFC",6.39125832450883,"Baseline","Baseline"
5618,"PFC_09",2,"OID30258","P43487","RANBP1",0,"Cardiometabolic_II","B13805","20212097_SS211647","PASS",-2.9298,2.5825,"Plate control","PASS","PFC",5.98976748270851,"Baseline","Baseline"
5619,"PFC_17",3,"OID30258","P43487","RANBP1",0,"Cardiometabolic_II","B13805","20212097_SS211647","PASS",-2.9298,3.1721,"Plate control","PASS","PFC",9.01357857998368,"1h","1h"
5620,"PFC_25",4,"OID30258","P43487","RANBP1",0,"Cardiometabolic_II","B13805","20212097_SS211647","PASS",-2.9298,3.1152,"Plate control","PASS","PFC",8.6650015052213,"24h","24h"
5621,"PFC_02",13,"OID30258","P43487","RANBP1",0,"Cardiometabolic_II","B13805","20212097_SS211647","PASS",-2.9298,2.5119,"Plate control","PASS","PFC",5.70370750487685,"Baseline","Baseline"
5622,"PFC_10",14,"OID30258","P43487","RANBP1",0,"Cardiometabolic_II","B13805","20212097_SS211647","PASS",-2.9298,2.6386,"Plate control","PASS","PFC",6.22727072306058,"1h","1h"
5623,"PFC_18",15,"OID30258","P43487","RANBP1",0,"Cardiometabolic_II","B13805","20212097_SS211647","PASS",-2.9298,2.5193,"Plate control","PASS","PFC",5.73303862941909,"1h","1h"
5624,"PFC_26",16,"OID30258","P43487","RANBP1",0,"Cardiometabolic_II","B13805","20212097_SS211647","PASS",-2.9298,3.2152,"Plate control","PASS","PFC",9.28691866613323,"24h","24h"
5625,"PFC_03",25,"OID30258","P43487","RANBP1",0,"Cardiometabolic_II","B13805","20212097_SS211647","PASS",-2.9298,1.8138,"Plate control","PASS","PFC",3.51567083439797,"Baseline","Baseline"
5626,"PFC_11",26,"OID30258","P43487","RANBP1",0,"Cardiometabolic_II","B13805","20212097_SS211647","PASS",-2.9298,2.8181,"Plate control","PASS","PFC",7.05233007658708,"1h","1h"
5627,"PFC_19",27,"OID30258","P43487","RANBP1",0,"Cardiometabolic_II","B13805","20212097_SS211647","PASS",-2.9298,3.0686,"Plate control","PASS","PFC",8.38958821033991,"24h","24h"
5628,"PFC_27",28,"OID30258","P43487","RANBP1",0,"Cardiometabolic_II","B13805","20212097_SS211647","PASS",-2.9298,3.2588,"Plate control","PASS","PFC",9.57186467166287,"24h","24h"
5629,"PFC_04",37,"OID30258","P43487","RANBP1",0,"Cardiometabolic_II","B13805","20212097_SS211647","PASS",-2.9298,3.0904,"Plate control","PASS","PFC",8.51732263555157,"Baseline","Baseline"
5630,"PFC_12",38,"OID30258","P43487","RANBP1",0,"Cardiometabolic_II","B13805","20212097_SS211647","PASS",-2.9298,2.4681,"Plate control","PASS","PFC",5.5331460312092,"1h","1h"
5631,"PFC_20",39,"OID30258","P43487","RANBP1",0,"Cardiometabolic_II","B13805","20212097_SS211647","PASS",-2.9298,2.9826,"Plate control","PASS","PFC",7.9040934281443,"24h","24h"
5632,"PFC_05",49,"OID30258","P43487","RANBP1",0,"Cardiometabolic_II","B13805","20212097_SS211647","PASS",-2.9298,2.1361,"Plate control","PASS","PFC",4.39572154794782,"Baseline","Baseline"
5633,"PFC_13",50,"OID30258","P43487","RANBP1",0,"Cardiometabolic_II","B13805","20212097_SS211647","PASS",-2.9298,3.223,"Plate control","PASS","PFC",9.33726481454774,"1h","1h"
5634,"PFC_21",51,"OID30258","P43487","RANBP1",0,"Cardiometabolic_II","B13805","20212097_SS211647","PASS",-2.9298,2.5827,"Plate control","PASS","PFC",5.99059789835578,"24h","24h"
5635,"PFC_06",61,"OID30258","P43487","RANBP1",0,"Cardiometabolic_II","B13805","20212097_SS211647","PASS",-2.9298,3.1166,"Plate control","PASS","PFC",8.67341415631294,"Baseline","Baseline"
5636,"PFC_14",62,"OID30258","P43487","RANBP1",0,"Cardiometabolic_II","B13805","20212097_SS211647","PASS",-2.9298,2.3424,"Plate control","PASS","PFC",5.07145599939893,"1h","1h"
5637,"PFC_22",63,"OID30258","P43487","RANBP1",0,"Cardiometabolic_II","B13805","20212097_SS211647","PASS",-2.9298,2.9109,"Plate control","PASS","PFC",7.52087229486281,"24h","24h"
5638,"PFC_07",73,"OID30258","P43487","RANBP1",0,"Cardiometabolic_II","B13805","20212097_SS211647","PASS",-2.9298,3.4334,"Plate control","PASS","PFC",10.8032987762171,"Baseline","Baseline"
5639,"PFC_15",74,"OID30258","P43487","RANBP1",0,"Cardiometabolic_II","B13805","20212097_SS211647","PASS",-2.9298,2.1259,"Plate control","PASS","PFC",4.36475295611367,"1h","1h"
5640,"PFC_23",75,"OID30258","P43487","RANBP1",0,"Cardiometabolic_II","B13805","20212097_SS211647","PASS",-2.9298,2.5367,"Plate control","PASS","PFC",5.80260208869022,"24h","24h"
5641,"PFC_08",85,"OID30258","P43487","RANBP1",0,"Cardiometabolic_II","B13805","20212097_SS211647","PASS",-2.9298,2.5082,"Plate control","PASS","PFC",5.68909826416908,"Baseline","Baseline"
5642,"PFC_16",86,"OID30258","P43487","RANBP1",0,"Cardiometabolic_II","B13805","20212097_SS211647","PASS",-2.9298,3.1592,"Plate control","PASS","PFC",8.93334203419232,"1h","1h"
5643,"PFC_24",87,"OID30258","P43487","RANBP1",0,"Cardiometabolic_II","B13805","20212097_SS211647","PASS",-2.9298,2.9986,"Plate control","PASS","PFC",7.99224051711123,"24h","24h"
5644,"PFC_01",1,"OID30259","Q92835","INPP5D",0,"Cardiometabolic_II","B13805","20212097_SS211647","PASS",-2.8778,1.9285,"Plate control","PASS","PFC",3.80659214104758,"Baseline","Baseline"
5645,"PFC_09",2,"OID30259","Q92835","INPP5D",0,"Cardiometabolic_II","B13805","20212097_SS211647","PASS",-2.8778,2.0939,"Plate control","PASS","PFC",4.26900543699608,"Baseline","Baseline"
5646,"PFC_17",3,"OID30259","Q92835","INPP5D",0,"Cardiometabolic_II","B13805","20212097_SS211647","PASS",-2.8778,2.3301,"Plate control","PASS","PFC",5.0284020282769,"1h","1h"
5647,"PFC_25",4,"OID30259","Q92835","INPP5D",0,"Cardiometabolic_II","B13805","20212097_SS211647","PASS",-2.8778,2.1616,"Plate control","PASS","PFC",4.47410774609118,"24h","24h"
5648,"PFC_02",13,"OID30259","Q92835","INPP5D",0,"Cardiometabolic_II","B13805","20212097_SS211647","PASS",-2.8778,2.0207,"Plate control","PASS","PFC",4.05780630148696,"Baseline","Baseline"
5649,"PFC_10",14,"OID30259","Q92835","INPP5D",0,"Cardiometabolic_II","B13805","20212097_SS211647","PASS",-2.8778,1.4667,"Plate control","PASS","PFC",2.76388961860771,"1h","1h"
5650,"PFC_18",15,"OID30259","Q92835","INPP5D",0,"Cardiometabolic_II","B13805","20212097_SS211647","PASS",-2.8778,2.0919,"Plate control","PASS","PFC",4.26309143904931,"1h","1h"
5651,"PFC_26",16,"OID30259","Q92835","INPP5D",0,"Cardiometabolic_II","B13805","20212097_SS211647","PASS",-2.8778,1.7634,"Plate control","PASS","PFC",3.39497276266506,"24h","24h"
5652,"PFC_03",25,"OID30259","Q92835","INPP5D",0,"Cardiometabolic_II","B13805","20212097_SS211647","PASS",-2.8778,0.4787,"Plate control","PASS","PFC",1.39348744099038,"Baseline","Baseline"
5653,"PFC_11",26,"OID30259","Q92835","INPP5D",0,"Cardiometabolic_II","B13805","20212097_SS211647","PASS",-2.8778,2.7508,"Plate control","PASS","PFC",6.73090269235764,"1h","1h"
5654,"PFC_19",27,"OID30259","Q92835","INPP5D",0,"Cardiometabolic_II","B13805","20212097_SS211647","PASS",-2.8778,2.3476,"Plate control","PASS","PFC",5.08976836201819,"24h","24h"
5655,"PFC_27",28,"OID30259","Q92835","INPP5D",0,"Cardiometabolic_II","B13805","20212097_SS211647","PASS",-2.8778,3.4409,"Plate control","PASS","PFC",10.8596070823487,"24h","24h"
5656,"PFC_04",37,"OID30259","Q92835","INPP5D",0,"Cardiometabolic_II","B13805","20212097_SS211647","PASS",-2.8778,2.1722,"Plate control","PASS","PFC",4.50710168764756,"Baseline","Baseline"
5657,"PFC_12",38,"OID30259","Q92835","INPP5D",0,"Cardiometabolic_II","B13805","20212097_SS211647","PASS",-2.8778,0.9765,"Plate control","PASS","PFC",1.96768597788746,"1h","1h"
5658,"PFC_20",39,"OID30259","Q92835","INPP5D",0,"Cardiometabolic_II","B13805","20212097_SS211647","PASS",-2.8778,2.9311,"Plate control","PASS","PFC",7.62691700158542,"24h","24h"
5659,"PFC_05",49,"OID30259","Q92835","INPP5D",0,"Cardiometabolic_II","B13805","20212097_SS211647","PASS",-2.8778,1.3859,"Plate control","PASS","PFC",2.61334935793432,"Baseline","Baseline"
5660,"PFC_13",50,"OID30259","Q92835","INPP5D",0,"Cardiometabolic_II","B13805","20212097_SS211647","PASS",-2.8778,2.2088,"Plate control","PASS","PFC",4.62290591095573,"1h","1h"
5661,"PFC_21",51,"OID30259","Q92835","INPP5D",0,"Cardiometabolic_II","B13805","20212097_SS211647","PASS",-2.8778,1.0375,"Plate control","PASS","PFC",2.05266756778085,"24h","24h"
5662,"PFC_06",61,"OID30259","Q92835","INPP5D",0,"Cardiometabolic_II","B13805","20212097_SS211647","PASS",-2.8778,1.6593,"Plate control","PASS","PFC",3.15863229763756,"Baseline","Baseline"
5663,"PFC_14",62,"OID30259","Q92835","INPP5D",0,"Cardiometabolic_II","B13805","20212097_SS211647","PASS",-2.8778,2.036,"Plate control","PASS","PFC",4.10106895155437,"1h","1h"
5664,"PFC_22",63,"OID30259","Q92835","INPP5D",0,"Cardiometabolic_II","B13805","20212097_SS211647","PASS",-2.8778,1.8298,"Plate control","PASS","PFC",3.55487787978974,"24h","24h"
5665,"PFC_07",73,"OID30259","Q92835","INPP5D",0,"Cardiometabolic_II","B13805","20212097_SS211647","PASS",-2.8778,2.2705,"Plate control","PASS","PFC",4.82490320505563,"Baseline","Baseline"
5666,"PFC_15",74,"OID30259","Q92835","INPP5D",0,"Cardiometabolic_II","B13805","20212097_SS211647","PASS",-2.8778,1.0485,"Plate control","PASS","PFC",2.06837819354706,"1h","1h"
5667,"PFC_23",75,"OID30259","Q92835","INPP5D",0,"Cardiometabolic_II","B13805","20212097_SS211647","PASS",-2.8778,2.1863,"Plate control","PASS","PFC",4.55136724330624,"24h","24h"
5668,"PFC_08",85,"OID30259","Q92835","INPP5D",0,"Cardiometabolic_II","B13805","20212097_SS211647","PASS",-2.8778,1.0763,"Plate control","PASS","PFC",2.10862127646323,"Baseline","Baseline"
5669,"PFC_16",86,"OID30259","Q92835","INPP5D",0,"Cardiometabolic_II","B13805","20212097_SS211647","PASS",-2.8778,2.0819,"Plate control","PASS","PFC",4.23364411548175,"1h","1h"
5670,"PFC_24",87,"OID30259","Q92835","INPP5D",0,"Cardiometabolic_II","B13805","20212097_SS211647","PASS",-2.8778,2.1668,"Plate control","PASS","PFC",4.49026316249502,"24h","24h"
5671,"PFC_01",1,"OID30260","Q09666","AHNAK",0,"Cardiometabolic_II","B13805","20212097_SS211647","PASS",-4.9506,0.111,"Plate control","PASS","PFC",1.07997655983288,"Baseline","Baseline"
5672,"PFC_09",2,"OID30260","Q09666","AHNAK",0,"Cardiometabolic_II","B13805","20212097_SS211647","PASS",-4.9506,-0.032,"Plate control","PASS","PFC",0.978063473447339,"Baseline","Baseline"
5673,"PFC_17",3,"OID30260","Q09666","AHNAK",0,"Cardiometabolic_II","B13805","20212097_SS211647","PASS",-4.9506,0.0465,"Plate control","PASS","PFC",1.03275639955982,"1h","1h"
5674,"PFC_25",4,"OID30260","Q09666","AHNAK",0,"Cardiometabolic_II","B13805","20212097_SS211647","PASS",-4.9506,-0.3247,"Plate control","PASS","PFC",0.79846440510009,"24h","24h"
5675,"PFC_02",13,"OID30260","Q09666","AHNAK",0,"Cardiometabolic_II","B13805","20212097_SS211647","PASS",-4.9506,0.5538,"Plate control","PASS","PFC",1.4679471212462,"Baseline","Baseline"
5676,"PFC_10",14,"OID30260","Q09666","AHNAK",0,"Cardiometabolic_II","B13805","20212097_SS211647","PASS",-4.9506,0.5541,"Plate control","PASS","PFC",1.46825240400847,"1h","1h"
5677,"PFC_18",15,"OID30260","Q09666","AHNAK",0,"Cardiometabolic_II","B13805","20212097_SS211647","PASS",-4.9506,0.4481,"Plate control","PASS","PFC",1.36424239367904,"1h","1h"
5678,"PFC_26",16,"OID30260","Q09666","AHNAK",0,"Cardiometabolic_II","B13805","20212097_SS211647","PASS",-4.9506,-0.3058,"Plate control","PASS","PFC",0.808993490899893,"24h","24h"
5679,"PFC_03",25,"OID30260","Q09666","AHNAK",0,"Cardiometabolic_II","B13805","20212097_SS211647","PASS",-4.9506,0.0332,"Plate control","PASS","PFC",1.02327931653677,"Baseline","Baseline"
5680,"PFC_11",26,"OID30260","Q09666","AHNAK",0,"Cardiometabolic_II","B13805","20212097_SS211647","PASS",-4.9506,0.9718,"Plate control","PASS","PFC",1.96128609714441,"1h","1h"
5681,"PFC_19",27,"OID30260","Q09666","AHNAK",0,"Cardiometabolic_II","B13805","20212097_SS211647","PASS",-4.9506,0.2411,"Plate control","PASS","PFC",1.18189346670763,"24h","24h"
5682,"PFC_27",28,"OID30260","Q09666","AHNAK",0,"Cardiometabolic_II","B13805","20212097_SS211647","PASS",-4.9506,0.2872,"Plate control","PASS","PFC",1.22026966384252,"24h","24h"
5683,"PFC_04",37,"OID30260","Q09666","AHNAK",0,"Cardiometabolic_II","B13805","20212097_SS211647","PASS",-4.9506,0.6382,"Plate control","PASS","PFC",1.55638609908341,"Baseline","Baseline"
5684,"PFC_12",38,"OID30260","Q09666","AHNAK",0,"Cardiometabolic_II","B13805","20212097_SS211647","PASS",-4.9506,0.3684,"Plate control","PASS","PFC",1.29092035996137,"1h","1h"
5685,"PFC_20",39,"OID30260","Q09666","AHNAK",0,"Cardiometabolic_II","B13805","20212097_SS211647","PASS",-4.9506,0.4773,"Plate control","PASS","PFC",1.39213584824612,"24h","24h"
5686,"PFC_05",49,"OID30260","Q09666","AHNAK",0,"Cardiometabolic_II","B13805","20212097_SS211647","PASS",-4.9506,0.4661,"Plate control","PASS","PFC",1.38137019411105,"Baseline","Baseline"
5687,"PFC_13",50,"OID30260","Q09666","AHNAK",0,"Cardiometabolic_II","B13805","20212097_SS211647","PASS",-4.9506,0.2051,"Plate control","PASS","PFC",1.15276624769712,"1h","1h"
5688,"PFC_21",51,"OID30260","Q09666","AHNAK",0,"Cardiometabolic_II","B13805","20212097_SS211647","PASS",-4.9506,0.2686,"Plate control","PASS","PFC",1.20463827204,"24h","24h"
5689,"PFC_06",61,"OID30260","Q09666","AHNAK",0,"Cardiometabolic_II","B13805","20212097_SS211647","PASS",-4.9506,-0.0728,"Plate control","PASS","PFC",0.950790899700319,"Baseline","Baseline"
5690,"PFC_14",62,"OID30260","Q09666","AHNAK",0,"Cardiometabolic_II","B13805","20212097_SS211647","PASS",-4.9506,0.8512,"Plate control","PASS","PFC",1.80400082704795,"1h","1h"
5691,"PFC_22",63,"OID30260","Q09666","AHNAK",0,"Cardiometabolic_II","B13805","20212097_SS211647","PASS",-4.9506,0.2376,"Plate control","PASS","PFC",1.17902965050834,"24h","24h"
5692,"PFC_07",73,"OID30260","Q09666","AHNAK",0,"Cardiometabolic_II","B13805","20212097_SS211647","PASS",-4.9506,-0.0353,"Plate control","PASS","PFC",0.975828821778009,"Baseline","Baseline"
5693,"PFC_15",74,"OID30260","Q09666","AHNAK",0,"Cardiometabolic_II","B13805","20212097_SS211647","PASS",-4.9506,-0.1699,"Plate control","PASS","PFC",0.888904293181696,"1h","1h"
5694,"PFC_23",75,"OID30260","Q09666","AHNAK",0,"Cardiometabolic_II","B13805","20212097_SS211647","PASS",-4.9506,0.5565,"Plate control","PASS","PFC",1.47069695279729,"24h","24h"
5695,"PFC_08",85,"OID30260","Q09666","AHNAK",0,"Cardiometabolic_II","B13805","20212097_SS211647","PASS",-4.9506,-0.0999,"Plate control","PASS","PFC",0.933097666696997,"Baseline","Baseline"
5696,"PFC_16",86,"OID30260","Q09666","AHNAK",0,"Cardiometabolic_II","B13805","20212097_SS211647","PASS",-4.9506,0.1097,"Plate control","PASS","PFC",1.07900384063335,"1h","1h"
5697,"PFC_24",87,"OID30260","Q09666","AHNAK",0,"Cardiometabolic_II","B13805","20212097_SS211647","PASS",-4.9506,-0.0892,"Plate control","PASS","PFC",0.940043875723358,"24h","24h"
5698,"PFC_01",1,"OID30261","Q9NYZ4","SIGLEC8",0,"Cardiometabolic_II","B13805","20212097_SS211647","PASS",-6.2129,0.4655,"Plate control","PASS","PFC",1.3807958178443,"Baseline","Baseline"
5699,"PFC_09",2,"OID30261","Q9NYZ4","SIGLEC8",0,"Cardiometabolic_II","B13805","20212097_SS211647","PASS",-6.2129,0.4374,"Plate control","PASS","PFC",1.35416168034869,"Baseline","Baseline"
5700,"PFC_17",3,"OID30261","Q9NYZ4","SIGLEC8",0,"Cardiometabolic_II","B13805","20212097_SS211647","PASS",-6.2129,-0.0432,"Plate control","PASS","PFC",0.970499920586786,"1h","1h"
5701,"PFC_25",4,"OID30261","Q9NYZ4","SIGLEC8",0,"Cardiometabolic_II","B13805","20212097_SS211647","PASS",-6.2129,-0.1225,"Plate control","PASS","PFC",0.918594467722301,"24h","24h"
5702,"PFC_02",13,"OID30261","Q9NYZ4","SIGLEC8",0,"Cardiometabolic_II","B13805","20212097_SS211647","PASS",-6.2129,0.077,"Plate control","PASS","PFC",1.05482231706806,"Baseline","Baseline"
5703,"PFC_10",14,"OID30261","Q9NYZ4","SIGLEC8",0,"Cardiometabolic_II","B13805","20212097_SS211647","PASS",-6.2129,0.4571,"Plate control","PASS","PFC",1.37277958178687,"1h","1h"
5704,"PFC_18",15,"OID30261","Q9NYZ4","SIGLEC8",0,"Cardiometabolic_II","B13805","20212097_SS211647","PASS",-6.2129,0.1495,"Plate control","PASS","PFC",1.10918499122155,"1h","1h"
5705,"PFC_26",16,"OID30261","Q9NYZ4","SIGLEC8",0,"Cardiometabolic_II","B13805","20212097_SS211647","PASS",-6.2129,-0.0826,"Plate control","PASS","PFC",0.944354213440377,"24h","24h"
5706,"PFC_03",25,"OID30261","Q9NYZ4","SIGLEC8",0,"Cardiometabolic_II","B13805","20212097_SS211647","PASS",-6.2129,0.3739,"Plate control","PASS","PFC",1.29585114076382,"Baseline","Baseline"
5707,"PFC_11",26,"OID30261","Q9NYZ4","SIGLEC8",0,"Cardiometabolic_II","B13805","20212097_SS211647","PASS",-6.2129,-0.0014,"Plate control","PASS","PFC",0.999030064638903,"1h","1h"
5708,"PFC_19",27,"OID30261","Q9NYZ4","SIGLEC8",0,"Cardiometabolic_II","B13805","20212097_SS211647","PASS",-6.2129,0.4645,"Plate control","PASS","PFC",1.37983905474336,"24h","24h"
5709,"PFC_27",28,"OID30261","Q9NYZ4","SIGLEC8",0,"Cardiometabolic_II","B13805","20212097_SS211647","PASS",-6.2129,0.6363,"Plate control","PASS","PFC",1.5543377194074,"24h","24h"
5710,"PFC_04",37,"OID30261","Q9NYZ4","SIGLEC8",0,"Cardiometabolic_II","B13805","20212097_SS211647","PASS",-6.2129,-0.0252,"Plate control","PASS","PFC",0.982684360123421,"Baseline","Baseline"
5711,"PFC_12",38,"OID30261","Q9NYZ4","SIGLEC8",0,"Cardiometabolic_II","B13805","20212097_SS211647","PASS",-6.2129,0.4973,"Plate control","PASS","PFC",1.41156934048469,"1h","1h"
5712,"PFC_20",39,"OID30261","Q9NYZ4","SIGLEC8",0,"Cardiometabolic_II","B13805","20212097_SS211647","PASS",-6.2129,0.018,"Plate control","PASS","PFC",1.01255480735049,"24h","24h"

Supplemental table 1_Olink proximity extension assay (PEA) data

,"SampleID","Index","OlinkID","UniProt","Assay","MissingFreq","Panel","Panel_Lot_Nr","PlateID","QC_Warning","LOD","NPX","Normalization","Assay_Warning","condition","Value","group","alt_group"
5713,"PFC_05",49,"OID30261","Q9NYZ4","SIGLEC8",0,"Cardiometabolic_II","B13805","20212097_SS211647","PASS",-6.2129,1.3181,"Plate control","PASS","PFC",2.49337521016029,"Baseline","Baseline"
5714,"PFC_13",50,"OID30261","Q9NYZ4","SIGLEC8",0,"Cardiometabolic_II","B13805","20212097_SS211647","PASS",-6.2129,-0.1603,"Plate control","PASS","PFC",0.894838975046085,"1h","1h"
5715,"PFC_21",51,"OID30261","Q9NYZ4","SIGLEC8",0,"Cardiometabolic_II","B13805","20212097_SS211647","PASS",-6.2129,0.5159,"Plate control","PASS","PFC",1.42988587079066,"24h","24h"
5716,"PFC_06",61,"OID30261","Q9NYZ4","SIGLEC8",0,"Cardiometabolic_II","B13805","20212097_SS211647","PASS",-6.2129,0.114,"Plate control","PASS","PFC",1.08222464452571,"Baseline","Baseline"
5717,"PFC_14",62,"OID30261","Q9NYZ4","SIGLEC8",0,"Cardiometabolic_II","B13805","20212097_SS211647","PASS",-6.2129,1.2559,"Plate control","PASS","PFC",2.38816083371719,"1h","1h"
5718,"PFC_22",63,"OID30261","Q9NYZ4","SIGLEC8",0,"Cardiometabolic_II","B13805","20212097_SS211647","PASS",-6.2129,-0.2144,"Plate control","PASS","PFC",0.861904541205104,"24h","24h"
5719,"PFC_07",73,"OID30261","Q9NYZ4","SIGLEC8",0,"Cardiometabolic_II","B13805","20212097_SS211647","PASS",-6.2129,0.1885,"Plate control","PASS","PFC",1.13957825651856,"Baseline","Baseline"
5720,"PFC_15",74,"OID30261","Q9NYZ4","SIGLEC8",0,"Cardiometabolic_II","B13805","20212097_SS211647","PASS",-6.2129,0.1358,"Plate control","PASS","PFC",1.0987018945948,"1h","1h"
5721,"PFC_23",75,"OID30261","Q9NYZ4","SIGLEC8",0,"Cardiometabolic_II","B13805","20212097_SS211647","PASS",-6.2129,1.2296,"Plate control","PASS","PFC",2.3450196308235,"24h","24h"
5722,"PFC_08",85,"OID30261","Q9NYZ4","SIGLEC8",0,"Cardiometabolic_II","B13805","20212097_SS211647","PASS",-6.2129,-0.2835,"Plate control","PASS","PFC",0.82159539468121,"Baseline","Baseline"
5723,"PFC_16",86,"OID30261","Q9NYZ4","SIGLEC8",0,"Cardiometabolic_II","B13805","20212097_SS211647","PASS",-6.2129,0.0324,"Plate control","PASS","PFC",1.02271204729319,"1h","1h"
5724,"PFC_24",87,"OID30261","Q9NYZ4","SIGLEC8",0,"Cardiometabolic_II","B13805","20212097_SS211647","PASS",-6.2129,0.3277,"Plate control","PASS","PFC",1.25501099194775,"24h","24h"
5725,"PFC_01",1,"OID30262","O60476","MAN1A2",0,"Cardiometabolic_II","B13805","20212097_SS211647","PASS",-5.4543,0.9133,"Plate control","PASS","PFC",1.88334851968144,"Baseline","Baseline"
5726,"PFC_09",2,"OID30262","O60476","MAN1A2",0,"Cardiometabolic_II","B13805","20212097_SS211647","PASS",-5.4543,0.919,"Plate control","PASS","PFC",1.89080423350149,"Baseline","Baseline"
5727,"PFC_17",3,"OID30262","O60476","MAN1A2",0,"Cardiometabolic_II","B13805","20212097_SS211647","PASS",-5.4543,0.5177,"Plate control","PASS","PFC",1.43167100263003,"1h","1h"
5728,"PFC_25",4,"OID30262","O60476","MAN1A2",0,"Cardiometabolic_II","B13805","20212097_SS211647","PASS",-5.4543,0.1738,"Plate control","PASS","PFC",1.12802574904323,"24h","24h"
5729,"PFC_02",13,"OID30262","O60476","MAN1A2",0,"Cardiometabolic_II","B13805","20212097_SS211647","PASS",-5.4543,0.5966,"Plate control","PASS","PFC",1.51214868251893,"Baseline","Baseline"
5730,"PFC_10",14,"OID30262","O60476","MAN1A2",0,"Cardiometabolic_II","B13805","20212097_SS211647","PASS",-5.4543,0.7676,"Plate control","PASS","PFC",1.70243533475568,"1h","1h"
5731,"PFC_18",15,"OID30262","O60476","MAN1A2",0,"Cardiometabolic_II","B13805","20212097_SS211647","PASS",-5.4543,0.446,"Plate control","PASS","PFC",1.36225803464049,"1h","1h"
5732,"PFC_26",16,"OID30262","O60476","MAN1A2",0,"Cardiometabolic_II","B13805","20212097_SS211647","PASS",-5.4543,0.4848,"Plate control","PASS","PFC",1.39939185524394,"24h","24h"
5733,"PFC_03",25,"OID30262","O60476","MAN1A2",0,"Cardiometabolic_II","B13805","20212097_SS211647","PASS",-5.4543,0.2374,"Plate control","PASS","PFC",1.17886621362158,"Baseline","Baseline"
5734,"PFC_11",26,"OID30262","O60476","MAN1A2",0,"Cardiometabolic_II","B13805","20212097_SS211647","PASS",-5.4543,0.4023,"Plate control","PASS","PFC",1.32161319882523,"1h","1h"
5735,"PFC_19",27,"OID30262","O60476","MAN1A2",0,"Cardiometabolic_II","B13805","20212097_SS211647","PASS",-5.4543,0.9029,"Plate control","PASS","PFC",1.86982078491048,"24h","24h"
5736,"PFC_27",28,"OID30262","O60476","MAN1A2",0,"Cardiometabolic_II","B13805","20212097_SS211647","PASS",-5.4543,0.8509,"Plate control","PASS","PFC",1.80362573462234,"24h","24h"
5737,"PFC_04",37,"OID30262","O60476","MAN1A2",0,"Cardiometabolic_II","B13805","20212097_SS211647","PASS",-5.4543,0.7574,"Plate control","PASS","PFC",1.69044139373121,"Baseline","Baseline"
5738,"PFC_12",38,"OID30262","O60476","MAN1A2",0,"Cardiometabolic_II","B13805","20212097_SS211647","PASS",-5.4543,0.1006,"Plate control","PASS","PFC",1.07221929329002,"1h","1h"
5739,"PFC_20",39,"OID30262","O60476","MAN1A2",0,"Cardiometabolic_II","B13805","20212097_SS211647","PASS",-5.4543,0.4335,"Plate control","PASS","PFC",1.35050595372236,"24h","24h"
5740,"PFC_05",49,"OID30262","O60476","MAN1A2",0,"Cardiometabolic_II","B13805","20212097_SS211647","PASS",-5.4543,0.3239,"Plate control","PASS","PFC",1.25170969374248,"Baseline","Baseline"
5741,"PFC_13",50,"OID30262","O60476","MAN1A2",0,"Cardiometabolic_II","B13805","20212097_SS211647","PASS",-5.4543,0.5728,"Plate control","PASS","PFC",1.48740754967138,"1h","1h"
5742,"PFC_21",51,"OID30262","O60476","MAN1A2",0,"Cardiometabolic_II","B13805","20212097_SS211647","PASS",-5.4543,0.2816,"Plate control","PASS","PFC",1.21554221659379,"24h","24h"
5743,"PFC_06",61,"OID30262","O60476","MAN1A2",0,"Cardiometabolic_II","B13805","20212097_SS211647","PASS",-5.4543,0.9065,"Plate control","PASS","PFC",1.87449243075384,"Baseline","Baseline"
5744,"PFC_14",62,"OID30262","O60476","MAN1A2",0,"Cardiometabolic_II","B13805","20212097_SS211647","PASS",-5.4543,0.1082,"Plate control","PASS","PFC",1.07788256093833,"1h","1h"
5745,"PFC_22",63,"OID30262","O60476","MAN1A2",0,"Cardiometabolic_II","B13805","20212097_SS211647","PASS",-5.4543,0.7151,"Plate control","PASS","PFC",1.64159700051079,"24h","24h"
5746,"PFC_07",73,"OID30262","O60476","MAN1A2",0,"Cardiometabolic_II","B13805","20212097_SS211647","PASS",-5.4543,0.643,"Plate control","PASS","PFC",1.56157298521478,"Baseline","Baseline"
5747,"PFC_15",74,"OID30262","O60476","MAN1A2",0,"Cardiometabolic_II","B13805","20212097_SS211647","PASS",-5.4543,0.5585,"Plate control","PASS","PFC",1.4727371855447,"1h","1h"
5748,"PFC_23",75,"OID30262","O60476","MAN1A2",0,"Cardiometabolic_II","B13805","20212097_SS211647","PASS",-5.4543,0.2606,"Plate control","PASS","PFC",1.19797682558582,"24h","24h"
5749,"PFC_08",85,"OID30262","O60476","MAN1A2",0,"Cardiometabolic_II","B13805","20212097_SS211647","PASS",-5.4543,0.4692,"Plate control","PASS","PFC",1.3843416132471,"Baseline","Baseline"
5750,"PFC_16",86,"OID30262","O60476","MAN1A2",0,"Cardiometabolic_II","B13805","20212097_SS211647","PASS",-5.4543,0.2259,"Plate control","PASS","PFC",1.16950659722237,"1h","1h"
5751,"PFC_24",87,"OID30262","O60476","MAN1A2",0,"Cardiometabolic_II","B13805","20212097_SS211647","PASS",-5.4543,0.8109,"Plate control","PASS","PFC",1.75430549406054,"24h","24h"
5752,"PFC_01",1,"OID30263","P07355","ANXA2",0,"Cardiometabolic_II","B13805","20212097_SS211647","PASS",-2.803,0.9848,"Plate control","PASS","PFC",1.97903894076043,"Baseline","Baseline"
5753,"PFC_09",2,"OID30263","P07355","ANXA2",0,"Cardiometabolic_II","B13805","20212097_SS211647","PASS",-2.803,0.9242,"Plate control","PASS","PFC",1.89763167965699,"Baseline","Baseline"
5754,"PFC_17",3,"OID30263","P07355","ANXA2",0,"Cardiometabolic_II","B13805","20212097_SS211647","PASS",-2.803,0.2022,"Plate control","PASS","PFC",1.15045136872088,"1h","1h"
5755,"PFC_25",4,"OID30263","P07355","ANXA2",0,"Cardiometabolic_II","B13805","20212097_SS211647","PASS",-2.803,0.7881,"Plate control","PASS","PFC",1.72679880557304,"24h","24h"
5756,"PFC_02",13,"OID30263","P07355","ANXA2",0,"Cardiometabolic_II","B13805","20212097_SS211647","PASS",-2.803,0.9637,"Plate control","PASS","PFC",1.95030532629456,"Baseline","Baseline"
5757,"PFC_10",14,"OID30263","P07355","ANXA2",0,"Cardiometabolic_II","B13805","20212097_SS211647","PASS",-2.803,0.9855,"Plate control","PASS","PFC",1.97999940943614,"1h","1h"
5758,"PFC_18",15,"OID30263","P07355","ANXA2",0,"Cardiometabolic_II","B13805","20212097_SS211647","PASS",-2.803,1.1032,"Plate control","PASS","PFC",2.14830674115011,"1h","1h"
5759,"PFC_26",16,"OID30263","P07355","ANXA2",0,"Cardiometabolic_II","B13805","20212097_SS211647","PASS",-2.803,0.1014,"Plate control","PASS","PFC",1.07281402279288,"24h","24h"
5760,"PFC_03",25,"OID30263","P07355","ANXA2",0,"Cardiometabolic_II","B13805","20212097_SS211647","PASS",-2.803,1.4252,"Plate control","PASS","PFC",2.68551727150144,"Baseline","Baseline"
5761,"PFC_11",26,"OID30263","P07355","ANXA2",0,"Cardiometabolic_II","B13805","20212097_SS211647","PASS",-2.803,1.6884,"Plate control","PASS","PFC",3.22299064307769,"1h","1h"
5762,"PFC_19",27,"OID30263","P07355","ANXA2",0,"Cardiometabolic_II","B13805","20212097_SS211647","PASS",-2.803,1.6234,"Plate control","PASS","PFC",3.08100281389856,"24h","24h"
5763,"PFC_27",28,"OID30263","P07355","ANXA2",0,"Cardiometabolic_II","B13805","20212097_SS211647","PASS",-2.803,1.2616,"Plate control","PASS","PFC",2.3976149753943,"24h","24h"
5764,"PFC_04",37,"OID30263","P07355","ANXA2",0,"Cardiometabolic_II","B13805","20212097_SS211647","PASS",-2.803,2.9221,"Plate control","PASS","PFC",7.57948591651594,"Baseline","Baseline"
5765,"PFC_12",38,"OID30263","P07355","ANXA2",0,"Cardiometabolic_II","B13805","20212097_SS211647","PASS",-2.803,0.5584,"Plate control","PASS","PFC",1.47263510671974,"1h","1h"
5766,"PFC_20",39,"OID30263","P07355","ANXA2",0,"Cardiometabolic_II","B13805","20212097_SS211647","PASS",-2.803,1.7249,"Plate control","PASS","PFC",3.30557213987491,"24h","24h"
5767,"PFC_05",49,"OID30263","P07355","ANXA2",0,"Cardiometabolic_II","B13805","20212097_SS211647","PASS",-2.803,1.4659,"Plate control","PASS","PFC",2.76235741762607,"Baseline","Baseline"
5768,"PFC_13",50,"OID30263","P07355","ANXA2",0,"Cardiometabolic_II","B13805","20212097_SS211647","PASS",-2.803,2.5582,"Plate control","PASS","PFC",5.88972388118112,"1h","1h"
5769,"PFC_21",51,"OID30263","P07355","ANXA2",0,"Cardiometabolic_II","B13805","20212097_SS211647","PASS",-2.803,1.0914,"Plate control","PASS","PFC",2.13080711007045,"24h","24h"
5770,"PFC_06",61,"OID30263","P07355","ANXA2",0,"Cardiometabolic_II","B13805","20212097_SS211647","PASS",-2.803,2.6049,"Plate control","PASS","PFC",6.08349332272508,"Baseline","Baseline"
5771,"PFC_14",62,"OID30263","P07355","ANXA2",0,"Cardiometabolic_II","B13805","20212097_SS211647","PASS",-2.803,1.9402,"Plate control","PASS","PFC",3.83758844314905,"1h","1h"
5772,"PFC_22",63,"OID30263","P07355","ANXA2",0,"Cardiometabolic_II","B13805","20212097_SS211647","PASS",-2.803,2.4361,"Plate control","PASS","PFC",5.41176802637583,"24h","24h"
5773,"PFC_07",73,"OID30263","P07355","ANXA2",0,"Cardiometabolic_II","B13805","20212097_SS211647","PASS",-2.803,1.0474,"Plate control","PASS","PFC",2.06680173505369,"Baseline","Baseline"
5774,"PFC_15",74,"OID30263","P07355","ANXA2",0,"Cardiometabolic_II","B13805","20212097_SS211647","PASS",-2.803,0.7857,"Plate control","PASS","PFC",1.72392857189188,"1h","1h"
5775,"PFC_23",75,"OID30263","P07355","ANXA2",0,"Cardiometabolic_II","B13805","20212097_SS211647","PASS",-2.803,1.9856,"Plate control","PASS","PFC",3.96027331458711,"24h","24h"
5776,"PFC_08",85,"OID30263","P07355","ANXA2",0,"Cardiometabolic_II","B13805","20212097_SS211647","PASS",-2.803,-0.742,"Plate control","PASS","PFC",0.597909898388062,"Baseline","Baseline"
5777,"PFC_16",86,"OID30263","P07355","ANXA2",0,"Cardiometabolic_II","B13805","20212097_SS211647","PASS",-2.803,0.7731,"Plate control","PASS","PFC",1.70893793222519,"1h","1h"
5778,"PFC_24",87,"OID30263","P07355","ANXA2",0,"Cardiometabolic_II","B13805","20212097_SS211647","PASS",-2.803,2.4004,"Plate control","PASS","PFC",5.27949522707895,"24h","24h"
5779,"PFC_01",1,"OID30264","P07492","GRP",0,"Cardiometabolic_II","B13805","20212097_SS211647","PASS",-3.9319,0.7339,"Plate control","PASS","PFC",1.66312891344272,"Baseline","Baseline"
5780,"PFC_09",2,"OID30264","P07492","GRP",0,"Cardiometabolic_II","B13805","20212097_SS211647","PASS",-3.9319,0.4077,"Plate control","PASS","PFC",1.3265692595916,"Baseline","Baseline"
5781,"PFC_17",3,"OID30264","P07492","GRP",0,"Cardiometabolic_II","B13805","20212097_SS211647","PASS",-3.9319,0.1517,"Plate control","PASS","PFC",1.11087770411126,"1h","1h"
5782,"PFC_25",4,"OID30264","P07492","GRP",0,"Cardiometabolic_II","B13805","20212097_SS211647","PASS",-3.9319,0.2793,"Plate control","PASS","PFC",1.21360589626348,"24h","24h"
5783,"PFC_02",13,"OID30264","P07492","GRP",0,"Cardiometabolic_II","B13805","20212097_SS211647","PASS",-3.9319,0.3871,"Plate control","PASS","PFC",1.30776199254904,"Baseline","Baseline"
5784,"PFC_10",14,"OID30264","P07492","GRP",0,"Cardiometabolic_II","B13805","20212097_SS211647","PASS",-3.9319,0.097,"Plate control","PASS","PFC",1.06954708788524,"1h","1h"
5785,"PFC_18",15,"OID30264","P07492","GRP",0,"Cardiometabolic_II","B13805","20212097_SS211647","PASS",-3.9319,-0.051,"Plate control","PASS","PFC",0.965267024871757,"1h","1h"
5786,"PFC_26",16,"OID30264","P07492","GRP",0,"Cardiometabolic_II","B13805","20212097_SS211647","PASS",-3.9319,0.1681,"Plate control","PASS","PFC",1.12357778080967,"24h","24h"
5787,"PFC_03",25,"OID30264","P07492","GRP",0,"Cardiometabolic_II","B13805","20212097_SS211647","PASS",-3.9319,0.1483,"Plate control","PASS","PFC",1.10826278067204,"Baseline","Baseline"
5788,"PFC_11",26,"OID30264","P07492","GRP",0,"Cardiometabolic_II","B13805","20212097_SS211647","PASS",-3.9319,0.1369,"Plate control","PASS","PFC",1.0995399323727,"1h","1h"
5789,"PFC_19",27,"OID30264","P07492","GRP",0,"Cardiometabolic_II","B13805","20212097_SS211647","PASS",-3.9319,0.3125,"Plate control","PASS","PFC",1.24185781207348,"24h","24h"
5790,"PFC_27",28,"OID30264","P07492","GRP",0,"Cardiometabolic_II","B13805","20212097_SS211647","PASS",-3.9319,0.463,"Plate control","PASS","PFC",1.37840515297563,"24h","24h"
5791,"PFC_04",37,"OID30264","P07492","GRP",0,"Cardiometabolic_II","B13805","20212097_SS211647","PASS",-3.9319,0.1803,"Plate control","PASS","PFC",1.13311948637218,"Baseline","Baseline"
5792,"PFC_12",38,"OID30264","P07492","GRP",0,"Cardiometabolic_II","B13805","20212097_SS211647","PASS",-3.9319,-0.6592,"Plate control","PASS","PFC",0.633229336550733,"1h","1h"
5793,"PFC_20",39,"OID30264","P07492","GRP",0,"Cardiometabolic_II","B13805","20212097_SS211647","PASS",-3.9319,-0.1504,"Plate control","PASS","PFC",0.901000617561454,"24h","24h"
5794,"PFC_05",49,"OID30264","P07492","GRP",0,"Cardiometabolic_II","B13805","20212097_SS211647","PASS",-3.9319,-0.9514,"Plate control","PASS","PFC",0.51713039189182,"Baseline","Baseline"
5795,"PFC_13",50,"OID30264","P07492","GRP",0,"Cardiometabolic_II","B13805","20212097_SS211647","PASS",-3.9319,-0.4641,"Plate control","PASS","PFC",0.724923166853005,"1h","1h"
5796,"PFC_21",51,"OID30264","P07492","GRP",0,"Cardiometabolic_II","B13805","20212097_SS211647","PASS",-3.9319,0.0369,"Plate control","PASS","PFC",1.02590703241588,"24h","24h"
5797,"PFC_06",61,"OID30264","P07492","GRP",0,"Cardiometabolic_II","B13805","20212097_SS211647","PASS",-3.9319,0.4853,"Plate control","PASS","PFC",1.39987693155589,"Baseline","Baseline"
5798,"PFC_14",62,"OID30264","P07492","GRP",0,"Cardiometabolic_II","B13805","20212097_SS211647","PASS",-3.9319,-0.7281,"Plate control","PASS","PFC",0.60369844910518,"1h","1h"
5799,"PFC_22",63,"OID30264","P07492","GRP",0,"Cardiometabolic_II","B13805","20212097_SS211647","PASS",-3.9319,-0.1028,"Plate control","PASS","PFC",0.931223905927429,"24h","24h"
5800,"PFC_07",73,"OID30264","P07492","GRP",0,"Cardiometabolic_II","B13805","20212097_SS211647","PASS",-3.9319,0.7815,"Plate control","PASS","PFC",1.71891713795783,"Baseline","Baseline"
5801,"PFC_15",74,"OID30264","P07492","GRP",0,"Cardiometabolic_II","B13805","20212097_SS211647","PASS",-3.9319,-0.2751,"Plate control","PASS","PFC",0.826393034968728,"1h","1h"
5802,"PFC_23",75,"OID30264","P07492","GRP",0,"Cardiometabolic_II","B13805","20212097_SS211647","PASS",-3.9319,-0.2895,"Plate control","PASS","PFC",0.818185570936827,"24h","24h"
5803,"PFC_08",85,"OID30264","P07492","GRP",0,"Cardiometabolic_II","B13805","20212097_SS211647","PASS",-3.9319,0.6927,"Plate control","PASS","PFC",1.61630560136241,"Baseline","Baseline"
5804,"PFC_16",86,"OID30264","P07492","GRP",0,"Cardiometabolic_II","B13805","20212097_SS211647","PASS",-3.9319,0.3928,"Plate control","PASS","PFC",1.31293910080026,"1h","1h"
5805,"PFC_24",87,"OID30264","P07492","GRP",0,"Cardiometabolic_II","B13805","20212097_SS211647","PASS",-3.9319,0.7823,"Plate control","PASS","PFC",1.71987057233577,"24h","24h"
5806,"PFC_01",1,"OID30265","P21754","ZP3",0,"Cardiometabolic_II","B13805","20212097_SS211647","PASS",-6.3052,1.8053,"Plate control","PASS","PFC",3.49501827672773,"Baseline","Baseline"
5807,"PFC_09",2,"OID30265","P21754","ZP3",0,"Cardiometabolic_II","B13805","20212097_SS211647","PASS",-6.3052,-0.8937,"Plate control","PASS","PFC",0.538231972948283,"Baseline","Baseline"
5808,"PFC_17",3,"OID30265","P21754","ZP3",0,"Cardiometabolic_II","B13805","20212097_SS211647","PASS",-6.3052,0.3999,"Plate control","PASS","PFC",1.31941645262382,"1h","1h"
5809,"PFC_25",4,"OID30265","P21754","ZP3",0,"Cardiometabolic_II","B13805","20212097_SS211647","PASS",-6.3052,1.0536,"Plate control","PASS","PFC",2.07570295423636,"24h","24h"
5810,"PFC_02",13,"OID30265","P21754","ZP3",0,"Cardiometabolic_II","B13805","20212097_SS211647","PASS",-6.3052,-4.2969,"Plate control","PASS","PFC",0.0508749753292609,"Baseline","Baseline"
5811,"PFC_10",14,"OID30265","P21754","ZP3",0,"Cardiometabolic_II","B13805","20212097_SS211647","PASS",-6.3052,1.7318,"Plate control","PASS","PFC",3.32141961791766,"1h","1h"
5812,"PFC_18",15,"OID30265","P21754","ZP3",0,"Cardiometabolic_II","B13805","20212097_SS211647","PASS",-6.3052,-1.3919,"Plate control","PASS","PFC",0.381062619917611,"1h","1h"
5813,"PFC_26",16,"OID30265","P21754","ZP3",0,"Cardiometabolic_II","B13805","20212097_SS211647","PASS",-6.3052,0.4619,"Plate control","PASS","PFC",1.37735457213046,"24h","24h"
5814,"PFC_03",25,"OID30265","P21754","ZP3",0,"Cardiometabolic_II","B13805","20212097_SS211647","PASS",-6.3052,1.6507,"Plate control","PASS","PFC",3.13985949137847,"Baseline","Baseline"
5815,"PFC_11",26,"OID30265","P21754","ZP3",0,"Cardiometabolic_II","B13805","20212097_SS211647","PASS",-6.3052,-4.4352,"Plate control","PASS","PFC",0.0462244511502345,"1h","1h"
5816,"PFC_19",27,"OID30265","P21754","ZP3",0,"Cardiometabolic_II","B13805","20212097_SS211647","PASS",-6.3052,1.9038,"Plate control","PASS","PFC",3.74197520745412,"24h","24h"
5817,"PFC_27",28,"OID30265","P21754","ZP3",0,"Cardiometabolic_II","B13805","20212097_SS211647","PASS",-6.3052,-0.9082,"Plate control","PASS","PFC",0.532849494288626,"24h","24h"
5818,"PFC_04",37,"OID30265","P21754","ZP3",0,"Cardiometabolic_II","B13805","20212097_SS211647","PASS",-6.3052,1.7267,"Plate control","PASS","PFC",3.30969896020006,"Baseline","Baseline"
5819,"PFC_12",38,"OID30265","P21754","ZP3",0,"Cardiometabolic_II","B13805","20212097_SS211647","PASS",-6.3052,1.5415,"Plate control","PASS","PFC",2.9109700578108,"1h","1h"
5820,"PFC_20",39,"OID30265","P21754","ZP3",0,"Cardiometabolic_II","B13805","20212097_SS211647","PASS",-6.3052,-4.8753,"Plate control","PASS","PFC",0.0340712809853981,"24h","24h"
5821,"PFC_05",49,"OID30265","P21754","ZP3",0,"Cardiometabolic_II","B13805","20212097_SS211647","PASS",-6.3052,0.547,"Plate control","PASS","PFC",1.46104437854568,"Baseline","Baseline"
5822,"PFC_13",50,"OID30265","P21754","ZP3",0,"Cardiometabolic_II","B13805","20212097_SS211647","PASS",-6.3052,1.6336,"Plate control","PASS","PFC",3.10286300152954,"1h","1h"
5823,"PFC_21",51,"OID30265","P21754","ZP3",0,"Cardiometabolic_II","B13805","20212097_SS211647","PASS",-6.3052,1.4385,"Plate control","PASS","PFC",2.71038914150854,"24h","24h"
5824,"PFC_06",61,"OID30265","P21754","ZP3",0,"Cardiometabolic_II","B13805","20212097_SS211647","PASS",-6.3052,-0.3962,"Plate control","PASS","PFC",0.759857082340057,"Baseline","Baseline"

Supplemental table 1_Olink proximity extension assay (PEA) data

**,"SampleID","Index","OlinkID","UniProt","Assay","MissingFreq","Panel","Panel_Lot_Nr","PlateID","QC_Warning","LOD","NPX","Normalization","Assay_Warning","condition","Value","group","alt_group"**
5825,"PFC_14",62,"OID30265","P21754","ZP3",0,"Cardiometabolic_II","B13805","20212097_SS211647","PASS",-6.3052,0.3588,"Plate control","PASS","PFC",1.28235881775962,"1h","1h"
5826,"PFC_22",63,"OID30265","P21754","ZP3",0,"Cardiometabolic_II","B13805","20212097_SS211647","PASS",-6.3052,1.6444,"Plate control","PASS","PFC",3.12617816117317,"24h","24h"
5827,"PFC_07",73,"OID30265","P21754","ZP3",0,"Cardiometabolic_II","B13805","20212097_SS211647","PASS",-6.3052,1.0685,"Plate control","PASS","PFC",2.09725167713801,"Baseline","Baseline"
5828,"PFC_15",74,"OID30265","P21754","ZP3",0,"Cardiometabolic_II","B13805","20212097_SS211647","PASS",-6.3052,-0.7323,"Plate control","PASS","PFC",0.601943506967142,"1h","1h"
5829,"PFC_23",75,"OID30265","P21754","ZP3",0,"Cardiometabolic_II","B13805","20212097_SS211647","PASS",-6.3052,0.5946,"Plate control","PASS","PFC",1.51005385168875,"24h","24h"
5830,"PFC_08",85,"OID30265","P21754","ZP3",0,"Cardiometabolic_II","B13805","20212097_SS211647","PASS",-6.3052,0.2925,"Plate control","PASS","PFC",1.22476078859744,"Baseline","Baseline"
5831,"PFC_16",86,"OID30265","P21754","ZP3",0,"Cardiometabolic_II","B13805","20212097_SS211647","PASS",-6.3052,0.9773,"Plate control","PASS","PFC",1.96877739725544,"1h","1h"
5832,"PFC_24",87,"OID30265","P21754","ZP3",0,"Cardiometabolic_II","B13805","20212097_SS211647","PASS",-6.3052,-0.2026,"Plate control","PASS","PFC",0.868983085023402,"24h","24h"
5833,"PFC_01",1,"OID30266","P07098","LIPF",0,"Cardiometabolic_II","B13805","20212097_SS211647","PASS",-4.189,0.7222,"Plate control","PASS","PFC",1.6496957777643,"Baseline","Baseline"
5834,"PFC_09",2,"OID30266","P07098","LIPF",0,"Cardiometabolic_II","B13805","20212097_SS211647","PASS",-4.189,0.2378,"Plate control","PASS","PFC",1.17919311005384,"Baseline","Baseline"
5835,"PFC_17",3,"OID30266","P07098","LIPF",0,"Cardiometabolic_II","B13805","20212097_SS211647","PASS",-4.189,0.7677,"Plate control","PASS","PFC",1.70255334267071,"1h","1h"
5836,"PFC_25",4,"OID30266","P07098","LIPF",0,"Cardiometabolic_II","B13805","20212097_SS211647","PASS",-4.189,0.4949,"Plate control","PASS","PFC",1.40922307185671,"24h","24h"
5837,"PFC_02",13,"OID30266","P07098","LIPF",0,"Cardiometabolic_II","B13805","20212097_SS211647","PASS",-4.189,-0.124,"Plate control","PASS","PFC",0.917639882311365,"Baseline","Baseline"
5838,"PFC_10",14,"OID30266","P07098","LIPF",0,"Cardiometabolic_II","B13805","20212097_SS211647","PASS",-4.189,0.6651,"Plate control","PASS","PFC",1.58567818024743,"1h","1h"
5839,"PFC_18",15,"OID30266","P07098","LIPF",0,"Cardiometabolic_II","B13805","20212097_SS211647","PASS",-4.189,0.0562,"Plate control","PASS","PFC",1.03972356147601,"1h","1h"
5840,"PFC_26",16,"OID30266","P07098","LIPF",0,"Cardiometabolic_II","B13805","20212097_SS211647","PASS",-4.189,1.817,"Plate control","PASS","PFC",3.52347749650792,"24h","24h"
5841,"PFC_03",25,"OID30266","P07098","LIPF",0,"Cardiometabolic_II","B13805","20212097_SS211647","PASS",-4.189,0.6986,"Plate control","PASS","PFC",1.62292912806975,"Baseline","Baseline"
5842,"PFC_11",26,"OID30266","P07098","LIPF",0,"Cardiometabolic_II","B13805","20212097_SS211647","PASS",-4.189,-0.123,"Plate control","PASS","PFC",0.918276162300924,"1h","1h"
5843,"PFC_19",27,"OID30266","P07098","LIPF",0,"Cardiometabolic_II","B13805","20212097_SS211647","PASS",-4.189,0.6746,"Plate control","PASS","PFC",1.59615416345421,"24h","24h"
5844,"PFC_27",28,"OID30266","P07098","LIPF",0,"Cardiometabolic_II","B13805","20212097_SS211647","PASS",-4.189,0.4252,"Plate control","PASS","PFC",1.34275863575072,"24h","24h"
5845,"PFC_04",37,"OID30266","P07098","LIPF",0,"Cardiometabolic_II","B13805","20212097_SS211647","PASS",-4.189,0.2978,"Plate control","PASS","PFC",1.22926844265089,"Baseline","Baseline"
5846,"PFC_12",38,"OID30266","P07098","LIPF",0,"Cardiometabolic_II","B13805","20212097_SS211647","PASS",-4.189,0.9311,"Plate control","PASS","PFC",1.90672925039636,"1h","1h"
5847,"PFC_20",39,"OID30266","P07098","LIPF",0,"Cardiometabolic_II","B13805","20212097_SS211647","PASS",-4.189,-0.2828,"Plate control","PASS","PFC",0.821994131979628,"24h","24h"
5848,"PFC_05",49,"OID30266","P07098","LIPF",0,"Cardiometabolic_II","B13805","20212097_SS211647","PASS",-4.189,0.0084,"Plate control","PASS","PFC",1.00583941964448,"Baseline","Baseline"
5849,"PFC_13",50,"OID30266","P07098","LIPF",0,"Cardiometabolic_II","B13805","20212097_SS211647","PASS",-4.189,0.2249,"Plate control","PASS","PFC",1.16869623790344,"1h","1h"
5850,"PFC_21",51,"OID30266","P07098","LIPF",0,"Cardiometabolic_II","B13805","20212097_SS211647","PASS",-4.189,0.5291,"Plate control","PASS","PFC",1.44302870652311,"24h","24h"
5851,"PFC_06",61,"OID30266","P07098","LIPF",0,"Cardiometabolic_II","B13805","20212097_SS211647","PASS",-4.189,-0.1506,"Plate control","PASS","PFC",0.900875721011274,"Baseline","Baseline"
5852,"PFC_14",62,"OID30266","P07098","LIPF",0,"Cardiometabolic_II","B13805","20212097_SS211647","PASS",-4.189,0.1506,"Plate control","PASS","PFC",1.11003102500915,"1h","1h"
5853,"PFC_22",63,"OID30266","P07098","LIPF",0,"Cardiometabolic_II","B13805","20212097_SS211647","PASS",-4.189,0.2675,"Plate control","PASS","PFC",1.20372013132409,"24h","24h"
5854,"PFC_07",73,"OID30266","P07098","LIPF",0,"Cardiometabolic_II","B13805","20212097_SS211647","PASS",-4.189,0.9055,"Plate control","PASS","PFC",1.87319358180923,"Baseline","Baseline"
5855,"PFC_15",74,"OID30266","P07098","LIPF",0,"Cardiometabolic_II","B13805","20212097_SS211647","PASS",-4.189,-0.1435,"Plate control","PASS","PFC",0.905320168579888,"1h","1h"
5856,"PFC_23",75,"OID30266","P07098","LIPF",0,"Cardiometabolic_II","B13805","20212097_SS211647","PASS",-4.189,-0.0958,"Plate control","PASS","PFC",0.935753211780196,"24h","24h"
5857,"PFC_08",85,"OID30266","P07098","LIPF",0,"Cardiometabolic_II","B13805","20212097_SS211647","PASS",-4.189,0.9707,"Plate control","PASS","PFC",1.95979126117321,"Baseline","Baseline"
5858,"PFC_16",86,"OID30266","P07098","LIPF",0,"Cardiometabolic_II","B13805","20212097_SS211647","PASS",-4.189,0.6697,"Plate control","PASS","PFC",1.59074214758462,"1h","1h"
5859,"PFC_24",87,"OID30266","P07098","LIPF",0,"Cardiometabolic_II","B13805","20212097_SS211647","PASS",-4.189,0.1399,"Plate control","PASS","PFC",1.10182874028119,"24h","24h"
5860,"PFC_01",1,"OID30267","Q9H3K6","BOLA2_BOLA2B",0,"Cardiometabolic_II","B13805","20212097_SS211647","PASS",-2.1861,2.9414,"Plate control","PASS","PFC",7.68156357474177,"Baseline","Baseline"
5861,"PFC_09",2,"OID30267","Q9H3K6","BOLA2_BOLA2B",0,"Cardiometabolic_II","B13805","20212097_SS211647","PASS",-2.1861,3.7014,"Plate control","PASS","PFC",13.0086558970547,"Baseline","Baseline"
5862,"PFC_17",3,"OID30267","Q9H3K6","BOLA2_BOLA2B",0,"Cardiometabolic_II","B13805","20212097_SS211647","PASS",-2.1861,4.2853,"Plate control","PASS","PFC",19.4986184209796,"1h","1h"
5863,"PFC_25",4,"OID30267","Q9H3K6","BOLA2_BOLA2B",0,"Cardiometabolic_II","B13805","20212097_SS211647","PASS",-2.1861,4.3901,"Plate control","PASS","PFC",20.9677477848909,"24h","24h"
5864,"PFC_02",13,"OID30267","Q9H3K6","BOLA2_BOLA2B",0,"Cardiometabolic_II","B13805","20212097_SS211647","PASS",-2.1861,3.0908,"Plate control","PASS","PFC",8.51968446622399,"Baseline","Baseline"
5865,"PFC_10",14,"OID30267","Q9H3K6","BOLA2_BOLA2B",0,"Cardiometabolic_II","B13805","20212097_SS211647","PASS",-2.1861,3.0652,"Plate control","PASS","PFC",8.36983974408172,"1h","1h"
5866,"PFC_18",15,"OID30267","Q9H3K6","BOLA2_BOLA2B",0,"Cardiometabolic_II","B13805","20212097_SS211647","PASS",-2.1861,3.6336,"Plate control","PASS","PFC",12.4114520061182,"1h","1h"
5867,"PFC_26",16,"OID30267","Q9H3K6","BOLA2_BOLA2B",0,"Cardiometabolic_II","B13805","20212097_SS211647","PASS",-2.1861,4.3874,"Plate control","PASS","PFC",20.9285433965874,"24h","24h"
5868,"PFC_03",25,"OID30267","Q9H3K6","BOLA2_BOLA2B",0,"Cardiometabolic_II","B13805","20212097_SS211647","PASS",-2.1861,2.2048,"Plate control","PASS","PFC",4.61010624646526,"Baseline","Baseline"
5869,"PFC_11",26,"OID30267","Q9H3K6","BOLA2_BOLA2B",0,"Cardiometabolic_II","B13805","20212097_SS211647","PASS",-2.1861,3.9985,"Plate control","PASS","PFC",15.9833731128244,"1h","1h"
5870,"PFC_19",27,"OID30267","Q9H3K6","BOLA2_BOLA2B",0,"Cardiometabolic_II","B13805","20212097_SS211647","PASS",-2.1861,3.5295,"Plate control","PASS","PFC",11.5474308360367,"24h","24h"
5871,"PFC_27",28,"OID30267","Q9H3K6","BOLA2_BOLA2B",0,"Cardiometabolic_II","B13805","20212097_SS211647","PASS",-2.1861,4.5368,"Plate control","PASS","PFC",23.2120172334308,"24h","24h"
5872,"PFC_04",37,"OID30267","Q9H3K6","BOLA2_BOLA2B",0,"Cardiometabolic_II","B13805","20212097_SS211647","PASS",-2.1861,3.9179,"Plate control","PASS","PFC",15.1149049342705,"Baseline","Baseline"
5873,"PFC_12",38,"OID30267","Q9H3K6","BOLA2_BOLA2B",0,"Cardiometabolic_II","B13805","20212097_SS211647","PASS",-2.1861,3.0206,"Plate control","PASS","PFC",8.1150500910699,"1h","1h"
5874,"PFC_20",39,"OID30267","Q9H3K6","BOLA2_BOLA2B",0,"Cardiometabolic_II","B13805","20212097_SS211647","PASS",-2.1861,4.0568,"Plate control","PASS","PFC",16.6424969644559,"24h","24h"
5875,"PFC_05",49,"OID30267","Q9H3K6","BOLA2_BOLA2B",0,"Cardiometabolic_II","B13805","20212097_SS211647","PASS",-2.1861,3.1479,"Plate control","PASS","PFC",8.8636443806149,"Baseline","Baseline"
5876,"PFC_13",50,"OID30267","Q9H3K6","BOLA2_BOLA2B",0,"Cardiometabolic_II","B13805","20212097_SS211647","PASS",-2.1861,4.0604,"Plate control","PASS","PFC",16.6840773407116,"1h","1h"
5877,"PFC_21",51,"OID30267","Q9H3K6","BOLA2_BOLA2B",0,"Cardiometabolic_II","B13805","20212097_SS211647","PASS",-2.1861,3.4352,"Plate control","PASS","PFC",10.8167860852462,"24h","24h"
5878,"PFC_06",61,"OID30267","Q9H3K6","BOLA2_BOLA2B",0,"Cardiometabolic_II","B13805","20212097_SS211647","PASS",-2.1861,3.7488,"Plate control","PASS","PFC",13.4431562893745,"Baseline","Baseline"
5879,"PFC_14",62,"OID30267","Q9H3K6","BOLA2_BOLA2B",0,"Cardiometabolic_II","B13805","20212097_SS211647","PASS",-2.1861,3.8638,"Plate control","PASS","PFC",14.5586027944974,"1h","1h"
5880,"PFC_22",63,"OID30267","Q9H3K6","BOLA2_BOLA2B",0,"Cardiometabolic_II","B13805","20212097_SS211647","PASS",-2.1861,3.547,"Plate control","PASS","PFC",11.6883550283654,"24h","24h"
5881,"PFC_07",73,"OID30267","Q9H3K6","BOLA2_BOLA2B",0,"Cardiometabolic_II","B13805","20212097_SS211647","PASS",-2.1861,4.7488,"Plate control","PASS","PFC",26.886312578749,"Baseline","Baseline"
5882,"PFC_15",74,"OID30267","Q9H3K6","BOLA2_BOLA2B",0,"Cardiometabolic_II","B13805","20212097_SS211647","PASS",-2.1861,2.4546,"Plate control","PASS","PFC",5.481611183872,"1h","1h"
5883,"PFC_23",75,"OID30267","Q9H3K6","BOLA2_BOLA2B",0,"Cardiometabolic_II","B13805","20212097_SS211647","PASS",-2.1861,4.0861,"Plate control","PASS","PFC",16.9839485400022,"24h","24h"
5884,"PFC_08",85,"OID30267","Q9H3K6","BOLA2_BOLA2B",0,"Cardiometabolic_II","B13805","20212097_SS211647","PASS",-2.1861,3.599,"Plate control","PASS","PFC",12.1173305270158,"Baseline","Baseline"
5885,"PFC_16",86,"OID30267","Q9H3K6","BOLA2_BOLA2B",0,"Cardiometabolic_II","B13805","20212097_SS211647","PASS",-2.1861,4.3248,"Plate control","PASS","PFC",20.0398527119318,"1h","1h"
5886,"PFC_24",87,"OID30267","Q9H3K6","BOLA2_BOLA2B",0,"Cardiometabolic_II","B13805","20212097_SS211647","PASS",-2.1861,4.0344,"Plate control","PASS","PFC",16.3860929667496,"24h","24h"
5887,"PFC_01",1,"OID30268","Q16621","NFE2",0,"Cardiometabolic_II","B13805","20212097_SS211647","PASS",-0.8986,1.4682,"Plate control","PASS","PFC",2.76676478647935,"Baseline","Baseline"
5888,"PFC_09",2,"OID30268","Q16621","NFE2",0,"Cardiometabolic_II","B13805","20212097_SS211647","PASS",-0.8986,3.2876,"Plate control","PASS","PFC",9.76486433072195,"Baseline","Baseline"
5889,"PFC_17",3,"OID30268","Q16621","NFE2",0,"Cardiometabolic_II","B13805","20212097_SS211647","PASS",-0.8986,3.6365,"Plate control","PASS","PFC",12.4364256904431,"1h","1h"
5890,"PFC_25",4,"OID30268","Q16621","NFE2",0,"Cardiometabolic_II","B13805","20212097_SS211647","PASS",-0.8986,3.219,"Plate control","PASS","PFC",9.31141227521709,"24h","24h"
5891,"PFC_02",13,"OID30268","Q16621","NFE2",0,"Cardiometabolic_II","B13805","20212097_SS211647","PASS",-0.8986,2.2388,"Plate control","PASS","PFC",4.72004299108925,"Baseline","Baseline"
5892,"PFC_10",14,"OID30268","Q16621","NFE2",0,"Cardiometabolic_II","B13805","20212097_SS211647","PASS",-0.8986,2.024,"Plate control","PASS","PFC",4.06709869316783,"1h","1h"
5893,"PFC_18",15,"OID30268","Q16621","NFE2",0,"Cardiometabolic_II","B13805","20212097_SS211647","PASS",-0.8986,3.162,"Plate control","PASS","PFC",8.95069680828569,"1h","1h"
5894,"PFC_26",16,"OID30268","Q16621","NFE2",0,"Cardiometabolic_II","B13805","20212097_SS211647","PASS",-0.8986,3.1719,"Plate control","PASS","PFC",9.0123291192758,"24h","24h"
5895,"PFC_03",25,"OID30268","Q16621","NFE2",0,"Cardiometabolic_II","B13805","20212097_SS211647","PASS",-0.8986,0.7966,"Plate control","PASS","PFC",1.73700270412043,"Baseline","Baseline"
5896,"PFC_11",26,"OID30268","Q16621","NFE2",0,"Cardiometabolic_II","B13805","20212097_SS211647","PASS",-0.8986,3.4531,"Plate control","PASS","PFC",10.9518296014071,"1h","1h"
5897,"PFC_19",27,"OID30268","Q16621","NFE2",0,"Cardiometabolic_II","B13805","20212097_SS211647","PASS",-0.8986,2.6732,"Plate control","PASS","PFC",6.37842398835725,"24h","24h"
5898,"PFC_27",28,"OID30268","Q16621","NFE2",0,"Cardiometabolic_II","B13805","20212097_SS211647","PASS",-0.8986,4.3373,"Plate control","PASS","PFC",20.214239185393,"24h","24h"
5899,"PFC_04",37,"OID30268","Q16621","NFE2",0,"Cardiometabolic_II","B13805","20212097_SS211647","PASS",-0.8986,3.0828,"Plate control","PASS","PFC",8.47257204816834,"Baseline","Baseline"
5900,"PFC_12",38,"OID30268","Q16621","NFE2",0,"Cardiometabolic_II","B13805","20212097_SS211647","PASS",-0.8986,1.4402,"Plate control","PASS","PFC",2.71358481155382,"1h","1h"
5901,"PFC_20",39,"OID30268","Q16621","NFE2",0,"Cardiometabolic_II","B13805","20212097_SS211647","PASS",-0.8986,3.78,"Plate control","PASS","PFC",13.7370469830041,"24h","24h"
5902,"PFC_05",49,"OID30268","Q16621","NFE2",0,"Cardiometabolic_II","B13805","20212097_SS211647","PASS",-0.8986,2.293,"Plate control","PASS","PFC",4.9007413276187,"Baseline","Baseline"
5903,"PFC_13",50,"OID30268","Q16621","NFE2",0,"Cardiometabolic_II","B13805","20212097_SS211647","PASS",-0.8986,3.4754,"Plate control","PASS","PFC",11.122429123429,"1h","1h"
5904,"PFC_21",51,"OID30268","Q16621","NFE2",0,"Cardiometabolic_II","B13805","20212097_SS211647","PASS",-0.8986,1.957,"Plate control","PASS","PFC",3.88253787907819,"24h","24h"
5905,"PFC_06",61,"OID30268","Q16621","NFE2",0,"Cardiometabolic_II","B13805","20212097_SS211647","PASS",-0.8986,3.0507,"Plate control","PASS","PFC",8.28613887517849,"Baseline","Baseline"
5906,"PFC_14",62,"OID30268","Q16621","NFE2",0,"Cardiometabolic_II","B13805","20212097_SS211647","PASS",-0.8986,3.426,"Plate control","PASS","PFC",10.7480274057704,"1h","1h"
5907,"PFC_22",63,"OID30268","Q16621","NFE2",0,"Cardiometabolic_II","B13805","20212097_SS211647","PASS",-0.8986,2.957,"Plate control","PASS","PFC",7.76507575815637,"24h","24h"
5908,"PFC_07",73,"OID30268","Q16621","NFE2",0,"Cardiometabolic_II","B13805","20212097_SS211647","PASS",-0.8986,3.5193,"Plate control","PASS","PFC",11.4660772588382,"Baseline","Baseline"
5909,"PFC_15",74,"OID30268","Q16621","NFE2",0,"Cardiometabolic_II","B13805","20212097_SS211647","PASS",-0.8986,2.2156,"Plate control","PASS","PFC",4.64474695185814,"1h","1h"
5910,"PFC_23",75,"OID30268","Q16621","NFE2",0,"Cardiometabolic_II","B13805","20212097_SS211647","PASS",-0.8986,3.2353,"Plate control","PASS","PFC",9.41721194833371,"24h","24h"
5911,"PFC_08",85,"OID30268","Q16621","NFE2",0,"Cardiometabolic_II","B13805","20212097_SS211647","PASS",-0.8986,2.5785,"Plate control","PASS","PFC",5.97318332208935,"Baseline","Baseline"
5912,"PFC_16",86,"OID30268","Q16621","NFE2",0,"Cardiometabolic_II","B13805","20212097_SS211647","PASS",-0.8986,3.7175,"Plate control","PASS","PFC",13.1546412580351,"1h","1h"
5913,"PFC_24",87,"OID30268","Q16621","NFE2",0,"Cardiometabolic_II","B13805","20212097_SS211647","PASS",-0.8986,4.0483,"Plate control","PASS","PFC",16.5447318025494,"24h","24h"
5914,"PFC_01",1,"OID30269","O94979","SEC31A",0,"Cardiometabolic_II","B13805","20212097_SS211647","PASS",-0.5452,2.7309,"Plate control","PASS","PFC",6.63869650939478,"Baseline","Baseline"
5915,"PFC_09",2,"OID30269","O94979","SEC31A",0,"Cardiometabolic_II","B13805","20212097_SS211647","PASS",-0.5452,2.9246,"Plate control","PASS","PFC",7.59263155128384,"Baseline","Baseline"
5916,"PFC_17",3,"OID30269","O94979","SEC31A",0,"Cardiometabolic_II","B13805","20212097_SS211647","PASS",-0.5452,4.021,"Plate control","PASS","PFC",16.2346007452941,"1h","1h"
5917,"PFC_25",4,"OID30269","O94979","SEC31A",0,"Cardiometabolic_II","B13805","20212097_SS211647","PASS",-0.5452,3.4771,"Plate control","PASS","PFC",11.1355429649199,"24h","24h"
5918,"PFC_02",13,"OID30269","O94979","SEC31A",0,"Cardiometabolic_II","B13805","20212097_SS211647","PASS",-0.5452,2.7094,"Plate control","PASS","PFC",6.54049578702312,"Baseline","Baseline"
5919,"PFC_10",14,"OID30269","O94979","SEC31A",0,"Cardiometabolic_II","B13805","20212097_SS211647","PASS",-0.5452,2.9391,"Plate control","PASS","PFC",7.66932708676518,"1h","1h"
5920,"PFC_18",15,"OID30269","O94979","SEC31A",0,"Cardiometabolic_II","B13805","20212097_SS211647","PASS",-0.5452,3.4913,"Plate control","PASS","PFC",11.2456878335791,"1h","1h"
5921,"PFC_26",16,"OID30269","O94979","SEC31A",0,"Cardiometabolic_II","B13805","20212097_SS211647","PASS",-0.5452,3.622,"Plate control","PASS","PFC",12.3120577612869,"24h","24h"
5922,"PFC_03",25,"OID30269","O94979","SEC31A",0,"Cardiometabolic_II","B13805","20212097_SS211647","PASS",-0.5452,1.6757,"Plate control","PASS","PFC",3.19474326853346,"Baseline","Baseline"
5923,"PFC_11",26,"OID30269","O94979","SEC31A",0,"Cardiometabolic_II","B13805","20212097_SS211647","PASS",-0.5452,3.4543,"Plate control","PASS","PFC",10.9609428667545,"1h","1h"
5924,"PFC_19",27,"OID30269","O94979","SEC31A",0,"Cardiometabolic_II","B13805","20212097_SS211647","PASS",-0.5452,3.6276,"Plate control","PASS","PFC",12.3599414158365,"24h","24h"
5925,"PFC_27",28,"OID30269","O94979","SEC31A",0,"Cardiometabolic_II","B13805","20212097_SS211647","PASS",-0.5452,4.215,"Plate control","PASS","PFC",18.5712626301395,"24h","24h"
5926,"PFC_04",37,"OID30269","O94979","SEC31A",0,"Cardiometabolic_II","B13805","20212097_SS211647","PASS",-0.5452,3.4784,"Plate control","PASS","PFC",11.1455816283902,"Baseline","Baseline"
5927,"PFC_12",38,"OID30269","O94979","SEC31A",0,"Cardiometabolic_II","B13805","20212097_SS211647","PASS",-0.5452,2.4001,"Plate control","PASS","PFC",5.27839750104633,"1h","1h"
5928,"PFC_20",39,"OID30269","O94979","SEC31A",0,"Cardiometabolic_II","B13805","20212097_SS211647","PASS",-0.5452,3.835,"Plate control","PASS","PFC",14.2708563107215,"24h","24h"
5929,"PFC_05",49,"OID30269","O94979","SEC31A",0,"Cardiometabolic_II","B13805","20212097_SS211647","PASS",-0.5452,2.2474,"Plate control","PASS","PFC",4.74826350632238,"Baseline","Baseline"
5930,"PFC_13",50,"OID30269","O94979","SEC31A",0,"Cardiometabolic_II","B13805","20212097_SS211647","PASS",-0.5452,3.5858,"Plate control","PASS","PFC",12.0069681455105,"1h","1h"
5931,"PFC_21",51,"OID30269","O94979","SEC31A",0,"Cardiometabolic_II","B13805","20212097_SS211647","PASS",-0.5452,2.754,"Plate control","PASS","PFC",6.7458488820009,"24h","24h"
5932,"PFC_06",61,"OID30269","O94979","SEC31A",0,"Cardiometabolic_II","B13805","20212097_SS211647","PASS",-0.5452,3.2996,"Plate control","PASS","PFC",9.84642491959614,"Baseline","Baseline"
5933,"PFC_14",62,"OID30269","O94979","SEC31A",0,"Cardiometabolic_II","B13805","20212097_SS211647","PASS",-0.5452,3.4171,"Plate control","PASS","PFC",10.6819268154525,"1h","1h"
5934,"PFC_22",63,"OID30269","O94979","SEC31A",0,"Cardiometabolic_II","B13805","20212097_SS211647","PASS",-0.5452,3.135,"Plate control","PASS","PFC",8.7847425102964,"24h","24h"
5935,"PFC_07",73,"OID30269","O94979","SEC31A",0,"Cardiometabolic_II","B13805","20212097_SS211647","PASS",-0.5452,3.6438,"Plate control","PASS","PFC",12.4995131621816,"Baseline","Baseline"
5936,"PFC_15",74,"OID30269","O94979","SEC31A",0,"Cardiometabolic_II","B13805","20212097_SS211647","PASS",-0.5452,2.7082,"Plate control","PASS","PFC",6.53505781746752,"1h","1h"

Supplemental table 1_Olink proximity extension assay (PEA) data

,"SampleID","Index","OlinkID","UniProt","Assay","MissingFreq","Panel","Panel_Lot_Nr","PlateID","QC_Warning","LOD","NPX","Normalization","Assay_Warning","condition","Value","group","alt_group"
5937,"PFC_23",75,"OID30269","O94979","SEC31A",0,"Cardiometabolic_II","B13805","20212097_SS211647","PASS",-0.5452,3.2874,"Plate control","PASS","PFC",9.76351072691291,"24h","24h"
5938,"PFC_08",85,"OID30269","O94979","SEC31A",0,"Cardiometabolic_II","B13805","20212097_SS211647","PASS",-0.5452,2.7207,"Plate control","PASS","PFC",6.59192578466444,"Baseline","Baseline"
5939,"PFC_16",86,"OID30269","O94979","SEC31A",0,"Cardiometabolic_II","B13805","20212097_SS211647","PASS",-0.5452,3.3318,"Plate control","PASS","PFC",10.0686614784209,"1h","1h"
5940,"PFC_24",87,"OID30269","O94979","SEC31A",0,"Cardiometabolic_II","B13805","20212097_SS211647","PASS",-0.5452,3.5772,"Plate control","PASS","PFC",11.935606725277,"24h","24h"
5941,"PFC_01",1,"OID30270","P20042","EIF2S2",0.1136,"Cardiometabolic_II","B13805","20212097_SS211647","PASS",0.63,1.5802,"Plate control","PASS","PFC",2.9901129862162 2,"Baseline","Baseline"
5942,"PFC_09",2,"OID30270","P20042","EIF2S2",0.1136,"Cardiometabolic_II","B13805","20212097_SS211647","PASS",0.63,2.879,"Plate control","PASS","PFC",7.35640036921326,"Baseline","Baseline"
5943,"PFC_17",3,"OID30270","P20042","EIF2S2",0.1136,"Cardiometabolic_II","B13805","20212097_SS211647","PASS",0.63,4.2543,"Plate control","PASS","PFC",19.0841099738194,"1h","1h"
5944,"PFC_25",4,"OID30270","P20042","EIF2S2",0.1136,"Cardiometabolic_II","B13805","20212097_SS211647","PASS",0.63,3.3259,"Plate control","PASS","PFC",10.0275690813124,"24h","24h"
5945,"PFC_02",13,"OID30270","P20042","EIF2S2",0.1136,"Cardiometabolic_II","B13805","20212097_SS211647","PASS",0.63,2.1869,"Plate control","PASS","PFC",4.55326049739351,"Baseline","Baseline"
5946,"PFC_10",14,"OID30270","P20042","EIF2S2",0.1136,"Cardiometabolic_II","B13805","20212097_SS211647","PASS",0.63,2.2045,"Plate control","PASS","PFC",4.60914770148658,"1h","1h"
5947,"PFC_18",15,"OID30270","P20042","EIF2S2",0.1136,"Cardiometabolic_II","B13805","20212097_SS211647","PASS",0.63,3.1236,"Plate control","PASS","PFC",8.71560008523037,"1h","1h"
5948,"PFC_26",16,"OID30270","P20042","EIF2S2",0.1136,"Cardiometabolic_II","B13805","20212097_SS211647","PASS",0.63,3.7984,"Plate control","PASS","PFC",13.913370031996,"24h","24h"
5949,"PFC_03",25,"OID30270","P20042","EIF2S2",0.1136,"Cardiometabolic_II","B13805","20212097_SS211647","PASS",0.63,0.5728,"Plate control","PASS","PFC",1.48740754967138,"Baseline","Baseline"
5950,"PFC_11",26,"OID30270","P20042","EIF2S2",0.1136,"Cardiometabolic_II","B13805","20212097_SS211647","PASS",0.63,3.5201,"Plate control","PASS","PFC",11.472437165315,"1h","1h"
5951,"PFC_19",27,"OID30270","P20042","EIF2S2",0.1136,"Cardiometabolic_II","B13805","20212097_SS211647","PASS",0.63,3.2543,"Plate control","PASS","PFC",9.54205498690972,"24h","24h"
5952,"PFC_27",28,"OID30270","P20042","EIF2S2",0.1136,"Cardiometabolic_II","B13805","20212097_SS211647","PASS",0.63,4.8365,"Plate control","PASS","PFC",28.5714034653097,"24h","24h"
5953,"PFC_04",37,"OID30270","P20042","EIF2S2",0.1136,"Cardiometabolic_II","B13805","20212097_SS211647","PASS",0.63,3.5817,"Plate control","PASS","PFC",11.9728939421613,"Baseline","Baseline"
5954,"PFC_12",38,"OID30270","P20042","EIF2S2",0.1136,"Cardiometabolic_II","B13805","20212097_SS211647","PASS",0.63,1.452,"Plate control","PASS","PFC",2.73587060780498,"1h","1h"
5955,"PFC_20",39,"OID30270","P20042","EIF2S2",0.1136,"Cardiometabolic_II","B13805","20212097_SS211647","PASS",0.63,4.234,"Plate control","PASS","PFC",18.8174600046554,"24h","24h"
5956,"PFC_05",49,"OID30270","P20042","EIF2S2",0.1136,"Cardiometabolic_II","B13805","20212097_SS211647","PASS",0.63,2.3387,"Plate control","PASS","PFC",5.05846618157066,"Baseline","Baseline"
5957,"PFC_13",50,"OID30270","P20042","EIF2S2",0.1136,"Cardiometabolic_II","B13805","20212097_SS211647","PASS",0.63,4.2269,"Plate control","PASS","PFC",18.7250802949486,"1h","1h"
5958,"PFC_21",51,"OID30270","P20042","EIF2S2",0.1136,"Cardiometabolic_II","B13805","20212097_SS211647","PASS",0.63,1.9092,"Plate control","PASS","PFC",3.75600764639382,"24h","24h"
5959,"PFC_06",61,"OID30270","P20042","EIF2S2",0.1136,"Cardiometabolic_II","B13805","20212097_SS211647","PASS",0.63,3.8858,"Plate control","PASS","PFC",14.7823117535557,"Baseline","Baseline"
5960,"PFC_14",62,"OID30270","P20042","EIF2S2",0.1136,"Cardiometabolic_II","B13805","20212097_SS211647","PASS",0.63,3.7079,"Plate control","PASS","PFC",13.0673980633558,"1h","1h"
5961,"PFC_22",63,"OID30270","P20042","EIF2S2",0.1136,"Cardiometabolic_II","B13805","20212097_SS211647","PASS",0.63,3.4531,"Plate control","PASS","PFC",10.9518296014071,"24h","24h"
5962,"PFC_07",73,"OID30270","P20042","EIF2S2",0.1136,"Cardiometabolic_II","B13805","20212097_SS211647","PASS",0.63,4.1986,"Plate control","PASS","PFC",18.3613470694926,"Baseline","Baseline"
5963,"PFC_15",74,"OID30270","P20042","EIF2S2",0.1136,"Cardiometabolic_II","B13805","20212097_SS211647","PASS",0.63,2.3097,"Plate control","PASS","PFC",4.95779974708403,"1h","1h"
5964,"PFC_23",75,"OID30270","P20042","EIF2S2",0.1136,"Cardiometabolic_II","B13805","20212097_SS211647","PASS",0.63,4.1104,"Plate control","PASS","PFC",17.2724400574954,"24h","24h"
5965,"PFC_08",85,"OID30270","P20042","EIF2S2",0.1136,"Cardiometabolic_II","B13805","20212097_SS211647","PASS",0.63,3.1101,"Plate control","PASS","PFC",8.63442436398409,"Baseline","Baseline"
5966,"PFC_16",86,"OID30270","P20042","EIF2S2",0.1136,"Cardiometabolic_II","B13805","20212097_SS211647","PASS",0.63,3.9615,"Plate control","PASS","PFC",15.5786682060096,"1h","1h"
5967,"PFC_24",87,"OID30270","P20042","EIF2S2",0.1136,"Cardiometabolic_II","B13805","20212097_SS211647","PASS",0.63,4.669,"Plate control","PASS","PFC",25.4395280303672,"24h","24h"
5968,"PFC_01",1,"OID30271","Q9UJ70","NAGK",0,"Cardiometabolic_II","B13805","20212097_SS211647","PASS",-3.1807,0.3935,"Plate control","PASS","PFC",1.3135762973978,"Baseline","Baseline"
5969,"PFC_09",2,"OID30271","Q9UJ70","NAGK",0,"Cardiometabolic_II","B13805","20212097_SS211647","PASS",-3.1807,1.0287,"Plate control","PASS","PFC",2.0401850298339,"Baseline","Baseline"
5970,"PFC_17",3,"OID30271","Q9UJ70","NAGK",0,"Cardiometabolic_II","B13805","20212097_SS211647","PASS",-3.1807,1.5844,"Plate control","PASS","PFC",2.99883054063166,"1h","1h"
5971,"PFC_25",4,"OID30271","Q9UJ70","NAGK",0,"Cardiometabolic_II","B13805","20212097_SS211647","PASS",-3.1807,1.541,"Plate control","PASS","PFC",2.90996136726951,"24h","24h"
5972,"PFC_02",13,"OID30271","Q9UJ70","NAGK",0,"Cardiometabolic_II","B13805","20212097_SS211647","PASS",-3.1807,0.4435,"Plate control","PASS","PFC",1.35989946548537,"Baseline","Baseline"
5973,"PFC_10",14,"OID30271","Q9UJ70","NAGK",0,"Cardiometabolic_II","B13805","20212097_SS211647","PASS",-3.1807,1.2223,"Plate control","PASS","PFC",2.33318386108061,"1h","1h"
5974,"PFC_18",15,"OID30271","Q9UJ70","NAGK",0,"Cardiometabolic_II","B13805","20212097_SS211647","PASS",-3.1807,1.3653,"Plate control","PASS","PFC",2.57629892962474,"1h","1h"
5975,"PFC_26",16,"OID30271","Q9UJ70","NAGK",0,"Cardiometabolic_II","B13805","20212097_SS211647","PASS",-3.1807,1.2747,"Plate control","PASS","PFC",2.41948500836117,"24h","24h"
5976,"PFC_03",25,"OID30271","Q9UJ70","NAGK",0,"Cardiometabolic_II","B13805","20212097_SS211647","PASS",-3.1807,0.351,"Plate control","PASS","PFC",1.27544439167854,"Baseline","Baseline"
5977,"PFC_11",26,"OID30271","Q9UJ70","NAGK",0,"Cardiometabolic_II","B13805","20212097_SS211647","PASS",-3.1807,1.6126,"Plate control","PASS","PFC",3.05802457377114,"1h","1h"
5978,"PFC_19",27,"OID30271","Q9UJ70","NAGK",0,"Cardiometabolic_II","B13805","20212097_SS211647","PASS",-3.1807,1.3499,"Plate control","PASS","PFC",2.54894456914099,"24h","24h"
5979,"PFC_27",28,"OID30271","Q9UJ70","NAGK",0,"Cardiometabolic_II","B13805","20212097_SS211647","PASS",-3.1807,2.3104,"Plate control","PASS","PFC",4.96020587020747,"24h","24h"
5980,"PFC_04",37,"OID30271","Q9UJ70","NAGK",0,"Cardiometabolic_II","B13805","20212097_SS211647","PASS",-3.1807,1.8069,"Plate control","PASS","PFC",3.4988965261916,"Baseline","Baseline"
5981,"PFC_12",38,"OID30271","Q9UJ70","NAGK",0,"Cardiometabolic_II","B13805","20212097_SS211647","PASS",-3.1807,0.4251,"Plate control","PASS","PFC",1.34266556604005,"1h","1h"
5982,"PFC_20",39,"OID30271","Q9UJ70","NAGK",0,"Cardiometabolic_II","B13805","20212097_SS211647","PASS",-3.1807,1.7733,"Plate control","PASS","PFC",3.41834971549825,"24h","24h"
5983,"PFC_05",49,"OID30271","Q9UJ70","NAGK",0,"Cardiometabolic_II","B13805","20212097_SS211647","PASS",-3.1807,1.0306,"Plate control","PASS","PFC",2.04287368204778,"Baseline","Baseline"
5984,"PFC_13",50,"OID30271","Q9UJ70","NAGK",0,"Cardiometabolic_II","B13805","20212097_SS211647","PASS",-3.1807,1.9843,"Plate control","PASS","PFC",3.9567063539402,"1h","1h"
5985,"PFC_21",51,"OID30271","Q9UJ70","NAGK",0,"Cardiometabolic_II","B13805","20212097_SS211647","PASS",-3.1807,0.8973,"Plate control","PASS","PFC",1.86257691137403,"24h","24h"
5986,"PFC_06",61,"OID30271","Q9UJ70","NAGK",0,"Cardiometabolic_II","B13805","20212097_SS211647","PASS",-3.1807,1.3107,"Plate control","PASS","PFC",2.48061871163532,"Baseline","Baseline"
5987,"PFC_14",62,"OID30271","Q9UJ70","NAGK",0,"Cardiometabolic_II","B13805","20212097_SS211647","PASS",-3.1807,1.8024,"Plate control","PASS","PFC",3.48799990301446,"1h","1h"
5988,"PFC_22",63,"OID30271","Q9UJ70","NAGK",0,"Cardiometabolic_II","B13805","20212097_SS211647","PASS",-3.1807,1.3475,"Plate control","PASS","PFC",2.5447077891 9295,"24h","24h"
5989,"PFC_07",73,"OID30271","Q9UJ70","NAGK",0,"Cardiometabolic_II","B13805","20212097_SS211647","PASS",-3.1807,1.7343,"Plate control","PASS","PFC",3.32718018923991,"Baseline","Baseline"
5990,"PFC_15",74,"OID30271","Q9UJ70","NAGK",0,"Cardiometabolic_II","B13805","20212097_SS211647","PASS",-3.1807,0.2339,"Plate control","PASS","PFC",1.17600973268276,"1h","1h"
5991,"PFC_23",75,"OID30271","Q9UJ70","NAGK",0,"Cardiometabolic_II","B13805","20212097_SS211647","PASS",-3.1807,1.8071,"Plate control","PASS","PFC",3.4993816098667,"24h","24h"
5992,"PFC_08",85,"OID30271","Q9UJ70","NAGK",0,"Cardiometabolic_II","B13805","20212097_SS211647","PASS",-3.1807,0.6678,"Plate control","PASS","PFC",1.58864855147321,"Baseline","Baseline"
5993,"PFC_16",86,"OID30271","Q9UJ70","NAGK",0,"Cardiometabolic_II","B13805","20212097_SS211647","PASS",-3.1807,1.7182,"Plate control","PASS","PFC",3.29025636962023,"1h","1h"
5994,"PFC_24",87,"OID30271","Q9UJ70","NAGK",0,"Cardiometabolic_II","B13805","20212097_SS211647","PASS",-3.1807,1.6512,"Plate control","PASS","PFC",3.14094787234653,"24h","24h"
5995,"PFC_01",1,"OID30272","O95825","CRYZL1",0,"Cardiometabolic_II","B13805","20212097_SS211647","PASS",-2.7105,3.6078,"Plate control","PASS","PFC",12.1914684291705,"Baseline","Baseline"
5996,"PFC_09",2,"OID30272","O95825","CRYZL1",0,"Cardiometabolic_II","B13805","20212097_SS211647","PASS",-2.7105,4.7616,"Plate control","PASS","PFC",27.1259169244116,"Baseline","Baseline"
5997,"PFC_17",3,"OID30272","O95825","CRYZL1",0,"Cardiometabolic_II","B13805","20212097_SS211647","PASS",-2.7105,6.1599,"Plate control","PASS","PFC",71.5014205640457,"1h","1h"
5998,"PFC_25",4,"OID30272","O95825","CRYZL1",0,"Cardiometabolic_II","B13805","20212097_SS211647","PASS",-2.7105,5.9465,"Plate control","PASS","PFC",61.6701307526453,"24h","24h"
5999,"PFC_02",13,"OID30272","O95825","CRYZL1",0,"Cardiometabolic_II","B13805","20212097_SS211647","PASS",-2.7105,4.2288,"Plate control","PASS","PFC",18.7497570903637,"Baseline","Baseline"
6000,"PFC_10",14,"OID30272","O95825","CRYZL1",0,"Cardiometabolic_II","B13805","20212097_SS211647","PASS",-2.7105,3.9525,"Plate control","PASS","PFC",15.4817859222775,"1h","1h"
6001,"PFC_18",15,"OID30272","O95825","CRYZL1",0,"Cardiometabolic_II","B13805","20212097_SS211647","PASS",-2.7105,5.0919,"Plate control","PASS","PFC",34.1047315123945,"1h","1h"
6002,"PFC_26",16,"OID30272","O95825","CRYZL1",0,"Cardiometabolic_II","B13805","20212097_SS211647","PASS",-2.7105,6.0557,"Plate control","PASS","PFC",66.5192501237434,"24h","24h"
6003,"PFC_03",25,"OID30272","O95825","CRYZL1",0,"Cardiometabolic_II","B13805","20212097_SS211647","PASS",-2.7105,3.0399,"Plate control","PASS","PFC",8.2243405256463,"Baseline","Baseline"
6004,"PFC_11",26,"OID30272","O95825","CRYZL1",0,"Cardiometabolic_II","B13805","20212097_SS211647","PASS",-2.7105,5.724,"Plate control","PASS","PFC",52.8561705558861,"1h","1h"
6005,"PFC_19",27,"OID30272","O95825","CRYZL1",0,"Cardiometabolic_II","B13805","20212097_SS211647","PASS",-2.7105,4.8388,"Plate control","PASS","PFC",28.6169894249411,"24h","24h"
6006,"PFC_27",28,"OID30272","O95825","CRYZL1",0,"Cardiometabolic_II","B13805","20212097_SS211647","PASS",-2.7105,6.5105,"Plate control","PASS","PFC",91.1708044364563,"24h","24h"
6007,"PFC_04",37,"OID30272","O95825","CRYZL1",0,"Cardiometabolic_II","B13805","20212097_SS211647","PASS",-2.7105,5.6449,"Plate control","PASS","PFC",50.036188795708,"Baseline","Baseline"
6008,"PFC_12",38,"OID30272","O95825","CRYZL1",0,"Cardiometabolic_II","B13805","20212097_SS211647","PASS",-2.7105,3.9606,"Plate control","PASS","PFC",15.5689527577811,"1h","1h"
6009,"PFC_20",39,"OID30272","O95825","CRYZL1",0,"Cardiometabolic_II","B13805","20212097_SS211647","PASS",-2.7105,5.9669,"Plate control","PASS","PFC",62.548353378551,"24h","24h"
6010,"PFC_05",49,"OID30272","O95825","CRYZL1",0,"Cardiometabolic_II","B13805","20212097_SS211647","PASS",-2.7105,4.1736,"Plate control","PASS","PFC",18.0459101169359,"Baseline","Baseline"
6011,"PFC_13",50,"OID30272","O95825","CRYZL1",0,"Cardiometabolic_II","B13805","20212097_SS211647","PASS",-2.7105,5.9748,"Plate control","PASS","PFC",62.891799047899,"1h","1h"
6012,"PFC_21",51,"OID30272","O95825","CRYZL1",0,"Cardiometabolic_II","B13805","20212097_SS211647","PASS",-2.7105,4.6787,"Plate control","PASS","PFC",25.6111476987949,"24h","24h"
6013,"PFC_06",61,"OID30272","O95825","CRYZL1",0,"Cardiometabolic_II","B13805","20212097_SS211647","PASS",-2.7105,5.8582,"Plate control","PASS","PFC",58.0088052196822,"Baseline","Baseline"
6014,"PFC_14",62,"OID30272","O95825","CRYZL1",0,"Cardiometabolic_II","B13805","20212097_SS211647","PASS",-2.7105,5.5254,"Plate control","PASS","PFC",46.0586429575913,"1h","1h"
6015,"PFC_22",63,"OID30272","O95825","CRYZL1",0,"Cardiometabolic_II","B13805","20212097_SS211647","PASS",-2.7105,5.2189,"Plate control","PASS","PFC",37.243067518674,"24h","24h"
6016,"PFC_07",73,"OID30272","O95825","CRYZL1",0,"Cardiometabolic_II","B13805","20212097_SS211647","PASS",-2.7105,6.504,"Plate control","PASS","PFC",90.760962283 4853,"Baseline","Baseline"
6017,"PFC_15",74,"OID30272","O95825","CRYZL1",0,"Cardiometabolic_II","B13805","20212097_SS211647","PASS",-2.7105,4.5301,"Plate control","PASS","PFC",23.1044685525817,"1h","1h"
6018,"PFC_23",75,"OID30272","O95825","CRYZL1",0,"Cardiometabolic_II","B13805","20212097_SS211647","PASS",-2.7105,5.7677,"Plate control","PASS","PFC",54.4817069654627,"24h","24h"
6019,"PFC_08",85,"OID30272","O95825","CRYZL1",0,"Cardiometabolic_II","B13805","20212097_SS211647","PASS",-2.7105,5.239,"Plate control","PASS","PFC",37.7655789867585,"Baseline","Baseline"
6020,"PFC_16",86,"OID30272","O95825","CRYZL1",0,"Cardiometabolic_II","B13805","20212097_SS211647","PASS",-2.7105,6.1933,"Plate control","PASS","PFC",73.1760684201661,"1h","1h"
6021,"PFC_24",87,"OID30272","O95825","CRYZL1",0,"Cardiometabolic_II","B13805","20212097_SS211647","PASS",-2.7105,6.3515,"Plate control","PASS","PFC",81.6567362322117,"24h","24h"
6022,"PFC_01",1,"OID30273","Q6UWR7","ENPP6",0,"Cardiometabolic_II","B13805","20212097_SS211647","PASS",-5.4418,1.6471,"Plate control","PASS","PFC",3.13203427357498,"Baseline","Baseline"
6023,"PFC_09",2,"OID30273","Q6UWR7","ENPP6",0,"Cardiometabolic_II","B13805","20212097_SS211647","PASS",-5.4418,1.1199,"Plate control","PASS","PFC",2.17331907683205,"Baseline","Baseline"
6024,"PFC_17",3,"OID30273","Q6UWR7","ENPP6",0,"Cardiometabolic_II","B13805","20212097_SS211647","PASS",-5.4418,1.3385,"Plate control","PASS","PFC",2.52888248893059,"1h","1h"
6025,"PFC_25",4,"OID30273","Q6UWR7","ENPP6",0,"Cardiometabolic_II","B13805","20212097_SS211647","PASS",-5.4418,0.1815,"Plate control","PASS","PFC",1.13406238074923,"24h","24h"
6026,"PFC_02",13,"OID30273","Q6UWR7","ENPP6",0,"Cardiometabolic_II","B13805","20212097_SS211647","PASS",-5.4418,0.9669,"Plate control","PASS","PFC",1.95463604307972,"Baseline","Baseline"
6027,"PFC_10",14,"OID30273","Q6UWR7","ENPP6",0,"Cardiometabolic_II","B13805","20212097_SS211647","PASS",-5.4418,1.1969,"Plate control","PASS","PFC",2.2924654643522,"1h","1h"
6028,"PFC_18",15,"OID30273","Q6UWR7","ENPP6",0,"Cardiometabolic_II","B13805","20212097_SS211647","PASS",-5.4418,0.8783,"Plate control","PASS","PFC",1.83820797181997,"1h","1h"
6029,"PFC_26",16,"OID30273","Q6UWR7","ENPP6",0,"Cardiometabolic_II","B13805","20212097_SS211647","PASS",-5.4418,1.1383,"Plate control","PASS","PFC",2.20121490091517,"24h","24h"
6030,"PFC_03",25,"OID30273","Q6UWR7","ENPP6",0,"Cardiometabolic_II","B13805","20212097_SS211647","PASS",-5.4418,0.6896,"Plate control","PASS","PFC",1.61283628327818,"Baseline","Baseline"
6031,"PFC_11",26,"OID30273","Q6UWR7","ENPP6",0,"Cardiometabolic_II","B13805","20212097_SS211647","PASS",-5.4418,0.8293,"Plate control","PASS","PFC",1.77682303323463,"1h","1h"
6032,"PFC_19",27,"OID30273","Q6UWR7","ENPP6",0,"Cardiometabolic_II","B13805","20212097_SS211647","PASS",-5.4418,1.4441,"Plate control","PASS","PFC",2.72093029879218,"24h","24h"
6033,"PFC_27",28,"OID30273","Q6UWR7","ENPP6",0,"Cardiometabolic_II","B13805","20212097_SS211647","PASS",-5.4418,1.3473,"Plate control","PASS","PFC",2.54435504223818,"24h","24h"
6034,"PFC_04",37,"OID30273","Q6UWR7","ENPP6",0,"Cardiometabolic_II","B13805","20212097_SS211647","PASS",-5.4418,1.2969,"Plate control","PASS","PFC",2.45700364847363,"Baseline","Baseline"
6035,"PFC_12",38,"OID30273","Q6UWR7","ENPP6",0,"Cardiometabolic_II","B13805","20212097_SS211647","PASS",-5.4418,0.8637,"Plate control","PASS","PFC",1.81969921300278,"1h","1h"
6036,"PFC_20",39,"OID30273","Q6UWR7","ENPP6",0,"Cardiometabolic_II","B13805","20212097_SS211647","PASS",-5.4418,0.5878,"Plate control","PASS","PFC",1.50295311008078,"24h","24h"
6037,"PFC_05",49,"OID30273","Q6UWR7","ENPP6",0,"Cardiometabolic_II","B13805","20212097_SS211647","PASS",-5.4418,0.6059,"Plate control","PASS","PFC",1.52192788518094,"Baseline","Baseline"
6038,"PFC_13",50,"OID30273","Q6UWR7","ENPP6",0,"Cardiometabolic_II","B13805","20212097_SS211647","PASS",-5.4418,0.9301,"Plate control","PASS","PFC",1.90540806433345,"1h","1h"
6039,"PFC_21",51,"OID30273","Q6UWR7","ENPP6",0,"Cardiometabolic_II","B13805","20212097_SS211647","PASS",-5.4418,0.8533,"Plate control","PASS","PFC",1.80662865911476,"24h","24h"
6040,"PFC_06",61,"OID30273","Q6UWR7","ENPP6",0,"Cardiometabolic_II","B13805","20212097_SS211647","PASS",-5.4418,0.8068,"Plate control","PASS","PFC",1.74932700478523,"Baseline","Baseline"
6041,"PFC_14",62,"OID30273","Q6UWR7","ENPP6",0,"Cardiometabolic_II","B13805","20212097_SS211647","PASS",-5.4418,0.4914,"Plate control","PASS","PFC",1.40580841903455,"1h","1h"
6042,"PFC_22",63,"OID30273","Q6UWR7","ENPP6",0,"Cardiometabolic_II","B13805","20212097_SS211647","PASS",-5.4418,1.0527,"Plate control","PASS","PFC",2.0744084671645,"24h","24h"
6043,"PFC_07",73,"OID30273","Q6UWR7","ENPP6",0,"Cardiometabolic_II","B13805","20212097_SS211647","PASS",-5.4418,0.824,"Plate control","PASS","PFC",1.77030752915909,"Baseline","Baseline"
6044,"PFC_15",74,"OID30273","Q6UWR7","ENPP6",0,"Cardiometabolic_II","B13805","20212097_SS211647","PASS",-5.4418,0.7743,"Plate control","PASS","PFC",1.71035997816685,"1h","1h"
6045,"PFC_23",75,"OID30273","Q6UWR7","ENPP6",0,"Cardiometabolic_II","B13805","20212097_SS211647","PASS",-5.4418,0.5472,"Plate control","PASS","PFC",1.46124693634392,"24h","24h"
6046,"PFC_08",85,"OID30273","Q6UWR7","ENPP6",0,"Cardiometabolic_II","B13805","20212097_SS211647","PASS",-5.4418,1.136,"Plate control","PASS","PFC",2.19770843515376,"Baseline","Baseline"
6047,"PFC_16",86,"OID30273","Q6UWR7","ENPP6",0,"Cardiometabolic_II","B13805","20212097_SS211647","PASS",-5.4418,0.3453,"Plate control","PASS","PFC",1.27041513047358,"1h","1h"
6048,"PFC_24",87,"OID30273","Q6UWR7","ENPP6",0,"Cardiometabolic_II","B13805","20212097_SS211647","PASS",-5.4418,1.0373,"Plate control","PASS","PFC",2.0523830273567,"24h","24h"

Supplemental table 1_Olink proximity extension assay (PEA) data

,"SampleID","Index","OlinkID","UniProt","Assay","MissingFreq","Panel","Panel_Lot_Nr","PlateID","QC_Warning","LOD","NPX","Normalization","Assay_Warning","condition","Value","group","alt_group"
6049,"PFC_01",1,"OID30274","Q9BV79","MECR",0,"Cardiometabolic_II","B13805","20212097_SS211647","PASS",-0.8992,1.6322,"Plate control","PASS","PFC",3.09985342498372,"Baseline","Baseline"
6050,"PFC_09",2,"OID30274","Q9BV79","MECR",0,"Cardiometabolic_II","B13805","20212097_SS211647","PASS",-0.8992,2.6927,"Plate control","PASS","PFC",6.46522240544964,"Baseline","Baseline"
6051,"PFC_17",3,"OID30274","Q9BV79","MECR",0,"Cardiometabolic_II","B13805","20212097_SS211647","PASS",-0.8992,5.3354,"Plate control","PASS","PFC",40.3752699326771,"1h","1h"
6052,"PFC_25",4,"OID30274","Q9BV79","MECR",0,"Cardiometabolic_II","B13805","20212097_SS211647","PASS",-0.8992,4.2644,"Plate control","PASS","PFC",19.2181825127666,"24h","24h"
6053,"PFC_02",13,"OID30274","Q9BV79","MECR",0,"Cardiometabolic_II","B13805","20212097_SS211647","PASS",-0.8992,2.2143,"Plate control","PASS","PFC",4.64056349574891,"Baseline","Baseline"
6054,"PFC_10",14,"OID30274","Q9BV79","MECR",0,"Cardiometabolic_II","B13805","20212097_SS211647","PASS",-0.8992,1.3958,"Plate control","PASS","PFC",2.63134424300348,"1h","1h"
6055,"PFC_18",15,"OID30274","Q9BV79","MECR",0,"Cardiometabolic_II","B13805","20212097_SS211647","PASS",-0.8992,3.181,"Plate control","PASS","PFC",9.06935530222738,"1h","1h"
6056,"PFC_26",16,"OID30274","Q9BV79","MECR",0,"Cardiometabolic_II","B13805","20212097_SS211647","PASS",-0.8992,4.2012,"Plate control","PASS","PFC",18.3944674064536,"24h","24h"
6057,"PFC_03",25,"OID30274","Q9BV79","MECR",0,"Cardiometabolic_II","B13805","20212097_SS211647","PASS",-0.8992,1.5019,"Plate control","PASS","PFC",2.83215455963055,"Baseline","Baseline"
6058,"PFC_11",26,"OID30274","Q9BV79","MECR",0,"Cardiometabolic_II","B13805","20212097_SS211647","PASS",-0.8992,3.4811,"Plate control","PASS","PFC",11.1664600861857,"1h","1h"
6059,"PFC_19",27,"OID30274","Q9BV79","MECR",0,"Cardiometabolic_II","B13805","20212097_SS211647","PASS",-0.8992,3.2799,"Plate control","PASS","PFC",9.71288580588577,"24h","24h"
6060,"PFC_27",28,"OID30274","Q9BV79","MECR",0,"Cardiometabolic_II","B13805","20212097_SS211647","PASS",-0.8992,3.8049,"Plate control","PASS","PFC",13.9761975448995,"24h","24h"
6061,"PFC_04",37,"OID30274","Q9BV79","MECR",0,"Cardiometabolic_II","B13805","20212097_SS211647","PASS",-0.8992,2.5215,"Plate control","PASS","PFC",5.74178774562787,"Baseline","Baseline"
6062,"PFC_12",38,"OID30274","Q9BV79","MECR",0,"Cardiometabolic_II","B13805","20212097_SS211647","PASS",-0.8992,2.3018,"Plate control","PASS","PFC",4.93072571074849,"1h","1h"
6063,"PFC_20",39,"OID30274","Q9BV79","MECR",0,"Cardiometabolic_II","B13805","20212097_SS211647","PASS",-0.8992,4.2371,"Plate control","PASS","PFC",18.8579376123149,"24h","24h"
6064,"PFC_05",49,"OID30274","Q9BV79","MECR",0,"Cardiometabolic_II","B13805","20212097_SS211647","PASS",-0.8992,1.5938,"Plate control","PASS","PFC",3.01843346430637,"Baseline","Baseline"
6065,"PFC_13",50,"OID30274","Q9BV79","MECR",0,"Cardiometabolic_II","B13805","20212097_SS211647","PASS",-0.8992,3.2632,"Plate control","PASS","PFC",9.60110196208348,"1h","1h"
6066,"PFC_21",51,"OID30274","Q9BV79","MECR",0,"Cardiometabolic_II","B13805","20212097_SS211647","PASS",-0.8992,3.1977,"Plate control","PASS","PFC",9.1749481186036,"24h","24h"
6067,"PFC_06",61,"OID30274","Q9BV79","MECR",0,"Cardiometabolic_II","B13805","20212097_SS211647","PASS",-0.8992,2.594,"Plate control","PASS","PFC",6.03770387408224,"Baseline","Baseline"
6068,"PFC_14",62,"OID30274","Q9BV79","MECR",0,"Cardiometabolic_II","B13805","20212097_SS211647","PASS",-0.8992,3.8049,"Plate control","PASS","PFC",13.9761975448995,"1h","1h"
6069,"PFC_22",63,"OID30274","Q9BV79","MECR",0,"Cardiometabolic_II","B13805","20212097_SS211647","PASS",-0.8992,2.4432,"Plate control","PASS","PFC",5.43846684696312,"24h","24h"
6070,"PFC_07",73,"OID30274","Q9BV79","MECR",0,"Cardiometabolic_II","B13805","20212097_SS211647","PASS",-0.8992,3.9,"Plate control","PASS","PFC",14.9285278645889,"Baseline","Baseline"
6071,"PFC_15",74,"OID30274","Q9BV79","MECR",0,"Cardiometabolic_II","B13805","20212097_SS211647","PASS",-0.8992,1.4756,"Plate control","PASS","PFC",2.7809927815969,"1h","1h"
6072,"PFC_23",75,"OID30274","Q9BV79","MECR",0,"Cardiometabolic_II","B13805","20212097_SS211647","PASS",-0.8992,3.1897,"Plate control","PASS","PFC",9.12421220307571,"24h","24h"
6073,"PFC_08",85,"OID30274","Q9BV79","MECR",0,"Cardiometabolic_II","B13805","20212097_SS211647","PASS",-0.8992,2.4136,"Plate control","PASS","PFC",5.32802185422954,"Baseline","Baseline"
6074,"PFC_16",86,"OID30274","Q9BV79","MECR",0,"Cardiometabolic_II","B13805","20212097_SS211647","PASS",-0.8992,3.3855,"Plate control","PASS","PFC",10.4504995363066,"1h","1h"
6075,"PFC_24",87,"OID30274","Q9BV79","MECR",0,"Cardiometabolic_II","B13805","20212097_SS211647","PASS",-0.8992,3.762,"Plate control","PASS","PFC",13.5667194341304,"24h","24h"
6076,"PFC_01",1,"OID30275","Q6UY14","ADAMTSL4",0,"Cardiometabolic_II","B13805","20212097_SS211647","PASS",-1.8959,1.3986,"Plate control","PASS","PFC",2.63645614678198,"Baseline","Baseline"
6077,"PFC_09",2,"OID30275","Q6UY14","ADAMTSL4",0,"Cardiometabolic_II","B13805","20212097_SS211647","PASS",-1.8959,1.3537,"Plate control","PASS","PFC",2.55566723508623,"Baseline","Baseline"
6078,"PFC_17",3,"OID30275","Q6UY14","ADAMTSL4",0,"Cardiometabolic_II","B13805","20212097_SS211647","PASS",-1.8959,0.9935,"Plate control","PASS","PFC",1.99100935534123,"1h","1h"
6079,"PFC_25",4,"OID30275","Q6UY14","ADAMTSL4",0,"Cardiometabolic_II","B13805","20212097_SS211647","PASS",-1.8959,0.7809,"Plate control","PASS","PFC",1.71820240905115,"24h","24h"
6080,"PFC_02",13,"OID30275","Q6UY14","ADAMTSL4",0,"Cardiometabolic_II","B13805","20212097_SS211647","PASS",-1.8959,1.1187,"Plate control","PASS","PFC",2.17151211244292,"Baseline","Baseline"
6081,"PFC_10",14,"OID30275","Q6UY14","ADAMTSL4",0,"Cardiometabolic_II","B13805","20212097_SS211647","PASS",-1.8959,1.3077,"Plate control","PASS","PFC",2.47546576951504,"1h","1h"
6082,"PFC_18",15,"OID30275","Q6UY14","ADAMTSL4",0,"Cardiometabolic_II","B13805","20212097_SS211647","PASS",-1.8959,1.1849,"Plate control","PASS","PFC",2.27347635563781,"1h","1h"
6083,"PFC_26",16,"OID30275","Q6UY14","ADAMTSL4",0,"Cardiometabolic_II","B13805","20212097_SS211647","PASS",-1.8959,1.0485,"Plate control","PASS","PFC",2.06837819354706,"24h","24h"
6084,"PFC_03",25,"OID30275","Q6UY14","ADAMTSL4",0,"Cardiometabolic_II","B13805","20212097_SS211647","PASS",-1.8959,0.6384,"Plate control","PASS","PFC",1.5566018749668,"Baseline","Baseline"
6085,"PFC_11",26,"OID30275","Q6UY14","ADAMTSL4",0,"Cardiometabolic_II","B13805","20212097_SS211647","PASS",-1.8959,1.1875,"Plate control","PASS","PFC",2.27757726951338,"1h","1h"
6086,"PFC_19",27,"OID30275","Q6UY14","ADAMTSL4",0,"Cardiometabolic_II","B13805","20212097_SS211647","PASS",-1.8959,1.5258,"Plate control","PASS","PFC",2.87946343096741,"24h","24h"
6087,"PFC_27",28,"OID30275","Q6UY14","ADAMTSL4",0,"Cardiometabolic_II","B13805","20212097_SS211647","PASS",-1.8959,1.4723,"Plate control","PASS","PFC",2.77463884820658,"24h","24h"
6088,"PFC_04",37,"OID30275","Q6UY14","ADAMTSL4",0,"Cardiometabolic_II","B13805","20212097_SS211647","PASS",-1.8959,0.6216,"Plate control","PASS","PFC",1.53858057590284,"Baseline","Baseline"
6089,"PFC_12",38,"OID30275","Q6UY14","ADAMTSL4",0,"Cardiometabolic_II","B13805","20212097_SS211647","PASS",-1.8959,0.6788,"Plate control","PASS","PFC",1.60080768686268,"1h","1h"
6090,"PFC_20",39,"OID30275","Q6UY14","ADAMTSL4",0,"Cardiometabolic_II","B13805","20212097_SS211647","PASS",-1.8959,1.1055,"Plate control","PASS","PFC",2.15173438601526,"24h","24h"
6091,"PFC_05",49,"OID30275","Q6UY14","ADAMTSL4",0,"Cardiometabolic_II","B13805","20212097_SS211647","PASS",-1.8959,0.9952,"Plate control","PASS","PFC",1.99335684443765,"Baseline","Baseline"
6092,"PFC_13",50,"OID30275","Q6UY14","ADAMTSL4",0,"Cardiometabolic_II","B13805","20212097_SS211647","PASS",-1.8959,0.5936,"Plate control","PASS","PFC",1.50900752479012,"1h","1h"
6093,"PFC_21",51,"OID30275","Q6UY14","ADAMTSL4",0,"Cardiometabolic_II","B13805","20212097_SS211647","PASS",-1.8959,0.6942,"Plate control","PASS","PFC",1.61798698179979,"24h","24h"
6094,"PFC_06",61,"OID30275","Q6UY14","ADAMTSL4",0,"Cardiometabolic_II","B13805","20212097_SS211647","PASS",-1.8959,0.9296,"Plate control","PASS","PFC",1.9047478146388,"Baseline","Baseline"
6095,"PFC_14",62,"OID30275","Q6UY14","ADAMTSL4",0,"Cardiometabolic_II","B13805","20212097_SS211647","PASS",-1.8959,1.2736,"Plate control","PASS","PFC",2.41764094633088,"1h","1h"
6096,"PFC_22",63,"OID30275","Q6UY14","ADAMTSL4",0,"Cardiometabolic_II","B13805","20212097_SS211647","PASS",-1.8959,0.794,"Plate control","PASS","PFC",1.73387512702937,"24h","24h"
6097,"PFC_07",73,"OID30275","Q6UY14","ADAMTSL4",0,"Cardiometabolic_II","B13805","20212097_SS211647","PASS",-1.8959,0.7529,"Plate control","PASS","PFC",1.68517684739992,"Baseline","Baseline"
6098,"PFC_15",74,"OID30275","Q6UY14","ADAMTSL4",0,"Cardiometabolic_II","B13805","20212097_SS211647","PASS",-1.8959,0.8415,"Plate control","PASS","PFC",1.7919122620405,"1h","1h"
6099,"PFC_23",75,"OID30275","Q6UY14","ADAMTSL4",0,"Cardiometabolic_II","B13805","20212097_SS211647","PASS",-1.8959,1.226,"Plate control","PASS","PFC",2.33917532810389,"24h","24h"
6100,"PFC_08",85,"OID30275","Q6UY14","ADAMTSL4",0,"Cardiometabolic_II","B13805","20212097_SS211647","PASS",-1.8959,0.7593,"Plate control","PASS","PFC",1.69266913725899,"Baseline","Baseline"
6101,"PFC_16",86,"OID30275","Q6UY14","ADAMTSL4",0,"Cardiometabolic_II","B13805","20212097_SS211647","PASS",-1.8959,0.8402,"Plate control","PASS","PFC",1.79029831269922,"1h","1h"
6102,"PFC_24",87,"OID30275","Q6UY14","ADAMTSL4",0,"Cardiometabolic_II","B13805","20212097_SS211647","PASS",-1.8959,1.2634,"Plate control","PASS","PFC",2.40060826242409,"24h","24h"
6103,"PFC_01",1,"OID30276","Q5FWE3","PRRT3",0,"Cardiometabolic_II","B13805","20212097_SS211647","PASS",-6.3119,1.1145,"Plate control","PASS","PFC",2.16519956001706,"Baseline","Baseline"
6104,"PFC_09",2,"OID30276","Q5FWE3","PRRT3",0,"Cardiometabolic_II","B13805","20212097_SS211647","PASS",-6.3119,1.6835,"Plate control","PASS","PFC",3.21206257804033,"Baseline","Baseline"
6105,"PFC_17",3,"OID30276","Q5FWE3","PRRT3",0,"Cardiometabolic_II","B13805","20212097_SS211647","PASS",-6.3119,1.1576,"Plate control","PASS","PFC",2.23086003315925,"1h","1h"
6106,"PFC_25",4,"OID30276","Q5FWE3","PRRT3",0,"Cardiometabolic_II","B13805","20212097_SS211647","PASS",-6.3119,1.2615,"Plate control","PASS","PFC",2.39744879114784,"24h","24h"
6107,"PFC_02",13,"OID30276","Q5FWE3","PRRT3",0,"Cardiometabolic_II","B13805","20212097_SS211647","PASS",-6.3119,1.2472,"Plate control","PASS","PFC",2.37380265142711,"Baseline","Baseline"
6108,"PFC_10",14,"OID30276","Q5FWE3","PRRT3",0,"Cardiometabolic_II","B13805","20212097_SS211647","PASS",-6.3119,1.2565,"Plate control","PASS","PFC",2.38915424844683,"1h","1h"
6109,"PFC_18",15,"OID30276","Q5FWE3","PRRT3",0,"Cardiometabolic_II","B13805","20212097_SS211647","PASS",-6.3119,1.301,"Plate control","PASS","PFC",2.46399614689151,"1h","1h"
6110,"PFC_26",16,"OID30276","Q5FWE3","PRRT3",0,"Cardiometabolic_II","B13805","20212097_SS211647","PASS",-6.3119,1.3956,"Plate control","PASS","PFC",2.63097948651843,"24h","24h"
6111,"PFC_03",25,"OID30276","Q5FWE3","PRRT3",0,"Cardiometabolic_II","B13805","20212097_SS211647","PASS",-6.3119,1.1324,"Plate control","PASS","PFC",2.19223126421001,"Baseline","Baseline"
6112,"PFC_11",26,"OID30276","Q5FWE3","PRRT3",0,"Cardiometabolic_II","B13805","20212097_SS211647","PASS",-6.3119,1.243,"Plate control","PASS","PFC",2.36690204350514,"1h","1h"
6113,"PFC_19",27,"OID30276","Q5FWE3","PRRT3",0,"Cardiometabolic_II","B13805","20212097_SS211647","PASS",-6.3119,1.3473,"Plate control","PASS","PFC",2.54435504223818,"24h","24h"
6114,"PFC_27",28,"OID30276","Q5FWE3","PRRT3",0,"Cardiometabolic_II","B13805","20212097_SS211647","PASS",-6.3119,1.7363,"Plate control","PASS","PFC",3.33179583896007,"24h","24h"
6115,"PFC_04",37,"OID30276","Q5FWE3","PRRT3",0,"Cardiometabolic_II","B13805","20212097_SS211647","PASS",-6.3119,1.2555,"Plate control","PASS","PFC",2.38749878672119,"Baseline","Baseline"
6116,"PFC_12",38,"OID30276","Q5FWE3","PRRT3",0,"Cardiometabolic_II","B13805","20212097_SS211647","PASS",-6.3119,1.0858,"Plate control","PASS","PFC",2.12255214929534,"1h","1h"
6117,"PFC_20",39,"OID30276","Q5FWE3","PRRT3",0,"Cardiometabolic_II","B13805","20212097_SS211647","PASS",-6.3119,1.1848,"Plate control","PASS","PFC",2.27331877572658,"24h","24h"
6118,"PFC_05",49,"OID30276","Q5FWE3","PRRT3",0,"Cardiometabolic_II","B13805","20212097_SS211647","PASS",-6.3119,0.6261,"Plate control","PASS","PFC",1.5433871507966,"Baseline","Baseline"
6119,"PFC_13",50,"OID30276","Q5FWE3","PRRT3",0,"Cardiometabolic_II","B13805","20212097_SS211647","PASS",-6.3119,1.0617,"Plate control","PASS","PFC",2.08738974921432,"1h","1h"
6120,"PFC_21",51,"OID30276","Q5FWE3","PRRT3",0,"Cardiometabolic_II","B13805","20212097_SS211647","PASS",-6.3119,1.0042,"Plate control","PASS","PFC",2.00583091973823,"24h","24h"
6121,"PFC_06",61,"OID30276","Q5FWE3","PRRT3",0,"Cardiometabolic_II","B13805","20212097_SS211647","PASS",-6.3119,1.1011,"Plate control","PASS","PFC",2.14518192116275,"Baseline","Baseline"
6122,"PFC_14",62,"OID30276","Q5FWE3","PRRT3",0,"Cardiometabolic_II","B13805","20212097_SS211647","PASS",-6.3119,0.3183,"Plate control","PASS","PFC",1.24686044465989,"1h","1h"
6123,"PFC_22",63,"OID30276","Q5FWE3","PRRT3",0,"Cardiometabolic_II","B13805","20212097_SS211647","PASS",-6.3119,0.9801,"Plate control","PASS","PFC",1.97260213460891,"24h","24h"
6124,"PFC_07",73,"OID30276","Q5FWE3","PRRT3",0,"Cardiometabolic_II","B13805","20212097_SS211647","PASS",-6.3119,1.4856,"Plate control","PASS","PFC",2.80033611609667,"Baseline","Baseline"
6125,"PFC_15",74,"OID30276","Q5FWE3","PRRT3",0,"Cardiometabolic_II","B13805","20212097_SS211647","PASS",-6.3119,0.8318,"Plate control","PASS","PFC",1.77990470221579,"1h","1h"
6126,"PFC_23",75,"OID30276","Q5FWE3","PRRT3",0,"Cardiometabolic_II","B13805","20212097_SS211647","PASS",-6.3119,0.4007,"Plate control","PASS","PFC",1.32014829535025,"24h","24h"
6127,"PFC_08",85,"OID30276","Q5FWE3","PRRT3",0,"Cardiometabolic_II","B13805","20212097_SS211647","PASS",-6.3119,1.1454,"Plate control","PASS","PFC",2.2120745389165,"Baseline","Baseline"
6128,"PFC_16",86,"OID30276","Q5FWE3","PRRT3",0,"Cardiometabolic_II","B13805","20212097_SS211647","PASS",-6.3119,1.2773,"Plate control","PASS","PFC",2.42384929375078,"1h","1h"
6129,"PFC_24",87,"OID30276","Q5FWE3","PRRT3",0,"Cardiometabolic_II","B13805","20212097_SS211647","PASS",-6.3119,1.1191,"Plate control","PASS","PFC",2.17211426691471,"24h","24h"
6130,"PFC_01",1,"OID30277","Q8IWT1","SCN4B",0,"Cardiometabolic_II","B13805","20212097_SS211647","PASS",-5.5582,0.2378,"Plate control","PASS","PFC",1.17919311005384,"Baseline","Baseline"
6131,"PFC_09",2,"OID30277","Q8IWT1","SCN4B",0,"Cardiometabolic_II","B13805","20212097_SS211647","PASS",-5.5582,0.5402,"Plate control","PASS","PFC",1.45417409468246,"Baseline","Baseline"
6132,"PFC_17",3,"OID30277","Q8IWT1","SCN4B",0,"Cardiometabolic_II","B13805","20212097_SS211647","PASS",-5.5582,-0.4488,"Plate control","PASS","PFC",0.732651997399163,"1h","1h"
6133,"PFC_25",4,"OID30277","Q8IWT1","SCN4B",0,"Cardiometabolic_II","B13805","20212097_SS211647","PASS",-5.5582,0.0935,"Plate control","PASS","PFC",1.06695549535811,"24h","24h"
6134,"PFC_02",13,"OID30277","Q8IWT1","SCN4B",0,"Cardiometabolic_II","B13805","20212097_SS211647","PASS",-5.5582,0.3765,"Plate control","PASS","PFC",1.2981886068697,"Baseline","Baseline"
6135,"PFC_10",14,"OID30277","Q8IWT1","SCN4B",0,"Cardiometabolic_II","B13805","20212097_SS211647","PASS",-5.5582,0.3377,"Plate control","PASS","PFC",1.26374028372397,"1h","1h"
6136,"PFC_18",15,"OID30277","Q8IWT1","SCN4B",0,"Cardiometabolic_II","B13805","20212097_SS211647","PASS",-5.5582,0.3652,"Plate control","PASS","PFC",1.28806018018981,"1h","1h"
6137,"PFC_26",16,"OID30277","Q8IWT1","SCN4B",0,"Cardiometabolic_II","B13805","20212097_SS211647","PASS",-5.5582,-0.3718,"Plate control","PASS","PFC",0.772817677501128,"24h","24h"
6138,"PFC_03",25,"OID30277","Q8IWT1","SCN4B",0,"Cardiometabolic_II","B13805","20212097_SS211647","PASS",-5.5582,-0.1422,"Plate control","PASS","PFC",0.906136312393786,"Baseline","Baseline"
6139,"PFC_11",26,"OID30277","Q8IWT1","SCN4B",0,"Cardiometabolic_II","B13805","20212097_SS211647","PASS",-5.5582,0.6444,"Plate control","PASS","PFC",1.56308908058659,"1h","1h"
6140,"PFC_19",27,"OID30277","Q8IWT1","SCN4B",0,"Cardiometabolic_II","B13805","20212097_SS211647","PASS",-5.5582,0.0665,"Plate control","PASS","PFC",1.0471731416475,"24h","24h"
6141,"PFC_27",28,"OID30277","Q8IWT1","SCN4B",0,"Cardiometabolic_II","B13805","20212097_SS211647","PASS",-5.5582,0.7192,"Plate control","PASS","PFC",1.6462688960678,"24h","24h"
6142,"PFC_04",37,"OID30277","Q8IWT1","SCN4B",0,"Cardiometabolic_II","B13805","20212097_SS211647","PASS",-5.5582,-0.1473,"Plate control","PASS","PFC",0.902938729798152,"Baseline","Baseline"
6143,"PFC_12",38,"OID30277","Q8IWT1","SCN4B",0,"Cardiometabolic_II","B13805","20212097_SS211647","PASS",-5.5582,-0.2322,"Plate control","PASS","PFC",0.851335679382856,"1h","1h"
6144,"PFC_20",39,"OID30277","Q8IWT1","SCN4B",0,"Cardiometabolic_II","B13805","20212097_SS211647","PASS",-5.5582,0.0931,"Plate control","PASS","PFC",1.06665971348673,"24h","24h"
6145,"PFC_05",49,"OID30277","Q8IWT1","SCN4B",0,"Cardiometabolic_II","B13805","20212097_SS211647","PASS",-5.5582,0.0669,"Plate control","PASS","PFC",1.0474635199449,"Baseline","Baseline"
6146,"PFC_13",50,"OID30277","Q8IWT1","SCN4B",0,"Cardiometabolic_II","B13805","20212097_SS211647","PASS",-5.5582,-0.1818,"Plate control","PASS","PFC",0.881602365474992,"1h","1h"
6147,"PFC_21",51,"OID30277","Q8IWT1","SCN4B",0,"Cardiometabolic_II","B13805","20212097_SS211647","PASS",-5.5582,0.0611,"Plate control","PASS","PFC",1.04326090435917,"24h","24h"
6148,"PFC_06",61,"OID30277","Q8IWT1","SCN4B",0,"Cardiometabolic_II","B13805","20212097_SS211647","PASS",-5.5582,0.8863,"Plate control","PASS","PFC",1.84842947503638,"Baseline","Baseline"
6149,"PFC_14",62,"OID30277","Q8IWT1","SCN4B",0,"Cardiometabolic_II","B13805","20212097_SS211647","PASS",-5.5582,0.2544,"Plate control","PASS","PFC",1.19283955183549,"1h","1h"
6150,"PFC_22",63,"OID30277","Q8IWT1","SCN4B",0,"Cardiometabolic_II","B13805","20212097_SS211647","PASS",-5.5582,-0.0935,"Plate control","PASS","PFC",0.937246215376921,"24h","24h"
6151,"PFC_07",73,"OID30277","Q8IWT1","SCN4B",0,"Cardiometabolic_II","B13805","20212097_SS211647","PASS",-5.5582,0.3702,"Plate control","PASS","PFC",1.29253200120053,"Baseline","Baseline"
6152,"PFC_15",74,"OID30277","Q8IWT1","SCN4B",0,"Cardiometabolic_II","B13805","20212097_SS211647","PASS",-5.5582,0.8671,"Plate control","PASS","PFC",1.82399275620156,"1h","1h"
6153,"PFC_23",75,"OID30277","Q8IWT1","SCN4B",0,"Cardiometabolic_II","B13805","20212097_SS211647","PASS",-5.5582,0.1916,"Plate control","PASS","PFC",1.14202956511989,"24h","24h"
6154,"PFC_08",85,"OID30277","Q8IWT1","SCN4B",0,"Cardiometabolic_II","B13805","20212097_SS211647","PASS",-5.5582,-0.498,"Plate control","PASS","PFC",0.708087719107272,"Baseline","Baseline"
6155,"PFC_16",86,"OID30277","Q8IWT1","SCN4B",0,"Cardiometabolic_II","B13805","20212097_SS211647","PASS",-5.5582,0.1938,"Plate control","PASS","PFC",1.14377240168963,"1h","1h"
6156,"PFC_24",87,"OID30277","Q8IWT1","SCN4B",0,"Cardiometabolic_II","B13805","20212097_SS211647","PASS",-5.5582,1.0959,"Plate control","PASS","PFC",2.13746381958513,"24h","24h"
6157,"PFC_01",1,"OID30278","Q9HB40","SCPEP1",0,"Cardiometabolic_II","B13805","20212097_SS211647","PASS",-3.4457,1.0613,"Plate control","PASS","PFC",2.08681108211056,"Baseline","Baseline"
6158,"PFC_09",2,"OID30278","Q9HB40","SCPEP1",0,"Cardiometabolic_II","B13805","20212097_SS211647","PASS",-3.4457,1.2387,"Plate control","PASS","PFC",2.35985791698932,"Baseline","Baseline"
6159,"PFC_17",3,"OID30278","Q9HB40","SCPEP1",0,"Cardiometabolic_II","B13805","20212097_SS211647","PASS",-3.4457,3.0035,"Plate control","PASS","PFC",8.01943168230282,"1h","1h"
6160,"PFC_25",4,"OID30278","Q9HB40","SCPEP1",0,"Cardiometabolic_II","B13805","20212097_SS211647","PASS",-3.4457,2.2538,"Plate control","PASS","PFC",4.76937426782585,"24h","24h"

Supplemental table 1_Olink proximity extension assay (PEA) data

,"SampleID","Index","OlinkID","UniProt","Assay","MissingFreq","Panel","Panel_Lot_Nr","PlateID","QC_Warning","LOD","NPX","Normalization","Assay_Warning","condition","Value","group","alt_group"
6161,"PFC_02",13,"OID30278","Q9HB40","SCPEP1",0,"Cardiometabolic_II","B13805","20212097_SS211647","PASS",-3.4457,1.2215,"Plate control","PASS","PFC",2.33189042787755,"Baseline","Baseline"
6162,"PFC_10",14,"OID30278","Q9HB40","SCPEP1",0,"Cardiometabolic_II","B13805","20212097_SS211647","PASS",-3.4457,1.3225,"Plate control","PASS","PFC",2.50099123249738,"1h","1h"
6163,"PFC_18",15,"OID30278","Q9HB40","SCPEP1",0,"Cardiometabolic_II","B13805","20212097_SS211647","PASS",-3.4457,1.3893,"Plate control","PASS","PFC",2.61951550247161,"1h","1h"
6164,"PFC_26",16,"OID30278","Q9HB40","SCPEP1",0,"Cardiometabolic_II","B13805","20212097_SS211647","PASS",-3.4457,2.5249,"Plate control","PASS","PFC",5.75533537676816,"24h","24h"
6165,"PFC_03",25,"OID30278","Q9HB40","SCPEP1",0,"Cardiometabolic_II","B13805","20212097_SS211647","PASS",-3.4457,0.8121,"Plate control","PASS","PFC",1.75576529137747,"Baseline","Baseline"
6166,"PFC_11",26,"OID30278","Q9HB40","SCPEP1",0,"Cardiometabolic_II","B13805","20212097_SS211647","PASS",-3.4457,2.1608,"Plate control","PASS","PFC",4.47162746169981,"1h","1h"
6167,"PFC_19",27,"OID30278","Q9HB40","SCPEP1",0,"Cardiometabolic_II","B13805","20212097_SS211647","PASS",-3.4457,1.9816,"Plate control","PASS","PFC",3.94930831320191,"24h","24h"
6168,"PFC_27",28,"OID30278","Q9HB40","SCPEP1",0,"Cardiometabolic_II","B13805","20212097_SS211647","PASS",-3.4457,2.5122,"Plate control","PASS","PFC",5.70489368083448,"24h","24h"
6169,"PFC_04",37,"OID30278","Q9HB40","SCPEP1",0,"Cardiometabolic_II","B13805","20212097_SS211647","PASS",-3.4457,1.9277,"Plate control","PASS","PFC",3.8044819032957,"Baseline","Baseline"
6170,"PFC_12",38,"OID30278","Q9HB40","SCPEP1",0,"Cardiometabolic_II","B13805","20212097_SS211647","PASS",-3.4457,1.3544,"Plate control","PASS","PFC",2.55690755344192,"1h","1h"
6171,"PFC_20",39,"OID30278","Q9HB40","SCPEP1",0,"Cardiometabolic_II","B13805","20212097_SS211647","PASS",-3.4457,2.3657,"Plate control","PASS","PFC",5.15402666078576,"24h","24h"
6172,"PFC_05",49,"OID30278","Q9HB40","SCPEP1",0,"Cardiometabolic_II","B13805","20212097_SS211647","PASS",-3.4457,1.1451,"Plate control","PASS","PFC",2.21161459877014,"Baseline","Baseline"
6173,"PFC_13",50,"OID30278","Q9HB40","SCPEP1",0,"Cardiometabolic_II","B13805","20212097_SS211647","PASS",-3.4457,2.1601,"Plate control","PASS","PFC",4.46945834079706,"1h","1h"
6174,"PFC_21",51,"OID30278","Q9HB40","SCPEP1",0,"Cardiometabolic_II","B13805","20212097_SS211647","PASS",-3.4457,1.5352,"Plate control","PASS","PFC",2.89828606902468,"24h","24h"
6175,"PFC_06",61,"OID30278","Q9HB40","SCPEP1",0,"Cardiometabolic_II","B13805","20212097_SS211647","PASS",-3.4457,1.5892,"Plate control","PASS","PFC",3.00882458552238,"Baseline","Baseline"
6176,"PFC_14",62,"OID30278","Q9HB40","SCPEP1",0,"Cardiometabolic_II","B13805","20212097_SS211647","PASS",-3.4457,2.3847,"Plate control","PASS","PFC",5.22235307764506,"1h","1h"
6177,"PFC_22",63,"OID30278","Q9HB40","SCPEP1",0,"Cardiometabolic_II","B13805","20212097_SS211647","PASS",-3.4457,1.7497,"Plate control","PASS","PFC",3.36288629575659,"24h","24h"
6178,"PFC_07",73,"OID30278","Q9HB40","SCPEP1",0,"Cardiometabolic_II","B13805","20212097_SS211647","PASS",-3.4457,2.4318,"Plate control","PASS","PFC",5.39566208791646,"Baseline","Baseline"
6179,"PFC_15",74,"OID30278","Q9HB40","SCPEP1",0,"Cardiometabolic_II","B13805","20212097_SS211647","PASS",-3.4457,0.8531,"Plate control","PASS","PFC",1.80637822456168,"1h","1h"
6180,"PFC_23",75,"OID30278","Q9HB40","SCPEP1",0,"Cardiometabolic_II","B13805","20212097_SS211647","PASS",-3.4457,2.1235,"Plate control","PASS","PFC",4.35749799340224,"24h","24h"
6181,"PFC_08",85,"OID30278","Q9HB40","SCPEP1",0,"Cardiometabolic_II","B13805","20212097_SS211647","PASS",-3.4457,1.6589,"Plate control","PASS","PFC",3.15775666020368,"Baseline","Baseline"
6182,"PFC_16",86,"OID30278","Q9HB40","SCPEP1",0,"Cardiometabolic_II","B13805","20212097_SS211647","PASS",-3.4457,2.2783,"Plate control","PASS","PFC",4.85105992092451,"1h","1h"
6183,"PFC_24",87,"OID30278","Q9HB40","SCPEP1",0,"Cardiometabolic_II","B13805","20212097_SS211647","PASS",-3.4457,2.1833,"Plate control","PASS","PFC",4.5419127746033 8,"24h","24h"
6184,"PFC_01",1,"OID30279","Q96DR5","BPIFA2",0.0455,"Cardiometabolic_II","B13805","20212097_SS211647","PASS",-3.6645,-0.417,"Plate control","PASS","PFC",0.748980467212091,"Baseline","Baseline"
6185,"PFC_09",2,"OID30279","Q96DR5","BPIFA2",0.0455,"Cardiometabolic_II","B13805","20212097_SS211647","PASS",-3.6645,-1.6264,"Plate control","PASS","PFC",0.323895426027321,"Baseline","Baseline"
6186,"PFC_17",3,"OID30279","Q96DR5","BPIFA2",0.0455,"Cardiometabolic_II","B13805","20212097_SS211647","PASS",-3.6645,-0.4897,"Plate control","PASS","PFC",0.712173174650568,"1h","1h"
6187,"PFC_25",4,"OID30279","Q96DR5","BPIFA2",0.0455,"Cardiometabolic_II","B13805","20212097_SS211647","PASS",-3.6645,-2.0124,"Plate control","PASS","PFC",0.247860451647576,"24h","24h"
6188,"PFC_02",13,"OID30279","Q96DR5","BPIFA2",0.0455,"Cardiometabolic_II","B13805","20212097_SS211647","PASS",-3.6645,-2.5427,"Plate control","PASS","PFC",0.171621237829741,"Baseline","Baseline"
6189,"PFC_10",14,"OID30279","Q96DR5","BPIFA2",0.0455,"Cardiometabolic_II","B13805","20212097_SS211647","PASS",-3.6645,-1.523,"Plate control","PASS","PFC",0.347961597957775,"1h","1h"
6190,"PFC_18",15,"OID30279","Q96DR5","BPIFA2",0.0455,"Cardiometabolic_II","B13805","20212097_SS211647","PASS",-3.6645,-2.7116,"Plate control","PASS","PFC",0.152660634731902,"1h","1h"
6191,"PFC_26",16,"OID30279","Q96DR5","BPIFA2",0.0455,"Cardiometabolic_II","B13805","20212097_SS211647","PASS",-3.6645,-0.8925,"Plate control","PASS","PFC",0.538679847957885,"24h","24h"
6192,"PFC_03",25,"OID30279","Q96DR5","BPIFA2",0.0455,"Cardiometabolic_II","B13805","20212097_SS211647","PASS",-3.6645,-2.3353,"Plate control","PASS","PFC",0.198154824730668,"Baseline","Baseline"
6193,"PFC_11",26,"OID30279","Q96DR5","BPIFA2",0.0455,"Cardiometabolic_II","B13805","20212097_SS211647","PASS",-3.6645,-3.1957,"Plate control","PASS","PFC",0.10914364231454,"1h","1h"
6194,"PFC_19",27,"OID30279","Q96DR5","BPIFA2",0.0455,"Cardiometabolic_II","B13805","20212097_SS211647","PASS",-3.6645,-1.2133,"Plate control","PASS","PFC",0.431280980587504,"24h","24h"
6195,"PFC_27",28,"OID30279","Q96DR5","BPIFA2",0.0455,"Cardiometabolic_II","B13805","20212097_SS211647","PASS",-3.6645,-0.9855,"Plate control","PASS","PFC",0.505050655689223,"24h","24h"
6196,"PFC_04",37,"OID30279","Q96DR5","BPIFA2",0.0455,"Cardiometabolic_II","B13805","20212097_SS211647","PASS",-3.6645,-0.8844,"Plate control","PASS","PFC",0.541712767927958,"Baseline","Baseline"
6197,"PFC_12",38,"OID30279","Q96DR5","BPIFA2",0.0455,"Cardiometabolic_II","B13805","20212097_SS211647","PASS",-3.6645,-2.7078,"Plate control","PASS","PFC",0.153063266653645,"1h","1h"
6198,"PFC_20",39,"OID30279","Q96DR5","BPIFA2",0.0455,"Cardiometabolic_II","B13805","20212097_SS211647","PASS",-3.6645,-3.5596,"Plate control","PASS","PFC",0.0848112818863216,"24h","24h"
6199,"PFC_05",49,"OID30279","Q96DR5","BPIFA2",0.0455,"Cardiometabolic_II","B13805","20212097_SS211647","PASS",-3.6645,-2.0066,"Plate control","PASS","PFC",0.248858919233993,"Baseline","Baseline"
6200,"PFC_13",50,"OID30279","Q96DR5","BPIFA2",0.0455,"Cardiometabolic_II","B13805","20212097_SS211647","PASS",-3.6645,-1.0789,"Plate control","PASS","PFC",0.473389627349726,"1h","1h"
6201,"PFC_21",51,"OID30279","Q96DR5","BPIFA2",0.0455,"Cardiometabolic_II","B13805","20212097_SS211647","PASS",-3.6645,-2.6259,"Plate control","PASS","PFC",0.162003849541154,"24h","24h"
6202,"PFC_06",61,"OID30279","Q96DR5","BPIFA2",0.0455,"Cardiometabolic_II","B13805","20212097_SS211647","PASS",-3.6645,-0.5998,"Plate control","PASS","PFC",0.659845423045163,"Baseline","Baseline"
6203,"PFC_14",62,"OID30279","Q96DR5","BPIFA2",0.0455,"Cardiometabolic_II","B13805","20212097_SS211647","PASS",-3.6645,-2.2564,"Plate control","PASS","PFC",0.20929358532847 3,"1h","1h"
6204,"PFC_22",63,"OID30279","Q96DR5","BPIFA2",0.0455,"Cardiometabolic_II","B13805","20212097_SS211647","PASS",-3.6645,-0.7819,"Plate control","PASS","PFC",0.581600332839892,"24h","24h"
6205,"PFC_07",73,"OID30279","Q96DR5","BPIFA2",0.0455,"Cardiometabolic_II","B13805","20212097_SS211647","PASS",-3.6645,-1.4696,"Plate control","PASS","PFC",0.36108239830179,"Baseline","Baseline"
6206,"PFC_15",74,"OID30279","Q96DR5","BPIFA2",0.0455,"Cardiometabolic_II","B13805","20212097_SS211647","PASS",-3.6645,-0.093,"Plate control","PASS","PFC",0.93757109645711,"1h","1h"
6207,"PFC_23",75,"OID30279","Q96DR5","BPIFA2",0.0455,"Cardiometabolic_II","B13805","20212097_SS211647","PASS",-3.6645,-1.8687,"Plate control","PASS","PFC",0.27382005105762,"24h","24h"
6208,"PFC_08",85,"OID30279","Q96DR5","BPIFA2",0.0455,"Cardiometabolic_II","B13805","20212097_SS211647","PASS",-3.6645,-0.5701,"Plate control","PASS","PFC",0.673570098493246,"Baseline","Baseline"
6209,"PFC_16",86,"OID30279","Q96DR5","BPIFA2",0.0455,"Cardiometabolic_II","B13805","20212097_SS211647","PASS",-3.6645,-1.481,"Plate control","PASS","PFC",0.358240412593275,"1h","1h"
6210,"PFC_24",87,"OID30279","Q96DR5","BPIFA2",0.0455,"Cardiometabolic_II","B13805","20212097_SS211647","PASS",-3.6645,-0.1033,"Plate control","PASS","PFC",0.930901224234703,"24h","24h"
6211,"PFC_01",1,"OID30280","P07942","LAMB1",0,"Cardiometabolic_II","B13805","20212097_SS211647","PASS",-4.4422,0.2369,"Plate control","PASS","PFC",1.17845772051601,"Baseline","Baseline"
6212,"PFC_09",2,"OID30280","P07942","LAMB1",0,"Cardiometabolic_II","B13805","20212097_SS211647","PASS",-4.4422,0.216,"Plate control","PASS","PFC",1.16150873192572,"Baseline","Baseline"
6213,"PFC_17",3,"OID30280","P07942","LAMB1",0,"Cardiometabolic_II","B13805","20212097_SS211647","PASS",-4.4422,0.2008,"Plate control","PASS","PFC",1.14933550525714,"1h","1h"
6214,"PFC_25",4,"OID30280","P07942","LAMB1",0,"Cardiometabolic_II","B13805","20212097_SS211647","PASS",-4.4422,0.214,"Plate control","PASS","PFC",1.15989965450543,"24h","24h"
6215,"PFC_02",13,"OID30280","P07942","LAMB1",0,"Cardiometabolic_II","B13805","20212097_SS211647","PASS",-4.4422,0.3345,"Plate control","PASS","PFC",1.26094032447929,"Baseline","Baseline"
6216,"PFC_10",14,"OID30280","P07942","LAMB1",0,"Cardiometabolic_II","B13805","20212097_SS211647","PASS",-4.4422,0.1957,"Plate control","PASS","PFC",1.1452797189942,"1h","1h"
6217,"PFC_18",15,"OID30280","P07942","LAMB1",0,"Cardiometabolic_II","B13805","20212097_SS211647","PASS",-4.4422,-0.1696,"Plate control","PASS","PFC",0.889089154852864,"1h","1h"
6218,"PFC_26",16,"OID30280","P07942","LAMB1",0,"Cardiometabolic_II","B13805","20212097_SS211647","PASS",-4.4422,0.2644,"Plate control","PASS","PFC",1.20113640704791,"24h","24h"
6219,"PFC_03",25,"OID30280","P07942","LAMB1",0,"Cardiometabolic_II","B13805","20212097_SS211647","PASS",-4.4422,-0.293,"Plate control","PASS","PFC",0.816203046150902,"Baseline","Baseline"
6220,"PFC_11",26,"OID30280","P07942","LAMB1",0,"Cardiometabolic_II","B13805","20212097_SS211647","PASS",-4.4422,0.4479,"Plate control","PASS","PFC",1.36405328263376,"1h","1h"
6221,"PFC_19",27,"OID30280","P07942","LAMB1",0,"Cardiometabolic_II","B13805","20212097_SS211647","PASS",-4.4422,0.3416,"Plate control","PASS","PFC",1.26716114165595,"24h","24h"
6222,"PFC_27",28,"OID30280","P07942","LAMB1",0,"Cardiometabolic_II","B13805","20212097_SS211647","PASS",-4.4422,0.1332,"Plate control","PASS","PFC",1.09672361622639,"24h","24h"
6223,"PFC_04",37,"OID30280","P07942","LAMB1",0,"Cardiometabolic_II","B13805","20212097_SS211647","PASS",-4.4422,-0.0872,"Plate control","PASS","PFC",0.941347956958861,"Baseline","Baseline"
6224,"PFC_12",38,"OID30280","P07942","LAMB1",0,"Cardiometabolic_II","B13805","20212097_SS211647","PASS",-4.4422,-0.4642,"Plate control","PASS","PFC",0.7248729207495,"1h","1h"
6225,"PFC_20",39,"OID30280","P07942","LAMB1",0,"Cardiometabolic_II","B13805","20212097_SS211647","PASS",-4.4422,0.4054,"Plate control","PASS","PFC",1.32445607669112,"24h","24h"
6226,"PFC_05",49,"OID30280","P07942","LAMB1",0,"Cardiometabolic_II","B13805","20212097_SS211647","PASS",-4.4422,-0.15,"Plate control","PASS","PFC",0.90125046261083,"Baseline","Baseline"
6227,"PFC_13",50,"OID30280","P07942","LAMB1",0,"Cardiometabolic_II","B13805","20212097_SS211647","PASS",-4.4422,-0.1484,"Plate control","PASS","PFC",0.902250535814082,"1h","1h"
6228,"PFC_21",51,"OID30280","P07942","LAMB1",0,"Cardiometabolic_II","B13805","20212097_SS211647","PASS",-4.4422,-0.3808,"Plate control","PASS","PFC",0.768011596486069,"24h","24h"
6229,"PFC_06",61,"OID30280","P07942","LAMB1",0,"Cardiometabolic_II","B13805","20212097_SS211647","PASS",-4.4422,0.4284,"Plate control","PASS","PFC",1.34574027523243,"Baseline","Baseline"
6230,"PFC_14",62,"OID30280","P07942","LAMB1",0,"Cardiometabolic_II","B13805","20212097_SS211647","PASS",-4.4422,-0.1075,"Plate control","PASS","PFC",0.928195108644738,"1h","1h"
6231,"PFC_22",63,"OID30280","P07942","LAMB1",0,"Cardiometabolic_II","B13805","20212097_SS211647","PASS",-4.4422,-0.0108,"Plate control","PASS","PFC",0.99254196068119,"24h","24h"
6232,"PFC_07",73,"OID30280","P07942","LAMB1",0,"Cardiometabolic_II","B13805","20212097_SS211647","PASS",-4.4422,0.5399,"Plate control","PASS","PFC",1.45387173911796,"Baseline","Baseline"
6233,"PFC_15",74,"OID30280","P07942","LAMB1",0,"Cardiometabolic_II","B13805","20212097_SS211647","PASS",-4.4422,0.2347,"Plate control","PASS","PFC",1.17666203178618,"1h","1h"
6234,"PFC_23",75,"OID30280","P07942","LAMB1",0,"Cardiometabolic_II","B13805","20212097_SS211647","PASS",-4.4422,-0.1805,"Plate control","PASS","PFC",0.88239712775012,"24h","24h"
6235,"PFC_08",85,"OID30280","P07942","LAMB1",0,"Cardiometabolic_II","B13805","20212097_SS211647","PASS",-4.4422,0.1849,"Plate control","PASS","PFC",1.13673817781891,"Baseline","Baseline"
6236,"PFC_16",86,"OID30280","P07942","LAMB1",0,"Cardiometabolic_II","B13805","20212097_SS211647","PASS",-4.4422,0.2555,"Plate control","PASS","PFC",1.19374939336059,"1h","1h"
6237,"PFC_24",87,"OID30280","P07942","LAMB1",0,"Cardiometabolic_II","B13805","20212097_SS211647","PASS",-4.4422,0.4236,"Plate control","PASS","PFC",1.34127029423498,"24h","24h"
6238,"PFC_01",1,"OID30281","Q8NFL0","B3GNT7",0,"Cardiometabolic_II","B13805","20212097_SS211647","PASS",-4.5288,1.1011,"Plate control","PASS","PFC",2.14518192116275,"Baseline","Baseline"
6239,"PFC_09",2,"OID30281","Q8NFL0","B3GNT7",0,"Cardiometabolic_II","B13805","20212097_SS211647","PASS",-4.5288,0.4587,"Plate control","PASS","PFC",1.37430288760548,"Baseline","Baseline"
6240,"PFC_17",3,"OID30281","Q8NFL0","B3GNT7",0,"Cardiometabolic_II","B13805","20212097_SS211647","PASS",-4.5288,-0.6024,"Plate control","PASS","PFC",0.658657331959377,"1h","1h"
6241,"PFC_25",4,"OID30281","Q8NFL0","B3GNT7",0,"Cardiometabolic_II","B13805","20212097_SS211647","PASS",-4.5288,-0.4288,"Plate control","PASS","PFC",0.742879438149981,"24h","24h"
6242,"PFC_02",13,"OID30281","Q8NFL0","B3GNT7",0,"Cardiometabolic_II","B13805","20212097_SS211647","PASS",-4.5288,0.8675,"Plate control","PASS","PFC",1.82449854448999,"Baseline","Baseline"
6243,"PFC_10",14,"OID30281","Q8NFL0","B3GNT7",0,"Cardiometabolic_II","B13805","20212097_SS211647","PASS",-4.5288,0.9652,"Plate control","PASS","PFC",1.95233415377606,"1h","1h"
6244,"PFC_18",15,"OID30281","Q8NFL0","B3GNT7",0,"Cardiometabolic_II","B13805","20212097_SS211647","PASS",-4.5288,0.1075,"Plate control","PASS","PFC",1.07735969591577,"1h","1h"
6245,"PFC_26",16,"OID30281","Q8NFL0","B3GNT7",0,"Cardiometabolic_II","B13805","20212097_SS211647","PASS",-4.5288,-0.6028,"Plate control","PASS","PFC",0.658474738684308,"24h","24h"
6246,"PFC_03",25,"OID30281","Q8NFL0","B3GNT7",0,"Cardiometabolic_II","B13805","20212097_SS211647","PASS",-4.5288,0.0811,"Plate control","PASS","PFC",1.0578242838693,"Baseline","Baseline"
6247,"PFC_11",26,"OID30281","Q8NFL0","B3GNT7",0,"Cardiometabolic_II","B13805","20212097_SS211647","PASS",-4.5288,0.833,"Plate control","PASS","PFC",1.78138580121336,"1h","1h"
6248,"PFC_19",27,"OID30281","Q8NFL0","B3GNT7",0,"Cardiometabolic_II","B13805","20212097_SS211647","PASS",-4.5288,1.1436,"Plate control","PASS","PFC",2.20931633211953,"24h","24h"
6249,"PFC_27",28,"OID30281","Q8NFL0","B3GNT7",0,"Cardiometabolic_II","B13805","20212097_SS211647","PASS",-4.5288,0.8268,"Plate control","PASS","PFC",1.77374669975468,"24h","24h"
6250,"PFC_04",37,"OID30281","Q8NFL0","B3GNT7",0,"Cardiometabolic_II","B13805","20212097_SS211647","PASS",-4.5288,0.609,"Plate control","PASS","PFC",1.52520165325628,"Baseline","Baseline"
6251,"PFC_12",38,"OID30281","Q8NFL0","B3GNT7",0,"Cardiometabolic_II","B13805","20212097_SS211647","PASS",-4.5288,0.0674,"Plate control","PASS","PFC",1.04782660605208,"1h","1h"
6252,"PFC_20",39,"OID30281","Q8NFL0","B3GNT7",0,"Cardiometabolic_II","B13805","20212097_SS211647","PASS",-4.5288,0.9663,"Plate control","PASS","PFC",1.95382330181886,"24h","24h"
6253,"PFC_05",49,"OID30281","Q8NFL0","B3GNT7",0,"Cardiometabolic_II","B13805","20212097_SS211647","PASS",-4.5288,1.0284,"Plate control","PASS","PFC",2.03976082939005,"Baseline","Baseline"
6254,"PFC_13",50,"OID30281","Q8NFL0","B3GNT7",0,"Cardiometabolic_II","B13805","20212097_SS211647","PASS",-4.5288,0.7395,"Plate control","PASS","PFC",1.66959710031341,"1h","1h"
6255,"PFC_21",51,"OID30281","Q8NFL0","B3GNT7",0,"Cardiometabolic_II","B13805","20212097_SS211647","PASS",-4.5288,0.264,"Plate control","PASS","PFC",1.20080342748521,"24h","24h"
6256,"PFC_06",61,"OID30281","Q8NFL0","B3GNT7",0,"Cardiometabolic_II","B13805","20212097_SS211647","PASS",-4.5288,-0.2915,"Plate control","PASS","PFC",0.817052110729759,"Baseline","Baseline"
6257,"PFC_14",62,"OID30281","Q8NFL0","B3GNT7",0,"Cardiometabolic_II","B13805","20212097_SS211647","PASS",-4.5288,0.9384,"Plate control","PASS","PFC",1.91640170216759,"1h","1h"
6258,"PFC_22",63,"OID30281","Q8NFL0","B3GNT7",0,"Cardiometabolic_II","B13805","20212097_SS211647","PASS",-4.5288,0.6707,"Plate control","PASS","PFC",1.59184514824595,"24h","24h"
6259,"PFC_07",73,"OID30281","Q8NFL0","B3GNT7",0,"Cardiometabolic_II","B13805","20212097_SS211647","PASS",-4.5288,-0.0422,"Plate control","PASS","PFC",0.971172853064156,"Baseline","Baseline"
6260,"PFC_15",74,"OID30281","Q8NFL0","B3GNT7",0,"Cardiometabolic_II","B13805","20212097_SS211647","PASS",-4.5288,-0.292,"Plate control","PASS","PFC",0.816768991110023,"1h","1h"
6261,"PFC_23",75,"OID30281","Q8NFL0","B3GNT7",0,"Cardiometabolic_II","B13805","20212097_SS211647","PASS",-4.5288,1.0539,"Plate control","PASS","PFC",2.07613462941209,"24h","24h"
6262,"PFC_08",85,"OID30281","Q8NFL0","B3GNT7",0,"Cardiometabolic_II","B13805","20212097_SS211647","PASS",-4.5288,-0.5824,"Plate control","PASS","PFC",0.667851845673418,"Baseline","Baseline"
6263,"PFC_16",86,"OID30281","Q8NFL0","B3GNT7",0,"Cardiometabolic_II","B13805","20212097_SS211647","PASS",-4.5288,-0.459,"Plate control","PASS","PFC",0.727490341817379,"1h","1h"
6264,"PFC_24",87,"OID30281","Q8NFL0","B3GNT7",0,"Cardiometabolic_II","B13805","20212097_SS211647","PASS",-4.5288,0.1268,"Plate control","PASS","PFC",1.09186917004598,"24h","24h"
6265,"PFC_01",1,"OID30282","P01189","POMC",0,"Cardiometabolic_II","B13805","20212097_SS211647","PASS",-2.4418,1.0056,"Plate control","PASS","PFC",2.00777833494254,"Baseline","Baseline"
6266,"PFC_09",2,"OID30282","P01189","POMC",0,"Cardiometabolic_II","B13805","20212097_SS211647","PASS",-2.4418,1.9283,"Plate control","PASS","PFC",3.80606447190164,"Baseline","Baseline"
6267,"PFC_17",3,"OID30282","P01189","POMC",0,"Cardiometabolic_II","B13805","20212097_SS211647","PASS",-2.4418,-0.9802,"Plate control","PASS","PFC",0.506909462450926,"1h","1h"
6268,"PFC_25",4,"OID30282","P01189","POMC",0,"Cardiometabolic_II","B13805","20212097_SS211647","PASS",-2.4418,1.0097,"Plate control","PASS","PFC",2.01349236261167,"24h","24h"
6269,"PFC_02",13,"OID30282","P01189","POMC",0,"Cardiometabolic_II","B13805","20212097_SS211647","PASS",-2.4418,0.7207,"Plate control","PASS","PFC",1.64798144616611,"Baseline","Baseline"
6270,"PFC_10",14,"OID30282","P01189","POMC",0,"Cardiometabolic_II","B13805","20212097_SS211647","PASS",-2.4418,0.6252,"Plate control","PASS","PFC",1.54242463604491,"1h","1h"
6271,"PFC_18",15,"OID30282","P01189","POMC",0,"Cardiometabolic_II","B13805","20212097_SS211647","PASS",-2.4418,1.0135,"Plate control","PASS","PFC",2.01880281019888,"1h","1h"
6272,"PFC_26",16,"OID30282","P01189","POMC",0,"Cardiometabolic_II","B13805","20212097_SS211647","PASS",-2.4418,-0.6594,"Plate control","PASS","PFC",0.633141558409335,"24h","24h"

Supplemental table 1_Olink proximity extension assay (PEA) data

,"SampleID","Index","OlinkID","UniProt","Assay","MissingFreq","Panel","Panel_Lot_Nr","PlateID","QC_Warning","LOD","NPX","Normalization","Assay_Warning","condition","Value","group","alt_group"
6273,"PFC_03",25,"OID30282","P01189","POMC",0,"Cardiometabolic_II","B13805","20212097_SS211647","PASS",-2.4418,1.5322,"Plate control","PASS","PFC",2.89226551443818,"Baseline","Baseline"
6274,"PFC_11",26,"OID30282","P01189","POMC",0,"Cardiometabolic_II","B13805","20212097_SS211647","PASS",-2.4418,0.6797,"Plate control","PASS","PFC",1.60180663421947,"1h","1h"
6275,"PFC_19",27,"OID30282","P01189","POMC",0,"Cardiometabolic_II","B13805","20212097_SS211647","PASS",-2.4418,1.4493,"Plate control","PASS","PFC",2.73075522131918,"24h","24h"
6276,"PFC_27",28,"OID30282","P01189","POMC",0,"Cardiometabolic_II","B13805","20212097_SS211647","PASS",-2.4418,1.9487,"Plate control","PASS","PFC",3.86026529641889,"24h","24h"
6277,"PFC_04",37,"OID30282","P01189","POMC",0,"Cardiometabolic_II","B13805","20212097_SS211647","PASS",-2.4418,0.2766,"Plate control","PASS","PFC",1.21133676000275,"Baseline","Baseline"
6278,"PFC_12",38,"OID30282","P01189","POMC",0,"Cardiometabolic_II","B13805","20212097_SS211647","PASS",-2.4418,1.5234,"Plate control","PASS","PFC",2.8746772723851,"1h","1h"
6279,"PFC_20",39,"OID30282","P01189","POMC",0,"Cardiometabolic_II","B13805","20212097_SS211647","PASS",-2.4418,0.514,"Plate control","PASS","PFC",1.42800397968506,"24h","24h"
6280,"PFC_05",49,"OID30282","P01189","POMC",0,"Cardiometabolic_II","B13805","20212097_SS211647","PASS",-2.4418,2.8171,"Plate control","PASS","PFC",7.04744346764337,"Baseline","Baseline"
6281,"PFC_13",50,"OID30282","P01189","POMC",0,"Cardiometabolic_II","B13805","20212097_SS211647","PASS",-2.4418,-0.3154,"Plate control","PASS","PFC",0.803628146818177,"1h","1h"
6282,"PFC_21",51,"OID30282","P01189","POMC",0,"Cardiometabolic_II","B13805","20212097_SS211647","PASS",-2.4418,2.3662,"Plate control","PASS","PFC",5.1558132198792,"24h","24h"
6283,"PFC_06",61,"OID30282","P01189","POMC",0,"Cardiometabolic_II","B13805","20212097_SS211647","PASS",-2.4418,0.9339,"Plate control","PASS","PFC",1.91043344701966,"Baseline","Baseline"
6284,"PFC_14",62,"OID30282","P01189","POMC",0,"Cardiometabolic_II","B13805","20212097_SS211647","PASS",-2.4418,2.777,"Plate control","PASS","PFC",6.85425563814856,"1h","1h"
6285,"PFC_22",63,"OID30282","P01189","POMC",0,"Cardiometabolic_II","B13805","20212097_SS211647","PASS",-2.4418,0.163,"Plate control","PASS","PFC",1.11961288865424,"24h","24h"
6286,"PFC_07",73,"OID30282","P01189","POMC",0,"Cardiometabolic_II","B13805","20212097_SS211647","PASS",-2.4418,0.1399,"Plate control","PASS","PFC",1.10182874028119,"Baseline","Baseline"
6287,"PFC_15",74,"OID30282","P01189","POMC",0,"Cardiometabolic_II","B13805","20212097_SS211647","PASS",-2.4418,-0.8166,"Plate control","PASS","PFC",0.567778450864739,"1h","1h"
6288,"PFC_23",75,"OID30282","P01189","POMC",0,"Cardiometabolic_II","B13805","20212097_SS211647","PASS",-2.4418,4.0774,"Plate control","PASS","PFC",16.8818370633798,"24h","24h"
6289,"PFC_08",85,"OID30282","P01189","POMC",0,"Cardiometabolic_II","B13805","20212097_SS211647","PASS",-2.4418,-0.429,"Plate control","PASS","PFC",0.742776460330414,"Baseline","Baseline"
6290,"PFC_16",86,"OID30282","P01189","POMC",0,"Cardiometabolic_II","B13805","20212097_SS211647","PASS",-2.4418,0.6954,"Plate control","PASS","PFC",1.61933334539658,"1h","1h"
6291,"PFC_24",87,"OID30282","P01189","POMC",0,"Cardiometabolic_II","B13805","20212097_SS211647","PASS",-2.4418,0.8401,"Plate control","PASS","PFC",1.79017422297711,"24h","24h"
6292,"PFC_01",1,"OID30283","Q7L266","ASRGL1",0,"Cardiometabolic_II","B13805","20212097_SS211647","PASS",-3.7636,2.4041,"Plate control","PASS","PFC",5.2930526333684,"Baseline","Baseline"
6293,"PFC_09",2,"OID30283","Q7L266","ASRGL1",0,"Cardiometabolic_II","B13805","20212097_SS211647","PASS",-3.7636,2.7991,"Plate control","PASS","PFC",6.96006123963216,"Baseline","Baseline"
6294,"PFC_17",3,"OID30283","Q7L266","ASRGL1",0,"Cardiometabolic_II","B13805","20212097_SS211647","PASS",-3.7636,4.5131,"Plate control","PASS","PFC",22.8338147560664,"1h","1h"
6295,"PFC_25",4,"OID30283","Q7L266","ASRGL1",0,"Cardiometabolic_II","B13805","20212097_SS211647","PASS",-3.7636,3.3762,"Plate control","PASS","PFC",10.3833494736263,"24h","24h"
6296,"PFC_02",13,"OID30283","Q7L266","ASRGL1",0,"Cardiometabolic_II","B13805","20212097_SS211647","PASS",-3.7636,1.9151,"Plate control","PASS","PFC",3.77139954810962,"Baseline","Baseline"
6297,"PFC_10",14,"OID30283","Q7L266","ASRGL1",0,"Cardiometabolic_II","B13805","20212097_SS211647","PASS",-3.7636,2.5231,"Plate control","PASS","PFC",5.74815912439702,"1h","1h"
6298,"PFC_18",15,"OID30283","Q7L266","ASRGL1",0,"Cardiometabolic_II","B13805","20212097_SS211647","PASS",-3.7636,2.9393,"Plate control","PASS","PFC",7.67039035495301,"1h","1h"
6299,"PFC_26",16,"OID30283","Q7L266","ASRGL1",0,"Cardiometabolic_II","B13805","20212097_SS211647","PASS",-3.7636,4.4867,"Plate control","PASS","PFC",22.4197766390443,"24h","24h"
6300,"PFC_03",25,"OID30283","Q7L266","ASRGL1",0,"Cardiometabolic_II","B13805","20212097_SS211647","PASS",-3.7636,0.5759,"Plate control","PASS","PFC",1.49060706220976,"Baseline","Baseline"
6301,"PFC_11",26,"OID30283","Q7L266","ASRGL1",0,"Cardiometabolic_II","B13805","20212097_SS211647","PASS",-3.7636,3.3685,"Plate control","PASS","PFC",10.3280787427469,"1h","1h"
6302,"PFC_19",27,"OID30283","Q7L266","ASRGL1",0,"Cardiometabolic_II","B13805","20212097_SS211647","PASS",-3.7636,3.5116,"Plate control","PASS","PFC",11.4050431511039,"24h","24h"
6303,"PFC_27",28,"OID30283","Q7L266","ASRGL1",0,"Cardiometabolic_II","B13805","20212097_SS211647","PASS",-3.7636,4.3901,"Plate control","PASS","PFC",20.9677477848909,"24h","24h"
6304,"PFC_04",37,"OID30283","Q7L266","ASRGL1",0,"Cardiometabolic_II","B13805","20212097_SS211647","PASS",-3.7636,3.995,"Plate control","PASS","PFC",15.9446442052459,"Baseline","Baseline"
6305,"PFC_12",38,"OID30283","Q7L266","ASRGL1",0,"Cardiometabolic_II","B13805","20212097_SS211647","PASS",-3.7636,0.9928,"Plate control","PASS","PFC",1.99004354590223,"1h","1h"
6306,"PFC_20",39,"OID30283","Q7L266","ASRGL1",0,"Cardiometabolic_II","B13805","20212097_SS211647","PASS",-3.7636,3.7811,"Plate control","PASS","PFC",13.7475249519465,"24h","24h"
6307,"PFC_05",49,"OID30283","Q7L266","ASRGL1",0,"Cardiometabolic_II","B13805","20212097_SS211647","PASS",-3.7636,1.9462,"Plate control","PASS","PFC",3.85358175891925,"Baseline","Baseline"
6308,"PFC_13",50,"OID30283","Q7L266","ASRGL1",0,"Cardiometabolic_II","B13805","20212097_SS211647","PASS",-3.7636,4.0608,"Plate control","PASS","PFC",16.6887037905116,"1h","1h"
6309,"PFC_21",51,"OID30283","Q7L266","ASRGL1",0,"Cardiometabolic_II","B13805","20212097_SS211647","PASS",-3.7636,1.371,"Plate control","PASS","PFC",2.58649786377497,"24h","24h"
6310,"PFC_06",61,"OID30283","Q7L266","ASRGL1",0,"Cardiometabolic_II","B13805","20212097_SS211647","PASS",-3.7636,3.3566,"Plate control","PASS","PFC",10.2432384681446,"Baseline","Baseline"
6311,"PFC_14",62,"OID30283","Q7L266","ASRGL1",0,"Cardiometabolic_II","B13805","20212097_SS211647","PASS",-3.7636,3.2767,"Plate control","PASS","PFC",9.69136581103005,"1h","1h"
6312,"PFC_22",63,"OID30283","Q7L266","ASRGL1",0,"Cardiometabolic_II","B13805","20212097_SS211647","PASS",-3.7636,3.2884,"Plate control","PASS","PFC",9.77028062284172,"24h","24h"
6313,"PFC_07",73,"OID30283","Q7L266","ASRGL1",0,"Cardiometabolic_II","B13805","20212097_SS211647","PASS",-3.7636,4.318,"Plate control","PASS","PFC",19.9456191084016,"Baseline","Baseline"
6314,"PFC_15",74,"OID30283","Q7L266","ASRGL1",0,"Cardiometabolic_II","B13805","20212097_SS211647","PASS",-3.7636,2.1034,"Plate control","PASS","PFC",4.2972091606928,"1h","1h"
6315,"PFC_23",75,"OID30283","Q7L266","ASRGL1",0,"Cardiometabolic_II","B13805","20212097_SS211647","PASS",-3.7636,3.3322,"Plate control","PASS","PFC",10.0714534911844,"24h","24h"
6316,"PFC_08",85,"OID30283","Q7L266","ASRGL1",0,"Cardiometabolic_II","B13805","20212097_SS211647","PASS",-3.7636,3.9043,"Plate control","PASS","PFC",14.9730892079715,"Baseline","Baseline"
6317,"PFC_16",86,"OID30283","Q7L266","ASRGL1",0,"Cardiometabolic_II","B13805","20212097_SS211647","PASS",-3.7636,3.7429,"Plate control","PASS","PFC",13.3882918450968,"1h","1h"
6318,"PFC_24",87,"OID30283","Q7L266","ASRGL1",0,"Cardiometabolic_II","B13805","20212097_SS211647","PASS",-3.7636,3.7184,"Plate control","PASS","PFC",13.1628501104926,"24h","24h"
6319,"PFC_01",1,"OID30284","P50914","RPL14",0,"Cardiometabolic_II","B13805","20212097_SS211647","PASS",-2.0459,-0.2588,"Plate control","PASS","PFC",0.835782814991492,"Baseline","Baseline"
6320,"PFC_09",2,"OID30284","P50914","RPL14",0,"Cardiometabolic_II","B13805","20212097_SS211647","PASS",-2.0459,0.1789,"Plate control","PASS","PFC",1.132020433714,"Baseline","Baseline"
6321,"PFC_17",3,"OID30284","P50914","RPL14",0,"Cardiometabolic_II","B13805","20212097_SS211647","PASS",-2.0459,0.2136,"Plate control","PASS","PFC",1.1595781066134,"1h","1h"
6322,"PFC_25",4,"OID30284","P50914","RPL14",0,"Cardiometabolic_II","B13805","20212097_SS211647","PASS",-2.0459,-0.4262,"Plate control","PASS","PFC",0.744219449709033,"24h","24h"
6323,"PFC_02",13,"OID30284","P50914","RPL14",0,"Cardiometabolic_II","B13805","20212097_SS211647","PASS",-2.0459,0.6671,"Plate control","PASS","PFC",1.58787792135932,"Baseline","Baseline"
6324,"PFC_10",14,"OID30284","P50914","RPL14",0,"Cardiometabolic_II","B13805","20212097_SS211647","PASS",-2.0459,-0.1109,"Plate control","PASS","PFC",0.92601020643923,"1h","1h"
6325,"PFC_18",15,"OID30284","P50914","RPL14",0,"Cardiometabolic_II","B13805","20212097_SS211647","PASS",-2.0459,0.4375,"Plate control","PASS","PFC",1.35425554693689,"1h","1h"
6326,"PFC_26",16,"OID30284","P50914","RPL14",0,"Cardiometabolic_II","B13805","20212097_SS211647","PASS",-2.0459,-0.0368,"Plate control","PASS","PFC",0.974814759545357,"24h","24h"
6327,"PFC_03",25,"OID30284","P50914","RPL14",0,"Cardiometabolic_II","B13805","20212097_SS211647","PASS",-2.0459,0.1497,"Plate control","PASS","PFC",1.10933876757014,"Baseline","Baseline"
6328,"PFC_11",26,"OID30284","P50914","RPL14",0,"Cardiometabolic_II","B13805","20212097_SS211647","PASS",-2.0459,0.4054,"Plate control","PASS","PFC",1.32445607669112,"1h","1h"
6329,"PFC_19",27,"OID30284","P50914","RPL14",0,"Cardiometabolic_II","B13805","20212097_SS211647","PASS",-2.0459,-0.1191,"Plate control","PASS","PFC",0.920761872643476,"24h","24h"
6330,"PFC_27",28,"OID30284","P50914","RPL14",0,"Cardiometabolic_II","B13805","20212097_SS211647","PASS",-2.0459,0.4282,"Plate control","PASS","PFC",1.34555372894762,"24h","24h"
6331,"PFC_04",37,"OID30284","P50914","RPL14",0,"Cardiometabolic_II","B13805","20212097_SS211647","PASS",-2.0459,-0.256,"Plate control","PASS","PFC",0.837406487508427,"Baseline","Baseline"
6332,"PFC_12",38,"OID30284","P50914","RPL14",0,"Cardiometabolic_II","B13805","20212097_SS211647","PASS",-2.0459,0.3959,"Plate control","PASS","PFC",1.31576332010456,"1h","1h"
6333,"PFC_20",39,"OID30284","P50914","RPL14",0,"Cardiometabolic_II","B13805","20212097_SS211647","PASS",-2.0459,0.1562,"Plate control","PASS","PFC",1.11434812156371,"24h","24h"
6334,"PFC_05",49,"OID30284","P50914","RPL14",0,"Cardiometabolic_II","B13805","20212097_SS211647","PASS",-2.0459,0.0252,"Plate control","PASS","PFC",1.01762075451613,"Baseline","Baseline"
6335,"PFC_13",50,"OID30284","P50914","RPL14",0,"Cardiometabolic_II","B13805","20212097_SS211647","PASS",-2.0459,-0.2356,"Plate control","PASS","PFC",0.849331698553624,"1h","1h"
6336,"PFC_21",51,"OID30284","P50914","RPL14",0,"Cardiometabolic_II","B13805","20212097_SS211647","PASS",-2.0459,0.6095,"Plate control","PASS","PFC",1.5257303394782,"24h","24h"
6337,"PFC_06",61,"OID30284","P50914","RPL14",0,"Cardiometabolic_II","B13805","20212097_SS211647","PASS",-2.0459,-0.3991,"Plate control","PASS","PFC",0.758331207351636,"Baseline","Baseline"
6338,"PFC_14",62,"OID30284","P50914","RPL14",0,"Cardiometabolic_II","B13805","20212097_SS211647","PASS",-2.0459,-0.2019,"Plate control","PASS","PFC",0.869404820551542,"1h","1h"
6339,"PFC_22",63,"OID30284","P50914","RPL14",0,"Cardiometabolic_II","B13805","20212097_SS211647","PASS",-2.0459,0.2545,"Plate control","PASS","PFC",1.19292223603829,"24h","24h"
6340,"PFC_07",73,"OID30284","P50914","RPL14",0,"Cardiometabolic_II","B13805","20212097_SS211647","PASS",-2.0459,0.1363,"Plate control","PASS","PFC",1.09908274164701,"Baseline","Baseline"
6341,"PFC_15",74,"OID30284","P50914","RPL14",0,"Cardiometabolic_II","B13805","20212097_SS211647","PASS",-2.0459,-1.8399,"Plate control","PASS","PFC",0.279341146299816,"1h","1h"
6342,"PFC_23",75,"OID30284","P50914","RPL14",0,"Cardiometabolic_II","B13805","20212097_SS211647","PASS",-2.0459,0.5176,"Plate control","PASS","PFC",1.43157177019731,"24h","24h"
6343,"PFC_08",85,"OID30284","P50914","RPL14",0,"Cardiometabolic_II","B13805","20212097_SS211647","PASS",-2.0459,-0.5683,"Plate control","PASS","PFC",0.67441101276020З,"Baseline","Baseline"
6344,"PFC_16",86,"OID30284","P50914","RPL14",0,"Cardiometabolic_II","B13805","20212097_SS211647","PASS",-2.0459,0.0041,"Plate control","PASS","PFC",1.002845945476,"1h","1h"
6345,"PFC_24",87,"OID30284","P50914","RPL14",0,"Cardiometabolic_II","B13805","20212097_SS211647","PASS",-2.0459,-0.3943,"Plate control","PASS","PFC",0.760858457900016,"24h","24h"
6346,"PFC_01",1,"OID30285","P09543","CNP",0,"Cardiometabolic_II","B13805","20212097_SS211647","PASS",-4.6074,3.0268,"Plate control","PASS","PFC",8.14999966475848,"Baseline","Baseline"
6347,"PFC_09",2,"OID30285","P09543","CNP",0,"Cardiometabolic_II","B13805","20212097_SS211647","PASS",-4.6074,3.0382,"Plate control","PASS","PFC",8.21465507983009,"Baseline","Baseline"
6348,"PFC_17",3,"OID30285","P09543","CNP",0,"Cardiometabolic_II","B13805","20212097_SS211647","PASS",-4.6074,4.3194,"Plate control","PASS","PFC",19.9649838522236,"1h","1h"
6349,"PFC_25",4,"OID30285","P09543","CNP",0,"Cardiometabolic_II","B13805","20212097_SS211647","PASS",-4.6074,3.9093,"Plate control","PASS","PFC",15.0250720081119,"24h","24h"
6350,"PFC_02",13,"OID30285","P09543","CNP",0,"Cardiometabolic_II","B13805","20212097_SS211647","PASS",-4.6074,2.8958,"Plate control","PASS","PFC",7.44256543145577,"Baseline","Baseline"
6351,"PFC_10",14,"OID30285","P09543","CNP",0,"Cardiometabolic_II","B13805","20212097_SS211647","PASS",-4.6074,3.0993,"Plate control","PASS","PFC",8.57002848758221,"1h","1h"
6352,"PFC_18",15,"OID30285","P09543","CNP",0,"Cardiometabolic_II","B13805","20212097_SS211647","PASS",-4.6074,3.4268,"Plate control","PASS","PFC",10.7539890304453,"1h","1h"
6353,"PFC_26",16,"OID30285","P09543","CNP",0,"Cardiometabolic_II","B13805","20212097_SS211647","PASS",-4.6074,4.1167,"Plate control","PASS","PFC",17.3480307448122,"24h","24h"
6354,"PFC_03",25,"OID30285","P09543","CNP",0,"Cardiometabolic_II","B13805","20212097_SS211647","PASS",-4.6074,1.6603,"Plate control","PASS","PFC",3.16082245367165,"Baseline","Baseline"
6355,"PFC_11",26,"OID30285","P09543","CNP",0,"Cardiometabolic_II","B13805","20212097_SS211647","PASS",-4.6074,3.8723,"Plate control","PASS","PFC",14.6446316389855,"1h","1h"
6356,"PFC_19",27,"OID30285","P09543","CNP",0,"Cardiometabolic_II","B13805","20212097_SS211647","PASS",-4.6074,3.79,"Plate control","PASS","PFC",13.8325957009258,"24h","24h"
6357,"PFC_27",28,"OID30285","P09543","CNP",0,"Cardiometabolic_II","B13805","20212097_SS211647","PASS",-4.6074,4.8355,"Plate control","PASS","PFC",28.5516061395759,"24h","24h"
6358,"PFC_04",37,"OID30285","P09543","CNP",0,"Cardiometabolic_II","B13805","20212097_SS211647","PASS",-4.6074,4.0332,"Plate control","PASS","PFC",16.3724690645883,"Baseline","Baseline"
6359,"PFC_12",38,"OID30285","P09543","CNP",0,"Cardiometabolic_II","B13805","20212097_SS211647","PASS",-4.6074,2.5386,"Plate control","PASS","PFC",5.81024903184679,"1h","1h"
6360,"PFC_20",39,"OID30285","P09543","CNP",0,"Cardiometabolic_II","B13805","20212097_SS211647","PASS",-4.6074,4.3681,"Plate control","PASS","PFC",20.6504311764365,"24h","24h"
6361,"PFC_05",49,"OID30285","P09543","CNP",0,"Cardiometabolic_II","B13805","20212097_SS211647","PASS",-4.6074,2.6808,"Plate control","PASS","PFC",6.41211366587613,"Baseline","Baseline"
6362,"PFC_13",50,"OID30285","P09543","CNP",0,"Cardiometabolic_II","B13805","20212097_SS211647","PASS",-4.6074,4.321,"Plate control","PASS","PFC",19.987138010454,"1h","1h"
6363,"PFC_21",51,"OID30285","P09543","CNP",0,"Cardiometabolic_II","B13805","20212097_SS211647","PASS",-4.6074,3.1235,"Plate control","PASS","PFC",8.71499598680447,"24h","24h"
6364,"PFC_06",61,"OID30285","P09543","CNP",0,"Cardiometabolic_II","B13805","20212097_SS211647","PASS",-4.6074,4.0135,"Plate control","PASS","PFC",16.150422481591,"Baseline","Baseline"
6365,"PFC_14",62,"OID30285","P09543","CNP",0,"Cardiometabolic_II","B13805","20212097_SS211647","PASS",-4.6074,3.7968,"Plate control","PASS","PFC",13.8979481641405,"1h","1h"
6366,"PFC_22",63,"OID30285","P09543","CNP",0,"Cardiometabolic_II","B13805","20212097_SS211647","PASS",-4.6074,3.8546,"Plate control","PASS","PFC",14.4660586418005,"24h","24h"
6367,"PFC_07",73,"OID30285","P09543","CNP",0,"Cardiometabolic_II","B13805","20212097_SS211647","PASS",-4.6074,4.4708,"Plate control","PASS","PFC",22.1740439821115,"Baseline","Baseline"
6368,"PFC_15",74,"OID30285","P09543","CNP",0,"Cardiometabolic_II","B13805","20212097_SS211647","PASS",-4.6074,3.0266,"Plate control","PASS","PFC",8.14886991321087,"1h","1h"
6369,"PFC_23",75,"OID30285","P09543","CNP",0,"Cardiometabolic_II","B13805","20212097_SS211647","PASS",-4.6074,3.9558,"Plate control","PASS","PFC",15.5172392702247,"24h","24h"
6370,"PFC_08",85,"OID30285","P09543","CNP",0,"Cardiometabolic_II","B13805","20212097_SS211647","PASS",-4.6074,3.5913,"Plate control","PASS","PFC",12.0528297879973,"Baseline","Baseline"
6371,"PFC_16",86,"OID30285","P09543","CNP",0,"Cardiometabolic_II","B13805","20212097_SS211647","PASS",-4.6074,4.348,"Plate control","PASS","PFC",20.3647189644151,"1h","1h"
6372,"PFC_24",87,"OID30285","P09543","CNP",0,"Cardiometabolic_II","B13805","20212097_SS211647","PASS",-4.6074,4.5077,"Plate control","PASS","PFC",22.7485076583472,"24h","24h"
6373,"PFC_01",1,"OID30286","P02458","COL2A1",0,"Cardiometabolic_II","B13805","20212097_SS211647","PASS",-5.0746,2.9354,"Plate control","PASS","PFC",7.64968318928597,"Baseline","Baseline"
6374,"PFC_09",2,"OID30286","P02458","COL2A1",0,"Cardiometabolic_II","B13805","20212097_SS211647","PASS",-5.0746,1.1405,"Plate control","PASS","PFC",2.20457414654624,"Baseline","Baseline"
6375,"PFC_17",3,"OID30286","P02458","COL2A1",0,"Cardiometabolic_II","B13805","20212097_SS211647","PASS",-5.0746,1.1106,"Plate control","PASS","PFC",2.15935433651256,"1h","1h"
6376,"PFC_25",4,"OID30286","P02458","COL2A1",0,"Cardiometabolic_II","B13805","20212097_SS211647","PASS",-5.0746,2.3025,"Plate control","PASS","PFC",4.93311869428013,"24h","24h"
6377,"PFC_02",13,"OID30286","P02458","COL2A1",0,"Cardiometabolic_II","B13805","20212097_SS211647","PASS",-5.0746,0.5257,"Plate control","PASS","PFC",1.43963192434431,"Baseline","Baseline"
6378,"PFC_10",14,"OID30286","P02458","COL2A1",0,"Cardiometabolic_II","B13805","20212097_SS211647","PASS",-5.0746,1.4729,"Plate control","PASS","PFC",2.77579302805172,"1h","1h"
6379,"PFC_18",15,"OID30286","P02458","COL2A1",0,"Cardiometabolic_II","B13805","20212097_SS211647","PASS",-5.0746,0.7325,"Plate control","PASS","PFC",1.66151578589951,"1h","1h"
6380,"PFC_26",16,"OID30286","P02458","COL2A1",0,"Cardiometabolic_II","B13805","20212097_SS211647","PASS",-5.0746,0.8919,"Plate control","PASS","PFC",1.85561832682363,"24h","24h"
6381,"PFC_03",25,"OID30286","P02458","COL2A1",0,"Cardiometabolic_II","B13805","20212097_SS211647","PASS",-5.0746,0.4096,"Plate control","PASS","PFC",1.32831747523112,"Baseline","Baseline"
6382,"PFC_11",26,"OID30286","P02458","COL2A1",0,"Cardiometabolic_II","B13805","20212097_SS211647","PASS",-5.0746,0.9281,"Plate control","PASS","PFC",1.90276843795055,"1h","1h"
6383,"PFC_19",27,"OID30286","P02458","COL2A1",0,"Cardiometabolic_II","B13805","20212097_SS211647","PASS",-5.0746,2.6121,"Plate control","PASS","PFC",6.11392985369775,"24h","24h"
6384,"PFC_27",28,"OID30286","P02458","COL2A1",0,"Cardiometabolic_II","B13805","20212097_SS211647","PASS",-5.0746,1.6855,"Plate control","PASS","PFC",3.21651853019688,"24h","24h"

Supplemental table 1_Olink proximity extension assay (PEA) data

,"SampleID","Index","OlinkID","UniProt","Assay","MissingFreq","Panel","Panel_Lot_Nr","PlateID","QC_Warning","LOD","NPX","Normalization","Assay_Warning","condition","Value","group","alt_group"
6385,"PFC_04",37,"OID30286","P02458","COL2A1",0,"Cardiometabolic_II","B13805","20212097_SS211647","PASS",-5.0746,2.043,"Plate control","PASS","PFC",4.1210158144803,"Baseline","Baseline"
6386,"PFC_12",38,"OID30286","P02458","COL2A1",0,"Cardiometabolic_II","B13805","20212097_SS211647","PASS",-5.0746,0.2681,"Plate control","PASS","PFC",1.20422084856722,"1h","1h"
6387,"PFC_20",39,"OID30286","P02458","COL2A1",0,"Cardiometabolic_II","B13805","20212097_SS211647","PASS",-5.0746,1.0714,"Plate control","PASS","PFC",2.10147165891846,"24h","24h"
6388,"PFC_05",49,"OID30286","P02458","COL2A1",0,"Cardiometabolic_II","B13805","20212097_SS211647","PASS",-5.0746,0.5129,"Plate control","PASS","PFC",1.42691559603774,"Baseline","Baseline"
6389,"PFC_13",50,"OID30286","P02458","COL2A1",0,"Cardiometabolic_II","B13805","20212097_SS211647","PASS",-5.0746,1.6896,"Plate control","PASS","PFC",3.22567256655635,"1h","1h"
6390,"PFC_21",51,"OID30286","P02458","COL2A1",0,"Cardiometabolic_II","B13805","20212097_SS211647","PASS",-5.0746,0.8026,"Plate control","PASS","PFC",1.74424173799606,"24h","24h"
6391,"PFC_06",61,"OID30286","P02458","COL2A1",0,"Cardiometabolic_II","B13805","20212097_SS211647","PASS",-5.0746,3.3437,"Plate control","PASS","PFC",10.1520558079938,"Baseline","Baseline"
6392,"PFC_14",62,"OID30286","P02458","COL2A1",0,"Cardiometabolic_II","B13805","20212097_SS211647","PASS",-5.0746,1.4263,"Plate control","PASS","PFC",2.68756565690771,"1h","1h"
6393,"PFC_22",63,"OID30286","P02458","COL2A1",0,"Cardiometabolic_II","B13805","20212097_SS211647","PASS",-5.0746,1.5349,"Plate control","PASS","PFC",2.89768345003724,"24h","24h"
6394,"PFC_07",73,"OID30286","P02458","COL2A1",0,"Cardiometabolic_II","B13805","20212097_SS211647","PASS",-5.0746,2.5258,"Plate control","PASS","PFC",5.75892686193483,"Baseline","Baseline"
6395,"PFC_15",74,"OID30286","P02458","COL2A1",0,"Cardiometabolic_II","B13805","20212097_SS211647","PASS",-5.0746,1.6555,"Plate control","PASS","PFC",3.15032353603929,"1h","1h"
6396,"PFC_23",75,"OID30286","P02458","COL2A1",0,"Cardiometabolic_II","B13805","20212097_SS211647","PASS",-5.0746,1.3262,"Plate control","PASS","PFC",2.50741361812459,"24h","24h"
6397,"PFC_08",85,"OID30286","P02458","COL2A1",0,"Cardiometabolic_II","B13805","20212097_SS211647","PASS",-5.0746,0.4162,"Plate control","PASS","PFC",1.33440814510465,"Baseline","Baseline"
6398,"PFC_16",86,"OID30286","P02458","COL2A1",0,"Cardiometabolic_II","B13805","20212097_SS211647","PASS",-5.0746,1.9734,"Plate control","PASS","PFC",3.92692487925496,"1h","1h"
6399,"PFC_24",87,"OID30286","P02458","COL2A1",0,"Cardiometabolic_II","B13805","20212097_SS211647","PASS",-5.0746,2.4408,"Plate control","PASS","PFC",5.42942718891631,"24h","24h"
6400,"PFC_01",1,"OID30287","Q24JP5","TMEM132A",0.3864,"Cardiometabolic_II","B13805","20212097_SS211647","PASS",-1.5155,-1.1287,"Plate control","PASS","PFC",0.457327633470956,"Baseline","Baseline"
6401,"PFC_09",2,"OID30287","Q24JP5","TMEM132A",0.3864,"Cardiometabolic_II","B13805","20212097_SS211647","PASS",-1.5155,-0.8614,"Plate control","PASS","PFC",0.550418169570074,"Baseline","Baseline"
6402,"PFC_17",3,"OID30287","Q24JP5","TMEM132A",0.3864,"Cardiometabolic_II","B13805","20212097_SS211647","PASS",-1.5155,-1.1825,"Plate control","PASS","PFC",0.440587356548347,"1h","1h"
6403,"PFC_25",4,"OID30287","Q24JP5","TMEM132A",0.3864,"Cardiometabolic_II","B13805","20212097_SS211647","PASS",-1.5155,-0.9253,"Plate control","PASS","PFC",0.52657100922837,"24h","24h"
6404,"PFC_02",13,"OID30287","Q24JP5","TMEM132A",0.3864,"Cardiometabolic_II","B13805","20212097_SS211647","PASS",-1.5155,-0.8789,"Plate control","PASS","PFC",0.54378188620929,"Baseline","Baseline"
6405,"PFC_10",14,"OID30287","Q24JP5","TMEM132A",0.3864,"Cardiometabolic_II","B13805","20212097_SS211647","PASS",-1.5155,-1.3176,"Plate control","PASS","PFC",0.401201804516834,"1h","1h"
6406,"PFC_18",15,"OID30287","Q24JP5","TMEM132A",0.3864,"Cardiometabolic_II","B13805","20212097_SS211647","PASS",-1.5155,-1.3413,"Plate control","PASS","PFC",0.394664866565022,"1h","1h"
6407,"PFC_26",16,"OID30287","Q24JP5","TMEM132A",0.3864,"Cardiometabolic_II","B13805","20212097_SS211647","PASS",-1.5155,-1.0291,"Plate control","PASS","PFC",0.490015741190817,"24h","24h"
6408,"PFC_03",25,"OID30287","Q24JP5","TMEM132A",0.3864,"Cardiometabolic_II","B13805","20212097_SS211647","PASS",-1.5155,-0.9653,"Plate control","PASS","PFC",0.512171897290309,"Baseline","Baseline"
6409,"PFC_11",26,"OID30287","Q24JP5","TMEM132A",0.3864,"Cardiometabolic_II","B13805","20212097_SS211647","PASS",-1.5155,-1.307,"Plate control","PASS","PFC",0.404160434402784,"1h","1h"
6410,"PFC_19",27,"OID30287","Q24JP5","TMEM132A",0.3864,"Cardiometabolic_II","B13805","20212097_SS211647","PASS",-1.5155,-0.9918,"Plate control","PASS","PFC",0.502849995178822,"24h","24h"
6411,"PFC_27",28,"OID30287","Q24JP5","TMEM132A",0.3864,"Cardiometabolic_II","B13805","20212097_SS211647","PASS",-1.5155,-1.0785,"Plate control","PASS","PFC",0.473520897020927,"24h","24h"
6412,"PFC_04",37,"OID30287","Q24JP5","TMEM132A",0.3864,"Cardiometabolic_II","B13805","20212097_SS211647","PASS",-1.5155,-2.2508,"Plate control","PASS","PFC",0.210107563132477,"Baseline","Baseline"
6413,"PFC_12",38,"OID30287","Q24JP5","TMEM132A",0.3864,"Cardiometabolic_II","B13805","20212097_SS211647","PASS",-1.5155,-2.9013,"Plate control","PASS","PFC",0.133851016468417,"1h","1h"
6414,"PFC_20",39,"OID30287","Q24JP5","TMEM132A",0.3864,"Cardiometabolic_II","B13805","20212097_SS211647","PASS",-1.5155,-1.6248,"Plate control","PASS","PFC",0.324254836812296,"24h","24h"
6415,"PFC_05",49,"OID30287","Q24JP5","TMEM132A",0.3864,"Cardiometabolic_II","B13805","20212097_SS211647","PASS",-1.5155,-1.2669,"Plate control","PASS","PFC",0.415551733649837,"Baseline","Baseline"
6416,"PFC_13",50,"OID30287","Q24JP5","TMEM132A",0.3864,"Cardiometabolic_II","B13805","20212097_SS211647","PASS",-1.5155,-2.5737,"Plate control","PASS","PFC",0.167972853556732,"1h","1h"
6417,"PFC_21",51,"OID30287","Q24JP5","TMEM132A",0.3864,"Cardiometabolic_II","B13805","20212097_SS211647","PASS",-1.5155,-1.757,"Plate control","PASS","PFC",0.295862755438143,"24h","24h"
6418,"PFC_06",61,"OID30287","Q24JP5","TMEM132A",0.3864,"Cardiometabolic_II","B13805","20212097_SS211647","PASS",-1.5155,-0.2029,"Plate control","PASS","PFC",0.868802403857248,"Baseline","Baseline"
6419,"PFC_14",62,"OID30287","Q24JP5","TMEM132A",0.3864,"Cardiometabolic_II","B13805","20212097_SS211647","PASS",-1.5155,-2.9799,"Plate control","PASS","PFC",0.126753720567668,"1h","1h"
6420,"PFC_22",63,"OID30287","Q24JP5","TMEM132A",0.3864,"Cardiometabolic_II","B13805","20212097_SS211647","PASS",-1.5155,-0.9708,"Plate control","PASS","PFC",0.510223056656327,"24h","24h"
6421,"PFC_07",73,"OID30287","Q24JP5","TMEM132A",0.3864,"Cardiometabolic_II","B13805","20212097_SS211647","PASS",-1.5155,-0.8431,"Plate control","PASS","PFC",0.557444468918493,"Baseline","Baseline"
6422,"PFC_15",74,"OID30287","Q24JP5","TMEM132A",0.3864,"Cardiometabolic_II","B13805","20212097_SS211647","PASS",-1.5155,-0.8255,"Plate control","PASS","PFC",0.564286601676511,"1h","1h"
6423,"PFC_23",75,"OID30287","Q24JP5","TMEM132A",0.3864,"Cardiometabolic_II","B13805","20212097_SS211647","PASS",-1.5155,-2.151,"Plate control","PASS","PFC",0.225156494961982,"24h","24h"
6424,"PFC_08",85,"OID30287","Q24JP5","TMEM132A",0.3864,"Cardiometabolic_II","B13805","20212097_SS211647","PASS",-1.5155,-0.6937,"Plate control","PASS","PFC",0.618266181932502,"Baseline","Baseline"
6425,"PFC_16",86,"OID30287","Q24JP5","TMEM132A",0.3864,"Cardiometabolic_II","B13805","20212097_SS211647","PASS",-1.5155,-0.7839,"Plate control","PASS","PFC",0.580794622183156,"1h","1h"
6426,"PFC_24",87,"OID30287","Q24JP5","TMEM132A",0.3864,"Cardiometabolic_II","B13805","20212097_SS211647","PASS",-1.5155,-0.2207,"Plate control","PASS","PFC",0.858148958935882,"24h","24h"
6427,"PFC_01",1,"OID30288","P09681","GIP",0,"Cardiometabolic_II","B13805","20212097_SS211647","PASS",-1.2123,0.4515,"Plate control","PASS","PFC",1.36746129579704,"Baseline","Baseline"
6428,"PFC_09",2,"OID30288","P09681","GIP",0,"Cardiometabolic_II","B13805","20212097_SS211647","PASS",-1.2123,-0.2296,"Plate control","PASS","PFC",0.852871325131576,"Baseline","Baseline"
6429,"PFC_17",3,"OID30288","P09681","GIP",0,"Cardiometabolic_II","B13805","20212097_SS211647","PASS",-1.2123,-0.642,"Plate control","PASS","PFC",0.640823962080097,"1h","1h"
6430,"PFC_25",4,"OID30288","P09681","GIP",0,"Cardiometabolic_II","B13805","20212097_SS211647","PASS",-1.2123,-0.6728,"Plate control","PASS","PFC",0.627288056822724,"24h","24h"
6431,"PFC_02",13,"OID30288","P09681","GIP",0,"Cardiometabolic_II","B13805","20212097_SS211647","PASS",-1.2123,0.9939,"Plate control","PASS","PFC",1.99156145688367,"Baseline","Baseline"
6432,"PFC_10",14,"OID30288","P09681","GIP",0,"Cardiometabolic_II","B13805","20212097_SS211647","PASS",-1.2123,-0.5703,"Plate control","PASS","PFC",0.673476728322386,"1h","1h"
6433,"PFC_18",15,"OID30288","P09681","GIP",0,"Cardiometabolic_II","B13805","20212097_SS211647","PASS",-1.2123,-0.1556,"Plate control","PASS","PFC",0.89775892779182,"1h","1h"
6434,"PFC_26",16,"OID30288","P09681","GIP",0,"Cardiometabolic_II","B13805","20212097_SS211647","PASS",-1.2123,-0.5378,"Plate control","PASS","PFC",0.688820507371476,"24h","24h"
6435,"PFC_03",25,"OID30288","P09681","GIP",0,"Cardiometabolic_II","B13805","20212097_SS211647","PASS",-1.2123,0.1199,"Plate control","PASS","PFC",1.08665953841603,"Baseline","Baseline"
6436,"PFC_11",26,"OID30288","P09681","GIP",0,"Cardiometabolic_II","B13805","20212097_SS211647","PASS",-1.2123,0.5427,"Plate control","PASS","PFC",1.45669617094835,"1h","1h"
6437,"PFC_19",27,"OID30288","P09681","GIP",0,"Cardiometabolic_II","B13805","20212097_SS211647","PASS",-1.2123,0.0468,"Plate control","PASS","PFC",1.03297117754593,"24h","24h"
6438,"PFC_27",28,"OID30288","P09681","GIP",0,"Cardiometabolic_II","B13805","20212097_SS211647","PASS",-1.2123,0.8111,"Plate control","PASS","PFC",1.75454870929995,"24h","24h"
6439,"PFC_04",37,"OID30288","P09681","GIP",0,"Cardiometabolic_II","B13805","20212097_SS211647","PASS",-1.2123,-0.411,"Plate control","PASS","PFC",0.752101875694344,"Baseline","Baseline"
6440,"PFC_12",38,"OID30288","P09681","GIP",0,"Cardiometabolic_II","B13805","20212097_SS211647","PASS",-1.2123,-0.8833,"Plate control","PASS","PFC",0.542125960775381,"1h","1h"
6441,"PFC_20",39,"OID30288","P09681","GIP",0,"Cardiometabolic_II","B13805","20212097_SS211647","PASS",-1.2123,-0.6011,"Plate control","PASS","PFC",0.659251109857643,"24h","24h"
6442,"PFC_05",49,"OID30288","P09681","GIP",0,"Cardiometabolic_II","B13805","20212097_SS211647","PASS",-1.2123,0.1814,"Plate control","PASS","PFC",1.1339837762593,"Baseline","Baseline"
6443,"PFC_13",50,"OID30288","P09681","GIP",0,"Cardiometabolic_II","B13805","20212097_SS211647","PASS",-1.2123,-0.1961,"Plate control","PASS","PFC",0.872907083728363,"1h","1h"
6444,"PFC_21",51,"OID30288","P09681","GIP",0,"Cardiometabolic_II","B13805","20212097_SS211647","PASS",-1.2123,-0.9691,"Plate control","PASS","PFC",0.510824632464611,"24h","24h"
6445,"PFC_06",61,"OID30288","P09681","GIP",0,"Cardiometabolic_II","B13805","20212097_SS211647","PASS",-1.2123,-0.7116,"Plate control","PASS","PFC",0.610642538927607,"Baseline","Baseline"
6446,"PFC_14",62,"OID30288","P09681","GIP",0,"Cardiometabolic_II","B13805","20212097_SS211647","PASS",-1.2123,-0.0553,"Plate control","PASS","PFC",0.962394298024694,"1h","1h"
6447,"PFC_22",63,"OID30288","P09681","GIP",0,"Cardiometabolic_II","B13805","20212097_SS211647","PASS",-1.2123,-0.2757,"Plate control","PASS","PFC",0.82604941922523,"24h","24h"
6448,"PFC_07",73,"OID30288","P09681","GIP",0,"Cardiometabolic_II","B13805","20212097_SS211647","PASS",-1.2123,-0.5442,"Plate control","PASS","PFC",0.685771569579381,"Baseline","Baseline"
6449,"PFC_15",74,"OID30288","P09681","GIP",0,"Cardiometabolic_II","B13805","20212097_SS211647","PASS",-1.2123,-0.5409,"Plate control","PASS","PFC",0.687341989051054,"1h","1h"
6450,"PFC_23",75,"OID30288","P09681","GIP",0,"Cardiometabolic_II","B13805","20212097_SS211647","PASS",-1.2123,0.0107,"Plate control","PASS","PFC",1.00744424648595,"24h","24h"
6451,"PFC_08",85,"OID30288","P09681","GIP",0,"Cardiometabolic_II","B13805","20212097_SS211647","PASS",-1.2123,-0.3637,"Plate control","PASS","PFC",0.7771688597037,"Baseline","Baseline"
6452,"PFC_16",86,"OID30288","P09681","GIP",0,"Cardiometabolic_II","B13805","20212097_SS211647","PASS",-1.2123,-0.3994,"Plate control","PASS","PFC",0.758173533204421,"1h","1h"
6453,"PFC_24",87,"OID30288","P09681","GIP",0,"Cardiometabolic_II","B13805","20212097_SS211647","PASS",-1.2123,-0.0479,"Plate control","PASS","PFC",0.967343378423709,"24h","24h"
6454,"PFC_01",1,"OID30289","Q9UBQ7","GRHPR",0,"Cardiometabolic_II","B13805","20212097_SS211647","PASS",-2.1039,2.5842,"Plate control","PASS","PFC",5.99682968651886,"Baseline","Baseline"
6455,"PFC_09",2,"OID30289","Q9UBQ7","GRHPR",0,"Cardiometabolic_II","B13805","20212097_SS211647","PASS",-2.1039,2.881,"Plate control","PASS","PFC",7.36660557764032,"Baseline","Baseline"
6456,"PFC_17",3,"OID30289","Q9UBQ7","GRHPR",0,"Cardiometabolic_II","B13805","20212097_SS211647","PASS",-2.1039,4.2914,"Plate control","PASS","PFC",19.5812369772313,"1h","1h"
6457,"PFC_25",4,"OID30289","Q9UBQ7","GRHPR",0,"Cardiometabolic_II","B13805","20212097_SS211647","PASS",-2.1039,3.2659,"Plate control","PASS","PFC",9.61908722375842,"24h","24h"
6458,"PFC_02",13,"OID30289","Q9UBQ7","GRHPR",0,"Cardiometabolic_II","B13805","20212097_SS211647","PASS",-2.1039,1.8942,"Plate control","PASS","PFC",3.71715796880006,"Baseline","Baseline"
6459,"PFC_10",14,"OID30289","Q9UBQ7","GRHPR",0,"Cardiometabolic_II","B13805","20212097_SS211647","PASS",-2.1039,2.8476,"Plate control","PASS","PFC",7.19801944690362,"1h","1h"
6460,"PFC_18",15,"OID30289","Q9UBQ7","GRHPR",0,"Cardiometabolic_II","B13805","20212097_SS211647","PASS",-2.1039,3.1894,"Plate control","PASS","PFC",9.12231507374201,"1h","1h"
6461,"PFC_26",16,"OID30289","Q9UBQ7","GRHPR",0,"Cardiometabolic_II","B13805","20212097_SS211647","PASS",-2.1039,4.1112,"Plate control","PASS","PFC",17.2820205880388,"24h","24h"
6462,"PFC_03",25,"OID30289","Q9UBQ7","GRHPR",0,"Cardiometabolic_II","B13805","20212097_SS211647","PASS",-2.1039,1.643,"Plate control","PASS","PFC",3.12314597042956,"Baseline","Baseline"
6463,"PFC_11",26,"OID30289","Q9UBQ7","GRHPR",0,"Cardiometabolic_II","B13805","20212097_SS211647","PASS",-2.1039,3.2149,"Plate control","PASS","PFC",9.28498770645922,"1h","1h"
6464,"PFC_19",27,"OID30289","Q9UBQ7","GRHPR",0,"Cardiometabolic_II","B13805","20212097_SS211647","PASS",-2.1039,3.5239,"Plate control","PASS","PFC",11.5026949290866,"24h","24h"
6465,"PFC_27",28,"OID30289","Q9UBQ7","GRHPR",0,"Cardiometabolic_II","B13805","20212097_SS211647","PASS",-2.1039,4.4283,"Plate control","PASS","PFC",21.5303519817187,"24h","24h"
6466,"PFC_04",37,"OID30289","Q9UBQ7","GRHPR",0,"Cardiometabolic_II","B13805","20212097_SS211647","PASS",-2.1039,3.5452,"Plate control","PASS","PFC",11.6737809714259,"Baseline","Baseline"
6467,"PFC_12",38,"OID30289","Q9UBQ7","GRHPR",0,"Cardiometabolic_II","B13805","20212097_SS211647","PASS",-2.1039,2.0248,"Plate control","PASS","PFC",4.06935459697315,"1h","1h"
6468,"PFC_20",39,"OID30289","Q9UBQ7","GRHPR",0,"Cardiometabolic_II","B13805","20212097_SS211647","PASS",-2.1039,3.3399,"Plate control","PASS","PFC",10.1253508915956,"24h","24h"
6469,"PFC_05",49,"OID30289","Q9UBQ7","GRHPR",0,"Cardiometabolic_II","B13805","20212097_SS211647","PASS",-2.1039,2.1557,"Plate control","PASS","PFC",4.45584793940264,"Baseline","Baseline"
6470,"PFC_13",50,"OID30289","Q9UBQ7","GRHPR",0,"Cardiometabolic_II","B13805","20212097_SS211647","PASS",-2.1039,3.795,"Plate control","PASS","PFC",13.8806189944331,"1h","1h"
6471,"PFC_21",51,"OID30289","Q9UBQ7","GRHPR",0,"Cardiometabolic_II","B13805","20212097_SS211647","PASS",-2.1039,2.6955,"Plate control","PASS","PFC",6.47778237168406,"24h","24h"
6472,"PFC_06",61,"OID30289","Q9UBQ7","GRHPR",0,"Cardiometabolic_II","B13805","20212097_SS211647","PASS",-2.1039,2.8952,"Plate control","PASS","PFC",7.43947079906412,"Baseline","Baseline"
6473,"PFC_14",62,"OID30289","Q9UBQ7","GRHPR",0,"Cardiometabolic_II","B13805","20212097_SS211647","PASS",-2.1039,3.0776,"Plate control","PASS","PFC",8.44208877257928,"1h","1h"
6474,"PFC_22",63,"OID30289","Q9UBQ7","GRHPR",0,"Cardiometabolic_II","B13805","20212097_SS211647","PASS",-2.1039,3.332,"Plate control","PASS","PFC",10.0700573880389,"24h","24h"
6475,"PFC_07",73,"OID30289","Q9UBQ7","GRHPR",0,"Cardiometabolic_II","B13805","20212097_SS211647","PASS",-2.1039,3.9554,"Plate control","PASS","PFC",15.5129375743339,"Baseline","Baseline"
6476,"PFC_15",74,"OID30289","Q9UBQ7","GRHPR",0,"Cardiometabolic_II","B13805","20212097_SS211647","PASS",-2.1039,2.0063,"Plate control","PASS","PFC",4.01750550288556,"1h","1h"
6477,"PFC_23",75,"OID30289","Q9UBQ7","GRHPR",0,"Cardiometabolic_II","B13805","20212097_SS211647","PASS",-2.1039,3.5731,"Plate control","PASS","PFC",11.901735036294,"24h","24h"
6478,"PFC_08",85,"OID30289","Q9UBQ7","GRHPR",0,"Cardiometabolic_II","B13805","20212097_SS211647","PASS",-2.1039,3.2686,"Plate control","PASS","PFC",9.63710617631997,"Baseline","Baseline"
6479,"PFC_16",86,"OID30289","Q9UBQ7","GRHPR",0,"Cardiometabolic_II","B13805","20212097_SS211647","PASS",-2.1039,3.3779,"Plate control","PASS","PFC",10.3955919071479,"1h","1h"
6480,"PFC_24",87,"OID30289","Q9UBQ7","GRHPR",0,"Cardiometabolic_II","B13805","20212097_SS211647","PASS",-2.1039,3.5286,"Plate control","PASS","PFC",11.5402294202947,"24h","24h"
6481,"PFC_01",1,"OID30290","Q9BVM4","GGACT",0,"Cardiometabolic_II","B13805","20212097_SS211647","PASS",-0.5554,1.318,"Plate control","PASS","PFC",2.49320238855019,"Baseline","Baseline"
6482,"PFC_09",2,"OID30290","Q9BVM4","GGACT",0,"Cardiometabolic_II","B13805","20212097_SS211647","PASS",-0.5554,2.3617,"Plate control","PASS","PFC",5.13975645643731,"Baseline","Baseline"
6483,"PFC_17",3,"OID30290","Q9BVM4","GGACT",0,"Cardiometabolic_II","B13805","20212097_SS211647","PASS",-0.5554,3.0868,"Plate control","PASS","PFC",8.49609560137734,"1h","1h"
6484,"PFC_25",4,"OID30290","Q9BVM4","GGACT",0,"Cardiometabolic_II","B13805","20212097_SS211647","PASS",-0.5554,3.5041,"Plate control","PASS","PFC",11.3459066965042,"24h","24h"
6485,"PFC_02",13,"OID30290","Q9BVM4","GGACT",0,"Cardiometabolic_II","B13805","20212097_SS211647","PASS",-0.5554,1.4171,"Plate control","PASS","PFC",2.67048170386314,"Baseline","Baseline"
6486,"PFC_10",14,"OID30290","Q9BVM4","GGACT",0,"Cardiometabolic_II","B13805","20212097_SS211647","PASS",-0.5554,1.7612,"Plate control","PASS","PFC",3.38979963322488,"1h","1h"
6487,"PFC_18",15,"OID30290","Q9BVM4","GGACT",0,"Cardiometabolic_II","B13805","20212097_SS211647","PASS",-0.5554,2.5171,"Plate control","PASS","PFC",5.72430284477843,"1h","1h"
6488,"PFC_26",16,"OID30290","Q9BVM4","GGACT",0,"Cardiometabolic_II","B13805","20212097_SS211647","PASS",-0.5554,3.66,"Plate control","PASS","PFC",12.640660989814,"24h","24h"
6489,"PFC_03",25,"OID30290","Q9BVM4","GGACT",0,"Cardiometabolic_II","B13805","20212097_SS211647","PASS",-0.5554,1.0817,"Plate control","PASS","PFC",2.11652862423239,"Baseline","Baseline"
6490,"PFC_11",26,"OID30290","Q9BVM4","GGACT",0,"Cardiometabolic_II","B13805","20212097_SS211647","PASS",-0.5554,2.7563,"Plate control","PASS","PFC",6.75661195127707,"1h","1h"
6491,"PFC_19",27,"OID30290","Q9BVM4","GGACT",0,"Cardiometabolic_II","B13805","20212097_SS211647","PASS",-0.5554,2.5942,"Plate control","PASS","PFC",6.03854093558506,"24h","24h"
6492,"PFC_27",28,"OID30290","Q9BVM4","GGACT",0,"Cardiometabolic_II","B13805","20212097_SS211647","PASS",-0.5554,3.8897,"Plate control","PASS","PFC",14.8223264536221,"24h","24h"
6493,"PFC_04",37,"OID30290","Q9BVM4","GGACT",0,"Cardiometabolic_II","B13805","20212097_SS211647","PASS",-0.5554,3.1512,"Plate control","PASS","PFC",8.88394215955875,"Baseline","Baseline"
6494,"PFC_12",38,"OID30290","Q9BVM4","GGACT",0,"Cardiometabolic_II","B13805","20212097_SS211647","PASS",-0.5554,1.6711,"Plate control","PASS","PFC",3.18457312525341,"1h","1h"
6495,"PFC_20",39,"OID30290","Q9BVM4","GGACT",0,"Cardiometabolic_II","B13805","20212097_SS211647","PASS",-0.5554,2.898,"Plate control","PASS","PFC",7.45392343443818,"24h","24h"
6496,"PFC_05",49,"OID30290","Q9BVM4","GGACT",0,"Cardiometabolic_II","B13805","20212097_SS211647","PASS",-0.5554,0.8908,"Plate control","PASS","PFC",1.85420402779413,"Baseline","Baseline"

Supplemental table 1_Olink proximity extension assay (PEA) data

,"SampleID","Index","OlinkID","UniProt","Assay","MissingFreq","Panel","Panel_Lot_Nr","PlateID","QC_Warning","LOD","NPX","Normalization","Assay_Warning","condition","Value","group","alt_group"
6497,"PFC_13",50,"OID30290","Q9BVM4","GGACT",0,"Cardiometabolic_II","B13805","20212097_SS211647","PASS",-0.5554,3.5561,"Plate control","PASS","PFC",11.7623139643329,"1h","1h"
6498,"PFC_21",51,"OID30290","Q9BVM4","GGACT",0,"Cardiometabolic_II","B13805","20212097_SS211647","PASS",-0.5554,2.1272,"Plate control","PASS","PFC",4.36868776972763,"24h","24h"
6499,"PFC_06",61,"OID30290","Q9BVM4","GGACT",0,"Cardiometabolic_II","B13805","20212097_SS211647","PASS",-0.5554,3.476,"Plate control","PASS","PFC",11.1270557736799,"Baseline","Baseline"
6500,"PFC_14",62,"OID30290","Q9BVM4","GGACT",0,"Cardiometabolic_II","B13805","20212097_SS211647","PASS",-0.5554,2.2681,"Plate control","PASS","PFC",4.81688339426888,"1h","1h"
6501,"PFC_22",63,"OID30290","Q9BVM4","GGACT",0,"Cardiometabolic_II","B13805","20212097_SS211647","PASS",-0.5554,2.7304,"Plate control","PASS","PFC",6.6363961111624,"24h","24h"
6502,"PFC_07",73,"OID30290","Q9BVM4","GGACT",0,"Cardiometabolic_II","B13805","20212097_SS211647","PASS",-0.5554,4.0994,"Plate control","PASS","PFC",17.141245074557,"Baseline","Baseline"
6503,"PFC_15",74,"OID30290","Q9BVM4","GGACT",0,"Cardiometabolic_II","B13805","20212097_SS211647","PASS",-0.5554,2.4917,"Plate control","PASS","PFC",5.62440311629357,"1h","1h"
6504,"PFC_23",75,"OID30290","Q9BVM4","GGACT",0,"Cardiometabolic_II","B13805","20212097_SS211647","PASS",-0.5554,2.6235,"Plate control","PASS","PFC",6.16243276028299,"24h","24h"
6505,"PFC_08",85,"OID30290","Q9BVM4","GGACT",0,"Cardiometabolic_II","B13805","20212097_SS211647","PASS",-0.5554,2.8296,"Plate control","PASS","PFC",7.10877020646256,"Baseline","Baseline"
6506,"PFC_16",86,"OID30290","Q9BVM4","GGACT",0,"Cardiometabolic_II","B13805","20212097_SS211647","PASS",-0.5554,3.6108,"Plate control","PASS","PFC",12.2168462517808,"1h","1h"
6507,"PFC_24",87,"OID30290","Q9BVM4","GGACT",0,"Cardiometabolic_II","B13805","20212097_SS211647","PASS",-0.5554,3.7293,"Plate control","PASS","PFC",13.2626760810433,"24h","24h"
6508,"PFC_01",1,"OID30291","P33681","CD80",0,"Cardiometabolic_II","B13805","20212097_SS211647","PASS",-3.5574,-0.3812,"Plate control","PASS","PFC",0.767798687973723,"Baseline","Baseline"
6509,"PFC_09",2,"OID30291","P33681","CD80",0,"Cardiometabolic_II","B13805","20212097_SS211647","PASS",-3.5574,0.3073,"Plate control","PASS","PFC",1.23738975990318,"Baseline","Baseline"
6510,"PFC_17",3,"OID30291","P33681","CD80",0,"Cardiometabolic_II","B13805","20212097_SS211647","PASS",-3.5574,-0.0573,"Plate control","PASS","PFC",0.961061060579464,"1h","1h"
6511,"PFC_25",4,"OID30291","P33681","CD80",0,"Cardiometabolic_II","B13805","20212097_SS211647","PASS",-3.5574,-0.9922,"Plate control","PASS","PFC",0.502710594882138,"24h","24h"
6512,"PFC_02",13,"OID30291","P33681","CD80",0,"Cardiometabolic_II","B13805","20212097_SS211647","PASS",-3.5574,0.2549,"Plate control","PASS","PFC",1.19325303016779,"Baseline","Baseline"
6513,"PFC_10",14,"OID30291","P33681","CD80",0,"Cardiometabolic_II","B13805","20212097_SS211647","PASS",-3.5574,0.1608,"Plate control","PASS","PFC",1.11790686542495,"1h","1h"
6514,"PFC_18",15,"OID30291","P33681","CD80",0,"Cardiometabolic_II","B13805","20212097_SS211647","PASS",-3.5574,-1.0211,"Plate control","PASS","PFC",0.492740513116223,"1h","1h"
6515,"PFC_26",16,"OID30291","P33681","CD80",0,"Cardiometabolic_II","B13805","20212097_SS211647","PASS",-3.5574,0.0909,"Plate control","PASS","PFC",1.06503437827721,"24h","24h"
6516,"PFC_03",25,"OID30291","P33681","CD80",0,"Cardiometabolic_II","B13805","20212097_SS211647","PASS",-3.5574,-0.5007,"Plate control","PASS","PFC",0.706763774057111,"Baseline","Baseline"
6517,"PFC_11",26,"OID30291","P33681","CD80",0,"Cardiometabolic_II","B13805","20212097_SS211647","PASS",-3.5574,-0.0436,"Plate control","PASS","PFC",0.970230878172232,"1h","1h"
6518,"PFC_19",27,"OID30291","P33681","CD80",0,"Cardiometabolic_II","B13805","20212097_SS211647","PASS",-3.5574,-0.0168,"Plate control","PASS","PFC",0.988422666480253,"24h","24h"
6519,"PFC_27",28,"OID30291","P33681","CD80",0,"Cardiometabolic_II","B13805","20212097_SS211647","PASS",-3.5574,-0.036,"Plate control","PASS","PFC",0.975355461527642,"24h","24h"
6520,"PFC_04",37,"OID30291","P33681","CD80",0,"Cardiometabolic_II","B13805","20212097_SS211647","PASS",-3.5574,0.215,"Plate control","PASS","PFC",1.16070391438372,"Baseline","Baseline"
6521,"PFC_12",38,"OID30291","P33681","CD80",0,"Cardiometabolic_II","B13805","20212097_SS211647","PASS",-3.5574,-0.5243,"Plate control","PASS","PFC",0.695296388002077,"1h","1h"
6522,"PFC_20",39,"OID30291","P33681","CD80",0,"Cardiometabolic_II","B13805","20212097_SS211647","PASS",-3.5574,0.1502,"Plate control","PASS","PFC",1.1097233017205,"24h","24h"
6523,"PFC_05",49,"OID30291","P33681","CD80",0,"Cardiometabolic_II","B13805","20212097_SS211647","PASS",-3.5574,-0.0455,"Plate control","PASS","PFC",0.96895394488874,"Baseline","Baseline"
6524,"PFC_13",50,"OID30291","P33681","CD80",0,"Cardiometabolic_II","B13805","20212097_SS211647","PASS",-3.5574,0.0485,"Plate control","PASS","PFC",1.03418909677353,"1h","1h"
6525,"PFC_21",51,"OID30291","P33681","CD80",0,"Cardiometabolic_II","B13805","20212097_SS211647","PASS",-3.5574,-0.2983,"Plate control","PASS","PFC",0.813210078267105,"24h","24h"
6526,"PFC_06",61,"OID30291","P33681","CD80",0,"Cardiometabolic_II","B13805","20212097_SS211647","PASS",-3.5574,-0.3224,"Plate control","PASS","PFC",0.799738363034366,"Baseline","Baseline"
6527,"PFC_14",62,"OID30291","P33681","CD80",0,"Cardiometabolic_II","B13805","20212097_SS211647","PASS",-3.5574,0.3225,"Plate control","PASS","PFC",1.25049561624869,"1h","1h"
6528,"PFC_22",63,"OID30291","P33681","CD80",0,"Cardiometabolic_II","B13805","20212097_SS211647","PASS",-3.5574,0.1638,"Plate control","PASS","PFC",1.12023390603458,"24h","24h"
6529,"PFC_07",73,"OID30291","P33681","CD80",0,"Cardiometabolic_II","B13805","20212097_SS211647","PASS",-3.5574,-0.5353,"Plate control","PASS","PFC",0.690015177156509,"Baseline","Baseline"
6530,"PFC_15",74,"OID30291","P33681","CD80",0,"Cardiometabolic_II","B13805","20212097_SS211647","PASS",-3.5574,-0.5542,"Plate control","PASS","PFC",0.681034599333361,"1h","1h"
6531,"PFC_23",75,"OID30291","P33681","CD80",0,"Cardiometabolic_II","B13805","20212097_SS211647","PASS",-3.5574,-0.1333,"Plate control","PASS","PFC",0.911743554063987,"24h","24h"
6532,"PFC_08",85,"OID30291","P33681","CD80",0,"Cardiometabolic_II","B13805","20212097_SS211647","PASS",-3.5574,-1.2351,"Plate control","PASS","PFC",0.42481305275181,"Baseline","Baseline"
6533,"PFC_16",86,"OID30291","P33681","CD80",0,"Cardiometabolic_II","B13805","20212097_SS211647","PASS",-3.5574,0.0436,"Plate control","PASS","PFC",1.03068251330431,"1h","1h"
6534,"PFC_24",87,"OID30291","P33681","CD80",0,"Cardiometabolic_II","B13805","20212097_SS211647","PASS",-3.5574,0.1832,"Plate control","PASS","PFC",1.13539949102765,"24h","24h"
6535,"PFC_01",1,"OID30292","Q12982","BNIP2",0.1364,"Cardiometabolic_II","B13805","20212097_SS211647","PASS",0.3563,0.7434,"Plate control","PASS","PFC",1.67411658470223,"Baseline","Baseline"
6536,"PFC_09",2,"OID30292","Q12982","BNIP2",0.1364,"Cardiometabolic_II","B13805","20212097_SS211647","PASS",0.3563,1.0611,"Plate control","PASS","PFC",2.08652180871834,"Baseline","Baseline"
6537,"PFC_17",3,"OID30292","Q12982","BNIP2",0.1364,"Cardiometabolic_II","B13805","20212097_SS211647","PASS",0.3563,2.674,"Plate control","PASS","PFC",6.38196191847226,"1h","1h"
6538,"PFC_25",4,"OID30292","Q12982","BNIP2",0.1364,"Cardiometabolic_II","B13805","20212097_SS211647","PASS",0.3563,2.101,"Plate control","PASS","PFC",4.29006646727177,"24h","24h"
6539,"PFC_02",13,"OID30292","Q12982","BNIP2",0.1364,"Cardiometabolic_II","B13805","20212097_SS211647","PASS",0.3563,0.7392,"Plate control","PASS","PFC",1.66924995345141,"Baseline","Baseline"
6540,"PFC_10",14,"OID30292","Q12982","BNIP2",0.1364,"Cardiometabolic_II","B13805","20212097_SS211647","PASS",0.3563,1.1632,"Plate control","PASS","PFC",2.23953622143316,"1h","1h"
6541,"PFC_18",15,"OID30292","Q12982","BNIP2",0.1364,"Cardiometabolic_II","B13805","20212097_SS211647","PASS",0.3563,2.018,"Plate control","PASS","PFC",4.05021922940197,"1h","1h"
6542,"PFC_26",16,"OID30292","Q12982","BNIP2",0.1364,"Cardiometabolic_II","B13805","20212097_SS211647","PASS",0.3563,2.7752,"Plate control","PASS","PFC",6.84570915648317,"24h","24h"
6543,"PFC_03",25,"OID30292","Q12982","BNIP2",0.1364,"Cardiometabolic_II","B13805","20212097_SS211647","PASS",0.3563,0.3566,"Plate control","PASS","PFC",1.28040480851807,"Baseline","Baseline"
6544,"PFC_11",26,"OID30292","Q12982","BNIP2",0.1364,"Cardiometabolic_II","B13805","20212097_SS211647","PASS",0.3563,1.8465,"Plate control","PASS","PFC",3.59626666153792,"1h","1h"
6545,"PFC_19",27,"OID30292","Q12982","BNIP2",0.1364,"Cardiometabolic_II","B13805","20212097_SS211647","PASS",0.3563,1.4907,"Plate control","PASS","PFC",2.81025296397488,"24h","24h"
6546,"PFC_27",28,"OID30292","Q12982","BNIP2",0.1364,"Cardiometabolic_II","B13805","20212097_SS211647","PASS",0.3563,2.9348,"Plate control","PASS","PFC",7.64650243694977,"24h","24h"
6547,"PFC_04",37,"OID30292","Q12982","BNIP2",0.1364,"Cardiometabolic_II","B13805","20212097_SS211647","PASS",0.3563,1.6975,"Plate control","PASS","PFC",3.24338435613798,"Baseline","Baseline"
6548,"PFC_12",38,"OID30292","Q12982","BNIP2",0.1364,"Cardiometabolic_II","B13805","20212097_SS211647","PASS",0.3563,0.8607,"Plate control","PASS","PFC",1.81591918640017,"1h","1h"
6549,"PFC_20",39,"OID30292","Q12982","BNIP2",0.1364,"Cardiometabolic_II","B13805","20212097_SS211647","PASS",0.3563,2.415,"Plate control","PASS","PFC",5.33319470836479,"24h","24h"
6550,"PFC_05",49,"OID30292","Q12982","BNIP2",0.1364,"Cardiometabolic_II","B13805","20212097_SS211647","PASS",0.3563,0.5604,"Plate control","PASS","PFC",1.47467802818225,"Baseline","Baseline"
6551,"PFC_13",50,"OID30292","Q12982","BNIP2",0.1364,"Cardiometabolic_II","B13805","20212097_SS211647","PASS",0.3563,1.9587,"Plate control","PASS","PFC",3.88711556492035,"1h","1h"
6552,"PFC_21",51,"OID30292","Q12982","BNIP2",0.1364,"Cardiometabolic_II","B13805","20212097_SS211647","PASS",0.3563,1.2524,"Plate control","PASS","PFC",2.38237414153659,"24h","24h"
6553,"PFC_06",61,"OID30292","Q12982","BNIP2",0.1364,"Cardiometabolic_II","B13805","20212097_SS211647","PASS",0.3563,1.4686,"Plate control","PASS","PFC",2.76753200291763,"Baseline","Baseline"
6554,"PFC_14",62,"OID30292","Q12982","BNIP2",0.1364,"Cardiometabolic_II","B13805","20212097_SS211647","PASS",0.3563,1.6698,"Plate control","PASS","PFC",3.1817048264991,"1h","1h"
6555,"PFC_22",63,"OID30292","Q12982","BNIP2",0.1364,"Cardiometabolic_II","B13805","20212097_SS211647","PASS",0.3563,1.785,"Plate control","PASS","PFC",3.44618463884807,"24h","24h"
6556,"PFC_07",73,"OID30292","Q12982","BNIP2",0.1364,"Cardiometabolic_II","B13805","20212097_SS211647","PASS",0.3563,2.6241,"Plate control","PASS","PFC",6.16499617702948,"Baseline","Baseline"
6557,"PFC_15",74,"OID30292","Q12982","BNIP2",0.1364,"Cardiometabolic_II","B13805","20212097_SS211647","PASS",0.3563,0.9486,"Plate control","PASS","PFC",1.92999886624571,"1h","1h"
6558,"PFC_23",75,"OID30292","Q12982","BNIP2",0.1364,"Cardiometabolic_II","B13805","20212097_SS211647","PASS",0.3563,2.0758,"Plate control","PASS","PFC",4.21578122127791,"24h","24h"
6559,"PFC_08",85,"OID30292","Q12982","BNIP2",0.1364,"Cardiometabolic_II","B13805","20212097_SS211647","PASS",0.3563,1.4886,"Plate control","PASS","PFC",2.80616530998062,"Baseline","Baseline"
6560,"PFC_16",86,"OID30292","Q12982","BNIP2",0.1364,"Cardiometabolic_II","B13805","20212097_SS211647","PASS",0.3563,1.8014,"Plate control","PASS","PFC",3.48558304343236,"1h","1h"
6561,"PFC_24",87,"OID30292","Q12982","BNIP2",0.1364,"Cardiometabolic_II","B13805","20212097_SS211647","PASS",0.3563,2.1857,"Plate control","PASS","PFC",4.54947477643751,"24h","24h"
6562,"PFC_01",1,"OID30293","Q96DC8","ECHDC3",0,"Cardiometabolic_II","B13805","20212097_SS211647","PASS",-2.9801,-0.6704,"Plate control","PASS","PFC",0.628332452360821,"Baseline","Baseline"
6563,"PFC_09",2,"OID30293","Q96DC8","ECHDC3",0,"Cardiometabolic_II","B13805","20212097_SS211647","PASS",-2.9801,0.0795,"Plate control","PASS","PFC",1.05665176949768,"Baseline","Baseline"
6564,"PFC_17",3,"OID30293","Q96DC8","ECHDC3",0,"Cardiometabolic_II","B13805","20212097_SS211647","PASS",-2.9801,-0.2248,"Plate control","PASS","PFC",0.855713644560397,"1h","1h"
6565,"PFC_25",4,"OID30293","Q96DC8","ECHDC3",0,"Cardiometabolic_II","B13805","20212097_SS211647","PASS",-2.9801,-0.6101,"Plate control","PASS","PFC",0.655151288728436,"24h","24h"
6566,"PFC_02",13,"OID30293","Q96DC8","ECHDC3",0,"Cardiometabolic_II","B13805","20212097_SS211647","PASS",-2.9801,-0.2272,"Plate control","PASS","PFC",0.85429130275884,"Baseline","Baseline"
6567,"PFC_10",14,"OID30293","Q96DC8","ECHDC3",0,"Cardiometabolic_II","B13805","20212097_SS211647","PASS",-2.9801,-0.1516,"Plate control","PASS","PFC",0.900251497909454,"1h","1h"
6568,"PFC_18",15,"OID30293","Q96DC8","ECHDC3",0,"Cardiometabolic_II","B13805","20212097_SS211647","PASS",-2.9801,0.114,"Plate control","PASS","PFC",1.08222464452571,"1h","1h"
6569,"PFC_26",16,"OID30293","Q96DC8","ECHDC3",0,"Cardiometabolic_II","B13805","20212097_SS211647","PASS",-2.9801,-0.4177,"Plate control","PASS","PFC",0.748617147771665,"24h","24h"
6570,"PFC_03",25,"OID30293","Q96DC8","ECHDC3",0,"Cardiometabolic_II","B13805","20212097_SS211647","PASS",-2.9801,-0.7632,"Plate control","PASS","PFC",0.589188019441137,"Baseline","Baseline"
6571,"PFC_11",26,"OID30293","Q96DC8","ECHDC3",0,"Cardiometabolic_II","B13805","20212097_SS211647","PASS",-2.9801,0.1439,"Plate control","PASS","PFC",1.10488789715254,"1h","1h"
6572,"PFC_19",27,"OID30293","Q96DC8","ECHDC3",0,"Cardiometabolic_II","B13805","20212097_SS211647","PASS",-2.9801,0.3466,"Plate control","PASS","PFC",1.27156040646079,"24h","24h"
6573,"PFC_27",28,"OID30293","Q96DC8","ECHDC3",0,"Cardiometabolic_II","B13805","20212097_SS211647","PASS",-2.9801,1.4288,"Plate control","PASS","PFC",2.69222689078684,"24h","24h"
6574,"PFC_04",37,"OID30293","Q96DC8","ECHDC3",0,"Cardiometabolic_II","B13805","20212097_SS211647","PASS",-2.9801,-1.257,"Plate control","PASS","PFC",0.418413121341696,"Baseline","Baseline"
6575,"PFC_12",38,"OID30293","Q96DC8","ECHDC3",0,"Cardiometabolic_II","B13805","20212097_SS211647","PASS",-2.9801,-1.2505,"Plate control","PASS","PFC",0.420302516629798,"1h","1h"
6576,"PFC_20",39,"OID30293","Q96DC8","ECHDC3",0,"Cardiometabolic_II","B13805","20212097_SS211647","PASS",-2.9801,0.3155,"Plate control","PASS","PFC",1.24444286960328,"24h","24h"
6577,"PFC_05",49,"OID30293","Q96DC8","ECHDC3",0,"Cardiometabolic_II","B13805","20212097_SS211647","PASS",-2.9801,-1.5201,"Plate control","PASS","PFC",0.34866174835922,"Baseline","Baseline"
6578,"PFC_13",50,"OID30293","Q96DC8","ECHDC3",0,"Cardiometabolic_II","B13805","20212097_SS211647","PASS",-2.9801,-0.9632,"Plate control","PASS","PFC",0.51291796221181,"1h","1h"
6579,"PFC_21",51,"OID30293","Q96DC8","ECHDC3",0,"Cardiometabolic_II","B13805","20212097_SS211647","PASS",-2.9801,-1.1624,"Plate control","PASS","PFC",0.446768693420614,"24h","24h"
6580,"PFC_06",61,"OID30293","Q96DC8","ECHDC3",0,"Cardiometabolic_II","B13805","20212097_SS211647","PASS",-2.9801,-0.0056,"Plate control","PASS","PFC",0.996125899554165,"Baseline","Baseline"
6581,"PFC_14",62,"OID30293","Q96DC8","ECHDC3",0,"Cardiometabolic_II","B13805","20212097_SS211647","PASS",-2.9801,-1.3015,"Plate control","PASS","PFC",0.405704159773357,"1h","1h"
6582,"PFC_22",63,"OID30293","Q96DC8","ECHDC3",0,"Cardiometabolic_II","B13805","20212097_SS211647","PASS",-2.9801,-0.9118,"Plate control","PASS","PFC",0.531521516600218,"24h","24h"
6583,"PFC_07",73,"OID30293","Q96DC8","ECHDC3",0,"Cardiometabolic_II","B13805","20212097_SS211647","PASS",-2.9801,-0.2191,"Plate control","PASS","PFC",0.859101204525575,"Baseline","Baseline"
6584,"PFC_15",74,"OID30293","Q96DC8","ECHDC3",0,"Cardiometabolic_II","B13805","20212097_SS211647","PASS",-2.9801,-0.4143,"Plate control","PASS","PFC",0.750383494671379,"1h","1h"
6585,"PFC_23",75,"OID30293","Q96DC8","ECHDC3",0,"Cardiometabolic_II","B13805","20212097_SS211647","PASS",-2.9801,-1.0301,"Plate control","PASS","PFC",0.489676205848957,"24h","24h"
6586,"PFC_08",85,"OID30293","Q96DC8","ECHDC3",0,"Cardiometabolic_II","B13805","20212097_SS211647","PASS",-2.9801,-1.4221,"Plate control","PASS","PFC",0.37316872884254,"Baseline","Baseline"
6587,"PFC_16",86,"OID30293","Q96DC8","ECHDC3",0,"Cardiometabolic_II","B13805","20212097_SS211647","PASS",-2.9801,-1.1781,"Plate control","PASS","PFC",0.441933131999726,"1h","1h"
6588,"PFC_24",87,"OID30293","Q96DC8","ECHDC3",0,"Cardiometabolic_II","B13805","20212097_SS211647","PASS",-2.9801,0.0575,"Plate control","PASS","PFC",1.04066086960447,"24h","24h"
6589,"PFC_01",1,"OID30294","Q8TCD5","NT5C",0,"Cardiometabolic_II","B13805","20212097_SS211647","PASS",-0.9683,3.486,"Plate control","PASS","PFC",11.2044505670976,"Baseline","Baseline"
6590,"PFC_09",2,"OID30294","Q8TCD5","NT5C",0,"Cardiometabolic_II","B13805","20212097_SS211647","PASS",-0.9683,4.1925,"Plate control","PASS","PFC",18.2838755600327,"Baseline","Baseline"
6591,"PFC_17",3,"OID30294","Q8TCD5","NT5C",0,"Cardiometabolic_II","B13805","20212097_SS211647","PASS",-0.9683,5.8701,"Plate control","PASS","PFC",58.4892668412186,"1h","1h"
6592,"PFC_25",4,"OID30294","Q8TCD5","NT5C",0,"Cardiometabolic_II","B13805","20212097_SS211647","PASS",-0.9683,4.7397,"Plate control","PASS","PFC",26.7172571465914,"24h","24h"
6593,"PFC_02",13,"OID30294","Q8TCD5","NT5C",0,"Cardiometabolic_II","B13805","20212097_SS211647","PASS",-0.9683,3.7319,"Plate control","PASS","PFC",13.2865993966445,"Baseline","Baseline"
6594,"PFC_10",14,"OID30294","Q8TCD5","NT5C",0,"Cardiometabolic_II","B13805","20212097_SS211647","PASS",-0.9683,4.085,"Plate control","PASS","PFC",16.9710038618915,"1h","1h"
6595,"PFC_18",15,"OID30294","Q8TCD5","NT5C",0,"Cardiometabolic_II","B13805","20212097_SS211647","PASS",-0.9683,4.5663,"Plate control","PASS","PFC",23.6915387728071,"1h","1h"
6596,"PFC_26",16,"OID30294","Q8TCD5","NT5C",0,"Cardiometabolic_II","B13805","20212097_SS211647","PASS",-0.9683,5.7858,"Plate control","PASS","PFC",55.169538228999,"24h","24h"
6597,"PFC_03",25,"OID30294","Q8TCD5","NT5C",0,"Cardiometabolic_II","B13805","20212097_SS211647","PASS",-0.9683,2.8158,"Plate control","PASS","PFC",7.04109593768431,"Baseline","Baseline"
6598,"PFC_11",26,"OID30294","Q8TCD5","NT5C",0,"Cardiometabolic_II","B13805","20212097_SS211647","PASS",-0.9683,5.1543,"Plate control","PASS","PFC",35.6122084393803,"1h","1h"
6599,"PFC_19",27,"OID30294","Q8TCD5","NT5C",0,"Cardiometabolic_II","B13805","20212097_SS211647","PASS",-0.9683,4.8146,"Plate control","PASS","PFC",28.1409670150071,"24h","24h"
6600,"PFC_27",28,"OID30294","Q8TCD5","NT5C",0,"Cardiometabolic_II","B13805","20212097_SS211647","PASS",-0.9683,6.3316,"Plate control","PASS","PFC",80.5381260984289,"24h","24h"
6601,"PFC_04",37,"OID30294","Q8TCD5","NT5C",0,"Cardiometabolic_II","B13805","20212097_SS211647","PASS",-0.9683,4.9203,"Plate control","PASS","PFC",30.2801406188252,"Baseline","Baseline"
6602,"PFC_12",38,"OID30294","Q8TCD5","NT5C",0,"Cardiometabolic_II","B13805","20212097_SS211647","PASS",-0.9683,3.2435,"Plate control","PASS","PFC",9.47088996563511,"1h","1h"
6603,"PFC_20",39,"OID30294","Q8TCD5","NT5C",0,"Cardiometabolic_II","B13805","20212097_SS211647","PASS",-0.9683,5.4264,"Plate control","PASS","PFC",43.0040312195179,"24h","24h"
6604,"PFC_05",49,"OID30294","Q8TCD5","NT5C",0,"Cardiometabolic_II","B13805","20212097_SS211647","PASS",-0.9683,3.2143,"Plate control","PASS","PFC",9.28112699149781,"Baseline","Baseline"
6605,"PFC_13",50,"OID30294","Q8TCD5","NT5C",0,"Cardiometabolic_II","B13805","20212097_SS211647","PASS",-0.9683,4.908,"Plate control","PASS","PFC",30.0230782880215,"1h","1h"
6606,"PFC_21",51,"OID30294","Q8TCD5","NT5C",0,"Cardiometabolic_II","B13805","20212097_SS211647","PASS",-0.9683,3.9519,"Plate control","PASS","PFC",15.4753485672234,"24h","24h"
6607,"PFC_06",61,"OID30294","Q8TCD5","NT5C",0,"Cardiometabolic_II","B13805","20212097_SS211647","PASS",-0.9683,4.6107,"Plate control","PASS","PFC",24.4319989477495,"Baseline","Baseline"
6608,"PFC_14",62,"OID30294","Q8TCD5","NT5C",0,"Cardiometabolic_II","B13805","20212097_SS211647","PASS",-0.9683,4.36,"Plate control","PASS","PFC",20.5348143610065,"1h","1h"

Supplemental table 1_Olink proximity extension assay (PEA) data

,"SampleID","Index","OlinkID","UniProt","Assay","MissingFreq","Panel","Panel_Lot_Nr","PlateID","QC_Warning","LOD","NPX","Normalization","Assay_Warning","condition","Value","group","alt_group"
6609,"PFC_22",63,"OID30294","Q8TCD5","NT5C",0,"Cardiometabolic_II","B13805","20212097_SS211647","PASS",-0.9683,4.2997,"Plate control","PASS","PFC",19.6942148908352,"24h","24h"
6610,"PFC_07",73,"OID30294","Q8TCD5","NT5C",0,"Cardiometabolic_II","B13805","20212097_SS211647","PASS",-0.9683,5.3025,"Plate control","PASS","PFC",39.4649495542411,"Baseline","Baseline"
6611,"PFC_15",74,"OID30294","Q8TCD5","NT5C",0,"Cardiometabolic_II","B13805","20212097_SS211647","PASS",-0.9683,3.3183,"Plate control","PASS","PFC",9.97488355727914,"1h","1h"
6612,"PFC_23",75,"OID30294","Q8TCD5","NT5C",0,"Cardiometabolic_II","B13805","20212097_SS211647","PASS",-0.9683,4.9572,"Plate control","PASS","PFC",31.0646092033945,"24h","24h"
6613,"PFC_08",85,"OID30294","Q8TCD5","NT5C",0,"Cardiometabolic_II","B13805","20212097_SS211647","PASS",-0.9683,4.5546,"Plate control","PASS","PFC",23.5001815952646,"Baseline","Baseline"
6614,"PFC_16",86,"OID30294","Q8TCD5","NT5C",0,"Cardiometabolic_II","B13805","20212097_SS211647","PASS",-0.9683,4.5417,"Plate control","PASS","PFC",23.2909890553715,"1h","1h"
6615,"PFC_24",87,"OID30294","Q8TCD5","NT5C",0,"Cardiometabolic_II","B13805","20212097_SS211647","PASS",-0.9683,5.2008,"Plate control","PASS","PFC",36.7787361682283,"24h","24h"
6616,"PFC_01",1,"OID30295","Q7Z7M9","GALNT5",0,"Cardiometabolic_II","B13805","20212097_SS211647","PASS",-1.0491,0.2213,"Plate control","PASS","PFC",1.16578359081435,"Baseline","Baseline"
6617,"PFC_09",2,"OID30295","Q7Z7M9","GALNT5",0,"Cardiometabolic_II","B13805","20212097_SS211647","PASS",-1.0491,0.6053,"Plate control","PASS","PFC",1.52129506476779,"Baseline","Baseline"
6618,"PFC_17",3,"OID30295","Q7Z7M9","GALNT5",0,"Cardiometabolic_II","B13805","20212097_SS211647","PASS",-1.0491,-0.6808,"Plate control","PASS","PFC",0.623819259675188,"1h","1h"
6619,"PFC_25",4,"OID30295","Q7Z7M9","GALNT5",0,"Cardiometabolic_II","B13805","20212097_SS211647","PASS",-1.0491,0.5516,"Plate control","PASS","PFC",1.46571031965737,"24h","24h"
6620,"PFC_02",13,"OID30295","Q7Z7M9","GALNT5",0,"Cardiometabolic_II","B13805","20212097_SS211647","PASS",-1.0491,0.4688,"Plate control","PASS","PFC",1.38395784545661,"Baseline","Baseline"
6621,"PFC_10",14,"OID30295","Q7Z7M9","GALNT5",0,"Cardiometabolic_II","B13805","20212097_SS211647","PASS",-1.0491,0.7097,"Plate control","PASS","PFC",1.63546399657631,"1h","1h"
6622,"PFC_18",15,"OID30295","Q7Z7M9","GALNT5",0,"Cardiometabolic_II","B13805","20212097_SS211647","PASS",-1.0491,0.2302,"Plate control","PASS","PFC",1.17299754994991,"1h","1h"
6623,"PFC_26",16,"OID30295","Q7Z7M9","GALNT5",0,"Cardiometabolic_II","B13805","20212097_SS211647","PASS",-1.0491,-0.6223,"Plate control","PASS","PFC",0.649634429499439,"24h","24h"
6624,"PFC_03",25,"OID30295","Q7Z7M9","GALNT5",0,"Cardiometabolic_II","B13805","20212097_SS211647","PASS",-1.0491,-0.0131,"Plate control","PASS","PFC",0.990960872709977,"Baseline","Baseline"
6625,"PFC_11",26,"OID30295","Q7Z7M9","GALNT5",0,"Cardiometabolic_II","B13805","20212097_SS211647","PASS",-1.0491,0.5207,"Plate control","PASS","PFC",1.43465117626147,"1h","1h"
6626,"PFC_19",27,"OID30295","Q7Z7M9","GALNT5",0,"Cardiometabolic_II","B13805","20212097_SS211647","PASS",-1.0491,0.6607,"Plate control","PASS","PFC",1.58084947062091,"24h","24h"
6627,"PFC_27",28,"OID30295","Q7Z7M9","GALNT5",0,"Cardiometabolic_II","B13805","20212097_SS211647","PASS",-1.0491,0.7286,"Plate control","PASS","PFC",1.65703031892262,"24h","24h"
6628,"PFC_04",37,"OID30295","Q7Z7M9","GALNT5",0,"Cardiometabolic_II","B13805","20212097_SS211647","PASS",-1.0491,0.249,"Plate control","PASS","PFC",1.18838310505693,"Baseline","Baseline"
6629,"PFC_12",38,"OID30295","Q7Z7M9","GALNT5",0,"Cardiometabolic_II","B13805","20212097_SS211647","PASS",-1.0491,-0.9365,"Plate control","PASS","PFC",0.522498934387825,"1h","1h"
6630,"PFC_20",39,"OID30295","Q7Z7M9","GALNT5",0,"Cardiometabolic_II","B13805","20212097_SS211647","PASS",-1.0491,0.7534,"Plate control","PASS","PFC",1.68576098640791,"24h","24h"
6631,"PFC_05",49,"OID30295","Q7Z7M9","GALNT5",0,"Cardiometabolic_II","B13805","20212097_SS211647","PASS",-1.0491,0.8427,"Plate control","PASS","PFC",1.79340335280399,"Baseline","Baseline"
6632,"PFC_13",50,"OID30295","Q7Z7M9","GALNT5",0,"Cardiometabolic_II","B13805","20212097_SS211647","PASS",-1.0491,0.2505,"Plate control","PASS","PFC",1.18961933421017,"1h","1h"
6633,"PFC_21",51,"OID30295","Q7Z7M9","GALNT5",0,"Cardiometabolic_II","B13805","20212097_SS211647","PASS",-1.0491,-0.1359,"Plate control","PASS","PFC",0.910101905351615,"24h","24h"
6634,"PFC_06",61,"OID30295","Q7Z7M9","GALNT5",0,"Cardiometabolic_II","B13805","20212097_SS211647","PASS",-1.0491,0.0226,"Plate control","PASS","PFC",1.01578846758206,"Baseline","Baseline"
6635,"PFC_14",62,"OID30295","Q7Z7M9","GALNT5",0,"Cardiometabolic_II","B13805","20212097_SS211647","PASS",-1.0491,0.6809,"Plate control","PASS","PFC",1.60313953378274,"1h","1h"
6636,"PFC_22",63,"OID30295","Q7Z7M9","GALNT5",0,"Cardiometabolic_II","B13805","20212097_SS211647","PASS",-1.0491,0.2058,"Plate control","PASS","PFC",1.15332570908443,"24h","24h"
6637,"PFC_07",73,"OID30295","Q7Z7M9","GALNT5",0,"Cardiometabolic_II","B13805","20212097_SS211647","PASS",-1.0491,0.3627,"Plate control","PASS","PFC",1.28583007478123,"Baseline","Baseline"
6638,"PFC_15",74,"OID30295","Q7Z7M9","GALNT5",0,"Cardiometabolic_II","B13805","20212097_SS211647","PASS",-1.0491,0.1038,"Plate control","PASS","PFC",1.07460019131056,"1h","1h"
6639,"PFC_23",75,"OID30295","Q7Z7M9","GALNT5",0,"Cardiometabolic_II","B13805","20212097_SS211647","PASS",-1.0491,1.0271,"Plate control","PASS","PFC",2.0379236464409,"24h","24h"
6640,"PFC_08",85,"OID30295","Q7Z7M9","GALNT5",0,"Cardiometabolic_II","B13805","20212097_SS211647","PASS",-1.0491,-0.4081,"Plate control","PASS","PFC",0.753615216342522,"Baseline","Baseline"
6641,"PFC_16",86,"OID30295","Q7Z7M9","GALNT5",0,"Cardiometabolic_II","B13805","20212097_SS211647","PASS",-1.0491,0.5689,"Plate control","PASS","PFC",1.48339210937171,"1h","1h"
6642,"PFC_24",87,"OID30295","Q7Z7M9","GALNT5",0,"Cardiometabolic_II","B13805","20212097_SS211647","PASS",-1.0491,-0.0305,"Plate control","PASS","PFC",0.979080915191884,"24h","24h"
6643,"PFC_01",1,"OID30296","Q00872","MYBPC1",0,"Cardiometabolic_II","B13805","20212097_SS211647","PASS",-2.7369,-0.757,"Plate control","PASS","PFC",0.591725510876286,"Baseline","Baseline"
6644,"PFC_09",2,"OID30296","Q00872","MYBPC1",0,"Cardiometabolic_II","B13805","20212097_SS211647","PASS",-2.7369,-0.5439,"Plate control","PASS","PFC",0.685914186596044,"Baseline","Baseline"
6645,"PFC_17",3,"OID30296","Q00872","MYBPC1",0,"Cardiometabolic_II","B13805","20212097_SS211647","PASS",-2.7369,-1.5305,"Plate control","PASS","PFC",0.346157377231256,"1h","1h"
6646,"PFC_25",4,"OID30296","Q00872","MYBPC1",0,"Cardiometabolic_II","B13805","20212097_SS211647","PASS",-2.7369,-2.0808,"Plate control","PASS","PFC",0.236383296599483,"24h","24h"
6647,"PFC_02",13,"OID30296","Q00872","MYBPC1",0,"Cardiometabolic_II","B13805","20212097_SS211647","PASS",-2.7369,0.0394,"Plate control","PASS","PFC",1.02768633503647,"Baseline","Baseline"
6648,"PFC_10",14,"OID30296","Q00872","MYBPC1",0,"Cardiometabolic_II","B13805","20212097_SS211647","PASS",-2.7369,-0.1531,"Plate control","PASS","PFC",0.899315974154115,"1h","1h"
6649,"PFC_18",15,"OID30296","Q00872","MYBPC1",0,"Cardiometabolic_II","B13805","20212097_SS211647","PASS",-2.7369,0.7029,"Plate control","PASS","PFC",1.62777353756662,"1h","1h"
6650,"PFC_26",16,"OID30296","Q00872","MYBPC1",0,"Cardiometabolic_II","B13805","20212097_SS211647","PASS",-2.7369,-1.5084,"Plate control","PASS","PFC",0.35150082974302,"24h","24h"
6651,"PFC_03",25,"OID30296","Q00872","MYBPC1",0,"Cardiometabolic_II","B13805","20212097_SS211647","PASS",-2.7369,0.1293,"Plate control","PASS","PFC",1.09376287543469,"Baseline","Baseline"
6652,"PFC_11",26,"OID30296","Q00872","MYBPC1",0,"Cardiometabolic_II","B13805","20212097_SS211647","PASS",-2.7369,1.7852,"Plate control","PASS","PFC",3.44666241459742,"1h","1h"
6653,"PFC_19",27,"OID30296","Q00872","MYBPC1",0,"Cardiometabolic_II","B13805","20212097_SS211647","PASS",-2.7369,-0.9576,"Plate control","PASS","PFC",0.514912786065874,"24h","24h"
6654,"PFC_27",28,"OID30296","Q00872","MYBPC1",0,"Cardiometabolic_II","B13805","20212097_SS211647","PASS",-2.7369,-0.4822,"Plate control","PASS","PFC",0.71588512097063,"24h","24h"
6655,"PFC_04",37,"OID30296","Q00872","MYBPC1",0,"Cardiometabolic_II","B13805","20212097_SS211647","PASS",-2.7369,-0.6843,"Plate control","PASS","PFC",0.622307698987522,"Baseline","Baseline"
6656,"PFC_12",38,"OID30296","Q00872","MYBPC1",0,"Cardiometabolic_II","B13805","20212097_SS211647","PASS",-2.7369,1.1485,"Plate control","PASS","PFC",2.21683285833249,"1h","1h"
6657,"PFC_20",39,"OID30296","Q00872","MYBPC1",0,"Cardiometabolic_II","B13805","20212097_SS211647","PASS",-2.7369,0.612,"Plate control","PASS","PFC",1.52837652076517,"24h","24h"
6658,"PFC_05",49,"OID30296","Q00872","MYBPC1",0,"Cardiometabolic_II","B13805","20212097_SS211647","PASS",-2.7369,0.3389,"Plate control","PASS","PFC",1.26479187062359,"Baseline","Baseline"
6659,"PFC_13",50,"OID30296","Q00872","MYBPC1",0,"Cardiometabolic_II","B13805","20212097_SS211647","PASS",-2.7369,-0.4895,"Plate control","PASS","PFC",0.712271909659815,"1h","1h"
6660,"PFC_21",51,"OID30296","Q00872","MYBPC1",0,"Cardiometabolic_II","B13805","20212097_SS211647","PASS",-2.7369,1.059,"Plate control","PASS","PFC",2.08348685801644,"24h","24h"
6661,"PFC_06",61,"OID30296","Q00872","MYBPC1",0,"Cardiometabolic_II","B13805","20212097_SS211647","PASS",-2.7369,-1.2412,"Plate control","PASS","PFC",0.423020651120798,"Baseline","Baseline"
6662,"PFC_14",62,"OID30296","Q00872","MYBPC1",0,"Cardiometabolic_II","B13805","20212097_SS211647","PASS",-2.7369,-0.0038,"Plate control","PASS","PFC",0.997369506541016,"1h","1h"
6663,"PFC_22",63,"OID30296","Q00872","MYBPC1",0,"Cardiometabolic_II","B13805","20212097_SS211647","PASS",-2.7369,-1.2236,"Plate control","PASS","PFC",0.428212851628171,"24h","24h"
6664,"PFC_07",73,"OID30296","Q00872","MYBPC1",0,"Cardiometabolic_II","B13805","20212097_SS211647","PASS",-2.7369,-1.4301,"Plate control","PASS","PFC",0.37110516871561,"Baseline","Baseline"
6665,"PFC_15",74,"OID30296","Q00872","MYBPC1",0,"Cardiometabolic_II","B13805","20212097_SS211647","PASS",-2.7369,-0.0549,"Plate control","PASS","PFC",0.962661167376638,"1h","1h"
6666,"PFC_23",75,"OID30296","Q00872","MYBPC1",0,"Cardiometabolic_II","B13805","20212097_SS211647","PASS",-2.7369,0.149,"Plate control","PASS","PFC",1.10880064360307,"24h","24h"
6667,"PFC_08",85,"OID30296","Q00872","MYBPC1",0,"Cardiometabolic_II","B13805","20212097_SS211647","PASS",-2.7369,-2.1018,"Plate control","PASS","PFC",0.232967401227164,"Baseline","Baseline"
6668,"PFC_16",86,"OID30296","Q00872","MYBPC1",0,"Cardiometabolic_II","B13805","20212097_SS211647","PASS",-2.7369,-1.532,"Plate control","PASS","PFC",0.345797657252741,"1h","1h"
6669,"PFC_24",87,"OID30296","Q00872","MYBPC1",0,"Cardiometabolic_II","B13805","20212097_SS211647","PASS",-2.7369,-0.3057,"Plate control","PASS","PFC",0.809049567999085,"24h","24h"
6670,"PFC_01",1,"OID30297","Q14914","PTGR1",0,"Cardiometabolic_II","B13805","20212097_SS211647","PASS",-4.2836,1.2235,"Plate control","PASS","PFC",2.33512535619183,"Baseline","Baseline"
6671,"PFC_09",2,"OID30297","Q14914","PTGR1",0,"Cardiometabolic_II","B13805","20212097_SS211647","PASS",-4.2836,1.2801,"Plate control","PASS","PFC",2.42855809777602,"Baseline","Baseline"
6672,"PFC_17",3,"OID30297","Q14914","PTGR1",0,"Cardiometabolic_II","B13805","20212097_SS211647","PASS",-4.2836,2.276,"Plate control","PASS","PFC",4.84333233580221,"1h","1h"
6673,"PFC_25",4,"OID30297","Q14914","PTGR1",0,"Cardiometabolic_II","B13805","20212097_SS211647","PASS",-4.2836,2.4132,"Plate control","PASS","PFC",5.32654481766926,"24h","24h"
6674,"PFC_02",13,"OID30297","Q14914","PTGR1",0,"Cardiometabolic_II","B13805","20212097_SS211647","PASS",-4.2836,1.0028,"Plate control","PASS","PFC",2.0038853934008,"Baseline","Baseline"
6675,"PFC_10",14,"OID30297","Q14914","PTGR1",0,"Cardiometabolic_II","B13805","20212097_SS211647","PASS",-4.2836,1.1507,"Plate control","PASS","PFC",2.22021593832666,"1h","1h"
6676,"PFC_18",15,"OID30297","Q14914","PTGR1",0,"Cardiometabolic_II","B13805","20212097_SS211647","PASS",-4.2836,0.9503,"Plate control","PASS","PFC",1.93227442124622,"1h","1h"
6677,"PFC_26",16,"OID30297","Q14914","PTGR1",0,"Cardiometabolic_II","B13805","20212097_SS211647","PASS",-4.2836,2.3254,"Plate control","PASS","PFC",5.01204719642327,"24h","24h"
6678,"PFC_03",25,"OID30297","Q14914","PTGR1",0,"Cardiometabolic_II","B13805","20212097_SS211647","PASS",-4.2836,1.7307,"Plate control","PASS","PFC",3.31888812721492,"Baseline","Baseline"
6679,"PFC_11",26,"OID30297","Q14914","PTGR1",0,"Cardiometabolic_II","B13805","20212097_SS211647","PASS",-4.2836,1.3751,"Plate control","PASS","PFC",2.59385889566906,"1h","1h"
6680,"PFC_19",27,"OID30297","Q14914","PTGR1",0,"Cardiometabolic_II","B13805","20212097_SS211647","PASS",-4.2836,1.5157,"Plate control","PASS","PFC",2.85937532051582,"24h","24h"
6681,"PFC_27",28,"OID30297","Q14914","PTGR1",0,"Cardiometabolic_II","B13805","20212097_SS211647","PASS",-4.2836,2.0394,"Plate control","PASS","PFC",4.11074534014587,"24h","24h"
6682,"PFC_04",37,"OID30297","Q14914","PTGR1",0,"Cardiometabolic_II","B13805","20212097_SS211647","PASS",-4.2836,2.2015,"Plate control","PASS","PFC",4.59957320653571,"Baseline","Baseline"
6683,"PFC_12",38,"OID30297","Q14914","PTGR1",0,"Cardiometabolic_II","B13805","20212097_SS211647","PASS",-4.2836,1.3732,"Plate control","PASS","PFC",2.59044508475964,"1h","1h"
6684,"PFC_20",39,"OID30297","Q14914","PTGR1",0,"Cardiometabolic_II","B13805","20212097_SS211647","PASS",-4.2836,1.3341,"Plate control","PASS","PFC",2.52118153209663,"24h","24h"
6685,"PFC_05",49,"OID30297","Q14914","PTGR1",0,"Cardiometabolic_II","B13805","20212097_SS211647","PASS",-4.2836,0.4773,"Plate control","PASS","PFC",1.39213584824612,"Baseline","Baseline"
6686,"PFC_13",50,"OID30297","Q14914","PTGR1",0,"Cardiometabolic_II","B13805","20212097_SS211647","PASS",-4.2836,2.4405,"Plate control","PASS","PFC",5.42829828865007,"1h","1h"
6687,"PFC_21",51,"OID30297","Q14914","PTGR1",0,"Cardiometabolic_II","B13805","20212097_SS211647","PASS",-4.2836,1.3937,"Plate control","PASS","PFC",2.62751682072403,"24h","24h"
6688,"PFC_06",61,"OID30297","Q14914","PTGR1",0,"Cardiometabolic_II","B13805","20212097_SS211647","PASS",-4.2836,2.9449,"Plate control","PASS","PFC",7.70022178761343,"Baseline","Baseline"
6689,"PFC_14",62,"OID30297","Q14914","PTGR1",0,"Cardiometabolic_II","B13805","20212097_SS211647","PASS",-4.2836,0.8138,"Plate control","PASS","PFC",1.75783541719898,"1h","1h"
6690,"PFC_22",63,"OID30297","Q14914","PTGR1",0,"Cardiometabolic_II","B13805","20212097_SS211647","PASS",-4.2836,2.1157,"Plate control","PASS","PFC",4.33400254317682,"24h","24h"
6691,"PFC_07",73,"OID30297","Q14914","PTGR1",0,"Cardiometabolic_II","B13805","20212097_SS211647","PASS",-4.2836,2.6815,"Plate control","PASS","PFC",6.41522559773071,"Baseline","Baseline"
6692,"PFC_15",74,"OID30297","Q14914","PTGR1",0,"Cardiometabolic_II","B13805","20212097_SS211647","PASS",-4.2836,3.0378,"Plate control","PASS","PFC",8.21237780953824,"1h","1h"
6693,"PFC_23",75,"OID30297","Q14914","PTGR1",0,"Cardiometabolic_II","B13805","20212097_SS211647","PASS",-4.2836,0.9311,"Plate control","PASS","PFC",1.90672925039636,"24h","24h"
6694,"PFC_08",85,"OID30297","Q14914","PTGR1",0,"Cardiometabolic_II","B13805","20212097_SS211647","PASS",-4.2836,2.1638,"Plate control","PASS","PFC",4.48093562413831,"Baseline","Baseline"
6695,"PFC_16",86,"OID30297","Q14914","PTGR1",0,"Cardiometabolic_II","B13805","20212097_SS211647","PASS",-4.2836,2.4569,"Plate control","PASS","PFC",5.49035714927221,"1h","1h"
6696,"PFC_24",87,"OID30297","Q14914","PTGR1",0,"Cardiometabolic_II","B13805","20212097_SS211647","PASS",-4.2836,3.25,"Plate control","PASS","PFC",9.51365692002177,"24h","24h"
6697,"PFC_01",1,"OID30298","Q6UW49","SPESP1",0.0909,"Cardiometabolic_II","B13805","20212097_SS211647","PASS",-0.3033,1.7751,"Plate control","PASS","PFC",3.42261733226396,"Baseline","Baseline"
6698,"PFC_09",2,"OID30298","Q6UW49","SPESP1",0.0909,"Cardiometabolic_II","B13805","20212097_SS211647","PASS",-0.3033,1.555,"Plate control","PASS","PFC",2.93833726655662,"Baseline","Baseline"
6699,"PFC_17",3,"OID30298","Q6UW49","SPESP1",0.0909,"Cardiometabolic_II","B13805","20212097_SS211647","PASS",-0.3033,1.4187,"Plate control","PASS","PFC",2.67344500574491,"1h","1h"
6700,"PFC_25",4,"OID30298","Q6UW49","SPESP1",0.0909,"Cardiometabolic_II","B13805","20212097_SS211647","PASS",-0.3033,0.0943,"Plate control","PASS","PFC",1.06754730518219,"24h","24h"
6701,"PFC_02",13,"OID30298","Q6UW49","SPESP1",0.0909,"Cardiometabolic_II","B13805","20212097_SS211647","PASS",-0.3033,-0.0719,"Plate control","PASS","PFC",0.951384218975276,"Baseline","Baseline"
6702,"PFC_10",14,"OID30298","Q6UW49","SPESP1",0.0909,"Cardiometabolic_II","B13805","20212097_SS211647","PASS",-0.3033,1.6631,"Plate control","PASS","PFC",3.16696297302295,"1h","1h"
6703,"PFC_18",15,"OID30298","Q6UW49","SPESP1",0.0909,"Cardiometabolic_II","B13805","20212097_SS211647","PASS",-0.3033,1.43,"Plate control","PASS","PFC",2.69446715373138,"1h","1h"
6704,"PFC_26",16,"OID30298","Q6UW49","SPESP1",0.0909,"Cardiometabolic_II","B13805","20212097_SS211647","PASS",-0.3033,1.505,"Plate control","PASS","PFC",2.83824671240076,"24h","24h"
6705,"PFC_03",25,"OID30298","Q6UW49","SPESP1",0.0909,"Cardiometabolic_II","B13805","20212097_SS211647","PASS",-0.3033,0.5365,"Plate control","PASS","PFC",1.45044943324741,"Baseline","Baseline"
6706,"PFC_11",26,"OID30298","Q6UW49","SPESP1",0.0909,"Cardiometabolic_II","B13805","20212097_SS211647","PASS",-0.3033,-0.1023,"Plate control","PASS","PFC",0.931546699472488,"1h","1h"
6707,"PFC_19",27,"OID30298","Q6UW49","SPESP1",0.0909,"Cardiometabolic_II","B13805","20212097_SS211647","PASS",-0.3033,1.9148,"Plate control","PASS","PFC",3.77061538915402,"24h","24h"
6708,"PFC_27",28,"OID30298","Q6UW49","SPESP1",0.0909,"Cardiometabolic_II","B13805","20212097_SS211647","PASS",-0.3033,1.8952,"Plate control","PASS","PFC",3.71973539953206,"24h","24h"
6709,"PFC_04",37,"OID30298","Q6UW49","SPESP1",0.0909,"Cardiometabolic_II","B13805","20212097_SS211647","PASS",-0.3033,1.1944,"Plate control","PASS","PFC",2.28849636437562,"Baseline","Baseline"
6710,"PFC_12",38,"OID30298","Q6UW49","SPESP1",0.0909,"Cardiometabolic_II","B13805","20212097_SS211647","PASS",-0.3033,0.5106,"Plate control","PASS","PFC",1.42464256459504,"1h","1h"
6711,"PFC_20",39,"OID30298","Q6UW49","SPESP1",0.0909,"Cardiometabolic_II","B13805","20212097_SS211647","PASS",-0.3033,0.1295,"Plate control","PASS","PFC",1.09391451367587,"24h","24h"
6712,"PFC_05",49,"OID30298","Q6UW49","SPESP1",0.0909,"Cardiometabolic_II","B13805","20212097_SS211647","PASS",-0.3033,1.3333,"Plate control","PASS","PFC",2.51978387974744,"Baseline","Baseline"
6713,"PFC_13",50,"OID30298","Q6UW49","SPESP1",0.0909,"Cardiometabolic_II","B13805","20212097_SS211647","PASS",-0.3033,0.8999,"Plate control","PASS","PFC",1.86593664171881,"1h","1h"
6714,"PFC_21",51,"OID30298","Q6UW49","SPESP1",0.0909,"Cardiometabolic_II","B13805","20212097_SS211647","PASS",-0.3033,0.4357,"Plate control","PASS","PFC",1.35256694341622,"24h","24h"
6715,"PFC_06",61,"OID30298","Q6UW49","SPESP1",0.0909,"Cardiometabolic_II","B13805","20212097_SS211647","PASS",-0.3033,0.7578,"Plate control","PASS","PFC",1.69091014858581,"Baseline","Baseline"
6716,"PFC_14",62,"OID30298","Q6UW49","SPESP1",0.0909,"Cardiometabolic_II","B13805","20212097_SS211647","PASS",-0.3033,1.1057,"Plate control","PASS","PFC",2.15203270041696,"1h","1h"
6717,"PFC_22",63,"OID30298","Q6UW49","SPESP1",0.0909,"Cardiometabolic_II","B13805","20212097_SS211647","PASS",-0.3033,1.051,"Plate control","PASS","PFC",2.07196552712004,"24h","24h"
6718,"PFC_07",73,"OID30298","Q6UW49","SPESP1",0.0909,"Cardiometabolic_II","B13805","20212097_SS211647","PASS",-0.3033,-0.1373,"Plate control","PASS","PFC",0.909219165331413,"Baseline","Baseline"
6719,"PFC_15",74,"OID30298","Q6UW49","SPESP1",0.0909,"Cardiometabolic_II","B13805","20212097_SS211647","PASS",-0.3033,0.7482,"Plate control","PASS","PFC",1.67969582503379,"1h","1h"
6720,"PFC_23",75,"OID30298","Q6UW49","SPESP1",0.0909,"Cardiometabolic_II","B13805","20212097_SS211647","PASS",-0.3033,1.0174,"Plate control","PASS","PFC",2.02426757039946,"24h","24h"

Supplemental table 1_Olink proximity extension assay (PEA) data

,"SampleID","Index","OlinkID","UniProt","Assay","MissingFreq","Panel","Panel_Lot_Nr","PlateID","QC_Warning","LOD","NPX","Normalization","Assay_Warning","condition","Value","group","alt_group"
6721,"PFC_08",85,"OID30298","Q6UW49","SPESP1",0.0909,"Cardiometabolic_II","B13805","20212097_SS211647","PASS",-0.3033,1.2613,"Plate control","PASS","PFC",2.39711645720998,"Baseline","Baseline"
6722,"PFC_16",86,"OID30298","Q6UW49","SPESP1",0.0909,"Cardiometabolic_II","B13805","20212097_SS211647","PASS",-0.3033,0.1146,"Plate control","PASS","PFC",1.0826748227078,"1h","1h"
6723,"PFC_24",87,"OID30298","Q6UW49","SPESP1",0.0909,"Cardiometabolic_II","B13805","20212097_SS211647","PASS",-0.3033,0.5078,"Plate control","PASS","PFC",1.42188028246193,"24h","24h"
6724,"PFC_01",1,"OID30299","P51511","MMP15",1,"Cardiometabolic_II","B13805","20212097_SS211647","PASS",0.5313,0.0659,"Plate control","PASS","PFC",1.04673772512968,"Baseline","Baseline"
6725,"PFC_09",2,"OID30299","P51511","MMP15",1,"Cardiometabolic_II","B13805","20212097_SS211647","PASS",0.5313,0.1707,"Plate control","PASS","PFC",1.12560449892362,"Baseline","Baseline"
6726,"PFC_17",3,"OID30299","P51511","MMP15",1,"Cardiometabolic_II","B13805","20212097_SS211647","PASS",0.5313,0.303,"Plate control","PASS","PFC",1.2337071698095,"1h","1h"
6727,"PFC_25",4,"OID30299","P51511","MMP15",1,"Cardiometabolic_II","B13805","20212097_SS211647","PASS",0.5313,0.0515,"Plate control","PASS","PFC",1.03634187005067,"24h","24h"
6728,"PFC_02",13,"OID30299","P51511","MMP15",1,"Cardiometabolic_II","B13805","20212097_SS211647","PASS",0.5313,-0.3917,"Plate control","PASS","PFC",0.762230900151113,"Baseline","Baseline"
6729,"PFC_10",14,"OID30299","P51511","MMP15",1,"Cardiometabolic_II","B13805","20212097_SS211647","PASS",0.5313,-0.3574,"Plate control","PASS","PFC",0.780570042632728,"1h","1h"
6730,"PFC_18",15,"OID30299","P51511","MMP15",1,"Cardiometabolic_II","B13805","20212097_SS211647","PASS",0.5313,-0.3054,"Plate control","PASS","PFC",0.809217822620359,"1h","1h"
6731,"PFC_26",16,"OID30299","P51511","MMP15",1,"Cardiometabolic_II","B13805","20212097_SS211647","PASS",0.5313,0.4088,"Plate control","PASS","PFC",1.32758110380523,"24h","24h"
6732,"PFC_03",25,"OID30299","P51511","MMP15",1,"Cardiometabolic_II","B13805","20212097_SS211647","PASS",0.5313,-0.0101,"Plate control","PASS","PFC",0.993023661896327,"Baseline","Baseline"
6733,"PFC_11",26,"OID30299","P51511","MMP15",1,"Cardiometabolic_II","B13805","20212097_SS211647","PASS",0.5313,0.226,"Plate control","PASS","PFC",1.16958766405195,"1h","1h"
6734,"PFC_19",27,"OID30299","P51511","MMP15",1,"Cardiometabolic_II","B13805","20212097_SS211647","PASS",0.5313,-0.0516,"Plate control","PASS","PFC",0.964865665067901,"24h","24h"
6735,"PFC_27",28,"OID30299","P51511","MMP15",1,"Cardiometabolic_II","B13805","20212097_SS211647","PASS",0.5313,0.2164,"Plate control","PASS","PFC",1.16183081517497,"24h","24h"
6736,"PFC_04",37,"OID30299","P51511","MMP15",1,"Cardiometabolic_II","B13805","20212097_SS211647","PASS",0.5313,0.0395,"Plate control","PASS","PFC",1.02775757129386,"Baseline","Baseline"
6737,"PFC_12",38,"OID30299","P51511","MMP15",1,"Cardiometabolic_II","B13805","20212097_SS211647","PASS",0.5313,-0.3195,"Plate control","PASS","PFC",0.801347555368276,"1h","1h"
6738,"PFC_20",39,"OID30299","P51511","MMP15",1,"Cardiometabolic_II","B13805","20212097_SS211647","PASS",0.5313,0.1665,"Plate control","PASS","PFC",1.12233238389855,"24h","24h"
6739,"PFC_05",49,"OID30299","P51511","MMP15",1,"Cardiometabolic_II","B13805","20212097_SS211647","PASS",0.5313,-0.1139,"Plate control","PASS","PFC",0.924086623030708,"Baseline","Baseline"
6740,"PFC_13",50,"OID30299","P51511","MMP15",1,"Cardiometabolic_II","B13805","20212097_SS211647","PASS",0.5313,0.1445,"Plate control","PASS","PFC",1.10534750267665,"1h","1h"
6741,"PFC_21",51,"OID30299","P51511","MMP15",1,"Cardiometabolic_II","B13805","20212097_SS211647","PASS",0.5313,-0.2316,"Plate control","PASS","PFC",0.851689813573411,"24h","24h"
6742,"PFC_06",61,"OID30299","P51511","MMP15",1,"Cardiometabolic_II","B13805","20212097_SS211647","PASS",0.5313,-0.2132,"Plate control","PASS","PFC",0.862621751486302,"Baseline","Baseline"
6743,"PFC_14",62,"OID30299","P51511","MMP15",1,"Cardiometabolic_II","B13805","20212097_SS211647","PASS",0.5313,-0.0415,"Plate control","PASS","PFC",0.97164418340782,"1h","1h"
6744,"PFC_22",63,"OID30299","P51511","MMP15",1,"Cardiometabolic_II","B13805","20212097_SS211647","PASS",0.5313,0.0159,"Plate control","PASS","PFC",1.01108199555891,"24h","24h"
6745,"PFC_07",73,"OID30299","P51511","MMP15",1,"Cardiometabolic_II","B13805","20212097_SS211647","PASS",0.5313,-0.0537,"Plate control","PASS","PFC",0.963462219527479,"Baseline","Baseline"
6746,"PFC_15",74,"OID30299","P51511","MMP15",1,"Cardiometabolic_II","B13805","20212097_SS211647","PASS",0.5313,-0.1646,"Plate control","PASS","PFC",0.89217584880104,"1h","1h"
6747,"PFC_23",75,"OID30299","P51511","MMP15",1,"Cardiometabolic_II","B13805","20212097_SS211647","PASS",0.5313,0.0046,"Plate control","PASS","PFC",1.00319356563032,"24h","24h"
6748,"PFC_08",85,"OID30299","P51511","MMP15",1,"Cardiometabolic_II","B13805","20212097_SS211647","PASS",0.5313,0.075,"Plate control","PASS","PFC",1.05336103595484,"Baseline","Baseline"
6749,"PFC_16",86,"OID30299","P51511","MMP15",1,"Cardiometabolic_II","B13805","20212097_SS211647","PASS",0.5313,0.0948,"Plate control","PASS","PFC",1.06791735300523,"1h","1h"
6750,"PFC_24",87,"OID30299","P51511","MMP15",1,"Cardiometabolic_II","B13805","20212097_SS211647","PASS",0.5313,-0.0534,"Plate control","PASS","PFC",0.963662586695661,"24h","24h"
6751,"PFC_01",1,"OID30300","P08138","NGFR",0.7955,"Cardiometabolic_II","B13805","20212097_SS211647","PASS",0.5796,0.2551,"Plate control","PASS","PFC",1.19341846162907,"Baseline","Baseline"
6752,"PFC_09",2,"OID30300","P08138","NGFR",0.7955,"Cardiometabolic_II","B13805","20212097_SS211647","PASS",0.5796,0.4258,"Plate control","PASS","PFC",1.34331718950818,"Baseline","Baseline"
6753,"PFC_17",3,"OID30300","P08138","NGFR",0.7955,"Cardiometabolic_II","B13805","20212097_SS211647","PASS",0.5796,0.413,"Plate control","PASS","PFC",1.33145161323637,"1h","1h"
6754,"PFC_25",4,"OID30300","P08138","NGFR",0.7955,"Cardiometabolic_II","B13805","20212097_SS211647","PASS",0.5796,0.0308,"Plate control","PASS","PFC",1.02157845205269,"24h","24h"
6755,"PFC_02",13,"OID30300","P08138","NGFR",0.7955,"Cardiometabolic_II","B13805","20212097_SS211647","PASS",0.5796,0.2151,"Plate control","PASS","PFC",1.16078437103667,"Baseline","Baseline"
6756,"PFC_10",14,"OID30300","P08138","NGFR",0.7955,"Cardiometabolic_II","B13805","20212097_SS211647","PASS",0.5796,0.266,"Plate control","PASS","PFC",1.20246924889818,"1h","1h"
6757,"PFC_18",15,"OID30300","P08138","NGFR",0.7955,"Cardiometabolic_II","B13805","20212097_SS211647","PASS",0.5796,1.2752,"Plate control","PASS","PFC",2.42032368329005,"1h","1h"
6758,"PFC_26",16,"OID30300","P08138","NGFR",0.7955,"Cardiometabolic_II","B13805","20212097_SS211647","PASS",0.5796,0.5142,"Plate control","PASS","PFC",1.42820195679394,"24h","24h"
6759,"PFC_03",25,"OID30300","P08138","NGFR",0.7955,"Cardiometabolic_II","B13805","20212097_SS211647","PASS",0.5796,0.0508,"Plate control","PASS","PFC",1.03583915580805,"Baseline","Baseline"
6760,"PFC_11",26,"OID30300","P08138","NGFR",0.7955,"Cardiometabolic_II","B13805","20212097_SS211647","PASS",0.5796,-0.1404,"Plate control","PASS","PFC",0.907267572458037,"1h","1h"
6761,"PFC_19",27,"OID30300","P08138","NGFR",0.7955,"Cardiometabolic_II","B13805","20212097_SS211647","PASS",0.5796,0.3597,"Plate control","PASS","PFC",1.28315904439644,"24h","24h"
6762,"PFC_27",28,"OID30300","P08138","NGFR",0.7955,"Cardiometabolic_II","B13805","20212097_SS211647","PASS",0.5796,0.5383,"Plate control","PASS","PFC",1.45226023753437,"24h","24h"
6763,"PFC_04",37,"OID30300","P08138","NGFR",0.7955,"Cardiometabolic_II","B13805","20212097_SS211647","PASS",0.5796,0.1121,"Plate control","PASS","PFC",1.08080031481214,"Baseline","Baseline"
6764,"PFC_12",38,"OID30300","P08138","NGFR",0.7955,"Cardiometabolic_II","B13805","20212097_SS211647","PASS",0.5796,0.2152,"Plate control","PASS","PFC",1.16086483326665,"1h","1h"
6765,"PFC_20",39,"OID30300","P08138","NGFR",0.7955,"Cardiometabolic_II","B13805","20212097_SS211647","PASS",0.5796,0.6775,"Plate control","PASS","PFC",1.59936586263271,"24h","24h"
6766,"PFC_05",49,"OID30300","P08138","NGFR",0.7955,"Cardiometabolic_II","B13805","20212097_SS211647","PASS",0.5796,0.1496,"Plate control","PASS","PFC",1.1092618767311,"Baseline","Baseline"
6767,"PFC_13",50,"OID30300","P08138","NGFR",0.7955,"Cardiometabolic_II","B13805","20212097_SS211647","PASS",0.5796,0.1224,"Plate control","PASS","PFC",1.0885442083747,"1h","1h"
6768,"PFC_21",51,"OID30300","P08138","NGFR",0.7955,"Cardiometabolic_II","B13805","20212097_SS211647","PASS",0.5796,0.348,"Plate control","PASS","PFC",1.27279493527594,"24h","24h"
6769,"PFC_06",61,"OID30300","P08138","NGFR",0.7955,"Cardiometabolic_II","B13805","20212097_SS211647","PASS",0.5796,-0.5357,"Plate control","PASS","PFC",0.689823890845812,"Baseline","Baseline"
6770,"PFC_14",62,"OID30300","P08138","NGFR",0.7955,"Cardiometabolic_II","B13805","20212097_SS211647","PASS",0.5796,0.4056,"Plate control","PASS","PFC",1.32463969801756,"1h","1h"
6771,"PFC_22",63,"OID30300","P08138","NGFR",0.7955,"Cardiometabolic_II","B13805","20212097_SS211647","PASS",0.5796,0.1406,"Plate control","PASS","PFC",1.10236348063887,"24h","24h"
6772,"PFC_07",73,"OID30300","P08138","NGFR",0.7955,"Cardiometabolic_II","B13805","20212097_SS211647","PASS",0.5796,-0.8844,"Plate control","PASS","PFC",0.541712767927958,"Baseline","Baseline"
6773,"PFC_15",74,"OID30300","P08138","NGFR",0.7955,"Cardiometabolic_II","B13805","20212097_SS211647","PASS",0.5796,0.3527,"Plate control","PASS","PFC",1.27694819767248,"1h","1h"
6774,"PFC_23",75,"OID30300","P08138","NGFR",0.7955,"Cardiometabolic_II","B13805","20212097_SS211647","PASS",0.5796,0.2661,"Plate control","PASS","PFC",1.20255260060385,"24h","24h"
6775,"PFC_08",85,"OID30300","P08138","NGFR",0.7955,"Cardiometabolic_II","B13805","20212097_SS211647","PASS",0.5796,-0.4685,"Plate control","PASS","PFC",0.72271562971345 5,"Baseline","Baseline"
6776,"PFC_16",86,"OID30300","P08138","NGFR",0.7955,"Cardiometabolic_II","B13805","20212097_SS211647","PASS",0.5796,-0.0418,"Plate control","PASS","PFC",0.971442156685815,"1h","1h"
6777,"PFC_24",87,"OID30300","P08138","NGFR",0.7955,"Cardiometabolic_II","B13805","20212097_SS211647","PASS",0.5796,0.1537,"Plate control","PASS","PFC",1.11241877555088,"24h","24h"
6778,"PFC_01",1,"OID30301","P15502","ELN",0,"Cardiometabolic_II","B13805","20212097_SS211647","PASS",-1.9768,0.4408,"Plate control","PASS","PFC",1.35735679722908,"Baseline","Baseline"
6779,"PFC_09",2,"OID30301","P15502","ELN",0,"Cardiometabolic_II","B13805","20212097_SS211647","PASS",-1.9768,0.2996,"Plate control","PASS","PFC",1.23080311494952,"Baseline","Baseline"
6780,"PFC_17",3,"OID30301","P15502","ELN",0,"Cardiometabolic_II","B13805","20212097_SS211647","PASS",-1.9768,-0.0996,"Plate control","PASS","PFC",0.933291719077384,"1h","1h"
6781,"PFC_25",4,"OID30301","P15502","ELN",0,"Cardiometabolic_II","B13805","20212097_SS211647","PASS",-1.9768,0.0289,"Plate control","PASS","PFC",1.02023393956966,"24h","24h"
6782,"PFC_02",13,"OID30301","P15502","ELN",0,"Cardiometabolic_II","B13805","20212097_SS211647","PASS",-1.9768,1.1797,"Plate control","PASS","PFC",2.26529666641238,"Baseline","Baseline"
6783,"PFC_10",14,"OID30301","P15502","ELN",0,"Cardiometabolic_II","B13805","20212097_SS211647","PASS",-1.9768,0.5577,"Plate control","PASS","PFC",1.47192075302649,"1h","1h"
6784,"PFC_18",15,"OID30301","P15502","ELN",0,"Cardiometabolic_II","B13805","20212097_SS211647","PASS",-1.9768,0.4868,"Plate control","PASS","PFC",1.40133316958752,"1h","1h"
6785,"PFC_26",16,"OID30301","P15502","ELN",0,"Cardiometabolic_II","B13805","20212097_SS211647","PASS",-1.9768,-0.0749,"Plate control","PASS","PFC",0.949407926612596,"24h","24h"
6786,"PFC_03",25,"OID30301","P15502","ELN",0,"Cardiometabolic_II","B13805","20212097_SS211647","PASS",-1.9768,-0.6263,"Plate control","PASS","PFC",0.647835755051113,"Baseline","Baseline"
6787,"PFC_11",26,"OID30301","P15502","ELN",0,"Cardiometabolic_II","B13805","20212097_SS211647","PASS",-1.9768,0.5032,"Plate control","PASS","PFC",1.41735386985716,"1h","1h"
6788,"PFC_19",27,"OID30301","P15502","ELN",0,"Cardiometabolic_II","B13805","20212097_SS211647","PASS",-1.9768,0.2615,"Plate control","PASS","PFC",1.19872439557392,"24h","24h"
6789,"PFC_27",28,"OID30301","P15502","ELN",0,"Cardiometabolic_II","B13805","20212097_SS211647","PASS",-1.9768,0.474,"Plate control","PASS","PFC",1.38895513576511,"24h","24h"
6790,"PFC_04",37,"OID30301","P15502","ELN",0,"Cardiometabolic_II","B13805","20212097_SS211647","PASS",-1.9768,1.0616,"Plate control","PASS","PFC",2.08724506739673,"Baseline","Baseline"
6791,"PFC_12",38,"OID30301","P15502","ELN",0,"Cardiometabolic_II","B13805","20212097_SS211647","PASS",-1.9768,-0.5722,"Plate control","PASS","PFC",0.672590356975731,"1h","1h"
6792,"PFC_20",39,"OID30301","P15502","ELN",0,"Cardiometabolic_II","B13805","20212097_SS211647","PASS",-1.9768,0.6369,"Plate control","PASS","PFC",1.55498428473227,"24h","24h"
6793,"PFC_05",49,"OID30301","P15502","ELN",0,"Cardiometabolic_II","B13805","20212097_SS211647","PASS",-1.9768,0.4068,"Plate control","PASS","PFC",1.32574196069849,"Baseline","Baseline"
6794,"PFC_13",50,"OID30301","P15502","ELN",0,"Cardiometabolic_II","B13805","20212097_SS211647","PASS",-1.9768,0.2709,"Plate control","PASS","PFC",1.20656028443629,"1h","1h"
6795,"PFC_21",51,"OID30301","P15502","ELN",0,"Cardiometabolic_II","B13805","20212097_SS211647","PASS",-1.9768,-0.483,"Plate control","PASS","PFC",0.715488260011456,"24h","24h"
6796,"PFC_06",61,"OID30301","P15502","ELN",0,"Cardiometabolic_II","B13805","20212097_SS211647","PASS",-1.9768,0.1947,"Plate control","PASS","PFC",1.14448614664902,"Baseline","Baseline"
6797,"PFC_14",62,"OID30301","P15502","ELN",0,"Cardiometabolic_II","B13805","20212097_SS211647","PASS",-1.9768,0.389,"Plate control","PASS","PFC",1.30948542308357,"1h","1h"
6798,"PFC_22",63,"OID30301","P15502","ELN",0,"Cardiometabolic_II","B13805","20212097_SS211647","PASS",-1.9768,0.6406,"Plate control","PASS","PFC",1.55897738498429,"24h","24h"
6799,"PFC_07",73,"OID30301","P15502","ELN",0,"Cardiometabolic_II","B13805","20212097_SS211647","PASS",-1.9768,0.2493,"Plate control","PASS","PFC",1.18863024807162,"Baseline","Baseline"
6800,"PFC_15",74,"OID30301","P15502","ELN",0,"Cardiometabolic_II","B13805","20212097_SS211647","PASS",-1.9768,-0.091,"Plate control","PASS","PFC",0.938871747315452,"1h","1h"
6801,"PFC_23",75,"OID30301","P15502","ELN",0,"Cardiometabolic_II","B13805","20212097_SS211647","PASS",-1.9768,0.5015,"Plate control","PASS","PFC",1.41568471424935,"24h","24h"
6802,"PFC_08",85,"OID30301","P15502","ELN",0,"Cardiometabolic_II","B13805","20212097_SS211647","PASS",-1.9768,-0.5743,"Plate control","PASS","PFC",0.671612040541431,"Baseline","Baseline"
6803,"PFC_16",86,"OID30301","P15502","ELN",0,"Cardiometabolic_II","B13805","20212097_SS211647","PASS",-1.9768,-0.0745,"Plate control","PASS","PFC",0.949671194878654,"1h","1h"
6804,"PFC_24",87,"OID30301","P15502","ELN",0,"Cardiometabolic_II","B13805","20212097_SS211647","PASS",-1.9768,0.0774,"Plate control","PASS","PFC",1.05511481646124,"24h","24h"
6805,"PFC_01",1,"OID30302","Q8NDI1","EHBP1",0,"Cardiometabolic_II","B13805","20212097_SS211647","PASS",-0.636,1.2781,"Plate control","PASS","PFC",2.42519373391755,"Baseline","Baseline"
6806,"PFC_09",2,"OID30302","Q8NDI1","EHBP1",0,"Cardiometabolic_II","B13805","20212097_SS211647","PASS",-0.636,2.7581,"Plate control","PASS","PFC",6.76504720010838,"Baseline","Baseline"
6807,"PFC_17",3,"OID30302","Q8NDI1","EHBP1",0,"Cardiometabolic_II","B13805","20212097_SS211647","PASS",-0.636,3.0281,"Plate control","PASS","PFC",8.15734686858815,"1h","1h"
6808,"PFC_25",4,"OID30302","Q8NDI1","EHBP1",0,"Cardiometabolic_II","B13805","20212097_SS211647","PASS",-0.636,2.8105,"Plate control","PASS","PFC",7.01527665888157,"24h","24h"
6809,"PFC_02",13,"OID30302","Q8NDI1","EHBP1",0,"Cardiometabolic_II","B13805","20212097_SS211647","PASS",-0.636,1.9797,"Plate control","PASS","PFC",3.94411057795626,"Baseline","Baseline"
6810,"PFC_10",14,"OID30302","Q8NDI1","EHBP1",0,"Cardiometabolic_II","B13805","20212097_SS211647","PASS",-0.636,1.7466,"Plate control","PASS","PFC",3.35566803070123,"1h","1h"
6811,"PFC_18",15,"OID30302","Q8NDI1","EHBP1",0,"Cardiometabolic_II","B13805","20212097_SS211647","PASS",-0.636,3.7129,"Plate control","PASS","PFC",13.1127647831053,"1h","1h"
6812,"PFC_26",16,"OID30302","Q8NDI1","EHBP1",0,"Cardiometabolic_II","B13805","20212097_SS211647","PASS",-0.636,2.8142,"Plate control","PASS","PFC",7.03329144094056,"24h","24h"
6813,"PFC_03",25,"OID30302","Q8NDI1","EHBP1",0,"Cardiometabolic_II","B13805","20212097_SS211647","PASS",-0.636,0.9209,"Plate control","PASS","PFC",1.89329602464498,"Baseline","Baseline"
6814,"PFC_11",26,"OID30302","Q8NDI1","EHBP1",0,"Cardiometabolic_II","B13805","20212097_SS211647","PASS",-0.636,2.9346,"Plate control","PASS","PFC",7.64544248010102,"1h","1h"
6815,"PFC_19",27,"OID30302","Q8NDI1","EHBP1",0,"Cardiometabolic_II","B13805","20212097_SS211647","PASS",-0.636,2.1747,"Plate control","PASS","PFC",4.51491867065937,"24h","24h"
6816,"PFC_27",28,"OID30302","Q8NDI1","EHBP1",0,"Cardiometabolic_II","B13805","20212097_SS211647","PASS",-0.636,3.8168,"Plate control","PASS","PFC",14.0919562906607,"24h","24h"
6817,"PFC_04",37,"OID30302","Q8NDI1","EHBP1",0,"Cardiometabolic_II","B13805","20212097_SS211647","PASS",-0.636,2.9224,"Plate control","PASS","PFC",7.58106219018664,"Baseline","Baseline"
6818,"PFC_12",38,"OID30302","Q8NDI1","EHBP1",0,"Cardiometabolic_II","B13805","20212097_SS211647","PASS",-0.636,0.9782,"Plate control","PASS","PFC",1.97000596767851,"1h","1h"
6819,"PFC_20",39,"OID30302","Q8NDI1","EHBP1",0,"Cardiometabolic_II","B13805","20212097_SS211647","PASS",-0.636,3.288,"Plate control","PASS","PFC",9.76757210135308,"24h","24h"
6820,"PFC_05",49,"OID30302","Q8NDI1","EHBP1",0,"Cardiometabolic_II","B13805","20212097_SS211647","PASS",-0.636,2.0384,"Plate control","PASS","PFC",4.10789697588521,"Baseline","Baseline"
6821,"PFC_13",50,"OID30302","Q8NDI1","EHBP1",0,"Cardiometabolic_II","B13805","20212097_SS211647","PASS",-0.636,2.9469,"Plate control","PASS","PFC",7.71090396426684,"1h","1h"
6822,"PFC_21",51,"OID30302","Q8NDI1","EHBP1",0,"Cardiometabolic_II","B13805","20212097_SS211647","PASS",-0.636,1.691,"Plate control","PASS","PFC",3.22880429801906,"24h","24h"
6823,"PFC_06",61,"OID30302","Q8NDI1","EHBP1",0,"Cardiometabolic_II","B13805","20212097_SS211647","PASS",-0.636,2.4565,"Plate control","PASS","PFC",5.48883511005009,"Baseline","Baseline"
6824,"PFC_14",62,"OID30302","Q8NDI1","EHBP1",0,"Cardiometabolic_II","B13805","20212097_SS211647","PASS",-0.636,3.4891,"Plate control","PASS","PFC",11.2285520852612,"1h","1h"
6825,"PFC_22",63,"OID30302","Q8NDI1","EHBP1",0,"Cardiometabolic_II","B13805","20212097_SS211647","PASS",-0.636,2.7327,"Plate control","PASS","PFC",6.64698454744936,"24h","24h"
6826,"PFC_07",73,"OID30302","Q8NDI1","EHBP1",0,"Cardiometabolic_II","B13805","20212097_SS211647","PASS",-0.636,2.9764,"Plate control","PASS","PFC",7.87019837206191,"Baseline","Baseline"
6827,"PFC_15",74,"OID30302","Q8NDI1","EHBP1",0,"Cardiometabolic_II","B13805","20212097_SS211647","PASS",-0.636,1.8319,"Plate control","PASS","PFC",3.56005616016858,"1h","1h"
6828,"PFC_23",75,"OID30302","Q8NDI1","EHBP1",0,"Cardiometabolic_II","B13805","20212097_SS211647","PASS",-0.636,3.2962,"Plate control","PASS","PFC",9.82324716814838,"24h","24h"
6829,"PFC_08",85,"OID30302","Q8NDI1","EHBP1",0,"Cardiometabolic_II","B13805","20212097_SS211647","PASS",-0.636,1.8681,"Plate control","PASS","PFC",3.65051497982109,"Baseline","Baseline"
6830,"PFC_16",86,"OID30302","Q8NDI1","EHBP1",0,"Cardiometabolic_II","B13805","20212097_SS211647","PASS",-0.636,3.1377,"Plate control","PASS","PFC",8.80119852684727,"1h","1h"
6831,"PFC_24",87,"OID30302","Q8NDI1","EHBP1",0,"Cardiometabolic_II","B13805","20212097_SS211647","PASS",-0.636,3.1321,"Plate control","PASS","PFC",8.76710179971053,"24h","24h"
6832,"PFC_01",1,"OID30303","O43681","GET3",0,"Cardiometabolic_II","B13805","20212097_SS211647","PASS",-0.1928,1.0228,"Plate control","PASS","PFC",2.03185859105151,"Baseline","Baseline"

Supplemental table 1_Olink proximity extension assay (PEA) data

,"SampleID","Index","OlinkID","UniProt","Assay","MissingFreq","Panel","Panel_Lot_Nr","PlateID","QC_Warning","LOD","NPX","Normalization","Assay_Warning","condition","Value","group","alt_group"
6833,"PFC_09",2,"OID30303","O43681","GET3",0,"Cardiometabolic_II","B13805","20212097_SS211647","PASS",-0.1928,1.1885,"Plate control","PASS","PFC",2.27915651303712,"Baseline","Baseline"
6834,"PFC_17",3,"OID30303","O43681","GET3",0,"Cardiometabolic_II","B13805","20212097_SS211647","PASS",-0.1928,2.1069,"Plate control","PASS","PFC",4.30764691109272,"1h","1h"
6835,"PFC_25",4,"OID30303","O43681","GET3",0,"Cardiometabolic_II","B13805","20212097_SS211647","PASS",-0.1928,1.8583,"Plate control","PASS","PFC",3.62580163893853,"24h","24h"
6836,"PFC_02",13,"OID30303","O43681","GET3",0,"Cardiometabolic_II","B13805","20212097_SS211647","PASS",-0.1928,1.7771,"Plate control","PASS","PFC",3.42736537770586,"Baseline","Baseline"
6837,"PFC_10",14,"OID30303","O43681","GET3",0,"Cardiometabolic_II","B13805","20212097_SS211647","PASS",-0.1928,0.92,"Plate control","PASS","PFC",1.89211529345119,"1h","1h"
6838,"PFC_18",15,"OID30303","O43681","GET3",0,"Cardiometabolic_II","B13805","20212097_SS211647","PASS",-0.1928,1.5481,"Plate control","PASS","PFC",2.92431758802436,"1h","1h"
6839,"PFC_26",16,"OID30303","O43681","GET3",0,"Cardiometabolic_II","B13805","20212097_SS211647","PASS",-0.1928,2.139,"Plate control","PASS","PFC",4.40456639239185,"24h","24h"
6840,"PFC_03",25,"OID30303","O43681","GET3",0,"Cardiometabolic_II","B13805","20212097_SS211647","PASS",-0.1928,0.1367,"Plate control","PASS","PFC",1.09938751433695,"Baseline","Baseline"
6841,"PFC_11",26,"OID30303","O43681","GET3",0,"Cardiometabolic_II","B13805","20212097_SS211647","PASS",-0.1928,1.4955,"Plate control","PASS","PFC",2.81961854628963,"1h","1h"
6842,"PFC_19",27,"OID30303","O43681","GET3",0,"Cardiometabolic_II","B13805","20212097_SS211647","PASS",-0.1928,1.4635,"Plate control","PASS","PFC",2.75776590918057,"24h","24h"
6843,"PFC_27",28,"OID30303","O43681","GET3",0,"Cardiometabolic_II","B13805","20212097_SS211647","PASS",-0.1928,2.8925,"Plate control","PASS","PFC",7.4255608691579,"24h","24h"
6844,"PFC_04",37,"OID30303","O43681","GET3",0,"Cardiometabolic_II","B13805","20212097_SS211647","PASS",-0.1928,1.1664,"Plate control","PASS","PFC",2.24450918488374,"Baseline","Baseline"
6845,"PFC_12",38,"OID30303","O43681","GET3",0,"Cardiometabolic_II","B13805","20212097_SS211647","PASS",-0.1928,0.3765,"Plate control","PASS","PFC",1.2981886068697,"1h","1h"
6846,"PFC_20",39,"OID30303","O43681","GET3",0,"Cardiometabolic_II","B13805","20212097_SS211647","PASS",-0.1928,1.6366,"Plate control","PASS","PFC",3.10932193691493,"24h","24h"
6847,"PFC_05",49,"OID30303","O43681","GET3",0,"Cardiometabolic_II","B13805","20212097_SS211647","PASS",-0.1928,0.6068,"Plate control","PASS","PFC",1.5228776094049,"Baseline","Baseline"
6848,"PFC_13",50,"OID30303","O43681","GET3",0,"Cardiometabolic_II","B13805","20212097_SS211647","PASS",-0.1928,1.129,"Plate control","PASS","PFC",2.18707091496926,"1h","1h"
6849,"PFC_21",51,"OID30303","O43681","GET3",0,"Cardiometabolic_II","B13805","20212097_SS211647","PASS",-0.1928,0.9571,"Plate control","PASS","PFC",1.94140350271186,"24h","24h"
6850,"PFC_06",61,"OID30303","O43681","GET3",0,"Cardiometabolic_II","B13805","20212097_SS211647","PASS",-0.1928,0.981,"Plate control","PASS","PFC",1.97383309177168,"Baseline","Baseline"
6851,"PFC_14",62,"OID30303","O43681","GET3",0,"Cardiometabolic_II","B13805","20212097_SS211647","PASS",-0.1928,1.3738,"Plate control","PASS","PFC",2.59152264464052,"1h","1h"
6852,"PFC_22",63,"OID30303","O43681","GET3",0,"Cardiometabolic_II","B13805","20212097_SS211647","PASS",-0.1928,1.34,"Plate control","PASS","PFC",2.53151318794056,"24h","24h"
6853,"PFC_07",73,"OID30303","O43681","GET3",0,"Cardiometabolic_II","B13805","20212097_SS211647","PASS",-0.1928,2.0021,"Plate control","PASS","PFC",4.00582667596913,"Baseline","Baseline"
6854,"PFC_15",74,"OID30303","O43681","GET3",0,"Cardiometabolic_II","B13805","20212097_SS211647","PASS",-0.1928,-0.083,"Plate control","PASS","PFC",0.94409241915025,"1h","1h"
6855,"PFC_23",75,"OID30303","O43681","GET3",0,"Cardiometabolic_II","B13805","20212097_SS211647","PASS",-0.1928,1.6781,"Plate control","PASS","PFC",3.20006231706106,"24h","24h"
6856,"PFC_08",85,"OID30303","O43681","GET3",0,"Cardiometabolic_II","B13805","20212097_SS211647","PASS",-0.1928,0.9045,"Plate control","PASS","PFC",1.87189563284615,"Baseline","Baseline"
6857,"PFC_16",86,"OID30303","O43681","GET3",0,"Cardiometabolic_II","B13805","20212097_SS211647","PASS",-0.1928,1.3938,"Plate control","PASS","PFC",2.62769895262378,"1h","1h"
6858,"PFC_24",87,"OID30303","O43681","GET3",0,"Cardiometabolic_II","B13805","20212097_SS211647","PASS",-0.1928,1.3585,"Plate control","PASS","PFC",2.56418437292618,"24h","24h"
6859,"PFC_01",1,"OID30304","O75223","GGCT",0,"Cardiometabolic_II","B13805","20212097_SS211647","PASS",-0.6264,1.6411,"Plate control","PASS","PFC",3.11903555802303,"Baseline","Baseline"
6860,"PFC_09",2,"OID30304","O75223","GGCT",0,"Cardiometabolic_II","B13805","20212097_SS211647","PASS",-0.6264,1.9457,"Plate control","PASS","PFC",3.85224644066001,"Baseline","Baseline"
6861,"PFC_17",3,"OID30304","O75223","GGCT",0,"Cardiometabolic_II","B13805","20212097_SS211647","PASS",-0.6264,3.3314,"Plate control","PASS","PFC",10.0658702396603,"1h","1h"
6862,"PFC_25",4,"OID30304","O75223","GGCT",0,"Cardiometabolic_II","B13805","20212097_SS211647","PASS",-0.6264,2.5817,"Plate control","PASS","PFC",5.98644697108063,"24h","24h"
6863,"PFC_02",13,"OID30304","O75223","GGCT",0,"Cardiometabolic_II","B13805","20212097_SS211647","PASS",-0.6264,1.4717,"Plate control","PASS","PFC",2.77348514827154,"Baseline","Baseline"
6864,"PFC_10",14,"OID30304","O75223","GGCT",0,"Cardiometabolic_II","B13805","20212097_SS211647","PASS",-0.6264,2.0451,"Plate control","PASS","PFC",4.12701877043396,"1h","1h"
6865,"PFC_18",15,"OID30304","O75223","GGCT",0,"Cardiometabolic_II","B13805","20212097_SS211647","PASS",-0.6264,2.6196,"Plate control","PASS","PFC",6.14579651229898,"1h","1h"
6866,"PFC_26",16,"OID30304","O75223","GGCT",0,"Cardiometabolic_II","B13805","20212097_SS211647","PASS",-0.6264,3.1056,"Plate control","PASS","PFC",8.60753415218688,"24h","24h"
6867,"PFC_03",25,"OID30304","O75223","GGCT",0,"Cardiometabolic_II","B13805","20212097_SS211647","PASS",-0.6264,1.6972,"Plate control","PASS","PFC",3.24270998343976,"Baseline","Baseline"
6868,"PFC_11",26,"OID30304","O75223","GGCT",0,"Cardiometabolic_II","B13805","20212097_SS211647","PASS",-0.6264,2.8381,"Plate control","PASS","PFC",7.15077693576381,"1h","1h"
6869,"PFC_19",27,"OID30304","O75223","GGCT",0,"Cardiometabolic_II","B13805","20212097_SS211647","PASS",-0.6264,2.7996,"Plate control","PASS","PFC",6.96247383109064,"24h","24h"
6870,"PFC_27",28,"OID30304","O75223","GGCT",0,"Cardiometabolic_II","B13805","20212097_SS211647","PASS",-0.6264,3.629,"Plate control","PASS","PFC",12.3719413992851,"24h","24h"
6871,"PFC_04",37,"OID30304","O75223","GGCT",0,"Cardiometabolic_II","B13805","20212097_SS211647","PASS",-0.6264,2.5901,"Plate control","PASS","PFC",6.02140434712422,"Baseline","Baseline"
6872,"PFC_12",38,"OID30304","O75223","GGCT",0,"Cardiometabolic_II","B13805","20212097_SS211647","PASS",-0.6264,1.3815,"Plate control","PASS","PFC",2.60539118246131,"1h","1h"
6873,"PFC_20",39,"OID30304","O75223","GGCT",0,"Cardiometabolic_II","B13805","20212097_SS211647","PASS",-0.6264,2.5892,"Plate control","PASS","PFC",6.01764917104477,"24h","24h"
6874,"PFC_05",49,"OID30304","O75223","GGCT",0,"Cardiometabolic_II","B13805","20212097_SS211647","PASS",-0.6264,1.7387,"Plate control","PASS","PFC",3.33734307147982,"Baseline","Baseline"
6875,"PFC_13",50,"OID30304","O75223","GGCT",0,"Cardiometabolic_II","B13805","20212097_SS211647","PASS",-0.6264,2.7132,"Plate control","PASS","PFC",6.55774589470482,"1h","1h"
6876,"PFC_21",51,"OID30304","O75223","GGCT",0,"Cardiometabolic_II","B13805","20212097_SS211647","PASS",-0.6264,2.0795,"Plate control","PASS","PFC",4.22660707799071,"24h","24h"
6877,"PFC_06",61,"OID30304","O75223","GGCT",0,"Cardiometabolic_II","B13805","20212097_SS211647","PASS",-0.6264,2.4594,"Plate control","PASS","PFC",5.49987946128976,"Baseline","Baseline"
6878,"PFC_14",62,"OID30304","O75223","GGCT",0,"Cardiometabolic_II","B13805","20212097_SS211647","PASS",-0.6264,2.282,"Plate control","PASS","PFC",4.86351713273239,"1h","1h"
6879,"PFC_22",63,"OID30304","O75223","GGCT",0,"Cardiometabolic_II","B13805","20212097_SS211647","PASS",-0.6264,2.0853,"Plate control","PASS","PFC",4.24363331247,"24h","24h"
6880,"PFC_07",73,"OID30304","O75223","GGCT",0,"Cardiometabolic_II","B13805","20212097_SS211647","PASS",-0.6264,3.3506,"Plate control","PASS","PFC",10.2007264993894,"Baseline","Baseline"
6881,"PFC_15",74,"OID30304","O75223","GGCT",0,"Cardiometabolic_II","B13805","20212097_SS211647","PASS",-0.6264,1.4244,"Plate control","PASS","PFC",2.68402851732982,"1h","1h"
6882,"PFC_23",75,"OID30304","O75223","GGCT",0,"Cardiometabolic_II","B13805","20212097_SS211647","PASS",-0.6264,3.0679,"Plate control","PASS","PFC",8.38551854413709,"24h","24h"
6883,"PFC_08",85,"OID30304","O75223","GGCT",0,"Cardiometabolic_II","B13805","20212097_SS211647","PASS",-0.6264,2.4751,"Plate control","PASS","PFC",5.56005825988358,"Baseline","Baseline"
6884,"PFC_16",86,"OID30304","O75223","GGCT",0,"Cardiometabolic_II","B13805","20212097_SS211647","PASS",-0.6264,2.755,"Plate control","PASS","PFC",6.75052636904014,"1h","1h"
6885,"PFC_24",87,"OID30304","O75223","GGCT",0,"Cardiometabolic_II","B13805","20212097_SS211647","PASS",-0.6264,3.0906,"Plate control","PASS","PFC",8.51850346903296,"24h","24h"
6886,"PFC_01",1,"OID30305","O43405","COCH",0,"Cardiometabolic_II","B13805","20212097_SS211647","PASS",-3.9914,1.1075,"Plate control","PASS","PFC",2.15471939183154,"Baseline","Baseline"
6887,"PFC_09",2,"OID30305","O43405","COCH",0,"Cardiometabolic_II","B13805","20212097_SS211647","PASS",-3.9914,0.2682,"Plate control","PASS","PFC",1.20430432168874,"Baseline","Baseline"
6888,"PFC_17",3,"OID30305","O43405","COCH",0,"Cardiometabolic_II","B13805","20212097_SS211647","PASS",-3.9914,0.7941,"Plate control","PASS","PFC",1.73399531426027,"1h","1h"
6889,"PFC_25",4,"OID30305","O43405","COCH",0,"Cardiometabolic_II","B13805","20212097_SS211647","PASS",-3.9914,0.6698,"Plate control","PASS","PFC",1.59085241324955,"24h","24h"
6890,"PFC_02",13,"OID30305","O43405","COCH",0,"Cardiometabolic_II","B13805","20212097_SS211647","PASS",-3.9914,0.4914,"Plate control","PASS","PFC",1.40580841903455,"Baseline","Baseline"
6891,"PFC_10",14,"OID30305","O43405","COCH",0,"Cardiometabolic_II","B13805","20212097_SS211647","PASS",-3.9914,1.1861,"Plate control","PASS","PFC",2.27536816678205,"1h","1h"
6892,"PFC_18",15,"OID30305","O43405","COCH",0,"Cardiometabolic_II","B13805","20212097_SS211647","PASS",-3.9914,1.0238,"Plate control","PASS","PFC",2.03326745632428,"1h","1h"
6893,"PFC_26",16,"OID30305","O43405","COCH",0,"Cardiometabolic_II","B13805","20212097_SS211647","PASS",-3.9914,0.7498,"Plate control","PASS","PFC",1.68155970067538,"24h","24h"
6894,"PFC_03",25,"OID30305","O43405","COCH",0,"Cardiometabolic_II","B13805","20212097_SS211647","PASS",-3.9914,0.144,"Plate control","PASS","PFC",1.10496448479991,"Baseline","Baseline"
6895,"PFC_11",26,"OID30305","O43405","COCH",0,"Cardiometabolic_II","B13805","20212097_SS211647","PASS",-3.9914,0.7559,"Plate control","PASS","PFC",1.68868472008554,"1h","1h"
6896,"PFC_19",27,"OID30305","O43405","COCH",0,"Cardiometabolic_II","B13805","20212097_SS211647","PASS",-3.9914,1.2045,"Plate control","PASS","PFC",2.30457385074329,"24h","24h"
6897,"PFC_27",28,"OID30305","O43405","COCH",0,"Cardiometabolic_II","B13805","20212097_SS211647","PASS",-3.9914,0.8046,"Plate control","PASS","PFC",1.74666144730905,"24h","24h"
6898,"PFC_04",37,"OID30305","O43405","COCH",0,"Cardiometabolic_II","B13805","20212097_SS211647","PASS",-3.9914,0.0653,"Plate control","PASS","PFC",1.04630248965885,"Baseline","Baseline"
6899,"PFC_12",38,"OID30305","O43405","COCH",0,"Cardiometabolic_II","B13805","20212097_SS211647","PASS",-3.9914,0.0114,"Plate control","PASS","PFC",1.00793318008971,"1h","1h"
6900,"PFC_20",39,"OID30305","O43405","COCH",0,"Cardiometabolic_II","B13805","20212097_SS211647","PASS",-3.9914,0.8621,"Plate control","PASS","PFC",1.81768222066123,"24h","24h"
6901,"PFC_05",49,"OID30305","O43405","COCH",0,"Cardiometabolic_II","B13805","20212097_SS211647","PASS",-3.9914,0.3545,"Plate control","PASS","PFC",1.2785423954553,"Baseline","Baseline"
6902,"PFC_13",50,"OID30305","O43405","COCH",0,"Cardiometabolic_II","B13805","20212097_SS211647","PASS",-3.9914,0.0421,"Plate control","PASS","PFC",1.02961144818789,"1h","1h"
6903,"PFC_21",51,"OID30305","O43405","COCH",0,"Cardiometabolic_II","B13805","20212097_SS211647","PASS",-3.9914,0.2245,"Plate control","PASS","PFC",1.16837225141868,"24h","24h"
6904,"PFC_06",61,"OID30305","O43405","COCH",0,"Cardiometabolic_II","B13805","20212097_SS211647","PASS",-3.9914,0.2659,"Plate control","PASS","PFC",1.2023859029698,"Baseline","Baseline"
6905,"PFC_14",62,"OID30305","O43405","COCH",0,"Cardiometabolic_II","B13805","20212097_SS211647","PASS",-3.9914,0.7692,"Plate control","PASS","PFC",1.70432444331006,"1h","1h"
6906,"PFC_22",63,"OID30305","O43405","COCH",0,"Cardiometabolic_II","B13805","20212097_SS211647","PASS",-3.9914,0.0524,"Plate control","PASS","PFC",1.0369885754484,"24h","24h"
6907,"PFC_07",73,"OID30305","O43405","COCH",0,"Cardiometabolic_II","B13805","20212097_SS211647","PASS",-3.9914,0.9232,"Plate control","PASS","PFC",1.89631679736462,"Baseline","Baseline"
6908,"PFC_15",74,"OID30305","O43405","COCH",0,"Cardiometabolic_II","B13805","20212097_SS211647","PASS",-3.9914,-0.1107,"Plate control","PASS","PFC",0.926138587610481,"1h","1h"
6909,"PFC_23",75,"OID30305","O43405","COCH",0,"Cardiometabolic_II","B13805","20212097_SS211647","PASS",-3.9914,0.5544,"Plate control","PASS","PFC",1.46855775025911,"24h","24h"
6910,"PFC_08",85,"OID30305","O43405","COCH",0,"Cardiometabolic_II","B13805","20212097_SS211647","PASS",-3.9914,0.013,"Plate control","PASS","PFC",1.00905163384468,"Baseline","Baseline"
6911,"PFC_16",86,"OID30305","O43405","COCH",0,"Cardiometabolic_II","B13805","20212097_SS211647","PASS",-3.9914,0.7446,"Plate control","PASS","PFC",1.6755096550123,"1h","1h"
6912,"PFC_24",87,"OID30305","O43405","COCH",0,"Cardiometabolic_II","B13805","20212097_SS211647","PASS",-3.9914,0.2123,"Plate control","PASS","PFC",1.15853369145698,"24h","24h"
6913,"PFC_01",1,"OID30306","Q9Y2E5","MAN2B2",0,"Cardiometabolic_II","B13805","20212097_SS211647","PASS",-4.5568,1.0674,"Plate control","PASS","PFC",2.09565321210417,"Baseline","Baseline"
6914,"PFC_09",2,"OID30306","Q9Y2E5","MAN2B2",0,"Cardiometabolic_II","B13805","20212097_SS211647","PASS",-4.5568,0.172,"Plate control","PASS","PFC",1.12661922849728,"Baseline","Baseline"
6915,"PFC_17",3,"OID30306","Q9Y2E5","MAN2B2",0,"Cardiometabolic_II","B13805","20212097_SS211647","PASS",-4.5568,-1.3169,"Plate control","PASS","PFC",0.401396516080079,"1h","1h"
6916,"PFC_25",4,"OID30306","Q9Y2E5","MAN2B2",0,"Cardiometabolic_II","B13805","20212097_SS211647","PASS",-4.5568,0.4073,"Plate control","PASS","PFC",1.32620150747839,"24h","24h"
6917,"PFC_02",13,"OID30306","Q9Y2E5","MAN2B2",0,"Cardiometabolic_II","B13805","20212097_SS211647","PASS",-4.5568,0.4464,"Plate control","PASS","PFC",1.36263578513177,"Baseline","Baseline"
6918,"PFC_10",14,"OID30306","Q9Y2E5","MAN2B2",0,"Cardiometabolic_II","B13805","20212097_SS211647","PASS",-4.5568,1.1921,"Plate control","PASS","PFC",2.2848508620017,"1h","1h"
6919,"PFC_18",15,"OID30306","Q9Y2E5","MAN2B2",0,"Cardiometabolic_II","B13805","20212097_SS211647","PASS",-4.5568,0.2487,"Plate control","PASS","PFC",1.18813601342884,"1h","1h"
6920,"PFC_26",16,"OID30306","Q9Y2E5","MAN2B2",0,"Cardiometabolic_II","B13805","20212097_SS211647","PASS",-4.5568,-1.1961,"Plate control","PASS","PFC",0.436453541864181,"24h","24h"
6921,"PFC_03",25,"OID30306","Q9Y2E5","MAN2B2",0,"Cardiometabolic_II","B13805","20212097_SS211647","PASS",-4.5568,-0.4775,"Plate control","PASS","PFC",0.718221128657936,"Baseline","Baseline"
6922,"PFC_11",26,"OID30306","Q9Y2E5","MAN2B2",0,"Cardiometabolic_II","B13805","20212097_SS211647","PASS",-4.5568,0.445,"Plate control","PASS","PFC",1.36131411649947,"1h","1h"
6923,"PFC_19",27,"OID30306","Q9Y2E5","MAN2B2",0,"Cardiometabolic_II","B13805","20212097_SS211647","PASS",-4.5568,1.297,"Plate control","PASS","PFC",2.4571739608913,"24h","24h"
6924,"PFC_27",28,"OID30306","Q9Y2E5","MAN2B2",0,"Cardiometabolic_II","B13805","20212097_SS211647","PASS",-4.5568,0.6062,"Plate control","PASS","PFC",1.52224439409467,"24h","24h"
6925,"PFC_04",37,"OID30306","Q9Y2E5","MAN2B2",0,"Cardiometabolic_II","B13805","20212097_SS211647","PASS",-4.5568,-0.8551,"Plate control","PASS","PFC",0.552827006283992,"Baseline","Baseline"
6926,"PFC_12",38,"OID30306","Q9Y2E5","MAN2B2",0,"Cardiometabolic_II","B13805","20212097_SS211647","PASS",-4.5568,-0.537,"Plate control","PASS","PFC",0.689202576487815,"1h","1h"
6927,"PFC_20",39,"OID30306","Q9Y2E5","MAN2B2",0,"Cardiometabolic_II","B13805","20212097_SS211647","PASS",-4.5568,0.5594,"Plate control","PASS","PFC",1.47365621343969,"24h","24h"
6928,"PFC_05",49,"OID30306","Q9Y2E5","MAN2B2",0,"Cardiometabolic_II","B13805","20212097_SS211647","PASS",-4.5568,1.0846,"Plate control","PASS","PFC",2.12078739409269,"Baseline","Baseline"
6929,"PFC_13",50,"OID30306","Q9Y2E5","MAN2B2",0,"Cardiometabolic_II","B13805","20212097_SS211647","PASS",-4.5568,-0.8427,"Plate control","PASS","PFC",0.557599046771324,"1h","1h"
6930,"PFC_21",51,"OID30306","Q9Y2E5","MAN2B2",0,"Cardiometabolic_II","B13805","20212097_SS211647","PASS",-4.5568,-0.4461,"Plate control","PASS","PFC",0.734024437556754,"24h","24h"
6931,"PFC_06",61,"OID30306","Q9Y2E5","MAN2B2",0,"Cardiometabolic_II","B13805","20212097_SS211647","PASS",-4.5568,-0.0378,"Plate control","PASS","PFC",0.974139303565458,"Baseline","Baseline"
6932,"PFC_14",62,"OID30306","Q9Y2E5","MAN2B2",0,"Cardiometabolic_II","B13805","20212097_SS211647","PASS",-4.5568,1.1414,"Plate control","PASS","PFC",2.20594986052775,"1h","1h"
6933,"PFC_22",63,"OID30306","Q9Y2E5","MAN2B2",0,"Cardiometabolic_II","B13805","20212097_SS211647","PASS",-4.5568,-0.6905,"Plate control","PASS","PFC",0.619639062217292,"24h","24h"
6934,"PFC_07",73,"OID30306","Q9Y2E5","MAN2B2",0,"Cardiometabolic_II","B13805","20212097_SS211647","PASS",-4.5568,0.2933,"Plate control","PASS","PFC",1.22544012852303,"Baseline","Baseline"
6935,"PFC_15",74,"OID30306","Q9Y2E5","MAN2B2",0,"Cardiometabolic_II","B13805","20212097_SS211647","PASS",-4.5568,-0.0621,"Plate control","PASS","PFC",0.957868821514291,"1h","1h"
6936,"PFC_23",75,"OID30306","Q9Y2E5","MAN2B2",0,"Cardiometabolic_II","B13805","20212097_SS211647","PASS",-4.5568,1.1944,"Plate control","PASS","PFC",2.28849636437562,"24h","24h"
6937,"PFC_08",85,"OID30306","Q9Y2E5","MAN2B2",0,"Cardiometabolic_II","B13805","20212097_SS211647","PASS",-4.5568,-1.5715,"Plate control","PASS","PFC",0.33645838951827,"Baseline","Baseline"
6938,"PFC_16",86,"OID30306","Q9Y2E5","MAN2B2",0,"Cardiometabolic_II","B13805","20212097_SS211647","PASS",-4.5568,0.4843,"Plate control","PASS","PFC",1.39890694701751,"1h","1h"
6939,"PFC_24",87,"OID30306","Q9Y2E5","MAN2B2",0,"Cardiometabolic_II","B13805","20212097_SS211647","PASS",-4.5568,0.1046,"Plate control","PASS","PFC",1.07519624142974,"24h","24h"
6940,"PFC_01",1,"OID30307","P02008","HBZ",0,"Cardiometabolic_II","B13805","20212097_SS211647","PASS",-0.892,-0.629,"Plate control","PASS","PFC",0.646624465943743,"Baseline","Baseline"
6941,"PFC_09",2,"OID30307","P02008","HBZ",0,"Cardiometabolic_II","B13805","20212097_SS211647","PASS",-0.892,2.1874,"Plate control","PASS","PFC",4.55483881071663,"Baseline","Baseline"
6942,"PFC_17",3,"OID30307","P02008","HBZ",0,"Cardiometabolic_II","B13805","20212097_SS211647","PASS",-0.892,1.1034,"Plate control","PASS","PFC",2.1486045803464,"1h","1h"
6943,"PFC_25",4,"OID30307","P02008","HBZ",0,"Cardiometabolic_II","B13805","20212097_SS211647","PASS",-0.892,-0.4946,"Plate control","PASS","PFC",0.709758437646239,"24h","24h"
6944,"PFC_02",13,"OID30307","P02008","HBZ",0,"Cardiometabolic_II","B13805","20212097_SS211647","PASS",-0.892,1.5301,"Plate control","PASS","PFC",2.88805856907271,"Baseline","Baseline"

Supplemental table 1_Olink proximity extension assay (PEA) data

,"SampleID","Index","OlinkID","UniProt","Assay","MissingFreq","Panel","Panel_Lot_Nr","PlateID","QC_Warning","LOD","NPX","Normalization","Assay_Warning","condition","Value","group","alt_group"
6945,"PFC_10",14,"OID30307","P02008","HBZ",0,"Cardiometabolic_II","B13805","20212097_SS211647","PASS",-0.892,-0.8841,"Plate control","PASS","PFC",0.541825425644138,"1h","1h"
6946,"PFC_18",15,"OID30307","P02008","HBZ",0,"Cardiometabolic_II","B13805","20212097_SS211647","PASS",-0.892,1.5586,"Plate control","PASS","PFC",2.94567854289095,"1h","1h"
6947,"PFC_26",16,"OID30307","P02008","HBZ",0,"Cardiometabolic_II","B13805","20212097_SS211647","PASS",-0.892,0.8546,"Plate control","PASS","PFC",1.80825733022507,"24h","24h"
6948,"PFC_03",25,"OID30307","P02008","HBZ",0,"Cardiometabolic_II","B13805","20212097_SS211647","PASS",-0.892,-0.25,"Plate control","PASS","PFC",0.840896415253715,"Baseline","Baseline"
6949,"PFC_11",26,"OID30307","P02008","HBZ",0,"Cardiometabolic_II","B13805","20212097_SS211647","PASS",-0.892,1.1576,"Plate control","PASS","PFC",2.23086003315925,"1h","1h"
6950,"PFC_19",27,"OID30307","P02008","HBZ",0,"Cardiometabolic_II","B13805","20212097_SS211647","PASS",-0.892,-0.7962,"Plate control","PASS","PFC",0.57586398394154,"24h","24h"
6951,"PFC_27",28,"OID30307","P02008","HBZ",0,"Cardiometabolic_II","B13805","20212097_SS211647","PASS",-0.892,2.2282,"Plate control","PASS","PFC",4.6854902267222,"24h","24h"
6952,"PFC_04",37,"OID30307","P02008","HBZ",0,"Cardiometabolic_II","B13805","20212097_SS211647","PASS",-0.892,-0.3914,"Plate control","PASS","PFC",0.762389418091795,"Baseline","Baseline"
6953,"PFC_12",38,"OID30307","P02008","HBZ",0,"Cardiometabolic_II","B13805","20212097_SS211647","PASS",-0.892,0.2952,"Plate control","PASS","PFC",1.22705507141613,"1h","1h"
6954,"PFC_20",39,"OID30307","P02008","HBZ",0,"Cardiometabolic_II","B13805","20212097_SS211647","PASS",-0.892,0.8714,"Plate control","PASS","PFC",1.82943733642231,"24h","24h"
6955,"PFC_05",49,"OID30307","P02008","HBZ",0,"Cardiometabolic_II","B13805","20212097_SS211647","PASS",-0.892,1.5903,"Plate control","PASS","PFC",3.01111957443809,"Baseline","Baseline"
6956,"PFC_13",50,"OID30307","P02008","HBZ",0,"Cardiometabolic_II","B13805","20212097_SS211647","PASS",-0.892,-0.469,"Plate control","PASS","PFC",0.722465198961763,"1h","1h"
6957,"PFC_21",51,"OID30307","P02008","HBZ",0,"Cardiometabolic_II","B13805","20212097_SS211647","PASS",-0.892,-0.073,"Plate control","PASS","PFC",0.950659101229818,"24h","24h"
6958,"PFC_06",61,"OID30307","P02008","HBZ",0,"Cardiometabolic_II","B13805","20212097_SS211647","PASS",-0.892,1.0541,"Plate control","PASS","PFC",2.07642246273568,"Baseline","Baseline"
6959,"PFC_14",62,"OID30307","P02008","HBZ",0,"Cardiometabolic_II","B13805","20212097_SS211647","PASS",-0.892,1.5932,"Plate control","PASS","PFC",3.01717839412167,"1h","1h"
6960,"PFC_22",63,"OID30307","P02008","HBZ",0,"Cardiometabolic_II","B13805","20212097_SS211647","PASS",-0.892,-0.4587,"Plate control","PASS","PFC",0.727641634910885,"24h","24h"
6961,"PFC_07",73,"OID30307","P02008","HBZ",0,"Cardiometabolic_II","B13805","20212097_SS211647","PASS",-0.892,-0.4237,"Plate control","PASS","PFC",0.74551020177069,"Baseline","Baseline"
6962,"PFC_15",74,"OID30307","P02008","HBZ",0,"Cardiometabolic_II","B13805","20212097_SS211647","PASS",-0.892,0.2345,"Plate control","PASS","PFC",1.17649892309831,"1h","1h"
6963,"PFC_23",75,"OID30307","P02008","HBZ",0,"Cardiometabolic_II","B13805","20212097_SS211647","PASS",-0.892,2.069,"Plate control","PASS","PFC",4.19595731029978,"24h","24h"
6964,"PFC_08",85,"OID30307","P02008","HBZ",0,"Cardiometabolic_II","B13805","20212097_SS211647","PASS",-0.892,0.6391,"Plate control","PASS","PFC",1.5573573261662,"Baseline","Baseline"
6965,"PFC_16",86,"OID30307","P02008","HBZ",0,"Cardiometabolic_II","B13805","20212097_SS211647","PASS",-0.892,-0.4112,"Plate control","PASS","PFC",0.751997619462076,"1h","1h"
6966,"PFC_24",87,"OID30307","P02008","HBZ",0,"Cardiometabolic_II","B13805","20212097_SS211647","PASS",-0.892,0.9907,"Plate control","PASS","PFC",1.98714892767623,"24h","24h"
6967,"PFC_01",1,"OID30308","P23919","DTYMK",0,"Cardiometabolic_II","B13805","20212097_SS211647","PASS",-1.3526,2.854,"Plate control","PASS","PFC",7.23002181400869,"Baseline","Baseline"
6968,"PFC_09",2,"OID30308","P23919","DTYMK",0,"Cardiometabolic_II","B13805","20212097_SS211647","PASS",-1.3526,3.6138,"Plate control","PASS","PFC",12.2422769009956,"Baseline","Baseline"
6969,"PFC_17",3,"OID30308","P23919","DTYMK",0,"Cardiometabolic_II","B13805","20212097_SS211647","PASS",-1.3526,4.4582,"Plate control","PASS","PFC",21.9812267687368,"1h","1h"
6970,"PFC_25",4,"OID30308","P23919","DTYMK",0,"Cardiometabolic_II","B13805","20212097_SS211647","PASS",-1.3526,4.0717,"Plate control","PASS","PFC",16.8152695448229,"24h","24h"
6971,"PFC_02",13,"OID30308","P23919","DTYMK",0,"Cardiometabolic_II","B13805","20212097_SS211647","PASS",-1.3526,2.9262,"Plate control","PASS","PFC",7.60105672017042,"Baseline","Baseline"
6972,"PFC_10",14,"OID30308","P23919","DTYMK",0,"Cardiometabolic_II","B13805","20212097_SS211647","PASS",-1.3526,3.2787,"Plate control","PASS","PFC",9.70481021360203,"1h","1h"
6973,"PFC_18",15,"OID30308","P23919","DTYMK",0,"Cardiometabolic_II","B13805","20212097_SS211647","PASS",-1.3526,4.1166,"Plate control","PASS","PFC",17.3468283126259,"1h","1h"
6974,"PFC_26",16,"OID30308","P23919","DTYMK",0,"Cardiometabolic_II","B13805","20212097_SS211647","PASS",-1.3526,4.5132,"Plate control","PASS","PFC",22.8353975303525,"24h","24h"
6975,"PFC_03",25,"OID30308","P23919","DTYMK",0,"Cardiometabolic_II","B13805","20212097_SS211647","PASS",-1.3526,1.3835,"Plate control","PASS","PFC",2.60900552625945,"Baseline","Baseline"
6976,"PFC_11",26,"OID30308","P23919","DTYMK",0,"Cardiometabolic_II","B13805","20212097_SS211647","PASS",-1.3526,4.2738,"Plate control","PASS","PFC",19.3438090061146,"1h","1h"
6977,"PFC_19",27,"OID30308","P23919","DTYMK",0,"Cardiometabolic_II","B13805","20212097_SS211647","PASS",-1.3526,3.8112,"Plate control","PASS","PFC",14.0373626365123,"24h","24h"
6978,"PFC_27",28,"OID30308","P23919","DTYMK",0,"Cardiometabolic_II","B13805","20212097_SS211647","PASS",-1.3526,5.0887,"Plate control","PASS","PFC",34.0291686301193,"24h","24h"
6979,"PFC_04",37,"OID30308","P23919","DTYMK",0,"Cardiometabolic_II","B13805","20212097_SS211647","PASS",-1.3526,3.9735,"Plate control","PASS","PFC",15.7087883295181,"Baseline","Baseline"
6980,"PFC_12",38,"OID30308","P23919","DTYMK",0,"Cardiometabolic_II","B13805","20212097_SS211647","PASS",-1.3526,2.314,"Plate control","PASS","PFC",4.97259867561576,"1h","1h"
6981,"PFC_20",39,"OID30308","P23919","DTYMK",0,"Cardiometabolic_II","B13805","20212097_SS211647","PASS",-1.3526,4.6051,"Plate control","PASS","PFC",24.3373469297334,"24h","24h"
6982,"PFC_05",49,"OID30308","P23919","DTYMK",0,"Cardiometabolic_II","B13805","20212097_SS211647","PASS",-1.3526,2.7528,"Plate control","PASS","PFC",6.74024017556005,"Baseline","Baseline"
6983,"PFC_13",50,"OID30308","P23919","DTYMK",0,"Cardiometabolic_II","B13805","20212097_SS211647","PASS",-1.3526,4.17,"Plate control","PASS","PFC",18.000935755021,"1h","1h"
6984,"PFC_21",51,"OID30308","P23919","DTYMK",0,"Cardiometabolic_II","B13805","20212097_SS211647","PASS",-1.3526,2.7725,"Plate control","PASS","PFC",6.83290941076242,"24h","24h"
6985,"PFC_06",61,"OID30308","P23919","DTYMK",0,"Cardiometabolic_II","B13805","20212097_SS211647","PASS",-1.3526,3.9728,"Plate control","PASS","PFC",15.7011682266777,"Baseline","Baseline"
6986,"PFC_14",62,"OID30308","P23919","DTYMK",0,"Cardiometabolic_II","B13805","20212097_SS211647","PASS",-1.3526,3.9216,"Plate control","PASS","PFC",15.1537190440303,"1h","1h"
6987,"PFC_22",63,"OID30308","P23919","DTYMK",0,"Cardiometabolic_II","B13805","20212097_SS211647","PASS",-1.3526,3.8056,"Plate control","PASS","PFC",13.9829804836639,"24h","24h"
6988,"PFC_07",73,"OID30308","P23919","DTYMK",0,"Cardiometabolic_II","B13805","20212097_SS211647","PASS",-1.3526,4.4377,"Plate control","PASS","PFC",21.6710927941351,"Baseline","Baseline"
6989,"PFC_15",74,"OID30308","P23919","DTYMK",0,"Cardiometabolic_II","B13805","20212097_SS211647","PASS",-1.3526,2.6678,"Plate control","PASS","PFC",6.35459420589285,"1h","1h"
6990,"PFC_23",75,"OID30308","P23919","DTYMK",0,"Cardiometabolic_II","B13805","20212097_SS211647","PASS",-1.3526,4.3241,"Plate control","PASS","PFC",20.0301316732769,"24h","24h"
6991,"PFC_08",85,"OID30308","P23919","DTYMK",0,"Cardiometabolic_II","B13805","20212097_SS211647","PASS",-1.3526,3.4019,"Plate control","PASS","PFC",10.5699745650668,"Baseline","Baseline"
6992,"PFC_16",86,"OID30308","P23919","DTYMK",0,"Cardiometabolic_II","B13805","20212097_SS211647","PASS",-1.3526,3.8909,"Plate control","PASS","PFC",14.8346604470234,"1h","1h"
6993,"PFC_24",87,"OID30308","P23919","DTYMK",0,"Cardiometabolic_II","B13805","20212097_SS211647","PASS",-1.3526,4.4205,"Plate control","PASS","PFC",21.4142612080481,"24h","24h"
6994,"PFC_01",1,"OID30309","Q7Z304","MAMDC2",0,"Cardiometabolic_II","B13805","20212097_SS211647","PASS",-2.948,-0.8133,"Plate control","PASS","PFC",0.569078665651102,"Baseline","Baseline"
6995,"PFC_09",2,"OID30309","Q7Z304","MAMDC2",0,"Cardiometabolic_II","B13805","20212097_SS211647","PASS",-2.948,-0.6222,"Plate control","PASS","PFC",0.649679460287389,"Baseline","Baseline"
6996,"PFC_17",3,"OID30309","Q7Z304","MAMDC2",0,"Cardiometabolic_II","B13805","20212097_SS211647","PASS",-2.948,-1.5806,"Plate control","PASS","PFC",0.334342810512169,"1h","1h"
6997,"PFC_25",4,"OID30309","Q7Z304","MAMDC2",0,"Cardiometabolic_II","B13805","20212097_SS211647","PASS",-2.948,-1.0098,"Plate control","PASS","PFC",0.496615088416406,"24h","24h"
6998,"PFC_02",13,"OID30309","Q7Z304","MAMDC2",0,"Cardiometabolic_II","B13805","20212097_SS211647","PASS",-2.948,-0.2288,"Plate control","PASS","PFC",0.853344388564005,"Baseline","Baseline"
6999,"PFC_10",14,"OID30309","Q7Z304","MAMDC2",0,"Cardiometabolic_II","B13805","20212097_SS211647","PASS",-2.948,-0.7106,"Plate control","PASS","PFC",0.611065950808218,"1h","1h"
7000,"PFC_18",15,"OID30309","Q7Z304","MAMDC2",0,"Cardiometabolic_II","B13805","20212097_SS211647","PASS",-2.948,-0.5076,"Plate control","PASS","PFC",0.703391594483547,"1h","1h"
7001,"PFC_26",16,"OID30309","Q7Z304","MAMDC2",0,"Cardiometabolic_II","B13805","20212097_SS211647","PASS",-2.948,-1.0824,"Plate control","PASS","PFC",0.472242568903626,"24h","24h"
7002,"PFC_03",25,"OID30309","Q7Z304","MAMDC2",0,"Cardiometabolic_II","B13805","20212097_SS211647","PASS",-2.948,-0.8019,"Plate control","PASS","PFC",0.573593269190924,"Baseline","Baseline"
7003,"PFC_11",26,"OID30309","Q7Z304","MAMDC2",0,"Cardiometabolic_II","B13805","20212097_SS211647","PASS",-2.948,-0.4066,"Plate control","PASS","PFC",0.754399173213672,"1h","1h"
7004,"PFC_19",27,"OID30309","Q7Z304","MAMDC2",0,"Cardiometabolic_II","B13805","20212097_SS211647","PASS",-2.948,-0.5144,"Plate control","PASS","PFC",0.700084029024167,"24h","24h"
7005,"PFC_27",28,"OID30309","Q7Z304","MAMDC2",0,"Cardiometabolic_II","B13805","20212097_SS211647","PASS",-2.948,0.1639,"Plate control","PASS","PFC",1.12031155742309,"24h","24h"
7006,"PFC_04",37,"OID30309","Q7Z304","MAMDC2",0,"Cardiometabolic_II","B13805","20212097_SS211647","PASS",-2.948,-0.2543,"Plate control","PASS","PFC",0.838393827218016,"Baseline","Baseline"
7007,"PFC_12",38,"OID30309","Q7Z304","MAMDC2",0,"Cardiometabolic_II","B13805","20212097_SS211647","PASS",-2.948,-1.0266,"Plate control","PASS","PFC",0.490865609906871,"1h","1h"
7008,"PFC_20",39,"OID30309","Q7Z304","MAMDC2",0,"Cardiometabolic_II","B13805","20212097_SS211647","PASS",-2.948,-0.4083,"Plate control","PASS","PFC",0.753510750331234,"24h","24h"
7009,"PFC_05",49,"OID30309","Q7Z304","MAMDC2",0,"Cardiometabolic_II","B13805","20212097_SS211647","PASS",-2.948,-1.3057,"Plate control","PASS","PFC",0.404524783999543,"Baseline","Baseline"
7010,"PFC_13",50,"OID30309","Q7Z304","MAMDC2",0,"Cardiometabolic_II","B13805","20212097_SS211647","PASS",-2.948,-0.6691,"Plate control","PASS","PFC",0.628898892457776,"1h","1h"
7011,"PFC_21",51,"OID30309","Q7Z304","MAMDC2",0,"Cardiometabolic_II","B13805","20212097_SS211647","PASS",-2.948,-1.0888,"Plate control","PASS","PFC",0.470152273681638,"24h","24h"
7012,"PFC_06",61,"OID30309","Q7Z304","MAMDC2",0,"Cardiometabolic_II","B13805","20212097_SS211647","PASS",-2.948,-1.6856,"Plate control","PASS","PFC",0.310873597741391,"Baseline","Baseline"
7013,"PFC_14",62,"OID30309","Q7Z304","MAMDC2",0,"Cardiometabolic_II","B13805","20212097_SS211647","PASS",-2.948,-0.9595,"Plate control","PASS","PFC",0.514235102754185,"1h","1h"
7014,"PFC_22",63,"OID30309","Q7Z304","MAMDC2",0,"Cardiometabolic_II","B13805","20212097_SS211647","PASS",-2.948,-0.3583,"Plate control","PASS","PFC",0.780083249555324,"24h","24h"
7015,"PFC_07",73,"OID30309","Q7Z304","MAMDC2",0,"Cardiometabolic_II","B13805","20212097_SS211647","PASS",-2.948,-1.196,"Plate control","PASS","PFC",0.436483795566881,"Baseline","Baseline"
7016,"PFC_15",74,"OID30309","Q7Z304","MAMDC2",0,"Cardiometabolic_II","B13805","20212097_SS211647","PASS",-2.948,-1.2639,"Plate control","PASS","PFC",0.416416748249453,"1h","1h"
7017,"PFC_23",75,"OID30309","Q7Z304","MAMDC2",0,"Cardiometabolic_II","B13805","20212097_SS211647","PASS",-2.948,-0.6633,"Plate control","PASS","PFC",0.631432314611567,"24h","24h"
7018,"PFC_08",85,"OID30309","Q7Z304","MAMDC2",0,"Cardiometabolic_II","B13805","20212097_SS211647","PASS",-2.948,-1.2696,"Plate control","PASS","PFC",0.41477475694765,"Baseline","Baseline"
7019,"PFC_16",86,"OID30309","Q7Z304","MAMDC2",0,"Cardiometabolic_II","B13805","20212097_SS211647","PASS",-2.948,-0.8172,"Plate control","PASS","PFC",0.56754236754085,"1h","1h"
7020,"PFC_24",87,"OID30309","Q7Z304","MAMDC2",0,"Cardiometabolic_II","B13805","20212097_SS211647","PASS",-2.948,-1.001,"Plate control","PASS","PFC",0.499653546495226,"24h","24h"
7021,"PFC_01",1,"OID30310","P78539","SRPX",0,"Cardiometabolic_II","B13805","20212097_SS211647","PASS",-3.2253,0.225,"Plate control","PASS","PFC",1.16877724856125,"Baseline","Baseline"
7022,"PFC_09",2,"OID30310","P78539","SRPX",0,"Cardiometabolic_II","B13805","20212097_SS211647","PASS",-3.2253,0.0112,"Plate control","PASS","PFC",1.00779346056616,"Baseline","Baseline"
7023,"PFC_17",3,"OID30310","P78539","SRPX",0,"Cardiometabolic_II","B13805","20212097_SS211647","PASS",-3.2253,0.3403,"Plate control","PASS","PFC",1.26601982802264,"1h","1h"
7024,"PFC_25",4,"OID30310","P78539","SRPX",0,"Cardiometabolic_II","B13805","20212097_SS211647","PASS",-3.2253,0.7265,"Plate control","PASS","PFC",1.65462008515057,"24h","24h"
7025,"PFC_02",13,"OID30310","P78539","SRPX",0,"Cardiometabolic_II","B13805","20212097_SS211647","PASS",-3.2253,-0.1713,"Plate control","PASS","PFC",0.888042113475109,"Baseline","Baseline"
7026,"PFC_10",14,"OID30310","P78539","SRPX",0,"Cardiometabolic_II","B13805","20212097_SS211647","PASS",-3.2253,0.3936,"Plate control","PASS","PFC",1.31366735072413,"1h","1h"
7027,"PFC_18",15,"OID30310","P78539","SRPX",0,"Cardiometabolic_II","B13805","20212097_SS211647","PASS",-3.2253,0.3887,"Plate control","PASS","PFC",1.30921315155448,"1h","1h"
7028,"PFC_26",16,"OID30310","P78539","SRPX",0,"Cardiometabolic_II","B13805","20212097_SS211647","PASS",-3.2253,0.3246,"Plate control","PASS","PFC",1.25231717443786,"24h","24h"
7029,"PFC_03",25,"OID30310","P78539","SRPX",0,"Cardiometabolic_II","B13805","20212097_SS211647","PASS",-3.2253,-0.2192,"Plate control","PASS","PFC",0.859041658231543,"Baseline","Baseline"
7030,"PFC_11",26,"OID30310","P78539","SRPX",0,"Cardiometabolic_II","B13805","20212097_SS211647","PASS",-3.2253,-0.0417,"Plate control","PASS","PFC",0.971509494258729,"1h","1h"
7031,"PFC_19",27,"OID30310","P78539","SRPX",0,"Cardiometabolic_II","B13805","20212097_SS211647","PASS",-3.2253,0.4279,"Plate control","PASS","PFC",1.34527395800494,"24h","24h"
7032,"PFC_27",28,"OID30310","P78539","SRPX",0,"Cardiometabolic_II","B13805","20212097_SS211647","PASS",-3.2253,-0.2099,"Plate control","PASS","PFC",0.864597158539198,"24h","24h"
7033,"PFC_04",37,"OID30310","P78539","SRPX",0,"Cardiometabolic_II","B13805","20212097_SS211647","PASS",-3.2253,1.1796,"Plate control","PASS","PFC",2.26513965345435,"Baseline","Baseline"
7034,"PFC_12",38,"OID30310","P78539","SRPX",0,"Cardiometabolic_II","B13805","20212097_SS211647","PASS",-3.2253,-0.1499,"Plate control","PASS","PFC",0.901312934697636,"1h","1h"
7035,"PFC_20",39,"OID30310","P78539","SRPX",0,"Cardiometabolic_II","B13805","20212097_SS211647","PASS",-3.2253,0.306,"Plate control","PASS","PFC",1.23627526092095,"24h","24h"
7036,"PFC_05",49,"OID30310","P78539","SRPX",0,"Cardiometabolic_II","B13805","20212097_SS211647","PASS",-3.2253,0.0306,"Plate control","PASS","PFC",1.02143684102389,"Baseline","Baseline"
7037,"PFC_13",50,"OID30310","P78539","SRPX",0,"Cardiometabolic_II","B13805","20212097_SS211647","PASS",-3.2253,0.9401,"Plate control","PASS","PFC",1.91866122550339,"1h","1h"
7038,"PFC_21",51,"OID30310","P78539","SRPX",0,"Cardiometabolic_II","B13805","20212097_SS211647","PASS",-3.2253,0.0161,"Plate control","PASS","PFC",1.01122217100181,"24h","24h"
7039,"PFC_06",61,"OID30310","P78539","SRPX",0,"Cardiometabolic_II","B13805","20212097_SS211647","PASS",-3.2253,-0.1232,"Plate control","PASS","PFC",0.918148871017712,"Baseline","Baseline"
7040,"PFC_14",62,"OID30310","P78539","SRPX",0,"Cardiometabolic_II","B13805","20212097_SS211647","PASS",-3.2253,0.2122,"Plate control","PASS","PFC",1.15845339080384,"1h","1h"
7041,"PFC_22",63,"OID30310","P78539","SRPX",0,"Cardiometabolic_II","B13805","20212097_SS211647","PASS",-3.2253,1.1703,"Plate control","PASS","PFC",2.25058491669936,"24h","24h"
7042,"PFC_07",73,"OID30310","P78539","SRPX",0,"Cardiometabolic_II","B13805","20212097_SS211647","PASS",-3.2253,0.7608,"Plate control","PASS","PFC",1.69442995574031,"Baseline","Baseline"
7043,"PFC_15",74,"OID30310","P78539","SRPX",0,"Cardiometabolic_II","B13805","20212097_SS211647","PASS",-3.2253,0.077,"Plate control","PASS","PFC",1.05482231706806,"1h","1h"
7044,"PFC_23",75,"OID30310","P78539","SRPX",0,"Cardiometabolic_II","B13805","20212097_SS211647","PASS",-3.2253,0.0533,"Plate control","PASS","PFC",1.03763568440781,"24h","24h"
7045,"PFC_08",85,"OID30310","P78539","SRPX",0,"Cardiometabolic_II","B13805","20212097_SS211647","PASS",-3.2253,-0.0108,"Plate control","PASS","PFC",0.99254196068119,"Baseline","Baseline"
7046,"PFC_16",86,"OID30310","P78539","SRPX",0,"Cardiometabolic_II","B13805","20212097_SS211647","PASS",-3.2253,0.4522,"Plate control","PASS","PFC",1.36812495314749,"1h","1h"
7047,"PFC_24",87,"OID30310","P78539","SRPX",0,"Cardiometabolic_II","B13805","20212097_SS211647","PASS",-3.2253,-0.1335,"Plate control","PASS","PFC",0.911617168329803,"24h","24h"
7048,"PFC_01",1,"OID30311","Q8N4F0","BPIFB2",0,"Cardiometabolic_II","B13805","20212097_SS211647","PASS",-3.6705,-2.6174,"Plate control","PASS","PFC",0.16296115321757,"Baseline","Baseline"
7049,"PFC_09",2,"OID30311","Q8N4F0","BPIFB2",0,"Cardiometabolic_II","B13805","20212097_SS211647","PASS",-3.6705,-1.9728,"Plate control","PASS","PFC",0.254758113679952,"Baseline","Baseline"
7050,"PFC_17",3,"OID30311","Q8N4F0","BPIFB2",0,"Cardiometabolic_II","B13805","20212097_SS211647","PASS",-3.6705,-2.4512,"Plate control","PASS","PFC",0.182858551253012,"1h","1h"
7051,"PFC_25",4,"OID30311","Q8N4F0","BPIFB2",0,"Cardiometabolic_II","B13805","20212097_SS211647","PASS",-3.6705,-1.6987,"Plate control","PASS","PFC",0.308063571720299,"24h","24h"
7052,"PFC_02",13,"OID30311","Q8N4F0","BPIFB2",0,"Cardiometabolic_II","B13805","20212097_SS211647","PASS",-3.6705,-2.7252,"Plate control","PASS","PFC",0.151228295005922,"Baseline","Baseline"
7053,"PFC_10",14,"OID30311","Q8N4F0","BPIFB2",0,"Cardiometabolic_II","B13805","20212097_SS211647","PASS",-3.6705,-2.3359,"Plate control","PASS","PFC",0.198072431590181,"1h","1h"
7054,"PFC_18",15,"OID30311","Q8N4F0","BPIFB2",0,"Cardiometabolic_II","B13805","20212097_SS211647","PASS",-3.6705,-1.7587,"Plate control","PASS","PFC",0.295514330822468,"1h","1h"
7055,"PFC_26",16,"OID30311","Q8N4F0","BPIFB2",0,"Cardiometabolic_II","B13805","20212097_SS211647","PASS",-3.6705,-2.4951,"Plate control","PASS","PFC",0.177378124184262,"24h","24h"
7056,"PFC_03",25,"OID30311","Q8N4F0","BPIFB2",0,"Cardiometabolic_II","B13805","20212097_SS211647","PASS",-3.6705,-2.89,"Plate control","PASS","PFC",0.134903529563053,"Baseline","Baseline"

Supplemental table 1_Olink proximity extension assay (PEA) data

,"SampleID","Index","OlinkID","UniProt","Assay","MissingFreq","Panel","Panel_Lot_Nr","PlateID","QC_Warning","LOD","NPX","Normalization","Assay_Warning","condition","Value","group","alt_group"
7057,"PFC_11",26,"OID30311","Q8N4F0","BPIFB2",0,"Cardiometabolic_II","B13805","20212097_SS211647","PASS",-3.6705,-2.2675,"Plate control","PASS","PFC",0.207689473237438,"1h","1h"
7058,"PFC_19",27,"OID30311","Q8N4F0","BPIFB2",0,"Cardiometabolic_II","B13805","20212097_SS211647","PASS",-3.6705,-2.5888,"Plate control","PASS","PFC",0.16622393045528,"24h","24h"
7059,"PFC_27",28,"OID30311","Q8N4F0","BPIFB2",0,"Cardiometabolic_II","B13805","20212097_SS211647","PASS",-3.6705,-2.2783,"Plate control","PASS","PFC",0.206140516979931,"24h","24h"
7060,"PFC_04",37,"OID30311","Q8N4F0","BPIFB2",0,"Cardiometabolic_II","B13805","20212097_SS211647","PASS",-3.6705,-2.5695,"Plate control","PASS","PFC",0.168462571670378,"Baseline","Baseline"
7061,"PFC_12",38,"OID30311","Q8N4F0","BPIFB2",0,"Cardiometabolic_II","B13805","20212097_SS211647","PASS",-3.6705,-2.8222,"Plate control","PASS","PFC",0.141394705028777,"1h","1h"
7062,"PFC_20",39,"OID30311","Q8N4F0","BPIFB2",0,"Cardiometabolic_II","B13805","20212097_SS211647","PASS",-3.6705,-1.7993,"Plate control","PASS","PFC",0.287313960537895,"24h","24h"
7063,"PFC_05",49,"OID30311","Q8N4F0","BPIFB2",0,"Cardiometabolic_II","B13805","20212097_SS211647","PASS",-3.6705,-0.1821,"Plate control","PASS","PFC",0.881419060476053,"Baseline","Baseline"
7064,"PFC_13",50,"OID30311","Q8N4F0","BPIFB2",0,"Cardiometabolic_II","B13805","20212097_SS211647","PASS",-3.6705,-0.7631,"Plate control","PASS","PFC",0.589228860258005,"1h","1h"
7065,"PFC_21",51,"OID30311","Q8N4F0","BPIFB2",0,"Cardiometabolic_II","B13805","20212097_SS211647","PASS",-3.6705,-1.3995,"Plate control","PASS","PFC",0.37906049122051,"24h","24h"
7066,"PFC_06",61,"OID30311","Q8N4F0","BPIFB2",0,"Cardiometabolic_II","B13805","20212097_SS211647","PASS",-3.6705,-0.5981,"Plate control","PASS","PFC",0.660623410316676,"Baseline","Baseline"
7067,"PFC_14",62,"OID30311","Q8N4F0","BPIFB2",0,"Cardiometabolic_II","B13805","20212097_SS211647","PASS",-3.6705,-1.2,"Plate control","PASS","PFC",0.435275281648062,"1h","1h"
7068,"PFC_22",63,"OID30311","Q8N4F0","BPIFB2",0,"Cardiometabolic_II","B13805","20212097_SS211647","PASS",-3.6705,-1.9444,"Plate control","PASS","PFC",0.259822810626832,"24h","24h"
7069,"PFC_07",73,"OID30311","Q8N4F0","BPIFB2",0,"Cardiometabolic_II","B13805","20212097_SS211647","PASS",-3.6705,-1.825,"Plate control","PASS","PFC",0.282241101201533,"Baseline","Baseline"
7070,"PFC_15",74,"OID30311","Q8N4F0","BPIFB2",0,"Cardiometabolic_II","B13805","20212097_SS211647","PASS",-3.6705,0.0024,"Plate control","PASS","PFC",1.00166493770563,"1h","1h"
7071,"PFC_23",75,"OID30311","Q8N4F0","BPIFB2",0,"Cardiometabolic_II","B13805","20212097_SS211647","PASS",-3.6705,-1.2009,"Plate control","PASS","PFC",0.43500382747701,"24h","24h"
7072,"PFC_08",85,"OID30311","Q8N4F0","BPIFB2",0,"Cardiometabolic_II","B13805","20212097_SS211647","PASS",-3.6705,-2.3876,"Plate control","PASS","PFC",0.191100041933266,"Baseline","Baseline"
7073,"PFC_16",86,"OID30311","Q8N4F0","BPIFB2",0,"Cardiometabolic_II","B13805","20212097_SS211647","PASS",-3.6705,-1.5041,"Plate control","PASS","PFC",0.352550052366679,"1h","1h"
7074,"PFC_24",87,"OID30311","Q8N4F0","BPIFB2",0,"Cardiometabolic_II","B13805","20212097_SS211647","PASS",-3.6705,-0.1418,"Plate control","PASS","PFC",0.906387581557534,"24h","24h"
7075,"PFC_01",1,"OID30312","P54687","BCAT1",0,"Cardiometabolic_II","B13805","20212097_SS211647","PASS",-1.1693,0.2493,"Plate control","PASS","PFC",1.18863024807162,"Baseline","Baseline"
7076,"PFC_09",2,"OID30312","P54687","BCAT1",0,"Cardiometabolic_II","B13805","20212097_SS211647","PASS",-1.1693,0.1012,"Plate control","PASS","PFC",1.07266530949811,"Baseline","Baseline"
7077,"PFC_17",3,"OID30312","P54687","BCAT1",0,"Cardiometabolic_II","B13805","20212097_SS211647","PASS",-1.1693,0.5256,"Plate control","PASS","PFC",1.43953214012167,"1h","1h"
7078,"PFC_25",4,"OID30312","P54687","BCAT1",0,"Cardiometabolic_II","B13805","20212097_SS211647","PASS",-1.1693,0.439,"Plate control","PASS","PFC",1.3556643268,"24h","24h"
7079,"PFC_02",13,"OID30312","P54687","BCAT1",0,"Cardiometabolic_II","B13805","20212097_SS211647","PASS",-1.1693,0.4814,"Plate control","PASS","PFC",1.39609779096541,"Baseline","Baseline"
7080,"PFC_10",14,"OID30312","P54687","BCAT1",0,"Cardiometabolic_II","B13805","20212097_SS211647","PASS",-1.1693,0.3062,"Plate control","PASS","PFC",1.23644665694324,"1h","1h"
7081,"PFC_18",15,"OID30312","P54687","BCAT1",0,"Cardiometabolic_II","B13805","20212097_SS211647","PASS",-1.1693,0.1303,"Plate control","PASS","PFC",1.09452127689955,"1h","1h"
7082,"PFC_26",16,"OID30312","P54687","BCAT1",0,"Cardiometabolic_II","B13805","20212097_SS211647","PASS",-1.1693,0.5944,"Plate control","PASS","PFC",1.50984452828432,"24h","24h"
7083,"PFC_03",25,"OID30312","P54687","BCAT1",0,"Cardiometabolic_II","B13805","20212097_SS211647","PASS",-1.1693,0.3688,"Plate control","PASS","PFC",1.29127832870722,"Baseline","Baseline"
7084,"PFC_11",26,"OID30312","P54687","BCAT1",0,"Cardiometabolic_II","B13805","20212097_SS211647","PASS",-1.1693,0.7482,"Plate control","PASS","PFC",1.67969582503379,"1h","1h"
7085,"PFC_19",27,"OID30312","P54687","BCAT1",0,"Cardiometabolic_II","B13805","20212097_SS211647","PASS",-1.1693,0.2355,"Plate control","PASS","PFC",1.17731469270132,"24h","24h"
7086,"PFC_27",28,"OID30312","P54687","BCAT1",0,"Cardiometabolic_II","B13805","20212097_SS211647","PASS",-1.1693,0.4094,"Plate control","PASS","PFC",1.32813334409185,"24h","24h"
7087,"PFC_04",37,"OID30312","P54687","BCAT1",0,"Cardiometabolic_II","B13805","20212097_SS211647","PASS",-1.1693,0.6174,"Plate control","PASS","PFC",1.53410794575211,"Baseline","Baseline"
7088,"PFC_12",38,"OID30312","P54687","BCAT1",0,"Cardiometabolic_II","B13805","20212097_SS211647","PASS",-1.1693,0.4495,"Plate control","PASS","PFC",1.36556690530844,"1h","1h"
7089,"PFC_20",39,"OID30312","P54687","BCAT1",0,"Cardiometabolic_II","B13805","20212097_SS211647","PASS",-1.1693,0.6688,"Plate control","PASS","PFC",1.58975010046125,"24h","24h"
7090,"PFC_05",49,"OID30312","P54687","BCAT1",0,"Cardiometabolic_II","B13805","20212097_SS211647","PASS",-1.1693,0.4021,"Plate control","PASS","PFC",1.32142999703159,"Baseline","Baseline"
7091,"PFC_13",50,"OID30312","P54687","BCAT1",0,"Cardiometabolic_II","B13805","20212097_SS211647","PASS",-1.1693,0.4868,"Plate control","PASS","PFC",1.40133316958752,"1h","1h"
7092,"PFC_21",51,"OID30312","P54687","BCAT1",0,"Cardiometabolic_II","B13805","20212097_SS211647","PASS",-1.1693,0.4732,"Plate control","PASS","PFC",1.38818514900148,"24h","24h"
7093,"PFC_06",61,"OID30312","P54687","BCAT1",0,"Cardiometabolic_II","B13805","20212097_SS211647","PASS",-1.1693,0.311,"Plate control","PASS","PFC",1.24056729771539,"Baseline","Baseline"
7094,"PFC_14",62,"OID30312","P54687","BCAT1",0,"Cardiometabolic_II","B13805","20212097_SS211647","PASS",-1.1693,0.6523,"Plate control","PASS","PFC",1.57167181932446,"1h","1h"
7095,"PFC_22",63,"OID30312","P54687","BCAT1",0,"Cardiometabolic_II","B13805","20212097_SS211647","PASS",-1.1693,0.209,"Plate control","PASS","PFC",1.1558867065548,"24h","24h"
7096,"PFC_07",73,"OID30312","P54687","BCAT1",0,"Cardiometabolic_II","B13805","20212097_SS211647","PASS",-1.1693,0.6374,"Plate control","PASS","PFC",1.55552329461656,"Baseline","Baseline"
7097,"PFC_15",74,"OID30312","P54687","BCAT1",0,"Cardiometabolic_II","B13805","20212097_SS211647","PASS",-1.1693,0.1823,"Plate control","PASS","PFC",1.13469141285128,"1h","1h"
7098,"PFC_23",75,"OID30312","P54687","BCAT1",0,"Cardiometabolic_II","B13805","20212097_SS211647","PASS",-1.1693,0.9989,"Plate control","PASS","PFC",1.99847565740319,"24h","24h"
7099,"PFC_08",85,"OID30312","P54687","BCAT1",0,"Cardiometabolic_II","B13805","20212097_SS211647","PASS",-1.1693,0.4114,"Plate control","PASS","PFC",1.32997580465885,"Baseline","Baseline"
7100,"PFC_16",86,"OID30312","P54687","BCAT1",0,"Cardiometabolic_II","B13805","20212097_SS211647","PASS",-1.1693,0.5187,"Plate control","PASS","PFC",1.43266370535379,"1h","1h"
7101,"PFC_24",87,"OID30312","P54687","BCAT1",0,"Cardiometabolic_II","B13805","20212097_SS211647","PASS",-1.1693,0.59,"Plate control","PASS","PFC",1.50524674741107,"24h","24h"
7102,"PFC_01",1,"OID30313","Q969H8","MYDGF",0,"Cardiometabolic_II","B13805","20212097_SS211647","PASS",-1.4485,2.3763,"Plate control","PASS","PFC",5.19203460875585,"Baseline","Baseline"
7103,"PFC_09",2,"OID30313","Q969H8","MYDGF",0,"Cardiometabolic_II","B13805","20212097_SS211647","PASS",-1.4485,3.9548,"Plate control","PASS","PFC",15.5064872663656,"Baseline","Baseline"
7104,"PFC_17",3,"OID30313","Q969H8","MYDGF",0,"Cardiometabolic_II","B13805","20212097_SS211647","PASS",-1.4485,5.5753,"Plate control","PASS","PFC",47.6795924816764,"1h","1h"
7105,"PFC_25",4,"OID30313","Q969H8","MYDGF",0,"Cardiometabolic_II","B13805","20212097_SS211647","PASS",-1.4485,4.5399,"Plate control","PASS","PFC",23.2619478258873,"24h","24h"
7106,"PFC_02",13,"OID30313","Q969H8","MYDGF",0,"Cardiometabolic_II","B13805","20212097_SS211647","PASS",-1.4485,2.7421,"Plate control","PASS","PFC",6.69043492885144,"Baseline","Baseline"
7107,"PFC_10",14,"OID30313","Q969H8","MYDGF",0,"Cardiometabolic_II","B13805","20212097_SS211647","PASS",-1.4485,3.62,"Plate control","PASS","PFC",12.2950014503041,"1h","1h"
7108,"PFC_18",15,"OID30313","Q969H8","MYDGF",0,"Cardiometabolic_II","B13805","20212097_SS211647","PASS",-1.4485,4.5767,"Plate control","PASS","PFC",23.8629417518631,"1h","1h"
7109,"PFC_26",16,"OID30313","Q969H8","MYDGF",0,"Cardiometabolic_II","B13805","20212097_SS211647","PASS",-1.4485,5.6464,"Plate control","PASS","PFC",50.0882395149106,"24h","24h"
7110,"PFC_03",25,"OID30313","Q969H8","MYDGF",0,"Cardiometabolic_II","B13805","20212097_SS211647","PASS",-1.4485,1.3284,"Plate control","PASS","PFC",2.51124014966335,"Baseline","Baseline"
7111,"PFC_11",26,"OID30313","Q969H8","MYDGF",0,"Cardiometabolic_II","B13805","20212097_SS211647","PASS",-1.4485,4.8378,"Plate control","PASS","PFC",28.5971605123764,"1h","1h"
7112,"PFC_19",27,"OID30313","Q969H8","MYDGF",0,"Cardiometabolic_II","B13805","20212097_SS211647","PASS",-1.4485,4.5336,"Plate control","PASS","PFC",23.1605883891679,"24h","24h"
7113,"PFC_27",28,"OID30313","Q969H8","MYDGF",0,"Cardiometabolic_II","B13805","20212097_SS211647","PASS",-1.4485,6.0976,"Plate control","PASS","PFC",68.4794875214859,"24h","24h"
7114,"PFC_04",37,"OID30313","Q969H8","MYDGF",0,"Cardiometabolic_II","B13805","20212097_SS211647","PASS",-1.4485,3.9399,"Plate control","PASS","PFC",15.3471620881223,"Baseline","Baseline"
7115,"PFC_12",38,"OID30313","Q969H8","MYDGF",0,"Cardiometabolic_II","B13805","20212097_SS211647","PASS",-1.4485,1.7691,"Plate control","PASS","PFC",3.40841262527188,"1h","1h"
7116,"PFC_20",39,"OID30313","Q969H8","MYDGF",0,"Cardiometabolic_II","B13805","20212097_SS211647","PASS",-1.4485,5.5511,"Plate control","PASS","PFC",46.8864777979209,"24h","24h"
7117,"PFC_05",49,"OID30313","Q969H8","MYDGF",0,"Cardiometabolic_II","B13805","20212097_SS211647","PASS",-1.4485,2.453,"Plate control","PASS","PFC",5.47553525236735,"Baseline","Baseline"
7118,"PFC_13",50,"OID30313","Q969H8","MYDGF",0,"Cardiometabolic_II","B13805","20212097_SS211647","PASS",-1.4485,4.2939,"Plate control","PASS","PFC",19.6151980918032,"1h","1h"
7119,"PFC_21",51,"OID30313","Q969H8","MYDGF",0,"Cardiometabolic_II","B13805","20212097_SS211647","PASS",-1.4485,2.9579,"Plate control","PASS","PFC",7.76992137575775,"24h","24h"
7120,"PFC_06",61,"OID30313","Q969H8","MYDGF",0,"Cardiometabolic_II","B13805","20212097_SS211647","PASS",-1.4485,4.0466,"Plate control","PASS","PFC",16.5252477961518,"Baseline","Baseline"
7121,"PFC_14",62,"OID30313","Q969H8","MYDGF",0,"Cardiometabolic_II","B13805","20212097_SS211647","PASS",-1.4485,5.0033,"Plate control","PASS","PFC",32.0732801202656,"1h","1h"
7122,"PFC_22",63,"OID30313","Q969H8","MYDGF",0,"Cardiometabolic_II","B13805","20212097_SS211647","PASS",-1.4485,3.9918,"Plate control","PASS","PFC",15.9093170462397,"24h","24h"
7123,"PFC_07",73,"OID30313","Q969H8","MYDGF",0,"Cardiometabolic_II","B13805","20212097_SS211647","PASS",-1.4485,4.9594,"Plate control","PASS","PFC",31.1120165022929,"Baseline","Baseline"
7124,"PFC_15",74,"OID30313","Q969H8","MYDGF",0,"Cardiometabolic_II","B13805","20212097_SS211647","PASS",-1.4485,2.8321,"Plate control","PASS","PFC",7.12109944591394,"1h","1h"
7125,"PFC_23",75,"OID30313","Q969H8","MYDGF",0,"Cardiometabolic_II","B13805","20212097_SS211647","PASS",-1.4485,4.9764,"Plate control","PASS","PFC",31.4807934882477,"24h","24h"
7126,"PFC_08",85,"OID30313","Q969H8","MYDGF",0,"Cardiometabolic_II","B13805","20212097_SS211647","PASS",-1.4485,4.1719,"Plate control","PASS","PFC",18.0246582385516,"Baseline","Baseline"
7127,"PFC_16",86,"OID30313","Q969H8","MYDGF",0,"Cardiometabolic_II","B13805","20212097_SS211647","PASS",-1.4485,4.2478,"Plate control","PASS","PFC",18.9983207481177,"1h","1h"
7128,"PFC_24",87,"OID30313","Q969H8","MYDGF",0,"Cardiometabolic_II","B13805","20212097_SS211647","PASS",-1.4485,5.152,"Plate control","PASS","PFC",35.555479316963,"24h","24h"
7129,"PFC_01",1,"OID30314","Q96EM0","L3HYPDH",0,"Cardiometabolic_II","B13805","20212097_SS211647","PASS",-1.4233,2.4369,"Plate control","PASS","PFC",5.41476977996119,"Baseline","Baseline"
7130,"PFC_09",2,"OID30314","Q96EM0","L3HYPDH",0,"Cardiometabolic_II","B13805","20212097_SS211647","PASS",-1.4233,3.4238,"Plate control","PASS","PFC",10.7316499733479,"Baseline","Baseline"
7131,"PFC_17",3,"OID30314","Q96EM0","L3HYPDH",0,"Cardiometabolic_II","B13805","20212097_SS211647","PASS",-1.4233,5.2996,"Plate control","PASS","PFC",39.3856996783846,"1h","1h"
7132,"PFC_25",4,"OID30314","Q96EM0","L3HYPDH",0,"Cardiometabolic_II","B13805","20212097_SS211647","PASS",-1.4233,3.9308,"Plate control","PASS","PFC",15.2506623873321,"24h","24h"
7133,"PFC_02",13,"OID30314","Q96EM0","L3HYPDH",0,"Cardiometabolic_II","B13805","20212097_SS211647","PASS",-1.4233,3.0454,"Plate control","PASS","PFC",8.25575409819066,"Baseline","Baseline"
7134,"PFC_10",14,"OID30314","Q96EM0","L3HYPDH",0,"Cardiometabolic_II","B13805","20212097_SS211647","PASS",-1.4233,3.0112,"Plate control","PASS","PFC",8.06234768452927,"1h","1h"
7135,"PFC_18",15,"OID30314","Q96EM0","L3HYPDH",0,"Cardiometabolic_II","B13805","20212097_SS211647","PASS",-1.4233,4.2689,"Plate control","PASS","PFC",19.2782207291696,"1h","1h"
7136,"PFC_26",16,"OID30314","Q96EM0","L3HYPDH",0,"Cardiometabolic_II","B13805","20212097_SS211647","PASS",-1.4233,4.6816,"Plate control","PASS","PFC",25.6626811307828,"24h","24h"
7137,"PFC_03",25,"OID30314","Q96EM0","L3HYPDH",0,"Cardiometabolic_II","B13805","20212097_SS211647","PASS",-1.4233,1.4603,"Plate control","PASS","PFC",2.75165576752289,"Baseline","Baseline"
7138,"PFC_11",26,"OID30314","Q96EM0","L3HYPDH",0,"Cardiometabolic_II","B13805","20212097_SS211647","PASS",-1.4233,4.2765,"Plate control","PASS","PFC",19.3800447911463,"1h","1h"
7139,"PFC_19",27,"OID30314","Q96EM0","L3HYPDH",0,"Cardiometabolic_II","B13805","20212097_SS211647","PASS",-1.4233,3.4079,"Plate control","PASS","PFC",10.6140253911016,"24h","24h"
7140,"PFC_27",28,"OID30314","Q96EM0","L3HYPDH",0,"Cardiometabolic_II","B13805","20212097_SS211647","PASS",-1.4233,5.7709,"Plate control","PASS","PFC",54.6026853782571,"24h","24h"
7141,"PFC_04",37,"OID30314","Q96EM0","L3HYPDH",0,"Cardiometabolic_II","B13805","20212097_SS211647","PASS",-1.4233,2.4446,"Plate control","PASS","PFC",5.44374692960701,"Baseline","Baseline"
7142,"PFC_12",38,"OID30314","Q96EM0","L3HYPDH",0,"Cardiometabolic_II","B13805","20212097_SS211647","PASS",-1.4233,1.1197,"Plate control","PASS","PFC",2.17301781171653,"1h","1h"
7143,"PFC_20",39,"OID30314","Q96EM0","L3HYPDH",0,"Cardiometabolic_II","B13805","20212097_SS211647","PASS",-1.4233,5.1652,"Plate control","PASS","PFC",35.8822884935534,"24h","24h"
7144,"PFC_05",49,"OID30314","Q96EM0","L3HYPDH",0,"Cardiometabolic_II","B13805","20212097_SS211647","PASS",-1.4233,1.4852,"Plate control","PASS","PFC",2.79955980568772,"Baseline","Baseline"
7145,"PFC_13",50,"OID30314","Q96EM0","L3HYPDH",0,"Cardiometabolic_II","B13805","20212097_SS211647","PASS",-1.4233,2.8418,"Plate control","PASS","PFC",7.16913967387296,"1h","1h"
7146,"PFC_21",51,"OID30314","Q96EM0","L3HYPDH",0,"Cardiometabolic_II","B13805","20212097_SS211647","PASS",-1.4233,2.2187,"Plate control","PASS","PFC",4.65473811138489,"24h","24h"
7147,"PFC_06",61,"OID30314","Q96EM0","L3HYPDH",0,"Cardiometabolic_II","B13805","20212097_SS211647","PASS",-1.4233,2.7923,"Plate control","PASS","PFC",6.92733287277091,"Baseline","Baseline"
7148,"PFC_14",62,"OID30314","Q96EM0","L3HYPDH",0,"Cardiometabolic_II","B13805","20212097_SS211647","PASS",-1.4233,3.7019,"Plate control","PASS","PFC",13.0131651349799,"1h","1h"
7149,"PFC_22",63,"OID30314","Q96EM0","L3HYPDH",0,"Cardiometabolic_II","B13805","20212097_SS211647","PASS",-1.4233,2.6966,"Plate control","PASS","PFC",6.48272331733202,"24h","24h"
7150,"PFC_07",73,"OID30314","Q96EM0","L3HYPDH",0,"Cardiometabolic_II","B13805","20212097_SS211647","PASS",-1.4233,4.1353,"Plate control","PASS","PFC",17.5731388721799,"Baseline","Baseline"
7151,"PFC_15",74,"OID30314","Q96EM0","L3HYPDH",0,"Cardiometabolic_II","B13805","20212097_SS211647","PASS",-1.4233,1.3765,"Plate control","PASS","PFC",2.59637721373941,"1h","1h"
7152,"PFC_23",75,"OID30314","Q96EM0","L3HYPDH",0,"Cardiometabolic_II","B13805","20212097_SS211647","PASS",-1.4233,4.1343,"Plate control","PASS","PFC",17.5609623210757,"24h","24h"
7153,"PFC_08",85,"OID30314","Q96EM0","L3HYPDH",0,"Cardiometabolic_II","B13805","20212097_SS211647","PASS",-1.4233,2.1452,"Plate control","PASS","PFC",4.42353580305102,"Baseline","Baseline"
7154,"PFC_16",86,"OID30314","Q96EM0","L3HYPDH",0,"Cardiometabolic_II","B13805","20212097_SS211647","PASS",-1.4233,2.8232,"Plate control","PASS","PFC",7.07730453738643,"1h","1h"
7155,"PFC_24",87,"OID30314","Q96EM0","L3HYPDH",0,"Cardiometabolic_II","B13805","20212097_SS211647","PASS",-1.4233,3.7902,"Plate control","PASS","PFC",13.8345134387922,"24h","24h"
7156,"PFC_01",1,"OID30315","Q9BXN1","ASPN",0,"Cardiometabolic_II","B13805","20212097_SS211647","PASS",-1.0486,0.2969,"Plate control","PASS","PFC",1.22850182423682,"Baseline","Baseline"
7157,"PFC_09",2,"OID30315","Q9BXN1","ASPN",0,"Cardiometabolic_II","B13805","20212097_SS211647","PASS",-1.0486,0.7925,"Plate control","PASS","PFC",1.73207331789726,"Baseline","Baseline"
7158,"PFC_17",3,"OID30315","Q9BXN1","ASPN",0,"Cardiometabolic_II","B13805","20212097_SS211647","PASS",-1.0486,0.1083,"Plate control","PASS","PFC",1.07795727665356,"1h","1h"
7159,"PFC_25",4,"OID30315","Q9BXN1","ASPN",0,"Cardiometabolic_II","B13805","20212097_SS211647","PASS",-1.0486,-0.2027,"Plate control","PASS","PFC",0.868922853793348,"24h","24h"
7160,"PFC_02",13,"OID30315","Q9BXN1","ASPN",0,"Cardiometabolic_II","B13805","20212097_SS211647","PASS",-1.0486,0.2253,"Plate control","PASS","PFC",1.16902031422877,"Baseline","Baseline"
7161,"PFC_10",14,"OID30315","Q9BXN1","ASPN",0,"Cardiometabolic_II","B13805","20212097_SS211647","PASS",-1.0486,0.5122,"Plate control","PASS","PFC",1.42622342020862,"1h","1h"
7162,"PFC_18",15,"OID30315","Q9BXN1","ASPN",0,"Cardiometabolic_II","B13805","20212097_SS211647","PASS",-1.0486,0.5928,"Plate control","PASS","PFC",1.50817098530055,"1h","1h"
7163,"PFC_26",16,"OID30315","Q9BXN1","ASPN",0,"Cardiometabolic_II","B13805","20212097_SS211647","PASS",-1.0486,0.0674,"Plate control","PASS","PFC",1.04782660605208,"24h","24h"
7164,"PFC_03",25,"OID30315","Q9BXN1","ASPN",0,"Cardiometabolic_II","B13805","20212097_SS211647","PASS",-1.0486,0.2551,"Plate control","PASS","PFC",1.19341846162907,"Baseline","Baseline"
7165,"PFC_11",26,"OID30315","Q9BXN1","ASPN",0,"Cardiometabolic_II","B13805","20212097_SS211647","PASS",-1.0486,0.5779,"Plate control","PASS","PFC",1.49267491537016,"1h","1h"
7166,"PFC_19",27,"OID30315","Q9BXN1","ASPN",0,"Cardiometabolic_II","B13805","20212097_SS211647","PASS",-1.0486,0.6474,"Plate control","PASS","PFC",1.56634281475665,"24h","24h"
7167,"PFC_27",28,"OID30315","Q9BXN1","ASPN",0,"Cardiometabolic_II","B13805","20212097_SS211647","PASS",-1.0486,0.0934,"Plate control","PASS","PFC",1.06688154220183,"24h","24h"
7168,"PFC_04",37,"OID30315","Q9BXN1","ASPN",0,"Cardiometabolic_II","B13805","20212097_SS211647","PASS",-1.0486,0.661,"Plate control","PASS","PFC",1.58117823320789,"Baseline","Baseline"

,"SampleID","Index","OlinkID","UniProt","Assay","MissingFreq","Panel","Panel_Lot_Nr","PlateID","QC_Warning","LOD","NPX","Normalization","Assay_Warning","condition","Value","group","alt_group"
7169,"PFC_12",38,"OID30315","Q9BXN1","ASPN",0,"Cardiometabolic_II","B13805","20212097_SS211647","PASS",-1.0486,-0.3081,"Plate control","PASS","PFC",0.807704789839462,"1h","1h"
7170,"PFC_20",39,"OID30315","Q9BXN1","ASPN",0,"Cardiometabolic_II","B13805","20212097_SS211647","PASS",-1.0486,0.8346,"Plate control","PASS","PFC",1.78336251720781,"24h","24h"
7171,"PFC_05",49,"OID30315","Q9BXN1","ASPN",0,"Cardiometabolic_II","B13805","20212097_SS211647","PASS",-1.0486,0.3946,"Plate control","PASS","PFC",1.31457823119512,"Baseline","Baseline"
7172,"PFC_13",50,"OID30315","Q9BXN1","ASPN",0,"Cardiometabolic_II","B13805","20212097_SS211647","PASS",-1.0486,0.4616,"Plate control","PASS","PFC",1.37706818907584,"1h","1h"
7173,"PFC_21",51,"OID30315","Q9BXN1","ASPN",0,"Cardiometabolic_II","B13805","20212097_SS211647","PASS",-1.0486,-0.003,"Plate control","PASS","PFC",0.997922718999051,"24h","24h"
7174,"PFC_06",61,"OID30315","Q9BXN1","ASPN",0,"Cardiometabolic_II","B13805","20212097_SS211647","PASS",-1.0486,0.2295,"Plate control","PASS","PFC",1.17242854604109,"Baseline","Baseline"
7175,"PFC_14",62,"OID30315","Q9BXN1","ASPN",0,"Cardiometabolic_II","B13805","20212097_SS211647","PASS",-1.0486,1.0687,"Plate control","PASS","PFC",2.09754243810895,"1h","1h"
7176,"PFC_22",63,"OID30315","Q9BXN1","ASPN",0,"Cardiometabolic_II","B13805","20212097_SS211647","PASS",-1.0486,0.5588,"Plate control","PASS","PFC",1.47304346447641,"24h","24h"
7177,"PFC_07",73,"OID30315","Q9BXN1","ASPN",0,"Cardiometabolic_II","B13805","20212097_SS211647","PASS",-1.0486,0.6947,"Plate control","PASS","PFC",1.61854783053916,"Baseline","Baseline"
7178,"PFC_15",74,"OID30315","Q9BXN1","ASPN",0,"Cardiometabolic_II","B13805","20212097_SS211647","PASS",-1.0486,0.036,"Plate control","PASS","PFC",1.02526723788859,"1h","1h"
7179,"PFC_23",75,"OID30315","Q9BXN1","ASPN",0,"Cardiometabolic_II","B13805","20212097_SS211647","PASS",-1.0486,-0.1723,"Plate control","PASS","PFC",0.887426782869909,"24h","24h"
7180,"PFC_08",85,"OID30315","Q9BXN1","ASPN",0,"Cardiometabolic_II","B13805","20212097_SS211647","PASS",-1.0486,0.0124,"Plate control","PASS","PFC",1.0086320683199,"Baseline","Baseline"
7181,"PFC_16",86,"OID30315","Q9BXN1","ASPN",0,"Cardiometabolic_II","B13805","20212097_SS211647","PASS",-1.0486,0.0731,"Plate control","PASS","PFC",1.05197469400613,"1h","1h"
7182,"PFC_24",87,"OID30315","Q9BXN1","ASPN",0,"Cardiometabolic_II","B13805","20212097_SS211647","PASS",-1.0486,0.2825,"Plate control","PASS","PFC",1.21630074786166,"24h","24h"
7183,"PFC_01",1,"OID30316","Q13428","TCOF1",0,"Cardiometabolic_II","B13805","20212097_SS211647","PASS",-1.6152,0.039,"Plate control","PASS","PFC",1.02740143937896,"Baseline","Baseline"
7184,"PFC_09",2,"OID30316","Q13428","TCOF1",0,"Cardiometabolic_II","B13805","20212097_SS211647","PASS",-1.6152,0.4955,"Plate control","PASS","PFC",1.40980927314481,"Baseline","Baseline"
7185,"PFC_17",3,"OID30316","Q13428","TCOF1",0,"Cardiometabolic_II","B13805","20212097_SS211647","PASS",-1.6152,0.0046,"Plate control","PASS","PFC",1.00319356563032,"1h","1h"
7186,"PFC_25",4,"OID30316","Q13428","TCOF1",0,"Cardiometabolic_II","B13805","20212097_SS211647","PASS",-1.6152,-0.3251,"Plate control","PASS","PFC",0.798243054446755,"24h","24h"
7187,"PFC_02",13,"OID30316","Q13428","TCOF1",0,"Cardiometabolic_II","B13805","20212097_SS211647","PASS",-1.6152,0.255,"Plate control","PASS","PFC",1.19333574303172,"Baseline","Baseline"
7188,"PFC_10",14,"OID30316","Q13428","TCOF1",0,"Cardiometabolic_II","B13805","20212097_SS211647","PASS",-1.6152,0.1365,"Plate control","PASS","PFC",1.09923511742936,"1h","1h"
7189,"PFC_18",15,"OID30316","Q13428","TCOF1",0,"Cardiometabolic_II","B13805","20212097_SS211647","PASS",-1.6152,0.28,"Plate control","PASS","PFC",1.21419488439505,"1h","1h"
7190,"PFC_26",16,"OID30316","Q13428","TCOF1",0,"Cardiometabolic_II","B13805","20212097_SS211647","PASS",-1.6152,0.0828,"Plate control","PASS","PFC",1.05907150602104,"24h","24h"
7191,"PFC_03",25,"OID30316","Q13428","TCOF1",0,"Cardiometabolic_II","B13805","20212097_SS211647","PASS",-1.6152,0.7761,"Plate control","PASS","PFC",1.71249526610568,"Baseline","Baseline"
7192,"PFC_11",26,"OID30316","Q13428","TCOF1",0,"Cardiometabolic_II","B13805","20212097_SS211647","PASS",-1.6152,0.3803,"Plate control","PASS","PFC",1.30161249001081,"1h","1h"
7193,"PFC_19",27,"OID30316","Q13428","TCOF1",0,"Cardiometabolic_II","B13805","20212097_SS211647","PASS",-1.6152,0.3073,"Plate control","PASS","PFC",1.23738975990318,"24h","24h"
7194,"PFC_27",28,"OID30316","Q13428","TCOF1",0,"Cardiometabolic_II","B13805","20212097_SS211647","PASS",-1.6152,0.5857,"Plate control","PASS","PFC",1.50076698934276,"24h","24h"
7195,"PFC_04",37,"OID30316","Q13428","TCOF1",0,"Cardiometabolic_II","B13805","20212097_SS211647","PASS",-1.6152,0.4708,"Plate control","PASS","PFC",1.38587774888197,"Baseline","Baseline"
7196,"PFC_12",38,"OID30316","Q13428","TCOF1",0,"Cardiometabolic_II","B13805","20212097_SS211647","PASS",-1.6152,0.5563,"Plate control","PASS","PFC",1.4704930850394,"1h","1h"
7197,"PFC_20",39,"OID30316","Q13428","TCOF1",0,"Cardiometabolic_II","B13805","20212097_SS211647","PASS",-1.6152,0.3462,"Plate control","PASS","PFC",1.27120790392602,"24h","24h"
7198,"PFC_05",49,"OID30316","Q13428","TCOF1",0,"Cardiometabolic_II","B13805","20212097_SS211647","PASS",-1.6152,0.3211,"Plate control","PASS","PFC",1.24928271633159,"Baseline","Baseline"
7199,"PFC_13",50,"OID30316","Q13428","TCOF1",0,"Cardiometabolic_II","B13805","20212097_SS211647","PASS",-1.6152,0.2914,"Plate control","PASS","PFC",1.22382731107696,"1h","1h"
7200,"PFC_21",51,"OID30316","Q13428","TCOF1",0,"Cardiometabolic_II","B13805","20212097_SS211647","PASS",-1.6152,0.6518,"Plate control","PASS","PFC",1.5711272137577,"24h","24h"
7201,"PFC_06",61,"OID30316","Q13428","TCOF1",0,"Cardiometabolic_II","B13805","20212097_SS211647","PASS",-1.6152,0.0455,"Plate control","PASS","PFC",1.03204079541141,"Baseline","Baseline"
7202,"PFC_14",62,"OID30316","Q13428","TCOF1",0,"Cardiometabolic_II","B13805","20212097_SS211647","PASS",-1.6152,0.4311,"Plate control","PASS","PFC",1.34826118284201,"1h","1h"
7203,"PFC_22",63,"OID30316","Q13428","TCOF1",0,"Cardiometabolic_II","B13805","20212097_SS211647","PASS",-1.6152,0.5554,"Plate control","PASS","PFC",1.46957602979122,"24h","24h"
7204,"PFC_07",73,"OID30316","Q13428","TCOF1",0,"Cardiometabolic_II","B13805","20212097_SS211647","PASS",-1.6152,0.1956,"Plate control","PASS","PFC",1.14520033700458,"Baseline","Baseline"
7205,"PFC_15",74,"OID30316","Q13428","TCOF1",0,"Cardiometabolic_II","B13805","20212097_SS211647","PASS",-1.6152,-0.0534,"Plate control","PASS","PFC",0.963662586695661,"1h","1h"
7206,"PFC_23",75,"OID30316","Q13428","TCOF1",0,"Cardiometabolic_II","B13805","20212097_SS211647","PASS",-1.6152,0.5436,"Plate control","PASS","PFC",1.45760518881574,"24h","24h"
7207,"PFC_08",85,"OID30316","Q13428","TCOF1",0,"Cardiometabolic_II","B13805","20212097_SS211647","PASS",-1.6152,-0.0389,"Plate control","PASS","PFC",0.973396842547633,"Baseline","Baseline"
7208,"PFC_16",86,"OID30316","Q13428","TCOF1",0,"Cardiometabolic_II","B13805","20212097_SS211647","PASS",-1.6152,-0.0197,"Plate control","PASS","PFC",0.986437807143117,"1h","1h"
7209,"PFC_24",87,"OID30316","Q13428","TCOF1",0,"Cardiometabolic_II","B13805","20212097_SS211647","PASS",-1.6152,0.0096,"Plate control","PASS","PFC",1.00667640139654,"24h","24h"
7210,"PFC_01",1,"OID30317","P14854","COX6B1",0.0455,"Cardiometabolic_II","B13805","20212097_SS211647","PASS",0.4232,1.4514,"Plate control","PASS","PFC",2.73473302777559,"Baseline","Baseline"
7211,"PFC_09",2,"OID30317","P14854","COX6B1",0.0455,"Cardiometabolic_II","B13805","20212097_SS211647","PASS",0.4232,1.9835,"Plate control","PASS","PFC",3.95451289826862,"Baseline","Baseline"
7212,"PFC_17",3,"OID30317","P14854","COX6B1",0.0455,"Cardiometabolic_II","B13805","20212097_SS211647","PASS",0.4232,3.4657,"Plate control","PASS","PFC",11.0478980004694,"1h","1h"
7213,"PFC_25",4,"OID30317","P14854","COX6B1",0.0455,"Cardiometabolic_II","B13805","20212097_SS211647","PASS",0.4232,2.6176,"Plate control","PASS","PFC",6.13728253205432,"24h","24h"
7214,"PFC_02",13,"OID30317","P14854","COX6B1",0.0455,"Cardiometabolic_II","B13805","20212097_SS211647","PASS",0.4232,1.6098,"Plate control","PASS","PFC",3.0520952783446,"Baseline","Baseline"
7215,"PFC_10",14,"OID30317","P14854","COX6B1",0.0455,"Cardiometabolic_II","B13805","20212097_SS211647","PASS",0.4232,2.11,"Plate control","PASS","PFC",4.31691294601771,"1h","1h"
7216,"PFC_18",15,"OID30317","P14854","COX6B1",0.0455,"Cardiometabolic_II","B13805","20212097_SS211647","PASS",0.4232,2.3969,"Plate control","PASS","PFC",5.26670261557778,"1h","1h"
7217,"PFC_26",16,"OID30317","P14854","COX6B1",0.0455,"Cardiometabolic_II","B13805","20212097_SS211647","PASS",0.4232,3.2796,"Plate control","PASS","PFC",9.71086627804411,"24h","24h"
7218,"PFC_03",25,"OID30317","P14854","COX6B1",0.0455,"Cardiometabolic_II","B13805","20212097_SS211647","PASS",0.4232,0.5354,"Plate control","PASS","PFC",1.44934394231961,"Baseline","Baseline"
7219,"PFC_11",26,"OID30317","P14854","COX6B1",0.0455,"Cardiometabolic_II","B13805","20212097_SS211647","PASS",0.4232,2.9118,"Plate control","PASS","PFC",7.52556552288848,"1h","1h"
7220,"PFC_19",27,"OID30317","P14854","COX6B1",0.0455,"Cardiometabolic_II","B13805","20212097_SS211647","PASS",0.4232,2.258,"Plate control","PASS","PFC",4.7832791871045,"24h","24h"
7221,"PFC_27",28,"OID30317","P14854","COX6B1",0.0455,"Cardiometabolic_II","B13805","20212097_SS211647","PASS",0.4232,3.4116,"Plate control","PASS","PFC",10.6412815298808,"24h","24h"
7222,"PFC_04",37,"OID30317","P14854","COX6B1",0.0455,"Cardiometabolic_II","B13805","20212097_SS211647","PASS",0.4232,1.2616,"Plate control","PASS","PFC",2.3976149753943,"Baseline","Baseline"
7223,"PFC_12",38,"OID30317","P14854","COX6B1",0.0455,"Cardiometabolic_II","B13805","20212097_SS211647","PASS",0.4232,0.1063,"Plate control","PASS","PFC",1.07646394589657,"1h","1h"
7224,"PFC_20",39,"OID30317","P14854","COX6B1",0.0455,"Cardiometabolic_II","B13805","20212097_SS211647","PASS",0.4232,3.3647,"Plate control","PASS","PFC",10.3009107991702,"24h","24h"
7225,"PFC_05",49,"OID30317","P14854","COX6B1",0.0455,"Cardiometabolic_II","B13805","20212097_SS211647","PASS",0.4232,0.5443,"Plate control","PASS","PFC",1.45831259486854,"Baseline","Baseline"
7226,"PFC_13",50,"OID30317","P14854","COX6B1",0.0455,"Cardiometabolic_II","B13805","20212097_SS211647","PASS",0.4232,1.5826,"Plate control","PASS","PFC",2.99509133807107,"1h","1h"
7227,"PFC_21",51,"OID30317","P14854","COX6B1",0.0455,"Cardiometabolic_II","B13805","20212097_SS211647","PASS",0.4232,0.9025,"Plate control","PASS","PFC",1.86930243237064,"24h","24h"
7228,"PFC_06",61,"OID30317","P14854","COX6B1",0.0455,"Cardiometabolic_II","B13805","20212097_SS211647","PASS",0.4232,1.7057,"Plate control","PASS","PFC",3.2618716156941,"Baseline","Baseline"
7229,"PFC_14",62,"OID30317","P14854","COX6B1",0.0455,"Cardiometabolic_II","B13805","20212097_SS211647","PASS",0.4232,2.3206,"Plate control","PASS","PFC",4.99539929181743,"1h","1h"
7230,"PFC_22",63,"OID30317","P14854","COX6B1",0.0455,"Cardiometabolic_II","B13805","20212097_SS211647","PASS",0.4232,1.5154,"Plate control","PASS","PFC",2.85878079194986,"24h","24h"
7231,"PFC_07",73,"OID30317","P14854","COX6B1",0.0455,"Cardiometabolic_II","B13805","20212097_SS211647","PASS",0.4232,2.9569,"Plate control","PASS","PFC",7.76453754277285,"Baseline","Baseline"
7232,"PFC_15",74,"OID30317","P14854","COX6B1",0.0455,"Cardiometabolic_II","B13805","20212097_SS211647","PASS",0.4232,0.3486,"Plate control","PASS","PFC",1.27332438589696,"1h","1h"
7233,"PFC_23",75,"OID30317","P14854","COX6B1",0.0455,"Cardiometabolic_II","B13805","20212097_SS211647","PASS",0.4232,2.7163,"Plate control","PASS","PFC",6.57185204215462,"24h","24h"
7234,"PFC_08",85,"OID30317","P14854","COX6B1",0.0455,"Cardiometabolic_II","B13805","20212097_SS211647","PASS",0.4232,0.8748,"Plate control","PASS","PFC",1.83375385652478,"Baseline","Baseline"
7235,"PFC_16",86,"OID30317","P14854","COX6B1",0.0455,"Cardiometabolic_II","B13805","20212097_SS211647","PASS",0.4232,1.5547,"Plate control","PASS","PFC",2.93772632002265,"1h","1h"
7236,"PFC_24",87,"OID30317","P14854","COX6B1",0.0455,"Cardiometabolic_II","B13805","20212097_SS211647","PASS",0.4232,2.5127,"Plate control","PASS","PFC",5.70687118897587,"24h","24h"
7237,"PFC_01",1,"OID30318","P16035","TIMP2",0,"Cardiometabolic_II","B13805","20212097_SS211647","PASS",-0.5561,0.4344,"Plate control","PASS","PFC",1.35134870601788,"Baseline","Baseline"
7238,"PFC_09",2,"OID30318","P16035","TIMP2",0,"Cardiometabolic_II","B13805","20212097_SS211647","PASS",-0.5561,0.2989,"Plate control","PASS","PFC",1.23020607040887,"Baseline","Baseline"
7239,"PFC_17",3,"OID30318","P16035","TIMP2",0,"Cardiometabolic_II","B13805","20212097_SS211647","PASS",-0.5561,-0.1705,"Plate control","PASS","PFC",0.888534685142091,"1h","1h"
7240,"PFC_25",4,"OID30318","P16035","TIMP2",0,"Cardiometabolic_II","B13805","20212097_SS211647","PASS",-0.5561,0.6189,"Plate control","PASS","PFC",1.5357038191356,"24h","24h"
7241,"PFC_02",13,"OID30318","P16035","TIMP2",0,"Cardiometabolic_II","B13805","20212097_SS211647","PASS",-0.5561,0.2154,"Plate control","PASS","PFC",1.16102577445923,"Baseline","Baseline"
7242,"PFC_10",14,"OID30318","P16035","TIMP2",0,"Cardiometabolic_II","B13805","20212097_SS211647","PASS",-0.5561,0.5843,"Plate control","PASS","PFC",1.49931134237103,"1h","1h"
7243,"PFC_18",15,"OID30318","P16035","TIMP2",0,"Cardiometabolic_II","B13805","20212097_SS211647","PASS",-0.5561,0.195,"Plate control","PASS","PFC",1.14472416059868,"1h","1h"
7244,"PFC_26",16,"OID30318","P16035","TIMP2",0,"Cardiometabolic_II","B13805","20212097_SS211647","PASS",-0.5561,0.2047,"Plate control","PASS","PFC",1.15244667733125,"24h","24h"
7245,"PFC_03",25,"OID30318","P16035","TIMP2",0,"Cardiometabolic_II","B13805","20212097_SS211647","PASS",-0.5561,0.1185,"Plate control","PASS","PFC",1.08560554890424,"Baseline","Baseline"
7246,"PFC_11",26,"OID30318","P16035","TIMP2",0,"Cardiometabolic_II","B13805","20212097_SS211647","PASS",-0.5561,0.3856,"Plate control","PASS","PFC",1.30640299185512,"1h","1h"
7247,"PFC_19",27,"OID30318","P16035","TIMP2",0,"Cardiometabolic_II","B13805","20212097_SS211647","PASS",-0.5561,0.3758,"Plate control","PASS","PFC",1.29755887461518,"24h","24h"
7248,"PFC_27",28,"OID30318","P16035","TIMP2",0,"Cardiometabolic_II","B13805","20212097_SS211647","PASS",-0.5561,0.4768,"Plate control","PASS","PFC",1.39165345432437,"24h","24h"
7249,"PFC_04",37,"OID30318","P16035","TIMP2",0,"Cardiometabolic_II","B13805","20212097_SS211647","PASS",-0.5561,0.5124,"Plate control","PASS","PFC",1.42642115046243,"Baseline","Baseline"
7250,"PFC_12",38,"OID30318","P16035","TIMP2",0,"Cardiometabolic_II","B13805","20212097_SS211647","PASS",-0.5561,0.1681,"Plate control","PASS","PFC",1.12357778080967,"1h","1h"
7251,"PFC_20",39,"OID30318","P16035","TIMP2",0,"Cardiometabolic_II","B13805","20212097_SS211647","PASS",-0.5561,0.094,"Plate control","PASS","PFC",1.06732533803996,"24h","24h"
7252,"PFC_05",49,"OID30318","P16035","TIMP2",0,"Cardiometabolic_II","B13805","20212097_SS211647","PASS",-0.5561,0.1429,"Plate control","PASS","PFC",1.10412231258384,"Baseline","Baseline"
7253,"PFC_13",50,"OID30318","P16035","TIMP2",0,"Cardiometabolic_II","B13805","20212097_SS211647","PASS",-0.5561,0.4148,"Plate control","PASS","PFC",1.33311385545858,"1h","1h"
7254,"PFC_21",51,"OID30318","P16035","TIMP2",0,"Cardiometabolic_II","B13805","20212097_SS211647","PASS",-0.5561,0.4918,"Plate control","PASS","PFC",1.40619824593036,"24h","24h"
7255,"PFC_06",61,"OID30318","P16035","TIMP2",0,"Cardiometabolic_II","B13805","20212097_SS211647","PASS",-0.5561,0.2463,"Plate control","PASS","PFC",1.18616112904015,"Baseline","Baseline"
7256,"PFC_14",62,"OID30318","P16035","TIMP2",0,"Cardiometabolic_II","B13805","20212097_SS211647","PASS",-0.5561,0.3499,"Plate control","PASS","PFC",1.2744722845705,"1h","1h"
7257,"PFC_22",63,"OID30318","P16035","TIMP2",0,"Cardiometabolic_II","B13805","20212097_SS211647","PASS",-0.5561,0.7255,"Plate control","PASS","PFC",1.65347358729543,"24h","24h"
7258,"PFC_07",73,"OID30318","P16035","TIMP2",0,"Cardiometabolic_II","B13805","20212097_SS211647","PASS",-0.5561,0.4477,"Plate control","PASS","PFC",1.36386419780303,"Baseline","Baseline"
7259,"PFC_15",74,"OID30318","P16035","TIMP2",0,"Cardiometabolic_II","B13805","20212097_SS211647","PASS",-0.5561,0.0029,"Plate control","PASS","PFC",1.00201214848292,"1h","1h"
7260,"PFC_23",75,"OID30318","P16035","TIMP2",0,"Cardiometabolic_II","B13805","20212097_SS211647","PASS",-0.5561,0.3112,"Plate control","PASS","PFC",1.24073928878157,"24h","24h"
7261,"PFC_08",85,"OID30318","P16035","TIMP2",0,"Cardiometabolic_II","B13805","20212097_SS211647","PASS",-0.5561,0.4851,"Plate control","PASS","PFC",1.39968288085712,"Baseline","Baseline"
7262,"PFC_16",86,"OID30318","P16035","TIMP2",0,"Cardiometabolic_II","B13805","20212097_SS211647","PASS",-0.5561,0.6557,"Plate control","PASS","PFC",1.57538014694386,"1h","1h"
7263,"PFC_24",87,"OID30318","P16035","TIMP2",0,"Cardiometabolic_II","B13805","20212097_SS211647","PASS",-0.5561,0.2228,"Plate control","PASS","PFC",1.16699631056373,"24h","24h"
7264,"PFC_01",1,"OID30319","P53674","CRYBB1",0,"Cardiometabolic_II","B13805","20212097_SS211647","PASS",-4.4235,1.1642,"Plate control","PASS","PFC",2.2410890877711,"Baseline","Baseline"
7265,"PFC_09",2,"OID30319","P53674","CRYBB1",0,"Cardiometabolic_II","B13805","20212097_SS211647","PASS",-4.4235,2.352,"Plate control","PASS","PFC",5.10531507531581,"Baseline","Baseline"
7266,"PFC_17",3,"OID30319","P53674","CRYBB1",0,"Cardiometabolic_II","B13805","20212097_SS211647","PASS",-4.4235,2.9624,"Plate control","PASS","PFC",7.79419485847041,"1h","1h"
7267,"PFC_25",4,"OID30319","P53674","CRYBB1",0,"Cardiometabolic_II","B13805","20212097_SS211647","PASS",-4.4235,2.8587,"Plate control","PASS","PFC",7.25361412797383,"24h","24h"
7268,"PFC_02",13,"OID30319","P53674","CRYBB1",0,"Cardiometabolic_II","B13805","20212097_SS211647","PASS",-4.4235,1.8718,"Plate control","PASS","PFC",3.6598892689564,"Baseline","Baseline"
7269,"PFC_10",14,"OID30319","P53674","CRYBB1",0,"Cardiometabolic_II","B13805","20212097_SS211647","PASS",-4.4235,1.7458,"Plate control","PASS","PFC",3.35380776905544,"1h","1h"
7270,"PFC_18",15,"OID30319","P53674","CRYBB1",0,"Cardiometabolic_II","B13805","20212097_SS211647","PASS",-4.4235,2.6634,"Plate control","PASS","PFC",6.33524318587072,"1h","1h"
7271,"PFC_26",16,"OID30319","P53674","CRYBB1",0,"Cardiometabolic_II","B13805","20212097_SS211647","PASS",-4.4235,2.965,"Plate control","PASS","PFC",7.8082540862098,"24h","24h"
7272,"PFC_03",25,"OID30319","P53674","CRYBB1",0,"Cardiometabolic_II","B13805","20212097_SS211647","PASS",-4.4235,1.0844,"Plate control","PASS","PFC",2.12049341090997,"Baseline","Baseline"
7273,"PFC_11",26,"OID30319","P53674","CRYBB1",0,"Cardiometabolic_II","B13805","20212097_SS211647","PASS",-4.4235,3.1179,"Plate control","PASS","PFC",8.68123321696654,"1h","1h"
7274,"PFC_19",27,"OID30319","P53674","CRYBB1",0,"Cardiometabolic_II","B13805","20212097_SS211647","PASS",-4.4235,2.1661,"Plate control","PASS","PFC",4.48808500168712,"24h","24h"
7275,"PFC_27",28,"OID30319","P53674","CRYBB1",0,"Cardiometabolic_II","B13805","20212097_SS211647","PASS",-4.4235,3.753,"Plate control","PASS","PFC",13.4823492720504,"24h","24h"
7276,"PFC_04",37,"OID30319","P53674","CRYBB1",0,"Cardiometabolic_II","B13805","20212097_SS211647","PASS",-4.4235,2.8168,"Plate control","PASS","PFC",7.04597814533033,"Baseline","Baseline"
7277,"PFC_12",38,"OID30319","P53674","CRYBB1",0,"Cardiometabolic_II","B13805","20212097_SS211647","PASS",-4.4235,1.2359,"Plate control","PASS","PFC",2.35528231780202,"1h","1h"
7278,"PFC_20",39,"OID30319","P53674","CRYBB1",0,"Cardiometabolic_II","B13805","20212097_SS211647","PASS",-4.4235,3.3725,"Plate control","PASS","PFC",10.3567539913574,"24h","24h"
7279,"PFC_05",49,"OID30319","P53674","CRYBB1",0,"Cardiometabolic_II","B13805","20212097_SS211647","PASS",-4.4235,1.6319,"Plate control","PASS","PFC",3.0992088956006,"Baseline","Baseline"
7280,"PFC_13",50,"OID30319","P53674","CRYBB1",0,"Cardiometabolic_II","B13805","20212097_SS211647","PASS",-4.4235,2.8711,"Plate control","PASS","PFC",7.31622782069272,"1h","1h"

Supplemental table 1_Olink proximity extension assay (PEA) data

,"SampleID","Index","OlinkID","UniProt","Assay","MissingFreq","Panel","Panel_Lot_Nr","PlateID","QC_Warning","LOD","NPX","Normalization","Assay_Warning","condition","Value","group","alt_group"
7281,"PFC_21",51,"OID30319","P53674","CRYBB1",0,"Cardiometabolic_II","B13805","20212097_SS211647","PASS",-4.4235,1.6668,"Plate control","PASS","PFC",3.17509553151238,"24h","24h"
7282,"PFC_06",61,"OID30319","P53674","CRYBB1",0,"Cardiometabolic_II","B13805","20212097_SS211647","PASS",-4.4235,2.1594,"Plate control","PASS","PFC",4.46729027210305,"Baseline","Baseline"
7283,"PFC_14",62,"OID30319","P53674","CRYBB1",0,"Cardiometabolic_II","B13805","20212097_SS211647","PASS",-4.4235,2.7951,"Plate control","PASS","PFC",6.94079057948541,"1h","1h"
7284,"PFC_22",63,"OID30319","P53674","CRYBB1",0,"Cardiometabolic_II","B13805","20212097_SS211647","PASS",-4.4235,2.4934,"Plate control","PASS","PFC",5.63103453917158,"24h","24h"
7285,"PFC_07",73,"OID30319","P53674","CRYBB1",0,"Cardiometabolic_II","B13805","20212097_SS211647","PASS",-4.4235,3.2616,"Plate control","PASS","PFC",9.59045990157718,"Baseline","Baseline"
7286,"PFC_15",74,"OID30319","P53674","CRYBB1",0,"Cardiometabolic_II","B13805","20212097_SS211647","PASS",-4.4235,1.5638,"Plate control","PASS","PFC",2.95631500185729,"1h","1h"
7287,"PFC_23",75,"OID30319","P53674","CRYBB1",0,"Cardiometabolic_II","B13805","20212097_SS211647","PASS",-4.4235,2.916,"Plate control","PASS","PFC",7.54750600716288,"24h","24h"
7288,"PFC_08",85,"OID30319","P53674","CRYBB1",0,"Cardiometabolic_II","B13805","20212097_SS211647","PASS",-4.4235,2.0123,"Plate control","PASS","PFC",4.03424863078164,"Baseline","Baseline"
7289,"PFC_16",86,"OID30319","P53674","CRYBB1",0,"Cardiometabolic_II","B13805","20212097_SS211647","PASS",-4.4235,2.7704,"Plate control","PASS","PFC",6.82297060105261,"1h","1h"
7290,"PFC_24",87,"OID30319","P53674","CRYBB1",0,"Cardiometabolic_II","B13805","20212097_SS211647","PASS",-4.4235,2.7767,"Plate control","PASS","PFC",6.85283048393881,"24h","24h"
7291,"PFC_01",1,"OID30320","O14960","LECT2",0,"Cardiometabolic_II","B13805","20212097_SS211647","PASS",-5.3241,-2.1836,"Plate control","PASS","PFC",0.220125776755373,"Baseline","Baseline"
7292,"PFC_09",2,"OID30320","O14960","LECT2",0,"Cardiometabolic_II","B13805","20212097_SS211647","PASS",-5.3241,-2.6252,"Plate control","PASS","PFC",0.162082473371957,"Baseline","Baseline"
7293,"PFC_17",3,"OID30320","O14960","LECT2",0,"Cardiometabolic_II","B13805","20212097_SS211647","PASS",-5.3241,-4.6623,"Plate control","PASS","PFC",0.0394918838663742,"1h","1h"
7294,"PFC_25",4,"OID30320","O14960","LECT2",0,"Cardiometabolic_II","B13805","20212097_SS211647","PASS",-5.3241,-3.3439,"Plate control","PASS","PFC",0.0984885622265006,"24h","24h"
7295,"PFC_02",13,"OID30320","O14960","LECT2",0,"Cardiometabolic_II","B13805","20212097_SS211647","PASS",-5.3241,-1.356,"Plate control","PASS","PFC",0.390663940086159,"Baseline","Baseline"
7296,"PFC_10",14,"OID30320","O14960","LECT2",0,"Cardiometabolic_II","B13805","20212097_SS211647","PASS",-5.3241,-2.0347,"Plate control","PASS","PFC",0.244058685494702,"1h","1h"
7297,"PFC_18",15,"OID30320","O14960","LECT2",0,"Cardiometabolic_II","B13805","20212097_SS211647","PASS",-5.3241,3.7031,"Plate control","PASS","PFC",13.0239936842908,"1h","1h"
7298,"PFC_26",16,"OID30320","O14960","LECT2",0,"Cardiometabolic_II","B13805","20212097_SS211647","PASS",-5.3241,-4.8813,"Plate control","PASS","PFC",0.0339298767569206,"24h","24h"
7299,"PFC_03",25,"OID30320","O14960","LECT2",0,"Cardiometabolic_II","B13805","20212097_SS211647","PASS",-5.3241,-3.2758,"Plate control","PASS","PFC",0.103249020478373,"Baseline","Baseline"
7300,"PFC_11",26,"OID30320","O14960","LECT2",0,"Cardiometabolic_II","B13805","20212097_SS211647","PASS",-5.3241,-1.3707,"Plate control","PASS","PFC",0.386703573114983,"1h","1h"
7301,"PFC_19",27,"OID30320","O14960","LECT2",0,"Cardiometabolic_II","B13805","20212097_SS211647","PASS",-5.3241,-2.1072,"Plate control","PASS","PFC",0.232097035365209,"24h","24h"
7302,"PFC_27",28,"OID30320","O14960","LECT2",0,"Cardiometabolic_II","B13805","20212097_SS211647","PASS",-5.3241,-2.5446,"Plate control","PASS","PFC",0.171395364920861,"24h","24h"
7303,"PFC_04",37,"OID30320","O14960","LECT2",0,"Cardiometabolic_II","B13805","20212097_SS211647","PASS",-5.3241,-2.6197,"Plate control","PASS","PFC",0.162701561251351,"Baseline","Baseline"
7304,"PFC_12",38,"OID30320","O14960","LECT2",0,"Cardiometabolic_II","B13805","20212097_SS211647","PASS",-5.3241,-3.8271,"Plate control","PASS","PFC",0.0704576418382481,"1h","1h"
7305,"PFC_20",39,"OID30320","O14960","LECT2",0,"Cardiometabolic_II","B13805","20212097_SS211647","PASS",-5.3241,-1.7971,"Plate control","PASS","PFC",0.287752426662343,"24h","24h"
7306,"PFC_05",49,"OID30320","O14960","LECT2",0,"Cardiometabolic_II","B13805","20212097_SS211647","PASS",-5.3241,-4.0002,"Plate control","PASS","PFC",0.0624913362607815,"Baseline","Baseline"
7307,"PFC_13",50,"OID30320","O14960","LECT2",0,"Cardiometabolic_II","B13805","20212097_SS211647","PASS",-5.3241,-2.9773,"Plate control","PASS","PFC",0.126982359889193,"1h","1h"
7308,"PFC_21",51,"OID30320","O14960","LECT2",0,"Cardiometabolic_II","B13805","20212097_SS211647","PASS",-5.3241,-3.3557,"Plate control","PASS","PFC",0.0976862962039086,"24h","24h"
7309,"PFC_06",61,"OID30320","O14960","LECT2",0,"Cardiometabolic_II","B13805","20212097_SS211647","PASS",-5.3241,-2.8289,"Plate control","PASS","PFC",0.140739578253195,"Baseline","Baseline"
7310,"PFC_14",62,"OID30320","O14960","LECT2",0,"Cardiometabolic_II","B13805","20212097_SS211647","PASS",-5.3241,-3.676,"Plate control","PASS","PFC",0.0782372786658747,"1h","1h"
7311,"PFC_22",63,"OID30320","O14960","LECT2",0,"Cardiometabolic_II","B13805","20212097_SS211647","PASS",-5.3241,-3.1568,"Plate control","PASS","PFC",0.112126562922562,"24h","24h"
7312,"PFC_07",73,"OID30320","O14960","LECT2",0,"Cardiometabolic_II","B13805","20212097_SS211647","PASS",-5.3241,-3.3855,"Plate control","PASS","PFC",0.0956892057193869,"Baseline","Baseline"
7313,"PFC_15",74,"OID30320","O14960","LECT2",0,"Cardiometabolic_II","B13805","20212097_SS211647","PASS",-5.3241,-3.0683,"Plate control","PASS","PFC",0.119220150107407,"1h","1h"
7314,"PFC_23",75,"OID30320","O14960","LECT2",0,"Cardiometabolic_II","B13805","20212097_SS211647","PASS",-5.3241,-4.1982,"Plate control","PASS","PFC",0.0544773372850175,"24h","24h"
7315,"PFC_08",85,"OID30320","O14960","LECT2",0,"Cardiometabolic_II","B13805","20212097_SS211647","PASS",-5.3241,-4.9501,"Plate control","PASS","PFC",0.0323497864759997,"Baseline","Baseline"
7316,"PFC_16",86,"OID30320","O14960","LECT2",0,"Cardiometabolic_II","B13805","20212097_SS211647","PASS",-5.3241,-3.7008,"Plate control","PASS","PFC",0.0769038694480632,"1h","1h"
7317,"PFC_24",87,"OID30320","O14960","LECT2",0,"Cardiometabolic_II","B13805","20212097_SS211647","PASS",-5.3241,-2.8989,"Plate control","PASS","PFC",0.134073870072672,"24h","24h"
7318,"PFC_01",1,"OID30321","O14933","UBE2L6",0,"Cardiometabolic_II","B13805","20212097_SS211647","PASS",-1.3374,1.0948,"Plate control","PASS","PFC",2.13583470601047,"Baseline","Baseline"
7319,"PFC_09",2,"OID30321","O14933","UBE2L6",0,"Cardiometabolic_II","B13805","20212097_SS211647","PASS",-1.3374,1.5625,"Plate control","PASS","PFC",2.953652291879,"Baseline","Baseline"
7320,"PFC_17",3,"OID30321","O14933","UBE2L6",0,"Cardiometabolic_II","B13805","20212097_SS211647","PASS",-1.3374,2.329,"Plate control","PASS","PFC",5.02456952457411,"1h","1h"
7321,"PFC_25",4,"OID30321","O14933","UBE2L6",0,"Cardiometabolic_II","B13805","20212097_SS211647","PASS",-1.3374,2.257,"Plate control","PASS","PFC",4.77996481942713,"24h","24h"
7322,"PFC_02",13,"OID30321","O14933","UBE2L6",0,"Cardiometabolic_II","B13805","20212097_SS211647","PASS",-1.3374,1.5958,"Plate control","PASS","PFC",3.02262080336901,"Baseline","Baseline"
7323,"PFC_10",14,"OID30321","O14933","UBE2L6",0,"Cardiometabolic_II","B13805","20212097_SS211647","PASS",-1.3374,1.3845,"Plate control","PASS","PFC",2.61081457798116,"1h","1h"
7324,"PFC_18",15,"OID30321","O14933","UBE2L6",0,"Cardiometabolic_II","B13805","20212097_SS211647","PASS",-1.3374,1.952,"Plate control","PASS","PFC",3.86910531845573,"1h","1h"
7325,"PFC_26",16,"OID30321","O14933","UBE2L6",0,"Cardiometabolic_II","B13805","20212097_SS211647","PASS",-1.3374,2.3362,"Plate control","PASS","PFC",5.0497081181167,"24h","24h"
7326,"PFC_03",25,"OID30321","O14933","UBE2L6",0,"Cardiometabolic_II","B13805","20212097_SS211647","PASS",-1.3374,0.3927,"Plate control","PASS","PFC",1.31284809795062,"Baseline","Baseline"
7327,"PFC_11",26,"OID30321","O14933","UBE2L6",0,"Cardiometabolic_II","B13805","20212097_SS211647","PASS",-1.3374,2.2739,"Plate control","PASS","PFC",4.836287465813,"1h","1h"
7328,"PFC_19",27,"OID30321","O14933","UBE2L6",0,"Cardiometabolic_II","B13805","20212097_SS211647","PASS",-1.3374,1.7939,"Plate control","PASS","PFC",3.46750989625786,"24h","24h"
7329,"PFC_27",28,"OID30321","O14933","UBE2L6",0,"Cardiometabolic_II","B13805","20212097_SS211647","PASS",-1.3374,3.0811,"Plate control","PASS","PFC",8.46259427095441,"24h","24h"
7330,"PFC_04",37,"OID30321","O14933","UBE2L6",0,"Cardiometabolic_II","B13805","20212097_SS211647","PASS",-1.3374,2.5759,"Plate control","PASS","PFC",5.96242824883902,"Baseline","Baseline"
7331,"PFC_12",38,"OID30321","O14933","UBE2L6",0,"Cardiometabolic_II","B13805","20212097_SS211647","PASS",-1.3374,0.9124,"Plate control","PASS","PFC",1.88217399212895,"1h","1h"
7332,"PFC_20",39,"OID30321","O14933","UBE2L6",0,"Cardiometabolic_II","B13805","20212097_SS211647","PASS",-1.3374,2.319,"Plate control","PASS","PFC",4.98986228765618,"24h","24h"
7333,"PFC_05",49,"OID30321","O14933","UBE2L6",0,"Cardiometabolic_II","B13805","20212097_SS211647","PASS",-1.3374,0.8524,"Plate control","PASS","PFC",1.8055019769765,"Baseline","Baseline"
7334,"PFC_13",50,"OID30321","O14933","UBE2L6",0,"Cardiometabolic_II","B13805","20212097_SS211647","PASS",-1.3374,2.7766,"Plate control","PASS","PFC",6.85235549838787,"1h","1h"
7335,"PFC_21",51,"OID30321","O14933","UBE2L6",0,"Cardiometabolic_II","B13805","20212097_SS211647","PASS",-1.3374,1.2548,"Plate control","PASS","PFC",2.38634064607376,"24h","24h"
7336,"PFC_06",61,"OID30321","O14933","UBE2L6",0,"Cardiometabolic_II","B13805","20212097_SS211647","PASS",-1.3374,2.3008,"Plate control","PASS","PFC",4.92730917634136,"Baseline","Baseline"
7337,"PFC_14",62,"OID30321","O14933","UBE2L6",0,"Cardiometabolic_II","B13805","20212097_SS211647","PASS",-1.3374,1.9319,"Plate control","PASS","PFC",3.81557371760754,"1h","1h"
7338,"PFC_22",63,"OID30321","O14933","UBE2L6",0,"Cardiometabolic_II","B13805","20212097_SS211647","PASS",-1.3374,2.2835,"Plate control","PASS","PFC",4.86857646220383,"24h","24h"
7339,"PFC_07",73,"OID30321","O14933","UBE2L6",0,"Cardiometabolic_II","B13805","20212097_SS211647","PASS",-1.3374,2.6083,"Plate control","PASS","PFC",6.09784720120891,"Baseline","Baseline"
7340,"PFC_15",74,"OID30321","O14933","UBE2L6",0,"Cardiometabolic_II","B13805","20212097_SS211647","PASS",-1.3374,1.539,"Plate control","PASS","PFC",2.90593009914274,"1h","1h"
7341,"PFC_23",75,"OID30321","O14933","UBE2L6",0,"Cardiometabolic_II","B13805","20212097_SS211647","PASS",-1.3374,2.106,"Plate control","PASS","PFC",4.30496049913519,"24h","24h"
7342,"PFC_08",85,"OID30321","O14933","UBE2L6",0,"Cardiometabolic_II","B13805","20212097_SS211647","PASS",-1.3374,1.6696,"Plate control","PASS","PFC",3.1812637791249,"Baseline","Baseline"
7343,"PFC_16",86,"OID30321","O14933","UBE2L6",0,"Cardiometabolic_II","B13805","20212097_SS211647","PASS",-1.3374,2.5479,"Plate control","PASS","PFC",5.84782443924954,"1h","1h"
7344,"PFC_24",87,"OID30321","O14933","UBE2L6",0,"Cardiometabolic_II","B13805","20212097_SS211647","PASS",-1.3374,2.5677,"Plate control","PASS","PFC",5.92863508601475,"24h","24h"
7345,"PFC_01",1,"OID30322","Q8N436","CPXM2",0,"Cardiometabolic_II","B13805","20212097_SS211647","PASS",-1.204,0.3489,"Plate control","PASS","PFC",1.27358919379104,"Baseline","Baseline"
7346,"PFC_09",2,"OID30322","Q8N436","CPXM2",0,"Cardiometabolic_II","B13805","20212097_SS211647","PASS",-1.204,0.6372,"Plate control","PASS","PFC",1.55530766824579,"Baseline","Baseline"
7347,"PFC_17",3,"OID30322","Q8N436","CPXM2",0,"Cardiometabolic_II","B13805","20212097_SS211647","PASS",-1.204,-0.0089,"Plate control","PASS","PFC",0.993849979366229,"1h","1h"
7348,"PFC_25",4,"OID30322","Q8N436","CPXM2",0,"Cardiometabolic_II","B13805","20212097_SS211647","PASS",-1.204,0.1744,"Plate control","PASS","PFC",1.12849497933069,"24h","24h"
7349,"PFC_02",13,"OID30322","Q8N436","CPXM2",0,"Cardiometabolic_II","B13805","20212097_SS211647","PASS",-1.204,0.6636,"Plate control","PASS","PFC",1.58403037448494,"Baseline","Baseline"
7350,"PFC_10",14,"OID30322","Q8N436","CPXM2",0,"Cardiometabolic_II","B13805","20212097_SS211647","PASS",-1.204,0.4514,"Plate control","PASS","PFC",1.36736651388779,"1h","1h"
7351,"PFC_18",15,"OID30322","Q8N436","CPXM2",0,"Cardiometabolic_II","B13805","20212097_SS211647","PASS",-1.204,0.8841,"Plate control","PASS","PFC",1.84561290901248,"1h","1h"
7352,"PFC_26",16,"OID30322","Q8N436","CPXM2",0,"Cardiometabolic_II","B13805","20212097_SS211647","PASS",-1.204,-0.0103,"Plate control","PASS","PFC",0.992886009127616,"24h","24h"
7353,"PFC_03",25,"OID30322","Q8N436","CPXM2",0,"Cardiometabolic_II","B13805","20212097_SS211647","PASS",-1.204,0.2467,"Plate control","PASS","PFC",1.18649004833285,"Baseline","Baseline"
7354,"PFC_11",26,"OID30322","Q8N436","CPXM2",0,"Cardiometabolic_II","B13805","20212097_SS211647","PASS",-1.204,0.4539,"Plate control","PASS","PFC",1.3697380336694,"1h","1h"
7355,"PFC_19",27,"OID30322","Q8N436","CPXM2",0,"Cardiometabolic_II","B13805","20212097_SS211647","PASS",-1.204,0.4026,"Plate control","PASS","PFC",1.32188804913976,"24h","24h"
7356,"PFC_27",28,"OID30322","Q8N436","CPXM2",0,"Cardiometabolic_II","B13805","20212097_SS211647","PASS",-1.204,1.0147,"Plate control","PASS","PFC",2.02048270172046,"24h","24h"
7357,"PFC_04",37,"OID30322","Q8N436","CPXM2",0,"Cardiometabolic_II","B13805","20212097_SS211647","PASS",-1.204,0.3975,"Plate control","PASS","PFC",1.3172233577887,"Baseline","Baseline"
7358,"PFC_12",38,"OID30322","Q8N436","CPXM2",0,"Cardiometabolic_II","B13805","20212097_SS211647","PASS",-1.204,0.395,"Plate control","PASS","PFC",1.31494276020507,"1h","1h"
7359,"PFC_20",39,"OID30322","Q8N436","CPXM2",0,"Cardiometabolic_II","B13805","20212097_SS211647","PASS",-1.204,0.3374,"Plate control","PASS","PFC",1.26347752364025,"24h","24h"
7360,"PFC_05",49,"OID30322","Q8N436","CPXM2",0,"Cardiometabolic_II","B13805","20212097_SS211647","PASS",-1.204,0.4596,"Plate control","PASS","PFC",1.37516048983232,"Baseline","Baseline"
7361,"PFC_13",50,"OID30322","Q8N436","CPXM2",0,"Cardiometabolic_II","B13805","20212097_SS211647","PASS",-1.204,-0.0178,"Plate control","PASS","PFC",0.987737781486253,"1h","1h"
7362,"PFC_21",51,"OID30322","Q8N436","CPXM2",0,"Cardiometabolic_II","B13805","20212097_SS211647","PASS",-1.204,0.4162,"Plate control","PASS","PFC",1.33440814510465,"24h","24h"
7363,"PFC_06",61,"OID30322","Q8N436","CPXM2",0,"Cardiometabolic_II","B13805","20212097_SS211647","PASS",-1.204,0.1234,"Plate control","PASS","PFC",1.08929899128125,"Baseline","Baseline"
7364,"PFC_14",62,"OID30322","Q8N436","CPXM2",0,"Cardiometabolic_II","B13805","20212097_SS211647","PASS",-1.204,0.7366,"Plate control","PASS","PFC",1.66624436923368,"1h","1h"
7365,"PFC_22",63,"OID30322","Q8N436","CPXM2",0,"Cardiometabolic_II","B13805","20212097_SS211647","PASS",-1.204,0.1071,"Plate control","PASS","PFC",1.07706102978733,"24h","24h"
7366,"PFC_07",73,"OID30322","Q8N436","CPXM2",0,"Cardiometabolic_II","B13805","20212097_SS211647","PASS",-1.204,0.3342,"Plate control","PASS","PFC",1.26067814657019,"Baseline","Baseline"
7367,"PFC_15",74,"OID30322","Q8N436","CPXM2",0,"Cardiometabolic_II","B13805","20212097_SS211647","PASS",-1.204,-0.0833,"Plate control","PASS","PFC",0.943896121060921,"1h","1h"
7368,"PFC_23",75,"OID30322","Q8N436","CPXM2",0,"Cardiometabolic_II","B13805","20212097_SS211647","PASS",-1.204,0.7658,"Plate control","PASS","PFC",1.70031259041361,"24h","24h"
7369,"PFC_08",85,"OID30322","Q8N436","CPXM2",0,"Cardiometabolic_II","B13805","20212097_SS211647","PASS",-1.204,0.0448,"Plate control","PASS","PFC",1.03154016755701,"Baseline","Baseline"
7370,"PFC_16",86,"OID30322","Q8N436","CPXM2",0,"Cardiometabolic_II","B13805","20212097_SS211647","PASS",-1.204,0.0942,"Plate control","PASS","PFC",1.06747331100619,"1h","1h"
7371,"PFC_24",87,"OID30322","Q8N436","CPXM2",0,"Cardiometabolic_II","B13805","20212097_SS211647","PASS",-1.204,0.0337,"Plate control","PASS","PFC",1.02363401958517,"24h","24h"
7372,"PFC_01",1,"OID30323","Q13442","PDAP1",0,"Cardiometabolic_II","B13805","20212097_SS211647","PASS",-0.9555,2.0802,"Plate control","PASS","PFC",4.22865833813489,"Baseline","Baseline"
7373,"PFC_09",2,"OID30323","Q13442","PDAP1",0,"Cardiometabolic_II","B13805","20212097_SS211647","PASS",-0.9555,3.2444,"Plate control","PASS","PFC",9.47680005750639,"Baseline","Baseline"
7374,"PFC_17",3,"OID30323","Q13442","PDAP1",0,"Cardiometabolic_II","B13805","20212097_SS211647","PASS",-0.9555,4.5531,"Plate control","PASS","PFC",23.4757606660152,"1h","1h"
7375,"PFC_25",4,"OID30323","Q13442","PDAP1",0,"Cardiometabolic_II","B13805","20212097_SS211647","PASS",-0.9555,4.2628,"Plate control","PASS","PFC",19.1968806807551,"24h","24h"
7376,"PFC_02",13,"OID30323","Q13442","PDAP1",0,"Cardiometabolic_II","B13805","20212097_SS211647","PASS",-0.9555,2.5905,"Plate control","PASS","PFC",6.02307406636425,"Baseline","Baseline"
7377,"PFC_10",14,"OID30323","Q13442","PDAP1",0,"Cardiometabolic_II","B13805","20212097_SS211647","PASS",-0.9555,3.2337,"Plate control","PASS","PFC",9.40677371533186,"1h","1h"
7378,"PFC_18",15,"OID30323","Q13442","PDAP1",0,"Cardiometabolic_II","B13805","20212097_SS211647","PASS",-0.9555,4.0468,"Plate control","PASS","PFC",16.5275388407349,"1h","1h"
7379,"PFC_26",16,"OID30323","Q13442","PDAP1",0,"Cardiometabolic_II","B13805","20212097_SS211647","PASS",-0.9555,4.6501,"Plate control","PASS","PFC",25.1084314562275,"24h","24h"
7380,"PFC_03",25,"OID30323","Q13442","PDAP1",0,"Cardiometabolic_II","B13805","20212097_SS211647","PASS",-0.9555,4.8226,"Plate control","PASS","PFC",28.2974471245847,"Baseline","Baseline"
7381,"PFC_11",26,"OID30323","Q13442","PDAP1",0,"Cardiometabolic_II","B13805","20212097_SS211647","PASS",-0.9555,3.9714,"Plate control","PASS","PFC",15.6859391084041,"1h","1h"
7382,"PFC_19",27,"OID30323","Q13442","PDAP1",0,"Cardiometabolic_II","B13805","20212097_SS211647","PASS",-0.9555,3.9123,"Plate control","PASS","PFC",15.0563482743259,"24h","24h"
7383,"PFC_27",28,"OID30323","Q13442","PDAP1",0,"Cardiometabolic_II","B13805","20212097_SS211647","PASS",-0.9555,5.0316,"Plate control","PASS","PFC",32.7086429607448,"24h","24h"
7384,"PFC_04",37,"OID30323","Q13442","PDAP1",0,"Cardiometabolic_II","B13805","20212097_SS211647","PASS",-0.9555,3.8614,"Plate control","PASS","PFC",14.5344039173865,"Baseline","Baseline"
7385,"PFC_12",38,"OID30323","Q13442","PDAP1",0,"Cardiometabolic_II","B13805","20212097_SS211647","PASS",-0.9555,4.9588,"Plate control","PASS","PFC",31.099080068623,"1h","1h"
7386,"PFC_20",39,"OID30323","Q13442","PDAP1",0,"Cardiometabolic_II","B13805","20212097_SS211647","PASS",-0.9555,4.2838,"Plate control","PASS","PFC",19.4783558379442,"24h","24h"
7387,"PFC_05",49,"OID30323","Q13442","PDAP1",0,"Cardiometabolic_II","B13805","20212097_SS211647","PASS",-0.9555,2.4657,"Plate control","PASS","PFC",5.52394900023472,"Baseline","Baseline"
7388,"PFC_13",50,"OID30323","Q13442","PDAP1",0,"Cardiometabolic_II","B13805","20212097_SS211647","PASS",-0.9555,4.1068,"Plate control","PASS","PFC",17.2293933524378,"1h","1h"
7389,"PFC_21",51,"OID30323","Q13442","PDAP1",0,"Cardiometabolic_II","B13805","20212097_SS211647","PASS",-0.9555,5.0447,"Plate control","PASS","PFC",33.0069974118116,"24h","24h"
7390,"PFC_06",61,"OID30323","Q13442","PDAP1",0,"Cardiometabolic_II","B13805","20212097_SS211647","PASS",-0.9555,3.9472,"Plate control","PASS","PFC",15.4250151367877,"Baseline","Baseline"
7391,"PFC_14",62,"OID30323","Q13442","PDAP1",0,"Cardiometabolic_II","B13805","20212097_SS211647","PASS",-0.9555,3.6436,"Plate control","PASS","PFC",12.4977804818233,"1h","1h"
7392,"PFC_22",63,"OID30323","Q13442","PDAP1",0,"Cardiometabolic_II","B13805","20212097_SS211647","PASS",-0.9555,3.7568,"Plate control","PASS","PFC",13.5179080407307,"24h","24h"

Supplemental table 1_Olink proximity extension assay (PEA) data

,"SampleID","Index","OlinkID","UniProt","Assay","MissingFreq","Panel","Panel_Lot_Nr","PlateID","QC_Warning","LOD","NPX","Normalization","Assay_Warning","condition","Value","group","alt_group"
7393,"PFC_07",73,"OID30323","Q13442","PDAP1",0,"Cardiometabolic_II","B13805","20212097_SS211647","PASS",-0.9555,4.6593,"Plate control","PASS","PFC",25.2690583811005,"Baseline","Baseline"
7394,"PFC_15",74,"OID30323","Q13442","PDAP1",0,"Cardiometabolic_II","B13805","20212097_SS211647","PASS",-0.9555,2.4834,"Plate control","PASS","PFC",5.59213814239804,"1h","1h"
7395,"PFC_23",75,"OID30323","Q13442","PDAP1",0,"Cardiometabolic_II","B13805","20212097_SS211647","PASS",-0.9555,4.0026,"Plate control","PASS","PFC",16.028860921226,"24h","24h"
7396,"PFC_08",85,"OID30323","Q13442","PDAP1",0,"Cardiometabolic_II","B13805","20212097_SS211647","PASS",-0.9555,3.6905,"Plate control","PASS","PFC",12.9107418944395,"Baseline","Baseline"
7397,"PFC_16",86,"OID30323","Q13442","PDAP1",0,"Cardiometabolic_II","B13805","20212097_SS211647","PASS",-0.9555,3.9175,"Plate control","PASS","PFC",15.1107147736811,"1h","1h"
7398,"PFC_24",87,"OID30323","Q13442","PDAP1",0,"Cardiometabolic_II","B13805","20212097_SS211647","PASS",-0.9555,4.3311,"Plate control","PASS","PFC",20.1275546368005,"24h","24h"
7399,"PFC_01",1,"OID30324","P23467","PTPRB",0,"Cardiometabolic_II","B13805","20212097_SS211647","PASS",-3.194,0.5136,"Plate control","PASS","PFC",1.42760810779416,"Baseline","Baseline"
7400,"PFC_09",2,"OID30324","P23467","PTPRB",0,"Cardiometabolic_II","B13805","20212097_SS211647","PASS",-3.194,0.8866,"Plate control","PASS","PFC",1.84881388510664,"Baseline","Baseline"
7401,"PFC_17",3,"OID30324","P23467","PTPRB",0,"Cardiometabolic_II","B13805","20212097_SS211647","PASS",-3.194,-0.1332,"Plate control","PASS","PFC",0.911806753501677,"1h","1h"
7402,"PFC_25",4,"OID30324","P23467","PTPRB",0,"Cardiometabolic_II","B13805","20212097_SS211647","PASS",-3.194,0.0526,"Plate control","PASS","PFC",1.03713234255481,"24h","24h"
7403,"PFC_02",13,"OID30324","P23467","PTPRB",0,"Cardiometabolic_II","B13805","20212097_SS211647","PASS",-3.194,0.2224,"Plate control","PASS","PFC",1.16667279533358,"Baseline","Baseline"
7404,"PFC_10",14,"OID30324","P23467","PTPRB",0,"Cardiometabolic_II","B13805","20212097_SS211647","PASS",-3.194,0.4123,"Plate control","PASS","PFC",1.33080574558527,"1h","1h"
7405,"PFC_18",15,"OID30324","P23467","PTPRB",0,"Cardiometabolic_II","B13805","20212097_SS211647","PASS",-3.194,0.6932,"Plate control","PASS","PFC",1.61686586727874,"1h","1h"
7406,"PFC_26",16,"OID30324","P23467","PTPRB",0,"Cardiometabolic_II","B13805","20212097_SS211647","PASS",-3.194,-0.1699,"Plate control","PASS","PFC",0.888904293181696,"24h","24h"
7407,"PFC_03",25,"OID30324","P23467","PTPRB",0,"Cardiometabolic_II","B13805","20212097_SS211647","PASS",-3.194,0.02,"Plate control","PASS","PFC",1.01395947979003,"Baseline","Baseline"
7408,"PFC_11",26,"OID30324","P23467","PTPRB",0,"Cardiometabolic_II","B13805","20212097_SS211647","PASS",-3.194,0.2163,"Plate control","PASS","PFC",1.16175028599055,"1h","1h"
7409,"PFC_19",27,"OID30324","P23467","PTPRB",0,"Cardiometabolic_II","B13805","20212097_SS211647","PASS",-3.194,0.7273,"Plate control","PASS","PFC",1.65553785578464,"24h","24h"
7410,"PFC_27",28,"OID30324","P23467","PTPRB",0,"Cardiometabolic_II","B13805","20212097_SS211647","PASS",-3.194,1.3291,"Plate control","PASS","PFC",2.51245890663234,"24h","24h"
7411,"PFC_04",37,"OID30324","P23467","PTPRB",0,"Cardiometabolic_II","B13805","20212097_SS211647","PASS",-3.194,0.3589,"Plate control","PASS","PFC",1.28244770718015,"Baseline","Baseline"
7412,"PFC_12",38,"OID30324","P23467","PTPRB",0,"Cardiometabolic_II","B13805","20212097_SS211647","PASS",-3.194,-0.086,"Plate control","PASS","PFC",0.942131273904809,"1h","1h"
7413,"PFC_20",39,"OID30324","P23467","PTPRB",0,"Cardiometabolic_II","B13805","20212097_SS211647","PASS",-3.194,0.3477,"Plate control","PASS","PFC",1.27253029252611,"24h","24h"
7414,"PFC_05",49,"OID30324","P23467","PTPRB",0,"Cardiometabolic_II","B13805","20212097_SS211647","PASS",-3.194,0.5557,"Plate control","PASS","PFC",1.46988165131072,"Baseline","Baseline"
7415,"PFC_13",50,"OID30324","P23467","PTPRB",0,"Cardiometabolic_II","B13805","20212097_SS211647","PASS",-3.194,0.362,"Plate control","PASS","PFC",1.28520633746937,"1h","1h"
7416,"PFC_21",51,"OID30324","P23467","PTPRB",0,"Cardiometabolic_II","B13805","20212097_SS211647","PASS",-3.194,-0.0669,"Plate control","PASS","PFC",0.954687185719468,"24h","24h"
7417,"PFC_06",61,"OID30324","P23467","PTPRB",0,"Cardiometabolic_II","B13805","20212097_SS211647","PASS",-3.194,0.6648,"Plate control","PASS","PFC",1.58534848202005,"Baseline","Baseline"
7418,"PFC_14",62,"OID30324","P23467","PTPRB",0,"Cardiometabolic_II","B13805","20212097_SS211647","PASS",-3.194,0.8262,"Plate control","PASS","PFC",1.77300917261539,"1h","1h"
7419,"PFC_22",63,"OID30324","P23467","PTPRB",0,"Cardiometabolic_II","B13805","20212097_SS211647","PASS",-3.194,0.3868,"Plate control","PASS","PFC",1.30749007936,"24h","24h"
7420,"PFC_07",73,"OID30324","P23467","PTPRB",0,"Cardiometabolic_II","B13805","20212097_SS211647","PASS",-3.194,0.2957,"Plate control","PASS","PFC",1.22748040999901,"Baseline","Baseline"
7421,"PFC_15",74,"OID30324","P23467","PTPRB",0,"Cardiometabolic_II","B13805","20212097_SS211647","PASS",-3.194,0.5878,"Plate control","PASS","PFC",1.50295311008078,"1h","1h"
7422,"PFC_23",75,"OID30324","P23467","PTPRB",0,"Cardiometabolic_II","B13805","20212097_SS211647","PASS",-3.194,0.7224,"Plate control","PASS","PFC",1.64992449001248,"24h","24h"
7423,"PFC_08",85,"OID30324","P23467","PTPRB",0,"Cardiometabolic_II","B13805","20212097_SS211647","PASS",-3.194,-0.3643,"Plate control","PASS","PFC",0.776845711462792,"Baseline","Baseline"
7424,"PFC_16",86,"OID30324","P23467","PTPRB",0,"Cardiometabolic_II","B13805","20212097_SS211647","PASS",-3.194,0.0848,"Plate control","PASS","PFC",1.06054070901648,"1h","1h"
7425,"PFC_24",87,"OID30324","P23467","PTPRB",0,"Cardiometabolic_II","B13805","20212097_SS211647","PASS",-3.194,0.6039,"Plate control","PASS","PFC",1.51981950688981,"24h","24h"
7426,"PFC_01",1,"OID30325","O75154","RAB11FIP3",0,"Cardiometabolic_II","B13805","20212097_SS211647","PASS",-1.0211,3.4448,"Plate control","PASS","PFC",10.8890032909718,"Baseline","Baseline"
7427,"PFC_09",2,"OID30325","O75154","RAB11FIP3",0,"Cardiometabolic_II","B13805","20212097_SS211647","PASS",-1.0211,4.6907,"Plate control","PASS","PFC",25.8250636547479,"Baseline","Baseline"
7428,"PFC_17",3,"OID30325","O75154","RAB11FIP3",0,"Cardiometabolic_II","B13805","20212097_SS211647","PASS",-1.0211,5.1091,"Plate control","PASS","PFC",34.5137660432755,"1h","1h"
7429,"PFC_25",4,"OID30325","O75154","RAB11FIP3",0,"Cardiometabolic_II","B13805","20212097_SS211647","PASS",-1.0211,4.8969,"Plate control","PASS","PFC",29.7929690717438,"24h","24h"
7430,"PFC_02",13,"OID30325","O75154","RAB11FIP3",0,"Cardiometabolic_II","B13805","20212097_SS211647","PASS",-1.0211,3.9445,"Plate control","PASS","PFC",15.3961742574899,"Baseline","Baseline"
7431,"PFC_10",14,"OID30325","O75154","RAB11FIP3",0,"Cardiometabolic_II","B13805","20212097_SS211647","PASS",-1.0211,4.1513,"Plate control","PASS","PFC",17.7691159376937,"1h","1h"
7432,"PFC_18",15,"OID30325","O75154","RAB11FIP3",0,"Cardiometabolic_II","B13805","20212097_SS211647","PASS",-1.0211,5.0873,"Plate control","PASS","PFC",33.9961625361563,"1h","1h"
7433,"PFC_26",16,"OID30325","O75154","RAB11FIP3",0,"Cardiometabolic_II","B13805","20212097_SS211647","PASS",-1.0211,4.9657,"Plate control","PASS","PFC",31.248174375957,"24h","24h"
7434,"PFC_03",25,"OID30325","O75154","RAB11FIP3",0,"Cardiometabolic_II","B13805","20212097_SS211647","PASS",-1.0211,2.6038,"Plate control","PASS","PFC",6.07885665872046,"Baseline","Baseline"
7435,"PFC_11",26,"OID30325","O75154","RAB11FIP3",0,"Cardiometabolic_II","B13805","20212097_SS211647","PASS",-1.0211,5.4844,"Plate control","PASS","PFC",44.768125287,"1h","1h"
7436,"PFC_19",27,"OID30325","O75154","RAB11FIP3",0,"Cardiometabolic_II","B13805","20212097_SS211647","PASS",-1.0211,4.8868,"Plate control","PASS","PFC",29.5851232463871,"24h","24h"
7437,"PFC_27",28,"OID30325","O75154","RAB11FIP3",0,"Cardiometabolic_II","B13805","20212097_SS211647","PASS",-1.0211,6.3571,"Plate control","PASS","PFC",81.974312954576,"24h","24h"
7438,"PFC_04",37,"OID30325","O75154","RAB11FIP3",0,"Cardiometabolic_II","B13805","20212097_SS211647","PASS",-1.0211,4.269,"Plate control","PASS","PFC",19.2795570399166,"Baseline","Baseline"
7439,"PFC_12",38,"OID30325","O75154","RAB11FIP3",0,"Cardiometabolic_II","B13805","20212097_SS211647","PASS",-1.0211,3.2956,"Plate control","PASS","PFC",9.81916264391306,"1h","1h"
7440,"PFC_20",39,"OID30325","O75154","RAB11FIP3",0,"Cardiometabolic_II","B13805","20212097_SS211647","PASS",-1.0211,6.1375,"Plate control","PASS","PFC",70.3998280498058,"24h","24h"
7441,"PFC_05",49,"OID30325","O75154","RAB11FIP3",0,"Cardiometabolic_II","B13805","20212097_SS211647","PASS",-1.0211,2.7951,"Plate control","PASS","PFC",6.94079057948541,"Baseline","Baseline"
7442,"PFC_13",50,"OID30325","O75154","RAB11FIP3",0,"Cardiometabolic_II","B13805","20212097_SS211647","PASS",-1.0211,4.9457,"Plate control","PASS","PFC",30.8179715252801,"1h","1h"
7443,"PFC_21",51,"OID30325","O75154","RAB11FIP3",0,"Cardiometabolic_II","B13805","20212097_SS211647","PASS",-1.0211,4.131,"Plate control","PASS","PFC",17.5208395326972,"24h","24h"
7444,"PFC_06",61,"OID30325","O75154","RAB11FIP3",0,"Cardiometabolic_II","B13805","20212097_SS211647","PASS",-1.0211,4.6133,"Plate control","PASS","PFC",24.4760695725635,"Baseline","Baseline"
7445,"PFC_14",62,"OID30325","O75154","RAB11FIP3",0,"Cardiometabolic_II","B13805","20212097_SS211647","PASS",-1.0211,4.8307,"Plate control","PASS","PFC",28.4567697590059,"1h","1h"
7446,"PFC_22",63,"OID30325","O75154","RAB11FIP3",0,"Cardiometabolic_II","B13805","20212097_SS211647","PASS",-1.0211,4.3335,"Plate control","PASS","PFC",20.1610657614375,"24h","24h"
7447,"PFC_07",73,"OID30325","O75154","RAB11FIP3",0,"Cardiometabolic_II","B13805","20212097_SS211647","PASS",-1.0211,5.1104,"Plate control","PASS","PFC",34.5448801149908,"Baseline","Baseline"
7448,"PFC_15",74,"OID30325","O75154","RAB11FIP3",0,"Cardiometabolic_II","B13805","20212097_SS211647","PASS",-1.0211,3.8086,"Plate control","PASS","PFC",14.0120875268671,"1h","1h"
7449,"PFC_23",75,"OID30325","O75154","RAB11FIP3",0,"Cardiometabolic_II","B13805","20212097_SS211647","PASS",-1.0211,4.7607,"Plate control","PASS","PFC",27.1090001740166,"24h","24h"
7450,"PFC_08",85,"OID30325","O75154","RAB11FIP3",0,"Cardiometabolic_II","B13805","20212097_SS211647","PASS",-1.0211,3.3936,"Plate control","PASS","PFC",10.509338805793,"Baseline","Baseline"
7451,"PFC_16",86,"OID30325","O75154","RAB11FIP3",0,"Cardiometabolic_II","B13805","20212097_SS211647","PASS",-1.0211,4.874,"Plate control","PASS","PFC",29.3237966296112,"1h","1h"
7452,"PFC_24",87,"OID30325","O75154","RAB11FIP3",0,"Cardiometabolic_II","B13805","20212097_SS211647","PASS",-1.0211,5.5814,"Plate control","PASS","PFC",47.8816180307926,"24h","24h"
7453,"PFC_01",1,"OID30326","Q6NUS6","TCTN3",0,"Cardiometabolic_II","B13805","20212097_SS211647","PASS",-3.7775,0.1352,"Plate control","PASS","PFC",1.09824505232675,"Baseline","Baseline"
7454,"PFC_09",2,"OID30326","Q6NUS6","TCTN3",0,"Cardiometabolic_II","B13805","20212097_SS211647","PASS",-3.7775,0.6268,"Plate control","PASS","PFC",1.54413618861608,"Baseline","Baseline"
7455,"PFC_17",3,"OID30326","Q6NUS6","TCTN3",0,"Cardiometabolic_II","B13805","20212097_SS211647","PASS",-3.7775,-0.1919,"Plate control","PASS","PFC",0.875452009300441,"1h","1h"
7456,"PFC_25",4,"OID30326","Q6NUS6","TCTN3",0,"Cardiometabolic_II","B13805","20212097_SS211647","PASS",-3.7775,0.0328,"Plate control","PASS","PFC",1.02299564259487,"24h","24h"
7457,"PFC_02",13,"OID30326","Q6NUS6","TCTN3",0,"Cardiometabolic_II","B13805","20212097_SS211647","PASS",-3.7775,0.2942,"Plate control","PASS","PFC",1.22620483635605,"Baseline","Baseline"
7458,"PFC_10",14,"OID30326","Q6NUS6","TCTN3",0,"Cardiometabolic_II","B13805","20212097_SS211647","PASS",-3.7775,0.2461,"Plate control","PASS","PFC",1.18599670358906,"1h","1h"
7459,"PFC_18",15,"OID30326","Q6NUS6","TCTN3",0,"Cardiometabolic_II","B13805","20212097_SS211647","PASS",-3.7775,0.4859,"Plate control","PASS","PFC",1.40045924508513,"1h","1h"
7460,"PFC_26",16,"OID30326","Q6NUS6","TCTN3",0,"Cardiometabolic_II","B13805","20212097_SS211647","PASS",-3.7775,-0.2198,"Plate control","PASS","PFC",0.858684467130461,"24h","24h"
7461,"PFC_03",25,"OID30326","Q6NUS6","TCTN3",0,"Cardiometabolic_II","B13805","20212097_SS211647","PASS",-3.7775,-0.3207,"Plate control","PASS","PFC",0.800681290340142,"Baseline","Baseline"
7462,"PFC_11",26,"OID30326","Q6NUS6","TCTN3",0,"Cardiometabolic_II","B13805","20212097_SS211647","PASS",-3.7775,0.4042,"Plate control","PASS","PFC",1.32335488313374,"1h","1h"
7463,"PFC_19",27,"OID30326","Q6NUS6","TCTN3",0,"Cardiometabolic_II","B13805","20212097_SS211647","PASS",-3.7775,0.1858,"Plate control","PASS","PFC",1.13744753323206,"24h","24h"
7464,"PFC_27",28,"OID30326","Q6NUS6","TCTN3",0,"Cardiometabolic_II","B13805","20212097_SS211647","PASS",-3.7775,0.9635,"Plate control","PASS","PFC",1.95003497530667,"24h","24h"
7465,"PFC_04",37,"OID30326","Q6NUS6","TCTN3",0,"Cardiometabolic_II","B13805","20212097_SS211647","PASS",-3.7775,-0.2163,"Plate control","PASS","PFC",0.860770177600917,"Baseline","Baseline"
7466,"PFC_12",38,"OID30326","Q6NUS6","TCTN3",0,"Cardiometabolic_II","B13805","20212097_SS211647","PASS",-3.7775,-0.2472,"Plate control","PASS","PFC",0.84253002194506,"1h","1h"
7467,"PFC_20",39,"OID30326","Q6NUS6","TCTN3",0,"Cardiometabolic_II","B13805","20212097_SS211647","PASS",-3.7775,0.3265,"Plate control","PASS","PFC",1.25396753717178,"24h","24h"
7468,"PFC_05",49,"OID30326","Q6NUS6","TCTN3",0,"Cardiometabolic_II","B13805","20212097_SS211647","PASS",-3.7775,0.3561,"Plate control","PASS","PFC",1.27996113091448,"Baseline","Baseline"
7469,"PFC_13",50,"OID30326","Q6NUS6","TCTN3",0,"Cardiometabolic_II","B13805","20212097_SS211647","PASS",-3.7775,-0.4725,"Plate control","PASS","PFC",0.72071461179848,"1h","1h"
7470,"PFC_21",51,"OID30326","Q6NUS6","TCTN3",0,"Cardiometabolic_II","B13805","20212097_SS211647","PASS",-3.7775,-0.1938,"Plate control","PASS","PFC",0.874299815700005,"24h","24h"
7471,"PFC_06",61,"OID30326","Q6NUS6","TCTN3",0,"Cardiometabolic_II","B13805","20212097_SS211647","PASS",-3.7775,0.0565,"Plate control","PASS","PFC",1.03993978839336,"Baseline","Baseline"
7472,"PFC_14",62,"OID30326","Q6NUS6","TCTN3",0,"Cardiometabolic_II","B13805","20212097_SS211647","PASS",-3.7775,0.5045,"Plate control","PASS","PFC",1.41863161074169,"1h","1h"
7473,"PFC_22",63,"OID30326","Q6NUS6","TCTN3",0,"Cardiometabolic_II","B13805","20212097_SS211647","PASS",-3.7775,-0.2535,"Plate control","PASS","PFC",0.838858860394702,"24h","24h"
7474,"PFC_07",73,"OID30326","Q6NUS6","TCTN3",0,"Cardiometabolic_II","B13805","20212097_SS211647","PASS",-3.7775,0.4233,"Plate control","PASS","PFC",1.34099141391491,"Baseline","Baseline"
7475,"PFC_15",74,"OID30326","Q6NUS6","TCTN3",0,"Cardiometabolic_II","B13805","20212097_SS211647","PASS",-3.7775,-0.0362,"Plate control","PASS","PFC",0.975220257921818,"1h","1h"
7476,"PFC_23",75,"OID30326","Q6NUS6","TCTN3",0,"Cardiometabolic_II","B13805","20212097_SS211647","PASS",-3.7775,0.7229,"Plate control","PASS","PFC",1.65049640936703,"24h","24h"
7477,"PFC_08",85,"OID30326","Q6NUS6","TCTN3",0,"Cardiometabolic_II","B13805","20212097_SS211647","PASS",-3.7775,-0.5837,"Plate control","PASS","PFC",0.667250321217339,"Baseline","Baseline"
7478,"PFC_16",86,"OID30326","Q6NUS6","TCTN3",0,"Cardiometabolic_II","B13805","20212097_SS211647","PASS",-3.7775,0.3302,"Plate control","PASS","PFC",1.2571876456564,"1h","1h"
7479,"PFC_24",87,"OID30326","Q6NUS6","TCTN3",0,"Cardiometabolic_II","B13805","20212097_SS211647","PASS",-3.7775,0.1128,"Plate control","PASS","PFC",1.08132484963846,"24h","24h"
7480,"PFC_01",1,"OID30327","Q96FZ7","CHMP6",0,"Cardiometabolic_II","B13805","20212097_SS211647","PASS",-3.0793,1.5371,"Plate control","PASS","PFC",2.90210556732606,"Baseline","Baseline"
7481,"PFC_09",2,"OID30327","Q96FZ7","CHMP6",0,"Cardiometabolic_II","B13805","20212097_SS211647","PASS",-3.0793,2.0726,"Plate control","PASS","PFC",4.20644068212117,"Baseline","Baseline"
7482,"PFC_17",3,"OID30327","Q96FZ7","CHMP6",0,"Cardiometabolic_II","B13805","20212097_SS211647","PASS",-3.0793,2.8255,"Plate control","PASS","PFC",7.08859644747172,"1h","1h"
7483,"PFC_25",4,"OID30327","Q96FZ7","CHMP6",0,"Cardiometabolic_II","B13805","20212097_SS211647","PASS",-3.0793,2.3572,"Plate control","PASS","PFC",5.12374969861843,"24h","24h"
7484,"PFC_02",13,"OID30327","Q96FZ7","CHMP6",0,"Cardiometabolic_II","B13805","20212097_SS211647","PASS",-3.0793,1.7876,"Plate control","PASS","PFC",3.45240089281006,"Baseline","Baseline"
7485,"PFC_10",14,"OID30327","Q96FZ7","CHMP6",0,"Cardiometabolic_II","B13805","20212097_SS211647","PASS",-3.0793,2.0017,"Plate control","PASS","PFC",4.00471617893732,"1h","1h"
7486,"PFC_18",15,"OID30327","Q96FZ7","CHMP6",0,"Cardiometabolic_II","B13805","20212097_SS211647","PASS",-3.0793,2.3151,"Plate control","PASS","PFC",4.97639153841596,"1h","1h"
7487,"PFC_26",16,"OID30327","Q96FZ7","CHMP6",0,"Cardiometabolic_II","B13805","20212097_SS211647","PASS",-3.0793,2.8186,"Plate control","PASS","PFC",7.05477465152965,"24h","24h"
7488,"PFC_03",25,"OID30327","Q96FZ7","CHMP6",0,"Cardiometabolic_II","B13805","20212097_SS211647","PASS",-3.0793,0.86,"Plate control","PASS","PFC",1.81503831063432,"Baseline","Baseline"
7489,"PFC_11",26,"OID30327","Q96FZ7","CHMP6",0,"Cardiometabolic_II","B13805","20212097_SS211647","PASS",-3.0793,2.4509,"Plate control","PASS","PFC",5.46757081150313,"1h","1h"
7490,"PFC_19",27,"OID30327","Q96FZ7","CHMP6",0,"Cardiometabolic_II","B13805","20212097_SS211647","PASS",-3.0793,2.4558,"Plate control","PASS","PFC",5.48617255663505,"24h","24h"
7491,"PFC_27",28,"OID30327","Q96FZ7","CHMP6",0,"Cardiometabolic_II","B13805","20212097_SS211647","PASS",-3.0793,3.3642,"Plate control","PASS","PFC",10.2973413940979,"24h","24h"
7492,"PFC_04",37,"OID30327","Q96FZ7","CHMP6",0,"Cardiometabolic_II","B13805","20212097_SS211647","PASS",-3.0793,2.1364,"Plate control","PASS","PFC",4.39663570759085,"Baseline","Baseline"
7493,"PFC_12",38,"OID30327","Q96FZ7","CHMP6",0,"Cardiometabolic_II","B13805","20212097_SS211647","PASS",-3.0793,1.0933,"Plate control","PASS","PFC",2.1336151883428,"1h","1h"
7494,"PFC_20",39,"OID30327","Q96FZ7","CHMP6",0,"Cardiometabolic_II","B13805","20212097_SS211647","PASS",-3.0793,2.7412,"Plate control","PASS","PFC",6.68626251993193,"24h","24h"
7495,"PFC_05",49,"OID30327","Q96FZ7","CHMP6",0,"Cardiometabolic_II","B13805","20212097_SS211647","PASS",-3.0793,1.4808,"Plate control","PASS","PFC",2.79103458187362,"Baseline","Baseline"
7496,"PFC_13",50,"OID30327","Q96FZ7","CHMP6",0,"Cardiometabolic_II","B13805","20212097_SS211647","PASS",-3.0793,2.214,"Plate control","PASS","PFC",4.63959861802174,"1h","1h"
7497,"PFC_21",51,"OID30327","Q96FZ7","CHMP6",0,"Cardiometabolic_II","B13805","20212097_SS211647","PASS",-3.0793,1.6326,"Plate control","PASS","PFC",3.10071300600612,"24h","24h"
7498,"PFC_06",61,"OID30327","Q96FZ7","CHMP6",0,"Cardiometabolic_II","B13805","20212097_SS211647","PASS",-3.0793,2.2576,"Plate control","PASS","PFC",4.78195316434583,"Baseline","Baseline"
7499,"PFC_14",62,"OID30327","Q96FZ7","CHMP6",0,"Cardiometabolic_II","B13805","20212097_SS211647","PASS",-3.0793,2.523,"Plate control","PASS","PFC",5.74776070617625,"1h","1h"
7500,"PFC_22",63,"OID30327","Q96FZ7","CHMP6",0,"Cardiometabolic_II","B13805","20212097_SS211647","PASS",-3.0793,1.8384,"Plate control","PASS","PFC",3.57613202631932,"24h","24h"
7501,"PFC_07",73,"OID30327","Q96FZ7","CHMP6",0,"Cardiometabolic_II","B13805","20212097_SS211647","PASS",-3.0793,2.6516,"Plate control","PASS","PFC",6.28363769749743,"Baseline","Baseline"
7502,"PFC_15",74,"OID30327","Q96FZ7","CHMP6",0,"Cardiometabolic_II","B13805","20212097_SS211647","PASS",-3.0793,1.6038,"Plate control","PASS","PFC",3.03942832936023,"1h","1h"
7503,"PFC_23",75,"OID30327","Q96FZ7","CHMP6",0,"Cardiometabolic_II","B13805","20212097_SS211647","PASS",-3.0793,2.7082,"Plate control","PASS","PFC",6.53505781746752,"24h","24h"
7504,"PFC_08",85,"OID30327","Q96FZ7","CHMP6",0,"Cardiometabolic_II","B13805","20212097_SS211647","PASS",-3.0793,1.8797,"Plate control","PASS","PFC",3.67998529150249,"Baseline","Baseline"

Supplemental table 1_Olink proximity extension assay (PEA) data

,"SampleID","Index","OlinkID","UniProt","Assay","MissingFreq","Panel","Panel_Lot_Nr","PlateID","QC_Warning","LOD","NPX","Normalization","Assay_Warning","condition","Value","group","alt_group"
7505,"PFC_16",86,"OID30327","Q96FZ7","CHMP6",0,"Cardiometabolic_II","B13805","20212097_SS211647","PASS",-3.0793,2.3072,"Plate control","PASS","PFC",4.94921597421328,"1h","1h"
7506,"PFC_24",87,"OID30327","Q96FZ7","CHMP6",0,"Cardiometabolic_II","B13805","20212097_SS211647","PASS",-3.0793,2.8701,"Plate control","PASS","PFC",7.31115835515232,"24h","24h"
7507,"PFC_01",1,"OID30328","P98161","PKD1",0,"Cardiometabolic_II","B13805","20212097_SS211647","PASS",-3.2213,0.2046,"Plate control","PASS","PFC",1.15236679858316,"Baseline","Baseline"
7508,"PFC_09",2,"OID30328","P98161","PKD1",0,"Cardiometabolic_II","B13805","20212097_SS211647","PASS",-3.2213,0.1084,"Plate control","PASS","PFC",1.07803199754786,"Baseline","Baseline"
7509,"PFC_17",3,"OID30328","P98161","PKD1",0,"Cardiometabolic_II","B13805","20212097_SS211647","PASS",-3.2213,-0.4622,"Plate control","PASS","PFC",0.725878504848802,"1h","1h"
7510,"PFC_25",4,"OID30328","P98161","PKD1",0,"Cardiometabolic_II","B13805","20212097_SS211647","PASS",-3.2213,0.1078,"Plate control","PASS","PFC",1.07758374986102,"24h","24h"
7511,"PFC_02",13,"OID30328","P98161","PKD1",0,"Cardiometabolic_II","B13805","20212097_SS211647","PASS",-3.2213,0.3788,"Plate control","PASS","PFC",1.3002598797597,"Baseline","Baseline"
7512,"PFC_10",14,"OID30328","P98161","PKD1",0,"Cardiometabolic_II","B13805","20212097_SS211647","PASS",-3.2213,0.3319,"Plate control","PASS","PFC",1.25866992613007,"1h","1h"
7513,"PFC_18",15,"OID30328","P98161","PKD1",0,"Cardiometabolic_II","B13805","20212097_SS211647","PASS",-3.2213,-0.1148,"Plate control","PASS","PFC",0.923510327571693,"1h","1h"
7514,"PFC_26",16,"OID30328","P98161","PKD1",0,"Cardiometabolic_II","B13805","20212097_SS211647","PASS",-3.2213,-0.3665,"Plate control","PASS","PFC",0.775661983727288,"24h","24h"
7515,"PFC_03",25,"OID30328","P98161","PKD1",0,"Cardiometabolic_II","B13805","20212097_SS211647","PASS",-3.2213,0.0248,"Plate control","PASS","PFC",1.01733864924329,"Baseline","Baseline"
7516,"PFC_11",26,"OID30328","P98161","PKD1",0,"Cardiometabolic_II","B13805","20212097_SS211647","PASS",-3.2213,0.3705,"Plate control","PASS","PFC",1.29280080362123,"1h","1h"
7517,"PFC_19",27,"OID30328","P98161","PKD1",0,"Cardiometabolic_II","B13805","20212097_SS211647","PASS",-3.2213,0.3488,"Plate control","PASS","PFC",1.27350091837458,"24h","24h"
7518,"PFC_27",28,"OID30328","P98161","PKD1",0,"Cardiometabolic_II","B13805","20212097_SS211647","PASS",-3.2213,0.2488,"Plate control","PASS","PFC",1.18821837159591,"24h","24h"
7519,"PFC_04",37,"OID30328","P98161","PKD1",0,"Cardiometabolic_II","B13805","20212097_SS211647","PASS",-3.2213,0.7668,"Plate control","PASS","PFC",1.70149156584627,"Baseline","Baseline"
7520,"PFC_12",38,"OID30328","P98161","PKD1",0,"Cardiometabolic_II","B13805","20212097_SS211647","PASS",-3.2213,-0.1388,"Plate control","PASS","PFC",0.90827432255142,"1h","1h"
7521,"PFC_20",39,"OID30328","P98161","PKD1",0,"Cardiometabolic_II","B13805","20212097_SS211647","PASS",-3.2213,0.2728,"Plate control","PASS","PFC",1.20815034657893,"24h","24h"
7522,"PFC_05",49,"OID30328","P98161","PKD1",0,"Cardiometabolic_II","B13805","20212097_SS211647","PASS",-3.2213,0.2168,"Plate control","PASS","PFC",1.16215298773704,"Baseline","Baseline"
7523,"PFC_13",50,"OID30328","P98161","PKD1",0,"Cardiometabolic_II","B13805","20212097_SS211647","PASS",-3.2213,0.711,"Plate control","PASS","PFC",1.63693836318161,"1h","1h"
7524,"PFC_21",51,"OID30328","P98161","PKD1",0,"Cardiometabolic_II","B13805","20212097_SS211647","PASS",-3.2213,0.0276,"Plate control","PASS","PFC",1.01931502968036,"24h","24h"
7525,"PFC_06",61,"OID30328","P98161","PKD1",0,"Cardiometabolic_II","B13805","20212097_SS211647","PASS",-3.2213,-0.1876,"Plate control","PASS","PFC",0.878065215234427,"Baseline","Baseline"
7526,"PFC_14",62,"OID30328","P98161","PKD1",0,"Cardiometabolic_II","B13805","20212097_SS211647","PASS",-3.2213,0.2108,"Plate control","PASS","PFC",1.15732976589592,"1h","1h"
7527,"PFC_22",63,"OID30328","P98161","PKD1",0,"Cardiometabolic_II","B13805","20212097_SS211647","PASS",-3.2213,0.7697,"Plate control","PASS","PFC",1.70491521951918,"24h","24h"
7528,"PFC_07",73,"OID30328","P98161","PKD1",0,"Cardiometabolic_II","B13805","20212097_SS211647","PASS",-3.2213,0.2125,"Plate control","PASS","PFC",1.15869430946228,"Baseline","Baseline"
7529,"PFC_15",74,"OID30328","P98161","PKD1",0,"Cardiometabolic_II","B13805","20212097_SS211647","PASS",-3.2213,-0.1854,"Plate control","PASS","PFC",0.879405219218972,"1h","1h"
7530,"PFC_23",75,"OID30328","P98161","PKD1",0,"Cardiometabolic_II","B13805","20212097_SS211647","PASS",-3.2213,0.235,"Plate control","PASS","PFC",1.17690673721877,"24h","24h"
7531,"PFC_08",85,"OID30328","P98161","PKD1",0,"Cardiometabolic_II","B13805","20212097_SS211647","PASS",-3.2213,-0.5045,"Plate control","PASS","PFC",0.704904636572407,"Baseline","Baseline"
7532,"PFC_16",86,"OID30328","P98161","PKD1",0,"Cardiometabolic_II","B13805","20212097_SS211647","PASS",-3.2213,0.0319,"Plate control","PASS","PFC",1.02235766372068,"1h","1h"
7533,"PFC_24",87,"OID30328","P98161","PKD1",0,"Cardiometabolic_II","B13805","20212097_SS211647","PASS",-3.2213,-0.2056,"Plate control","PASS","PFC",0.867177962970736,"24h","24h"
7534,"PFC_01",1,"OID30329","Q9BXD5","NPL",0,"Cardiometabolic_II","B13805","20212097_SS211647","PASS",-1.1925,2.4297,"Plate control","PASS","PFC",5.38781382657909,"Baseline","Baseline"
7535,"PFC_09",2,"OID30329","Q9BXD5","NPL",0,"Cardiometabolic_II","B13805","20212097_SS211647","PASS",-1.1925,2.8141,"Plate control","PASS","PFC",7.03280394722277,"Baseline","Baseline"
7536,"PFC_17",3,"OID30329","Q9BXD5","NPL",0,"Cardiometabolic_II","B13805","20212097_SS211647","PASS",-1.1925,2.2436,"Plate control","PASS","PFC",4.73577323022746,"1h","1h"
7537,"PFC_25",4,"OID30329","Q9BXD5","NPL",0,"Cardiometabolic_II","B13805","20212097_SS211647","PASS",-1.1925,1.9291,"Plate control","PASS","PFC",3.80817558745924,"24h","24h"
7538,"PFC_02",13,"OID30329","Q9BXD5","NPL",0,"Cardiometabolic_II","B13805","20212097_SS211647","PASS",-1.1925,3.075,"Plate control","PASS","PFC",8.42688828763869,"Baseline","Baseline"
7539,"PFC_10",14,"OID30329","Q9BXD5","NPL",0,"Cardiometabolic_II","B13805","20212097_SS211647","PASS",-1.1925,2.4584,"Plate control","PASS","PFC",5.49606855625937,"1h","1h"
7540,"PFC_18",15,"OID30329","Q9BXD5","NPL",0,"Cardiometabolic_II","B13805","20212097_SS211647","PASS",-1.1925,2.9621,"Plate control","PASS","PFC",7.79257426971497,"1h","1h"
7541,"PFC_26",16,"OID30329","Q9BXD5","NPL",0,"Cardiometabolic_II","B13805","20212097_SS211647","PASS",-1.1925,2.2226,"Plate control","PASS","PFC",4.6673381669459,"24h","24h"
7542,"PFC_03",25,"OID30329","Q9BXD5","NPL",0,"Cardiometabolic_II","B13805","20212097_SS211647","PASS",-1.1925,1.9663,"Plate control","PASS","PFC",3.90764660363772,"Baseline","Baseline"
7543,"PFC_11",26,"OID30329","Q9BXD5","NPL",0,"Cardiometabolic_II","B13805","20212097_SS211647","PASS",-1.1925,3.2078,"Plate control","PASS","PFC",9.23940533409109,"1h","1h"
7544,"PFC_19",27,"OID30329","Q9BXD5","NPL",0,"Cardiometabolic_II","B13805","20212097_SS211647","PASS",-1.1925,2.5555,"Plate control","PASS","PFC",5.87871158627911,"24h","24h"
7545,"PFC_27",28,"OID30329","Q9BXD5","NPL",0,"Cardiometabolic_II","B13805","20212097_SS211647","PASS",-1.1925,3.4209,"Plate control","PASS","PFC",10.71009966256,"24h","24h"
7546,"PFC_04",37,"OID30329","Q9BXD5","NPL",0,"Cardiometabolic_II","B13805","20212097_SS211647","PASS",-1.1925,2.8083,"Plate control","PASS","PFC",7.00458704905195,"Baseline","Baseline"
7547,"PFC_12",38,"OID30329","Q9BXD5","NPL",0,"Cardiometabolic_II","B13805","20212097_SS211647","PASS",-1.1925,2.3778,"Plate control","PASS","PFC",5.19743568229911,"1h","1h"
7548,"PFC_20",39,"OID30329","Q9BXD5","NPL",0,"Cardiometabolic_II","B13805","20212097_SS211647","PASS",-1.1925,3.0281,"Plate control","PASS","PFC",8.15734686858815,"24h","24h"
7549,"PFC_05",49,"OID30329","Q9BXD5","NPL",0,"Cardiometabolic_II","B13805","20212097_SS211647","PASS",-1.1925,2.9704,"Plate control","PASS","PFC",7.83753510562226,"Baseline","Baseline"
7550,"PFC_13",50,"OID30329","Q9BXD5","NPL",0,"Cardiometabolic_II","B13805","20212097_SS211647","PASS",-1.1925,2.8916,"Plate control","PASS","PFC",7.42093000782642,"1h","1h"
7551,"PFC_21",51,"OID30329","Q9BXD5","NPL",0,"Cardiometabolic_II","B13805","20212097_SS211647","PASS",-1.1925,2.3733,"Plate control","PASS","PFC",5.18124929390681,"24h","24h"
7552,"PFC_06",61,"OID30329","Q9BXD5","NPL",0,"Cardiometabolic_II","B13805","20212097_SS211647","PASS",-1.1925,2.0624,"Plate control","PASS","PFC",4.1768056055727,"Baseline","Baseline"
7553,"PFC_14",62,"OID30329","Q9BXD5","NPL",0,"Cardiometabolic_II","B13805","20212097_SS211647","PASS",-1.1925,3.2019,"Plate control","PASS","PFC",9.20169731164462,"1h","1h"
7554,"PFC_22",63,"OID30329","Q9BXD5","NPL",0,"Cardiometabolic_II","B13805","20212097_SS211647","PASS",-1.1925,2.6416,"Plate control","PASS","PFC",6.24023344142996,"24h","24h"
7555,"PFC_07",73,"OID30329","Q9BXD5","NPL",0,"Cardiometabolic_II","B13805","20212097_SS211647","PASS",-1.1925,2.0955,"Plate control","PASS","PFC",4.27374254185116,"Baseline","Baseline"
7556,"PFC_15",74,"OID30329","Q9BXD5","NPL",0,"Cardiometabolic_II","B13805","20212097_SS211647","PASS",-1.1925,2.4411,"Plate control","PASS","PFC",5.43055632395517,"1h","1h"
7557,"PFC_23",75,"OID30329","Q9BXD5","NPL",0,"Cardiometabolic_II","B13805","20212097_SS211647","PASS",-1.1925,3.1832,"Plate control","PASS","PFC",9.0831959282212,"24h","24h"
7558,"PFC_08",85,"OID30329","Q9BXD5","NPL",0,"Cardiometabolic_II","B13805","20212097_SS211647","PASS",-1.1925,1.8172,"Plate control","PASS","PFC",3.52396598806528,"Baseline","Baseline"
7559,"PFC_16",86,"OID30329","Q9BXD5","NPL",0,"Cardiometabolic_II","B13805","20212097_SS211647","PASS",-1.1925,2.0582,"Plate control","PASS","PFC",4.16466369569952,"1h","1h"
7560,"PFC_24",87,"OID30329","Q9BXD5","NPL",0,"Cardiometabolic_II","B13805","20212097_SS211647","PASS",-1.1925,2.3004,"Plate control","PASS","PFC",4.92594322552577,"24h","24h"
7561,"PFC_01",1,"OID30330","Q9P2J2","IGSF9",0,"Cardiometabolic_II","B13805","20212097_SS211647","PASS",-3.2624,-1.3734,"Plate control","PASS","PFC",0.385980535180996,"Baseline","Baseline"
7562,"PFC_09",2,"OID30330","Q9P2J2","IGSF9",0,"Cardiometabolic_II","B13805","20212097_SS211647","PASS",-3.2624,-1.595,"Plate control","PASS","PFC",0.331022227598849,"Baseline","Baseline"
7563,"PFC_17",3,"OID30330","Q9P2J2","IGSF9",0,"Cardiometabolic_II","B13805","20212097_SS211647","PASS",-3.2624,-2.4123,"Plate control","PASS","PFC",0.187856117115013,"1h","1h"
7564,"PFC_25",4,"OID30330","Q9P2J2","IGSF9",0,"Cardiometabolic_II","B13805","20212097_SS211647","PASS",-3.2624,-1.4366,"Plate control","PASS","PFC",0.369436931364133,"24h","24h"
7565,"PFC_02",13,"OID30330","Q9P2J2","IGSF9",0,"Cardiometabolic_II","B13805","20212097_SS211647","PASS",-3.2624,-0.8148,"Plate control","PASS","PFC",0.568487290228112,"Baseline","Baseline"
7566,"PFC_10",14,"OID30330","Q9P2J2","IGSF9",0,"Cardiometabolic_II","B13805","20212097_SS211647","PASS",-3.2624,-1.0244,"Plate control","PASS","PFC",0.491614713574502,"1h","1h"
7567,"PFC_18",15,"OID30330","Q9P2J2","IGSF9",0,"Cardiometabolic_II","B13805","20212097_SS211647","PASS",-3.2624,-1.5501,"Plate control","PASS","PFC",0.341486393335165,"1h","1h"
7568,"PFC_26",16,"OID30330","Q9P2J2","IGSF9",0,"Cardiometabolic_II","B13805","20212097_SS211647","PASS",-3.2624,-2.2951,"Plate control","PASS","PFC",0.203753959462921,"24h","24h"
7569,"PFC_03",25,"OID30330","Q9P2J2","IGSF9",0,"Cardiometabolic_II","B13805","20212097_SS211647","PASS",-3.2624,-1.8296,"Plate control","PASS","PFC",0.281342615095619,"Baseline","Baseline"
7570,"PFC_11",26,"OID30330","Q9P2J2","IGSF9",0,"Cardiometabolic_II","B13805","20212097_SS211647","PASS",-3.2624,-0.6837,"Plate control","PASS","PFC",0.622566563309309,"1h","1h"
7571,"PFC_19",27,"OID30330","Q9P2J2","IGSF9",0,"Cardiometabolic_II","B13805","20212097_SS211647","PASS",-3.2624,-0.5436,"Plate control","PASS","PFC",0.686056833272164,"24h","24h"
7572,"PFC_27",28,"OID30330","Q9P2J2","IGSF9",0,"Cardiometabolic_II","B13805","20212097_SS211647","PASS",-3.2624,-1.1751,"Plate control","PASS","PFC",0.442853062252155,"24h","24h"
7573,"PFC_04",37,"OID30330","Q9P2J2","IGSF9",0,"Cardiometabolic_II","B13805","20212097_SS211647","PASS",-3.2624,-1.0025,"Plate control","PASS","PFC",0.499134316298696,"Baseline","Baseline"
7574,"PFC_12",38,"OID30330","Q9P2J2","IGSF9",0,"Cardiometabolic_II","B13805","20212097_SS211647","PASS",-3.2624,-1.9184,"Plate control","PASS","PFC",0.264547740341486,"1h","1h"
7575,"PFC_20",39,"OID30330","Q9P2J2","IGSF9",0,"Cardiometabolic_II","B13805","20212097_SS211647","PASS",-3.2624,-0.7227,"Plate control","PASS","PFC",0.605962323437695,"24h","24h"
7576,"PFC_05",49,"OID30330","Q9P2J2","IGSF9",0,"Cardiometabolic_II","B13805","20212097_SS211647","PASS",-3.2624,-0.16,"Plate control","PASS","PFC",0.895025070927972,"Baseline","Baseline"
7577,"PFC_13",50,"OID30330","Q9P2J2","IGSF9",0,"Cardiometabolic_II","B13805","20212097_SS211647","PASS",-3.2624,-1.1986,"Plate control","PASS","PFC",0.435697880429045,"1h","1h"
7578,"PFC_21",51,"OID30330","Q9P2J2","IGSF9",0,"Cardiometabolic_II","B13805","20212097_SS211647","PASS",-3.2624,-1.5701,"Plate control","PASS","PFC",0.336785049246623,"24h","24h"
7579,"PFC_06",61,"OID30330","Q9P2J2","IGSF9",0,"Cardiometabolic_II","B13805","20212097_SS211647","PASS",-3.2624,-1.2082,"Plate control","PASS","PFC",0.432808278633145,"Baseline","Baseline"
7580,"PFC_14",62,"OID30330","Q9P2J2","IGSF9",0,"Cardiometabolic_II","B13805","20212097_SS211647","PASS",-3.2624,0.2708,"Plate control","PASS","PFC",1.20647665494876,"1h","1h"
7581,"PFC_22",63,"OID30330","Q9P2J2","IGSF9",0,"Cardiometabolic_II","B13805","20212097_SS211647","PASS",-3.2624,-1.2194,"Plate control","PASS","PFC",0.429461289012534,"24h","24h"
7582,"PFC_07",73,"OID30330","Q9P2J2","IGSF9",0,"Cardiometabolic_II","B13805","20212097_SS211647","PASS",-3.2624,-1.8849,"Plate control","PASS","PFC",0.27076252855938,"Baseline","Baseline"
7583,"PFC_15",74,"OID30330","Q9P2J2","IGSF9",0,"Cardiometabolic_II","B13805","20212097_SS211647","PASS",-3.2624,-1.1843,"Plate control","PASS","PFC",0.440037993938673,"1h","1h"
7584,"PFC_23",75,"OID30330","Q9P2J2","IGSF9",0,"Cardiometabolic_II","B13805","20212097_SS211647","PASS",-3.2624,0.0566,"Plate control","PASS","PFC",1.04001187402485,"24h","24h"
7585,"PFC_08",85,"OID30330","Q9P2J2","IGSF9",0,"Cardiometabolic_II","B13805","20212097_SS211647","PASS",-3.2624,-2.2546,"Plate control","PASS","PFC",0.209554876561974,"Baseline","Baseline"
7586,"PFC_16",86,"OID30330","Q9P2J2","IGSF9",0,"Cardiometabolic_II","B13805","20212097_SS211647","PASS",-3.2624,-1.1159,"Plate control","PASS","PFC",0.461403227253119,"1h","1h"
7587,"PFC_24",87,"OID30330","Q9P2J2","IGSF9",0,"Cardiometabolic_II","B13805","20212097_SS211647","PASS",-3.2624,-1.231,"Plate control","PASS","PFC",0.426022047537433,"24h","24h"
7588,"PFC_01",1,"OID30331","Q9BW04","SARG",0,"Cardiometabolic_II","B13805","20212097_SS211647","PASS",-2.1394,3.7763,"Plate control","PASS","PFC",13.70186147852,"Baseline","Baseline"
7589,"PFC_09",2,"OID30331","Q9BW04","SARG",0,"Cardiometabolic_II","B13805","20212097_SS211647","PASS",-2.1394,4.0463,"Plate control","PASS","PFC",16.5218118247339,"Baseline","Baseline"
7590,"PFC_17",3,"OID30331","Q9BW04","SARG",0,"Cardiometabolic_II","B13805","20212097_SS211647","PASS",-2.1394,4.4435,"Plate control","PASS","PFC",21.7583914477659,"1h","1h"
7591,"PFC_25",4,"OID30331","Q9BW04","SARG",0,"Cardiometabolic_II","B13805","20212097_SS211647","PASS",-2.1394,4.1044,"Plate control","PASS","PFC",17.2007551665956,"24h","24h"
7592,"PFC_02",13,"OID30331","Q9BW04","SARG",0,"Cardiometabolic_II","B13805","20212097_SS211647","PASS",-2.1394,3.4908,"Plate control","PASS","PFC",11.2417910504715,"Baseline","Baseline"
7593,"PFC_10",14,"OID30331","Q9BW04","SARG",0,"Cardiometabolic_II","B13805","20212097_SS211647","PASS",-2.1394,4.1082,"Plate control","PASS","PFC",17.2461209750133,"1h","1h"
7594,"PFC_18",15,"OID30331","Q9BW04","SARG",0,"Cardiometabolic_II","B13805","20212097_SS211647","PASS",-2.1394,4.9318,"Plate control","PASS","PFC",30.5224740108552,"1h","1h"
7595,"PFC_26",16,"OID30331","Q9BW04","SARG",0,"Cardiometabolic_II","B13805","20212097_SS211647","PASS",-2.1394,4.0189,"Plate control","PASS","PFC",16.2109867036286,"24h","24h"
7596,"PFC_03",25,"OID30331","Q9BW04","SARG",0,"Cardiometabolic_II","B13805","20212097_SS211647","PASS",-2.1394,2.3097,"Plate control","PASS","PFC",4.95779974708403,"Baseline","Baseline"
7597,"PFC_11",26,"OID30331","Q9BW04","SARG",0,"Cardiometabolic_II","B13805","20212097_SS211647","PASS",-2.1394,4.6039,"Plate control","PASS","PFC",24.3171121102369,"1h","1h"
7598,"PFC_19",27,"OID30331","Q9BW04","SARG",0,"Cardiometabolic_II","B13805","20212097_SS211647","PASS",-2.1394,4.9962,"Plate control","PASS","PFC",31.9158242093125,"24h","24h"
7599,"PFC_27",28,"OID30331","Q9BW04","SARG",0,"Cardiometabolic_II","B13805","20212097_SS211647","PASS",-2.1394,6.2458,"Plate control","PASS","PFC",75.8880069214474,"24h","24h"
7600,"PFC_04",37,"OID30331","Q9BW04","SARG",0,"Cardiometabolic_II","B13805","20212097_SS211647","PASS",-2.1394,4.5166,"Plate control","PASS","PFC",22.8892771853291,"Baseline","Baseline"
7601,"PFC_12",38,"OID30331","Q9BW04","SARG",0,"Cardiometabolic_II","B13805","20212097_SS211647","PASS",-2.1394,2.8369,"Plate control","PASS","PFC",7.14483155967279,"1h","1h"
7602,"PFC_20",39,"OID30331","Q9BW04","SARG",0,"Cardiometabolic_II","B13805","20212097_SS211647","PASS",-2.1394,5.1564,"Plate control","PASS","PFC",35.6640836391607,"24h","24h"
7603,"PFC_05",49,"OID30331","Q9BW04","SARG",0,"Cardiometabolic_II","B13805","20212097_SS211647","PASS",-2.1394,2.6562,"Plate control","PASS","PFC",6.30370490688154,"Baseline","Baseline"
7604,"PFC_13",50,"OID30331","Q9BW04","SARG",0,"Cardiometabolic_II","B13805","20212097_SS211647","PASS",-2.1394,4.4828,"Plate control","PASS","PFC",22.3592516843029,"1h","1h"
7605,"PFC_21",51,"OID30331","Q9BW04","SARG",0,"Cardiometabolic_II","B13805","20212097_SS211647","PASS",-2.1394,3.4888,"Plate control","PASS","PFC",11.2262174162441,"24h","24h"
7606,"PFC_06",61,"OID30331","Q9BW04","SARG",0,"Cardiometabolic_II","B13805","20212097_SS211647","PASS",-2.1394,4.779,"Plate control","PASS","PFC",27.4550569737181,"Baseline","Baseline"
7607,"PFC_14",62,"OID30331","Q9BW04","SARG",0,"Cardiometabolic_II","B13805","20212097_SS211647","PASS",-2.1394,4.018,"Plate control","PASS","PFC",16.2008769176079,"1h","1h"
7608,"PFC_22",63,"OID30331","Q9BW04","SARG",0,"Cardiometabolic_II","B13805","20212097_SS211647","PASS",-2.1394,4.2956,"Plate control","PASS","PFC",19.6383252878261,"24h","24h"
7609,"PFC_07",73,"OID30331","Q9BW04","SARG",0,"Cardiometabolic_II","B13805","20212097_SS211647","PASS",-2.1394,4.6866,"Plate control","PASS","PFC",25.7517755057485,"Baseline","Baseline"
7610,"PFC_15",74,"OID30331","Q9BW04","SARG",0,"Cardiometabolic_II","B13805","20212097_SS211647","PASS",-2.1394,3.319,"Plate control","PASS","PFC",9.97972457531236,"1h","1h"
7611,"PFC_23",75,"OID30331","Q9BW04","SARG",0,"Cardiometabolic_II","B13805","20212097_SS211647","PASS",-2.1394,4.1662,"Plate control","PASS","PFC",17.9535844112618,"24h","24h"
7612,"PFC_08",85,"OID30331","Q9BW04","SARG",0,"Cardiometabolic_II","B13805","20212097_SS211647","PASS",-2.1394,3.1812,"Plate control","PASS","PFC",9.07061266899083,"Baseline","Baseline"
7613,"PFC_16",86,"OID30331","Q9BW04","SARG",0,"Cardiometabolic_II","B13805","20212097_SS211647","PASS",-2.1394,4.2441,"Plate control","PASS","PFC",18.9496592344242,"1h","1h"
7614,"PFC_24",87,"OID30331","Q9BW04","SARG",0,"Cardiometabolic_II","B13805","20212097_SS211647","PASS",-2.1394,5.3572,"Plate control","PASS","PFC",40.9899975889475,"24h","24h"
7615,"PFC_01",1,"OID30332","Q8WWV6","FCAMR",0,"Cardiometabolic_II","B13805","20212097_SS211647","PASS",-3.4193,0.2229,"Plate control","PASS","PFC",1.16707720338747,"Baseline","Baseline"
7616,"PFC_09",2,"OID30332","Q8WWV6","FCAMR",0,"Cardiometabolic_II","B13805","20212097_SS211647","PASS",-3.4193,0.418,"Plate control","PASS","PFC",1.3360740783901,"Baseline","Baseline"

Supplemental table 1_Olink proximity extension assay (PEA) data

,"SampleID","Index","OlinkID","UniProt","Assay","MissingFreq","Panel","Panel_Lot_Nr","PlateID","QC_Warning","LOD","NPX","Normalization","Assay_Warning","condition","Value","group","alt_group"
7617,"PFC_17",3,"OID30332","Q8WWV6","FCAMR",0,"Cardiometabolic_II","B13805","20212097_SS211647","PASS",-3.4193,-1.3273,"Plate control","PASS","PFC",0.398513361129854,"1h","1h"
7618,"PFC_25",4,"OID30332","Q8WWV6","FCAMR",0,"Cardiometabolic_II","B13805","20212097_SS211647","PASS",-3.4193,-1.5151,"Plate control","PASS","PFC",0.349872214264406,"24h","24h"
7619,"PFC_02",13,"OID30332","Q8WWV6","FCAMR",0,"Cardiometabolic_II","B13805","20212097_SS211647","PASS",-3.4193,0.1919,"Plate control","PASS","PFC",1.14226706818468,"Baseline","Baseline"
7620,"PFC_10",14,"OID30332","Q8WWV6","FCAMR",0,"Cardiometabolic_II","B13805","20212097_SS211647","PASS",-3.4193,0.4124,"Plate control","PASS","PFC",1.33089799320734,"1h","1h"
7621,"PFC_18",15,"OID30332","Q8WWV6","FCAMR",0,"Cardiometabolic_II","B13805","20212097_SS211647","PASS",-3.4193,0.4051,"Plate control","PASS","PFC",1.32418069242579,"1h","1h"
7622,"PFC_26",16,"OID30332","Q8WWV6","FCAMR",0,"Cardiometabolic_II","B13805","20212097_SS211647","PASS",-3.4193,-1.1744,"Plate control","PASS","PFC",0.44306798803531,"24h","24h"
7623,"PFC_03",25,"OID30332","Q8WWV6","FCAMR",0,"Cardiometabolic_II","B13805","20212097_SS211647","PASS",-3.4193,-2.1306,"Plate control","PASS","PFC",0.228362869358015,"Baseline","Baseline"
7624,"PFC_11",26,"OID30332","Q8WWV6","FCAMR",0,"Cardiometabolic_II","B13805","20212097_SS211647","PASS",-3.4193,0.3114,"Plate control","PASS","PFC",1.24091130369242,"1h","1h"
7625,"PFC_19",27,"OID30332","Q8WWV6","FCAMR",0,"Cardiometabolic_II","B13805","20212097_SS211647","PASS",-3.4193,0.5103,"Plate control","PASS","PFC",1.42434634930114,"24h","24h"
7626,"PFC_27",28,"OID30332","Q8WWV6","FCAMR",0,"Cardiometabolic_II","B13805","20212097_SS211647","PASS",-3.4193,0.772,"Plate control","PASS","PFC",1.70763542878249,"24h","24h"
7627,"PFC_04",37,"OID30332","Q8WWV6","FCAMR",0,"Cardiometabolic_II","B13805","20212097_SS211647","PASS",-3.4193,-1.9731,"Plate control","PASS","PFC",0.254705143727072,"Baseline","Baseline"
7628,"PFC_12",38,"OID30332","Q8WWV6","FCAMR",0,"Cardiometabolic_II","B13805","20212097_SS211647","PASS",-3.4193,-2.1416,"Plate control","PASS","PFC",0.2266283105667,"1h","1h"
7629,"PFC_20",39,"OID30332","Q8WWV6","FCAMR",0,"Cardiometabolic_II","B13805","20212097_SS211647","PASS",-3.4193,0.2252,"Plate control","PASS","PFC",1.16893928672352,"24h","24h"
7630,"PFC_05",49,"OID30332","Q8WWV6","FCAMR",0,"Cardiometabolic_II","B13805","20212097_SS211647","PASS",-3.4193,0.521,"Plate control","PASS","PFC",1.4349495346067,"Baseline","Baseline"
7631,"PFC_13",50,"OID30332","Q8WWV6","FCAMR",0,"Cardiometabolic_II","B13805","20212097_SS211647","PASS",-3.4193,-1.6753,"Plate control","PASS","PFC",0.313100995364548,"1h","1h"
7632,"PFC_21",51,"OID30332","Q8WWV6","FCAMR",0,"Cardiometabolic_II","B13805","20212097_SS211647","PASS",-3.4193,-2.2546,"Plate control","PASS","PFC",0.209554876561974,"24h","24h"
7633,"PFC_06",61,"OID30332","Q8WWV6","FCAMR",0,"Cardiometabolic_II","B13805","20212097_SS211647","PASS",-3.4193,0.212,"Plate control","PASS","PFC",1.15829280619464,"Baseline","Baseline"
7634,"PFC_14",62,"OID30332","Q8WWV6","FCAMR",0,"Cardiometabolic_II","B13805","20212097_SS211647","PASS",-3.4193,0.5007,"Plate control","PASS","PFC",1.41489990956893,"1h","1h"
7635,"PFC_22",63,"OID30332","Q8WWV6","FCAMR",0,"Cardiometabolic_II","B13805","20212097_SS211647","PASS",-3.4193,-2.1639,"Plate control","PASS","PFC",0.22315221006471,"24h","24h"
7636,"PFC_07",73,"OID30332","Q8WWV6","FCAMR",0,"Cardiometabolic_II","B13805","20212097_SS211647","PASS",-3.4193,-1.0803,"Plate control","PASS","PFC",0.472930470010583,"Baseline","Baseline"
7637,"PFC_15",74,"OID30332","Q8WWV6","FCAMR",0,"Cardiometabolic_II","B13805","20212097_SS211647","PASS",-3.4193,0.4149,"Plate control","PASS","PFC",1.33320626307217,"1h","1h"
7638,"PFC_23",75,"OID30332","Q8WWV6","FCAMR",0,"Cardiometabolic_II","B13805","20212097_SS211647","PASS",-3.4193,0.4982,"Plate control","PASS","PFC",1.41245019798755,"24h","24h"
7639,"PFC_08",85,"OID30332","Q8WWV6","FCAMR",0,"Cardiometabolic_II","B13805","20212097_SS211647","PASS",-3.4193,-1.0121,"Plate control","PASS","PFC",0.495823996277511,"Baseline","Baseline"
7640,"PFC_16",86,"OID30332","Q8WWV6","FCAMR",0,"Cardiometabolic_II","B13805","20212097_SS211647","PASS",-3.4193,-1.305,"Plate control","PASS","PFC",0.404721108273704,"1h","1h"
7641,"PFC_24",87,"OID30332","Q8WWV6","FCAMR",0,"Cardiometabolic_II","B13805","20212097_SS211647","PASS",-3.4193,0.0476,"Plate control","PASS","PFC",1.03354413723686,"24h","24h"
7642,"PFC_01",1,"OID30333","O75711","SCRG1",0,"Cardiometabolic_II","B13805","20212097_SS211647","PASS",-1.9332,0.7991,"Plate control","PASS","PFC",1.74001530990804,"Baseline","Baseline"
7643,"PFC_09",2,"OID30333","O75711","SCRG1",0,"Cardiometabolic_II","B13805","20212097_SS211647","PASS",-1.9332,0.4659,"Plate control","PASS","PFC",1.38117870881304,"Baseline","Baseline"
7644,"PFC_17",3,"OID30333","O75711","SCRG1",0,"Cardiometabolic_II","B13805","20212097_SS211647","PASS",-1.9332,-0.4757,"Plate control","PASS","PFC",0.719117787216962,"1h","1h"
7645,"PFC_25",4,"OID30333","O75711","SCRG1",0,"Cardiometabolic_II","B13805","20212097_SS211647","PASS",-1.9332,-0.0693,"Plate control","PASS","PFC",0.953100333037743,"24h","24h"
7646,"PFC_02",13,"OID30333","O75711","SCRG1",0,"Cardiometabolic_II","B13805","20212097_SS211647","PASS",-1.9332,0.8029,"Plate control","PASS","PFC",1.74460448058273,"Baseline","Baseline"
7647,"PFC_10",14,"OID30333","O75711","SCRG1",0,"Cardiometabolic_II","B13805","20212097_SS211647","PASS",-1.9332,0.9302,"Plate control","PASS","PFC",1.90554014173361,"1h","1h"
7648,"PFC_18",15,"OID30333","O75711","SCRG1",0,"Cardiometabolic_II","B13805","20212097_SS211647","PASS",-1.9332,0.4844,"Plate control","PASS","PFC",1.39900391521875,"1h","1h"
7649,"PFC_26",16,"OID30333","O75711","SCRG1",0,"Cardiometabolic_II","B13805","20212097_SS211647","PASS",-1.9332,0.102,"Plate control","PASS","PFC",1.07326028639345,"24h","24h"
7650,"PFC_03",25,"OID30333","O75711","SCRG1",0,"Cardiometabolic_II","B13805","20212097_SS211647","PASS",-1.9332,0.1085,"Plate control","PASS","PFC",1.07810672362161,"Baseline","Baseline"
7651,"PFC_11",26,"OID30333","O75711","SCRG1",0,"Cardiometabolic_II","B13805","20212097_SS211647","PASS",-1.9332,0.987,"Plate control","PASS","PFC",1.98205912652816,"1h","1h"
7652,"PFC_19",27,"OID30333","O75711","SCRG1",0,"Cardiometabolic_II","B13805","20212097_SS211647","PASS",-1.9332,0.4155,"Plate control","PASS","PFC",1.33376084328331,"24h","24h"
7653,"PFC_27",28,"OID30333","O75711","SCRG1",0,"Cardiometabolic_II","B13805","20212097_SS211647","PASS",-1.9332,0.8393,"Plate control","PASS","PFC",1.78918181478422,"24h","24h"
7654,"PFC_04",37,"OID30333","O75711","SCRG1",0,"Cardiometabolic_II","B13805","20212097_SS211647","PASS",-1.9332,0.87,"Plate control","PASS","PFC",1.8276629004588,"Baseline","Baseline"
7655,"PFC_12",38,"OID30333","O75711","SCRG1",0,"Cardiometabolic_II","B13805","20212097_SS211647","PASS",-1.9332,0.2505,"Plate control","PASS","PFC",1.18961933421017,"1h","1h"
7656,"PFC_20",39,"OID30333","O75711","SCRG1",0,"Cardiometabolic_II","B13805","20212097_SS211647","PASS",-1.9332,0.7211,"Plate control","PASS","PFC",1.64843842699139,"24h","24h"
7657,"PFC_05",49,"OID30333","O75711","SCRG1",0,"Cardiometabolic_II","B13805","20212097_SS211647","PASS",-1.9332,0.5072,"Plate control","PASS","PFC",1.42128906202596,"Baseline","Baseline"
7658,"PFC_13",50,"OID30333","O75711","SCRG1",0,"Cardiometabolic_II","B13805","20212097_SS211647","PASS",-1.9332,0.7253,"Plate control","PASS","PFC",1.65324438307199,"1h","1h"
7659,"PFC_21",51,"OID30333","O75711","SCRG1",0,"Cardiometabolic_II","B13805","20212097_SS211647","PASS",-1.9332,0.1403,"Plate control","PASS","PFC",1.10213427442917,"24h","24h"
7660,"PFC_06",61,"OID30333","O75711","SCRG1",0,"Cardiometabolic_II","B13805","20212097_SS211647","PASS",-1.9332,0.5367,"Plate control","PASS","PFC",1.45065052217255,"Baseline","Baseline"
7661,"PFC_14",62,"OID30333","O75711","SCRG1",0,"Cardiometabolic_II","B13805","20212097_SS211647","PASS",-1.9332,0.5674,"Plate control","PASS","PFC",1.48185059729423,"1h","1h"
7662,"PFC_22",63,"OID30333","O75711","SCRG1",0,"Cardiometabolic_II","B13805","20212097_SS211647","PASS",-1.9332,0.4668,"Plate control","PASS","PFC",1.38204060173855,"24h","24h"
7663,"PFC_07",73,"OID30333","O75711","SCRG1",0,"Cardiometabolic_II","B13805","20212097_SS211647","PASS",-1.9332,-0.2271,"Plate control","PASS","PFC",0.85435051977191,"Baseline","Baseline"
7664,"PFC_15",74,"OID30333","O75711","SCRG1",0,"Cardiometabolic_II","B13805","20212097_SS211647","PASS",-1.9332,0.7439,"Plate control","PASS","PFC",1.67469688985095,"1h","1h"
7665,"PFC_23",75,"OID30333","O75711","SCRG1",0,"Cardiometabolic_II","B13805","20212097_SS211647","PASS",-1.9332,0.3156,"Plate control","PASS","PFC",1.24452913079947,"24h","24h"
7666,"PFC_08",85,"OID30333","O75711","SCRG1",0,"Cardiometabolic_II","B13805","20212097_SS211647","PASS",-1.9332,0.1363,"Plate control","PASS","PFC",1.09908274164701,"Baseline","Baseline"
7667,"PFC_16",86,"OID30333","O75711","SCRG1",0,"Cardiometabolic_II","B13805","20212097_SS211647","PASS",-1.9332,0.0795,"Plate control","PASS","PFC",1.05665176949768,"1h","1h"
7668,"PFC_24",87,"OID30333","O75711","SCRG1",0,"Cardiometabolic_II","B13805","20212097_SS211647","PASS",-1.9332,0.7575,"Plate control","PASS","PFC",1.69055857026078,"24h","24h"
7669,"PFC_01",1,"OID30334","Q6UXI7","VIT",0,"Cardiometabolic_II","B13805","20212097_SS211647","PASS",-2.7469,0.9254,"Plate control","PASS","PFC",1.89921074193769,"Baseline","Baseline"
7670,"PFC_09",2,"OID30334","Q6UXI7","VIT",0,"Cardiometabolic_II","B13805","20212097_SS211647","PASS",-2.7469,-0.6216,"Plate control","PASS","PFC",0.64994971057216,"Baseline","Baseline"
7671,"PFC_17",3,"OID30334","Q6UXI7","VIT",0,"Cardiometabolic_II","B13805","20212097_SS211647","PASS",-2.7469,-0.1965,"Plate control","PASS","PFC",0.872665096042938,"1h","1h"
7672,"PFC_25",4,"OID30334","Q6UXI7","VIT",0,"Cardiometabolic_II","B13805","20212097_SS211647","PASS",-2.7469,-0.2549,"Plate control","PASS","PFC",0.838045221523036,"24h","24h"
7673,"PFC_02",13,"OID30334","Q6UXI7","VIT",0,"Cardiometabolic_II","B13805","20212097_SS211647","PASS",-2.7469,0.3755,"Plate control","PASS","PFC",1.29728908288429,"Baseline","Baseline"
7674,"PFC_10",14,"OID30334","Q6UXI7","VIT",0,"Cardiometabolic_II","B13805","20212097_SS211647","PASS",-2.7469,1.2817,"Plate control","PASS","PFC",2.43125294895988,"1h","1h"
7675,"PFC_18",15,"OID30334","Q6UXI7","VIT",0,"Cardiometabolic_II","B13805","20212097_SS211647","PASS",-2.7469,-0.3854,"Plate control","PASS","PFC",0.765566708956628,"1h","1h"
7676,"PFC_26",16,"OID30334","Q6UXI7","VIT",0,"Cardiometabolic_II","B13805","20212097_SS211647","PASS",-2.7469,0.1396,"Plate control","PASS","PFC",1.10159964525606,"24h","24h"
7677,"PFC_03",25,"OID30334","Q6UXI7","VIT",0,"Cardiometabolic_II","B13805","20212097_SS211647","PASS",-2.7469,0.354,"Plate control","PASS","PFC",1.27809936320305,"Baseline","Baseline"
7678,"PFC_11",26,"OID30334","Q6UXI7","VIT",0,"Cardiometabolic_II","B13805","20212097_SS211647","PASS",-2.7469,0.4428,"Plate control","PASS","PFC",1.35923979819835,"1h","1h"
7679,"PFC_19",27,"OID30334","Q6UXI7","VIT",0,"Cardiometabolic_II","B13805","20212097_SS211647","PASS",-2.7469,1.2197,"Plate control","PASS","PFC",2.32898282421646,"24h","24h"
7680,"PFC_27",28,"OID30334","Q6UXI7","VIT",0,"Cardiometabolic_II","B13805","20212097_SS211647","PASS",-2.7469,-0.4851,"Plate control","PASS","PFC",0.714447546423968,"24h","24h"
7681,"PFC_04",37,"OID30334","Q6UXI7","VIT",0,"Cardiometabolic_II","B13805","20212097_SS211647","PASS",-2.7469,0.9434,"Plate control","PASS","PFC",1.9230549669207,"Baseline","Baseline"
7682,"PFC_12",38,"OID30334","Q6UXI7","VIT",0,"Cardiometabolic_II","B13805","20212097_SS211647","PASS",-2.7469,0.0934,"Plate control","PASS","PFC",1.06688154220183,"1h","1h"
7683,"PFC_20",39,"OID30334","Q6UXI7","VIT",0,"Cardiometabolic_II","B13805","20212097_SS211647","PASS",-2.7469,0.2717,"Plate control","PASS","PFC",1.20722952906061,"24h","24h"
7684,"PFC_05",49,"OID30334","Q6UXI7","VIT",0,"Cardiometabolic_II","B13805","20212097_SS211647","PASS",-2.7469,0.582,"Plate control","PASS","PFC",1.49692298679269,"Baseline","Baseline"
7685,"PFC_13",50,"OID30334","Q6UXI7","VIT",0,"Cardiometabolic_II","B13805","20212097_SS211647","PASS",-2.7469,0.2642,"Plate control","PASS","PFC",1.20096990572637,"1h","1h"
7686,"PFC_21",51,"OID30334","Q6UXI7","VIT",0,"Cardiometabolic_II","B13805","20212097_SS211647","PASS",-2.7469,0.5361,"Plate control","PASS","PFC",1.45004733901801,"24h","24h"
7687,"PFC_06",61,"OID30334","Q6UXI7","VIT",0,"Cardiometabolic_II","B13805","20212097_SS211647","PASS",-2.7469,0.7199,"Plate control","PASS","PFC",1.64706786453371,"Baseline","Baseline"
7688,"PFC_14",62,"OID30334","Q6UXI7","VIT",0,"Cardiometabolic_II","B13805","20212097_SS211647","PASS",-2.7469,0.6063,"Plate control","PASS","PFC",1.52234991169258,"1h","1h"
7689,"PFC_22",63,"OID30334","Q6UXI7","VIT",0,"Cardiometabolic_II","B13805","20212097_SS211647","PASS",-2.7469,0.5432,"Plate control","PASS","PFC",1.45720111086463,"24h","24h"
7690,"PFC_07",73,"OID30334","Q6UXI7","VIT",0,"Cardiometabolic_II","B13805","20212097_SS211647","PASS",-2.7469,-0.2532,"Plate control","PASS","PFC",0.839033314328603,"Baseline","Baseline"
7691,"PFC_15",74,"OID30334","Q6UXI7","VIT",0,"Cardiometabolic_II","B13805","20212097_SS211647","PASS",-2.7469,0.6057,"Plate control","PASS","PFC",1.52171691580003,"1h","1h"
7692,"PFC_23",75,"OID30334","Q6UXI7","VIT",0,"Cardiometabolic_II","B13805","20212097_SS211647","PASS",-2.7469,0.8103,"Plate control","PASS","PFC",1.75357605061032,"24h","24h"
7693,"PFC_08",85,"OID30334","Q6UXI7","VIT",0,"Cardiometabolic_II","B13805","20212097_SS211647","PASS",-2.7469,0.0127,"Plate control","PASS","PFC",1.00884182927074,"Baseline","Baseline"
7694,"PFC_16",86,"OID30334","Q6UXI7","VIT",0,"Cardiometabolic_II","B13805","20212097_SS211647","PASS",-2.7469,-0.4423,"Plate control","PASS","PFC",0.735960376513244,"1h","1h"
7695,"PFC_24",87,"OID30334","Q6UXI7","VIT",0,"Cardiometabolic_II","B13805","20212097_SS211647","PASS",-2.7469,0.6779,"Plate control","PASS","PFC",1.59980936248735,"24h","24h"
7696,"PFC_01",1,"OID30335","P29692","EEF1D",0,"Cardiometabolic_II","B13805","20212097_SS211647","PASS",-1.7379,1.8981,"Plate control","PASS","PFC",3.72722005947308,"Baseline","Baseline"
7697,"PFC_09",2,"OID30335","P29692","EEF1D",0,"Cardiometabolic_II","B13805","20212097_SS211647","PASS",-1.7379,3.2575,"Plate control","PASS","PFC",9.56324343219548,"Baseline","Baseline"
7698,"PFC_17",3,"OID30335","P29692","EEF1D",0,"Cardiometabolic_II","B13805","20212097_SS211647","PASS",-1.7379,4.2682,"Plate control","PASS","PFC",19.2688691470199,"1h","1h"
7699,"PFC_25",4,"OID30335","P29692","EEF1D",0,"Cardiometabolic_II","B13805","20212097_SS211647","PASS",-1.7379,3.7731,"Plate control","PASS","PFC",13.6715034578015,"24h","24h"
7700,"PFC_02",13,"OID30335","P29692","EEF1D",0,"Cardiometabolic_II","B13805","20212097_SS211647","PASS",-1.7379,2.4662,"Plate control","PASS","PFC",5.52586378686033,"Baseline","Baseline"
7701,"PFC_10",14,"OID30335","P29692","EEF1D",0,"Cardiometabolic_II","B13805","20212097_SS211647","PASS",-1.7379,2.9089,"Plate control","PASS","PFC",7.5104533755229,"1h","1h"
7702,"PFC_18",15,"OID30335","P29692","EEF1D",0,"Cardiometabolic_II","B13805","20212097_SS211647","PASS",-1.7379,3.6029,"Plate control","PASS","PFC",12.1501313064019,"1h","1h"
7703,"PFC_26",16,"OID30335","P29692","EEF1D",0,"Cardiometabolic_II","B13805","20212097_SS211647","PASS",-1.7379,4.0463,"Plate control","PASS","PFC",16.5218118247339,"24h","24h"
7704,"PFC_03",25,"OID30335","P29692","EEF1D",0,"Cardiometabolic_II","B13805","20212097_SS211647","PASS",-1.7379,1.4679,"Plate control","PASS","PFC",2.76618951373042,"Baseline","Baseline"
7705,"PFC_11",26,"OID30335","P29692","EEF1D",0,"Cardiometabolic_II","B13805","20212097_SS211647","PASS",-1.7379,4.0843,"Plate control","PASS","PFC",16.9627714768074,"1h","1h"
7706,"PFC_19",27,"OID30335","P29692","EEF1D",0,"Cardiometabolic_II","B13805","20212097_SS211647","PASS",-1.7379,3.3768,"Plate control","PASS","PFC",10.3876686853663,"24h","24h"
7707,"PFC_27",28,"OID30335","P29692","EEF1D",0,"Cardiometabolic_II","B13805","20212097_SS211647","PASS",-1.7379,4.3035,"Plate control","PASS","PFC",19.7461570277367,"24h","24h"
7708,"PFC_04",37,"OID30335","P29692","EEF1D",0,"Cardiometabolic_II","B13805","20212097_SS211647","PASS",-1.7379,3.3059,"Plate control","PASS","PFC",9.88951657455675,"Baseline","Baseline"
7709,"PFC_12",38,"OID30335","P29692","EEF1D",0,"Cardiometabolic_II","B13805","20212097_SS211647","PASS",-1.7379,1.7821,"Plate control","PASS","PFC",3.43926432834743,"1h","1h"
7710,"PFC_20",39,"OID30335","P29692","EEF1D",0,"Cardiometabolic_II","B13805","20212097_SS211647","PASS",-1.7379,4.4134,"Plate control","PASS","PFC",21.3091331390368,"24h","24h"
7711,"PFC_05",49,"OID30335","P29692","EEF1D",0,"Cardiometabolic_II","B13805","20212097_SS211647","PASS",-1.7379,2.4301,"Plate control","PASS","PFC",5.38930785287085,"Baseline","Baseline"
7712,"PFC_13",50,"OID30335","P29692","EEF1D",0,"Cardiometabolic_II","B13805","20212097_SS211647","PASS",-1.7379,3.659,"Plate control","PASS","PFC",12.6319021872089,"1h","1h"
7713,"PFC_21",51,"OID30335","P29692","EEF1D",0,"Cardiometabolic_II","B13805","20212097_SS211647","PASS",-1.7379,2.4797,"Plate control","PASS","PFC",5.57781467084492,"24h","24h"
7714,"PFC_06",61,"OID30335","P29692","EEF1D",0,"Cardiometabolic_II","B13805","20212097_SS211647","PASS",-1.7379,2.8167,"Plate control","PASS","PFC",7.04548977226767,"Baseline","Baseline"
7715,"PFC_14",62,"OID30335","P29692","EEF1D",0,"Cardiometabolic_II","B13805","20212097_SS211647","PASS",-1.7379,3.9756,"Plate control","PASS","PFC",15.731670834384,"1h","1h"
7716,"PFC_22",63,"OID30335","P29692","EEF1D",0,"Cardiometabolic_II","B13805","20212097_SS211647","PASS",-1.7379,3.2235,"Plate control","PASS","PFC",9.34050142476733,"24h","24h"
7717,"PFC_07",73,"OID30335","P29692","EEF1D",0,"Cardiometabolic_II","B13805","20212097_SS211647","PASS",-1.7379,3.9269,"Plate control","PASS","PFC",15.2094913415349,"Baseline","Baseline"
7718,"PFC_15",74,"OID30335","P29692","EEF1D",0,"Cardiometabolic_II","B13805","20212097_SS211647","PASS",-1.7379,2.4508,"Plate control","PASS","PFC",5.46719184150813,"1h","1h"
7719,"PFC_23",75,"OID30335","P29692","EEF1D",0,"Cardiometabolic_II","B13805","20212097_SS211647","PASS",-1.7379,4.1001,"Plate control","PASS","PFC",17.1495640765826,"24h","24h"
7720,"PFC_08",85,"OID30335","P29692","EEF1D",0,"Cardiometabolic_II","B13805","20212097_SS211647","PASS",-1.7379,2.5281,"Plate control","PASS","PFC",5.76811528726953,"Baseline","Baseline"
7721,"PFC_16",86,"OID30335","P29692","EEF1D",0,"Cardiometabolic_II","B13805","20212097_SS211647","PASS",-1.7379,3.657,"Plate control","PASS","PFC",12.6144027849005,"1h","1h"
7722,"PFC_24",87,"OID30335","P29692","EEF1D",0,"Cardiometabolic_II","B13805","20212097_SS211647","PASS",-1.7379,3.8428,"Plate control","PASS","PFC",14.3482213308806,"24h","24h"
7723,"PFC_01",1,"OID30336","Q9NQR4","NIT2",0,"Cardiometabolic_II","B13805","20212097_SS211647","PASS",-4.509,2.6461,"Plate control","PASS","PFC",6.25972813014536,"Baseline","Baseline"
7724,"PFC_09",2,"OID30336","Q9NQR4","NIT2",0,"Cardiometabolic_II","B13805","20212097_SS211647","PASS",-4.509,3.3911,"Plate control","PASS","PFC",10.4911432791617,"Baseline","Baseline"
7725,"PFC_17",3,"OID30336","Q9NQR4","NIT2",0,"Cardiometabolic_II","B13805","20212097_SS211647","PASS",-4.509,4.7006,"Plate control","PASS","PFC",26.0028887323481,"1h","1h"
7726,"PFC_25",4,"OID30336","Q9NQR4","NIT2",0,"Cardiometabolic_II","B13805","20212097_SS211647","PASS",-4.509,3.8693,"Plate control","PASS","PFC",14.6142106239159,"24h","24h"
7727,"PFC_02",13,"OID30336","Q9NQR4","NIT2",0,"Cardiometabolic_II","B13805","20212097_SS211647","PASS",-4.509,2.6292,"Plate control","PASS","PFC",6.18682831671654,"Baseline","Baseline"
7728,"PFC_10",14,"OID30336","Q9NQR4","NIT2",0,"Cardiometabolic_II","B13805","20212097_SS211647","PASS",-4.509,3.2306,"Plate control","PASS","PFC",9.38658255214008,"1h","1h"

Supplemental table 1_Olink proximity extension assay (PEA) data

,"SampleID","Index","OlinkID","UniProt","Assay","MissingFreq","Panel","Panel_Lot_Nr","PlateID","QC_Warning","LOD","NPX","Normalization","Assay_Warning","condition","Value","group","alt_group"
7729,"PFC_18",15,"OID30336","Q9NQR4","NIT2",0,"Cardiometabolic_II","B13805","20212097_SS211647","PASS",-4.509,3.7484,"Plate control","PASS","PFC",13.4394295716792,"1h","1h"
7730,"PFC_26",16,"OID30336","Q9NQR4","NIT2",0,"Cardiometabolic_II","B13805","20212097_SS211647","PASS",-4.509,4.7187,"Plate control","PASS","PFC",26.3311750656618,"24h","24h"
7731,"PFC_03",25,"OID30336","Q9NQR4","NIT2",0,"Cardiometabolic_II","B13805","20212097_SS211647","PASS",-4.509,1.8618,"Plate control","PASS","PFC",3.63460856713114,"Baseline","Baseline"
7732,"PFC_11",26,"OID30336","Q9NQR4","NIT2",0,"Cardiometabolic_II","B13805","20212097_SS211647","PASS",-4.509,3.916,"Plate control","PASS","PFC",15.0950120143258,"1h","1h"
7733,"PFC_19",27,"OID30336","Q9NQR4","NIT2",0,"Cardiometabolic_II","B13805","20212097_SS211647","PASS",-4.509,3.6998,"Plate control","PASS","PFC",12.9942368331067,"24h","24h"
7734,"PFC_27",28,"OID30336","Q9NQR4","NIT2",0,"Cardiometabolic_II","B13805","20212097_SS211647","PASS",-4.509,5.1118,"Plate control","PASS","PFC",34.5784189462576,"24h","24h"
7735,"PFC_04",37,"OID30336","Q9NQR4","NIT2",0,"Cardiometabolic_II","B13805","20212097_SS211647","PASS",-4.509,3.7767,"Plate control","PASS","PFC",13.7056609678776,"Baseline","Baseline"
7736,"PFC_12",38,"OID30336","Q9NQR4","NIT2",0,"Cardiometabolic_II","B13805","20212097_SS211647","PASS",-4.509,2.1626,"Plate control","PASS","PFC",4.47721003630855,"1h","1h"
7737,"PFC_20",39,"OID30336","Q9NQR4","NIT2",0,"Cardiometabolic_II","B13805","20212097_SS211647","PASS",-4.509,4.1239,"Plate control","PASS","PFC",17.4348252635317,"24h","24h"
7738,"PFC_05",49,"OID30336","Q9NQR4","NIT2",0,"Cardiometabolic_II","B13805","20212097_SS211647","PASS",-4.509,2.825,"Plate control","PASS","PFC",7.08614015281888,"Baseline","Baseline"
7739,"PFC_13",50,"OID30336","Q9NQR4","NIT2",0,"Cardiometabolic_II","B13805","20212097_SS211647","PASS",-4.509,3.978,"Plate control","PASS","PFC",15.7578630863288,"1h","1h"
7740,"PFC_21",51,"OID30336","Q9NQR4","NIT2",0,"Cardiometabolic_II","B13805","20212097_SS211647","PASS",-4.509,2.962,"Plate control","PASS","PFC",7.79203414834593,"24h","24h"
7741,"PFC_06",61,"OID30336","Q9NQR4","NIT2",0,"Cardiometabolic_II","B13805","20212097_SS211647","PASS",-4.509,3.7234,"Plate control","PASS","PFC",13.2085482157445,"Baseline","Baseline"
7742,"PFC_14",62,"OID30336","Q9NQR4","NIT2",0,"Cardiometabolic_II","B13805","20212097_SS211647","PASS",-4.509,3.7988,"Plate control","PASS","PFC",13.917228172107,"1h","1h"
7743,"PFC_22",63,"OID30336","Q9NQR4","NIT2",0,"Cardiometabolic_II","B13805","20212097_SS211647","PASS",-4.509,3.4588,"Plate control","PASS","PFC",10.9951852025934,"24h","24h"
7744,"PFC_07",73,"OID30336","Q9NQR4","NIT2",0,"Cardiometabolic_II","B13805","20212097_SS211647","PASS",-4.509,4.5261,"Plate control","PASS","PFC",23.0404980865156,"Baseline","Baseline"
7745,"PFC_15",74,"OID30336","Q9NQR4","NIT2",0,"Cardiometabolic_II","B13805","20212097_SS211647","PASS",-4.509,2.9083,"Plate control","PASS","PFC",7.50733051519928,"1h","1h"
7746,"PFC_23",75,"OID30336","Q9NQR4","NIT2",0,"Cardiometabolic_II","B13805","20212097_SS211647","PASS",-4.509,4.148,"Plate control","PASS","PFC",17.7285175658388,"24h","24h"
7747,"PFC_08",85,"OID30336","Q9NQR4","NIT2",0,"Cardiometabolic_II","B13805","20212097_SS211647","PASS",-4.509,3.5156,"Plate control","PASS","PFC",11.4367085223619,"Baseline","Baseline"
7748,"PFC_16",86,"OID30336","Q9NQR4","NIT2",0,"Cardiometabolic_II","B13805","20212097_SS211647","PASS",-4.509,4.041,"Plate control","PASS","PFC",16.4612273262972,"1h","1h"
7749,"PFC_24",87,"OID30336","Q9NQR4","NIT2",0,"Cardiometabolic_II","B13805","20212097_SS211647","PASS",-4.509,4.1851,"Plate control","PASS","PFC",18.1903323858399,"24h","24h"
7750,"PFC_01",1,"OID30337","Q9BQS7","HEPH",0,"Cardiometabolic_II","B13805","20212097_SS211647","PASS",-5.4168,0.0157,"Plate control","PASS","PFC",1.0109418395471,"Baseline","Baseline"
7751,"PFC_09",2,"OID30337","Q9BQS7","HEPH",0,"Cardiometabolic_II","B13805","20212097_SS211647","PASS",-5.4168,-0.0255,"Plate control","PASS","PFC",0.982480037899884,"Baseline","Baseline"
7752,"PFC_17",3,"OID30337","Q9BQS7","HEPH",0,"Cardiometabolic_II","B13805","20212097_SS211647","PASS",-5.4168,-0.5031,"Plate control","PASS","PFC",0.705589012305844,"1h","1h"
7753,"PFC_25",4,"OID30337","Q9BQS7","HEPH",0,"Cardiometabolic_II","B13805","20212097_SS211647","PASS",-5.4168,-0.2679,"Plate control","PASS","PFC",0.830527589881523,"24h","24h"
7754,"PFC_02",13,"OID30337","Q9BQS7","HEPH",0,"Cardiometabolic_II","B13805","20212097_SS211647","PASS",-5.4168,0.0571,"Plate control","PASS","PFC",1.04037237714079,"Baseline","Baseline"
7755,"PFC_10",14,"OID30337","Q9BQS7","HEPH",0,"Cardiometabolic_II","B13805","20212097_SS211647","PASS",-5.4168,-0.0945,"Plate control","PASS","PFC",0.936596790904614,"1h","1h"
7756,"PFC_18",15,"OID30337","Q9BQS7","HEPH",0,"Cardiometabolic_II","B13805","20212097_SS211647","PASS",-5.4168,-0.0092,"Plate control","PASS","PFC",0.99364333555883,"1h","1h"
7757,"PFC_26",16,"OID30337","Q9BQS7","HEPH",0,"Cardiometabolic_II","B13805","20212097_SS211647","PASS",-5.4168,-0.3436,"Plate control","PASS","PFC",0.78807235581421,"24h","24h"
7758,"PFC_03",25,"OID30337","Q9BQS7","HEPH",0,"Cardiometabolic_II","B13805","20212097_SS211647","PASS",-5.4168,-0.2401,"Plate control","PASS","PFC",0.846686622483995,"Baseline","Baseline"
7759,"PFC_11",26,"OID30337","Q9BQS7","HEPH",0,"Cardiometabolic_II","B13805","20212097_SS211647","PASS",-5.4168,0.199,"Plate control","PASS","PFC",1.147902413855,"1h","1h"
7760,"PFC_19",27,"OID30337","Q9BQS7","HEPH",0,"Cardiometabolic_II","B13805","20212097_SS211647","PASS",-5.4168,0.2347,"Plate control","PASS","PFC",1.17666203178618,"24h","24h"
7761,"PFC_27",28,"OID30337","Q9BQS7","HEPH",0,"Cardiometabolic_II","B13805","20212097_SS211647","PASS",-5.4168,0.3358,"Plate control","PASS","PFC",1.26207705895317,"24h","24h"
7762,"PFC_04",37,"OID30337","Q9BQS7","HEPH",0,"Cardiometabolic_II","B13805","20212097_SS211647","PASS",-5.4168,-0.1039,"Plate control","PASS","PFC",0.930514153793943,"Baseline","Baseline"
7763,"PFC_12",38,"OID30337","Q9BQS7","HEPH",0,"Cardiometabolic_II","B13805","20212097_SS211647","PASS",-5.4168,-0.2779,"Plate control","PASS","PFC",0.824790716764749,"1h","1h"
7764,"PFC_20",39,"OID30337","Q9BQS7","HEPH",0,"Cardiometabolic_II","B13805","20212097_SS211647","PASS",-5.4168,0.1953,"Plate control","PASS","PFC",1.14496222404711,"24h","24h"
7765,"PFC_05",49,"OID30337","Q9BQS7","HEPH",0,"Cardiometabolic_II","B13805","20212097_SS211647","PASS",-5.4168,0.1957,"Plate control","PASS","PFC",1.1452797189942,"Baseline","Baseline"
7766,"PFC_13",50,"OID30337","Q9BQS7","HEPH",0,"Cardiometabolic_II","B13805","20212097_SS211647","PASS",-5.4168,-0.2515,"Plate control","PASS","PFC",0.840022572139788,"1h","1h"
7767,"PFC_21",51,"OID30337","Q9BQS7","HEPH",0,"Cardiometabolic_II","B13805","20212097_SS211647","PASS",-5.4168,-0.2671,"Plate control","PASS","PFC",0.830988259880406,"24h","24h"
7768,"PFC_06",61,"OID30337","Q9BQS7","HEPH",0,"Cardiometabolic_II","B13805","20212097_SS211647","PASS",-5.4168,-0.2691,"Plate control","PASS","PFC",0.829837063674347,"Baseline","Baseline"
7769,"PFC_14",62,"OID30337","Q9BQS7","HEPH",0,"Cardiometabolic_II","B13805","20212097_SS211647","PASS",-5.4168,0.2353,"Plate control","PASS","PFC",1.17715149354171,"1h","1h"
7770,"PFC_22",63,"OID30337","Q9BQS7","HEPH",0,"Cardiometabolic_II","B13805","20212097_SS211647","PASS",-5.4168,-0.1057,"Plate control","PASS","PFC",0.929353907871611,"24h","24h"
7771,"PFC_07",73,"OID30337","Q9BQS7","HEPH",0,"Cardiometabolic_II","B13805","20212097_SS211647","PASS",-5.4168,0.0527,"Plate control","PASS","PFC",1.03720423358225,"Baseline","Baseline"
7772,"PFC_15",74,"OID30337","Q9BQS7","HEPH",0,"Cardiometabolic_II","B13805","20212097_SS211647","PASS",-5.4168,-0.1995,"Plate control","PASS","PFC",0.870852325418737,"1h","1h"
7773,"PFC_23",75,"OID30337","Q9BQS7","HEPH",0,"Cardiometabolic_II","B13805","20212097_SS211647","PASS",-5.4168,0.0815,"Plate control","PASS","PFC",1.0581176156998,"24h","24h"
7774,"PFC_08",85,"OID30337","Q9BQS7","HEPH",0,"Cardiometabolic_II","B13805","20212097_SS211647","PASS",-5.4168,-0.1904,"Plate control","PASS","PFC",0.876362708292673,"Baseline","Baseline"
7775,"PFC_16",86,"OID30337","Q9BQS7","HEPH",0,"Cardiometabolic_II","B13805","20212097_SS211647","PASS",-5.4168,-0.043,"Plate control","PASS","PFC",0.970634469769547,"1h","1h"
7776,"PFC_24",87,"OID30337","Q9BQS7","HEPH",0,"Cardiometabolic_II","B13805","20212097_SS211647","PASS",-5.4168,-0.2097,"Plate control","PASS","PFC",0.8647170254641,"24h","24h"
7777,"PFC_01",1,"OID30338","Q9H3S4","TPK1",0,"Cardiometabolic_II","B13805","20212097_SS211647","PASS",-5.2213,0.2833,"Plate control","PASS","PFC",1.21697539524362,"Baseline","Baseline"
7778,"PFC_09",2,"OID30338","Q9H3S4","TPK1",0,"Cardiometabolic_II","B13805","20212097_SS211647","PASS",-5.2213,0.1244,"Plate control","PASS","PFC",1.09005429754481,"Baseline","Baseline"
7779,"PFC_17",3,"OID30338","Q9H3S4","TPK1",0,"Cardiometabolic_II","B13805","20212097_SS211647","PASS",-5.2213,0.7202,"Plate control","PASS","PFC",1.64741039828037,"1h","1h"
7780,"PFC_25",4,"OID30338","Q9H3S4","TPK1",0,"Cardiometabolic_II","B13805","20212097_SS211647","PASS",-5.2213,0.4672,"Plate control","PASS","PFC",1.38242383788252,"24h","24h"
7781,"PFC_02",13,"OID30338","Q9H3S4","TPK1",0,"Cardiometabolic_II","B13805","20212097_SS211647","PASS",-5.2213,0.9229,"Plate control","PASS","PFC",1.89592251036841,"Baseline","Baseline"
7782,"PFC_10",14,"OID30338","Q9H3S4","TPK1",0,"Cardiometabolic_II","B13805","20212097_SS211647","PASS",-5.2213,0.4852,"Plate control","PASS","PFC",1.39977990284386,"1h","1h"
7783,"PFC_18",15,"OID30338","Q9H3S4","TPK1",0,"Cardiometabolic_II","B13805","20212097_SS211647","PASS",-5.2213,0.237,"Plate control","PASS","PFC",1.17853940781168,"1h","1h"
7784,"PFC_26",16,"OID30338","Q9H3S4","TPK1",0,"Cardiometabolic_II","B13805","20212097_SS211647","PASS",-5.2213,0.7991,"Plate control","PASS","PFC",1.74001530990804,"24h","24h"
7785,"PFC_03",25,"OID30338","Q9H3S4","TPK1",0,"Cardiometabolic_II","B13805","20212097_SS211647","PASS",-5.2213,0.283,"Plate control","PASS","PFC",1.21672235863407,"Baseline","Baseline"
7786,"PFC_11",26,"OID30338","Q9H3S4","TPK1",0,"Cardiometabolic_II","B13805","20212097_SS211647","PASS",-5.2213,1.072,"Plate control","PASS","PFC",2.10234581817541,"1h","1h"
7787,"PFC_19",27,"OID30338","Q9H3S4","TPK1",0,"Cardiometabolic_II","B13805","20212097_SS211647","PASS",-5.2213,0.6408,"Plate control","PASS","PFC",1.55919352012109,"24h","24h"
7788,"PFC_27",28,"OID30338","Q9H3S4","TPK1",0,"Cardiometabolic_II","B13805","20212097_SS211647","PASS",-5.2213,0.7944,"Plate control","PASS","PFC",1.73435592594147,"24h","24h"
7789,"PFC_04",37,"OID30338","Q9H3S4","TPK1",0,"Cardiometabolic_II","B13805","20212097_SS211647","PASS",-5.2213,1.127,"Plate control","PASS","PFC",2.1840410914913,"Baseline","Baseline"
7790,"PFC_12",38,"OID30338","Q9H3S4","TPK1",0,"Cardiometabolic_II","B13805","20212097_SS211647","PASS",-5.2213,0.2216,"Plate control","PASS","PFC",1.16602603390352,"1h","1h"
7791,"PFC_20",39,"OID30338","Q9H3S4","TPK1",0,"Cardiometabolic_II","B13805","20212097_SS211647","PASS",-5.2213,1.0439,"Plate control","PASS","PFC",2.0617937199861,"24h","24h"
7792,"PFC_05",49,"OID30338","Q9H3S4","TPK1",0,"Cardiometabolic_II","B13805","20212097_SS211647","PASS",-5.2213,0.0986,"Plate control","PASS","PFC",1.07073391155589,"Baseline","Baseline"
7793,"PFC_13",50,"OID30338","Q9H3S4","TPK1",0,"Cardiometabolic_II","B13805","20212097_SS211647","PASS",-5.2213,0.9597,"Plate control","PASS","PFC",1.94490542105933,"1h","1h"
7794,"PFC_21",51,"OID30338","Q9H3S4","TPK1",0,"Cardiometabolic_II","B13805","20212097_SS211647","PASS",-5.2213,0.2655,"Plate control","PASS","PFC",1.20205257702129,"24h","24h"
7795,"PFC_06",61,"OID30338","Q9H3S4","TPK1",0,"Cardiometabolic_II","B13805","20212097_SS211647","PASS",-5.2213,0.545,"Plate control","PASS","PFC",1.45902034424018,"Baseline","Baseline"
7796,"PFC_14",62,"OID30338","Q9H3S4","TPK1",0,"Cardiometabolic_II","B13805","20212097_SS211647","PASS",-5.2213,0.3102,"Plate control","PASS","PFC",1.23987957183135,"1h","1h"
7797,"PFC_22",63,"OID30338","Q9H3S4","TPK1",0,"Cardiometabolic_II","B13805","20212097_SS211647","PASS",-5.2213,0.7571,"Plate control","PASS","PFC",1.69008991287088,"24h","24h"
7798,"PFC_07",73,"OID30338","Q9H3S4","TPK1",0,"Cardiometabolic_II","B13805","20212097_SS211647","PASS",-5.2213,0.7093,"Plate control","PASS","PFC",1.63501061252833,"Baseline","Baseline"
7799,"PFC_15",74,"OID30338","Q9H3S4","TPK1",0,"Cardiometabolic_II","B13805","20212097_SS211647","PASS",-5.2213,0.4243,"Plate control","PASS","PFC",1.34192124054874,"1h","1h"
7800,"PFC_23",75,"OID30338","Q9H3S4","TPK1",0,"Cardiometabolic_II","B13805","20212097_SS211647","PASS",-5.2213,0.5953,"Plate control","PASS","PFC",1.51078671216629,"24h","24h"
7801,"PFC_08",85,"OID30338","Q9H3S4","TPK1",0,"Cardiometabolic_II","B13805","20212097_SS211647","PASS",-5.2213,0.3357,"Plate control","PASS","PFC",1.26198958146944,"Baseline","Baseline"
7802,"PFC_16",86,"OID30338","Q9H3S4","TPK1",0,"Cardiometabolic_II","B13805","20212097_SS211647","PASS",-5.2213,0.5634,"Plate control","PASS","PFC",1.47774772545653,"1h","1h"
7803,"PFC_24",87,"OID30338","Q9H3S4","TPK1",0,"Cardiometabolic_II","B13805","20212097_SS211647","PASS",-5.2213,0.7602,"Plate control","PASS","PFC",1.69372540864903,"24h","24h"
7804,"PFC_01",1,"OID30339","Q96C24","SYTL4",0.0227,"Cardiometabolic_II","B13805","20212097_SS211647","PASS",0.1016,1.6112,"Plate control","PASS","PFC",3.05505848760194,"Baseline","Baseline"
7805,"PFC_09",2,"OID30339","Q96C24","SYTL4",0.0227,"Cardiometabolic_II","B13805","20212097_SS211647","PASS",0.1016,2.51,"Plate control","PASS","PFC",5.69620078238829,"Baseline","Baseline"
7806,"PFC_17",3,"OID30339","Q96C24","SYTL4",0.0227,"Cardiometabolic_II","B13805","20212097_SS211647","PASS",0.1016,4.214,"Plate control","PASS","PFC",18.5583944720869,"1h","1h"
7807,"PFC_25",4,"OID30339","Q96C24","SYTL4",0.0227,"Cardiometabolic_II","B13805","20212097_SS211647","PASS",0.1016,2.9168,"Plate control","PASS","PFC",7.55169239377574,"24h","24h"
7808,"PFC_02",13,"OID30339","Q96C24","SYTL4",0.0227,"Cardiometabolic_II","B13805","20212097_SS211647","PASS",0.1016,1.7009,"Plate control","PASS","PFC",3.25103705541036,"Baseline","Baseline"
7809,"PFC_10",14,"OID30339","Q96C24","SYTL4",0.0227,"Cardiometabolic_II","B13805","20212097_SS211647","PASS",0.1016,2.2198,"Plate control","PASS","PFC",4.65828852519848,"1h","1h"
7810,"PFC_18",15,"OID30339","Q96C24","SYTL4",0.0227,"Cardiometabolic_II","B13805","20212097_SS211647","PASS",0.1016,3.6886,"Plate control","PASS","PFC",12.8937499016979,"1h","1h"
7811,"PFC_26",16,"OID30339","Q96C24","SYTL4",0.0227,"Cardiometabolic_II","B13805","20212097_SS211647","PASS",0.1016,3.7384,"Plate control","PASS","PFC",13.3465966505892,"24h","24h"
7812,"PFC_03",25,"OID30339","Q96C24","SYTL4",0.0227,"Cardiometabolic_II","B13805","20212097_SS211647","PASS",0.1016,0.3604,"Plate control","PASS","PFC",1.28378178811447,"Baseline","Baseline"
7813,"PFC_11",26,"OID30339","Q96C24","SYTL4",0.0227,"Cardiometabolic_II","B13805","20212097_SS211647","PASS",0.1016,3.5929,"Plate control","PASS","PFC",12.0662042189623,"1h","1h"
7814,"PFC_19",27,"OID30339","Q96C24","SYTL4",0.0227,"Cardiometabolic_II","B13805","20212097_SS211647","PASS",0.1016,2.9159,"Plate control","PASS","PFC",7.54698287204266,"24h","24h"
7815,"PFC_27",28,"OID30339","Q96C24","SYTL4",0.0227,"Cardiometabolic_II","B13805","20212097_SS211647","PASS",0.1016,4.5814,"Plate control","PASS","PFC",23.9408090153963,"24h","24h"
7816,"PFC_04",37,"OID30339","Q96C24","SYTL4",0.0227,"Cardiometabolic_II","B13805","20212097_SS211647","PASS",0.1016,2.2726,"Plate control","PASS","PFC",4.8319314919452,"Baseline","Baseline"
7817,"PFC_12",38,"OID30339","Q96C24","SYTL4",0.0227,"Cardiometabolic_II","B13805","20212097_SS211647","PASS",0.1016,0.5379,"Plate control","PASS","PFC",1.451857641313,"1h","1h"
7818,"PFC_20",39,"OID30339","Q96C24","SYTL4",0.0227,"Cardiometabolic_II","B13805","20212097_SS211647","PASS",0.1016,4.4621,"Plate control","PASS","PFC",22.040728436061,"24h","24h"
7819,"PFC_05",49,"OID30339","Q96C24","SYTL4",0.0227,"Cardiometabolic_II","B13805","20212097_SS211647","PASS",0.1016,1.0863,"Plate control","PASS","PFC",2.12328789730233,"Baseline","Baseline"
7820,"PFC_13",50,"OID30339","Q96C24","SYTL4",0.0227,"Cardiometabolic_II","B13805","20212097_SS211647","PASS",0.1016,2.4538,"Plate control","PASS","PFC",5.47857237581674,"1h","1h"
7821,"PFC_21",51,"OID30339","Q96C24","SYTL4",0.0227,"Cardiometabolic_II","B13805","20212097_SS211647","PASS",0.1016,1.4077,"Plate control","PASS","PFC",2.65313851918321,"24h","24h"
7822,"PFC_06",61,"OID30339","Q96C24","SYTL4",0.0227,"Cardiometabolic_II","B13805","20212097_SS211647","PASS",0.1016,2.2401,"Plate control","PASS","PFC",4.72429809776003,"Baseline","Baseline"
7823,"PFC_14",62,"OID30339","Q96C24","SYTL4",0.0227,"Cardiometabolic_II","B13805","20212097_SS211647","PASS",0.1016,2.8663,"Plate control","PASS","PFC",7.29192640092149,"1h","1h"
7824,"PFC_22",63,"OID30339","Q96C24","SYTL4",0.0227,"Cardiometabolic_II","B13805","20212097_SS211647","PASS",0.1016,2.3804,"Plate control","PASS","PFC",5.20681085616185,"24h","24h"
7825,"PFC_07",73,"OID30339","Q96C24","SYTL4",0.0227,"Cardiometabolic_II","B13805","20212097_SS211647","PASS",0.1016,4.0204,"Plate control","PASS","PFC",16.2278503684523,"Baseline","Baseline"
7826,"PFC_15",74,"OID30339","Q96C24","SYTL4",0.0227,"Cardiometabolic_II","B13805","20212097_SS211647","PASS",0.1016,1.3182,"Plate control","PASS","PFC",2.49354804374987,"1h","1h"
7827,"PFC_23",75,"OID30339","Q96C24","SYTL4",0.0227,"Cardiometabolic_II","B13805","20212097_SS211647","PASS",0.1016,3.1335,"Plate control","PASS","PFC",8.77561357763479,"24h","24h"
7828,"PFC_08",85,"OID30339","Q96C24","SYTL4",0.0227,"Cardiometabolic_II","B13805","20212097_SS211647","PASS",0.1016,1.4947,"Plate control","PASS","PFC",2.81805545119517,"Baseline","Baseline"
7829,"PFC_16",86,"OID30339","Q96C24","SYTL4",0.0227,"Cardiometabolic_II","B13805","20212097_SS211647","PASS",0.1016,2.4956,"Plate control","PASS","PFC",5.63962798825563,"1h","1h"
7830,"PFC_24",87,"OID30339","Q96C24","SYTL4",0.0227,"Cardiometabolic_II","B13805","20212097_SS211647","PASS",0.1016,3.7508,"Plate control","PASS","PFC",13.4618053847153,"24h","24h"
7831,"PFC_01",1,"OID30340","O60234","GMFG",0,"Cardiometabolic_II","B13805","20212097_SS211647","PASS",-1.4386,2.4096,"Plate control","PASS","PFC",5.3132699009245,"Baseline","Baseline"
7832,"PFC_09",2,"OID30340","O60234","GMFG",0,"Cardiometabolic_II","B13805","20212097_SS211647","PASS",-1.4386,3.3693,"Plate control","PASS","PFC",10.3338074338594,"Baseline","Baseline"
7833,"PFC_17",3,"OID30340","O60234","GMFG",0,"Cardiometabolic_II","B13805","20212097_SS211647","PASS",-1.4386,4.2267,"Plate control","PASS","PFC",18.7224846275482,"1h","1h"
7834,"PFC_25",4,"OID30340","O60234","GMFG",0,"Cardiometabolic_II","B13805","20212097_SS211647","PASS",-1.4386,4.0771,"Plate control","PASS","PFC",16.8783269490174,"24h","24h"
7835,"PFC_02",13,"OID30340","O60234","GMFG",0,"Cardiometabolic_II","B13805","20212097_SS211647","PASS",-1.4386,2.7984,"Plate control","PASS","PFC",6.95668501599801,"Baseline","Baseline"
7836,"PFC_10",14,"OID30340","O60234","GMFG",0,"Cardiometabolic_II","B13805","20212097_SS211647","PASS",-1.4386,3.0715,"Plate control","PASS","PFC",8.40646930752964,"1h","1h"
7837,"PFC_18",15,"OID30340","O60234","GMFG",0,"Cardiometabolic_II","B13805","20212097_SS211647","PASS",-1.4386,3.9762,"Plate control","PASS","PFC",15.7382148130424,"1h","1h"
7838,"PFC_26",16,"OID30340","O60234","GMFG",0,"Cardiometabolic_II","B13805","20212097_SS211647","PASS",-1.4386,4.4971,"Plate control","PASS","PFC",22.5819787037794,"24h","24h"
7839,"PFC_03",25,"OID30340","O60234","GMFG",0,"Cardiometabolic_II","B13805","20212097_SS211647","PASS",-1.4386,2.0157,"Plate control","PASS","PFC",4.0437673581884,"Baseline","Baseline"
7840,"PFC_11",26,"OID30340","O60234","GMFG",0,"Cardiometabolic_II","B13805","20212097_SS211647","PASS",-1.4386,4.1135,"Plate control","PASS","PFC",17.3095942245188,"1h","1h"

Supplemental table 1_Olink proximity extension assay (PEA) data

,"SampleID","Index","OlinkID","UniProt","Assay","MissingFreq","Panel","Panel_Lot_Nr","PlateID","QC_Warning","LOD","NPX","Normalization","Assay_Warning","condition","Value","group","alt_group"
7841,"PFC_19",27,"OID30340","O60234","GMFG",0,"Cardiometabolic_II","B13805","20212097_SS211647","PASS",-1.4386,3.735,"Plate control","PASS","PFC",13.3151797553844,"24h","24h"
7842,"PFC_27",28,"OID30340","O60234","GMFG",0,"Cardiometabolic_II","B13805","20212097_SS211647","PASS",-1.4386,4.7927,"Plate control","PASS","PFC",27.7170152142239,"24h","24h"
7843,"PFC_04",37,"OID30340","O60234","GMFG",0,"Cardiometabolic_II","B13805","20212097_SS211647","PASS",-1.4386,4.2066,"Plate control","PASS","PFC",18.4634468160967,"Baseline","Baseline"
7844,"PFC_12",38,"OID30340","O60234","GMFG",0,"Cardiometabolic_II","B13805","20212097_SS211647","PASS",-1.4386,2.5471,"Plate control","PASS","PFC",5.84458261573896,"1h","1h"
7845,"PFC_20",39,"OID30340","O60234","GMFG",0,"Cardiometabolic_II","B13805","20212097_SS211647","PASS",-1.4386,4.344,"Plate control","PASS","PFC",20.3083341763169,"24h","24h"
7846,"PFC_05",49,"OID30340","O60234","GMFG",0,"Cardiometabolic_II","B13805","20212097_SS211647","PASS",-1.4386,2.6792,"Plate control","PASS","PFC",6.40500634612523,"Baseline","Baseline"
7847,"PFC_13",50,"OID30340","O60234","GMFG",0,"Cardiometabolic_II","B13805","20212097_SS211647","PASS",-1.4386,4.3023,"Plate control","PASS","PFC",19.7297394648709,"1h","1h"
7848,"PFC_21",51,"OID30340","O60234","GMFG",0,"Cardiometabolic_II","B13805","20212097_SS211647","PASS",-1.4386,3.4258,"Plate control","PASS","PFC",10.7465375160655,"24h","24h"
7849,"PFC_06",61,"OID30340","O60234","GMFG",0,"Cardiometabolic_II","B13805","20212097_SS211647","PASS",-1.4386,3.8852,"Plate control","PASS","PFC",14.7761652411471,"Baseline","Baseline"
7850,"PFC_14",62,"OID30340","O60234","GMFG",0,"Cardiometabolic_II","B13805","20212097_SS211647","PASS",-1.4386,4.0234,"Plate control","PASS","PFC",16.2616303442108,"1h","1h"
7851,"PFC_22",63,"OID30340","O60234","GMFG",0,"Cardiometabolic_II","B13805","20212097_SS211647","PASS",-1.4386,3.9269,"Plate control","PASS","PFC",15.2094913415349,"24h","24h"
7852,"PFC_07",73,"OID30340","O60234","GMFG",0,"Cardiometabolic_II","B13805","20212097_SS211647","PASS",-1.4386,4.3654,"Plate control","PASS","PFC",20.6118200899822,"Baseline","Baseline"
7853,"PFC_15",74,"OID30340","O60234","GMFG",0,"Cardiometabolic_II","B13805","20212097_SS211647","PASS",-1.4386,3.0551,"Plate control","PASS","PFC",8.31144891215346,"1h","1h"
7854,"PFC_23",75,"OID30340","O60234","GMFG",0,"Cardiometabolic_II","B13805","20212097_SS211647","PASS",-1.4386,4.234,"Plate control","PASS","PFC",18.8174600046554,"24h","24h"
7855,"PFC_08",85,"OID30340","O60234","GMFG",0,"Cardiometabolic_II","B13805","20212097_SS211647","PASS",-1.4386,3.9419,"Plate control","PASS","PFC",15.3684525263819,"Baseline","Baseline"
7856,"PFC_16",86,"OID30340","O60234","GMFG",0,"Cardiometabolic_II","B13805","20212097_SS211647","PASS",-1.4386,4.0822,"Plate control","PASS","PFC",16.938098274263,"1h","1h"
7857,"PFC_24",87,"OID30340","O60234","GMFG",0,"Cardiometabolic_II","B13805","20212097_SS211647","PASS",-1.4386,4.4614,"Plate control","PASS","PFC",22.0300368019364,"24h","24h"
7858,"PFC_01",1,"OID30341","P51688","SGSH",0,"Cardiometabolic_II","B13805","20212097_SS211647","PASS",-2.1094,0.0199,"Plate control","PASS","PFC",1.01388919991031,"Baseline","Baseline"
7859,"PFC_09",2,"OID30341","P51688","SGSH",0,"Cardiometabolic_II","B13805","20212097_SS211647","PASS",-2.1094,-0.8198,"Plate control","PASS","PFC",0.566520473618225,"Baseline","Baseline"
7860,"PFC_17",3,"OID30341","P51688","SGSH",0,"Cardiometabolic_II","B13805","20212097_SS211647","PASS",-2.1094,-0.3241,"Plate control","PASS","PFC",0.7987965461728,"1h","1h"
7861,"PFC_25",4,"OID30341","P51688","SGSH",0,"Cardiometabolic_II","B13805","20212097_SS211647","PASS",-2.1094,-0.9866,"Plate control","PASS","PFC",0.504665720575217,"24h","24h"
7862,"PFC_02",13,"OID30341","P51688","SGSH",0,"Cardiometabolic_II","B13805","20212097_SS211647","PASS",-2.1094,0.6226,"Plate control","PASS","PFC",1.53964740838434,"Baseline","Baseline"
7863,"PFC_10",14,"OID30341","P51688","SGSH",0,"Cardiometabolic_II","B13805","20212097_SS211647","PASS",-2.1094,0.5788,"Plate control","PASS","PFC",1.49360638494861,"1h","1h"
7864,"PFC_18",15,"OID30341","P51688","SGSH",0,"Cardiometabolic_II","B13805","20212097_SS211647","PASS",-2.1094,-0.8886,"Plate control","PASS","PFC",0.540138017214983,"1h","1h"
7865,"PFC_26",16,"OID30341","P51688","SGSH",0,"Cardiometabolic_II","B13805","20212097_SS211647","PASS",-2.1094,-0.3389,"Plate control","PASS","PFC",0.790643917964908,"24h","24h"
7866,"PFC_03",25,"OID30341","P51688","SGSH",0,"Cardiometabolic_II","B13805","20212097_SS211647","PASS",-2.1094,0.2178,"Plate control","PASS","PFC",1.16295881004833,"Baseline","Baseline"
7867,"PFC_11",26,"OID30341","P51688","SGSH",0,"Cardiometabolic_II","B13805","20212097_SS211647","PASS",-2.1094,0.1597,"Plate control","PASS","PFC",1.11705482889784,"1h","1h"
7868,"PFC_19",27,"OID30341","P51688","SGSH",0,"Cardiometabolic_II","B13805","20212097_SS211647","PASS",-2.1094,0.6363,"Plate control","PASS","PFC",1.5543377194074,"24h","24h"
7869,"PFC_27",28,"OID30341","P51688","SGSH",0,"Cardiometabolic_II","B13805","20212097_SS211647","PASS",-2.1094,-0.0248,"Plate control","PASS","PFC",0.982956855855045,"24h","24h"
7870,"PFC_04",37,"OID30341","P51688","SGSH",0,"Cardiometabolic_II","B13805","20212097_SS211647","PASS",-2.1094,0.1144,"Plate control","PASS","PFC",1.08252474251064,"Baseline","Baseline"
7871,"PFC_12",38,"OID30341","P51688","SGSH",0,"Cardiometabolic_II","B13805","20212097_SS211647","PASS",-2.1094,0.186,"Plate control","PASS","PFC",1.1376052278725,"1h","1h"
7872,"PFC_20",39,"OID30341","P51688","SGSH",0,"Cardiometabolic_II","B13805","20212097_SS211647","PASS",-2.1094,0.0411,"Plate control","PASS","PFC",1.02889802319833,"24h","24h"
7873,"PFC_05",49,"OID30341","P51688","SGSH",0,"Cardiometabolic_II","B13805","20212097_SS211647","PASS",-2.1094,1.9651,"Plate control","PASS","PFC",3.90439766594911,"Baseline","Baseline"
7874,"PFC_13",50,"OID30341","P51688","SGSH",0,"Cardiometabolic_II","B13805","20212097_SS211647","PASS",-2.1094,0.7777,"Plate control","PASS","PFC",1.71439553766989,"1h","1h"
7875,"PFC_21",51,"OID30341","P51688","SGSH",0,"Cardiometabolic_II","B13805","20212097_SS211647","PASS",-2.1094,0.4491,"Plate control","PASS","PFC",1.36518834225073,"24h","24h"
7876,"PFC_06",61,"OID30341","P51688","SGSH",0,"Cardiometabolic_II","B13805","20212097_SS211647","PASS",-2.1094,-0.209,"Plate control","PASS","PFC",0.865136690584988,"Baseline","Baseline"
7877,"PFC_14",62,"OID30341","P51688","SGSH",0,"Cardiometabolic_II","B13805","20212097_SS211647","PASS",-2.1094,1.9805,"Plate control","PASS","PFC",3.94629826375704,"1h","1h"
7878,"PFC_22",63,"OID30341","P51688","SGSH",0,"Cardiometabolic_II","B13805","20212097_SS211647","PASS",-2.1094,0.23,"Plate control","PASS","PFC",1.17283494923188,"24h","24h"
7879,"PFC_07",73,"OID30341","P51688","SGSH",0,"Cardiometabolic_II","B13805","20212097_SS211647","PASS",-2.1094,-0.856,"Plate control","PASS","PFC",0.552482242399956,"Baseline","Baseline"
7880,"PFC_15",74,"OID30341","P51688","SGSH",0,"Cardiometabolic_II","B13805","20212097_SS211647","PASS",-2.1094,-0.132,"Plate control","PASS","PFC",0.912565488543452,"1h","1h"
7881,"PFC_23",75,"OID30341","P51688","SGSH",0,"Cardiometabolic_II","B13805","20212097_SS211647","PASS",-2.1094,2.0188,"Plate control","PASS","PFC",4.05246577064951,"24h","24h"
7882,"PFC_08",85,"OID30341","P51688","SGSH",0,"Cardiometabolic_II","B13805","20212097_SS211647","PASS",-2.1094,-0.3568,"Plate control","PASS","PFC",0.780894740101556,"Baseline","Baseline"
7883,"PFC_16",86,"OID30341","P51688","SGSH",0,"Cardiometabolic_II","B13805","20212097_SS211647","PASS",-2.1094,-0.9351,"Plate control","PASS","PFC",0.52300621661139,"1h","1h"
7884,"PFC_24",87,"OID30341","P51688","SGSH",0,"Cardiometabolic_II","B13805","20212097_SS211647","PASS",-2.1094,-0.1493,"Plate control","PASS","PFC",0.901687858167069,"24h","24h"
7885,"PFC_01",1,"OID30342","Q969X0","RILPL2",0,"Cardiometabolic_II","B13805","20212097_SS211647","PASS",-2.516,2.699,"Plate control","PASS","PFC",6.49351664781823,"Baseline","Baseline"
7886,"PFC_09",2,"OID30342","Q969X0","RILPL2",0,"Cardiometabolic_II","B13805","20212097_SS211647","PASS",-2.516,3.8553,"Plate control","PASS","PFC",14.473079320323,"Baseline","Baseline"
7887,"PFC_17",3,"OID30342","Q969X0","RILPL2",0,"Cardiometabolic_II","B13805","20212097_SS211647","PASS",-2.516,4.6861,"Plate control","PASS","PFC",25.7428521668415,"1h","1h"
7888,"PFC_25",4,"OID30342","Q969X0","RILPL2",0,"Cardiometabolic_II","B13805","20212097_SS211647","PASS",-2.516,4.0741,"Plate control","PASS","PFC",16.8432659211184,"24h","24h"
7889,"PFC_02",13,"OID30342","Q969X0","RILPL2",0,"Cardiometabolic_II","B13805","20212097_SS211647","PASS",-2.516,3.1041,"Plate control","PASS","PFC",8.59858937098633,"Baseline","Baseline"
7890,"PFC_10",14,"OID30342","Q969X0","RILPL2",0,"Cardiometabolic_II","B13805","20212097_SS211647","PASS",-2.516,3.3341,"Plate control","PASS","PFC",10.0847261283865,"1h","1h"
7891,"PFC_18",15,"OID30342","Q969X0","RILPL2",0,"Cardiometabolic_II","B13805","20212097_SS211647","PASS",-2.516,4.9798,"Plate control","PASS","PFC",31.5550717787518,"1h","1h"
7892,"PFC_26",16,"OID30342","Q969X0","RILPL2",0,"Cardiometabolic_II","B13805","20212097_SS211647","PASS",-2.516,4.423,"Plate control","PASS","PFC",21.4514014652854,"24h","24h"
7893,"PFC_03",25,"OID30342","Q969X0","RILPL2",0,"Cardiometabolic_II","B13805","20212097_SS211647","PASS",-2.516,2.009,"Plate control","PASS","PFC",4.02503129399113,"Baseline","Baseline"
7894,"PFC_11",26,"OID30342","Q969X0","RILPL2",0,"Cardiometabolic_II","B13805","20212097_SS211647","PASS",-2.516,4.6199,"Plate control","PASS","PFC",24.5882985105605,"1h","1h"
7895,"PFC_19",27,"OID30342","Q969X0","RILPL2",0,"Cardiometabolic_II","B13805","20212097_SS211647","PASS",-2.516,4.1334,"Plate control","PASS","PFC",17.5500106390695,"24h","24h"
7896,"PFC_27",28,"OID30342","Q969X0","RILPL2",0,"Cardiometabolic_II","B13805","20212097_SS211647","PASS",-2.516,5.0972,"Plate control","PASS","PFC",34.230251803928,"24h","24h"
7897,"PFC_04",37,"OID30342","Q969X0","RILPL2",0,"Cardiometabolic_II","B13805","20212097_SS211647","PASS",-2.516,4.275,"Plate control","PASS","PFC",19.3599054275087,"Baseline","Baseline"
7898,"PFC_12",38,"OID30342","Q969X0","RILPL2",0,"Cardiometabolic_II","B13805","20212097_SS211647","PASS",-2.516,2.6827,"Plate control","PASS","PFC",6.42056385218341,"1h","1h"
7899,"PFC_20",39,"OID30342","Q969X0","RILPL2",0,"Cardiometabolic_II","B13805","20212097_SS211647","PASS",-2.516,5.1884,"Plate control","PASS","PFC",36.4639766307364,"24h","24h"
7900,"PFC_05",49,"OID30342","Q969X0","RILPL2",0,"Cardiometabolic_II","B13805","20212097_SS211647","PASS",-2.516,2.8672,"Plate control","PASS","PFC",7.29647676050802,"Baseline","Baseline"
7901,"PFC_13",50,"OID30342","Q969X0","RILPL2",0,"Cardiometabolic_II","B13805","20212097_SS211647","PASS",-2.516,4.4959,"Plate control","PASS","PFC",22.5632033514674,"1h","1h"
7902,"PFC_21",51,"OID30342","Q969X0","RILPL2",0,"Cardiometabolic_II","B13805","20212097_SS211647","PASS",-2.516,3.3966,"Plate control","PASS","PFC",10.5312150988348,"24h","24h"
7903,"PFC_06",61,"OID30342","Q969X0","RILPL2",0,"Cardiometabolic_II","B13805","20212097_SS211647","PASS",-2.516,4.3842,"Plate control","PASS","PFC",20.8821738463596,"Baseline","Baseline"
7904,"PFC_14",62,"OID30342","Q969X0","RILPL2",0,"Cardiometabolic_II","B13805","20212097_SS211647","PASS",-2.516,4.3174,"Plate control","PASS","PFC",19.9373256833006,"1h","1h"
7905,"PFC_22",63,"OID30342","Q969X0","RILPL2",0,"Cardiometabolic_II","B13805","20212097_SS211647","PASS",-2.516,4.1403,"Plate control","PASS","PFC",17.6341483908667,"24h","24h"
7906,"PFC_07",73,"OID30342","Q969X0","RILPL2",0,"Cardiometabolic_II","B13805","20212097_SS211647","PASS",-2.516,4.5782,"Plate control","PASS","PFC",23.8877654506773,"Baseline","Baseline"
7907,"PFC_15",74,"OID30342","Q969X0","RILPL2",0,"Cardiometabolic_II","B13805","20212097_SS211647","PASS",-2.516,3.4859,"Plate control","PASS","PFC",11.203673960681,"1h","1h"
7908,"PFC_23",75,"OID30342","Q969X0","RILPL2",0,"Cardiometabolic_II","B13805","20212097_SS211647","PASS",-2.516,4.2151,"Plate control","PASS","PFC",18.5725499365868,"24h","24h"
7909,"PFC_08",85,"OID30342","Q969X0","RILPL2",0,"Cardiometabolic_II","B13805","20212097_SS211647","PASS",-2.516,3.6845,"Plate control","PASS","PFC",12.8571591278439,"Baseline","Baseline"
7910,"PFC_16",86,"OID30342","Q969X0","RILPL2",0,"Cardiometabolic_II","B13805","20212097_SS211647","PASS",-2.516,4.2529,"Plate control","PASS","PFC",19.0655996207208,"1h","1h"
7911,"PFC_24",87,"OID30342","Q969X0","RILPL2",0,"Cardiometabolic_II","B13805","20212097_SS211647","PASS",-2.516,4.8431,"Plate control","PASS","PFC",28.7024105397294,"24h","24h"
7912,"PFC_01",1,"OID30343","P23471","PTPRZ1",0,"Cardiometabolic_II","B13805","20212097_SS211647","PASS",-5.1386,0.4329,"Plate control","PASS","PFC",1.34994441086352,"Baseline","Baseline"
7913,"PFC_09",2,"OID30343","P23471","PTPRZ1",0,"Cardiometabolic_II","B13805","20212097_SS211647","PASS",-5.1386,0.267,"Plate control","PASS","PFC",1.2033030259995,"Baseline","Baseline"
7914,"PFC_17",3,"OID30343","P23471","PTPRZ1",0,"Cardiometabolic_II","B13805","20212097_SS211647","PASS",-5.1386,0.0085,"Plate control","PASS","PFC",1.00590914153661,"1h","1h"
7915,"PFC_25",4,"OID30343","P23471","PTPRZ1",0,"Cardiometabolic_II","B13805","20212097_SS211647","PASS",-5.1386,0.2122,"Plate control","PASS","PFC",1.15845339080384,"24h","24h"
7916,"PFC_02",13,"OID30343","P23471","PTPRZ1",0,"Cardiometabolic_II","B13805","20212097_SS211647","PASS",-5.1386,0.089,"Plate control","PASS","PFC",1.06363267289318,"Baseline","Baseline"
7917,"PFC_10",14,"OID30343","P23471","PTPRZ1",0,"Cardiometabolic_II","B13805","20212097_SS211647","PASS",-5.1386,0.4277,"Plate control","PASS","PFC",1.34508747636095,"1h","1h"
7918,"PFC_18",15,"OID30343","P23471","PTPRZ1",0,"Cardiometabolic_II","B13805","20212097_SS211647","PASS",-5.1386,0.017,"Plate control","PASS","PFC",1.01185320102693,"1h","1h"
7919,"PFC_26",16,"OID30343","P23471","PTPRZ1",0,"Cardiometabolic_II","B13805","20212097_SS211647","PASS",-5.1386,0.0573,"Plate control","PASS","PFC",1.04051661337424,"24h","24h"
7920,"PFC_03",25,"OID30343","P23471","PTPRZ1",0,"Cardiometabolic_II","B13805","20212097_SS211647","PASS",-5.1386,-0.2622,"Plate control","PASS","PFC",0.833815444447526,"Baseline","Baseline"
7921,"PFC_11",26,"OID30343","P23471","PTPRZ1",0,"Cardiometabolic_II","B13805","20212097_SS211647","PASS",-5.1386,0.159,"Plate control","PASS","PFC",1.11651296198257,"1h","1h"
7922,"PFC_19",27,"OID30343","P23471","PTPRZ1",0,"Cardiometabolic_II","B13805","20212097_SS211647","PASS",-5.1386,0.6083,"Plate control","PASS","PFC",1.52446180030223,"24h","24h"
7923,"PFC_27",28,"OID30343","P23471","PTPRZ1",0,"Cardiometabolic_II","B13805","20212097_SS211647","PASS",-5.1386,0.5017,"Plate control","PASS","PFC",1.41588098342703,"24h","24h"
7924,"PFC_04",37,"OID30343","P23471","PTPRZ1",0,"Cardiometabolic_II","B13805","20212097_SS211647","PASS",-5.1386,0.7129,"Plate control","PASS","PFC",1.63909559788817,"Baseline","Baseline"
7925,"PFC_12",38,"OID30343","P23471","PTPRZ1",0,"Cardiometabolic_II","B13805","20212097_SS211647","PASS",-5.1386,-0.5505,"Plate control","PASS","PFC",0.682783452654219,"1h","1h"
7926,"PFC_20",39,"OID30343","P23471","PTPRZ1",0,"Cardiometabolic_II","B13805","20212097_SS211647","PASS",-5.1386,0.0882,"Plate control","PASS","PFC",1.06304303320044,"24h","24h"
7927,"PFC_05",49,"OID30343","P23471","PTPRZ1",0,"Cardiometabolic_II","B13805","20212097_SS211647","PASS",-5.1386,0.3533,"Plate control","PASS","PFC",1.27747937594601,"Baseline","Baseline"
7928,"PFC_13",50,"OID30343","P23471","PTPRZ1",0,"Cardiometabolic_II","B13805","20212097_SS211647","PASS",-5.1386,0.5003,"Plate control","PASS","PFC",1.4145076703941,"1h","1h"
7929,"PFC_21",51,"OID30343","P23471","PTPRZ1",0,"Cardiometabolic_II","B13805","20212097_SS211647","PASS",-5.1386,-0.3566,"Plate control","PASS","PFC",0.781003002603051,"24h","24h"
7930,"PFC_06",61,"OID30343","P23471","PTPRZ1",0,"Cardiometabolic_II","B13805","20212097_SS211647","PASS",-5.1386,-0.2577,"Plate control","PASS","PFC",0.836420310545592,"Baseline","Baseline"
7931,"PFC_14",62,"OID30343","P23471","PTPRZ1",0,"Cardiometabolic_II","B13805","20212097_SS211647","PASS",-5.1386,0.2821,"Plate control","PASS","PFC",1.21596356443373,"1h","1h"
7932,"PFC_22",63,"OID30343","P23471","PTPRZ1",0,"Cardiometabolic_II","B13805","20212097_SS211647","PASS",-5.1386,0.4893,"Plate control","PASS","PFC",1.40376360012575,"24h","24h"
7933,"PFC_07",73,"OID30343","P23471","PTPRZ1",0,"Cardiometabolic_II","B13805","20212097_SS211647","PASS",-5.1386,0.6338,"Plate control","PASS","PFC",1.55164658974738,"Baseline","Baseline"
7934,"PFC_15",74,"OID30343","P23471","PTPRZ1",0,"Cardiometabolic_II","B13805","20212097_SS211647","PASS",-5.1386,-0.1812,"Plate control","PASS","PFC",0.881969089844297,"1h","1h"
7935,"PFC_23",75,"OID30343","P23471","PTPRZ1",0,"Cardiometabolic_II","B13805","20212097_SS211647","PASS",-5.1386,0.5101,"Plate control","PASS","PFC",1.4241489066559,"24h","24h"
7936,"PFC_08",85,"OID30343","P23471","PTPRZ1",0,"Cardiometabolic_II","B13805","20212097_SS211647","PASS",-5.1386,-0.0542,"Plate control","PASS","PFC",0.963128366822566,"Baseline","Baseline"
7937,"PFC_16",86,"OID30343","P23471","PTPRZ1",0,"Cardiometabolic_II","B13805","20212097_SS211647","PASS",-5.1386,0.0745,"Plate control","PASS","PFC",1.05299603209275,"1h","1h"
7938,"PFC_24",87,"OID30343","P23471","PTPRZ1",0,"Cardiometabolic_II","B13805","20212097_SS211647","PASS",-5.1386,-0.3393,"Plate control","PASS","PFC",0.790424735310454,"24h","24h"
7939,"PFC_01",1,"OID30344","P52209","PGD",0,"Cardiometabolic_II","B13805","20212097_SS211647","PASS",-2.2823,-0.3682,"Plate control","PASS","PFC",0.774748520563688,"Baseline","Baseline"
7940,"PFC_09",2,"OID30344","P52209","PGD",0,"Cardiometabolic_II","B13805","20212097_SS211647","PASS",-2.2823,0.3071,"Plate control","PASS","PFC",1.23721823314812,"Baseline","Baseline"
7941,"PFC_17",3,"OID30344","P52209","PGD",0,"Cardiometabolic_II","B13805","20212097_SS211647","PASS",-2.2823,0.7243,"Plate control","PASS","PFC",1.65209883845046,"1h","1h"
7942,"PFC_25",4,"OID30344","P52209","PGD",0,"Cardiometabolic_II","B13805","20212097_SS211647","PASS",-2.2823,0.774,"Plate control","PASS","PFC",1.71000435578395,"24h","24h"
7943,"PFC_02",13,"OID30344","P52209","PGD",0,"Cardiometabolic_II","B13805","20212097_SS211647","PASS",-2.2823,1.0268,"Plate control","PASS","PFC",2.03749991618962,"Baseline","Baseline"
7944,"PFC_10",14,"OID30344","P52209","PGD",0,"Cardiometabolic_II","B13805","20212097_SS211647","PASS",-2.2823,0.3945,"Plate control","PASS","PFC",1.31448711473356,"1h","1h"
7945,"PFC_18",15,"OID30344","P52209","PGD",0,"Cardiometabolic_II","B13805","20212097_SS211647","PASS",-2.2823,0.6869,"Plate control","PASS","PFC",1.60982068710789,"1h","1h"
7946,"PFC_26",16,"OID30344","P52209","PGD",0,"Cardiometabolic_II","B13805","20212097_SS211647","PASS",-2.2823,1.0133,"Plate control","PASS","PFC",2.01852296410158,"24h","24h"
7947,"PFC_03",25,"OID30344","P52209","PGD",0,"Cardiometabolic_II","B13805","20212097_SS211647","PASS",-2.2823,-0.4736,"Plate control","PASS","PFC",0.7201653037994,"Baseline","Baseline"
7948,"PFC_11",26,"OID30344","P52209","PGD",0,"Cardiometabolic_II","B13805","20212097_SS211647","PASS",-2.2823,2.0873,"Plate control","PASS","PFC",4.24952031701953,"1h","1h"
7949,"PFC_19",27,"OID30344","P52209","PGD",0,"Cardiometabolic_II","B13805","20212097_SS211647","PASS",-2.2823,0.2784,"Plate control","PASS","PFC",1.21284904560725,"24h","24h"
7950,"PFC_27",28,"OID30344","P52209","PGD",0,"Cardiometabolic_II","B13805","20212097_SS211647","PASS",-2.2823,1.1469,"Plate control","PASS","PFC",2.21437567482336,"24h","24h"
7951,"PFC_04",37,"OID30344","P52209","PGD",0,"Cardiometabolic_II","B13805","20212097_SS211647","PASS",-2.2823,0.5836,"Plate control","PASS","PFC",1.49858404842709,"Baseline","Baseline"
7952,"PFC_12",38,"OID30344","P52209","PGD",0,"Cardiometabolic_II","B13805","20212097_SS211647","PASS",-2.2823,-0.0084,"Plate control","PASS","PFC",0.99419448121595,"1h","1h"

Supplemental table 1_Olink proximity extension assay (PEA) data

,"SampleID","Index","OlinkID","UniProt","Assay","MissingFreq","Panel","Panel_Lot_Nr","PlateID","QC_Warning","LOD","NPX","Normalization","Assay_Warning","condition","Value","group","alt_group"
7953,"PFC_20",39,"OID30344","P52209","PGD",0,"Cardiometabolic_II","B13805","20212097_SS211647","PASS",-2.2823,2.1428,"Plate control","PASS","PFC",4.41618313323751,"24h","24h"
7954,"PFC_05",49,"OID30344","P52209","PGD",0,"Cardiometabolic_II","B13805","20212097_SS211647","PASS",-2.2823,-0.1225,"Plate control","PASS","PFC",0.918594467722301,"Baseline","Baseline"
7955,"PFC_13",50,"OID30344","P52209","PGD",0,"Cardiometabolic_II","B13805","20212097_SS211647","PASS",-2.2823,0.794,"Plate control","PASS","PFC",1.73387512702937,"1h","1h"
7956,"PFC_21",51,"OID30344","P52209","PGD",0,"Cardiometabolic_II","B13805","20212097_SS211647","PASS",-2.2823,0.2422,"Plate control","PASS","PFC",1.18279495907733,"24h","24h"
7957,"PFC_06",61,"OID30344","P52209","PGD",0,"Cardiometabolic_II","B13805","20212097_SS211647","PASS",-2.2823,0.4298,"Plate control","PASS","PFC",1.34704682357967,"Baseline","Baseline"
7958,"PFC_14",62,"OID30344","P52209","PGD",0,"Cardiometabolic_II","B13805","20212097_SS211647","PASS",-2.2823,0.8152,"Plate control","PASS","PFC",1.7595420592616,"1h","1h"
7959,"PFC_22",63,"OID30344","P52209","PGD",0,"Cardiometabolic_II","B13805","20212097_SS211647","PASS",-2.2823,0.5969,"Plate control","PASS","PFC",1.51246315769319,"24h","24h"
7960,"PFC_07",73,"OID30344","P52209","PGD",0,"Cardiometabolic_II","B13805","20212097_SS211647","PASS",-2.2823,0.9324,"Plate control","PASS","PFC",1.9084481619334,"Baseline","Baseline"
7961,"PFC_15",74,"OID30344","P52209","PGD",0,"Cardiometabolic_II","B13805","20212097_SS211647","PASS",-2.2823,-0.1375,"Plate control","PASS","PFC",0.909093129527559,"1h","1h"
7962,"PFC_23",75,"OID30344","P52209","PGD",0,"Cardiometabolic_II","B13805","20212097_SS211647","PASS",-2.2823,1.4977,"Plate control","PASS","PFC",2.82392152973707,"24h","24h"
7963,"PFC_08",85,"OID30344","P52209","PGD",0,"Cardiometabolic_II","B13805","20212097_SS211647","PASS",-2.2823,0.0328,"Plate control","PASS","PFC",1.02299564259487,"Baseline","Baseline"
7964,"PFC_16",86,"OID30344","P52209","PGD",0,"Cardiometabolic_II","B13805","20212097_SS211647","PASS",-2.2823,1.1561,"Plate control","PASS","PFC",2.2285417670295,"1h","1h"
7965,"PFC_24",87,"OID30344","P52209","PGD",0,"Cardiometabolic_II","B13805","20212097_SS211647","PASS",-2.2823,0.9178,"Plate control","PASS","PFC",1.88923216065071,"24h","24h"
7966,"PFC_01",1,"OID30345","P32320","CDA",0,"Cardiometabolic_II","B13805","20212097_SS211647","PASS",-4.4526,0.6231,"Plate control","PASS","PFC",1.54018110199114,"Baseline","Baseline"
7967,"PFC_09",2,"OID30345","P32320","CDA",0,"Cardiometabolic_II","B13805","20212097_SS211647","PASS",-4.4526,0.5505,"Plate control","PASS","PFC",1.46459319731995,"Baseline","Baseline"
7968,"PFC_17",3,"OID30345","P32320","CDA",0,"Cardiometabolic_II","B13805","20212097_SS211647","PASS",-4.4526,0.0561,"Plate control","PASS","PFC",1.03965149582813,"1h","1h"
7969,"PFC_25",4,"OID30345","P32320","CDA",0,"Cardiometabolic_II","B13805","20212097_SS211647","PASS",-4.4526,-0.5868,"Plate control","PASS","PFC",0.665818102208657,"24h","24h"
7970,"PFC_02",13,"OID30345","P32320","CDA",0,"Cardiometabolic_II","B13805","20212097_SS211647","PASS",-4.4526,0.771,"Plate control","PASS","PFC",1.70645219622413,"Baseline","Baseline"
7971,"PFC_10",14,"OID30345","P32320","CDA",0,"Cardiometabolic_II","B13805","20212097_SS211647","PASS",-4.4526,0.6845,"Plate control","PASS","PFC",1.60714489098048,"1h","1h"
7972,"PFC_18",15,"OID30345","P32320","CDA",0,"Cardiometabolic_II","B13805","20212097_SS211647","PASS",-4.4526,0.8406,"Plate control","PASS","PFC",1.79079475760904,"1h","1h"
7973,"PFC_26",16,"OID30345","P32320","CDA",0,"Cardiometabolic_II","B13805","20212097_SS211647","PASS",-4.4526,-0.6237,"Plate control","PASS","PFC",0.649004326094481,"24h","24h"
7974,"PFC_03",25,"OID30345","P32320","CDA",0,"Cardiometabolic_II","B13805","20212097_SS211647","PASS",-4.4526,0.4368,"Plate control","PASS","PFC",1.35359861743199,"Baseline","Baseline"
7975,"PFC_11",26,"OID30345","P32320","CDA",0,"Cardiometabolic_II","B13805","20212097_SS211647","PASS",-4.4526,0.7871,"Plate control","PASS","PFC",1.72560229457658,"1h","1h"
7976,"PFC_19",27,"OID30345","P32320","CDA",0,"Cardiometabolic_II","B13805","20212097_SS211647","PASS",-4.4526,0.8223,"Plate control","PASS","PFC",1.76822271547729,"24h","24h"
7977,"PFC_27",28,"OID30345","P32320","CDA",0,"Cardiometabolic_II","B13805","20212097_SS211647","PASS",-4.4526,0.8887,"Plate control","PASS","PFC",1.85150699496557,"24h","24h"
7978,"PFC_04",37,"OID30345","P32320","CDA",0,"Cardiometabolic_II","B13805","20212097_SS211647","PASS",-4.4526,-0.0397,"Plate control","PASS","PFC",0.972857226353255,"Baseline","Baseline"
7979,"PFC_12",38,"OID30345","P32320","CDA",0,"Cardiometabolic_II","B13805","20212097_SS211647","PASS",-4.4526,0.634,"Plate control","PASS","PFC",1.55186170854971,"1h","1h"
7980,"PFC_20",39,"OID30345","P32320","CDA",0,"Cardiometabolic_II","B13805","20212097_SS211647","PASS",-4.4526,0.637,"Plate control","PASS","PFC",1.55509207176512,"24h","24h"
7981,"PFC_05",49,"OID30345","P32320","CDA",0,"Cardiometabolic_II","B13805","20212097_SS211647","PASS",-4.4526,1.1972,"Plate control","PASS","PFC",2.29294221871157,"Baseline","Baseline"
7982,"PFC_13",50,"OID30345","P32320","CDA",0,"Cardiometabolic_II","B13805","20212097_SS211647","PASS",-4.4526,0.3618,"Plate control","PASS","PFC",1.28502818238858,"1h","1h"
7983,"PFC_21",51,"OID30345","P32320","CDA",0,"Cardiometabolic_II","B13805","20212097_SS211647","PASS",-4.4526,0.3448,"Plate control","PASS","PFC",1.2699749144287,"24h","24h"
7984,"PFC_06",61,"OID30345","P32320","CDA",0,"Cardiometabolic_II","B13805","20212097_SS211647","PASS",-4.4526,0.215,"Plate control","PASS","PFC",1.16070391438372,"Baseline","Baseline"
7985,"PFC_14",62,"OID30345","P32320","CDA",0,"Cardiometabolic_II","B13805","20212097_SS211647","PASS",-4.4526,0.9997,"Plate control","PASS","PFC",1.99958415492944,"1h","1h"
7986,"PFC_22",63,"OID30345","P32320","CDA",0,"Cardiometabolic_II","B13805","20212097_SS211647","PASS",-4.4526,-0.271,"Plate control","PASS","PFC",0.828744904487992,"24h","24h"
7987,"PFC_07",73,"OID30345","P32320","CDA",0,"Cardiometabolic_II","B13805","20212097_SS211647","PASS",-4.4526,0.0532,"Plate control","PASS","PFC",1.03756376347552,"Baseline","Baseline"
7988,"PFC_15",74,"OID30345","P32320","CDA",0,"Cardiometabolic_II","B13805","20212097_SS211647","PASS",-4.4526,0.5229,"Plate control","PASS","PFC",1.43684057889269,"1h","1h"
7989,"PFC_23",75,"OID30345","P32320","CDA",0,"Cardiometabolic_II","B13805","20212097_SS211647","PASS",-4.4526,0.9948,"Plate control","PASS","PFC",1.99280424517708,"24h","24h"
7990,"PFC_08",85,"OID30345","P32320","CDA",0,"Cardiometabolic_II","B13805","20212097_SS211647","PASS",-4.4526,-0.5222,"Plate control","PASS","PFC",0.696309204690925,"Baseline","Baseline"
7991,"PFC_16",86,"OID30345","P32320","CDA",0,"Cardiometabolic_II","B13805","20212097_SS211647","PASS",-4.4526,-0.2177,"Plate control","PASS","PFC",0.859935286167884,"1h","1h"
7992,"PFC_24",87,"OID30345","P32320","CDA",0,"Cardiometabolic_II","B13805","20212097_SS211647","PASS",-4.4526,0.3116,"Plate control","PASS","PFC",1.24108334245126,"24h","24h"
7993,"PFC_01",1,"OID30346","Q6PI73","LILRA6",0,"Cardiometabolic_II","B13805","20212097_SS211647","PASS",-4.5708,1.1792,"Plate control","PASS","PFC",2.26451171044401,"Baseline","Baseline"
7994,"PFC_09",2,"OID30346","Q6PI73","LILRA6",0,"Cardiometabolic_II","B13805","20212097_SS211647","PASS",-4.5708,0.1534,"Plate control","PASS","PFC",1.11218747861877,"Baseline","Baseline"
7995,"PFC_17",3,"OID30346","Q6PI73","LILRA6",0,"Cardiometabolic_II","B13805","20212097_SS211647","PASS",-4.5708,-1.4812,"Plate control","PASS","PFC",0.358190753369079,"1h","1h"
7996,"PFC_25",4,"OID30346","Q6PI73","LILRA6",0,"Cardiometabolic_II","B13805","20212097_SS211647","PASS",-4.5708,0.5427,"Plate control","PASS","PFC",1.45669617094835,"24h","24h"
7997,"PFC_02",13,"OID30346","Q6PI73","LILRA6",0,"Cardiometabolic_II","B13805","20212097_SS211647","PASS",-4.5708,4e-04,"Plate control","PASS","PFC",1.00027729731202,"Baseline","Baseline"
7998,"PFC_10",14,"OID30346","Q6PI73","LILRA6",0,"Cardiometabolic_II","B13805","20212097_SS211647","PASS",-4.5708,1.1302,"Plate control","PASS","PFC",2.18889082619021,"1h","1h"
7999,"PFC_18",15,"OID30346","Q6PI73","LILRA6",0,"Cardiometabolic_II","B13805","20212097_SS211647","PASS",-4.5708,-0.0585,"Plate control","PASS","PFC",0.960262004826095,"1h","1h"
8000,"PFC_26",16,"OID30346","Q6PI73","LILRA6",0,"Cardiometabolic_II","B13805","20212097_SS211647","PASS",-4.5708,-1.7489,"Plate control","PASS","PFC",0.297528546469255,"24h","24h"
8001,"PFC_03",25,"OID30346","Q6PI73","LILRA6",0,"Cardiometabolic_II","B13805","20212097_SS211647","PASS",-4.5708,-0.1921,"Plate control","PASS","PFC",0.875330654293931,"Baseline","Baseline"
8002,"PFC_11",26,"OID30346","Q6PI73","LILRA6",0,"Cardiometabolic_II","B13805","20212097_SS211647","PASS",-4.5708,0.0643,"Plate control","PASS","PFC",1.04557749932966,"1h","1h"
8003,"PFC_19",27,"OID30346","Q6PI73","LILRA6",0,"Cardiometabolic_II","B13805","20212097_SS211647","PASS",-4.5708,1.2678,"Plate control","PASS","PFC",2.4079409278307,"24h","24h"
8004,"PFC_27",28,"OID30346","Q6PI73","LILRA6",0,"Cardiometabolic_II","B13805","20212097_SS211647","PASS",-4.5708,0.3578,"Plate control","PASS","PFC",1.28147026234604,"24h","24h"
8005,"PFC_04",37,"OID30346","Q6PI73","LILRA6",0,"Cardiometabolic_II","B13805","20212097_SS211647","PASS",-4.5708,-0.2455,"Plate control","PASS","PFC",0.843523402530895,"Baseline","Baseline"
8006,"PFC_12",38,"OID30346","Q6PI73","LILRA6",0,"Cardiometabolic_II","B13805","20212097_SS211647","PASS",-4.5708,-0.3485,"Plate control","PASS","PFC",0.785400270502085,"1h","1h"
8007,"PFC_20",39,"OID30346","Q6PI73","LILRA6",0,"Cardiometabolic_II","B13805","20212097_SS211647","PASS",-4.5708,0.019,"Plate control","PASS","PFC",1.01325690015909,"24h","24h"
8008,"PFC_05",49,"OID30346","Q6PI73","LILRA6",0,"Cardiometabolic_II","B13805","20212097_SS211647","PASS",-4.5708,-0.489,"Plate control","PASS","PFC",0.712518807074392,"Baseline","Baseline"
8009,"PFC_13",50,"OID30346","Q6PI73","LILRA6",0,"Cardiometabolic_II","B13805","20212097_SS211647","PASS",-4.5708,-0.418,"Plate control","PASS","PFC",0.748461493396346,"1h","1h"
8010,"PFC_21",51,"OID30346","Q6PI73","LILRA6",0,"Cardiometabolic_II","B13805","20212097_SS211647","PASS",-4.5708,-0.2049,"Plate control","PASS","PFC",0.867598822435677,"24h","24h"
8011,"PFC_06",61,"OID30346","Q6PI73","LILRA6",0,"Cardiometabolic_II","B13805","20212097_SS211647","PASS",-4.5708,-0.4623,"Plate control","PASS","PFC",0.725828192528607,"Baseline","Baseline"
8012,"PFC_14",62,"OID30346","Q6PI73","LILRA6",0,"Cardiometabolic_II","B13805","20212097_SS211647","PASS",-4.5708,-0.5156,"Plate control","PASS","PFC",0.699501957609375,"1h","1h"
8013,"PFC_22",63,"OID30346","Q6PI73","LILRA6",0,"Cardiometabolic_II","B13805","20212097_SS211647","PASS",-4.5708,-0.2957,"Plate control","PASS","PFC",0.81467695276767,"24h","24h"
8014,"PFC_07",73,"OID30346","Q6PI73","LILRA6",0,"Cardiometabolic_II","B13805","20212097_SS211647","PASS",-4.5708,0.6748,"Plate control","PASS","PFC",1.59637545274409,"Baseline","Baseline"
8015,"PFC_15",74,"OID30346","Q6PI73","LILRA6",0,"Cardiometabolic_II","B13805","20212097_SS211647","PASS",-4.5708,-0.2194,"Plate control","PASS","PFC",0.858922578025067,"1h","1h"
8016,"PFC_23",75,"OID30346","Q6PI73","LILRA6",0,"Cardiometabolic_II","B13805","20212097_SS211647","PASS",-4.5708,-0.5588,"Plate control","PASS","PFC",0.678866594310199,"24h","24h"
8017,"PFC_08",85,"OID30346","Q6PI73","LILRA6",0,"Cardiometabolic_II","B13805","20212097_SS211647","PASS",-4.5708,-1.5451,"Plate control","PASS","PFC",0.342671948212242,"Baseline","Baseline"
8018,"PFC_16",86,"OID30346","Q6PI73","LILRA6",0,"Cardiometabolic_II","B13805","20212097_SS211647","PASS",-4.5708,0.5001,"Plate control","PASS","PFC",1.41431159158484,"1h","1h"
8019,"PFC_24",87,"OID30346","Q6PI73","LILRA6",0,"Cardiometabolic_II","B13805","20212097_SS211647","PASS",-4.5708,-0.2356,"Plate control","PASS","PFC",0.849331698553624,"24h","24h"
8020,"PFC_01",1,"OID30347","P08582","MELTF",0,"Cardiometabolic_II","B13805","20212097_SS211647","PASS",-3.2054,0.3586,"Plate control","PASS","PFC",1.28218105740151,"Baseline","Baseline"
8021,"PFC_09",2,"OID30347","P08582","MELTF",0,"Cardiometabolic_II","B13805","20212097_SS211647","PASS",-3.2054,0.0967,"Plate control","PASS","PFC",1.06932470494313,"Baseline","Baseline"
8022,"PFC_17",3,"OID30347","P08582","MELTF",0,"Cardiometabolic_II","B13805","20212097_SS211647","PASS",-3.2054,-0.0126,"Plate control","PASS","PFC",0.991304373098102,"1h","1h"
8023,"PFC_25",4,"OID30347","P08582","MELTF",0,"Cardiometabolic_II","B13805","20212097_SS211647","PASS",-3.2054,0.0669,"Plate control","PASS","PFC",1.0474635199449,"24h","24h"
8024,"PFC_02",13,"OID30347","P08582","MELTF",0,"Cardiometabolic_II","B13805","20212097_SS211647","PASS",-3.2054,0.6066,"Plate control","PASS","PFC",1.52266650837339,"Baseline","Baseline"
8025,"PFC_10",14,"OID30347","P08582","MELTF",0,"Cardiometabolic_II","B13805","20212097_SS211647","PASS",-3.2054,0.1581,"Plate control","PASS","PFC",1.11581666216188,"1h","1h"
8026,"PFC_18",15,"OID30347","P08582","MELTF",0,"Cardiometabolic_II","B13805","20212097_SS211647","PASS",-3.2054,-0.1958,"Plate control","PASS","PFC",0.873088618527452,"1h","1h"
8027,"PFC_26",16,"OID30347","P08582","MELTF",0,"Cardiometabolic_II","B13805","20212097_SS211647","PASS",-3.2054,0.2155,"Plate control","PASS","PFC",1.1611062534226,"24h","24h"
8028,"PFC_03",25,"OID30347","P08582","MELTF",0,"Cardiometabolic_II","B13805","20212097_SS211647","PASS",-3.2054,0.5479,"Plate control","PASS","PFC",1.46195610981238,"Baseline","Baseline"
8029,"PFC_11",26,"OID30347","P08582","MELTF",0,"Cardiometabolic_II","B13805","20212097_SS211647","PASS",-3.2054,0.6848,"Plate control","PASS","PFC",1.60747912211496,"1h","1h"
8030,"PFC_19",27,"OID30347","P08582","MELTF",0,"Cardiometabolic_II","B13805","20212097_SS211647","PASS",-3.2054,0.3138,"Plate control","PASS","PFC",1.24297734371128,"24h","24h"
8031,"PFC_27",28,"OID30347","P08582","MELTF",0,"Cardiometabolic_II","B13805","20212097_SS211647","PASS",-3.2054,0.3535,"Plate control","PASS","PFC",1.27765648446748,"24h","24h"
8032,"PFC_04",37,"OID30347","P08582","MELTF",0,"Cardiometabolic_II","B13805","20212097_SS211647","PASS",-3.2054,0.3294,"Plate control","PASS","PFC",1.25649070604933,"Baseline","Baseline"
8033,"PFC_12",38,"OID30347","P08582","MELTF",0,"Cardiometabolic_II","B13805","20212097_SS211647","PASS",-3.2054,0.1109,"Plate control","PASS","PFC",1.07990170415646,"1h","1h"
8034,"PFC_20",39,"OID30347","P08582","MELTF",0,"Cardiometabolic_II","B13805","20212097_SS211647","PASS",-3.2054,0.6198,"Plate control","PASS","PFC",1.5366621399155,"24h","24h"
8035,"PFC_05",49,"OID30347","P08582","MELTF",0,"Cardiometabolic_II","B13805","20212097_SS211647","PASS",-3.2054,0.3549,"Plate control","PASS","PFC",1.27889693182486,"Baseline","Baseline"
8036,"PFC_13",50,"OID30347","P08582","MELTF",0,"Cardiometabolic_II","B13805","20212097_SS211647","PASS",-3.2054,0.1879,"Plate control","PASS","PFC",1.13910441778407,"1h","1h"
8037,"PFC_21",51,"OID30347","P08582","MELTF",0,"Cardiometabolic_II","B13805","20212097_SS211647","PASS",-3.2054,0.1235,"Plate control","PASS","PFC",1.08937449835056,"24h","24h"
8038,"PFC_06",61,"OID30347","P08582","MELTF",0,"Cardiometabolic_II","B13805","20212097_SS211647","PASS",-3.2054,0.4498,"Plate control","PASS","PFC",1.36585089648965,"Baseline","Baseline"
8039,"PFC_14",62,"OID30347","P08582","MELTF",0,"Cardiometabolic_II","B13805","20212097_SS211647","PASS",-3.2054,0.2725,"Plate control","PASS","PFC",1.20789914489587,"1h","1h"
8040,"PFC_22",63,"OID30347","P08582","MELTF",0,"Cardiometabolic_II","B13805","20212097_SS211647","PASS",-3.2054,0.1803,"Plate control","PASS","PFC",1.13311948637218,"24h","24h"
8041,"PFC_07",73,"OID30347","P08582","MELTF",0,"Cardiometabolic_II","B13805","20212097_SS211647","PASS",-3.2054,0.312,"Plate control","PASS","PFC",1.24142749152611,"Baseline","Baseline"
8042,"PFC_15",74,"OID30347","P08582","MELTF",0,"Cardiometabolic_II","B13805","20212097_SS211647","PASS",-3.2054,0.6261,"Plate control","PASS","PFC",1.5433871507966,"1h","1h"
8043,"PFC_23",75,"OID30347","P08582","MELTF",0,"Cardiometabolic_II","B13805","20212097_SS211647","PASS",-3.2054,0.4731,"Plate control","PASS","PFC",1.38808893067398,"24h","24h"
8044,"PFC_08",85,"OID30347","P08582","MELTF",0,"Cardiometabolic_II","B13805","20212097_SS211647","PASS",-3.2054,0.2219,"Plate control","PASS","PFC",1.16626852741256,"Baseline","Baseline"
8045,"PFC_16",86,"OID30347","P08582","MELTF",0,"Cardiometabolic_II","B13805","20212097_SS211647","PASS",-3.2054,-0.0744,"Plate control","PASS","PFC",0.949737023351187,"1h","1h"
8046,"PFC_24",87,"OID30347","P08582","MELTF",0,"Cardiometabolic_II","B13805","20212097_SS211647","PASS",-3.2054,0.5885,"Plate control","PASS","PFC",1.50368252442102,"24h","24h"
8047,"PFC_01",1,"OID30348","Q96MK3","FAM20A",0,"Cardiometabolic_II","B13805","20212097_SS211647","PASS",-3.1715,-0.0361,"Plate control","PASS","PFC",0.97528785738183,"Baseline","Baseline"
8048,"PFC_09",2,"OID30348","Q96MK3","FAM20A",0,"Cardiometabolic_II","B13805","20212097_SS211647","PASS",-3.1715,0.4735,"Plate control","PASS","PFC",1.3884738440033,"Baseline","Baseline"
8049,"PFC_17",3,"OID30348","Q96MK3","FAM20A",0,"Cardiometabolic_II","B13805","20212097_SS211647","PASS",-3.1715,-0.0436,"Plate control","PASS","PFC",0.970230878172232,"1h","1h"
8050,"PFC_25",4,"OID30348","Q96MK3","FAM20A",0,"Cardiometabolic_II","B13805","20212097_SS211647","PASS",-3.1715,0.1649,"Plate control","PASS","PFC",1.12108836741119,"24h","24h"
8051,"PFC_02",13,"OID30348","Q96MK3","FAM20A",0,"Cardiometabolic_II","B13805","20212097_SS211647","PASS",-3.1715,0.1663,"Plate control","PASS","PFC",1.1221768063771,"Baseline","Baseline"
8052,"PFC_10",14,"OID30348","Q96MK3","FAM20A",0,"Cardiometabolic_II","B13805","20212097_SS211647","PASS",-3.1715,0.2731,"Plate control","PASS","PFC",1.20840160050335,"1h","1h"
8053,"PFC_18",15,"OID30348","Q96MK3","FAM20A",0,"Cardiometabolic_II","B13805","20212097_SS211647","PASS",-3.1715,0.766,"Plate control","PASS","PFC",1.70054832012839,"1h","1h"
8054,"PFC_26",16,"OID30348","Q96MK3","FAM20A",0,"Cardiometabolic_II","B13805","20212097_SS211647","PASS",-3.1715,0.1853,"Plate control","PASS","PFC",1.13705339226008,"24h","24h"
8055,"PFC_03",25,"OID30348","Q96MK3","FAM20A",0,"Cardiometabolic_II","B13805","20212097_SS211647","PASS",-3.1715,-0.4499,"Plate control","PASS","PFC",0.732093591075026,"Baseline","Baseline"
8056,"PFC_11",26,"OID30348","Q96MK3","FAM20A",0,"Cardiometabolic_II","B13805","20212097_SS211647","PASS",-3.1715,0.4901,"Plate control","PASS","PFC",1.40454222781251,"1h","1h"
8057,"PFC_19",27,"OID30348","Q96MK3","FAM20A",0,"Cardiometabolic_II","B13805","20212097_SS211647","PASS",-3.1715,0.5535,"Plate control","PASS","PFC",1.46764190195909,"24h","24h"
8058,"PFC_27",28,"OID30348","Q96MK3","FAM20A",0,"Cardiometabolic_II","B13805","20212097_SS211647","PASS",-3.1715,0.994,"Plate control","PASS","PFC",1.99169950618892,"24h","24h"
8059,"PFC_04",37,"OID30348","Q96MK3","FAM20A",0,"Cardiometabolic_II","B13805","20212097_SS211647","PASS",-3.1715,0.4325,"Plate control","PASS","PFC",1.34957017868059,"Baseline","Baseline"
8060,"PFC_12",38,"OID30348","Q96MK3","FAM20A",0,"Cardiometabolic_II","B13805","20212097_SS211647","PASS",-3.1715,-0.3167,"Plate control","PASS","PFC",0.802904330619964,"1h","1h"
8061,"PFC_20",39,"OID30348","Q96MK3","FAM20A",0,"Cardiometabolic_II","B13805","20212097_SS211647","PASS",-3.1715,0.7103,"Plate control","PASS","PFC",1.63614430838825,"24h","24h"
8062,"PFC_05",49,"OID30348","Q96MK3","FAM20A",0,"Cardiometabolic_II","B13805","20212097_SS211647","PASS",-3.1715,0.621,"Plate control","PASS","PFC",1.53794083127027,"Baseline","Baseline"
8063,"PFC_13",50,"OID30348","Q96MK3","FAM20A",0,"Cardiometabolic_II","B13805","20212097_SS211647","PASS",-3.1715,0.4982,"Plate control","PASS","PFC",1.41245019798755,"1h","1h"
8064,"PFC_21",51,"OID30348","Q96MK3","FAM20A",0,"Cardiometabolic_II","B13805","20212097_SS211647","PASS",-3.1715,0.0076,"Plate control","PASS","PFC",1.00528181845239,"24h","24h"

Supplemental table 1_Olink proximity extension assay (PEA) data

**,"SampleID","Index","OlinkID","UniProt","Assay","MissingFreq","Panel","Panel_Lot_Nr","PlateID","QC_Warning","LOD","NPX","Normalization","Assay_Warning","condition","Value","group","alt_group"**
8065,"PFC_06",61,"OID30348","Q96MK3","FAM20A",0,"Cardiometabolic_II","B13805","20212097_SS211647","PASS",-3.1715,0.2294,"Plate control","PASS","PFC",1.1723472823034,"Baseline","Baseline"
8066,"PFC_14",62,"OID30348","Q96MK3","FAM20A",0,"Cardiometabolic_II","B13805","20212097_SS211647","PASS",-3.1715,0.7703,"Plate control","PASS","PFC",1.70562442128983,"1h","1h"
8067,"PFC_22",63,"OID30348","Q96MK3","FAM20A",0,"Cardiometabolic_II","B13805","20212097_SS211647","PASS",-3.1715,0.8538,"Plate control","PASS","PFC",1.80725489740801,"24h","24h"
8068,"PFC_07",73,"OID30348","Q96MK3","FAM20A",0,"Cardiometabolic_II","B13805","20212097_SS211647","PASS",-3.1715,0.0909,"Plate control","PASS","PFC",1.06503437827721,"Baseline","Baseline"
8069,"PFC_15",74,"OID30348","Q96MK3","FAM20A",0,"Cardiometabolic_II","B13805","20212097_SS211647","PASS",-3.1715,0.2134,"Plate control","PASS","PFC",1.15941736609629,"1h","1h"
8070,"PFC_23",75,"OID30348","Q96MK3","FAM20A",0,"Cardiometabolic_II","B13805","20212097_SS211647","PASS",-3.1715,0.9735,"Plate control","PASS","PFC",1.96359854118976,"24h","24h"
8071,"PFC_08",85,"OID30348","Q96MK3","FAM20A",0,"Cardiometabolic_II","B13805","20212097_SS211647","PASS",-3.1715,0.0155,"Plate control","PASS","PFC",1.01080170296369,"Baseline","Baseline"
8072,"PFC_16",86,"OID30348","Q96MK3","FAM20A",0,"Cardiometabolic_II","B13805","20212097_SS211647","PASS",-3.1715,0.3252,"Plate control","PASS","PFC",1.25283810682638,"1h","1h"
8073,"PFC_24",87,"OID30348","Q96MK3","FAM20A",0,"Cardiometabolic_II","B13805","20212097_SS211647","PASS",-3.1715,0.3434,"Plate control","PASS","PFC",1.26874312085149,"24h","24h"
8074,"PFC_01",1,"OID30349","Q8IZF2","ADGRF5",0,"Cardiometabolic_II","B13805","20212097_SS211647","PASS",-4.0752,0.6278,"Plate control","PASS","PFC",1.54520687328978,"Baseline","Baseline"
8075,"PFC_09",2,"OID30349","Q8IZF2","ADGRF5",0,"Cardiometabolic_II","B13805","20212097_SS211647","PASS",-4.0752,0.6415,"Plate control","PASS","PFC",1.55995022909968,"Baseline","Baseline"
8076,"PFC_17",3,"OID30349","Q8IZF2","ADGRF5",0,"Cardiometabolic_II","B13805","20212097_SS211647","PASS",-4.0752,0.5016,"Plate control","PASS","PFC",1.4157828454371,"1h","1h"
8077,"PFC_25",4,"OID30349","Q8IZF2","ADGRF5",0,"Cardiometabolic_II","B13805","20212097_SS211647","PASS",-4.0752,0.621,"Plate control","PASS","PFC",1.53794083127027,"24h","24h"
8078,"PFC_02",13,"OID30349","Q8IZF2","ADGRF5",0,"Cardiometabolic_II","B13805","20212097_SS211647","PASS",-4.0752,0.3367,"Plate control","PASS","PFC",1.26286462922313,"Baseline","Baseline"
8079,"PFC_10",14,"OID30349","Q8IZF2","ADGRF5",0,"Cardiometabolic_II","B13805","20212097_SS211647","PASS",-4.0752,0.2419,"Plate control","PASS","PFC",1.18254902935072,"1h","1h"
8080,"PFC_18",15,"OID30349","Q8IZF2","ADGRF5",0,"Cardiometabolic_II","B13805","20212097_SS211647","PASS",-4.0752,0.6122,"Plate control","PASS","PFC",1.52858841342735,"1h","1h"
8081,"PFC_26",16,"OID30349","Q8IZF2","ADGRF5",0,"Cardiometabolic_II","B13805","20212097_SS211647","PASS",-4.0752,0.2419,"Plate control","PASS","PFC",1.18254902935072,"24h","24h"
8082,"PFC_03",25,"OID30349","Q8IZF2","ADGRF5",0,"Cardiometabolic_II","B13805","20212097_SS211647","PASS",-4.0752,-0.0379,"Plate control","PASS","PFC",0.974071783714371,"Baseline","Baseline"
8083,"PFC_11",26,"OID30349","Q8IZF2","ADGRF5",0,"Cardiometabolic_II","B13805","20212097_SS211647","PASS",-4.0752,0.4895,"Plate control","PASS","PFC",1.40395821657154,"1h","1h"
8084,"PFC_19",27,"OID30349","Q8IZF2","ADGRF5",0,"Cardiometabolic_II","B13805","20212097_SS211647","PASS",-4.0752,0.5158,"Plate control","PASS","PFC",1.42978676208956,"24h","24h"
8085,"PFC_27",28,"OID30349","Q8IZF2","ADGRF5",0,"Cardiometabolic_II","B13805","20212097_SS211647","PASS",-4.0752,1.0134,"Plate control","PASS","PFC",2.01866288230087,"24h","24h"
8086,"PFC_04",37,"OID30349","Q8IZF2","ADGRF5",0,"Cardiometabolic_II","B13805","20212097_SS211647","PASS",-4.0752,0.621,"Plate control","PASS","PFC",1.53794083127027,"Baseline","Baseline"
8087,"PFC_12",38,"OID30349","Q8IZF2","ADGRF5",0,"Cardiometabolic_II","B13805","20212097_SS211647","PASS",-4.0752,0.0098,"Plate control","PASS","PFC",1.00681596605207,"1h","1h"
8088,"PFC_20",39,"OID30349","Q8IZF2","ADGRF5",0,"Cardiometabolic_II","B13805","20212097_SS211647","PASS",-4.0752,0.5361,"Plate control","PASS","PFC",1.45004733901801,"24h","24h"
8089,"PFC_05",49,"OID30349","Q8IZF2","ADGRF5",0,"Cardiometabolic_II","B13805","20212097_SS211647","PASS",-4.0752,0.5041,"Plate control","PASS","PFC",1.41823833706302,"Baseline","Baseline"
8090,"PFC_13",50,"OID30349","Q8IZF2","ADGRF5",0,"Cardiometabolic_II","B13805","20212097_SS211647","PASS",-4.0752,0.5158,"Plate control","PASS","PFC",1.42978676208956,"1h","1h"
8091,"PFC_21",51,"OID30349","Q8IZF2","ADGRF5",0,"Cardiometabolic_II","B13805","20212097_SS211647","PASS",-4.0752,0.342,"Plate control","PASS","PFC",1.26751252203442,"24h","24h"
8092,"PFC_06",61,"OID30349","Q8IZF2","ADGRF5",0,"Cardiometabolic_II","B13805","20212097_SS211647","PASS",-4.0752,0.6272,"Plate control","PASS","PFC",1.54456437343057,"Baseline","Baseline"
8093,"PFC_14",62,"OID30349","Q8IZF2","ADGRF5",0,"Cardiometabolic_II","B13805","20212097_SS211647","PASS",-4.0752,1.0265,"Plate control","PASS","PFC",2.03707627404141,"1h","1h"
8094,"PFC_22",63,"OID30349","Q8IZF2","ADGRF5",0,"Cardiometabolic_II","B13805","20212097_SS211647","PASS",-4.0752,0.558,"Plate control","PASS","PFC",1.47222686216818,"24h","24h"
8095,"PFC_07",73,"OID30349","Q8IZF2","ADGRF5",0,"Cardiometabolic_II","B13805","20212097_SS211647","PASS",-4.0752,0.5862,"Plate control","PASS","PFC",1.50128720568785,"Baseline","Baseline"
8096,"PFC_15",74,"OID30349","Q8IZF2","ADGRF5",0,"Cardiometabolic_II","B13805","20212097_SS211647","PASS",-4.0752,0.6327,"Plate control","PASS","PFC",1.5504639692514,"1h","1h"
8097,"PFC_23",75,"OID30349","Q8IZF2","ADGRF5",0,"Cardiometabolic_II","B13805","20212097_SS211647","PASS",-4.0752,0.9792,"Plate control","PASS","PFC",1.9713719451177,"24h","24h"
8098,"PFC_08",85,"OID30349","Q8IZF2","ADGRF5",0,"Cardiometabolic_II","B13805","20212097_SS211647","PASS",-4.0752,-0.1009,"Plate control","PASS","PFC",0.93245111678315,"Baseline","Baseline"
8099,"PFC_16",86,"OID30349","Q8IZF2","ADGRF5",0,"Cardiometabolic_II","B13805","20212097_SS211647","PASS",-4.0752,0.5768,"Plate control","PASS","PFC",1.49153724139182,"1h","1h"
8100,"PFC_24",87,"OID30349","Q8IZF2","ADGRF5",0,"Cardiometabolic_II","B13805","20212097_SS211647","PASS",-4.0752,0.8307,"Plate control","PASS","PFC",1.77854810993787,"24h","24h"
8101,"PFC_01",1,"OID30350","P49593","PPM1F",0,"Cardiometabolic_II","B13805","20212097_SS211647","PASS",-0.4288,1.5759,"Plate control","PASS","PFC",2.98121412441951,"Baseline","Baseline"
8102,"PFC_09",2,"OID30350","P49593","PPM1F",0,"Cardiometabolic_II","B13805","20212097_SS211647","PASS",-0.4288,1.7657,"Plate control","PASS","PFC",3.40038947562061,"Baseline","Baseline"
8103,"PFC_17",3,"OID30350","P49593","PPM1F",0,"Cardiometabolic_II","B13805","20212097_SS211647","PASS",-0.4288,2.7352,"Plate control","PASS","PFC",6.65851287959894,"1h","1h"
8104,"PFC_25",4,"OID30350","P49593","PPM1F",0,"Cardiometabolic_II","B13805","20212097_SS211647","PASS",-0.4288,2.3319,"Plate control","PASS","PFC",5.03467970452029,"24h","24h"
8105,"PFC_02",13,"OID30350","P49593","PPM1F",0,"Cardiometabolic_II","B13805","20212097_SS211647","PASS",-0.4288,1.5498,"Plate control","PASS","PFC",2.92776548927803,"Baseline","Baseline"
8106,"PFC_10",14,"OID30350","P49593","PPM1F",0,"Cardiometabolic_II","B13805","20212097_SS211647","PASS",-0.4288,1.8535,"Plate control","PASS","PFC",3.61375825677568,"1h","1h"
8107,"PFC_18",15,"OID30350","P49593","PPM1F",0,"Cardiometabolic_II","B13805","20212097_SS211647","PASS",-0.4288,2.302,"Plate control","PASS","PFC",4.93140930185522,"1h","1h"
8108,"PFC_26",16,"OID30350","P49593","PPM1F",0,"Cardiometabolic_II","B13805","20212097_SS211647","PASS",-0.4288,2.6434,"Plate control","PASS","PFC",6.24802402082441,"24h","24h"
8109,"PFC_03",25,"OID30350","P49593","PPM1F",0,"Cardiometabolic_II","B13805","20212097_SS211647","PASS",-0.4288,0.8999,"Plate control","PASS","PFC",1.86593664171881,"Baseline","Baseline"
8110,"PFC_11",26,"OID30350","P49593","PPM1F",0,"Cardiometabolic_II","B13805","20212097_SS211647","PASS",-0.4288,2.47,"Plate control","PASS","PFC",5.5404378724437,"1h","1h"
8111,"PFC_19",27,"OID30350","P49593","PPM1F",0,"Cardiometabolic_II","B13805","20212097_SS211647","PASS",-0.4288,2.3572,"Plate control","PASS","PFC",5.12374969861843,"24h","24h"
8112,"PFC_27",28,"OID30350","P49593","PPM1F",0,"Cardiometabolic_II","B13805","20212097_SS211647","PASS",-0.4288,2.931,"Plate control","PASS","PFC",7.62638836230527,"24h","24h"
8113,"PFC_04",37,"OID30350","P49593","PPM1F",0,"Cardiometabolic_II","B13805","20212097_SS211647","PASS",-0.4288,2.5751,"Plate control","PASS","PFC",5.95912289309894,"Baseline","Baseline"
8114,"PFC_12",38,"OID30350","P49593","PPM1F",0,"Cardiometabolic_II","B13805","20212097_SS211647","PASS",-0.4288,1.3755,"Plate control","PASS","PFC",2.59457816576858,"1h","1h"
8115,"PFC_20",39,"OID30350","P49593","PPM1F",0,"Cardiometabolic_II","B13805","20212097_SS211647","PASS",-0.4288,2.7064,"Plate control","PASS","PFC",6.52690933938795,"24h","24h"
8116,"PFC_05",49,"OID30350","P49593","PPM1F",0,"Cardiometabolic_II","B13805","20212097_SS211647","PASS",-0.4288,1.7289,"Plate control","PASS","PFC",3.31474984903766,"Baseline","Baseline"
8117,"PFC_13",50,"OID30350","P49593","PPM1F",0,"Cardiometabolic_II","B13805","20212097_SS211647","PASS",-0.4288,2.4052,"Plate control","PASS","PFC",5.29708992327298,"1h","1h"
8118,"PFC_21",51,"OID30350","P49593","PPM1F",0,"Cardiometabolic_II","B13805","20212097_SS211647","PASS",-0.4288,1.9298,"Plate control","PASS","PFC",3.81002377411593,"24h","24h"
8119,"PFC_06",61,"OID30350","P49593","PPM1F",0,"Cardiometabolic_II","B13805","20212097_SS211647","PASS",-0.4288,2.2543,"Plate control","PASS","PFC",4.77102749345486,"Baseline","Baseline"
8120,"PFC_14",62,"OID30350","P49593","PPM1F",0,"Cardiometabolic_II","B13805","20212097_SS211647","PASS",-0.4288,2.43,"Plate control","PASS","PFC",5.38893430746276,"1h","1h"
8121,"PFC_22",63,"OID30350","P49593","PPM1F",0,"Cardiometabolic_II","B13805","20212097_SS211647","PASS",-0.4288,2.0203,"Plate control","PASS","PFC",4.05668139464052,"24h","24h"
8122,"PFC_07",73,"OID30350","P49593","PPM1F",0,"Cardiometabolic_II","B13805","20212097_SS211647","PASS",-0.4288,2.7093,"Plate control","PASS","PFC",6.54004245011334,"Baseline","Baseline"
8123,"PFC_15",74,"OID30350","P49593","PPM1F",0,"Cardiometabolic_II","B13805","20212097_SS211647","PASS",-0.4288,1.6406,"Plate control","PASS","PFC",3.11795476996859,"1h","1h"
8124,"PFC_23",75,"OID30350","P49593","PPM1F",0,"Cardiometabolic_II","B13805","20212097_SS211647","PASS",-0.4288,2.7955,"Plate control","PASS","PFC",6.94271524205638,"24h","24h"
8125,"PFC_08",85,"OID30350","P49593","PPM1F",0,"Cardiometabolic_II","B13805","20212097_SS211647","PASS",-0.4288,1.9197,"Plate control","PASS","PFC",3.78344376008467,"Baseline","Baseline"
8126,"PFC_16",86,"OID30350","P49593","PPM1F",0,"Cardiometabolic_II","B13805","20212097_SS211647","PASS",-0.4288,2.4617,"Plate control","PASS","PFC",5.50865457390908,"1h","1h"
8127,"PFC_24",87,"OID30350","P49593","PPM1F",0,"Cardiometabolic_II","B13805","20212097_SS211647","PASS",-0.4288,2.6665,"Plate control","PASS","PFC",6.34887071519942,"24h","24h"
8128,"PFC_01",1,"OID30351","P05413","FABP3",0,"Cardiometabolic_II","B13805","20212097_SS211647","PASS",-5.4384,-0.1163,"Plate control","PASS","PFC",0.922550633695314,"Baseline","Baseline"
8129,"PFC_09",2,"OID30351","P05413","FABP3",0,"Cardiometabolic_II","B13805","20212097_SS211647","PASS",-5.4384,-0.0079,"Plate control","PASS","PFC",0.994539102481607,"Baseline","Baseline"
8130,"PFC_17",3,"OID30351","P05413","FABP3",0,"Cardiometabolic_II","B13805","20212097_SS211647","PASS",-5.4384,-0.4091,"Plate control","PASS","PFC",0.753093031076616,"1h","1h"
8131,"PFC_25",4,"OID30351","P05413","FABP3",0,"Cardiometabolic_II","B13805","20212097_SS211647","PASS",-5.4384,-1.0412,"Plate control","PASS","PFC",0.485923126072235,"24h","24h"
8132,"PFC_02",13,"OID30351","P05413","FABP3",0,"Cardiometabolic_II","B13805","20212097_SS211647","PASS",-5.4384,0.3124,"Plate control","PASS","PFC",1.24177173603258,"Baseline","Baseline"
8133,"PFC_10",14,"OID30351","P05413","FABP3",0,"Cardiometabolic_II","B13805","20212097_SS211647","PASS",-5.4384,0.6708,"Plate control","PASS","PFC",1.59195549036771,"1h","1h"
8134,"PFC_18",15,"OID30351","P05413","FABP3",0,"Cardiometabolic_II","B13805","20212097_SS211647","PASS",-5.4384,1.0503,"Plate control","PASS","PFC",2.07096044702937,"1h","1h"
8135,"PFC_26",16,"OID30351","P05413","FABP3",0,"Cardiometabolic_II","B13805","20212097_SS211647","PASS",-5.4384,-1.0504,"Plate control","PASS","PFC",0.482834275815589,"24h","24h"
8136,"PFC_03",25,"OID30351","P05413","FABP3",0,"Cardiometabolic_II","B13805","20212097_SS211647","PASS",-5.4384,0.2184,"Plate control","PASS","PFC",1.16344257160893,"Baseline","Baseline"
8137,"PFC_11",26,"OID30351","P05413","FABP3",0,"Cardiometabolic_II","B13805","20212097_SS211647","PASS",-5.4384,2.2231,"Plate control","PASS","PFC",4.66895602342844,"1h","1h"
8138,"PFC_19",27,"OID30351","P05413","FABP3",0,"Cardiometabolic_II","B13805","20212097_SS211647","PASS",-5.4384,-0.4835,"Plate control","PASS","PFC",0.715240333641229,"24h","24h"
8139,"PFC_27",28,"OID30351","P05413","FABP3",0,"Cardiometabolic_II","B13805","20212097_SS211647","PASS",-5.4384,0.4658,"Plate control","PASS","PFC",1.38108297611813,"24h","24h"
8140,"PFC_04",37,"OID30351","P05413","FABP3",0,"Cardiometabolic_II","B13805","20212097_SS211647","PASS",-5.4384,0.5635,"Plate control","PASS","PFC",1.47785015867351,"Baseline","Baseline"
8141,"PFC_12",38,"OID30351","P05413","FABP3",0,"Cardiometabolic_II","B13805","20212097_SS211647","PASS",-5.4384,0.9907,"Plate control","PASS","PFC",1.98714892767623,"1h","1h"
8142,"PFC_20",39,"OID30351","P05413","FABP3",0,"Cardiometabolic_II","B13805","20212097_SS211647","PASS",-5.4384,0.2072,"Plate control","PASS","PFC",1.15444544654549,"24h","24h"
8143,"PFC_05",49,"OID30351","P05413","FABP3",0,"Cardiometabolic_II","B13805","20212097_SS211647","PASS",-5.4384,0.3149,"Plate control","PASS","PFC",1.24392542796984,"Baseline","Baseline"
8144,"PFC_13",50,"OID30351","P05413","FABP3",0,"Cardiometabolic_II","B13805","20212097_SS211647","PASS",-5.4384,0.7005,"Plate control","PASS","PFC",1.62506790074446,"1h","1h"
8145,"PFC_21",51,"OID30351","P05413","FABP3",0,"Cardiometabolic_II","B13805","20212097_SS211647","PASS",-5.4384,0.3037,"Plate control","PASS","PFC",1.23430591350635,"24h","24h"
8146,"PFC_06",61,"OID30351","P05413","FABP3",0,"Cardiometabolic_II","B13805","20212097_SS211647","PASS",-5.4384,-0.1441,"Plate control","PASS","PFC",0.904943734789107,"Baseline","Baseline"
8147,"PFC_14",62,"OID30351","P05413","FABP3",0,"Cardiometabolic_II","B13805","20212097_SS211647","PASS",-5.4384,0.8915,"Plate control","PASS","PFC",1.85510391149546,"1h","1h"
8148,"PFC_22",63,"OID30351","P05413","FABP3",0,"Cardiometabolic_II","B13805","20212097_SS211647","PASS",-5.4384,-0.1371,"Plate control","PASS","PFC",0.909345218608751,"24h","24h"
8149,"PFC_07",73,"OID30351","P05413","FABP3",0,"Cardiometabolic_II","B13805","20212097_SS211647","PASS",-5.4384,-0.3649,"Plate control","PASS","PFC",0.776522697587517,"Baseline","Baseline"
8150,"PFC_15",74,"OID30351","P05413","FABP3",0,"Cardiometabolic_II","B13805","20212097_SS211647","PASS",-5.4384,0.7608,"Plate control","PASS","PFC",1.69442995574031,"1h","1h"
8151,"PFC_23",75,"OID30351","P05413","FABP3",0,"Cardiometabolic_II","B13805","20212097_SS211647","PASS",-5.4384,0.0859,"Plate control","PASS","PFC",1.06134963924909,"24h","24h"
8152,"PFC_08",85,"OID30351","P05413","FABP3",0,"Cardiometabolic_II","B13805","20212097_SS211647","PASS",-5.4384,-0.7375,"Plate control","PASS","PFC",0.599777788020451,"Baseline","Baseline"
8153,"PFC_16",86,"OID30351","P05413","FABP3",0,"Cardiometabolic_II","B13805","20212097_SS211647","PASS",-5.4384,-0.3191,"Plate control","PASS","PFC",0.801569766891372,"1h","1h"
8154,"PFC_24",87,"OID30351","P05413","FABP3",0,"Cardiometabolic_II","B13805","20212097_SS211647","PASS",-5.4384,-0.0499,"Plate control","PASS","PFC",0.966003284849634,"24h","24h"
8155,"PFC_01",1,"OID30352","Q9UBR1","UPB1",0,"Cardiometabolic_II","B13805","20212097_SS211647","PASS",-2.1127,-0.118,"Plate control","PASS","PFC",0.921464186198704,"Baseline","Baseline"
8156,"PFC_09",2,"OID30352","Q9UBR1","UPB1",0,"Cardiometabolic_II","B13805","20212097_SS211647","PASS",-2.1127,0.3157,"Plate control","PASS","PFC",1.24461539797504,"Baseline","Baseline"
8157,"PFC_17",3,"OID30352","Q9UBR1","UPB1",0,"Cardiometabolic_II","B13805","20212097_SS211647","PASS",-2.1127,-0.9197,"Plate control","PASS","PFC",0.528618932069243,"1h","1h"
8158,"PFC_25",4,"OID30352","Q9UBR1","UPB1",0,"Cardiometabolic_II","B13805","20212097_SS211647","PASS",-2.1127,-1.213,"Plate control","PASS","PFC",0.431370672271328,"24h","24h"
8159,"PFC_02",13,"OID30352","Q9UBR1","UPB1",0,"Cardiometabolic_II","B13805","20212097_SS211647","PASS",-2.1127,0.5982,"Plate control","PASS","PFC",1.51382663935719,"Baseline","Baseline"
8160,"PFC_10",14,"OID30352","Q9UBR1","UPB1",0,"Cardiometabolic_II","B13805","20212097_SS211647","PASS",-2.1127,0.2912,"Plate control","PASS","PFC",1.22365766434621,"1h","1h"
8161,"PFC_18",15,"OID30352","Q9UBR1","UPB1",0,"Cardiometabolic_II","B13805","20212097_SS211647","PASS",-2.1127,-0.1785,"Plate control","PASS","PFC",0.883621238205257,"1h","1h"
8162,"PFC_26",16,"OID30352","Q9UBR1","UPB1",0,"Cardiometabolic_II","B13805","20212097_SS211647","PASS",-2.1127,-1.2345,"Plate control","PASS","PFC",0.42498976427726,"24h","24h"
8163,"PFC_03",25,"OID30352","Q9UBR1","UPB1",0,"Cardiometabolic_II","B13805","20212097_SS211647","PASS",-2.1127,-0.8248,"Plate control","PASS","PFC",0.564560461676874,"Baseline","Baseline"
8164,"PFC_11",26,"OID30352","Q9UBR1","UPB1",0,"Cardiometabolic_II","B13805","20212097_SS211647","PASS",-2.1127,0.461,"Plate control","PASS","PFC",1.3764956015907,"1h","1h"
8165,"PFC_19",27,"OID30352","Q9UBR1","UPB1",0,"Cardiometabolic_II","B13805","20212097_SS211647","PASS",-2.1127,0.6885,"Plate control","PASS","PFC",1.61160702575404,"24h","24h"
8166,"PFC_27",28,"OID30352","Q9UBR1","UPB1",0,"Cardiometabolic_II","B13805","20212097_SS211647","PASS",-2.1127,1.2033,"Plate control","PASS","PFC",2.30265775709426,"24h","24h"
8167,"PFC_04",37,"OID30352","Q9UBR1","UPB1",0,"Cardiometabolic_II","B13805","20212097_SS211647","PASS",-2.1127,-0.4466,"Plate control","PASS","PFC",0.733770088150016,"Baseline","Baseline"
8168,"PFC_12",38,"OID30352","Q9UBR1","UPB1",0,"Cardiometabolic_II","B13805","20212097_SS211647","PASS",-2.1127,-1.0778,"Plate control","PASS","PFC",0.473750706540851,"1h","1h"
8169,"PFC_20",39,"OID30352","Q9UBR1","UPB1",0,"Cardiometabolic_II","B13805","20212097_SS211647","PASS",-2.1127,0.176,"Plate control","PASS","PFC",1.12974721457012,"24h","24h"
8170,"PFC_05",49,"OID30352","Q9UBR1","UPB1",0,"Cardiometabolic_II","B13805","20212097_SS211647","PASS",-2.1127,0.8584,"Plate control","PASS","PFC",1.8130264845336,"Baseline","Baseline"
8171,"PFC_13",50,"OID30352","Q9UBR1","UPB1",0,"Cardiometabolic_II","B13805","20212097_SS211647","PASS",-2.1127,-0.7186,"Plate control","PASS","PFC",0.607686859170707,"1h","1h"
8172,"PFC_21",51,"OID30352","Q9UBR1","UPB1",0,"Cardiometabolic_II","B13805","20212097_SS211647","PASS",-2.1127,-1.0398,"Plate control","PASS","PFC",0.486394897682954,"24h","24h"
8173,"PFC_06",61,"OID30352","Q9UBR1","UPB1",0,"Cardiometabolic_II","B13805","20212097_SS211647","PASS",-2.1127,-0.1047,"Plate control","PASS","PFC",0.92999831021963,"Baseline","Baseline"
8174,"PFC_14",62,"OID30352","Q9UBR1","UPB1",0,"Cardiometabolic_II","B13805","20212097_SS211647","PASS",-2.1127,1.0449,"Plate control","PASS","PFC",2.06322334190196,"1h","1h"
8175,"PFC_22",63,"OID30352","Q9UBR1","UPB1",0,"Cardiometabolic_II","B13805","20212097_SS211647","PASS",-2.1127,-0.3065,"Plate control","PASS","PFC",0.808601060021801,"24h","24h"
8176,"PFC_07",73,"OID30352","Q9UBR1","UPB1",0,"Cardiometabolic_II","B13805","20212097_SS211647","PASS",-2.1127,-1.2833,"Plate control","PASS","PFC",0.410854649941306,"Baseline","Baseline"

Supplemental table 1_Olink proximity extension assay (PEA) data

,"SampleID","Index","OlinkID","UniProt","Assay","MissingFreq","Panel","Panel_Lot_Nr","PlateID","QC_Warning","LOD","NPX","Normalization","Assay_Warning","condition","Value","group","alt_group"
8177,"PFC_15",74,"OID30352","Q9UBR1","UPB1",0,"Cardiometabolic_II","B13805","20212097_SS211647","PASS",-2.1127,-0.9242,"Plate control","PASS","PFC",0.526972652659738,"1h","1h"
8178,"PFC_23",75,"OID30352","Q9UBR1","UPB1",0,"Cardiometabolic_II","B13805","20212097_SS211647","PASS",-2.1127,1.349,"Plate control","PASS","PFC",2.547354950653,"24h","24h"
8179,"PFC_08",85,"OID30352","Q9UBR1","UPB1",0,"Cardiometabolic_II","B13805","20212097_SS211647","PASS",-2.1127,-0.9457,"Plate control","PASS","PFC",0.519177583991053,"Baseline","Baseline"
8180,"PFC_16",86,"OID30352","Q9UBR1","UPB1",0,"Cardiometabolic_II","B13805","20212097_SS211647","PASS",-2.1127,-0.9633,"Plate control","PASS","PFC",0.51288241068001,"1h","1h"
8181,"PFC_24",87,"OID30352","Q9UBR1","UPB1",0,"Cardiometabolic_II","B13805","20212097_SS211647","PASS",-2.1127,0.236,"Plate control","PASS","PFC",1.17772278959498,"24h","24h"
8182,"PFC_01",1,"OID30353","Q15388","TOMM20",0,"Cardiometabolic_II","B13805","20212097_SS211647","PASS",-1.1925,2.0712,"Plate control","PASS","PFC",4.20236070655923,"Baseline","Baseline"
8183,"PFC_09",2,"OID30353","Q15388","TOMM20",0,"Cardiometabolic_II","B13805","20212097_SS211647","PASS",-1.1925,2.9181,"Plate control","PASS","PFC",7.55850022513258,"Baseline","Baseline"
8184,"PFC_17",3,"OID30353","Q15388","TOMM20",0,"Cardiometabolic_II","B13805","20212097_SS211647","PASS",-1.1925,4.4067,"Plate control","PASS","PFC",21.2104011272477,"1h","1h"
8185,"PFC_25",4,"OID30353","Q15388","TOMM20",0,"Cardiometabolic_II","B13805","20212097_SS211647","PASS",-1.1925,2.8711,"Plate control","PASS","PFC",7.31622782069272,"24h","24h"
8186,"PFC_02",13,"OID30353","Q15388","TOMM20",0,"Cardiometabolic_II","B13805","20212097_SS211647","PASS",-1.1925,2.7567,"Plate control","PASS","PFC",6.75848554160951,"Baseline","Baseline"
8187,"PFC_10",14,"OID30353","Q15388","TOMM20",0,"Cardiometabolic_II","B13805","20212097_SS211647","PASS",-1.1925,2.5181,"Plate control","PASS","PFC",5.72827200460301,"1h","1h"
8188,"PFC_18",15,"OID30353","Q15388","TOMM20",0,"Cardiometabolic_II","B13805","20212097_SS211647","PASS",-1.1925,3.0289,"Plate control","PASS","PFC",8.16187151655725,"1h","1h"
8189,"PFC_26",16,"OID30353","Q15388","TOMM20",0,"Cardiometabolic_II","B13805","20212097_SS211647","PASS",-1.1925,3.6053,"Plate control","PASS","PFC",12.1703605181423,"24h","24h"
8190,"PFC_03",25,"OID30353","Q15388","TOMM20",0,"Cardiometabolic_II","B13805","20212097_SS211647","PASS",-1.1925,0.8163,"Plate control","PASS","PFC",1.76088415462407,"Baseline","Baseline"
8191,"PFC_11",26,"OID30353","Q15388","TOMM20",0,"Cardiometabolic_II","B13805","20212097_SS211647","PASS",-1.1925,3.6732,"Plate control","PASS","PFC",12.7568479767145,"1h","1h"
8192,"PFC_19",27,"OID30353","Q15388","TOMM20",0,"Cardiometabolic_II","B13805","20212097_SS211647","PASS",-1.1925,2.7728,"Plate control","PASS","PFC",6.83433042207072,"24h","24h"
8193,"PFC_27",28,"OID30353","Q15388","TOMM20",0,"Cardiometabolic_II","B13805","20212097_SS211647","PASS",-1.1925,4.6156,"Plate control","PASS","PFC",24.5151213860604,"24h","24h"
8194,"PFC_04",37,"OID30353","Q15388","TOMM20",0,"Cardiometabolic_II","B13805","20212097_SS211647","PASS",-1.1925,1.7022,"Plate control","PASS","PFC",3.25396785699152,"Baseline","Baseline"
8195,"PFC_12",38,"OID30353","Q15388","TOMM20",0,"Cardiometabolic_II","B13805","20212097_SS211647","PASS",-1.1925,0.3326,"Plate control","PASS","PFC",1.25928078477071,"1h","1h"
8196,"PFC_20",39,"OID30353","Q15388","TOMM20",0,"Cardiometabolic_II","B13805","20212097_SS211647","PASS",-1.1925,4.4732,"Plate control","PASS","PFC",22.2109623840237,"24h","24h"
8197,"PFC_05",49,"OID30353","Q15388","TOMM20",0,"Cardiometabolic_II","B13805","20212097_SS211647","PASS",-1.1925,1.1189,"Plate control","PASS","PFC",2.17181316880973,"Baseline","Baseline"
8198,"PFC_13",50,"OID30353","Q15388","TOMM20",0,"Cardiometabolic_II","B13805","20212097_SS211647","PASS",-1.1925,2.1897,"Plate control","PASS","PFC",4.56210610153786,"1h","1h"
8199,"PFC_21",51,"OID30353","Q15388","TOMM20",0,"Cardiometabolic_II","B13805","20212097_SS211647","PASS",-1.1925,1.535,"Plate control","PASS","PFC",2.89788430910976,"24h","24h"
8200,"PFC_06",61,"OID30353","Q15388","TOMM20",0,"Cardiometabolic_II","B13805","20212097_SS211647","PASS",-1.1925,2.2417,"Plate control","PASS","PFC",4.72954041843308,"Baseline","Baseline"
8201,"PFC_14",62,"OID30353","Q15388","TOMM20",0,"Cardiometabolic_II","B13805","20212097_SS211647","PASS",-1.1925,3.1267,"Plate control","PASS","PFC",8.73434792052161,"1h","1h"
8202,"PFC_22",63,"OID30353","Q15388","TOMM20",0,"Cardiometabolic_II","B13805","20212097_SS211647","PASS",-1.1925,2.2082,"Plate control","PASS","PFC",4.62098369817744,"24h","24h"
8203,"PFC_07",73,"OID30353","Q15388","TOMM20",0,"Cardiometabolic_II","B13805","20212097_SS211647","PASS",-1.1925,3.5946,"Plate control","PASS","PFC",12.08043081351,"Baseline","Baseline"
8204,"PFC_15",74,"OID30353","Q15388","TOMM20",0,"Cardiometabolic_II","B13805","20212097_SS211647","PASS",-1.1925,1.0134,"Plate control","PASS","PFC",2.01866288230087,"1h","1h"
8205,"PFC_23",75,"OID30353","Q15388","TOMM20",0,"Cardiometabolic_II","B13805","20212097_SS211647","PASS",-1.1925,3.6697,"Plate control","PASS","PFC",12.7259371806769,"24h","24h"
8206,"PFC_08",85,"OID30353","Q15388","TOMM20",0,"Cardiometabolic_II","B13805","20212097_SS211647","PASS",-1.1925,1.4605,"Plate control","PASS","PFC",2.75203725445237,"Baseline","Baseline"
8207,"PFC_16",86,"OID30353","Q15388","TOMM20",0,"Cardiometabolic_II","B13805","20212097_SS211647","PASS",-1.1925,2.1357,"Plate control","PASS","PFC",4.39450296408823,"1h","1h"
8208,"PFC_24",87,"OID30353","Q15388","TOMM20",0,"Cardiometabolic_II","B13805","20212097_SS211647","PASS",-1.1925,3.2192,"Plate control","PASS","PFC",9.31270320052827,"24h","24h"
8209,"PFC_01",1,"OID30354","O00194","RAB27B",0,"Cardiometabolic_II","B13805","20212097_SS211647","PASS",-1.7969,3.7557,"Plate control","PASS","PFC",13.5076050792076,"Baseline","Baseline"
8210,"PFC_09",2,"OID30354","O00194","RAB27B",0,"Cardiometabolic_II","B13805","20212097_SS211647","PASS",-1.7969,4.2491,"Plate control","PASS","PFC",19.0154476856269,"Baseline","Baseline"
8211,"PFC_17",3,"OID30354","O00194","RAB27B",0,"Cardiometabolic_II","B13805","20212097_SS211647","PASS",-1.7969,4.7527,"Plate control","PASS","PFC",26.9590919756165,"1h","1h"
8212,"PFC_25",4,"OID30354","O00194","RAB27B",0,"Cardiometabolic_II","B13805","20212097_SS211647","PASS",-1.7969,4.5452,"Plate control","PASS","PFC",23.3475619428518,"24h","24h"
8213,"PFC_02",13,"OID30354","O00194","RAB27B",0,"Cardiometabolic_II","B13805","20212097_SS211647","PASS",-1.7969,3.9424,"Plate control","PASS","PFC",15.373779749235,"Baseline","Baseline"
8214,"PFC_10",14,"OID30354","O00194","RAB27B",0,"Cardiometabolic_II","B13805","20212097_SS211647","PASS",-1.7969,4.0812,"Plate control","PASS","PFC",16.9263617472404,"1h","1h"
8215,"PFC_18",15,"OID30354","O00194","RAB27B",0,"Cardiometabolic_II","B13805","20212097_SS211647","PASS",-1.7969,4.4635,"Plate control","PASS","PFC",22.0621272734446,"1h","1h"
8216,"PFC_26",16,"OID30354","O00194","RAB27B",0,"Cardiometabolic_II","B13805","20212097_SS211647","PASS",-1.7969,4.6165,"Plate control","PASS","PFC",24.5304194858327,"24h","24h"
8217,"PFC_03",25,"OID30354","O00194","RAB27B",0,"Cardiometabolic_II","B13805","20212097_SS211647","PASS",-1.7969,3.126,"Plate control","PASS","PFC",8.73011101643948,"Baseline","Baseline"
8218,"PFC_11",26,"OID30354","O00194","RAB27B",0,"Cardiometabolic_II","B13805","20212097_SS211647","PASS",-1.7969,4.5874,"Plate control","PASS","PFC",24.040583372144,"1h","1h"
8219,"PFC_19",27,"OID30354","O00194","RAB27B",0,"Cardiometabolic_II","B13805","20212097_SS211647","PASS",-1.7969,4.3714,"Plate control","PASS","PFC",20.6977207414411,"24h","24h"
8220,"PFC_27",28,"OID30354","O00194","RAB27B",0,"Cardiometabolic_II","B13805","20212097_SS211647","PASS",-1.7969,4.6544,"Plate control","PASS","PFC",25.1833795988755,"24h","24h"
8221,"PFC_04",37,"OID30354","O00194","RAB27B",0,"Cardiometabolic_II","B13805","20212097_SS211647","PASS",-1.7969,4.2274,"Plate control","PASS","PFC",18.7315710379498,"Baseline","Baseline"
8222,"PFC_12",38,"OID30354","O00194","RAB27B",0,"Cardiometabolic_II","B13805","20212097_SS211647","PASS",-1.7969,3.2407,"Plate control","PASS","PFC",9.45252657345042,"1h","1h"
8223,"PFC_20",39,"OID30354","O00194","RAB27B",0,"Cardiometabolic_II","B13805","20212097_SS211647","PASS",-1.7969,4.6673,"Plate control","PASS","PFC",25.4095690118222,"24h","24h"
8224,"PFC_05",49,"OID30354","O00194","RAB27B",0,"Cardiometabolic_II","B13805","20212097_SS211647","PASS",-1.7969,3.5292,"Plate control","PASS","PFC",11.5450298649413,"Baseline","Baseline"
8225,"PFC_13",50,"OID30354","O00194","RAB27B",0,"Cardiometabolic_II","B13805","20212097_SS211647","PASS",-1.7969,4.4957,"Plate control","PASS","PFC",22.5600756441111,"1h","1h"
8226,"PFC_21",51,"OID30354","O00194","RAB27B",0,"Cardiometabolic_II","B13805","20212097_SS211647","PASS",-1.7969,3.8963,"Plate control","PASS","PFC",14.8902905502105,"24h","24h"
8227,"PFC_06",61,"OID30354","O00194","RAB27B",0,"Cardiometabolic_II","B13805","20212097_SS211647","PASS",-1.7969,4.4428,"Plate control","PASS","PFC",21.7478367711736,"Baseline","Baseline"
8228,"PFC_14",62,"OID30354","O00194","RAB27B",0,"Cardiometabolic_II","B13805","20212097_SS211647","PASS",-1.7969,4.5904,"Plate control","PASS","PFC",24.0906263726088,"1h","1h"
8229,"PFC_22",63,"OID30354","O00194","RAB27B",0,"Cardiometabolic_II","B13805","20212097_SS211647","PASS",-1.7969,4.2051,"Plate control","PASS","PFC",18.4442599631539,"24h","24h"
8230,"PFC_07",73,"OID30354","O00194","RAB27B",0,"Cardiometabolic_II","B13805","20212097_SS211647","PASS",-1.7969,4.5966,"Plate control","PASS","PFC",24.1943789203029,"Baseline","Baseline"
8231,"PFC_15",74,"OID30354","O00194","RAB27B",0,"Cardiometabolic_II","B13805","20212097_SS211647","PASS",-1.7969,3.7335,"Plate control","PASS","PFC",13.3013428809146,"1h","1h"
8232,"PFC_23",75,"OID30354","O00194","RAB27B",0,"Cardiometabolic_II","B13805","20212097_SS211647","PASS",-1.7969,4.596,"Plate control","PASS","PFC",24.1843188530596,"24h","24h"
8233,"PFC_08",85,"OID30354","O00194","RAB27B",0,"Cardiometabolic_II","B13805","20212097_SS211647","PASS",-1.7969,4.2623,"Plate control","PASS","PFC",19.1902287016621,"Baseline","Baseline"
8234,"PFC_16",86,"OID30354","O00194","RAB27B",0,"Cardiometabolic_II","B13805","20212097_SS211647","PASS",-1.7969,4.5044,"Plate control","PASS","PFC",22.6965325135907,"1h","1h"
8235,"PFC_24",87,"OID30354","O00194","RAB27B",0,"Cardiometabolic_II","B13805","20212097_SS211647","PASS",-1.7969,4.6963,"Plate control","PASS","PFC",25.9255016522574,"24h","24h"
8236,"PFC_01",1,"OID30355","Q6ZMM2","ADAMTSL5",0,"Cardiometabolic_II","B13805","20212097_SS211647","PASS",-3.4862,0.4105,"Plate control","PASS","PFC",1.32914638131506,"Baseline","Baseline"
8237,"PFC_09",2,"OID30355","Q6ZMM2","ADAMTSL5",0,"Cardiometabolic_II","B13805","20212097_SS211647","PASS",-3.4862,0.8244,"Plate control","PASS","PFC",1.77079843067837,"Baseline","Baseline"
8238,"PFC_17",3,"OID30355","Q6ZMM2","ADAMTSL5",0,"Cardiometabolic_II","B13805","20212097_SS211647","PASS",-3.4862,0.6701,"Plate control","PASS","PFC",1.59118325610625,"1h","1h"
8239,"PFC_25",4,"OID30355","Q6ZMM2","ADAMTSL5",0,"Cardiometabolic_II","B13805","20212097_SS211647","PASS",-3.4862,1.5381,"Plate control","PASS","PFC",2.90411785094153,"24h","24h"
8240,"PFC_02",13,"OID30355","Q6ZMM2","ADAMTSL5",0,"Cardiometabolic_II","B13805","20212097_SS211647","PASS",-3.4862,0.506,"Plate control","PASS","PFC",1.42010735854343,"Baseline","Baseline"
8241,"PFC_10",14,"OID30355","Q6ZMM2","ADAMTSL5",0,"Cardiometabolic_II","B13805","20212097_SS211647","PASS",-3.4862,0.4512,"Plate control","PASS","PFC",1.36717696977752,"1h","1h"
8242,"PFC_18",15,"OID30355","Q6ZMM2","ADAMTSL5",0,"Cardiometabolic_II","B13805","20212097_SS211647","PASS",-3.4862,0.6715,"Plate control","PASS","PFC",1.59272809941048,"1h","1h"
8243,"PFC_26",16,"OID30355","Q6ZMM2","ADAMTSL5",0,"Cardiometabolic_II","B13805","20212097_SS211647","PASS",-3.4862,0.4648,"Plate control","PASS","PFC",1.38012601404321,"24h","24h"
8244,"PFC_03",25,"OID30355","Q6ZMM2","ADAMTSL5",0,"Cardiometabolic_II","B13805","20212097_SS211647","PASS",-3.4862,0.5767,"Plate control","PASS","PFC",1.49143385949145,"Baseline","Baseline"
8245,"PFC_11",26,"OID30355","Q6ZMM2","ADAMTSL5",0,"Cardiometabolic_II","B13805","20212097_SS211647","PASS",-3.4862,1.6581,"Plate control","PASS","PFC",3.15600611350241,"1h","1h"
8246,"PFC_19",27,"OID30355","Q6ZMM2","ADAMTSL5",0,"Cardiometabolic_II","B13805","20212097_SS211647","PASS",-3.4862,0.2587,"Plate control","PASS","PFC",1.19640015294444,"24h","24h"
8247,"PFC_27",28,"OID30355","Q6ZMM2","ADAMTSL5",0,"Cardiometabolic_II","B13805","20212097_SS211647","PASS",-3.4862,1.3959,"Plate control","PASS","PFC",2.63152664020912,"24h","24h"
8248,"PFC_04",37,"OID30355","Q6ZMM2","ADAMTSL5",0,"Cardiometabolic_II","B13805","20212097_SS211647","PASS",-3.4862,0.321,"Plate control","PASS","PFC",1.24919612565338,"Baseline","Baseline"
8249,"PFC_12",38,"OID30355","Q6ZMM2","ADAMTSL5",0,"Cardiometabolic_II","B13805","20212097_SS211647","PASS",-3.4862,0.0497,"Plate control","PASS","PFC",1.03504966893372,"1h","1h"
8250,"PFC_20",39,"OID30355","Q6ZMM2","ADAMTSL5",0,"Cardiometabolic_II","B13805","20212097_SS211647","PASS",-3.4862,1.7633,"Plate control","PASS","PFC",3.39473744924064,"24h","24h"
8251,"PFC_05",49,"OID30355","Q6ZMM2","ADAMTSL5",0,"Cardiometabolic_II","B13805","20212097_SS211647","PASS",-3.4862,-0.6232,"Plate control","PASS","PFC",0.649229292835397,"Baseline","Baseline"
8252,"PFC_13",50,"OID30355","Q6ZMM2","ADAMTSL5",0,"Cardiometabolic_II","B13805","20212097_SS211647","PASS",-3.4862,0.1752,"Plate control","PASS","PFC",1.12912092335375,"1h","1h"
8253,"PFC_21",51,"OID30355","Q6ZMM2","ADAMTSL5",0,"Cardiometabolic_II","B13805","20212097_SS211647","PASS",-3.4862,1.1022,"Plate control","PASS","PFC",2.14681816435051,"24h","24h"
8254,"PFC_06",61,"OID30355","Q6ZMM2","ADAMTSL5",0,"Cardiometabolic_II","B13805","20212097_SS211647","PASS",-3.4862,0.0224,"Plate control","PASS","PFC",1.01564765915991,"Baseline","Baseline"
8255,"PFC_14",62,"OID30355","Q6ZMM2","ADAMTSL5",0,"Cardiometabolic_II","B13805","20212097_SS211647","PASS",-3.4862,1.4061,"Plate control","PASS","PFC",2.65019772543222,"1h","1h"
8256,"PFC_22",63,"OID30355","Q6ZMM2","ADAMTSL5",0,"Cardiometabolic_II","B13805","20212097_SS211647","PASS",-3.4862,0.8201,"Plate control","PASS","PFC",1.76552836544101,"24h","24h"
8257,"PFC_07",73,"OID30355","Q6ZMM2","ADAMTSL5",0,"Cardiometabolic_II","B13805","20212097_SS211647","PASS",-3.4862,1.3754,"Plate control","PASS","PFC",2.59439832954727,"Baseline","Baseline"
8258,"PFC_15",74,"OID30355","Q6ZMM2","ADAMTSL5",0,"Cardiometabolic_II","B13805","20212097_SS211647","PASS",-3.4862,0.954,"Plate control","PASS","PFC",1.93723637845326,"1h","1h"
8259,"PFC_23",75,"OID30355","Q6ZMM2","ADAMTSL5",0,"Cardiometabolic_II","B13805","20212097_SS211647","PASS",-3.4862,0.2467,"Plate control","PASS","PFC",1.18649004833285,"24h","24h"
8260,"PFC_08",85,"OID30355","Q6ZMM2","ADAMTSL5",0,"Cardiometabolic_II","B13805","20212097_SS211647","PASS",-3.4862,-0.1995,"Plate control","PASS","PFC",0.870852325418737,"Baseline","Baseline"
8261,"PFC_16",86,"OID30355","Q6ZMM2","ADAMTSL5",0,"Cardiometabolic_II","B13805","20212097_SS211647","PASS",-3.4862,0.8836,"Plate control","PASS","PFC",1.84497337914881,"1h","1h"
8262,"PFC_24",87,"OID30355","Q6ZMM2","ADAMTSL5",0,"Cardiometabolic_II","B13805","20212097_SS211647","PASS",-3.4862,1.3232,"Plate control","PASS","PFC",2.50220501545413,"24h","24h"
8263,"PFC_01",1,"OID30356","P45954","ACADSB",0,"Cardiometabolic_II","B13805","20212097_SS211647","PASS",-1.0193,1.3457,"Plate control","PASS","PFC",2.54153482634881,"Baseline","Baseline"
8264,"PFC_09",2,"OID30356","P45954","ACADSB",0,"Cardiometabolic_II","B13805","20212097_SS211647","PASS",-1.0193,3.1872,"Plate control","PASS","PFC",9.10841483949283,"Baseline","Baseline"
8265,"PFC_17",3,"OID30356","P45954","ACADSB",0,"Cardiometabolic_II","B13805","20212097_SS211647","PASS",-1.0193,4.4663,"Plate control","PASS","PFC",22.1049872953025,"1h","1h"
8266,"PFC_25",4,"OID30356","P45954","ACADSB",0,"Cardiometabolic_II","B13805","20212097_SS211647","PASS",-1.0193,2.6918,"Plate control","PASS","PFC",6.46119044759966,"24h","24h"
8267,"PFC_02",13,"OID30356","P45954","ACADSB",0,"Cardiometabolic_II","B13805","20212097_SS211647","PASS",-1.0193,1.8767,"Plate control","PASS","PFC",3.67234092797268,"Baseline","Baseline"
8268,"PFC_10",14,"OID30356","P45954","ACADSB",0,"Cardiometabolic_II","B13805","20212097_SS211647","PASS",-1.0193,1.702,"Plate control","PASS","PFC",3.2535167925285,"1h","1h"
8269,"PFC_18",15,"OID30356","P45954","ACADSB",0,"Cardiometabolic_II","B13805","20212097_SS211647","PASS",-1.0193,3.9381,"Plate control","PASS","PFC",15.3280259125365,"1h","1h"
8270,"PFC_26",16,"OID30356","P45954","ACADSB",0,"Cardiometabolic_II","B13805","20212097_SS211647","PASS",-1.0193,3.9812,"Plate control","PASS","PFC",15.7928539368619,"24h","24h"
8271,"PFC_03",25,"OID30356","P45954","ACADSB",0,"Cardiometabolic_II","B13805","20212097_SS211647","PASS",-1.0193,0.4993,"Plate control","PASS","PFC",1.41352754811422,"Baseline","Baseline"
8272,"PFC_11",26,"OID30356","P45954","ACADSB",0,"Cardiometabolic_II","B13805","20212097_SS211647","PASS",-1.0193,3.185,"Plate control","PASS","PFC",9.09453578588811,"1h","1h"
8273,"PFC_19",27,"OID30356","P45954","ACADSB",0,"Cardiometabolic_II","B13805","20212097_SS211647","PASS",-1.0193,2.2612,"Plate control","PASS","PFC",4.79390061503396,"24h","24h"
8274,"PFC_27",28,"OID30356","P45954","ACADSB",0,"Cardiometabolic_II","B13805","20212097_SS211647","PASS",-1.0193,5.7845,"Plate control","PASS","PFC",55.1198478273173,"24h","24h"
8275,"PFC_04",37,"OID30356","P45954","ACADSB",0,"Cardiometabolic_II","B13805","20212097_SS211647","PASS",-1.0193,1.0776,"Plate control","PASS","PFC",2.11052219314482,"Baseline","Baseline"
8276,"PFC_12",38,"OID30356","P45954","ACADSB",0,"Cardiometabolic_II","B13805","20212097_SS211647","PASS",-1.0193,-0.3579,"Plate control","PASS","PFC",0.780299564543576,"1h","1h"
8277,"PFC_20",39,"OID30356","P45954","ACADSB",0,"Cardiometabolic_II","B13805","20212097_SS211647","PASS",-1.0193,4.0603,"Plate control","PASS","PFC",16.6829209286733,"24h","24h"
8278,"PFC_05",49,"OID30356","P45954","ACADSB",0,"Cardiometabolic_II","B13805","20212097_SS211647","PASS",-1.0193,0.3445,"Plate control","PASS","PFC",1.26971085802475,"Baseline","Baseline"
8279,"PFC_13",50,"OID30356","P45954","ACADSB",0,"Cardiometabolic_II","B13805","20212097_SS211647","PASS",-1.0193,1.375,"Plate control","PASS","PFC",2.59367910930202,"1h","1h"
8280,"PFC_21",51,"OID30356","P45954","ACADSB",0,"Cardiometabolic_II","B13805","20212097_SS211647","PASS",-1.0193,0.8386,"Plate control","PASS","PFC",1.78831390892522,"24h","24h"
8281,"PFC_06",61,"OID30356","P45954","ACADSB",0,"Cardiometabolic_II","B13805","20212097_SS211647","PASS",-1.0193,1.6904,"Plate control","PASS","PFC",3.22746175525488,"Baseline","Baseline"
8282,"PFC_14",62,"OID30356","P45954","ACADSB",0,"Cardiometabolic_II","B13805","20212097_SS211647","PASS",-1.0193,2.1342,"Plate control","PASS","PFC",4.38993628252717,"1h","1h"
8283,"PFC_22",63,"OID30356","P45954","ACADSB",0,"Cardiometabolic_II","B13805","20212097_SS211647","PASS",-1.0193,1.2913,"Plate control","PASS","PFC",2.44748496954366,"24h","24h"
8284,"PFC_07",73,"OID30356","P45954","ACADSB",0,"Cardiometabolic_II","B13805","20212097_SS211647","PASS",-1.0193,3.2058,"Plate control","PASS","PFC",9.22660567267544,"Baseline","Baseline"
8285,"PFC_15",74,"OID30356","P45954","ACADSB",0,"Cardiometabolic_II","B13805","20212097_SS211647","PASS",-1.0193,0.1289,"Plate control","PASS","PFC",1.09345966200961,"1h","1h"
8286,"PFC_23",75,"OID30356","P45954","ACADSB",0,"Cardiometabolic_II","B13805","20212097_SS211647","PASS",-1.0193,2.9208,"Plate control","PASS","PFC",7.57265918365171,"24h","24h"
8287,"PFC_08",85,"OID30356","P45954","ACADSB",0,"Cardiometabolic_II","B13805","20212097_SS211647","PASS",-1.0193,1.0889,"Plate control","PASS","PFC",2.12711790009883,"Baseline","Baseline"
8288,"PFC_16",86,"OID30356","P45954","ACADSB",0,"Cardiometabolic_II","B13805","20212097_SS211647","PASS",-1.0193,1.6246,"Plate control","PASS","PFC",3.08356658609013,"1h","1h"

Supplemental table 1_Olink proximity extension assay (PEA) data

,"SampleID","Index","OlinkID","UniProt","Assay","MissingFreq","Panel","Panel_Lot_Nr","PlateID","QC_Warning","LOD","NPX","Normalization","Assay_Warning","condition","Value","group","alt_group"
8289,"PFC_24",87,"OID30356","P45954","ACADSB",0,"Cardiometabolic_II","B13805","20212097_SS211647","PASS",-1.0193,2.5106,"Plate control","PASS","PFC",5.69857025838014,"24h","24h"
8290,"PFC_01",1,"OID30357","P61026","RAB10",0.0227,"Cardiometabolic_II","B13805","20212097_SS211647","PASS",-0.0412,1.397,"Plate control","PASS","PFC",2.63353384411849,"Baseline","Baseline"
8291,"PFC_09",2,"OID30357","P61026","RAB10",0.0227,"Cardiometabolic_II","B13805","20212097_SS211647","PASS",-0.0412,1.9693,"Plate control","PASS","PFC",3.91578078065726,"Baseline","Baseline"
8292,"PFC_17",3,"OID30357","P61026","RAB10",0.0227,"Cardiometabolic_II","B13805","20212097_SS211647","PASS",-0.0412,3.3465,"Plate control","PASS","PFC",10.1717781733143,"1h","1h"
8293,"PFC_25",4,"OID30357","P61026","RAB10",0.0227,"Cardiometabolic_II","B13805","20212097_SS211647","PASS",-0.0412,2.3498,"Plate control","PASS","PFC",5.09753579177988,"24h","24h"
8294,"PFC_02",13,"OID30357","P61026","RAB10",0.0227,"Cardiometabolic_II","B13805","20212097_SS211647","PASS",-0.0412,1.2363,"Plate control","PASS","PFC",2.35593543125779,"Baseline","Baseline"
8295,"PFC_10",14,"OID30357","P61026","RAB10",0.0227,"Cardiometabolic_II","B13805","20212097_SS211647","PASS",-0.0412,1.7573,"Plate control","PASS","PFC",3.38064845064681,"1h","1h"
8296,"PFC_18",15,"OID30357","P61026","RAB10",0.0227,"Cardiometabolic_II","B13805","20212097_SS211647","PASS",-0.0412,2.2203,"Plate control","PASS","PFC",4.65990324477064,"1h","1h"
8297,"PFC_26",16,"OID30357","P61026","RAB10",0.0227,"Cardiometabolic_II","B13805","20212097_SS211647","PASS",-0.0412,2.9607,"Plate control","PASS","PFC",7.7850159763768,"24h","24h"
8298,"PFC_03",25,"OID30357","P61026","RAB10",0.0227,"Cardiometabolic_II","B13805","20212097_SS211647","PASS",-0.0412,0.5812,"Plate control","PASS","PFC",1.49609314653628,"Baseline","Baseline"
8299,"PFC_11",26,"OID30357","P61026","RAB10",0.0227,"Cardiometabolic_II","B13805","20212097_SS211647","PASS",-0.0412,2.3051,"Plate control","PASS","PFC",4.94201709115726,"1h","1h"
8300,"PFC_19",27,"OID30357","P61026","RAB10",0.0227,"Cardiometabolic_II","B13805","20212097_SS211647","PASS",-0.0412,2.2045,"Plate control","PASS","PFC",4.60914770148658,"24h","24h"
8301,"PFC_27",28,"OID30357","P61026","RAB10",0.0227,"Cardiometabolic_II","B13805","20212097_SS211647","PASS",-0.0412,3.9306,"Plate control","PASS","PFC",15.2485483431428,"24h","24h"
8302,"PFC_04",37,"OID30357","P61026","RAB10",0.0227,"Cardiometabolic_II","B13805","20212097_SS211647","PASS",-0.0412,1.337,"Plate control","PASS","PFC",2.5262545236916,"Baseline","Baseline"
8303,"PFC_12",38,"OID30357","P61026","RAB10",0.0227,"Cardiometabolic_II","B13805","20212097_SS211647","PASS",-0.0412,0.1306,"Plate control","PASS","PFC",1.09474889986631,"1h","1h"
8304,"PFC_20",39,"OID30357","P61026","RAB10",0.0227,"Cardiometabolic_II","B13805","20212097_SS211647","PASS",-0.0412,2.9052,"Plate control","PASS","PFC",7.49121641060251,"24h","24h"
8305,"PFC_05",49,"OID30357","P61026","RAB10",0.0227,"Cardiometabolic_II","B13805","20212097_SS211647","PASS",-0.0412,0.8422,"Plate control","PASS","PFC",1.7927819142585,"Baseline","Baseline"
8306,"PFC_13",50,"OID30357","P61026","RAB10",0.0227,"Cardiometabolic_II","B13805","20212097_SS211647","PASS",-0.0412,1.6009,"Plate control","PASS","PFC",3.03332482940654,"1h","1h"
8307,"PFC_21",51,"OID30357","P61026","RAB10",0.0227,"Cardiometabolic_II","B13805","20212097_SS211647","PASS",-0.0412,1.3446,"Plate control","PASS","PFC",2.53959774145027,"24h","24h"
8308,"PFC_06",61,"OID30357","P61026","RAB10",0.0227,"Cardiometabolic_II","B13805","20212097_SS211647","PASS",-0.0412,1.6706,"Plate control","PASS","PFC",3.18346962754451,"Baseline","Baseline"
8309,"PFC_14",62,"OID30357","P61026","RAB10",0.0227,"Cardiometabolic_II","B13805","20212097_SS211647","PASS",-0.0412,2.0215,"Plate control","PASS","PFC",4.06005705106729,"1h","1h"
8310,"PFC_22",63,"OID30357","P61026","RAB10",0.0227,"Cardiometabolic_II","B13805","20212097_SS211647","PASS",-0.0412,1.5484,"Plate control","PASS","PFC",2.92492574600098,"24h","24h"
8311,"PFC_07",73,"OID30357","P61026","RAB10",0.0227,"Cardiometabolic_II","B13805","20212097_SS211647","PASS",-0.0412,3.0875,"Plate control","PASS","PFC",8.50021893292122,"Baseline","Baseline"
8312,"PFC_15",74,"OID30357","P61026","RAB10",0.0227,"Cardiometabolic_II","B13805","20212097_SS211647","PASS",-0.0412,1.1744,"Plate control","PASS","PFC",2.25698995866139,"1h","1h"
8313,"PFC_23",75,"OID30357","P61026","RAB10",0.0227,"Cardiometabolic_II","B13805","20212097_SS211647","PASS",-0.0412,2.5305,"Plate control","PASS","PFC",5.77771883990174,"24h","24h"
8314,"PFC_08",85,"OID30357","P61026","RAB10",0.0227,"Cardiometabolic_II","B13805","20212097_SS211647","PASS",-0.0412,1.3841,"Plate control","PASS","PFC",2.61009080681631,"Baseline","Baseline"
8315,"PFC_16",86,"OID30357","P61026","RAB10",0.0227,"Cardiometabolic_II","B13805","20212097_SS211647","PASS",-0.0412,1.8001,"Plate control","PASS","PFC",3.48244362941726,"1h","1h"
8316,"PFC_24",87,"OID30357","P61026","RAB10",0.0227,"Cardiometabolic_II","B13805","20212097_SS211647","PASS",-0.0412,2.3901,"Plate control","PASS","PFC",5.24193694622272,"24h","24h"
8317,"PFC_01",1,"OID30358","P62072","TIMM10",0.0227,"Cardiometabolic_II","B13805","20212097_SS211647","PASS",0.2689,0.979,"Plate control","PASS","PFC",1.97109867387874,"Baseline","Baseline"
8318,"PFC_09",2,"OID30358","P62072","TIMM10",0.0227,"Cardiometabolic_II","B13805","20212097_SS211647","PASS",0.2689,1.5317,"Plate control","PASS","PFC",2.89126330527444,"Baseline","Baseline"
8319,"PFC_17",3,"OID30358","P62072","TIMM10",0.0227,"Cardiometabolic_II","B13805","20212097_SS211647","PASS",0.2689,2.4282,"Plate control","PASS","PFC",5.38221491579048,"1h","1h"
8320,"PFC_25",4,"OID30358","P62072","TIMM10",0.0227,"Cardiometabolic_II","B13805","20212097_SS211647","PASS",0.2689,2.2891,"Plate control","PASS","PFC",4.88751117141202,"24h","24h"
8321,"PFC_02",13,"OID30358","P62072","TIMM10",0.0227,"Cardiometabolic_II","B13805","20212097_SS211647","PASS",0.2689,1.0185,"Plate control","PASS","PFC",2.02581158584617,"Baseline","Baseline"
8322,"PFC_10",14,"OID30358","P62072","TIMM10",0.0227,"Cardiometabolic_II","B13805","20212097_SS211647","PASS",0.2689,1.2099,"Plate control","PASS","PFC",2.3132160223371,"1h","1h"
8323,"PFC_18",15,"OID30358","P62072","TIMM10",0.0227,"Cardiometabolic_II","B13805","20212097_SS211647","PASS",0.2689,1.5731,"Plate control","PASS","PFC",2.9754337590735,"1h","1h"
8324,"PFC_26",16,"OID30358","P62072","TIMM10",0.0227,"Cardiometabolic_II","B13805","20212097_SS211647","PASS",0.2689,2.2472,"Plate control","PASS","PFC",4.74760530285423,"24h","24h"
8325,"PFC_03",25,"OID30358","P62072","TIMM10",0.0227,"Cardiometabolic_II","B13805","20212097_SS211647","PASS",0.2689,0.5329,"Plate control","PASS","PFC",1.44683459546271,"Baseline","Baseline"
8326,"PFC_11",26,"OID30358","P62072","TIMM10",0.0227,"Cardiometabolic_II","B13805","20212097_SS211647","PASS",0.2689,1.635,"Plate control","PASS","PFC",3.10587550000416,"1h","1h"
8327,"PFC_19",27,"OID30358","P62072","TIMM10",0.0227,"Cardiometabolic_II","B13805","20212097_SS211647","PASS",0.2689,1.4948,"Plate control","PASS","PFC",2.81825079068411,"24h","24h"
8328,"PFC_27",28,"OID30358","P62072","TIMM10",0.0227,"Cardiometabolic_II","B13805","20212097_SS211647","PASS",0.2689,2.4972,"Plate control","PASS","PFC",5.6458860053789,"24h","24h"
8329,"PFC_04",37,"OID30358","P62072","TIMM10",0.0227,"Cardiometabolic_II","B13805","20212097_SS211647","PASS",0.2689,1.0326,"Plate control","PASS","PFC",2.04570767023074,"Baseline","Baseline"
8330,"PFC_12",38,"OID30358","P62072","TIMM10",0.0227,"Cardiometabolic_II","B13805","20212097_SS211647","PASS",0.2689,0.4888,"Plate control","PASS","PFC",1.40327717703052,"1h","1h"
8331,"PFC_20",39,"OID30358","P62072","TIMM10",0.0227,"Cardiometabolic_II","B13805","20212097_SS211647","PASS",0.2689,2.1284,"Plate control","PASS","PFC",4.37232305372255,"24h","24h"
8332,"PFC_05",49,"OID30358","P62072","TIMM10",0.0227,"Cardiometabolic_II","B13805","20212097_SS211647","PASS",0.2689,0.261,"Plate control","PASS","PFC",1.19830902133942,"Baseline","Baseline"
8333,"PFC_13",50,"OID30358","P62072","TIMM10",0.0227,"Cardiometabolic_II","B13805","20212097_SS211647","PASS",0.2689,1.0446,"Plate control","PASS","PFC",2.06279435127386,"1h","1h"
8334,"PFC_21",51,"OID30358","P62072","TIMM10",0.0227,"Cardiometabolic_II","B13805","20212097_SS211647","PASS",0.2689,0.8487,"Plate control","PASS","PFC",1.80087743882172,"24h","24h"
8335,"PFC_06",61,"OID30358","P62072","TIMM10",0.0227,"Cardiometabolic_II","B13805","20212097_SS211647","PASS",0.2689,0.7441,"Plate control","PASS","PFC",1.67492906822945,"Baseline","Baseline"
8336,"PFC_14",62,"OID30358","P62072","TIMM10",0.0227,"Cardiometabolic_II","B13805","20212097_SS211647","PASS",0.2689,1.4127,"Plate control","PASS","PFC",2.66234954888269,"1h","1h"
8337,"PFC_22",63,"OID30358","P62072","TIMM10",0.0227,"Cardiometabolic_II","B13805","20212097_SS211647","PASS",0.2689,0.8982,"Plate control","PASS","PFC",1.86373920981731,"24h","24h"
8338,"PFC_07",73,"OID30358","P62072","TIMM10",0.0227,"Cardiometabolic_II","B13805","20212097_SS211647","PASS",0.2689,1.8342,"Plate control","PASS","PFC",3.56573626533388,"Baseline","Baseline"
8339,"PFC_15",74,"OID30358","P62072","TIMM10",0.0227,"Cardiometabolic_II","B13805","20212097_SS211647","PASS",0.2689,0.341,"Plate control","PASS","PFC",1.26663425372322,"1h","1h"
8340,"PFC_23",75,"OID30358","P62072","TIMM10",0.0227,"Cardiometabolic_II","B13805","20212097_SS211647","PASS",0.2689,1.6997,"Plate control","PASS","PFC",3.24833404311479,"24h","24h"
8341,"PFC_08",85,"OID30358","P62072","TIMM10",0.0227,"Cardiometabolic_II","B13805","20212097_SS211647","PASS",0.2689,0.9698,"Plate control","PASS","PFC",1.95856906102846,"Baseline","Baseline"
8342,"PFC_16",86,"OID30358","P62072","TIMM10",0.0227,"Cardiometabolic_II","B13805","20212097_SS211647","PASS",0.2689,1.3323,"Plate control","PASS","PFC",2.51803790383461,"1h","1h"
8343,"PFC_24",87,"OID30358","P62072","TIMM10",0.0227,"Cardiometabolic_II","B13805","20212097_SS211647","PASS",0.2689,1.757,"Plate control","PASS","PFC",3.37994553765005,"24h","24h"
8344,"PFC_01",1,"OID30359","P07093","SERPINE2",0,"Cardiometabolic_II","B13805","20212097_SS211647","PASS",-0.9814,2.5359,"Plate control","PASS","PFC",5.79938533482404,"Baseline","Baseline"
8345,"PFC_09",2,"OID30359","P07093","SERPINE2",0,"Cardiometabolic_II","B13805","20212097_SS211647","PASS",-0.9814,2.6843,"Plate control","PASS","PFC",6.42768843533174,"Baseline","Baseline"
8346,"PFC_17",3,"OID30359","P07093","SERPINE2",0,"Cardiometabolic_II","B13805","20212097_SS211647","PASS",-0.9814,2.8696,"Plate control","PASS","PFC",7.30862493978486,"1h","1h"
8347,"PFC_25",4,"OID30359","P07093","SERPINE2",0,"Cardiometabolic_II","B13805","20212097_SS211647","PASS",-0.9814,1.7692,"Plate control","PASS","PFC",3.40864888662012,"24h","24h"
8348,"PFC_02",13,"OID30359","P07093","SERPINE2",0,"Cardiometabolic_II","B13805","20212097_SS211647","PASS",-0.9814,1.8478,"Plate control","PASS","PFC",3.59950868672503,"Baseline","Baseline"
8349,"PFC_10",14,"OID30359","P07093","SERPINE2",0,"Cardiometabolic_II","B13805","20212097_SS211647","PASS",-0.9814,1.7865,"Plate control","PASS","PFC",3.44976957193898,"1h","1h"
8350,"PFC_18",15,"OID30359","P07093","SERPINE2",0,"Cardiometabolic_II","B13805","20212097_SS211647","PASS",-0.9814,2.9153,"Plate control","PASS","PFC",7.54384482268721,"1h","1h"
8351,"PFC_26",16,"OID30359","P07093","SERPINE2",0,"Cardiometabolic_II","B13805","20212097_SS211647","PASS",-0.9814,2.7166,"Plate control","PASS","PFC",6.57321876246467,"24h","24h"
8352,"PFC_03",25,"OID30359","P07093","SERPINE2",0,"Cardiometabolic_II","B13805","20212097_SS211647","PASS",-0.9814,1.0796,"Plate control","PASS","PFC",2.11345002711116,"Baseline","Baseline"
8353,"PFC_11",26,"OID30359","P07093","SERPINE2",0,"Cardiometabolic_II","B13805","20212097_SS211647","PASS",-0.9814,3.0103,"Plate control","PASS","PFC",8.05731969879309,"1h","1h"
8354,"PFC_19",27,"OID30359","P07093","SERPINE2",0,"Cardiometabolic_II","B13805","20212097_SS211647","PASS",-0.9814,2.7386,"Plate control","PASS","PFC",6.67422350500553,"24h","24h"
8355,"PFC_27",28,"OID30359","P07093","SERPINE2",0,"Cardiometabolic_II","B13805","20212097_SS211647","PASS",-0.9814,4.4219,"Plate control","PASS","PFC",21.435051822778,"24h","24h"
8356,"PFC_04",37,"OID30359","P07093","SERPINE2",0,"Cardiometabolic_II","B13805","20212097_SS211647","PASS",-0.9814,2.4909,"Plate control","PASS","PFC",5.6212851495275,"Baseline","Baseline"
8357,"PFC_12",38,"OID30359","P07093","SERPINE2",0,"Cardiometabolic_II","B13805","20212097_SS211647","PASS",-0.9814,1.0015,"Plate control","PASS","PFC",2.00208052293571,"1h","1h"
8358,"PFC_20",39,"OID30359","P07093","SERPINE2",0,"Cardiometabolic_II","B13805","20212097_SS211647","PASS",-0.9814,3.6335,"Plate control","PASS","PFC",12.4105917396366,"24h","24h"
8359,"PFC_05",49,"OID30359","P07093","SERPINE2",0,"Cardiometabolic_II","B13805","20212097_SS211647","PASS",-0.9814,0.1273,"Plate control","PASS","PFC",1.09224764864591,"Baseline","Baseline"
8360,"PFC_13",50,"OID30359","P07093","SERPINE2",0,"Cardiometabolic_II","B13805","20212097_SS211647","PASS",-0.9814,2.3503,"Plate control","PASS","PFC",5.09930276923699,"1h","1h"
8361,"PFC_21",51,"OID30359","P07093","SERPINE2",0,"Cardiometabolic_II","B13805","20212097_SS211647","PASS",-0.9814,2.0811,"Plate control","PASS","PFC",4.2312971354772,"24h","24h"
8362,"PFC_06",61,"OID30359","P07093","SERPINE2",0,"Cardiometabolic_II","B13805","20212097_SS211647","PASS",-0.9814,3.0808,"Plate control","PASS","PFC",8.46083470689854,"Baseline","Baseline"
8363,"PFC_14",62,"OID30359","P07093","SERPINE2",0,"Cardiometabolic_II","B13805","20212097_SS211647","PASS",-0.9814,0.9898,"Plate control","PASS","PFC",1.98590966625326,"1h","1h"
8364,"PFC_22",63,"OID30359","P07093","SERPINE2",0,"Cardiometabolic_II","B13805","20212097_SS211647","PASS",-0.9814,2.1397,"Plate control","PASS","PFC",4.40670401988511,"24h","24h"
8365,"PFC_07",73,"OID30359","P07093","SERPINE2",0,"Cardiometabolic_II","B13805","20212097_SS211647","PASS",-0.9814,2.3311,"Plate control","PASS","PFC",5.03188865920012,"Baseline","Baseline"
8366,"PFC_15",74,"OID30359","P07093","SERPINE2",0,"Cardiometabolic_II","B13805","20212097_SS211647","PASS",-0.9814,1.6368,"Plate control","PASS","PFC",3.10975301034078,"1h","1h"
8367,"PFC_23",75,"OID30359","P07093","SERPINE2",0,"Cardiometabolic_II","B13805","20212097_SS211647","PASS",-0.9814,1.143,"Plate control","PASS","PFC",2.20839769432619,"24h","24h"
8368,"PFC_08",85,"OID30359","P07093","SERPINE2",0,"Cardiometabolic_II","B13805","20212097_SS211647","PASS",-0.9814,1.5193,"Plate control","PASS","PFC",2.86651931470955,"Baseline","Baseline"
8369,"PFC_16",86,"OID30359","P07093","SERPINE2",0,"Cardiometabolic_II","B13805","20212097_SS211647","PASS",-0.9814,1.3009,"Plate control","PASS","PFC",2.46382536161233,"1h","1h"
8370,"PFC_24",87,"OID30359","P07093","SERPINE2",0,"Cardiometabolic_II","B13805","20212097_SS211647","PASS",-0.9814,2.4957,"Plate control","PASS","PFC",5.64001891102778,"24h","24h"
8371,"PFC_01",1,"OID30360","Q7Z7K0","CMC1",0,"Cardiometabolic_II","B13805","20212097_SS211647","PASS",-0.1928,2.453,"Plate control","PASS","PFC",5.47553525236735,"Baseline","Baseline"
8372,"PFC_09",2,"OID30360","Q7Z7K0","CMC1",0,"Cardiometabolic_II","B13805","20212097_SS211647","PASS",-0.1928,2.7862,"Plate control","PASS","PFC",6.89810457420689,"Baseline","Baseline"
8373,"PFC_17",3,"OID30360","Q7Z7K0","CMC1",0,"Cardiometabolic_II","B13805","20212097_SS211647","PASS",-0.1928,4.933,"Plate control","PASS","PFC",30.547872452471,"1h","1h"
8374,"PFC_25",4,"OID30360","Q7Z7K0","CMC1",0,"Cardiometabolic_II","B13805","20212097_SS211647","PASS",-0.1928,4.0261,"Plate control","PASS","PFC",16.2920924388909,"24h","24h"
8375,"PFC_02",13,"OID30360","Q7Z7K0","CMC1",0,"Cardiometabolic_II","B13805","20212097_SS211647","PASS",-0.1928,2.5989,"Plate control","PASS","PFC",6.05824532338755,"Baseline","Baseline"
8376,"PFC_10",14,"OID30360","Q7Z7K0","CMC1",0,"Cardiometabolic_II","B13805","20212097_SS211647","PASS",-0.1928,3.171,"Plate control","PASS","PFC",9.0067086890941,"1h","1h"
8377,"PFC_18",15,"OID30360","Q7Z7K0","CMC1",0,"Cardiometabolic_II","B13805","20212097_SS211647","PASS",-0.1928,4.5373,"Plate control","PASS","PFC",23.2200632997775,"1h","1h"
8378,"PFC_26",16,"OID30360","Q7Z7K0","CMC1",0,"Cardiometabolic_II","B13805","20212097_SS211647","PASS",-0.1928,4.6266,"Plate control","PASS","PFC",24.7027542515837,"24h","24h"
8379,"PFC_03",25,"OID30360","Q7Z7K0","CMC1",0,"Cardiometabolic_II","B13805","20212097_SS211647","PASS",-0.1928,1.6106,"Plate control","PASS","PFC",3.05378818866521,"Baseline","Baseline"
8380,"PFC_11",26,"OID30360","Q7Z7K0","CMC1",0,"Cardiometabolic_II","B13805","20212097_SS211647","PASS",-0.1928,3.8386,"Plate control","PASS","PFC",14.3065112714018,"1h","1h"
8381,"PFC_19",27,"OID30360","Q7Z7K0","CMC1",0,"Cardiometabolic_II","B13805","20212097_SS211647","PASS",-0.1928,3.7372,"Plate control","PASS","PFC",13.3354998792403,"24h","24h"
8382,"PFC_27",28,"OID30360","Q7Z7K0","CMC1",0,"Cardiometabolic_II","B13805","20212097_SS211647","PASS",-0.1928,5.0485,"Plate control","PASS","PFC",33.0940510967529,"24h","24h"
8383,"PFC_04",37,"OID30360","Q7Z7K0","CMC1",0,"Cardiometabolic_II","B13805","20212097_SS211647","PASS",-0.1928,3.107,"Plate control","PASS","PFC",8.61589100954439,"Baseline","Baseline"
8384,"PFC_12",38,"OID30360","Q7Z7K0","CMC1",0,"Cardiometabolic_II","B13805","20212097_SS211647","PASS",-0.1928,1.8874,"Plate control","PASS","PFC",3.69967873902097,"1h","1h"
8385,"PFC_20",39,"OID30360","Q7Z7K0","CMC1",0,"Cardiometabolic_II","B13805","20212097_SS211647","PASS",-0.1928,4.5038,"Plate control","PASS","PFC",22.6870952536376,"24h","24h"
8386,"PFC_05",49,"OID30360","Q7Z7K0","CMC1",0,"Cardiometabolic_II","B13805","20212097_SS211647","PASS",-0.1928,2.0665,"Plate control","PASS","PFC",4.18869256659,"Baseline","Baseline"
8387,"PFC_13",50,"OID30360","Q7Z7K0","CMC1",0,"Cardiometabolic_II","B13805","20212097_SS211647","PASS",-0.1928,3.2044,"Plate control","PASS","PFC",9.21765646157062,"1h","1h"
8388,"PFC_21",51,"OID30360","Q7Z7K0","CMC1",0,"Cardiometabolic_II","B13805","20212097_SS211647","PASS",-0.1928,2.6423,"Plate control","PASS","PFC",6.24326195624399,"24h","24h"
8389,"PFC_06",61,"OID30360","Q7Z7K0","CMC1",0,"Cardiometabolic_II","B13805","20212097_SS211647","PASS",-0.1928,3.2255,"Plate control","PASS","PFC",9.35345908871557,"Baseline","Baseline"
8390,"PFC_14",62,"OID30360","Q7Z7K0","CMC1",0,"Cardiometabolic_II","B13805","20212097_SS211647","PASS",-0.1928,3.8023,"Plate control","PASS","PFC",13.951032566654,"1h","1h"
8391,"PFC_22",63,"OID30360","Q7Z7K0","CMC1",0,"Cardiometabolic_II","B13805","20212097_SS211647","PASS",-0.1928,3.0962,"Plate control","PASS","PFC",8.55163335562863,"24h","24h"
8392,"PFC_07",73,"OID30360","Q7Z7K0","CMC1",0,"Cardiometabolic_II","B13805","20212097_SS211647","PASS",-0.1928,4.593,"Plate control","PASS","PFC",24.1340812269853,"Baseline","Baseline"
8393,"PFC_15",74,"OID30360","Q7Z7K0","CMC1",0,"Cardiometabolic_II","B13805","20212097_SS211647","PASS",-0.1928,1.9276,"Plate control","PASS","PFC",3.80421820584439,"1h","1h"
8394,"PFC_23",75,"OID30360","Q7Z7K0","CMC1",0,"Cardiometabolic_II","B13805","20212097_SS211647","PASS",-0.1928,4.1979,"Plate control","PASS","PFC",18.3524402493087,"24h","24h"
8395,"PFC_08",85,"OID30360","Q7Z7K0","CMC1",0,"Cardiometabolic_II","B13805","20212097_SS211647","PASS",-0.1928,2.9775,"Plate control","PASS","PFC",7.87620138669931,"Baseline","Baseline"
8396,"PFC_16",86,"OID30360","Q7Z7K0","CMC1",0,"Cardiometabolic_II","B13805","20212097_SS211647","PASS",-0.1928,3.6096,"Plate control","PASS","PFC",12.2066887896945,"1h","1h"
8397,"PFC_24",87,"OID30360","Q7Z7K0","CMC1",0,"Cardiometabolic_II","B13805","20212097_SS211647","PASS",-0.1928,4.0924,"Plate control","PASS","PFC",17.058276680046,"24h","24h"
8398,"PFC_01",1,"OID30361","Q96AG4","LRRC59",0.0227,"Cardiometabolic_II","B13805","20212097_SS211647","PASS",0.1149,1.0089,"Plate control","PASS","PFC",2.01237615487539,"Baseline","Baseline"
8399,"PFC_09",2,"OID30361","Q96AG4","LRRC59",0.0227,"Cardiometabolic_II","B13805","20212097_SS211647","PASS",0.1149,2.3727,"Plate control","PASS","PFC",5.17909492092322,"Baseline","Baseline"
8400,"PFC_17",3,"OID30361","Q96AG4","LRRC59",0.0227,"Cardiometabolic_II","B13805","20212097_SS211647","PASS",0.1149,4.0083,"Plate control","PASS","PFC",16.092315241359,"1h","1h"

Supplemental table 1_Olink proximity extension assay (PEA) data

,"SampleID","Index","OlinkID","UniProt","Assay","MissingFreq","Panel","Panel_Lot_Nr","PlateID","QC_Warning","LOD","NPX","Normalization","Assay_Warning","condition","Value","group","alt_group"
8401,"PFC_25",4,"OID30361","Q96AG4","LRRC59",0.0227,"Cardiometabolic_II","B13805","20212097_SS211647","PASS",0.1149,2.1254,"Plate control","PASS","PFC",4.36324051011304,"24h","24h"
8402,"PFC_02",13,"OID30361","Q96AG4","LRRC59",0.0227,"Cardiometabolic_II","B13805","20212097_SS211647","PASS",0.1149,1.203,"Plate control","PASS","PFC",2.30217898265553,"Baseline","Baseline"
8403,"PFC_10",14,"OID30361","Q96AG4","LRRC59",0.0227,"Cardiometabolic_II","B13805","20212097_SS211647","PASS",0.1149,1.7146,"Plate control","PASS","PFC",3.28205632984384,"1h","1h"
8404,"PFC_18",15,"OID30361","Q96AG4","LRRC59",0.0227,"Cardiometabolic_II","B13805","20212097_SS211647","PASS",0.1149,3.2319,"Plate control","PASS","PFC",9.39504453227644,"1h","1h"
8405,"PFC_26",16,"OID30361","Q96AG4","LRRC59",0.0227,"Cardiometabolic_II","B13805","20212097_SS211647","PASS",0.1149,2.938,"Plate control","PASS","PFC",7.66348174578157,"24h","24h"
8406,"PFC_03",25,"OID30361","Q96AG4","LRRC59",0.0227,"Cardiometabolic_II","B13805","20212097_SS211647","PASS",0.1149,0.5906,"Plate control","PASS","PFC",1.50587289212858,"Baseline","Baseline"
8407,"PFC_11",26,"OID30361","Q96AG4","LRRC59",0.0227,"Cardiometabolic_II","B13805","20212097_SS211647","PASS",0.1149,3.1506,"Plate control","PASS","PFC",8.88024820007318,"1h","1h"
8408,"PFC_19",27,"OID30361","Q96AG4","LRRC59",0.0227,"Cardiometabolic_II","B13805","20212097_SS211647","PASS",0.1149,2.0495,"Plate control","PASS","PFC",4.13962476210867,"24h","24h"
8409,"PFC_27",28,"OID30361","Q96AG4","LRRC59",0.0227,"Cardiometabolic_II","B13805","20212097_SS211647","PASS",0.1149,4.5501,"Plate control","PASS","PFC",23.4269949144008,"24h","24h"
8410,"PFC_04",37,"OID30361","Q96AG4","LRRC59",0.0227,"Cardiometabolic_II","B13805","20212097_SS211647","PASS",0.1149,1.4894,"Plate control","PASS","PFC",2.8077218099524,"Baseline","Baseline"
8411,"PFC_12",38,"OID30361","Q96AG4","LRRC59",0.0227,"Cardiometabolic_II","B13805","20212097_SS211647","PASS",0.1149,0.1234,"Plate control","PASS","PFC",1.08929899128125,"1h","1h"
8412,"PFC_20",39,"OID30361","Q96AG4","LRRC59",0.0227,"Cardiometabolic_II","B13805","20212097_SS211647","PASS",0.1149,3.9819,"Plate control","PASS","PFC",15.8005185366805,"24h","24h"
8413,"PFC_05",49,"OID30361","Q96AG4","LRRC59",0.0227,"Cardiometabolic_II","B13805","20212097_SS211647","PASS",0.1149,0.3947,"Plate control","PASS","PFC",1.31466935397262,"Baseline","Baseline"
8414,"PFC_13",50,"OID30361","Q96AG4","LRRC59",0.0227,"Cardiometabolic_II","B13805","20212097_SS211647","PASS",0.1149,1.9733,"Plate control","PASS","PFC",3.92665269499743,"1h","1h"
8415,"PFC_21",51,"OID30361","Q96AG4","LRRC59",0.0227,"Cardiometabolic_II","B13805","20212097_SS211647","PASS",0.1149,1.078,"Plate control","PASS","PFC",2.11110743527593,"24h","24h"
8416,"PFC_06",61,"OID30361","Q96AG4","LRRC59",0.0227,"Cardiometabolic_II","B13805","20212097_SS211647","PASS",0.1149,1.5153,"Plate control","PASS","PFC",2.85858264323268,"Baseline","Baseline"
8417,"PFC_14",62,"OID30361","Q96AG4","LRRC59",0.0227,"Cardiometabolic_II","B13805","20212097_SS211647","PASS",0.1149,2.9073,"Plate control","PASS","PFC",7.50212863326231,"1h","1h"
8418,"PFC_22",63,"OID30361","Q96AG4","LRRC59",0.0227,"Cardiometabolic_II","B13805","20212097_SS211647","PASS",0.1149,1.8039,"Plate control","PASS","PFC",3.49162833491345,"24h","24h"
8419,"PFC_07",73,"OID30361","Q96AG4","LRRC59",0.0227,"Cardiometabolic_II","B13805","20212097_SS211647","PASS",0.1149,3.2556,"Plate control","PASS","PFC",9.55065712504794,"Baseline","Baseline"
8420,"PFC_15",74,"OID30361","Q96AG4","LRRC59",0.0227,"Cardiometabolic_II","B13805","20212097_SS211647","PASS",0.1149,0.784,"Plate control","PASS","PFC",1.72189837667781,"1h","1h"
8421,"PFC_23",75,"OID30361","Q96AG4","LRRC59",0.0227,"Cardiometabolic_II","B13805","20212097_SS211647","PASS",0.1149,3.0874,"Plate control","PASS","PFC",8.49962976306178,"24h","24h"
8422,"PFC_08",85,"OID30361","Q96AG4","LRRC59",0.0227,"Cardiometabolic_II","B13805","20212097_SS211647","PASS",0.1149,0.9108,"Plate control","PASS","PFC",1.88008775144672,"Baseline","Baseline"
8423,"PFC_16",86,"OID30361","Q96AG4","LRRC59",0.0227,"Cardiometabolic_II","B13805","20212097_SS211647","PASS",0.1149,1.6349,"Plate control","PASS","PFC",3.10566022458052,"1h","1h"
8424,"PFC_24",87,"OID30361","Q96AG4","LRRC59",0.0227,"Cardiometabolic_II","B13805","20212097_SS211647","PASS",0.1149,2.5697,"Plate control","PASS","PFC",5.93685961889824,"24h","24h"
8425,"PFC_01",1,"OID30363","P16410","CTLA4",0.9545,"Cardiometabolic_II","B13805","20212097_SS211647","PASS",0.5725,-0.0225,"Plate control","PASS","PFC",0.984525173337414,"Baseline","Baseline"
8426,"PFC_09",2,"OID30363","P16410","CTLA4",0.9545,"Cardiometabolic_II","B13805","20212097_SS211647","PASS",0.5725,-1.2677,"Plate control","PASS","PFC",0.415321366716971,"Baseline","Baseline"
8427,"PFC_17",3,"OID30363","P16410","CTLA4",0.9545,"Cardiometabolic_II","B13805","20212097_SS211647","PASS",0.5725,0.2555,"Plate control","PASS","PFC",1.19374939336059,"1h","1h"
8428,"PFC_25",4,"OID30363","P16410","CTLA4",0.9545,"Cardiometabolic_II","B13805","20212097_SS211647","PASS",0.5725,0.1361,"Plate control","PASS","PFC",1.09893038698696,"24h","24h"
8429,"PFC_02",13,"OID30363","P16410","CTLA4",0.9545,"Cardiometabolic_II","B13805","20212097_SS211647","PASS",0.5725,0.3272,"Plate control","PASS","PFC",1.25457611364545,"Baseline","Baseline"
8430,"PFC_10",14,"OID30363","P16410","CTLA4",0.9545,"Cardiometabolic_II","B13805","20212097_SS211647","PASS",0.5725,0.6191,"Plate control","PASS","PFC",1.53591672764744,"1h","1h"
8431,"PFC_18",15,"OID30363","P16410","CTLA4",0.9545,"Cardiometabolic_II","B13805","20212097_SS211647","PASS",0.5725,-0.0651,"Plate control","PASS","PFC",0.955879058809957,"1h","1h"
8432,"PFC_26",16,"OID30363","P16410","CTLA4",0.9545,"Cardiometabolic_II","B13805","20212097_SS211647","PASS",0.5725,-0.4825,"Plate control","PASS","PFC",0.715736272321307,"24h","24h"
8433,"PFC_03",25,"OID30363","P16410","CTLA4",0.9545,"Cardiometabolic_II","B13805","20212097_SS211647","PASS",0.5725,0.3585,"Plate control","PASS","PFC",1.28209218646309,"Baseline","Baseline"
8434,"PFC_11",26,"OID30363","P16410","CTLA4",0.9545,"Cardiometabolic_II","B13805","20212097_SS211647","PASS",0.5725,0.2985,"Plate control","PASS","PFC",1.22986503214132,"1h","1h"
8435,"PFC_19",27,"OID30363","P16410","CTLA4",0.9545,"Cardiometabolic_II","B13805","20212097_SS211647","PASS",0.5725,0.0807,"Plate control","PASS","PFC",1.05753103335638,"24h","24h"
8436,"PFC_27",28,"OID30363","P16410","CTLA4",0.9545,"Cardiometabolic_II","B13805","20212097_SS211647","PASS",0.5725,0.0501,"Plate control","PASS","PFC",1.03533668542473,"24h","24h"
8437,"PFC_04",37,"OID30363","P16410","CTLA4",0.9545,"Cardiometabolic_II","B13805","20212097_SS211647","PASS",0.5725,-0.0207,"Plate control","PASS","PFC",0.985754297472065,"Baseline","Baseline"
8438,"PFC_12",38,"OID30363","P16410","CTLA4",0.9545,"Cardiometabolic_II","B13805","20212097_SS211647","PASS",0.5725,0.5628,"Plate control","PASS","PFC",1.47713327523503,"1h","1h"
8439,"PFC_20",39,"OID30363","P16410","CTLA4",0.9545,"Cardiometabolic_II","B13805","20212097_SS211647","PASS",0.5725,0.0679,"Plate control","PASS","PFC",1.04818981801714,"24h","24h"
8440,"PFC_05",49,"OID30363","P16410","CTLA4",0.9545,"Cardiometabolic_II","B13805","20212097_SS211647","PASS",0.5725,-0.3247,"Plate control","PASS","PFC",0.79846440510009,"Baseline","Baseline"
8441,"PFC_13",50,"OID30363","P16410","CTLA4",0.9545,"Cardiometabolic_II","B13805","20212097_SS211647","PASS",0.5725,0.1991,"Plate control","PASS","PFC",1.1479819831448,"1h","1h"
8442,"PFC_21",51,"OID30363","P16410","CTLA4",0.9545,"Cardiometabolic_II","B13805","20212097_SS211647","PASS",0.5725,0.8081,"Plate control","PASS","PFC",1.75090401860088,"24h","24h"
8443,"PFC_06",61,"OID30363","P16410","CTLA4",0.9545,"Cardiometabolic_II","B13805","20212097_SS211647","PASS",0.5725,0.2806,"Plate control","PASS","PFC",1.21469995887151,"Baseline","Baseline"
8444,"PFC_14",62,"OID30363","P16410","CTLA4",0.9545,"Cardiometabolic_II","B13805","20212097_SS211647","PASS",0.5725,-0.1663,"Plate control","PASS","PFC",0.891125172358942,"1h","1h"
8445,"PFC_22",63,"OID30363","P16410","CTLA4",0.9545,"Cardiometabolic_II","B13805","20212097_SS211647","PASS",0.5725,-0.3354,"Plate control","PASS","PFC",0.792564360643477,"24h","24h"
8446,"PFC_07",73,"OID30363","P16410","CTLA4",0.9545,"Cardiometabolic_II","B13805","20212097_SS211647","PASS",0.5725,0.0919,"Plate control","PASS","PFC",1.06577285976233,"Baseline","Baseline"
8447,"PFC_15",74,"OID30363","P16410","CTLA4",0.9545,"Cardiometabolic_II","B13805","20212097_SS211647","PASS",0.5725,-0.8608,"Plate control","PASS","PFC",0.550647129659106,"1h","1h"
8448,"PFC_23",75,"OID30363","P16410","CTLA4",0.9545,"Cardiometabolic_II","B13805","20212097_SS211647","PASS",0.5725,0.2216,"Plate control","PASS","PFC",1.16602603390352,"24h","24h"
8449,"PFC_08",85,"OID30363","P16410","CTLA4",0.9545,"Cardiometabolic_II","B13805","20212097_SS211647","PASS",0.5725,-0.2674,"Plate control","PASS","PFC",0.830815478694623,"Baseline","Baseline"
8450,"PFC_16",86,"OID30363","P16410","CTLA4",0.9545,"Cardiometabolic_II","B13805","20212097_SS211647","PASS",0.5725,-0.1557,"Plate control","PASS","PFC",0.897696702041463,"1h","1h"
8451,"PFC_24",87,"OID30363","P16410","CTLA4",0.9545,"Cardiometabolic_II","B13805","20212097_SS211647","PASS",0.5725,-0.1878,"Plate control","PASS","PFC",0.877943497985761,"24h","24h"
8452,"PFC_01",1,"OID30362","P40199","CEACAM6",0,"Cardiometabolic_II","B13805","20212097_SS211647","PASS",-1.198,-0.1771,"Plate control","PASS","PFC",0.884479125785508,"Baseline","Baseline"
8453,"PFC_09",2,"OID30362","P40199","CEACAM6",0,"Cardiometabolic_II","B13805","20212097_SS211647","PASS",-1.198,0.6251,"Plate control","PASS","PFC",1.54231772702137,"Baseline","Baseline"
8454,"PFC_17",3,"OID30362","P40199","CEACAM6",0,"Cardiometabolic_II","B13805","20212097_SS211647","PASS",-1.198,-0.9511,"Plate control","PASS","PFC",0.51723793731509,"1h","1h"
8455,"PFC_25",4,"OID30362","P40199","CEACAM6",0,"Cardiometabolic_II","B13805","20212097_SS211647","PASS",-1.198,0.9146,"Plate control","PASS","PFC",1.88504635354961,"24h","24h"
8456,"PFC_02",13,"OID30362","P40199","CEACAM6",0,"Cardiometabolic_II","B13805","20212097_SS211647","PASS",-1.198,0.321,"Plate control","PASS","PFC",1.24919612565338,"Baseline","Baseline"
8457,"PFC_10",14,"OID30362","P40199","CEACAM6",0,"Cardiometabolic_II","B13805","20212097_SS211647","PASS",-1.198,-0.1377,"Plate control","PASS","PFC",0.908967111194767,"1h","1h"
8458,"PFC_18",15,"OID30362","P40199","CEACAM6",0,"Cardiometabolic_II","B13805","20212097_SS211647","PASS",-1.198,0.8543,"Plate control","PASS","PFC",1.80788135277653,"1h","1h"
8459,"PFC_26",16,"OID30362","P40199","CEACAM6",0,"Cardiometabolic_II","B13805","20212097_SS211647","PASS",-1.198,-0.6046,"Plate control","PASS","PFC",0.657653695148966,"24h","24h"
8460,"PFC_03",25,"OID30362","P40199","CEACAM6",0,"Cardiometabolic_II","B13805","20212097_SS211647","PASS",-1.198,-0.2191,"Plate control","PASS","PFC",0.85910120452575,"Baseline","Baseline"
8461,"PFC_11",26,"OID30362","P40199","CEACAM6",0,"Cardiometabolic_II","B13805","20212097_SS211647","PASS",-1.198,0.2901,"Plate control","PASS","PFC",1.22272502759537,"1h","1h"
8462,"PFC_19",27,"OID30362","P40199","CEACAM6",0,"Cardiometabolic_II","B13805","20212097_SS211647","PASS",-1.198,-0.3649,"Plate control","PASS","PFC",0.776522697587517,"24h","24h"
8463,"PFC_27",28,"OID30362","P40199","CEACAM6",0,"Cardiometabolic_II","B13805","20212097_SS211647","PASS",-1.198,0.8115,"Plate control","PASS","PFC",1.75503524094084,"24h","24h"
8464,"PFC_04",37,"OID30362","P40199","CEACAM6",0,"Cardiometabolic_II","B13805","20212097_SS211647","PASS",-1.198,0.1124,"Plate control","PASS","PFC",1.08102508428837,"Baseline","Baseline"
8465,"PFC_12",38,"OID30362","P40199","CEACAM6",0,"Cardiometabolic_II","B13805","20212097_SS211647","PASS",-1.198,-0.0346,"Plate control","PASS","PFC",0.976302411759932,"1h","1h"
8466,"PFC_20",39,"OID30362","P40199","CEACAM6",0,"Cardiometabolic_II","B13805","20212097_SS211647","PASS",-1.198,0.3963,"Plate control","PASS","PFC",1.31612817773648,"24h","24h"
8467,"PFC_05",49,"OID30362","P40199","CEACAM6",0,"Cardiometabolic_II","B13805","20212097_SS211647","PASS",-1.198,0.7376,"Plate control","PASS","PFC",1.66739972218891,"Baseline","Baseline"
8468,"PFC_13",50,"OID30362","P40199","CEACAM6",0,"Cardiometabolic_II","B13805","20212097_SS211647","PASS",-1.198,0.3235,"Plate control","PASS","PFC",1.25136269423101,"1h","1h"
8469,"PFC_21",51,"OID30362","P40199","CEACAM6",0,"Cardiometabolic_II","B13805","20212097_SS211647","PASS",-1.198,0.1278,"Plate control","PASS","PFC",1.09262625843927,"24h","24h"
8470,"PFC_06",61,"OID30362","P40199","CEACAM6",0,"Cardiometabolic_II","B13805","20212097_SS211647","PASS",-1.198,-0.0075,"Plate control","PASS","PFC",0.994814885501422,"Baseline","Baseline"
8471,"PFC_14",62,"OID30362","P40199","CEACAM6",0,"Cardiometabolic_II","B13805","20212097_SS211647","PASS",-1.198,0.6725,"Plate control","PASS","PFC",1.59383247710592,"1h","1h"
8472,"PFC_22",63,"OID30362","P40199","CEACAM6",0,"Cardiometabolic_II","B13805","20212097_SS211647","PASS",-1.198,-0.1338,"Plate control","PASS","PFC",0.911427622576958,"24h","24h"
8473,"PFC_07",73,"OID30362","P40199","CEACAM6",0,"Cardiometabolic_II","B13805","20212097_SS211647","PASS",-1.198,0.795,"Plate control","PASS","PFC",1.73507737430414,"Baseline","Baseline"
8474,"PFC_15",74,"OID30362","P40199","CEACAM6",0,"Cardiometabolic_II","B13805","20212097_SS211647","PASS",-1.198,0.313,"Plate control","PASS","PFC",1.24228828178444,"1h","1h"
8475,"PFC_23",75,"OID30362","P40199","CEACAM6",0,"Cardiometabolic_II","B13805","20212097_SS211647","PASS",-1.198,0.4747,"Plate control","PASS","PFC",1.38962922452223,"24h","24h"
8476,"PFC_08",85,"OID30362","P40199","CEACAM6",0,"Cardiometabolic_II","B13805","20212097_SS211647","PASS",-1.198,-0.644,"Plate control","PASS","PFC",0.639936206922154,"Baseline","Baseline"
8477,"PFC_16",86,"OID30362","P40199","CEACAM6",0,"Cardiometabolic_II","B13805","20212097_SS211647","PASS",-1.198,0.5153,"Plate control","PASS","PFC",1.42929132161634,"1h","1h"
8478,"PFC_24",87,"OID30362","P40199","CEACAM6",0,"Cardiometabolic_II","B13805","20212097_SS211647","PASS",-1.198,0.1443,"Plate control","PASS","PFC",1.1051942795965,"24h","24h"
8479,"PFC_01",1,"OID30364","P07288","KLK3",0.0455,"Cardiometabolic_II","B13805","20212097_SS211647","PASS",-1.2858,1.3523,"Plate control","PASS","PFC",2.55318840306372,"Baseline","Baseline"
8480,"PFC_09",2,"OID30364","P07288","KLK3",0.0455,"Cardiometabolic_II","B13805","20212097_SS211647","PASS",-1.2858,1.58,"Plate control","PASS","PFC",2.98969849726988,"Baseline","Baseline"
8481,"PFC_17",3,"OID30364","P07288","KLK3",0.0455,"Cardiometabolic_II","B13805","20212097_SS211647","PASS",-1.2858,-0.8664,"Plate control","PASS","PFC",0.548513867368596,"1h","1h"
8482,"PFC_25",4,"OID30364","P07288","KLK3",0.0455,"Cardiometabolic_II","B13805","20212097_SS211647","PASS",-1.2858,-1.1342,"Plate control","PASS","PFC",0.455587478105411,"24h","24h"
8483,"PFC_02",13,"OID30364","P07288","KLK3",0.0455,"Cardiometabolic_II","B13805","20212097_SS211647","PASS",-1.2858,0.8027,"Plate control","PASS","PFC",1.74436264381058,"Baseline","Baseline"
8484,"PFC_10",14,"OID30364","P07288","KLK3",0.0455,"Cardiometabolic_II","B13805","20212097_SS211647","PASS",-1.2858,1.5834,"Plate control","PASS","PFC",2.99675262992961,"1h","1h"
8485,"PFC_18",15,"OID30364","P07288","KLK3",0.0455,"Cardiometabolic_II","B13805","20212097_SS211647","PASS",-1.2858,1.2686,"Plate control","PASS","PFC",2.40927654407999,"1h","1h"
8486,"PFC_26",16,"OID30364","P07288","KLK3",0.0455,"Cardiometabolic_II","B13805","20212097_SS211647","PASS",-1.2858,0.4694,"Plate control","PASS","PFC",1.38453353704717,"24h","24h"
8487,"PFC_03",25,"OID30364","P07288","KLK3",0.0455,"Cardiometabolic_II","B13805","20212097_SS211647","PASS",-1.2858,1.7742,"Plate control","PASS","PFC",3.42048285831166,"Baseline","Baseline"
8488,"PFC_11",26,"OID30364","P07288","KLK3",0.0455,"Cardiometabolic_II","B13805","20212097_SS211647","PASS",-1.2858,0.7519,"Plate control","PASS","PFC",1.68400917655003,"1h","1h"
8489,"PFC_19",27,"OID30364","P07288","KLK3",0.0455,"Cardiometabolic_II","B13805","20212097_SS211647","PASS",-1.2858,1.6346,"Plate control","PASS","PFC",3.10501448783291,"24h","24h"
8490,"PFC_27",28,"OID30364","P07288","KLK3",0.0455,"Cardiometabolic_II","B13805","20212097_SS211647","PASS",-1.2858,1.2323,"Plate control","PASS","PFC",2.34941243821788,"24h","24h"
8491,"PFC_04",37,"OID30364","P07288","KLK3",0.0455,"Cardiometabolic_II","B13805","20212097_SS211647","PASS",-1.2858,3.2003,"Plate control","PASS","PFC",9.19149795953506,"Baseline","Baseline"
8492,"PFC_12",38,"OID30364","P07288","KLK3",0.0455,"Cardiometabolic_II","B13805","20212097_SS211647","PASS",-1.2858,1.875,"Plate control","PASS","PFC",3.66801617281868,"1h","1h"
8493,"PFC_20",39,"OID30364","P07288","KLK3",0.0455,"Cardiometabolic_II","B13805","20212097_SS211647","PASS",-1.2858,0.8057,"Plate control","PASS","PFC",1.74799371795066,"24h","24h"
8494,"PFC_05",49,"OID30364","P07288","KLK3",0.0455,"Cardiometabolic_II","B13805","20212097_SS211647","PASS",-1.2858,1.676,"Plate control","PASS","PFC",3.19540766579659,"Baseline","Baseline"
8495,"PFC_13",50,"OID30364","P07288","KLK3",0.0455,"Cardiometabolic_II","B13805","20212097_SS211647","PASS",-1.2858,3.0642,"Plate control","PASS","PFC",8.36404022345426,"1h","1h"
8496,"PFC_21",51,"OID30364","P07288","KLK3",0.0455,"Cardiometabolic_II","B13805","20212097_SS211647","PASS",-1.2858,1.8329,"Plate control","PASS","PFC",3.56252465847613,"24h","24h"
8497,"PFC_06",61,"OID30364","P07288","KLK3",0.0455,"Cardiometabolic_II","B13805","20212097_SS211647","PASS",-1.2858,1.2612,"Plate control","PASS","PFC",2.39695030751698,"Baseline","Baseline"
8498,"PFC_14",62,"OID30364","P07288","KLK3",0.0455,"Cardiometabolic_II","B13805","20212097_SS211647","PASS",-1.2858,1.7566,"Plate control","PASS","PFC",3.3790085476625,"1h","1h"
8499,"PFC_22",63,"OID30364","P07288","KLK3",0.0455,"Cardiometabolic_II","B13805","20212097_SS211647","PASS",-1.2858,2.9224,"Plate control","PASS","PFC",7.58106219018664,"24h","24h"
8500,"PFC_07",73,"OID30364","P07288","KLK3",0.0455,"Cardiometabolic_II","B13805","20212097_SS211647","PASS",-1.2858,-1.0387,"Plate control","PASS","PFC",0.486765896678445,"Baseline","Baseline"
8501,"PFC_15",74,"OID30364","P07288","KLK3",0.0455,"Cardiometabolic_II","B13805","20212097_SS211647","PASS",-1.2858,1.1874,"Plate control","PASS","PFC",2.27741940535831,"1h","1h"
8502,"PFC_23",75,"OID30364","P07288","KLK3",0.0455,"Cardiometabolic_II","B13805","20212097_SS211647","PASS",-1.2858,1.5593,"Plate control","PASS","PFC",2.94710814182956,"24h","24h"
8503,"PFC_08",85,"OID30364","P07288","KLK3",0.0455,"Cardiometabolic_II","B13805","20212097_SS211647","PASS",-1.2858,-1.3936,"Plate control","PASS","PFC",0.380613859151166,"Baseline","Baseline"
8504,"PFC_16",86,"OID30364","P07288","KLK3",0.0455,"Cardiometabolic_II","B13805","20212097_SS211647","PASS",-1.2858,-0.9234,"Plate control","PASS","PFC",0.527264949380846,"1h","1h"
8505,"PFC_24",87,"OID30364","P07288","KLK3",0.0455,"Cardiometabolic_II","B13805","20212097_SS211647","PASS",-1.2858,1.2296,"Plate control","PASS","PFC",2.3450196308235,"24h","24h"
8506,"PFC_01",1,"OID30365","Q6H9L7","ISM2",0.7727,"Cardiometabolic_II","B13805","20212097_SS211647","PASS",0.2879,0.2197,"Plate control","PASS","PFC",1.16449141210823,"Baseline","Baseline"
8507,"PFC_09",2,"OID30365","Q6H9L7","ISM2",0.7727,"Cardiometabolic_II","B13805","20212097_SS211647","PASS",0.2879,0.0816,"Plate control","PASS","PFC",1.05819096136594,"Baseline","Baseline"
8508,"PFC_17",3,"OID30365","Q6H9L7","ISM2",0.7727,"Cardiometabolic_II","B13805","20212097_SS211647","PASS",0.2879,0.3296,"Plate control","PASS","PFC",1.25666490472164,"1h","1h"
8509,"PFC_25",4,"OID30365","Q6H9L7","ISM2",0.7727,"Cardiometabolic_II","B13805","20212097_SS211647","PASS",0.2879,0.1242,"Plate control","PASS","PFC",1.08990319440612,"24h","24h"
8510,"PFC_02",13,"OID30365","Q6H9L7","ISM2",0.7727,"Cardiometabolic_II","B13805","20212097_SS211647","PASS",0.2879,0.3073,"Plate control","PASS","PFC",1.23738975990318,"Baseline","Baseline"
8511,"PFC_10",14,"OID30365","Q6H9L7","ISM2",0.7727,"Cardiometabolic_II","B13805","20212097_SS211647","PASS",0.2879,0.2647,"Plate control","PASS","PFC",1.20138620231295,"1h","1h"
8512,"PFC_18",15,"OID30365","Q6H9L7","ISM2",0.7727,"Cardiometabolic_II","B13805","20212097_SS211647","PASS",0.2879,0.183,"Plate control","PASS","PFC",1.13524210214606,"1h","1h"

Supplemental table 1_Olink proximity extension assay (PEA) data

,"SampleID","Index","OlinkID","UniProt","Assay","MissingFreq","Panel","Panel_Lot_Nr","PlateID","QC_Warning","LOD","NPX","Normalization","Assay_Warning","condition","Value","group","alt_group"
8513,"PFC_26",16,"OID30365","Q6H9L7","ISM2",0.7727,"Cardiometabolic_II","B13805","20212097_SS211647","PASS",0.2879,0.0677,"Plate control","PASS","PFC",1.04804451812538,"24h","24h"
8514,"PFC_03",25,"OID30365","Q6H9L7","ISM2",0.7727,"Cardiometabolic_II","B13805","20212097_SS211647","PASS",0.2879,-0.0287,"Plate control","PASS","PFC",0.980303242477391,"Baseline","Baseline"
8515,"PFC_11",26,"OID30365","Q6H9L7","ISM2",0.7727,"Cardiometabolic_II","B13805","20212097_SS211647","PASS",0.2879,-0.2367,"Plate control","PASS","PFC",0.848684362310161,"1h","1h"
8516,"PFC_19",27,"OID30365","Q6H9L7","ISM2",0.7727,"Cardiometabolic_II","B13805","20212097_SS211647","PASS",0.2879,-0.0827,"Plate control","PASS","PFC",0.944288758062864,"24h","24h"
8517,"PFC_27",28,"OID30365","Q6H9L7","ISM2",0.7727,"Cardiometabolic_II","B13805","20212097_SS211647","PASS",0.2879,-0.2254,"Plate control","PASS","PFC",0.855357837253519,"24h","24h"
8518,"PFC_04",37,"OID30365","Q6H9L7","ISM2",0.7727,"Cardiometabolic_II","B13805","20212097_SS211647","PASS",0.2879,0.4077,"Plate control","PASS","PFC",1.3265692595916,"Baseline","Baseline"
8519,"PFC_12",38,"OID30365","Q6H9L7","ISM2",0.7727,"Cardiometabolic_II","B13805","20212097_SS211647","PASS",0.2879,0.0448,"Plate control","PASS","PFC",1.03154016755701,"1h","1h"
8520,"PFC_20",39,"OID30365","Q6H9L7","ISM2",0.7727,"Cardiometabolic_II","B13805","20212097_SS211647","PASS",0.2879,0.0474,"Plate control","PASS","PFC",1.03340086752684,"24h","24h"
8521,"PFC_05",49,"OID30365","Q6H9L7","ISM2",0.7727,"Cardiometabolic_II","B13805","20212097_SS211647","PASS",0.2879,0.0999,"Plate control","PASS","PFC",1.07169917543554,"Baseline","Baseline"
8522,"PFC_13",50,"OID30365","Q6H9L7","ISM2",0.7727,"Cardiometabolic_II","B13805","20212097_SS211647","PASS",0.2879,0.2027,"Plate control","PASS","PFC",1.15085015388239,"1h","1h"
8523,"PFC_21",51,"OID30365","Q6H9L7","ISM2",0.7727,"Cardiometabolic_II","B13805","20212097_SS211647","PASS",0.2879,-0.0694,"Plate control","PASS","PFC",0.953034271446426,"24h","24h"
8524,"PFC_06",61,"OID30365","Q6H9L7","ISM2",0.7727,"Cardiometabolic_II","B13805","20212097_SS211647","PASS",0.2879,0.1852,"Plate control","PASS","PFC",1.13697458045622,"Baseline","Baseline"
8525,"PFC_14",62,"OID30365","Q6H9L7","ISM2",0.7727,"Cardiometabolic_II","B13805","20212097_SS211647","PASS",0.2879,0.5108,"Plate control","PASS","PFC",1.42484007568054,"1h","1h"
8526,"PFC_22",63,"OID30365","Q6H9L7","ISM2",0.7727,"Cardiometabolic_II","B13805","20212097_SS211647","PASS",0.2879,0.1038,"Plate control","PASS","PFC",1.07460019131056,"24h","24h"
8527,"PFC_07",73,"OID30365","Q6H9L7","ISM2",0.7727,"Cardiometabolic_II","B13805","20212097_SS211647","PASS",0.2879,0.2891,"Plate control","PASS","PFC",1.221877792853,"Baseline","Baseline"
8528,"PFC_15",74,"OID30365","Q6H9L7","ISM2",0.7727,"Cardiometabolic_II","B13805","20212097_SS211647","PASS",0.2879,0.0737,"Plate control","PASS","PFC",1.0524122889710 4,"1h","1h"
8529,"PFC_23",75,"OID30365","Q6H9L7","ISM2",0.7727,"Cardiometabolic_II","B13805","20212097_SS211647","PASS",0.2879,0.2316,"Plate control","PASS","PFC",1.17413638634978,"24h","24h"
8530,"PFC_08",85,"OID30365","Q6H9L7","ISM2",0.7727,"Cardiometabolic_II","B13805","20212097_SS211647","PASS",0.2879,0.0044,"Plate control","PASS","PFC",1.00305450311131,"Baseline","Baseline"
8531,"PFC_16",86,"OID30365","Q6H9L7","ISM2",0.7727,"Cardiometabolic_II","B13805","20212097_SS211647","PASS",0.2879,0.5595,"Plate control","PASS","PFC",1.47375836304483,"1h","1h"
8532,"PFC_24",87,"OID30365","Q6H9L7","ISM2",0.7727,"Cardiometabolic_II","B13805","20212097_SS211647","PASS",0.2879,0.2281,"Plate control","PASS","PFC",1.17129136613593,"24h","24h"
8533,"PFC_01",1,"OID30366","Q9Y303","AMDHD2",0.5909,"Cardiometabolic_II","B13805","20212097_SS211647","PASS",0.2051,0.3408,"Plate control","PASS","PFC",1.26645867310147,"Baseline","Baseline"
8534,"PFC_09",2,"OID30366","Q9Y303","AMDHD2",0.5909,"Cardiometabolic_II","B13805","20212097_SS211647","PASS",0.2051,0.0818,"Plate control","PASS","PFC",1.05833766795091,"Baseline","Baseline"
8535,"PFC_17",3,"OID30366","Q9Y303","AMDHD2",0.5909,"Cardiometabolic_II","B13805","20212097_SS211647","PASS",0.2051,0.1586,"Plate control","PASS","PFC",1.11620344176851,"1h","1h"
8536,"PFC_25",4,"OID30366","Q9Y303","AMDHD2",0.5909,"Cardiometabolic_II","B13805","20212097_SS211647","PASS",0.2051,0.2733,"Plate control","PASS","PFC",1.20856913214796,"24h","24h"
8537,"PFC_02",13,"OID30366","Q9Y303","AMDHD2",0.5909,"Cardiometabolic_II","B13805","20212097_SS211647","PASS",0.2051,-0.1419,"Plate control","PASS","PFC",0.906324757735202,"Baseline","Baseline"
8538,"PFC_10",14,"OID30366","Q9Y303","AMDHD2",0.5909,"Cardiometabolic_II","B13805","20212097_SS211647","PASS",0.2051,-0.0835,"Plate control","PASS","PFC",0.943765278343445,"1h","1h"
8539,"PFC_18",15,"OID30366","Q9Y303","AMDHD2",0.5909,"Cardiometabolic_II","B13805","20212097_SS211647","PASS",0.2051,-0.5089,"Plate control","PASS","PFC",0.70275805989197 1,"1h","1h"
8540,"PFC_26",16,"OID30366","Q9Y303","AMDHD2",0.5909,"Cardiometabolic_II","B13805","20212097_SS211647","PASS",0.2051,0.0366,"Plate control","PASS","PFC",1.02569372322474,"24h","24h"
8541,"PFC_03",25,"OID30366","Q9Y303","AMDHD2",0.5909,"Cardiometabolic_II","B13805","20212097_SS211647","PASS",0.2051,0.0713,"Plate control","PASS","PFC",1.05066300052555,"Baseline","Baseline"
8542,"PFC_11",26,"OID30366","Q9Y303","AMDHD2",0.5909,"Cardiometabolic_II","B13805","20212097_SS211647","PASS",0.2051,-0.2455,"Plate control","PASS","PFC",0.843523402530895,"1h","1h"
8543,"PFC_19",27,"OID30366","Q9Y303","AMDHD2",0.5909,"Cardiometabolic_II","B13805","20212097_SS211647","PASS",0.2051,0.0312,"Plate control","PASS","PFC",1.02186173301146,"24h","24h"
8544,"PFC_27",28,"OID30366","Q9Y303","AMDHD2",0.5909,"Cardiometabolic_II","B13805","20212097_SS211647","PASS",0.2051,0.6982,"Plate control","PASS","PFC",1.62247921894353,"24h","24h"
8545,"PFC_04",37,"OID30366","Q9Y303","AMDHD2",0.5909,"Cardiometabolic_II","B13805","20212097_SS211647","PASS",0.2051,0.2248,"Plate control","PASS","PFC",1.16861523286067,"Baseline","Baseline"
8546,"PFC_12",38,"OID30366","Q9Y303","AMDHD2",0.5909,"Cardiometabolic_II","B13805","20212097_SS211647","PASS",0.2051,0.6174,"Plate control","PASS","PFC",1.53410794575211,"1h","1h"
8547,"PFC_20",39,"OID30366","Q9Y303","AMDHD2",0.5909,"Cardiometabolic_II","B13805","20212097_SS211647","PASS",0.2051,-0.0454,"Plate control","PASS","PFC",0.969021109985978,"24h","24h"
8548,"PFC_05",49,"OID30366","Q9Y303","AMDHD2",0.5909,"Cardiometabolic_II","B13805","20212097_SS211647","PASS",0.2051,-0.0244,"Plate control","PASS","PFC",0.983229427149003,"Baseline","Baseline"
8549,"PFC_13",50,"OID30366","Q9Y303","AMDHD2",0.5909,"Cardiometabolic_II","B13805","20212097_SS211647","PASS",0.2051,0.1012,"Plate control","PASS","PFC",1.07266530949811,"1h","1h"
8550,"PFC_21",51,"OID30366","Q9Y303","AMDHD2",0.5909,"Cardiometabolic_II","B13805","20212097_SS211647","PASS",0.2051,-0.0362,"Plate control","PASS","PFC",0.975220257921818,"24h","24h"
8551,"PFC_06",61,"OID30366","Q9Y303","AMDHD2",0.5909,"Cardiometabolic_II","B13805","20212097_SS211647","PASS",0.2051,-0.0728,"Plate control","PASS","PFC",0.950790899700319,"Baseline","Baseline"
8552,"PFC_14",62,"OID30366","Q9Y303","AMDHD2",0.5909,"Cardiometabolic_II","B13805","20212097_SS211647","PASS",0.2051,-0.2132,"Plate control","PASS","PFC",0.862621751486302,"1h","1h"
8553,"PFC_22",63,"OID30366","Q9Y303","AMDHD2",0.5909,"Cardiometabolic_II","B13805","20212097_SS211647","PASS",0.2051,0.1649,"Plate control","PASS","PFC",1.12108836741119,"24h","24h"
8554,"PFC_07",73,"OID30366","Q9Y303","AMDHD2",0.5909,"Cardiometabolic_II","B13805","20212097_SS211647","PASS",0.2051,0.3182,"Plate control","PASS","PFC",1.24677402187494,"Baseline","Baseline"
8555,"PFC_15",74,"OID30366","Q9Y303","AMDHD2",0.5909,"Cardiometabolic_II","B13805","20212097_SS211647","PASS",0.2051,0.1753,"Plate control","PASS","PFC",1.12919919076471,"1h","1h"
8556,"PFC_23",75,"OID30366","Q9Y303","AMDHD2",0.5909,"Cardiometabolic_II","B13805","20212097_SS211647","PASS",0.2051,-0.1335,"Plate control","PASS","PFC",0.911617168329803,"24h","24h"
8557,"PFC_08",85,"OID30366","Q9Y303","AMDHD2",0.5909,"Cardiometabolic_II","B13805","20212097_SS211647","PASS",0.2051,0.2105,"Plate control","PASS","PFC",1.15708913095683,"Baseline","Baseline"
8558,"PFC_16",86,"OID30366","Q9Y303","AMDHD2",0.5909,"Cardiometabolic_II","B13805","20212097_SS211647","PASS",0.2051,0.2404,"Plate control","PASS","PFC",1.18132014752006,"1h","1h"
8559,"PFC_24",87,"OID30366","Q9Y303","AMDHD2",0.5909,"Cardiometabolic_II","B13805","20212097_SS211647","PASS",0.2051,0.1119,"Plate control","PASS","PFC",1.08065049445894,"24h","24h"
8560,"PFC_01",1,"OID30367","P13796","LCP1",0,"Cardiometabolic_II","B13805","20212097_SS211647","PASS",-3.2548,0.4684,"Plate control","PASS","PFC",1.3835741840544,"Baseline","Baseline"
8561,"PFC_09",2,"OID30367","P13796","LCP1",0,"Cardiometabolic_II","B13805","20212097_SS211647","PASS",-3.2548,-0.0762,"Plate control","PASS","PFC",0.948552808684341,"Baseline","Baseline"
8562,"PFC_17",3,"OID30367","P13796","LCP1",0,"Cardiometabolic_II","B13805","20212097_SS211647","PASS",-3.2548,1.2194,"Plate control","PASS","PFC",2.32849857620302,"1h","1h"
8563,"PFC_25",4,"OID30367","P13796","LCP1",0,"Cardiometabolic_II","B13805","20212097_SS211647","PASS",-3.2548,0.0412,"Plate control","PASS","PFC",1.02896934344646,"24h","24h"
8564,"PFC_02",13,"OID30367","P13796","LCP1",0,"Cardiometabolic_II","B13805","20212097_SS211647","PASS",-3.2548,0.181,"Plate control","PASS","PFC",1.13366941277842,"Baseline","Baseline"
8565,"PFC_10",14,"OID30367","P13796","LCP1",0,"Cardiometabolic_II","B13805","20212097_SS211647","PASS",-3.2548,1.1423,"Plate control","PASS","PFC",2.20732643299206,"1h","1h"
8566,"PFC_18",15,"OID30367","P13796","LCP1",0,"Cardiometabolic_II","B13805","20212097_SS211647","PASS",-3.2548,0.1397,"Plate control","PASS","PFC",1.10167600497128,"1h","1h"
8567,"PFC_26",16,"OID30367","P13796","LCP1",0,"Cardiometabolic_II","B13805","20212097_SS211647","PASS",-3.2548,1.2709,"Plate control","PASS","PFC",2.41312056887257,"24h","24h"
8568,"PFC_03",25,"OID30367","P13796","LCP1",0,"Cardiometabolic_II","B13805","20212097_SS211647","PASS",-3.2548,-0.2923,"Plate control","PASS","PFC",0.816599166430652,"Baseline","Baseline"
8569,"PFC_11",26,"OID30367","P13796","LCP1",0,"Cardiometabolic_II","B13805","20212097_SS211647","PASS",-3.2548,-0.0541,"Plate control","PASS","PFC",0.963195128107507,"1h","1h"
8570,"PFC_19",27,"OID30367","P13796","LCP1",0,"Cardiometabolic_II","B13805","20212097_SS211647","PASS",-3.2548,0.8376,"Plate control","PASS","PFC",1.78707477368246,"24h","24h"
8571,"PFC_27",28,"OID30367","P13796","LCP1",0,"Cardiometabolic_II","B13805","20212097_SS211647","PASS",-3.2548,0.1872,"Plate control","PASS","PFC",1.1385518549366,"24h","24h"
8572,"PFC_04",37,"OID30367","P13796","LCP1",0,"Cardiometabolic_II","B13805","20212097_SS211647","PASS",-3.2548,0.2059,"Plate control","PASS","PFC",1.15340565430144,"Baseline","Baseline"
8573,"PFC_12",38,"OID30367","P13796","LCP1",0,"Cardiometabolic_II","B13805","20212097_SS211647","PASS",-3.2548,0.211,"Plate control","PASS","PFC",1.15749021699012,"1h","1h"
8574,"PFC_20",39,"OID30367","P13796","LCP1",0,"Cardiometabolic_II","B13805","20212097_SS211647","PASS",-3.2548,-0.1059,"Plate control","PASS","PFC",0.92922508099322,"24h","24h"
8575,"PFC_05",49,"OID30367","P13796","LCP1",0,"Cardiometabolic_II","B13805","20212097_SS211647","PASS",-3.2548,1.1364,"Plate control","PASS","PFC",2.19831785379543,"Baseline","Baseline"
8576,"PFC_13",50,"OID30367","P13796","LCP1",0,"Cardiometabolic_II","B13805","20212097_SS211647","PASS",-3.2548,0.7789,"Plate control","PASS","PFC",1.7158221250086,"1h","1h"
8577,"PFC_21",51,"OID30367","P13796","LCP1",0,"Cardiometabolic_II","B13805","20212097_SS211647","PASS",-3.2548,0.1102,"Plate control","PASS","PFC",1.07937785967714,"24h","24h"
8578,"PFC_06",61,"OID30367","P13796","LCP1",0,"Cardiometabolic_II","B13805","20212097_SS211647","PASS",-3.2548,-0.459,"Plate control","PASS","PFC",0.727490341817379,"Baseline","Baseline"
8579,"PFC_14",62,"OID30367","P13796","LCP1",0,"Cardiometabolic_II","B13805","20212097_SS211647","PASS",-3.2548,1.2267,"Plate control","PASS","PFC",2.34031057844353,"1h","1h"
8580,"PFC_22",63,"OID30367","P13796","LCP1",0,"Cardiometabolic_II","B13805","20212097_SS211647","PASS",-3.2548,0.1487,"Plate control","PASS","PFC",1.10857009896213,"24h","24h"
8581,"PFC_07",73,"OID30367","P13796","LCP1",0,"Cardiometabolic_II","B13805","20212097_SS211647","PASS",-3.2548,-0.1145,"Plate control","PASS","PFC",0.923702386113659,"Baseline","Baseline"
8582,"PFC_15",74,"OID30367","P13796","LCP1",0,"Cardiometabolic_II","B13805","20212097_SS211647","PASS",-3.2548,0.2329,"Plate control","PASS","PFC",1.17519486729569,"1h","1h"
8583,"PFC_23",75,"OID30367","P13796","LCP1",0,"Cardiometabolic_II","B13805","20212097_SS211647","PASS",-3.2548,1.0901,"Plate control","PASS","PFC",2.12888792305884,"24h","24h"
8584,"PFC_08",85,"OID30367","P13796","LCP1",0,"Cardiometabolic_II","B13805","20212097_SS211647","PASS",-3.2548,1.289,"Plate control","PASS","PFC",2.443586203347,"Baseline","Baseline"
8585,"PFC_16",86,"OID30367","P13796","LCP1",0,"Cardiometabolic_II","B13805","20212097_SS211647","PASS",-3.2548,-0.2706,"Plate control","PASS","PFC",0.828974713222355,"1h","1h"
8586,"PFC_24",87,"OID30367","P13796","LCP1",0,"Cardiometabolic_II","B13805","20212097_SS211647","PASS",-3.2548,-0.0795,"Plate control","PASS","PFC",0.946385582144429,"24h","24h"
8587,"PFC_01",1,"OID30369","P02730","SLC4A1",0.0682,"Cardiometabolic_II","B13805","20212097_SS211647","PASS",-0.869,2.9161,"Plate control","PASS","PFC",7.54802917854532,"Baseline","Baseline"
8588,"PFC_09",2,"OID30369","P02730","SLC4A1",0.0682,"Cardiometabolic_II","B13805","20212097_SS211647","PASS",-0.869,2.7415,"Plate control","PASS","PFC",6.68765303370578,"Baseline","Baseline"
8589,"PFC_17",3,"OID30369","P02730","SLC4A1",0.0682,"Cardiometabolic_II","B13805","20212097_SS211647","PASS",-0.869,1.0508,"Plate control","PASS","PFC",2.07167831161609,"1h","1h"
8590,"PFC_25",4,"OID30369","P02730","SLC4A1",0.0682,"Cardiometabolic_II","B13805","20212097_SS211647","PASS",-0.869,-0.033,"Plate control","PASS","PFC",0.977385766410805,"24h","24h"
8591,"PFC_02",13,"OID30369","P02730","SLC4A1",0.0682,"Cardiometabolic_II","B13805","20212097_SS211647","PASS",-0.869,1.8728,"Plate control","PASS","PFC",3.66242699028993,"Baseline","Baseline"
8592,"PFC_10",14,"OID30369","P02730","SLC4A1",0.0682,"Cardiometabolic_II","B13805","20212097_SS211647","PASS",-0.869,1.4173,"Plate control","PASS","PFC",2.6708519368979,"1h","1h"
8593,"PFC_18",15,"OID30369","P02730","SLC4A1",0.0682,"Cardiometabolic_II","B13805","20212097_SS211647","PASS",-0.869,1.4465,"Plate control","PASS","PFC",2.72546047824103,"1h","1h"
8594,"PFC_26",16,"OID30369","P02730","SLC4A1",0.0682,"Cardiometabolic_II","B13805","20212097_SS211647","PASS",-0.869,1.495,"Plate control","PASS","PFC",2.81864151028404,"24h","24h"
8595,"PFC_03",25,"OID30369","P02730","SLC4A1",0.0682,"Cardiometabolic_II","B13805","20212097_SS211647","PASS",-0.869,-0.5605,"Plate control","PASS","PFC",0.678067122839494,"Baseline","Baseline"
8596,"PFC_11",26,"OID30369","P02730","SLC4A1",0.0682,"Cardiometabolic_II","B13805","20212097_SS211647","PASS",-0.869,0.1278,"Plate control","PASS","PFC",1.09262625843927,"1h","1h"
8597,"PFC_19",27,"OID30369","P02730","SLC4A1",0.0682,"Cardiometabolic_II","B13805","20212097_SS211647","PASS",-0.869,1.5486,"Plate control","PASS","PFC",2.9253312549149,"24h","24h"
8598,"PFC_27",28,"OID30369","P02730","SLC4A1",0.0682,"Cardiometabolic_II","B13805","20212097_SS211647","PASS",-0.869,-0.019,"Plate control","PASS","PFC",0.986916545885842,"24h","24h"
8599,"PFC_04",37,"OID30369","P02730","SLC4A1",0.0682,"Cardiometabolic_II","B13805","20212097_SS211647","PASS",-0.869,-0.0388,"Plate control","PASS","PFC",0.973464315613742,"Baseline","Baseline"
8600,"PFC_12",38,"OID30369","P02730","SLC4A1",0.0682,"Cardiometabolic_II","B13805","20212097_SS211647","PASS",-0.869,-0.4861,"Plate control","PASS","PFC",0.713952500711097,"1h","1h"
8601,"PFC_20",39,"OID30369","P02730","SLC4A1",0.0682,"Cardiometabolic_II","B13805","20212097_SS211647","PASS",-0.869,0.0944,"Plate control","PASS","PFC",1.06762130448725,"24h","24h"
8602,"PFC_05",49,"OID30369","P02730","SLC4A1",0.0682,"Cardiometabolic_II","B13805","20212097_SS211647","PASS",-0.869,1.0622,"Plate control","PASS","PFC",2.08811330875009,"Baseline","Baseline"
8603,"PFC_13",50,"OID30369","P02730","SLC4A1",0.0682,"Cardiometabolic_II","B13805","20212097_SS211647","PASS",-0.869,0.762,"Plate control","PASS","PFC",1.6958399292663,"1h","1h"
8604,"PFC_21",51,"OID30369","P02730","SLC4A1",0.0682,"Cardiometabolic_II","B13805","20212097_SS211647","PASS",-0.869,1.0469,"Plate control","PASS","PFC",2.06608556026678,"24h","24h"
8605,"PFC_06",61,"OID30369","P02730","SLC4A1",0.0682,"Cardiometabolic_II","B13805","20212097_SS211647","PASS",-0.869,0.3897,"Plate control","PASS","PFC",1.31012094353962,"Baseline","Baseline"
8606,"PFC_14",62,"OID30369","P02730","SLC4A1",0.0682,"Cardiometabolic_II","B13805","20212097_SS211647","PASS",-0.869,-1.2573,"Plate control","PASS","PFC",0.418326123824712,"1h","1h"
8607,"PFC_22",63,"OID30369","P02730","SLC4A1",0.0682,"Cardiometabolic_II","B13805","20212097_SS211647","PASS",-0.869,-0.4393,"Plate control","PASS","PFC",0.737492355371402,"24h","24h"
8608,"PFC_07",73,"OID30369","P02730","SLC4A1",0.0682,"Cardiometabolic_II","B13805","20212097_SS211647","PASS",-0.869,0.8126,"Plate control","PASS","PFC",1.75637389871571,"Baseline","Baseline"
8609,"PFC_15",74,"OID30369","P02730","SLC4A1",0.0682,"Cardiometabolic_II","B13805","20212097_SS211647","PASS",-0.869,-1.1113,"Plate control","PASS","PFC",0.462876748741393,"1h","1h"
8610,"PFC_23",75,"OID30369","P02730","SLC4A1",0.0682,"Cardiometabolic_II","B13805","20212097_SS211647","PASS",-0.869,1.5595,"Plate control","PASS","PFC",2.94751672608967,"24h","24h"
8611,"PFC_08",85,"OID30369","P02730","SLC4A1",0.0682,"Cardiometabolic_II","B13805","20212097_SS211647","PASS",-0.869,-0.378,"Plate control","PASS","PFC",0.769503610080431,"Baseline","Baseline"
8612,"PFC_16",86,"OID30369","P02730","SLC4A1",0.0682,"Cardiometabolic_II","B13805","20212097_SS211647","PASS",-0.869,-1.1194,"Plate control","PASS","PFC",0.460285212750263,"1h","1h"
8613,"PFC_24",87,"OID30369","P02730","SLC4A1",0.0682,"Cardiometabolic_II","B13805","20212097_SS211647","PASS",-0.869,-0.083,"Plate control","PASS","PFC",0.94409241915025,"24h","24h"
8614,"PFC_01",1,"OID30370","Q86TH1","ADAMTSL2",0,"Cardiometabolic_II","B13805","20212097_SS211647","PASS",-2.9668,0.1548,"Plate control","PASS","PFC",1.11326727591604,"Baseline","Baseline"
8615,"PFC_09",2,"OID30370","Q86TH1","ADAMTSL2",0,"Cardiometabolic_II","B13805","20212097_SS211647","PASS",-2.9668,0.3706,"Plate control","PASS","PFC",1.29289041685016,"Baseline","Baseline"
8616,"PFC_17",3,"OID30370","Q86TH1","ADAMTSL2",0,"Cardiometabolic_II","B13805","20212097_SS211647","PASS",-2.9668,0.0746,"Plate control","PASS","PFC",1.05306902274546,"1h","1h"
8617,"PFC_25",4,"OID30370","Q86TH1","ADAMTSL2",0,"Cardiometabolic_II","B13805","20212097_SS211647","PASS",-2.9668,0.0945,"Plate control","PASS","PFC",1.06769530892173,"24h","24h"
8618,"PFC_02",13,"OID30370","Q86TH1","ADAMTSL2",0,"Cardiometabolic_II","B13805","20212097_SS211647","PASS",-2.9668,0.2705,"Plate control","PASS","PFC",1.20622580126391,"Baseline","Baseline"
8619,"PFC_10",14,"OID30370","Q86TH1","ADAMTSL2",0,"Cardiometabolic_II","B13805","20212097_SS211647","PASS",-2.9668,0.5206,"Plate control","PASS","PFC",1.43455173726601,"1h","1h"
8620,"PFC_18",15,"OID30370","Q86TH1","ADAMTSL2",0,"Cardiometabolic_II","B13805","20212097_SS211647","PASS",-2.9668,0.268,"Plate control","PASS","PFC",1.20413738123142,"1h","1h"
8621,"PFC_26",16,"OID30370","Q86TH1","ADAMTSL2",0,"Cardiometabolic_II","B13805","20212097_SS211647","PASS",-2.9668,-0.0037,"Plate control","PASS","PFC",0.997438641323161,"24h","24h"
8622,"PFC_03",25,"OID30370","Q86TH1","ADAMTSL2",0,"Cardiometabolic_II","B13805","20212097_SS211647","PASS",-2.9668,-0.2769,"Plate control","PASS","PFC",0.825362616307007,"Baseline","Baseline"
8623,"PFC_11",26,"OID30370","Q86TH1","ADAMTSL2",0,"Cardiometabolic_II","B13805","20212097_SS211647","PASS",-2.9668,0.2097,"Plate control","PASS","PFC",1.15644768236556,"1h","1h"
8624,"PFC_19",27,"OID30370","Q86TH1","ADAMTSL2",0,"Cardiometabolic_II","B13805","20212097_SS211647","PASS",-2.9668,0.4027,"Plate control","PASS","PFC",1.32197967861279,"24h","24h"

Supplemental table 1_Olink proximity extension assay (PEA) data

,"SampleID","Index","OlinkID","UniProt","Assay","MissingFreq","Panel","Panel_Lot_Nr","PlateID","QC_Warning","LOD","NPX","Normalization","Assay_Warning","condition","Value","group","alt_group"
8625,"PFC_27",28,"OID30370","Q86TH1","ADAMTSL2",0,"Cardiometabolic_II","B13805","20212097_SS211647","PASS",-2.9668,0.083,"Plate control","PASS","PFC",1.05921833468388,"24h","24h"
8626,"PFC_04",37,"OID30370","Q86TH1","ADAMTSL2",0,"Cardiometabolic_II","B13805","20212097_SS211647","PASS",-2.9668,0.4838,"Plate control","PASS","PFC",1.39842220681834,"Baseline","Baseline"
8627,"PFC_12",38,"OID30370","Q86TH1","ADAMTSL2",0,"Cardiometabolic_II","B13805","20212097_SS211647","PASS",-2.9668,-0.0241,"Plate control","PASS","PFC",0.983433905219858,"1h","1h"
8628,"PFC_20",39,"OID30370","Q86TH1","ADAMTSL2",0,"Cardiometabolic_II","B13805","20212097_SS211647","PASS",-2.9668,0.3681,"Plate control","PASS","PFC",1.29065194852728,"24h","24h"
8629,"PFC_05",49,"OID30370","Q86TH1","ADAMTSL2",0,"Cardiometabolic_II","B13805","20212097_SS211647","PASS",-2.9668,0.3524,"Plate control","PASS","PFC",1.2766826913658,"Baseline","Baseline"
8630,"PFC_13",50,"OID30370","Q86TH1","ADAMTSL2",0,"Cardiometabolic_II","B13805","20212097_SS211647","PASS",-2.9668,0.7902,"Plate control","PASS","PFC",1.72931417984903,"1h","1h"
8631,"PFC_21",51,"OID30370","Q86TH1","ADAMTSL2",0,"Cardiometabolic_II","B13805","20212097_SS211647","PASS",-2.9668,0.0856,"Plate control","PASS","PFC",1.06112896074128,"24h","24h"
8632,"PFC_06",61,"OID30370","Q86TH1","ADAMTSL2",0,"Cardiometabolic_II","B13805","20212097_SS211647","PASS",-2.9668,-0.3592,"Plate control","PASS","PFC",0.779596760060543,"Baseline","Baseline"
8633,"PFC_14",62,"OID30370","Q86TH1","ADAMTSL2",0,"Cardiometabolic_II","B13805","20212097_SS211647","PASS",-2.9668,0.5435,"Plate control","PASS","PFC",1.45750415882451,"1h","1h"
8634,"PFC_22",63,"OID30370","Q86TH1","ADAMTSL2",0,"Cardiometabolic_II","B13805","20212097_SS211647","PASS",-2.9668,0.6722,"Plate control","PASS","PFC",1.59350108341646,"24h","24h"
8635,"PFC_07",73,"OID30370","Q86TH1","ADAMTSL2",0,"Cardiometabolic_II","B13805","20212097_SS211647","PASS",-2.9668,-0.1937,"Plate control","PASS","PFC",0.874360419645575,"Baseline","Baseline"
8636,"PFC_15",74,"OID30370","Q86TH1","ADAMTSL2",0,"Cardiometabolic_II","B13805","20212097_SS211647","PASS",-2.9668,-0.4256,"Plate control","PASS","PFC",0.744529026247173,"1h","1h"
8637,"PFC_23",75,"OID30370","Q86TH1","ADAMTSL2",0,"Cardiometabolic_II","B13805","20212097_SS211647","PASS",-2.9668,0.3485,"Plate control","PASS","PFC",1.27323612883495,"24h","24h"
8638,"PFC_08",85,"OID30370","Q86TH1","ADAMTSL2",0,"Cardiometabolic_II","B13805","20212097_SS211647","PASS",-2.9668,0.1302,"Plate control","PASS","PFC",1.09444541309511,"Baseline","Baseline"
8639,"PFC_16",86,"OID30370","Q86TH1","ADAMTSL2",0,"Cardiometabolic_II","B13805","20212097_SS211647","PASS",-2.9668,0.1945,"Plate control","PASS","PFC",1.1443274981768,"1h","1h"
8640,"PFC_24",87,"OID30370","Q86TH1","ADAMTSL2",0,"Cardiometabolic_II","B13805","20212097_SS211647","PASS",-2.9668,-0.2432,"Plate control","PASS","PFC",0.84486925254567,"24h","24h"
8641,"PFC_01",1,"OID30372","P30046","DDT",0,"Cardiometabolic_II","B13805","20212097_SS211647","PASS",-0.7025,0.4707,"Plate control","PASS","PFC",1.38578169048571,"Baseline","Baseline"
8642,"PFC_09",2,"OID30372","P30046","DDT",0,"Cardiometabolic_II","B13805","20212097_SS211647","PASS",-0.7025,0.8328,"Plate control","PASS","PFC",1.78113886582089,"Baseline","Baseline"
8643,"PFC_17",3,"OID30372","P30046","DDT",0,"Cardiometabolic_II","B13805","20212097_SS211647","PASS",-0.7025,0.58,"Plate control","PASS","PFC",1.49484924863494,"1h","1h"
8644,"PFC_25",4,"OID30372","P30046","DDT",0,"Cardiometabolic_II","B13805","20212097_SS211647","PASS",-0.7025,0.7303,"Plate control","PASS","PFC",1.65898403179476,"24h","24h"
8645,"PFC_02",13,"OID30372","P30046","DDT",0,"Cardiometabolic_II","B13805","20212097_SS211647","PASS",-0.7025,0.8445,"Plate control","PASS","PFC",1.79564231571097,"Baseline","Baseline"
8646,"PFC_10",14,"OID30372","P30046","DDT",0,"Cardiometabolic_II","B13805","20212097_SS211647","PASS",-0.7025,0.5871,"Plate control","PASS","PFC",1.50222404956873,"1h","1h"
8647,"PFC_18",15,"OID30372","P30046","DDT",0,"Cardiometabolic_II","B13805","20212097_SS211647","PASS",-0.7025,0.748,"Plate control","PASS","PFC",1.67946298588828,"1h","1h"
8648,"PFC_26",16,"OID30372","P30046","DDT",0,"Cardiometabolic_II","B13805","20212097_SS211647","PASS",-0.7025,0.5379,"Plate control","PASS","PFC",1.451857641313,"24h","24h"
8649,"PFC_03",25,"OID30372","P30046","DDT",0,"Cardiometabolic_II","B13805","20212097_SS211647","PASS",-0.7025,-0.241,"Plate control","PASS","PFC",0.84615859660029,"Baseline","Baseline"
8650,"PFC_11",26,"OID30372","P30046","DDT",0,"Cardiometabolic_II","B13805","20212097_SS211647","PASS",-0.7025,1.2998,"Plate control","PASS","PFC",2.46194750463743,"1h","1h"
8651,"PFC_19",27,"OID30372","P30046","DDT",0,"Cardiometabolic_II","B13805","20212097_SS211647","PASS",-0.7025,0.5898,"Plate control","PASS","PFC",1.5050380903666,"24h","24h"
8652,"PFC_27",28,"OID30372","P30046","DDT",0,"Cardiometabolic_II","B13805","20212097_SS211647","PASS",-0.7025,1.6703,"Plate control","PASS","PFC",3.18280771246856,"24h","24h"
8653,"PFC_04",37,"OID30372","P30046","DDT",0,"Cardiometabolic_II","B13805","20212097_SS211647","PASS",-0.7025,1.0622,"Plate control","PASS","PFC",2.08811330875009,"Baseline","Baseline"
8654,"PFC_12",38,"OID30372","P30046","DDT",0,"Cardiometabolic_II","B13805","20212097_SS211647","PASS",-0.7025,0.4085,"Plate control","PASS","PFC",1.32730506977633,"1h","1h"
8655,"PFC_20",39,"OID30372","P30046","DDT",0,"Cardiometabolic_II","B13805","20212097_SS211647","PASS",-0.7025,1.0576,"Plate control","PASS","PFC",2.08146601043847,"24h","24h"
8656,"PFC_05",49,"OID30372","P30046","DDT",0,"Cardiometabolic_II","B13805","20212097_SS211647","PASS",-0.7025,0.4231,"Plate control","PASS","PFC",1.34080552591644,"Baseline","Baseline"
8657,"PFC_13",50,"OID30372","P30046","DDT",0,"Cardiometabolic_II","B13805","20212097_SS211647","PASS",-0.7025,1.0113,"Plate control","PASS","PFC",2.01572663581356,"1h","1h"
8658,"PFC_21",51,"OID30372","P30046","DDT",0,"Cardiometabolic_II","B13805","20212097_SS211647","PASS",-0.7025,0.2937,"Plate control","PASS","PFC",1.22577993977671,"24h","24h"
8659,"PFC_06",61,"OID30372","P30046","DDT",0,"Cardiometabolic_II","B13805","20212097_SS211647","PASS",-0.7025,0.705,"Plate control","PASS","PFC",1.63014466480525,"Baseline","Baseline"
8660,"PFC_14",62,"OID30372","P30046","DDT",0,"Cardiometabolic_II","B13805","20212097_SS211647","PASS",-0.7025,0.8397,"Plate control","PASS","PFC",1.78967795009217,"1h","1h"
8661,"PFC_22",63,"OID30372","P30046","DDT",0,"Cardiometabolic_II","B13805","20212097_SS211647","PASS",-0.7025,0.5501,"Plate control","PASS","PFC",1.46418718215005,"24h","24h"
8662,"PFC_07",73,"OID30372","P30046","DDT",0,"Cardiometabolic_II","B13805","20212097_SS211647","PASS",-0.7025,0.975,"Plate control","PASS","PFC",1.9656411970905,"Baseline","Baseline"
8663,"PFC_15",74,"OID30372","P30046","DDT",0,"Cardiometabolic_II","B13805","20212097_SS211647","PASS",-0.7025,-0.0397,"Plate control","PASS","PFC",0.972857226353255,"1h","1h"
8664,"PFC_23",75,"OID30372","P30046","DDT",0,"Cardiometabolic_II","B13805","20212097_SS211647","PASS",-0.7025,1.1013,"Plate control","PASS","PFC",2.14547932713698,"24h","24h"
8665,"PFC_08",85,"OID30372","P30046","DDT",0,"Cardiometabolic_II","B13805","20212097_SS211647","PASS",-0.7025,0.1296,"Plate control","PASS","PFC",1.0939903406799,"Baseline","Baseline"
8666,"PFC_16",86,"OID30372","P30046","DDT",0,"Cardiometabolic_II","B13805","20212097_SS211647","PASS",-0.7025,0.6632,"Plate control","PASS","PFC",1.58359124888829,"1h","1h"
8667,"PFC_24",87,"OID30372","P30046","DDT",0,"Cardiometabolic_II","B13805","20212097_SS211647","PASS",-0.7025,0.7945,"Plate control","PASS","PFC",1.73447614649997,"24h","24h"
8668,"PFC_01",1,"OID30373","P23560","BDNF",0,"Cardiometabolic_II","B13805","20212097_SS211647","PASS",-4.5706,2.7802,"Plate control","PASS","PFC",6.86947573704369,"Baseline","Baseline"
8669,"PFC_09",2,"OID30373","P23560","BDNF",0,"Cardiometabolic_II","B13805","20212097_SS211647","PASS",-4.5706,2.0952,"Plate control","PASS","PFC",4.27285393446672,"Baseline","Baseline"
8670,"PFC_17",3,"OID30373","P23560","BDNF",0,"Cardiometabolic_II","B13805","20212097_SS211647","PASS",-4.5706,2.3819,"Plate control","PASS","PFC",5.21222730086593,"1h","1h"
8671,"PFC_25",4,"OID30373","P23560","BDNF",0,"Cardiometabolic_II","B13805","20212097_SS211647","PASS",-4.5706,3.1305,"Plate control","PASS","PFC",8.7573841622783,"24h","24h"
8672,"PFC_02",13,"OID30373","P23560","BDNF",0,"Cardiometabolic_II","B13805","20212097_SS211647","PASS",-4.5706,0.9083,"Plate control","PASS","PFC",1.87683262879982,"Baseline","Baseline"
8673,"PFC_10",14,"OID30373","P23560","BDNF",0,"Cardiometabolic_II","B13805","20212097_SS211647","PASS",-4.5706,1.1786,"Plate control","PASS","PFC",2.26357012231087,"1h","1h"
8674,"PFC_18",15,"OID30373","P23560","BDNF",0,"Cardiometabolic_II","B13805","20212097_SS211647","PASS",-4.5706,1.7106,"Plate control","PASS","PFC",3.27296914081815,"1h","1h"
8675,"PFC_26",16,"OID30373","P23560","BDNF",0,"Cardiometabolic_II","B13805","20212097_SS211647","PASS",-4.5706,1.9977,"Plate control","PASS","PFC",3.99362812643154,"24h","24h"
8676,"PFC_03",25,"OID30373","P23560","BDNF",0,"Cardiometabolic_II","B13805","20212097_SS211647","PASS",-4.5706,0.1913,"Plate control","PASS","PFC",1.14179211143735,"Baseline","Baseline"
8677,"PFC_11",26,"OID30373","P23560","BDNF",0,"Cardiometabolic_II","B13805","20212097_SS211647","PASS",-4.5706,1.5676,"Plate control","PASS","PFC",2.96411207929356,"1h","1h"
8678,"PFC_19",27,"OID30373","P23560","BDNF",0,"Cardiometabolic_II","B13805","20212097_SS211647","PASS",-4.5706,2.0014,"Plate control","PASS","PFC",4.00388350819631,"24h","24h"
8679,"PFC_27",28,"OID30373","P23560","BDNF",0,"Cardiometabolic_II","B13805","20212097_SS211647","PASS",-4.5706,2.6946,"Plate control","PASS","PFC",6.47374258096287,"24h","24h"
8680,"PFC_04",37,"OID30373","P23560","BDNF",0,"Cardiometabolic_II","B13805","20212097_SS211647","PASS",-4.5706,2.818,"Plate control","PASS","PFC",7.05184126325736,"Baseline","Baseline"
8681,"PFC_12",38,"OID30373","P23560","BDNF",0,"Cardiometabolic_II","B13805","20212097_SS211647","PASS",-4.5706,0.1151,"Plate control","PASS","PFC",1.08305011423752,"1h","1h"
8682,"PFC_20",39,"OID30373","P23560","BDNF",0,"Cardiometabolic_II","B13805","20212097_SS211647","PASS",-4.5706,2.2563,"Plate control","PASS","PFC",4.77764612859409,"24h","24h"
8683,"PFC_05",49,"OID30373","P23560","BDNF",0,"Cardiometabolic_II","B13805","20212097_SS211647","PASS",-4.5706,1.0655,"Plate control","PASS","PFC",2.09289509607488,"Baseline","Baseline"
8684,"PFC_13",50,"OID30373","P23560","BDNF",0,"Cardiometabolic_II","B13805","20212097_SS211647","PASS",-4.5706,2.2664,"Plate control","PASS","PFC",4.81121076155472,"1h","1h"
8685,"PFC_21",51,"OID30373","P23560","BDNF",0,"Cardiometabolic_II","B13805","20212097_SS211647","PASS",-4.5706,1.0034,"Plate control","PASS","PFC",2.00471895923029,"24h","24h"
8686,"PFC_06",61,"OID30373","P23560","BDNF",0,"Cardiometabolic_II","B13805","20212097_SS211647","PASS",-4.5706,3.4754,"Plate control","PASS","PFC",11.122429123429,"Baseline","Baseline"
8687,"PFC_14",62,"OID30373","P23560","BDNF",0,"Cardiometabolic_II","B13805","20212097_SS211647","PASS",-4.5706,2.3322,"Plate control","PASS","PFC",5.03572674559222,"1h","1h"
8688,"PFC_22",63,"OID30373","P23560","BDNF",0,"Cardiometabolic_II","B13805","20212097_SS211647","PASS",-4.5706,2.212,"Plate control","PASS","PFC",4.63317122477857,"24h","24h"
8689,"PFC_07",73,"OID30373","P23560","BDNF",0,"Cardiometabolic_II","B13805","20212097_SS211647","PASS",-4.5706,3.1181,"Plate control","PASS","PFC",8.68243677485451,"Baseline","Baseline"
8690,"PFC_15",74,"OID30373","P23560","BDNF",0,"Cardiometabolic_II","B13805","20212097_SS211647","PASS",-4.5706,1.9071,"Plate control","PASS","PFC",3.75054434474266,"1h","1h"
8691,"PFC_23",75,"OID30373","P23560","BDNF",0,"Cardiometabolic_II","B13805","20212097_SS211647","PASS",-4.5706,1.9641,"Plate control","PASS","PFC",3.90169228143831,"24h","24h"
8692,"PFC_08",85,"OID30373","P23560","BDNF",0,"Cardiometabolic_II","B13805","20212097_SS211647","PASS",-4.5706,1.1076,"Plate control","PASS","PFC",2.154868750775,"Baseline","Baseline"
8693,"PFC_16",86,"OID30373","P23560","BDNF",0,"Cardiometabolic_II","B13805","20212097_SS211647","PASS",-4.5706,2.625,"Plate control","PASS","PFC",6.16884330163176,"1h","1h"
8694,"PFC_24",87,"OID30373","P23560","BDNF",0,"Cardiometabolic_II","B13805","20212097_SS211647","PASS",-4.5706,2.3793,"Plate control","PASS","PFC",5.20284237437107,"24h","24h"
8695,"PFC_01",1,"OID30374","Q9NRR1","CYTL1",0,"Cardiometabolic_II","B13805","20212097_SS211647","PASS",-2.6281,0.4652,"Plate control","PASS","PFC",1.38050871927715,"Baseline","Baseline"
8696,"PFC_09",2,"OID30374","Q9NRR1","CYTL1",0,"Cardiometabolic_II","B13805","20212097_SS211647","PASS",-2.6281,0.2132,"Plate control","PASS","PFC",1.15925664786101,"Baseline","Baseline"
8697,"PFC_17",3,"OID30374","Q9NRR1","CYTL1",0,"Cardiometabolic_II","B13805","20212097_SS211647","PASS",-2.6281,0.3098,"Plate control","PASS","PFC",1.23953585187148,"1h","1h"
8698,"PFC_25",4,"OID30374","Q9NRR1","CYTL1",0,"Cardiometabolic_II","B13805","20212097_SS211647","PASS",-2.6281,-0.3892,"Plate control","PASS","PFC",0.763552890736301,"24h","24h"
8699,"PFC_02",13,"OID30374","Q9NRR1","CYTL1",0,"Cardiometabolic_II","B13805","20212097_SS211647","PASS",-2.6281,0.7925,"Plate control","PASS","PFC",1.73207331789726,"Baseline","Baseline"
8700,"PFC_10",14,"OID30374","Q9NRR1","CYTL1",0,"Cardiometabolic_II","B13805","20212097_SS211647","PASS",-2.6281,0.8954,"Plate control","PASS","PFC",1.8601255500489,"1h","1h"
8701,"PFC_18",15,"OID30374","Q9NRR1","CYTL1",0,"Cardiometabolic_II","B13805","20212097_SS211647","PASS",-2.6281,0.1052,"Plate control","PASS","PFC",1.07564349597324,"1h","1h"
8702,"PFC_26",16,"OID30374","Q9NRR1","CYTL1",0,"Cardiometabolic_II","B13805","20212097_SS211647","PASS",-2.6281,0.5495,"Plate control","PASS","PFC",1.4635783704274,"24h","24h"
8703,"PFC_03",25,"OID30374","Q9NRR1","CYTL1",0,"Cardiometabolic_II","B13805","20212097_SS211647","PASS",-2.6281,1.0512,"Plate control","PASS","PFC",2.07225278244327,"Baseline","Baseline"
8704,"PFC_11",26,"OID30374","Q9NRR1","CYTL1",0,"Cardiometabolic_II","B13805","20212097_SS211647","PASS",-2.6281,0.9651,"Plate control","PASS","PFC",1.95219883297456,"1h","1h"
8705,"PFC_19",27,"OID30374","Q9NRR1","CYTL1",0,"Cardiometabolic_II","B13805","20212097_SS211647","PASS",-2.6281,0.4795,"Plate control","PASS","PFC",1.39426036878436,"24h","24h"
8706,"PFC_27",28,"OID30374","Q9NRR1","CYTL1",0,"Cardiometabolic_II","B13805","20212097_SS211647","PASS",-2.6281,0.0027,"Plate control","PASS","PFC",1.00187324973175,"24h","24h"
8707,"PFC_04",37,"OID30374","Q9NRR1","CYTL1",0,"Cardiometabolic_II","B13805","20212097_SS211647","PASS",-2.6281,0.8839,"Plate control","PASS","PFC",1.84535707046941,"Baseline","Baseline"
8708,"PFC_12",38,"OID30374","Q9NRR1","CYTL1",0,"Cardiometabolic_II","B13805","20212097_SS211647","PASS",-2.6281,1.027,"Plate control","PASS","PFC",2.03778239323345,"1h","1h"
8709,"PFC_20",39,"OID30374","Q9NRR1","CYTL1",0,"Cardiometabolic_II","B13805","20212097_SS211647","PASS",-2.6281,0.7301,"Plate control","PASS","PFC",1.65875406371446,"24h","24h"
8710,"PFC_05",49,"OID30374","Q9NRR1","CYTL1",0,"Cardiometabolic_II","B13805","20212097_SS211647","PASS",-2.6281,0.3753,"Plate control","PASS","PFC",1.29710925289541,"Baseline","Baseline"
8711,"PFC_13",50,"OID30374","Q9NRR1","CYTL1",0,"Cardiometabolic_II","B13805","20212097_SS211647","PASS",-2.6281,0.5209,"Plate control","PASS","PFC",1.43485007493134,"1h","1h"
8712,"PFC_21",51,"OID30374","Q9NRR1","CYTL1",0,"Cardiometabolic_II","B13805","20212097_SS211647","PASS",-2.6281,0.9538,"Plate control","PASS","PFC",1.93696783908066,"24h","24h"
8713,"PFC_06",61,"OID30374","Q9NRR1","CYTL1",0,"Cardiometabolic_II","B13805","20212097_SS211647","PASS",-2.6281,0.3746,"Plate control","PASS","PFC",1.29648004421966,"Baseline","Baseline"
8714,"PFC_14",62,"OID30374","Q9NRR1","CYTL1",0,"Cardiometabolic_II","B13805","20212097_SS211647","PASS",-2.6281,0.8582,"Plate control","PASS","PFC",1.81277516311507,"1h","1h"
8715,"PFC_22",63,"OID30374","Q9NRR1","CYTL1",0,"Cardiometabolic_II","B13805","20212097_SS211647","PASS",-2.6281,0.4762,"Plate control","PASS","PFC",1.39107480225911,"24h","24h"
8716,"PFC_07",73,"OID30374","Q9NRR1","CYTL1",0,"Cardiometabolic_II","B13805","20212097_SS211647","PASS",-2.6281,-0.6591,"Plate control","PASS","PFC",0.633273230184881,"Baseline","Baseline"
8717,"PFC_15",74,"OID30374","Q9NRR1","CYTL1",0,"Cardiometabolic_II","B13805","20212097_SS211647","PASS",-2.6281,0.3788,"Plate control","PASS","PFC",1.3002598797597,"1h","1h"
8718,"PFC_23",75,"OID30374","Q9NRR1","CYTL1",0,"Cardiometabolic_II","B13805","20212097_SS211647","PASS",-2.6281,0.5499,"Plate control","PASS","PFC",1.46398421677544,"24h","24h"
8719,"PFC_08",85,"OID30374","Q9NRR1","CYTL1",0,"Cardiometabolic_II","B13805","20212097_SS211647","PASS",-2.6281,0.1384,"Plate control","PASS","PFC",1.10068374139672,"Baseline","Baseline"
8720,"PFC_16",86,"OID30374","Q9NRR1","CYTL1",0,"Cardiometabolic_II","B13805","20212097_SS211647","PASS",-2.6281,-0.3924,"Plate control","PASS","PFC",0.761861153119994,"1h","1h"
8721,"PFC_24",87,"OID30374","Q9NRR1","CYTL1",0,"Cardiometabolic_II","B13805","20212097_SS211647","PASS",-2.6281,0.7374,"Plate control","PASS","PFC",1.66716858752705,"24h","24h"
8722,"PFC_01",1,"OID30375","P13667","PDIA4",0,"Cardiometabolic_II","B13805","20212097_SS211647","PASS",-1.003,1.4646,"Plate control","PASS","PFC",2.75986940242645,"Baseline","Baseline"
8723,"PFC_09",2,"OID30375","P13667","PDIA4",0,"Cardiometabolic_II","B13805","20212097_SS211647","PASS",-1.003,2.324,"Plate control","PASS","PFC",5.00718583461598,"Baseline","Baseline"
8724,"PFC_17",3,"OID30375","P13667","PDIA4",0,"Cardiometabolic_II","B13805","20212097_SS211647","PASS",-1.003,3.6166,"Plate control","PASS","PFC",12.2660599319366,"1h","1h"
8725,"PFC_25",4,"OID30375","P13667","PDIA4",0,"Cardiometabolic_II","B13805","20212097_SS211647","PASS",-1.003,2.7447,"Plate control","PASS","PFC",6.70250318606701,"24h","24h"
8726,"PFC_02",13,"OID30375","P13667","PDIA4",0,"Cardiometabolic_II","B13805","20212097_SS211647","PASS",-1.003,1.6755,"Plate control","PASS","PFC",3.19430041377269,"Baseline","Baseline"
8727,"PFC_10",14,"OID30375","P13667","PDIA4",0,"Cardiometabolic_II","B13805","20212097_SS211647","PASS",-1.003,2.1034,"Plate control","PASS","PFC",4.2972091606928,"1h","1h"
8728,"PFC_18",15,"OID30375","P13667","PDIA4",0,"Cardiometabolic_II","B13805","20212097_SS211647","PASS",-1.003,2.651,"Plate control","PASS","PFC",6.28102494938858,"1h","1h"
8729,"PFC_26",16,"OID30375","P13667","PDIA4",0,"Cardiometabolic_II","B13805","20212097_SS211647","PASS",-1.003,3.3594,"Plate control","PASS","PFC",10.2631379737181,"24h","24h"
8730,"PFC_03",25,"OID30375","P13667","PDIA4",0,"Cardiometabolic_II","B13805","20212097_SS211647","PASS",-1.003,-0.0375,"Plate control","PASS","PFC",0.974341891201707,"Baseline","Baseline"
8731,"PFC_11",26,"OID30375","P13667","PDIA4",0,"Cardiometabolic_II","B13805","20212097_SS211647","PASS",-1.003,2.8239,"Plate control","PASS","PFC",7.08073930017794,"1h","1h"
8732,"PFC_19",27,"OID30375","P13667","PDIA4",0,"Cardiometabolic_II","B13805","20212097_SS211647","PASS",-1.003,2.3897,"Plate control","PASS","PFC",5.24048377415847,"24h","24h"
8733,"PFC_27",28,"OID30375","P13667","PDIA4",0,"Cardiometabolic_II","B13805","20212097_SS211647","PASS",-1.003,3.8101,"Plate control","PASS","PFC",14.0266637616055,"24h","24h"
8734,"PFC_04",37,"OID30375","P13667","PDIA4",0,"Cardiometabolic_II","B13805","20212097_SS211647","PASS",-1.003,2.4024,"Plate control","PASS","PFC",5.28681923698568,"Baseline","Baseline"
8735,"PFC_12",38,"OID30375","P13667","PDIA4",0,"Cardiometabolic_II","B13805","20212097_SS211647","PASS",-1.003,0.7775,"Plate control","PASS","PFC",1.7141578884562,"1h","1h"
8736,"PFC_20",39,"OID30375","P13667","PDIA4",0,"Cardiometabolic_II","B13805","20212097_SS211647","PASS",-1.003,3.4719,"Plate control","PASS","PFC",11.0954786464221,"24h","24h"

Supplemental table 1_Olink proximity extension assay (PEA) data

,"SampleID","Index","OlinkID","UniProt","Assay","MissingFreq","Panel","Panel_Lot_Nr","PlateID","QC_Warning","LOD","NPX","Normalization","Assay_Warning","condition","Value","group","alt_group"
8737,"PFC_05",49,"OID30375","P13667","PDIA4",0,"Cardiometabolic_II","B13805","20212097_SS211647","PASS",-1.003,1.2108,"Plate control","PASS","PFC",2.31465953179183,"Baseline","Baseline"
8738,"PFC_13",50,"OID30375","P13667","PDIA4",0,"Cardiometabolic_II","B13805","20212097_SS211647","PASS",-1.003,2.7306,"Plate control","PASS","PFC",6.63731617478558,"1h","1h"
8739,"PFC_21",51,"OID30375","P13667","PDIA4",0,"Cardiometabolic_II","B13805","20212097_SS211647","PASS",-1.003,1.5321,"Plate control","PASS","PFC",2.89206504481733,"24h","24h"
8740,"PFC_06",61,"OID30375","P13667","PDIA4",0,"Cardiometabolic_II","B13805","20212097_SS211647","PASS",-1.003,2.1312,"Plate control","PASS","PFC",4.3808171512921,"Baseline","Baseline"
8741,"PFC_14",62,"OID30375","P13667","PDIA4",0,"Cardiometabolic_II","B13805","20212097_SS211647","PASS",-1.003,2.9561,"Plate control","PASS","PFC",7.76023316246588,"1h","1h"
8742,"PFC_22",63,"OID30375","P13667","PDIA4",0,"Cardiometabolic_II","B13805","20212097_SS211647","PASS",-1.003,2.2138,"Plate control","PASS","PFC",4.63895547766166,"24h","24h"
8743,"PFC_07",73,"OID30375","P13667","PDIA4",0,"Cardiometabolic_II","B13805","20212097_SS211647","PASS",-1.003,2.9643,"Plate control","PASS","PFC",7.804466416665,"Baseline","Baseline"
8744,"PFC_15",74,"OID30375","P13667","PDIA4",0,"Cardiometabolic_II","B13805","20212097_SS211647","PASS",-1.003,1.4469,"Plate control","PASS","PFC",2.72621624110566,"1h","1h"
8745,"PFC_23",75,"OID30375","P13667","PDIA4",0,"Cardiometabolic_II","B13805","20212097_SS211647","PASS",-1.003,2.8356,"Plate control","PASS","PFC",7.13839631367391,"24h","24h"
8746,"PFC_08",85,"OID30375","P13667","PDIA4",0,"Cardiometabolic_II","B13805","20212097_SS211647","PASS",-1.003,2.4791,"Plate control","PASS","PFC",5.57549540524841,"Baseline","Baseline"
8747,"PFC_16",86,"OID30375","P13667","PDIA4",0,"Cardiometabolic_II","B13805","20212097_SS211647","PASS",-1.003,2.6643,"Plate control","PASS","PFC",6.33919654921791,"1h","1h"
8748,"PFC_24",87,"OID30375","P13667","PDIA4",0,"Cardiometabolic_II","B13805","20212097_SS211647","PASS",-1.003,3.1643,"Plate control","PASS","PFC",8.96497773445269,"24h","24h"
8749,"PFC_01",1,"OID30376","Q8N6C8","LILRA3",0.0227,"Cardiometabolic_II","B13805","20212097_SS211647","PASS",-4.8333,-0.4749,"Plate control","PASS","PFC",0.7195166613715,"Baseline","Baseline"
8750,"PFC_09",2,"OID30376","Q8N6C8","LILRA3",0.0227,"Cardiometabolic_II","B13805","20212097_SS211647","PASS",-4.8333,0.3727,"Plate control","PASS","PFC",1.29477373023081,"Baseline","Baseline"
8751,"PFC_17",3,"OID30376","Q8N6C8","LILRA3",0.0227,"Cardiometabolic_II","B13805","20212097_SS211647","PASS",-4.8333,-0.5172,"Plate control","PASS","PFC",0.698726615134464,"1h","1h"
8752,"PFC_25",4,"OID30376","Q8N6C8","LILRA3",0.0227,"Cardiometabolic_II","B13805","20212097_SS211647","PASS",-4.8333,0.0287,"Plate control","PASS","PFC",1.02009251491695,"24h","24h"
8753,"PFC_02",13,"OID30376","Q8N6C8","LILRA3",0.0227,"Cardiometabolic_II","B13805","20212097_SS211647","PASS",-4.8333,0.1111,"Plate control","PASS","PFC",1.08005142069808,"Baseline","Baseline"
8754,"PFC_10",14,"OID30376","Q8N6C8","LILRA3",0.0227,"Cardiometabolic_II","B13805","20212097_SS211647","PASS",-4.8333,-0.3421,"Plate control","PASS","PFC",0.788892157119846,"1h","1h"
8755,"PFC_18",15,"OID30376","Q8N6C8","LILRA3",0.0227,"Cardiometabolic_II","B13805","20212097_SS211647","PASS",-4.8333,0.4968,"Plate control","PASS","PFC",1.41108021259473,"1h","1h"
8756,"PFC_26",16,"OID30376","Q8N6C8","LILRA3",0.0227,"Cardiometabolic_II","B13805","20212097_SS211647","PASS",-4.8333,-0.3614,"Plate control","PASS","PFC",0.778408840382573,"24h","24h"
8757,"PFC_03",25,"OID30376","Q8N6C8","LILRA3",0.0227,"Cardiometabolic_II","B13805","20212097_SS211647","PASS",-4.8333,0.0422,"Plate control","PASS","PFC",1.02968281788859,"Baseline","Baseline"
8758,"PFC_11",26,"OID30376","Q8N6C8","LILRA3",0.0227,"Cardiometabolic_II","B13805","20212097_SS211647","PASS",-4.8333,0.0757,"Plate control","PASS","PFC",1.05387225392959,"1h","1h"
8759,"PFC_19",27,"OID30376","Q8N6C8","LILRA3",0.0227,"Cardiometabolic_II","B13805","20212097_SS211647","PASS",-4.8333,-0.4146,"Plate control","PASS","PFC",0.750227473032734,"24h","24h"
8760,"PFC_27",28,"OID30376","Q8N6C8","LILRA3",0.0227,"Cardiometabolic_II","B13805","20212097_SS211647","PASS",-4.8333,0.4957,"Plate control","PASS","PFC",1.41000472775694,"24h","24h"
8761,"PFC_04",37,"OID30376","Q8N6C8","LILRA3",0.0227,"Cardiometabolic_II","B13805","20212097_SS211647","PASS",-4.8333,-0.05,"Plate control","PASS","PFC",0.965936328924846,"Baseline","Baseline"
8762,"PFC_12",38,"OID30376","Q8N6C8","LILRA3",0.0227,"Cardiometabolic_II","B13805","20212097_SS211647","PASS",-4.8333,0.2628,"Plate control","PASS","PFC",1.19980504254719,"1h","1h"
8763,"PFC_20",39,"OID30376","Q8N6C8","LILRA3",0.0227,"Cardiometabolic_II","B13805","20212097_SS211647","PASS",-4.8333,0.1469,"Plate control","PASS","PFC",1.10718783741168,"24h","24h"
8764,"PFC_05",49,"OID30376","Q8N6C8","LILRA3",0.0227,"Cardiometabolic_II","B13805","20212097_SS211647","PASS",-4.8333,-0.6538,"Plate control","PASS","PFC",0.63560395196301,"Baseline","Baseline"
8765,"PFC_13",50,"OID30376","Q8N6C8","LILRA3",0.0227,"Cardiometabolic_II","B13805","20212097_SS211647","PASS",-4.8333,-0.0903,"Plate control","PASS","PFC",0.93932740126204l,"1h","1h"
8766,"PFC_21",51,"OID30376","Q8N6C8","LILRA3",0.0227,"Cardiometabolic_II","B13805","20212097_SS211647","PASS",-4.8333,0.0965,"Plate control","PASS","PFC",1.06917647533699,"24h","24h"
8767,"PFC_06",61,"OID30376","Q8N6C8","LILRA3",0.0227,"Cardiometabolic_II","B13805","20212097_SS211647","PASS",-4.8333,-1.3205,"Plate control","PASS","PFC",0.400396148015039,"Baseline","Baseline"
8768,"PFC_14",62,"OID30376","Q8N6C8","LILRA3",0.0227,"Cardiometabolic_II","B13805","20212097_SS211647","PASS",-4.8333,-0.6461,"Plate control","PASS","PFC",0.639005387588153,"1h","1h"
8769,"PFC_22",63,"OID30376","Q8N6C8","LILRA3",0.0227,"Cardiometabolic_II","B13805","20212097_SS211647","PASS",-4.8333,-0.1923,"Plate control","PASS","PFC",0.875209316109631,"24h","24h"
8770,"PFC_07",73,"OID30376","Q8N6C8","LILRA3",0.0227,"Cardiometabolic_II","B13805","20212097_SS211647","PASS",-4.8333,0.0472,"Plate control","PASS","PFC",1.03325761767685,"Baseline","Baseline"
8771,"PFC_15",74,"OID30376","Q8N6C8","LILRA3",0.0227,"Cardiometabolic_II","B13805","20212097_SS211647","PASS",-4.8333,-1.3397,"Plate control","PASS","PFC",0.395102806708956,"1h","1h"
8772,"PFC_23",75,"OID30376","Q8N6C8","LILRA3",0.0227,"Cardiometabolic_II","B13805","20212097_SS211647","PASS",-4.8333,-0.3728,"Plate control","PASS","PFC",0.77228218671 5285,"24h","24h"
8773,"PFC_08",85,"OID30376","Q8N6C8","LILRA3",0.0227,"Cardiometabolic_II","B13805","20212097_SS211647","PASS",-4.8333,-0.471,"Plate control","PASS","PFC",0.721464343430814,"Baseline","Baseline"
8774,"PFC_16",86,"OID30376","Q8N6C8","LILRA3",0.0227,"Cardiometabolic_II","B13805","20212097_SS211647","PASS",-4.8333,0.0286,"Plate control","PASS","PFC",1.02002180994236,"1h","1h"
8775,"PFC_24",87,"OID30376","Q8N6C8","LILRA3",0.0227,"Cardiometabolic_II","B13805","20212097_SS211647","PASS",-4.8333,-1.2955,"Plate control","PASS","PFC",0.407394949401444,"24h","24h"
8776,"PFC_01",1,"OID30377","Q02817","MUC2",0.0227,"Cardiometabolic_II","B13805","20212097_SS211647","PASS",-2.301,-0.278,"Plate control","PASS","PFC",0.824733548610081,"Baseline","Baseline"
8777,"PFC_09",2,"OID30377","Q02817","MUC2",0.0227,"Cardiometabolic_II","B13805","20212097_SS211647","PASS",-2.301,0.299,"Plate control","PASS","PFC",1.23029134475114,"Baseline","Baseline"
8778,"PFC_17",3,"OID30377","Q02817","MUC2",0.0227,"Cardiometabolic_II","B13805","20212097_SS211647","PASS",-2.301,-1.2413,"Plate control","PASS","PFC",0.422991330579817,"1h","1h"
8779,"PFC_25",4,"OID30377","Q02817","MUC2",0.0227,"Cardiometabolic_II","B13805","20212097_SS211647","PASS",-2.301,-1.975,"Plate control","PASS","PFC",0.254369923025672,"24h","24h"
8780,"PFC_02",13,"OID30377","Q02817","MUC2",0.0227,"Cardiometabolic_II","B13805","20212097_SS211647","PASS",-2.301,-1.1022,"Plate control","PASS","PFC",0.465805635803596,"Baseline","Baseline"
8781,"PFC_10",14,"OID30377","Q02817","MUC2",0.0227,"Cardiometabolic_II","B13805","20212097_SS211647","PASS",-2.301,-0.4563,"Plate control","PASS","PFC",0.728853112905036,"1h","1h"
8782,"PFC_18",15,"OID30377","Q02817","MUC2",0.0227,"Cardiometabolic_II","B13805","20212097_SS211647","PASS",-2.301,0.4601,"Plate control","PASS","PFC",1.37563716673753,"1h","1h"
8783,"PFC_26",16,"OID30377","Q02817","MUC2",0.0227,"Cardiometabolic_II","B13805","20212097_SS211647","PASS",-2.301,-0.9408,"Plate control","PASS","PFC",0.520943927661498,"24h","24h"
8784,"PFC_03",25,"OID30377","Q02817","MUC2",0.0227,"Cardiometabolic_II","B13805","20212097_SS211647","PASS",-2.301,0.0437,"Plate control","PASS","PFC",1.03075395724815,"Baseline","Baseline"
8785,"PFC_11",26,"OID30377","Q02817","MUC2",0.0227,"Cardiometabolic_II","B13805","20212097_SS211647","PASS",-2.301,-1.163,"Plate control","PASS","PFC",0.44658292617638,"1h","1h"
8786,"PFC_19",27,"OID30377","Q02817","MUC2",0.0227,"Cardiometabolic_II","B13805","20212097_SS211647","PASS",-2.301,-0.0703,"Plate control","PASS","PFC",0.952439923136179,"24h","24h"
8787,"PFC_27",28,"OID30377","Q02817","MUC2",0.0227,"Cardiometabolic_II","B13805","20212097_SS211647","PASS",-2.301,0.5031,"Plate control","PASS","PFC",1.41725562977806,"24h","24h"
8788,"PFC_04",37,"OID30377","Q02817","MUC2",0.0227,"Cardiometabolic_II","B13805","20212097_SS211647","PASS",-2.301,1.3423,"Plate control","PASS","PFC",2.53555224251945,"Baseline","Baseline"
8789,"PFC_12",38,"OID30377","Q02817","MUC2",0.0227,"Cardiometabolic_II","B13805","20212097_SS211647","PASS",-2.301,-0.099,"Plate control","PASS","PFC",0.933679944915313,"1h","1h"
8790,"PFC_20",39,"OID30377","Q02817","MUC2",0.0227,"Cardiometabolic_II","B13805","20212097_SS211647","PASS",-2.301,-1.1973,"Plate control","PASS","PFC",0.436090660952645,"24h","24h"
8791,"PFC_05",49,"OID30377","Q02817","MUC2",0.0227,"Cardiometabolic_II","B13805","20212097_SS211647","PASS",-2.301,-0.7169,"Plate control","PASS","PFC",0.608403349162133,"Baseline","Baseline"
8792,"PFC_13",50,"OID30377","Q02817","MUC2",0.0227,"Cardiometabolic_II","B13805","20212097_SS211647","PASS",-2.301,1.1631,"Plate control","PASS","PFC",2.23938099399123,"1h","1h"
8793,"PFC_21",51,"OID30377","Q02817","MUC2",0.0227,"Cardiometabolic_II","B13805","20212097_SS211647","PASS",-2.301,-0.1902,"Plate control","PASS","PFC",0.876484206382165,"24h","24h"
8794,"PFC_06",61,"OID30377","Q02817","MUC2",0.0227,"Cardiometabolic_II","B13805","20212097_SS211647","PASS",-2.301,1.008,"Plate control","PASS","PFC",2.01112116079694,"Baseline","Baseline"
8795,"PFC_14",62,"OID30377","Q02817","MUC2",0.0227,"Cardiometabolic_II","B13805","20212097_SS211647","PASS",-2.301,-1.2175,"Plate control","PASS","PFC",0.430027253387625,"1h","1h"
8796,"PFC_22",63,"OID30377","Q02817","MUC2",0.0227,"Cardiometabolic_II","B13805","20212097_SS211647","PASS",-2.301,0.9416,"Plate control","PASS","PFC",1.92065713484615,"24h","24h"
8797,"PFC_07",73,"OID30377","Q02817","MUC2",0.0227,"Cardiometabolic_II","B13805","20212097_SS211647","PASS",-2.301,-2.3199,"Plate control","PASS","PFC",0.200281351361689,"Baseline","Baseline"
8798,"PFC_15",74,"OID30377","Q02817","MUC2",0.0227,"Cardiometabolic_II","B13805","20212097_SS211647","PASS",-2.301,0.9158,"Plate control","PASS","PFC",1.88661494329554,"1h","1h"
8799,"PFC_23",75,"OID30377","Q02817","MUC2",0.0227,"Cardiometabolic_II","B13805","20212097_SS211647","PASS",-2.301,-0.897,"Plate control","PASS","PFC",0.537002235810062,"24h","24h"
8800,"PFC_08",85,"OID30377","Q02817","MUC2",0.0227,"Cardiometabolic_II","B13805","20212097_SS211647","PASS",-2.301,-1.4833,"Plate control","PASS","PFC",0.357669746939235,"Baseline","Baseline"
8801,"PFC_16",86,"OID30377","Q02817","MUC2",0.0227,"Cardiometabolic_II","B13805","20212097_SS211647","PASS",-2.301,-2.1722,"Plate control","PASS","PFC",0.221872074184761,"1h","1h"
8802,"PFC_24",87,"OID30377","Q02817","MUC2",0.0227,"Cardiometabolic_II","B13805","20212097_SS211647","PASS",-2.301,1.1081,"Plate control","PASS","PFC",2.15561570080364,"24h","24h"
8803,"PFC_01",1,"OID30378","P98095","FBLN2",0,"Cardiometabolic_II","B13805","20212097_SS211647","PASS",-3.6645,0.1878,"Plate control","PASS","PFC",1.13902546381888,"Baseline","Baseline"
8804,"PFC_09",2,"OID30378","P98095","FBLN2",0,"Cardiometabolic_II","B13805","20212097_SS211647","PASS",-3.6645,0.5876,"Plate control","PASS","PFC",1.50274477097992,"Baseline","Baseline"
8805,"PFC_17",3,"OID30378","P98095","FBLN2",0,"Cardiometabolic_II","B13805","20212097_SS211647","PASS",-3.6645,-0.1524,"Plate control","PASS","PFC",0.899752430862952,"1h","1h"
8806,"PFC_25",4,"OID30378","P98095","FBLN2",0,"Cardiometabolic_II","B13805","20212097_SS211647","PASS",-3.6645,0.4568,"Plate control","PASS","PFC",1.37249414997584,"24h","24h"
8807,"PFC_02",13,"OID30378","P98095","FBLN2",0,"Cardiometabolic_II","B13805","20212097_SS211647","PASS",-3.6645,0.675,"Plate control","PASS","PFC",1.5965967727133,"Baseline","Baseline"
8808,"PFC_10",14,"OID30378","P98095","FBLN2",0,"Cardiometabolic_II","B13805","20212097_SS211647","PASS",-3.6645,0.6132,"Plate control","PASS","PFC",1.52964831746866,"1h","1h"
8809,"PFC_18",15,"OID30378","P98095","FBLN2",0,"Cardiometabolic_II","B13805","20212097_SS211647","PASS",-3.6645,0.6045,"Plate control","PASS","PFC",1.52045171350806,"1h","1h"
8810,"PFC_26",16,"OID30378","P98095","FBLN2",0,"Cardiometabolic_II","B13805","20212097_SS211647","PASS",-3.6645,0.0477,"Plate control","PASS","PFC",1.03361577954023,"24h","24h"
8811,"PFC_03",25,"OID30378","P98095","FBLN2",0,"Cardiometabolic_II","B13805","20212097_SS211647","PASS",-3.6645,0.2913,"Plate control","PASS","PFC",1.22374248477183,"Baseline","Baseline"
8812,"PFC_11",26,"OID30378","P98095","FBLN2",0,"Cardiometabolic_II","B13805","20212097_SS211647","PASS",-3.6645,0.8292,"Plate control","PASS","PFC",1.77669987751534,"1h","1h"
8813,"PFC_19",27,"OID30378","P98095","FBLN2",0,"Cardiometabolic_II","B13805","20212097_SS211647","PASS",-3.6645,0.4512,"Plate control","PASS","PFC",1.36717696977752,"24h","24h"
8814,"PFC_27",28,"OID30378","P98095","FBLN2",0,"Cardiometabolic_II","B13805","20212097_SS211647","PASS",-3.6645,0.6909,"Plate control","PASS","PFC",1.61429025105787,"24h","24h"
8815,"PFC_04",37,"OID30378","P98095","FBLN2",0,"Cardiometabolic_II","B13805","20212097_SS211647","PASS",-3.6645,1.2901,"Plate control","PASS","PFC",2.44545005519075,"Baseline","Baseline"
8816,"PFC_12",38,"OID30378","P98095","FBLN2",0,"Cardiometabolic_II","B13805","20212097_SS211647","PASS",-3.6645,0.3127,"Plate control","PASS","PFC",1.24202998205534,"1h","1h"
8817,"PFC_20",39,"OID30378","P98095","FBLN2",0,"Cardiometabolic_II","B13805","20212097_SS211647","PASS",-3.6645,0.582,"Plate control","PASS","PFC",1.49692298679269,"24h","24h"
8818,"PFC_05",49,"OID30378","P98095","FBLN2",0,"Cardiometabolic_II","B13805","20212097_SS211647","PASS",-3.6645,0.4969,"Plate control","PASS","PFC",1.41117802461169,"Baseline","Baseline"
8819,"PFC_13",50,"OID30378","P98095","FBLN2",0,"Cardiometabolic_II","B13805","20212097_SS211647","PASS",-3.6645,1.015,"Plate control","PASS","PFC",2.02090289297353,"1h","1h"
8820,"PFC_21",51,"OID30378","P98095","FBLN2",0,"Cardiometabolic_II","B13805","20212097_SS211647","PASS",-3.6645,0.2263,"Plate control","PASS","PFC",1.16983089825816,"24h","24h"
8821,"PFC_06",61,"OID30378","P98095","FBLN2",0,"Cardiometabolic_II","B13805","20212097_SS211647","PASS",-3.6645,0.094,"Plate control","PASS","PFC",1.06732533803996,"Baseline","Baseline"
8822,"PFC_14",62,"OID30378","P98095","FBLN2",0,"Cardiometabolic_II","B13805","20212097_SS211647","PASS",-3.6645,0.6806,"Plate control","PASS","PFC",1.60280620494647,"1h","1h"
8823,"PFC_22",63,"OID30378","P98095","FBLN2",0,"Cardiometabolic_II","B13805","20212097_SS211647","PASS",-3.6645,0.9209,"Plate control","PASS","PFC",1.89329602464498,"24h","24h"
8824,"PFC_07",73,"OID30378","P98095","FBLN2",0,"Cardiometabolic_II","B13805","20212097_SS211647","PASS",-3.6645,0.3648,"Plate control","PASS","PFC",1.28770310358051,"Baseline","Baseline"
8825,"PFC_15",74,"OID30378","P98095","FBLN2",0,"Cardiometabolic_II","B13805","20212097_SS211647","PASS",-3.6645,0.3292,"Plate control","PASS","PFC",1.25631653152441,"1h","1h"
8826,"PFC_23",75,"OID30378","P98095","FBLN2",0,"Cardiometabolic_II","B13805","20212097_SS211647","PASS",-3.6645,0.5882,"Plate control","PASS","PFC",1.50336987493829,"24h","24h"
8827,"PFC_08",85,"OID30378","P98095","FBLN2",0,"Cardiometabolic_II","B13805","20212097_SS211647","PASS",-3.6645,-0.2371,"Plate control","PASS","PFC",0.848449089658215,"Baseline","Baseline"
8828,"PFC_16",86,"OID30378","P98095","FBLN2",0,"Cardiometabolic_II","B13805","20212097_SS211647","PASS",-3.6645,0.4792,"Plate control","PASS","PFC",1.39397047063364,"1h","1h"
8829,"PFC_24",87,"OID30378","P98095","FBLN2",0,"Cardiometabolic_II","B13805","20212097_SS211647","PASS",-3.6645,0.3622,"Plate control","PASS","PFC",1.28538451724942,"24h","24h"
8830,"PFC_01",1,"OID30379","P02461","COL3A1",0,"Cardiometabolic_II","B13805","20212097_SS211647","PASS",-2.3393,0.4764,"Plate control","PASS","PFC",1.39126765954208,"Baseline","Baseline"
8831,"PFC_09",2,"OID30379","P02461","COL3A1",0,"Cardiometabolic_II","B13805","20212097_SS211647","PASS",-2.3393,0.2311,"Plate control","PASS","PFC",1.17372953219341,"Baseline","Baseline"
8832,"PFC_17",3,"OID30379","P02461","COL3A1",0,"Cardiometabolic_II","B13805","20212097_SS211647","PASS",-2.3393,0.0167,"Plate control","PASS","PFC",1.01164281394404,"1h","1h"
8833,"PFC_25",4,"OID30379","P02461","COL3A1",0,"Cardiometabolic_II","B13805","20212097_SS211647","PASS",-2.3393,0.7771,"Plate control","PASS","PFC",1.71368268885293,"24h","24h"
8834,"PFC_02",13,"OID30379","P02461","COL3A1",0,"Cardiometabolic_II","B13805","20212097_SS211647","PASS",-2.3393,0.4332,"Plate control","PASS","PFC",1.35022515310055,"Baseline","Baseline"
8835,"PFC_10",14,"OID30379","P02461","COL3A1",0,"Cardiometabolic_II","B13805","20212097_SS211647","PASS",-2.3393,0.5196,"Plate control","PASS","PFC",1.4335577263117,"1h","1h"
8836,"PFC_18",15,"OID30379","P02461","COL3A1",0,"Cardiometabolic_II","B13805","20212097_SS211647","PASS",-2.3393,0.2884,"Plate control","PASS","PFC",1.22128507785521,"1h","1h"
8837,"PFC_26",16,"OID30379","P02461","COL3A1",0,"Cardiometabolic_II","B13805","20212097_SS211647","PASS",-2.3393,0.4577,"Plate control","PASS","PFC",1.37335062350141,"24h","24h"
8838,"PFC_03",25,"OID30379","P02461","COL3A1",0,"Cardiometabolic_II","B13805","20212097_SS211647","PASS",-2.3393,1.3192,"Plate control","PASS","PFC",2.49527703870075,"Baseline","Baseline"
8839,"PFC_11",26,"OID30379","P02461","COL3A1",0,"Cardiometabolic_II","B13805","20212097_SS211647","PASS",-2.3393,0.2123,"Plate control","PASS","PFC",1.15853369145698,"1h","1h"
8840,"PFC_19",27,"OID30379","P02461","COL3A1",0,"Cardiometabolic_II","B13805","20212097_SS211647","PASS",-2.3393,0.6928,"Plate control","PASS","PFC",1.61641763901234,"24h","24h"
8841,"PFC_27",28,"OID30379","P02461","COL3A1",0,"Cardiometabolic_II","B13805","20212097_SS211647","PASS",-2.3393,0.3661,"Plate control","PASS","PFC",1.28886396463121,"24h","24h"
8842,"PFC_04",37,"OID30379","P02461","COL3A1",0,"Cardiometabolic_II","B13805","20212097_SS211647","PASS",-2.3393,0.4263,"Plate control","PASS","PFC",1.34378282845386,"Baseline","Baseline"
8843,"PFC_12",38,"OID30379","P02461","COL3A1",0,"Cardiometabolic_II","B13805","20212097_SS211647","PASS",-2.3393,1.269,"Plate control","PASS","PFC",2.40994462998957,"1h","1h"
8844,"PFC_20",39,"OID30379","P02461","COL3A1",0,"Cardiometabolic_II","B13805","20212097_SS211647","PASS",-2.3393,0.3353,"Plate control","PASS","PFC",1.26163973216297,"24h","24h"
8845,"PFC_05",49,"OID30379","P02461","COL3A1",0,"Cardiometabolic_II","B13805","20212097_SS211647","PASS",-2.3393,0.7083,"Plate control","PASS","PFC",1.63387770221423,"Baseline","Baseline"
8846,"PFC_13",50,"OID30379","P02461","COL3A1",0,"Cardiometabolic_II","B13805","20212097_SS211647","PASS",-2.3393,0.2237,"Plate control","PASS","PFC",1.1677245478713,"1h","1h"
8847,"PFC_21",51,"OID30379","P02461","COL3A1",0,"Cardiometabolic_II","B13805","20212097_SS211647","PASS",-2.3393,1.3148,"Plate control","PASS","PFC",2.48767841723537,"24h","24h"
8848,"PFC_06",61,"OID30379","P02461","COL3A1",0,"Cardiometabolic_II","B13805","20212097_SS211647","PASS",-2.3393,0.5996,"Plate control","PASS","PFC",1.51529637889762,"Baseline","Baseline"

Supplemental table 1_Olink proximity extension assay (PEA) data

,"SampleID","Index","OlinkID","UniProt","Assay","MissingFreq","Panel","Panel_Lot_Nr","PlateID","QC_Warning","LOD","NPX","Normalization","Assay_Warning","condition","Value","group","alt_group"
8849,"PFC_14",62,"OID30379","P02461","COL3A1",0,"Cardiometabolic_II","B13805","20212097_SS211647","PASS",-2.3393,0.531,"Plate control","PASS","PFC",1.44493039792353,"1h","1h"
8850,"PFC_22",63,"OID30379","P02461","COL3A1",0,"Cardiometabolic_II","B13805","20212097_SS211647","PASS",-2.3393,0.1735,"Plate control","PASS","PFC",1.12779120706963,"24h","24h"
8851,"PFC_07",73,"OID30379","P02461","COL3A1",0,"Cardiometabolic_II","B13805","20212097_SS211647","PASS",-2.3393,0.4469,"Plate control","PASS","PFC",1.36310812055283,"Baseline","Baseline"
8852,"PFC_15",74,"OID30379","P02461","COL3A1",0,"Cardiometabolic_II","B13805","20212097_SS211647","PASS",-2.3393,0.4704,"Plate control","PASS","PFC",1.38549355524328,"1h","1h"
8853,"PFC_23",75,"OID30379","P02461","COL3A1",0,"Cardiometabolic_II","B13805","20212097_SS211647","PASS",-2.3393,0.6388,"Plate control","PASS","PFC",1.55703351648261,"24h","24h"
8854,"PFC_08",85,"OID30379","P02461","COL3A1",0,"Cardiometabolic_II","B13805","20212097_SS211647","PASS",-2.3393,0.3624,"Plate control","PASS","PFC",1.28556272173214,"Baseline","Baseline"
8855,"PFC_16",86,"OID30379","P02461","COL3A1",0,"Cardiometabolic_II","B13805","20212097_SS211647","PASS",-2.3393,0.4015,"Plate control","PASS","PFC",1.320880544009,"1h","1h"
8856,"PFC_24",87,"OID30379","P02461","COL3A1",0,"Cardiometabolic_II","B13805","20212097_SS211647","PASS",-2.3393,0.7092,"Plate control","PASS","PFC",1.63489728615635,"24h","24h"
8857,"PFC_01",1,"OID30380","P08575","PTPRC",0,"Cardiometabolic_II","B13805","20212097_SS211647","PASS",-2.869,0.2184,"Plate control","PASS","PFC",1.16344257160893,"Baseline","Baseline"
8858,"PFC_09",2,"OID30380","P08575","PTPRC",0,"Cardiometabolic_II","B13805","20212097_SS211647","PASS",-2.869,0.901,"Plate control","PASS","PFC",1.86735988983063,"Baseline","Baseline"
8859,"PFC_17",3,"OID30380","P08575","PTPRC",0,"Cardiometabolic_II","B13805","20212097_SS211647","PASS",-2.869,-0.0294,"Plate control","PASS","PFC",0.979827711751162,"1h","1h"
8860,"PFC_25",4,"OID30380","P08575","PTPRC",0,"Cardiometabolic_II","B13805","20212097_SS211647","PASS",-2.869,0.0481,"Plate control","PASS","PFC",1.03390239841756,"24h","24h"
8861,"PFC_02",13,"OID30380","P08575","PTPRC",0,"Cardiometabolic_II","B13805","20212097_SS211647","PASS",-2.869,0.2794,"Plate control","PASS","PFC",1.21369001992948,"Baseline","Baseline"
8862,"PFC_10",14,"OID30380","P08575","PTPRC",0,"Cardiometabolic_II","B13805","20212097_SS211647","PASS",-2.869,0.5605,"Plate control","PASS","PFC",1.47478024861664,"1h","1h"
8863,"PFC_18",15,"OID30380","P08575","PTPRC",0,"Cardiometabolic_II","B13805","20212097_SS211647","PASS",-2.869,0.646,"Plate control","PASS","PFC",1.56482356347302,"1h","1h"
8864,"PFC_26",16,"OID30380","P08575","PTPRC",0,"Cardiometabolic_II","B13805","20212097_SS211647","PASS",-2.869,-0.0115,"Plate control","PASS","PFC",0.992060493132249,"24h","24h"
8865,"PFC_03",25,"OID30380","P08575","PTPRC",0,"Cardiometabolic_II","B13805","20212097_SS211647","PASS",-2.869,-0.2708,"Plate control","PASS","PFC",0.82885980089061,"Baseline","Baseline"
8866,"PFC_11",26,"OID30380","P08575","PTPRC",0,"Cardiometabolic_II","B13805","20212097_SS211647","PASS",-2.869,0.2803,"Plate control","PASS","PFC",1.21444739537646,"1h","1h"
8867,"PFC_19",27,"OID30380","P08575","PTPRC",0,"Cardiometabolic_II","B13805","20212097_SS211647","PASS",-2.869,0.348,"Plate control","PASS","PFC",1.27279493527594,"24h","24h"
8868,"PFC_27",28,"OID30380","P08575","PTPRC",0,"Cardiometabolic_II","B13805","20212097_SS211647","PASS",-2.869,0.8142,"Plate control","PASS","PFC",1.75832286023514,"24h","24h"
8869,"PFC_04",37,"OID30380","P08575","PTPRC",0,"Cardiometabolic_II","B13805","20212097_SS211647","PASS",-2.869,0.3423,"Plate control","PASS","PFC",1.26777612125973,"Baseline","Baseline"
8870,"PFC_12",38,"OID30380","P08575","PTPRC",0,"Cardiometabolic_II","B13805","20212097_SS211647","PASS",-2.869,-0.1109,"Plate control","PASS","PFC",0.92601020643923,"1h","1h"
8871,"PFC_20",39,"OID30380","P08575","PTPRC",0,"Cardiometabolic_II","B13805","20212097_SS211647","PASS",-2.869,0.4549,"Plate control","PASS","PFC",1.37068779284897,"24h","24h"
8872,"PFC_05",49,"OID30380","P08575","PTPRC",0,"Cardiometabolic_II","B13805","20212097_SS211647","PASS",-2.869,0.5314,"Plate control","PASS","PFC",1.44533107323892,"Baseline","Baseline"
8873,"PFC_13",50,"OID30380","P08575","PTPRC",0,"Cardiometabolic_II","B13805","20212097_SS211647","PASS",-2.869,0.4633,"Plate control","PASS","PFC",1.37869181407298,"1h","1h"
8874,"PFC_21",51,"OID30380","P08575","PTPRC",0,"Cardiometabolic_II","B13805","20212097_SS211647","PASS",-2.869,-0.1634,"Plate control","PASS","PFC",0.892918248522594,"24h","24h"
8875,"PFC_06",61,"OID30380","P08575","PTPRC",0,"Cardiometabolic_II","B13805","20212097_SS211647","PASS",-2.869,0.3086,"Plate control","PASS","PFC",1.23850526360339,"Baseline","Baseline"
8876,"PFC_14",62,"OID30380","P08575","PTPRC",0,"Cardiometabolic_II","B13805","20212097_SS211647","PASS",-2.869,0.5588,"Plate control","PASS","PFC",1.47304346447641,"1h","1h"
8877,"PFC_22",63,"OID30380","P08575","PTPRC",0,"Cardiometabolic_II","B13805","20212097_SS211647","PASS",-2.869,0.3185,"Plate control","PASS","PFC",1.24703330820196,"24h","24h"
8878,"PFC_07",73,"OID30380","P08575","PTPRC",0,"Cardiometabolic_II","B13805","20212097_SS211647","PASS",-2.869,0.34,"Plate control","PASS","PFC",1.26575659397028,"Baseline","Baseline"
8879,"PFC_15",74,"OID30380","P08575","PTPRC",0,"Cardiometabolic_II","B13805","20212097_SS211647","PASS",-2.869,0.3777,"Plate control","PASS","PFC",1.29926885899888,"1h","1h"
8880,"PFC_23",75,"OID30380","P08575","PTPRC",0,"Cardiometabolic_II","B13805","20212097_SS211647","PASS",-2.869,0.5168,"Plate control","PASS","PFC",1.43077815830456,"24h","24h"
8881,"PFC_08",85,"OID30380","P08575","PTPRC",0,"Cardiometabolic_II","B13805","20212097_SS211647","PASS",-2.869,0.0547,"Plate control","PASS","PFC",1.03864310107911,"Baseline","Baseline"
8882,"PFC_16",86,"OID30380","P08575","PTPRC",0,"Cardiometabolic_II","B13805","20212097_SS211647","PASS",-2.869,0.3639,"Plate control","PASS","PFC",1.28690004309607,"1h","1h"
8883,"PFC_24",87,"OID30380","P08575","PTPRC",0,"Cardiometabolic_II","B13805","20212097_SS211647","PASS",-2.869,0.0949,"Plate control","PASS","PFC",1.06799137796094,"24h","24h"
8884,"PFC_01",1,"OID30381","P13727","PRG2",0,"Cardiometabolic_II","B13805","20212097_SS211647","PASS",-3.8724,0.7531,"Plate control","PASS","PFC",1.68541047870973,"Baseline","Baseline"
8885,"PFC_09",2,"OID30381","P13727","PRG2",0,"Cardiometabolic_II","B13805","20212097_SS211647","PASS",-3.8724,1.8908,"Plate control","PASS","PFC",3.70840805558827,"Baseline","Baseline"
8886,"PFC_17",3,"OID30381","P13727","PRG2",0,"Cardiometabolic_II","B13805","20212097_SS211647","PASS",-3.8724,0.6617,"Plate control","PASS","PFC",1.58194561182406,"1h","1h"
8887,"PFC_25",4,"OID30381","P13727","PRG2",0,"Cardiometabolic_II","B13805","20212097_SS211647","PASS",-3.8724,1.1748,"Plate control","PASS","PFC",2.25761581591018,"24h","24h"
8888,"PFC_02",13,"OID30381","P13727","PRG2",0,"Cardiometabolic_II","B13805","20212097_SS211647","PASS",-3.8724,1.3694,"Plate control","PASS","PFC",2.58363093590832,"Baseline","Baseline"
8889,"PFC_10",14,"OID30381","P13727","PRG2",0,"Cardiometabolic_II","B13805","20212097_SS211647","PASS",-3.8724,0.6523,"Plate control","PASS","PFC",1.57167181932446,"1h","1h"
8890,"PFC_18",15,"OID30381","P13727","PRG2",0,"Cardiometabolic_II","B13805","20212097_SS211647","PASS",-3.8724,1.7715,"Plate control","PASS","PFC",3.41408741996805,"1h","1h"
8891,"PFC_26",16,"OID30381","P13727","PRG2",0,"Cardiometabolic_II","B13805","20212097_SS211647","PASS",-3.8724,0.6492,"Plate control","PASS","PFC",1.56829830539203,"24h","24h"
8892,"PFC_03",25,"OID30381","P13727","PRG2",0,"Cardiometabolic_II","B13805","20212097_SS211647","PASS",-3.8724,1.3385,"Plate control","PASS","PFC",2.52888248893059,"Baseline","Baseline"
8893,"PFC_11",26,"OID30381","P13727","PRG2",0,"Cardiometabolic_II","B13805","20212097_SS211647","PASS",-3.8724,1.4393,"Plate control","PASS","PFC",2.71189251716758,"1h","1h"
8894,"PFC_19",27,"OID30381","P13727","PRG2",0,"Cardiometabolic_II","B13805","20212097_SS211647","PASS",-3.8724,0.6164,"Plate control","PASS","PFC",1.53304495160309,"24h","24h"
8895,"PFC_27",28,"OID30381","P13727","PRG2",0,"Cardiometabolic_II","B13805","20212097_SS211647","PASS",-3.8724,1.9713,"Plate control","PASS","PFC",3.92121296990956,"24h","24h"
8896,"PFC_04",37,"OID30381","P13727","PRG2",0,"Cardiometabolic_II","B13805","20212097_SS211647","PASS",-3.8724,1.5951,"Plate control","PASS","PFC",3.02115457434573,"Baseline","Baseline"
8897,"PFC_12",38,"OID30381","P13727","PRG2",0,"Cardiometabolic_II","B13805","20212097_SS211647","PASS",-3.8724,1.3472,"Plate control","PASS","PFC",2.54417868709787,"1h","1h"
8898,"PFC_20",39,"OID30381","P13727","PRG2",0,"Cardiometabolic_II","B13805","20212097_SS211647","PASS",-3.8724,1.3524,"Plate control","PASS","PFC",2.5533653827316,"24h","24h"
8899,"PFC_05",49,"OID30381","P13727","PRG2",0,"Cardiometabolic_II","B13805","20212097_SS211647","PASS",-3.8724,1.051,"Plate control","PASS","PFC",2.07196552712004,"Baseline","Baseline"
8900,"PFC_13",50,"OID30381","P13727","PRG2",0,"Cardiometabolic_II","B13805","20212097_SS211647","PASS",-3.8724,1.6198,"Plate control","PASS","PFC",3.073324279831,"1h","1h"
8901,"PFC_21",51,"OID30381","P13727","PRG2",0,"Cardiometabolic_II","B13805","20212097_SS211647","PASS",-3.8724,1.4454,"Plate control","PASS","PFC",2.72338321048958,"24h","24h"
8902,"PFC_06",61,"OID30381","P13727","PRG2",0,"Cardiometabolic_II","B13805","20212097_SS211647","PASS",-3.8724,1.5312,"Plate control","PASS","PFC",2.89026144338975,"Baseline","Baseline"
8903,"PFC_14",62,"OID30381","P13727","PRG2",0,"Cardiometabolic_II","B13805","20212097_SS211647","PASS",-3.8724,1.0342,"Plate control","PASS","PFC",2.04797769117115,"1h","1h"
8904,"PFC_22",63,"OID30381","P13727","PRG2",0,"Cardiometabolic_II","B13805","20212097_SS211647","PASS",-3.8724,1.4941,"Plate control","PASS","PFC",2.81688369855678,"24h","24h"
8905,"PFC_07",73,"OID30381","P13727","PRG2",0,"Cardiometabolic_II","B13805","20212097_SS211647","PASS",-3.8724,1.1398,"Plate control","PASS","PFC",2.20350473995906,"Baseline","Baseline"
8906,"PFC_15",74,"OID30381","P13727","PRG2",0,"Cardiometabolic_II","B13805","20212097_SS211647","PASS",-3.8724,1.6506,"Plate control","PASS","PFC",3.13964186044569,"1h","1h"
8907,"PFC_23",75,"OID30381","P13727","PRG2",0,"Cardiometabolic_II","B13805","20212097_SS211647","PASS",-3.8724,1.0858,"Plate control","PASS","PFC",2.12255214929534,"24h","24h"
8908,"PFC_08",85,"OID30381","P13727","PRG2",0,"Cardiometabolic_II","B13805","20212097_SS211647","PASS",-3.8724,0.5213,"Plate control","PASS","PFC",1.43524795500026,"Baseline","Baseline"
8909,"PFC_16",86,"OID30381","P13727","PRG2",0,"Cardiometabolic_II","B13805","20212097_SS211647","PASS",-3.8724,1.1471,"Plate control","PASS","PFC",2.21468267375355,"1h","1h"
8910,"PFC_24",87,"OID30381","P13727","PRG2",0,"Cardiometabolic_II","B13805","20212097_SS211647","PASS",-3.8724,1.7589,"Plate control","PASS","PFC",3.38439978955354,"24h","24h"
8911,"PFC_01",1,"OID30382","P35579","MYH9",0,"Cardiometabolic_II","B13805","20212097_SS211647","PASS",-0.9351,4.5672,"Plate control","PASS","PFC",23.7063229347209,"Baseline","Baseline"
8912,"PFC_09",2,"OID30382","P35579","MYH9",0,"Cardiometabolic_II","B13805","20212097_SS211647","PASS",-0.9351,5.7632,"Plate control","PASS","PFC",54.3120344340216,"Baseline","Baseline"
8913,"PFC_17",3,"OID30382","P35579","MYH9",0,"Cardiometabolic_II","B13805","20212097_SS211647","PASS",-0.9351,5.4029,"Plate control","PASS","PFC",42.3092146116401,"1h","1h"
8914,"PFC_25",4,"OID30382","P35579","MYH9",0,"Cardiometabolic_II","B13805","20212097_SS211647","PASS",-0.9351,4.5679,"Plate control","PASS","PFC",23.7178281052995,"24h","24h"
8915,"PFC_02",13,"OID30382","P35579","MYH9",0,"Cardiometabolic_II","B13805","20212097_SS211647","PASS",-0.9351,4.3875,"Plate control","PASS","PFC",20.9299941029493,"Baseline","Baseline"
8916,"PFC_10",14,"OID30382","P35579","MYH9",0,"Cardiometabolic_II","B13805","20212097_SS211647","PASS",-0.9351,5.2375,"Plate control","PASS","PFC",37.72633373545,"1h","1h"
8917,"PFC_18",15,"OID30382","P35579","MYH9",0,"Cardiometabolic_II","B13805","20212097_SS211647","PASS",-0.9351,4.5374,"Plate control","PASS","PFC",23.2216728477004,"1h","1h"
8918,"PFC_26",16,"OID30382","P35579","MYH9",0,"Cardiometabolic_II","B13805","20212097_SS211647","PASS",-0.9351,4.3644,"Plate control","PASS","PFC",20.5975380153623,"24h","24h"
8919,"PFC_03",25,"OID30382","P35579","MYH9",0,"Cardiometabolic_II","B13805","20212097_SS211647","PASS",-0.9351,1.2005,"Plate control","PASS","PFC",2.29819306500999,"Baseline","Baseline"
8920,"PFC_11",26,"OID30382","P35579","MYH9",0,"Cardiometabolic_II","B13805","20212097_SS211647","PASS",-0.9351,4.9176,"Plate control","PASS","PFC",30.2235244098321,"1h","1h"
8921,"PFC_19",27,"OID30382","P35579","MYH9",0,"Cardiometabolic_II","B13805","20212097_SS211647","PASS",-0.9351,4.3495,"Plate control","PASS","PFC",20.3859035968575,"24h","24h"
8922,"PFC_27",28,"OID30382","P35579","MYH9",0,"Cardiometabolic_II","B13805","20212097_SS211647","PASS",-0.9351,5.4764,"Plate control","PASS","PFC",44.5205651053465,"24h","24h"
8923,"PFC_04",37,"OID30382","P35579","MYH9",0,"Cardiometabolic_II","B13805","20212097_SS211647","PASS",-0.9351,3.9126,"Plate control","PASS","PFC",15.0594794794793,"Baseline","Baseline"
8924,"PFC_12",38,"OID30382","P35579","MYH9",0,"Cardiometabolic_II","B13805","20212097_SS211647","PASS",-0.9351,3.1693,"Plate control","PASS","PFC",8.99610188254,"1h","1h"
8925,"PFC_20",39,"OID30382","P35579","MYH9",0,"Cardiometabolic_II","B13805","20212097_SS211647","PASS",-0.9351,5.4892,"Plate control","PASS","PFC",44.9173216646312,"24h","24h"
8926,"PFC_05",49,"OID30382","P35579","MYH9",0,"Cardiometabolic_II","B13805","20212097_SS211647","PASS",-0.9351,4.0503,"Plate control","PASS","PFC",16.5676835762349,"Baseline","Baseline"
8927,"PFC_13",50,"OID30382","P35579","MYH9",0,"Cardiometabolic_II","B13805","20212097_SS211647","PASS",-0.9351,4.7805,"Plate control","PASS","PFC",27.4836174115856,"1h","1h"
8928,"PFC_21",51,"OID30382","P35579","MYH9",0,"Cardiometabolic_II","B13805","20212097_SS211647","PASS",-0.9351,3.2513,"Plate control","PASS","PFC",9.5222334573754,"24h","24h"
8929,"PFC_06",61,"OID30382","P35579","MYH9",0,"Cardiometabolic_II","B13805","20212097_SS211647","PASS",-0.9351,4.0801,"Plate control","PASS","PFC",16.9134609601302,"Baseline","Baseline"
8930,"PFC_14",62,"OID30382","P35579","MYH9",0,"Cardiometabolic_II","B13805","20212097_SS211647","PASS",-0.9351,5.0821,"Plate control","PASS","PFC",33.8738485108912,"1h","1h"
8931,"PFC_22",63,"OID30382","P35579","MYH9",0,"Cardiometabolic_II","B13805","20212097_SS211647","PASS",-0.9351,3.9416,"Plate control","PASS","PFC",15.3652570787692,"24h","24h"
8932,"PFC_07",73,"OID30382","P35579","MYH9",0,"Cardiometabolic_II","B13805","20212097_SS211647","PASS",-0.9351,4.1725,"Plate control","PASS","PFC",18.0321560421911,"Baseline","Baseline"
8933,"PFC_15",74,"OID30382","P35579","MYH9",0,"Cardiometabolic_II","B13805","20212097_SS211647","PASS",-0.9351,2.7487,"Plate control","PASS","PFC",6.72111225653989,"1h","1h"
8934,"PFC_23",75,"OID30382","P35579","MYH9",0,"Cardiometabolic_II","B13805","20212097_SS211647","PASS",-0.9351,3.8537,"Plate control","PASS","PFC",14.457037059086,"24h","24h"
8935,"PFC_08",85,"OID30382","P35579","MYH9",0,"Cardiometabolic_II","B13805","20212097_SS211647","PASS",-0.9351,3.0705,"Plate control","PASS","PFC",8.40064440602091,"Baseline","Baseline"
8936,"PFC_16",86,"OID30382","P35579","MYH9",0,"Cardiometabolic_II","B13805","20212097_SS211647","PASS",-0.9351,4.0586,"Plate control","PASS","PFC",16.6632741829912,"1h","1h"
8937,"PFC_24",87,"OID30382","P35579","MYH9",0,"Cardiometabolic_II","B13805","20212097_SS211647","PASS",-0.9351,3.9169,"Plate control","PASS","PFC",15.1044317106929,"24h","24h"
8938,"PFC_01",1,"OID30383","Q9Y2Y8","PRG3",0,"Cardiometabolic_II","B13805","20212097_SS211647","PASS",-3.5989,-0.2599,"Plate control","PASS","PFC",0.835145805318205,"Baseline","Baseline"
8939,"PFC_09",2,"OID30383","Q9Y2Y8","PRG3",0,"Cardiometabolic_II","B13805","20212097_SS211647","PASS",-3.5989,1.1352,"Plate control","PASS","PFC",2.1964901046535,"Baseline","Baseline"
8940,"PFC_17",3,"OID30383","Q9Y2Y8","PRG3",0,"Cardiometabolic_II","B13805","20212097_SS211647","PASS",-3.5989,-0.0061,"Plate control","PASS","PFC",0.995780728441836,"1h","1h"
8941,"PFC_25",4,"OID30383","Q9Y2Y8","PRG3",0,"Cardiometabolic_II","B13805","20212097_SS211647","PASS",-3.5989,0.7296,"Plate control","PASS","PFC",1.65817928297087,"24h","24h"
8942,"PFC_02",13,"OID30383","Q9Y2Y8","PRG3",0,"Cardiometabolic_II","B13805","20212097_SS211647","PASS",-3.5989,0.6563,"Plate control","PASS","PFC",1.57603546538835,"Baseline","Baseline"
8943,"PFC_10",14,"OID30383","Q9Y2Y8","PRG3",0,"Cardiometabolic_II","B13805","20212097_SS211647","PASS",-3.5989,-0.0154,"Plate control","PASS","PFC",0.989382303361927,"1h","1h"
8944,"PFC_18",15,"OID30383","Q9Y2Y8","PRG3",0,"Cardiometabolic_II","B13805","20212097_SS211647","PASS",-3.5989,1.2016,"Plate control","PASS","PFC",2.29994601785267,"1h","1h"
8945,"PFC_26",16,"OID30383","Q9Y2Y8","PRG3",0,"Cardiometabolic_II","B13805","20212097_SS211647","PASS",-3.5989,0.0125,"Plate control","PASS","PFC",1.0087019837904,"24h","24h"
8946,"PFC_03",25,"OID30383","Q9Y2Y8","PRG3",0,"Cardiometabolic_II","B13805","20212097_SS211647","PASS",-3.5989,0.3049,"Plate control","PASS","PFC",1.23533300740015,"Baseline","Baseline"
8947,"PFC_11",26,"OID30383","Q9Y2Y8","PRG3",0,"Cardiometabolic_II","B13805","20212097_SS211647","PASS",-3.5989,0.8831,"Plate control","PASS","PFC",1.84433407089091,"1h","1h"
8948,"PFC_19",27,"OID30383","Q9Y2Y8","PRG3",0,"Cardiometabolic_II","B13805","20212097_SS211647","PASS",-3.5989,-0.2313,"Plate control","PASS","PFC",0.851866935906447,"24h","24h"
8949,"PFC_27",28,"OID30383","Q9Y2Y8","PRG3",0,"Cardiometabolic_II","B13805","20212097_SS211647","PASS",-3.5989,1.5005,"Plate control","PASS","PFC",2.82940755277508,"24h","24h"
8950,"PFC_04",37,"OID30383","Q9Y2Y8","PRG3",0,"Cardiometabolic_II","B13805","20212097_SS211647","PASS",-3.5989,0.5596,"Plate control","PASS","PFC",1.47386051973069,"Baseline","Baseline"
8951,"PFC_12",38,"OID30383","Q9Y2Y8","PRG3",0,"Cardiometabolic_II","B13805","20212097_SS211647","PASS",-3.5989,0.4156,"Plate control","PASS","PFC",1.33385329574423,"1h","1h"
8952,"PFC_20",39,"OID30383","Q9Y2Y8","PRG3",0,"Cardiometabolic_II","B13805","20212097_SS211647","PASS",-3.5989,0.7741,"Plate control","PASS","PFC",1.71012288836172,"24h","24h"
8953,"PFC_05",49,"OID30383","Q9Y2Y8","PRG3",0,"Cardiometabolic_II","B13805","20212097_SS211647","PASS",-3.5989,0.5141,"Plate control","PASS","PFC",1.42810296480882,"Baseline","Baseline"
8954,"PFC_13",50,"OID30383","Q9Y2Y8","PRG3",0,"Cardiometabolic_II","B13805","20212097_SS211647","PASS",-3.5989,0.4827,"Plate control","PASS","PFC",1.39735636954925,"1h","1h"
8955,"PFC_21",51,"OID30383","Q9Y2Y8","PRG3",0,"Cardiometabolic_II","B13805","20212097_SS211647","PASS",-3.5989,0.319,"Plate control","PASS","PFC",1.24746557191405,"24h","24h"
8956,"PFC_06",61,"OID30383","Q9Y2Y8","PRG3",0,"Cardiometabolic_II","B13805","20212097_SS211647","PASS",-3.5989,0.8971,"Plate control","PASS","PFC",1.86231872128387,"Baseline","Baseline"
8957,"PFC_14",62,"OID30383","Q9Y2Y8","PRG3",0,"Cardiometabolic_II","B13805","20212097_SS211647","PASS",-3.5989,0.4012,"Plate control","PASS","PFC",1.32060590317749,"1h","1h"
8958,"PFC_22",63,"OID30383","Q9Y2Y8","PRG3",0,"Cardiometabolic_II","B13805","20212097_SS211647","PASS",-3.5989,0.3312,"Plate control","PASS","PFC",1.25805936380801,"24h","24h"
8959,"PFC_07",73,"OID30383","Q9Y2Y8","PRG3",0,"Cardiometabolic_II","B13805","20212097_SS211647","PASS",-3.5989,0.915,"Plate control","PASS","PFC",1.88556907183648,"Baseline","Baseline"
8960,"PFC_15",74,"OID30383","Q9Y2Y8","PRG3",0,"Cardiometabolic_II","B13805","20212097_SS211647","PASS",-3.5989,0.9164,"Plate control","PASS","PFC",1.88739972757278,"1h","1h"

Supplemental table 1_Olink proximity extension assay (PEA) data

,"SampleID","Index","OlinkID","UniProt","Assay","MissingFreq","Panel","Panel_Lot_Nr","PlateID","QC_Warning","LOD","NPX","Normalization","Assay_Warning","condition","Value","group","alt_group"
8961,"PFC_23",75,"OID30383","Q9Y2Y8","PRG3",0,"Cardiometabolic_II","B13805","20212097_SS211647","PASS",-3.5989,0.4192,"Plate control","PASS","PFC",1.33718585587781,"24h","24h"
8962,"PFC_08",85,"OID30383","Q9Y2Y8","PRG3",0,"Cardiometabolic_II","B13805","20212097_SS211647","PASS",-3.5989,0.0903,"Plate control","PASS","PFC",1.06459153502436,"Baseline","Baseline"
8963,"PFC_16",86,"OID30383","Q9Y2Y8","PRG3",0,"Cardiometabolic_II","B13805","20212097_SS211647","PASS",-3.5989,0.7173,"Plate control","PASS","PFC",1.64410222049165,"1h","1h"
8964,"PFC_24",87,"OID30383","Q9Y2Y8","PRG3",0,"Cardiometabolic_II","B13805","20212097_SS211647","PASS",-3.5989,0.9496,"Plate control","PASS","PFC",1.93133710326236,"24h","24h"
8965,"PFC_01",1,"OID30384","P32971","TNFSF8",0,"Cardiometabolic_II","B13805","20212097_SS211647","PASS",-2.1277,0.3194,"Plate control","PASS","PFC",1.24781149076397,"Baseline","Baseline"
8966,"PFC_09",2,"OID30384","P32971","TNFSF8",0,"Cardiometabolic_II","B13805","20212097_SS211647","PASS",-2.1277,0.4967,"Plate control","PASS","PFC",1.41098240735735,"Baseline","Baseline"
8967,"PFC_17",3,"OID30384","P32971","TNFSF8",0,"Cardiometabolic_II","B13805","20212097_SS211647","PASS",-2.1277,0.157,"Plate control","PASS","PFC",1.1149662187277,"1h","1h"
8968,"PFC_25",4,"OID30384","P32971","TNFSF8",0,"Cardiometabolic_II","B13805","20212097_SS211647","PASS",-2.1277,0.3151,"Plate control","PASS","PFC",1.24409788460399,"24h","24h"
8969,"PFC_02",13,"OID30384","P32971","TNFSF8",0,"Cardiometabolic_II","B13805","20212097_SS211647","PASS",-2.1277,0.109,"Plate control","PASS","PFC",1.07848043169445,"Baseline","Baseline"
8970,"PFC_10",14,"OID30384","P32971","TNFSF8",0,"Cardiometabolic_II","B13805","20212097_SS211647","PASS",-2.1277,0.6086,"Plate control","PASS","PFC",1.52477883618359,"1h","1h"
8971,"PFC_18",15,"OID30384","P32971","TNFSF8",0,"Cardiometabolic_II","B13805","20212097_SS211647","PASS",-2.1277,0.4238,"Plate control","PASS","PFC",1.34145624666849,"1h","1h"
8972,"PFC_26",16,"OID30384","P32971","TNFSF8",0,"Cardiometabolic_II","B13805","20212097_SS211647","PASS",-2.1277,0.3642,"Plate control","PASS","PFC",1.28716767426223,"24h","24h"
8973,"PFC_03",25,"OID30384","P32971","TNFSF8",0,"Cardiometabolic_II","B13805","20212097_SS211647","PASS",-2.1277,0.1091,"Plate control","PASS","PFC",1.07855518885236,"Baseline","Baseline"
8974,"PFC_11",26,"OID30384","P32971","TNFSF8",0,"Cardiometabolic_II","B13805","20212097_SS211647","PASS",-2.1277,0.3468,"Plate control","PASS","PFC",1.27173669438199,"1h","1h"
8975,"PFC_19",27,"OID30384","P32971","TNFSF8",0,"Cardiometabolic_II","B13805","20212097_SS211647","PASS",-2.1277,0.4513,"Plate control","PASS","PFC",1.36727173854811,"24h","24h"
8976,"PFC_27",28,"OID30384","P32971","TNFSF8",0,"Cardiometabolic_II","B13805","20212097_SS211647","PASS",-2.1277,1.0431,"Plate control","PASS","PFC",2.06065073571479,"24h","24h"
8977,"PFC_04",37,"OID30384","P32971","TNFSF8",0,"Cardiometabolic_II","B13805","20212097_SS211647","PASS",-2.1277,0.2341,"Plate control","PASS","PFC",1.17617277354974,"Baseline","Baseline"
8978,"PFC_12",38,"OID30384","P32971","TNFSF8",0,"Cardiometabolic_II","B13805","20212097_SS211647","PASS",-2.1277,0.0804,"Plate control","PASS","PFC",1.05731114882278,"1h","1h"
8979,"PFC_20",39,"OID30384","P32971","TNFSF8",0,"Cardiometabolic_II","B13805","20212097_SS211647","PASS",-2.1277,0.2668,"Plate control","PASS","PFC",1.20313622434161,"24h","24h"
8980,"PFC_05",49,"OID30384","P32971","TNFSF8",0,"Cardiometabolic_II","B13805","20212097_SS211647","PASS",-2.1277,0.8902,"Plate control","PASS","PFC",1.8534330463499,"Baseline","Baseline"
8981,"PFC_13",50,"OID30384","P32971","TNFSF8",0,"Cardiometabolic_II","B13805","20212097_SS211647","PASS",-2.1277,0.0402,"Plate control","PASS","PFC",1.02825636337578,"1h","1h"
8982,"PFC_21",51,"OID30384","P32971","TNFSF8",0,"Cardiometabolic_II","B13805","20212097_SS211647","PASS",-2.1277,0.463,"Plate control","PASS","PFC",1.37840515297563,"24h","24h"
8983,"PFC_06",61,"OID30384","P32971","TNFSF8",0,"Cardiometabolic_II","B13805","20212097_SS211647","PASS",-2.1277,0.0677,"Plate control","PASS","PFC",1.04804451812538,"Baseline","Baseline"
8984,"PFC_14",62,"OID30384","P32971","TNFSF8",0,"Cardiometabolic_II","B13805","20212097_SS211647","PASS",-2.1277,0.6406,"Plate control","PASS","PFC",1.55897738498429,"1h","1h"
8985,"PFC_22",63,"OID30384","P32971","TNFSF8",0,"Cardiometabolic_II","B13805","20212097_SS211647","PASS",-2.1277,-0.0707,"Plate control","PASS","PFC",0.952175887322057,"24h","24h"
8986,"PFC_07",73,"OID30384","P32971","TNFSF8",0,"Cardiometabolic_II","B13805","20212097_SS211647","PASS",-2.1277,0.7064,"Plate control","PASS","PFC",1.63172733484699,"Baseline","Baseline"
8987,"PFC_15",74,"OID30384","P32971","TNFSF8",0,"Cardiometabolic_II","B13805","20212097_SS211647","PASS",-2.1277,-0.0921,"Plate control","PASS","PFC",0.938156166216765,"1h","1h"
8988,"PFC_23",75,"OID30384","P32971","TNFSF8",0,"Cardiometabolic_II","B13805","20212097_SS211647","PASS",-2.1277,0.632,"Plate control","PASS","PFC",1.54971186191834,"24h","24h"
8989,"PFC_08",85,"OID30384","P32971","TNFSF8",0,"Cardiometabolic_II","B13805","20212097_SS211647","PASS",-2.1277,0.0929,"Plate control","PASS","PFC",1.06651185330125,"Baseline","Baseline"
8990,"PFC_16",86,"OID30384","P32971","TNFSF8",0,"Cardiometabolic_II","B13805","20212097_SS211647","PASS",-2.1277,0.3329,"Plate control","PASS","PFC",1.25954267207638,"1h","1h"
8991,"PFC_24",87,"OID30384","P32971","TNFSF8",0,"Cardiometabolic_II","B13805","20212097_SS211647","PASS",-2.1277,0.3927,"Plate control","PASS","PFC",1.31284809795062,"24h","24h"
8992,"PFC_01",1,"OID30385","P30405","PPIF",0.2273,"Cardiometabolic_II","B13805","20212097_SS211647","PASS",0.364,0.8424,"Plate control","PASS","PFC",1.79303046383209,"Baseline","Baseline"
8993,"PFC_09",2,"OID30385","P30405","PPIF",0.2273,"Cardiometabolic_II","B13805","20212097_SS211647","PASS",0.364,2.0625,"Plate control","PASS","PFC",4.17709512970966,"Baseline","Baseline"
8994,"PFC_17",3,"OID30385","P30405","PPIF",0.2273,"Cardiometabolic_II","B13805","20212097_SS211647","PASS",0.364,2.4905,"Plate control","PASS","PFC",5.61972681438759,"1h","1h"
8995,"PFC_25",4,"OID30385","P30405","PPIF",0.2273,"Cardiometabolic_II","B13805","20212097_SS211647","PASS",0.364,1.449,"Plate control","PASS","PFC",2.73018743577033,"24h","24h"
8996,"PFC_02",13,"OID30385","P30405","PPIF",0.2273,"Cardiometabolic_II","B13805","20212097_SS211647","PASS",0.364,0.2206,"Plate control","PASS","PFC",1.16521808629132,"Baseline","Baseline"
8997,"PFC_10",14,"OID30385","P30405","PPIF",0.2273,"Cardiometabolic_II","B13805","20212097_SS211647","PASS",0.364,2.0115,"Plate control","PASS","PFC",4.03201218846114,"1h","1h"
8998,"PFC_18",15,"OID30385","P30405","PPIF",0.2273,"Cardiometabolic_II","B13805","20212097_SS211647","PASS",0.364,0.8605,"Plate control","PASS","PFC",1.81566746399581,"1h","1h"
8999,"PFC_26",16,"OID30385","P30405","PPIF",0.2273,"Cardiometabolic_II","B13805","20212097_SS211647","PASS",0.364,1.6093,"Plate control","PASS","PFC",3.05103768600348,"24h","24h"
9000,"PFC_03",25,"OID30385","P30405","PPIF",0.2273,"Cardiometabolic_II","B13805","20212097_SS211647","PASS",0.364,0.2001,"Plate control","PASS","PFC",1.14877797945918,"Baseline","Baseline"
9001,"PFC_11",26,"OID30385","P30405","PPIF",0.2273,"Cardiometabolic_II","B13805","20212097_SS211647","PASS",0.364,1.0191,"Plate control","PASS","PFC",2.02665427241921,"1h","1h"
9002,"PFC_19",27,"OID30385","P30405","PPIF",0.2273,"Cardiometabolic_II","B13805","20212097_SS211647","PASS",0.364,1.4416,"Plate control","PASS","PFC",2.71621936876808,"24h","24h"
9003,"PFC_27",28,"OID30385","P30405","PPIF",0.2273,"Cardiometabolic_II","B13805","20212097_SS211647","PASS",0.364,2.9771,"Plate control","PASS","PFC",7.87401794268903,"24h","24h"
9004,"PFC_04",37,"OID30385","P30405","PPIF",0.2273,"Cardiometabolic_II","B13805","20212097_SS211647","PASS",0.364,0.4222,"Plate control","PASS","PFC",1.33996934874781,"Baseline","Baseline"
9005,"PFC_12",38,"OID30385","P30405","PPIF",0.2273,"Cardiometabolic_II","B13805","20212097_SS211647","PASS",0.364,0.093,"Plate control","PASS","PFC",1.06658578083176,"1h","1h"
9006,"PFC_20",39,"OID30385","P30405","PPIF",0.2273,"Cardiometabolic_II","B13805","20212097_SS211647","PASS",0.364,1.6148,"Plate control","PASS","PFC",3.06269138557805,"24h","24h"
9007,"PFC_05",49,"OID30385","P30405","PPIF",0.2273,"Cardiometabolic_II","B13805","20212097_SS211647","PASS",0.364,0.4532,"Plate control","PASS","PFC",1.36907359383724,"Baseline","Baseline"
9008,"PFC_13",50,"OID30385","P30405","PPIF",0.2273,"Cardiometabolic_II","B13805","20212097_SS211647","PASS",0.364,1.5302,"Plate control","PASS","PFC",2.8882587609762,"1h","1h"
9009,"PFC_21",51,"OID30385","P30405","PPIF",0.2273,"Cardiometabolic_II","B13805","20212097_SS211647","PASS",0.364,0.4154,"Plate control","PASS","PFC",1.33366839723047,"24h","24h"
9010,"PFC_06",61,"OID30385","P30405","PPIF",0.2273,"Cardiometabolic_II","B13805","20212097_SS211647","PASS",0.364,1.059,"Plate control","PASS","PFC",2.08348685801644,"Baseline","Baseline"
9011,"PFC_14",62,"OID30385","P30405","PPIF",0.2273,"Cardiometabolic_II","B13805","20212097_SS211647","PASS",0.364,1.3352,"Plate control","PASS","PFC",2.52310456998274,"1h","1h"
9012,"PFC_22",63,"OID30385","P30405","PPIF",0.2273,"Cardiometabolic_II","B13805","20212097_SS211647","PASS",0.364,0.5195,"Plate control","PASS","PFC",1.43345836310579,"24h","24h"
9013,"PFC_07",73,"OID30385","P30405","PPIF",0.2273,"Cardiometabolic_II","B13805","20212097_SS211647","PASS",0.364,1.2042,"Plate control","PASS","PFC",2.3040946779055,"Baseline","Baseline"
9014,"PFC_15",74,"OID30385","P30405","PPIF",0.2273,"Cardiometabolic_II","B13805","20212097_SS211647","PASS",0.364,0.019,"Plate control","PASS","PFC",1.01325690015909,"1h","1h"
9015,"PFC_23",75,"OID30385","P30405","PPIF",0.2273,"Cardiometabolic_II","B13805","20212097_SS211647","PASS",0.364,0.4103,"Plate control","PASS","PFC",1.32896213527297,"24h","24h"
9016,"PFC_08",85,"OID30385","P30405","PPIF",0.2273,"Cardiometabolic_II","B13805","20212097_SS211647","PASS",0.364,0.6576,"Plate control","PASS","PFC",1.57745625732495,"Baseline","Baseline"
9017,"PFC_16",86,"OID30385","P30405","PPIF",0.2273,"Cardiometabolic_II","B13805","20212097_SS211647","PASS",0.364,0.7615,"Plate control","PASS","PFC",1.69525229776813,"1h","1h"
9018,"PFC_24",87,"OID30385","P30405","PPIF",0.2273,"Cardiometabolic_II","B13805","20212097_SS211647","PASS",0.364,1.3086,"Plate control","PASS","PFC",2.47701052720678,"24h","24h"
9019,"PFC_01",1,"OID30386","Q6IBS0","TWF2",0,"Cardiometabolic_II","B13805","20212097_SS211647","PASS",-1.5638,2.4952,"Plate control","PASS","PFC",5.63806456810592,"Baseline","Baseline"
9020,"PFC_09",2,"OID30386","Q6IBS0","TWF2",0,"Cardiometabolic_II","B13805","20212097_SS211647","PASS",-1.5638,3.0094,"Plate control","PASS","PFC",8.05229484869947,"Baseline","Baseline"
9021,"PFC_17",3,"OID30386","Q6IBS0","TWF2",0,"Cardiometabolic_II","B13805","20212097_SS211647","PASS",-1.5638,4.3443,"Plate control","PASS","PFC",20.3125576147942,"1h","1h"
9022,"PFC_25",4,"OID30386","Q6IBS0","TWF2",0,"Cardiometabolic_II","B13805","20212097_SS211647","PASS",-1.5638,3.7558,"Plate control","PASS","PFC",13.5085413874949,"24h","24h"
9023,"PFC_02",13,"OID30386","Q6IBS0","TWF2",0,"Cardiometabolic_II","B13805","20212097_SS211647","PASS",-1.5638,2.6165,"Plate control","PASS","PFC",6.13260487145818,"Baseline","Baseline"
9024,"PFC_10",14,"OID30386","Q6IBS0","TWF2",0,"Cardiometabolic_II","B13805","20212097_SS211647","PASS",-1.5638,3.0967,"Plate control","PASS","PFC",8.55459763954503,"1h","1h"
9025,"PFC_18",15,"OID30386","Q6IBS0","TWF2",0,"Cardiometabolic_II","B13805","20212097_SS211647","PASS",-1.5638,3.7856,"Plate control","PASS","PFC",13.7904726592817,"1h","1h"
9026,"PFC_26",16,"OID30386","Q6IBS0","TWF2",0,"Cardiometabolic_II","B13805","20212097_SS211647","PASS",-1.5638,4.1124,"Plate control","PASS","PFC",17.2964013486138,"24h","24h"
9027,"PFC_03",25,"OID30386","Q6IBS0","TWF2",0,"Cardiometabolic_II","B13805","20212097_SS211647","PASS",-1.5638,1.3013,"Plate control","PASS","PFC",2.46450857376239,"Baseline","Baseline"
9028,"PFC_11",26,"OID30386","Q6IBS0","TWF2",0,"Cardiometabolic_II","B13805","20212097_SS211647","PASS",-1.5638,4.0238,"Plate control","PASS","PFC",16.2661396505943,"1h","1h"
9029,"PFC_19",27,"OID30386","Q6IBS0","TWF2",0,"Cardiometabolic_II","B13805","20212097_SS211647","PASS",-1.5638,3.6425,"Plate control","PASS","PFC",12.4882550322463,"24h","24h"
9030,"PFC_27",28,"OID30386","Q6IBS0","TWF2",0,"Cardiometabolic_II","B13805","20212097_SS211647","PASS",-1.5638,5.3043,"Plate control","PASS","PFC",39.5142193172533,"24h","24h"
9031,"PFC_04",37,"OID30386","Q6IBS0","TWF2",0,"Cardiometabolic_II","B13805","20212097_SS211647","PASS",-1.5638,4.0283,"Plate control","PASS","PFC",16.3169555907455,"Baseline","Baseline"
9032,"PFC_12",38,"OID30386","Q6IBS0","TWF2",0,"Cardiometabolic_II","B13805","20212097_SS211647","PASS",-1.5638,2.1744,"Plate control","PASS","PFC",4.51397991732278,"1h","1h"
9033,"PFC_20",39,"OID30386","Q6IBS0","TWF2",0,"Cardiometabolic_II","B13805","20212097_SS211647","PASS",-1.5638,4.5589,"Plate control","PASS","PFC",23.570329145721,"24h","24h"
9034,"PFC_05",49,"OID30386","Q6IBS0","TWF2",0,"Cardiometabolic_II","B13805","20212097_SS211647","PASS",-1.5638,2.5158,"Plate control","PASS","PFC",5.71914704835824,"Baseline","Baseline"
9035,"PFC_13",50,"OID30386","Q6IBS0","TWF2",0,"Cardiometabolic_II","B13805","20212097_SS211647","PASS",-1.5638,4.4502,"Plate control","PASS","PFC",21.8596742923499,"1h","1h"
9036,"PFC_21",51,"OID30386","Q6IBS0","TWF2",0,"Cardiometabolic_II","B13805","20212097_SS211647","PASS",-1.5638,2.8253,"Plate control","PASS","PFC",7.08761382745498,"24h","24h"
9037,"PFC_06",61,"OID30386","Q6IBS0","TWF2",0,"Cardiometabolic_II","B13805","20212097_SS211647","PASS",-1.5638,3.7003,"Plate control","PASS","PFC",12.9987410728991,"Baseline","Baseline"
9038,"PFC_14",62,"OID30386","Q6IBS0","TWF2",0,"Cardiometabolic_II","B13805","20212097_SS211647","PASS",-1.5638,4.2812,"Plate control","PASS","PFC",19.4432838951398,"1h","1h"
9039,"PFC_22",63,"OID30386","Q6IBS0","TWF2",0,"Cardiometabolic_II","B13805","20212097_SS211647","PASS",-1.5638,3.5769,"Plate control","PASS","PFC",11.9331250436666,"24h","24h"
9040,"PFC_07",73,"OID30386","Q6IBS0","TWF2",0,"Cardiometabolic_II","B13805","20212097_SS211647","PASS",-1.5638,4.1823,"Plate control","PASS","PFC",18.1550626056204,"Baseline","Baseline"
9041,"PFC_15",74,"OID30386","Q6IBS0","TWF2",0,"Cardiometabolic_II","B13805","20212097_SS211647","PASS",-1.5638,2.6523,"Plate control","PASS","PFC",6.28668727729786,"1h","1h"
9042,"PFC_23",75,"OID30386","Q6IBS0","TWF2",0,"Cardiometabolic_II","B13805","20212097_SS211647","PASS",-1.5638,4.2749,"Plate control","PASS","PFC",19.3585635476289,"24h","24h"
9043,"PFC_08",85,"OID30386","Q6IBS0","TWF2",0,"Cardiometabolic_II","B13805","20212097_SS211647","PASS",-1.5638,3.1742,"Plate control","PASS","PFC",9.02670838041064,"Baseline","Baseline"
9044,"PFC_16",86,"OID30386","Q6IBS0","TWF2",0,"Cardiometabolic_II","B13805","20212097_SS211647","PASS",-1.5638,3.9292,"Plate control","PASS","PFC",15.2337582368994,"1h","1h"
9045,"PFC_24",87,"OID30386","Q6IBS0","TWF2",0,"Cardiometabolic_II","B13805","20212097_SS211647","PASS",-1.5638,4.4232,"Plate control","PASS","PFC",21.4543754671117,"24h","24h"
9046,"PFC_01",1,"OID30387","Q8N114","SHISA5",0,"Cardiometabolic_II","B13805","20212097_SS211647","PASS",-2.6338,-0.5969,"Plate control","PASS","PFC",0.661173130012104,"Baseline","Baseline"
9047,"PFC_09",2,"OID30387","Q8N114","SHISA5",0,"Cardiometabolic_II","B13805","20212097_SS211647","PASS",-2.6338,0.2217,"Plate control","PASS","PFC",1.16610685947048,"Baseline","Baseline"
9048,"PFC_17",3,"OID30387","Q8N114","SHISA5",0,"Cardiometabolic_II","B13805","20212097_SS211647","PASS",-2.6338,-0.4227,"Plate control","PASS","PFC",0.746027129197824,"1h","1h"
9049,"PFC_25",4,"OID30387","Q8N114","SHISA5",0,"Cardiometabolic_II","B13805","20212097_SS211647","PASS",-2.6338,0.0802,"Plate control","PASS","PFC",1.05716458453372,"24h","24h"
9050,"PFC_02",13,"OID30387","Q8N114","SHISA5",0,"Cardiometabolic_II","B13805","20212097_SS211647","PASS",-2.6338,0.189,"Plate control","PASS","PFC",1.13997327269346,"Baseline","Baseline"
9051,"PFC_10",14,"OID30387","Q8N114","SHISA5",0,"Cardiometabolic_II","B13805","20212097_SS211647","PASS",-2.6338,-0.2178,"Plate control","PASS","PFC",0.859875682061722,"1h","1h"
9052,"PFC_18",15,"OID30387","Q8N114","SHISA5",0,"Cardiometabolic_II","B13805","20212097_SS211647","PASS",-2.6338,0.0758,"Plate control","PASS","PFC",1.05394530531948,"1h","1h"
9053,"PFC_26",16,"OID30387","Q8N114","SHISA5",0,"Cardiometabolic_II","B13805","20212097_SS211647","PASS",-2.6338,0.0855,"Plate control","PASS","PFC",1.0610554114356,"24h","24h"
9054,"PFC_03",25,"OID30387","Q8N114","SHISA5",0,"Cardiometabolic_II","B13805","20212097_SS211647","PASS",-2.6338,0.0358,"Plate control","PASS","PFC",1.02512511552094,"Baseline","Baseline"
9055,"PFC_11",26,"OID30387","Q8N114","SHISA5",0,"Cardiometabolic_II","B13805","20212097_SS211647","PASS",-2.6338,0.5658,"Plate control","PASS","PFC",1.48020808336406,"1h","1h"
9056,"PFC_19",27,"OID30387","Q8N114","SHISA5",0,"Cardiometabolic_II","B13805","20212097_SS211647","PASS",-2.6338,-0.6285,"Plate control","PASS","PFC",0.646848607745039,"24h","24h"
9057,"PFC_27",28,"OID30387","Q8N114","SHISA5",0,"Cardiometabolic_II","B13805","20212097_SS211647","PASS",-2.6338,0.365,"Plate control","PASS","PFC",1.28788162950983,"24h","24h"
9058,"PFC_04",37,"OID30387","Q8N114","SHISA5",0,"Cardiometabolic_II","B13805","20212097_SS211647","PASS",-2.6338,0.6825,"Plate control","PASS","PFC",1.60491845868245,"Baseline","Baseline"
9059,"PFC_12",38,"OID30387","Q8N114","SHISA5",0,"Cardiometabolic_II","B13805","20212097_SS211647","PASS",-2.6338,0.1767,"Plate control","PASS","PFC",1.13029550434286,"1h","1h"
9060,"PFC_20",39,"OID30387","Q8N114","SHISA5",0,"Cardiometabolic_II","B13805","20212097_SS211647","PASS",-2.6338,0.1458,"Plate control","PASS","PFC",1.10634397062006,"24h","24h"
9061,"PFC_05",49,"OID30387","Q8N114","SHISA5",0,"Cardiometabolic_II","B13805","20212097_SS211647","PASS",-2.6338,0.0865,"Plate control","PASS","PFC",1.06179113395499,"Baseline","Baseline"
9062,"PFC_13",50,"OID30387","Q8N114","SHISA5",0,"Cardiometabolic_II","B13805","20212097_SS211647","PASS",-2.6338,0.3194,"Plate control","PASS","PFC",1.24781149076397,"1h","1h"
9063,"PFC_21",51,"OID30387","Q8N114","SHISA5",0,"Cardiometabolic_II","B13805","20212097_SS211647","PASS",-2.6338,0.1014,"Plate control","PASS","PFC",1.07281402279288,"24h","24h"
9064,"PFC_06",61,"OID30387","Q8N114","SHISA5",0,"Cardiometabolic_II","B13805","20212097_SS211647","PASS",-2.6338,0.0861,"Plate control","PASS","PFC",1.06149678375014,"Baseline","Baseline"
9065,"PFC_14",62,"OID30387","Q8N114","SHISA5",0,"Cardiometabolic_II","B13805","20212097_SS211647","PASS",-2.6338,0.2861,"Plate control","PASS","PFC",1.21933960932843,"1h","1h"
9066,"PFC_22",63,"OID30387","Q8N114","SHISA5",0,"Cardiometabolic_II","B13805","20212097_SS211647","PASS",-2.6338,0.1175,"Plate control","PASS","PFC",1.0848533252098,"24h","24h"
9067,"PFC_07",73,"OID30387","Q8N114","SHISA5",0,"Cardiometabolic_II","B13805","20212097_SS211647","PASS",-2.6338,-0.1263,"Plate control","PASS","PFC",0.916178110983499,"Baseline","Baseline"
9068,"PFC_15",74,"OID30387","Q8N114","SHISA5",0,"Cardiometabolic_II","B13805","20212097_SS211647","PASS",-2.6338,0.5155,"Plate control","PASS","PFC",1.42948947720108,"1h","1h"
9069,"PFC_23",75,"OID30387","Q8N114","SHISA5",0,"Cardiometabolic_II","B13805","20212097_SS211647","PASS",-2.6338,0.2957,"Plate control","PASS","PFC",1.22748040999901,"24h","24h"
9070,"PFC_08",85,"OID30387","Q8N114","SHISA5",0,"Cardiometabolic_II","B13805","20212097_SS211647","PASS",-2.6338,-0.3726,"Plate control","PASS","PFC",0.772389255180598,"Baseline","Baseline"
9071,"PFC_16",86,"OID30387","Q8N114","SHISA5",0,"Cardiometabolic_II","B13805","20212097_SS211647","PASS",-2.6338,0.3335,"Plate control","PASS","PFC",1.26006661008984,"1h","1h"
9072,"PFC_24",87,"OID30387","Q8N114","SHISA5",0,"Cardiometabolic_II","B13805","20212097_SS211647","PASS",-2.6338,0.3273,"Plate control","PASS","PFC",1.25466307724894,"24h","24h"

Supplemental table 1_Olink proximity extension assay (PEA) data

,"SampleID","Index","OlinkID","UniProt","Assay","MissingFreq","Panel","Panel_Lot_Nr","PlateID","QC_Warning","LOD","NPX","Normalization","Assay_Warning","condition","Value","group","alt_group"
9073,"PFC_01",1,"OID30388","O43280","TREH",0,"Cardiometabolic_II","B13805","20212097_SS211647","PASS",-4.0871,0.423,"Plate control","PASS","PFC",1.34071259158034,"Baseline","Baseline"
9074,"PFC_09",2,"OID30388","O43280","TREH",0,"Cardiometabolic_II","B13805","20212097_SS211647","PASS",-4.0871,-1.4865,"Plate control","PASS","PFC",0.356877289242521,"Baseline","Baseline"
9075,"PFC_17",3,"OID30388","O43280","TREH",0,"Cardiometabolic_II","B13805","20212097_SS211647","PASS",-4.0871,0.7993,"Plate control","PASS","PFC",1.74025654396996,"1h","1h"
9076,"PFC_25",4,"OID30388","O43280","TREH",0,"Cardiometabolic_II","B13805","20212097_SS211647","PASS",-4.0871,0.9441,"Plate control","PASS","PFC",1.92398826541189,"24h","24h"
9077,"PFC_02",13,"OID30388","O43280","TREH",0,"Cardiometabolic_II","B13805","20212097_SS211647","PASS",-4.0871,-0.0749,"Plate control","PASS","PFC",0.949407926612596,"Baseline","Baseline"
9078,"PFC_10",14,"OID30388","O43280","TREH",0,"Cardiometabolic_II","B13805","20212097_SS211647","PASS",-4.0871,0.5681,"Plate control","PASS","PFC",1.48256977014701,"1h","1h"
9079,"PFC_18",15,"OID30388","O43280","TREH",0,"Cardiometabolic_II","B13805","20212097_SS211647","PASS",-4.0871,-1.4928,"Plate control","PASS","PFC",0.35532226550649,"1h","1h"
9080,"PFC_26",16,"OID30388","O43280","TREH",0,"Cardiometabolic_II","B13805","20212097_SS211647","PASS",-4.0871,0.9471,"Plate control","PASS","PFC",1.92799324915832,"24h","24h"
9081,"PFC_03",25,"OID30388","O43280","TREH",0,"Cardiometabolic_II","B13805","20212097_SS211647","PASS",-4.0871,0.7268,"Plate control","PASS","PFC",1.65496418950065,"Baseline","Baseline"
9082,"PFC_11",26,"OID30388","O43280","TREH",0,"Cardiometabolic_II","B13805","20212097_SS211647","PASS",-4.0871,0.1426,"Plate control","PASS","PFC",1.10389274067335,"1h","1h"
9083,"PFC_19",27,"OID30388","O43280","TREH",0,"Cardiometabolic_II","B13805","20212097_SS211647","PASS",-4.0871,0.704,"Plate control","PASS","PFC",1.62901512614043,"24h","24h"
9084,"PFC_27",28,"OID30388","O43280","TREH",0,"Cardiometabolic_II","B13805","20212097_SS211647","PASS",-4.0871,-1.2574,"Plate control","PASS","PFC",0.41829712867229,"24h","24h"
9085,"PFC_04",37,"OID30388","O43280","TREH",0,"Cardiometabolic_II","B13805","20212097_SS211647","PASS",-4.0871,-0.249,"Plate control","PASS","PFC",0.84147948228538,"Baseline","Baseline"
9086,"PFC_12",38,"OID30388","O43280","TREH",0,"Cardiometabolic_II","B13805","20212097_SS211647","PASS",-4.0871,0.9748,"Plate control","PASS","PFC",1.96536872024684,"1h","1h"
9087,"PFC_20",39,"OID30388","O43280","TREH",0,"Cardiometabolic_II","B13805","20212097_SS211647","PASS",-4.0871,0.1022,"Plate control","PASS","PFC",1.07340908217526,"24h","24h"
9088,"PFC_05",49,"OID30388","O43280","TREH",0,"Cardiometabolic_II","B13805","20212097_SS211647","PASS",-4.0871,0.5855,"Plate control","PASS","PFC",1.50055895328158,"Baseline","Baseline"
9089,"PFC_13",50,"OID30388","O43280","TREH",0,"Cardiometabolic_II","B13805","20212097_SS211647","PASS",-4.0871,-0.1244,"Plate control","PASS","PFC",0.917385493779856,"1h","1h"
9090,"PFC_21",51,"OID30388","O43280","TREH",0,"Cardiometabolic_II","B13805","20212097_SS211647","PASS",-4.0871,0.809,"Plate control","PASS","PFC",1.75199663013425,"24h","24h"
9091,"PFC_06",61,"OID30388","O43280","TREH",0,"Cardiometabolic_II","B13805","20212097_SS211647","PASS",-4.0871,-2.5143,"Plate control","PASS","PFC",0.17503313920827,"Baseline","Baseline"
9092,"PFC_14",62,"OID30388","O43280","TREH",0,"Cardiometabolic_II","B13805","20212097_SS211647","PASS",-4.0871,0.5159,"Plate control","PASS","PFC",1.42988587079066,"1h","1h"
9093,"PFC_22",63,"OID30388","O43280","TREH",0,"Cardiometabolic_II","B13805","20212097_SS211647","PASS",-4.0871,-0.4329,"Plate control","PASS","PFC",0.740771243580565,"24h","24h"
9094,"PFC_07",73,"OID30388","O43280","TREH",0,"Cardiometabolic_II","B13805","20212097_SS211647","PASS",-4.0871,0.7674,"Plate control","PASS","PFC",1.70219934346325,"Baseline","Baseline"
9095,"PFC_15",74,"OID30388","O43280","TREH",0,"Cardiometabolic_II","B13805","20212097_SS211647","PASS",-4.0871,-2.6285,"Plate control","PASS","PFC",0.16171215193626,"1h","1h"
9096,"PFC_23",75,"OID30388","O43280","TREH",0,"Cardiometabolic_II","B13805","20212097_SS211647","PASS",-4.0871,0.7085,"Plate control","PASS","PFC",1.63410422145952,"24h","24h"
9097,"PFC_08",85,"OID30388","O43280","TREH",0,"Cardiometabolic_II","B13805","20212097_SS211647","PASS",-4.0871,0.7524,"Plate control","PASS","PFC",1.684592910804,"Baseline","Baseline"
9098,"PFC_16",86,"OID30388","O43280","TREH",0,"Cardiometabolic_II","B13805","20212097_SS211647","PASS",-4.0871,0.7183,"Plate control","PASS","PFC",1.64524222035854,"1h","1h"
9099,"PFC_24",87,"OID30388","O43280","TREH",0,"Cardiometabolic_II","B13805","20212097_SS211647","PASS",-4.0871,-2.5376,"Plate control","PASS","PFC",0.172229001197305,"24h","24h"
9100,"PFC_01",1,"OID30389","P02818","BGLAP",0,"Cardiometabolic_II","B13805","20212097_SS211647","PASS",-5.4176,-0.4514,"Plate control","PASS","PFC",0.731332813728726,"Baseline","Baseline"
9101,"PFC_09",2,"OID30389","P02818","BGLAP",0,"Cardiometabolic_II","B13805","20212097_SS211647","PASS",-5.4176,-1.2643,"Plate control","PASS","PFC",0.416301309015473,"Baseline","Baseline"
9102,"PFC_17",3,"OID30389","P02818","BGLAP",0,"Cardiometabolic_II","B13805","20212097_SS211647","PASS",-5.4176,-2.5552,"Plate control","PASS","PFC",0.170140676421435,"1h","1h"
9103,"PFC_25",4,"OID30389","P02818","BGLAP",0,"Cardiometabolic_II","B13805","20212097_SS211647","PASS",-5.4176,-0.7086,"Plate control","PASS","PFC",0.611913655538479,"24h","24h"
9104,"PFC_02",13,"OID30389","P02818","BGLAP",0,"Cardiometabolic_II","B13805","20212097_SS211647","PASS",-5.4176,-0.6736,"Plate control","PASS","PFC",0.626940310888892,"Baseline","Baseline"
9105,"PFC_10",14,"OID30389","P02818","BGLAP",0,"Cardiometabolic_II","B13805","20212097_SS211647","PASS",-5.4176,-0.5564,"Plate control","PASS","PFC",0.67999686490016,"1h","1h"
9106,"PFC_18",15,"OID30389","P02818","BGLAP",0,"Cardiometabolic_II","B13805","20212097_SS211647","PASS",-5.4176,-1.4885,"Plate control","PASS","PFC",0.356382895035961,"1h","1h"
9107,"PFC_26",16,"OID30389","P02818","BGLAP",0,"Cardiometabolic_II","B13805","20212097_SS211647","PASS",-5.4176,-2.5593,"Plate control","PASS","PFC",0.169657839460754,"24h","24h"
9108,"PFC_03",25,"OID30389","P02818","BGLAP",0,"Cardiometabolic_II","B13805","20212097_SS211647","PASS",-5.4176,-1.6715,"Plate control","PASS","PFC",0.313926777699888,"Baseline","Baseline"
9109,"PFC_11",26,"OID30389","P02818","BGLAP",0,"Cardiometabolic_II","B13805","20212097_SS211647","PASS",-5.4176,-1.0425,"Plate control","PASS","PFC",0.485485462171175,"1h","1h"
9110,"PFC_19",27,"OID30389","P02818","BGLAP",0,"Cardiometabolic_II","B13805","20212097_SS211647","PASS",-5.4176,-0.6626,"Plate control","PASS","PFC",0.631738761820123,"24h","24h"
9111,"PFC_27",28,"OID30389","P02818","BGLAP",0,"Cardiometabolic_II","B13805","20212097_SS211647","PASS",-5.4176,-1.5046,"Plate control","PASS","PFC",0.352427888999798,"24h","24h"
9112,"PFC_04",37,"OID30389","P02818","BGLAP",0,"Cardiometabolic_II","B13805","20212097_SS211647","PASS",-5.4176,-0.875,"Plate control","PASS","PFC",0.545253866332629,"Baseline","Baseline"
9113,"PFC_12",38,"OID30389","P02818","BGLAP",0,"Cardiometabolic_II","B13805","20212097_SS211647","PASS",-5.4176,-1.9455,"Plate control","PASS","PFC",0.259624781137901,"1h","1h"
9114,"PFC_20",39,"OID30389","P02818","BGLAP",0,"Cardiometabolic_II","B13805","20212097_SS211647","PASS",-5.4176,-1.5287,"Plate control","PASS","PFC",0.346589535187462,"24h","24h"
9115,"PFC_05",49,"OID30389","P02818","BGLAP",0,"Cardiometabolic_II","B13805","20212097_SS211647","PASS",-5.4176,-1.525,"Plate control","PASS","PFC",0.347479554960584,"Baseline","Baseline"
9116,"PFC_13",50,"OID30389","P02818","BGLAP",0,"Cardiometabolic_II","B13805","20212097_SS211647","PASS",-5.4176,-1.4689,"Plate control","PASS","PFC",0.361257639084464,"1h","1h"
9117,"PFC_21",51,"OID30389","P02818","BGLAP",0,"Cardiometabolic_II","B13805","20212097_SS211647","PASS",-5.4176,-1.7066,"Plate control","PASS","PFC",0.306381268125984,"24h","24h"
9118,"PFC_06",61,"OID30389","P02818","BGLAP",0,"Cardiometabolic_II","B13805","20212097_SS211647","PASS",-5.4176,-1.1587,"Plate control","PASS","PFC",0.447915966868849,"Baseline","Baseline"
9119,"PFC_14",62,"OID30389","P02818","BGLAP",0,"Cardiometabolic_II","B13805","20212097_SS211647","PASS",-5.4176,-1.5986,"Plate control","PASS","PFC",0.330197247679885,"1h","1h"
9120,"PFC_22",63,"OID30389","P02818","BGLAP",0,"Cardiometabolic_II","B13805","20212097_SS211647","PASS",-5.4176,-1.1607,"Plate control","PASS","PFC",0.447295453696055,"24h","24h"
9121,"PFC_07",73,"OID30389","P02818","BGLAP",0,"Cardiometabolic_II","B13805","20212097_SS211647","PASS",-5.4176,-0.5277,"Plate control","PASS","PFC",0.693659712051645,"Baseline","Baseline"
9122,"PFC_15",74,"OID30389","P02818","BGLAP",0,"Cardiometabolic_II","B13805","20212097_SS211647","PASS",-5.4176,-1.8458,"Plate control","PASS","PFC",0.278201094333914,"1h","1h"
9123,"PFC_23",75,"OID30389","P02818","BGLAP",0,"Cardiometabolic_II","B13805","20212097_SS211647","PASS",-5.4176,-1.5645,"Plate control","PASS","PFC",0.338094862704963,"24h","24h"
9124,"PFC_08",85,"OID30389","P02818","BGLAP",0,"Cardiometabolic_II","B13805","20212097_SS211647","PASS",-5.4176,-2.0989,"Plate control","PASS","PFC",0.233436166230112,"Baseline","Baseline"
9125,"PFC_16",86,"OID30389","P02818","BGLAP",0,"Cardiometabolic_II","B13805","20212097_SS211647","PASS",-5.4176,-0.9149,"Plate control","PASS","PFC",0.530380632594034,"1h","1h"
9126,"PFC_24",87,"OID30389","P02818","BGLAP",0,"Cardiometabolic_II","B13805","20212097_SS211647","PASS",-5.4176,-1.6439,"Plate control","PASS","PFC",0.319990282728621,"24h","24h"
9127,"PFC_01",1,"OID30390","P47972","NPTX2",0,"Cardiometabolic_II","B13805","20212097_SS211647","PASS",-1.9201,0.6545,"Plate control","PASS","PFC",1.57407032738941,"Baseline","Baseline"
9128,"PFC_09",2,"OID30390","P47972","NPTX2",0,"Cardiometabolic_II","B13805","20212097_SS211647","PASS",-1.9201,0.8998,"Plate control","PASS","PFC",1.86580730932895,"Baseline","Baseline"
9129,"PFC_17",3,"OID30390","P47972","NPTX2",0,"Cardiometabolic_II","B13805","20212097_SS211647","PASS",-1.9201,0.8883,"Plate control","PASS","PFC",1.85099371938263,"1h","1h"
9130,"PFC_25",4,"OID30390","P47972","NPTX2",0,"Cardiometabolic_II","B13805","20212097_SS211647","PASS",-1.9201,0.6673,"Plate control","PASS","PFC",1.58809806323889,"24h","24h"
9131,"PFC_02",13,"OID30390","P47972","NPTX2",0,"Cardiometabolic_II","B13805","20212097_SS211647","PASS",-1.9201,0.4747,"Plate control","PASS","PFC",1.38962922452223,"Baseline","Baseline"
9132,"PFC_10",14,"OID30390","P47972","NPTX2",0,"Cardiometabolic_II","B13805","20212097_SS211647","PASS",-1.9201,0.9171,"Plate control","PASS","PFC",1.88831572183623,"1h","1h"
9133,"PFC_18",15,"OID30390","P47972","NPTX2",0,"Cardiometabolic_II","B13805","20212097_SS211647","PASS",-1.9201,0.8882,"Plate control","PASS","PFC",1.85086542272132,"1h","1h"
9134,"PFC_26",16,"OID30390","P47972","NPTX2",0,"Cardiometabolic_II","B13805","20212097_SS211647","PASS",-1.9201,0.8692,"Plate control","PASS","PFC",1.82664970989166,"24h","24h"
9135,"PFC_03",25,"OID30390","P47972","NPTX2",0,"Cardiometabolic_II","B13805","20212097_SS211647","PASS",-1.9201,1.3624,"Plate control","PASS","PFC",2.57112544346428,"Baseline","Baseline"
9136,"PFC_11",26,"OID30390","P47972","NPTX2",0,"Cardiometabolic_II","B13805","20212097_SS211647","PASS",-1.9201,0.6902,"Plate control","PASS","PFC",1.61350718253156,"1h","1h"
9137,"PFC_19",27,"OID30390","P47972","NPTX2",0,"Cardiometabolic_II","B13805","20212097_SS211647","PASS",-1.9201,0.9711,"Plate control","PASS","PFC",1.96033470602205,"24h","24h"
9138,"PFC_27",28,"OID30390","P47972","NPTX2",0,"Cardiometabolic_II","B13805","20212097_SS211647","PASS",-1.9201,1.0436,"Plate control","PASS","PFC",2.06136502660861,"24h","24h"
9139,"PFC_04",37,"OID30390","P47972","NPTX2",0,"Cardiometabolic_II","B13805","20212097_SS211647","PASS",-1.9201,0.7527,"Plate control","PASS","PFC",1.68494324847603,"Baseline","Baseline"
9140,"PFC_12",38,"OID30390","P47972","NPTX2",0,"Cardiometabolic_II","B13805","20212097_SS211647","PASS",-1.9201,1.7821,"Plate control","PASS","PFC",3.43926432834743,"1h","1h"
9141,"PFC_20",39,"OID30390","P47972","NPTX2",0,"Cardiometabolic_II","B13805","20212097_SS211647","PASS",-1.9201,0.7745,"Plate control","PASS","PFC",1.71059710084188,"24h","24h"
9142,"PFC_05",49,"OID30390","P47972","NPTX2",0,"Cardiometabolic_II","B13805","20212097_SS211647","PASS",-1.9201,0.6965,"Plate control","PASS","PFC",1.62056849619148,"Baseline","Baseline"
9143,"PFC_13",50,"OID30390","P47972","NPTX2",0,"Cardiometabolic_II","B13805","20212097_SS211647","PASS",-1.9201,0.8479,"Plate control","PASS","PFC",1.79987909715098,"1h","1h"
9144,"PFC_21",51,"OID30390","P47972","NPTX2",0,"Cardiometabolic_II","B13805","20212097_SS211647","PASS",-1.9201,1.6505,"Plate control","PASS","PFC",3.13942424459741,"24h","24h"
9145,"PFC_06",61,"OID30390","P47972","NPTX2",0,"Cardiometabolic_II","B13805","20212097_SS211647","PASS",-1.9201,0.5163,"Plate control","PASS","PFC",1.43028237429912,"Baseline","Baseline"
9146,"PFC_14",62,"OID30390","P47972","NPTX2",0,"Cardiometabolic_II","B13805","20212097_SS211647","PASS",-1.9201,0.5554,"Plate control","PASS","PFC",1.46957602979122,"1h","1h"
9147,"PFC_22",63,"OID30390","P47972","NPTX2",0,"Cardiometabolic_II","B13805","20212097_SS211647","PASS",-1.9201,0.6039,"Plate control","PASS","PFC",1.51981950688981,"24h","24h"
9148,"PFC_07",73,"OID30390","P47972","NPTX2",0,"Cardiometabolic_II","B13805","20212097_SS211647","PASS",-1.9201,0.5923,"Plate control","PASS","PFC",1.50764838363262,"Baseline","Baseline"
9149,"PFC_15",74,"OID30390","P47972","NPTX2",0,"Cardiometabolic_II","B13805","20212097_SS211647","PASS",-1.9201,0.932,"Plate control","PASS","PFC",1.90791910109512,"1h","1h"
9150,"PFC_23",75,"OID30390","P47972","NPTX2",0,"Cardiometabolic_II","B13805","20212097_SS211647","PASS",-1.9201,0.5528,"Plate control","PASS","PFC",1.46692997039624,"24h","24h"
9151,"PFC_08",85,"OID30390","P47972","NPTX2",0,"Cardiometabolic_II","B13805","20212097_SS211647","PASS",-1.9201,0.6485,"Plate control","PASS","PFC",1.56753754688406,"Baseline","Baseline"
9152,"PFC_16",86,"OID30390","P47972","NPTX2",0,"Cardiometabolic_II","B13805","20212097_SS211647","PASS",-1.9201,0.5294,"Plate control","PASS","PFC",1.44332880710793,"1h","1h"
9153,"PFC_24",87,"OID30390","P47972","NPTX2",0,"Cardiometabolic_II","B13805","20212097_SS211647","PASS",-1.9201,0.8084,"Plate control","PASS","PFC",1.7512681467139,"24h","24h"
9154,"PFC_01",1,"OID30391","Q96NZ9","PRAP1",0,"Cardiometabolic_II","B13805","20212097_SS211647","PASS",-3.9963,0.8101,"Plate control","PASS","PFC",1.75333297020068,"Baseline","Baseline"
9155,"PFC_09",2,"OID30391","Q96NZ9","PRAP1",0,"Cardiometabolic_II","B13805","20212097_SS211647","PASS",-3.9963,0.7847,"Plate control","PASS","PFC",1.72273404970046,"Baseline","Baseline"
9156,"PFC_17",3,"OID30391","Q96NZ9","PRAP1",0,"Cardiometabolic_II","B13805","20212097_SS211647","PASS",-3.9963,-1.2793,"Plate control","PASS","PFC",0.411995361541527,"1h","1h"
9157,"PFC_25",4,"OID30391","Q96NZ9","PRAP1",0,"Cardiometabolic_II","B13805","20212097_SS211647","PASS",-3.9963,0.3495,"Plate control","PASS","PFC",1.27411897480359,"24h","24h"
9158,"PFC_02",13,"OID30391","Q96NZ9","PRAP1",0,"Cardiometabolic_II","B13805","20212097_SS211647","PASS",-3.9963,1.401,"Plate control","PASS","PFC",2.64084568203,"Baseline","Baseline"
9159,"PFC_10",14,"OID30391","Q96NZ9","PRAP1",0,"Cardiometabolic_II","B13805","20212097_SS211647","PASS",-3.9963,0.5845,"Plate control","PASS","PFC",1.49951920546462,"1h","1h"
9160,"PFC_18",15,"OID30391","Q96NZ9","PRAP1",0,"Cardiometabolic_II","B13805","20212097_SS211647","PASS",-3.9963,1.074,"Plate control","PASS","PFC",2.10526230941888,"1h","1h"
9161,"PFC_26",16,"OID30391","Q96NZ9","PRAP1",0,"Cardiometabolic_II","B13805","20212097_SS211647","PASS",-3.9963,-0.9839,"Plate control","PASS","PFC",0.505611085500906,"24h","24h"
9162,"PFC_03",25,"OID30391","Q96NZ9","PRAP1",0,"Cardiometabolic_II","B13805","20212097_SS211647","PASS",-3.9963,0.0892,"Plate control","PASS","PFC",1.06378013391184,"Baseline","Baseline"
9163,"PFC_11",26,"OID30391","Q96NZ9","PRAP1",0,"Cardiometabolic_II","B13805","20212097_SS211647","PASS",-3.9963,1.5021,"Plate control","PASS","PFC",2.83254720683574,"1h","1h"
9164,"PFC_19",27,"OID30391","Q96NZ9","PRAP1",0,"Cardiometabolic_II","B13805","20212097_SS211647","PASS",-3.9963,0.9047,"Plate control","PASS","PFC",1.87215515067019,"24h","24h"
9165,"PFC_27",28,"OID30391","Q96NZ9","PRAP1",0,"Cardiometabolic_II","B13805","20212097_SS211647","PASS",-3.9963,1.4358,"Plate control","PASS","PFC",2.70532139892372,"24h","24h"
9166,"PFC_04",37,"OID30391","Q96NZ9","PRAP1",0,"Cardiometabolic_II","B13805","20212097_SS211647","PASS",-3.9963,0.1308,"Plate control","PASS","PFC",1.09490067480897,"Baseline","Baseline"
9167,"PFC_12",38,"OID30391","Q96NZ9","PRAP1",0,"Cardiometabolic_II","B13805","20212097_SS211647","PASS",-3.9963,0.2799,"Plate control","PASS","PFC",1.21411072573572,"1h","1h"
9168,"PFC_20",39,"OID30391","Q96NZ9","PRAP1",0,"Cardiometabolic_II","B13805","20212097_SS211647","PASS",-3.9963,1.0412,"Plate control","PASS","PFC",2.05793868689292,"24h","24h"
9169,"PFC_05",49,"OID30391","Q96NZ9","PRAP1",0,"Cardiometabolic_II","B13805","20212097_SS211647","PASS",-3.9963,1.0549,"Plate control","PASS","PFC",2.07757419513475,"Baseline","Baseline"
9170,"PFC_13",50,"OID30391","Q96NZ9","PRAP1",0,"Cardiometabolic_II","B13805","20212097_SS211647","PASS",-3.9963,0.2905,"Plate control","PASS","PFC",1.22306408595886,"1h","1h"
9171,"PFC_21",51,"OID30391","Q96NZ9","PRAP1",0,"Cardiometabolic_II","B13805","20212097_SS211647","PASS",-3.9963,-0.0176,"Plate control","PASS","PFC",0.987874720509598,"24h","24h"
9172,"PFC_06",61,"OID30391","Q96NZ9","PRAP1",0,"Cardiometabolic_II","B13805","20212097_SS211647","PASS",-3.9963,0.7617,"Plate control","PASS","PFC",1.69548732592877,"Baseline","Baseline"
9173,"PFC_14",62,"OID30391","Q96NZ9","PRAP1",0,"Cardiometabolic_II","B13805","20212097_SS211647","PASS",-3.9963,1.2636,"Plate control","PASS","PFC",2.40094108046249,"1h","1h"
9174,"PFC_22",63,"OID30391","Q96NZ9","PRAP1",0,"Cardiometabolic_II","B13805","20212097_SS211647","PASS",-3.9963,0.1755,"Plate control","PASS","PFC",1.12935574186283,"24h","24h"
9175,"PFC_07",73,"OID30391","Q96NZ9","PRAP1",0,"Cardiometabolic_II","B13805","20212097_SS211647","PASS",-3.9963,-0.2589,"Plate control","PASS","PFC",0.83572488494904,"Baseline","Baseline"
9176,"PFC_15",74,"OID30391","Q96NZ9","PRAP1",0,"Cardiometabolic_II","B13805","20212097_SS211647","PASS",-3.9963,0.9449,"Plate control","PASS","PFC",1.92505544690336,"1h","1h"
9177,"PFC_23",75,"OID30391","Q96NZ9","PRAP1",0,"Cardiometabolic_II","B13805","20212097_SS211647","PASS",-3.9963,0.9855,"Plate control","PASS","PFC",1.97999940943614,"24h","24h"
9178,"PFC_08",85,"OID30391","Q96NZ9","PRAP1",0,"Cardiometabolic_II","B13805","20212097_SS211647","PASS",-3.9963,-1.4552,"Plate control","PASS","PFC",0.364704523772936,"Baseline","Baseline"
9179,"PFC_16",86,"OID30391","Q96NZ9","PRAP1",0,"Cardiometabolic_II","B13805","20212097_SS211647","PASS",-3.9963,0.0011,"Plate control","PASS","PFC",1.00076275264658,"1h","1h"
9180,"PFC_24",87,"OID30391","Q96NZ9","PRAP1",0,"Cardiometabolic_II","B13805","20212097_SS211647","PASS",-3.9963,1.1198,"Plate control","PASS","PFC",2.17316843905376,"24h","24h"
9181,"PFC_01",1,"OID30393","Q96CG8","CTHRC1",0,"Cardiometabolic_II","B13805","20212097_SS211647","PASS",-2.1541,0.469,"Plate control","PASS","PFC",1.38414971605148,"Baseline","Baseline"
9182,"PFC_09",2,"OID30393","Q96CG8","CTHRC1",0,"Cardiometabolic_II","B13805","20212097_SS211647","PASS",-2.1541,0.3154,"Plate control","PASS","PFC",1.24435661438605,"Baseline","Baseline"
9183,"PFC_17",3,"OID30393","Q96CG8","CTHRC1",0,"Cardiometabolic_II","B13805","20212097_SS211647","PASS",-2.1541,0.0176,"Plate control","PASS","PFC",1.01227410646175,"1h","1h"
9184,"PFC_25",4,"OID30393","Q96CG8","CTHRC1",0,"Cardiometabolic_II","B13805","20212097_SS211647","PASS",-2.1541,0.3845,"Plate control","PASS","PFC",1.30540728899058,"24h","24h"

Supplemental table 1_Olink proximity extension assay (PEA) data

,"SampleID","Index","OlinkID","UniProt","Assay","MissingFreq","Panel","Panel_Lot_Nr","PlateID","QC_Warning","LOD","NPX","Normalization","Assay_Warning","condition","Value","group","alt_group"
9185,"PFC_02",13,"OID30393","Q96CG8","CTHRC1",0,"Cardiometabolic_II","B13805","20212097_SS211647","PASS",-2.1541,0.8228,"Plate control","PASS","PFC",1.76883564097796,"Baseline","Baseline"
9186,"PFC_10",14,"OID30393","Q96CG8","CTHRC1",0,"Cardiometabolic_II","B13805","20212097_SS211647","PASS",-2.1541,0.4967,"Plate control","PASS","PFC",1.41098240735735,"1h","1h"
9187,"PFC_18",15,"OID30393","Q96CG8","CTHRC1",0,"Cardiometabolic_II","B13805","20212097_SS211647","PASS",-2.1541,0.451,"Plate control","PASS","PFC",1.36698745194181,"1h","1h"
9188,"PFC_26",16,"OID30393","Q96CG8","CTHRC1",0,"Cardiometabolic_II","B13805","20212097_SS211647","PASS",-2.1541,0.0193,"Plate control","PASS","PFC",1.01346762291667,"24h","24h"
9189,"PFC_03",25,"OID30393","Q96CG8","CTHRC1",0,"Cardiometabolic_II","B13805","20212097_SS211647","PASS",-2.1541,0.3291,"Plate control","PASS","PFC",1.25622945331617,"Baseline","Baseline"
9190,"PFC_11",26,"OID30393","Q96CG8","CTHRC1",0,"Cardiometabolic_II","B13805","20212097_SS211647","PASS",-2.1541,1.0809,"Plate control","PASS","PFC",2.1153552968991,"1h","1h"
9191,"PFC_19",27,"OID30393","Q96CG8","CTHRC1",0,"Cardiometabolic_II","B13805","20212097_SS211647","PASS",-2.1541,0.5307,"Plate control","PASS","PFC",1.44462996433188,"24h","24h"
9192,"PFC_27",28,"OID30393","Q96CG8","CTHRC1",0,"Cardiometabolic_II","B13805","20212097_SS211647","PASS",-2.1541,0.2583,"Plate control","PASS","PFC",1.19606848636818,"24h","24h"
9193,"PFC_04",37,"OID30393","Q96CG8","CTHRC1",0,"Cardiometabolic_II","B13805","20212097_SS211647","PASS",-2.1541,1.1797,"Plate control","PASS","PFC",2.26529666641238,"Baseline","Baseline"
9194,"PFC_12",38,"OID30393","Q96CG8","CTHRC1",0,"Cardiometabolic_II","B13805","20212097_SS211647","PASS",-2.1541,0.177,"Plate control","PASS","PFC",1.13053056712459,"1h","1h"
9195,"PFC_20",39,"OID30393","Q96CG8","CTHRC1",0,"Cardiometabolic_II","B13805","20212097_SS211647","PASS",-2.1541,0.962,"Plate control","PASS","PFC",1.94800853708648,"24h","24h"
9196,"PFC_05",49,"OID30393","Q96CG8","CTHRC1",0,"Cardiometabolic_II","B13805","20212097_SS211647","PASS",-2.1541,0.8957,"Plate control","PASS","PFC",1.86051239250247,"Baseline","Baseline"
9197,"PFC_13",50,"OID30393","Q96CG8","CTHRC1",0,"Cardiometabolic_II","B13805","20212097_SS211647","PASS",-2.1541,1.0101,"Plate control","PASS","PFC",2.01405069863159,"1h","1h"
9198,"PFC_21",51,"OID30393","Q96CG8","CTHRC1",0,"Cardiometabolic_II","B13805","20212097_SS211647","PASS",-2.1541,0.4501,"Plate control","PASS","PFC",1.36613494673131,"24h","24h"
9199,"PFC_06",61,"OID30393","Q96CG8","CTHRC1",0,"Cardiometabolic_II","B13805","20212097_SS211647","PASS",-2.1541,0.422,"Plate control","PASS","PFC",1.33978360242784,"Baseline","Baseline"
9200,"PFC_14",62,"OID30393","Q96CG8","CTHRC1",0,"Cardiometabolic_II","B13805","20212097_SS211647","PASS",-2.1541,1.02,"Plate control","PASS","PFC",2.02791895958006,"1h","1h"
9201,"PFC_22",63,"OID30393","Q96CG8","CTHRC1",0,"Cardiometabolic_II","B13805","20212097_SS211647","PASS",-2.1541,0.9161,"Plate control","PASS","PFC",1.88700729463633,"24h","24h"
9202,"PFC_07",73,"OID30393","Q96CG8","CTHRC1",0,"Cardiometabolic_II","B13805","20212097_SS211647","PASS",-2.1541,0.6063,"Plate control","PASS","PFC",1.52234991169258,"Baseline","Baseline"
9203,"PFC_15",74,"OID30393","Q96CG8","CTHRC1",0,"Cardiometabolic_II","B13805","20212097_SS211647","PASS",-2.1541,0.5531,"Plate control","PASS","PFC",1.46723504162595,"1h","1h"
9204,"PFC_23",75,"OID30393","Q96CG8","CTHRC1",0,"Cardiometabolic_II","B13805","20212097_SS211647","PASS",-2.1541,1.1571,"Plate control","PASS","PFC",2.23008700995059,"24h","24h"
9205,"PFC_08",85,"OID30393","Q96CG8","CTHRC1",0,"Cardiometabolic_II","B13805","20212097_SS211647","PASS",-2.1541,0.3181,"Plate control","PASS","PFC",1.24668760508014,"Baseline","Baseline"
9206,"PFC_16",86,"OID30393","Q96CG8","CTHRC1",0,"Cardiometabolic_II","B13805","20212097_SS211647","PASS",-2.1541,0.6919,"Plate control","PASS","PFC",1.61540957967892,"1h","1h"
9207,"PFC_24",87,"OID30393","Q96CG8","CTHRC1",0,"Cardiometabolic_II","B13805","20212097_SS211647","PASS",-2.1541,0.6463,"Plate control","PASS","PFC",1.56514899321979,"24h","24h"
9208,"PFC_01",1,"OID30394","Q9HCU0","CD248",0,"Cardiometabolic_II","B13805","20212097_SS211647","PASS",-5.0848,0.4026,"Plate control","PASS","PFC",1.32188804913976,"Baseline","Baseline"
9209,"PFC_09",2,"OID30394","Q9HCU0","CD248",0,"Cardiometabolic_II","B13805","20212097_SS211647","PASS",-5.0848,-0.1998,"Plate control","PASS","PFC",0.87067125559538,"Baseline","Baseline"
9210,"PFC_17",3,"OID30394","Q9HCU0","CD248",0,"Cardiometabolic_II","B13805","20212097_SS211647","PASS",-5.0848,-0.7751,"Plate control","PASS","PFC",0.58434811895172,"1h","1h"
9211,"PFC_25",4,"OID30394","Q9HCU0","CD248",0,"Cardiometabolic_II","B13805","20212097_SS211647","PASS",-5.0848,0.1766,"Plate control","PASS","PFC",1.13021716094387,"24h","24h"
9212,"PFC_02",13,"OID30394","Q9HCU0","CD248",0,"Cardiometabolic_II","B13805","20212097_SS211647","PASS",-5.0848,0.5645,"Plate control","PASS","PFC",1.4788748814451,"Baseline","Baseline"
9213,"PFC_10",14,"OID30394","Q9HCU0","CD248",0,"Cardiometabolic_II","B13805","20212097_SS211647","PASS",-5.0848,0.4582,"Plate control","PASS","PFC",1.37382667304605,"1h","1h"
9214,"PFC_18",15,"OID30394","Q9HCU0","CD248",0,"Cardiometabolic_II","B13805","20212097_SS211647","PASS",-5.0848,-0.0478,"Plate control","PASS","PFC",0.967410431881117,"1h","1h"
9215,"PFC_26",16,"OID30394","Q9HCU0","CD248",0,"Cardiometabolic_II","B13805","20212097_SS211647","PASS",-5.0848,-0.4165,"Plate control","PASS","PFC",0.7492400890481,"24h","24h"
9216,"PFC_03",25,"OID30394","Q9HCU0","CD248",0,"Cardiometabolic_II","B13805","20212097_SS211647","PASS",-5.0848,0.11,"Plate control","PASS","PFC",1.07922823650443,"Baseline","Baseline"
9217,"PFC_11",26,"OID30394","Q9HCU0","CD248",0,"Cardiometabolic_II","B13805","20212097_SS211647","PASS",-5.0848,0.7011,"Plate control","PASS","PFC",1.62574388804249,"1h","1h"
9218,"PFC_19",27,"OID30394","Q9HCU0","CD248",0,"Cardiometabolic_II","B13805","20212097_SS211647","PASS",-5.0848,0.4091,"Plate control","PASS","PFC",1.32785719523975,"24h","24h"
9219,"PFC_27",28,"OID30394","Q9HCU0","CD248",0,"Cardiometabolic_II","B13805","20212097_SS211647","PASS",-5.0848,0.1479,"Plate control","PASS","PFC",1.10795554757686,"24h","24h"
9220,"PFC_04",37,"OID30394","Q9HCU0","CD248",0,"Cardiometabolic_II","B13805","20212097_SS211647","PASS",-5.0848,1.839,"Plate control","PASS","PFC",3.57761960713042,"Baseline","Baseline"
9221,"PFC_12",38,"OID30394","Q9HCU0","CD248",0,"Cardiometabolic_II","B13805","20212097_SS211647","PASS",-5.0848,0.0262,"Plate control","PASS","PFC",1.01832635998898,"1h","1h"
9222,"PFC_20",39,"OID30394","Q9HCU0","CD248",0,"Cardiometabolic_II","B13805","20212097_SS211647","PASS",-5.0848,0.6002,"Plate control","PASS","PFC",1.5159267040086,"24h","24h"
9223,"PFC_05",49,"OID30394","Q9HCU0","CD248",0,"Cardiometabolic_II","B13805","20212097_SS211647","PASS",-5.0848,0.4054,"Plate control","PASS","PFC",1.32445607669112,"Baseline","Baseline"
9224,"PFC_13",50,"OID30394","Q9HCU0","CD248",0,"Cardiometabolic_II","B13805","20212097_SS211647","PASS",-5.0848,1.5104,"Plate control","PASS","PFC",2.84889016177698,"1h","1h"
9225,"PFC_21",51,"OID30394","Q9HCU0","CD248",0,"Cardiometabolic_II","B13805","20212097_SS211647","PASS",-5.0848,0.1783,"Plate control","PASS","PFC",1.13154973753613,"24h","24h"
9226,"PFC_06",61,"OID30394","Q9HCU0","CD248",0,"Cardiometabolic_II","B13805","20212097_SS211647","PASS",-5.0848,-0.4648,"Plate control","PASS","PFC",0.72457151725617,"Baseline","Baseline"
9227,"PFC_14",62,"OID30394","Q9HCU0","CD248",0,"Cardiometabolic_II","B13805","20212097_SS211647","PASS",-5.0848,0.1232,"Plate control","PASS","PFC",1.08914799284299,"1h","1h"
9228,"PFC_22",63,"OID30394","Q9HCU0","CD248",0,"Cardiometabolic_II","B13805","20212097_SS211647","PASS",-5.0848,1.3625,"Plate control","PASS","PFC",2.57130366647615,"24h","24h"
9229,"PFC_07",73,"OID30394","Q9HCU0","CD248",0,"Cardiometabolic_II","B13805","20212097_SS211647","PASS",-5.0848,-0.0022,"Plate control","PASS","PFC",0.998476238308279,"Baseline","Baseline"
9230,"PFC_15",74,"OID30394","Q9HCU0","CD248",0,"Cardiometabolic_II","B13805","20212097_SS211647","PASS",-5.0848,-0.406,"Plate control","PASS","PFC",0.75471298426028,"1h","1h"
9231,"PFC_23",75,"OID30394","Q9HCU0","CD248",0,"Cardiometabolic_II","B13805","20212097_SS211647","PASS",-5.0848,0.4295,"Plate control","PASS","PFC",1.34676674218897,"24h","24h"
9232,"PFC_08",85,"OID30394","Q9HCU0","CD248",0,"Cardiometabolic_II","B13805","20212097_SS211647","PASS",-5.0848,-0.5068,"Plate control","PASS","PFC",0.703781745766892,"Baseline","Baseline"
9233,"PFC_16",86,"OID30394","Q9HCU0","CD248",0,"Cardiometabolic_II","B13805","20212097_SS211647","PASS",-5.0848,0.1356,"Plate control","PASS","PFC",1.0985495927277,"1h","1h"
9234,"PFC_24",87,"OID30394","Q9HCU0","CD248",0,"Cardiometabolic_II","B13805","20212097_SS211647","PASS",-5.0848,-0.2203,"Plate control","PASS","PFC",0.858386921335505,"24h","24h"
9235,"PFC_01",1,"OID30395","Q9BYJ0","FGFBP2",0,"Cardiometabolic_II","B13805","20212097_SS211647","PASS",-6.2614,0.0635,"Plate control","PASS","PFC",1.04499786877565,"Baseline","Baseline"
9236,"PFC_09",2,"OID30395","Q9BYJ0","FGFBP2",0,"Cardiometabolic_II","B13805","20212097_SS211647","PASS",-6.2614,0.0814,"Plate control","PASS","PFC",1.05804427511742,"Baseline","Baseline"
9237,"PFC_17",3,"OID30395","Q9BYJ0","FGFBP2",0,"Cardiometabolic_II","B13805","20212097_SS211647","PASS",-6.2614,-0.4466,"Plate control","PASS","PFC",0.733770088150016,"1h","1h"
9238,"PFC_25",4,"OID30395","Q9BYJ0","FGFBP2",0,"Cardiometabolic_II","B13805","20212097_SS211647","PASS",-6.2614,-0.2427,"Plate control","PASS","PFC",0.845162112661701,"24h","24h"
9239,"PFC_02",13,"OID30395","Q9BYJ0","FGFBP2",0,"Cardiometabolic_II","B13805","20212097_SS211647","PASS",-6.2614,0.4451,"Plate control","PASS","PFC",1.36140847887396,"Baseline","Baseline"
9240,"PFC_10",14,"OID30395","Q9BYJ0","FGFBP2",0,"Cardiometabolic_II","B13805","20212097_SS211647","PASS",-6.2614,0.3374,"Plate control","PASS","PFC",1.26347752364025,"1h","1h"
9241,"PFC_18",15,"OID30395","Q9BYJ0","FGFBP2",0,"Cardiometabolic_II","B13805","20212097_SS211647","PASS",-6.2614,0.1484,"Plate control","PASS","PFC",1.10833960225662,"1h","1h"
9242,"PFC_26",16,"OID30395","Q9BYJ0","FGFBP2",0,"Cardiometabolic_II","B13805","20212097_SS211647","PASS",-6.2614,-0.2973,"Plate control","PASS","PFC",0.81377394793982,"24h","24h"
9243,"PFC_03",25,"OID30395","Q9BYJ0","FGFBP2",0,"Cardiometabolic_II","B13805","20212097_SS211647","PASS",-6.2614,-0.3653,"Plate control","PASS","PFC",0.776307429623983,"Baseline","Baseline"
9244,"PFC_11",26,"OID30395","Q9BYJ0","FGFBP2",0,"Cardiometabolic_II","B13805","20212097_SS211647","PASS",-6.2614,0.3276,"Plate control","PASS","PFC",1.25492400422948,"1h","1h"
9245,"PFC_19",27,"OID30395","Q9BYJ0","FGFBP2",0,"Cardiometabolic_II","B13805","20212097_SS211647","PASS",-6.2614,0.2495,"Plate control","PASS","PFC",1.18879503863481,"24h","24h"
9246,"PFC_27",28,"OID30395","Q9BYJ0","FGFBP2",0,"Cardiometabolic_II","B13805","20212097_SS211647","PASS",-6.2614,0.2819,"Plate control","PASS","PFC",1.21579500777419,"24h","24h"
9247,"PFC_04",37,"OID30395","Q9BYJ0","FGFBP2",0,"Cardiometabolic_II","B13805","20212097_SS211647","PASS",-6.2614,1.6506,"Plate control","PASS","PFC",3.13964186044569,"Baseline","Baseline"
9248,"PFC_12",38,"OID30395","Q9BYJ0","FGFBP2",0,"Cardiometabolic_II","B13805","20212097_SS211647","PASS",-6.2614,-0.4248,"Plate control","PASS","PFC",0.744941995292233,"1h","1h"
9249,"PFC_20",39,"OID30395","Q9BYJ0","FGFBP2",0,"Cardiometabolic_II","B13805","20212097_SS211647","PASS",-6.2614,0.4025,"Plate control","PASS","PFC",1.32179642601778,"24h","24h"
9250,"PFC_05",49,"OID30395","Q9BYJ0","FGFBP2",0,"Cardiometabolic_II","B13805","20212097_SS211647","PASS",-6.2614,-0.0413,"Plate control","PASS","PFC",0.971778891230087,"Baseline","Baseline"
9251,"PFC_13",50,"OID30395","Q9BYJ0","FGFBP2",0,"Cardiometabolic_II","B13805","20212097_SS211647","PASS",-6.2614,1.462,"Plate control","PASS","PFC",2.75490009276628,"1h","1h"
9252,"PFC_21",51,"OID30395","Q9BYJ0","FGFBP2",0,"Cardiometabolic_II","B13805","20212097_SS211647","PASS",-6.2614,-0.2936,"Plate control","PASS","PFC",0.815863667423493,"24h","24h"
9253,"PFC_06",61,"OID30395","Q9BYJ0","FGFBP2",0,"Cardiometabolic_II","B13805","20212097_SS211647","PASS",-6.2614,0.6044,"Plate control","PASS","PFC",1.52034632747866,"Baseline","Baseline"
9254,"PFC_14",62,"OID30395","Q9BYJ0","FGFBP2",0,"Cardiometabolic_II","B13805","20212097_SS211647","PASS",-6.2614,-0.0077,"Plate control","PASS","PFC",0.994676984433603,"1h","1h"
9255,"PFC_22",63,"OID30395","Q9BYJ0","FGFBP2",0,"Cardiometabolic_II","B13805","20212097_SS211647","PASS",-6.2614,1.3575,"Plate control","PASS","PFC",2.5624076316004,"24h","24h"
9256,"PFC_07",73,"OID30395","Q9BYJ0","FGFBP2",0,"Cardiometabolic_II","B13805","20212097_SS211647","PASS",-6.2614,-0.0977,"Plate control","PASS","PFC",0.934521654994962,"Baseline","Baseline"
9257,"PFC_15",74,"OID30395","Q9BYJ0","FGFBP2",0,"Cardiometabolic_II","B13805","20212097_SS211647","PASS",-6.2614,0.7174,"Plate control","PASS","PFC",1.64421618492318,"1h","1h"
9258,"PFC_23",75,"OID30395","Q9BYJ0","FGFBP2",0,"Cardiometabolic_II","B13805","20212097_SS211647","PASS",-6.2614,0.1459,"Plate control","PASS","PFC",1.10642065919825,"24h","24h"
9259,"PFC_08",85,"OID30395","Q9BYJ0","FGFBP2",0,"Cardiometabolic_II","B13805","20212097_SS211647","PASS",-6.2614,-0.1378,"Plate control","PASS","PFC",0.908904108579262,"Baseline","Baseline"
9260,"PFC_16",86,"OID30395","Q9BYJ0","FGFBP2",0,"Cardiometabolic_II","B13805","20212097_SS211647","PASS",-6.2614,-0.362,"Plate control","PASS","PFC",0.778085176555419,"1h","1h"
9261,"PFC_24",87,"OID30395","Q9BYJ0","FGFBP2",0,"Cardiometabolic_II","B13805","20212097_SS211647","PASS",-6.2614,0.7063,"Plate control","PASS","PFC",1.63161423604658,"24h","24h"
9262,"PFC_01",1,"OID30396","P39059","COL15A1",0,"Cardiometabolic_II","B13805","20212097_SS211647","PASS",-4.7429,0.7241,"Plate control","PASS","PFC",1.65186982479447,"Baseline","Baseline"
9263,"PFC_09",2,"OID30396","P39059","COL15A1",0,"Cardiometabolic_II","B13805","20212097_SS211647","PASS",-4.7429,0.7839,"Plate control","PASS","PFC",1.72177902791367,"Baseline","Baseline"
9264,"PFC_17",3,"OID30396","P39059","COL15A1",0,"Cardiometabolic_II","B13805","20212097_SS211647","PASS",-4.7429,-0.482,"Plate control","PASS","PFC",0.715984370600572,"1h","1h"
9265,"PFC_25",4,"OID30396","P39059","COL15A1",0,"Cardiometabolic_II","B13805","20212097_SS211647","PASS",-4.7429,-0.0285,"Plate control","PASS","PFC",0.980439150783342,"24h","24h"
9266,"PFC_02",13,"OID30396","P39059","COL15A1",0,"Cardiometabolic_II","B13805","20212097_SS211647","PASS",-4.7429,0.3344,"Plate control","PASS","PFC",1.26085292578526,"Baseline","Baseline"
9267,"PFC_10",14,"OID30396","P39059","COL15A1",0,"Cardiometabolic_II","B13805","20212097_SS211647","PASS",-4.7429,0.8875,"Plate control","PASS","PFC",1.84996759504874,"1h","1h"
9268,"PFC_18",15,"OID30396","P39059","COL15A1",0,"Cardiometabolic_II","B13805","20212097_SS211647","PASS",-4.7429,0.785,"Plate control","PASS","PFC",1.72309231942403,"1h","1h"
9269,"PFC_26",16,"OID30396","P39059","COL15A1",0,"Cardiometabolic_II","B13805","20212097_SS211647","PASS",-4.7429,-0.1823,"Plate control","PASS","PFC",0.881296878317938,"24h","24h"
9270,"PFC_03",25,"OID30396","P39059","COL15A1",0,"Cardiometabolic_II","B13805","20212097_SS211647","PASS",-4.7429,0.2489,"Plate control","PASS","PFC",1.18830073547181,"Baseline","Baseline"
9271,"PFC_11",26,"OID30396","P39059","COL15A1",0,"Cardiometabolic_II","B13805","20212097_SS211647","PASS",-4.7429,0.6309,"Plate control","PASS","PFC",1.54853071601639,"1h","1h"
9272,"PFC_19",27,"OID30396","P39059","COL15A1",0,"Cardiometabolic_II","B13805","20212097_SS211647","PASS",-4.7429,0.6513,"Plate control","PASS","PFC",1.57058279690415,"24h","24h"
9273,"PFC_27",28,"OID30396","P39059","COL15A1",0,"Cardiometabolic_II","B13805","20212097_SS211647","PASS",-4.7429,0.6255,"Plate control","PASS","PFC",1.54274540758135,"24h","24h"
9274,"PFC_04",37,"OID30396","P39059","COL15A1",0,"Cardiometabolic_II","B13805","20212097_SS211647","PASS",-4.7429,0.4982,"Plate control","PASS","PFC",1.41245019798755,"Baseline","Baseline"
9275,"PFC_12",38,"OID30396","P39059","COL15A1",0,"Cardiometabolic_II","B13805","20212097_SS211647","PASS",-4.7429,0.3031,"Plate control","PASS","PFC",1.2337926868379,"1h","1h"
9276,"PFC_20",39,"OID30396","P39059","COL15A1",0,"Cardiometabolic_II","B13805","20212097_SS211647","PASS",-4.7429,0.3354,"Plate control","PASS","PFC",1.26172718539616,"24h","24h"
9277,"PFC_05",49,"OID30396","P39059","COL15A1",0,"Cardiometabolic_II","B13805","20212097_SS211647","PASS",-4.7429,0.4932,"Plate control","PASS","PFC",1.40756349153378,"Baseline","Baseline"
9278,"PFC_13",50,"OID30396","P39059","COL15A1",0,"Cardiometabolic_II","B13805","20212097_SS211647","PASS",-4.7429,0.3085,"Plate control","PASS","PFC",1.23841941993538,"1h","1h"
9279,"PFC_21",51,"OID30396","P39059","COL15A1",0,"Cardiometabolic_II","B13805","20212097_SS211647","PASS",-4.7429,0.3125,"Plate control","PASS","PFC",1.24185781207348,"24h","24h"
9280,"PFC_06",61,"OID30396","P39059","COL15A1",0,"Cardiometabolic_II","B13805","20212097_SS211647","PASS",-4.7429,0.3675,"Plate control","PASS","PFC",1.29011529307386,"Baseline","Baseline"
9281,"PFC_14",62,"OID30396","P39059","COL15A1",0,"Cardiometabolic_II","B13805","20212097_SS211647","PASS",-4.7429,1.0198,"Plate control","PASS","PFC",2.02763784980371,"1h","1h"
9282,"PFC_22",63,"OID30396","P39059","COL15A1",0,"Cardiometabolic_II","B13805","20212097_SS211647","PASS",-4.7429,0.1461,"Plate control","PASS","PFC",1.10657405230249,"24h","24h"
9283,"PFC_07",73,"OID30396","P39059","COL15A1",0,"Cardiometabolic_II","B13805","20212097_SS211647","PASS",-4.7429,-0.0126,"Plate control","PASS","PFC",0.991304373098102,"Baseline","Baseline"
9284,"PFC_15",74,"OID30396","P39059","COL15A1",0,"Cardiometabolic_II","B13805","20212097_SS211647","PASS",-4.7429,0.4791,"Plate control","PASS","PFC",1.3938738513121,"1h","1h"
9285,"PFC_23",75,"OID30396","P39059","COL15A1",0,"Cardiometabolic_II","B13805","20212097_SS211647","PASS",-4.7429,0.638,"Plate control","PASS","PFC",1.55617035311084,"24h","24h"
9286,"PFC_08",85,"OID30396","P39059","COL15A1",0,"Cardiometabolic_II","B13805","20212097_SS211647","PASS",-4.7429,-0.3841,"Plate control","PASS","PFC",0.766256865384514,"Baseline","Baseline"
9287,"PFC_16",86,"OID30396","P39059","COL15A1",0,"Cardiometabolic_II","B13805","20212097_SS211647","PASS",-4.7429,0.1182,"Plate control","PASS","PFC",1.0853798270462,"1h","1h"
9288,"PFC_24",87,"OID30396","P39059","COL15A1",0,"Cardiometabolic_II","B13805","20212097_SS211647","PASS",-4.7429,0.811,"Plate control","PASS","PFC",1.75442709746564,"24h","24h"
9289,"PFC_01",1,"OID30397","Q6UVK1","CSPG4",0,"Cardiometabolic_II","B13805","20212097_SS211647","PASS",-4.613,0.1914,"Plate control","PASS","PFC",1.14187125717858,"Baseline","Baseline"
9290,"PFC_09",2,"OID30397","Q6UVK1","CSPG4",0,"Cardiometabolic_II","B13805","20212097_SS211647","PASS",-4.613,0.0486,"Plate control","PASS","PFC",1.03426078378364,"Baseline","Baseline"
9291,"PFC_17",3,"OID30397","Q6UVK1","CSPG4",0,"Cardiometabolic_II","B13805","20212097_SS211647","PASS",-4.613,-0.2781,"Plate control","PASS","PFC",0.824676384417871,"1h","1h"
9292,"PFC_25",4,"OID30397","Q6UVK1","CSPG4",0,"Cardiometabolic_II","B13805","20212097_SS211647","PASS",-4.613,0.2932,"Plate control","PASS","PFC",1.22535519042979,"24h","24h"
9293,"PFC_02",13,"OID30397","Q6UVK1","CSPG4",0,"Cardiometabolic_II","B13805","20212097_SS211647","PASS",-4.613,0.1083,"Plate control","PASS","PFC",1.07795727665356,"Baseline","Baseline"
9294,"PFC_10",14,"OID30397","Q6UVK1","CSPG4",0,"Cardiometabolic_II","B13805","20212097_SS211647","PASS",-4.613,0.0831,"Plate control","PASS","PFC",1.05929175664869,"1h","1h"
9295,"PFC_18",15,"OID30397","Q6UVK1","CSPG4",0,"Cardiometabolic_II","B13805","20212097_SS211647","PASS",-4.613,0.0237,"Plate control","PASS","PFC",1.01656326292408,"1h","1h"
9296,"PFC_26",16,"OID30397","Q6UVK1","CSPG4",0,"Cardiometabolic_II","B13805","20212097_SS211647","PASS",-4.613,-0.189,"Plate control","PASS","PFC",0.877213548732822,"24h","24h"

Supplemental table 1_Olink proximity extension assay (PEA) data

,"SampleID","Index","OlinkID","UniProt","Assay","MissingFreq","Panel","Panel_Lot_Nr","PlateID","QC_Warning","LOD","NPX","Normalization","Assay_Warning","condition","Value","group","alt_group"
9297,"PFC_03",25,"OID30397","Q6UVK1","CSPG4",0,"Cardiometabolic_II","B13805","20212097_SS211647","PASS",-4.613,-0.1626,"Plate control","PASS","PFC",0.893413524842804,"Baseline","Baseline"
9298,"PFC_11",26,"OID30397","Q6UVK1","CSPG4",0,"Cardiometabolic_II","B13805","20212097_SS211647","PASS",-4.613,0.0196,"Plate control","PASS","PFC",1.01367838949737,"1h","1h"
9299,"PFC_19",27,"OID30397","Q6UVK1","CSPG4",0,"Cardiometabolic_II","B13805","20212097_SS211647","PASS",-4.613,0.0282,"Plate control","PASS","PFC",1.01973903904787,"24h","24h"
9300,"PFC_27",28,"OID30397","Q6UVK1","CSPG4",0,"Cardiometabolic_II","B13805","20212097_SS211647","PASS",-4.613,0.2581,"Plate control","PASS","PFC",1.19590268756093,"24h","24h"
9301,"PFC_04",37,"OID30397","Q6UVK1","CSPG4",0,"Cardiometabolic_II","B13805","20212097_SS211647","PASS",-4.613,0.9931,"Plate control","PASS","PFC",1.99045740685263,"Baseline","Baseline"
9302,"PFC_12",38,"OID30397","Q6UVK1","CSPG4",0,"Cardiometabolic_II","B13805","20212097_SS211647","PASS",-4.613,-0.1867,"Plate control","PASS","PFC",0.878613151712397,"1h","1h"
9303,"PFC_20",39,"OID30397","Q6UVK1","CSPG4",0,"Cardiometabolic_II","B13805","20212097_SS211647","PASS",-4.613,0.2119,"Plate control","PASS","PFC",1.15821252223782,"24h","24h"
9304,"PFC_05",49,"OID30397","Q6UVK1","CSPG4",0,"Cardiometabolic_II","B13805","20212097_SS211647","PASS",-4.613,0.1291,"Plate control","PASS","PFC",1.09361125821357,"Baseline","Baseline"
9305,"PFC_13",50,"OID30397","Q6UVK1","CSPG4",0,"Cardiometabolic_II","B13805","20212097_SS211647","PASS",-4.613,0.8815,"Plate control","PASS","PFC",1.84228977276203,"1h","1h"
9306,"PFC_21",51,"OID30397","Q6UVK1","CSPG4",0,"Cardiometabolic_II","B13805","20212097_SS211647","PASS",-4.613,0.1378,"Plate control","PASS","PFC",1.10022607507313,"24h","24h"
9307,"PFC_06",61,"OID30397","Q6UVK1","CSPG4",0,"Cardiometabolic_II","B13805","20212097_SS211647","PASS",-4.613,-0.3898,"Plate control","PASS","PFC",0.763235404040326,"Baseline","Baseline"
9308,"PFC_14",62,"OID30397","Q6UVK1","CSPG4",0,"Cardiometabolic_II","B13805","20212097_SS211647","PASS",-4.613,0.1021,"Plate control","PASS","PFC",1.07333468170592,"1h","1h"
9309,"PFC_22",63,"OID30397","Q6UVK1","CSPG4",0,"Cardiometabolic_II","B13805","20212097_SS211647","PASS",-4.613,0.7829,"Plate control","PASS","PFC",1.72058599515633,"24h","24h"
9310,"PFC_07",73,"OID30397","Q6UVK1","CSPG4",0,"Cardiometabolic_II","B13805","20212097_SS211647","PASS",-4.613,0.211,"Plate control","PASS","PFC",1.15749021699012,"Baseline","Baseline"
9311,"PFC_15",74,"OID30397","Q6UVK1","CSPG4",0,"Cardiometabolic_II","B13805","20212097_SS211647","PASS",-4.613,-0.3966,"Plate control","PASS","PFC",0.759646434425707,"1h","1h"
9312,"PFC_23",75,"OID30397","Q6UVK1","CSPG4",0,"Cardiometabolic_II","B13805","20212097_SS211647","PASS",-4.613,0.3508,"Plate control","PASS","PFC",1.27526758979699,"24h","24h"
9313,"PFC_08",85,"OID30397","Q6UVK1","CSPG4",0,"Cardiometabolic_II","B13805","20212097_SS211647","PASS",-4.613,-0.0392,"Plate control","PASS","PFC",0.973194451408296,"Baseline","Baseline"
9314,"PFC_16",86,"OID30397","Q6UVK1","CSPG4",0,"Cardiometabolic_II","B13805","20212097_SS211647","PASS",-4.613,0.1014,"Plate control","PASS","PFC",1.07281402279288,"1h","1h"
9315,"PFC_24",87,"OID30397","Q6UVK1","CSPG4",0,"Cardiometabolic_II","B13805","20212097_SS211647","PASS",-4.613,-0.1294,"Plate control","PASS","PFC",0.914211581085851,"24h","24h"
9316,"PFC_01",1,"OID30398","Q6UWP8","SBSN",0,"Cardiometabolic_II","B13805","20212097_SS211647","PASS",-3.021,0.6973,"Plate control","PASS","PFC",1.62146737937901,"Baseline","Baseline"
9317,"PFC_09",2,"OID30398","Q6UWP8","SBSN",0,"Cardiometabolic_II","B13805","20212097_SS211647","PASS",-3.021,0.9992,"Plate control","PASS","PFC",1.9988912719442,"Baseline","Baseline"
9318,"PFC_17",3,"OID30398","Q6UWP8","SBSN",0,"Cardiometabolic_II","B13805","20212097_SS211647","PASS",-3.021,-0.2091,"Plate control","PASS","PFC",0.86507672595744,"1h","1h"
9319,"PFC_25",4,"OID30398","Q6UWP8","SBSN",0,"Cardiometabolic_II","B13805","20212097_SS211647","PASS",-3.021,0.5539,"Plate control","PASS","PFC",1.46804887511351,"24h","24h"
9320,"PFC_02",13,"OID30398","Q6UWP8","SBSN",0,"Cardiometabolic_II","B13805","20212097_SS211647","PASS",-3.021,0.6662,"Plate control","PASS","PFC",1.58688766047604,"Baseline","Baseline"
9321,"PFC_10",14,"OID30398","Q6UWP8","SBSN",0,"Cardiometabolic_II","B13805","20212097_SS211647","PASS",-3.021,0.7077,"Plate control","PASS","PFC",1.6331983328613,"1h","1h"
9322,"PFC_18",15,"OID30398","Q6UWP8","SBSN",0,"Cardiometabolic_II","B13805","20212097_SS211647","PASS",-3.021,0.9376,"Plate control","PASS","PFC",1.91533931800091,"1h","1h"
9323,"PFC_26",16,"OID30398","Q6UWP8","SBSN",0,"Cardiometabolic_II","B13805","20212097_SS211647","PASS",-3.021,-0.0762,"Plate control","PASS","PFC",0.948552808684341,"24h","24h"
9324,"PFC_03",25,"OID30398","Q6UWP8","SBSN",0,"Cardiometabolic_II","B13805","20212097_SS211647","PASS",-3.021,0.535,"Plate control","PASS","PFC",1.44894215455488,"Baseline","Baseline"
9325,"PFC_11",26,"OID30398","Q6UWP8","SBSN",0,"Cardiometabolic_II","B13805","20212097_SS211647","PASS",-3.021,0.7344,"Plate control","PASS","PFC",1.6637054098948,"1h","1h"
9326,"PFC_19",27,"OID30398","Q6UWP8","SBSN",0,"Cardiometabolic_II","B13805","20212097_SS211647","PASS",-3.021,0.8649,"Plate control","PASS","PFC",1.82121342591368,"24h","24h"
9327,"PFC_27",28,"OID30398","Q6UWP8","SBSN",0,"Cardiometabolic_II","B13805","20212097_SS211647","PASS",-3.021,0.3374,"Plate control","PASS","PFC",1.26347752364025,"24h","24h"
9328,"PFC_04",37,"OID30398","Q6UWP8","SBSN",0,"Cardiometabolic_II","B13805","20212097_SS211647","PASS",-3.021,0.5261,"Plate control","PASS","PFC",1.44003113040723,"Baseline","Baseline"
9329,"PFC_12",38,"OID30398","Q6UWP8","SBSN",0,"Cardiometabolic_II","B13805","20212097_SS211647","PASS",-3.021,0.5348,"Plate control","PASS","PFC",1.44874130244336,"1h","1h"
9330,"PFC_20",39,"OID30398","Q6UWP8","SBSN",0,"Cardiometabolic_II","B13805","20212097_SS211647","PASS",-3.021,0.5822,"Plate control","PASS","PFC",1.49713051876694,"24h","24h"
9331,"PFC_05",49,"OID30398","Q6UWP8","SBSN",0,"Cardiometabolic_II","B13805","20212097_SS211647","PASS",-3.021,0.0214,"Plate control","PASS","PFC",1.01494390977765,"Baseline","Baseline"
9332,"PFC_13",50,"OID30398","Q6UWP8","SBSN",0,"Cardiometabolic_II","B13805","20212097_SS211647","PASS",-3.021,-0.0234,"Plate control","PASS","PFC",0.983911186106629,"1h","1h"
9333,"PFC_21",51,"OID30398","Q6UWP8","SBSN",0,"Cardiometabolic_II","B13805","20212097_SS211647","PASS",-3.021,0.3723,"Plate control","PASS","PFC",1.29441479248821,"24h","24h"
9334,"PFC_06",61,"OID30398","Q6UWP8","SBSN",0,"Cardiometabolic_II","B13805","20212097_SS211647","PASS",-3.021,0.0086,"Plate control","PASS","PFC",1.00597886826167,"Baseline","Baseline"
9335,"PFC_14",62,"OID30398","Q6UWP8","SBSN",0,"Cardiometabolic_II","B13805","20212097_SS211647","PASS",-3.021,0.2172,"Plate control","PASS","PFC",1.16247524963669,"1h","1h"
9336,"PFC_22",63,"OID30398","Q6UWP8","SBSN",0,"Cardiometabolic_II","B13805","20212097_SS211647","PASS",-3.021,0.4483,"Plate control","PASS","PFC",1.36443153094249,"24h","24h"
9337,"PFC_07",73,"OID30398","Q6UWP8","SBSN",0,"Cardiometabolic_II","B13805","20212097_SS211647","PASS",-3.021,0.1047,"Plate control","PASS","PFC",1.07527077093703,"Baseline","Baseline"
9338,"PFC_15",74,"OID30398","Q6UWP8","SBSN",0,"Cardiometabolic_II","B13805","20212097_SS211647","PASS",-3.021,0.0131,"Plate control","PASS","PFC",1.00912157839825,"1h","1h"
9339,"PFC_23",75,"OID30398","Q6UWP8","SBSN",0,"Cardiometabolic_II","B13805","20212097_SS211647","PASS",-3.021,0.0935,"Plate control","PASS","PFC",1.06695549535811,"24h","24h"
9340,"PFC_08",85,"OID30398","Q6UWP8","SBSN",0,"Cardiometabolic_II","B13805","20212097_SS211647","PASS",-3.021,-0.4259,"Plate control","PASS","PFC",0.744374221884445,"Baseline","Baseline"
9341,"PFC_16",86,"OID30398","Q6UWP8","SBSN",0,"Cardiometabolic_II","B13805","20212097_SS211647","PASS",-3.021,0.151,"Plate control","PASS","PFC",1.11033883362864,"1h","1h"
9342,"PFC_24",87,"OID30398","Q6UWP8","SBSN",0,"Cardiometabolic_II","B13805","20212097_SS211647","PASS",-3.021,0.3313,"Plate control","PASS","PFC",1.25814656886037,"24h","24h"
9343,"PFC_01",1,"OID30399","O75339","CILP",0,"Cardiometabolic_II","B13805","20212097_SS211647","PASS",-4.5404,0.3675,"Plate control","PASS","PFC",1.29011529307386,"Baseline","Baseline"
9344,"PFC_09",2,"OID30399","O75339","CILP",0,"Cardiometabolic_II","B13805","20212097_SS211647","PASS",-4.5404,1.0038,"Plate control","PASS","PFC",2.00527486240903,"Baseline","Baseline"
9345,"PFC_17",3,"OID30399","O75339","CILP",0,"Cardiometabolic_II","B13805","20212097_SS211647","PASS",-4.5404,-0.2818,"Plate control","PASS","PFC",0.822564092405058,"1h","1h"
9346,"PFC_25",4,"OID30399","O75339","CILP",0,"Cardiometabolic_II","B13805","20212097_SS211647","PASS",-4.5404,0.0041,"Plate control","PASS","PFC",1.002845945476,"24h","24h"
9347,"PFC_02",13,"OID30399","O75339","CILP",0,"Cardiometabolic_II","B13805","20212097_SS211647","PASS",-4.5404,0.4512,"Plate control","PASS","PFC",1.36717696977752,"Baseline","Baseline"
9348,"PFC_10",14,"OID30399","O75339","CILP",0,"Cardiometabolic_II","B13805","20212097_SS211647","PASS",-4.5404,1.0369,"Plate control","PASS","PFC",2.05181406483176,"1h","1h"
9349,"PFC_18",15,"OID30399","O75339","CILP",0,"Cardiometabolic_II","B13805","20212097_SS211647","PASS",-4.5404,1.1207,"Plate control","PASS","PFC",2.17452455502314,"1h","1h"
9350,"PFC_26",16,"OID30399","O75339","CILP",0,"Cardiometabolic_II","B13805","20212097_SS211647","PASS",-4.5404,-0.8835,"Plate control","PASS","PFC",0.542050811368217,"24h","24h"
9351,"PFC_03",25,"OID30399","O75339","CILP",0,"Cardiometabolic_II","B13805","20212097_SS211647","PASS",-4.5404,-0.3135,"Plate control","PASS","PFC",0.804687205954719,"Baseline","Baseline"
9352,"PFC_11",26,"OID30399","O75339","CILP",0,"Cardiometabolic_II","B13805","20212097_SS211647","PASS",-4.5404,1.5742,"Plate control","PASS","PFC",2.97770327904795,"1h","1h"
9353,"PFC_19",27,"OID30399","O75339","CILP",0,"Cardiometabolic_II","B13805","20212097_SS211647","PASS",-4.5404,0.9685,"Plate control","PASS","PFC",1.95680500632567,"24h","24h"
9354,"PFC_27",28,"OID30399","O75339","CILP",0,"Cardiometabolic_II","B13805","20212097_SS211647","PASS",-4.5404,0.4555,"Plate control","PASS","PFC",1.37125796443202,"24h","24h"
9355,"PFC_04",37,"OID30399","O75339","CILP",0,"Cardiometabolic_II","B13805","20212097_SS211647","PASS",-4.5404,-0.259,"Plate control","PASS","PFC",0.835666958921853,"Baseline","Baseline"
9356,"PFC_12",38,"OID30399","O75339","CILP",0,"Cardiometabolic_II","B13805","20212097_SS211647","PASS",-4.5404,-0.8858,"Plate control","PASS","PFC",0.541187341558787,"1h","1h"
9357,"PFC_20",39,"OID30399","O75339","CILP",0,"Cardiometabolic_II","B13805","20212097_SS211647","PASS",-4.5404,2.1428,"Plate control","PASS","PFC",4.41618313323751,"24h","24h"
9358,"PFC_05",49,"OID30399","O75339","CILP",0,"Cardiometabolic_II","B13805","20212097_SS211647","PASS",-4.5404,-0.9755,"Plate control","PASS","PFC",0.508563560806048,"Baseline","Baseline"
9359,"PFC_13",50,"OID30399","O75339","CILP",0,"Cardiometabolic_II","B13805","20212097_SS211647","PASS",-4.5404,-0.439,"Plate control","PASS","PFC",0.73764572854142,"1h","1h"
9360,"PFC_21",51,"OID30399","O75339","CILP",0,"Cardiometabolic_II","B13805","20212097_SS211647","PASS",-4.5404,0.0637,"Plate control","PASS","PFC",1.04514274628285,"24h","24h"
9361,"PFC_06",61,"OID30399","O75339","CILP",0,"Cardiometabolic_II","B13805","20212097_SS211647","PASS",-4.5404,-0.6257,"Plate control","PASS","PFC",0.648105238400964,"Baseline","Baseline"
9362,"PFC_14",62,"OID30399","O75339","CILP",0,"Cardiometabolic_II","B13805","20212097_SS211647","PASS",-4.5404,2.0479,"Plate control","PASS","PFC",4.13503631618177,"1h","1h"
9363,"PFC_22",63,"OID30399","O75339","CILP",0,"Cardiometabolic_II","B13805","20212097_SS211647","PASS",-4.5404,0.1703,"Plate control","PASS","PFC",1.12529245834968,"24h","24h"
9364,"PFC_07",73,"OID30399","O75339","CILP",0,"Cardiometabolic_II","B13805","20212097_SS211647","PASS",-4.5404,-0.5586,"Plate control","PASS","PFC",0.678960711726935,"Baseline","Baseline"
9365,"PFC_15",74,"OID30399","O75339","CILP",0,"Cardiometabolic_II","B13805","20212097_SS211647","PASS",-4.5404,-0.1903,"Plate control","PASS","PFC",0.876423455232017,"1h","1h"
9366,"PFC_23",75,"OID30399","O75339","CILP",0,"Cardiometabolic_II","B13805","20212097_SS211647","PASS",-4.5404,0.6115,"Plate control","PASS","PFC",1.5278469176056,"24h","24h"
9367,"PFC_08",85,"OID30399","O75339","CILP",0,"Cardiometabolic_II","B13805","20212097_SS211647","PASS",-4.5404,-1.2197,"Plate control","PASS","PFC",0.429371994332517,"Baseline","Baseline"
9368,"PFC_16",86,"OID30399","O75339","CILP",0,"Cardiometabolic_II","B13805","20212097_SS211647","PASS",-4.5404,-0.6506,"Plate control","PASS","PFC",0.637015331333392,"1h","1h"
9369,"PFC_24",87,"OID30399","O75339","CILP",0,"Cardiometabolic_II","B13805","20212097_SS211647","PASS",-4.5404,1.241,"Plate control","PASS","PFC",2.36362309386873,"24h","24h"
9370,"PFC_01",1,"OID30400","P12277","CKB",0,"Cardiometabolic_II","B13805","20212097_SS211647","PASS",-2.8302,0.6397,"Plate control","PASS","PFC",1.55800514757139,"Baseline","Baseline"
9371,"PFC_09",2,"OID30400","P12277","CKB",0,"Cardiometabolic_II","B13805","20212097_SS211647","PASS",-2.8302,-0.0993,"Plate control","PASS","PFC",0.933485811814025,"Baseline","Baseline"
9372,"PFC_17",3,"OID30400","P12277","CKB",0,"Cardiometabolic_II","B13805","20212097_SS211647","PASS",-2.8302,1.4397,"Plate control","PASS","PFC",2.71264451767307,"1h","1h"
9373,"PFC_25",4,"OID30400","P12277","CKB",0,"Cardiometabolic_II","B13805","20212097_SS211647","PASS",-2.8302,0.1365,"Plate control","PASS","PFC",1.09923511742936,"24h","24h"
9374,"PFC_02",13,"OID30400","P12277","CKB",0,"Cardiometabolic_II","B13805","20212097_SS211647","PASS",-2.8302,1.1333,"Plate control","PASS","PFC",2.19359927589862,"Baseline","Baseline"
9375,"PFC_10",14,"OID30400","P12277","CKB",0,"Cardiometabolic_II","B13805","20212097_SS211647","PASS",-2.8302,0.4559,"Plate control","PASS","PFC",1.37163821057964,"1h","1h"
9376,"PFC_18",15,"OID30400","P12277","CKB",0,"Cardiometabolic_II","B13805","20212097_SS211647","PASS",-2.8302,-0.4632,"Plate control","PASS","PFC",0.725375538544712,"1h","1h"
9377,"PFC_26",16,"OID30400","P12277","CKB",0,"Cardiometabolic_II","B13805","20212097_SS211647","PASS",-2.8302,0.9209,"Plate control","PASS","PFC",1.89329602464498,"24h","24h"
9378,"PFC_03",25,"OID30400","P12277","CKB",0,"Cardiometabolic_II","B13805","20212097_SS211647","PASS",-2.8302,0.584,"Plate control","PASS","PFC",1.49899960175555,"Baseline","Baseline"
9379,"PFC_11",26,"OID30400","P12277","CKB",0,"Cardiometabolic_II","B13805","20212097_SS211647","PASS",-2.8302,0.7822,"Plate control","PASS","PFC",1.71975136412344,"1h","1h"
9380,"PFC_19",27,"OID30400","P12277","CKB",0,"Cardiometabolic_II","B13805","20212097_SS211647","PASS",-2.8302,0.7467,"Plate control","PASS","PFC",1.67795031797303,"24h","24h"
9381,"PFC_27",28,"OID30400","P12277","CKB",0,"Cardiometabolic_II","B13805","20212097_SS211647","PASS",-2.8302,-0.2508,"Plate control","PASS","PFC",0.84043025252991,"24h","24h"
9382,"PFC_04",37,"OID30400","P12277","CKB",0,"Cardiometabolic_II","B13805","20212097_SS211647","PASS",-2.8302,1.4445,"Plate control","PASS","PFC",2.72168480545022,"Baseline","Baseline"
9383,"PFC_12",38,"OID30400","P12277","CKB",0,"Cardiometabolic_II","B13805","20212097_SS211647","PASS",-2.8302,0.4194,"Plate control","PASS","PFC",1.33737124204868,"1h","1h"
9384,"PFC_20",39,"OID30400","P12277","CKB",0,"Cardiometabolic_II","B13805","20212097_SS211647","PASS",-2.8302,0.4403,"Plate control","PASS","PFC",1.35688645471943,"24h","24h"
9385,"PFC_05",49,"OID30400","P12277","CKB",0,"Cardiometabolic_II","B13805","20212097_SS211647","PASS",-2.8302,-0.4238,"Plate control","PASS","PFC",0.745458528732118,"Baseline","Baseline"
9386,"PFC_13",50,"OID30400","P12277","CKB",0,"Cardiometabolic_II","B13805","20212097_SS211647","PASS",-2.8302,1.4463,"Plate control","PASS","PFC",2.72508267537969,"1h","1h"
9387,"PFC_21",51,"OID30400","P12277","CKB",0,"Cardiometabolic_II","B13805","20212097_SS211647","PASS",-2.8302,0.6955,"Plate control","PASS","PFC",1.61944559292101,"24h","24h"
9388,"PFC_06",61,"OID30400","P12277","CKB",0,"Cardiometabolic_II","B13805","20212097_SS211647","PASS",-2.8302,0.4941,"Plate control","PASS","PFC",1.40844184927839,"Baseline","Baseline"
9389,"PFC_14",62,"OID30400","P12277","CKB",0,"Cardiometabolic_II","B13805","20212097_SS211647","PASS",-2.8302,-0.2548,"Plate control","PASS","PFC",0.838103312404538,"1h","1h"
9390,"PFC_22",63,"OID30400","P12277","CKB",0,"Cardiometabolic_II","B13805","20212097_SS211647","PASS",-2.8302,1.2545,"Plate control","PASS","PFC",2.38584447207658,"24h","24h"
9391,"PFC_07",73,"OID30400","P12277","CKB",0,"Cardiometabolic_II","B13805","20212097_SS211647","PASS",-2.8302,0.2995,"Plate control","PASS","PFC",1.23071780513527,"Baseline","Baseline"
9392,"PFC_15",74,"OID30400","P12277","CKB",0,"Cardiometabolic_II","B13805","20212097_SS211647","PASS",-2.8302,0.686,"Plate control","PASS","PFC",1.60881674188383,"1h","1h"
9393,"PFC_23",75,"OID30400","P12277","CKB",0,"Cardiometabolic_II","B13805","20212097_SS211647","PASS",-2.8302,-0.4515,"Plate control","PASS","PFC",0.731282123357752,"24h","24h"
9394,"PFC_08",85,"OID30400","P12277","CKB",0,"Cardiometabolic_II","B13805","20212097_SS211647","PASS",-2.8302,0.6967,"Plate control","PASS","PFC",1.62079317026114,"Baseline","Baseline"
9395,"PFC_16",86,"OID30400","P12277","CKB",0,"Cardiometabolic_II","B13805","20212097_SS211647","PASS",-2.8302,0.7048,"Plate control","PASS","PFC",1.62991869443302,"1h","1h"
9396,"PFC_24",87,"OID30400","P12277","CKB",0,"Cardiometabolic_II","B13805","20212097_SS211647","PASS",-2.8302,0.5775,"Plate control","PASS","PFC",1.49226111537404,"24h","24h"
9397,"PFC_01",1,"OID30401","Q8WWQ8","STAB2",0,"Cardiometabolic_II","B13805","20212097_SS211647","PASS",-6.3747,0.6396,"Plate control","PASS","PFC",1.55789715862651,"Baseline","Baseline"
9398,"PFC_09",2,"OID30401","Q8WWQ8","STAB2",0,"Cardiometabolic_II","B13805","20212097_SS211647","PASS",-6.3747,1.1191,"Plate control","PASS","PFC",2.17211426691471,"Baseline","Baseline"
9399,"PFC_17",3,"OID30401","Q8WWQ8","STAB2",0,"Cardiometabolic_II","B13805","20212097_SS211647","PASS",-6.3747,0.3273,"Plate control","PASS","PFC",1.25466307724894,"1h","1h"
9400,"PFC_25",4,"OID30401","Q8WWQ8","STAB2",0,"Cardiometabolic_II","B13805","20212097_SS211647","PASS",-6.3747,-0.4725,"Plate control","PASS","PFC",0.720714611790848,"24h","24h"
9401,"PFC_02",13,"OID30401","Q8WWQ8","STAB2",0,"Cardiometabolic_II","B13805","20212097_SS211647","PASS",-6.3747,0.6619,"Plate control","PASS","PFC",1.5821649312539,"Baseline","Baseline"
9402,"PFC_10",14,"OID30401","Q8WWQ8","STAB2",0,"Cardiometabolic_II","B13805","20212097_SS211647","PASS",-6.3747,0.7616,"Plate control","PASS","PFC",1.69536980777572,"1h","1h"
9403,"PFC_18",15,"OID30401","Q8WWQ8","STAB2",0,"Cardiometabolic_II","B13805","20212097_SS211647","PASS",-6.3747,1.0018,"Plate control","PASS","PFC",2.00249688716538,"1h","1h"
9404,"PFC_26",16,"OID30401","Q8WWQ8","STAB2",0,"Cardiometabolic_II","B13805","20212097_SS211647","PASS",-6.3747,0.2191,"Plate control","PASS","PFC",1.16400721443783,"24h","24h"
9405,"PFC_03",25,"OID30401","Q8WWQ8","STAB2",0,"Cardiometabolic_II","B13805","20212097_SS211647","PASS",-6.3747,0.3934,"Plate control","PASS","PFC",1.31348525038259,"Baseline","Baseline"
9406,"PFC_11",26,"OID30401","Q8WWQ8","STAB2",0,"Cardiometabolic_II","B13805","20212097_SS211647","PASS",-6.3747,0.8763,"Plate control","PASS","PFC",1.83566144000325,"1h","1h"
9407,"PFC_19",27,"OID30401","Q8WWQ8","STAB2",0,"Cardiometabolic_II","B13805","20212097_SS211647","PASS",-6.3747,0.6584,"Plate control","PASS","PFC",1.57833122738152,"24h","24h"
9408,"PFC_27",28,"OID30401","Q8WWQ8","STAB2",0,"Cardiometabolic_II","B13805","20212097_SS211647","PASS",-6.3747,1.117,"Plate control","PASS","PFC",2.16895481768613,"24h","24h"

Supplemental table 1_Olink proximity extension assay (PEA) data

,"SampleID","Index","OlinkID","UniProt","Assay","MissingFreq","Panel","Panel_Lot_Nr","PlateID","QC_Warning","LOD","NPX","Normalization","Assay_Warning","condition","Value","group","alt_group"
9409,"PFC_04",37,"OID30401","Q8WWQ8","STAB2",0,"Cardiometabolic_II","B13805","20212097_SS211647","PASS",-6.3747,0.1264,"Plate control","PASS","PFC",1.09156648159474,"Baseline","Baseline"
9410,"PFC_12",38,"OID30401","Q8WWQ8","STAB2",0,"Cardiometabolic_II","B13805","20212097_SS211647","PASS",-6.3747,0.3886,"Plate control","PASS","PFC",1.30912240695901,"1h","1h"
9411,"PFC_20",39,"OID30401","Q8WWQ8","STAB2",0,"Cardiometabolic_II","B13805","20212097_SS211647","PASS",-6.3747,0.8371,"Plate control","PASS","PFC",1.78645552807533,"24h","24h"
9412,"PFC_05",49,"OID30401","Q8WWQ8","STAB2",0,"Cardiometabolic_II","B13805","20212097_SS211647","PASS",-6.3747,0.6832,"Plate control","PASS","PFC",1.60569735892281,"Baseline","Baseline"
9413,"PFC_13",50,"OID30401","Q8WWQ8","STAB2",0,"Cardiometabolic_II","B13805","20212097_SS211647","PASS",-6.3747,0.1579,"Plate control","PASS","PFC",1.11566198784865,"1h","1h"
9414,"PFC_21",51,"OID30401","Q8WWQ8","STAB2",0,"Cardiometabolic_II","B13805","20212097_SS211647","PASS",-6.3747,0.3804,"Plate control","PASS","PFC",1.30170271404046,"24h","24h"
9415,"PFC_06",61,"OID30401","Q8WWQ8","STAB2",0,"Cardiometabolic_II","B13805","20212097_SS211647","PASS",-6.3747,0.5113,"Plate control","PASS","PFC",1.42533397320211,"Baseline","Baseline"
9416,"PFC_14",62,"OID30401","Q8WWQ8","STAB2",0,"Cardiometabolic_II","B13805","20212097_SS211647","PASS",-6.3747,0.7468,"Plate control","PASS","PFC",1.67806662865721,"1h","1h"
9417,"PFC_22",63,"OID30401","Q8WWQ8","STAB2",0,"Cardiometabolic_II","B13805","20212097_SS211647","PASS",-6.3747,-0.0125,"Plate control","PASS","PFC",0.991373087462662,"24h","24h"
9418,"PFC_07",73,"OID30401","Q8WWQ8","STAB2",0,"Cardiometabolic_II","B13805","20212097_SS211647","PASS",-6.3747,-0.1443,"Plate control","PASS","PFC",0.904818291644697,"Baseline","Baseline"
9419,"PFC_15",74,"OID30401","Q8WWQ8","STAB2",0,"Cardiometabolic_II","B13805","20212097_SS211647","PASS",-6.3747,0.305,"Plate control","PASS","PFC",1.23541863712693,"1h","1h"
9420,"PFC_23",75,"OID30401","Q8WWQ8","STAB2",0,"Cardiometabolic_II","B13805","20212097_SS211647","PASS",-6.3747,0.7893,"Plate control","PASS","PFC",1.72823571395176,"24h","24h"
9421,"PFC_08",85,"OID30401","Q8WWQ8","STAB2",0,"Cardiometabolic_II","B13805","20212097_SS211647","PASS",-6.3747,0.4115,"Plate control","PASS","PFC",1.3300679947518,"Baseline","Baseline"
9422,"PFC_16",86,"OID30401","Q8WWQ8","STAB2",0,"Cardiometabolic_II","B13805","20212097_SS211647","PASS",-6.3747,-0.4834,"Plate control","PASS","PFC",0.715289912041534,"1h","1h"
9423,"PFC_24",87,"OID30401","Q8WWQ8","STAB2",0,"Cardiometabolic_II","B13805","20212097_SS211647","PASS",-6.3747,0.4636,"Plate control","PASS","PFC",1.37897853478603,"24h","24h"
9424,"PFC_01",1,"OID30402","Q8TDY8","IGDCC4",0,"Cardiometabolic_II","B13805","20212097_SS211647","PASS",-4.0923,0.471,"Plate control","PASS","PFC",1.38606988565041,"Baseline","Baseline"
9425,"PFC_09",2,"OID30402","Q8TDY8","IGDCC4",0,"Cardiometabolic_II","B13805","20212097_SS211647","PASS",-4.0923,0.4285,"Plate control","PASS","PFC",1.34583355807308,"Baseline","Baseline"
9426,"PFC_17",3,"OID30402","Q8TDY8","IGDCC4",0,"Cardiometabolic_II","B13805","20212097_SS211647","PASS",-4.0923,0.4485,"Plate control","PASS","PFC",1.36462069442774,"1h","1h"
9427,"PFC_25",4,"OID30402","Q8TDY8","IGDCC4",0,"Cardiometabolic_II","B13805","20212097_SS211647","PASS",-4.0923,0.3983,"Plate control","PASS","PFC",1.31795398406789,"24h","24h"
9428,"PFC_02",13,"OID30402","Q8TDY8","IGDCC4",0,"Cardiometabolic_II","B13805","20212097_SS211647","PASS",-4.0923,0.2022,"Plate control","PASS","PFC",1.15045136872088,"Baseline","Baseline"
9429,"PFC_10",14,"OID30402","Q8TDY8","IGDCC4",0,"Cardiometabolic_II","B13805","20212097_SS211647","PASS",-4.0923,0.5201,"Plate control","PASS","PFC",1.43405464566437,"1h","1h"
9430,"PFC_18",15,"OID30402","Q8TDY8","IGDCC4",0,"Cardiometabolic_II","B13805","20212097_SS211647","PASS",-4.0923,0.3694,"Plate control","PASS","PFC",1.29181546795416,"1h","1h"
9431,"PFC_26",16,"OID30402","Q8TDY8","IGDCC4",0,"Cardiometabolic_II","B13805","20212097_SS211647","PASS",-4.0923,0.5486,"Plate control","PASS","PFC",1.46266562745745,"24h","24h"
9432,"PFC_03",25,"OID30402","Q8TDY8","IGDCC4",0,"Cardiometabolic_II","B13805","20212097_SS211647","PASS",-4.0923,0.4246,"Plate control","PASS","PFC",1.34220031424094,"Baseline","Baseline"
9433,"PFC_11",26,"OID30402","Q8TDY8","IGDCC4",0,"Cardiometabolic_II","B13805","20212097_SS211647","PASS",-4.0923,0.1239,"Plate control","PASS","PFC",1.08967657897073,"1h","1h"
9434,"PFC_19",27,"OID30402","Q8TDY8","IGDCC4",0,"Cardiometabolic_II","B13805","20212097_SS211647","PASS",-4.0923,0.5108,"Plate control","PASS","PFC",1.42484007568054,"24h","24h"
9435,"PFC_27",28,"OID30402","Q8TDY8","IGDCC4",0,"Cardiometabolic_II","B13805","20212097_SS211647","PASS",-4.0923,0.584,"Plate control","PASS","PFC",1.49899960175555,"24h","24h"
9436,"PFC_04",37,"OID30402","Q8TDY8","IGDCC4",0,"Cardiometabolic_II","B13805","20212097_SS211647","PASS",-4.0923,0.3289,"Plate control","PASS","PFC",1.25605531500606,"Baseline","Baseline"
9437,"PFC_12",38,"OID30402","Q8TDY8","IGDCC4",0,"Cardiometabolic_II","B13805","20212097_SS211647","PASS",-4.0923,0.3353,"Plate control","PASS","PFC",1.26163973216297,"1h","1h"
9438,"PFC_20",39,"OID30402","Q8TDY8","IGDCC4",0,"Cardiometabolic_II","B13805","20212097_SS211647","PASS",-4.0923,0.4144,"Plate control","PASS","PFC",1.33274428904962,"24h","24h"
9439,"PFC_05",49,"OID30402","Q8TDY8","IGDCC4",0,"Cardiometabolic_II","B13805","20212097_SS211647","PASS",-4.0923,0.1253,"Plate control","PASS","PFC",1.09073452095217,"Baseline","Baseline"
9440,"PFC_13",50,"OID30402","Q8TDY8","IGDCC4",0,"Cardiometabolic_II","B13805","20212097_SS211647","PASS",-4.0923,0.3556,"Plate control","PASS","PFC",1.27951760705119,"1h","1h"
9441,"PFC_21",51,"OID30402","Q8TDY8","IGDCC4",0,"Cardiometabolic_II","B13805","20212097_SS211647","PASS",-4.0923,0.4641,"Plate control","PASS","PFC",1.37945653515412,"24h","24h"
9442,"PFC_06",61,"OID30402","Q8TDY8","IGDCC4",0,"Cardiometabolic_II","B13805","20212097_SS211647","PASS",-4.0923,0.6984,"Plate control","PASS","PFC",1.62270415791398,"Baseline","Baseline"
9443,"PFC_14",62,"OID30402","Q8TDY8","IGDCC4",0,"Cardiometabolic_II","B13805","20212097_SS211647","PASS",-4.0923,0.1568,"Plate control","PASS","PFC",1.11481166230279,"1h","1h"
9444,"PFC_22",63,"OID30402","Q8TDY8","IGDCC4",0,"Cardiometabolic_II","B13805","20212097_SS211647","PASS",-4.0923,0.1344,"Plate control","PASS","PFC",1.09763622477592,"24h","24h"
9445,"PFC_07",73,"OID30402","Q8TDY8","IGDCC4",0,"Cardiometabolic_II","B13805","20212097_SS211647","PASS",-4.0923,0.5877,"Plate control","PASS","PFC",1.50284893692011,"Baseline","Baseline"
9446,"PFC_15",74,"OID30402","Q8TDY8","IGDCC4",0,"Cardiometabolic_II","B13805","20212097_SS211647","PASS",-4.0923,0.615,"Plate control","PASS","PFC",1.53155799709438,"1h","1h"
9447,"PFC_23",75,"OID30402","Q8TDY8","IGDCC4",0,"Cardiometabolic_II","B13805","20212097_SS211647","PASS",-4.0923,0.2768,"Plate control","PASS","PFC",1.21150469857507,"24h","24h"
9448,"PFC_08",85,"OID30402","Q8TDY8","IGDCC4",0,"Cardiometabolic_II","B13805","20212097_SS211647","PASS",-4.0923,0.4783,"Plate control","PASS","PFC",1.39310113778951,"Baseline","Baseline"
9449,"PFC_16",86,"OID30402","Q8TDY8","IGDCC4",0,"Cardiometabolic_II","B13805","20212097_SS211647","PASS",-4.0923,0.2333,"Plate control","PASS","PFC",1.17552074567349,"1h","1h"
9450,"PFC_24",87,"OID30402","Q8TDY8","IGDCC4",0,"Cardiometabolic_II","B13805","20212097_SS211647","PASS",-4.0923,0.6046,"Plate control","PASS","PFC",1.52055710684251,"24h","24h"
9451,"PFC_01",1,"OID30403","P10645","CHGA",0,"Cardiometabolic_II","B13805","20212097_SS211647","PASS",-3.6842,2.0966,"Plate control","PASS","PFC",4.27700235028575,"Baseline","Baseline"
9452,"PFC_09",2,"OID30403","P10645","CHGA",0,"Cardiometabolic_II","B13805","20212097_SS211647","PASS",-3.6842,0.8218,"Plate control","PASS","PFC",1.76761000236359,"Baseline","Baseline"
9453,"PFC_17",3,"OID30403","P10645","CHGA",0,"Cardiometabolic_II","B13805","20212097_SS211647","PASS",-3.6842,0.6674,"Plate control","PASS","PFC",1.58820814562351,"1h","1h"
9454,"PFC_25",4,"OID30403","P10645","CHGA",0,"Cardiometabolic_II","B13805","20212097_SS211647","PASS",-3.6842,0.9612,"Plate control","PASS","PFC",1.94692863122748,"24h","24h"
9455,"PFC_02",13,"OID30403","P10645","CHGA",0,"Cardiometabolic_II","B13805","20212097_SS211647","PASS",-3.6842,1.6887,"Plate control","PASS","PFC",3.22366091482799,"Baseline","Baseline"
9456,"PFC_10",14,"OID30403","P10645","CHGA",0,"Cardiometabolic_II","B13805","20212097_SS211647","PASS",-3.6842,1.42,"Plate control","PASS","PFC",2.67585510957222,"1h","1h"
9457,"PFC_18",15,"OID30403","P10645","CHGA",0,"Cardiometabolic_II","B13805","20212097_SS211647","PASS",-3.6842,0.562,"Plate control","PASS","PFC",1.47631440568283,"1h","1h"
9458,"PFC_26",16,"OID30403","P10645","CHGA",0,"Cardiometabolic_II","B13805","20212097_SS211647","PASS",-3.6842,0.9886,"Plate control","PASS","PFC",1.98425851981774,"24h","24h"
9459,"PFC_03",25,"OID30403","P10645","CHGA",0,"Cardiometabolic_II","B13805","20212097_SS211647","PASS",-3.6842,1.2501,"Plate control","PASS","PFC",2.37857909483093,"Baseline","Baseline"
9460,"PFC_11",26,"OID30403","P10645","CHGA",0,"Cardiometabolic_II","B13805","20212097_SS211647","PASS",-3.6842,1.7384,"Plate control","PASS","PFC",3.3366491626473,"1h","1h"
9461,"PFC_19",27,"OID30403","P10645","CHGA",0,"Cardiometabolic_II","B13805","20212097_SS211647","PASS",-3.6842,2.1114,"Plate control","PASS","PFC",4.32110413771986,"24h","24h"
9462,"PFC_27",28,"OID30403","P10645","CHGA",0,"Cardiometabolic_II","B13805","20212097_SS211647","PASS",-3.6842,0.3654,"Plate control","PASS","PFC",1.28823875562389,"24h","24h"
9463,"PFC_04",37,"OID30403","P10645","CHGA",0,"Cardiometabolic_II","B13805","20212097_SS211647","PASS",-3.6842,2.0369,"Plate control","PASS","PFC",4.10362812966352,"Baseline","Baseline"
9464,"PFC_12",38,"OID30403","P10645","CHGA",0,"Cardiometabolic_II","B13805","20212097_SS211647","PASS",-3.6842,1.1044,"Plate control","PASS","PFC",2.15009439582445,"1h","1h"
9465,"PFC_20",39,"OID30403","P10645","CHGA",0,"Cardiometabolic_II","B13805","20212097_SS211647","PASS",-3.6842,1.7257,"Plate control","PASS","PFC",3.30740564659105,"24h","24h"
9466,"PFC_05",49,"OID30403","P10645","CHGA",0,"Cardiometabolic_II","B13805","20212097_SS211647","PASS",-3.6842,0.6299,"Plate control","PASS","PFC",1.54745772822876,"Baseline","Baseline"
9467,"PFC_13",50,"OID30403","P10645","CHGA",0,"Cardiometabolic_II","B13805","20212097_SS211647","PASS",-3.6842,1.524,"Plate control","PASS","PFC",2.87587306569391,"1h","1h"
9468,"PFC_21",51,"OID30403","P10645","CHGA",0,"Cardiometabolic_II","B13805","20212097_SS211647","PASS",-3.6842,1.2608,"Plate control","PASS","PFC",2.39628582389919,"24h","24h"
9469,"PFC_06",61,"OID30403","P10645","CHGA",0,"Cardiometabolic_II","B13805","20212097_SS211647","PASS",-3.6842,1.3107,"Plate control","PASS","PFC",2.48061871163532,"Baseline","Baseline"
9470,"PFC_14",62,"OID30403","P10645","CHGA",0,"Cardiometabolic_II","B13805","20212097_SS211647","PASS",-3.6842,0.197,"Plate control","PASS","PFC",1.14631218572838,"1h","1h"
9471,"PFC_22",63,"OID30403","P10645","CHGA",0,"Cardiometabolic_II","B13805","20212097_SS211647","PASS",-3.6842,2.1333,"Plate control","PASS","PFC",4.38719855179725,"24h","24h"
9472,"PFC_07",73,"OID30403","P10645","CHGA",0,"Cardiometabolic_II","B13805","20212097_SS211647","PASS",-3.6842,0.4872,"Plate control","PASS","PFC",1.40172175550869,"Baseline","Baseline"
9473,"PFC_15",74,"OID30403","P10645","CHGA",0,"Cardiometabolic_II","B13805","20212097_SS211647","PASS",-3.6842,1.295,"Plate control","PASS","PFC",2.45376995450761,"1h","1h"
9474,"PFC_23",75,"OID30403","P10645","CHGA",0,"Cardiometabolic_II","B13805","20212097_SS211647","PASS",-3.6842,0.4735,"Plate control","PASS","PFC",1.3884738440033,"24h","24h"
9475,"PFC_08",85,"OID30403","P10645","CHGA",0,"Cardiometabolic_II","B13805","20212097_SS211647","PASS",-3.6842,0.7988,"Plate control","PASS","PFC",1.73965352151338,"Baseline","Baseline"
9476,"PFC_16",86,"OID30403","P10645","CHGA",0,"Cardiometabolic_II","B13805","20212097_SS211647","PASS",-3.6842,0.617,"Plate control","PASS","PFC",1.53368265967309,"1h","1h"
9477,"PFC_24",87,"OID30403","P10645","CHGA",0,"Cardiometabolic_II","B13805","20212097_SS211647","PASS",-3.6842,2.1243,"Plate control","PASS","PFC",4.3599149734282,"24h","24h"
9478,"PFC_01",1,"OID30404","P55000","SLURP1",0,"Cardiometabolic_II","B13805","20212097_SS211647","PASS",-1.8687,0.4121,"Plate control","PASS","PFC",1.33062126952239,"Baseline","Baseline"
9479,"PFC_09",2,"OID30404","P55000","SLURP1",0,"Cardiometabolic_II","B13805","20212097_SS211647","PASS",-1.8687,0.1686,"Plate control","PASS","PFC",1.12396725068121,"Baseline","Baseline"
9480,"PFC_17",3,"OID30404","P55000","SLURP1",0,"Cardiometabolic_II","B13805","20212097_SS211647","PASS",-1.8687,-0.3538,"Plate control","PASS","PFC",0.78252025455921,"1h","1h"
9481,"PFC_25",4,"OID30404","P55000","SLURP1",0,"Cardiometabolic_II","B13805","20212097_SS211647","PASS",-1.8687,-0.3179,"Plate control","PASS","PFC",0.802236771240958,"24h","24h"
9482,"PFC_02",13,"OID30404","P55000","SLURP1",0,"Cardiometabolic_II","B13805","20212097_SS211647","PASS",-1.8687,0.0094,"Plate control","PASS","PFC",1.00653685608743,"Baseline","Baseline"
9483,"PFC_10",14,"OID30404","P55000","SLURP1",0,"Cardiometabolic_II","B13805","20212097_SS211647","PASS",-1.8687,0.9177,"Plate control","PASS","PFC",1.88910121359449,"1h","1h"
9484,"PFC_18",15,"OID30404","P55000","SLURP1",0,"Cardiometabolic_II","B13805","20212097_SS211647","PASS",-1.8687,0.2311,"Plate control","PASS","PFC",1.17372953219341,"1h","1h"
9485,"PFC_26",16,"OID30404","P55000","SLURP1",0,"Cardiometabolic_II","B13805","20212097_SS211647","PASS",-1.8687,-0.4179,"Plate control","PASS","PFC",0.748513374591781,"24h","24h"
9486,"PFC_03",25,"OID30404","P55000","SLURP1",0,"Cardiometabolic_II","B13805","20212097_SS211647","PASS",-1.8687,0.1767,"Plate control","PASS","PFC",1.13029550434286,"Baseline","Baseline"
9487,"PFC_11",26,"OID30404","P55000","SLURP1",0,"Cardiometabolic_II","B13805","20212097_SS211647","PASS",-1.8687,-0.0811,"Plate control","PASS","PFC",0.945336588740626,"1h","1h"
9488,"PFC_19",27,"OID30404","P55000","SLURP1",0,"Cardiometabolic_II","B13805","20212097_SS211647","PASS",-1.8687,0.0612,"Plate control","PASS","PFC",1.04333322020086,"24h","24h"
9489,"PFC_27",28,"OID30404","P55000","SLURP1",0,"Cardiometabolic_II","B13805","20212097_SS211647","PASS",-1.8687,0.0191,"Plate control","PASS","PFC",1.01332713620961,"24h","24h"
9490,"PFC_04",37,"OID30404","P55000","SLURP1",0,"Cardiometabolic_II","B13805","20212097_SS211647","PASS",-1.8687,0.4215,"Plate control","PASS","PFC",1.33931934926814,"Baseline","Baseline"
9491,"PFC_12",38,"OID30404","P55000","SLURP1",0,"Cardiometabolic_II","B13805","20212097_SS211647","PASS",-1.8687,0.3684,"Plate control","PASS","PFC",1.29092035996137,"1h","1h"
9492,"PFC_20",39,"OID30404","P55000","SLURP1",0,"Cardiometabolic_II","B13805","20212097_SS211647","PASS",-1.8687,-0.6395,"Plate control","PASS","PFC",0.641935388085608,"24h","24h"
9493,"PFC_05",49,"OID30404","P55000","SLURP1",0,"Cardiometabolic_II","B13805","20212097_SS211647","PASS",-1.8687,0.165,"Plate control","PASS","PFC",1.12116607802851,"Baseline","Baseline"
9494,"PFC_13",50,"OID30404","P55000","SLURP1",0,"Cardiometabolic_II","B13805","20212097_SS211647","PASS",-1.8687,-0.0743,"Plate control","PASS","PFC",0.949802856386761,"1h","1h"
9495,"PFC_21",51,"OID30404","P55000","SLURP1",0,"Cardiometabolic_II","B13805","20212097_SS211647","PASS",-1.8687,0.2634,"Plate control","PASS","PFC",1.20030413121205,"24h","24h"
9496,"PFC_06",61,"OID30404","P55000","SLURP1",0,"Cardiometabolic_II","B13805","20212097_SS211647","PASS",-1.8687,-0.7023,"Plate control","PASS","PFC",0.614591619604164,"Baseline","Baseline"
9497,"PFC_14",62,"OID30404","P55000","SLURP1",0,"Cardiometabolic_II","B13805","20212097_SS211647","PASS",-1.8687,0.2523,"Plate control","PASS","PFC",1.19110450683381,"1h","1h"
9498,"PFC_22",63,"OID30404","P55000","SLURP1",0,"Cardiometabolic_II","B13805","20212097_SS211647","PASS",-1.8687,0.0542,"Plate control","PASS","PFC",1.03828319718074,"24h","24h"
9499,"PFC_07",73,"OID30404","P55000","SLURP1",0,"Cardiometabolic_II","B13805","20212097_SS211647","PASS",-1.8687,-0.8447,"Plate control","PASS","PFC",0.556826585909797,"Baseline","Baseline"
9500,"PFC_15",74,"OID30404","P55000","SLURP1",0,"Cardiometabolic_II","B13805","20212097_SS211647","PASS",-1.8687,-0.379,"Plate control","PASS","PFC",0.768970415635134,"1h","1h"
9501,"PFC_23",75,"OID30404","P55000","SLURP1",0,"Cardiometabolic_II","B13805","20212097_SS211647","PASS",-1.8687,0.162,"Plate control","PASS","PFC",1.11883710103571,"24h","24h"
9502,"PFC_08",85,"OID30404","P55000","SLURP1",0,"Cardiometabolic_II","B13805","20212097_SS211647","PASS",-1.8687,-0.8877,"Plate control","PASS","PFC",0.540475077968143,"Baseline","Baseline"
9503,"PFC_16",86,"OID30404","P55000","SLURP1",0,"Cardiometabolic_II","B13805","20212097_SS211647","PASS",-1.8687,-0.3124,"Plate control","PASS","PFC",0.80530098325073,"1h","1h"
9504,"PFC_24",87,"OID30404","P55000","SLURP1",0,"Cardiometabolic_II","B13805","20212097_SS211647","PASS",-1.8687,-0.5397,"Plate control","PASS","PFC",0.687913941880723,"24h","24h"
9505,"PFC_01",1,"OID30405","Q9H2X3","CLEC4M",0,"Cardiometabolic_II","B13805","20212097_SS211647","PASS",-5.8832,0.528,"Plate control","PASS","PFC",1.44192887146022,"Baseline","Baseline"
9506,"PFC_09",2,"OID30405","Q9H2X3","CLEC4M",0,"Cardiometabolic_II","B13805","20212097_SS211647","PASS",-5.8832,1.1497,"Plate control","PASS","PFC",2.21867753514028,"Baseline","Baseline"
9507,"PFC_17",3,"OID30405","Q9H2X3","CLEC4M",0,"Cardiometabolic_II","B13805","20212097_SS211647","PASS",-5.8832,0.5044,"Plate control","PASS","PFC",1.41853328209942,"1h","1h"
9508,"PFC_25",4,"OID30405","Q9H2X3","CLEC4M",0,"Cardiometabolic_II","B13805","20212097_SS211647","PASS",-5.8832,0.2797,"Plate control","PASS","PFC",1.21394242591636,"24h","24h"
9509,"PFC_02",13,"OID30405","Q9H2X3","CLEC4M",0,"Cardiometabolic_II","B13805","20212097_SS211647","PASS",-5.8832,0.1882,"Plate control","PASS","PFC",1.13934131251831,"Baseline","Baseline"
9510,"PFC_10",14,"OID30405","Q9H2X3","CLEC4M",0,"Cardiometabolic_II","B13805","20212097_SS211647","PASS",-5.8832,0.4707,"Plate control","PASS","PFC",1.38578169048571,"1h","1h"
9511,"PFC_18",15,"OID30405","Q9H2X3","CLEC4M",0,"Cardiometabolic_II","B13805","20212097_SS211647","PASS",-5.8832,1.4325,"Plate control","PASS","PFC",2.69914035736118,"1h","1h"
9512,"PFC_26",16,"OID30405","Q9H2X3","CLEC4M",0,"Cardiometabolic_II","B13805","20212097_SS211647","PASS",-5.8832,0.5056,"Plate control","PASS","PFC",1.41971367575731,"24h","24h"
9513,"PFC_03",25,"OID30405","Q9H2X3","CLEC4M",0,"Cardiometabolic_II","B13805","20212097_SS211647","PASS",-5.8832,1.0747,"Plate control","PASS","PFC",2.10628403691535,"Baseline","Baseline"
9514,"PFC_11",26,"OID30405","Q9H2X3","CLEC4M",0,"Cardiometabolic_II","B13805","20212097_SS211647","PASS",-5.8832,0.4272,"Plate control","PASS","PFC",1.34462138533711,"1h","1h"
9515,"PFC_19",27,"OID30405","Q9H2X3","CLEC4M",0,"Cardiometabolic_II","B13805","20212097_SS211647","PASS",-5.8832,0.5954,"Plate control","PASS","PFC",1.51089143555068,"24h","24h"
9516,"PFC_27",28,"OID30405","Q9H2X3","CLEC4M",0,"Cardiometabolic_II","B13805","20212097_SS211647","PASS",-5.8832,1.7262,"Plate control","PASS","PFC",3.30855210469508,"24h","24h"
9517,"PFC_04",37,"OID30405","Q9H2X3","CLEC4M",0,"Cardiometabolic_II","B13805","20212097_SS211647","PASS",-5.8832,1.1232,"Plate control","PASS","PFC",2.17829598568598,"Baseline","Baseline"
9518,"PFC_12",38,"OID30405","Q9H2X3","CLEC4M",0,"Cardiometabolic_II","B13805","20212097_SS211647","PASS",-5.8832,1.1478,"Plate control","PASS","PFC",2.21575750522401,"1h","1h"
9519,"PFC_20",39,"OID30405","Q9H2X3","CLEC4M",0,"Cardiometabolic_II","B13805","20212097_SS211647","PASS",-5.8832,0.4089,"Plate control","PASS","PFC",1.32767312790441,"24h","24h"
9520,"PFC_05",49,"OID30405","Q9H2X3","CLEC4M",0,"Cardiometabolic_II","B13805","20212097_SS211647","PASS",-5.8832,-0.2419,"Plate control","PASS","PFC",0.845630900013548,"Baseline","Baseline"

Supplemental table 1_Olink proximity extension assay (PEA) data

,"SampleID","Index","OlinkID","UniProt","Assay","MissingFreq","Panel","Panel_Lot_Nr","PlateID","QC_Warning","LOD","NPX","Normalization","Assay_Warning","condition","Value","group","alt_group"
9521,"PFC_13",50,"OID30405","Q9H2X3","CLEC4M",0,"Cardiometabolic_II","B13805","20212097_SS211647","PASS",-5.8832,0.7949,"Plate control","PASS","PFC",1.73495711207315,"1h","1h"
9522,"PFC_21",51,"OID30405","Q9H2X3","CLEC4M",0,"Cardiometabolic_II","B13805","20212097_SS211647","PASS",-5.8832,0.9456,"Plate control","PASS","PFC",1.925989716269,"24h","24h"
9523,"PFC_06",61,"OID30405","Q9H2X3","CLEC4M",0,"Cardiometabolic_II","B13805","20212097_SS211647","PASS",-5.8832,0.7215,"Plate control","PASS","PFC",1.64889553453622,"Baseline","Baseline"
9524,"PFC_14",62,"OID30405","Q9H2X3","CLEC4M",0,"Cardiometabolic_II","B13805","20212097_SS211647","PASS",-5.8832,-0.1967,"Plate control","PASS","PFC",0.872544127357863,"1h","1h"
9525,"PFC_22",63,"OID30405","Q9H2X3","CLEC4M",0,"Cardiometabolic_II","B13805","20212097_SS211647","PASS",-5.8832,1.079,"Plate control","PASS","PFC",2.11257125070371,"24h","24h"
9526,"PFC_07",73,"OID30405","Q9H2X3","CLEC4M",0,"Cardiometabolic_II","B13805","20212097_SS211647","PASS",-5.8832,0.3186,"Plate control","PASS","PFC",1.2471197489599,"Baseline","Baseline"
9527,"PFC_15",74,"OID30405","Q9H2X3","CLEC4M",0,"Cardiometabolic_II","B13805","20212097_SS211647","PASS",-5.8832,1.1966,"Plate control","PASS","PFC",2.29198880912081,"1h","1h"
9528,"PFC_23",75,"OID30405","Q9H2X3","CLEC4M",0,"Cardiometabolic_II","B13805","20212097_SS211647","PASS",-5.8832,-0.302,"Plate control","PASS","PFC",0.811127155577043,"24h","24h"
9529,"PFC_08",85,"OID30405","Q9H2X3","CLEC4M",0,"Cardiometabolic_II","B13805","20212097_SS211647","PASS",-5.8832,0.4179,"Plate control","PASS","PFC",1.33598147200158,"Baseline","Baseline"
9530,"PFC_16",86,"OID30405","Q9H2X3","CLEC4M",0,"Cardiometabolic_II","B13805","20212097_SS211647","PASS",-5.8832,0.4407,"Plate control","PASS","PFC",1.35726271568603,"1h","1h"
9531,"PFC_24",87,"OID30405","Q9H2X3","CLEC4M",0,"Cardiometabolic_II","B13805","20212097_SS211647","PASS",-5.8832,1.0522,"Plate control","PASS","PFC",2.07368965654191,"24h","24h"
9532,"PFC_01",1,"OID30406","Q9Y646","CPQ",0,"Cardiometabolic_II","B13805","20212097_SS211647","PASS",-5.656,0.454,"Plate control","PASS","PFC",1.36983297996557,"Baseline","Baseline"
9533,"PFC_09",2,"OID30406","Q9Y646","CPQ",0,"Cardiometabolic_II","B13805","20212097_SS211647","PASS",-5.656,0.4391,"Plate control","PASS","PFC",1.35575829754733,"Baseline","Baseline"
9534,"PFC_17",3,"OID30406","Q9Y646","CPQ",0,"Cardiometabolic_II","B13805","20212097_SS211647","PASS",-5.656,0.206,"Plate control","PASS","PFC",1.15348560506002,"1h","1h"
9535,"PFC_25",4,"OID30406","Q9Y646","CPQ",0,"Cardiometabolic_II","B13805","20212097_SS211647","PASS",-5.656,0.8841,"Plate control","PASS","PFC",1.84561290901248,"24h","24h"
9536,"PFC_02",13,"OID30406","Q9Y646","CPQ",0,"Cardiometabolic_II","B13805","20212097_SS211647","PASS",-5.656,0.5512,"Plate control","PASS","PFC",1.46530399479833,"Baseline","Baseline"
9537,"PFC_10",14,"OID30406","Q9Y646","CPQ",0,"Cardiometabolic_II","B13805","20212097_SS211647","PASS",-5.656,0.7054,"Plate control","PASS","PFC",1.630596699539,"1h","1h"
9538,"PFC_18",15,"OID30406","Q9Y646","CPQ",0,"Cardiometabolic_II","B13805","20212097_SS211647","PASS",-5.656,0.591,"Plate control","PASS","PFC",1.50629046663381,"1h","1h"
9539,"PFC_26",16,"OID30406","Q9Y646","CPQ",0,"Cardiometabolic_II","B13805","20212097_SS211647","PASS",-5.656,0.2898,"Plate control","PASS","PFC",1.22247079550769,"24h","24h"
9540,"PFC_03",25,"OID30406","Q9Y646","CPQ",0,"Cardiometabolic_II","B13805","20212097_SS211647","PASS",-5.656,0.2729,"Plate control","PASS","PFC",1.20823409208194,"Baseline","Baseline"
9541,"PFC_11",26,"OID30406","Q9Y646","CPQ",0,"Cardiometabolic_II","B13805","20212097_SS211647","PASS",-5.656,0.5643,"Plate control","PASS","PFC",1.47866988006415,"1h","1h"
9542,"PFC_19",27,"OID30406","Q9Y646","CPQ",0,"Cardiometabolic_II","B13805","20212097_SS211647","PASS",-5.656,0.6993,"Plate control","PASS","PFC",1.6237167692618,"24h","24h"
9543,"PFC_27",28,"OID30406","Q9Y646","CPQ",0,"Cardiometabolic_II","B13805","20212097_SS211647","PASS",-5.656,0.6959,"Plate control","PASS","PFC",1.61989466083089,"24h","24h"
9544,"PFC_04",37,"OID30406","Q9Y646","CPQ",0,"Cardiometabolic_II","B13805","20212097_SS211647","PASS",-5.656,1.015,"Plate control","PASS","PFC",2.02090289297353,"Baseline","Baseline"
9545,"PFC_12",38,"OID30406","Q9Y646","CPQ",0,"Cardiometabolic_II","B13805","20212097_SS211647","PASS",-5.656,0.4025,"Plate control","PASS","PFC",1.32179642601778,"1h","1h"
9546,"PFC_20",39,"OID30406","Q9Y646","CPQ",0,"Cardiometabolic_II","B13805","20212097_SS211647","PASS",-5.656,0.6536,"Plate control","PASS","PFC",1.57308867744531,"24h","24h"
9547,"PFC_05",49,"OID30406","Q9Y646","CPQ",0,"Cardiometabolic_II","B13805","20212097_SS211647","PASS",-5.656,1.1499,"Plate control","PASS","PFC",2.21898513047629,"Baseline","Baseline"
9548,"PFC_13",50,"OID30406","Q9Y646","CPQ",0,"Cardiometabolic_II","B13805","20212097_SS211647","PASS",-5.656,1.1529,"Plate control","PASS","PFC",2.22360418119552,"1h","1h"
9549,"PFC_21",51,"OID30406","Q9Y646","CPQ",0,"Cardiometabolic_II","B13805","20212097_SS211647","PASS",-5.656,0.361,"Plate control","PASS","PFC",1.28431580898943,"24h","24h"
9550,"PFC_06",61,"OID30406","Q9Y646","CPQ",0,"Cardiometabolic_II","B13805","20212097_SS211647","PASS",-5.656,0.542,"Plate control","PASS","PFC",1.45598954899909,"Baseline","Baseline"
9551,"PFC_14",62,"OID30406","Q9Y646","CPQ",0,"Cardiometabolic_II","B13805","20212097_SS211647","PASS",-5.656,1.2185,"Plate control","PASS","PFC",2.32704643619543,"1h","1h"
9552,"PFC_22",63,"OID30406","Q9Y646","CPQ",0,"Cardiometabolic_II","B13805","20212097_SS211647","PASS",-5.656,0.9717,"Plate control","PASS","PFC",1.96115015586298,"24h","24h"
9553,"PFC_07",73,"OID30406","Q9Y646","CPQ",0,"Cardiometabolic_II","B13805","20212097_SS211647","PASS",-5.656,0.8329,"Plate control","PASS","PFC",1.78126232923806,"Baseline","Baseline"
9554,"PFC_15",74,"OID30406","Q9Y646","CPQ",0,"Cardiometabolic_II","B13805","20212097_SS211647","PASS",-5.656,0.5813,"Plate control","PASS","PFC",1.49619685140501,"1h","1h"
9555,"PFC_23",75,"OID30406","Q9Y646","CPQ",0,"Cardiometabolic_II","B13805","20212097_SS211647","PASS",-5.656,1.2464,"Plate control","PASS","PFC",2.37248670062783,"24h","24h"
9556,"PFC_08",85,"OID30406","Q9Y646","CPQ",0,"Cardiometabolic_II","B13805","20212097_SS211647","PASS",-5.656,0.3685,"Plate control","PASS","PFC",1.29100984284336,"Baseline","Baseline"
9557,"PFC_16",86,"OID30406","Q9Y646","CPQ",0,"Cardiometabolic_II","B13805","20212097_SS211647","PASS",-5.656,0.8294,"Plate control","PASS","PFC",1.77694619749071,"1h","1h"
9558,"PFC_24",87,"OID30406","Q9Y646","CPQ",0,"Cardiometabolic_II","B13805","20212097_SS211647","PASS",-5.656,0.6657,"Plate control","PASS","PFC",1.58633778241428,"24h","24h"
9559,"PFC_01",1,"OID30407","Q04721","NOTCH2",0,"Cardiometabolic_II","B13805","20212097_SS211647","PASS",-5.3498,0.2954,"Plate control","PASS","PFC",1.22722518916015,"Baseline","Baseline"
9560,"PFC_09",2,"OID30407","Q04721","NOTCH2",0,"Cardiometabolic_II","B13805","20212097_SS211647","PASS",-5.3498,0.3432,"Plate control","PASS","PFC",1.26856724789894,"Baseline","Baseline"
9561,"PFC_17",3,"OID30407","Q04721","NOTCH2",0,"Cardiometabolic_II","B13805","20212097_SS211647","PASS",-5.3498,-0.0513,"Plate control","PASS","PFC",0.965066324104723,"1h","1h"
9562,"PFC_25",4,"OID30407","Q04721","NOTCH2",0,"Cardiometabolic_II","B13805","20212097_SS211647","PASS",-5.3498,0.47,"Plate control","PASS","PFC",1.38510946811092,"24h","24h"
9563,"PFC_02",13,"OID30407","Q04721","NOTCH2",0,"Cardiometabolic_II","B13805","20212097_SS211647","PASS",-5.3498,0.1948,"Plate control","PASS","PFC",1.14456547913301,"Baseline","Baseline"
9564,"PFC_10",14,"OID30407","Q04721","NOTCH2",0,"Cardiometabolic_II","B13805","20212097_SS211647","PASS",-5.3498,0.3052,"Plate control","PASS","PFC",1.23558991438772,"1h","1h"
9565,"PFC_18",15,"OID30407","Q04721","NOTCH2",0,"Cardiometabolic_II","B13805","20212097_SS211647","PASS",-5.3498,0.3423,"Plate control","PASS","PFC",1.26777612125973,"1h","1h"
9566,"PFC_26",16,"OID30407","Q04721","NOTCH2",0,"Cardiometabolic_II","B13805","20212097_SS211647","PASS",-5.3498,0.1538,"Plate control","PASS","PFC",1.11249588521706,"24h","24h"
9567,"PFC_03",25,"OID30407","Q04721","NOTCH2",0,"Cardiometabolic_II","B13805","20212097_SS211647","PASS",-5.3498,0.2539,"Plate control","PASS","PFC",1.19242621677902,"Baseline","Baseline"
9568,"PFC_11",26,"OID30407","Q04721","NOTCH2",0,"Cardiometabolic_II","B13805","20212097_SS211647","PASS",-5.3498,0.1917,"Plate control","PASS","PFC",1.14210872732073,"1h","1h"
9569,"PFC_19",27,"OID30407","Q04721","NOTCH2",0,"Cardiometabolic_II","B13805","20212097_SS211647","PASS",-5.3498,0.3259,"Plate control","PASS","PFC",1.25344613516405,"24h","24h"
9570,"PFC_27",28,"OID30407","Q04721","NOTCH2",0,"Cardiometabolic_II","B13805","20212097_SS211647","PASS",-5.3498,0.53,"Plate control","PASS","PFC",1.4439291955225,"24h","24h"
9571,"PFC_04",37,"OID30407","Q04721","NOTCH2",0,"Cardiometabolic_II","B13805","20212097_SS211647","PASS",-5.3498,0.4795,"Plate control","PASS","PFC",1.39426036878436,"Baseline","Baseline"
9572,"PFC_12",38,"OID30407","Q04721","NOTCH2",0,"Cardiometabolic_II","B13805","20212097_SS211647","PASS",-5.3498,0.1852,"Plate control","PASS","PFC",1.13697458045622,"1h","1h"
9573,"PFC_20",39,"OID30407","Q04721","NOTCH2",0,"Cardiometabolic_II","B13805","20212097_SS211647","PASS",-5.3498,0.1474,"Plate control","PASS","PFC",1.10757162597725,"24h","24h"
9574,"PFC_05",49,"OID30407","Q04721","NOTCH2",0,"Cardiometabolic_II","B13805","20212097_SS211647","PASS",-5.3498,0.2429,"Plate control","PASS","PFC",1.18336899402156,"Baseline","Baseline"
9575,"PFC_13",50,"OID30407","Q04721","NOTCH2",0,"Cardiometabolic_II","B13805","20212097_SS211647","PASS",-5.3498,0.4151,"Plate control","PASS","PFC",1.3333910975161,"1h","1h"
9576,"PFC_21",51,"OID30407","Q04721","NOTCH2",0,"Cardiometabolic_II","B13805","20212097_SS211647","PASS",-5.3498,0.019,"Plate control","PASS","PFC",1.01325690015909,"24h","24h"
9577,"PFC_06",61,"OID30407","Q04721","NOTCH2",0,"Cardiometabolic_II","B13805","20212097_SS211647","PASS",-5.3498,0.3586,"Plate control","PASS","PFC",1.28218105740151,"Baseline","Baseline"
9578,"PFC_14",62,"OID30407","Q04721","NOTCH2",0,"Cardiometabolic_II","B13805","20212097_SS211647","PASS",-5.3498,0.1308,"Plate control","PASS","PFC",1.09490067480897,"1h","1h"
9579,"PFC_22",63,"OID30407","Q04721","NOTCH2",0,"Cardiometabolic_II","B13805","20212097_SS211647","PASS",-5.3498,0.1918,"Plate control","PASS","PFC",1.14218789500887,"24h","24h"
9580,"PFC_07",73,"OID30407","Q04721","NOTCH2",0,"Cardiometabolic_II","B13805","20212097_SS211647","PASS",-5.3498,0.6302,"Plate control","PASS","PFC",1.54777954647612,"Baseline","Baseline"
9581,"PFC_15",74,"OID30407","Q04721","NOTCH2",0,"Cardiometabolic_II","B13805","20212097_SS211647","PASS",-5.3498,0.334,"Plate control","PASS","PFC",1.26050339158299,"1h","1h"
9582,"PFC_23",75,"OID30407","Q04721","NOTCH2",0,"Cardiometabolic_II","B13805","20212097_SS211647","PASS",-5.3498,0.0557,"Plate control","PASS","PFC",1.03936328318349,"24h","24h"
9583,"PFC_08",85,"OID30407","Q04721","NOTCH2",0,"Cardiometabolic_II","B13805","20212097_SS211647","PASS",-5.3498,0.1025,"Plate control","PASS","PFC",1.07363231452805,"Baseline","Baseline"
9584,"PFC_16",86,"OID30407","Q04721","NOTCH2",0,"Cardiometabolic_II","B13805","20212097_SS211647","PASS",-5.3498,0.3775,"Plate control","PASS","PFC",1.29908875457377,"1h","1h"
9585,"PFC_24",87,"OID30407","Q04721","NOTCH2",0,"Cardiometabolic_II","B13805","20212097_SS211647","PASS",-5.3498,0.3082,"Plate control","PASS","PFC",1.23816192462985,"24h","24h"
9586,"PFC_01",1,"OID30408","O95965","ITGBL1",0,"Cardiometabolic_II","B13805","20212097_SS211647","PASS",-3.5483,0.1792,"Plate control","PASS","PFC",1.13225585522201,"Baseline","Baseline"
9587,"PFC_09",2,"OID30408","O95965","ITGBL1",0,"Cardiometabolic_II","B13805","20212097_SS211647","PASS",-3.5483,-0.1622,"Plate control","PASS","PFC",0.893661266011763,"Baseline","Baseline"
9588,"PFC_17",3,"OID30408","O95965","ITGBL1",0,"Cardiometabolic_II","B13805","20212097_SS211647","PASS",-3.5483,0.0607,"Plate control","PASS","PFC",1.04297169111272,"1h","1h"
9589,"PFC_25",4,"OID30408","O95965","ITGBL1",0,"Cardiometabolic_II","B13805","20212097_SS211647","PASS",-3.5483,0.5801,"Plate control","PASS","PFC",1.49495286728025,"24h","24h"
9590,"PFC_02",13,"OID30408","O95965","ITGBL1",0,"Cardiometabolic_II","B13805","20212097_SS211647","PASS",-3.5483,0.2831,"Plate control","PASS","PFC",1.21680669832427,"Baseline","Baseline"
9591,"PFC_10",14,"OID30408","O95965","ITGBL1",0,"Cardiometabolic_II","B13805","20212097_SS211647","PASS",-3.5483,0.3892,"Plate control","PASS","PFC",1.30966696889287,"1h","1h"
9592,"PFC_18",15,"OID30408","O95965","ITGBL1",0,"Cardiometabolic_II","B13805","20212097_SS211647","PASS",-3.5483,-0.1119,"Plate control","PASS","PFC",0.925368567476275,"1h","1h"
9593,"PFC_26",16,"OID30408","O95965","ITGBL1",0,"Cardiometabolic_II","B13805","20212097_SS211647","PASS",-3.5483,0.0302,"Plate control","PASS","PFC",1.02115367785386,"24h","24h"
9594,"PFC_03",25,"OID30408","O95965","ITGBL1",0,"Cardiometabolic_II","B13805","20212097_SS211647","PASS",-3.5483,0.2485,"Plate control","PASS","PFC",1.18797131421962,"Baseline","Baseline"
9595,"PFC_11",26,"OID30408","O95965","ITGBL1",0,"Cardiometabolic_II","B13805","20212097_SS211647","PASS",-3.5483,0.4201,"Plate control","PASS","PFC",1.33802029607148,"1h","1h"
9596,"PFC_19",27,"OID30408","O95965","ITGBL1",0,"Cardiometabolic_II","B13805","20212097_SS211647","PASS",-3.5483,0.637,"Plate control","PASS","PFC",1.55509207176512,"24h","24h"
9597,"PFC_27",28,"OID30408","O95965","ITGBL1",0,"Cardiometabolic_II","B13805","20212097_SS211647","PASS",-3.5483,-0.1247,"Plate control","PASS","PFC",0.917194748662159,"24h","24h"
9598,"PFC_04",37,"OID30408","O95965","ITGBL1",0,"Cardiometabolic_II","B13805","20212097_SS211647","PASS",-3.5483,0.5933,"Plate control","PASS","PFC",1.50869376811981,"Baseline","Baseline"
9599,"PFC_12",38,"OID30408","O95965","ITGBL1",0,"Cardiometabolic_II","B13805","20212097_SS211647","PASS",-3.5483,-0.2038,"Plate control","PASS","PFC",0.868260585733659,"1h","1h"
9600,"PFC_20",39,"OID30408","O95965","ITGBL1",0,"Cardiometabolic_II","B13805","20212097_SS211647","PASS",-3.5483,0.4569,"Plate control","PASS","PFC",1.37258928731805,"24h","24h"
9601,"PFC_05",49,"OID30408","O95965","ITGBL1",0,"Cardiometabolic_II","B13805","20212097_SS211647","PASS",-3.5483,0.3028,"Plate control","PASS","PFC",1.23353615353444,"Baseline","Baseline"
9602,"PFC_13",50,"OID30408","O95965","ITGBL1",0,"Cardiometabolic_II","B13805","20212097_SS211647","PASS",-3.5483,0.4109,"Plate control","PASS","PFC",1.32951495003388,"1h","1h"
9603,"PFC_21",51,"OID30408","O95965","ITGBL1",0,"Cardiometabolic_II","B13805","20212097_SS211647","PASS",-3.5483,0.0588,"Plate control","PASS","PFC",1.0415990227138,"24h","24h"
9604,"PFC_06",61,"OID30408","O95965","ITGBL1",0,"Cardiometabolic_II","B13805","20212097_SS211647","PASS",-3.5483,-0.3311,"Plate control","PASS","PFC",0.794930148680167,"Baseline","Baseline"
9605,"PFC_14",62,"OID30408","O95965","ITGBL1",0,"Cardiometabolic_II","B13805","20212097_SS211647","PASS",-3.5483,0.3603,"Plate control","PASS","PFC",1.2836928062257,"1h","1h"
9606,"PFC_22",63,"OID30408","O95965","ITGBL1",0,"Cardiometabolic_II","B13805","20212097_SS211647","PASS",-3.5483,0.4648,"Plate control","PASS","PFC",1.38012601404321,"24h","24h"
9607,"PFC_07",73,"OID30408","O95965","ITGBL1",0,"Cardiometabolic_II","B13805","20212097_SS211647","PASS",-3.5483,0.5784,"Plate control","PASS","PFC",1.49319232673008,"Baseline","Baseline"
9608,"PFC_15",74,"OID30408","O95965","ITGBL1",0,"Cardiometabolic_II","B13805","20212097_SS211647","PASS",-3.5483,-0.4269,"Plate control","PASS","PFC",0.743858439768375,"1h","1h"
9609,"PFC_23",75,"OID30408","O95965","ITGBL1",0,"Cardiometabolic_II","B13805","20212097_SS211647","PASS",-3.5483,0.2676,"Plate control","PASS","PFC",1.20380356973733,"24h","24h"
9610,"PFC_08",85,"OID30408","O95965","ITGBL1",0,"Cardiometabolic_II","B13805","20212097_SS211647","PASS",-3.5483,0.1905,"Plate control","PASS","PFC",1.14115914296343,"Baseline","Baseline"
9611,"PFC_16",86,"OID30408","O95965","ITGBL1",0,"Cardiometabolic_II","B13805","20212097_SS211647","PASS",-3.5483,0.5412,"Plate control","PASS","PFC",1.45518240076813,"1h","1h"
9612,"PFC_24",87,"OID30408","O95965","ITGBL1",0,"Cardiometabolic_II","B13805","20212097_SS211647","PASS",-3.5483,-0.155,"Plate control","PASS","PFC",0.898132372883934,"24h","24h"
9613,"PFC_01",1,"OID30409","Q9Y251","HPSE",0,"Cardiometabolic_II","B13805","20212097_SS211647","PASS",-4.6777,3.3747,"Plate control","PASS","PFC",10.3725592998636,"Baseline","Baseline"
9614,"PFC_09",2,"OID30409","Q9Y251","HPSE",0,"Cardiometabolic_II","B13805","20212097_SS211647","PASS",-4.6777,3.0016,"Plate control","PASS","PFC",8.00887720556929,"Baseline","Baseline"
9615,"PFC_17",3,"OID30409","Q9Y251","HPSE",0,"Cardiometabolic_II","B13805","20212097_SS211647","PASS",-4.6777,4.5451,"Plate control","PASS","PFC",23.3459436692641,"1h","1h"
9616,"PFC_25",4,"OID30409","Q9Y251","HPSE",0,"Cardiometabolic_II","B13805","20212097_SS211647","PASS",-4.6777,3.7634,"Plate control","PASS","PFC",13.5798910506602,"24h","24h"
9617,"PFC_02",13,"OID30409","Q9Y251","HPSE",0,"Cardiometabolic_II","B13805","20212097_SS211647","PASS",-4.6777,2.6281,"Plate control","PASS","PFC",6.18211289374539,"Baseline","Baseline"
9618,"PFC_10",14,"OID30409","Q9Y251","HPSE",0,"Cardiometabolic_II","B13805","20212097_SS211647","PASS",-4.6777,3.8326,"Plate control","PASS","PFC",14.2471357172685,"1h","1h"
9619,"PFC_18",15,"OID30409","Q9Y251","HPSE",0,"Cardiometabolic_II","B13805","20212097_SS211647","PASS",-4.6777,3.191,"Plate control","PASS","PFC",9.13243765701419,"1h","1h"
9620,"PFC_26",16,"OID30409","Q9Y251","HPSE",0,"Cardiometabolic_II","B13805","20212097_SS211647","PASS",-4.6777,4.4706,"Plate control","PASS","PFC",22.1709702199598,"24h","24h"
9621,"PFC_03",25,"OID30409","Q9Y251","HPSE",0,"Cardiometabolic_II","B13805","20212097_SS211647","PASS",-4.6777,1.7864,"Plate control","PASS","PFC",3.44953046042081,"Baseline","Baseline"
9622,"PFC_11",26,"OID30409","Q9Y251","HPSE",0,"Cardiometabolic_II","B13805","20212097_SS211647","PASS",-4.6777,3.7062,"Plate control","PASS","PFC",13.0520091606825,"1h","1h"
9623,"PFC_19",27,"OID30409","Q9Y251","HPSE",0,"Cardiometabolic_II","B13805","20212097_SS211647","PASS",-4.6777,3.8928,"Plate control","PASS","PFC",14.8542102633734,"24h","24h"
9624,"PFC_27",28,"OID30409","Q9Y251","HPSE",0,"Cardiometabolic_II","B13805","20212097_SS211647","PASS",-4.6777,4.4823,"Plate control","PASS","PFC",22.3515039008368,"24h","24h"
9625,"PFC_04",37,"OID30409","Q9Y251","HPSE",0,"Cardiometabolic_II","B13805","20212097_SS211647","PASS",-4.6777,3.1795,"Plate control","PASS","PFC",9.05993060551701,"Baseline","Baseline"
9626,"PFC_12",38,"OID30409","Q9Y251","HPSE",0,"Cardiometabolic_II","B13805","20212097_SS211647","PASS",-4.6777,2.2756,"Plate control","PASS","PFC",4.84198966508327,"1h","1h"
9627,"PFC_20",39,"OID30409","Q9Y251","HPSE",0,"Cardiometabolic_II","B13805","20212097_SS211647","PASS",-4.6777,4.1531,"Plate control","PASS","PFC",17.7912996764562,"24h","24h"
9628,"PFC_05",49,"OID30409","Q9Y251","HPSE",0,"Cardiometabolic_II","B13805","20212097_SS211647","PASS",-4.6777,2.1989,"Plate control","PASS","PFC",4.59129140032133,"Baseline","Baseline"
9629,"PFC_13",50,"OID30409","Q9Y251","HPSE",0,"Cardiometabolic_II","B13805","20212097_SS211647","PASS",-4.6777,3.5104,"Plate control","PASS","PFC",11.3955606471079,"1h","1h"
9630,"PFC_21",51,"OID30409","Q9Y251","HPSE",0,"Cardiometabolic_II","B13805","20212097_SS211647","PASS",-4.6777,2.5909,"Plate control","PASS","PFC",6.02474424861293,"24h","24h"
9631,"PFC_06",61,"OID30409","Q9Y251","HPSE",0,"Cardiometabolic_II","B13805","20212097_SS211647","PASS",-4.6777,3.8618,"Plate control","PASS","PFC",14.5384342685246,"Baseline","Baseline"
9632,"PFC_14",62,"OID30409","Q9Y251","HPSE",0,"Cardiometabolic_II","B13805","20212097_SS211647","PASS",-4.6777,3.3694,"Plate control","PASS","PFC",10.3345237436333,"1h","1h"

Supplemental table 1_Olink proximity extension assay (PEA) data

,"SampleID","Index","OlinkID","UniProt","Assay","MissingFreq","Panel","Panel_Lot_Nr","PlateID","QC_Warning","LOD","NPX","Normalization","Assay_Warning","condition","Value","group","alt_group"
9633,"PFC_22",63,"OID30409","Q9Y251","HPSE",0,"Cardiometabolic_II","B13805","20212097_SS211647","PASS",-4.6777,2.8585,"Plate control","PASS","PFC",7.25260863323469,"24h","24h"
9634,"PFC_07",73,"OID30409","Q9Y251","HPSE",0,"Cardiometabolic_II","B13805","20212097_SS211647","PASS",-4.6777,4.1074,"Plate control","PASS","PFC",17.2365603459239,"Baseline","Baseline"
9635,"PFC_15",74,"OID30409","Q9Y251","HPSE",0,"Cardiometabolic_II","B13805","20212097_SS211647","PASS",-4.6777,2.717,"Plate control","PASS","PFC",6.57504149835881,"1h","1h"
9636,"PFC_23",75,"OID30409","Q9Y251","HPSE",0,"Cardiometabolic_II","B13805","20212097_SS211647","PASS",-4.6777,3.7937,"Plate control","PASS","PFC",13.8681169225368,"24h","24h"
9637,"PFC_08",85,"OID30409","Q9Y251","HPSE",0,"Cardiometabolic_II","B13805","20212097_SS211647","PASS",-4.6777,3.3511,"Plate control","PASS","PFC",10.2042624144878,"Baseline","Baseline"
9638,"PFC_16",86,"OID30409","Q9Y251","HPSE",0,"Cardiometabolic_II","B13805","20212097_SS211647","PASS",-4.6777,3.4631,"Plate control","PASS","PFC",11.0280056004124,"1h","1h"
9639,"PFC_24",87,"OID30409","Q9Y251","HPSE",0,"Cardiometabolic_II","B13805","20212097_SS211647","PASS",-4.6777,3.9227,"Plate control","PASS","PFC",15.1652775833367,"24h","24h"
9640,"PFC_01",1,"OID30410","Q15063","POSTN",0,"Cardiometabolic_II","B13805","20212097_SS211647","PASS",-4.1821,0.271,"Plate control","PASS","PFC",1.20664391972076,"Baseline","Baseline"
9641,"PFC_09",2,"OID30410","Q15063","POSTN",0,"Cardiometabolic_II","B13805","20212097_SS211647","PASS",-4.1821,0.4564,"Plate control","PASS","PFC",1.37211366654433,"Baseline","Baseline"
9642,"PFC_17",3,"OID30410","Q15063","POSTN",0,"Cardiometabolic_II","B13805","20212097_SS211647","PASS",-4.1821,0.7171,"Plate control","PASS","PFC",1.64387431532546,"1h","1h"
9643,"PFC_25",4,"OID30410","Q15063","POSTN",0,"Cardiometabolic_II","B13805","20212097_SS211647","PASS",-4.1821,1.1688,"Plate control","PASS","PFC",2.24824615285629,"24h","24h"
9644,"PFC_02",13,"OID30410","Q15063","POSTN",0,"Cardiometabolic_II","B13805","20212097_SS211647","PASS",-4.1821,-0.3191,"Plate control","PASS","PFC",0.801569766891372,"Baseline","Baseline"
9645,"PFC_10",14,"OID30410","Q15063","POSTN",0,"Cardiometabolic_II","B13805","20212097_SS211647","PASS",-4.1821,0.583,"Plate control","PASS","PFC",1.49796093442418,"1h","1h"
9646,"PFC_18",15,"OID30410","Q15063","POSTN",0,"Cardiometabolic_II","B13805","20212097_SS211647","PASS",-4.1821,0.367,"Plate control","PASS","PFC",1.28966825065589,"1h","1h"
9647,"PFC_26",16,"OID30410","Q15063","POSTN",0,"Cardiometabolic_II","B13805","20212097_SS211647","PASS",-4.1821,0.6335,"Plate control","PASS","PFC",1.55132396745458,"24h","24h"
9648,"PFC_03",25,"OID30410","Q15063","POSTN",0,"Cardiometabolic_II","B13805","20212097_SS211647","PASS",-4.1821,0.4271,"Plate control","PASS","PFC",1.34452818651495,"Baseline","Baseline"
9649,"PFC_11",26,"OID30410","Q15063","POSTN",0,"Cardiometabolic_II","B13805","20212097_SS211647","PASS",-4.1821,0.0377,"Plate control","PASS","PFC",1.02647607382671,"1h","1h"
9650,"PFC_19",27,"OID30410","Q15063","POSTN",0,"Cardiometabolic_II","B13805","20212097_SS211647","PASS",-4.1821,0.4362,"Plate control","PASS","PFC",1.35303578863789,"24h","24h"
9651,"PFC_27",28,"OID30410","Q15063","POSTN",0,"Cardiometabolic_II","B13805","20212097_SS211647","PASS",-4.1821,-0.0973,"Plate control","PASS","PFC",0.934780795337914,"24h","24h"
9652,"PFC_04",37,"OID30410","Q15063","POSTN",0,"Cardiometabolic_II","B13805","20212097_SS211647","PASS",-4.1821,0.9914,"Plate control","PASS","PFC",1.98811333229693,"Baseline","Baseline"
9653,"PFC_12",38,"OID30410","Q15063","POSTN",0,"Cardiometabolic_II","B13805","20212097_SS211647","PASS",-4.1821,0.0686,"Plate control","PASS","PFC",1.04869852629249,"1h","1h"
9654,"PFC_20",39,"OID30410","Q15063","POSTN",0,"Cardiometabolic_II","B13805","20212097_SS211647","PASS",-4.1821,0.1426,"Plate control","PASS","PFC",1.10389274067335,"24h","24h"
9655,"PFC_05",49,"OID30410","Q15063","POSTN",0,"Cardiometabolic_II","B13805","20212097_SS211647","PASS",-4.1821,1.0245,"Plate control","PASS","PFC",2.03425424322419,"Baseline","Baseline"
9656,"PFC_13",50,"OID30410","Q15063","POSTN",0,"Cardiometabolic_II","B13805","20212097_SS211647","PASS",-4.1821,0.8276,"Plate control","PASS","PFC",1.77473054652889,"1h","1h"
9657,"PFC_21",51,"OID30410","Q15063","POSTN",0,"Cardiometabolic_II","B13805","20212097_SS211647","PASS",-4.1821,0.1847,"Plate control","PASS","PFC",1.13658060336879,"24h","24h"
9658,"PFC_06",61,"OID30410","Q15063","POSTN",0,"Cardiometabolic_II","B13805","20212097_SS211647","PASS",-4.1821,-0.4163,"Plate control","PASS","PFC",0.749343962978983,"Baseline","Baseline"
9659,"PFC_14",62,"OID30410","Q15063","POSTN",0,"Cardiometabolic_II","B13805","20212097_SS211647","PASS",-4.1821,0.9555,"Plate control","PASS","PFC",1.93925161081189,"1h","1h"
9660,"PFC_22",63,"OID30410","Q15063","POSTN",0,"Cardiometabolic_II","B13805","20212097_SS211647","PASS",-4.1821,0.8028,"Plate control","PASS","PFC",1.74448355800594,"24h","24h"
9661,"PFC_07",73,"OID30410","Q15063","POSTN",0,"Cardiometabolic_II","B13805","20212097_SS211647","PASS",-4.1821,1.4979,"Plate control","PASS","PFC",2.82431303552285,"Baseline","Baseline"
9662,"PFC_15",74,"OID30410","Q15063","POSTN",0,"Cardiometabolic_II","B13805","20212097_SS211647","PASS",-4.1821,-0.5292,"Plate control","PASS","PFC",0.692938874440984,"1h","1h"
9663,"PFC_23",75,"OID30410","Q15063","POSTN",0,"Cardiometabolic_II","B13805","20212097_SS211647","PASS",-4.1821,0.9027,"Plate control","PASS","PFC",1.86956159067583,"24h","24h"
9664,"PFC_08",85,"OID30410","Q15063","POSTN",0,"Cardiometabolic_II","B13805","20212097_SS211647","PASS",-4.1821,0.5353,"Plate control","PASS","PFC",1.44924348493451,"Baseline","Baseline"
9665,"PFC_16",86,"OID30410","Q15063","POSTN",0,"Cardiometabolic_II","B13805","20212097_SS211647","PASS",-4.1821,1.4325,"Plate control","PASS","PFC",2.69914035736118,"1h","1h"
9666,"PFC_24",87,"OID30410","Q15063","POSTN",0,"Cardiometabolic_II","B13805","20212097_SS211647","PASS",-4.1821,-0.2682,"Plate control","PASS","PFC",0.830354904479414,"24h","24h"
9667,"PFC_01",1,"OID30411","P08217","CELA2A",0,"Cardiometabolic_II","B13805","20212097_SS211647","PASS",-6.3485,0.729,"Plate control","PASS","PFC",1.65748980897598,"Baseline","Baseline"
9668,"PFC_09",2,"OID30411","P08217","CELA2A",0,"Cardiometabolic_II","B13805","20212097_SS211647","PASS",-6.3485,0.4236,"Plate control","PASS","PFC",1.34127029423498,"Baseline","Baseline"
9669,"PFC_17",3,"OID30411","P08217","CELA2A",0,"Cardiometabolic_II","B13805","20212097_SS211647","PASS",-6.3485,0.4785,"Plate control","PASS","PFC",1.3932942760017,"1h","1h"
9670,"PFC_25",4,"OID30411","P08217","CELA2A",0,"Cardiometabolic_II","B13805","20212097_SS211647","PASS",-6.3485,0.5377,"Plate control","PASS","PFC",1.45165638505721,"24h","24h"
9671,"PFC_02",13,"OID30411","P08217","CELA2A",0,"Cardiometabolic_II","B13805","20212097_SS211647","PASS",-6.3485,0.6904,"Plate control","PASS","PFC",1.61373087762744,"Baseline","Baseline"
9672,"PFC_10",14,"OID30411","P08217","CELA2A",0,"Cardiometabolic_II","B13805","20212097_SS211647","PASS",-6.3485,0.8626,"Plate control","PASS","PFC",1.8183122904909,"1h","1h"
9673,"PFC_18",15,"OID30411","P08217","CELA2A",0,"Cardiometabolic_II","B13805","20212097_SS211647","PASS",-6.3485,0.2058,"Plate control","PASS","PFC",1.15332570908443,"1h","1h"
9674,"PFC_26",16,"OID30411","P08217","CELA2A",0,"Cardiometabolic_II","B13805","20212097_SS211647","PASS",-6.3485,0.651,"Plate control","PASS","PFC",1.57025623734714,"24h","24h"
9675,"PFC_03",25,"OID30411","P08217","CELA2A",0,"Cardiometabolic_II","B13805","20212097_SS211647","PASS",-6.3485,-0.0955,"Plate control","PASS","PFC",0.935947816423077,"Baseline","Baseline"
9676,"PFC_11",26,"OID30411","P08217","CELA2A",0,"Cardiometabolic_II","B13805","20212097_SS211647","PASS",-6.3485,0.7039,"Plate control","PASS","PFC",1.62890221532949,"1h","1h"
9677,"PFC_19",27,"OID30411","P08217","CELA2A",0,"Cardiometabolic_II","B13805","20212097_SS211647","PASS",-6.3485,0.7322,"Plate control","PASS","PFC",1.66117031932489,"24h","24h"
9678,"PFC_27",28,"OID30411","P08217","CELA2A",0,"Cardiometabolic_II","B13805","20212097_SS211647","PASS",-6.3485,0.4597,"Plate control","PASS","PFC",1.37525581199753,"24h","24h"
9679,"PFC_04",37,"OID30411","P08217","CELA2A",0,"Cardiometabolic_II","B13805","20212097_SS211647","PASS",-6.3485,0.9812,"Plate control","PASS","PFC",1.97410674210773,"Baseline","Baseline"
9680,"PFC_12",38,"OID30411","P08217","CELA2A",0,"Cardiometabolic_II","B13805","20212097_SS211647","PASS",-6.3485,-0.135,"Plate control","PASS","PFC",0.910669833591978,"1h","1h"
9681,"PFC_20",39,"OID30411","P08217","CELA2A",0,"Cardiometabolic_II","B13805","20212097_SS211647","PASS",-6.3485,0.9126,"Plate control","PASS","PFC",1.88243493493491,"24h","24h"
9682,"PFC_05",49,"OID30411","P08217","CELA2A",0,"Cardiometabolic_II","B13805","20212097_SS211647","PASS",-6.3485,0.0204,"Plate control","PASS","PFC",1.01424064802827,"Baseline","Baseline"
9683,"PFC_13",50,"OID30411","P08217","CELA2A",0,"Cardiometabolic_II","B13805","20212097_SS211647","PASS",-6.3485,0.7536,"Plate control","PASS","PFC",1.6859946987022,"1h","1h"
9684,"PFC_21",51,"OID30411","P08217","CELA2A",0,"Cardiometabolic_II","B13805","20212097_SS211647","PASS",-6.3485,-0.1291,"Plate control","PASS","PFC",0.914401705806788,"24h","24h"
9685,"PFC_06",61,"OID30411","P08217","CELA2A",0,"Cardiometabolic_II","B13805","20212097_SS211647","PASS",-6.3485,0.8504,"Plate control","PASS","PFC",1.80300075388313,"Baseline","Baseline"
9686,"PFC_14",62,"OID30411","P08217","CELA2A",0,"Cardiometabolic_II","B13805","20212097_SS211647","PASS",-6.3485,0.0807,"Plate control","PASS","PFC",1.05753103335638,"1h","1h"
9687,"PFC_22",63,"OID30411","P08217","CELA2A",0,"Cardiometabolic_II","B13805","20212097_SS211647","PASS",-6.3485,0.5391,"Plate control","PASS","PFC",1.45306576492461,"24h","24h"
9688,"PFC_07",73,"OID30411","P08217","CELA2A",0,"Cardiometabolic_II","B13805","20212097_SS211647","PASS",-6.3485,0.2365,"Plate control","PASS","PFC",1.17813102794876,"Baseline","Baseline"
9689,"PFC_15",74,"OID30411","P08217","CELA2A",0,"Cardiometabolic_II","B13805","20212097_SS211647","PASS",-6.3485,0.9959,"Plate control","PASS","PFC",1.99432426188921,"1h","1h"
9690,"PFC_23",75,"OID30411","P08217","CELA2A",0,"Cardiometabolic_II","B13805","20212097_SS211647","PASS",-6.3485,0.2877,"Plate control","PASS","PFC",1.22069265037478,"24h","24h"
9691,"PFC_08",85,"OID30411","P08217","CELA2A",0,"Cardiometabolic_II","B13805","20212097_SS211647","PASS",-6.3485,0.2284,"Plate control","PASS","PFC",1.17153495465387,"Baseline","Baseline"
9692,"PFC_16",86,"OID30411","P08217","CELA2A",0,"Cardiometabolic_II","B13805","20212097_SS211647","PASS",-6.3485,0.4156,"Plate control","PASS","PFC",1.33385329574423,"1h","1h"
9693,"PFC_24",87,"OID30411","P08217","CELA2A",0,"Cardiometabolic_II","B13805","20212097_SS211647","PASS",-6.3485,1.1448,"Plate control","PASS","PFC",2.2111547542557,"24h","24h"
9694,"PFC_01",1,"OID30412","Q9UQP3","TNN",0,"Cardiometabolic_II","B13805","20212097_SS211647","PASS",-1.7094,0.8313,"Plate control","PASS","PFC",1.77928794113551,"Baseline","Baseline"
9695,"PFC_09",2,"OID30412","Q9UQP3","TNN",0,"Cardiometabolic_II","B13805","20212097_SS211647","PASS",-1.7094,0.8335,"Plate control","PASS","PFC",1.78200328948254,"Baseline","Baseline"
9696,"PFC_17",3,"OID30412","Q9UQP3","TNN",0,"Cardiometabolic_II","B13805","20212097_SS211647","PASS",-1.7094,-0.0523,"Plate control","PASS","PFC",0.964397622884072,"1h","1h"
9697,"PFC_25",4,"OID30412","Q9UQP3","TNN",0,"Cardiometabolic_II","B13805","20212097_SS211647","PASS",-1.7094,0.0034,"Plate control","PASS","PFC",1.00235947961514,"24h","24h"
9698,"PFC_02",13,"OID30412","Q9UQP3","TNN",0,"Cardiometabolic_II","B13805","20212097_SS211647","PASS",-1.7094,0.2921,"Plate control","PASS","PFC",1.2244212598733,"Baseline","Baseline"
9699,"PFC_10",14,"OID30412","Q9UQP3","TNN",0,"Cardiometabolic_II","B13805","20212097_SS211647","PASS",-1.7094,0.826,"Plate control","PASS","PFC",1.77276339838973,"1h","1h"
9700,"PFC_18",15,"OID30412","Q9UQP3","TNN",0,"Cardiometabolic_II","B13805","20212097_SS211647","PASS",-1.7094,0.3835,"Plate control","PASS","PFC",1.30450276312972,"1h","1h"
9701,"PFC_26",16,"OID30412","Q9UQP3","TNN",0,"Cardiometabolic_II","B13805","20212097_SS211647","PASS",-1.7094,0.0958,"Plate control","PASS","PFC",1.06865783350888,"24h","24h"
9702,"PFC_03",25,"OID30412","Q9UQP3","TNN",0,"Cardiometabolic_II","B13805","20212097_SS211647","PASS",-1.7094,0.3017,"Plate control","PASS","PFC",1.23259598768267,"Baseline","Baseline"
9703,"PFC_11",26,"OID30412","Q9UQP3","TNN",0,"Cardiometabolic_II","B13805","20212097_SS211647","PASS",-1.7094,0.3394,"Plate control","PASS","PFC",1.26523029005106,"1h","1h"
9704,"PFC_19",27,"OID30412","Q9UQP3","TNN",0,"Cardiometabolic_II","B13805","20212097_SS211647","PASS",-1.7094,0.7565,"Plate control","PASS","PFC",1.6893871703774,"24h","24h"
9705,"PFC_27",28,"OID30412","Q9UQP3","TNN",0,"Cardiometabolic_II","B13805","20212097_SS211647","PASS",-1.7094,0.5534,"Plate control","PASS","PFC",1.46754017630003,"24h","24h"
9706,"PFC_04",37,"OID30412","Q9UQP3","TNN",0,"Cardiometabolic_II","B13805","20212097_SS211647","PASS",-1.7094,1.1563,"Plate control","PASS","PFC",2.2288507299332,"Baseline","Baseline"
9707,"PFC_12",38,"OID30412","Q9UQP3","TNN",0,"Cardiometabolic_II","B13805","20212097_SS211647","PASS",-1.7094,0.2386,"Plate control","PASS","PFC",1.17984717488601,"1h","1h"
9708,"PFC_20",39,"OID30412","Q9UQP3","TNN",0,"Cardiometabolic_II","B13805","20212097_SS211647","PASS",-1.7094,0.4124,"Plate control","PASS","PFC",1.33089799320734,"24h","24h"
9709,"PFC_05",49,"OID30412","Q9UQP3","TNN",0,"Cardiometabolic_II","B13805","20212097_SS211647","PASS",-1.7094,0.6182,"Plate control","PASS","PFC",1.5349588717349,"Baseline","Baseline"
9710,"PFC_13",50,"OID30412","Q9UQP3","TNN",0,"Cardiometabolic_II","B13805","20212097_SS211647","PASS",-1.7094,1.0522,"Plate control","PASS","PFC",2.07368965654191,"1h","1h"
9711,"PFC_21",51,"OID30412","Q9UQP3","TNN",0,"Cardiometabolic_II","B13805","20212097_SS211647","PASS",-1.7094,0.0694,"Plate control","PASS","PFC",1.04928020949582,"24h","24h"
9712,"PFC_06",61,"OID30412","Q9UQP3","TNN",0,"Cardiometabolic_II","B13805","20212097_SS211647","PASS",-1.7094,0.0991,"Plate control","PASS","PFC",1.07110506396395,"Baseline","Baseline"
9713,"PFC_14",62,"OID30412","Q9UQP3","TNN",0,"Cardiometabolic_II","B13805","20212097_SS211647","PASS",-1.7094,0.5807,"Plate control","PASS","PFC",1.49557473000302,"1h","1h"
9714,"PFC_22",63,"OID30412","Q9UQP3","TNN",0,"Cardiometabolic_II","B13805","20212097_SS211647","PASS",-1.7094,0.8325,"Plate control","PASS","PFC",1.78076852691223,"24h","24h"
9715,"PFC_07",73,"OID30412","Q9UQP3","TNN",0,"Cardiometabolic_II","B13805","20212097_SS211647","PASS",-1.7094,0.274,"Plate control","PASS","PFC",1.20915567583336,"Baseline","Baseline"
9716,"PFC_15",74,"OID30412","Q9UQP3","TNN",0,"Cardiometabolic_II","B13805","20212097_SS211647","PASS",-1.7094,0.0115,"Plate control","PASS","PFC",1.00800304711529,"1h","1h"
9717,"PFC_23",75,"OID30412","Q9UQP3","TNN",0,"Cardiometabolic_II","B13805","20212097_SS211647","PASS",-1.7094,0.5905,"Plate control","PASS","PFC",1.50576851659106,"24h","24h"
9718,"PFC_08",85,"OID30412","Q9UQP3","TNN",0,"Cardiometabolic_II","B13805","20212097_SS211647","PASS",-1.7094,0.2835,"Plate control","PASS","PFC",1.21714411555096,"Baseline","Baseline"
9719,"PFC_16",86,"OID30412","Q9UQP3","TNN",0,"Cardiometabolic_II","B13805","20212097_SS211647","PASS",-1.7094,-0.0363,"Plate control","PASS","PFC",0.975152663147281,"1h","1h"
9720,"PFC_24",87,"OID30412","Q9UQP3","TNN",0,"Cardiometabolic_II","B13805","20212097_SS211647","PASS",-1.7094,0.075,"Plate control","PASS","PFC",1.05336103595484,"24h","24h"
9721,"PFC_01",1,"OID30413","P17900","GM2A",0,"Cardiometabolic_II","B13805","20212097_SS211647","PASS",-3.3388,0.6225,"Plate control","PASS","PFC",1.53954069185688,"Baseline","Baseline"
9722,"PFC_09",2,"OID30413","P17900","GM2A",0,"Cardiometabolic_II","B13805","20212097_SS211647","PASS",-3.3388,0.482,"Plate control","PASS","PFC",1.3966785324674,"Baseline","Baseline"
9723,"PFC_17",3,"OID30413","P17900","GM2A",0,"Cardiometabolic_II","B13805","20212097_SS211647","PASS",-3.3388,-0.1726,"Plate control","PASS","PFC",0.887242266843338,"1h","1h"
9724,"PFC_25",4,"OID30413","P17900","GM2A",0,"Cardiometabolic_II","B13805","20212097_SS211647","PASS",-3.3388,0.367,"Plate control","PASS","PFC",1.28966825065589,"24h","24h"
9725,"PFC_02",13,"OID30413","P17900","GM2A",0,"Cardiometabolic_II","B13805","20212097_SS211647","PASS",-3.3388,0.6731,"Plate control","PASS","PFC",1.59449547125479,"Baseline","Baseline"
9726,"PFC_10",14,"OID30413","P17900","GM2A",0,"Cardiometabolic_II","B13805","20212097_SS211647","PASS",-3.3388,0.7002,"Plate control","PASS","PFC",1.62473001250653,"1h","1h"
9727,"PFC_18",15,"OID30413","P17900","GM2A",0,"Cardiometabolic_II","B13805","20212097_SS211647","PASS",-3.3388,0.6687,"Plate control","PASS","PFC",1.58963991120017,"1h","1h"
9728,"PFC_26",16,"OID30413","P17900","GM2A",0,"Cardiometabolic_II","B13805","20212097_SS211647","PASS",-3.3388,0.0871,"Plate control","PASS","PFC",1.06223281231156,"24h","24h"
9729,"PFC_03",25,"OID30413","P17900","GM2A",0,"Cardiometabolic_II","B13805","20212097_SS211647","PASS",-3.3388,0.4419,"Plate control","PASS","PFC",1.35839212471852,"Baseline","Baseline"
9730,"PFC_11",26,"OID30413","P17900","GM2A",0,"Cardiometabolic_II","B13805","20212097_SS211647","PASS",-3.3388,0.9644,"Plate control","PASS","PFC",1.95125184995077,"1h","1h"
9731,"PFC_19",27,"OID30413","P17900","GM2A",0,"Cardiometabolic_II","B13805","20212097_SS211647","PASS",-3.3388,0.5984,"Plate control","PASS","PFC",1.51403651483769,"24h","24h"
9732,"PFC_27",28,"OID30413","P17900","GM2A",0,"Cardiometabolic_II","B13805","20212097_SS211647","PASS",-3.3388,0.8078,"Plate control","PASS","PFC",1.7505399661983,"24h","24h"
9733,"PFC_04",37,"OID30413","P17900","GM2A",0,"Cardiometabolic_II","B13805","20212097_SS211647","PASS",-3.3388,0.8221,"Plate control","PASS","PFC",1.76797760474949,"Baseline","Baseline"
9734,"PFC_12",38,"OID30413","P17900","GM2A",0,"Cardiometabolic_II","B13805","20212097_SS211647","PASS",-3.3388,0.3321,"Plate control","PASS","PFC",1.25884442692738,"1h","1h"
9735,"PFC_20",39,"OID30413","P17900","GM2A",0,"Cardiometabolic_II","B13805","20212097_SS211647","PASS",-3.3388,0.7165,"Plate control","PASS","PFC",1.64319078936242,"24h","24h"
9736,"PFC_05",49,"OID30413","P17900","GM2A",0,"Cardiometabolic_II","B13805","20212097_SS211647","PASS",-3.3388,0.5765,"Plate control","PASS","PFC",1.49122711718712,"Baseline","Baseline"
9737,"PFC_13",50,"OID30413","P17900","GM2A",0,"Cardiometabolic_II","B13805","20212097_SS211647","PASS",-3.3388,0.6784,"Plate control","PASS","PFC",1.60036391025212,"1h","1h"
9738,"PFC_21",51,"OID30413","P17900","GM2A",0,"Cardiometabolic_II","B13805","20212097_SS211647","PASS",-3.3388,0.3686,"Plate control","PASS","PFC",1.29109933192804,"24h","24h"
9739,"PFC_06",61,"OID30413","P17900","GM2A",0,"Cardiometabolic_II","B13805","20212097_SS211647","PASS",-3.3388,0.1625,"Plate control","PASS","PFC",1.1192249276281,"Baseline","Baseline"
9740,"PFC_14",62,"OID30413","P17900","GM2A",0,"Cardiometabolic_II","B13805","20212097_SS211647","PASS",-3.3388,0.6962,"Plate control","PASS","PFC",1.62023154348115,"1h","1h"
9741,"PFC_22",63,"OID30413","P17900","GM2A",0,"Cardiometabolic_II","B13805","20212097_SS211647","PASS",-3.3388,0.6254,"Plate control","PASS","PFC",1.54263847632439,"24h","24h"
9742,"PFC_07",73,"OID30413","P17900","GM2A",0,"Cardiometabolic_II","B13805","20212097_SS211647","PASS",-3.3388,0.4247,"Plate control","PASS","PFC",1.34229335170169,"Baseline","Baseline"
9743,"PFC_15",74,"OID30413","P17900","GM2A",0,"Cardiometabolic_II","B13805","20212097_SS211647","PASS",-3.3388,0.2633,"Plate control","PASS","PFC",1.20022093535299,"1h","1h"
9744,"PFC_23",75,"OID30413","P17900","GM2A",0,"Cardiometabolic_II","B13805","20212097_SS211647","PASS",-3.3388,0.6937,"Plate control","PASS","PFC",1.61742632740209,"24h","24h"

Supplemental table 1_Olink proximity extension assay (PEA) data

,"SampleID","Index","OlinkID","UniProt","Assay","MissingFreq","Panel","Panel_Lot_Nr","PlateID","QC_Warning","LOD","NPX","Normalization","Assay_Warning","condition","Value","group","alt_group"
9745,"PFC_08",85,"OID30413","P17900","GM2A",0,"Cardiometabolic_II","B13805","20212097_SS211647","PASS",-3.3388,-0.1429,"Plate control","PASS","PFC",0.905696758957641,"Baseline","Baseline"
9746,"PFC_16",86,"OID30413","P17900","GM2A",0,"Cardiometabolic_II","B13805","20212097_SS211647","PASS",-3.3388,0.517,"Plate control","PASS","PFC",1.43097652002291,"1h","1h"
9747,"PFC_24",87,"OID30413","P17900","GM2A",0,"Cardiometabolic_II","B13805","20212097_SS211647","PASS",-3.3388,0.4147,"Plate control","PASS","PFC",1.33302145424997,"24h","24h"
9748,"PFC_01",1,"OID30414","P37837","TALDO1",0,"Cardiometabolic_II","B13805","20212097_SS211647","PASS",-3.478,0.7099,"Plate control","PASS","PFC",1.63569073574393,"Baseline","Baseline"
9749,"PFC_09",2,"OID30414","P37837","TALDO1",0,"Cardiometabolic_II","B13805","20212097_SS211647","PASS",-3.478,1.2605,"Plate control","PASS","PFC",2.39578758207543,"Baseline","Baseline"
9750,"PFC_17",3,"OID30414","P37837","TALDO1",0,"Cardiometabolic_II","B13805","20212097_SS211647","PASS",-3.478,1.5417,"Plate control","PASS","PFC",2.91137363192143,"1h","1h"
9751,"PFC_25",4,"OID30414","P37837","TALDO1",0,"Cardiometabolic_II","B13805","20212097_SS211647","PASS",-3.478,1.3236,"Plate control","PASS","PFC",2.50289887017904,"24h","24h"
9752,"PFC_02",13,"OID30414","P37837","TALDO1",0,"Cardiometabolic_II","B13805","20212097_SS211647","PASS",-3.478,0.8889,"Plate control","PASS","PFC",1.85176368612831,"Baseline","Baseline"
9753,"PFC_10",14,"OID30414","P37837","TALDO1",0,"Cardiometabolic_II","B13805","20212097_SS211647","PASS",-3.478,0.9602,"Plate control","PASS","PFC",1.94557959073182,"1h","1h"
9754,"PFC_18",15,"OID30414","P37837","TALDO1",0,"Cardiometabolic_II","B13805","20212097_SS211647","PASS",-3.478,1.3705,"Plate control","PASS","PFC",2.58560160724247,"1h","1h"
9755,"PFC_26",16,"OID30414","P37837","TALDO1",0,"Cardiometabolic_II","B13805","20212097_SS211647","PASS",-3.478,1.7743,"Plate control","PASS","PFC",3.4207199563337,"24h","24h"
9756,"PFC_03",25,"OID30414","P37837","TALDO1",0,"Cardiometabolic_II","B13805","20212097_SS211647","PASS",-3.478,0.2034,"Plate control","PASS","PFC",1.1514086853496,"Baseline","Baseline"
9757,"PFC_11",26,"OID30414","P37837","TALDO1",0,"Cardiometabolic_II","B13805","20212097_SS211647","PASS",-3.478,1.5278,"Plate control","PASS","PFC",2.8834579830576,"1h","1h"
9758,"PFC_19",27,"OID30414","P37837","TALDO1",0,"Cardiometabolic_II","B13805","20212097_SS211647","PASS",-3.478,1.1543,"Plate control","PASS","PFC",2.22576302746127,"24h","24h"
9759,"PFC_27",28,"OID30414","P37837","TALDO1",0,"Cardiometabolic_II","B13805","20212097_SS211647","PASS",-3.478,2.3467,"Plate control","PASS","PFC",5.08659418946622,"24h","24h"
9760,"PFC_04",37,"OID30414","P37837","TALDO1",0,"Cardiometabolic_II","B13805","20212097_SS211647","PASS",-3.478,1.724,"Plate control","PASS","PFC",3.30351065974288,"Baseline","Baseline"
9761,"PFC_12",38,"OID30414","P37837","TALDO1",0,"Cardiometabolic_II","B13805","20212097_SS211647","PASS",-3.478,0.6146,"Plate control","PASS","PFC",1.53113341791326,"1h","1h"
9762,"PFC_20",39,"OID30414","P37837","TALDO1",0,"Cardiometabolic_II","B13805","20212097_SS211647","PASS",-3.478,1.7167,"Plate control","PASS","PFC",3.28683719953044,"24h","24h"
9763,"PFC_05",49,"OID30414","P37837","TALDO1",0,"Cardiometabolic_II","B13805","20212097_SS211647","PASS",-3.478,1.0974,"Plate control","PASS","PFC",2.13968734083558,"Baseline","Baseline"
9764,"PFC_13",50,"OID30414","P37837","TALDO1",0,"Cardiometabolic_II","B13805","20212097_SS211647","PASS",-3.478,1.9667,"Plate control","PASS","PFC",3.90873018353722,"1h","1h"
9765,"PFC_21",51,"OID30414","P37837","TALDO1",0,"Cardiometabolic_II","B13805","20212097_SS211647","PASS",-3.478,0.6986,"Plate control","PASS","PFC",1.62292912806975,"24h","24h"
9766,"PFC_06",61,"OID30414","P37837","TALDO1",0,"Cardiometabolic_II","B13805","20212097_SS211647","PASS",-3.478,1.2627,"Plate control","PASS","PFC",2.39944376256226,"Baseline","Baseline"
9767,"PFC_14",62,"OID30414","P37837","TALDO1",0,"Cardiometabolic_II","B13805","20212097_SS211647","PASS",-3.478,1.4824,"Plate control","PASS","PFC",2.79413165534041,"1h","1h"
9768,"PFC_22",63,"OID30414","P37837","TALDO1",0,"Cardiometabolic_II","B13805","20212097_SS211647","PASS",-3.478,1.2756,"Plate control","PASS","PFC",2.42099483254164,"24h","24h"
9769,"PFC_07",73,"OID30414","P37837","TALDO1",0,"Cardiometabolic_II","B13805","20212097_SS211647","PASS",-3.478,1.7085,"Plate control","PASS","PFC",3.26820844291904,"Baseline","Baseline"
9770,"PFC_15",74,"OID30414","P37837","TALDO1",0,"Cardiometabolic_II","B13805","20212097_SS211647","PASS",-3.478,0.5282,"Plate control","PASS","PFC",1.44212877910281,"1h","1h"
9771,"PFC_23",75,"OID30414","P37837","TALDO1",0,"Cardiometabolic_II","B13805","20212097_SS211647","PASS",-3.478,1.8964,"Plate control","PASS","PFC",3.72283067556826,"24h","24h"
9772,"PFC_08",85,"OID30414","P37837","TALDO1",0,"Cardiometabolic_II","B13805","20212097_SS211647","PASS",-3.478,1.0604,"Plate control","PASS","PFC",2.08550966758895,"Baseline","Baseline"
9773,"PFC_16",86,"OID30414","P37837","TALDO1",0,"Cardiometabolic_II","B13805","20212097_SS211647","PASS",-3.478,1.3983,"Plate control","PASS","PFC",2.63590796813578,"1h","1h"
9774,"PFC_24",87,"OID30414","P37837","TALDO1",0,"Cardiometabolic_II","B13805","20212097_SS211647","PASS",-3.478,1.5781,"Plate control","PASS","PFC",2.98576371679169,"24h","24h"
9775,"PFC_01",1,"OID30415","Q13510","ASAH1",0,"Cardiometabolic_II","B13805","20212097_SS211647","PASS",-1.5334,0.849,"Plate control","PASS","PFC",1.80125195969586,"Baseline","Baseline"
9776,"PFC_09",2,"OID30415","Q13510","ASAH1",0,"Cardiometabolic_II","B13805","20212097_SS211647","PASS",-1.5334,1.0865,"Plate control","PASS","PFC",2.12358226790998,"Baseline","Baseline"
9777,"PFC_17",3,"OID30415","Q13510","ASAH1",0,"Cardiometabolic_II","B13805","20212097_SS211647","PASS",-1.5334,1.4852,"Plate control","PASS","PFC",2.79955980568772,"1h","1h"
9778,"PFC_25",4,"OID30415","Q13510","ASAH1",0,"Cardiometabolic_II","B13805","20212097_SS211647","PASS",-1.5334,1.3784,"Plate control","PASS","PFC",2.59979884228864,"24h","24h"
9779,"PFC_02",13,"OID30415","Q13510","ASAH1",0,"Cardiometabolic_II","B13805","20212097_SS211647","PASS",-1.5334,0.6649,"Plate control","PASS","PFC",1.58545837381162,"Baseline","Baseline"
9780,"PFC_10",14,"OID30415","Q13510","ASAH1",0,"Cardiometabolic_II","B13805","20212097_SS211647","PASS",-1.5334,1.2166,"Plate control","PASS","PFC",2.32398378058071,"1h","1h"
9781,"PFC_18",15,"OID30415","Q13510","ASAH1",0,"Cardiometabolic_II","B13805","20212097_SS211647","PASS",-1.5334,1.0622,"Plate control","PASS","PFC",2.08811330875009,"1h","1h"
9782,"PFC_26",16,"OID30415","Q13510","ASAH1",0,"Cardiometabolic_II","B13805","20212097_SS211647","PASS",-1.5334,1.1503,"Plate control","PASS","PFC",2.2196004490883 8,"24h","24h"
9783,"PFC_03",25,"OID30415","Q13510","ASAH1",0,"Cardiometabolic_II","B13805","20212097_SS211647","PASS",-1.5334,-0.362,"Plate control","PASS","PFC",0.778085176555419,"Baseline","Baseline"
9784,"PFC_11",26,"OID30415","Q13510","ASAH1",0,"Cardiometabolic_II","B13805","20212097_SS211647","PASS",-1.5334,1.0123,"Plate control","PASS","PFC",2.01712431539082,"1h","1h"
9785,"PFC_19",27,"OID30415","Q13510","ASAH1",0,"Cardiometabolic_II","B13805","20212097_SS211647","PASS",-1.5334,0.9841,"Plate control","PASS","PFC",1.97807893799398,"24h","24h"
9786,"PFC_27",28,"OID30415","Q13510","ASAH1",0,"Cardiometabolic_II","B13805","20212097_SS211647","PASS",-1.5334,1.7347,"Plate control","PASS","PFC",3.32810280736298,"24h","24h"
9787,"PFC_04",37,"OID30415","Q13510","ASAH1",0,"Cardiometabolic_II","B13805","20212097_SS211647","PASS",-1.5334,1.3064,"Plate control","PASS","PFC",2.47323615345544,"Baseline","Baseline"
9788,"PFC_12",38,"OID30415","Q13510","ASAH1",0,"Cardiometabolic_II","B13805","20212097_SS211647","PASS",-1.5334,-0.2321,"Plate control","PASS","PFC",0.851394691520625,"1h","1h"
9789,"PFC_20",39,"OID30415","Q13510","ASAH1",0,"Cardiometabolic_II","B13805","20212097_SS211647","PASS",-1.5334,1.3934,"Plate control","PASS","PFC",2.62697050076517,"24h","24h"
9790,"PFC_05",49,"OID30415","Q13510","ASAH1",0,"Cardiometabolic_II","B13805","20212097_SS211647","PASS",-1.5334,1.0255,"Plate control","PASS","PFC",2.03566476961215,"Baseline","Baseline"
9791,"PFC_13",50,"OID30415","Q13510","ASAH1",0,"Cardiometabolic_II","B13805","20212097_SS211647","PASS",-1.5334,1.3301,"Plate control","PASS","PFC",2.51420101413845,"1h","1h"
9792,"PFC_21",51,"OID30415","Q13510","ASAH1",0,"Cardiometabolic_II","B13805","20212097_SS211647","PASS",-1.5334,0.1834,"Plate control","PASS","PFC",1.13555690172948,"24h","24h"
9793,"PFC_06",61,"OID30415","Q13510","ASAH1",0,"Cardiometabolic_II","B13805","20212097_SS211647","PASS",-1.5334,1.0299,"Plate control","PASS","PFC",2.04188271398451,"Baseline","Baseline"
9794,"PFC_14",62,"OID30415","Q13510","ASAH1",0,"Cardiometabolic_II","B13805","20212097_SS211647","PASS",-1.5334,1.4535,"Plate control","PASS","PFC",2.73871662857932,"1h","1h"
9795,"PFC_22",63,"OID30415","Q13510","ASAH1",0,"Cardiometabolic_II","B13805","20212097_SS211647","PASS",-1.5334,0.8968,"Plate control","PASS","PFC",1.86193150325384,"24h","24h"
9796,"PFC_07",73,"OID30415","Q13510","ASAH1",0,"Cardiometabolic_II","B13805","20212097_SS211647","PASS",-1.5334,1.4241,"Plate control","PASS","PFC",2.68347044731573,"Baseline","Baseline"
9797,"PFC_15",74,"OID30415","Q13510","ASAH1",0,"Cardiometabolic_II","B13805","20212097_SS211647","PASS",-1.5334,0.4259,"Plate control","PASS","PFC",1.34341030438751,"1h","1h"
9798,"PFC_23",75,"OID30415","Q13510","ASAH1",0,"Cardiometabolic_II","B13805","20212097_SS211647","PASS",-1.5334,1.3647,"Plate control","PASS","PFC",2.57522769979255,"24h","24h"
9799,"PFC_08",85,"OID30415","Q13510","ASAH1",0,"Cardiometabolic_II","B13805","20212097_SS211647","PASS",-1.5334,0.3625,"Plate control","PASS","PFC",1.28565183323808,"Baseline","Baseline"
9800,"PFC_16",86,"OID30415","Q13510","ASAH1",0,"Cardiometabolic_II","B13805","20212097_SS211647","PASS",-1.5334,1.08,"Plate control","PASS","PFC",2.11403608112276,"1h","1h"
9801,"PFC_24",87,"OID30415","Q13510","ASAH1",0,"Cardiometabolic_II","B13805","20212097_SS211647","PASS",-1.5334,1.3015,"Plate control","PASS","PFC",2.46485025087897,"24h","24h"
9802,"PFC_01",1,"OID30416","P11279","LAMP1",0,"Cardiometabolic_II","B13805","20212097_SS211647","PASS",-2.2441,0.4576,"Plate control","PASS","PFC",1.37325543338923,"Baseline","Baseline"
9803,"PFC_09",2,"OID30416","P11279","LAMP1",0,"Cardiometabolic_II","B13805","20212097_SS211647","PASS",-2.2441,0.6251,"Plate control","PASS","PFC",1.54231772702137,"Baseline","Baseline"
9804,"PFC_17",3,"OID30416","P11279","LAMP1",0,"Cardiometabolic_II","B13805","20212097_SS211647","PASS",-2.2441,0.4813,"Plate control","PASS","PFC",1.39600102419437,"1h","1h"
9805,"PFC_25",4,"OID30416","P11279","LAMP1",0,"Cardiometabolic_II","B13805","20212097_SS211647","PASS",-2.2441,0.4184,"Plate control","PASS","PFC",1.33644456814069,"24h","24h"
9806,"PFC_02",13,"OID30416","P11279","LAMP1",0,"Cardiometabolic_II","B13805","20212097_SS211647","PASS",-2.2441,0.5105,"Plate control","PASS","PFC",1.42454381931963,"Baseline","Baseline"
9807,"PFC_10",14,"OID30416","P11279","LAMP1",0,"Cardiometabolic_II","B13805","20212097_SS211647","PASS",-2.2441,0.4977,"Plate control","PASS","PFC",1.41196076486853,"1h","1h"
9808,"PFC_18",15,"OID30416","P11279","LAMP1",0,"Cardiometabolic_II","B13805","20212097_SS211647","PASS",-2.2441,0.5689,"Plate control","PASS","PFC",1.48339210937171,"1h","1h"
9809,"PFC_26",16,"OID30416","P11279","LAMP1",0,"Cardiometabolic_II","B13805","20212097_SS211647","PASS",-2.2441,0.4494,"Plate control","PASS","PFC",1.36547225470379,"24h","24h"
9810,"PFC_03",25,"OID30416","P11279","LAMP1",0,"Cardiometabolic_II","B13805","20212097_SS211647","PASS",-2.2441,0.2276,"Plate control","PASS","PFC",1.17088549781758,"Baseline","Baseline"
9811,"PFC_11",26,"OID30416","P11279","LAMP1",0,"Cardiometabolic_II","B13805","20212097_SS211647","PASS",-2.2441,0.4873,"Plate control","PASS","PFC",1.40181891882435,"1h","1h"
9812,"PFC_19",27,"OID30416","P11279","LAMP1",0,"Cardiometabolic_II","B13805","20212097_SS211647","PASS",-2.2441,0.6079,"Plate control","PASS","PFC",1.52403918833189,"24h","24h"
9813,"PFC_27",28,"OID30416","P11279","LAMP1",0,"Cardiometabolic_II","B13805","20212097_SS211647","PASS",-2.2441,0.856,"Plate control","PASS","PFC",1.81001292576581,"24h","24h"
9814,"PFC_04",37,"OID30416","P11279","LAMP1",0,"Cardiometabolic_II","B13805","20212097_SS211647","PASS",-2.2441,0.5106,"Plate control","PASS","PFC",1.42464256459504,"Baseline","Baseline"
9815,"PFC_12",38,"OID30416","P11279","LAMP1",0,"Cardiometabolic_II","B13805","20212097_SS211647","PASS",-2.2441,0.2433,"Plate control","PASS","PFC",1.18369713906273,"1h","1h"
9816,"PFC_20",39,"OID30416","P11279","LAMP1",0,"Cardiometabolic_II","B13805","20212097_SS211647","PASS",-2.2441,0.9091,"Plate control","PASS","PFC",1.87787365440276,"24h","24h"
9817,"PFC_05",49,"OID30416","P11279","LAMP1",0,"Cardiometabolic_II","B13805","20212097_SS211647","PASS",-2.2441,0.5495,"Plate control","PASS","PFC",1.4635783704274,"Baseline","Baseline"
9818,"PFC_13",50,"OID30416","P11279","LAMP1",0,"Cardiometabolic_II","B13805","20212097_SS211647","PASS",-2.2441,0.3939,"Plate control","PASS","PFC",1.3139405485742,"1h","1h"
9819,"PFC_21",51,"OID30416","P11279","LAMP1",0,"Cardiometabolic_II","B13805","20212097_SS211647","PASS",-2.2441,0.3942,"Plate control","PASS","PFC",1.31421380324007,"24h","24h"
9820,"PFC_06",61,"OID30416","P11279","LAMP1",0,"Cardiometabolic_II","B13805","20212097_SS211647","PASS",-2.2441,0.2173,"Plate control","PASS","PFC",1.1625558290735,"Baseline","Baseline"
9821,"PFC_14",62,"OID30416","P11279","LAMP1",0,"Cardiometabolic_II","B13805","20212097_SS211647","PASS",-2.2441,0.6039,"Plate control","PASS","PFC",1.51981950688981,"1h","1h"
9822,"PFC_22",63,"OID30416","P11279","LAMP1",0,"Cardiometabolic_II","B13805","20212097_SS211647","PASS",-2.2441,0.3547,"Plate control","PASS","PFC",1.27871965135278,"24h","24h"
9823,"PFC_07",73,"OID30416","P11279","LAMP1",0,"Cardiometabolic_II","B13805","20212097_SS211647","PASS",-2.2441,0.6026,"Plate control","PASS","PFC",1.51845062753708,"Baseline","Baseline"
9824,"PFC_15",74,"OID30416","P11279","LAMP1",0,"Cardiometabolic_II","B13805","20212097_SS211647","PASS",-2.2441,0.2959,"Plate control","PASS","PFC",1.22765058671156,"1h","1h"
9825,"PFC_23",75,"OID30416","P11279","LAMP1",0,"Cardiometabolic_II","B13805","20212097_SS211647","PASS",-2.2441,0.6558,"Plate control","PASS","PFC",1.57548934775914,"24h","24h"
9826,"PFC_08",85,"OID30416","P11279","LAMP1",0,"Cardiometabolic_II","B13805","20212097_SS211647","PASS",-2.2441,0.3312,"Plate control","PASS","PFC",1.25805936380801,"Baseline","Baseline"
9827,"PFC_16",86,"OID30416","P11279","LAMP1",0,"Cardiometabolic_II","B13805","20212097_SS211647","PASS",-2.2441,0.4537,"Plate control","PASS","PFC",1.36954816081946,"1h","1h"
9828,"PFC_24",87,"OID30416","P11279","LAMP1",0,"Cardiometabolic_II","B13805","20212097_SS211647","PASS",-2.2441,0.216,"Plate control","PASS","PFC",1.16150873192572,"24h","24h"
9829,"PFC_01",1,"OID30417","P17174","GOT1",0,"Cardiometabolic_II","B13805","20212097_SS211647","PASS",-2.3916,0.8631,"Plate control","PASS","PFC",1.81894257872399,"Baseline","Baseline"
9830,"PFC_09",2,"OID30417","P17174","GOT1",0,"Cardiometabolic_II","B13805","20212097_SS211647","PASS",-2.3916,0.5747,"Plate control","PASS","PFC",1.48936772560782,"Baseline","Baseline"
9831,"PFC_17",3,"OID30417","P17174","GOT1",0,"Cardiometabolic_II","B13805","20212097_SS211647","PASS",-2.3916,0.6442,"Plate control","PASS","PFC",1.56287240544788,"1h","1h"
9832,"PFC_25",4,"OID30417","P17174","GOT1",0,"Cardiometabolic_II","B13805","20212097_SS211647","PASS",-2.3916,0.228,"Plate control","PASS","PFC",1.17121018121881,"24h","24h"
9833,"PFC_02",13,"OID30417","P17174","GOT1",0,"Cardiometabolic_II","B13805","20212097_SS211647","PASS",-2.3916,0.7286,"Plate control","PASS","PFC",1.65703031892262,"Baseline","Baseline"
9834,"PFC_10",14,"OID30417","P17174","GOT1",0,"Cardiometabolic_II","B13805","20212097_SS211647","PASS",-2.3916,0.6254,"Plate control","PASS","PFC",1.54263847632439,"1h","1h"
9835,"PFC_18",15,"OID30417","P17174","GOT1",0,"Cardiometabolic_II","B13805","20212097_SS211647","PASS",-2.3916,0.8351,"Plate control","PASS","PFC",1.78398069067328,"1h","1h"
9836,"PFC_26",16,"OID30417","P17174","GOT1",0,"Cardiometabolic_II","B13805","20212097_SS211647","PASS",-2.3916,0.5146,"Plate control","PASS","PFC",1.42859799335758,"24h","24h"
9837,"PFC_03",25,"OID30417","P17174","GOT1",0,"Cardiometabolic_II","B13805","20212097_SS211647","PASS",-2.3916,0.2177,"Plate control","PASS","PFC",1.16287820267998,"Baseline","Baseline"
9838,"PFC_11",26,"OID30417","P17174","GOT1",0,"Cardiometabolic_II","B13805","20212097_SS211647","PASS",-2.3916,1.0032,"Plate control","PASS","PFC",2.00444106543398,"1h","1h"
9839,"PFC_19",27,"OID30417","P17174","GOT1",0,"Cardiometabolic_II","B13805","20212097_SS211647","PASS",-2.3916,0.8615,"Plate control","PASS","PFC",1.81692642505156,"24h","24h"
9840,"PFC_27",28,"OID30417","P17174","GOT1",0,"Cardiometabolic_II","B13805","20212097_SS211647","PASS",-2.3916,1.1253,"Plate control","PASS","PFC",2.18146904190434,"24h","24h"
9841,"PFC_04",37,"OID30417","P17174","GOT1",0,"Cardiometabolic_II","B13805","20212097_SS211647","PASS",-2.3916,1.1311,"Plate control","PASS","PFC",2.19025675335502,"Baseline","Baseline"
9842,"PFC_12",38,"OID30417","P17174","GOT1",0,"Cardiometabolic_II","B13805","20212097_SS211647","PASS",-2.3916,0.5435,"Plate control","PASS","PFC",1.45750415882451,"1h","1h"
9843,"PFC_20",39,"OID30417","P17174","GOT1",0,"Cardiometabolic_II","B13805","20212097_SS211647","PASS",-2.3916,1.2054,"Plate control","PASS","PFC",2.30601196724886,"24h","24h"
9844,"PFC_05",49,"OID30417","P17174","GOT1",0,"Cardiometabolic_II","B13805","20212097_SS211647","PASS",-2.3916,1.0038,"Plate control","PASS","PFC",2.00527486240903,"Baseline","Baseline"
9845,"PFC_13",50,"OID30417","P17174","GOT1",0,"Cardiometabolic_II","B13805","20212097_SS211647","PASS",-2.3916,1.1557,"Plate control","PASS","PFC",2.22792396970132,"1h","1h"
9846,"PFC_21",51,"OID30417","P17174","GOT1",0,"Cardiometabolic_II","B13805","20212097_SS211647","PASS",-2.3916,0.3917,"Plate control","PASS","PFC",1.31193841630108,"24h","24h"
9847,"PFC_06",61,"OID30417","P17174","GOT1",0,"Cardiometabolic_II","B13805","20212097_SS211647","PASS",-2.3916,0.6165,"Plate control","PASS","PFC",1.53315121786455,"Baseline","Baseline"
9848,"PFC_14",62,"OID30417","P17174","GOT1",0,"Cardiometabolic_II","B13805","20212097_SS211647","PASS",-2.3916,1.0426,"Plate control","PASS","PFC",2.05993669233244,"1h","1h"
9849,"PFC_22",63,"OID30417","P17174","GOT1",0,"Cardiometabolic_II","B13805","20212097_SS211647","PASS",-2.3916,0.925,"Plate control","PASS","PFC",1.89868424190104,"24h","24h"
9850,"PFC_07",73,"OID30417","P17174","GOT1",0,"Cardiometabolic_II","B13805","20212097_SS211647","PASS",-2.3916,0.8718,"Plate control","PASS","PFC",1.8299446344782,"Baseline","Baseline"
9851,"PFC_15",74,"OID30417","P17174","GOT1",0,"Cardiometabolic_II","B13805","20212097_SS211647","PASS",-2.3916,0.3124,"Plate control","PASS","PFC",1.24177173603258,"1h","1h"
9852,"PFC_23",75,"OID30417","P17174","GOT1",0,"Cardiometabolic_II","B13805","20212097_SS211647","PASS",-2.3916,1.4439,"Plate control","PASS","PFC",2.72055312390353,"24h","24h"
9853,"PFC_08",85,"OID30417","P17174","GOT1",0,"Cardiometabolic_II","B13805","20212097_SS211647","PASS",-2.3916,0.6698,"Plate control","PASS","PFC",1.59085241324955,"Baseline","Baseline"
9854,"PFC_16",86,"OID30417","P17174","GOT1",0,"Cardiometabolic_II","B13805","20212097_SS211647","PASS",-2.3916,0.5696,"Plate control","PASS","PFC",1.4841120303523,"1h","1h"
9855,"PFC_24",87,"OID30417","P17174","GOT1",0,"Cardiometabolic_II","B13805","20212097_SS211647","PASS",-2.3916,0.5583,"Plate control","PASS","PFC",1.47253303497009,"24h","24h"
9856,"PFC_01",1,"OID30418","P61916","NPC2",0,"Cardiometabolic_II","B13805","20212097_SS211647","PASS",-4.1621,0.485,"Plate control","PASS","PFC",1.3995858655952,"Baseline","Baseline"

Supplemental table 1_Olink proximity extension assay (PEA) data

,"SampleID","Index","OlinkID","UniProt","Assay","MissingFreq","Panel","Panel_Lot_Nr","PlateID","QC_Warning","LOD","NPX","Normalization","Assay_Warning","condition","Value","group","alt_group"
9857,"PFC_09",2,"OID30418","P61916","NPC2",0,"Cardiometabolic_II","B13805","20212097_SS211647","PASS",-4.1621,0.2912,"Plate control","PASS","PFC",1.22365766434621,"Baseline","Baseline"
9858,"PFC_17",3,"OID30418","P61916","NPC2",0,"Cardiometabolic_II","B13805","20212097_SS211647","PASS",-4.1621,-0.1774,"Plate control","PASS","PFC",0.884295222643272,"1h","1h"
9859,"PFC_25",4,"OID30418","P61916","NPC2",0,"Cardiometabolic_II","B13805","20212097_SS211647","PASS",-4.1621,0.1291,"Plate control","PASS","PFC",1.09361125821357,"24h","24h"
9860,"PFC_02",13,"OID30418","P61916","NPC2",0,"Cardiometabolic_II","B13805","20212097_SS211647","PASS",-4.1621,0.6866,"Plate control","PASS","PFC",1.60948596910928,"Baseline","Baseline"
9861,"PFC_10",14,"OID30418","P61916","NPC2",0,"Cardiometabolic_II","B13805","20212097_SS211647","PASS",-4.1621,0.549,"Plate control","PASS","PFC",1.46307122070433,"1h","1h"
9862,"PFC_18",15,"OID30418","P61916","NPC2",0,"Cardiometabolic_II","B13805","20212097_SS211647","PASS",-4.1621,0.2041,"Plate control","PASS","PFC",1.15196748788372,"1h","1h"
9863,"PFC_26",16,"OID30418","P61916","NPC2",0,"Cardiometabolic_II","B13805","20212097_SS211647","PASS",-4.1621,0.1206,"Plate control","PASS","PFC",1.08718691684518,"24h","24h"
9864,"PFC_03",25,"OID30418","P61916","NPC2",0,"Cardiometabolic_II","B13805","20212097_SS211647","PASS",-4.1621,0.1364,"Plate control","PASS","PFC",1.09915892689771,"Baseline","Baseline"
9865,"PFC_11",26,"OID30418","P61916","NPC2",0,"Cardiometabolic_II","B13805","20212097_SS211647","PASS",-4.1621,0.7705,"Plate control","PASS","PFC",1.70586088743177,"1h","1h"
9866,"PFC_19",27,"OID30418","P61916","NPC2",0,"Cardiometabolic_II","B13805","20212097_SS211647","PASS",-4.1621,0.5226,"Plate control","PASS","PFC",1.43654182735674,"24h","24h"
9867,"PFC_27",28,"OID30418","P61916","NPC2",0,"Cardiometabolic_II","B13805","20212097_SS211647","PASS",-4.1621,0.4288,"Plate control","PASS","PFC",1.34611344539342,"24h","24h"
9868,"PFC_04",37,"OID30418","P61916","NPC2",0,"Cardiometabolic_II","B13805","20212097_SS211647","PASS",-4.1621,0.8001,"Plate control","PASS","PFC",1.74122181470863,"Baseline","Baseline"
9869,"PFC_12",38,"OID30418","P61916","NPC2",0,"Cardiometabolic_II","B13805","20212097_SS211647","PASS",-4.1621,0.1446,"Plate control","PASS","PFC",1.10542412218255,"1h","1h"
9870,"PFC_20",39,"OID30418","P61916","NPC2",0,"Cardiometabolic_II","B13805","20212097_SS211647","PASS",-4.1621,0.4934,"Plate control","PASS","PFC",1.40775863479289,"24h","24h"
9871,"PFC_05",49,"OID30418","P61916","NPC2",0,"Cardiometabolic_II","B13805","20212097_SS211647","PASS",-4.1621,0.4339,"Plate control","PASS","PFC",1.35088044539319,"Baseline","Baseline"
9872,"PFC_13",50,"OID30418","P61916","NPC2",0,"Cardiometabolic_II","B13805","20212097_SS211647","PASS",-4.1621,0.4435,"Plate control","PASS","PFC",1.35989946548537,"1h","1h"
9873,"PFC_21",51,"OID30418","P61916","NPC2",0,"Cardiometabolic_II","B13805","20212097_SS211647","PASS",-4.1621,0.2196,"Plate control","PASS","PFC",1.16441069851167,"24h","24h"
9874,"PFC_06",61,"OID30418","P61916","NPC2",0,"Cardiometabolic_II","B13805","20212097_SS211647","PASS",-4.1621,0.175,"Plate control","PASS","PFC",1.12896440480613,"Baseline","Baseline"
9875,"PFC_14",62,"OID30418","P61916","NPC2",0,"Cardiometabolic_II","B13805","20212097_SS211647","PASS",-4.1621,0.5064,"Plate control","PASS","PFC",1.42050115049673,"1h","1h"
9876,"PFC_22",63,"OID30418","P61916","NPC2",0,"Cardiometabolic_II","B13805","20212097_SS211647","PASS",-4.1621,0.4585,"Plate control","PASS","PFC",1.37411238197627,"24h","24h"
9877,"PFC_07",73,"OID30418","P61916","NPC2",0,"Cardiometabolic_II","B13805","20212097_SS211647","PASS",-4.1621,0.2055,"Plate control","PASS","PFC",1.15308590667899,"Baseline","Baseline"
9878,"PFC_15",74,"OID30418","P61916","NPC2",0,"Cardiometabolic_II","B13805","20212097_SS211647","PASS",-4.1621,0.0129,"Plate control","PASS","PFC",1.00898169413913,"1h","1h"
9879,"PFC_23",75,"OID30418","P61916","NPC2",0,"Cardiometabolic_II","B13805","20212097_SS211647","PASS",-4.1621,0.6071,"Plate control","PASS","PFC",1.52319431582878,"24h","24h"
9880,"PFC_08",85,"OID30418","P61916","NPC2",0,"Cardiometabolic_II","B13805","20212097_SS211647","PASS",-4.1621,-0.1469,"Plate control","PASS","PFC",0.903189112280842,"Baseline","Baseline"
9881,"PFC_16",86,"OID30418","P61916","NPC2",0,"Cardiometabolic_II","B13805","20212097_SS211647","PASS",-4.1621,-0.0436,"Plate control","PASS","PFC",0.970230878172232,"1h","1h"
9882,"PFC_24",87,"OID30418","P61916","NPC2",0,"Cardiometabolic_II","B13805","20212097_SS211647","PASS",-4.1621,0.2533,"Plate control","PASS","PFC",1.19193040376548,"24h","24h"
9883,"PFC_01",1,"OID30419","P07602","PSAP",0,"Cardiometabolic_II","B13805","20212097_SS211647","PASS",-3.517,0.3596,"Plate control","PASS","PFC",1.28307010567147,"Baseline","Baseline"
9884,"PFC_09",2,"OID30419","P07602","PSAP",0,"Cardiometabolic_II","B13805","20212097_SS211647","PASS",-3.517,0.1354,"Plate control","PASS","PFC",1.09839731197266,"Baseline","Baseline"
9885,"PFC_17",3,"OID30419","P07602","PSAP",0,"Cardiometabolic_II","B13805","20212097_SS211647","PASS",-3.517,0.1072,"Plate control","PASS","PFC",1.07713568855639,"1h","1h"
9886,"PFC_25",4,"OID30419","P07602","PSAP",0,"Cardiometabolic_II","B13805","20212097_SS211647","PASS",-3.517,-0.0426,"Plate control","PASS","PFC",0.970903624098965,"24h","24h"
9887,"PFC_02",13,"OID30419","P07602","PSAP",0,"Cardiometabolic_II","B13805","20212097_SS211647","PASS",-3.517,0.4915,"Plate control","PASS","PFC",1.40590586562595,"Baseline","Baseline"
9888,"PFC_10",14,"OID30419","P07602","PSAP",0,"Cardiometabolic_II","B13805","20212097_SS211647","PASS",-3.517,0.5257,"Plate control","PASS","PFC",1.43963192434431,"1h","1h"
9889,"PFC_18",15,"OID30419","P07602","PSAP",0,"Cardiometabolic_II","B13805","20212097_SS211647","PASS",-3.517,0.2114,"Plate control","PASS","PFC",1.15781118591598,"1h","1h"
9890,"PFC_26",16,"OID30419","P07602","PSAP",0,"Cardiometabolic_II","B13805","20212097_SS211647","PASS",-3.517,0.1608,"Plate control","PASS","PFC",1.11790686542495,"24h","24h"
9891,"PFC_03",25,"OID30419","P07602","PSAP",0,"Cardiometabolic_II","B13805","20212097_SS211647","PASS",-3.517,0.0999,"Plate control","PASS","PFC",1.07169917543554,"Baseline","Baseline"
9892,"PFC_11",26,"OID30419","P07602","PSAP",0,"Cardiometabolic_II","B13805","20212097_SS211647","PASS",-3.517,0.8493,"Plate control","PASS","PFC",1.80162655845752,"1h","1h"
9893,"PFC_19",27,"OID30419","P07602","PSAP",0,"Cardiometabolic_II","B13805","20212097_SS211647","PASS",-3.517,0.4376,"Plate control","PASS","PFC",1.35434942003166,"24h","24h"
9894,"PFC_27",28,"OID30419","P07602","PSAP",0,"Cardiometabolic_II","B13805","20212097_SS211647","PASS",-3.517,0.4338,"Plate control","PASS","PFC",1.35078681274109,"24h","24h"
9895,"PFC_04",37,"OID30419","P07602","PSAP",0,"Cardiometabolic_II","B13805","20212097_SS211647","PASS",-3.517,0.8497,"Plate control","PASS","PFC",1.80212614465944,"Baseline","Baseline"
9896,"PFC_12",38,"OID30419","P07602","PSAP",0,"Cardiometabolic_II","B13805","20212097_SS211647","PASS",-3.517,0.217,"Plate control","PASS","PFC",1.16231410751812,"1h","1h"
9897,"PFC_20",39,"OID30419","P07602","PSAP",0,"Cardiometabolic_II","B13805","20212097_SS211647","PASS",-3.517,0.6625,"Plate control","PASS","PFC",1.58282307199771,"24h","24h"
9898,"PFC_05",49,"OID30419","P07602","PSAP",0,"Cardiometabolic_II","B13805","20212097_SS211647","PASS",-3.517,0.504,"Plate control","PASS","PFC",1.41814003567946,"Baseline","Baseline"
9899,"PFC_13",50,"OID30419","P07602","PSAP",0,"Cardiometabolic_II","B13805","20212097_SS211647","PASS",-3.517,0.7742,"Plate control","PASS","PFC",1.71024142915583,"1h","1h"
9900,"PFC_21",51,"OID30419","P07602","PSAP",0,"Cardiometabolic_II","B13805","20212097_SS211647","PASS",-3.517,0.0684,"Plate control","PASS","PFC",1.04855315588368,"24h","24h"
9901,"PFC_06",61,"OID30419","P07602","PSAP",0,"Cardiometabolic_II","B13805","20212097_SS211647","PASS",-3.517,0.4933,"Plate control","PASS","PFC",1.40766105978176,"Baseline","Baseline"
9902,"PFC_14",62,"OID30419","P07602","PSAP",0,"Cardiometabolic_II","B13805","20212097_SS211647","PASS",-3.517,0.8768,"Plate control","PASS","PFC",1.83629774203542,"1h","1h"
9903,"PFC_22",63,"OID30419","P07602","PSAP",0,"Cardiometabolic_II","B13805","20212097_SS211647","PASS",-3.517,0.5239,"Plate control","PASS","PFC",1.43783686613583,"24h","24h"
9904,"PFC_07",73,"OID30419","P07602","PSAP",0,"Cardiometabolic_II","B13805","20212097_SS211647","PASS",-3.517,0.2519,"Plate control","PASS","PFC",1.1907743083189,"Baseline","Baseline"
9905,"PFC_15",74,"OID30419","P07602","PSAP",0,"Cardiometabolic_II","B13805","20212097_SS211647","PASS",-3.517,0.4339,"Plate control","PASS","PFC",1.35088044539319,"1h","1h"
9906,"PFC_23",75,"OID30419","P07602","PSAP",0,"Cardiometabolic_II","B13805","20212097_SS211647","PASS",-3.517,0.817,"Plate control","PASS","PFC",1.76173874825396,"24h","24h"
9907,"PFC_08",85,"OID30419","P07602","PSAP",0,"Cardiometabolic_II","B13805","20212097_SS211647","PASS",-3.517,-0.0031,"Plate control","PASS","PFC",0.997853550664361,"Baseline","Baseline"
9908,"PFC_16",86,"OID30419","P07602","PSAP",0,"Cardiometabolic_II","B13805","20212097_SS211647","PASS",-3.517,0.3753,"Plate control","PASS","PFC",1.29710925289541,"1h","1h"
9909,"PFC_24",87,"OID30419","P07602","PSAP",0,"Cardiometabolic_II","B13805","20212097_SS211647","PASS",-3.517,0.3108,"Plate control","PASS","PFC",1.24039533049058,"24h","24h"
9910,"PFC_01",1,"OID20466","Q13574","DGKZ",0.9091,"Inflammation","B04411","20212097_SS211642","PASS",0.7369,-0.0575,"Plate control","PASS","PFC",0.960927838461034,"Baseline","Baseline"
9911,"PFC_09",2,"OID20466","Q13574","DGKZ",0.9091,"Inflammation","B04411","20212097_SS211642","PASS",0.7369,-0.0822,"Plate control","PASS","PFC",0.944616080325356,"Baseline","Baseline"
9912,"PFC_17",3,"OID20466","Q13574","DGKZ",0.9091,"Inflammation","B04411","20212097_SS211642","PASS",0.7369,0.2853,"Plate control","PASS","PFC",1.21866365131123,"1h","1h"
9913,"PFC_25",4,"OID20466","Q13574","DGKZ",0.9091,"Inflammation","B04411","20212097_SS211642","PASS",0.7369,0.6828,"Plate control","PASS","PFC",1.60525222679521,"24h","24h"
9914,"PFC_02",13,"OID20466","Q13574","DGKZ",0.9091,"Inflammation","B04411","20212097_SS211642","PASS",0.7369,0.0392,"Plate control","PASS","PFC",1.02754387733398,"Baseline","Baseline"
9915,"PFC_10",14,"OID20466","Q13574","DGKZ",0.9091,"Inflammation","B04411","20212097_SS211642","PASS",0.7369,0.2641,"Plate control","PASS","PFC",1.20088666372094,"1h","1h"
9916,"PFC_18",15,"OID20466","Q13574","DGKZ",0.9091,"Inflammation","B04411","20212097_SS211642","PASS",0.7369,0.1445,"Plate control","PASS","PFC",1.10534750267665,"1h","1h"
9917,"PFC_26",16,"OID20466","Q13574","DGKZ",0.9091,"Inflammation","B04411","20212097_SS211642","PASS",0.7369,0.1502,"Plate control","PASS","PFC",1.1097233017205,"24h","24h"
9918,"PFC_03",25,"OID20466","Q13574","DGKZ",0.9091,"Inflammation","B04411","20212097_SS211642","PASS",0.7369,-0.5113,"Plate control","PASS","PFC",0.701589956319801,"Baseline","Baseline"
9919,"PFC_11",26,"OID20466","Q13574","DGKZ",0.9091,"Inflammation","B04411","20212097_SS211642","PASS",0.7369,0.5947,"Plate control","PASS","PFC",1.51015852427336,"1h","1h"
9920,"PFC_19",27,"OID20466","Q13574","DGKZ",0.9091,"Inflammation","B04411","20212097_SS211642","PASS",0.7369,0.518,"Plate control","PASS","PFC",1.43196874120114,"24h","24h"
9921,"PFC_27",28,"OID20466","Q13574","DGKZ",0.9091,"Inflammation","B04411","20212097_SS211642","PASS",0.7369,0.849,"Plate control","PASS","PFC",1.80125195969586,"24h","24h"
9922,"PFC_04",37,"OID20466","Q13574","DGKZ",0.9091,"Inflammation","B04411","20212097_SS211642","PASS",0.7369,-0.3207,"Plate control","PASS","PFC",0.800681290340142,"Baseline","Baseline"
9923,"PFC_12",38,"OID20466","Q13574","DGKZ",0.9091,"Inflammation","B04411","20212097_SS211642","PASS",0.7369,-0.5304,"Plate control","PASS","PFC",0.692362743727686,"1h","1h"
9924,"PFC_20",39,"OID20466","Q13574","DGKZ",0.9091,"Inflammation","B04411","20212097_SS211642","PASS",0.7369,0.1809,"Plate control","PASS","PFC",1.13359083552602,"24h","24h"
9925,"PFC_05",49,"OID20466","Q13574","DGKZ",0.9091,"Inflammation","B04411","20212097_SS211642","PASS",0.7369,-0.1744,"Plate control","PASS","PFC",0.886135976070621,"Baseline","Baseline"
9926,"PFC_13",50,"OID20466","Q13574","DGKZ",0.9091,"Inflammation","B04411","20212097_SS211642","PASS",0.7369,0.1482,"Plate control","PASS","PFC",1.10818596441215,"1h","1h"
9927,"PFC_21",51,"OID20466","Q13574","DGKZ",0.9091,"Inflammation","B04411","20212097_SS211642","PASS",0.7369,-0.2286,"Plate control","PASS","PFC",0.853462695415617,"24h","24h"
9928,"PFC_06",61,"OID20466","Q13574","DGKZ",0.9091,"Inflammation","B04411","20212097_SS211642","PASS",0.7369,0.6553,"Plate control","PASS","PFC",1.57494341936709,"Baseline","Baseline"
9929,"PFC_14",62,"OID20466","Q13574","DGKZ",0.9091,"Inflammation","B04411","20212097_SS211642","PASS",0.7369,0.4684,"Plate control","PASS","PFC",1.3835741840544,"1h","1h"
9930,"PFC_22",63,"OID20466","Q13574","DGKZ",0.9091,"Inflammation","B04411","20212097_SS211642","PASS",0.7369,0.1774,"Plate control","PASS","PFC",1.13084406021201,"24h","24h"
9931,"PFC_07",73,"OID20466","Q13574","DGKZ",0.9091,"Inflammation","B04411","20212097_SS211642","PASS",0.7369,-0.4639,"Plate control","PASS","PFC",0.725023669509003,"Baseline","Baseline"
9932,"PFC_15",74,"OID20466","Q13574","DGKZ",0.9091,"Inflammation","B04411","20212097_SS211642","PASS",0.7369,-0.105,"Plate control","PASS","PFC",0.929804942613162,"1h","1h"
9933,"PFC_23",75,"OID20466","Q13574","DGKZ",0.9091,"Inflammation","B04411","20212097_SS211642","PASS",0.7369,0.3141,"Plate control","PASS","PFC",1.24323584045919,"24h","24h"
9934,"PFC_08",85,"OID20466","Q13574","DGKZ",0.9091,"Inflammation","B04411","20212097_SS211642","PASS",0.7369,-0.0693,"Plate control","PASS","PFC",0.953100333037743,"Baseline","Baseline"
9935,"PFC_16",86,"OID20466","Q13574","DGKZ",0.9091,"Inflammation","B04411","20212097_SS211642","PASS",0.7369,0.4327,"Plate control","PASS","PFC",1.34975728180215,"1h","1h"
9936,"PFC_24",87,"OID20466","Q13574","DGKZ",0.9091,"Inflammation","B04411","20212097_SS211642","PASS",0.7369,0.7458,"Plate control","PASS","PFC",1.67690388452772,"24h","24h"
9937,"PFC_01",1,"OID20467","Q9Y2J8","PADI2",0.5455,"Inflammation","B04411","20212097_SS211642","PASS",0.1246,-0.0557,"Plate control","PASS","PFC",0.96212750265439,"Baseline","Baseline"
9938,"PFC_09",2,"OID20467","Q9Y2J8","PADI2",0.5455,"Inflammation","B04411","20212097_SS211642","PASS",0.1246,0.2854,"Plate control","PASS","PFC",1.21874812556624,"Baseline","Baseline"
9939,"PFC_17",3,"OID20467","Q9Y2J8","PADI2",0.5455,"Inflammation","B04411","20212097_SS211642","PASS",0.1246,0.2771,"Plate control","PASS","PFC",1.2117566500899,"1h","1h"
9940,"PFC_25",4,"OID20467","Q9Y2J8","PADI2",0.5455,"Inflammation","B04411","20212097_SS211642","PASS",0.1246,0.0534,"Plate control","PASS","PFC",1.03770761032545,"24h","24h"
9941,"PFC_02",13,"OID20467","Q9Y2J8","PADI2",0.5455,"Inflammation","B04411","20212097_SS211642","PASS",0.1246,-0.0965,"Plate control","PASS","PFC",0.935299291620507,"Baseline","Baseline"
9942,"PFC_10",14,"OID20467","Q9Y2J8","PADI2",0.5455,"Inflammation","B04411","20212097_SS211642","PASS",0.1246,-0.1099,"Plate control","PASS","PFC",0.926652290306596,"1h","1h"
9943,"PFC_18",15,"OID20467","Q9Y2J8","PADI2",0.5455,"Inflammation","B04411","20212097_SS211642","PASS",0.1246,0.0972,"Plate control","PASS","PFC",1.06969536887275,"1h","1h"
9944,"PFC_26",16,"OID20467","Q9Y2J8","PADI2",0.5455,"Inflammation","B04411","20212097_SS211642","PASS",0.1246,-0.0384,"Plate control","PASS","PFC",0.973734254651807,"24h","24h"
9945,"PFC_03",25,"OID20467","Q9Y2J8","PADI2",0.5455,"Inflammation","B04411","20212097_SS211642","PASS",0.1246,0.5195,"Plate control","PASS","PFC",1.43345836310579,"Baseline","Baseline"
9946,"PFC_11",26,"OID20467","Q9Y2J8","PADI2",0.5455,"Inflammation","B04411","20212097_SS211642","PASS",0.1246,-0.0483,"Plate control","PASS","PFC",0.967075211067162,"1h","1h"
9947,"PFC_19",27,"OID20467","Q9Y2J8","PADI2",0.5455,"Inflammation","B04411","20212097_SS211642","PASS",0.1246,-0.2243,"Plate control","PASS","PFC",0.856010263707656,"24h","24h"
9948,"PFC_27",28,"OID20467","Q9Y2J8","PADI2",0.5455,"Inflammation","B04411","20212097_SS211642","PASS",0.1246,-0.0678,"Plate control","PASS","PFC",0.954091806589203,"24h","24h"
9949,"PFC_04",37,"OID20467","Q9Y2J8","PADI2",0.5455,"Inflammation","B04411","20212097_SS211642","PASS",0.1246,0.1483,"Plate control","PASS","PFC",1.10826278067204,"Baseline","Baseline"
9950,"PFC_12",38,"OID20467","Q9Y2J8","PADI2",0.5455,"Inflammation","B04411","20212097_SS211642","PASS",0.1246,0.5845,"Plate control","PASS","PFC",1.49951920546462,"1h","1h"
9951,"PFC_20",39,"OID20467","Q9Y2J8","PADI2",0.5455,"Inflammation","B04411","20212097_SS211642","PASS",0.1246,-0.2534,"Plate control","PASS","PFC",0.838917007675307,"24h","24h"
9952,"PFC_05",49,"OID20467","Q9Y2J8","PADI2",0.5455,"Inflammation","B04411","20212097_SS211642","PASS",0.1246,0.0195,"Plate control","PASS","PFC",1.01360812910067,"Baseline","Baseline"
9953,"PFC_13",50,"OID20467","Q9Y2J8","PADI2",0.5455,"Inflammation","B04411","20212097_SS211642","PASS",0.1246,0.3114,"Plate control","PASS","PFC",1.24091130369242,"1h","1h"
9954,"PFC_21",51,"OID20467","Q9Y2J8","PADI2",0.5455,"Inflammation","B04411","20212097_SS211642","PASS",0.1246,0.7186,"Plate control","PASS","PFC",1.64558437443369,"24h","24h"
9955,"PFC_06",61,"OID20467","Q9Y2J8","PADI2",0.5455,"Inflammation","B04411","20212097_SS211642","PASS",0.1246,0.3936,"Plate control","PASS","PFC",1.31366735072413,"Baseline","Baseline"
9956,"PFC_14",62,"OID20467","Q9Y2J8","PADI2",0.5455,"Inflammation","B04411","20212097_SS211642","PASS",0.1246,0.3491,"Plate control","PASS","PFC",1.27376576298138,"1h","1h"
9957,"PFC_22",63,"OID20467","Q9Y2J8","PADI2",0.5455,"Inflammation","B04411","20212097_SS211642","PASS",0.1246,-0.3238,"Plate control","PASS","PFC",0.798962668516433,"24h","24h"
9958,"PFC_07",73,"OID20467","Q9Y2J8","PADI2",0.5455,"Inflammation","B04411","20212097_SS211642","PASS",0.1246,0.0139,"Plate control","PASS","PFC",1.00968130939582,"Baseline","Baseline"
9959,"PFC_15",74,"OID20467","Q9Y2J8","PADI2",0.5455,"Inflammation","B04411","20212097_SS211642","PASS",0.1246,-0.0891,"Plate control","PASS","PFC",0.940109036857851,"1h","1h"
9960,"PFC_23",75,"OID20467","Q9Y2J8","PADI2",0.5455,"Inflammation","B04411","20212097_SS211642","PASS",0.1246,-0.1383,"Plate control","PASS","PFC",0.90858916099854,"24h","24h"
9961,"PFC_08",85,"OID20467","Q9Y2J8","PADI2",0.5455,"Inflammation","B04411","20212097_SS211642","PASS",0.1246,0.1535,"Plate control","PASS","PFC",1.11226457225211,"Baseline","Baseline"
9962,"PFC_16",86,"OID20467","Q9Y2J8","PADI2",0.5455,"Inflammation","B04411","20212097_SS211642","PASS",0.1246,-0.1602,"Plate control","PASS","PFC",0.894901002707036,"1h","1h"
9963,"PFC_24",87,"OID20467","Q9Y2J8","PADI2",0.5455,"Inflammation","B04411","20212097_SS211642","PASS",0.1246,-0.2999,"Plate control","PASS","PFC",0.81230869935337,"24h","24h"
9964,"PFC_01",1,"OID20468","Q04759","PRKCQ",1,"Inflammation","B04411","20212097_SS211642","PASS",1.187,-0.186,"Plate control","PASS","PFC",0.879039560911787,"Baseline","Baseline"
9965,"PFC_09",2,"OID20468","Q04759","PRKCQ",1,"Inflammation","B04411","20212097_SS211642","PASS",1.187,-0.5258,"Plate control","PASS","PFC",0.694573849589768,"Baseline","Baseline"
9966,"PFC_17",3,"OID20468","Q04759","PRKCQ",1,"Inflammation","B04411","20212097_SS211642","PASS",1.187,-0.6864,"Plate control","PASS","PFC",0.621402521203159,"1h","1h"
9967,"PFC_25",4,"OID20468","Q04759","PRKCQ",1,"Inflammation","B04411","20212097_SS211642","PASS",1.187,0.1291,"Plate control","PASS","PFC",1.09361125821357,"24h","24h"
9968,"PFC_02",13,"OID20468","Q04759","PRKCQ",1,"Inflammation","B04411","20212097_SS211642","PASS",1.187,0.248,"Plate control","PASS","PFC",1.18755966607341,"Baseline","Baseline"

Supplemental table 1_Olink proximity extension assay (PEA) data

,"SampleID","Index","OlinkID","UniProt","Assay","MissingFreq","Panel","Panel_Lot_Nr","PlateID","QC_Warning","LOD","NPX","Normalization","Assay_Warning","condition","Value","group","alt_group"
9969,"PFC_10",14,"OID20468","Q04759","PRKCQ",1,"Inflammation","B04411","20212097_SS211642","PASS",1.187,0.0674,"Plate control","PASS","PFC",1.04782660605208,"1h","1h"
9970,"PFC_18",15,"OID20468","Q04759","PRKCQ",1,"Inflammation","B04411","20212097_SS211642","PASS",1.187,0.1118,"Plate control","PASS","PFC",1.08057559207055,"1h","1h"
9971,"PFC_26",16,"OID20468","Q04759","PRKCQ",1,"Inflammation","B04411","20212097_SS211642","PASS",1.187,0.6539,"Plate control","PASS","PFC",1.57341582605291,"24h","24h"
9972,"PFC_03",25,"OID20468","Q04759","PRKCQ",1,"Inflammation","B04411","20212097_SS211642","PASS",1.187,-0.2722,"Plate control","PASS","PFC",0.828055860460334,"Baseline","Baseline"
9973,"PFC_11",26,"OID20468","Q04759","PRKCQ",1,"Inflammation","B04411","20212097_SS211642","PASS",1.187,0.8344,"Plate control","PASS","PFC",1.78311530780331,"1h","1h"
9974,"PFC_19",27,"OID20468","Q04759","PRKCQ",1,"Inflammation","B04411","20212097_SS211642","PASS",1.187,0.3305,"Plate control","PASS","PFC",1.25744909766077,"24h","24h"
9975,"PFC_27",28,"OID20468","Q04759","PRKCQ",1,"Inflammation","B04411","20212097_SS211642","PASS",1.187,0.242,"Plate control","PASS","PFC",1.18263100024414,"24h","24h"
9976,"PFC_04",37,"OID20468","Q04759","PRKCQ",1,"Inflammation","B04411","20212097_SS211642","PASS",1.187,-0.3448,"Plate control","PASS","PFC",0.787417128195681,"Baseline","Baseline"
9977,"PFC_12",38,"OID20468","Q04759","PRKCQ",1,"Inflammation","B04411","20212097_SS211642","PASS",1.187,-0.2917,"Plate control","PASS","PFC",0.816938851107114,"1h","1h"
9978,"PFC_20",39,"OID20468","Q04759","PRKCQ",1,"Inflammation","B04411","20212097_SS211642","PASS",1.187,0.1012,"Plate control","PASS","PFC",1.07266530949811,"24h","24h"
9979,"PFC_05",49,"OID20468","Q04759","PRKCQ",1,"Inflammation","B04411","20212097_SS211642","PASS",1.187,-0.0908,"Plate control","PASS","PFC",0.939001911598456,"Baseline","Baseline"
9980,"PFC_13",50,"OID20468","Q04759","PRKCQ",1,"Inflammation","B04411","20212097_SS211642","PASS",1.187,0.2493,"Plate control","PASS","PFC",1.18863024807162,"1h","1h"
9981,"PFC_21",51,"OID20468","Q04759","PRKCQ",1,"Inflammation","B04411","20212097_SS211642","PASS",1.187,0.1414,"Plate control","PASS","PFC",1.10297493026388,"24h","24h"
9982,"PFC_06",61,"OID20468","Q04759","PRKCQ",1,"Inflammation","B04411","20212097_SS211642","PASS",1.187,-0.0518,"Plate control","PASS","PFC",0.964731915555852,"Baseline","Baseline"
9983,"PFC_14",62,"OID20468","Q04759","PRKCQ",1,"Inflammation","B04411","20212097_SS211642","PASS",1.187,0.0641,"Plate control","PASS","PFC",1.04543256155707,"1h","1h"
9984,"PFC_22",63,"OID20468","Q04759","PRKCQ",1,"Inflammation","B04411","20212097_SS211642","PASS",1.187,0.2516,"Plate control","PASS","PFC",1.19052671950576,"24h","24h"
9985,"PFC_07",73,"OID20468","Q04759","PRKCQ",1,"Inflammation","B04411","20212097_SS211642","PASS",1.187,0.1687,"Plate control","PASS","PFC",1.12404516085442,"Baseline","Baseline"
9986,"PFC_15",74,"OID20468","Q04759","PRKCQ",1,"Inflammation","B04411","20212097_SS211642","PASS",1.187,-0.2338,"Plate control","PASS","PFC",0.850392041262259,"1h","1h"
9987,"PFC_23",75,"OID20468","Q04759","PRKCQ",1,"Inflammation","B04411","20212097_SS211642","PASS",1.187,-0.4529,"Plate control","PASS","PFC",0.730572826967369,"24h","24h"
9988,"PFC_08",85,"OID20468","Q04759","PRKCQ",1,"Inflammation","B04411","20212097_SS211642","PASS",1.187,-0.2525,"Plate control","PASS","PFC",0.839440514611375,"Baseline","Baseline"
9989,"PFC_16",86,"OID20468","Q04759","PRKCQ",1,"Inflammation","B04411","20212097_SS211642","PASS",1.187,0.2487,"Plate control","PASS","PFC",1.18813601342884,"1h","1h"
9990,"PFC_24",87,"OID20468","Q04759","PRKCQ",1,"Inflammation","B04411","20212097_SS211642","PASS",1.187,0.6952,"Plate control","PASS","PFC",1.61910887368758,"24h","24h"
9991,"PFC_01",1,"OID20469","Q16552","IL17A",0.8409,"Inflammation","B04411","20212097_SS211642","PASS",0.16,-0.1629,"Plate control","PASS","PFC",0.893227764037664,"Baseline","Baseline"
9992,"PFC_09",2,"OID20469","Q16552","IL17A",0.8409,"Inflammation","B04411","20212097_SS211642","PASS",0.16,0.3696,"Plate control","PASS","PFC",1.29199456401775,"Baseline","Baseline"
9993,"PFC_17",3,"OID20469","Q16552","IL17A",0.8409,"Inflammation","B04411","20212097_SS211642","PASS",0.16,-0.4662,"Plate control","PASS","PFC",0.72386872971994,"1h","1h"
9994,"PFC_25",4,"OID20469","Q16552","IL17A",0.8409,"Inflammation","B04411","20212097_SS211642","PASS",0.16,-0.4121,"Plate control","PASS","PFC",0.751528645231213,"24h","24h"
9995,"PFC_02",13,"OID20469","Q16552","IL17A",0.8409,"Inflammation","B04411","20212097_SS211642","PASS",0.16,0.0475,"Plate control","PASS","PFC",1.03347249989918,"Baseline","Baseline"
9996,"PFC_10",14,"OID20469","Q16552","IL17A",0.8409,"Inflammation","B04411","20212097_SS211642","PASS",0.16,-0.248,"Plate control","PASS","PFC",0.842062953608416,"1h","1h"
9997,"PFC_18",15,"OID20469","Q16552","IL17A",0.8409,"Inflammation","B04411","20212097_SS211642","PASS",0.16,-0.1414,"Plate control","PASS","PFC",0.906638920397546,"1h","1h"
9998,"PFC_26",16,"OID20469","Q16552","IL17A",0.8409,"Inflammation","B04411","20212097_SS211642","PASS",0.16,0.2453,"Plate control","PASS","PFC",1.18533922967938,"24h","24h"
9999,"PFC_03",25,"OID20469","Q16552","IL17A",0.8409,"Inflammation","B04411","20212097_SS211642","PASS",0.16,0.0284,"Plate control","PASS","PFC",1.01988041469503,"Baseline","Baseline"
10000,"PFC_11",26,"OID20469","Q16552","IL17A",0.8409,"Inflammation","B04411","20212097_SS211642","PASS",0.16,-0.3433,"Plate control","PASS","PFC",0.788236247893273,"1h","1h"
10001,"PFC_19",27,"OID20469","Q16552","IL17A",0.8409,"Inflammation","B04411","20212097_SS211642","PASS",0.16,-0.2979,"Plate control","PASS","PFC",0.813435579235915,"24h","24h"
10002,"PFC_27",28,"OID20469","Q16552","IL17A",0.8409,"Inflammation","B04411","20212097_SS211642","PASS",0.16,-0.3112,"Plate control","PASS","PFC",0.805971092429919,"24h","24h"
10003,"PFC_04",37,"OID20469","Q16552","IL17A",0.8409,"Inflammation","B04411","20212097_SS211642","PASS",0.16,-0.5466,"Plate control","PASS","PFC",0.684631700446837,"Baseline","Baseline"
10004,"PFC_12",38,"OID20469","Q16552","IL17A",0.8409,"Inflammation","B04411","20212097_SS211642","PASS",0.16,-0.1249,"Plate control","PASS","PFC",0.91706760728432,"1h","1h"
10005,"PFC_20",39,"OID20469","Q16552","IL17A",0.8409,"Inflammation","B04411","20212097_SS211642","PASS",0.16,-0.67,"Plate control","PASS","PFC",0.628506687260914,"24h","24h"
10006,"PFC_05",49,"OID20469","Q16552","IL17A",0.8409,"Inflammation","B04411","20212097_SS211642","PASS",0.16,-0.3461,"Plate control","PASS","PFC",0.786707913026457,"Baseline","Baseline"
10007,"PFC_13",50,"OID20469","Q16552","IL17A",0.8409,"Inflammation","B04411","20212097_SS211642","PASS",0.16,-0.4185,"Plate control","PASS","PFC",0.748202141354273,"1h","1h"
10008,"PFC_21",51,"OID20469","Q16552","IL17A",0.8409,"Inflammation","B04411","20212097_SS211642","PASS",0.16,0.1355,"Plate control","PASS","PFC",1.09847344971135,"24h","24h"
10009,"PFC_06",61,"OID20469","Q16552","IL17A",0.8409,"Inflammation","B04411","20212097_SS211642","PASS",0.16,-0.1832,"Plate control","PASS","PFC",0.880747268166291,"Baseline","Baseline"
10010,"PFC_14",62,"OID20469","Q16552","IL17A",0.8409,"Inflammation","B04411","20212097_SS211642","PASS",0.16,-0.3203,"Plate control","PASS","PFC",0.800903317109737,"1h","1h"
10011,"PFC_22",63,"OID20469","Q16552","IL17A",0.8409,"Inflammation","B04411","20212097_SS211642","PASS",0.16,-0.7732,"Plate control","PASS","PFC",0.585118200508404,"24h","24h"
10012,"PFC_07",73,"OID20469","Q16552","IL17A",0.8409,"Inflammation","B04411","20212097_SS211642","PASS",0.16,-0.5542,"Plate control","PASS","PFC",0.681034599333361,"Baseline","Baseline"
10013,"PFC_15",74,"OID20469","Q16552","IL17A",0.8409,"Inflammation","B04411","20212097_SS211642","PASS",0.16,-0.1384,"Plate control","PASS","PFC",0.908526184579639,"1h","1h"
10014,"PFC_23",75,"OID20469","Q16552","IL17A",0.8409,"Inflammation","B04411","20212097_SS211642","PASS",0.16,-0.5443,"Plate control","PASS","PFC",0.685724037163752,"24h","24h"
10015,"PFC_08",85,"OID20469","Q16552","IL17A",0.8409,"Inflammation","B04411","20212097_SS211642","PASS",0.16,-0.0133,"Plate control","PASS","PFC",0.990823505884747,"Baseline","Baseline"
10016,"PFC_16",86,"OID20469","Q16552","IL17A",0.8409,"Inflammation","B04411","20212097_SS211642","PASS",0.16,-0.4121,"Plate control","PASS","PFC",0.751528645231213,"1h","1h"
10017,"PFC_24",87,"OID20469","Q16552","IL17A",0.8409,"Inflammation","B04411","20212097_SS211642","PASS",0.16,-0.0453,"Plate control","PASS","PFC",0.969088279738906,"24h","24h"
10018,"PFC_01",1,"OID20470","Q12968","NFATC3",0.7273,"Inflammation","B04411","20212097_SS211642","PASS",0.9119,0.2621,"Plate control","PASS","PFC",1.19922303471687,"Baseline","Baseline"
10019,"PFC_09",2,"OID20470","Q12968","NFATC3",0.7273,"Inflammation","B04411","20212097_SS211642","PASS",0.9119,0.7315,"Plate control","PASS","PFC",1.66036450996498,"Baseline","Baseline"
10020,"PFC_17",3,"OID20470","Q12968","NFATC3",0.7273,"Inflammation","B04411","20212097_SS211642","PASS",0.9119,-0.5517,"Plate control","PASS","PFC",0.682215765471243,"1h","1h"
10021,"PFC_25",4,"OID20470","Q12968","NFATC3",0.7273,"Inflammation","B04411","20212097_SS211642","PASS",0.9119,1.1273,"Plate control","PASS","PFC",2.18449529729183,"24h","24h"
10022,"PFC_02",13,"OID20470","Q12968","NFATC3",0.7273,"Inflammation","B04411","20212097_SS211642","PASS",0.9119,1.0966,"Plate control","PASS","PFC",2.13850117514288,"Baseline","Baseline"
10023,"PFC_10",14,"OID20470","Q12968","NFATC3",0.7273,"Inflammation","B04411","20212097_SS211642","PASS",0.9119,-0.1313,"Plate control","PASS","PFC",0.913008375516635,"1h","1h"
10024,"PFC_18",15,"OID20470","Q12968","NFATC3",0.7273,"Inflammation","B04411","20212097_SS211642","PASS",0.9119,0.5062,"Plate control","PASS","PFC",1.42030424087229,"1h","1h"
10025,"PFC_26",16,"OID20470","Q12968","NFATC3",0.7273,"Inflammation","B04411","20212097_SS211642","PASS",0.9119,-0.0676,"Plate control","PASS","PFC",0.954224080966699,"24h","24h"
10026,"PFC_03",25,"OID20470","Q12968","NFATC3",0.7273,"Inflammation","B04411","20212097_SS211642","PASS",0.9119,0.8035,"Plate control","PASS","PFC",1.74533019208588,"Baseline","Baseline"
10027,"PFC_11",26,"OID20470","Q12968","NFATC3",0.7273,"Inflammation","B04411","20212097_SS211642","PASS",0.9119,1.0539,"Plate control","PASS","PFC",2.07613462941209,"1h","1h"
10028,"PFC_19",27,"OID20470","Q12968","NFATC3",0.7273,"Inflammation","B04411","20212097_SS211642","PASS",0.9119,0.3451,"Plate control","PASS","PFC",1.27023902574734,"24h","24h"
10029,"PFC_27",28,"OID20470","Q12968","NFATC3",0.7273,"Inflammation","B04411","20212097_SS211642","PASS",0.9119,1.2228,"Plate control","PASS","PFC",2.33399262112747,"24h","24h"
10030,"PFC_04",37,"OID20470","Q12968","NFATC3",0.7273,"Inflammation","B04411","20212097_SS211642","PASS",0.9119,0.2538,"Plate control","PASS","PFC",1.19234356695646,"Baseline","Baseline"
10031,"PFC_12",38,"OID20470","Q12968","NFATC3",0.7273,"Inflammation","B04411","20212097_SS211642","PASS",0.9119,1.0667,"Plate control","PASS","PFC",2.09463664146491,"1h","1h"
10032,"PFC_20",39,"OID20470","Q12968","NFATC3",0.7273,"Inflammation","B04411","20212097_SS211642","PASS",0.9119,0.9461,"Plate control","PASS","PFC",1.92665732912162,"24h","24h"
10033,"PFC_05",49,"OID20470","Q12968","NFATC3",0.7273,"Inflammation","B04411","20212097_SS211642","PASS",0.9119,0.0249,"Plate control","PASS","PFC",1.0174091682289,"Baseline","Baseline"
10034,"PFC_13",50,"OID20470","Q12968","NFATC3",0.7273,"Inflammation","B04411","20212097_SS211642","PASS",0.9119,-0.2777,"Plate control","PASS","PFC",0.824905064962554,"1h","1h"
10035,"PFC_21",51,"OID20470","Q12968","NFATC3",0.7273,"Inflammation","B04411","20212097_SS211642","PASS",0.9119,0.9013,"Plate control","PASS","PFC",1.86774823677928,"24h","24h"
10036,"PFC_06",61,"OID20470","Q12968","NFATC3",0.7273,"Inflammation","B04411","20212097_SS211642","PASS",0.9119,0.4148,"Plate control","PASS","PFC",1.33311385545858,"Baseline","Baseline"
10037,"PFC_14",62,"OID20470","Q12968","NFATC3",0.7273,"Inflammation","B04411","20212097_SS211642","PASS",0.9119,-0.1838,"Plate control","PASS","PFC",0.880381051832686,"1h","1h"
10038,"PFC_22",63,"OID20470","Q12968","NFATC3",0.7273,"Inflammation","B04411","20212097_SS211642","PASS",0.9119,0.0635,"Plate control","PASS","PFC",1.04499786877565,"24h","24h"
10039,"PFC_07",73,"OID20470","Q12968","NFATC3",0.7273,"Inflammation","B04411","20212097_SS211642","PASS",0.9119,0.7898,"Plate control","PASS","PFC",1.728834778612,"Baseline","Baseline"
10040,"PFC_15",74,"OID20470","Q12968","NFATC3",0.7273,"Inflammation","B04411","20212097_SS211642","PASS",0.9119,0.2304,"Plate control","PASS","PFC",1.17316017321074,"1h","1h"
10041,"PFC_23",75,"OID20470","Q12968","NFATC3",0.7273,"Inflammation","B04411","20212097_SS211642","PASS",0.9119,-0.5265,"Plate control","PASS","PFC",0.694236922001648,"24h","24h"
10042,"PFC_08",85,"OID20470","Q12968","NFATC3",0.7273,"Inflammation","B04411","20212097_SS211642","PASS",0.9119,-0.3304,"Plate control","PASS","PFC",0.795315944781226,"Baseline","Baseline"
10043,"PFC_16",86,"OID20470","Q12968","NFATC3",0.7273,"Inflammation","B04411","20212097_SS211642","PASS",0.9119,1.1344,"Plate control","PASS","PFC",2.19527244955185,"1h","1h"
10044,"PFC_24",87,"OID20470","Q12968","NFATC3",0.7273,"Inflammation","B04411","20212097_SS211642","PASS",0.9119,0.1613,"Plate control","PASS","PFC",1.11829436956637,"24h","24h"
10045,"PFC_01",1,"OID20471","Q14435","GALNT3",0,"Inflammation","B04411","20212097_SS211642","PASS",-1.4405,0.171,"Plate control","PASS","PFC",1.12583858613676,"Baseline","Baseline"
10046,"PFC_09",2,"OID20471","Q14435","GALNT3",0,"Inflammation","B04411","20212097_SS211642","PASS",-1.4405,0.517,"Plate control","PASS","PFC",1.43097652002291,"Baseline","Baseline"
10047,"PFC_17",3,"OID20471","Q14435","GALNT3",0,"Inflammation","B04411","20212097_SS211642","PASS",-1.4405,0.125,"Plate control","PASS","PFC",1.09050773266526,"1h","1h"
10048,"PFC_25",4,"OID20471","Q14435","GALNT3",0,"Inflammation","B04411","20212097_SS211642","PASS",-1.4405,-0.2733,"Plate control","PASS","PFC",0.827424740050016,"24h","24h"
10049,"PFC_02",13,"OID20471","Q14435","GALNT3",0,"Inflammation","B04411","20212097_SS211642","PASS",-1.4405,-0.2804,"Plate control","PASS","PFC",0.823362701003769,"Baseline","Baseline"
10050,"PFC_10",14,"OID20471","Q14435","GALNT3",0,"Inflammation","B04411","20212097_SS211642","PASS",-1.4405,-0.5354,"Plate control","PASS","PFC",0.689967350606611,"1h","1h"
10051,"PFC_18",15,"OID20471","Q14435","GALNT3",0,"Inflammation","B04411","20212097_SS211642","PASS",-1.4405,0.1059,"Plate control","PASS","PFC",1.07616552808834,"1h","1h"
10052,"PFC_26",16,"OID20471","Q14435","GALNT3",0,"Inflammation","B04411","20212097_SS211642","PASS",-1.4405,0.1432,"Plate control","PASS","PFC",1.10435193223743,"24h","24h"
10053,"PFC_03",25,"OID20471","Q14435","GALNT3",0,"Inflammation","B04411","20212097_SS211642","PASS",-1.4405,-0.5313,"Plate control","PASS","PFC",0.691930960066655,"Baseline","Baseline"
10054,"PFC_11",26,"OID20471","Q14435","GALNT3",0,"Inflammation","B04411","20212097_SS211642","PASS",-1.4405,-0.3445,"Plate control","PASS","PFC",0.787580884009823,"1h","1h"
10055,"PFC_19",27,"OID20471","Q14435","GALNT3",0,"Inflammation","B04411","20212097_SS211642","PASS",-1.4405,0.0883,"Plate control","PASS","PFC",1.06311672028233,"24h","24h"
10056,"PFC_27",28,"OID20471","Q14435","GALNT3",0,"Inflammation","B04411","20212097_SS211642","PASS",-1.4405,0.4127,"Plate control","PASS","PFC",1.33117477444134,"24h","24h"
10057,"PFC_04",37,"OID20471","Q14435","GALNT3",0,"Inflammation","B04411","20212097_SS211642","PASS",-1.4405,0.4402,"Plate control","PASS","PFC",1.35679240577691,"Baseline","Baseline"
10058,"PFC_12",38,"OID20471","Q14435","GALNT3",0,"Inflammation","B04411","20212097_SS211642","PASS",-1.4405,-0.274,"Plate control","PASS","PFC",0.827023368443266,"1h","1h"
10059,"PFC_20",39,"OID20471","Q14435","GALNT3",0,"Inflammation","B04411","20212097_SS211642","PASS",-1.4405,-0.947,"Plate control","PASS","PFC",0.518709968282806,"24h","24h"
10060,"PFC_05",49,"OID20471","Q14435","GALNT3",0,"Inflammation","B04411","20212097_SS211642","PASS",-1.4405,-0.1584,"Plate control","PASS","PFC",0.89601823612101,"Baseline","Baseline"
10061,"PFC_13",50,"OID20471","Q14435","GALNT3",0,"Inflammation","B04411","20212097_SS211642","PASS",-1.4405,0.1501,"Plate control","PASS","PFC",1.10964638422851,"1h","1h"
10062,"PFC_21",51,"OID20471","Q14435","GALNT3",0,"Inflammation","B04411","20212097_SS211642","PASS",-1.4405,0.0373,"Plate control","PASS","PFC",1.02619151367835,"24h","24h"
10063,"PFC_06",61,"OID20471","Q14435","GALNT3",0,"Inflammation","B04411","20212097_SS211642","PASS",-1.4405,-0.2673,"Plate control","PASS","PFC",0.830873068431171,"Baseline","Baseline"
10064,"PFC_14",62,"OID20471","Q14435","GALNT3",0,"Inflammation","B04411","20212097_SS211642","PASS",-1.4405,0.2888,"Plate control","PASS","PFC",1.22162373692451,"1h","1h"
10065,"PFC_22",63,"OID20471","Q14435","GALNT3",0,"Inflammation","B04411","20212097_SS211642","PASS",-1.4405,0.5947,"Plate control","PASS","PFC",1.51015852427336,"24h","24h"
10066,"PFC_07",73,"OID20471","Q14435","GALNT3",0,"Inflammation","B04411","20212097_SS211642","PASS",-1.4405,-0.6052,"Plate control","PASS","PFC",0.657380241533242,"Baseline","Baseline"
10067,"PFC_15",74,"OID20471","Q14435","GALNT3",0,"Inflammation","B04411","20212097_SS211642","PASS",-1.4405,-0.4415,"Plate control","PASS","PFC",0.73636859277235,"1h","1h"
10068,"PFC_23",75,"OID20471","Q14435","GALNT3",0,"Inflammation","B04411","20212097_SS211642","PASS",-1.4405,0.3315,"Plate control","PASS","PFC",1.25832099709992,"24h","24h"
10069,"PFC_08",85,"OID20471","Q14435","GALNT3",0,"Inflammation","B04411","20212097_SS211642","PASS",-1.4405,-0.226,"Plate control","PASS","PFC",0.855002177891973,"Baseline","Baseline"
10070,"PFC_16",86,"OID20471","Q14435","GALNT3",0,"Inflammation","B04411","20212097_SS211642","PASS",-1.4405,-0.386,"Plate control","PASS","PFC",0.765248384911334,"1h","1h"
10071,"PFC_24",87,"OID20471","Q14435","GALNT3",0,"Inflammation","B04411","20212097_SS211642","PASS",-1.4405,-0.1437,"Plate control","PASS","PFC",0.905194673254291,"24h","24h"
10072,"PFC_01",1,"OID20472","P05113","IL5",0.3864,"Inflammation","B04411","20212097_SS211642","PASS",-1.0427,1.5871,"Plate control","PASS","PFC",3.00444809913745,"Baseline","Baseline"
10073,"PFC_09",2,"OID20472","P05113","IL5",0.3864,"Inflammation","B04411","20212097_SS211642","PASS",-1.0427,-2.9363,"Plate control","PASS","PFC",0.130642843285356,"Baseline","Baseline"
10074,"PFC_17",3,"OID20472","P05113","IL5",0.3864,"Inflammation","B04411","20212097_SS211642","PASS",-1.0427,-0.7212,"Plate control","PASS","PFC",0.60659268269374,"1h","1h"
10075,"PFC_25",4,"OID20472","P05113","IL5",0.3864,"Inflammation","B04411","20212097_SS211642","PASS",-1.0427,-5.0967,"Plate control","PASS","PFC",0.0292240512685639,"24h","24h"
10076,"PFC_02",13,"OID20472","P05113","IL5",0.3864,"Inflammation","B04411","20212097_SS211642","PASS",-1.0427,-0.8111,"Plate control","PASS","PFC",0.569947129252964,"Baseline","Baseline"
10077,"PFC_10",14,"OID20472","P05113","IL5",0.3864,"Inflammation","B04411","20212097_SS211642","PASS",-1.0427,0.8085,"Plate control","PASS","PFC",1.75138953957884,"1h","1h"
10078,"PFC_18",15,"OID20472","P05113","IL5",0.3864,"Inflammation","B04411","20212097_SS211642","PASS",-1.0427,-1.0691,"Plate control","PASS","PFC",0.476616234979146,"1h","1h"
10079,"PFC_26",16,"OID20472","P05113","IL5",0.3864,"Inflammation","B04411","20212097_SS211642","PASS",-1.0427,-1.6206,"Plate control","PASS","PFC",0.32520018877639,"24h","24h"
10080,"PFC_03",25,"OID20472","P05113","IL5",0.3864,"Inflammation","B04411","20212097_SS211642","PASS",-1.0427,-1.6674,"Plate control","PASS","PFC",0.314820196192677,"Baseline","Baseline"

Supplemental table 1_Olink proximity extension assay (PEA) data

**,"SampleID","Index","OlinkID","UniProt","Assay","MissingFreq","Panel","Panel_Lot_Nr","PlateID","QC_Warning","LOD","NPX","Normalization","Assay_Warning","condition","Value","group","alt_group"**
10081,"PFC_11",26,"OID20472","P05113","IL5",0.3864,"Inflammation","B04411","20212097_SS211642","PASS",-1.0427,-0.0404,"Plate control","PASS","PFC",0.972385307580248,"1h","1h"
10082,"PFC_19",27,"OID20472","P05113","IL5",0.3864,"Inflammation","B04411","20212097_SS211642","PASS",-1.0427,0.9603,"Plate control","PASS","PFC",1.94571445270651,"24h","24h"
10083,"PFC_27",28,"OID20472","P05113","IL5",0.3864,"Inflammation","B04411","20212097_SS211642","PASS",-1.0427,0.2764,"Plate control","PASS","PFC",1.21116884471004,"24h","24h"
10084,"PFC_04",37,"OID20472","P05113","IL5",0.3864,"Inflammation","B04411","20212097_SS211642","PASS",-1.0427,0.7896,"Plate control","PASS","PFC",1.72859512783322,"Baseline","Baseline"
10085,"PFC_12",38,"OID20472","P05113","IL5",0.3864,"Inflammation","B04411","20212097_SS211642","PASS",-1.0427,-0.0521,"Plate control","PASS","PFC",0.964531326050103,"1h","1h"
10086,"PFC_20",39,"OID20472","P05113","IL5",0.3864,"Inflammation","B04411","20212097_SS211642","PASS",-1.0427,-1.1841,"Plate control","PASS","PFC",0.440099000386188,"24h","24h"
10087,"PFC_05",49,"OID20472","P05113","IL5",0.3864,"Inflammation","B04411","20212097_SS211642","PASS",-1.0427,-1.3444,"Plate control","PASS","PFC",0.393817738424383,"Baseline","Baseline"
10088,"PFC_13",50,"OID20472","P05113","IL5",0.3864,"Inflammation","B04411","20212097_SS211642","PASS",-1.0427,-0.0383,"Plate control","PASS","PFC",0.973801751106351,"1h","1h"
10089,"PFC_21",51,"OID20472","P05113","IL5",0.3864,"Inflammation","B04411","20212097_SS211642","PASS",-1.0427,0.126,"Plate control","PASS","PFC",1.09126387705493,"24h","24h"
10090,"PFC_06",61,"OID20472","P05113","IL5",0.3864,"Inflammation","B04411","20212097_SS211642","PASS",-1.0427,-0.6127,"Plate control","PASS","PFC",0.653971649711789,"Baseline","Baseline"
10091,"PFC_14",62,"OID20472","P05113","IL5",0.3864,"Inflammation","B04411","20212097_SS211642","PASS",-1.0427,-0.4982,"Plate control","PASS","PFC",0.707989564109797,"1h","1h"
10092,"PFC_22",63,"OID20472","P05113","IL5",0.3864,"Inflammation","B04411","20212097_SS211642","PASS",-1.0427,0.0553,"Plate control","PASS","PFC",1.03907515043729,"24h","24h"
10093,"PFC_07",73,"OID20472","P05113","IL5",0.3864,"Inflammation","B04411","20212097_SS211642","PASS",-1.0427,-1.7607,"Plate control","PASS","PFC",0.295104944802348,"Baseline","Baseline"
10094,"PFC_15",74,"OID20472","P05113","IL5",0.3864,"Inflammation","B04411","20212097_SS211642","PASS",-1.0427,-2.0093,"Plate control","PASS","PFC",0.248393615959529,"1h","1h"
10095,"PFC_23",75,"OID20472","P05113","IL5",0.3864,"Inflammation","B04411","20212097_SS211642","PASS",-1.0427,-1.4378,"Plate control","PASS","PFC",0.369129770125767,"24h","24h"
10096,"PFC_08",85,"OID20472","P05113","IL5",0.3864,"Inflammation","B04411","20212097_SS211642","PASS",-1.0427,-0.8129,"Plate control","PASS","PFC",0.569236469635414,"Baseline","Baseline"
10097,"PFC_16",86,"OID20472","P05113","IL5",0.3864,"Inflammation","B04411","20212097_SS211642","PASS",-1.0427,-2.4206,"Plate control","PASS","PFC",0.186778460946082,"1h","1h"
10098,"PFC_24",87,"OID20472","P05113","IL5",0.3864,"Inflammation","B04411","20212097_SS211642","PASS",-1.0427,-0.8255,"Plate control","PASS","PFC",0.564286601676511,"24h","24h"
10099,"PFC_01",1,"OID20473","P01375","TNF",0,"Inflammation","B04411","20212097_SS211642","PASS",-0.9716,0.0764,"Plate control","PASS","PFC",1.05438372000904,"Baseline","Baseline"
10100,"PFC_09",2,"OID20473","P01375","TNF",0,"Inflammation","B04411","20212097_SS211642","PASS",-0.9716,0.5944,"Plate control","PASS","PFC",1.50984452828432,"Baseline","Baseline"
10101,"PFC_17",3,"OID20473","P01375","TNF",0,"Inflammation","B04411","20212097_SS211642","PASS",-0.9716,0.3168,"Plate control","PASS","PFC",1.24556473166382,"1h","1h"
10102,"PFC_25",4,"OID20473","P01375","TNF",0,"Inflammation","B04411","20212097_SS211642","PASS",-0.9716,-0.2425,"Plate control","PASS","PFC",0.845279285130392,"24h","24h"
10103,"PFC_02",13,"OID20473","P01375","TNF",0,"Inflammation","B04411","20212097_SS211642","PASS",-0.9716,0.4626,"Plate control","PASS","PFC",1.37802303089327,"Baseline","Baseline"
10104,"PFC_10",14,"OID20473","P01375","TNF",0,"Inflammation","B04411","20212097_SS211642","PASS",-0.9716,-0.0353,"Plate control","PASS","PFC",0.975828821778009,"1h","1h"
10105,"PFC_18",15,"OID20473","P01375","TNF",0,"Inflammation","B04411","20212097_SS211642","PASS",-0.9716,0.4872,"Plate control","PASS","PFC",1.40172175550869,"1h","1h"
10106,"PFC_26",16,"OID20473","P01375","TNF",0,"Inflammation","B04411","20212097_SS211642","PASS",-0.9716,0.784,"Plate control","PASS","PFC",1.72189837667781,"24h","24h"
10107,"PFC_03",25,"OID20473","P01375","TNF",0,"Inflammation","B04411","20212097_SS211642","PASS",-0.9716,-0.1144,"Plate control","PASS","PFC",0.923766414503149,"Baseline","Baseline"
10108,"PFC_11",26,"OID20473","P01375","TNF",0,"Inflammation","B04411","20212097_SS211642","PASS",-0.9716,1.0696,"Plate control","PASS","PFC",2.09885136140529,"1h","1h"
10109,"PFC_19",27,"OID20473","P01375","TNF",0,"Inflammation","B04411","20212097_SS211642","PASS",-0.9716,0.1745,"Plate control","PASS","PFC",1.12857320335302,"24h","24h"
10110,"PFC_27",28,"OID20473","P01375","TNF",0,"Inflammation","B04411","20212097_SS211642","PASS",-0.9716,0.7778,"Plate control","PASS","PFC",1.71451437463175,"24h","24h"
10111,"PFC_04",37,"OID20473","P01375","TNF",0,"Inflammation","B04411","20212097_SS211642","PASS",-0.9716,0.7409,"Plate control","PASS","PFC",1.67121807381931,"Baseline","Baseline"
10112,"PFC_12",38,"OID20473","P01375","TNF",0,"Inflammation","B04411","20212097_SS211642","PASS",-0.9716,-0.1142,"Plate control","PASS","PFC",0.923894484597229,"1h","1h"
10113,"PFC_20",39,"OID20473","P01375","TNF",0,"Inflammation","B04411","20212097_SS211642","PASS",-0.9716,0.3611,"Plate control","PASS","PFC",1.28440483406296,"24h","24h"
10114,"PFC_05",49,"OID20473","P01375","TNF",0,"Inflammation","B04411","20212097_SS211642","PASS",-0.9716,0.4168,"Plate control","PASS","PFC",1.33496322526842,"Baseline","Baseline"
10115,"PFC_13",50,"OID20473","P01375","TNF",0,"Inflammation","B04411","20212097_SS211642","PASS",-0.9716,0.4432,"Plate control","PASS","PFC",1.35961671174078,"1h","1h"
10116,"PFC_21",51,"OID20473","P01375","TNF",0,"Inflammation","B04411","20212097_SS211642","PASS",-0.9716,0.1951,"Plate control","PASS","PFC",1.14480350958112,"24h","24h"
10117,"PFC_06",61,"OID20473","P01375","TNF",0,"Inflammation","B04411","20212097_SS211642","PASS",-0.9716,0.7922,"Plate control","PASS","PFC",1.73171318082171,"Baseline","Baseline"
10118,"PFC_14",62,"OID20473","P01375","TNF",0,"Inflammation","B04411","20212097_SS211642","PASS",-0.9716,0.4445,"Plate control","PASS","PFC",1.36084240272511,"1h","1h"
10119,"PFC_22",63,"OID20473","P01375","TNF",0,"Inflammation","B04411","20212097_SS211642","PASS",-0.9716,0.6481,"Plate control","PASS","PFC",1.56710299343633,"24h","24h"
10120,"PFC_07",73,"OID20473","P01375","TNF",0,"Inflammation","B04411","20212097_SS211642","PASS",-0.9716,-0.1552,"Plate control","PASS","PFC",0.898007873929337,"Baseline","Baseline"
10121,"PFC_15",74,"OID20473","P01375","TNF",0,"Inflammation","B04411","20212097_SS211642","PASS",-0.9716,0.6538,"Plate control","PASS","PFC",1.57330676895822,"1h","1h"
10122,"PFC_23",75,"OID20473","P01375","TNF",0,"Inflammation","B04411","20212097_SS211642","PASS",-0.9716,0.3417,"Plate control","PASS","PFC",1.26724897761734,"24h","24h"
10123,"PFC_08",85,"OID20473","P01375","TNF",0,"Inflammation","B04411","20212097_SS211642","PASS",-0.9716,0.4137,"Plate control","PASS","PFC",1.33209779434044,"Baseline","Baseline"
10124,"PFC_16",86,"OID20473","P01375","TNF",0,"Inflammation","B04411","20212097_SS211642","PASS",-0.9716,0.0639,"Plate control","PASS","PFC",1.04528764387573,"1h","1h"
10125,"PFC_24",87,"OID20473","P01375","TNF",0,"Inflammation","B04411","20212097_SS211642","PASS",-0.9716,1.0315,"Plate control","PASS","PFC",2.04414849055956,"24h","24h"
10126,"PFC_01",1,"OID20474","Q92844","TANK",0.1591,"Inflammation","B04411","20212097_SS211642","PASS",0.6792,1.3442,"Plate control","PASS","PFC",2.53889371304814,"Baseline","Baseline"
10127,"PFC_09",2,"OID20474","Q92844","TANK",0.1591,"Inflammation","B04411","20212097_SS211642","PASS",0.6792,1.6324,"Plate control","PASS","PFC",3.10028318570411,"Baseline","Baseline"
10128,"PFC_17",3,"OID20474","Q92844","TANK",0.1591,"Inflammation","B04411","20212097_SS211642","PASS",0.6792,2.3031,"Plate control","PASS","PFC",4.93517074735158,"1h","1h"
10129,"PFC_25",4,"OID20474","Q92844","TANK",0.1591,"Inflammation","B04411","20212097_SS211642","PASS",0.6792,1.9171,"Plate control","PASS","PFC",3.77663144367246,"24h","24h"
10130,"PFC_02",13,"OID20474","Q92844","TANK",0.1591,"Inflammation","B04411","20212097_SS211642","PASS",0.6792,1.4179,"Plate control","PASS","PFC",2.67196294400317,"Baseline","Baseline"
10131,"PFC_10",14,"OID20474","Q92844","TANK",0.1591,"Inflammation","B04411","20212097_SS211642","PASS",0.6792,0.956,"Plate control","PASS","PFC",1.93992382068347,"1h","1h"
10132,"PFC_18",15,"OID20474","Q92844","TANK",0.1591,"Inflammation","B04411","20212097_SS211642","PASS",0.6792,1.9605,"Plate control","PASS","PFC",3.89196840940255,"1h","1h"
10133,"PFC_26",16,"OID20474","Q92844","TANK",0.1591,"Inflammation","B04411","20212097_SS211642","PASS",0.6792,1.885,"Plate control","PASS","PFC",3.69352924291758,"24h","24h"
10134,"PFC_03",25,"OID20474","Q92844","TANK",0.1591,"Inflammation","B04411","20212097_SS211642","PASS",0.6792,0.5444,"Plate control","PASS","PFC",1.45841368089822,"Baseline","Baseline"
10135,"PFC_11",26,"OID20474","Q92844","TANK",0.1591,"Inflammation","B04411","20212097_SS211642","PASS",0.6792,1.5081,"Plate control","PASS","PFC",2.84435196979666,"1h","1h"
10136,"PFC_19",27,"OID20474","Q92844","TANK",0.1591,"Inflammation","B04411","20212097_SS211642","PASS",0.6792,1.5254,"Plate control","PASS","PFC",2.87866518484946,"24h","24h"
10137,"PFC_27",28,"OID20474","Q92844","TANK",0.1591,"Inflammation","B04411","20212097_SS211642","PASS",0.6792,2.6668,"Plate control","PASS","PFC",6.35019106302476,"24h","24h"
10138,"PFC_04",37,"OID20474","Q92844","TANK",0.1591,"Inflammation","B04411","20212097_SS211642","PASS",0.6792,1.4512,"Plate control","PASS","PFC",2.73435393955503,"Baseline","Baseline"
10139,"PFC_12",38,"OID20474","Q92844","TANK",0.1591,"Inflammation","B04411","20212097_SS211642","PASS",0.6792,-0.2395,"Plate control","PASS","PFC",0.847038822784028,"1h","1h"
10140,"PFC_20",39,"OID20474","Q92844","TANK",0.1591,"Inflammation","B04411","20212097_SS211642","PASS",0.6792,2.3111,"Plate control","PASS","PFC",4.9626131610724,"24h","24h"
10141,"PFC_05",49,"OID20474","Q92844","TANK",0.1591,"Inflammation","B04411","20212097_SS211642","PASS",0.6792,1.2375,"Plate control","PASS","PFC",2.35789585846562,"Baseline","Baseline"
10142,"PFC_13",50,"OID20474","Q92844","TANK",0.1591,"Inflammation","B04411","20212097_SS211642","PASS",0.6792,1.9569,"Plate control","PASS","PFC",3.88226877138643,"1h","1h"
10143,"PFC_21",51,"OID20474","Q92844","TANK",0.1591,"Inflammation","B04411","20212097_SS211642","PASS",0.6792,0.6129,"Plate control","PASS","PFC",1.52933026911241,"24h","24h"
10144,"PFC_06",61,"OID20474","Q92844","TANK",0.1591,"Inflammation","B04411","20212097_SS211642","PASS",0.6792,2.1227,"Plate control","PASS","PFC",4.35508235326306,"Baseline","Baseline"
10145,"PFC_14",62,"OID20474","Q92844","TANK",0.1591,"Inflammation","B04411","20212097_SS211642","PASS",0.6792,1.9091,"Plate control","PASS","PFC",3.75574730880551,"1h","1h"
10146,"PFC_22",63,"OID20474","Q92844","TANK",0.1591,"Inflammation","B04411","20212097_SS211642","PASS",0.6792,1.2048,"Plate control","PASS","PFC",2.30505312323263,"24h","24h"
10147,"PFC_07",73,"OID20474","Q92844","TANK",0.1591,"Inflammation","B04411","20212097_SS211642","PASS",0.6792,1.6569,"Plate control","PASS","PFC",3.1533821128576,"Baseline","Baseline"
10148,"PFC_15",74,"OID20474","Q92844","TANK",0.1591,"Inflammation","B04411","20212097_SS211642","PASS",0.6792,1.8318,"Plate control","PASS","PFC",3.55980940443158,"1h","1h"
10149,"PFC_23",75,"OID20474","Q92844","TANK",0.1591,"Inflammation","B04411","20212097_SS211642","PASS",0.6792,1.3381,"Plate control","PASS","PFC",2.52818143101548,"24h","24h"
10150,"PFC_08",85,"OID20474","Q92844","TANK",0.1591,"Inflammation","B04411","20212097_SS211642","PASS",0.6792,1.1456,"Plate control","PASS","PFC",2.21238121881941,"Baseline","Baseline"
10151,"PFC_16",86,"OID20474","Q92844","TANK",0.1591,"Inflammation","B04411","20212097_SS211642","PASS",0.6792,1.8264,"Plate control","PASS","PFC",3.5465099618299,"1h","1h"
10152,"PFC_24",87,"OID20474","Q92844","TANK",0.1591,"Inflammation","B04411","20212097_SS211642","PASS",0.6792,2.222,"Plate control","PASS","PFC",4.66539747915387,"24h","24h"
10153,"PFC_01",1,"OID20475","O43597","SPRY2",0,"Inflammation","B04411","20212097_SS211642","PASS",0.0779,2.78,"Plate control","PASS","PFC",6.86852349150203,"Baseline","Baseline"
10154,"PFC_09",2,"OID20475","O43597","SPRY2",0,"Inflammation","B04411","20212097_SS211642","PASS",0.0779,3.0793,"Plate control","PASS","PFC",8.45204237289333,"Baseline","Baseline"
10155,"PFC_17",3,"OID20475","O43597","SPRY2",0,"Inflammation","B04411","20212097_SS211642","PASS",0.0779,4.4905,"Plate control","PASS","PFC",22.4789072575504,"1h","1h"
10156,"PFC_25",4,"OID20475","O43597","SPRY2",0,"Inflammation","B04411","20212097_SS211642","PASS",0.0779,3.7517,"Plate control","PASS","PFC",13.4702059159078,"24h","24h"
10157,"PFC_02",13,"OID20475","O43597","SPRY2",0,"Inflammation","B04411","20212097_SS211642","PASS",0.0779,2.8301,"Plate control","PASS","PFC",7.11123434545357,"Baseline","Baseline"
10158,"PFC_10",14,"OID20475","O43597","SPRY2",0,"Inflammation","B04411","20212097_SS211642","PASS",0.0779,2.4339,"Plate control","PASS","PFC",5.40352178157276,"1h","1h"
10159,"PFC_18",15,"OID20475","O43597","SPRY2",0,"Inflammation","B04411","20212097_SS211642","PASS",0.0779,3.7521,"Plate control","PASS","PFC",13.4739411678006,"1h","1h"
10160,"PFC_26",16,"OID20475","O43597","SPRY2",0,"Inflammation","B04411","20212097_SS211642","PASS",0.0779,4.3442,"Plate control","PASS","PFC",20.3111497043851,"24h","24h"
10161,"PFC_03",25,"OID20475","O43597","SPRY2",0,"Inflammation","B04411","20212097_SS211642","PASS",0.0779,1.805,"Plate control","PASS","PFC",3.49429158366678,"Baseline","Baseline"
10162,"PFC_11",26,"OID20475","O43597","SPRY2",0,"Inflammation","B04411","20212097_SS211642","PASS",0.0779,3.9346,"Plate control","PASS","PFC",15.290884960202,"1h","1h"
10163,"PFC_19",27,"OID20475","O43597","SPRY2",0,"Inflammation","B04411","20212097_SS211642","PASS",0.0779,3.1438,"Plate control","PASS","PFC",8.83849051850912,"24h","24h"
10164,"PFC_27",28,"OID20475","O43597","SPRY2",0,"Inflammation","B04411","20212097_SS211642","PASS",0.0779,5.4513,"Plate control","PASS","PFC",43.7526956335394,"24h","24h"
10165,"PFC_04",37,"OID20475","O43597","SPRY2",0,"Inflammation","B04411","20212097_SS211642","PASS",0.0779,4.0403,"Plate control","PASS","PFC",16.4532422263344,"Baseline","Baseline"
10166,"PFC_12",38,"OID20475","O43597","SPRY2",0,"Inflammation","B04411","20212097_SS211642","PASS",0.0779,2.2558,"Plate control","PASS","PFC",4.77599060951838,"1h","1h"
10167,"PFC_20",39,"OID20475","O43597","SPRY2",0,"Inflammation","B04411","20212097_SS211642","PASS",0.0779,4.1345,"Plate control","PASS","PFC",17.5633969561319,"24h","24h"
10168,"PFC_05",49,"OID20475","O43597","SPRY2",0,"Inflammation","B04411","20212097_SS211642","PASS",0.0779,2.3863,"Plate control","PASS","PFC",5.22814806537327,"Baseline","Baseline"
10169,"PFC_13",50,"OID20475","O43597","SPRY2",0,"Inflammation","B04411","20212097_SS211642","PASS",0.0779,4.1962,"Plate control","PASS","PFC",18.3308273838059,"1h","1h"
10170,"PFC_21",51,"OID20475","O43597","SPRY2",0,"Inflammation","B04411","20212097_SS211642","PASS",0.0779,2.7007,"Plate control","PASS","PFC",6.50117279442912,"24h","24h"
10171,"PFC_06",61,"OID20475","O43597","SPRY2",0,"Inflammation","B04411","20212097_SS211642","PASS",0.0779,3.6571,"Plate control","PASS","PFC",12.6152771789768,"Baseline","Baseline"
10172,"PFC_14",62,"OID20475","O43597","SPRY2",0,"Inflammation","B04411","20212097_SS211642","PASS",0.0779,4.0604,"Plate control","PASS","PFC",16.6840773407116,"1h","1h"
10173,"PFC_22",63,"OID20475","O43597","SPRY2",0,"Inflammation","B04411","20212097_SS211642","PASS",0.0779,3.8469,"Plate control","PASS","PFC",14.3890555864658,"24h","24h"
10174,"PFC_07",73,"OID20475","O43597","SPRY2",0,"Inflammation","B04411","20212097_SS211642","PASS",0.0779,4.367,"Plate control","PASS","PFC",20.6346920104942,"Baseline","Baseline"
10175,"PFC_15",74,"OID20475","O43597","SPRY2",0,"Inflammation","B04411","20212097_SS211642","PASS",0.0779,2.2547,"Plate control","PASS","PFC",4.77235048655436,"1h","1h"
10176,"PFC_23",75,"OID20475","O43597","SPRY2",0,"Inflammation","B04411","20212097_SS211642","PASS",0.0779,4.0593,"Plate control","PASS","PFC",16.6713612158221,"24h","24h"
10177,"PFC_08",85,"OID20475","O43597","SPRY2",0,"Inflammation","B04411","20212097_SS211642","PASS",0.0779,2.8879,"Plate control","PASS","PFC",7.40192234436066,"Baseline","Baseline"
10178,"PFC_16",86,"OID20475","O43597","SPRY2",0,"Inflammation","B04411","20212097_SS211642","PASS",0.0779,3.5592,"Plate control","PASS","PFC",11.7876154837568,"1h","1h"
10179,"PFC_24",87,"OID20475","O43597","SPRY2",0,"Inflammation","B04411","20212097_SS211642","PASS",0.0779,4.9248,"Plate control","PASS","PFC",30.3747367459033,"24h","24h"
10180,"PFC_01",1,"OID20476","P13693","TPT1",0.9545,"Inflammation","B04411","20212097_SS211642","PASS",1.0508,0.2556,"Plate control","PASS","PFC",1.19383214063099,"Baseline","Baseline"
10181,"PFC_09",2,"OID20476","P13693","TPT1",0.9545,"Inflammation","B04411","20212097_SS211642","PASS",1.0508,0.8631,"Plate control","PASS","PFC",1.81894257872399,"Baseline","Baseline"
10182,"PFC_17",3,"OID20476","P13693","TPT1",0.9545,"Inflammation","B04411","20212097_SS211642","PASS",1.0508,0.8686,"Plate control","PASS","PFC",1.82589018558348,"1h","1h"
10183,"PFC_25",4,"OID20476","P13693","TPT1",0.9545,"Inflammation","B04411","20212097_SS211642","PASS",1.0508,0.7322,"Plate control","PASS","PFC",1.66117031932489,"24h","24h"
10184,"PFC_02",13,"OID20476","P13693","TPT1",0.9545,"Inflammation","B04411","20212097_SS211642","PASS",1.0508,0.5191,"Plate control","PASS","PFC",1.43306097914831,"Baseline","Baseline"
10185,"PFC_10",14,"OID20476","P13693","TPT1",0.9545,"Inflammation","B04411","20212097_SS211642","PASS",1.0508,0.4158,"Plate control","PASS","PFC",1.33403821989215,"1h","1h"
10186,"PFC_18",15,"OID20476","P13693","TPT1",0.9545,"Inflammation","B04411","20212097_SS211642","PASS",1.0508,0.8288,"Plate control","PASS","PFC",1.77620733999438,"1h","1h"
10187,"PFC_26",16,"OID20476","P13693","TPT1",0.9545,"Inflammation","B04411","20212097_SS211642","PASS",1.0508,0.931,"Plate control","PASS","PFC",1.90659709057632,"24h","24h"
10188,"PFC_03",25,"OID20476","P13693","TPT1",0.9545,"Inflammation","B04411","20212097_SS211642","PASS",1.0508,0.3331,"Plate control","PASS","PFC",1.25971729387035,"Baseline","Baseline"
10189,"PFC_11",26,"OID20476","P13693","TPT1",0.9545,"Inflammation","B04411","20212097_SS211642","PASS",1.0508,-0.1904,"Plate control","PASS","PFC",0.876362708292673,"1h","1h"
10190,"PFC_19",27,"OID20476","P13693","TPT1",0.9545,"Inflammation","B04411","20212097_SS211642","PASS",1.0508,0.1519,"Plate control","PASS","PFC",1.11103171513595,"24h","24h"
10191,"PFC_27",28,"OID20476","P13693","TPT1",0.9545,"Inflammation","B04411","20212097_SS211642","PASS",1.0508,-0.3351,"Plate control","PASS","PFC",0.79272918690581,"24h","24h"
10192,"PFC_04",37,"OID20476","P13693","TPT1",0.9545,"Inflammation","B04411","20212097_SS211642","PASS",1.0508,0.8777,"Plate control","PASS","PFC",1.83744364156523,"Baseline","Baseline"

Supplemental table 1_Olink proximity extension assay (PEA) data

,"SampleID","Index","OlinkID","UniProt","Assay","MissingFreq","Panel","Panel_Lot_Nr","PlateID","QC_Warning","LOD","NPX","Normalization","Assay_Warning","condition","Value","group","alt_group"
10193,"PFC_12",38,"OID20476","P13693","TPT1",0.9545,"Inflammation","B04411","20212097_SS211642","PASS",1.0508,0.4562,"Plate control","PASS","PFC",1.37192346438457,"1h","1h"
10194,"PFC_20",39,"OID20476","P13693","TPT1",0.9545,"Inflammation","B04411","20212097_SS211642","PASS",1.0508,-0.173,"Plate control","PASS","PFC",0.88699630515215,"24h","24h"
10195,"PFC_05",49,"OID20476","P13693","TPT1",0.9545,"Inflammation","B04411","20212097_SS211642","PASS",1.0508,-0.0397,"Plate control","PASS","PFC",0.972857226353255,"Baseline","Baseline"
10196,"PFC_13",50,"OID20476","P13693","TPT1",0.9545,"Inflammation","B04411","20212097_SS211642","PASS",1.0508,0.9866,"Plate control","PASS","PFC",1.98150965922592,"1h","1h"
10197,"PFC_21",51,"OID20476","P13693","TPT1",0.9545,"Inflammation","B04411","20212097_SS211642","PASS",1.0508,0.6216,"Plate control","PASS","PFC",1.53858057590284,"24h","24h"
10198,"PFC_06",61,"OID20476","P13693","TPT1",0.9545,"Inflammation","B04411","20212097_SS211642","PASS",1.0508,0.7238,"Plate control","PASS","PFC",1.65152636383255,"Baseline","Baseline"
10199,"PFC_14",62,"OID20476","P13693","TPT1",0.9545,"Inflammation","B04411","20212097_SS211642","PASS",1.0508,0.647,"Plate control","PASS","PFC",1.56590859251309,"1h","1h"
10200,"PFC_22",63,"OID20476","P13693","TPT1",0.9545,"Inflammation","B04411","20212097_SS211642","PASS",1.0508,1.0298,"Plate control","PASS","PFC",2.04174118636491,"24h","24h"
10201,"PFC_07",73,"OID20476","P13693","TPT1",0.9545,"Inflammation","B04411","20212097_SS211642","PASS",1.0508,0.5856,"Plate control","PASS","PFC",1.50066296770718,"Baseline","Baseline"
10202,"PFC_15",74,"OID20476","P13693","TPT1",0.9545,"Inflammation","B04411","20212097_SS211642","PASS",1.0508,0.2143,"Plate control","PASS","PFC",1.16014087393723,"1h","1h"
10203,"PFC_23",75,"OID20476","P13693","TPT1",0.9545,"Inflammation","B04411","20212097_SS211642","PASS",1.0508,0.3271,"Plate control","PASS","PFC",1.25448915606961,"24h","24h"
10204,"PFC_08",85,"OID20476","P13693","TPT1",0.9545,"Inflammation","B04411","20212097_SS211642","PASS",1.0508,0.2266,"Plate control","PASS","PFC",1.17007418304877,"Baseline","Baseline"
10205,"PFC_16",86,"OID20476","P13693","TPT1",0.9545,"Inflammation","B04411","20212097_SS211642","PASS",1.0508,1.2277,"Plate control","PASS","PFC",2.34193332045717,"1h","1h"
10206,"PFC_24",87,"OID20476","P13693","TPT1",0.9545,"Inflammation","B04411","20212097_SS211642","PASS",1.0508,0.6729,"Plate control","PASS","PFC",1.59427444256762,"24h","24h"
10207,"PFC_01",1,"OID20493","P18564","ITGB6",0,"Inflammation","B04411","20212097_SS211642","PASS",-1.3374,0.5951,"Plate control","PASS","PFC",1.51057728717286,"Baseline","Baseline"
10208,"PFC_09",2,"OID20493","P18564","ITGB6",0,"Inflammation","B04411","20212097_SS211642","PASS",-1.3374,0.8515,"Plate control","PASS","PFC",1.80437599747994,"Baseline","Baseline"
10209,"PFC_17",3,"OID20493","P18564","ITGB6",0,"Inflammation","B04411","20212097_SS211642","PASS",-1.3374,0.1344,"Plate control","PASS","PFC",1.09763622477592,"1h","1h"
10210,"PFC_25",4,"OID20493","P18564","ITGB6",0,"Inflammation","B04411","20212097_SS211642","PASS",-1.3374,0.5336,"Plate control","PASS","PFC",1.44753677432299,"24h","24h"
10211,"PFC_02",13,"OID20493","P18564","ITGB6",0,"Inflammation","B04411","20212097_SS211642","PASS",-1.3374,0.6958,"Plate control","PASS","PFC",1.61978238218052,"Baseline","Baseline"
10212,"PFC_10",14,"OID20493","P18564","ITGB6",0,"Inflammation","B04411","20212097_SS211642","PASS",-1.3374,0.8566,"Plate control","PASS","PFC",1.81076584553399,"1h","1h"
10213,"PFC_18",15,"OID20493","P18564","ITGB6",0,"Inflammation","B04411","20212097_SS211642","PASS",-1.3374,0.8737,"Plate control","PASS","PFC",1.832356221967,"1h","1h"
10214,"PFC_26",16,"OID20493","P18564","ITGB6",0,"Inflammation","B04411","20212097_SS211642","PASS",-1.3374,0.3754,"Plate control","PASS","PFC",1.29719916477363,"24h","24h"
10215,"PFC_03",25,"OID20493","P18564","ITGB6",0,"Inflammation","B04411","20212097_SS211642","PASS",-1.3374,0.1988,"Plate control","PASS","PFC",1.14774329182041,"Baseline","Baseline"
10216,"PFC_11",26,"OID20493","P18564","ITGB6",0,"Inflammation","B04411","20212097_SS211642","PASS",-1.3374,1.0075,"Plate control","PASS","PFC",2.01042428008295,"1h","1h"
10217,"PFC_19",27,"OID20493","P18564","ITGB6",0,"Inflammation","B04411","20212097_SS211642","PASS",-1.3374,0.9105,"Plate control","PASS","PFC",1.87969683883489,"24h","24h"
10218,"PFC_27",28,"OID20493","P18564","ITGB6",0,"Inflammation","B04411","20212097_SS211642","PASS",-1.3374,1.1884,"Plate control","PASS","PFC",2.27899853942103,"24h","24h"
10219,"PFC_04",37,"OID20493","P18564","ITGB6",0,"Inflammation","B04411","20212097_SS211642","PASS",-1.3374,1.3561,"Plate control","PASS","PFC",2.55992226182897,"Baseline","Baseline"
10220,"PFC_12",38,"OID20493","P18564","ITGB6",0,"Inflammation","B04411","20212097_SS211642","PASS",-1.3374,0.2032,"Plate control","PASS","PFC",1.15124907727626,"1h","1h"
10221,"PFC_20",39,"OID20493","P18564","ITGB6",0,"Inflammation","B04411","20212097_SS211642","PASS",-1.3374,0.4679,"Plate control","PASS","PFC",1.38309475686521,"24h","24h"
10222,"PFC_05",49,"OID20493","P18564","ITGB6",0,"Inflammation","B04411","20212097_SS211642","PASS",-1.3374,0.8082,"Plate control","PASS","PFC",1.75102538622551,"Baseline","Baseline"
10223,"PFC_13",50,"OID20493","P18564","ITGB6",0,"Inflammation","B04411","20212097_SS211642","PASS",-1.3374,0.7999,"Plate control","PASS","PFC",1.74098044684104,"1h","1h"
10224,"PFC_21",51,"OID20493","P18564","ITGB6",0,"Inflammation","B04411","20212097_SS211642","PASS",-1.3374,0.0163,"Plate control","PASS","PFC",1.01136236587851,"24h","24h"
10225,"PFC_06",61,"OID20493","P18564","ITGB6",0,"Inflammation","B04411","20212097_SS211642","PASS",-1.3374,0.3337,"Plate control","PASS","PFC",1.26024130452208,"Baseline","Baseline"
10226,"PFC_14",62,"OID20493","P18564","ITGB6",0,"Inflammation","B04411","20212097_SS211642","PASS",-1.3374,0.8716,"Plate control","PASS","PFC",1.82969096786864,"1h","1h"
10227,"PFC_22",63,"OID20493","P18564","ITGB6",0,"Inflammation","B04411","20212097_SS211642","PASS",-1.3374,0.9831,"Plate control","PASS","PFC",1.97670831323241,"24h","24h"
10228,"PFC_07",73,"OID20493","P18564","ITGB6",0,"Inflammation","B04411","20212097_SS211642","PASS",-1.3374,0.486,"Plate control","PASS","PFC",1.4005563208872,"Baseline","Baseline"
10229,"PFC_15",74,"OID20493","P18564","ITGB6",0,"Inflammation","B04411","20212097_SS211642","PASS",-1.3374,0.1368,"Plate control","PASS","PFC",1.09946372071362,"1h","1h"
10230,"PFC_23",75,"OID20493","P18564","ITGB6",0,"Inflammation","B04411","20212097_SS211642","PASS",-1.3374,0.7041,"Plate control","PASS","PFC",1.62912804477802,"24h","24h"
10231,"PFC_08",85,"OID20493","P18564","ITGB6",0,"Inflammation","B04411","20212097_SS211642","PASS",-1.3374,0.1852,"Plate control","PASS","PFC",1.13697458045622,"Baseline","Baseline"
10232,"PFC_16",86,"OID20493","P18564","ITGB6",0,"Inflammation","B04411","20212097_SS211642","PASS",-1.3374,0.2103,"Plate control","PASS","PFC",1.1569287354611,"1h","1h"
10233,"PFC_24",87,"OID20493","P18564","ITGB6",0,"Inflammation","B04411","20212097_SS211642","PASS",-1.3374,0.0822,"Plate control","PASS","PFC",1.05863114214144,"24h","24h"
10234,"PFC_01",1,"OID20494","P01591","JCHAIN",0.8409,"Inflammation","B04411","20212097_SS211642","PASS",1.2457,1.0427,"Plate control","PASS","PFC",2.06007948121211,"Baseline","Baseline"
10235,"PFC_09",2,"OID20494","P01591","JCHAIN",0.8409,"Inflammation","B04411","20212097_SS211642","PASS",1.2457,0.0448,"Plate control","PASS","PFC",1.03154016755701,"Baseline","Baseline"
10236,"PFC_17",3,"OID20494","P01591","JCHAIN",0.8409,"Inflammation","B04411","20212097_SS211642","PASS",1.2457,0.6593,"Plate control","PASS","PFC",1.57931614881878,"1h","1h"
10237,"PFC_25",4,"OID20494","P01591","JCHAIN",0.8409,"Inflammation","B04411","20212097_SS211642","PASS",1.2457,0.63,"Plate control","PASS","PFC",1.54756499354239,"24h","24h"
10238,"PFC_02",13,"OID20494","P01591","JCHAIN",0.8409,"Inflammation","B04411","20212097_SS211642","PASS",1.2457,0.0975,"Plate control","PASS","PFC",1.06991782890028,"Baseline","Baseline"
10239,"PFC_10",14,"OID20494","P01591","JCHAIN",0.8409,"Inflammation","B04411","20212097_SS211642","PASS",1.2457,0.6958,"Plate control","PASS","PFC",1.61978238218052,"1h","1h"
10240,"PFC_18",15,"OID20494","P01591","JCHAIN",0.8409,"Inflammation","B04411","20212097_SS211642","PASS",1.2457,-1.1165,"Plate control","PASS","PFC",0.461211374942808,"1h","1h"
10241,"PFC_26",16,"OID20494","P01591","JCHAIN",0.8409,"Inflammation","B04411","20212097_SS211642","PASS",1.2457,1.4045,"Plate control","PASS","PFC",2.647260191318,"24h","24h"
10242,"PFC_03",25,"OID20494","P01591","JCHAIN",0.8409,"Inflammation","B04411","20212097_SS211642","PASS",1.2457,0.0839,"Plate control","PASS","PFC",1.05987931561518,"Baseline","Baseline"
10243,"PFC_11",26,"OID20494","P01591","JCHAIN",0.8409,"Inflammation","B04411","20212097_SS211642","PASS",1.2457,-0.3483,"Plate control","PASS","PFC",0.785509157646014,"1h","1h"
10244,"PFC_19",27,"OID20494","P01591","JCHAIN",0.8409,"Inflammation","B04411","20212097_SS211642","PASS",1.2457,0.0293,"Plate control","PASS","PFC",1.0205168476987З,"24h","24h"
10245,"PFC_27",28,"OID20494","P01591","JCHAIN",0.8409,"Inflammation","B04411","20212097_SS211642","PASS",1.2457,-0.0191,"Plate control","PASS","PFC",0.986848140414499,"24h","24h"
10246,"PFC_04",37,"OID20494","P01591","JCHAIN",0.8409,"Inflammation","B04411","20212097_SS211642","PASS",1.2457,-0.3136,"Plate control","PASS","PFC",0.804631431220942,"Baseline","Baseline"
10247,"PFC_12",38,"OID20494","P01591","JCHAIN",0.8409,"Inflammation","B04411","20212097_SS211642","PASS",1.2457,1.3469,"Plate control","PASS","PFC",2.54364969501504,"1h","1h"
10248,"PFC_20",39,"OID20494","P01591","JCHAIN",0.8409,"Inflammation","B04411","20212097_SS211642","PASS",1.2457,-0.5141,"Plate control","PASS","PFC",0.700229622542565,"24h","24h"
10249,"PFC_05",49,"OID20494","P01591","JCHAIN",0.8409,"Inflammation","B04411","20212097_SS211642","PASS",1.2457,0.8757,"Plate control","PASS","PFC",1.83489816860112,"Baseline","Baseline"
10250,"PFC_13",50,"OID20494","P01591","JCHAIN",0.8409,"Inflammation","B04411","20212097_SS211642","PASS",1.2457,-0.2511,"Plate control","PASS","PFC",0.840255508141077,"1h","1h"
10251,"PFC_21",51,"OID20494","P01591","JCHAIN",0.8409,"Inflammation","B04411","20212097_SS211642","PASS",1.2457,1.5544,"Plate control","PASS","PFC",2.93711550051822,"24h","24h"
10252,"PFC_06",61,"OID20494","P01591","JCHAIN",0.8409,"Inflammation","B04411","20212097_SS211642","PASS",1.2457,0.7284,"Plate control","PASS","PFC",1.65680062166565,"Baseline","Baseline"
10253,"PFC_14",62,"OID20494","P01591","JCHAIN",0.8409,"Inflammation","B04411","20212097_SS211642","PASS",1.2457,1.2131,"Plate control","PASS","PFC",2.31835259419616,"1h","1h"
10254,"PFC_22",63,"OID20494","P01591","JCHAIN",0.8409,"Inflammation","B04411","20212097_SS211642","PASS",1.2457,-0.0778,"Plate control","PASS","PFC",0.947501413081701,"24h","24h"
10255,"PFC_07",73,"OID20494","P01591","JCHAIN",0.8409,"Inflammation","B04411","20212097_SS211642","PASS",1.2457,0.8335,"Plate control","PASS","PFC",1.78200328948254,"Baseline","Baseline"
10256,"PFC_15",74,"OID20494","P01591","JCHAIN",0.8409,"Inflammation","B04411","20212097_SS211642","PASS",1.2457,-0.1147,"Plate control","PASS","PFC",0.923574342648239,"1h","1h"
10257,"PFC_23",75,"OID20494","P01591","JCHAIN",0.8409,"Inflammation","B04411","20212097_SS211642","PASS",1.2457,0.8847,"Plate control","PASS","PFC",1.84638063747663,"24h","24h"
10258,"PFC_08",85,"OID20494","P01591","JCHAIN",0.8409,"Inflammation","B04411","20212097_SS211642","PASS",1.2457,0.759,"Plate control","PASS","PFC",1.69231719320058,"Baseline","Baseline"
10259,"PFC_16",86,"OID20494","P01591","JCHAIN",0.8409,"Inflammation","B04411","20212097_SS211642","PASS",1.2457,0.9589,"Plate control","PASS","PFC",1.94382723545623,"1h","1h"
10260,"PFC_24",87,"OID20494","P01591","JCHAIN",0.8409,"Inflammation","B04411","20212097_SS211642","PASS",1.2457,-1.2805,"Plate control","PASS","PFC",0.411652815914091,"24h","24h"
10261,"PFC_01",1,"OID20495","P01579","IFNG",0.1818,"Inflammation","B04411","20212097_SS211642","PASS",-1.9087,-0.6084,"Plate control","PASS","PFC",0.6559237414056,"Baseline","Baseline"
10262,"PFC_09",2,"OID20495","P01579","IFNG",0.1818,"Inflammation","B04411","20212097_SS211642","PASS",-1.9087,0.2273,"Plate control","PASS","PFC",1.17064204433635,"Baseline","Baseline"
10263,"PFC_17",3,"OID20495","P01579","IFNG",0.1818,"Inflammation","B04411","20212097_SS211642","PASS",-1.9087,-2.7729,"Plate control","PASS","PFC",0.146309971267264,"1h","1h"
10264,"PFC_25",4,"OID20495","P01579","IFNG",0.1818,"Inflammation","B04411","20212097_SS211642","PASS",-1.9087,-0.7101,"Plate control","PASS","PFC",0.611277766831487,"24h","24h"
10265,"PFC_02",13,"OID20495","P01579","IFNG",0.1818,"Inflammation","B04411","20212097_SS211642","PASS",-1.9087,1.0472,"Plate control","PASS","PFC",2.06651523535371,"Baseline","Baseline"
10266,"PFC_10",14,"OID20495","P01579","IFNG",0.1818,"Inflammation","B04411","20212097_SS211642","PASS",-1.9087,0.4021,"Plate control","PASS","PFC",1.32142999703159,"1h","1h"
10267,"PFC_18",15,"OID20495","P01579","IFNG",0.1818,"Inflammation","B04411","20212097_SS211642","PASS",-1.9087,-0.5933,"Plate control","PASS","PFC",0.662825035226491,"1h","1h"
10268,"PFC_26",16,"OID20495","P01579","IFNG",0.1818,"Inflammation","B04411","20212097_SS211642","PASS",-1.9087,-3.3367,"Plate control","PASS","PFC",0.0989813136795947,"24h","24h"
10269,"PFC_03",25,"OID20495","P01579","IFNG",0.1818,"Inflammation","B04411","20212097_SS211642","PASS",-1.9087,-1.3563,"Plate control","PASS","PFC",0.390582712249293,"Baseline","Baseline"
10270,"PFC_11",26,"OID20495","P01579","IFNG",0.1818,"Inflammation","B04411","20212097_SS211642","PASS",-1.9087,0.3941,"Plate control","PASS","PFC",1.31412271203785,"1h","1h"
10271,"PFC_19",27,"OID20495","P01579","IFNG",0.1818,"Inflammation","B04411","20212097_SS211642","PASS",-1.9087,-0.5267,"Plate control","PASS","PFC",0.694140686999278,"24h","24h"
10272,"PFC_27",28,"OID20495","P01579","IFNG",0.1818,"Inflammation","B04411","20212097_SS211642","PASS",-1.9087,0.5953,"Plate control","PASS","PFC",1.51078671216629,"24h","24h"
10273,"PFC_04",37,"OID20495","P01579","IFNG",0.1818,"Inflammation","B04411","20212097_SS211642","PASS",-1.9087,-0.3686,"Plate control","PASS","PFC",0.774533744438288,"Baseline","Baseline"
10274,"PFC_12",38,"OID20495","P01579","IFNG",0.1818,"Inflammation","B04411","20212097_SS211642","PASS",-1.9087,-1.875,"Plate control","PASS","PFC",0.272626933166314,"1h","1h"
10275,"PFC_20",39,"OID20495","P01579","IFNG",0.1818,"Inflammation","B04411","20212097_SS211642","PASS",-1.9087,-0.6327,"Plate control","PASS","PFC",0.644968228757242,"24h","24h"
10276,"PFC_05",49,"OID20495","P01579","IFNG",0.1818,"Inflammation","B04411","20212097_SS211642","PASS",-1.9087,-0.6873,"Plate control","PASS","PFC",0.621014991027671,"Baseline","Baseline"
10277,"PFC_13",50,"OID20495","P01579","IFNG",0.1818,"Inflammation","B04411","20212097_SS211642","PASS",-1.9087,-0.029,"Plate control","PASS","PFC",0.980099415341871,"1h","1h"
10278,"PFC_21",51,"OID20495","P01579","IFNG",0.1818,"Inflammation","B04411","20212097_SS211642","PASS",-1.9087,-1.7858,"Plate control","PASS","PFC",0.290015115471636,"24h","24h"
10279,"PFC_06",61,"OID20495","P01579","IFNG",0.1818,"Inflammation","B04411","20212097_SS211642","PASS",-1.9087,-0.5288,"Plate control","PASS","PFC",0.693131024528259,"Baseline","Baseline"
10280,"PFC_14",62,"OID20495","P01579","IFNG",0.1818,"Inflammation","B04411","20212097_SS211642","PASS",-1.9087,-0.6431,"Plate control","PASS","PFC",0.640335544448869,"1h","1h"
10281,"PFC_22",63,"OID20495","P01579","IFNG",0.1818,"Inflammation","B04411","20212097_SS211642","PASS",-1.9087,-0.438,"Plate control","PASS","PFC",0.738157202841417,"24h","24h"
10282,"PFC_07",73,"OID20495","P01579","IFNG",0.1818,"Inflammation","B04411","20212097_SS211642","PASS",-1.9087,-1.0218,"Plate control","PASS","PFC",0.492501491919627,"Baseline","Baseline"
10283,"PFC_15",74,"OID20495","P01579","IFNG",0.1818,"Inflammation","B04411","20212097_SS211642","PASS",-1.9087,-0.9004,"Plate control","PASS","PFC",0.535738172512964,"1h","1h"
10284,"PFC_23",75,"OID20495","P01579","IFNG",0.1818,"Inflammation","B04411","20212097_SS211642","PASS",-1.9087,0.0808,"Plate control","PASS","PFC",1.05760433836232,"24h","24h"
10285,"PFC_08",85,"OID20495","P01579","IFNG",0.1818,"Inflammation","B04411","20212097_SS211642","PASS",-1.9087,-1.4603,"Plate control","PASS","PFC",0.36341755091707,"Baseline","Baseline"
10286,"PFC_16",86,"OID20495","P01579","IFNG",0.1818,"Inflammation","B04411","20212097_SS211642","PASS",-1.9087,-0.8129,"Plate control","PASS","PFC",0.569236469635414,"1h","1h"
10287,"PFC_24",87,"OID20495","P01579","IFNG",0.1818,"Inflammation","B04411","20212097_SS211642","PASS",-1.9087,-0.112,"Plate control","PASS","PFC",0.925304428037852,"24h","24h"
10288,"PFC_01",1,"OID20496","Q13291","SLAMF1",0.0682,"Inflammation","B04411","20212097_SS211642","PASS",-1.0314,0.6571,"Plate control","PASS","PFC",1.5769096473721,"Baseline","Baseline"
10289,"PFC_09",2,"OID20496","Q13291","SLAMF1",0.0682,"Inflammation","B04411","20212097_SS211642","PASS",-1.0314,-0.7951,"Plate control","PASS","PFC",0.576303225719362,"Baseline","Baseline"
10290,"PFC_17",3,"OID20496","Q13291","SLAMF1",0.0682,"Inflammation","B04411","20212097_SS211642","PASS",-1.0314,0.1672,"Plate control","PASS","PFC",1.12287707510006,"1h","1h"
10291,"PFC_25",4,"OID20496","Q13291","SLAMF1",0.0682,"Inflammation","B04411","20212097_SS211642","PASS",-1.0314,-0.8526,"Plate control","PASS","PFC",0.553785812988627,"24h","24h"
10292,"PFC_02",13,"OID20496","Q13291","SLAMF1",0.0682,"Inflammation","B04411","20212097_SS211642","PASS",-1.0314,0.1194,"Plate control","PASS","PFC",1.08628299617195,"Baseline","Baseline"
10293,"PFC_10",14,"OID20496","Q13291","SLAMF1",0.0682,"Inflammation","B04411","20212097_SS211642","PASS",-1.0314,-0.4513,"Plate control","PASS","PFC",0.731383507613411,"1h","1h"
10294,"PFC_18",15,"OID20496","Q13291","SLAMF1",0.0682,"Inflammation","B04411","20212097_SS211642","PASS",-1.0314,-0.6453,"Plate control","PASS","PFC",0.639359825676391,"1h","1h"
10295,"PFC_26",16,"OID20496","Q13291","SLAMF1",0.0682,"Inflammation","B04411","20212097_SS211642","PASS",-1.0314,-0.3167,"Plate control","PASS","PFC",0.802904330619964,"24h","24h"
10296,"PFC_03",25,"OID20496","Q13291","SLAMF1",0.0682,"Inflammation","B04411","20212097_SS211642","PASS",-1.0314,-0.5544,"Plate control","PASS","PFC",0.680940194434683,"Baseline","Baseline"
10297,"PFC_11",26,"OID20496","Q13291","SLAMF1",0.0682,"Inflammation","B04411","20212097_SS211642","PASS",-1.0314,0.2837,"Plate control","PASS","PFC",1.21731285924951,"1h","1h"
10298,"PFC_19",27,"OID20496","Q13291","SLAMF1",0.0682,"Inflammation","B04411","20212097_SS211642","PASS",-1.0314,-0.4476,"Plate control","PASS","PFC",0.733261653712541,"24h","24h"
10299,"PFC_27",28,"OID20496","Q13291","SLAMF1",0.0682,"Inflammation","B04411","20212097_SS211642","PASS",-1.0314,-0.8074,"Plate control","PASS","PFC",0.571410717051122,"24h","24h"
10300,"PFC_04",37,"OID20496","Q13291","SLAMF1",0.0682,"Inflammation","B04411","20212097_SS211642","PASS",-1.0314,0.396,"Plate control","PASS","PFC",1.31585452502901,"Baseline","Baseline"
10301,"PFC_12",38,"OID20496","Q13291","SLAMF1",0.0682,"Inflammation","B04411","20212097_SS211642","PASS",-1.0314,0.5498,"Plate control","PASS","PFC",1.46388274463901,"1h","1h"
10302,"PFC_20",39,"OID20496","Q13291","SLAMF1",0.0682,"Inflammation","B04411","20212097_SS211642","PASS",-1.0314,0.1374,"Plate control","PASS","PFC",1.09992106991701,"24h","24h"
10303,"PFC_05",49,"OID20496","Q13291","SLAMF1",0.0682,"Inflammation","B04411","20212097_SS211642","PASS",-1.0314,0.1245,"Plate control","PASS","PFC",1.09012985696977,"Baseline","Baseline"
10304,"PFC_13",50,"OID20496","Q13291","SLAMF1",0.0682,"Inflammation","B04411","20212097_SS211642","PASS",-1.0314,0.2335,"Plate control","PASS","PFC",1.17568371874777,"1h","1h"

Supplemental table 1_Olink proximity extension assay (PEA) data

,"SampleID","Index","OlinkID","UniProt","Assay","MissingFreq","Panel","Panel_Lot_Nr","PlateID","QC_Warning","LOD","NPX","Normalization","Assay_Warning","condition","Value","group","alt_group"
10305,"PFC_21",51,"OID20496","Q13291","SLAMF1",0.0682,"Inflammation","B04411","20212097_SS211642","PASS",-1.0314,-1.007,"Plate control","PASS","PFC",0.497579860910041,"24h","24h"
10306,"PFC_06",61,"OID20496","Q13291","SLAMF1",0.0682,"Inflammation","B04411","20212097_SS211642","PASS",-1.0314,0.0415,"Plate control","PASS","PFC",1.02918333385451,"Baseline","Baseline"
10307,"PFC_14",62,"OID20496","Q13291","SLAMF1",0.0682,"Inflammation","B04411","20212097_SS211642","PASS",-1.0314,-0.4279,"Plate control","PASS","PFC",0.743343015041348,"1h","1h"
10308,"PFC_22",63,"OID20496","Q13291","SLAMF1",0.0682,"Inflammation","B04411","20212097_SS211642","PASS",-1.0314,0.1038,"Plate control","PASS","PFC",1.07460019131056,"24h","24h"
10309,"PFC_07",73,"OID20496","Q13291","SLAMF1",0.0682,"Inflammation","B04411","20212097_SS211642","PASS",-1.0314,0.2965,"Plate control","PASS","PFC",1.22816125842113,"Baseline","Baseline"
10310,"PFC_15",74,"OID20496","Q13291","SLAMF1",0.0682,"Inflammation","B04411","20212097_SS211642","PASS",-1.0314,-0.094,"Plate control","PASS","PFC",0.936921446872426,"1h","1h"
10311,"PFC_23",75,"OID20496","Q13291","SLAMF1",0.0682,"Inflammation","B04411","20212097_SS211642","PASS",-1.0314,-0.5197,"Plate control","PASS","PFC",0.697516862649686,"24h","24h"
10312,"PFC_08",85,"OID20496","Q13291","SLAMF1",0.0682,"Inflammation","B04411","20212097_SS211642","PASS",-1.0314,-2.8925,"Plate control","PASS","PFC",0.134669961989471,"Baseline","Baseline"
10313,"PFC_16",86,"OID20496","Q13291","SLAMF1",0.0682,"Inflammation","B04411","20212097_SS211642","PASS",-1.0314,-1.0575,"Plate control","PASS","PFC",0.480463919230517,"1h","1h"
10314,"PFC_24",87,"OID20496","Q13291","SLAMF1",0.0682,"Inflammation","B04411","20212097_SS211642","PASS",-1.0314,0.5085,"Plate control","PASS","PFC",1.42257035047616,"24h","24h"
10315,"PFC_01",1,"OID20497","Q8N8S7","ENAH",0,"Inflammation","B04411","20212097_SS211642","PASS",-0.3149,1.2389,"Plate control","PASS","PFC",2.36018508543872,"Baseline","Baseline"
10316,"PFC_09",2,"OID20497","Q8N8S7","ENAH",0,"Inflammation","B04411","20212097_SS211642","PASS",-0.3149,1.2398,"Plate control","PASS","PFC",2.36165790486106,"Baseline","Baseline"
10317,"PFC_17",3,"OID20497","Q8N8S7","ENAH",0,"Inflammation","B04411","20212097_SS211642","PASS",-0.3149,1.1418,"Plate control","PASS","PFC",2.20656156449452,"1h","1h"
10318,"PFC_25",4,"OID20497","Q8N8S7","ENAH",0,"Inflammation","B04411","20212097_SS211642","PASS",-0.3149,1.4618,"Plate control","PASS","PFC",2.75451820899065,"24h","24h"
10319,"PFC_02",13,"OID20497","Q8N8S7","ENAH",0,"Inflammation","B04411","20212097_SS211642","PASS",-0.3149,0.8769,"Plate control","PASS","PFC",1.83642502890706,"Baseline","Baseline"
10320,"PFC_10",14,"OID20497","Q8N8S7","ENAH",0,"Inflammation","B04411","20212097_SS211642","PASS",-0.3149,1.0562,"Plate control","PASS","PFC",2.07944712295203,"1h","1h"
10321,"PFC_18",15,"OID20497","Q8N8S7","ENAH",0,"Inflammation","B04411","20212097_SS211642","PASS",-0.3149,1.4335,"Plate control","PASS","PFC",2.70101190744472,"1h","1h"
10322,"PFC_26",16,"OID20497","Q8N8S7","ENAH",0,"Inflammation","B04411","20212097_SS211642","PASS",-0.3149,1.5793,"Plate control","PASS","PFC",2.98824823837446,"24h","24h"
10323,"PFC_03",25,"OID20497","Q8N8S7","ENAH",0,"Inflammation","B04411","20212097_SS211642","PASS",-0.3149,0.5542,"Plate control","PASS","PFC",1.4683541790371,"Baseline","Baseline"
10324,"PFC_11",26,"OID20497","Q8N8S7","ENAH",0,"Inflammation","B04411","20212097_SS211642","PASS",-0.3149,1.2498,"Plate control","PASS","PFC",2.37808453463516,"1h","1h"
10325,"PFC_19",27,"OID20497","Q8N8S7","ENAH",0,"Inflammation","B04411","20212097_SS211642","PASS",-0.3149,1.3171,"Plate control","PASS","PFC",2.49164753299973,"24h","24h"
10326,"PFC_27",28,"OID20497","Q8N8S7","ENAH",0,"Inflammation","B04411","20212097_SS211642","PASS",-0.3149,1.6918,"Plate control","PASS","PFC",3.23059522379983,"24h","24h"
10327,"PFC_04",37,"OID20497","Q8N8S7","ENAH",0,"Inflammation","B04411","20212097_SS211642","PASS",-0.3149,2.5636,"Plate control","PASS","PFC",5.91181039596341,"Baseline","Baseline"
10328,"PFC_12",38,"OID20497","Q8N8S7","ENAH",0,"Inflammation","B04411","20212097_SS211642","PASS",-0.3149,0.8175,"Plate control","PASS","PFC",1.76234942619339,"1h","1h"
10329,"PFC_20",39,"OID20497","Q8N8S7","ENAH",0,"Inflammation","B04411","20212097_SS211642","PASS",-0.3149,0.9499,"Plate control","PASS","PFC",1.93173875528186,"24h","24h"
10330,"PFC_05",49,"OID20497","Q8N8S7","ENAH",0,"Inflammation","B04411","20212097_SS211642","PASS",-0.3149,1.2782,"Plate control","PASS","PFC",2.42536184136354,"Baseline","Baseline"
10331,"PFC_13",50,"OID20497","Q8N8S7","ENAH",0,"Inflammation","B04411","20212097_SS211642","PASS",-0.3149,2.3164,"Plate control","PASS","PFC",4.98087774264576,"1h","1h"
10332,"PFC_21",51,"OID20497","Q8N8S7","ENAH",0,"Inflammation","B04411","20212097_SS211642","PASS",-0.3149,0.8018,"Plate control","PASS","PFC",1.74327479312056,"24h","24h"
10333,"PFC_06",61,"OID20497","Q8N8S7","ENAH",0,"Inflammation","B04411","20212097_SS211642","PASS",-0.3149,0.4624,"Plate control","PASS","PFC",1.37783200957844,"Baseline","Baseline"
10334,"PFC_14",62,"OID20497","Q8N8S7","ENAH",0,"Inflammation","B04411","20212097_SS211642","PASS",-0.3149,1.3098,"Plate control","PASS","PFC",2.47907170374295,"1h","1h"
10335,"PFC_22",63,"OID20497","Q8N8S7","ENAH",0,"Inflammation","B04411","20212097_SS211642","PASS",-0.3149,2.2075,"Plate control","PASS","PFC",4.61874212675477,"24h","24h"
10336,"PFC_07",73,"OID20497","Q8N8S7","ENAH",0,"Inflammation","B04411","20212097_SS211642","PASS",-0.3149,1.0297,"Plate control","PASS","PFC",2.04159966855493,"Baseline","Baseline"
10337,"PFC_15",74,"OID20497","Q8N8S7","ENAH",0,"Inflammation","B04411","20212097_SS211642","PASS",-0.3149,0.5959,"Plate control","PASS","PFC",1.51141516136955,"1h","1h"
10338,"PFC_23",75,"OID20497","Q8N8S7","ENAH",0,"Inflammation","B04411","20212097_SS211642","PASS",-0.3149,1.5245,"Plate control","PASS","PFC",2.87686994008267,"24h","24h"
10339,"PFC_08",85,"OID20497","Q8N8S7","ENAH",0,"Inflammation","B04411","20212097_SS211642","PASS",-0.3149,0.8541,"Plate control","PASS","PFC",1.80763074457527,"Baseline","Baseline"
10340,"PFC_16",86,"OID20497","Q8N8S7","ENAH",0,"Inflammation","B04411","20212097_SS211642","PASS",-0.3149,1.5087,"Plate control","PASS","PFC",2.84553514854374,"1h","1h"
10341,"PFC_24",87,"OID20497","Q8N8S7","ENAH",0,"Inflammation","B04411","20212097_SS211642","PASS",-0.3149,0.5654,"Plate control","PASS","PFC",1.47979773942858,"24h","24h"
10342,"PFC_01",1,"OID20498","Q13261","IL15RA",0.0909,"Inflammation","B04411","20212097_SS211642","PASS",-0.5571,0.5006,"Plate control","PASS","PFC",1.4148018395795,"Baseline","Baseline"
10343,"PFC_09",2,"OID20498","Q13261","IL15RA",0.0909,"Inflammation","B04411","20212097_SS211642","PASS",-0.5571,0.94,"Plate control","PASS","PFC",1.91852823865053,"Baseline","Baseline"
10344,"PFC_17",3,"OID20498","Q13261","IL15RA",0.0909,"Inflammation","B04411","20212097_SS211642","PASS",-0.5571,-0.9205,"Plate control","PASS","PFC",0.528325884748839,"1h","1h"
10345,"PFC_25",4,"OID20498","Q13261","IL15RA",0.0909,"Inflammation","B04411","20212097_SS211642","PASS",-0.5571,0.0711,"Plate control","PASS","PFC",1.05051735780166,"24h","24h"
10346,"PFC_02",13,"OID20498","Q13261","IL15RA",0.0909,"Inflammation","B04411","20212097_SS211642","PASS",-0.5571,1.0182,"Plate control","PASS","PFC",2.02539037396524,"Baseline","Baseline"
10347,"PFC_10",14,"OID20498","Q13261","IL15RA",0.0909,"Inflammation","B04411","20212097_SS211642","PASS",-0.5571,0.1504,"Plate control","PASS","PFC",1.10987715269995,"1h","1h"
10348,"PFC_18",15,"OID20498","Q13261","IL15RA",0.0909,"Inflammation","B04411","20212097_SS211642","PASS",-0.5571,-0.0388,"Plate control","PASS","PFC",0.973464315613742,"1h","1h"
10349,"PFC_26",16,"OID20498","Q13261","IL15RA",0.0909,"Inflammation","B04411","20212097_SS211642","PASS",-0.5571,0.0501,"Plate control","PASS","PFC",1.03533668542473,"24h","24h"
10350,"PFC_03",25,"OID20498","Q13261","IL15RA",0.0909,"Inflammation","B04411","20212097_SS211642","PASS",-0.5571,0.1929,"Plate control","PASS","PFC",1.14305910184868,"Baseline","Baseline"
10351,"PFC_11",26,"OID20498","Q13261","IL15RA",0.0909,"Inflammation","B04411","20212097_SS211642","PASS",-0.5571,0.883,"Plate control","PASS","PFC",1.84420623582526,"1h","1h"
10352,"PFC_19",27,"OID20498","Q13261","IL15RA",0.0909,"Inflammation","B04411","20212097_SS211642","PASS",-0.5571,0.2126,"Plate control","PASS","PFC",1.15877462681521,"24h","24h"
10353,"PFC_27",28,"OID20498","Q13261","IL15RA",0.0909,"Inflammation","B04411","20212097_SS211642","PASS",-0.5571,0.1793,"Plate control","PASS","PFC",1.13233433993742,"24h","24h"
10354,"PFC_04",37,"OID20498","Q13261","IL15RA",0.0909,"Inflammation","B04411","20212097_SS211642","PASS",-0.5571,0.1599,"Plate control","PASS","PFC",1.11720969631321,"Baseline","Baseline"
10355,"PFC_12",38,"OID20498","Q13261","IL15RA",0.0909,"Inflammation","B04411","20212097_SS211642","PASS",-0.5571,-0.4636,"Plate control","PASS","PFC",0.725174449619089,"1h","1h"
10356,"PFC_20",39,"OID20498","Q13261","IL15RA",0.0909,"Inflammation","B04411","20212097_SS211642","PASS",-0.5571,0.0927,"Plate control","PASS","PFC",1.06636401361212,"24h","24h"
10357,"PFC_05",49,"OID20498","Q13261","IL15RA",0.0909,"Inflammation","B04411","20212097_SS211642","PASS",-0.5571,0.2609,"Plate control","PASS","PFC",1.19822596376605,"Baseline","Baseline"
10358,"PFC_13",50,"OID20498","Q13261","IL15RA",0.0909,"Inflammation","B04411","20212097_SS211642","PASS",-0.5571,0.2762,"Plate control","PASS","PFC",1.21100095269371,"1h","1h"
10359,"PFC_21",51,"OID20498","Q13261","IL15RA",0.0909,"Inflammation","B04411","20212097_SS211642","PASS",-0.5571,-0.4728,"Plate control","PASS","PFC",0.72056475898155,"24h","24h"
10360,"PFC_06",61,"OID20498","Q13261","IL15RA",0.0909,"Inflammation","B04411","20212097_SS211642","PASS",-0.5571,0.3914,"Plate control","PASS","PFC",1.31166563473943,"Baseline","Baseline"
10361,"PFC_14",62,"OID20498","Q13261","IL15RA",0.0909,"Inflammation","B04411","20212097_SS211642","PASS",-0.5571,0.6725,"Plate control","PASS","PFC",1.59383247710592,"1h","1h"
10362,"PFC_22",63,"OID20498","Q13261","IL15RA",0.0909,"Inflammation","B04411","20212097_SS211642","PASS",-0.5571,0.5066,"Plate control","PASS","PFC",1.42069808742053,"24h","24h"
10363,"PFC_07",73,"OID20498","Q13261","IL15RA",0.0909,"Inflammation","B04411","20212097_SS211642","PASS",-0.5571,-0.116,"Plate control","PASS","PFC",0.922742492653798,"Baseline","Baseline"
10364,"PFC_15",74,"OID20498","Q13261","IL15RA",0.0909,"Inflammation","B04411","20212097_SS211642","PASS",-0.5571,0.4357,"Plate control","PASS","PFC",1.35256694341622,"1h","1h"
10365,"PFC_23",75,"OID20498","Q13261","IL15RA",0.0909,"Inflammation","B04411","20212097_SS211642","PASS",-0.5571,-0.0106,"Plate control","PASS","PFC",0.992679565751354,"24h","24h"
10366,"PFC_08",85,"OID20498","Q13261","IL15RA",0.0909,"Inflammation","B04411","20212097_SS211642","PASS",-0.5571,-0.6787,"Plate control","PASS","PFC",0.624727957847545,"Baseline","Baseline"
10367,"PFC_16",86,"OID20498","Q13261","IL15RA",0.0909,"Inflammation","B04411","20212097_SS211642","PASS",-0.5571,0.3089,"Plate control","PASS","PFC",1.23876283031168,"1h","1h"
10368,"PFC_24",87,"OID20498","Q13261","IL15RA",0.0909,"Inflammation","B04411","20212097_SS211642","PASS",-0.5571,0.4446,"Plate control","PASS","PFC",1.36093673240175,"24h","24h"
10369,"PFC_01",1,"OID20455","P20809","IL11",0.8636,"Inflammation","B04411","20212097_SS211642","PASS",1.2309,-0.3887,"Plate control","PASS","PFC",0.763817563864721,"Baseline","Baseline"
10370,"PFC_09",2,"OID20455","P20809","IL11",0.8636,"Inflammation","B04411","20212097_SS211642","PASS",1.2309,0.5847,"Plate control","PASS","PFC",1.49972709737615,"Baseline","Baseline"
10371,"PFC_17",3,"OID20455","P20809","IL11",0.8636,"Inflammation","B04411","20212097_SS211642","PASS",1.2309,0.0568,"Plate control","PASS","PFC",1.0401560602785,"1h","1h"
10372,"PFC_25",4,"OID20455","P20809","IL11",0.8636,"Inflammation","B04411","20212097_SS211642","PASS",1.2309,0.2933,"Plate control","PASS","PFC",1.22544012852303,"24h","24h"
10373,"PFC_02",13,"OID20455","P20809","IL11",0.8636,"Inflammation","B04411","20212097_SS211642","PASS",1.2309,-0.6024,"Plate control","PASS","PFC",0.658657331959377,"Baseline","Baseline"
10374,"PFC_10",14,"OID20455","P20809","IL11",0.8636,"Inflammation","B04411","20212097_SS211642","PASS",1.2309,-2e-04,"Plate control","PASS","PFC",0.999861380172504,"1h","1h"
10375,"PFC_18",15,"OID20455","P20809","IL11",0.8636,"Inflammation","B04411","20212097_SS211642","PASS",1.2309,0.3811,"Plate control","PASS","PFC",1.30233445738628,"1h","1h"
10376,"PFC_26",16,"OID20455","P20809","IL11",0.8636,"Inflammation","B04411","20212097_SS211642","PASS",1.2309,0.3644,"Plate control","PASS","PFC",1.28734612596014,"24h","24h"
10377,"PFC_03",25,"OID20455","P20809","IL11",0.8636,"Inflammation","B04411","20212097_SS211642","PASS",1.2309,2.0186,"Plate control","PASS","PFC",4.05190401854345,"Baseline","Baseline"
10378,"PFC_11",26,"OID20455","P20809","IL11",0.8636,"Inflammation","B04411","20212097_SS211642","PASS",1.2309,-0.3885,"Plate control","PASS","PFC",0.7639234588028,"1h","1h"
10379,"PFC_19",27,"OID20455","P20809","IL11",0.8636,"Inflammation","B04411","20212097_SS211642","PASS",1.2309,0.1854,"Plate control","PASS","PFC",1.13713220952695,"24h","24h"
10380,"PFC_27",28,"OID20455","P20809","IL11",0.8636,"Inflammation","B04411","20212097_SS211642","PASS",1.2309,-0.2935,"Plate control","PASS","PFC",0.815920220743539,"24h","24h"
10381,"PFC_04",37,"OID20455","P20809","IL11",0.8636,"Inflammation","B04411","20212097_SS211642","PASS",1.2309,0.172,"Plate control","PASS","PFC",1.12661922849728,"Baseline","Baseline"
10382,"PFC_12",38,"OID20455","P20809","IL11",0.8636,"Inflammation","B04411","20212097_SS211642","PASS",1.2309,1.8449,"Plate control","PASS","PFC",3.59228048499693,"1h","1h"
10383,"PFC_20",39,"OID20455","P20809","IL11",0.8636,"Inflammation","B04411","20212097_SS211642","PASS",1.2309,-0.0535,"Plate control","PASS","PFC",0.963595793010082,"24h","24h"
10384,"PFC_05",49,"OID20455","P20809","IL11",0.8636,"Inflammation","B04411","20212097_SS211642","PASS",1.2309,-0.2881,"Plate control","PASS","PFC",0.818979928529536,"Baseline","Baseline"
10385,"PFC_13",50,"OID20455","P20809","IL11",0.8636,"Inflammation","B04411","20212097_SS211642","PASS",1.2309,0.3677,"Plate control","PASS","PFC",1.29029415342683,"1h","1h"
10386,"PFC_21",51,"OID20455","P20809","IL11",0.8636,"Inflammation","B04411","20212097_SS211642","PASS",1.2309,2.142,"Plate control","PASS","PFC",4.41373496016784,"24h","24h"
10387,"PFC_06",61,"OID20455","P20809","IL11",0.8636,"Inflammation","B04411","20212097_SS211642","PASS",1.2309,-0.2847,"Plate control","PASS","PFC",0.820912294976113,"Baseline","Baseline"
10388,"PFC_14",62,"OID20455","P20809","IL11",0.8636,"Inflammation","B04411","20212097_SS211642","PASS",1.2309,0.1154,"Plate control","PASS","PFC",1.08327535159503,"1h","1h"
10389,"PFC_22",63,"OID20455","P20809","IL11",0.8636,"Inflammation","B04411","20212097_SS211642","PASS",1.2309,0.1369,"Plate control","PASS","PFC",1.0995399323727,"24h","24h"
10390,"PFC_07",73,"OID20455","P20809","IL11",0.8636,"Inflammation","B04411","20212097_SS211642","PASS",1.2309,-0.3686,"Plate control","PASS","PFC",0.774533744438288,"Baseline","Baseline"
10391,"PFC_15",74,"OID20455","P20809","IL11",0.8636,"Inflammation","B04411","20212097_SS211642","PASS",1.2309,0.4133,"Plate control","PASS","PFC",1.33172850960439,"1h","1h"
10392,"PFC_23",75,"OID20455","P20809","IL11",0.8636,"Inflammation","B04411","20212097_SS211642","PASS",1.2309,0.0027,"Plate control","PASS","PFC",1.00187324973175,"24h","24h"
10393,"PFC_08",85,"OID20455","P20809","IL11",0.8636,"Inflammation","B04411","20212097_SS211642","PASS",1.2309,-0.0286,"Plate control","PASS","PFC",0.980371194275255,"Baseline","Baseline"
10394,"PFC_16",86,"OID20455","P20809","IL11",0.8636,"Inflammation","B04411","20212097_SS211642","PASS",1.2309,-0.6933,"Plate control","PASS","PFC",0.618437625482863,"1h","1h"
10395,"PFC_24",87,"OID20455","P20809","IL11",0.8636,"Inflammation","B04411","20212097_SS211642","PASS",1.2309,0.1611,"Plate control","PASS","PFC",1.11813935179377,"24h","24h"
10396,"PFC_01",1,"OID20456","Q96PD4","IL17F",0.1591,"Inflammation","B04411","20212097_SS211642","PASS",-0.6214,-0.4716,"Plate control","PASS","PFC",0.721164357230201,"Baseline","Baseline"
10397,"PFC_09",2,"OID20456","Q96PD4","IL17F",0.1591,"Inflammation","B04411","20212097_SS211642","PASS",-0.6214,-0.0793,"Plate control","PASS","PFC",0.946516788138322,"Baseline","Baseline"
10398,"PFC_17",3,"OID20456","Q96PD4","IL17F",0.1591,"Inflammation","B04411","20212097_SS211642","PASS",-0.6214,0.0031,"Plate control","PASS","PFC",1.00215106649088,"1h","1h"
10399,"PFC_25",4,"OID20456","Q96PD4","IL17F",0.1591,"Inflammation","B04411","20212097_SS211642","PASS",-0.6214,-0.4289,"Plate control","PASS","PFC",0.742827947455728,"24h","24h"
10400,"PFC_02",13,"OID20456","Q96PD4","IL17F",0.1591,"Inflammation","B04411","20212097_SS211642","PASS",-0.6214,-0.4104,"Plate control","PASS","PFC",0.752414731123071,"Baseline","Baseline"
10401,"PFC_10",14,"OID20456","Q96PD4","IL17F",0.1591,"Inflammation","B04411","20212097_SS211642","PASS",-0.6214,-0.582,"Plate control","PASS","PFC",0.66803703919505,"1h","1h"
10402,"PFC_18",15,"OID20456","Q96PD4","IL17F",0.1591,"Inflammation","B04411","20212097_SS211642","PASS",-0.6214,0.0472,"Plate control","PASS","PFC",1.03325761767685,"1h","1h"
10403,"PFC_26",16,"OID20456","Q96PD4","IL17F",0.1591,"Inflammation","B04411","20212097_SS211642","PASS",-0.6214,-0.2166,"Plate control","PASS","PFC",0.860591204083296,"24h","24h"
10404,"PFC_03",25,"OID20456","Q96PD4","IL17F",0.1591,"Inflammation","B04411","20212097_SS211642","PASS",-0.6214,0.1253,"Plate control","PASS","PFC",1.09073452095217,"Baseline","Baseline"
10405,"PFC_11",26,"OID20456","Q96PD4","IL17F",0.1591,"Inflammation","B04411","20212097_SS211642","PASS",-0.6214,-0.4197,"Plate control","PASS","PFC",0.747580063059506,"1h","1h"
10406,"PFC_19",27,"OID20456","Q96PD4","IL17F",0.1591,"Inflammation","B04411","20212097_SS211642","PASS",-0.6214,-0.445,"Plate control","PASS","PFC",0.734584316639154,"24h","24h"
10407,"PFC_27",28,"OID20456","Q96PD4","IL17F",0.1591,"Inflammation","B04411","20212097_SS211642","PASS",-0.6214,-0.7727,"Plate control","PASS","PFC",0.585321022168177,"24h","24h"
10408,"PFC_04",37,"OID20456","Q96PD4","IL17F",0.1591,"Inflammation","B04411","20212097_SS211642","PASS",-0.6214,-0.4378,"Plate control","PASS","PFC",0.738259540251533,"Baseline","Baseline"
10409,"PFC_12",38,"OID20456","Q96PD4","IL17F",0.1591,"Inflammation","B04411","20212097_SS211642","PASS",-0.6214,0.6755,"Plate control","PASS","PFC",1.59715020688634,"1h","1h"
10410,"PFC_20",39,"OID20456","Q96PD4","IL17F",0.1591,"Inflammation","B04411","20212097_SS211642","PASS",-0.6214,-0.1863,"Plate control","PASS","PFC",0.87885678877767,"24h","24h"
10411,"PFC_05",49,"OID20456","Q96PD4","IL17F",0.1591,"Inflammation","B04411","20212097_SS211642","PASS",-0.6214,-0.1343,"Plate control","PASS","PFC",0.911111800564467,"Baseline","Baseline"
10412,"PFC_13",50,"OID20456","Q96PD4","IL17F",0.1591,"Inflammation","B04411","20212097_SS211642","PASS",-0.6214,-0.7884,"Plate control","PASS","PFC",0.5789858518769,"1h","1h"
10413,"PFC_21",51,"OID20456","Q96PD4","IL17F",0.1591,"Inflammation","B04411","20212097_SS211642","PASS",-0.6214,-0.1635,"Plate control","PASS","PFC",0.892856358290927,"24h","24h"
10414,"PFC_06",61,"OID20456","Q96PD4","IL17F",0.1591,"Inflammation","B04411","20212097_SS211642","PASS",-0.6214,-0.4048,"Plate control","PASS","PFC",0.755340998020259,"Baseline","Baseline"
10415,"PFC_14",62,"OID20456","Q96PD4","IL17F",0.1591,"Inflammation","B04411","20212097_SS211642","PASS",-0.6214,-0.3067,"Plate control","PASS","PFC",0.808488971882347,"1h","1h"
10416,"PFC_22",63,"OID20456","Q96PD4","IL17F",0.1591,"Inflammation","B04411","20212097_SS211642","PASS",-0.6214,-0.4648,"Plate control","PASS","PFC",0.72457151725617,"24h","24h"

Supplemental table 1_Olink proximity extension assay (PEA) data

**,"SampleID","Index","OlinkID","UniProt","Assay","MissingFreq","Panel","Panel_Lot_Nr","PlateID","QC_Warning","LOD","NPX","Normalization","Assay_Warning","condition","Value","group","alt_group"**
10417,"PFC_07",73,"OID20456","Q96PD4","IL17F",0.1591,"Inflammation","B04411","20212097_SS211642","PASS",-0.6214,-0.4762,"Plate control","PASS","PFC",0.718868603166412,"Baseline","Baseline"
10418,"PFC_15",74,"OID20456","Q96PD4","IL17F",0.1591,"Inflammation","B04411","20212097_SS211642","PASS",-0.6214,-0.135,"Plate control","PASS","PFC",0.910669833591978,"1h","1h"
10419,"PFC_23",75,"OID20456","Q96PD4","IL17F",0.1591,"Inflammation","B04411","20212097_SS211642","PASS",-0.6214,-0.6971,"Plate control","PASS","PFC",0.61681082935424,"24h","24h"
10420,"PFC_08",85,"OID20456","Q96PD4","IL17F",0.1591,"Inflammation","B04411","20212097_SS211642","PASS",-0.6214,-0.7516,"Plate control","PASS","PFC",0.593944486588336,"Baseline","Baseline"
10421,"PFC_16",86,"OID20456","Q96PD4","IL17F",0.1591,"Inflammation","B04411","20212097_SS211642","PASS",-0.6214,0.1634,"Plate control","PASS","PFC",1.11992335429876,"1h","1h"
10422,"PFC_24",87,"OID20456","Q96PD4","IL17F",0.1591,"Inflammation","B04411","20212097_SS211642","PASS",-0.6214,-0.2394,"Plate control","PASS","PFC",0.84709753707607,"24h","24h"
10423,"PFC_01",1,"OID20457","O76038","SCGN",0.1136,"Inflammation","B04411","20212097_SS211642","PASS",-0.2672,0.7556,"Plate control","PASS","PFC",1.68833360447725,"Baseline","Baseline"
10424,"PFC_09",2,"OID20457","O76038","SCGN",0.1136,"Inflammation","B04411","20212097_SS211642","PASS",-0.2672,0.3452,"Plate control","PASS","PFC",1.2703270750588,"Baseline","Baseline"
10425,"PFC_17",3,"OID20457","O76038","SCGN",0.1136,"Inflammation","B04411","20212097_SS211642","PASS",-0.2672,0.1691,"Plate control","PASS","PFC",1.12435685555611,"1h","1h"
10426,"PFC_25",4,"OID20457","O76038","SCGN",0.1136,"Inflammation","B04411","20212097_SS211642","PASS",-0.2672,1.0596,"Plate control","PASS","PFC",2.0843535360494,"24h","24h"
10427,"PFC_02",13,"OID20457","O76038","SCGN",0.1136,"Inflammation","B04411","20212097_SS211642","PASS",-0.2672,0.2076,"Plate control","PASS","PFC",1.15476557116469,"Baseline","Baseline"
10428,"PFC_10",14,"OID20457","O76038","SCGN",0.1136,"Inflammation","B04411","20212097_SS211642","PASS",-0.2672,0.4038,"Plate control","PASS","PFC",1.32298802211137,"1h","1h"
10429,"PFC_18",15,"OID20457","O76038","SCGN",0.1136,"Inflammation","B04411","20212097_SS211642","PASS",-0.2672,0.739,"Plate control","PASS","PFC",1.66901856231082,"1h","1h"
10430,"PFC_26",16,"OID20457","O76038","SCGN",0.1136,"Inflammation","B04411","20212097_SS211642","PASS",-0.2672,0.4819,"Plate control","PASS","PFC",1.39658172544382,"24h","24h"
10431,"PFC_03",25,"OID20457","O76038","SCGN",0.1136,"Inflammation","B04411","20212097_SS211642","PASS",-0.2672,0.3243,"Plate control","PASS","PFC",1.25205678947598,"Baseline","Baseline"
10432,"PFC_11",26,"OID20457","O76038","SCGN",0.1136,"Inflammation","B04411","20212097_SS211642","PASS",-0.2672,0.7305,"Plate control","PASS","PFC",1.65921403175763,"1h","1h"
10433,"PFC_19",27,"OID20457","O76038","SCGN",0.1136,"Inflammation","B04411","20212097_SS211642","PASS",-0.2672,0.1272,"Plate control","PASS","PFC",1.09217194243191,"24h","24h"
10434,"PFC_27",28,"OID20457","O76038","SCGN",0.1136,"Inflammation","B04411","20212097_SS211642","PASS",-0.2672,-0.0159,"Plate control","PASS","PFC",0.989039468996991,"24h","24h"
10435,"PFC_04",37,"OID20457","O76038","SCGN",0.1136,"Inflammation","B04411","20212097_SS211642","PASS",-0.2672,0.8579,"Plate control","PASS","PFC",1.81239824630726,"Baseline","Baseline"
10436,"PFC_12",38,"OID20457","O76038","SCGN",0.1136,"Inflammation","B04411","20212097_SS211642","PASS",-0.2672,0.4589,"Plate control","PASS","PFC",1.3744934196462,"1h","1h"
10437,"PFC_20",39,"OID20457","O76038","SCGN",0.1136,"Inflammation","B04411","20212097_SS211642","PASS",-0.2672,-0.2085,"Plate control","PASS","PFC",0.865436576077121,"24h","24h"
10438,"PFC_05",49,"OID20457","O76038","SCGN",0.1136,"Inflammation","B04411","20212097_SS211642","PASS",-0.2672,0.4519,"Plate control","PASS","PFC",1.36784048913865,"Baseline","Baseline"
10439,"PFC_13",50,"OID20457","O76038","SCGN",0.1136,"Inflammation","B04411","20212097_SS211642","PASS",-0.2672,0.5666,"Plate control","PASS","PFC",1.48102911262837,"1h","1h"
10440,"PFC_21",51,"OID20457","O76038","SCGN",0.1136,"Inflammation","B04411","20212097_SS211642","PASS",-0.2672,0.0345,"Plate control","PASS","PFC",1.02420180022052,"24h","24h"
10441,"PFC_06",61,"OID20457","O76038","SCGN",0.1136,"Inflammation","B04411","20212097_SS211642","PASS",-0.2672,0.7854,"Plate control","PASS","PFC",1.72357012829257,"Baseline","Baseline"
10442,"PFC_14",62,"OID20457","O76038","SCGN",0.1136,"Inflammation","B04411","20212097_SS211642","PASS",-0.2672,-0.0778,"Plate control","PASS","PFC",0.947501413081701,"1h","1h"
10443,"PFC_22",63,"OID20457","O76038","SCGN",0.1136,"Inflammation","B04411","20212097_SS211642","PASS",-0.2672,1.0606,"Plate control","PASS","PFC",2.08579880065888,"24h","24h"
10444,"PFC_07",73,"OID20457","O76038","SCGN",0.1136,"Inflammation","B04411","20212097_SS211642","PASS",-0.2672,0.6152,"Plate control","PASS","PFC",1.53177033083341,"Baseline","Baseline"
10445,"PFC_15",74,"OID20457","O76038","SCGN",0.1136,"Inflammation","B04411","20212097_SS211642","PASS",-0.2672,0.6684,"Plate control","PASS","PFC",1.58930938923965,"1h","1h"
10446,"PFC_23",75,"OID20457","O76038","SCGN",0.1136,"Inflammation","B04411","20212097_SS211642","PASS",-0.2672,-0.2792,"Plate control","PASS","PFC",0.824047839747196,"24h","24h"
10447,"PFC_08",85,"OID20457","O76038","SCGN",0.1136,"Inflammation","B04411","20212097_SS211642","PASS",-0.2672,-0.3123,"Plate control","PASS","PFC",0.805356804395925,"Baseline","Baseline"
10448,"PFC_16",86,"OID20457","O76038","SCGN",0.1136,"Inflammation","B04411","20212097_SS211642","PASS",-0.2672,0.4306,"Plate control","PASS","PFC",1.3477939920859,"1h","1h"
10449,"PFC_24",87,"OID20457","O76038","SCGN",0.1136,"Inflammation","B04411","20212097_SS211642","PASS",-0.2672,0.5767,"Plate control","PASS","PFC",1.49143385949145,"24h","24h"
10450,"PFC_01",1,"OID20458","O95715","CXCL14",0.9318,"Inflammation","B04411","20212097_SS211642","PASS",2.1607,1.0747,"Plate control","PASS","PFC",2.10628403691535,"Baseline","Baseline"
10451,"PFC_09",2,"OID20458","O95715","CXCL14",0.9318,"Inflammation","B04411","20212097_SS211642","PASS",2.1607,0.7844,"Plate control","PASS","PFC",1.72237585446923,"Baseline","Baseline"
10452,"PFC_17",3,"OID20458","O95715","CXCL14",0.9318,"Inflammation","B04411","20212097_SS211642","PASS",2.1607,0.348,"Plate control","PASS","PFC",1.27279493527594,"1h","1h"
10453,"PFC_25",4,"OID20458","O95715","CXCL14",0.9318,"Inflammation","B04411","20212097_SS211642","PASS",2.1607,1.2911,"Plate control","PASS","PFC",2.44714569959939,"24h","24h"
10454,"PFC_02",13,"OID20458","O95715","CXCL14",0.9318,"Inflammation","B04411","20212097_SS211642","PASS",2.1607,2.0885,"Plate control","PASS","PFC",4.25305643907925,"Baseline","Baseline"
10455,"PFC_10",14,"OID20458","O95715","CXCL14",0.9318,"Inflammation","B04411","20212097_SS211642","PASS",2.1607,0.4028,"Plate control","PASS","PFC",1.3220713144373,"1h","1h"
10456,"PFC_18",15,"OID20458","O95715","CXCL14",0.9318,"Inflammation","B04411","20212097_SS211642","PASS",2.1607,0.6848,"Plate control","PASS","PFC",1.60747912211496,"1h","1h"
10457,"PFC_26",16,"OID20458","O95715","CXCL14",0.9318,"Inflammation","B04411","20212097_SS211642","PASS",2.1607,0.4153,"Plate control","PASS","PFC",1.33357595758529,"24h","24h"
10458,"PFC_03",25,"OID20458","O95715","CXCL14",0.9318,"Inflammation","B04411","20212097_SS211642","PASS",2.1607,0.9017,"Plate control","PASS","PFC",1.86826615834487,"Baseline","Baseline"
10459,"PFC_11",26,"OID20458","O95715","CXCL14",0.9318,"Inflammation","B04411","20212097_SS211642","PASS",2.1607,2.1708,"Plate control","PASS","PFC",4.50273009034466,"1h","1h"
10460,"PFC_19",27,"OID20458","O95715","CXCL14",0.9318,"Inflammation","B04411","20212097_SS211642","PASS",2.1607,1.0082,"Plate control","PASS","PFC",2.01139998071529,"24h","24h"
10461,"PFC_27",28,"OID20458","O95715","CXCL14",0.9318,"Inflammation","B04411","20212097_SS211642","PASS",2.1607,0.5915,"Plate control","PASS","PFC",1.50681259760201,"24h","24h"
10462,"PFC_04",37,"OID20458","O95715","CXCL14",0.9318,"Inflammation","B04411","20212097_SS211642","PASS",2.1607,1.7429,"Plate control","PASS","PFC",3.3470729612742,"Baseline","Baseline"
10463,"PFC_12",38,"OID20458","O95715","CXCL14",0.9318,"Inflammation","B04411","20212097_SS211642","PASS",2.1607,0.967,"Plate control","PASS","PFC",1.95477153282161,"1h","1h"
10464,"PFC_20",39,"OID20458","O95715","CXCL14",0.9318,"Inflammation","B04411","20212097_SS211642","PASS",2.1607,1.7466,"Plate control","PASS","PFC",3.35566803070123,"24h","24h"
10465,"PFC_05",49,"OID20458","O95715","CXCL14",0.9318,"Inflammation","B04411","20212097_SS211642","PASS",2.1607,0.9003,"Plate control","PASS","PFC",1.86645406093395,"Baseline","Baseline"
10466,"PFC_13",50,"OID20458","O95715","CXCL14",0.9318,"Inflammation","B04411","20212097_SS211642","PASS",2.1607,0.9459,"Plate control","PASS","PFC",1.92639025621501,"1h","1h"
10467,"PFC_21",51,"OID20458","O95715","CXCL14",0.9318,"Inflammation","B04411","20212097_SS211642","PASS",2.1607,0.8486,"Plate control","PASS","PFC",1.80075261583588,"24h","24h"
10468,"PFC_06",61,"OID20458","O95715","CXCL14",0.9318,"Inflammation","B04411","20212097_SS211642","PASS",2.1607,0.5214,"Plate control","PASS","PFC",1.43534744225553,"Baseline","Baseline"
10469,"PFC_14",62,"OID20458","O95715","CXCL14",0.9318,"Inflammation","B04411","20212097_SS211642","PASS",2.1607,1.0973,"Plate control","PASS","PFC",2.1395390341508,"1h","1h"
10470,"PFC_22",63,"OID20458","O95715","CXCL14",0.9318,"Inflammation","B04411","20212097_SS211642","PASS",2.1607,1.4214,"Plate control","PASS","PFC",2.67845303588476,"24h","24h"
10471,"PFC_07",73,"OID20458","O95715","CXCL14",0.9318,"Inflammation","B04411","20212097_SS211642","PASS",2.1607,1.2173,"Plate control","PASS","PFC",2.32511165814701,"Baseline","Baseline"
10472,"PFC_15",74,"OID20458","O95715","CXCL14",0.9318,"Inflammation","B04411","20212097_SS211642","PASS",2.1607,0.3801,"Plate control","PASS","PFC",1.30143206071198,"1h","1h"
10473,"PFC_23",75,"OID20458","O95715","CXCL14",0.9318,"Inflammation","B04411","20212097_SS211642","PASS",2.1607,1.1602,"Plate control","PASS","PFC",2.23488407538944,"24h","24h"
10474,"PFC_08",85,"OID20458","O95715","CXCL14",0.9318,"Inflammation","B04411","20212097_SS211642","PASS",2.1607,0.1878,"Plate control","PASS","PFC",1.13902546381888,"Baseline","Baseline"
10475,"PFC_16",86,"OID20458","O95715","CXCL14",0.9318,"Inflammation","B04411","20212097_SS211642","PASS",2.1607,0.9724,"Plate control","PASS","PFC",1.96210194274008,"1h","1h"
10476,"PFC_24",87,"OID20458","O95715","CXCL14",0.9318,"Inflammation","B04411","20212097_SS211642","PASS",2.1607,0.433,"Plate control","PASS","PFC",1.35003798512275,"24h","24h"
10477,"PFC_01",1,"OID20459","Q03426","MVK",0.0227,"Inflammation","B04411","20212097_SS211642","PASS",0.0287,1.7361,"Plate control","PASS","PFC",3.33133398599562,"Baseline","Baseline"
10478,"PFC_09",2,"OID20459","Q03426","MVK",0.0227,"Inflammation","B04411","20212097_SS211642","PASS",0.0287,2.5652,"Plate control","PASS","PFC",5.91837044048486,"Baseline","Baseline"
10479,"PFC_17",3,"OID20459","Q03426","MVK",0.0227,"Inflammation","B04411","20212097_SS211642","PASS",0.0287,3.2421,"Plate control","PASS","PFC",9.46170381455638,"1h","1h"
10480,"PFC_25",4,"OID20459","Q03426","MVK",0.0227,"Inflammation","B04411","20212097_SS211642","PASS",0.0287,1.9667,"Plate control","PASS","PFC",3.90873018353722,"24h","24h"
10481,"PFC_02",13,"OID20459","Q03426","MVK",0.0227,"Inflammation","B04411","20212097_SS211642","PASS",0.0287,2.2353,"Plate control","PASS","PFC",4.70860597416686,"Baseline","Baseline"
10482,"PFC_10",14,"OID20459","Q03426","MVK",0.0227,"Inflammation","B04411","20212097_SS211642","PASS",0.0287,1.8993,"Plate control","PASS","PFC",3.73032156366426,"1h","1h"
10483,"PFC_18",15,"OID20459","Q03426","MVK",0.0227,"Inflammation","B04411","20212097_SS211642","PASS",0.0287,3.2342,"Plate control","PASS","PFC",9.4100344196757,"1h","1h"
10484,"PFC_26",16,"OID20459","Q03426","MVK",0.0227,"Inflammation","B04411","20212097_SS211642","PASS",0.0287,2.8216,"Plate control","PASS","PFC",7.06945990627994,"24h","24h"
10485,"PFC_03",25,"OID20459","Q03426","MVK",0.0227,"Inflammation","B04411","20212097_SS211642","PASS",0.0287,1.038,"Plate control","PASS","PFC",2.05337909144,"Baseline","Baseline"
10486,"PFC_11",26,"OID20459","Q03426","MVK",0.0227,"Inflammation","B04411","20212097_SS211642","PASS",0.0287,3.1338,"Plate control","PASS","PFC",8.7774386049228,"1h","1h"
10487,"PFC_19",27,"OID20459","Q03426","MVK",0.0227,"Inflammation","B04411","20212097_SS211642","PASS",0.0287,2.2566,"Plate control","PASS","PFC",4.77863971547893,"24h","24h"
10488,"PFC_27",28,"OID20459","Q03426","MVK",0.0227,"Inflammation","B04411","20212097_SS211642","PASS",0.0287,3.7501,"Plate control","PASS","PFC",13.4552752603481,"24h","24h"
10489,"PFC_04",37,"OID20459","Q03426","MVK",0.0227,"Inflammation","B04411","20212097_SS211642","PASS",0.0287,2.7402,"Plate control","PASS","PFC",6.6816295617642,"Baseline","Baseline"
10490,"PFC_12",38,"OID20459","Q03426","MVK",0.0227,"Inflammation","B04411","20212097_SS211642","PASS",0.0287,1.422,"Plate control","PASS","PFC",2.67956720485568,"1h","1h"
10491,"PFC_20",39,"OID20459","Q03426","MVK",0.0227,"Inflammation","B04411","20212097_SS211642","PASS",0.0287,3.2586,"Plate control","PASS","PFC",9.57053782143327,"24h","24h"
10492,"PFC_05",49,"OID20459","Q03426","MVK",0.0227,"Inflammation","B04411","20212097_SS211642","PASS",0.0287,1.7553,"Plate control","PASS","PFC",3.37596512374762,"Baseline","Baseline"
10493,"PFC_13",50,"OID20459","Q03426","MVK",0.0227,"Inflammation","B04411","20212097_SS211642","PASS",0.0287,2.7209,"Plate control","PASS","PFC",6.592839682964,"1h","1h"
10494,"PFC_21",51,"OID20459","Q03426","MVK",0.0227,"Inflammation","B04411","20212097_SS211642","PASS",0.0287,1.461,"Plate control","PASS","PFC",2.75299120318139,"24h","24h"
10495,"PFC_06",61,"OID20459","Q03426","MVK",0.0227,"Inflammation","B04411","20212097_SS211642","PASS",0.0287,3.6322,"Plate control","PASS","PFC",12.3994136999349,"Baseline","Baseline"
10496,"PFC_14",62,"OID20459","Q03426","MVK",0.0227,"Inflammation","B04411","20212097_SS211642","PASS",0.0287,2.7413,"Plate control","PASS","PFC",6.6867259923959,"1h","1h"
10497,"PFC_22",63,"OID20459","Q03426","MVK",0.0227,"Inflammation","B04411","20212097_SS211642","PASS",0.0287,2.3366,"Plate control","PASS","PFC",5.05110838860432,"24h","24h"
10498,"PFC_07",73,"OID20459","Q03426","MVK",0.0227,"Inflammation","B04411","20212097_SS211642","PASS",0.0287,2.4369,"Plate control","PASS","PFC",5.41476977996119,"Baseline","Baseline"
10499,"PFC_15",74,"OID20459","Q03426","MVK",0.0227,"Inflammation","B04411","20212097_SS211642","PASS",0.0287,2.3316,"Plate control","PASS","PFC",5.0336328811518,"1h","1h"
10500,"PFC_23",75,"OID20459","Q03426","MVK",0.0227,"Inflammation","B04411","20212097_SS211642","PASS",0.0287,2.9561,"Plate control","PASS","PFC",7.76023316246588,"24h","24h"
10501,"PFC_08",85,"OID20459","Q03426","MVK",0.0227,"Inflammation","B04411","20212097_SS211642","PASS",0.0287,2.0015,"Plate control","PASS","PFC",4.00416104587142,"Baseline","Baseline"
10502,"PFC_16",86,"OID20459","Q03426","MVK",0.0227,"Inflammation","B04411","20212097_SS211642","PASS",0.0287,2.6073,"Plate control","PASS","PFC",6.09362196014004,"1h","1h"
10503,"PFC_24",87,"OID20459","Q03426","MVK",0.0227,"Inflammation","B04411","20212097_SS211642","PASS",0.0287,3.5231,"Plate control","PASS","PFC",11.496318248794,"24h","24h"
10504,"PFC_01",1,"OID20460","O14904","WNT9A",0,"Inflammation","B04411","20212097_SS211642","PASS",-0.2489,0.6152,"Plate control","PASS","PFC",1.53177033083341,"Baseline","Baseline"
10505,"PFC_09",2,"OID20460","O14904","WNT9A",0,"Inflammation","B04411","20212097_SS211642","PASS",-0.2489,0.8854,"Plate control","PASS","PFC",1.84727672432425,"Baseline","Baseline"
10506,"PFC_17",3,"OID20460","O14904","WNT9A",0,"Inflammation","B04411","20212097_SS211642","PASS",-0.2489,0.3254,"Plate control","PASS","PFC",1.25301179910582,"1h","1h"
10507,"PFC_25",4,"OID20460","O14904","WNT9A",0,"Inflammation","B04411","20212097_SS211642","PASS",-0.2489,0.8072,"Plate control","PASS","PFC",1.7498120884615,"24h","24h"
10508,"PFC_02",13,"OID20460","O14904","WNT9A",0,"Inflammation","B04411","20212097_SS211642","PASS",-0.2489,0.7441,"Plate control","PASS","PFC",1.67492906822945,"Baseline","Baseline"
10509,"PFC_10",14,"OID20460","O14904","WNT9A",0,"Inflammation","B04411","20212097_SS211642","PASS",-0.2489,0.851,"Plate control","PASS","PFC",1.8037507567645,"1h","1h"
10510,"PFC_18",15,"OID20460","O14904","WNT9A",0,"Inflammation","B04411","20212097_SS211642","PASS",-0.2489,0.6931,"Plate control","PASS","PFC",1.61675379856107,"1h","1h"
10511,"PFC_26",16,"OID20460","O14904","WNT9A",0,"Inflammation","B04411","20212097_SS211642","PASS",-0.2489,0.6809,"Plate control","PASS","PFC",1.60313953378274,"24h","24h"
10512,"PFC_03",25,"OID20460","O14904","WNT9A",0,"Inflammation","B04411","20212097_SS211642","PASS",-0.2489,0.8705,"Plate control","PASS","PFC",1.82829642992798,"Baseline","Baseline"
10513,"PFC_11",26,"OID20460","O14904","WNT9A",0,"Inflammation","B04411","20212097_SS211642","PASS",-0.2489,0.9261,"Plate control","PASS","PFC",1.90013246833157,"1h","1h"
10514,"PFC_19",27,"OID20460","O14904","WNT9A",0,"Inflammation","B04411","20212097_SS211642","PASS",-0.2489,0.5666,"Plate control","PASS","PFC",1.48102911262837,"24h","24h"
10515,"PFC_27",28,"OID20460","O14904","WNT9A",0,"Inflammation","B04411","20212097_SS211642","PASS",-0.2489,0.334,"Plate control","PASS","PFC",1.26050339158299,"24h","24h"
10516,"PFC_04",37,"OID20460","O14904","WNT9A",0,"Inflammation","B04411","20212097_SS211642","PASS",-0.2489,0.999,"Plate control","PASS","PFC",1.99861418598091,"Baseline","Baseline"
10517,"PFC_12",38,"OID20460","O14904","WNT9A",0,"Inflammation","B04411","20212097_SS211642","PASS",-0.2489,0.6963,"Plate control","PASS","PFC",1.62034385326609,"1h","1h"
10518,"PFC_20",39,"OID20460","O14904","WNT9A",0,"Inflammation","B04411","20212097_SS211642","PASS",-0.2489,0.5175,"Plate control","PASS","PFC",1.43147254464261,"24h","24h"
10519,"PFC_05",49,"OID20460","O14904","WNT9A",0,"Inflammation","B04411","20212097_SS211642","PASS",-0.2489,0.3335,"Plate control","PASS","PFC",1.26006661008984,"Baseline","Baseline"
10520,"PFC_13",50,"OID20460","O14904","WNT9A",0,"Inflammation","B04411","20212097_SS211642","PASS",-0.2489,0.8678,"Plate control","PASS","PFC",1.8248779777457,"1h","1h"
10521,"PFC_21",51,"OID20460","O14904","WNT9A",0,"Inflammation","B04411","20212097_SS211642","PASS",-0.2489,0.938,"Plate control","PASS","PFC",1.91587043644539,"24h","24h"
10522,"PFC_06",61,"OID20460","O14904","WNT9A",0,"Inflammation","B04411","20212097_SS211642","PASS",-0.2489,0.6231,"Plate control","PASS","PFC",1.54018110199114,"Baseline","Baseline"
10523,"PFC_14",62,"OID20460","O14904","WNT9A",0,"Inflammation","B04411","20212097_SS211642","PASS",-0.2489,0.4496,"Plate control","PASS","PFC",1.365661562474,"1h","1h"
10524,"PFC_22",63,"OID20460","O14904","WNT9A",0,"Inflammation","B04411","20212097_SS211642","PASS",-0.2489,0.9903,"Plate control","PASS","PFC",1.98659804937708,"24h","24h"
10525,"PFC_07",73,"OID20460","O14904","WNT9A",0,"Inflammation","B04411","20212097_SS211642","PASS",-0.2489,0.4165,"Plate control","PASS","PFC",1.33468565633012,"Baseline","Baseline"
10526,"PFC_15",74,"OID20460","O14904","WNT9A",0,"Inflammation","B04411","20212097_SS211642","PASS",-0.2489,0.4341,"Plate control","PASS","PFC",1.35106773016888,"1h","1h"
10527,"PFC_23",75,"OID20460","O14904","WNT9A",0,"Inflammation","B04411","20212097_SS211642","PASS",-0.2489,0.3886,"Plate control","PASS","PFC",1.30912240695901,"24h","24h"
10528,"PFC_08",85,"OID20460","O14904","WNT9A",0,"Inflammation","B04411","20212097_SS211642","PASS",-0.2489,0.1563,"Plate control","PASS","PFC",1.1144253649666,"Baseline","Baseline"

Supplemental table 1_Olink proximity extension assay (PEA) data

,"SampleID","Index","OlinkID","UniProt","Assay","MissingFreq","Panel","Panel_Lot_Nr","PlateID","QC_Warning","LOD","NPX","Normalization","Assay_Warning","condition","Value","group","alt_group"
10529,"PFC_16",86,"OID20460","O14904","WNT9A",0,"Inflammation","B04411","20212097_SS211642","PASS",-0.2489,0.8582,"Plate control","PASS","PFC",1.81277516311507,"1h","1h"
10530,"PFC_24",87,"OID20460","O14904","WNT9A",0,"Inflammation","B04411","20212097_SS211642","PASS",-0.2489,0.6584,"Plate control","PASS","PFC",1.57833122738152,"24h","24h"
10531,"PFC_01",1,"OID20461","P26951","IL3RA",0.4545,"Inflammation","B04411","20212097_SS211642","PASS",0.2169,0.4366,"Plate control","PASS","PFC",1.35341098182514,"Baseline","Baseline"
10532,"PFC_09",2,"OID20461","P26951","IL3RA",0.4545,"Inflammation","B04411","20212097_SS211642","PASS",0.2169,1.3085,"Plate control","PASS","PFC",2.47683883987077,"Baseline","Baseline"
10533,"PFC_17",3,"OID20461","P26951","IL3RA",0.4545,"Inflammation","B04411","20212097_SS211642","PASS",0.2169,0.1678,"Plate control","PASS","PFC",1.1233441636689,"1h","1h"
10534,"PFC_25",4,"OID20461","P26951","IL3RA",0.4545,"Inflammation","B04411","20212097_SS211642","PASS",0.2169,0.3713,"Plate control","PASS","PFC",1.29351788340524,"24h","24h"
10535,"PFC_02",13,"OID20461","P26951","IL3RA",0.4545,"Inflammation","B04411","20212097_SS211642","PASS",0.2169,0.9391,"Plate control","PASS","PFC",1.91733177169129,"Baseline","Baseline"
10536,"PFC_10",14,"OID20461","P26951","IL3RA",0.4545,"Inflammation","B04411","20212097_SS211642","PASS",0.2169,0.1422,"Plate control","PASS","PFC",1.10358671904258,"1h","1h"
10537,"PFC_18",15,"OID20461","P26951","IL3RA",0.4545,"Inflammation","B04411","20212097_SS211642","PASS",0.2169,0.9671,"Plate control","PASS","PFC",1.95490703195526,"1h","1h"
10538,"PFC_26",16,"OID20461","P26951","IL3RA",0.4545,"Inflammation","B04411","20212097_SS211642","PASS",0.2169,0.121,"Plate control","PASS","PFC",1.08748839085488,"24h","24h"
10539,"PFC_03",25,"OID20461","P26951","IL3RA",0.4545,"Inflammation","B04411","20212097_SS211642","PASS",0.2169,0.0289,"Plate control","PASS","PFC",1.02023393956966,"Baseline","Baseline"
10540,"PFC_11",26,"OID20461","P26951","IL3RA",0.4545,"Inflammation","B04411","20212097_SS211642","PASS",0.2169,0.8884,"Plate control","PASS","PFC",1.85112202493709,"1h","1h"
10541,"PFC_19",27,"OID20461","P26951","IL3RA",0.4545,"Inflammation","B04411","20212097_SS211642","PASS",0.2169,0.6289,"Plate control","PASS","PFC",1.5463854839219,"24h","24h"
10542,"PFC_27",28,"OID20461","P26951","IL3RA",0.4545,"Inflammation","B04411","20212097_SS211642","PASS",0.2169,1.2323,"Plate control","PASS","PFC",2.34941243821788,"24h","24h"
10543,"PFC_04",37,"OID20461","P26951","IL3RA",0.4545,"Inflammation","B04411","20212097_SS211642","PASS",0.2169,0.2016,"Plate control","PASS","PFC",1.14997300892634,"Baseline","Baseline"
10544,"PFC_12",38,"OID20461","P26951","IL3RA",0.4545,"Inflammation","B04411","20212097_SS211642","PASS",0.2169,0.1643,"Plate control","PASS","PFC",1.12062221680659,"1h","1h"
10545,"PFC_20",39,"OID20461","P26951","IL3RA",0.4545,"Inflammation","B04411","20212097_SS211642","PASS",0.2169,0.6066,"Plate control","PASS","PFC",1.52266650837339,"24h","24h"
10546,"PFC_05",49,"OID20461","P26951","IL3RA",0.4545,"Inflammation","B04411","20212097_SS211642","PASS",0.2169,0.641,"Plate control","PASS","PFC",1.55940968522265,"Baseline","Baseline"
10547,"PFC_13",50,"OID20461","P26951","IL3RA",0.4545,"Inflammation","B04411","20212097_SS211642","PASS",0.2169,0.3855,"Plate control","PASS","PFC",1.30631244203833,"1h","1h"
10548,"PFC_21",51,"OID20461","P26951","IL3RA",0.4545,"Inflammation","B04411","20212097_SS211642","PASS",0.2169,-0.0165,"Plate control","PASS","PFC",0.988628224567155,"24h","24h"
10549,"PFC_06",61,"OID20461","P26951","IL3RA",0.4545,"Inflammation","B04411","20212097_SS211642","PASS",0.2169,-0.2784,"Plate control","PASS","PFC",0.824504915613236,"Baseline","Baseline"
10550,"PFC_14",62,"OID20461","P26951","IL3RA",0.4545,"Inflammation","B04411","20212097_SS211642","PASS",0.2169,0.4176,"Plate control","PASS","PFC",1.33570369134684,"1h","1h"
10551,"PFC_22",63,"OID20461","P26951","IL3RA",0.4545,"Inflammation","B04411","20212097_SS211642","PASS",0.2169,0.0453,"Plate control","PASS","PFC",1.03189773409438,"24h","24h"
10552,"PFC_07",73,"OID20461","P26951","IL3RA",0.4545,"Inflammation","B04411","20212097_SS211642","PASS",0.2169,0.2186,"Plate control","PASS","PFC",1.16360387017668,"Baseline","Baseline"
10553,"PFC_15",74,"OID20461","P26951","IL3RA",0.4545,"Inflammation","B04411","20212097_SS211642","PASS",0.2169,-0.0341,"Plate control","PASS","PFC",0.976640831032178,"1h","1h"
10554,"PFC_23",75,"OID20461","P26951","IL3RA",0.4545,"Inflammation","B04411","20212097_SS211642","PASS",0.2169,0.5104,"Plate control","PASS","PFC",1.42444508088849,"24h","24h"
10555,"PFC_08",85,"OID20461","P26951","IL3RA",0.4545,"Inflammation","B04411","20212097_SS211642","PASS",0.2169,-0.5184,"Plate control","PASS","PFC",0.698145672315369,"Baseline","Baseline"
10556,"PFC_16",86,"OID20461","P26951","IL3RA",0.4545,"Inflammation","B04411","20212097_SS211642","PASS",0.2169,-0.065,"Plate control","PASS","PFC",0.955945317593742,"1h","1h"
10557,"PFC_24",87,"OID20461","P26951","IL3RA",0.4545,"Inflammation","B04411","20212097_SS211642","PASS",0.2169,-0.0159,"Plate control","PASS","PFC",0.989039468996991,"24h","24h"
10558,"PFC_01",1,"OID20462","Q9Y3P8","SIT1",0.1818,"Inflammation","B04411","20212097_SS211642","PASS",-0.3968,0.3106,"Plate control","PASS","PFC",1.24022338710384,"Baseline","Baseline"
10559,"PFC_09",2,"OID20462","Q9Y3P8","SIT1",0.1818,"Inflammation","B04411","20212097_SS211642","PASS",-0.3968,0.0548,"Plate control","PASS","PFC",1.03871509682797,"Baseline","Baseline"
10560,"PFC_17",3,"OID20462","Q9Y3P8","SIT1",0.1818,"Inflammation","B04411","20212097_SS211642","PASS",-0.3968,-0.2981,"Plate control","PASS","PFC",0.813322820936242,"1h","1h"
10561,"PFC_25",4,"OID20462","Q9Y3P8","SIT1",0.1818,"Inflammation","B04411","20212097_SS211642","PASS",-0.3968,-0.4874,"Plate control","PASS","PFC",0.71330945406469,"24h","24h"
10562,"PFC_02",13,"OID20462","Q9Y3P8","SIT1",0.1818,"Inflammation","B04411","20212097_SS211642","PASS",-0.3968,0.3764,"Plate control","PASS","PFC",1.29809862641096,"Baseline","Baseline"
10563,"PFC_10",14,"OID20462","Q9Y3P8","SIT1",0.1818,"Inflammation","B04411","20212097_SS211642","PASS",-0.3968,-0.5632,"Plate control","PASS","PFC",0.676799308715995,"1h","1h"
10564,"PFC_18",15,"OID20462","Q9Y3P8","SIT1",0.1818,"Inflammation","B04411","20212097_SS211642","PASS",-0.3968,0.2637,"Plate control","PASS","PFC",1.20055375339219,"1h","1h"
10565,"PFC_26",16,"OID20462","Q9Y3P8","SIT1",0.1818,"Inflammation","B04411","20212097_SS211642","PASS",-0.3968,-0.3597,"Plate control","PASS","PFC",0.779326619226981,"24h","24h"
10566,"PFC_03",25,"OID20462","Q9Y3P8","SIT1",0.1818,"Inflammation","B04411","20212097_SS211642","PASS",-0.3968,-0.414,"Plate control","PASS","PFC",0.750539548757186,"Baseline","Baseline"
10567,"PFC_11",26,"OID20462","Q9Y3P8","SIT1",0.1818,"Inflammation","B04411","20212097_SS211642","PASS",-0.3968,0.5626,"Plate control","PASS","PFC",1.47692851527523,"1h","1h"
10568,"PFC_19",27,"OID20462","Q9Y3P8","SIT1",0.1818,"Inflammation","B04411","20212097_SS211642","PASS",-0.3968,-0.4312,"Plate control","PASS","PFC",0.741644646014651,"24h","24h"
10569,"PFC_27",28,"OID20462","Q9Y3P8","SIT1",0.1818,"Inflammation","B04411","20212097_SS211642","PASS",-0.3968,0.4171,"Plate control","PASS","PFC",1.33524085193157,"24h","24h"
10570,"PFC_04",37,"OID20462","Q9Y3P8","SIT1",0.1818,"Inflammation","B04411","20212097_SS211642","PASS",-0.3968,-0.3058,"Plate control","PASS","PFC",0.808993490899893,"Baseline","Baseline"
10571,"PFC_12",38,"OID20462","Q9Y3P8","SIT1",0.1818,"Inflammation","B04411","20212097_SS211642","PASS",-0.3968,-0.2737,"Plate control","PASS","PFC",0.827195360999897,"1h","1h"
10572,"PFC_20",39,"OID20462","Q9Y3P8","SIT1",0.1818,"Inflammation","B04411","20212097_SS211642","PASS",-0.3968,0.5279,"Plate control","PASS","PFC",1.44182892803086,"24h","24h"
10573,"PFC_05",49,"OID20462","Q9Y3P8","SIT1",0.1818,"Inflammation","B04411","20212097_SS211642","PASS",-0.3968,0.0279,"Plate control","PASS","PFC",1.01952701232155,"Baseline","Baseline"
10574,"PFC_13",50,"OID20462","Q9Y3P8","SIT1",0.1818,"Inflammation","B04411","20212097_SS211642","PASS",-0.3968,-0.5482,"Plate control","PASS","PFC",0.683872840473319,"1h","1h"
10575,"PFC_21",51,"OID20462","Q9Y3P8","SIT1",0.1818,"Inflammation","B04411","20212097_SS211642","PASS",-0.3968,0.0317,"Plate control","PASS","PFC",1.02221594467769,"24h","24h"
10576,"PFC_06",61,"OID20462","Q9Y3P8","SIT1",0.1818,"Inflammation","B04411","20212097_SS211642","PASS",-0.3968,0.0112,"Plate control","PASS","PFC",1.00779346056616,"Baseline","Baseline"
10577,"PFC_14",62,"OID20462","Q9Y3P8","SIT1",0.1818,"Inflammation","B04411","20212097_SS211642","PASS",-0.3968,-0.2116,"Plate control","PASS","PFC",0.863578960313363,"1h","1h"
10578,"PFC_22",63,"OID20462","Q9Y3P8","SIT1",0.1818,"Inflammation","B04411","20212097_SS211642","PASS",-0.3968,-0.0124,"Plate control","PASS","PFC",0.991441806590304,"24h","24h"
10579,"PFC_07",73,"OID20462","Q9Y3P8","SIT1",0.1818,"Inflammation","B04411","20212097_SS211642","PASS",-0.3968,-0.6416,"Plate control","PASS","PFC",0.641001660842258,"Baseline","Baseline"
10580,"PFC_15",74,"OID20462","Q9Y3P8","SIT1",0.1818,"Inflammation","B04411","20212097_SS211642","PASS",-0.3968,-0.3181,"Plate control","PASS","PFC",0.802125565318118,"1h","1h"
10581,"PFC_23",75,"OID20462","Q9Y3P8","SIT1",0.1818,"Inflammation","B04411","20212097_SS211642","PASS",-0.3968,0.4936,"Plate control","PASS","PFC",1.40795380510648,"24h","24h"
10582,"PFC_08",85,"OID20462","Q9Y3P8","SIT1",0.1818,"Inflammation","B04411","20212097_SS211642","PASS",-0.3968,-0.3166,"Plate control","PASS","PFC",0.8029599856361,"Baseline","Baseline"
10583,"PFC_16",86,"OID20462","Q9Y3P8","SIT1",0.1818,"Inflammation","B04411","20212097_SS211642","PASS",-0.3968,-0.2531,"Plate control","PASS","PFC",0.839091473701852,"1h","1h"
10584,"PFC_24",87,"OID20462","Q9Y3P8","SIT1",0.1818,"Inflammation","B04411","20212097_SS211642","PASS",-0.3968,0.6218,"Plate control","PASS","PFC",1.53879388324549,"24h","24h"
10585,"PFC_01",1,"OID20463","Q96DB9","FXYD5",0.0909,"Inflammation","B04411","20212097_SS211642","PASS",0.6645,0.8604,"Plate control","PASS","PFC",1.81554161587907,"Baseline","Baseline"
10586,"PFC_09",2,"OID20463","Q96DB9","FXYD5",0.0909,"Inflammation","B04411","20212097_SS211642","PASS",0.6645,1.5308,"Plate control","PASS","PFC",2.88946020384204,"Baseline","Baseline"
10587,"PFC_17",3,"OID20463","Q96DB9","FXYD5",0.0909,"Inflammation","B04411","20212097_SS211642","PASS",0.6645,2.1277,"Plate control","PASS","PFC",4.37020210393176,"1h","1h"
10588,"PFC_25",4,"OID20463","Q96DB9","FXYD5",0.0909,"Inflammation","B04411","20212097_SS211642","PASS",0.6645,1.784,"Plate control","PASS","PFC",3.44379675335562,"24h","24h"
10589,"PFC_02",13,"OID20463","Q96DB9","FXYD5",0.0909,"Inflammation","B04411","20212097_SS211642","PASS",0.6645,1.1343,"Plate control","PASS","PFC",2.19512029013446,"Baseline","Baseline"
10590,"PFC_10",14,"OID20463","Q96DB9","FXYD5",0.0909,"Inflammation","B04411","20212097_SS211642","PASS",0.6645,1.2404,"Plate control","PASS","PFC",2.36264029504011,"1h","1h"
10591,"PFC_18",15,"OID20463","Q96DB9","FXYD5",0.0909,"Inflammation","B04411","20212097_SS211642","PASS",0.6645,1.9755,"Plate control","PASS","PFC",3.9326451089616,"1h","1h"
10592,"PFC_26",16,"OID20463","Q96DB9","FXYD5",0.0909,"Inflammation","B04411","20212097_SS211642","PASS",0.6645,2.5945,"Plate control","PASS","PFC",6.03979674543695,"24h","24h"
10593,"PFC_03",25,"OID20463","Q96DB9","FXYD5",0.0909,"Inflammation","B04411","20212097_SS211642","PASS",0.6645,0.7909,"Plate control","PASS","PFC",1.73015345191488,"Baseline","Baseline"
10594,"PFC_11",26,"OID20463","Q96DB9","FXYD5",0.0909,"Inflammation","B04411","20212097_SS211642","PASS",0.6645,1.576,"Plate control","PASS","PFC",2.98142077359784,"1h","1h"
10595,"PFC_19",27,"OID20463","Q96DB9","FXYD5",0.0909,"Inflammation","B04411","20212097_SS211642","PASS",0.6645,1.749,"Plate control","PASS","PFC",3.36125500893313,"24h","24h"
10596,"PFC_27",28,"OID20463","Q96DB9","FXYD5",0.0909,"Inflammation","B04411","20212097_SS211642","PASS",0.6645,2.84,"Plate control","PASS","PFC",7.16020056742378,"24h","24h"
10597,"PFC_04",37,"OID20463","Q96DB9","FXYD5",0.0909,"Inflammation","B04411","20212097_SS211642","PASS",0.6645,1.8008,"Plate control","PASS","PFC",3.48413373159368,"Baseline","Baseline"
10598,"PFC_12",38,"OID20463","Q96DB9","FXYD5",0.0909,"Inflammation","B04411","20212097_SS211642","PASS",0.6645,0.9427,"Plate control","PASS","PFC",1.92212212115893,"1h","1h"
10599,"PFC_20",39,"OID20463","Q96DB9","FXYD5",0.0909,"Inflammation","B04411","20212097_SS211642","PASS",0.6645,1.8582,"Plate control","PASS","PFC",3.62555032623014,"24h","24h"
10600,"PFC_05",49,"OID20463","Q96DB9","FXYD5",0.0909,"Inflammation","B04411","20212097_SS211642","PASS",0.6645,0.9517,"Plate control","PASS","PFC",1.93415042213432,"Baseline","Baseline"
10601,"PFC_13",50,"OID20463","Q96DB9","FXYD5",0.0909,"Inflammation","B04411","20212097_SS211642","PASS",0.6645,1.5568,"Plate control","PASS","PFC",2.94200561486083,"1h","1h"
10602,"PFC_21",51,"OID20463","Q96DB9","FXYD5",0.0909,"Inflammation","B04411","20212097_SS211642","PASS",0.6645,1.2732,"Plate control","PASS","PFC",2.4169707268451,"24h","24h"
10603,"PFC_06",61,"OID20463","Q96DB9","FXYD5",0.0909,"Inflammation","B04411","20212097_SS211642","PASS",0.6645,1.1951,"Plate control","PASS","PFC",2.28960701916225,"Baseline","Baseline"
10604,"PFC_14",62,"OID20463","Q96DB9","FXYD5",0.0909,"Inflammation","B04411","20212097_SS211642","PASS",0.6645,1.1029,"Plate control","PASS","PFC",2.14786005976593,"1h","1h"
10605,"PFC_22",63,"OID20463","Q96DB9","FXYD5",0.0909,"Inflammation","B04411","20212097_SS211642","PASS",0.6645,1.4564,"Plate control","PASS","PFC",2.74422733308866,"24h","24h"
10606,"PFC_07",73,"OID20463","Q96DB9","FXYD5",0.0909,"Inflammation","B04411","20212097_SS211642","PASS",0.6645,2.1289,"Plate control","PASS","PFC",4.37383864803846,"Baseline","Baseline"
10607,"PFC_15",74,"OID20463","Q96DB9","FXYD5",0.0909,"Inflammation","B04411","20212097_SS211642","PASS",0.6645,0.7772,"Plate control","PASS","PFC",1.71380147640216,"1h","1h"
10608,"PFC_23",75,"OID20463","Q96DB9","FXYD5",0.0909,"Inflammation","B04411","20212097_SS211642","PASS",0.6645,1.4908,"Plate control","PASS","PFC",2.81044776261787,"24h","24h"
10609,"PFC_08",85,"OID20463","Q96DB9","FXYD5",0.0909,"Inflammation","B04411","20212097_SS211642","PASS",0.6645,1.721,"Plate control","PASS","PFC",3.29664833981296,"Baseline","Baseline"
10610,"PFC_16",86,"OID20463","Q96DB9","FXYD5",0.0909,"Inflammation","B04411","20212097_SS211642","PASS",0.6645,1.9812,"Plate control","PASS","PFC",3.94821348421547,"1h","1h"
10611,"PFC_24",87,"OID20463","Q96DB9","FXYD5",0.0909,"Inflammation","B04411","20212097_SS211642","PASS",0.6645,1.6735,"Plate control","PASS","PFC",3.18987524112599,"24h","24h"
10612,"PFC_01",1,"OID20464","P48061","CXCL12",0,"Inflammation","B04411","20212097_SS211642","PASS",-0.2881,1.3334,"Plate control","PASS","PFC",2.51995854390995,"Baseline","Baseline"
10613,"PFC_09",2,"OID20464","P48061","CXCL12",0,"Inflammation","B04411","20212097_SS211642","PASS",-0.2881,1.7423,"Plate control","PASS","PFC",3.34568124218237,"Baseline","Baseline"
10614,"PFC_17",3,"OID20464","P48061","CXCL12",0,"Inflammation","B04411","20212097_SS211642","PASS",-0.2881,1.2016,"Plate control","PASS","PFC",2.29994601785267,"1h","1h"
10615,"PFC_25",4,"OID20464","P48061","CXCL12",0,"Inflammation","B04411","20212097_SS211642","PASS",-0.2881,1.2507,"Plate control","PASS","PFC",2.37956852379863,"24h","24h"
10616,"PFC_02",13,"OID20464","P48061","CXCL12",0,"Inflammation","B04411","20212097_SS211642","PASS",-0.2881,0.5766,"Plate control","PASS","PFC",1.49133048475671,"Baseline","Baseline"
10617,"PFC_10",14,"OID20464","P48061","CXCL12",0,"Inflammation","B04411","20212097_SS211642","PASS",-0.2881,0.9496,"Plate control","PASS","PFC",1.93133710326236,"1h","1h"
10618,"PFC_18",15,"OID20464","P48061","CXCL12",0,"Inflammation","B04411","20212097_SS211642","PASS",-0.2881,1.4174,"Plate control","PASS","PFC",2.67103707266311,"1h","1h"
10619,"PFC_26",16,"OID20464","P48061","CXCL12",0,"Inflammation","B04411","20212097_SS211642","PASS",-0.2881,1.3197,"Plate control","PASS","PFC",2.49614198569803,"24h","24h"
10620,"PFC_03",25,"OID20464","P48061","CXCL12",0,"Inflammation","B04411","20212097_SS211642","PASS",-0.2881,0.8865,"Plate control","PASS","PFC",1.84868573953469,"Baseline","Baseline"
10621,"PFC_11",26,"OID20464","P48061","CXCL12",0,"Inflammation","B04411","20212097_SS211642","PASS",-0.2881,0.6179,"Plate control","PASS","PFC",1.53463971919474,"1h","1h"
10622,"PFC_19",27,"OID20464","P48061","CXCL12",0,"Inflammation","B04411","20212097_SS211642","PASS",-0.2881,1.5003,"Plate control","PASS","PFC",2.8290153407882,"24h","24h"
10623,"PFC_27",28,"OID20464","P48061","CXCL12",0,"Inflammation","B04411","20212097_SS211642","PASS",-0.2881,1.5551,"Plate control","PASS","PFC",2.93854094363463,"24h","24h"
10624,"PFC_04",37,"OID20464","P48061","CXCL12",0,"Inflammation","B04411","20212097_SS211642","PASS",-0.2881,1.582,"Plate control","PASS","PFC",2.99384597358538,"Baseline","Baseline"
10625,"PFC_12",38,"OID20464","P48061","CXCL12",0,"Inflammation","B04411","20212097_SS211642","PASS",-0.2881,0.4689,"Plate control","PASS","PFC",1.38405377742918,"1h","1h"
10626,"PFC_20",39,"OID20464","P48061","CXCL12",0,"Inflammation","B04411","20212097_SS211642","PASS",-0.2881,1.1047,"Plate control","PASS","PFC",2.15054154187407,"24h","24h"
10627,"PFC_05",49,"OID20464","P48061","CXCL12",0,"Inflammation","B04411","20212097_SS211642","PASS",-0.2881,1.6828,"Plate control","PASS","PFC",3.21050445359042,"Baseline","Baseline"
10628,"PFC_13",50,"OID20464","P48061","CXCL12",0,"Inflammation","B04411","20212097_SS211642","PASS",-0.2881,1.0993,"Plate control","PASS","PFC",2.14250712189555,"1h","1h"
10629,"PFC_21",51,"OID20464","P48061","CXCL12",0,"Inflammation","B04411","20212097_SS211642","PASS",-0.2881,1.1067,"Plate control","PASS","PFC",2.15352489291049,"24h","24h"
10630,"PFC_06",61,"OID20464","P48061","CXCL12",0,"Inflammation","B04411","20212097_SS211642","PASS",-0.2881,1.1336,"Plate control","PASS","PFC",2.19405546947438,"Baseline","Baseline"
10631,"PFC_14",62,"OID20464","P48061","CXCL12",0,"Inflammation","B04411","20212097_SS211642","PASS",-0.2881,0.8966,"Plate control","PASS","PFC",1.86167340263005,"1h","1h"
10632,"PFC_22",63,"OID20464","P48061","CXCL12",0,"Inflammation","B04411","20212097_SS211642","PASS",-0.2881,1.4295,"Plate control","PASS","PFC",2.69353348437794,"24h","24h"
10633,"PFC_07",73,"OID20464","P48061","CXCL12",0,"Inflammation","B04411","20212097_SS211642","PASS",-0.2881,1.8408,"Plate control","PASS","PFC",3.58208606337026,"Baseline","Baseline"
10634,"PFC_15",74,"OID20464","P48061","CXCL12",0,"Inflammation","B04411","20212097_SS211642","PASS",-0.2881,0.2068,"Plate control","PASS","PFC",1.15412541067138,"1h","1h"
10635,"PFC_23",75,"OID20464","P48061","CXCL12",0,"Inflammation","B04411","20212097_SS211642","PASS",-0.2881,1.5296,"Plate control","PASS","PFC",2.88705781767251,"24h","24h"
10636,"PFC_08",85,"OID20464","P48061","CXCL12",0,"Inflammation","B04411","20212097_SS211642","PASS",-0.2881,0.903,"Plate control","PASS","PFC",1.86995039550291,"Baseline","Baseline"
10637,"PFC_16",86,"OID20464","P48061","CXCL12",0,"Inflammation","B04411","20212097_SS211642","PASS",-0.2881,1.231,"Plate control","PASS","PFC",2.3472963565627,"1h","1h"
10638,"PFC_24",87,"OID20464","P48061","CXCL12",0,"Inflammation","B04411","20212097_SS211642","PASS",-0.2881,0.9786,"Plate control","PASS","PFC",1.970552245038,"24h","24h"
10639,"PFC_01",1,"OID20465","Q99748","NRTN",0.6591,"Inflammation","B04411","20212097_SS211642","PASS",0.6838,-0.3825,"Plate control","PASS","PFC",0.767107142848627,"Baseline","Baseline"
10640,"PFC_09",2,"OID20465","Q99748","NRTN",0.6591,"Inflammation","B04411","20212097_SS211642","PASS",0.6838,0.8177,"Plate control","PASS","PFC",1.76259375663588,"Baseline","Baseline"

Supplemental table 1_Olink proximity extension assay (PEA) data

,"SampleID","Index","OlinkID","UniProt","Assay","MissingFreq","Panel","Panel_Lot_Nr","PlateID","QC_Warning","LOD","NPX","Normalization","Assay_Warning","condition","Value","group","alt_group"
10641,"PFC_17",3,"OID20465","Q99748","NRTN",0.6591,"Inflammation","B04411","20212097_SS211642","PASS",0.6838,0.1123,"Plate control","PASS","PFC",1.08095015593629,"1h","1h"
10642,"PFC_25",4,"OID20465","Q99748","NRTN",0.6591,"Inflammation","B04411","20212097_SS211642","PASS",0.6838,-0.0424,"Plate control","PASS","PFC",0.971038229250796,"24h","24h"
10643,"PFC_02",13,"OID20465","Q99748","NRTN",0.6591,"Inflammation","B04411","20212097_SS211642","PASS",0.6838,0.8621,"Plate control","PASS","PFC",1.81768222066123,"Baseline","Baseline"
10644,"PFC_10",14,"OID20465","Q99748","NRTN",0.6591,"Inflammation","B04411","20212097_SS211642","PASS",0.6838,-0.0495,"Plate control","PASS","PFC",0.966271154963923,"1h","1h"
10645,"PFC_18",15,"OID20465","Q99748","NRTN",0.6591,"Inflammation","B04411","20212097_SS211642","PASS",0.6838,-0.1037,"Plate control","PASS","PFC",0.930643159388157,"1h","1h"
10646,"PFC_26",16,"OID20465","Q99748","NRTN",0.6591,"Inflammation","B04411","20212097_SS211642","PASS",0.6838,0.6772,"Plate control","PASS","PFC",1.5990333184277,"24h","24h"
10647,"PFC_03",25,"OID20465","Q99748","NRTN",0.6591,"Inflammation","B04411","20212097_SS211642","PASS",0.6838,-0.2736,"Plate control","PASS","PFC",0.827252699800311,"Baseline","Baseline"
10648,"PFC_11",26,"OID20465","Q99748","NRTN",0.6591,"Inflammation","B04411","20212097_SS211642","PASS",0.6838,0.642,"Plate control","PASS","PFC",1.56049096034741,"1h","1h"
10649,"PFC_19",27,"OID20465","Q99748","NRTN",0.6591,"Inflammation","B04411","20212097_SS211642","PASS",0.6838,-0.9301,"Plate control","PASS","PFC",0.524821962664371,"24h","24h"
10650,"PFC_27",28,"OID20465","Q99748","NRTN",0.6591,"Inflammation","B04411","20212097_SS211642","PASS",0.6838,0.2865,"Plate control","PASS","PFC",1.21967772892453,"24h","24h"
10651,"PFC_04",37,"OID20465","Q99748","NRTN",0.6591,"Inflammation","B04411","20212097_SS211642","PASS",0.6838,0.5736,"Plate control","PASS","PFC",1.48823257227459,"Baseline","Baseline"
10652,"PFC_12",38,"OID20465","Q99748","NRTN",0.6591,"Inflammation","B04411","20212097_SS211642","PASS",0.6838,-1.0905,"Plate control","PASS","PFC",0.469598595929858,"1h","1h"
10653,"PFC_20",39,"OID20465","Q99748","NRTN",0.6591,"Inflammation","B04411","20212097_SS211642","PASS",0.6838,0.379,"Plate control","PASS","PFC",1.30044014654848,"24h","24h"
10654,"PFC_05",49,"OID20465","Q99748","NRTN",0.6591,"Inflammation","B04411","20212097_SS211642","PASS",0.6838,0.7043,"Plate control","PASS","PFC",1.62935390553534,"Baseline","Baseline"
[truncated: 13,158,324 more chars]
